# Identification of copy number variations using high density whole-genome single nucleotide polymorphism markers in Chinese Dongxiang spotted pigs

**DOI:** 10.5713/ajas.18.0696

**Published:** 2019-02-07

**Authors:** Chengbin Wang, Hao Chen, Xiaopeng Wang, Zhongping Wu, Weiwei Liu, Yuanmei Guo, Jun Ren, Nengshui Ding

**Affiliations:** 1 State Key Laboratory of Pig Genetic Improvement and Production Technology, Jiangxi Agricultural University, Nanchang 330045, China

**Keywords:** Copy Number Variation, Pig1.4M Array Plate, Next Generation Sequence, Complex Traits, Chinese Indigenous Pigs

## Abstract

**Objective:**

Copy number variations (CNVs) are a major source of genetic diversity complementary to single nucleotide polymorphism (SNP) in animals. The aim of the study was to perform a comprehensive genomic analysis of CNVs based on high density whole-genome SNP markers in Chinese Dongxiang spotted pigs.

**Methods:**

We used customized Affymetrix Axiom Pig1.4M array plates containing 1.4 million SNPs and the PennCNV algorithm to identify porcine CNVs on autosomes in Chinese Dongxiang spotted pigs. Then, the next generation sequence data was used to confirm the detected CNVs. Next, functional analysis was performed for gene contents in copy number variation regions (CNVRs). In addition, we compared the identified CNVRs with those reported ones and quantitative trait loci (QTL) in the pig QTL database.

**Results:**

We identified 871 putative CNVs belonging to 2,221 CNVRs on 17 autosomes. We further discarded CNVRs that were detected only in one individual, leaving us 166 CNVRs in total. The 166 CNVRs ranged from 2.89 kb to 617.53 kb with a mean value of 93.65 kb and a genome coverage of 15.55 Mb, corresponding to 0.58% of the pig genome. A total of 119 (71.69%) of the identified CNVRs were confirmed by next generation sequence data. Moreover, functional annotation showed that these CNVRs are involved in a variety of molecular functions. More than half (56.63%) of the CNVRs (n = 94) have been reported in previous studies, while 72 CNVRs are reported for the first time. In addition, 162 (97.59%) CNVRs were found to overlap with 2,765 previously reported QTLs affecting 378 phenotypic traits.

**Conclusion:**

The findings improve the catalog of pig CNVs and provide insights and novel molecular markers for further genetic analyses of Chinese indigenous pigs.

## INTRODUCTION

Copy number variations (CNVs) refer to the insertion, deletion and duplication of DNA segments of 1 kb or larger with variable copy number compared with the reference genome. Since the milestone work by Iafrate et al [1] and Sebat et al [2] that first reported CNVs in the human genome, thousands of CNVs have been detected across the human genome. It has been reported that CNVs account for 3.7%, 4.6%, 4.2%, 1.4%, 3.6%, and 5.8% of human, cattle, dog, rat, horse, and pig assembled genomes, respectively [3–8].

A list of CNVs are known to affect phenotypic traits in humans [3]. In livestock, CNVs have also been repeatedly shown to cause phenotypic variations. For instance, the duplication of the proto-oncogene receptor tyrosine kinase (*KIT*) gene is responsible for the white coat phenotype in pigs [9]. The copy number alteration in intron 1 of the sex determining region Y-Box (*SOX5*) gene contributes to the pea-comb phenotype in chickens [10]. A 4.6-kb duplication in intron 6 of the syntaxin 17 gene causes hair graying and melanoma in horses [11], and CNV and missense mutations of the agouti signaling protein gene lead to different coat colors in goats [12].

Currently, CNVs can be identified using three different approaches, including single nucleotide polymorphism (SNP) genotyping array, comparative genomic hybridization and next-generation sequencing [13]. Of these three approaches, SNP genotyping array has been popular in large-scale CNV surveys due to it relatively lower cost [14]. In pigs, Fadista et al [15] first reported 37 copy number variation regions (CNVRs) that were identified in 12 unrelated Duroc pigs using the comparative genomic hybridization method. Then, Ramayo-Caldas et al [16] detected 49 CNVRs in 55 animals belonging to several generations of the Iberian boars crossing with Landrace sows using Porcine SNP60K BeadChip array. Afterwards, a number of CNVs were inferred from whole-genome 60K SNP data of diverse pig breeds [8,14,17].

Dongxiang spotted pigs, a representative Chinese local pig breed, was originally distributed in Dongxiang county, Jiangxi province of China. Dongxiang spotted pigs are well adapted to local environments and roughage feed occurring in the middle and lower area of the Yangtze River. This breed is also known for strong disease resistance and desirable meat quality, providing a valuable genetic resource for further genetic improvement of commercial breeds in Chinese pig industry [18]. We herein used a customized Affymetrix Axiom Pig1.4M array plate containing 1.4 million SNPs and the PennCNV algorithm to identify porcine autosomal CNVs in Chinese Dongxiang spotted pigs, aiming to improve the catalog of pig CNVs and facilitate the identification of trait-related CNVs for further selective breeding of pig breeds.

## MATERIALS AND METHODS

### Ethics statements

All animal work was carried out according to the approved guidelines established by the Ministry of Agriculture of China. The Ethics Committee of Jiangxi Agricultural University specifically approved this study.

### Experimental animals

Samples were collected from ear tissues of 96 Dongxiang spotted pigs as described in our previous report [19]. Briefly, these 96 pigs were sampled from the nucleus population of Dongxiang spotted pigs in a national conservation farm in Dongxiang county, Jiangxi Province, China. The farm is the only state-supported farm specifically for the conservation of Dongxiang spotted pigs. The nucleus population currently comprises ~120 sows and 15 boars and is managed to maintain its genetic diversity without intensive selection for any particular traits.

### Genotyping and quality control

Genomic DNA from the 96 individuals was extracted from ear tissue using a standard phenol/chloroform method [20]. DNA quality was measured by spectrophotometry and agarose gel electrophoresis and was finally diluted to a concentration of 50 ng/μL. All 96 pigs were genotyped with a customized Affymetrix Axiom Pig1.4M array plate as previously described [19]. Briefly, a total of 1,362,630 SNPs were successfully genotyped for these 96 pigs. Before CNV calling, we first performed a quality control by Plink v1.07 [21]. After quality control procedures, one individual was removed due to its missing genotype date rate (>0.1), 678 SNPs were discarded due to Hardy-Weinberg exact test, and 85,205 and 711,888 SNPs were discarded due to their genotype call rates (<0.90) and minor allele frequencies (<0.05), respectively. A final set of 566,795 SNPs were retained for subsequent CNV calling.

### Copy number variations calling

The CNVs were called using the PENNCNV software based on a hidden Markov model (HMM) and SNP chip data [22]. The PENNCNV algorithm incorporates various sets of information such as total signal intensity (Log R ratio [LRR]), the allelic intensity ratio (B allele frequency, [BAF]), the distance between SNPs, the population frequency of B allele (PFB) simultaneously for accurate CNV detection. Canonical genotype clustering files were generated successively by generating affy_geno_cluster.pl and normalize_affy_geno_cluster.pl programs from three original chip genotype information files: AxiomGT1.call.txt, AxiomGT1.confidences.txt and AxiomGT1.summary.txt. Individual-based signal intensity (LRR) was split by kcolumn.pl program from the canonical genotype clustering files that contain the LRR values and the BAF values for each marker in each individual [23].

The *Sscrofa* genome assembly (10.2 version, ftp://ftp.ensembl.org/pub/release-89/fasta/sus_scrofa/dna/) was explored to annotate SNPs. The PFB file was created from signal intensity data, which was then explored to detect raw CNV calls using the default –*test* algorithm of the hhall.hmm model program. To minimize false-positive rate in CNV identification, we used strict quality control and filtering criteria to generate raw CNV calls. Individuals with a standard deviation of LRR>0.3, BAF drift>0.02, a waviness factor>0.05 or <−0.05 were filtered. Five samples were discarded during quality control, leaving us 90 animals for further analyses. Finally, the PENNCNV software was explored to identify loss (deletion) and gain (duplication) events in target regions. Overlapped CNVs that were detected by more than one individual in a certain region were merged to form CNVRs. According to the distribution frequencies of these CNVs in their covered region, the location of the 2.5% cumulative frequencies at both end of the region was defined as the start and end position of CNVRs.

### Validation of copy number variation regions by whole-genome sequence data

Two DNA pools each comprising 12 Dongxiang spotted pigs were re-sequenced with paired-end reads of 150 bp at 30× coverage using a Hiseq/NovaSeq platform. One pool contained DNA of 12 sows and the other included DNA of 10 sows and two boars. Reads were aligned against the *Sscrofa* reference genome (version 10.2) using Burrows-Wheeler Aligner (BWA) [24]. The bam file of mapped paired-end reads was sorted and indexed with marked PCR duplicates and local realignment and qualities were recalibrated. CNV caller software was then explored to detect CNVs [25]. The main steps included: i) divided the reference genome into the specified size of sliding window; ii) counted the number of read segments in the matching window; iii) absolute copy number correction to the similarity between the windows; iv) corrected and normalized GC contents; v) detected copy number based on the standardized reading signal [25]. We selected 400 bp as a sliding window and adopted the suggested parameters.

### Gene contents and functional annotation in copy number variation regions

Gene contents in CNVRs were retrieved using the genes in ensemble genes 89 database downloaded from ftp://ftp.ensembl.org/pub/release-89/gff3/sus_scrofa//Sus_scrofa.Sscrofa10.2.89.chr.gff3.gz. We first converted the unannotated pig ensemble genes to orthologous mouse ensemble genes, then functional annotation was performed with the clueGO function in Cytoscape v3.6.0 (http://www.cytoscape.org/) for gene ontology (GO) and Kyoto encyclopedia of genes and genomes (KEGG) pathway enrichment analyses. We also compared the identified CNVRs with the reported quantitative trait loci (QTL) in the pig QTL database (http://www.animalgenome.org/cgi-bin/QTLdb/SS/index) or those CNVs reported in previous studies.

## RESULTS

### Genome-wide detection of copy number variations

**Figure 1:**
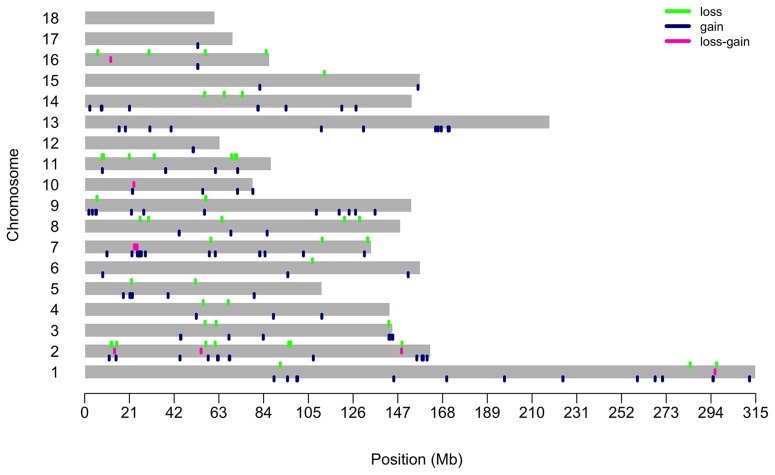
Distribution of copy number variation regions in the genome of Dongxiang spotted pigs. The X-axis values represent the chromosome position in Mb on the *Sus scrofa* 10.2 reference genome assembly. The Y-axis show all autosomal chromosomes.

In the present study, 90 Dongxiang spotted pigs were successfully genotyped using the Affymetrix Axiom Pig1.4M array plate. After quality control, the filtered SNPs were explored to detect CNVs using the PennCNV software. A total of 3,871 CNVs were identified on 17 autosomes. The average number of CNVs was 43.01 per individual. By aggregating the overlapping CNVs, we detected 2,221 CNVRs on the 17 autosomes. We further eliminated the CNVRs that were detected only in one individual, leaving us 166 CNVRs. The 166 CNVRs ranged from 2.89 kb to 617.53 kb, with a mean value of 93.65 kb and a genome coverage of 15.55 Mb, corresponding to 0.58% of the pig genome. Of the 166 CNVRs, 111 (66.87%) were identified as gain events, 46 (27.71%) as loss events, and 9 (5.42%) as gain-loss event (Supplementary Table S1). The 166 CNVRs were not uniformly distributed across the pig genome (Figure 1). Twenty-six (15.66%) CNVRs were detected on *Sus scrofa* chromosome (SSC) 2, while no CNVR was found on SSC18. On average, 1.84 CNVRs were detected per individual.

### Validation of copy number variation regions by whole-genome sequence data

Whole-genome sequence data of two DNA pools of Dongxiang spotted pigs were explored to detect CNVRs using CNVcaller [25]. We detected 700 discrete CNVRs based on the whole-genome sequence data, of which 119 perfectly overlapped with the 166 CNVRs inferred from the whole-genome 1.4 M SNP data (Supplementary Table S2).

### Comparison of the identified copy number variation regions with previous reports

We compared the predicted CNVRs in this study with the reported CNVRs inferring from whole-genome chip SNP markers in 11 previous studies (Table 1). In total, 94 (56.63%) CNVRs were consistent with previous studies. The other 72 (43.37%) CNVRs were reported for the first time, of which 39 were validated by the whole-genome sequence data (Supplementary Table S3). The best concordance (36.75% CNVR count, 9.52% CNVR length) was observed between this study and the previous report by Paudel et al [26]. The second best match (21.69% CNVR count and 7.58% CNVR length) was found when compared to the results of Chen et al [8].

### Analysis of genes content and overlapped quantitative trait loci within copy number variation regions

A total of 100 genes partially or entirely spanning 77 CNVRs were retrieved from the ensemble genes 89 database, including 92 protein-coding genes, three snRNA genes, one miRNA gene, one rRNA gene and three pseudogenes (Supplementary Table S4). To better understand the functional enrichment of the 100 genes, we performed GO and KEGG pathway analyses of these genes using the clueGO function in the Cytoscape software (http://www.cytoscape.org/). We found 8 statistically significant GO terms enriched for these genes after Bonferroni correction. The significant GO terms included sensory perception of chemical stimulus or smell, detection of chemical stimulus involved in sensory perception of smell and ATP hydrolysis coupled ion transmembrane transport activity (Table 2). For the KEGG analysis, CNVRs-harbored genes were significantly enriched in one pathway: olfactory transduction (Table 2). In addition, 162 (97.59%) CNVRs were found to overlap with 2,765 previously reported QTLs affecting 378 phenotypic traits related to meat and carcass quality, reproduction, exterior, health and production, etc. (Supplementary Table S5).

## DISCUSSION

We herein detected 166 CNVRs in this breed. These CNVRs covered 15.55 Mb, accounting for 0.58% of the pig autosomal genome, a proportion larger than 0.33% reported in highly inbred Iberian pigs using the 60k BeadChip SNP data [27], but within the range of 0.33% to 6.14% CNVR coverage as previously reported in other pig populations [28–30]. The different genome coverage and imperfect overlap of CNVRs among different studies may be due to several factors, such as sample size, genetic background, CNV calling platforms and algorithms, and filtering criteria. In this study, we detected 72 novel CNVRs, which could be attributed to the high-density SNP markers (n = 566,795) with a small average distance (3.08 Kb) among adjacent SNPs on the customized Axiom Pig1.4M array plate.

It has been shown that CNVs tend to occur near telomeres or centromeres and some genomic regions are particularly apt to structural rearrangements that create CNV hotspots [31]. We herein identified two or more genes in each of 30 CNVRs, one in each of 47 CNVRs, and none in 89 CNVRs (Supplementary Table S4), suggesting that CNVs are preferably located in gene-poor regions in pigs [32]. We further showed that CNVR-related genes were significantly enriched in GO terms related to sensory perception of chemical stimulus or smell and ATP hydrolysis coupled ion transmembrane transport. These genes were also overrepresented in the olfactory transduction KEGG pathway. Our finding is consistent with previous reports that most of CNVR-overlapped genes are related to the olfactory receptors in pigs [8,14,33].

A total of 162 (97.59%) CNVRs resided in 2,765 reported QTLs affecting 378 phenotypic traits. These QTLs mainly affect several economically important traits, such as meat and carcass quality, reproduction, exterior, health and production. We note that CNVR30 overlap with a QTL for mycoplasma hyopneumoniae antibody titer. This CNVR comprises the cytochrome P450 4F2 gene that may play a key role in swine mycoplasma pneumoniae susceptibility. Mycoplasma pneumonia of swine (MPS) is a chronic and endemic respiratory disease caused by *Mycoplasma hyopneumoniae* and inflicts significant economic loss in swine industry [34]. There are obvious sensitivity differences among various pig breeds. For instance, compared with Western modern breeds, Chinese local pig breeds have a more intense immune response and a higher antibody level after infection with mycoplasma pneumonia [35]. Cytochrome P450 enzymes is known to mediate the suppression of inflammatory response caused by *Mycoplasma hyopneumoniae* (MPS) via peroxisome proliferator activated receptor gamma signal pathway in pigs [36]. Therefore, we assume that CNVR30 may have effects on immunity and resistance to MPS in Dongxiang spotted pigs. It is deserved to be mentioned that 72 unique CNVRs were identified in Dongxiang spotted pigs. These CNVRs correspond to 32 QTLs affecting meat and carcass quality, immunity function and reproduction traits (Supplementary Table S6). CNVR65 is located in multiple overlapped QTLs for carcass quality and harbors the thyrotropin releasing hormone degrading enzyme gene related to growth and meat production traits in sheep [37,38]. Moreover, CNVR100 overlaps with QTLs for reproduction traits including litter size, teat number and uterine capacity. Lymphoid enhancer binding factor 1 (*LEF1*), one gene associated with teat number [39], falls in CNVR100. *LEF1* belonging to T-cell specific factor family, a small family in vertebrates including four members. As a major transcription factor of wingless signaling (Wnt), *LEF1* mediates β-catenin that binds to DNA through a high conserved sequence CTTTGT [40]. *LEF1* is an outstanding candidate gene for melanoma susceptibility as it can regulate microphthalmia-associated transcription factor, KIT-ligand (*KITLG*), tyrosinase, and other melanogenic genes [41,42]. *LEF1* has be shown to associate with the development of white/black coat color in mink skin [43]. Dongxiang spotted pigs also have white/black coat colors, and we suspect that *LEF1* CNVR may play a role in the formation of their coloring. Further studies are needed to test this assumption.

We are aware that there are some limits in the current study. First, a larger sample size would have enabled us to identify more CNVs. Second, SNPs in sex chromosomes were discarded. An improved algorithm is required to detect CNVs on the sex chromosomes. Finally, to reduce false-positive findings, we focused on the CNVs containing three or more consecutive SNPs (on average 3.08 kb) and discarded the CNVRs that were presented in only one individual. A proportion of true low-frequency CNVs that comprise sporadic cases may have not been analyzed [44].

In summary, we used a customized Affymetrix Axiom Pig1.4M data to uncover 3,871 CNVs and 166 CNVRs from 90 Dongxiang spotted pigs. The 166 CNVRs are unevenly distributed across the pig genome. The GO and KEGG enrichment analyses illustrate that 166 CNVRs are involved in a number of molecular functions especially in olfactory transduction. Of these CNVRs, 94 (56.63%) have been reported in previous studies, whereas 72 (43.37%) CNVRs are identified for the first time. These novel CNVRs improve the catalog of pig CNVs, which would benefit further identification of trait-related CNVs and contribute to selective breeding of Dongxiang spotted pigs and other breeds.

## Supplementary Data













## Figures and Tables

**Table 1:** Comparison of CNVRs identified in this study with those 11 previous studies

Study	Platform	Sample	CNVR	Total length (Mb)	Overlapping	Concordant length (Mb)
Chen et al [8]	60k SNP array	1,693	565	139.9	36	1.18
Wang et al [33]	60k SNP array	14	63	9.98	6	0.27
Paudel et al [26]	NGS	16	3,118	39.2	61	1.48
Wang et al [28]	60k SNP array	302	348	150.5	10	0.28
G. Schiavo et al [45]	60k SNP array	297	170	72.33	10	0.29
Fernandez et al [27]	60k SNP array	223	65	9.68	6	0.33
Dong et al [14]	60k SNP array	96	105	16.71	15	0.54
Wang et al [30]	NGS	49	3,131	42.1	16	0.13
Wang et al [46]	NGS	252	455	11.36	2	0.02
Xie et al [17]	60k SNP array	120	172	80.41	23	0.80
Revilla et al [29]	NGS	7	540	9.65	26	0.35

CNVRs, copy number variation regions; SNP, single nucleotide polymorphism; NGS, next-generation sequencing.

**Table 2:** Go ontology (GO) and Kyoto encyclopedia of genes and genomes (KEGG) pathway analyses of genes in the identified copy number variation regions

SUID	ID	Term	p value	Associated gene (%)	No. gene
62	GO:0007606	Sensory perception of chemical stimulus	1.05E-33	6.57	35
63	GO:0007608	Sensory perception of smell	2.04E-34	7.47	34
66	GO:0009593	Detection of chemical stimulus	6.32E-33	6.67	34
69	GO:0050906	Detection of stimulus involved in sensory perception	7.85E-33	6.53	34
73	GO:0050907	Detection of chemical stimulus involved in sensory perception	5.05E-34	7.25	34
81	GO:0050911	Detection of chemical stimulus involved in sensory perception of smell	2.19E-35	8.00	34
89	GO:0090662	ATP hydrolysis coupled transmembrane transport	3.06E-03	4.17	3
90	GO:0099131	ATP hydrolysis coupled ion transmembrane transport	5.42E-03	4.35	3
93	KEGG:04740	Olfactory transduction	8.94E-33	7.64	32
