# Supplementary material for: Identification of copy number variations using high density whole-genome single nucleotide polymorphism markers in Chinese Dongxiang spotted pigs
Source: Asian-Australas J Anim Sci. 2019 Feb 7;32(12):1809–15. doi: 10.5713/ajas.18.0696 (PMC6819687; doi:10.5713/ajas.18.0696)
Supplement: Supplementary file 1 [file ajas-18-0696-suppl1.pdf]

Table S1. Details of CNVRs identified in this study

CNVR_ID	CNV_ID	Num_ind	Chr.	Start	End	State	Length (bp)
CNVR1	83	2	1	88942731	88998960	gain	56229
CNVR2	88	7	1	91934647	91942954	loss	8307
CNVR3	92	2	1	95316086	95335767	gain	19681
CNVR4	99	4	1	99687218	99704572	gain	17354
CNVR5	101	2	1	99733649	99785971	gain	52322
CNVR6	103	2	1	100019309	100027849	gain	8540
CNVR7	141	2	1	145236440	145265439	gain	28999
CNVR8	156	2	1	170118820	170121900	gain	3080
CNVR9	191	2	1	197269718	197274380	gain	4662
CNVR10	220	2	1	224697068	224765844	gain	68776
CNVR11	255	2	1	259697345	259705356	gain	8011
CNVR12	261	2	1	268054795	268056507	gain	1712
CNVR13	266	2	1	271529620	271536665	gain	7045
CNVR14	279	2	1	284447951	284512355	loss	64404
CNVR15	288	2	1	295235629	295379020	gain	143391
CNVR16	290	2	1	296193901	296202305	loss-gain	8404
CNVR17	292	2	1	296918534	296982162	loss	63628
CNVR18	298	2	1	312363155	312415324	gain	52169
CNVR19	299	3	1	312415324	312437693	gain	22369
CNVR20	312	2	2	11511074	11530117	gain	19043
CNVR21	314	3	2	12364565	12398203	loss	33638
CNVR22	316	4	2	12689951	12700771	loss	10820
CNVR23	319	2	2	14000848	14032642	loss-gain	31794
CNVR24	320	2	2	14728311	14771718	gain	43407
CNVR25	321	2	2	15024071	15060448	loss	36377
CNVR26	343	2	2	44800086	44803691	gain	3605
CNVR27	348	2	2	54685639	54717573	loss-gain	31934
CNVR28	353	3	2	56935884	56944870	loss	8986
CNVR29	355	2	2	58030910	58035496	gain	4586
CNVR30	359	7	2	61357153	61369311	loss	12158

CNVR31	362	2	2	62416028	62428337	gain	12309
CNVR32	364	2	2	62628253	62738661	gain	110408
CNVR33	378	2	2	67915859	67952389	gain	36530
CNVR34	379	2	2	68061173	68181801	gain	120628
CNVR35	401	15	2	95897658	95915484	loss	17826
CNVR36	408	3	2	96833054	96835454	loss	2400
CNVR37	422	2	2	107437562	107451870	gain	14308
CNVR38	466	3	2	148904898	148913506	loss-gain	8608
CNVR39	467	6	2	149095217	149101135	loss	5918
CNVR40	471	2	2	156079099	156090392	gain	11293
CNVR41	472	3	2	158544152	158558867	gain	14715
CNVR42	473	2	2	158605784	158612295	gain	6511
CNVR43	473	2	2	158605784	158629757	gain	23973
CNVR44	479	2	2	159094998	159110351	gain	15353
CNVR45	487	2	2	160893564	160895760	gain	2196
CNVR46	504	2	3	45023435	45241189	gain	217754
CNVR47	510	6	3	56589884	56595835	loss	5951
CNVR48	519	3	3	61793403	61809935	loss	16532
CNVR49	524	2	3	67785626	67805349	gain	19723
CNVR50	539	2	3	83892565	83943514	gain	50949
CNVR51	581	5	3	142899740	142925257	loss	25517
CNVR52	582	2	3	142999513	143009221	gain	9708
CNVR53	585	2	3	143590087	143597682	gain	7595
CNVR54	589	2	3	144724140	144783726	gain	59586
CNVR55	639	2	4	52447075	52460832	gain	13757
CNVR56	649	2	4	55682568	55685476	loss	2908
CNVR57	668	2	4	67469665	67474606	loss	4941
CNVR58	683	2	4	88694499	88704809	gain	10310
CNVR59	698	2	4	111405421	111446020	gain	40599
CNVR60	727	2	5	18191073	18199920	gain	8847
CNVR61	730	2	5	21242609	21260003	gain	17394
CNVR62	731	2	5	21332629	21368821	gain	36192
CNVR63	736	2	5	22004036	22012384	loss	8348

CNVR64	739	2	5	22410640	22421100	gain	10460
CNVR65	755	2	5	39192570	39207538	gain	14968
CNVR66	766	2	5	52064895	52069376	loss	4481
CNVR67	800	2	5	79630280	79697936	gain	67656
CNVR68	833	2	6	8471113	8494128	gain	23015
CNVR69	862	2	6	95466297	95473943	gain	7646
CNVR70	871	2	6	107004191	107039849	loss	35658
CNVR71	917	2	6	151994674	152015484	gain	20810
CNVR72	923	2	7	10444634	10478517	gain	33883
CNVR73	934	2	7	22207228	22236111	gain	28883
CNVR74	938	2	7	23307667	23658110	loss-gain	350443
CNVR75	939	2	7	23481211	23658110	loss-gain	176899
CNVR76	942	7	7	24648309	24653067	loss-gain	4758
CNVR77	943	2	7	24735665	24740397	gain	4732
CNVR78	944	2	7	25359190	25399904	gain	40714
CNVR79	945	2	7	25488796	25557156	gain	68360
CNVR80	946	2	7	25785853	25888939	gain	103086
CNVR81	949	2	7	26267062	26297673	gain	30611
CNVR82	951	2	7	26600964	26608495	gain	7531
CNVR83	957	3	7	28525787	28565312	gain	39525
CNVR84	972	2	7	58579811	58584542	gain	4731
CNVR85	975	4	7	59278633	59284219	loss	5586
CNVR86	977	2	7	61355441	61417944	gain	62503
CNVR87	994	2	7	82234623	82374446	gain	139823
CNVR88	1002	2	7	84728780	84749202	gain	20422
CNVR89	1011	2	7	102790067	102823288	gain	33221
CNVR90	1021	8	7	111620166	111623056	loss	2890
CNVR91	1035	3	7	131449863	131460402	gain	10539
CNVR92	1040	3	7	132961208	132975832	loss	14624
CNVR93	1041	2	7	132985953	133003168	loss	17215
CNVR94	1059	3	8	26029928	26034839	loss	4911
CNVR95	1068	3	8	30016900	30024587	loss	7687
CNVR96	1086	5	8	44371710	44447879	gain	76169

CNVR97	1112	2	8	64473379	64515977	loss	42598
CNVR98	1116	2	8	68684763	68710413	gain	25650
CNVR99	1129	2	8	85727251	85735420	gain	8169
CNVR100	1169	3	8	122108843	122121928	loss	13085
CNVR101	1176	2	8	129139072	129153237	loss	14165
CNVR102	1198	2	9	1963636	2010455	gain	46819
CNVR103	1200	4	9	3614907	3618335	gain	3428
CNVR104	1203	4	9	5206397	5225462	gain	19065
CNVR105	1204	2	9	5488035	5492918	gain	4883
CNVR106	1205	2	9	5747061	5810398	loss	63337
CNVR107	1227	2	9	21980507	21992085	gain	11578
CNVR108	1234	5	9	27756709	27768971	gain	12262
CNVR109	1261	3	9	56272660	56332060	gain	59400
CNVR110	1262	2	9	56901872	56950458	loss	48586
CNVR111	1316	3	9	108875949	108885208	gain	9259
CNVR112	1331	2	9	119585370	119593230	gain	7860
CNVR113	1338	2	9	124260883	124271069	gain	10186
CNVR114	1339	2	9	127267298	127291319	gain	24021
CNVR115	1346	2	9	136449280	136453167	gain	3887
CNVR116	1380	2	10	22431073	22524502	gain	93429
CNVR117	1381	4	10	23114909	23120232	loss-gain	5323
CNVR118	1416	2	10	55474331	55489205	gain	14874
CNVR119	1427	6	10	71830224	71835029	gain	4805
CNVR120	1431	2	10	79004773	79065598	gain	60825
CNVR121	1435	2	11	8243425	8246938	loss	3513
CNVR122	1436	2	11	8308242	8320582	gain	12340
CNVR123	1437	2	11	8815180	8845277	loss	30097
CNVR124	1443	3	11	20978853	20984493	loss	5640
CNVR125	1457	4	11	32658621	32711857	loss	53236
CNVR126	1468	2	11	38006863	38074356	gain	67493
CNVR127	1505	2	11	61453183	61459945	gain	6762
CNVR128	1514	3	11	69030920	69069509	loss	38589
CNVR129	1518	2	11	70689891	70709813	loss	19922

CNVR130	1520	2	11	71317528	71375741	loss	58213
CNVR131	1524	2	11	71878763	71887995	gain	9232
CNVR132	1555	2	12	50924636	50947287	gain	22651
CNVR133	1556	2	12	51099806	51123003	gain	23197
CNVR134	1573	2	13	16191826	16221816	gain	29990
CNVR135	1578	2	13	19139627	19152872	gain	13245
CNVR136	1589	2	13	30640874	30643949	gain	3075
CNVR137	1593	2	13	40598705	40614627	gain	15922
CNVR138	1636	2	13	111201690	111225859	gain	24169
CNVR139	1656	2	13	131028348	131044332	gain	15984
CNVR140	1690	2	13	164895338	164898801	gain	3463
CNVR141	1693	2	13	166109149	166114547	gain	5398
CNVR142	1695	4	13	167567440	167600641	gain	33201
CNVR143	1699	2	13	170765271	170819670	gain	54399
CNVR144	1702	2	13	171285806	171311772	gain	25966
CNVR145	1777	2	14	2401741	2423505	gain	21764
CNVR146	1781	2	14	7794851	7822418	gain	27567
CNVR147	1782	3	14	8039487	8116098	gain	76611
CNVR148	1792	2	14	21068045	21091201	gain	23156
CNVR149	1816	5	14	56382005	56403580	loss	21575
CNVR150	1820	3	14	65589945	65600023	loss	10078
CNVR151	1829	2	14	74107606	74126308	loss	18702
CNVR152	1838	2	14	81377028	81553876	gain	176848
CNVR153	1849	2	14	94594494	94629904	gain	35410
CNVR154	1873	2	14	120810562	120829324	gain	18762
CNVR155	1880	2	14	127501895	127526173	gain	24278
CNVR156	1968	2	15	82278814	82319824	gain	41010
CNVR157	1990	3	15	112634645	112649752	loss	15107
CNVR158	2026	2	15	156637663	156646003	gain	8340
CNVR159	2034	2	16	6146177	6154424	loss	8247
CNVR160	2055	2	16	12229596	12241067	loss-gain	11471
CNVR161	2084	2	16	30196586	30215798	loss	19212
CNVR162	2084	2	16	30196586	30217974	loss	21388

CNVR163	2109	2	16	53083153	53202189	gain	119036
CNVR164	2111	8	16	56657347	56707690	loss	50343
CNVR165	2136	2	16	85295667	85298564	loss	2897
CNVR166	2168	2	17	53147051	53158077	gain	11026
