# Supplementary material for: Identification of copy number variations using high density whole-genome single nucleotide polymorphism markers in Chinese Dongxiang spotted pigs
Source: Asian-Australas J Anim Sci. 2019 Feb 7;32(12):1809–15. doi: 10.5713/ajas.18.0696 (PMC6819687; doi:10.5713/ajas.18.0696)
Supplement: Supplementary file 2 [file ajas-18-0696-suppl2.pdf]

**Table S2. Validation of the identified CNVRs by whole-genome sequencing**

| CNVRs identified using Affymetrix Axion Pig1.4M array plates | | | | | CNV identified using whole-genome sequencing | | | | Relationship | |
|---|---|---|---|---|---|---|---|---|---|---|
| CNVR_ID | Chr. | Start1 | End1 | Type | CNV_ID | Start2 | End2 | Type | Relationship | Overlap Length (bp) |
| 83 | 1 | 88942731 | 88998960 | gain | 1000 | 88947401 | 88948400 | DEL | region2inRegion1 | 999 |
| 92 | 1 | 95316086 | 95335767 | gain | 2000 | 95317601 | 95319600 | DEL | region2inRegion1 | 1999 |
| 92 | 1 | 95316086 | 95335767 | gain | 1200 | 95321401 | 95322600 | DEL | region2inRegion1 | 1199 |
| 92 | 1 | 95316086 | 95335767 | gain | 1000 | 95325401 | 95326400 | DEL | region2inRegion1 | 999 |
| 92 | 1 | 95316086 | 95335767 | gain | 2600 | 95333401 | 95336000 | DEL | overlapTail | 2367 |
| 99 | 1 | 99687218 | 99704572 | gain | 800 | 99693601 | 99694400 | DUP | region2inRegion1 | 799 |
| 99 | 1 | 99687218 | 99704572 | gain | 800 | 99696201 | 99697000 | DUP | region2inRegion1 | 799 |
| 99 | 1 | 99687218 | 99704572 | gain | 1400 | 99700601 | 99702000 | DUP | region2inRegion1 | 1399 |
| 101 | 1 | 99733649 | 99785971 | gain | 800 | 99736401 | 99737200 | DUP | region2inRegion1 | 799 |
| 101 | 1 | 99733649 | 99785971 | gain | 1000 | 99743601 | 99744600 | DUP | region2inRegion1 | 999 |
| 101 | 1 | 99733649 | 99785971 | gain | 1000 | 99745201 | 99746200 | DUP | region2inRegion1 | 999 |
| 101 | 1 | 99733649 | 99785971 | gain | 1200 | 99748201 | 99749400 | DUP | region2inRegion1 | 1199 |
| 101 | 1 | 99733649 | 99785971 | gain | 1000 | 99758201 | 99759200 | DUP | region2inRegion1 | 999 |
| 101 | 1 | 99733649 | 99785971 | gain | 1000 | 99771601 | 99772600 | DUP | region2inRegion1 | 999 |
| 101 | 1 | 99733649 | 99785971 | gain | 1600 | 99773801 | 99775400 | DUP | region2inRegion1 | 1599 |
| 101 | 1 | 99733649 | 99785971 | gain | 800 | 99777201 | 99778000 | DUP | region2inRegion1 | 799 |

| | | | | | | | | | | |
|---|---|---|---|---|---|---|---|---|---|---|
| 101 | 1 | 99733649 | 99785971 | gain | 800 | 99778001 | 99778800 | DUP | region2inRegion1 | 799 |
| 101 | 1 | 99733649 | 99785971 | gain | 1000 | 99779601 | 99780600 | DUP | region2inRegion1 | 999 |
| 101 | 1 | 99733649 | 99785971 | gain | 800 | 99781001 | 99781800 | DUP | region2inRegion1 | 799 |
| 101 | 1 | 99733649 | 99785971 | gain | 1000 | 99782201 | 99783200 | DUP | region2inRegion1 | 999 |
| 141 | 1 | 145236440 | 145265439 | gain | 1000 | 145246601 | 145247600 | DEL | region2inRegion1 | 999 |
| 220 | 1 | 224697068 | 224765844 | gain | 800 | 224699401 | 224700200 | DUP | region2inRegion1 | 799 |
| 220 | 1 | 224697068 | 224765844 | gain | 1000 | 224701001 | 224702000 | DUP | region2inRegion1 | 999 |
| 220 | 1 | 224697068 | 224765844 | gain | 800 | 224707401 | 224708200 | DUP | region2inRegion1 | 799 |
| 220 | 1 | 224697068 | 224765844 | gain | 1600 | 224709001 | 224710600 | DUP | region2inRegion1 | 1599 |
| 220 | 1 | 224697068 | 224765844 | gain | 800 | 224710801 | 224711600 | DUP | region2inRegion1 | 799 |
| 220 | 1 | 224697068 | 224765844 | gain | 1400 | 224712401 | 224713800 | DUP | region2inRegion1 | 1399 |
| 220 | 1 | 224697068 | 224765844 | gain | 800 | 224715201 | 224716000 | DUP | region2inRegion1 | 799 |
| 220 | 1 | 224697068 | 224765844 | gain | 2000 | 224717401 | 224719400 | DUP | region2inRegion1 | 1999 |
| 220 | 1 | 224697068 | 224765844 | gain | 1200 | 224720201 | 224721400 | DUP | region2inRegion1 | 1199 |
| 220 | 1 | 224697068 | 224765844 | gain | 1000 | 224728201 | 224729200 | DUP | region2inRegion1 | 999 |
| 220 | 1 | 224697068 | 224765844 | gain | 1800 | 224731401 | 224733200 | DUP | region2inRegion1 | 1799 |
| 220 | 1 | 224697068 | 224765844 | gain | 1200 | 224733601 | 224734800 | DUP | region2inRegion1 | 1199 |
| 220 | 1 | 224697068 | 224765844 | gain | 1600 | 224736001 | 224737600 | DUP | region2inRegion1 | 1599 |
| 220 | 1 | 224697068 | 224765844 | gain | 800 | 224739801 | 224740600 | DUP | region2inRegion1 | 799 |

| | | | | | | | | | | | |
|---|---|---|---|---|---|---|---|---|---|---|---|
| 220 | 1 | 224697068 | 224765844 | gain | 800 | 224742401 | 224743200 | DUP | region2inRegion1 | 799 |
| 220 | 1 | 224697068 | 224765844 | gain | 1000 | 224743401 | 224744400 | DUP | region2inRegion1 | 999 |
| 220 | 1 | 224697068 | 224765844 | gain | 800 | 224745801 | 224746600 | DUP | region2inRegion1 | 799 |
| 220 | 1 | 224697068 | 224765844 | gain | 1200 | 224748001 | 224749200 | DUP | region2inRegion1 | 1199 |
| 220 | 1 | 224697068 | 224765844 | gain | 1000 | 224750001 | 224751000 | DUP | region2inRegion1 | 999 |
| 220 | 1 | 224697068 | 224765844 | gain | 800 | 224751201 | 224752000 | DUP | region2inRegion1 | 799 |
| 220 | 1 | 224697068 | 224765844 | gain | 1400 | 224752201 | 224753600 | DUP | region2inRegion1 | 1399 |
| 220 | 1 | 224697068 | 224765844 | gain | 800 | 224753801 | 224754600 | DUP | region2inRegion1 | 799 |
| 220 | 1 | 224697068 | 224765844 | gain | 1000 | 224754801 | 224755800 | DUP | region2inRegion1 | 999 |
| 220 | 1 | 224697068 | 224765844 | gain | 800 | 224756201 | 224757000 | DUP | region2inRegion1 | 799 |
| 220 | 1 | 224697068 | 224765844 | gain | 800 | 224758001 | 224758800 | DUP | region2inRegion1 | 799 |
| 220 | 1 | 224697068 | 224765844 | gain | 1000 | 224759201 | 224760200 | DUP | region2inRegion1 | 999 |
| 220 | 1 | 224697068 | 224765844 | gain | 1200 | 224760401 | 224761600 | DUP | region2inRegion1 | 1199 |
| 220 | 1 | 224697068 | 224765844 | gain | 1400 | 224763201 | 224764600 | DUP | region2inRegion1 | 1399 |
| 266 | 1 | 271529620 | 271536665 | gain | 60800 | 271530401 | 271591200 | DUP | overlapTail | 6265 |
| 279 | 1 | 284447951 | 284512355 | loss | 1600 | 284447601 | 284449200 | DUP | overlapHead | 1250 |
| 279 | 1 | 284447951 | 284512355 | loss | 1400 | 284455601 | 284457000 | DUP | region2inRegion1 | 1399 |
| 279 | 1 | 284447951 | 284512355 | loss | 1600 | 284457401 | 284459000 | DUP | region2inRegion1 | 1599 |
| 279 | 1 | 284447951 | 284512355 | loss | 800 | 284459601 | 284460400 | DUP | region2inRegion1 | 799 |

| | | | | | | | | | | |
|---|---|---|---|---|---|---|---|---|---|---|
| 279 | 1 | 284447951 | 284512355 | loss | 1200 | 284462601 | 284463800 | DUP | region2inRegion1 | 1199 |
| 279 | 1 | 284447951 | 284512355 | loss | 800 | 284464201 | 284465000 | DUP | region2inRegion1 | 799 |
| 279 | 1 | 284447951 | 284512355 | loss | 1600 | 284465401 | 284467000 | DUP | region2inRegion1 | 1599 |
| 279 | 1 | 284447951 | 284512355 | loss | 1600 | 284471801 | 284473400 | DUP | region2inRegion1 | 1599 |
| 279 | 1 | 284447951 | 284512355 | loss | 1400 | 284473801 | 284475200 | DUP | region2inRegion1 | 1399 |
| 279 | 1 | 284447951 | 284512355 | loss | 1200 | 284475401 | 284476600 | DUP | region2inRegion1 | 1199 |
| 279 | 1 | 284447951 | 284512355 | loss | 1600 | 284477601 | 284479200 | DUP | region2inRegion1 | 1599 |
| 279 | 1 | 284447951 | 284512355 | loss | 800 | 284480201 | 284481000 | DUP | region2inRegion1 | 799 |
| 279 | 1 | 284447951 | 284512355 | loss | 1200 | 284481601 | 284482800 | DUP | region2inRegion1 | 1199 |
| 279 | 1 | 284447951 | 284512355 | loss | 1000 | 284483601 | 284484600 | DUP | region2inRegion1 | 999 |
| 279 | 1 | 284447951 | 284512355 | loss | 1000 | 284485401 | 284486400 | DUP | region2inRegion1 | 999 |
| 279 | 1 | 284447951 | 284512355 | loss | 1000 | 284489001 | 284490000 | DUP | region2inRegion1 | 999 |
| 279 | 1 | 284447951 | 284512355 | loss | 800 | 284492401 | 284493200 | DUP | region2inRegion1 | 799 |
| 279 | 1 | 284447951 | 284512355 | loss | 1200 | 284493401 | 284494600 | DUP | region2inRegion1 | 1199 |
| 279 | 1 | 284447951 | 284512355 | loss | 1400 | 284496401 | 284497800 | DUP | region2inRegion1 | 1399 |
| 279 | 1 | 284447951 | 284512355 | loss | 800 | 284502001 | 284502800 | DUP | region2inRegion1 | 799 |
| 279 | 1 | 284447951 | 284512355 | loss | 1400 | 284504401 | 284505800 | DUP | region2inRegion1 | 1399 |
| 279 | 1 | 284447951 | 284512355 | loss | 800 | 284506401 | 284507200 | DUP | region2inRegion1 | 799 |
| 279 | 1 | 284447951 | 284512355 | loss | 1000 | 284507801 | 284508800 | DUP | region2inRegion1 | 999 |

| | | | | | | | | | | |
|---|---|---|---|---|---|---|---|---|---|---|
| 279 | 1 | 284447951 | 284512355 | loss | 1400 | 284510601 | 284512000 | DUP | region2inRegion1 | 1399 |
| 288 | 1 | 295235629 | 295379020 | gain | 2000 | 295237601 | 295239600 | DUP | region2inRegion1 | 1999 |
| 288 | 1 | 295235629 | 295379020 | gain | 800 | 295241601 | 295242400 | DUP | region2inRegion1 | 799 |
| 288 | 1 | 295235629 | 295379020 | gain | 1000 | 295243401 | 295244400 | DUP | region2inRegion1 | 999 |
| 288 | 1 | 295235629 | 295379020 | gain | 1600 | 295245201 | 295246800 | DUP | region2inRegion1 | 1599 |
| 288 | 1 | 295235629 | 295379020 | gain | 1200 | 295250001 | 295251200 | DUP | region2inRegion1 | 1199 |
| 288 | 1 | 295235629 | 295379020 | gain | 800 | 295255401 | 295256200 | DUP | region2inRegion1 | 799 |
| 288 | 1 | 295235629 | 295379020 | gain | 1000 | 295266201 | 295267200 | DEL | region2inRegion1 | 999 |
| 288 | 1 | 295235629 | 295379020 | gain | 800 | 295274001 | 295274800 | DUP | region2inRegion1 | 799 |
| 288 | 1 | 295235629 | 295379020 | gain | 3200 | 295284801 | 295288000 | DUP | region2inRegion1 | 3199 |
| 288 | 1 | 295235629 | 295379020 | gain | 1800 | 295290601 | 295292400 | DUP | region2inRegion1 | 1799 |
| 288 | 1 | 295235629 | 295379020 | gain | 1000 | 295303601 | 295304600 | DUP | region2inRegion1 | 999 |
| 288 | 1 | 295235629 | 295379020 | gain | 1600 | 295304801 | 295306400 | DUP | region2inRegion1 | 1599 |
| 288 | 1 | 295235629 | 295379020 | gain | 4000 | 295362801 | 295366800 | DUP | region2inRegion1 | 3999 |
| 290 | 1 | 296193901 | 296202305 | loss-gain | 800 | 296199601 | 296200400 | DUP | region2inRegion1 | 799 |
| 290 | 1 | 296193901 | 296202305 | loss-gain | 1200 | 296201601 | 296202800 | DEL | overlapTail | 705 |
| 292 | 1 | 296918534 | 296982162 | loss | 800 | 296923001 | 296923800 | DUP | region2inRegion1 | 799 |
| 292 | 1 | 296918534 | 296982162 | loss | 1000 | 296927601 | 296928600 | DUP | region2inRegion1 | 999 |
| 292 | 1 | 296918534 | 296982162 | loss | 1200 | 296932401 | 296933600 | DUP | region2inRegion1 | 1199 |

| | | | | | | | | | | |
|---|---|---|---|---|---|---|---|---|---|---|
| 292 | 1 | 296918534 | 296982162 | loss | 800 | 296935801 | 296936600 | DUP | region2inRegion1 | 799 |
| 292 | 1 | 296918534 | 296982162 | loss | 1400 | 296936801 | 296938200 | DUP | region2inRegion1 | 1399 |
| 292 | 1 | 296918534 | 296982162 | loss | 800 | 296949001 | 296949800 | DUP | region2inRegion1 | 799 |
| 292 | 1 | 296918534 | 296982162 | loss | 800 | 296951401 | 296952200 | DUP | region2inRegion1 | 799 |
| 292 | 1 | 296918534 | 296982162 | loss | 1200 | 296961001 | 296962200 | DUP | region2inRegion1 | 1199 |
| 292 | 1 | 296918534 | 296982162 | loss | 1000 | 296965201 | 296966200 | DUP | region2inRegion1 | 999 |
| 292 | 1 | 296918534 | 296982162 | loss | 1000 | 296966801 | 296967800 | DEL | region2inRegion1 | 999 |
| 292 | 1 | 296918534 | 296982162 | loss | 1000 | 296972401 | 296973400 | DEL | region2inRegion1 | 999 |
| 292 | 1 | 296918534 | 296982162 | loss | 800 | 296980801 | 296981600 | DUP | region2inRegion1 | 799 |
| 298 | 1 | 312363155 | 312415324 | gain | 114000 | 312288001 | 312402000 | DUP | overlapHead | 38846 |
| 298 | 1 | 312363155 | 312415324 | gain | 1200 | 312402601 | 312403800 | DUP | region2inRegion1 | 1199 |
| 298 | 1 | 312363155 | 312415324 | gain | 1400 | 312403601 | 312405000 | DEL | region2inRegion1 | 1399 |
| 298 | 1 | 312363155 | 312415324 | gain | 1600 | 312407801 | 312409400 | DUP | region2inRegion1 | 1599 |
| 298 | 1 | 312363155 | 312415324 | gain | 1600 | 312415001 | 312416600 | DEL | overlapTail | 324 |
| 299 | 1 | 312415324 | 312437693 | gain | 1600 | 312415001 | 312416600 | DEL | overlapHead | 1277 |
| 299 | 1 | 312415324 | 312437693 | gain | 1400 | 312417401 | 312418800 | DUP | region2inRegion1 | 1399 |
| 299 | 1 | 312415324 | 312437693 | gain | 1000 | 312421601 | 312422600 | DUP | region2inRegion1 | 999 |
| 299 | 1 | 312415324 | 312437693 | gain | 2400 | 312425201 | 312427600 | DUP | region2inRegion1 | 2399 |
| 299 | 1 | 312415324 | 312437693 | gain | 1200 | 312436001 | 312437200 | DUP | region2inRegion1 | 1199 |

| | | | | | | | | | | | |
|---|---|---|---|---|---|---|---|---|---|---|---|
| 1380 | 10 | 22431073 | 22524502 | gain | 23600 | 22414201 | 22437800 | DUP | overlapHead | 6728 |
| 1380 | 10 | 22431073 | 22524502 | gain | 2400 | 22438001 | 22440400 | DUP | region2inRegion1 | 2399 |
| 1380 | 10 | 22431073 | 22524502 | gain | 6200 | 22440601 | 22446800 | DUP | region2inRegion1 | 6199 |
| 1380 | 10 | 22431073 | 22524502 | gain | 800 | 22449001 | 22449800 | DUP | region2inRegion1 | 799 |
| 1380 | 10 | 22431073 | 22524502 | gain | 1400 | 22450401 | 22451800 | DUP | region2inRegion1 | 1399 |
| 1380 | 10 | 22431073 | 22524502 | gain | 800 | 22453401 | 22454200 | DUP | region2inRegion1 | 799 |
| 1380 | 10 | 22431073 | 22524502 | gain | 1400 | 22454601 | 22456000 | DUP | region2inRegion1 | 1399 |
| 1380 | 10 | 22431073 | 22524502 | gain | 1200 | 22456401 | 22457600 | DUP | region2inRegion1 | 1199 |
| 1380 | 10 | 22431073 | 22524502 | gain | 3800 | 22458801 | 22462600 | DUP | region2inRegion1 | 3799 |
| 1380 | 10 | 22431073 | 22524502 | gain | 1200 | 22464401 | 22465600 | DUP | region2inRegion1 | 1199 |
| 1380 | 10 | 22431073 | 22524502 | gain | 1400 | 22467401 | 22468800 | DUP | region2inRegion1 | 1399 |
| 1380 | 10 | 22431073 | 22524502 | gain | 1000 | 22471801 | 22472800 | DUP | region2inRegion1 | 999 |
| 1380 | 10 | 22431073 | 22524502 | gain | 1000 | 22473801 | 22474800 | DUP | region2inRegion1 | 999 |
| 1380 | 10 | 22431073 | 22524502 | gain | 1400 | 22476201 | 22477600 | DUP | region2inRegion1 | 1399 |
| 1380 | 10 | 22431073 | 22524502 | gain | 1200 | 22478801 | 22480000 | DUP | region2inRegion1 | 1199 |
| 1380 | 10 | 22431073 | 22524502 | gain | 1200 | 22481401 | 22482600 | DUP | region2inRegion1 | 1199 |
| 1380 | 10 | 22431073 | 22524502 | gain | 3200 | 22483601 | 22486800 | DUP | region2inRegion1 | 3199 |
| 1380 | 10 | 22431073 | 22524502 | gain | 800 | 22487201 | 22488000 | DUP | region2inRegion1 | 799 |
| 1380 | 10 | 22431073 | 22524502 | gain | 800 | 22488401 | 22489200 | DUP | region2inRegion1 | 799 |

| | | | | | | | | | | |
|---|---|---|---|---|---|---|---|---|---|---|
| 1380 | 10 | 22431073 | 22524502 | gain | 800 | 22489801 | 22490600 | DUP | region2inRegion1 | 799 |
| 1380 | 10 | 22431073 | 22524502 | gain | 1800 | 22490601 | 22492400 | DUP | region2inRegion1 | 1799 |
| 1380 | 10 | 22431073 | 22524502 | gain | 1000 | 22493001 | 22494000 | DUP | region2inRegion1 | 999 |
| 1380 | 10 | 22431073 | 22524502 | gain | 3000 | 22494601 | 22497600 | DUP | region2inRegion1 | 2999 |
| 1380 | 10 | 22431073 | 22524502 | gain | 800 | 22498201 | 22499000 | DUP | region2inRegion1 | 799 |
| 1380 | 10 | 22431073 | 22524502 | gain | 1200 | 22499401 | 22500600 | DUP | region2inRegion1 | 1199 |
| 1380 | 10 | 22431073 | 22524502 | gain | 1000 | 22506001 | 22507000 | DUP | region2inRegion1 | 999 |
| 1380 | 10 | 22431073 | 22524502 | gain | 1000 | 22510601 | 22511600 | DUP | region2inRegion1 | 999 |
| 1380 | 10 | 22431073 | 22524502 | gain | 3400 | 22514601 | 22518000 | DUP | region2inRegion1 | 3399 |
| 1380 | 10 | 22431073 | 22524502 | gain | 4200 | 22519201 | 22523400 | DUP | region2inRegion1 | 4199 |
| 1380 | 10 | 22431073 | 22524502 | gain | 1400 | 22523801 | 22525200 | DUP | overlapTail | 702 |
| 1381 | 10 | 23114909 | 23120232 | loss-gain | 1400 | 23114601 | 23116000 | DUP | overlapHead | 1092 |
| 1381 | 10 | 23114909 | 23120232 | loss-gain | 800 | 23116401 | 23117200 | DUP | region2inRegion1 | 799 |
| 1381 | 10 | 23114909 | 23120232 | loss-gain | 1000 | 23119601 | 23120600 | DUP | overlapTail | 632 |
| 1416 | 10 | 55474331 | 55489205 | gain | 1000 | 55482401 | 55483400 | DUP | region2inRegion1 | 999 |
| 1427 | 10 | 71830224 | 71835029 | gain | 1800 | 71829001 | 71830800 | DUP | overlapHead | 577 |
| 1427 | 10 | 71830224 | 71835029 | gain | 5200 | 71831801 | 71837000 | DUP | overlapTail | 3229 |
| 1431 | 10 | 79004773 | 79065598 | gain | 3400 | 79004001 | 79007400 | DUP | overlapHead | 2628 |
| 1431 | 10 | 79004773 | 79065598 | gain | 5000 | 79008801 | 79013800 | DUP | region2inRegion1 | 4999 |

| | | | | | | | | | | |
|---|---|---|---|---|---|---|---|---|---|---|
| 1431 | 10 | 79004773 | 79065598 | gain | 2400 | 79014801 | 79017200 | DUP | region2inRegion1 | 2399 |
| 1431 | 10 | 79004773 | 79065598 | gain | 1200 | 79018401 | 79019600 | DUP | region2inRegion1 | 1199 |
| 1431 | 10 | 79004773 | 79065598 | gain | 5600 | 79020201 | 79025800 | DUP | region2inRegion1 | 5599 |
| 1431 | 10 | 79004773 | 79065598 | gain | 1200 | 79026201 | 79027400 | DUP | region2inRegion1 | 1199 |
| 1431 | 10 | 79004773 | 79065598 | gain | 1000 | 79029201 | 79030200 | DUP | region2inRegion1 | 999 |
| 1431 | 10 | 79004773 | 79065598 | gain | 2200 | 79030801 | 79033000 | DUP | region2inRegion1 | 2199 |
| 1431 | 10 | 79004773 | 79065598 | gain | 800 | 79033401 | 79034200 | DUP | region2inRegion1 | 799 |
| 1431 | 10 | 79004773 | 79065598 | gain | 1000 | 79035201 | 79036200 | DUP | region2inRegion1 | 999 |
| 1431 | 10 | 79004773 | 79065598 | gain | 2200 | 79036401 | 79038600 | DUP | region2inRegion1 | 2199 |
| 1431 | 10 | 79004773 | 79065598 | gain | 3400 | 79039801 | 79043200 | DUP | region2inRegion1 | 3399 |
| 1431 | 10 | 79004773 | 79065598 | gain | 1200 | 79044001 | 79045200 | DUP | region2inRegion1 | 1199 |
| 1431 | 10 | 79004773 | 79065598 | gain | 1000 | 79047401 | 79048400 | DUP | region2inRegion1 | 999 |
| 1431 | 10 | 79004773 | 79065598 | gain | 1200 | 79048601 | 79049800 | DUP | region2inRegion1 | 1199 |
| 1431 | 10 | 79004773 | 79065598 | gain | 2000 | 79050201 | 79052200 | DUP | region2inRegion1 | 1999 |
| 1431 | 10 | 79004773 | 79065598 | gain | 1800 | 79052601 | 79054400 | DUP | region2inRegion1 | 1799 |
| 1431 | 10 | 79004773 | 79065598 | gain | 1200 | 79054801 | 79056000 | DUP | region2inRegion1 | 1199 |
| 1431 | 10 | 79004773 | 79065598 | gain | 7600 | 79057001 | 79064600 | DUP | region2inRegion1 | 7599 |
| 1431 | 10 | 79004773 | 79065598 | gain | 1600 | 79065401 | 79067000 | DUP | overlapTail | 198 |
| 1437 | 11 | 8815180 | 8845277 | loss | 1200 | 8820201 | 8821400 | DUP | region2inRegion1 | 1199 |

| | | | | | | | | | | | |
|---|---|---|---|---|---|---|---|---|---|---|---|
| 1443 | 11 | 20978853 | 20984493 | loss | 1000 | 20978201 | 20979200 | DEL | overlapHead | 348 |
| 1443 | 11 | 20978853 | 20984493 | loss | 1200 | 20982201 | 20983400 | DEL | region2inRegion1 | 1199 |
| 1457 | 11 | 32658621 | 32711857 | loss | 1200 | 32669801 | 32671000 | DEL | region2inRegion1 | 1199 |
| 1457 | 11 | 32658621 | 32711857 | loss | 1000 | 32672801 | 32673800 | DEL | region2inRegion1 | 999 |
| 1457 | 11 | 32658621 | 32711857 | loss | 1600 | 32693201 | 32694800 | DEL | region2inRegion1 | 1599 |
| 1457 | 11 | 32658621 | 32711857 | loss | 1000 | 32703601 | 32704600 | DEL | region2inRegion1 | 999 |
| 1457 | 11 | 32658621 | 32711857 | loss | 800 | 32707601 | 32708400 | DEL | region2inRegion1 | 799 |
| 1468 | 11 | 38006863 | 38074356 | gain | 1000 | 38014001 | 38015000 | DUP | region2inRegion1 | 999 |
| 1468 | 11 | 38006863 | 38074356 | gain | 1000 | 38028601 | 38029600 | DUP | region2inRegion1 | 999 |
| 1468 | 11 | 38006863 | 38074356 | gain | 1200 | 38032001 | 38033200 | DUP | region2inRegion1 | 1199 |
| 1468 | 11 | 38006863 | 38074356 | gain | 1200 | 38039001 | 38040200 | DUP | region2inRegion1 | 1199 |
| 1505 | 11 | 61453183 | 61459945 | gain | 1000 | 61453001 | 61454000 | DUP | overlapHead | 818 |
| 1514 | 11 | 69030920 | 69069509 | loss | 1000 | 69042001 | 69043000 | DEL | region2inRegion1 | 999 |
| 1514 | 11 | 69030920 | 69069509 | loss | 1000 | 69052601 | 69053600 | DEL | region2inRegion1 | 999 |
| 1514 | 11 | 69030920 | 69069509 | loss | 1400 | 69062601 | 69064000 | DEL | region2inRegion1 | 1399 |
| 1518 | 11 | 70689891 | 70709813 | loss | 1000 | 70695201 | 70696200 | DEL | region2inRegion1 | 999 |
| 1518 | 11 | 70689891 | 70709813 | loss | 1400 | 70701401 | 70702800 | DEL | region2inRegion1 | 1399 |
| 1518 | 11 | 70689891 | 70709813 | loss | 1000 | 70706001 | 70707000 | DUP | region2inRegion1 | 999 |
| 1520 | 11 | 71317528 | 71375741 | loss | 2000 | 71317001 | 71319000 | DEL | overlapHead | 1473 |

| | | | | | | | | | | | |
|---|---|---|---|---|---|---|---|---|---|---|---|
| 1520 | 11 | 71317528 | 71375741 | loss | 800 | 71321001 | 71321800 | DEL | region2inRegion1 | 799 |
| 1520 | 11 | 71317528 | 71375741 | loss | 800 | 71329801 | 71330600 | DUP | region2inRegion1 | 799 |
| 1520 | 11 | 71317528 | 71375741 | loss | 1000 | 71333401 | 71334400 | DEL | region2inRegion1 | 999 |
| 1520 | 11 | 71317528 | 71375741 | loss | 1800 | 71335801 | 71337600 | DEL | region2inRegion1 | 1799 |
| 1520 | 11 | 71317528 | 71375741 | loss | 800 | 71340401 | 71341200 | DUP | region2inRegion1 | 799 |
| 1520 | 11 | 71317528 | 71375741 | loss | 1600 | 71349201 | 71350800 | DUP | region2inRegion1 | 1599 |
| 1520 | 11 | 71317528 | 71375741 | loss | 1400 | 71351601 | 71353000 | DEL | region2inRegion1 | 1399 |
| 1520 | 11 | 71317528 | 71375741 | loss | 1400 | 71354401 | 71355800 | DUP | region2inRegion1 | 1399 |
| 1520 | 11 | 71317528 | 71375741 | loss | 1000 | 71356201 | 71357200 | DUP | region2inRegion1 | 999 |
| 1520 | 11 | 71317528 | 71375741 | loss | 2200 | 71367001 | 71369200 | DUP | region2inRegion1 | 2199 |
| 1520 | 11 | 71317528 | 71375741 | loss | 1400 | 71373401 | 71374800 | DUP | region2inRegion1 | 1399 |
| 1520 | 11 | 71317528 | 71375741 | loss | 1400 | 71375201 | 71376600 | DUP | overlapTail | 541 |
| 1555 | 12 | 50924636 | 50947287 | gain | 78800 | 50876201 | 50955000 | DUP | region1inRegion2 | 22651 |
| 1556 | 12 | 51099806 | 51123003 | gain | 32200 | 51097401 | 51129600 | DUP | region1inRegion2 | 23197 |
| 1573 | 13 | 16191826 | 16221816 | gain | 1000 | 16213801 | 16214800 | DUP | region2inRegion1 | 999 |
| 1589 | 13 | 30640874 | 30643949 | gain | 12600 | 30632601 | 30645200 | DUP | region1inRegion2 | 3075 |
| 1593 | 13 | 40598705 | 40614627 | gain | 1000 | 40600801 | 40601800 | DUP | region2inRegion1 | 999 |
| 1636 | 13 | 111201690 | 111225859 | gain | 1200 | 111202601 | 111203800 | DEL | region2inRegion1 | 1199 |
| 1636 | 13 | 111201690 | 111225859 | gain | 800 | 111207601 | 111208400 | DUP | region2inRegion1 | 799 |

| | | | | | | | | | | | |
|---|---|---|---|---|---|---|---|---|---|---|---|
| 1636 | 13 | 111201690 | 111225859 | gain | 1000 | 111218201 | 111219200 | DEL | region2inRegion1 | 999 |
| 1690 | 13 | 164895338 | 164898801 | gain | 1400 | 164897001 | 164898400 | DUP | region2inRegion1 | 1399 |
| 1699 | 13 | 170765271 | 170819670 | gain | 2000 | 170765801 | 170767800 | DUP | region2inRegion1 | 1999 |
| 1699 | 13 | 170765271 | 170819670 | gain | 2400 | 170769201 | 170771600 | DUP | region2inRegion1 | 2399 |
| 1699 | 13 | 170765271 | 170819670 | gain | 5600 | 170772001 | 170777600 | DUP | region2inRegion1 | 5599 |
| 1699 | 13 | 170765271 | 170819670 | gain | 1000 | 170778201 | 170779200 | DUP | region2inRegion1 | 999 |
| 1699 | 13 | 170765271 | 170819670 | gain | 1800 | 170780801 | 170782600 | DUP | region2inRegion1 | 1799 |
| 1699 | 13 | 170765271 | 170819670 | gain | 2600 | 170783001 | 170785600 | DUP | region2inRegion1 | 2599 |
| 1699 | 13 | 170765271 | 170819670 | gain | 1400 | 170786801 | 170788200 | DUP | region2inRegion1 | 1399 |
| 1699 | 13 | 170765271 | 170819670 | gain | 2000 | 170790401 | 170792400 | DUP | region2inRegion1 | 1999 |
| 1699 | 13 | 170765271 | 170819670 | gain | 1200 | 170793401 | 170794600 | DUP | region2inRegion1 | 1199 |
| 1699 | 13 | 170765271 | 170819670 | gain | 800 | 170803001 | 170803800 | DUP | region2inRegion1 | 799 |
| 1699 | 13 | 170765271 | 170819670 | gain | 1600 | 170806401 | 170808000 | DUP | region2inRegion1 | 1599 |
| 1699 | 13 | 170765271 | 170819670 | gain | 800 | 170808401 | 170809200 | DUP | region2inRegion1 | 799 |
| 1699 | 13 | 170765271 | 170819670 | gain | 1800 | 170810801 | 170812600 | DUP | region2inRegion1 | 1799 |
| 1699 | 13 | 170765271 | 170819670 | gain | 1000 | 170812801 | 170813800 | DUP | region2inRegion1 | 999 |
| 1699 | 13 | 170765271 | 170819670 | gain | 1800 | 170815801 | 170817600 | DUP | region2inRegion1 | 1799 |
| 1699 | 13 | 170765271 | 170819670 | gain | 800 | 170817801 | 170818600 | DUP | region2inRegion1 | 799 |
| 1699 | 13 | 170765271 | 170819670 | gain | 3000 | 170819001 | 170822000 | DUP | overlapTail | 670 |

| | | | | | | | | | | |
|---|---|---|---|---|---|---|---|---|---|---|
| 1702 | 13 | 171285806 | 171311772 | gain | 1000 | 171292401 | 171293400 | DUP | region2inRegion1 | 999 |
| 1702 | 13 | 171285806 | 171311772 | gain | 1600 | 171295401 | 171297000 | DUP | region2inRegion1 | 1599 |
| 1702 | 13 | 171285806 | 171311772 | gain | 1400 | 171300001 | 171301400 | DUP | region2inRegion1 | 1399 |
| 1702 | 13 | 171285806 | 171311772 | gain | 1000 | 171306001 | 171307000 | DUP | region2inRegion1 | 999 |
| 1777 | 14 | 2401741 | 2423505 | gain | 800 | 2418001 | 2418800 | DEL | region2inRegion1 | 799 |
| 1781 | 14 | 7794851 | 7822418 | gain | 2400 | 7796001 | 7798400 | DEL | region2inRegion1 | 2399 |
| 1781 | 14 | 7794851 | 7822418 | gain | 800 | 7799001 | 7799800 | DUP | region2inRegion1 | 799 |
| 1781 | 14 | 7794851 | 7822418 | gain | 1000 | 7803601 | 7804600 | DEL | region2inRegion1 | 999 |
| 1782 | 14 | 8039487 | 8116098 | gain | 1000 | 8045401 | 8046400 | DUP | region2inRegion1 | 999 |
| 1782 | 14 | 8039487 | 8116098 | gain | 800 | 8056201 | 8057000 | DUP | region2inRegion1 | 799 |
| 1782 | 14 | 8039487 | 8116098 | gain | 2200 | 8074601 | 8076800 | DUP | region2inRegion1 | 2199 |
| 1782 | 14 | 8039487 | 8116098 | gain | 1000 | 8077401 | 8078400 | DUP | region2inRegion1 | 999 |
| 1782 | 14 | 8039487 | 8116098 | gain | 1000 | 8079201 | 8080200 | DUP | region2inRegion1 | 999 |
| 1782 | 14 | 8039487 | 8116098 | gain | 1000 | 8080801 | 8081800 | DUP | region2inRegion1 | 999 |
| 1782 | 14 | 8039487 | 8116098 | gain | 800 | 8083601 | 8084400 | DUP | region2inRegion1 | 799 |
| 1782 | 14 | 8039487 | 8116098 | gain | 1600 | 8086001 | 8087600 | DUP | region2inRegion1 | 1599 |
| 1782 | 14 | 8039487 | 8116098 | gain | 1000 | 8089401 | 8090400 | DUP | region2inRegion1 | 999 |
| 1782 | 14 | 8039487 | 8116098 | gain | 1400 | 8094601 | 8096000 | DUP | region2inRegion1 | 1399 |
| 1782 | 14 | 8039487 | 8116098 | gain | 1200 | 8099801 | 8101000 | DUP | region2inRegion1 | 1199 |

| | | | | | | | | | | | |
|---|---|---|---|---|---|---|---|---|---|---|---|
| 1782 | 14 | 8039487 | 8116098 | gain | 1000 | 8101601 | 8102600 | DUP | region2inRegion1 | 999 |
| 1782 | 14 | 8039487 | 8116098 | gain | 1000 | 8104601 | 8105600 | DUP | region2inRegion1 | 999 |
| 1782 | 14 | 8039487 | 8116098 | gain | 3600 | 8108001 | 8111600 | DUP | region2inRegion1 | 3599 |
| 1782 | 14 | 8039487 | 8116098 | gain | 3000 | 8112801 | 8115800 | DUP | region2inRegion1 | 2999 |
| 1792 | 14 | 21068045 | 21091201 | gain | 1000 | 21067401 | 21068400 | DUP | overlapHead | 356 |
| 1792 | 14 | 21068045 | 21091201 | gain | 2000 | 21069001 | 21071000 | DUP | region2inRegion1 | 1999 |
| 1792 | 14 | 21068045 | 21091201 | gain | 1000 | 21078801 | 21079800 | DUP | region2inRegion1 | 999 |
| 1792 | 14 | 21068045 | 21091201 | gain | 1800 | 21082801 | 21084600 | DUP | region2inRegion1 | 1799 |
| 1792 | 14 | 21068045 | 21091201 | gain | 1200 | 21087001 | 21088200 | DUP | region2inRegion1 | 1199 |
| 1816 | 14 | 56382005 | 56403580 | loss | 1000 | 56390401 | 56391400 | DUP | region2inRegion1 | 999 |
| 1820 | 14 | 65589945 | 65600023 | loss | 1200 | 65589601 | 65590800 | DEL | overlapHead | 856 |
| 1820 | 14 | 65589945 | 65600023 | loss | 1200 | 65591601 | 65592800 | DEL | region2inRegion1 | 1199 |
| 1820 | 14 | 65589945 | 65600023 | loss | 1600 | 65593201 | 65594800 | DEL | region2inRegion1 | 1599 |
| 1820 | 14 | 65589945 | 65600023 | loss | 4800 | 65597201 | 65602000 | DEL | overlapTail | 2823 |
| 1829 | 14 | 74107606 | 74126308 | loss | 1200 | 74110801 | 74112000 | DEL | region2inRegion1 | 1199 |
| 1829 | 14 | 74107606 | 74126308 | loss | 1000 | 74114001 | 74115000 | DEL | region2inRegion1 | 999 |
| 1829 | 14 | 74107606 | 74126308 | loss | 1600 | 74117401 | 74119000 | DUP | region2inRegion1 | 1599 |
| 1829 | 14 | 74107606 | 74126308 | loss | 800 | 74119201 | 74120000 | DUP | region2inRegion1 | 799 |
| 1829 | 14 | 74107606 | 74126308 | loss | 800 | 74120401 | 74121200 | DUP | region2inRegion1 | 799 |

| | | | | | | | | | | | |
|---|---|---|---|---|---|---|---|---|---|---|---|
| 1838 | 14 | 81377028 | 81553876 | gain | 800 | 81425401 | 81426200 | DUP | region2inRegion1 | 799 |
| 1838 | 14 | 81377028 | 81553876 | gain | 800 | 81426201 | 81427000 | DUP | region2inRegion1 | 799 |
| 1849 | 14 | 94594494 | 94629904 | gain | 81000 | 94572801 | 94653800 | DUP | region1inRegion2 | 35410 |
| 1873 | 14 | 120810562 | 120829324 | gain | 5200 | 120810801 | 120816000 | DUP | region2inRegion1 | 5199 |
| 1873 | 14 | 120810562 | 120829324 | gain | 800 | 120818201 | 120819000 | DUP | region2inRegion1 | 799 |
| 1873 | 14 | 120810562 | 120829324 | gain | 800 | 120819201 | 120820000 | DUP | region2inRegion1 | 799 |
| 1873 | 14 | 120810562 | 120829324 | gain | 1000 | 120820401 | 120821400 | DUP | region2inRegion1 | 999 |
| 1873 | 14 | 120810562 | 120829324 | gain | 1600 | 120822601 | 120824200 | DUP | region2inRegion1 | 1599 |
| 1873 | 14 | 120810562 | 120829324 | gain | 1200 | 120825801 | 120827000 | DUP | region2inRegion1 | 1199 |
| 1873 | 14 | 120810562 | 120829324 | gain | 2600 | 120827401 | 120830000 | DUP | overlapTail | 1924 |
| 1990 | 15 | 112634645 | 112649752 | loss | 800 | 112642401 | 112643200 | DEL | region2inRegion1 | 799 |
| 2034 | 16 | 6146177 | 6154424 | loss | 3000 | 6146601 | 6149600 | DUP | region2inRegion1 | 2999 |
| 2034 | 16 | 6146177 | 6154424 | loss | 2400 | 6151201 | 6153600 | DUP | region2inRegion1 | 2399 |
| 2034 | 16 | 6146177 | 6154424 | loss | 1200 | 6154001 | 6155200 | DUP | overlapTail | 424 |
| 2055 | 16 | 12229596 | 12241067 | loss-gain | 2200 | 12229601 | 12231800 | DEL | region2inRegion1 | 2199 |
| 2109 | 16 | 53083153 | 53202189 | gain | 1000 | 53195201 | 53196200 | DUP | region2inRegion1 | 999 |
| 2111 | 16 | 56657347 | 56707690 | loss | 1200 | 56662201 | 56663400 | DEL | region2inRegion1 | 1199 |
| 2111 | 16 | 56657347 | 56707690 | loss | 1600 | 56667401 | 56669000 | DEL | region2inRegion1 | 1599 |
| 2111 | 16 | 56657347 | 56707690 | loss | 2800 | 56671801 | 56674600 | DEL | region2inRegion1 | 2799 |

| | | | | | | | | | | | |
|---|---|---|---|---|---|---|---|---|---|---|---|
| 2111 | 16 | 56657347 | 56707690 | loss | 1000 | 56675601 | 56676600 | DEL | region2inRegion1 | 999 |
| 2111 | 16 | 56657347 | 56707690 | loss | 1200 | 56681201 | 56682400 | DEL | region2inRegion1 | 1199 |
| 2111 | 16 | 56657347 | 56707690 | loss | 800 | 56683401 | 56684200 | DUP | region2inRegion1 | 799 |
| 2111 | 16 | 56657347 | 56707690 | loss | 800 | 56698201 | 56699000 | DEL | region2inRegion1 | 799 |
| 2111 | 16 | 56657347 | 56707690 | loss | 1200 | 56703801 | 56705000 | DEL | region2inRegion1 | 1199 |
| 2168 | 17 | 53147051 | 53158077 | gain | 800 | 53152601 | 53153400 | DUP | region2inRegion1 | 799 |
| 2168 | 17 | 53147051 | 53158077 | gain | 6600 | 53154001 | 53160600 | DUP | overlapTail | 4077 |
| 312 | 2 | 11511074 | 11530117 | gain | 2400 | 11508801 | 11511200 | DUP | overlapHead | 127 |
| 312 | 2 | 11511074 | 11530117 | gain | 1000 | 11514001 | 11515000 | DEL | region2inRegion1 | 999 |
| 312 | 2 | 11511074 | 11530117 | gain | 1200 | 11514801 | 11516000 | DUP | region2inRegion1 | 1199 |
| 312 | 2 | 11511074 | 11530117 | gain | 1400 | 11516401 | 11517800 | DUP | region2inRegion1 | 1399 |
| 312 | 2 | 11511074 | 11530117 | gain | 1600 | 11519001 | 11520600 | DUP | region2inRegion1 | 1599 |
| 312 | 2 | 11511074 | 11530117 | gain | 1000 | 11520401 | 11521400 | DEL | region2inRegion1 | 999 |
| 312 | 2 | 11511074 | 11530117 | gain | 1400 | 11522801 | 11524200 | DUP | region2inRegion1 | 1399 |
| 312 | 2 | 11511074 | 11530117 | gain | 1000 | 11529801 | 11530800 | DUP | overlapTail | 317 |
| 314 | 2 | 12364565 | 12398203 | loss | 1000 | 12374601 | 12375600 | DUP | region2inRegion1 | 999 |
| 316 | 2 | 12689951 | 12700771 | loss | 3400 | 12690201 | 12693600 | DUP | region2inRegion1 | 3399 |
| 316 | 2 | 12689951 | 12700771 | loss | 1200 | 12694401 | 12695600 | DUP | region2inRegion1 | 1199 |
| 316 | 2 | 12689951 | 12700771 | loss | 1200 | 12697401 | 12698600 | DUP | region2inRegion1 | 1199 |

| | | | | | | | | | | |
|---|---|---|---|---|---|---|---|---|---|---|
| 316 | 2 | 12689951 | 12700771 | loss | 2000 | 12699401 | 12701400 | DUP | overlapTail | 1371 |
| 319 | 2 | 14000848 | 14032642 | loss-gain | 1400 | 14000801 | 14002200 | DUP | overlapHead | 1353 |
| 319 | 2 | 14000848 | 14032642 | loss-gain | 2200 | 14003401 | 14005600 | DUP | region2inRegion1 | 2199 |
| 319 | 2 | 14000848 | 14032642 | loss-gain | 5000 | 14006001 | 14011000 | DUP | region2inRegion1 | 4999 |
| 319 | 2 | 14000848 | 14032642 | loss-gain | 6800 | 14011401 | 14018200 | DUP | region2inRegion1 | 6799 |
| 319 | 2 | 14000848 | 14032642 | loss-gain | 1600 | 14018601 | 14020200 | DUP | region2inRegion1 | 1599 |
| 319 | 2 | 14000848 | 14032642 | loss-gain | 4600 | 14020601 | 14025200 | DUP | region2inRegion1 | 4599 |
| 319 | 2 | 14000848 | 14032642 | loss-gain | 1000 | 14026001 | 14027000 | DUP | region2inRegion1 | 999 |
| 319 | 2 | 14000848 | 14032642 | loss-gain | 2800 | 14028201 | 14031000 | DUP | region2inRegion1 | 2799 |
| 320 | 2 | 14728311 | 14771718 | gain | 1000 | 14730001 | 14731000 | DUP | region2inRegion1 | 999 |
| 320 | 2 | 14728311 | 14771718 | gain | 4000 | 14731801 | 14735800 | DUP | region2inRegion1 | 3999 |
| 320 | 2 | 14728311 | 14771718 | gain | 2400 | 14738801 | 14741200 | DUP | region2inRegion1 | 2399 |
| 320 | 2 | 14728311 | 14771718 | gain | 4200 | 14741601 | 14745800 | DUP | region2inRegion1 | 4199 |
| 320 | 2 | 14728311 | 14771718 | gain | 1800 | 14748801 | 14750600 | DUP | region2inRegion1 | 1799 |
| 320 | 2 | 14728311 | 14771718 | gain | 3800 | 14751001 | 14754800 | DUP | region2inRegion1 | 3799 |
| 321 | 2 | 15024071 | 15060448 | loss | 4800 | 15024801 | 15029600 | DUP | region2inRegion1 | 4799 |
| 321 | 2 | 15024071 | 15060448 | loss | 1200 | 15030001 | 15031200 | DUP | region2inRegion1 | 1199 |
| 321 | 2 | 15024071 | 15060448 | loss | 4400 | 15031601 | 15036000 | DUP | region2inRegion1 | 4399 |
| 321 | 2 | 15024071 | 15060448 | loss | 1000 | 15051001 | 15052000 | DUP | region2inRegion1 | 999 |

| | | | | | | | | | | |
|---|---|---|---|---|---|---|---|---|---|---|
| 321 | 2 | 15024071 | 15060448 | loss | 1400 | 15052601 | 15054000 | DUP | region2inRegion1 | 1399 |
| 321 | 2 | 15024071 | 15060448 | loss | 800 | 15055601 | 15056400 | DUP | region2inRegion1 | 799 |
| 321 | 2 | 15024071 | 15060448 | loss | 1600 | 15057201 | 15058800 | DUP | region2inRegion1 | 1599 |
| 348 | 2 | 54685639 | 54717573 | loss-gain | 800 | 54686401 | 54687200 | DEL | region2inRegion1 | 799 |
| 348 | 2 | 54685639 | 54717573 | loss-gain | 1000 | 54688001 | 54689000 | DEL | region2inRegion1 | 999 |
| 348 | 2 | 54685639 | 54717573 | loss-gain | 1000 | 54691801 | 54692800 | DEL | region2inRegion1 | 999 |
| 348 | 2 | 54685639 | 54717573 | loss-gain | 3200 | 54695801 | 54699000 | DEL | region2inRegion1 | 3199 |
| 348 | 2 | 54685639 | 54717573 | loss-gain | 4800 | 54699601 | 54704400 | DEL | region2inRegion1 | 4799 |
| 348 | 2 | 54685639 | 54717573 | loss-gain | 800 | 54706001 | 54706800 | DEL | region2inRegion1 | 799 |
| 348 | 2 | 54685639 | 54717573 | loss-gain | 800 | 54709601 | 54710400 | DEL | region2inRegion1 | 799 |
| 348 | 2 | 54685639 | 54717573 | loss-gain | 1200 | 54711401 | 54712600 | DEL | region2inRegion1 | 1199 |
| 348 | 2 | 54685639 | 54717573 | loss-gain | 3000 | 54713801 | 54716800 | DEL | region2inRegion1 | 2999 |
| 359 | 2 | 61357153 | 61369311 | loss | 1400 | 61357401 | 61358800 | DUP | region2inRegion1 | 1399 |
| 362 | 2 | 62416028 | 62428337 | gain | 1000 | 62416001 | 62417000 | DUP | overlapHead | 973 |
| 362 | 2 | 62416028 | 62428337 | gain | 800 | 62420201 | 62421000 | DUP | region2inRegion1 | 799 |
| 362 | 2 | 62416028 | 62428337 | gain | 4000 | 62424601 | 62428600 | DUP | overlapTail | 3737 |
| 364 | 2 | 62628253 | 62738661 | gain | 1400 | 62627001 | 62628400 | DUP | overlapHead | 148 |
| 364 | 2 | 62628253 | 62738661 | gain | 1400 | 62628601 | 62630000 | DUP | region2inRegion1 | 1399 |
| 364 | 2 | 62628253 | 62738661 | gain | 2000 | 62631601 | 62633600 | DUP | region2inRegion1 | 1999 |

| | | | | | | | | | | |
|---|---|---|---|---|---|---|---|---|---|---|
| 364 | 2 | 62628253 | 62738661 | gain | 2200 | 62634001 | 62636200 | DUP | region2inRegion1 | 2199 |
| 364 | 2 | 62628253 | 62738661 | gain | 1600 | 62638001 | 62639600 | DUP | region2inRegion1 | 1599 |
| 364 | 2 | 62628253 | 62738661 | gain | 2400 | 62640801 | 62643200 | DUP | region2inRegion1 | 2399 |
| 364 | 2 | 62628253 | 62738661 | gain | 1200 | 62644001 | 62645200 | DUP | region2inRegion1 | 1199 |
| 364 | 2 | 62628253 | 62738661 | gain | 1000 | 62645801 | 62646800 | DUP | region2inRegion1 | 999 |
| 364 | 2 | 62628253 | 62738661 | gain | 1800 | 62647801 | 62649600 | DUP | region2inRegion1 | 1799 |
| 364 | 2 | 62628253 | 62738661 | gain | 1600 | 62655201 | 62656800 | DUP | region2inRegion1 | 1599 |
| 364 | 2 | 62628253 | 62738661 | gain | 3600 | 62660601 | 62664200 | DUP | region2inRegion1 | 3599 |
| 364 | 2 | 62628253 | 62738661 | gain | 1200 | 62665001 | 62666200 | DUP | region2inRegion1 | 1199 |
| 364 | 2 | 62628253 | 62738661 | gain | 800 | 62669201 | 62670000 | DUP | region2inRegion1 | 799 |
| 364 | 2 | 62628253 | 62738661 | gain | 2400 | 62723201 | 62725600 | DUP | region2inRegion1 | 2399 |
| 378 | 2 | 67915859 | 67952389 | gain | 4800 | 67913001 | 67917800 | DUP | overlapHead | 1942 |
| 378 | 2 | 67915859 | 67952389 | gain | 22800 | 67919401 | 67942200 | DUP | region2inRegion1 | 22799 |
| 378 | 2 | 67915859 | 67952389 | gain | 800 | 67944801 | 67945600 | DUP | region2inRegion1 | 799 |
| 378 | 2 | 67915859 | 67952389 | gain | 1000 | 67945601 | 67946600 | DUP | region2inRegion1 | 999 |
| 378 | 2 | 67915859 | 67952389 | gain | 3800 | 67947001 | 67950800 | DUP | region2inRegion1 | 3799 |
| 378 | 2 | 67915859 | 67952389 | gain | 9800 | 67952201 | 67962000 | DUP | overlapTail | 189 |
| 379 | 2 | 68061173 | 68181801 | gain | 75600 | 68022201 | 68097800 | DUP | overlapHead | 36628 |
| 379 | 2 | 68061173 | 68181801 | gain | 1600 | 68100801 | 68102400 | DUP | region2inRegion1 | 1599 |

| | | | | | | | | | | |
|---|---|---|---|---|---|---|---|---|---|---|
| 379 | 2 | 68061173 | 68181801 | gain | 44600 | 68102801 | 68147400 | DUP | region2inRegion1 | 44599 |
| 379 | 2 | 68061173 | 68181801 | gain | 1600 | 68147601 | 68149200 | DUP | region2inRegion1 | 1599 |
| 379 | 2 | 68061173 | 68181801 | gain | 5800 | 68150201 | 68156000 | DUP | region2inRegion1 | 5799 |
| 379 | 2 | 68061173 | 68181801 | gain | 119000 | 68159401 | 68278400 | DUP | overlapTail | 22401 |
| 401 | 2 | 95897658 | 95915484 | loss | 1600 | 95897801 | 95899400 | DEL | region2inRegion1 | 1599 |
| 401 | 2 | 95897658 | 95915484 | loss | 1000 | 95901601 | 95902600 | DEL | region2inRegion1 | 999 |
| 401 | 2 | 95897658 | 95915484 | loss | 800 | 95905801 | 95906600 | DEL | region2inRegion1 | 799 |
| 401 | 2 | 95897658 | 95915484 | loss | 1400 | 95907601 | 95909000 | DEL | region2inRegion1 | 1399 |
| 408 | 2 | 96833054 | 96835454 | loss | 1600 | 96834201 | 96835800 | DEL | overlapTail | 1254 |
| 466 | 2 | 148904898 | 148913506 | loss-gain | 800 | 148908201 | 148909000 | DEL | region2inRegion1 | 799 |
| 466 | 2 | 148904898 | 148913506 | loss-gain | 1200 | 148910201 | 148911400 | DUP | region2inRegion1 | 1199 |
| 471 | 2 | 156079099 | 156090392 | gain | 1400 | 156081601 | 156083000 | DUP | region2inRegion1 | 1399 |
| 472 | 2 | 158544152 | 158558867 | gain | 1400 | 158544001 | 158545400 | DUP | overlapHead | 1249 |
| 472 | 2 | 158544152 | 158558867 | gain | 3400 | 158546601 | 158550000 | DUP | region2inRegion1 | 3399 |
| 472 | 2 | 158544152 | 158558867 | gain | 1200 | 158551801 | 158553000 | DUP | region2inRegion1 | 1199 |
| 472 | 2 | 158544152 | 158558867 | gain | 1000 | 158558801 | 158559800 | DUP | overlapTail | 67 |
| 473 | 2 | 158605784 | 158612295 | gain | 2000 | 158603801 | 158605800 | DUP | overlapHead | 17 |
| 473 | 2 | 158605784 | 158629757 | gain | 2000 | 158603801 | 158605800 | DUP | overlapHead | 17 |
| 473 | 2 | 158605784 | 158612295 | gain | 5000 | 158608401 | 158613400 | DUP | overlapTail | 3895 |

| | | | | | | | | | | | |
|---|---|---|---|---|---|---|---|---|---|---|---|
| 473 | 2 | 158605784 | 158629757 | gain | 5000 | 158608401 | 158613400 | DUP | region2inRegion1 | 4999 |
| 473 | 2 | 158605784 | 158629757 | gain | 1200 | 158613601 | 158614800 | DUP | region2inRegion1 | 1199 |
| 473 | 2 | 158605784 | 158629757 | gain | 1400 | 158616001 | 158617400 | DUP | region2inRegion1 | 1399 |
| 473 | 2 | 158605784 | 158629757 | gain | 800 | 158617801 | 158618600 | DUP | region2inRegion1 | 799 |
| 473 | 2 | 158605784 | 158629757 | gain | 1200 | 158619401 | 158620600 | DUP | region2inRegion1 | 1199 |
| 473 | 2 | 158605784 | 158629757 | gain | 800 | 158622601 | 158623400 | DUP | region2inRegion1 | 799 |
| 473 | 2 | 158605784 | 158629757 | gain | 1000 | 158623601 | 158624600 | DUP | region2inRegion1 | 999 |
| 473 | 2 | 158605784 | 158629757 | gain | 800 | 158625401 | 158626200 | DUP | region2inRegion1 | 799 |
| 473 | 2 | 158605784 | 158629757 | gain | 1000 | 158626801 | 158627800 | DUP | region2inRegion1 | 999 |
| 473 | 2 | 158605784 | 158629757 | gain | 800 | 158629201 | 158630000 | DUP | overlapTail | 557 |
| 479 | 2 | 159094998 | 159110351 | gain | 8200 | 159090001 | 159098200 | DUP | overlapHead | 3203 |
| 479 | 2 | 159094998 | 159110351 | gain | 30200 | 159098601 | 159128800 | DUP | overlapTail | 11751 |
| 504 | 3 | 45023435 | 45241189 | gain | 1200 | 45043201 | 45044400 | DUP | region2inRegion1 | 1199 |
| 504 | 3 | 45023435 | 45241189 | gain | 32400 | 45069801 | 45102200 | DUP | region2inRegion1 | 32399 |
| 504 | 3 | 45023435 | 45241189 | gain | 5000 | 45164201 | 45169200 | DUP | region2inRegion1 | 4999 |
| 504 | 3 | 45023435 | 45241189 | gain | 2000 | 45169601 | 45171600 | DUP | region2inRegion1 | 1999 |
| 504 | 3 | 45023435 | 45241189 | gain | 1600 | 45172201 | 45173800 | DUP | region2inRegion1 | 1599 |
| 504 | 3 | 45023435 | 45241189 | gain | 2800 | 45174201 | 45177000 | DUP | region2inRegion1 | 2799 |
| 504 | 3 | 45023435 | 45241189 | gain | 3600 | 45177401 | 45181000 | DUP | region2inRegion1 | 3599 |

| | | | | | | | | | | |
|---|---|---|---|---|---|---|---|---|---|---|
| 504 | 3 | 45023435 | 45241189 | gain | 5000 | 45181601 | 45186600 | DUP | region2inRegion1 | 4999 |
| 504 | 3 | 45023435 | 45241189 | gain | 7800 | 45187201 | 45195000 | DUP | region2inRegion1 | 7799 |
| 504 | 3 | 45023435 | 45241189 | gain | 1600 | 45195601 | 45197200 | DUP | region2inRegion1 | 1599 |
| 504 | 3 | 45023435 | 45241189 | gain | 1200 | 45198001 | 45199200 | DUP | region2inRegion1 | 1199 |
| 504 | 3 | 45023435 | 45241189 | gain | 2000 | 45200001 | 45202000 | DUP | region2inRegion1 | 1999 |
| 504 | 3 | 45023435 | 45241189 | gain | 1600 | 45202401 | 45204000 | DUP | region2inRegion1 | 1599 |
| 504 | 3 | 45023435 | 45241189 | gain | 1200 | 45204401 | 45205600 | DUP | region2inRegion1 | 1199 |
| 504 | 3 | 45023435 | 45241189 | gain | 1600 | 45206001 | 45207600 | DUP | region2inRegion1 | 1599 |
| 504 | 3 | 45023435 | 45241189 | gain | 2800 | 45211601 | 45214400 | DUP | region2inRegion1 | 2799 |
| 504 | 3 | 45023435 | 45241189 | gain | 1000 | 45216201 | 45217200 | DUP | region2inRegion1 | 999 |
| 504 | 3 | 45023435 | 45241189 | gain | 1600 | 45218201 | 45219800 | DUP | region2inRegion1 | 1599 |
| 504 | 3 | 45023435 | 45241189 | gain | 2200 | 45220401 | 45222600 | DUP | region2inRegion1 | 2199 |
| 504 | 3 | 45023435 | 45241189 | gain | 2200 | 45224201 | 45226400 | DUP | region2inRegion1 | 2199 |
| 504 | 3 | 45023435 | 45241189 | gain | 1800 | 45228801 | 45230600 | DUP | region2inRegion1 | 1799 |
| 504 | 3 | 45023435 | 45241189 | gain | 1400 | 45231601 | 45233000 | DUP | region2inRegion1 | 1399 |
| 504 | 3 | 45023435 | 45241189 | gain | 1600 | 45234601 | 45236200 | DUP | region2inRegion1 | 1599 |
| 504 | 3 | 45023435 | 45241189 | gain | 1000 | 45236401 | 45237400 | DUP | region2inRegion1 | 999 |
| 524 | 3 | 67785626 | 67805349 | gain | 800 | 67787801 | 67788600 | DUP | region2inRegion1 | 799 |
| 524 | 3 | 67785626 | 67805349 | gain | 1200 | 67802801 | 67804000 | DEL | region2inRegion1 | 1199 |

| | | | | | | | | | | |
|---|---|---|---|---|---|---|---|---|---|---|
| 539 | 3 | 83892565 | 83943514 | gain | 1200 | 83897601 | 83898800 | DUP | region2inRegion1 | 1199 |
| 539 | 3 | 83892565 | 83943514 | gain | 800 | 83929601 | 83930400 | DUP | region2inRegion1 | 799 |
| 581 | 3 | 142899740 | 142925257 | loss | 8800 | 142901001 | 142909800 | DUP | region2inRegion1 | 8799 |
| 581 | 3 | 142899740 | 142925257 | loss | 6800 | 142914801 | 142921600 | DUP | region2inRegion1 | 6799 |
| 581 | 3 | 142899740 | 142925257 | loss | 7800 | 142922401 | 142930200 | DUP | overlapTail | 2857 |
| 582 | 3 | 142999513 | 143009221 | gain | 800 | 142999201 | 143000000 | DUP | overlapHead | 488 |
| 582 | 3 | 142999513 | 143009221 | gain | 800 | 143004401 | 143005200 | DUP | region2inRegion1 | 799 |
| 582 | 3 | 142999513 | 143009221 | gain | 20200 | 143007601 | 143027800 | DUP | overlapTail | 1621 |
| 585 | 3 | 143590087 | 143597682 | gain | 1600 | 143588801 | 143590400 | DUP | overlapHead | 314 |
| 585 | 3 | 143590087 | 143597682 | gain | 4000 | 143590801 | 143594800 | DUP | region2inRegion1 | 3999 |
| 585 | 3 | 143590087 | 143597682 | gain | 30000 | 143595201 | 143625200 | DUP | overlapTail | 2482 |
| 589 | 3 | 144724140 | 144783726 | gain | 3000 | 144723401 | 144726400 | DUP | overlapHead | 2261 |
| 589 | 3 | 144724140 | 144783726 | gain | 1200 | 144727401 | 144728600 | DUP | region2inRegion1 | 1199 |
| 589 | 3 | 144724140 | 144783726 | gain | 800 | 144729001 | 144729800 | DUP | region2inRegion1 | 799 |
| 589 | 3 | 144724140 | 144783726 | gain | 1600 | 144730201 | 144731800 | DUP | region2inRegion1 | 1599 |
| 589 | 3 | 144724140 | 144783726 | gain | 2800 | 144733401 | 144736200 | DUP | region2inRegion1 | 2799 |
| 589 | 3 | 144724140 | 144783726 | gain | 1000 | 144736801 | 144737800 | DUP | region2inRegion1 | 999 |
| 589 | 3 | 144724140 | 144783726 | gain | 1800 | 144738201 | 144740000 | DUP | region2inRegion1 | 1799 |
| 589 | 3 | 144724140 | 144783726 | gain | 1800 | 144740401 | 144742200 | DUP | region2inRegion1 | 1799 |

| | | | | | | | | | | |
|---|---|---|---|---|---|---|---|---|---|---|
| 589 | 3 | 144724140 | 144783726 | gain | 800 | 144744001 | 144744800 | DUP | region2inRegion1 | 799 |
| 589 | 3 | 144724140 | 144783726 | gain | 800 | 144746801 | 144747600 | DUP | region2inRegion1 | 799 |
| 589 | 3 | 144724140 | 144783726 | gain | 800 | 144747801 | 144748600 | DUP | region2inRegion1 | 799 |
| 589 | 3 | 144724140 | 144783726 | gain | 2000 | 144748801 | 144750800 | DUP | region2inRegion1 | 1999 |
| 589 | 3 | 144724140 | 144783726 | gain | 800 | 144751201 | 144752000 | DUP | region2inRegion1 | 799 |
| 589 | 3 | 144724140 | 144783726 | gain | 1000 | 144752201 | 144753200 | DUP | region2inRegion1 | 999 |
| 589 | 3 | 144724140 | 144783726 | gain | 2000 | 144753401 | 144755400 | DUP | region2inRegion1 | 1999 |
| 589 | 3 | 144724140 | 144783726 | gain | 3600 | 144756201 | 144759800 | DUP | region2inRegion1 | 3599 |
| 589 | 3 | 144724140 | 144783726 | gain | 1400 | 144760201 | 144761600 | DUP | region2inRegion1 | 1399 |
| 589 | 3 | 144724140 | 144783726 | gain | 2600 | 144763201 | 144765800 | DUP | region2inRegion1 | 2599 |
| 589 | 3 | 144724140 | 144783726 | gain | 800 | 144767401 | 144768200 | DUP | region2inRegion1 | 799 |
| 589 | 3 | 144724140 | 144783726 | gain | 8800 | 144768601 | 144777400 | DUP | region2inRegion1 | 8799 |
| 589 | 3 | 144724140 | 144783726 | gain | 2200 | 144778801 | 144781000 | DUP | region2inRegion1 | 2199 |
| 589 | 3 | 144724140 | 144783726 | gain | 3600 | 144781601 | 144785200 | DUP | overlapTail | 2126 |
| 698 | 4 | 111405421 | 111446020 | gain | 1800 | 111404601 | 111406400 | DUP | overlapHead | 980 |
| 698 | 4 | 111405421 | 111446020 | gain | 1400 | 111411201 | 111412600 | DUP | region2inRegion1 | 1399 |
| 698 | 4 | 111405421 | 111446020 | gain | 1000 | 111414801 | 111415800 | DUP | region2inRegion1 | 999 |
| 698 | 4 | 111405421 | 111446020 | gain | 800 | 111417801 | 111418600 | DUP | region2inRegion1 | 799 |
| 698 | 4 | 111405421 | 111446020 | gain | 800 | 111418801 | 111419600 | DUP | region2inRegion1 | 799 |

| | | | | | | | | | | | |
|---|---|---|---|---|---|---|---|---|---|---|---|
| 698 | 4 | 111405421 | 111446020 | gain | 1600 | 111421601 | 111423200 | DUP | region2inRegion1 | 1599 |
| 698 | 4 | 111405421 | 111446020 | gain | 800 | 111423601 | 111424400 | DUP | region2inRegion1 | 799 |
| 698 | 4 | 111405421 | 111446020 | gain | 1000 | 111426001 | 111427000 | DUP | region2inRegion1 | 999 |
| 698 | 4 | 111405421 | 111446020 | gain | 1600 | 111427201 | 111428800 | DUP | region2inRegion1 | 1599 |
| 698 | 4 | 111405421 | 111446020 | gain | 1000 | 111430001 | 111431000 | DUP | region2inRegion1 | 999 |
| 698 | 4 | 111405421 | 111446020 | gain | 800 | 111432001 | 111432800 | DUP | region2inRegion1 | 799 |
| 698 | 4 | 111405421 | 111446020 | gain | 1600 | 111438401 | 111440000 | DUP | region2inRegion1 | 1599 |
| 698 | 4 | 111405421 | 111446020 | gain | 800 | 111440201 | 111441000 | DUP | region2inRegion1 | 799 |
| 698 | 4 | 111405421 | 111446020 | gain | 1600 | 111443001 | 111444600 | DUP | region2inRegion1 | 1599 |
| 727 | 5 | 18191073 | 18199920 | gain | 2000 | 18190201 | 18192200 | DUP | overlapHead | 1128 |
| 727 | 5 | 18191073 | 18199920 | gain | 1000 | 18193201 | 18194200 | DUP | region2inRegion1 | 999 |
| 727 | 5 | 18191073 | 18199920 | gain | 1000 | 18195801 | 18196800 | DUP | region2inRegion1 | 999 |
| 730 | 5 | 21242609 | 21260003 | gain | 1000 | 21246201 | 21247200 | DUP | region2inRegion1 | 999 |
| 730 | 5 | 21242609 | 21260003 | gain | 800 | 21248601 | 21249400 | DUP | region2inRegion1 | 799 |
| 730 | 5 | 21242609 | 21260003 | gain | 800 | 21250601 | 21251400 | DUP | region2inRegion1 | 799 |
| 730 | 5 | 21242609 | 21260003 | gain | 1400 | 21253401 | 21254800 | DUP | region2inRegion1 | 1399 |
| 730 | 5 | 21242609 | 21260003 | gain | 1000 | 21255201 | 21256200 | DUP | region2inRegion1 | 999 |
| 730 | 5 | 21242609 | 21260003 | gain | 3200 | 21257201 | 21260400 | DUP | overlapTail | 2803 |
| 731 | 5 | 21332629 | 21368821 | gain | 1000 | 21334401 | 21335400 | DUP | region2inRegion1 | 999 |

| | | | | | | | | | | | |
|---|---|---|---|---|---|---|---|---|---|---|---|
| 731 | 5 | 21332629 | 21368821 | gain | 800 | 21335801 | 21336600 | DUP | region2inRegion1 | 799 |
| 731 | 5 | 21332629 | 21368821 | gain | 1000 | 21338401 | 21339400 | DUP | region2inRegion1 | 999 |
| 731 | 5 | 21332629 | 21368821 | gain | 800 | 21343601 | 21344400 | DUP | region2inRegion1 | 799 |
| 731 | 5 | 21332629 | 21368821 | gain | 1000 | 21344801 | 21345800 | DUP | region2inRegion1 | 999 |
| 731 | 5 | 21332629 | 21368821 | gain | 2400 | 21348201 | 21350600 | DUP | region2inRegion1 | 2399 |
| 731 | 5 | 21332629 | 21368821 | gain | 2400 | 21351201 | 21353600 | DUP | region2inRegion1 | 2399 |
| 731 | 5 | 21332629 | 21368821 | gain | 2200 | 21359801 | 21362000 | DUP | region2inRegion1 | 2199 |
| 731 | 5 | 21332629 | 21368821 | gain | 1000 | 21367401 | 21368400 | DUP | region2inRegion1 | 999 |
| 736 | 5 | 22004036 | 22012384 | loss | 1400 | 22006601 | 22008000 | DUP | region2inRegion1 | 1399 |
| 736 | 5 | 22004036 | 22012384 | loss | 1800 | 22009801 | 22011600 | DUP | region2inRegion1 | 1799 |
| 800 | 5 | 79630280 | 79697936 | gain | 1400 | 79637801 | 79639200 | DEL | region2inRegion1 | 1399 |
| 833 | 6 | 8471113 | 8494128 | gain | 1000 | 8492801 | 8493800 | DUP | region2inRegion1 | 999 |
| 871 | 6 | 107004191 | 107039849 | loss | 1200 | 107004001 | 107005200 | DEL | overlapHead | 1010 |
| 871 | 6 | 107004191 | 107039849 | loss | 3400 | 107008201 | 107011600 | DEL | region2inRegion1 | 3399 |
| 871 | 6 | 107004191 | 107039849 | loss | 9600 | 107012201 | 107021800 | DEL | region2inRegion1 | 9599 |
| 871 | 6 | 107004191 | 107039849 | loss | 1200 | 107024001 | 107025200 | DEL | region2inRegion1 | 1199 |
| 871 | 6 | 107004191 | 107039849 | loss | 2000 | 107028401 | 107030400 | DEL | region2inRegion1 | 1999 |
| 871 | 6 | 107004191 | 107039849 | loss | 1600 | 107032201 | 107033800 | DEL | region2inRegion1 | 1599 |
| 871 | 6 | 107004191 | 107039849 | loss | 1000 | 107035001 | 107036000 | DEL | region2inRegion1 | 999 |

| | | | | | | | | | | |
|---|---|---|---|---|---|---|---|---|---|---|
| 871 | 6 | 107004191 | 107039849 | loss | 2200 | 107036601 | 107038800 | DEL | region2inRegion1 | 2199 |
| 917 | 6 | 151994674 | 152015484 | gain | 1000 | 151996201 | 151997200 | DUP | region2inRegion1 | 999 |
| 917 | 6 | 151994674 | 152015484 | gain | 5600 | 151998401 | 152004000 | DUP | region2inRegion1 | 5599 |
| 917 | 6 | 151994674 | 152015484 | gain | 4400 | 152005401 | 152009800 | DUP | region2inRegion1 | 4399 |
| 917 | 6 | 151994674 | 152015484 | gain | 1000 | 152011001 | 152012000 | DUP | region2inRegion1 | 999 |
| 917 | 6 | 151994674 | 152015484 | gain | 3000 | 152013801 | 152016800 | DUP | overlapTail | 1684 |
| 934 | 7 | 22207228 | 22236111 | gain | 24000 | 22204201 | 22228200 | DUP | overlapHead | 20973 |
| 938 | 7 | 23307667 | 23658110 | loss-gain | 1600 | 23306601 | 23308200 | DEL | overlapHead | 534 |
| 938 | 7 | 23307667 | 23658110 | loss-gain | 1200 | 23309401 | 23310600 | DEL | region2inRegion1 | 1199 |
| 938 | 7 | 23307667 | 23658110 | loss-gain | 1000 | 23312001 | 23313000 | DEL | region2inRegion1 | 999 |
| 938 | 7 | 23307667 | 23658110 | loss-gain | 800 | 23314001 | 23314800 | DEL | region2inRegion1 | 799 |
| 938 | 7 | 23307667 | 23658110 | loss-gain | 1000 | 23315401 | 23316400 | DEL | region2inRegion1 | 999 |
| 938 | 7 | 23307667 | 23658110 | loss-gain | 1000 | 23317201 | 23318200 | DEL | region2inRegion1 | 999 |
| 938 | 7 | 23307667 | 23658110 | loss-gain | 1200 | 23318601 | 23319800 | DEL | region2inRegion1 | 1199 |
| 938 | 7 | 23307667 | 23658110 | loss-gain | 800 | 23320001 | 23320800 | DEL | region2inRegion1 | 799 |
| 938 | 7 | 23307667 | 23658110 | loss-gain | 800 | 23322601 | 23323400 | DEL | region2inRegion1 | 799 |
| 938 | 7 | 23307667 | 23658110 | loss-gain | 1200 | 23327601 | 23328800 | DEL | region2inRegion1 | 1199 |
| 938 | 7 | 23307667 | 23658110 | loss-gain | 1000 | 23332201 | 23333200 | DEL | region2inRegion1 | 999 |
| 938 | 7 | 23307667 | 23658110 | loss-gain | 1600 | 23335801 | 23337400 | DEL | region2inRegion1 | 1599 |

| | | | | | | | | | | |
|---|---|---|---|---|---|---|---|---|---|---|
| 938 | 7 | 23307667 | 23658110 | loss-gain | 800 | 23337801 | 23338600 | DEL | region2inRegion1 | 799 |
| 938 | 7 | 23307667 | 23658110 | loss-gain | 1200 | 23338801 | 23340000 | DEL | region2inRegion1 | 1199 |
| 938 | 7 | 23307667 | 23658110 | loss-gain | 1000 | 23341001 | 23342000 | DUP | region2inRegion1 | 999 |
| 938 | 7 | 23307667 | 23658110 | loss-gain | 800 | 23347601 | 23348400 | DEL | region2inRegion1 | 799 |
| 938 | 7 | 23307667 | 23658110 | loss-gain | 1600 | 23353001 | 23354600 | DEL | region2inRegion1 | 1599 |
| 938 | 7 | 23307667 | 23658110 | loss-gain | 1400 | 23356801 | 23358200 | DEL | region2inRegion1 | 1399 |
| 938 | 7 | 23307667 | 23658110 | loss-gain | 1800 | 23359401 | 23361200 | DEL | region2inRegion1 | 1799 |
| 938 | 7 | 23307667 | 23658110 | loss-gain | 1400 | 23363601 | 23365000 | DEL | region2inRegion1 | 1399 |
| 938 | 7 | 23307667 | 23658110 | loss-gain | 2000 | 23367401 | 23369400 | DEL | region2inRegion1 | 1999 |
| 938 | 7 | 23307667 | 23658110 | loss-gain | 2000 | 23372201 | 23374200 | DEL | region2inRegion1 | 1999 |
| 938 | 7 | 23307667 | 23658110 | loss-gain | 1000 | 23380201 | 23381200 | DUP | region2inRegion1 | 999 |
| 938 | 7 | 23307667 | 23658110 | loss-gain | 800 | 23396201 | 23397000 | DUP | region2inRegion1 | 799 |
| 938 | 7 | 23307667 | 23658110 | loss-gain | 1200 | 23406401 | 23407600 | DEL | region2inRegion1 | 1199 |
| 938 | 7 | 23307667 | 23658110 | loss-gain | 1000 | 23411201 | 23412200 | DEL | region2inRegion1 | 999 |
| 938 | 7 | 23307667 | 23658110 | loss-gain | 800 | 23463201 | 23464000 | DEL | region2inRegion1 | 799 |
| 938 | 7 | 23307667 | 23658110 | loss-gain | 800 | 23467801 | 23468600 | DEL | region2inRegion1 | 799 |
| 938 | 7 | 23307667 | 23658110 | loss-gain | 1000 | 23472401 | 23473400 | DEL | region2inRegion1 | 999 |
| 938 | 7 | 23307667 | 23658110 | loss-gain | 1400 | 23475401 | 23476800 | DEL | region2inRegion1 | 1399 |
| 938 | 7 | 23307667 | 23658110 | loss-gain | 800 | 23479601 | 23480400 | DEL | region2inRegion1 | 799 |

| | | | | | | | | | | |
|---|---|---|---|---|---|---|---|---|---|---|
| 938 | 7 | 23307667 | 23658110 | loss-gain | 800 | 23488801 | 23489600 | DEL | region2inRegion1 | 799 |
| 939 | 7 | 23481211 | 23658110 | loss-gain | 800 | 23488801 | 23489600 | DEL | region2inRegion1 | 799 |
| 938 | 7 | 23307667 | 23658110 | loss-gain | 800 | 23491401 | 23492200 | DEL | region2inRegion1 | 799 |
| 939 | 7 | 23481211 | 23658110 | loss-gain | 800 | 23491401 | 23492200 | DEL | region2inRegion1 | 799 |
| 938 | 7 | 23307667 | 23658110 | loss-gain | 1000 | 23496001 | 23497000 | DEL | region2inRegion1 | 999 |
| 939 | 7 | 23481211 | 23658110 | loss-gain | 1000 | 23496001 | 23497000 | DEL | region2inRegion1 | 999 |
| 938 | 7 | 23307667 | 23658110 | loss-gain | 1800 | 23499001 | 23500800 | DEL | region2inRegion1 | 1799 |
| 939 | 7 | 23481211 | 23658110 | loss-gain | 1800 | 23499001 | 23500800 | DEL | region2inRegion1 | 1799 |
| 938 | 7 | 23307667 | 23658110 | loss-gain | 800 | 23505401 | 23506200 | DEL | region2inRegion1 | 799 |
| 939 | 7 | 23481211 | 23658110 | loss-gain | 800 | 23505401 | 23506200 | DEL | region2inRegion1 | 799 |
| 938 | 7 | 23307667 | 23658110 | loss-gain | 800 | 23506401 | 23507200 | DEL | region2inRegion1 | 799 |
| 939 | 7 | 23481211 | 23658110 | loss-gain | 800 | 23506401 | 23507200 | DEL | region2inRegion1 | 799 |
| 938 | 7 | 23307667 | 23658110 | loss-gain | 800 | 23514001 | 23514800 | DEL | region2inRegion1 | 799 |
| 939 | 7 | 23481211 | 23658110 | loss-gain | 800 | 23514001 | 23514800 | DEL | region2inRegion1 | 799 |
| 938 | 7 | 23307667 | 23658110 | loss-gain | 1200 | 23517401 | 23518600 | DEL | region2inRegion1 | 1199 |
| 939 | 7 | 23481211 | 23658110 | loss-gain | 1200 | 23517401 | 23518600 | DEL | region2inRegion1 | 1199 |
| 938 | 7 | 23307667 | 23658110 | loss-gain | 1200 | 23524601 | 23525800 | DEL | region2inRegion1 | 1199 |
| 939 | 7 | 23481211 | 23658110 | loss-gain | 1200 | 23524601 | 23525800 | DEL | region2inRegion1 | 1199 |
| 938 | 7 | 23307667 | 23658110 | loss-gain | 800 | 23527401 | 23528200 | DEL | region2inRegion1 | 799 |

| | | | | | | | | | | |
|---|---|---|---|---|---|---|---|---|---|---|
| 939 | 7 | 23481211 | 23658110 | loss-gain | 800 | 23527401 | 23528200 | DEL | region2inRegion1 | 799 |
| 938 | 7 | 23307667 | 23658110 | loss-gain | 2400 | 23545001 | 23547400 | DUP | region2inRegion1 | 2399 |
| 939 | 7 | 23481211 | 23658110 | loss-gain | 2400 | 23545001 | 23547400 | DUP | region2inRegion1 | 2399 |
| 938 | 7 | 23307667 | 23658110 | loss-gain | 1400 | 23547201 | 23548600 | DEL | region2inRegion1 | 1399 |
| 939 | 7 | 23481211 | 23658110 | loss-gain | 1400 | 23547201 | 23548600 | DEL | region2inRegion1 | 1399 |
| 938 | 7 | 23307667 | 23658110 | loss-gain | 1200 | 23569601 | 23570800 | DUP | region2inRegion1 | 1199 |
| 939 | 7 | 23481211 | 23658110 | loss-gain | 1200 | 23569601 | 23570800 | DUP | region2inRegion1 | 1199 |
| 938 | 7 | 23307667 | 23658110 | loss-gain | 800 | 23587001 | 23587800 | DEL | region2inRegion1 | 799 |
| 939 | 7 | 23481211 | 23658110 | loss-gain | 800 | 23587001 | 23587800 | DEL | region2inRegion1 | 799 |
| 938 | 7 | 23307667 | 23658110 | loss-gain | 800 | 23588001 | 23588800 | DEL | region2inRegion1 | 799 |
| 939 | 7 | 23481211 | 23658110 | loss-gain | 800 | 23588001 | 23588800 | DEL | region2inRegion1 | 799 |
| 938 | 7 | 23307667 | 23658110 | loss-gain | 800 | 23590601 | 23591400 | DEL | region2inRegion1 | 799 |
| 939 | 7 | 23481211 | 23658110 | loss-gain | 800 | 23590601 | 23591400 | DEL | region2inRegion1 | 799 |
| 938 | 7 | 23307667 | 23658110 | loss-gain | 1000 | 23596601 | 23597600 | DEL | region2inRegion1 | 999 |
| 939 | 7 | 23481211 | 23658110 | loss-gain | 1000 | 23596601 | 23597600 | DEL | region2inRegion1 | 999 |
| 938 | 7 | 23307667 | 23658110 | loss-gain | 1200 | 23597801 | 23599000 | DEL | region2inRegion1 | 1199 |
| 939 | 7 | 23481211 | 23658110 | loss-gain | 1200 | 23597801 | 23599000 | DEL | region2inRegion1 | 1199 |
| 942 | 7 | 24648309 | 24653067 | loss-gain | 1600 | 24647401 | 24649000 | DUP | overlapHead | 692 |
| 942 | 7 | 24648309 | 24653067 | loss-gain | 1400 | 24650201 | 24651600 | DUP | region2inRegion1 | 1399 |

| | | | | | | | | | | |
|---|---|---|---|---|---|---|---|---|---|---|
| 942 | 7 | 24648309 | 24653067 | loss-gain | 1400 | 24652001 | 24653400 | DUP | overlapTail | 1067 |
| 943 | 7 | 24735665 | 24740397 | gain | 1200 | 24735401 | 24736600 | DUP | overlapHead | 936 |
| 944 | 7 | 25359190 | 25399904 | gain | 6400 | 25358001 | 25364400 | DUP | overlapHead | 5211 |
| 944 | 7 | 25359190 | 25399904 | gain | 4400 | 25364801 | 25369200 | DUP | region2inRegion1 | 4399 |
| 944 | 7 | 25359190 | 25399904 | gain | 193400 | 25369601 | 25563000 | DUP | overlapTail | 30304 |
| 945 | 7 | 25488796 | 25557156 | gain | 193400 | 25369601 | 25563000 | DUP | region1inRegion2 | 68360 |
| 946 | 7 | 25785853 | 25888939 | gain | 19200 | 25766801 | 25786000 | DUP | overlapHead | 148 |
| 946 | 7 | 25785853 | 25888939 | gain | 2000 | 25786201 | 25788200 | DUP | region2inRegion1 | 1999 |
| 946 | 7 | 25785853 | 25888939 | gain | 800 | 25790201 | 25791000 | DUP | region2inRegion1 | 799 |
| 946 | 7 | 25785853 | 25888939 | gain | 2600 | 25793601 | 25796200 | DUP | region2inRegion1 | 2599 |
| 946 | 7 | 25785853 | 25888939 | gain | 8000 | 25846201 | 25854200 | DUP | region2inRegion1 | 7999 |
| 946 | 7 | 25785853 | 25888939 | gain | 7000 | 25855801 | 25862800 | DUP | region2inRegion1 | 6999 |
| 946 | 7 | 25785853 | 25888939 | gain | 4400 | 25863201 | 25867600 | DUP | region2inRegion1 | 4399 |
| 946 | 7 | 25785853 | 25888939 | gain | 3400 | 25868001 | 25871400 | DUP | region2inRegion1 | 3399 |
| 946 | 7 | 25785853 | 25888939 | gain | 30200 | 25873201 | 25903400 | DUP | overlapTail | 15739 |
| 949 | 7 | 26267062 | 26297673 | gain | 800 | 26266801 | 26267600 | DUP | overlapHead | 539 |
| 949 | 7 | 26267062 | 26297673 | gain | 1000 | 26269001 | 26270000 | DUP | region2inRegion1 | 999 |
| 949 | 7 | 26267062 | 26297673 | gain | 800 | 26270401 | 26271200 | DUP | region2inRegion1 | 799 |
| 949 | 7 | 26267062 | 26297673 | gain | 800 | 26271401 | 26272200 | DUP | region2inRegion1 | 799 |

| | | | | | | | | | | |
|---|---|---|---|---|---|---|---|---|---|---|
| 949 | 7 | 26267062 | 26297673 | gain | 800 | 26273001 | 26273800 | DUP | region2inRegion1 | 799 |
| 949 | 7 | 26267062 | 26297673 | gain | 1200 | 26274801 | 26276000 | DUP | region2inRegion1 | 1199 |
| 949 | 7 | 26267062 | 26297673 | gain | 1400 | 26276801 | 26278200 | DUP | region2inRegion1 | 1399 |
| 949 | 7 | 26267062 | 26297673 | gain | 3000 | 26283801 | 26286800 | DUP | region2inRegion1 | 2999 |
| 949 | 7 | 26267062 | 26297673 | gain | 800 | 26287001 | 26287800 | DUP | region2inRegion1 | 799 |
| 949 | 7 | 26267062 | 26297673 | gain | 1400 | 26290201 | 26291600 | DUP | region2inRegion1 | 1399 |
| 949 | 7 | 26267062 | 26297673 | gain | 2400 | 26293601 | 26296000 | DUP | region2inRegion1 | 2399 |
| 949 | 7 | 26267062 | 26297673 | gain | 800 | 26297001 | 26297800 | DUP | overlapTail | 673 |
| 951 | 7 | 26600964 | 26608495 | gain | 2600 | 26599001 | 26601600 | DUP | overlapHead | 637 |
| 951 | 7 | 26600964 | 26608495 | gain | 1000 | 26603801 | 26604800 | DUP | region2inRegion1 | 999 |
| 951 | 7 | 26600964 | 26608495 | gain | 2600 | 26606201 | 26608800 | DUP | overlapTail | 2295 |
| 957 | 7 | 28525787 | 28565312 | gain | 2400 | 28523401 | 28525800 | DUP | overlapHead | 14 |
| 957 | 7 | 28525787 | 28565312 | gain | 1800 | 28531601 | 28533400 | DUP | region2inRegion1 | 1799 |
| 957 | 7 | 28525787 | 28565312 | gain | 2200 | 28533801 | 28536000 | DUP | region2inRegion1 | 2199 |
| 957 | 7 | 28525787 | 28565312 | gain | 800 | 28536801 | 28537600 | DUP | region2inRegion1 | 799 |
| 957 | 7 | 28525787 | 28565312 | gain | 2200 | 28538001 | 28540200 | DUP | region2inRegion1 | 2199 |
| 957 | 7 | 28525787 | 28565312 | gain | 1600 | 28546001 | 28547600 | DEL | region2inRegion1 | 1599 |
| 957 | 7 | 28525787 | 28565312 | gain | 1400 | 28554401 | 28555800 | DEL | region2inRegion1 | 1399 |
| 957 | 7 | 28525787 | 28565312 | gain | 1000 | 28556201 | 28557200 | DEL | region2inRegion1 | 999 |

| | | | | | | | | | | | |
|---|---|---|---|---|---|---|---|---|---|---|---|
| 972 | 7 | 58579811 | 58584542 | gain | 3000 | 58581401 | 58584400 | DUP | region2inRegion1 | 2999 |
| 975 | 7 | 59278633 | 59284219 | loss | 1000 | 59282401 | 59283400 | DEL | region2inRegion1 | 999 |
| 977 | 7 | 61355441 | 61417944 | gain | 1000 | 61371001 | 61372000 | DUP | region2inRegion1 | 999 |
| 977 | 7 | 61355441 | 61417944 | gain | 1200 | 61377601 | 61378800 | DEL | region2inRegion1 | 1199 |
| 994 | 7 | 82234623 | 82374446 | gain | 352000 | 82227601 | 82579600 | DUP | region1inRegion2 | 139823 |
| 1002 | 7 | 84728780 | 84749202 | gain | 2200 | 84731401 | 84733600 | DUP | region2inRegion1 | 2199 |
| 1002 | 7 | 84728780 | 84749202 | gain | 1200 | 84735401 | 84736600 | DUP | region2inRegion1 | 1199 |
| 1002 | 7 | 84728780 | 84749202 | gain | 1000 | 84740201 | 84741200 | DUP | region2inRegion1 | 999 |
| 1002 | 7 | 84728780 | 84749202 | gain | 2200 | 84743001 | 84745200 | DUP | region2inRegion1 | 2199 |
| 1002 | 7 | 84728780 | 84749202 | gain | 1200 | 84745801 | 84747000 | DUP | region2inRegion1 | 1199 |
| 1011 | 7 | 102790067 | 102823288 | gain | 1400 | 102788801 | 102790200 | DUP | overlapHead | 134 |
| 1011 | 7 | 102790067 | 102823288 | gain | 1000 | 102791601 | 102792600 | DUP | region2inRegion1 | 999 |
| 1011 | 7 | 102790067 | 102823288 | gain | 1400 | 102793001 | 102794400 | DUP | region2inRegion1 | 1399 |
| 1011 | 7 | 102790067 | 102823288 | gain | 2200 | 102800801 | 102803000 | DUP | region2inRegion1 | 2199 |
| 1011 | 7 | 102790067 | 102823288 | gain | 1000 | 102806601 | 102807600 | DUP | region2inRegion1 | 999 |
| 1011 | 7 | 102790067 | 102823288 | gain | 800 | 102811001 | 102811800 | DUP | region2inRegion1 | 799 |
| 1011 | 7 | 102790067 | 102823288 | gain | 2400 | 102813001 | 102815400 | DUP | region2inRegion1 | 2399 |
| 1011 | 7 | 102790067 | 102823288 | gain | 1200 | 102818001 | 102819200 | DUP | region2inRegion1 | 1199 |
| 1011 | 7 | 102790067 | 102823288 | gain | 1400 | 102819801 | 102821200 | DUP | region2inRegion1 | 1399 |

| | | | | | | | | | | |
|---|---|---|---|---|---|---|---|---|---|---|
| 1011 | 7 | 102790067 | 102823288 | gain | 1800 | 102822001 | 102823800 | DUP | overlapTail | 1288 |
| 1021 | 7 | 111620166 | 111623056 | loss | 800 | 111620801 | 111621600 | DEL | region2inRegion1 | 799 |
| 1035 | 7 | 131449863 | 131460402 | gain | 12400 | 131446601 | 131459000 | DUP | overlapHead | 9138 |
| 1040 | 7 | 132961208 | 132975832 | loss | 800 | 132967601 | 132968400 | DUP | region2inRegion1 | 799 |
| 1041 | 7 | 132985953 | 133003168 | loss | 1200 | 132990201 | 132991400 | DUP | region2inRegion1 | 1199 |
| 1041 | 7 | 132985953 | 133003168 | loss | 800 | 132993001 | 132993800 | DUP | region2inRegion1 | 799 |
| 1059 | 8 | 26029928 | 26034839 | loss | 4400 | 26031201 | 26035600 | DEL | overlapTail | 3639 |
| 1068 | 8 | 30016900 | 30024587 | loss | 1000 | 30016401 | 30017400 | DEL | overlapHead | 501 |
| 1068 | 8 | 30016900 | 30024587 | loss | 7000 | 30018001 | 30025000 | DEL | overlapTail | 6587 |
| 1086 | 8 | 44371710 | 44447879 | gain | 800 | 44375001 | 44375800 | DUP | region2inRegion1 | 799 |
| 1086 | 8 | 44371710 | 44447879 | gain | 1000 | 44376801 | 44377800 | DUP | region2inRegion1 | 999 |
| 1086 | 8 | 44371710 | 44447879 | gain | 800 | 44379401 | 44380200 | DUP | region2inRegion1 | 799 |
| 1086 | 8 | 44371710 | 44447879 | gain | 2600 | 44381401 | 44384000 | DUP | region2inRegion1 | 2599 |
| 1086 | 8 | 44371710 | 44447879 | gain | 1400 | 44384601 | 44386000 | DUP | region2inRegion1 | 1399 |
| 1086 | 8 | 44371710 | 44447879 | gain | 1200 | 44386401 | 44387600 | DUP | region2inRegion1 | 1199 |
| 1086 | 8 | 44371710 | 44447879 | gain | 1000 | 44389201 | 44390200 | DUP | region2inRegion1 | 999 |
| 1086 | 8 | 44371710 | 44447879 | gain | 1000 | 44391001 | 44392000 | DUP | region2inRegion1 | 999 |
| 1086 | 8 | 44371710 | 44447879 | gain | 1400 | 44397801 | 44399200 | DUP | region2inRegion1 | 1399 |
| 1086 | 8 | 44371710 | 44447879 | gain | 1600 | 44401401 | 44403000 | DUP | region2inRegion1 | 1599 |

| | | | | | | | | | | |
|---|---|---|---|---|---|---|---|---|---|---|
| 1086 | 8 | 44371710 | 44447879 | gain | 800 | 44411201 | 44412000 | DUP | region2inRegion1 | 799 |
| 1086 | 8 | 44371710 | 44447879 | gain | 800 | 44412801 | 44413600 | DUP | region2inRegion1 | 799 |
| 1086 | 8 | 44371710 | 44447879 | gain | 1000 | 44418201 | 44419200 | DUP | region2inRegion1 | 999 |
| 1086 | 8 | 44371710 | 44447879 | gain | 1200 | 44424801 | 44426000 | DUP | region2inRegion1 | 1199 |
| 1086 | 8 | 44371710 | 44447879 | gain | 800 | 44426601 | 44427400 | DUP | region2inRegion1 | 799 |
| 1086 | 8 | 44371710 | 44447879 | gain | 2600 | 44428001 | 44430600 | DUP | region2inRegion1 | 2599 |
| 1086 | 8 | 44371710 | 44447879 | gain | 1200 | 44431201 | 44432400 | DUP | region2inRegion1 | 1199 |
| 1086 | 8 | 44371710 | 44447879 | gain | 800 | 44432401 | 44433200 | DUP | region2inRegion1 | 799 |
| 1086 | 8 | 44371710 | 44447879 | gain | 800 | 44434601 | 44435400 | DUP | region2inRegion1 | 799 |
| 1086 | 8 | 44371710 | 44447879 | gain | 1600 | 44436001 | 44437600 | DUP | region2inRegion1 | 1599 |
| 1086 | 8 | 44371710 | 44447879 | gain | 1000 | 44438001 | 44439000 | DUP | region2inRegion1 | 999 |
| 1086 | 8 | 44371710 | 44447879 | gain | 1200 | 44442201 | 44443400 | DUP | region2inRegion1 | 1199 |
| 1086 | 8 | 44371710 | 44447879 | gain | 1600 | 44446801 | 44448400 | DUP | overlapTail | 1079 |
| 1112 | 8 | 64473379 | 64515977 | loss | 1000 | 64511401 | 64512400 | DUP | region2inRegion1 | 999 |
| 1198 | 9 | 1963636 | 2010455 | gain | 1200 | 1965001 | 1966200 | DUP | region2inRegion1 | 1199 |
| 1198 | 9 | 1963636 | 2010455 | gain | 1200 | 1967001 | 1968200 | DUP | region2inRegion1 | 1199 |
| 1198 | 9 | 1963636 | 2010455 | gain | 1400 | 1970601 | 1972000 | DUP | region2inRegion1 | 1399 |
| 1198 | 9 | 1963636 | 2010455 | gain | 1000 | 1977601 | 1978600 | DUP | region2inRegion1 | 999 |
| 1198 | 9 | 1963636 | 2010455 | gain | 800 | 1980201 | 1981000 | DUP | region2inRegion1 | 799 |

| | | | | | | | | | | | |
|---|---|---|---|---|---|---|---|---|---|---|---|
| 1198 | 9 | 1963636 | 2010455 | gain | 1600 | 1986401 | 1988000 | DUP | region2inRegion1 | 1599 |
| 1198 | 9 | 1963636 | 2010455 | gain | 800 | 1988601 | 1989400 | DUP | region2inRegion1 | 799 |
| 1198 | 9 | 1963636 | 2010455 | gain | 2000 | 1990401 | 1992400 | DUP | region2inRegion1 | 1999 |
| 1198 | 9 | 1963636 | 2010455 | gain | 800 | 1992601 | 1993400 | DUP | region2inRegion1 | 799 |
| 1198 | 9 | 1963636 | 2010455 | gain | 1400 | 1993601 | 1995000 | DUP | region2inRegion1 | 1399 |
| 1198 | 9 | 1963636 | 2010455 | gain | 1200 | 1995401 | 1996600 | DUP | region2inRegion1 | 1199 |
| 1198 | 9 | 1963636 | 2010455 | gain | 800 | 1996401 | 1997200 | DEL | region2inRegion1 | 799 |
| 1198 | 9 | 1963636 | 2010455 | gain | 800 | 2000201 | 2001000 | DUP | region2inRegion1 | 799 |
| 1198 | 9 | 1963636 | 2010455 | gain | 1000 | 2001201 | 2002200 | DUP | region2inRegion1 | 999 |
| 1198 | 9 | 1963636 | 2010455 | gain | 800 | 2007601 | 2008400 | DEL | region2inRegion1 | 799 |
| 1200 | 9 | 3614907 | 3618335 | gain | 2000 | 3614001 | 3616000 | DUP | overlapHead | 1094 |
| 1203 | 9 | 5206397 | 5225462 | gain | 1000 | 5209201 | 5210200 | DUP | region2inRegion1 | 999 |
| 1203 | 9 | 5206397 | 5225462 | gain | 800 | 5211001 | 5211800 | DUP | region2inRegion1 | 799 |
| 1203 | 9 | 5206397 | 5225462 | gain | 800 | 5213001 | 5213800 | DUP | region2inRegion1 | 799 |
| 1203 | 9 | 5206397 | 5225462 | gain | 800 | 5214401 | 5215200 | DUP | region2inRegion1 | 799 |
| 1203 | 9 | 5206397 | 5225462 | gain | 1000 | 5215401 | 5216400 | DUP | region2inRegion1 | 999 |
| 1203 | 9 | 5206397 | 5225462 | gain | 1400 | 5216601 | 5218000 | DUP | region2inRegion1 | 1399 |
| 1203 | 9 | 5206397 | 5225462 | gain | 800 | 5218801 | 5219600 | DUP | region2inRegion1 | 799 |
| 1203 | 9 | 5206397 | 5225462 | gain | 1200 | 5220001 | 5221200 | DUP | region2inRegion1 | 1199 |

| | | | | | | | | | | | |
|---|---|---|---|---|---|---|---|---|---|---|---|
| 1203 | 9 | 5206397 | 5225462 | gain | 1000 | 5223801 | 5224800 | DUP | region2inRegion1 | 999 |
| 1204 | 9 | 5488035 | 5492918 | gain | 1200 | 5487001 | 5488200 | DUP | overlapHead | 166 |
| 1204 | 9 | 5488035 | 5492918 | gain | 1000 | 5492401 | 5493400 | DUP | overlapTail | 518 |
| 1205 | 9 | 5747061 | 5810398 | loss | 2000 | 5767601 | 5769600 | DUP | region2inRegion1 | 1999 |
| 1205 | 9 | 5747061 | 5810398 | loss | 1200 | 5790801 | 5792000 | DUP | region2inRegion1 | 1199 |
| 1261 | 9 | 56272660 | 56332060 | gain | 1800 | 56282601 | 56284400 | DEL | region2inRegion1 | 1799 |
| 1261 | 9 | 56272660 | 56332060 | gain | 800 | 56285201 | 56286000 | DEL | region2inRegion1 | 799 |
| 1261 | 9 | 56272660 | 56332060 | gain | 800 | 56286801 | 56287600 | DEL | region2inRegion1 | 799 |
| 1261 | 9 | 56272660 | 56332060 | gain | 2600 | 56294401 | 56297000 | DUP | region2inRegion1 | 2599 |
| 1261 | 9 | 56272660 | 56332060 | gain | 800 | 56300401 | 56301200 | DUP | region2inRegion1 | 799 |
| 1261 | 9 | 56272660 | 56332060 | gain | 800 | 56305201 | 56306000 | DEL | region2inRegion1 | 799 |
| 1261 | 9 | 56272660 | 56332060 | gain | 800 | 56306601 | 56307400 | DUP | region2inRegion1 | 799 |
| 1261 | 9 | 56272660 | 56332060 | gain | 1000 | 56311401 | 56312400 | DEL | region2inRegion1 | 999 |
| 1261 | 9 | 56272660 | 56332060 | gain | 800 | 56314001 | 56314800 | DUP | region2inRegion1 | 799 |
| 1261 | 9 | 56272660 | 56332060 | gain | 800 | 56323001 | 56323800 | DUP | region2inRegion1 | 799 |
| 1262 | 9 | 56901872 | 56950458 | loss | 800 | 56933601 | 56934400 | DEL | region2inRegion1 | 799 |
| 1262 | 9 | 56901872 | 56950458 | loss | 1000 | 56941201 | 56942200 | DEL | region2inRegion1 | 999 |
| 1331 | 9 | 119585370 | 119593230 | gain | 1200 | 119584401 | 119585600 | DUP | overlapHead | 231 |
| 1331 | 9 | 119585370 | 119593230 | gain | 1800 | 119586001 | 119587800 | DUP | region2inRegion1 | 1799 |
