# Supplementary material for: Identification of copy number variations using high density whole-genome single nucleotide polymorphism markers in Chinese Dongxiang spotted pigs
Source: Asian-Australas J Anim Sci. 2019 Feb 7;32(12):1809–15. doi: 10.5713/ajas.18.0696 (PMC6819687; doi:10.5713/ajas.18.0696)
Supplement: Supplementary file 3 [file ajas-18-0696-suppl3.pdf]

Table S3. Detailed comparison of CNVRs identified in this studies with those in 11 previous studies

**Our results**						**Previous results**				
CNVR ID	CNV ID	Chr.	Start1	End1	Type	CNV ID	Start2	End2	Type	Relationship
CNVR3	92	1	95316086	95335767	gain	CNVR21_Chen	92347854	100449909	gain/loss	region1inRegion2
CNVR4	99	1	99687218	99704572	gain	CNVR21_Chen	92347854	100449909	gain/loss	region1inRegion2
CNVR5	101	1	99733649	99785971	gain	CNVR21_Chen	92347854	100449909	gain/loss	region1inRegion2
CNVR6	103	1	100019309	100027849	gain	CNVR21_Chen	92347854	100449909	gain/loss	region1inRegion2
CNVR12	261	1	268054795	268056507	gain	CNVR41_Chen	267977629	268124492	loss	region1inRegion2
CNVR14	279	1	284447951	284512355	loss	CNVR47_Chen	284386253	284552738	gain/loss	region1inRegion2
CNVR15	288	1	295235629	295379020	gain	CNVR50_Chen	294821198	295462741	gain/loss	region1inRegion2
CNVR16	290	1	296193901	296202305	loss-gain	CNVR51_Chen	295676469	297172866	gain/loss	region1inRegion2
CNVR17	292	1	296918534	296982162	loss	CNVR51_Chen	295676469	297172866	gain/loss	region1inRegion2
CNVR18	298	1	312363155	312415324	gain	CNVR58_Chen	312227906	312829468	gain/loss	region1inRegion2
CNVR19	299	1	312415324	312437693	gain	CNVR58_Chen	312227906	312829468	gain/loss	region1inRegion2
CNVR23	319	2	14000848	14032642	loss-gain	CNVR66_Chen	13743143	14105818	gain/loss	region1inRegion2
CNVR28	353	2	56935884	56944870	loss	CNVR79_Chen	56784798	57021596	loss	region1inRegion2
CNVR39	467	2	149095217	149101135	loss	CNVR94_Chen	149012060	149184709	gain	region1inRegion2
CNVR54	589	3	144724140	144783726	gain	CNVR133_Chen	144353522	144740773	gain	overlapHead
CNVR61	730	5	21242609	21260003	gain	CNVR161_Chen	21238084	22568532	gain/loss	region1inRegion2

CNVR62	731	5	21332629	21368821	gain	CNVR161_Chen	21238084	22568532	gain/loss	region1inRegion2
CNVR63	736	5	22004036	22012384	loss	CNVR161_Chen	21238084	22568532	gain/loss	region1inRegion2
CNVR64	739	5	22410640	22421100	gain	CNVR161_Chen	21238084	22568532	gain/loss	region1inRegion2
CNVR77	943	7	24735665	24740397	gain	CNVR235_Chen	24703970	24777963	gain	region1inRegion2
CNVR78	944	7	25359190	25399904	gain	CNVR236_Chen	25171813	26269643	gain/loss	region1inRegion2
CNVR79	945	7	25488796	25557156	gain	CNVR236_Chen	25171813	26269643	gain/loss	region1inRegion2
CNVR80	946	7	25785853	25888939	gain	CNVR236_Chen	25171813	26269643	gain/loss	region1inRegion2
CNVR81	949	7	26267062	26297673	gain	CNVR236_Chen	25171813	26269643	gain/loss	overlapHead
CNVR87	994	7	82234623	82374446	gain	CNVR242_Chen	82147895	82456371	gain	region1inRegion2
CNVR90	1021	7	111620166	111623056	loss	CNVR250_Chen	111308562	111876616	gain	region1inRegion2
CNVR103	1200	9	3614907	3618335	gain	CNVR290_Chen	3598721	3721967	gain	region1inRegion2
CNVR104	1203	9	5206397	5225462	gain	CNVR291_Chen	5186145	5532623	gain	region1inRegion2
CNVR105	1204	9	5488035	5492918	gain	CNVR291_Chen	5186145	5532623	gain	region1inRegion2
CNVR106	1205	9	5747061	5810398	loss	CNVR292_Chen	5757660	5851208	loss	overlapTail
CNVR122	1436	11	8308242	8320582	gain	CNVR339_Chen	8253141	8372688	gain	region1inRegion2
CNVR125	1457	11	32658621	32711857	loss	CNVR349_Chen	32581919	32878292	gain/loss	region1inRegion2
CNVR129	1518	11	70689891	70709813	loss	CNVR365_Chen	70508069	71882063	gain/loss	region1inRegion2
CNVR130	1520	11	71317528	71375741	loss	CNVR365_Chen	70508069	71882063	gain/loss	region1inRegion2
CNVR131	1524	11	71878763	71887995	gain	CNVR365_Chen	70508069	71882063	gain/loss	overlapHead

CNVR132	1555	12	50924636	50947287	gain	CNVR383_Chen	50854966	50930355	gain	overlapHead
CNVR14	279	1	284447951	284512355	loss	CNVR7_Dong	284443528	284486575	both	overlapHead
CNVR15	288	1	295235629	295379020	gain	CNVR8_Dong	294821198	295462741	gain	region1inRegion2
CNVR18	298	1	312363155	312415324	gain	CNVR11_Dong	312227906	312613914	both	region1inRegion2
CNVR19	299	1	312415324	312437693	gain	CNVR11_Dong	312227906	312613914	both	region1inRegion2
CNVR38	466	2	148904898	148913506	loss-gain	CNVR20_Dong	148906133	149106718	gain	overlapTail
CNVR39	467	2	149095217	149101135	loss	CNVR20_Dong	148906133	149106718	gain	region1inRegion2
CNVR61	730	5	21242609	21260003	gain	CNVR32_Dong	21238084	21339891	gain	region1inRegion2
CNVR62	731	5	21332629	21368821	gain	CNVR32_Dong	21238084	21339891	gain	overlapHead
CNVR80	946	7	25785853	25888939	gain	CNVR44_Dong	25773312	26187687	both	region1inRegion2
CNVR103	1200	9	3614907	3618335	gain	CNVR53_Dong	3598721	3721967	gain	region1inRegion2
CNVR106	1205	9	5747061	5810398	loss	CNVR54_Dong	5757660	5815331	loss	overlapTail
CNVR129	1518	11	70689891	70709813	loss	CNVR64_Dong	70384747	71641281	gain	region1inRegion2
CNVR130	1520	11	71317528	71375741	loss	CNVR64_Dong	70384747	71641281	gain	region1inRegion2
CNVR131	1524	11	71878763	71887995	gain	CNVR65_Dong	71882063	71912745	gain	overlapTail
CNVR132	1555	12	50924636	50947287	gain	CNVR68_Dong	50892615	50930355	gain	overlapHead
CNVR15	288	1	295235629	295379020	gain	7_Fernandez	295241692	295462741	loss-gain	overlapTail
CNVR54	589	3	144724140	144783726	gain	11_Fernandez	144514114	144742787	loss	overlapHead
CNVR64	739	5	22410640	22421100	gain	13_Fernandez	22169288	22568532	loss	region1inRegion2

CNVR87	994	7	82234623	82374446	gain	25_Fernandez	82305261	82585580	gain	overlapTail
CNVR129	1518	11	70689891	70709813	loss	35_Fernandez	70656747	71089190	loss-gain	region1inRegion2
CNVR147	1782	14	8039487	8116098	gain	46_Fernandez	7976149	8116098	gain	region1inRegion2
CNVR10	220	1	224697068	224765844	gain	CNVR223_Paudel	224693001	224700000	NA	overlapHead
CNVR13	266	1	271529620	271536665	gain	CNVR266_Paudel	271523001	271535000	NA	overlapHead
CNVR14	279	1	284447951	284512355	loss	CNVR287_Paudel	284434001	284469000	NA	overlapHead
CNVR14	279	1	284447951	284512355	loss	CNVR288_Paudel	284490001	284501000	NA	region2inRegion1
CNVR14	279	1	284447951	284512355	loss	CNVR289_Paudel	284509001	284516000	NA	overlapTail
CNVR15	288	1	295235629	295379020	gain	CNVR304_Paudel	295238001	295261000	NA	region2inRegion1
CNVR15	288	1	295235629	295379020	gain	CNVR305_Paudel	295272001	295280000	NA	region2inRegion1
CNVR15	288	1	295235629	295379020	gain	CNVR306_Paudel	295375001	295383000	NA	overlapTail
CNVR16	290	1	296193901	296202305	loss-gain	CNVR318_Paudel	296197001	296214000	NA	overlapTail
CNVR17	292	1	296918534	296982162	loss	CNVR333_Paudel	296924001	296931000	NA	region2inRegion1
CNVR17	292	1	296918534	296982162	loss	CNVR334_Paudel	296946001	296952000	NA	region2inRegion1
CNVR17	292	1	296918534	296982162	loss	CNVR335_Paudel	296959001	296967000	NA	region2inRegion1
CNVR17	292	1	296918534	296982162	loss	CNVR336_Paudel	296969001	296975000	NA	region2inRegion1
CNVR18	298	1	312363155	312415324	gain	CNVR359_Paudel	312353001	312430000	NA	region1inRegion2
CNVR19	299	1	312415324	312437693	gain	CNVR359_Paudel	312353001	312430000	NA	overlapHead
CNVR116	1380	10	22431073	22524502	gain	CNVR433_Paudel	22460001	22477000	NA	region2inRegion1

CNVR116	1380	10	22431073	22524502	gain	CNVR434_Paudel	22503001	22510000	NA	region2inRegion1
CNVR116	1380	10	22431073	22524502	gain	CNVR435_Paudel	22513001	22519000	NA	region2inRegion1
CNVR117	1381	10	23114909	23120232	loss-gain	CNVR450_Paudel	23104001	23121000	NA	region1inRegion2
CNVR120	1431	10	79004773	79065598	gain	CNVR522_Paudel	78992001	79021000	NA	overlapHead
CNVR120	1431	10	79004773	79065598	gain	CNVR523_Paudel	79023001	79039000	NA	region2inRegion1
CNVR120	1431	10	79004773	79065598	gain	CNVR524_Paudel	79044001	79068000	NA	overlapTail
CNVR126	1468	11	38006863	38074356	gain	CNVR569_Paudel	38056001	38067000	NA	region2inRegion1
CNVR129	1518	11	70689891	70709813	loss	CNVR625_Paudel	70708001	70719000	NA	overlapTail
CNVR130	1520	11	71317528	71375741	loss	CNVR639_Paudel	71290001	71319000	NA	overlapHead
CNVR130	1520	11	71317528	71375741	loss	CNVR640_Paudel	71322001	71331000	NA	region2inRegion1
CNVR130	1520	11	71317528	71375741	loss	CNVR641_Paudel	71333001	71364000	NA	region2inRegion1
CNVR131	1524	11	71878763	71887995	gain	CNVR651_Paudel	71868001	71886000	NA	overlapHead
CNVR132	1555	12	50924636	50947287	gain	CNVR699_Paudel	50925001	50944000	NA	region2inRegion1
CNVR132	1555	12	50924636	50947287	gain	CNVR700_Paudel	50946001	50956000	NA	overlapTail
CNVR133	1556	12	51099806	51123003	gain	CNVR705_Paudel	51101001	51124000	NA	overlapTail
CNVR143	1699	13	170765271	170819670	gain	CNVR897_Paudel	170768001	170780000	NA	region2inRegion1
CNVR144	1702	13	171285806	171311772	gain	CNVR904_Paudel	171282001	171288000	NA	overlapHead
CNVR146	1781	14	7794851	7822418	gain	CNVR994_Paudel	7795001	7801000	NA	region2inRegion1
CNVR148	1792	14	21068045	21091201	gain	CNVR1009_Paudel	21080001	21094000	NA	overlapTail

CNVR153	1849	14	94594494	94629904	gain	CNVR1070_Paudel	94620001	94636000	NA	overlapTail
CNVR154	1873	14	120810562	120829324	gain	CNVR1113_Paudel	120813001	120827000	NA	region2inRegion1
CNVR159	2034	16	6146177	6154424	loss	CNVR1340_Paudel	6140001	6153000	NA	overlapHead
CNVR20	312	2	11511074	11530117	gain	CNVR1550_Paudel	11501001	11543000	NA	region1inRegion2
CNVR22	316	2	12689951	12700771	loss	CNVR1563_Paudel	12685001	12707000	NA	region1inRegion2
CNVR23	319	2	14000848	14032642	loss-gain	CNVR1570_Paudel	13981001	14025000	NA	overlapHead
CNVR23	319	2	14000848	14032642	loss-gain	CNVR1571_Paudel	14029001	14044000	NA	overlapTail
CNVR24	320	2	14728311	14771718	gain	CNVR1585_Paudel	14729001	14736000	NA	region2inRegion1
CNVR24	320	2	14728311	14771718	gain	CNVR1586_Paudel	14738001	14748000	NA	region2inRegion1
CNVR24	320	2	14728311	14771718	gain	CNVR1587_Paudel	14750001	14758000	NA	region2inRegion1
CNVR24	320	2	14728311	14771718	gain	CNVR1588_Paudel	14763001	14775000	NA	overlapTail
CNVR25	321	2	15024071	15060448	loss	CNVR1591_Paudel	15018001	15025000	NA	overlapHead
CNVR25	321	2	15024071	15060448	loss	CNVR1592_Paudel	15030001	15037000	NA	region2inRegion1
CNVR25	321	2	15024071	15060448	loss	CNVR1593_Paudel	15041001	15047000	NA	region2inRegion1
CNVR25	321	2	15024071	15060448	loss	CNVR1594_Paudel	15052001	15068000	NA	overlapTail
CNVR31	362	2	62416028	62428337	gain	CNVR1655_Paudel	62406001	62419000	NA	overlapHead
CNVR32	364	2	62628253	62738661	gain	CNVR1663_Paudel	62621001	62633000	NA	overlapHead
CNVR32	364	2	62628253	62738661	gain	CNVR1664_Paudel	62636001	62652000	NA	region2inRegion1
CNVR32	364	2	62628253	62738661	gain	CNVR1665_Paudel	62659001	62667000	NA	region2inRegion1

CNVR32	364	2	62628253	62738661	gain	CNVR1666_Paudel	62722001	62741000	NA	overlapTail
CNVR33	378	2	67915859	67952389	gain	CNVR1696_Paudel	67920001	67940000	NA	region2inRegion1
CNVR33	378	2	67915859	67952389	gain	CNVR1697_Paudel	67952001	67961000	NA	overlapTail
CNVR34	379	2	68061173	68181801	gain	CNVR1702_Paudel	68058001	68079000	NA	overlapHead
CNVR34	379	2	68061173	68181801	gain	CNVR1703_Paudel	68082001	68098000	NA	region2inRegion1
CNVR34	379	2	68061173	68181801	gain	CNVR1704_Paudel	68104001	68113000	NA	region2inRegion1
CNVR34	379	2	68061173	68181801	gain	CNVR1705_Paudel	68115001	68148000	NA	region2inRegion1
CNVR34	379	2	68061173	68181801	gain	CNVR1706_Paudel	68160001	68167000	NA	region2inRegion1
CNVR34	379	2	68061173	68181801	gain	CNVR1707_Paudel	68169001	68193000	NA	overlapTail
CNVR44	479	2	159094998	159110351	gain	CNVR1790_Paudel	159099001	159150000	NA	overlapTail
CNVR46	504	3	45023435	45241189	gain	CNVR1840_Paudel	45014001	45033000	NA	overlapHead
CNVR46	504	3	45023435	45241189	gain	CNVR1841_Paudel	45057001	45068000	NA	region2inRegion1
CNVR46	504	3	45023435	45241189	gain	CNVR1842_Paudel	45071001	45081000	NA	region2inRegion1
CNVR46	504	3	45023435	45241189	gain	CNVR1843_Paudel	45090001	45104000	NA	region2inRegion1
CNVR46	504	3	45023435	45241189	gain	CNVR1844_Paudel	45106001	45112000	NA	region2inRegion1
CNVR46	504	3	45023435	45241189	gain	CNVR1845_Paudel	45164001	45172000	NA	region2inRegion1
CNVR46	504	3	45023435	45241189	gain	CNVR1846_Paudel	45201001	45215000	NA	region2inRegion1
CNVR46	504	3	45023435	45241189	gain	CNVR1847_Paudel	45223001	45237000	NA	region2inRegion1
CNVR51	581	3	142899740	142925257	loss	CNVR1927_Paudel	142917001	142946000	NA	overlapTail

CNVR52	582	3	142999513	143009221	gain	CNVR1930_Paudel	142981001	143013000	NA	region1inRegion2
CNVR53	585	3	143590087	143597682	gain	CNVR1942_Paudel	143592001	143601000	NA	overlapTail
CNVR54	589	3	144724140	144783726	gain	CNVR1953_Paudel	144748001	144786000	NA	overlapTail
CNVR61	730	5	21242609	21260003	gain	CNVR2106_Paudel	21235001	21244000	NA	overlapHead
CNVR61	730	5	21242609	21260003	gain	CNVR2107_Paudel	21246001	21260000	NA	region2inRegion1
CNVR62	731	5	21332629	21368821	gain	CNVR2110_Paudel	21337001	21379000	NA	overlapTail
CNVR63	736	5	22004036	22012384	loss	CNVR2133_Paudel	22003001	22016000	NA	region1inRegion2
CNVR64	739	5	22410640	22421100	gain	CNVR2145_Paudel	22391001	22421000	NA	overlapHead
CNVR71	917	6	151994674	152015484	gain	CNVR2345_Paudel	151991001	152016000	NA	region1inRegion2
CNVR73	934	7	22207228	22236111	gain	CNVR2368_Paudel	22200001	22211000	NA	overlapHead
CNVR73	934	7	22207228	22236111	gain	CNVR2369_Paudel	22214001	22241000	NA	overlapTail
CNVR74	938	7	23307667	23658110	loss-gain	CNVR2378_Paudel	23323001	23335000	NA	region2inRegion1
CNVR74	938	7	23307667	23658110	loss-gain	CNVR2379_Paudel	23361001	23400000	NA	region2inRegion1
CNVR74	938	7	23307667	23658110	loss-gain	CNVR2380_Paudel	23513001	23522000	NA	region2inRegion1
CNVR75	939	7	23481211	23658110	loss-gain	CNVR2380_Paudel	23513001	23522000	NA	region2inRegion1
CNVR74	938	7	23307667	23658110	loss-gain	CNVR2381_Paudel	23529001	23548000	NA	region2inRegion1
CNVR75	939	7	23481211	23658110	loss-gain	CNVR2381_Paudel	23529001	23548000	NA	region2inRegion1
CNVR74	938	7	23307667	23658110	loss-gain	CNVR2382_Paudel	23554001	23563000	NA	region2inRegion1
CNVR75	939	7	23481211	23658110	loss-gain	CNVR2382_Paudel	23554001	23563000	NA	region2inRegion1

CNVR74	938	7	23307667	23658110	loss-gain	CNVR2383_Paudel	23566001	23580000	NA	region2inRegion1
CNVR75	939	7	23481211	23658110	loss-gain	CNVR2383_Paudel	23566001	23580000	NA	region2inRegion1
CNVR74	938	7	23307667	23658110	loss-gain	CNVR2384_Paudel	23587001	23601000	NA	region2inRegion1
CNVR75	939	7	23481211	23658110	loss-gain	CNVR2384_Paudel	23587001	23601000	NA	region2inRegion1
CNVR74	938	7	23307667	23658110	loss-gain	CNVR2385_Paudel	23651001	23671000	NA	overlapTail
CNVR75	939	7	23481211	23658110	loss-gain	CNVR2385_Paudel	23651001	23671000	NA	overlapTail
CNVR76	942	7	24648309	24653067	loss-gain	CNVR2394_Paudel	24653001	24663000	NA	overlapTail
CNVR77	943	7	24735665	24740397	gain	CNVR2399_Paudel	24735001	24745000	NA	region1inRegion2
CNVR78	944	7	25359190	25399904	gain	CNVR2401_Paudel	25357001	25380000	NA	overlapHead
CNVR78	944	7	25359190	25399904	gain	CNVR2402_Paudel	25382001	25420000	NA	overlapTail
CNVR79	945	7	25488796	25557156	gain	CNVR2406_Paudel	25480001	25492000	NA	overlapHead
CNVR79	945	7	25488796	25557156	gain	CNVR2407_Paudel	25509001	25523000	NA	region2inRegion1
CNVR79	945	7	25488796	25557156	gain	CNVR2408_Paudel	25528001	25538000	NA	region2inRegion1
CNVR79	945	7	25488796	25557156	gain	CNVR2409_Paudel	25542001	25561000	NA	overlapTail
CNVR80	946	7	25785853	25888939	gain	CNVR2417_Paudel	25759001	25786000	NA	overlapHead
CNVR80	946	7	25785853	25888939	gain	CNVR2418_Paudel	25855001	25903000	NA	overlapTail
CNVR81	949	7	26267062	26297673	gain	CNVR2431_Paudel	26266001	26275000	NA	overlapHead
CNVR81	949	7	26267062	26297673	gain	CNVR2432_Paudel	26289001	26299000	NA	overlapTail
CNVR85	975	7	59278633	59284219	loss	CNVR2468_Paudel	59241001	59281000	NA	overlapHead

CNVR87	994	7	82234623	82374446	gain	CNVR2482_Paudel	82228001	82243000	NA	overlapHead
CNVR87	994	7	82234623	82374446	gain	CNVR2483_Paudel	82246001	82255000	NA	region2inRegion1
CNVR87	994	7	82234623	82374446	gain	CNVR2484_Paudel	82258001	82268000	NA	region2inRegion1
CNVR87	994	7	82234623	82374446	gain	CNVR2485_Paudel	82274001	82293000	NA	region2inRegion1
CNVR87	994	7	82234623	82374446	gain	CNVR2486_Paudel	82296001	82314000	NA	region2inRegion1
CNVR87	994	7	82234623	82374446	gain	CNVR2487_Paudel	82320001	82328000	NA	region2inRegion1
CNVR87	994	7	82234623	82374446	gain	CNVR2488_Paudel	82330001	82350000	NA	region2inRegion1
CNVR87	994	7	82234623	82374446	gain	CNVR2489_Paudel	82353001	82392000	NA	overlapTail
CNVR89	1011	7	102790067	102823288	gain	CNVR2525_Paudel	102786001	102795000	NA	overlapHead
CNVR91	1035	7	131449863	131460402	gain	CNVR2537_Paudel	131455001	131466000	NA	overlapTail
CNVR102	1198	9	1963636	2010455	gain	CNVR2747_Paudel	1954001	1976000	NA	overlapHead
CNVR102	1198	9	1963636	2010455	gain	CNVR2748_Paudel	1998001	2030000	NA	overlapTail
CNVR105	1204	9	5488035	5492918	gain	CNVR2767_Paudel	5485001	5507000	NA	region1inRegion2
CNVR106	1205	9	5747061	5810398	loss	CNVR2770_Paudel	5758001	5764000	NA	region2inRegion1
CNVR109	1261	9	56272660	56332060	gain	CNVR2824_Paudel	56323001	56330000	NA	region2inRegion1
CNVR15	288	1	295235629	295379020	gain	36_Revilla	295244060	295251086	GAIN	region2inRegion1
CNVR18	298	1	312363155	312415324	gain	43_Revilla	312183024	313013937	GAIN	region1inRegion2
CNVR19	299	1	312415324	312437693	gain	43_Revilla	312183024	313013937	GAIN	region1inRegion2
CNVR23	319	2	14000848	14032642	loss-gain	57_Revilla	14008896	14020416	GAIN	region2inRegion1

CNVR25	321	2	15024071	15060448	loss	58_Revilla	14913236	15153792	GAIN	region1inRegion2
CNVR32	364	2	62628253	62738661	gain	74_Revilla	62615164	62652443	GAIN	overlapHead
CNVR34	379	2	68061173	68181801	gain	80_Revilla	68114844	68120460	GAIN	region2inRegion1
CNVR41	472	2	158544152	158558867	gain	94_Revilla	158551416	158612136	GAIN	overlapTail
CNVR42	473	2	158605784	158612295	gain	94_Revilla	158551416	158612136	GAIN	overlapHead
CNVR42	473	2	158605784	158629757	gain	94_Revilla	158551416	158612136	GAIN	overlapHead
CNVR42	473	2	158605784	158629757	gain	95_Revilla	158614974	159025436	GAIN	overlapTail
CNVR54	589	3	144724140	144783726	gain	131_Revilla	144773232	144786208	mixed	overlapTail
CNVR59	698	4	111405421	111446020	gain	151_Revilla	111407040	111420153	GAIN	region2inRegion1
CNVR61	730	5	21242609	21260003	gain	160_Revilla	21244896	21284214	GAIN	overlapTail
CNVR71	917	6	151994674	152015484	gain	214_Revilla	151988436	151995708	GAIN	overlapHead
CNVR78	944	7	25359190	25399904	gain	220_Revilla	25380332	25393908	GAIN	region2inRegion1
CNVR87	994	7	82234623	82374446	gain	233_Revilla	82260387	82263724	GAIN	region2inRegion1
CNVR104	1203	9	5206397	5225462	gain	294_Revilla	5148816	5348944	GAIN	region1inRegion2
CNVR106	1205	9	5747061	5810398	loss	297_Revilla	5748516	5788512	LOSS	region2inRegion1
CNVR112	1331	9	119585370	119593230	gain	315_Revilla	119588040	119591676	GAIN	region2inRegion1
CNVR128	1514	11	69030920	69069509	loss	369_Revilla	69061112	69064506	LOSS	region2inRegion1
CNVR132	1555	12	50924636	50947287	gain	375_Revilla	50883552	50930817	GAIN	overlapHead
CNVR157	1990	15	112634645	112649752	loss	484_Revilla	112647900	112651144	GAIN	overlapTail

CNVR159	2034	16	6146177	6154424	loss	494_Revilla	6142560	6147380	GAIN	overlapHead
CNVR161	2084	16	30196586	30215798	loss	504_Revilla	30196418	30206600	LOSS	overlapHead
CNVR161	2084	16	30196586	30217974	loss	504_Revilla	30196418	30206600	LOSS	overlapHead
CNVR14	279	1	284447951	284512355	loss	4_Schiavo	284443528	284486575	gain	overlapHead
CNVR6	103	1	100019309	100027849	gain	6_Schiavo	99869938	100080552	loss	region1inRegion2
CNVR61	730	5	21242609	21260003	gain	13_Schiavo	21238084	21339891	gain/loss	region1inRegion2
CNVR62	731	5	21332629	21368821	gain	13_Schiavo	21238084	21339891	gain/loss	overlapHead
CNVR80	946	7	25785853	25888939	gain	19_Schiavo	25773312	26018707	gain/loss	region1inRegion2
CNVR125	1457	11	32658621	32711857	loss	36_Schiavo	32619321	32878292	loss	region1inRegion2
CNVR129	1518	11	70689891	70709813	loss	49_Schiavo	70656747	70967934	loss	region1inRegion2
CNVR123	1437	11	8815180	8845277	loss	55_Schiavo	8638031	9216774	loss	region1inRegion2
CNVR141	1693	13	166109149	166114547	gain	84_Schiavo	165948194	166379880	loss	region1inRegion2
CNVR165	2136	16	85295667	85298564	loss	165_Schiavo	85253075	85764477	loss	region1inRegion2
CNVR6	103	1	100019309	100027849	gain	13_WAGNYN	99869938	100098510	loss	region1inRegion2
CNVR87	994	7	82234623	82374446	gain	154_WAGNYN	81753305	82401282	loss	region1inRegion2
CNVR94	1059	8	26029928	26034839	loss	162_WAGNYN	25948033	28118022	loss	region1inRegion2
CNVR101	1176	8	129139072	129153237	loss	176_WAGNYN	129127706	129213551	loss	region1inRegion2
CNVR123	1437	11	8815180	8845277	loss	217_WAGNYN	8771604	9895994	loss	region1inRegion2
CNVR129	1518	11	70689891	70709813	loss	230_WAGNYN	70508069	71130475	gain	region1inRegion2

CNVR140	1690	13	164895338	164898801	gain	260_WAGNYN	161588754	167903653	gain	region1inRegion2
CNVR141	1693	13	166109149	166114547	gain	260_WAGNYN	161588754	167903653	gain	region1inRegion2
CNVR142	1695	13	167567440	167600641	gain	260_WAGNYN	161588754	167903653	gain	region1inRegion2
CNVR151	1829	14	74107606	74126308	loss	284_WAGNYN	73559713	76660742	both	region1inRegion2
CNVR15	288	1	295235629	295379020	gain	305_WangHY	295260301	295266005	Loss	region2inRegion1
CNVR25	321	2	15024071	15060448	loss	386_WangHY	15032401	15069889	Loss	overlapTail
CNVR46	504	3	45023435	45241189	gain	721_WangHY	45096901	45115452	Loss	region2inRegion1
CNVR46	504	3	45023435	45241189	gain	722_WangHY	45155949	45165000	Loss	region2inRegion1
CNVR46	504	3	45023435	45241189	gain	723_WangHY	45200026	45206700	Loss	region2inRegion1
CNVR54	589	3	144724140	144783726	gain	848_WangHY	144742501	144747900	Loss	region2inRegion1
CNVR54	589	3	144724140	144783726	gain	849_WangHY	144765601	144772800	Loss	region2inRegion1
CNVR61	730	5	21242609	21260003	gain	1061_WangHY	21257701	21264600	Loss	overlapTail
CNVR67	800	5	79630280	79697936	gain	1126_WangHY	79684801	79686973	Gain	region2inRegion1
CNVR68	833	6	8471113	8494128	gain	1182_WangHY	8488117	8494200	Loss	overlapTail
CNVR106	1205	9	5747061	5810398	loss	1703_WangHY	5734903	5747400	Loss	overlapHead
CNVR106	1205	9	5747061	5810398	loss	1704_WangHY	5793901	5802900	Loss	region2inRegion1
CNVR132	1555	12	50924636	50947287	gain	2167_WangHY	50937601	50952637	Loss	overlapTail
CNVR136	1589	13	30640874	30643949	gain	2212_WangHY	30639301	30661896	Loss	region1inRegion2
CNVR154	1873	14	120810562	120829324	gain	2617_WangHY	120797951	120822780	Loss	overlapHead

CNVR158	2026	15	156637663	156646003	gain	2877_WangHY	156624601	156641497	Loss	overlapHead
CNVR15	288	1	295235629	295379020	gain	6_WangJY	295241692	295379020	gain	region2inRegion1
CNVR125	1457	11	32658621	32711857	loss	10_WangJY	32619321	32711857	loss	region1inRegion2
CNVR129	1518	11	70689891	70709813	loss	12_WangJY	70508069	71089190	gain	region1inRegion2
CNVR63	736	5	22004036	22012384	loss	46_WangJY	21932283	22169288	gain	region1inRegion2
CNVR104	1203	9	5206397	5225462	gain	59_WangJY	5159477	5419279	gain	region1inRegion2
CNVR106	1205	9	5747061	5810398	loss	60_WangJY	5532623	5779850	gain	overlapHead
CNVR46	504	3	45023435	45241189	gain	CNV67_WangZ	45228988	45256960	NA	overlapTail
CNVR126	1468	11	38006863	38074356	gain	CNV274_WangZ	37947843	38010860	NA	overlapHead
CNVR4	99	1	99687218	99704572	gain	CNVR6_XIEJIAN	99692675	100080552	gain	overlapTail
CNVR5	101	1	99733649	99785971	gain	CNVR6_XIEJIAN	99692675	100080552	gain	region1inRegion2
CNVR6	103	1	100019309	100027849	gain	CNVR6_XIEJIAN	99692675	100080552	gain	region1inRegion2
CNVR14	279	1	284447951	284512355	loss	CNVR12_XIEJIAN	284443528	284486575	gain	overlapHead
CNVR15	288	1	295235629	295379020	gain	CNVR14_XIEJIAN	295241692	295462741	gain	overlapTail
CNVR18	298	1	312363155	312415324	gain	CNVR17_XIEJIAN	311929352	312829468	both	region1inRegion2
CNVR19	299	1	312415324	312437693	gain	CNVR17_XIEJIAN	311929352	312829468	both	region1inRegion2
CNVR23	319	2	14000848	14032642	loss-gain	CNVR19_XIEJIAN	13853695	14043003	gain	region1inRegion2
CNVR41	472	2	158544152	158558867	gain	CNVR28_XIEJIAN	158549163	159250446	gain	overlapTail
CNVR42	473	2	158605784	158612295	gain	CNVR28_XIEJIAN	158549163	159250446	gain	region1inRegion2

CNVR42	473	2	158605784	158629757	gain	CNVR28_XIEJIAN	158549163	159250446	gain	region1inRegion2
CNVR44	479	2	159094998	159110351	gain	CNVR28_XIEJIAN	158549163	159250446	gain	region1inRegion2
CNVR61	730	5	21242609	21260003	gain	CNVR44_XIEJIAN	21238084	21339891	gain	region1inRegion2
CNVR62	731	5	21332629	21368821	gain	CNVR44_XIEJIAN	21238084	21339891	gain	overlapHead
CNVR78	944	7	25359190	25399904	gain	CNVR54_XIEJIAN	25215084	26187687	both	region1inRegion2
CNVR79	945	7	25488796	25557156	gain	CNVR54_XIEJIAN	25215084	26187687	both	region1inRegion2
CNVR80	946	7	25785853	25888939	gain	CNVR54_XIEJIAN	25215084	26187687	both	region1inRegion2
CNVR103	1200	9	3614907	3618335	gain	CNVR66_XIEJIAN	3598721	3721967	gain	region1inRegion2
CNVR129	1518	11	70689891	70709813	loss	CNVR79_XIEJIAN	70685626	71912745	both	region1inRegion2
CNVR130	1520	11	71317528	71375741	loss	CNVR79_XIEJIAN	70685626	71912745	both	region1inRegion2
CNVR131	1524	11	71878763	71887995	gain	CNVR79_XIEJIAN	70685626	71912745	both	region1inRegion2
CNVR132	1555	12	50924636	50947287	gain	CNVR82_XIEJIAN	50892615	50930355	gain	overlapHead
CNVR143	1699	13	170765271	170819670	gain	CNVR91_XIEJIAN	170612751	170944031	gain	region1inRegion2
