# Supplementary material for: Identification of copy number variations using high density whole-genome single nucleotide polymorphism markers in Chinese Dongxiang spotted pigs
Source: Asian-Australas J Anim Sci. 2019 Feb 7;32(12):1809–15. doi: 10.5713/ajas.18.0696 (PMC6819687; doi:10.5713/ajas.18.0696)
Supplement: Supplementary file 4 [file ajas-18-0696-suppl4.pdf]

Table S4. Annotated genes in the CNVRs identified in this study

CNVR ID	CNV_ID	CNVR_Start	CNVR_End	Chr.	Ensembl Gene ID	Gene Start	Gene End	Gene Name	Gene_Biotype	Relationship
CNVR2	88	91934647	91942954	1	ENSSSCG00000004435	91861519	91997751	NT5DC1	protein_coding	region1inRegion2
CNVR7	141	145236440	145265439	1	ENSSSCG00000004751	145223057	145259690	CHP1	protein_coding	overlapHead
CNVR7	141	145236440	145265439	1	ENSSSCG00000026091	145247884	145248054	RPS29	protein_coding	region2inRegion1
CNVR7	141	145236440	145265439	1	ENSSSCG00000004752	145262870	145313560	Exd1	protein_coding	overlapTail
CNVR10	220	224697068	224765844	1	ENSSSCG00000005149	224699121	224699633	IFN-DELTA-1	protein_coding	region2inRegion1
CNVR10	220	224697068	224765844	1	ENSSSCG00000021817	224724570	224724673		snRNA	region2inRegion1
CNVR10	220	224697068	224765844	1	ENSSSCG00000027162	224730487	224801189	IFNW1	protein_coding	overlapTail
CNVR12	261	268054795	268056507	1	ENSSSCG00000005373	268051416	268082286	NANS	protein_coding	region1inRegion2
CNVR14	279	284447951	284512355	1	ENSSSCG00000005474	284447110	284451960	SAL1	protein_coding	overlapHead
CNVR15	288	295235629	295379020	1	ENSSSCG00000027732	295277041	295278003	OR1J4	protein_coding	region2inRegion1
CNVR15	288	295235629	295379020	1	ENSSSCG00000029564	295289239	295290180	OR1J4	protein_coding	region2inRegion1
CNVR15	288	295235629	295379020	1	ENSSSCG00000026287	295304653	295305610		pseudogene	region2inRegion1
CNVR15	288	295235629	295379020	1	ENSSSCG00000026287	295304653	295305610		pseudogene	region2inRegion1
CNVR18	298	312363155	312415324	1	ENSSSCG00000005817	312377137	312378084	OR8H3	protein_coding	region2inRegion1
CNVR18	298	312363155	312415324	1	ENSSSCG00000024808	312400631	312401569	OR8K3	protein_coding	region2inRegion1
CNVR18	298	312363155	312415324	1	ENSSSCG00000005805	312412169	312413113	OR8K3	protein_coding	region2inRegion1
CNVR20	312	11511074	11530117	2	ENSSSCG00000021797	11525200	11526114	OR5AN1	protein_coding	region2inRegion1

CNVR21	314	12364565	12398203	2	ENSSSCG00000026361	12368666	12369604	OR5B3		protein_coding	region2inRegion1
CNVR21	314	12364565	12398203	2	ENSSSCG00000026611	12384880	12385824	OR5B17		protein_coding	region2inRegion1
CNVR21	314	12364565	12398203	2	ENSSSCG00000021043	12393163	12394137	OR1S1		protein_coding	region2inRegion1
CNVR22	316	12689951	12700771	2	ENSSSCG00000024984	12698820	12699782	OR5G3		protein_coding	region2inRegion1
CNVR23	319	14000848	14032642	2	ENSSSCG00000013201	14030126	14031853	OR9G4		protein_coding	region2inRegion1
CNVR24	320	14728311	14771718	2	ENSSSCG00000014530	14733194	14734129	Olfr140		protein_coding	region2inRegion1
CNVR24	320	14728311	14771718	2	ENSSSCG00000014528	14753116	14754048	Olfr140		protein_coding	region2inRegion1
CNVR24	320	14728311	14771718	2	ENSSSCG00000021435	14765540	14766469	OR4C13		protein_coding	region2inRegion1
CNVR25	321	15024071	15060448	2	ENSSSCG00000030330	15043603	15044923	OR4C13		protein_coding	region2inRegion1
CNVR26	343	44800086	44803691	2	ENSSSCG00000013378	44725269	44812585	ABCC8		protein_coding	region1inRegion2
CNVR28	353	56935884	56944870	2	ENSSSCG00000023473	56938410	56938658	RPS29		protein_coding	region2inRegion1
CNVR30	359	61357153	61369311	2	ENSSSCG00000013842	61344295	61358739	CYP4F2		protein_coding	overlapHead
CNVR32	364	62628253	62738661	2	ENSSSCG00000013822	62644870	62645796	OLF4_CANLF		protein_coding	region2inRegion1
CNVR32	364	62628253	62738661	2	ENSSSCG00000013819	62669703	62670662	OR7A5		protein_coding	region2inRegion1
CNVR33	378	67915859	67952389	2	ENSSSCG00000021192	67934296	67935234	Olfr18		protein_coding	region2inRegion1
CNVR34	379	68061173	68181801	2	ENSSSCG00000023573	68082738	68083649	OR7E24		protein_coding	region2inRegion1
CNVR34	379	68061173	68181801	2	ENSSSCG00000013696	68108471	68109823	OR7E24		protein_coding	region2inRegion1
CNVR34	379	68061173	68181801	2	ENSSSCG00000013690	68169183	68170608	OR7E24		protein_coding	region2inRegion1
CNVR37	422	107437562	107451870	2	ENSSSCG00000014173	107369918	107462862	LNPEP		protein_coding	region1inRegion2

CNVR38	466	148904898	148913506	2	ENSSSCG00000014378	148910217	148913280	PCDHB4	protein_coding	region2inRegion1
CNVR41	472	158544152	158558867	2	ENSSSCG00000024251	158546202	158547137	OR4P4	protein_coding	region2inRegion1
CNVR42	473	158605784	158629757	2	ENSSSCG00000014455	158613163	158614101	OR5D13	protein_coding	region2inRegion1
CNVR42	473	158605784	158629757	2	ENSSSCG00000014456	158620781	158621734	OR5D18	protein_coding	region2inRegion1
CNVR42	473	158605784	158629757	2	ENSSSCG00000014457	158629441	158630382	OR5D13	protein_coding	overlapTail
CNVR44	479	159094998	159110351	2	ENSSSCG00000014474	159095777	159096721	OR8K3	protein_coding	region2inRegion1
CNVR46	504	45023435	45241189	3	ENSSSCG00000026202	45093945	45102159	NUTM2G	protein_coding	region2inRegion1
CNVR46	504	45023435	45241189	3	ENSSSCG00000028738	45106277	45107791	ZNF169	protein_coding	region2inRegion1
CNVR46	504	45023435	45241189	3	ENSSSCG00000008087	45180185	45185893	IL1B2	protein_coding	region2inRegion1
CNVR51	581	142899740	142925257	3	ENSSSCG00000008662	142921464	142923089	BTN1A1	protein_coding	region2inRegion1
CNVR56	649	55682568	55685476	4	ENSSSCG00000006138	55660235	55715444	ATP6V0D2	protein_coding	region1inRegion2
CNVR58	683	88694499	88704809	4	ENSSSCG00000006282	88564488	88734989	KIFAP3	protein_coding	region1inRegion2
CNVR59	698	111405421	111446020	4	ENSSSCG00000030938	111398740	111406269	PHGDH	protein_coding	overlapHead
CNVR59	698	111405421	111446020	4	ENSSSCG00000006716	111411282	111430642	HMGCS2	protein_coding	region2inRegion1
CNVR59	698	111405421	111446020	4	ENSSSCG00000006717	111436317	111468486	PHGDH	protein_coding	overlapTail
CNVR61	730	21242609	21260003	5	ENSSSCG00000026447	21256500	21258129	OR6C6	protein_coding	region2inRegion1
CNVR62	731	21332629	21368821	5	ENSSSCG00000000351	21337563	21338498	OR6C2	protein_coding	region2inRegion1
CNVR62	731	21332629	21368821	5	ENSSSCG00000000320	21362491	21363420	OR6C75	protein_coding	region2inRegion1
CNVR64	739	22410640	22421100	5	ENSSSCG00000029157	22416614	22417558	OR6C2	protein_coding	region2inRegion1

CNVR65	755	39192570	39207538	5	ENSSSCG00000000515	39065887	39518064	TRHDE	protein_coding	region1inRegion2
CNVR67	800	79630280	79697936	5	ENSSSCG00000030685	79566481	79726285	ARID2	protein_coding	region1inRegion2
CNVR71	917	151994674	152015484	6	ENSSSCG00000024778	151929629	152153725	CYP4A24	protein_coding	region1inRegion2
CNVR71	917	151994674	152015484	6	ENSSSCG00000024129	151954326	152007678	CYP4X1	protein_coding	overlapHead
CNVR71	917	151994674	152015484	6	ENSSSCG00000023702	151996428	151997949	CYP4X1	protein_coding	region2inRegion1
CNVR71	917	151994674	152015484	6	ENSSSCG00000003891	152012736	152187414	CYP4A24	protein_coding	overlapTail
CNVR73	934	22207228	22236111	7	ENSSSCG00000001147	22214209	22215141	OR2M2	protein_coding	region2inRegion1
CNVR74	938	23307667	23658110	7	ENSSSCG00000029559	23337179	23338117	OR2B8P	protein_coding	region2inRegion1
CNVR74	938	23307667	23658110	7	ENSSSCG00000001190	23371588	23372520	OR2B8P	protein_coding	region2inRegion1
CNVR74	938	23307667	23658110	7	ENSSSCG00000026574	23398414	23399343	OR2B8P	protein_coding	region2inRegion1
CNVR74	938	23307667	23658110	7	ENSSSCG00000020709	23483415	23484344	OR2B8P	protein_coding	region2inRegion1
CNVR75	939	23481211	23658110	7	ENSSSCG00000020709	23483415	23484344	OR2B8P	protein_coding	region2inRegion1
CNVR74	938	23307667	23658110	7	ENSSSCG00000026329	23515850	23516788	OR2B8P	protein_coding	region2inRegion1
CNVR75	939	23481211	23658110	7	ENSSSCG00000026329	23515850	23516788	OR2B8P	protein_coding	region2inRegion1
CNVR74	938	23307667	23658110	7	ENSSSCG00000023340	23519655	23521580	OR2B8P	protein_coding	region2inRegion1
CNVR75	939	23481211	23658110	7	ENSSSCG00000023340	23519655	23521580	OR2B8P	protein_coding	region2inRegion1
CNVR74	938	23307667	23658110	7	ENSSSCG00000025476	23555120	23556049	OR2B8P	protein_coding	region2inRegion1
CNVR75	939	23481211	23658110	7	ENSSSCG00000025476	23555120	23556049	OR2B8P	protein_coding	region2inRegion1
CNVR76	942	24648309	24653067	7	ENSSSCG00000030604	24650159	24650269		rRNA	region2inRegion1

CNVR78	944	25359190	25399904	7	ENSSSCG00000026875	25369478	25370439	OR2G6	protein_coding	region2inRegion1
CNVR78	944	25359190	25399904	7	ENSSSCG00000001254	25373473	25374327	OR2G3	protein_coding	region2inRegion1
CNVR78	944	25359190	25399904	7	ENSSSCG00000001255	25382745	25383668	OR6N2	protein_coding	region2inRegion1
CNVR78	944	25359190	25399904	7	ENSSSCG00000028462	25386139	25386245		snRNA	region2inRegion1
CNVR78	944	25359190	25399904	7	ENSSSCG00000001256	25396945	25397917		pseudogene	region2inRegion1
CNVR78	944	25359190	25399904	7	ENSSSCG00000001256	25396945	25397917		pseudogene	region2inRegion1
CNVR79	945	25488796	25557156	7	ENSSSCG00000028426	25543388	25544314	OR12D2	protein_coding	region2inRegion1
CNVR79	945	25488796	25557156	7	ENSSSCG00000021520	25555450	25556864	OR5V1	protein_coding	region2inRegion1
CNVR80	946	25785853	25888939	7	ENSSSCG00000001286	25794642	25795595	OR5V1	protein_coding	region2inRegion1
CNVR80	946	25785853	25888939	7	ENSSSCG00000023411	25861917	25862846	OR2G3	protein_coding	region2inRegion1
CNVR80	946	25785853	25888939	7	ENSSSCG00000026436	25869234	25870169	OR2G6	protein_coding	region2inRegion1
CNVR81	949	26267062	26297673	7	ENSSSCG00000025314	26269415	26270275	OR2W1	protein_coding	region2inRegion1
CNVR81	949	26267062	26297673	7	ENSSSCG00000022756	26295011	26295958	OR2B8P	protein_coding	region2inRegion1
CNVR82	951	26600964	26608495	7	ENSSSCG00000026831	26600985	26601947	OR2W1	protein_coding	region2inRegion1
CNVR84	972	58579811	58584542	7	ENSSSCG00000001822	58578282	58587684	RCCD1	protein_coding	region1inRegion2
CNVR87	994	82234623	82374446	7	ENSSSCG00000021543	82253358	82390901	TVA1_HUMAN	protein_coding	overlapTail
CNVR87	994	82234623	82374446	7	ENSSSCG00000021202	82257955	82258446	TCRA	protein_coding	region2inRegion1
CNVR87	994	82234623	82374446	7	ENSSSCG00000026335	82276655	82280677	TVA3_MOUSE	protein_coding	region2inRegion1
CNVR87	994	82234623	82374446	7	ENSSSCG00000023346	82281866	82282491	TVA1_HUMAN	protein_coding	region2inRegion1

CNVR87	994	82234623	82374446	7	ENSSSCG00000029524	82299859	82300402	TVA2_MOUSE	protein_coding	region2inRegion1
CNVR87	994	82234623	82374446	7	ENSSSCG00000002078	82302487	82302994	TVA3_HUMAN	protein_coding	region2inRegion1
CNVR87	994	82234623	82374446	7	ENSSSCG00000024807	82306610	82307158	ENSSSCG00000024807	protein_coding	region2inRegion1
CNVR87	994	82234623	82374446	7	ENSSSCG00000025373	82313420	82313986	TCRA	protein_coding	region2inRegion1
CNVR87	994	82234623	82374446	7	ENSSSCG00000002081	82322889	82324569	TVA3_MOUSE	protein_coding	region2inRegion1
CNVR87	994	82234623	82374446	7	ENSSSCG00000021666	82333128	82334537	ENSSSCG00000021666	protein_coding	region2inRegion1
CNVR87	994	82234623	82374446	7	ENSSSCG00000002085	82362291	82362868	ENSSSCG00000002085	protein_coding	region2inRegion1
CNVR89	1011	102790067	102823288	7	ENSSSCG00000002349	102786805	102791429	ACOT4	protein_coding	overlapHead
CNVR99	1129	85727251	85735420	8	ENSSSCG00000024987	85602111	85800834	NR3C2	protein_coding	region1inRegion2
CNVR99	1129	85727251	85735420	8	ENSSSCG00000009028	85707729	85739412	ARHGAP10	protein_coding	region1inRegion2
CNVR100	1169	122108843	122121928	8	ENSSSCG00000009148	122085975	122204345	LEF1	protein_coding	region1inRegion2
CNVR102	1198	1963636	2010455	9	ENSSSCG00000023830	1968198	1969151	Olfr477	protein_coding	region2inRegion1
CNVR104	1203	5206397	5225462	9	ENSSSCG00000014693	5209262	5210203	OR52D1	protein_coding	region2inRegion1
CNVR104	1203	5206397	5225462	9	ENSSSCG00000014694	5214883	5215877		pseudogene	region2inRegion1
CNVR104	1203	5206397	5225462	9	ENSSSCG00000014694	5214883	5215877		pseudogene	region2inRegion1
CNVR105	1204	5488035	5492918	9	ENSSSCG00000024371	5487546	5488490	OR51V1	protein_coding	overlapHead
CNVR106	1205	5747061	5810398	9	ENSSSCG00000014740	5752638	5753734	OR52E2	protein_coding	region2inRegion1
CNVR106	1205	5747061	5810398	9	ENSSSCG00000014745	5768665	5769600	OR52J3	protein_coding	region2inRegion1
CNVR106	1205	5747061	5810398	9	ENSSSCG00000014746	5791044	5791985	OR51A4	protein_coding	region2inRegion1

CNVR106	1205	5747061	5810398	9	ENSSSCG00000014747	5803253	5804194	OR51G2	protein_coding	region2inRegion1
CNVR107	1227	21980507	21992085	9	ENSSSCG00000014909	21887327	22005568	SYTL2	protein_coding	region1inRegion2
CNVR109	1261	56272660	56332060	9	ENSSSCG00000025623	56306596	56307760	OR8B3	protein_coding	region2inRegion1
CNVR110	1262	56901872	56950458	9	ENSSSCG00000022266	56912897	56913003		snRNA	region2inRegion1
CNVR110	1262	56901872	56950458	9	ENSSSCG00000015184	56925449	56927199	OR8B3	protein_coding	region2inRegion1
CNVR112	1331	119585370	119593230	9	ENSSSCG00000029596	119542539	119595213	TCC3_MOUSE	protein_coding	region1inRegion2
CNVR112	1331	119585370	119593230	9	ENSSSCG00000023584	119580426	119585606	TCC1_MOUSE	protein_coding	overlapHead
CNVR113	1338	124260883	124271069	9	ENSSSCG00000015462	124255189	124629474	TPK1	protein_coding	region1inRegion2
CNVR119	1427	71830224	71835029	10	ENSSSCG00000011147	71833306	71847875	DDBX_BOVIN	protein_coding	overlapTail
CNVR122	1436	8308242	8320582	11	ENSSSCG00000009336	8312524	8336889	RXFP2	protein_coding	overlapTail
CNVR129	1518	70689891	70709813	11	ENSSSCG00000009498	70591415	70739460	ABCC4	protein_coding	region1inRegion2
CNVR130	1520	71317528	71375741	11	ENSSSCG00000009497	71298810	71453998	ABCC4	protein_coding	region1inRegion2
CNVR130	1520	71317528	71375741	11	ENSSSCG00000024407	71345954	71346034		miRNA	region2inRegion1
CNVR132	1555	50924636	50947287	12	ENSSSCG00000026065	50933615	50934550	OR1A1	protein_coding	region2inRegion1
CNVR132	1555	50924636	50947287	12	ENSSSCG00000024366	50940999	50941928	OR1A1	protein_coding	region2inRegion1
CNVR133	1556	51099806	51123003	12	ENSSSCG00000027631	51103565	51104494	OR1A1	protein_coding	region2inRegion1
CNVR139	1656	131028348	131044332	13	ENSSSCG00000011775	131013295	131042066	KLHL24	protein_coding	overlapHead
CNVR141	1693	166109149	166114547	13	ENSSSCG00000011947	166070148	166110307	ZPLD1	protein_coding	overlapHead
CNVR145	1777	2401741	2423505	14	ENSSSCG00000027841	2406324	2406923	Diras2	protein_coding	region2inRegion1

CNVR146	1781	7794851	7822418	14	ENSSSCG00000009633	7785333	7817139	TNFRSF10B	protein_coding	overlapHead
CNVR147	1782	8039487	8116098	14	ENSSSCG00000009636	8076243	8084520	TNFRSF10B	protein_coding	region2inRegion1
CNVR147	1782	8039487	8116098	14	ENSSSCG00000030644	8111572	8120119	TNFRSF10B	protein_coding	overlapTail
CNVR148	1792	21068045	21091201	14	ENSSSCG00000009708	21067835	21091630	AADAT	protein_coding	region1inRegion2
CNVR150	1820	65589945	65600023	14	ENSSSCG00000028345	65569354	65661554	ZNF33A	protein_coding	region1inRegion2
CNVR150	1820	65589945	65600023	14	ENSSSCG00000010195	65578719	65630736	ZNF37A	protein_coding	region1inRegion2
CNVR152	1838	81377028	81553876	14	ENSSSCG00000010289	81253334	81512159	MICU1	protein_coding	overlapHead
CNVR153	1849	94594494	94629904	14	ENSSSCG00000010356	94625362	94627832	Ralgds	protein_coding	region2inRegion1
CNVR154	1873	120810562	120829324	14	ENSSSCG00000010548	120756666	120854124	CHUK	protein_coding	region1inRegion2
CNVR154	1873	120810562	120829324	14	ENSSSCG00000010549	120804242	120834261	Cyp2c23	protein_coding	region1inRegion2
CNVR157	1990	112634645	112649752	15	ENSSSCG00000016082	112634532	112667533	BOLL	protein_coding	region1inRegion2
CNVR159	2034	6146177	6154424	16	ENSSSCG00000016793	6142034	6158561	ZNF622	protein_coding	region1inRegion2
CNVR163	2109	53083153	53202189	16	ENSSSCG00000024289	53191857	53193197	DRD1	protein_coding	region2inRegion1
