# Supplementary material for: Identification of copy number variations using high density whole-genome single nucleotide polymorphism markers in Chinese Dongxiang spotted pigs
Source: Asian-Australas J Anim Sci. 2019 Feb 7;32(12):1809–15. doi: 10.5713/ajas.18.0696 (PMC6819687; doi:10.5713/ajas.18.0696)
Supplement: Supplementary file 5 [file ajas-18-0696-suppl5.pdf]

Table S5. CNVRs identified in this study and their overlapped QTLs

CNVR ID	CNV ID	Chr.	CNVR Start	CNVR End	State	QTL ID	QTL Start	QTL End	QTL Name	Relationship	Overlap Length (bp)
CNVR1	83	1	88942731	88998960	gain	38049	2701298	274130998	Marbling	region1inRegion2	56229
CNVR2	88	1	91934647	91942954	loss	38049	2701298	274130998	Marbling	region1inRegion2	8307
CNVR3	92	1	95316086	95335767	gain	38049	2701298	274130998	Marbling	region1inRegion2	19681
CNVR4	99	1	99687218	99704572	gain	38049	2701298	274130998	Marbling	region1inRegion2	17354
CNVR5	101	1	99733649	99785971	gain	38049	2701298	274130998	Marbling	region1inRegion2	52322
CNVR6	103	1	100019309	100027849	gain	38049	2701298	274130998	Marbling	region1inRegion2	8540
CNVR7	141	1	145236440	145265439	gain	38049	2701298	274130998	Marbling	region1inRegion2	28999
CNVR8	156	1	170118820	170121900	gain	38049	2701298	274130998	Marbling	region1inRegion2	3080
CNVR9	191	1	197269718	197274380	gain	38049	2701298	274130998	Marbling	region1inRegion2	4662
CNVR10	220	1	224697068	224765844	gain	38049	2701298	274130998	Marbling	region1inRegion2	68776
CNVR11	255	1	259697345	259705356	gain	38049	2701298	274130998	Marbling	region1inRegion2	8011
CNVR12	261	1	268054795	268056507	gain	38049	2701298	274130998	Marbling	region1inRegion2	1712
CNVR13	266	1	271529620	271536665	gain	38049	2701298	274130998	Marbling	region1inRegion2	7045
CNVR1	83	1	88942731	88998960	gain	38050	2701298	292955427	Loin_fat_percentage	region1inRegion2	56229
CNVR2	88	1	91934647	91942954	loss	38050	2701298	292955427	Loin_fat_percentage	region1inRegion2	8307
CNVR3	92	1	95316086	95335767	gain	38050	2701298	292955427	Loin_fat_percentage	region1inRegion2	19681
CNVR4	99	1	99687218	99704572	gain	38050	2701298	292955427	Loin_fat_percentage	region1inRegion2	17354
CNVR5	101	1	99733649	99785971	gain	38050	2701298	292955427	Loin_fat_percentage	region1inRegion2	52322
CNVR6	103	1	100019309	100027849	gain	38050	2701298	292955427	Loin_fat_percentage	region1inRegion2	8540
CNVR7	141	1	145236440	145265439	gain	38050	2701298	292955427	Loin_fat_percentage	region1inRegion2	28999
CNVR8	156	1	170118820	170121900	gain	38050	2701298	292955427	Loin_fat_percentage	region1inRegion2	3080
CNVR9	191	1	197269718	197274380	gain	38050	2701298	292955427	Loin_fat_percentage	region1inRegion2	4662
CNVR10	220	1	224697068	224765844	gain	38050	2701298	292955427	Loin_fat_percentage	region1inRegion2	68776
CNVR11	255	1	259697345	259705356	gain	38050	2701298	292955427	Loin_fat_percentage	region1inRegion2	8011
CNVR12	261	1	268054795	268056507	gain	38050	2701298	292955427	Loin_fat_percentage	region1inRegion2	1712
CNVR13	266	1	271529620	271536665	gain	38050	2701298	292955427	Loin_fat_percentage	region1inRegion2	7045
CNVR14	279	1	284447951	284512355	loss	38050	2701298	292955427	Loin_fat_percentage	region1inRegion2	64404
CNVR1	83	1	88942731	88998960	gain	38072	2701298	292955427	Loin_muscle_area	region1inRegion2	56229
CNVR2	88	1	91934647	91942954	loss	38072	2701298	292955427	Loin_muscle_area	region1inRegion2	8307
CNVR3	92	1	95316086	95335767	gain	38072	2701298	292955427	Loin_muscle_area	region1inRegion2	19681
CNVR4	99	1	99687218	99704572	gain	38072	2701298	292955427	Loin_muscle_area	region1inRegion2	17354
CNVR5	101	1	99733649	99785971	gain	38072	2701298	292955427	Loin_muscle_area	region1inRegion2	52322
CNVR6	103	1	100019309	100027849	gain	38072	2701298	292955427	Loin_muscle_area	region1inRegion2	8540
CNVR7	141	1	145236440	145265439	gain	38072	2701298	292955427	Loin_muscle_area	region1inRegion2	28999
CNVR8	156	1	170118820	170121900	gain	38072	2701298	292955427	Loin_muscle_area	region1inRegion2	3080
CNVR9	191	1	197269718	197274380	gain	38072	2701298	292955427	Loin_muscle_area	region1inRegion2	4662
CNVR10	220	1	224697068	224765844	gain	38072	2701298	292955427	Loin_muscle_area	region1inRegion2	68776
CNVR11	255	1	259697345	259705356	gain	38072	2701298	292955427	Loin_muscle_area	region1inRegion2	8011
CNVR12	261	1	268054795	268056507	gain	38072	2701298	292955427	Loin_muscle_area	region1inRegion2	1712
CNVR13	266	1	271529620	271536665	gain	38072	2701298	292955427	Loin_muscle_area	region1inRegion2	7045
CNVR14	279	1	284447951	284512355	loss	38072	2701298	292955427	Loin_muscle_area	region1inRegion2	64404
CNVR1	83	1	88942731	88998960	gain	38073	2701298	292955427	Muscle_moisture_percentage	region1inRegion2	56229
CNVR2	88	1	91934647	91942954	loss	38073	2701298	292955427	Muscle_moisture_percentage	region1inRegion2	8307
CNVR3	92	1	95316086	95335767	gain	38073	2701298	292955427	Muscle_moisture_percentage	region1inRegion2	19681
CNVR4	99	1	99687218	99704572	gain	38073	2701298	292955427	Muscle_moisture_percentage	region1inRegion2	17354
CNVR5	101	1	99733649	99785971	gain	38073	2701298	292955427	Muscle_moisture_percentage	region1inRegion2	52322
CNVR6	103	1	100019309	100027849	gain	38073	2701298	292955427	Muscle_moisture_percentage	region1inRegion2	8540
CNVR7	141	1	145236440	145265439	gain	38073	2701298	292955427	Muscle_moisture_percentage	region1inRegion2	28999
CNVR8	156	1	170118820	170121900	gain	38073	2701298	292955427	Muscle_moisture_percentage	region1inRegion2	3080
CNVR9	191	1	197269718	197274380	gain	38073	2701298	292955427	Muscle_moisture_percentage	region1inRegion2	4662
CNVR10	220	1	224697068	224765844	gain	38073	2701298	292955427	Muscle_moisture_percentage	region1inRegion2	68776
CNVR11	255	1	259697345	259705356	gain	38073	2701298	292955427	Muscle_moisture_percentage	region1inRegion2	8011
CNVR12	261	1	268054795	268056507	gain	38073	2701298	292955427	Muscle_moisture_percentage	region1inRegion2	1712
CNVR13	266	1	271529620	271536665	gain	38073	2701298	292955427	Muscle_moisture_percentage	region1inRegion2	7045
CNVR14	279	1	284447951	284512355	loss	38073	2701298	292955427	Muscle_moisture_percentage	region1inRegion2	64404
CNVR1	83	1	88942731	88998960	gain	5671	7964370	252283830	Shoulder_subcutaneous_fat_thickness	region1inRegion2	56229
CNVR2	88	1	91934647	91942954	loss	5671	7964370	252283830	Shoulder_subcutaneous_fat_thickness	region1inRegion2	8307
CNVR3	92	1	95316086	95335767	gain	5671	7964370	252283830	Shoulder_subcutaneous_fat_thickness	region1inRegion2	19681
CNVR4	99	1	99687218	99704572	gain	5671	7964370	252283830	Shoulder_subcutaneous_fat_thickness	region1inRegion2	17354
CNVR5	101	1	99733649	99785971	gain	5671	7964370	252283830	Shoulder_subcutaneous_fat_thickness	region1inRegion2	52322
CNVR6	103	1	100019309	100027849	gain	5671	7964370	252283830	Shoulder_subcutaneous_fat_thickness	region1inRegion2	8540
CNVR7	141	1	145236440	145265439	gain	5671	7964370	252283830	Shoulder_subcutaneous_fat_thickness	region1inRegion2	28999
CNVR8	156	1	170118820	170121900	gain	5671	7964370	252283830	Shoulder_subcutaneous_fat_thickness	region1inRegion2	3080
CNVR9	191	1	197269718	197274380	gain	5671	7964370	252283830	Shoulder_subcutaneous_fat_thickness	region1inRegion2	4662
CNVR10	220	1	224697068	224765844	gain	5671	7964370	252283830	Shoulder_subcutaneous_fat_thickness	region1inRegion2	68776
CNVR1	83	1	88942731	88998960	gain	5670	7964370	257944209	Average_backfat_thickness	region1inRegion2	56229
CNVR2	88	1	91934647	91942954	loss	5670	7964370	257944209	Average_backfat_thickness	region1inRegion2	8307
CNVR3	92	1	95316086	95335767	gain	5670	7964370	257944209	Average_backfat_thickness	region1inRegion2	19681
CNVR4	99	1	99687218	99704572	gain	5670	7964370	257944209	Average_backfat_thickness	region1inRegion2	17354
CNVR5	101	1	99733649	99785971	gain	5670	7964370	257944209	Average_backfat_thickness	region1inRegion2	52322
CNVR6	103	1	100019309	100027849	gain	5670	7964370	257944209	Average_backfat_thickness	region1inRegion2	8540
CNVR7	141	1	145236440	145265439	gain	5670	7964370	257944209	Average_backfat_thickness	region1inRegion2	28999
CNVR8	156	1	170118820	170121900	gain	5670	7964370	257944209	Average_backfat_thickness	region1inRegion2	3080
CNVR9	191	1	197269718	197274380	gain	5670	7964370	257944209	Average_backfat_thickness	region1inRegion2	4662
CNVR10	220	1	224697068	224765844	gain	5670	7964370	257944209	Average_backfat_thickness	region1inRegion2	68776
CNVR1	83	1	88942731	88998960	gain	5680	7964370	292955427	Average_daily_gain	region1inRegion2	56229
CNVR2	88	1	91934647	91942954	loss	5680	7964370	292955427	Average_daily_gain	region1inRegion2	8307
CNVR3	92	1	95316086	95335767	gain	5680	7964370	292955427	Average_daily_gain	region1inRegion2	19681
CNVR4	99	1	99687218	99704572	gain	5680	7964370	292955427	Average_daily_gain	region1inRegion2	17354
CNVR5	101	1	99733649	99785971	gain	5680	7964370	292955427	Average_daily_gain	region1inRegion2	52322
CNVR6	103	1	100019309	100027849	gain	5680	7964370	292955427	Average_daily_gain	region1inRegion2	8540
CNVR7	141	1	145236440	145265439	gain	5680	7964370	292955427	Average_daily_gain	region1inRegion2	28999
CNVR8	156	1	170118820	170121900	gain	5680	7964370	292955427	Average_daily_gain	region1inRegion2	3080
CNVR9	191	1	197269718	197274380	gain	5680	7964370	292955427	Average_daily_gain	region1inRegion2	4662
CNVR10	220	1	224697068	224765844	gain	5680	7964370	292955427	Average_daily_gain	region1inRegion2	68776
CNVR11	255	1	259697345	259705356	gain	5680	7964370	292955427	Average_daily_gain	region1inRegion2	8011
CNVR12	261	1	268054795	268056507	gain	5680	7964370	292955427	Average_daily_gain	region1inRegion2	1712
CNVR13	266	1	271529620	271536665	gain	5680	7964370	292955427	Average_daily_gain	region1inRegion2	7045
CNVR14	279	1	284447951	284512355	loss	5680	7964370	292955427	Average_daily_gain	region1inRegion2	64404
CNVR1	83	1	88942731	88998960	gain	21209	8025530	292730648	Shoulder_subcutaneous_fat_thickness	region1inRegion2	56229
CNVR2	88	1	91934647	91942954	loss	21209	8025530	292730648	Shoulder_subcutaneous_fat_thickness	region1inRegion2	8307
CNVR3	92	1	95316086	95335767	gain	21209	8025530	292730648	Shoulder_subcutaneous_fat_thickness	region1inRegion2	19681
CNVR4	99	1	99687218	99704572	gain	21209	8025530	292730648	Shoulder_subcutaneous_fat_thickness	region1inRegion2	17354
CNVR5	101	1	99733649	99785971	gain	21209	8025530	292730648	Shoulder_subcutaneous_fat_thickness	region1inRegion2	52322
CNVR6	103	1	100019309	100027849	gain	21209	8025530	292730648	Shoulder_subcutaneous_fat_thickness	region1inRegion2	8540
CNVR7	141	1	145236440	145265439	gain	21209	8025530	292730648	Shoulder_subcutaneous_fat_thickness	region1inRegion2	28999
CNVR8	156	1	170118820	170121900	gain	21209	8025530	292730648	Shoulder_subcutaneous_fat_thickness	region1inRegion2	3080
CNVR9	191	1	197269718	197274380	gain	21209	8025530	292730648	Shoulder_subcutaneous_fat_thickness	region1inRegion2	4662
CNVR10	220	1	224697068	224765844	gain	21209	8025530	292730648	Shoulder_subcutaneous_fat_thickness	region1inRegion2	68776
CNVR11	255	1	259697345	259705356	gain	21209	8025530	292730648	Shoulder_subcutaneous_fat_thickness	region1inRegion2	8011
CNVR12	261	1	268054795	268056507	gain	21209	8025530	292730648	Shoulder_subcutaneous_fat_thickness	region1inRegion2	1712
CNVR13	266	1	271529620	271536665	gain	21209	8025530	292730648	Shoulder_subcutaneous_fat_thickness	region1inRegion2	7045
CNVR14	279	1	284447951	284512355	loss	21209	8025530	292730648	Shoulder_subcutaneous_fat_thickness	region1inRegion2	64404
CNVR1	83	1	88942731	88998960	gain	21208	10067182	292041505	backfat_at_last_rib	region1inRegion2	56229
CNVR2	88	1	91934647	91942954	loss	21208	10067182	292041505	backfat_at_last_rib	region1inRegion2	8307
CNVR3	92	1	95316086	95335767	gain	21208	10067182	292041505	backfat_at_last_rib	region1inRegion2	19681
CNVR4	99	1	99687218	99704572	gain	21208	10067182	292041505	backfat_at_last_rib	region1inRegion2	17354
CNVR5	101	1	99733649	99785971	gain	21208	10067182	292041505	backfat_at_last_rib	region1inRegion2	52322
CNVR6	103	1	100019309	100027849	gain	21208	10067182	292041505	backfat_at_last_rib	region1inRegion2	8540
CNVR7	141	1	145236440	145265439	gain	21208	10067182	292041505	backfat_at_last_rib	region1inRegion2	28999
CNVR8	156	1	170118820	170121900	gain	21208	10067182	292041505	backfat_at_last_rib	region1inRegion2	3080
CNVR9	191	1	197269718	197274380	gain	21208	10067182	292041505	backfat_at_last_rib	region1inRegion2	4662
CNVR10	220	1	224697068	224765844	gain	21208	10067182	292041505	backfat_at_last_rib	region1inRegion2	68776
CNVR11	255	1	259697345	259705356	gain	21208	10067182	292041505	backfat_at_last_rib	region1inRegion2	8011
CNVR12	261	1	268054795	268056507	gain	21208	10067182	292041505	backfat_at_last_rib	region1inRegion2	1712
CNVR13	266	1	271529620	271536665	gain	21208	10067182	292041505	backfat_at_last_rib	region1inRegion2	7045
CNVR14	279	1	284447951	284512355	loss	21208	10067182	292041505	backfat_at_last_rib	region1inRegion2	64404
CNVR1	83	1	88942731	88998960	gain	7702	11830368	116064120	HDL_cholesterol	region1inRegion2	56229
CNVR2	88	1	91934647	91942954	loss	7702	11830368	116064120	HDL_cholesterol	region1inRegion2	8307

CNVR3	92	1	95316086	95335767	gain	7702	11830368	116064120	HDL_cholesterol	region1inRegion2	19681
CNVR4	99	1	99687218	99704572	gain	7702	11830368	116064120	HDL_cholesterol	region1inRegion2	17354
CNVR5	101	1	99733649	99785971	gain	7702	11830368	116064120	HDL_cholesterol	region1inRegion2	52322
CNVR6	103	1	100019309	100027849	gain	7702	11830368	116064120	HDL_cholesterol	region1inRegion2	8540
CNVR1	83	1	88942731	88998960	gain	7567	15380966	242497921	Melanoma_susceptibility	region1inRegion2	56229
CNVR2	88	1	91934647	91942954	loss	7567	15380966	242497921	Melanoma_susceptibility	region1inRegion2	8307
CNVR3	92	1	95316086	95335767	gain	7567	15380966	242497921	Melanoma_susceptibility	region1inRegion2	19681
CNVR4	99	1	99687218	99704572	gain	7567	15380966	242497921	Melanoma_susceptibility	region1inRegion2	17354
CNVR5	101	1	99733649	99785971	gain	7567	15380966	242497921	Melanoma_susceptibility	region1inRegion2	52322
CNVR6	103	1	100019309	100027849	gain	7567	15380966	242497921	Melanoma_susceptibility	region1inRegion2	8540
CNVR7	141	1	145236440	145265439	gain	7567	15380966	242497921	Melanoma_susceptibility	region1inRegion2	28999
CNVR8	156	1	170118820	170121900	gain	7567	15380966	242497921	Melanoma_susceptibility	region1inRegion2	3080
CNVR9	191	1	197269718	197274380	gain	7567	15380966	242497921	Melanoma_susceptibility	region1inRegion2	4662
CNVR10	220	1	224697068	224765844	gain	7567	15380966	242497921	Melanoma_susceptibility	region1inRegion2	68776
CNVR1	83	1	88942731	88998960	gain	5674	16114132	252283830	backfat_at_last_rib	region1inRegion2	56229
CNVR2	88	1	91934647	91942954	loss	5674	16114132	252283830	backfat_at_last_rib	region1inRegion2	8307
CNVR3	92	1	95316086	95335767	gain	5674	16114132	252283830	backfat_at_last_rib	region1inRegion2	19681
CNVR4	99	1	99687218	99704572	gain	5674	16114132	252283830	backfat_at_last_rib	region1inRegion2	17354
CNVR5	101	1	99733649	99785971	gain	5674	16114132	252283830	backfat_at_last_rib	region1inRegion2	52322
CNVR6	103	1	100019309	100027849	gain	5674	16114132	252283830	backfat_at_last_rib	region1inRegion2	8540
CNVR7	141	1	145236440	145265439	gain	5674	16114132	252283830	backfat_at_last_rib	region1inRegion2	28999
CNVR8	156	1	170118820	170121900	gain	5674	16114132	252283830	backfat_at_last_rib	region1inRegion2	3080
CNVR9	191	1	197269718	197274380	gain	5674	16114132	252283830	backfat_at_last_rib	region1inRegion2	4662
CNVR10	220	1	224697068	224765844	gain	5674	16114132	252283830	backfat_at_last_rib	region1inRegion2	68776
CNVR1	83	1	88942731	88998960	gain	5677	16114132	252283830	Fat_to_meat_ratio	region1inRegion2	56229
CNVR2	88	1	91934647	91942954	loss	5677	16114132	252283830	Fat_to_meat_ratio	region1inRegion2	8307
CNVR3	92	1	95316086	95335767	gain	5677	16114132	252283830	Fat_to_meat_ratio	region1inRegion2	19681
CNVR4	99	1	99687218	99704572	gain	5677	16114132	252283830	Fat_to_meat_ratio	region1inRegion2	17354
CNVR5	101	1	99733649	99785971	gain	5677	16114132	252283830	Fat_to_meat_ratio	region1inRegion2	52322
CNVR6	103	1	100019309	100027849	gain	5677	16114132	252283830	Fat_to_meat_ratio	region1inRegion2	8540
CNVR7	141	1	145236440	145265439	gain	5677	16114132	252283830	Fat_to_meat_ratio	region1inRegion2	28999
CNVR8	156	1	170118820	170121900	gain	5677	16114132	252283830	Fat_to_meat_ratio	region1inRegion2	3080
CNVR9	191	1	197269718	197274380	gain	5677	16114132	252283830	Fat_to_meat_ratio	region1inRegion2	4662
CNVR10	220	1	224697068	224765844	gain	5677	16114132	252283830	Fat_to_meat_ratio	region1inRegion2	68776
CNVR1	83	1	88942731	88998960	gain	5678	16114132	252283830	Estimated_carcass_lean_content	region1inRegion2	56229
CNVR2	88	1	91934647	91942954	loss	5678	16114132	252283830	Estimated_carcass_lean_content	region1inRegion2	8307
CNVR3	92	1	95316086	95335767	gain	5678	16114132	252283830	Estimated_carcass_lean_content	region1inRegion2	19681
CNVR4	99	1	99687218	99704572	gain	5678	16114132	252283830	Estimated_carcass_lean_content	region1inRegion2	17354
CNVR5	101	1	99733649	99785971	gain	5678	16114132	252283830	Estimated_carcass_lean_content	region1inRegion2	52322
CNVR6	103	1	100019309	100027849	gain	5678	16114132	252283830	Estimated_carcass_lean_content	region1inRegion2	8540
CNVR7	141	1	145236440	145265439	gain	5678	16114132	252283830	Estimated_carcass_lean_content	region1inRegion2	28999
CNVR8	156	1	170118820	170121900	gain	5678	16114132	252283830	Estimated_carcass_lean_content	region1inRegion2	3080
CNVR9	191	1	197269718	197274380	gain	5678	16114132	252283830	Estimated_carcass_lean_content	region1inRegion2	4662
CNVR10	220	1	224697068	224765844	gain	5678	16114132	252283830	Estimated_carcass_lean_content	region1inRegion2	68776
CNVR1	83	1	88942731	88998960	gain	5679	16114132	252283830	Belly_meat_content	region1inRegion2	56229
CNVR2	88	1	91934647	91942954	loss	5679	16114132	252283830	Belly_meat_content	region1inRegion2	8307
CNVR3	92	1	95316086	95335767	gain	5679	16114132	252283830	Belly_meat_content	region1inRegion2	19681
CNVR4	99	1	99687218	99704572	gain	5679	16114132	252283830	Belly_meat_content	region1inRegion2	17354
CNVR5	101	1	99733649	99785971	gain	5679	16114132	252283830	Belly_meat_content	region1inRegion2	52322
CNVR6	103	1	100019309	100027849	gain	5679	16114132	252283830	Belly_meat_content	region1inRegion2	8540
CNVR7	141	1	145236440	145265439	gain	5679	16114132	252283830	Belly_meat_content	region1inRegion2	28999
CNVR8	156	1	170118820	170121900	gain	5679	16114132	252283830	Belly_meat_content	region1inRegion2	3080
CNVR9	191	1	197269718	197274380	gain	5679	16114132	252283830	Belly_meat_content	region1inRegion2	4662
CNVR10	220	1	224697068	224765844	gain	5679	16114132	252283830	Belly_meat_content	region1inRegion2	68776
CNVR1	83	1	88942731	88998960	gain	5232	16114132	289571736	Body_weight_(birth)	region1inRegion2	56229
CNVR2	88	1	91934647	91942954	loss	5232	16114132	289571736	Body_weight_(birth)	region1inRegion2	8307
CNVR3	92	1	95316086	95335767	gain	5232	16114132	289571736	Body_weight_(birth)	region1inRegion2	19681
CNVR4	99	1	99687218	99704572	gain	5232	16114132	289571736	Body_weight_(birth)	region1inRegion2	17354
CNVR5	101	1	99733649	99785971	gain	5232	16114132	289571736	Body_weight_(birth)	region1inRegion2	52322
CNVR6	103	1	100019309	100027849	gain	5232	16114132	289571736	Body_weight_(birth)	region1inRegion2	8540
CNVR7	141	1	145236440	145265439	gain	5232	16114132	289571736	Body_weight_(birth)	region1inRegion2	28999
CNVR8	156	1	170118820	170121900	gain	5232	16114132	289571736	Body_weight_(birth)	region1inRegion2	3080
CNVR9	191	1	197269718	197274380	gain	5232	16114132	289571736	Body_weight_(birth)	region1inRegion2	4662
CNVR10	220	1	224697068	224765844	gain	5232	16114132	289571736	Body_weight_(birth)	region1inRegion2	68776
CNVR11	255	1	259697345	259705356	gain	5232	16114132	289571736	Body_weight_(birth)	region1inRegion2	8011
CNVR12	261	1	268054795	268056507	gain	5232	16114132	289571736	Body_weight_(birth)	region1inRegion2	1712
CNVR13	266	1	271529620	271536665	gain	5232	16114132	289571736	Body_weight_(birth)	region1inRegion2	7045
CNVR14	279	1	284447951	284512355	loss	5232	16114132	289571736	Body_weight_(birth)	region1inRegion2	64404
CNVR1	83	1	88942731	88998960	gain	5217	16114132	292187438	Shoulder_subcutaneous_fat_thickness	region1inRegion2	56229
CNVR2	88	1	91934647	91942954	loss	5217	16114132	292187438	Shoulder_subcutaneous_fat_thickness	region1inRegion2	8307
CNVR3	92	1	95316086	95335767	gain	5217	16114132	292187438	Shoulder_subcutaneous_fat_thickness	region1inRegion2	19681
CNVR4	99	1	99687218	99704572	gain	5217	16114132	292187438	Shoulder_subcutaneous_fat_thickness	region1inRegion2	17354
CNVR5	101	1	99733649	99785971	gain	5217	16114132	292187438	Shoulder_subcutaneous_fat_thickness	region1inRegion2	52322
CNVR6	103	1	100019309	100027849	gain	5217	16114132	292187438	Shoulder_subcutaneous_fat_thickness	region1inRegion2	8540
CNVR7	141	1	145236440	145265439	gain	5217	16114132	292187438	Shoulder_subcutaneous_fat_thickness	region1inRegion2	28999
CNVR8	156	1	170118820	170121900	gain	5217	16114132	292187438	Shoulder_subcutaneous_fat_thickness	region1inRegion2	3080
CNVR9	191	1	197269718	197274380	gain	5217	16114132	292187438	Shoulder_subcutaneous_fat_thickness	region1inRegion2	4662
CNVR10	220	1	224697068	224765844	gain	5217	16114132	292187438	Shoulder_subcutaneous_fat_thickness	region1inRegion2	68776
CNVR11	255	1	259697345	259705356	gain	5217	16114132	292187438	Shoulder_subcutaneous_fat_thickness	region1inRegion2	8011
CNVR12	261	1	268054795	268056507	gain	5217	16114132	292187438	Shoulder_subcutaneous_fat_thickness	region1inRegion2	1712
CNVR13	266	1	271529620	271536665	gain	5217	16114132	292187438	Shoulder_subcutaneous_fat_thickness	region1inRegion2	7045
CNVR14	279	1	284447951	284512355	loss	5217	16114132	292187438	Shoulder_subcutaneous_fat_thickness	region1inRegion2	64404
CNVR1	83	1	88942731	88998960	gain	5237	16114132	292845492	backfat_above_muscle_dorsi	region1inRegion2	56229
CNVR2	88	1	91934647	91942954	loss	5237	16114132	292845492	backfat_above_muscle_dorsi	region1inRegion2	8307
CNVR3	92	1	95316086	95335767	gain	5237	16114132	292845492	backfat_above_muscle_dorsi	region1inRegion2	19681
CNVR4	99	1	99687218	99704572	gain	5237	16114132	292845492	backfat_above_muscle_dorsi	region1inRegion2	17354
CNVR5	101	1	99733649	99785971	gain	5237	16114132	292845492	backfat_above_muscle_dorsi	region1inRegion2	52322
CNVR6	103	1	100019309	100027849	gain	5237	16114132	292845492	backfat_above_muscle_dorsi	region1inRegion2	8540
CNVR7	141	1	145236440	145265439	gain	5237	16114132	292845492	backfat_above_muscle_dorsi	region1inRegion2	28999
CNVR8	156	1	170118820	170121900	gain	5237	16114132	292845492	backfat_above_muscle_dorsi	region1inRegion2	3080
CNVR9	191	1	197269718	197274380	gain	5237	16114132	292845492	backfat_above_muscle_dorsi	region1inRegion2	4662
CNVR10	220	1	224697068	224765844	gain	5237	16114132	292845492	backfat_above_muscle_dorsi	region1inRegion2	68776
CNVR11	255	1	259697345	259705356	gain	5237	16114132	292845492	backfat_above_muscle_dorsi	region1inRegion2	8011
CNVR12	261	1	268054795	268056507	gain	5237	16114132	292845492	backfat_above_muscle_dorsi	region1inRegion2	1712
CNVR13	266	1	271529620	271536665	gain	5237	16114132	292845492	backfat_above_muscle_dorsi	region1inRegion2	7045
CNVR14	279	1	284447951	284512355	loss	5237	16114132	292845492	backfat_above_muscle_dorsi	region1inRegion2	64404
CNVR1	83	1	88942731	88998960	gain	7592	18004420	242497921	Melanoma_susceptibility	region1inRegion2	56229
CNVR2	88	1	91934647	91942954	loss	7592	18004420	242497921	Melanoma_susceptibility	region1inRegion2	8307
CNVR3	92	1	95316086	95335767	gain	7592	18004420	242497921	Melanoma_susceptibility	region1inRegion2	19681
CNVR4	99	1	99687218	99704572	gain	7592	18004420	242497921	Melanoma_susceptibility	region1inRegion2	17354
CNVR5	101	1	99733649	99785971	gain	7592	18004420	242497921	Melanoma_susceptibility	region1inRegion2	52322
CNVR6	103	1	100019309	100027849	gain	7592	18004420	242497921	Melanoma_susceptibility	region1inRegion2	8540
CNVR7	141	1	145236440	145265439	gain	7592	18004420	242497921	Melanoma_susceptibility	region1inRegion2	28999
CNVR8	156	1	170118820	170121900	gain	7592	18004420	242497921	Melanoma_susceptibility	region1inRegion2	3080
CNVR9	191	1	197269718	197274380	gain	7592	18004420	242497921	Melanoma_susceptibility	region1inRegion2	4662
CNVR10	220	1	224697068	224765844	gain	7592	18004420	242497921	Melanoma_susceptibility	region1inRegion2	68776
CNVR1	83	1	88942731	88998960	gain	29689	20064508	210644363	Body_weight_(20_weeks)	region1inRegion2	56229
CNVR2	88	1	91934647	91942954	loss	29689	20064508	210644363	Body_weight_(20_weeks)	region1inRegion2	8307
CNVR3	92	1	95316086	95335767	gain	29689	20064508	210644363	Body_weight_(20_weeks)	region1inRegion2	19681
CNVR4	99	1	99687218	99704572	gain	29689	20064508	210644363	Body_weight_(20_weeks)	region1inRegion2	17354
CNVR5	101	1	99733649	99785971	gain	29689	20064508	210644363	Body_weight_(20_weeks)	region1inRegion2	52322
CNVR6	103	1	100019309	100027849	gain	29689	20064508	210644363	Body_weight_(20_weeks)	region1inRegion2	8540
CNVR7	141	1	145236440	145265439	gain	29689	20064508	210644363	Body_weight_(20_weeks)	region1inRegion2	28999
CNVR8	156	1	170118820	170121900	gain	29689	20064508	210644363	Body_weight_(20_weeks)	region1inRegion2	3080
CNVR9	191	1	197269718	197274380	gain	29689	20064508	210644363	Body_weight_(20_weeks)	region1inRegion2	4662
CNVR1	83	1	88942731	88998960	gain	15047	20064508	274130998	White_blood_cell_counts	region1inRegion2	56229
CNVR2	88	1	91934647	91942954	loss	15047	20064508	274130998	White_blood_cell_counts	region1inRegion2	8307
CNVR3	92	1	95316086	95335767	gain	15047	20064508	274130998	White_blood_cell_counts	region1inRegion2	19681
CNVR4	99	1	99687218	99704572	gain	15047	20064508	274130998	White_blood_cell_counts	region1inRegion2	17354
CNVR5	101	1	99733649	99785971	gain	15047	20064508	274130998	White_blood_cell_counts	region1inRegion2	52322
CNVR6	103	1	100019309	100027849	gain	15047	20064508	274130998	White_blood_cell_counts	region1inRegion2	8540
CNVR7	141	1	145236440	145265439	gain	15047	20064508	274130998	White_blood_cell_counts	region1inRegion2	28999

CNVR8	156	1	170118820	170121900	gain	15047	20064508	274130998	White_blood_cell_counts	region1inRegion2	3080
CNVR9	191	1	197269718	197274380	gain	15047	20064508	274130998	White_blood_cell_counts	region1inRegion2	4662
CNVR10	220	1	224697068	224765844	gain	15047	20064508	274130998	White_blood_cell_counts	region1inRegion2	68776
CNVR11	255	1	259697345	259705356	gain	15047	20064508	274130998	White_blood_cell_counts	region1inRegion2	8011
CNVR12	261	1	268054795	268056507	gain	15047	20064508	274130998	White_blood_cell_counts	region1inRegion2	1712
CNVR13	266	1	271529620	271536665	gain	15047	20064508	274130998	White_blood_cell_counts	region1inRegion2	7045
CNVR1	83	1	88942731	88998960	gain	16901	24647055	119841750	Carcass_weight_(hot)	region1inRegion2	56229
CNVR2	88	1	91934647	91942954	loss	16901	24647055	119841750	Carcass_weight_(hot)	region1inRegion2	8307
CNVR3	92	1	95316086	95335767	gain	16901	24647055	119841750	Carcass_weight_(hot)	region1inRegion2	19681
CNVR4	99	1	99687218	99704572	gain	16901	24647055	119841750	Carcass_weight_(hot)	region1inRegion2	17354
CNVR5	101	1	99733649	99785971	gain	16901	24647055	119841750	Carcass_weight_(hot)	region1inRegion2	52322
CNVR6	103	1	100019309	100027849	gain	16901	24647055	119841750	Carcass_weight_(hot)	region1inRegion2	8540
CNVR1	83	1	88942731	88998960	gain	5676	24647055	257944209	Subcutanous_fat_area	region1inRegion2	56229
CNVR2	88	1	91934647	91942954	loss	5676	24647055	257944209	Subcutanous_fat_area	region1inRegion2	8307
CNVR3	92	1	95316086	95335767	gain	5676	24647055	257944209	Subcutanous_fat_area	region1inRegion2	19681
CNVR4	99	1	99687218	99704572	gain	5676	24647055	257944209	Subcutanous_fat_area	region1inRegion2	17354
CNVR5	101	1	99733649	99785971	gain	5676	24647055	257944209	Subcutanous_fat_area	region1inRegion2	52322
CNVR6	103	1	100019309	100027849	gain	5676	24647055	257944209	Subcutanous_fat_area	region1inRegion2	8540
CNVR7	141	1	145236440	145265439	gain	5676	24647055	257944209	Subcutanous_fat_area	region1inRegion2	28999
CNVR8	156	1	170118820	170121900	gain	5676	24647055	257944209	Subcutanous_fat_area	region1inRegion2	3080
CNVR9	191	1	197269718	197274380	gain	5676	24647055	257944209	Subcutanous_fat_area	region1inRegion2	4662
CNVR10	220	1	224697068	224765844	gain	5676	24647055	257944209	Subcutanous_fat_area	region1inRegion2	68776
CNVR1	83	1	88942731	88998960	gain	5262	24673417	292845492	Body_weight_(weaning)	region1inRegion2	56229
CNVR2	88	1	91934647	91942954	loss	5262	24673417	292845492	Body_weight_(weaning)	region1inRegion2	8307
CNVR3	92	1	95316086	95335767	gain	5262	24673417	292845492	Body_weight_(weaning)	region1inRegion2	19681
CNVR4	99	1	99687218	99704572	gain	5262	24673417	292845492	Body_weight_(weaning)	region1inRegion2	17354
CNVR5	101	1	99733649	99785971	gain	5262	24673417	292845492	Body_weight_(weaning)	region1inRegion2	52322
CNVR6	103	1	100019309	100027849	gain	5262	24673417	292845492	Body_weight_(weaning)	region1inRegion2	8540
CNVR7	141	1	145236440	145265439	gain	5262	24673417	292845492	Body_weight_(weaning)	region1inRegion2	28999
CNVR8	156	1	170118820	170121900	gain	5262	24673417	292845492	Body_weight_(weaning)	region1inRegion2	3080
CNVR9	191	1	197269718	197274380	gain	5262	24673417	292845492	Body_weight_(weaning)	region1inRegion2	4662
CNVR10	220	1	224697068	224765844	gain	5262	24673417	292845492	Body_weight_(weaning)	region1inRegion2	68776
CNVR11	255	1	259697345	259705356	gain	5262	24673417	292845492	Body_weight_(weaning)	region1inRegion2	8011
CNVR12	261	1	268054795	268056507	gain	5262	24673417	292845492	Body_weight_(weaning)	region1inRegion2	1712
CNVR13	266	1	271529620	271536665	gain	5262	24673417	292845492	Body_weight_(weaning)	region1inRegion2	7045
CNVR14	279	1	284447951	284512355	loss	5262	24673417	292845492	Body_weight_(weaning)	region1inRegion2	64404
CNVR1	83	1	88942731	88998960	gain	17635	32319237	289379107	Front_feet_conformation	region1inRegion2	56229
CNVR2	88	1	91934647	91942954	loss	17635	32319237	289379107	Front_feet_conformation	region1inRegion2	8307
CNVR3	92	1	95316086	95335767	gain	17635	32319237	289379107	Front_feet_conformation	region1inRegion2	19681
CNVR4	99	1	99687218	99704572	gain	17635	32319237	289379107	Front_feet_conformation	region1inRegion2	17354
CNVR5	101	1	99733649	99785971	gain	17635	32319237	289379107	Front_feet_conformation	region1inRegion2	52322
CNVR6	103	1	100019309	100027849	gain	17635	32319237	289379107	Front_feet_conformation	region1inRegion2	8540
CNVR7	141	1	145236440	145265439	gain	17635	32319237	289379107	Front_feet_conformation	region1inRegion2	28999
CNVR8	156	1	170118820	170121900	gain	17635	32319237	289379107	Front_feet_conformation	region1inRegion2	3080
CNVR9	191	1	197269718	197274380	gain	17635	32319237	289379107	Front_feet_conformation	region1inRegion2	4662
CNVR10	220	1	224697068	224765844	gain	17635	32319237	289379107	Front_feet_conformation	region1inRegion2	68776
CNVR11	255	1	259697345	259705356	gain	17635	32319237	289379107	Front_feet_conformation	region1inRegion2	8011
CNVR12	261	1	268054795	268056507	gain	17635	32319237	289379107	Front_feet_conformation	region1inRegion2	1712
CNVR13	266	1	271529620	271536665	gain	17635	32319237	289379107	Front_feet_conformation	region1inRegion2	7045
CNVR14	279	1	284447951	284512355	loss	17635	32319237	289379107	Front_feet_conformation	region1inRegion2	64404
CNVR1	83	1	88942731	88998960	gain	16831	35389848	120316411	backfat_at_last_rib	region1inRegion2	56229
CNVR2	88	1	91934647	91942954	loss	16831	35389848	120316411	backfat_at_last_rib	region1inRegion2	8307
CNVR3	92	1	95316086	95335767	gain	16831	35389848	120316411	backfat_at_last_rib	region1inRegion2	19681
CNVR4	99	1	99687218	99704572	gain	16831	35389848	120316411	backfat_at_last_rib	region1inRegion2	17354
CNVR5	101	1	99733649	99785971	gain	16831	35389848	120316411	backfat_at_last_rib	region1inRegion2	52322
CNVR6	103	1	100019309	100027849	gain	16831	35389848	120316411	backfat_at_last_rib	region1inRegion2	8540
CNVR1	83	1	88942731	88998960	gain	3222	35389848	169149638	Dressing_percentage	region1inRegion2	56229
CNVR2	88	1	91934647	91942954	loss	3222	35389848	169149638	Dressing_percentage	region1inRegion2	8307
CNVR3	92	1	95316086	95335767	gain	3222	35389848	169149638	Dressing_percentage	region1inRegion2	19681
CNVR4	99	1	99687218	99704572	gain	3222	35389848	169149638	Dressing_percentage	region1inRegion2	17354
CNVR5	101	1	99733649	99785971	gain	3222	35389848	169149638	Dressing_percentage	region1inRegion2	52322
CNVR6	103	1	100019309	100027849	gain	3222	35389848	169149638	Dressing_percentage	region1inRegion2	8540
CNVR7	141	1	145236440	145265439	gain	3222	35389848	169149638	Dressing_percentage	region1inRegion2	28999
CNVR1	83	1	88942731	88998960	gain	4260	35389848	228164160	CD2-positive_leukocyte_number	region1inRegion2	56229
CNVR2	88	1	91934647	91942954	loss	4260	35389848	228164160	CD2-positive_leukocyte_number	region1inRegion2	8307
CNVR3	92	1	95316086	95335767	gain	4260	35389848	228164160	CD2-positive_leukocyte_number	region1inRegion2	19681
CNVR4	99	1	99687218	99704572	gain	4260	35389848	228164160	CD2-positive_leukocyte_number	region1inRegion2	17354
CNVR5	101	1	99733649	99785971	gain	4260	35389848	228164160	CD2-positive_leukocyte_number	region1inRegion2	52322
CNVR6	103	1	100019309	100027849	gain	4260	35389848	228164160	CD2-positive_leukocyte_number	region1inRegion2	8540
CNVR7	141	1	145236440	145265439	gain	4260	35389848	228164160	CD2-positive_leukocyte_number	region1inRegion2	28999
CNVR8	156	1	170118820	170121900	gain	4260	35389848	228164160	CD2-positive_leukocyte_number	region1inRegion2	3080
CNVR9	191	1	197269718	197274380	gain	4260	35389848	228164160	CD2-positive_leukocyte_number	region1inRegion2	4662
CNVR10	220	1	224697068	224765844	gain	4260	35389848	228164160	CD2-positive_leukocyte_number	region1inRegion2	68776
CNVR1	83	1	88942731	88998960	gain	4261	35389848	228164160	CD4-positive_leukocyte_number	region1inRegion2	56229
CNVR2	88	1	91934647	91942954	loss	4261	35389848	228164160	CD4-positive_leukocyte_number	region1inRegion2	8307
CNVR3	92	1	95316086	95335767	gain	4261	35389848	228164160	CD4-positive_leukocyte_number	region1inRegion2	19681
CNVR4	99	1	99687218	99704572	gain	4261	35389848	228164160	CD4-positive_leukocyte_number	region1inRegion2	17354
CNVR5	101	1	99733649	99785971	gain	4261	35389848	228164160	CD4-positive_leukocyte_number	region1inRegion2	52322
CNVR6	103	1	100019309	100027849	gain	4261	35389848	228164160	CD4-positive_leukocyte_number	region1inRegion2	8540
CNVR7	141	1	145236440	145265439	gain	4261	35389848	228164160	CD4-positive_leukocyte_number	region1inRegion2	28999
CNVR8	156	1	170118820	170121900	gain	4261	35389848	228164160	CD4-positive_leukocyte_number	region1inRegion2	3080
CNVR9	191	1	197269718	197274380	gain	4261	35389848	228164160	CD4-positive_leukocyte_number	region1inRegion2	4662
CNVR10	220	1	224697068	224765844	gain	4261	35389848	228164160	CD4-positive_leukocyte_number	region1inRegion2	68776
CNVR1	83	1	88942731	88998960	gain	79	35389848	230570794	Muscle_fat_content	region1inRegion2	56229
CNVR2	88	1	91934647	91942954	loss	79	35389848	230570794	Muscle_fat_content	region1inRegion2	8307
CNVR3	92	1	95316086	95335767	gain	79	35389848	230570794	Muscle_fat_content	region1inRegion2	19681
CNVR4	99	1	99687218	99704572	gain	79	35389848	230570794	Muscle_fat_content	region1inRegion2	17354
CNVR5	101	1	99733649	99785971	gain	79	35389848	230570794	Muscle_fat_content	region1inRegion2	52322
CNVR6	103	1	100019309	100027849	gain	79	35389848	230570794	Muscle_fat_content	region1inRegion2	8540
CNVR7	141	1	145236440	145265439	gain	79	35389848	230570794	Muscle_fat_content	region1inRegion2	28999
CNVR8	156	1	170118820	170121900	gain	79	35389848	230570794	Muscle_fat_content	region1inRegion2	3080
CNVR9	191	1	197269718	197274380	gain	79	35389848	230570794	Muscle_fat_content	region1inRegion2	4662
CNVR10	220	1	224697068	224765844	gain	79	35389848	230570794	Muscle_fat_content	region1inRegion2	68776
CNVR1	83	1	88942731	88998960	gain	5960	36144885	105086765	Estimated_carcass_lean_content	region1inRegion2	56229
CNVR2	88	1	91934647	91942954	loss	5960	36144885	105086765	Estimated_carcass_lean_content	region1inRegion2	8307
CNVR3	92	1	95316086	95335767	gain	5960	36144885	105086765	Estimated_carcass_lean_content	region1inRegion2	19681
CNVR4	99	1	99687218	99704572	gain	5960	36144885	105086765	Estimated_carcass_lean_content	region1inRegion2	17354
CNVR5	101	1	99733649	99785971	gain	5960	36144885	105086765	Estimated_carcass_lean_content	region1inRegion2	52322
CNVR6	103	1	100019309	100027849	gain	5960	36144885	105086765	Estimated_carcass_lean_content	region1inRegion2	8540
CNVR1	83	1	88942731	88998960	gain	5672	36144885	154329026	Backfat_at_tenth_rib	region1inRegion2	56229
CNVR2	88	1	91934647	91942954	loss	5672	36144885	154329026	Backfat_at_tenth_rib	region1inRegion2	8307
CNVR3	92	1	95316086	95335767	gain	5672	36144885	154329026	Backfat_at_tenth_rib	region1inRegion2	19681
CNVR4	99	1	99687218	99704572	gain	5672	36144885	154329026	Backfat_at_tenth_rib	region1inRegion2	17354
CNVR5	101	1	99733649	99785971	gain	5672	36144885	154329026	Backfat_at_tenth_rib	region1inRegion2	52322
CNVR6	103	1	100019309	100027849	gain	5672	36144885	154329026	Backfat_at_tenth_rib	region1inRegion2	8540
CNVR7	141	1	145236440	145265439	gain	5672	36144885	154329026	Backfat_at_tenth_rib	region1inRegion2	28999
CNVR1	83	1	88942731	88998960	gain	5675	36144885	154329026	Side_fat_thickness	region1inRegion2	56229
CNVR2	88	1	91934647	91942954	loss	5675	36144885	154329026	Side_fat_thickness	region1inRegion2	8307
CNVR3	92	1	95316086	95335767	gain	5675	36144885	154329026	Side_fat_thickness	region1inRegion2	19681
CNVR4	99	1	99687218	99704572	gain	5675	36144885	154329026	Side_fat_thickness	region1inRegion2	17354
CNVR5	101	1	99733649	99785971	gain	5675	36144885	154329026	Side_fat_thickness	region1inRegion2	52322
CNVR6	103	1	100019309	100027849	gain	5675	36144885	154329026	Side_fat_thickness	region1inRegion2	8540
CNVR7	141	1	145236440	145265439	gain	5675	36144885	154329026	Side_fat_thickness	region1inRegion2	28999
CNVR1	83	1	88942731	88998960	gain	9658	36144885	204653903	PRRSV_antibody_titer	region1inRegion2	56229
CNVR2	88	1	91934647	91942954	loss	9658	36144885	204653903	PRRSV_antibody_titer	region1inRegion2	8307
CNVR3	92	1	95316086	95335767	gain	9658	36144885	204653903	PRRSV_antibody_titer	region1inRegion2	19681
CNVR4	99	1	99687218	99704572	gain	9658	36144885	204653903	PRRSV_antibody_titer	region1inRegion2	17354
CNVR5	101	1	99733649	99785971	gain	9658	36144885	204653903	PRRSV_antibody_titer	region1inRegion2	52322
CNVR6	103	1	100019309	100027849	gain	9658	36144885	204653903	PRRSV_antibody_titer	region1inRegion2	8540
CNVR7	141	1	145236440	145265439	gain	9658	36144885	204653903	PRRSV_antibody_titer	region1inRegion2	28999
CNVR8	156	1	170118820	170121900	gain	9658	36144885	204653903	PRRSV_antibody_titer	region1inRegion2	3080
CNVR9	191	1	197269718	197274380	gain	9658	36144885	204653903	PRRSV_antibody_titer	region1inRegion2	4662

CNVR1	83	1	88942731	88998960	gain	31809	42824511	293329173	Skin_thickness	region1inRegion2	56229
CNVR2	88	1	91934647	91942954	loss	31809	42824511	293329173	Skin_thickness	region1inRegion2	8307
CNVR3	92	1	95316086	95335767	gain	31809	42824511	293329173	Skin_thickness	region1inRegion2	19681
CNVR4	99	1	99687218	99704572	gain	31809	42824511	293329173	Skin_thickness	region1inRegion2	17354
CNVR5	101	1	99733649	99785971	gain	31809	42824511	293329173	Skin_thickness	region1inRegion2	52322
CNVR6	103	1	100019309	100027849	gain	31809	42824511	293329173	Skin_thickness	region1inRegion2	8540
CNVR7	141	1	145236440	145265439	gain	31809	42824511	293329173	Skin_thickness	region1inRegion2	28999
CNVR8	156	1	170118820	170121900	gain	31809	42824511	293329173	Skin_thickness	region1inRegion2	3080
CNVR9	191	1	197269718	197274380	gain	31809	42824511	293329173	Skin_thickness	region1inRegion2	4662
CNVR10	220	1	224697068	224765844	gain	31809	42824511	293329173	Skin_thickness	region1inRegion2	68776
CNVR11	255	1	259697345	259705356	gain	31809	42824511	293329173	Skin_thickness	region1inRegion2	8011
CNVR12	261	1	268054795	268056507	gain	31809	42824511	293329173	Skin_thickness	region1inRegion2	1712
CNVR13	266	1	271529620	271536665	gain	31809	42824511	293329173	Skin_thickness	region1inRegion2	7045
CNVR14	279	1	284447951	284512355	loss	31809	42824511	293329173	Skin_thickness	region1inRegion2	64404
CNVR1	83	1	88942731	88998960	gain	21206	44131186	289788010	Backfat_at_rump	region1inRegion2	56229
CNVR2	88	1	91934647	91942954	loss	21206	44131186	289788010	Backfat_at_rump	region1inRegion2	8307
CNVR3	92	1	95316086	95335767	gain	21206	44131186	289788010	Backfat_at_rump	region1inRegion2	19681
CNVR4	99	1	99687218	99704572	gain	21206	44131186	289788010	Backfat_at_rump	region1inRegion2	17354
CNVR5	101	1	99733649	99785971	gain	21206	44131186	289788010	Backfat_at_rump	region1inRegion2	52322
CNVR6	103	1	100019309	100027849	gain	21206	44131186	289788010	Backfat_at_rump	region1inRegion2	8540
CNVR7	141	1	145236440	145265439	gain	21206	44131186	289788010	Backfat_at_rump	region1inRegion2	28999
CNVR8	156	1	170118820	170121900	gain	21206	44131186	289788010	Backfat_at_rump	region1inRegion2	3080
CNVR9	191	1	197269718	197274380	gain	21206	44131186	289788010	Backfat_at_rump	region1inRegion2	4662
CNVR10	220	1	224697068	224765844	gain	21206	44131186	289788010	Backfat_at_rump	region1inRegion2	68776
CNVR11	255	1	259697345	259705356	gain	21206	44131186	289788010	Backfat_at_rump	region1inRegion2	8011
CNVR12	261	1	268054795	268056507	gain	21206	44131186	289788010	Backfat_at_rump	region1inRegion2	1712
CNVR13	266	1	271529620	271536665	gain	21206	44131186	289788010	Backfat_at_rump	region1inRegion2	7045
CNVR14	279	1	284447951	284512355	loss	21206	44131186	289788010	Backfat_at_rump	region1inRegion2	64404
CNVR1	83	1	88942731	88998960	gain	21207	45762028	290786847	Leaf_fat_weight	region1inRegion2	56229
CNVR2	88	1	91934647	91942954	loss	21207	45762028	290786847	Leaf_fat_weight	region1inRegion2	8307
CNVR3	92	1	95316086	95335767	gain	21207	45762028	290786847	Leaf_fat_weight	region1inRegion2	19681
CNVR4	99	1	99687218	99704572	gain	21207	45762028	290786847	Leaf_fat_weight	region1inRegion2	17354
CNVR5	101	1	99733649	99785971	gain	21207	45762028	290786847	Leaf_fat_weight	region1inRegion2	52322
CNVR6	103	1	100019309	100027849	gain	21207	45762028	290786847	Leaf_fat_weight	region1inRegion2	8540
CNVR7	141	1	145236440	145265439	gain	21207	45762028	290786847	Leaf_fat_weight	region1inRegion2	28999
CNVR8	156	1	170118820	170121900	gain	21207	45762028	290786847	Leaf_fat_weight	region1inRegion2	3080
CNVR9	191	1	197269718	197274380	gain	21207	45762028	290786847	Leaf_fat_weight	region1inRegion2	4662
CNVR10	220	1	224697068	224765844	gain	21207	45762028	290786847	Leaf_fat_weight	region1inRegion2	68776
CNVR11	255	1	259697345	259705356	gain	21207	45762028	290786847	Leaf_fat_weight	region1inRegion2	8011
CNVR12	261	1	268054795	268056507	gain	21207	45762028	290786847	Leaf_fat_weight	region1inRegion2	1712
CNVR13	266	1	271529620	271536665	gain	21207	45762028	290786847	Leaf_fat_weight	region1inRegion2	7045
CNVR14	279	1	284447951	284512355	loss	21207	45762028	290786847	Leaf_fat_weight	region1inRegion2	64404
CNVR1	83	1	88942731	88998960	gain	5223	45939845	292033322	Teat_number	region1inRegion2	56229
CNVR2	88	1	91934647	91942954	loss	5223	45939845	292033322	Teat_number	region1inRegion2	8307
CNVR3	92	1	95316086	95335767	gain	5223	45939845	292033322	Teat_number	region1inRegion2	19681
CNVR4	99	1	99687218	99704572	gain	5223	45939845	292033322	Teat_number	region1inRegion2	17354
CNVR5	101	1	99733649	99785971	gain	5223	45939845	292033322	Teat_number	region1inRegion2	52322
CNVR6	103	1	100019309	100027849	gain	5223	45939845	292033322	Teat_number	region1inRegion2	8540
CNVR7	141	1	145236440	145265439	gain	5223	45939845	292033322	Teat_number	region1inRegion2	28999
CNVR8	156	1	170118820	170121900	gain	5223	45939845	292033322	Teat_number	region1inRegion2	3080
CNVR9	191	1	197269718	197274380	gain	5223	45939845	292033322	Teat_number	region1inRegion2	4662
CNVR10	220	1	224697068	224765844	gain	5223	45939845	292033322	Teat_number	region1inRegion2	68776
CNVR11	255	1	259697345	259705356	gain	5223	45939845	292033322	Teat_number	region1inRegion2	8011
CNVR12	261	1	268054795	268056507	gain	5223	45939845	292033322	Teat_number	region1inRegion2	1712
CNVR13	266	1	271529620	271536665	gain	5223	45939845	292033322	Teat_number	region1inRegion2	7045
CNVR14	279	1	284447951	284512355	loss	5223	45939845	292033322	Teat_number	region1inRegion2	64404
CNVR1	83	1	88942731	88998960	gain	4019	48054350	117332062	Marbling	region1inRegion2	56229
CNVR2	88	1	91934647	91942954	loss	4019	48054350	117332062	Marbling	region1inRegion2	8307
CNVR3	92	1	95316086	95335767	gain	4019	48054350	117332062	Marbling	region1inRegion2	19681
CNVR4	99	1	99687218	99704572	gain	4019	48054350	117332062	Marbling	region1inRegion2	17354
CNVR5	101	1	99733649	99785971	gain	4019	48054350	117332062	Marbling	region1inRegion2	52322
CNVR6	103	1	100019309	100027849	gain	4019	48054350	117332062	Marbling	region1inRegion2	8540
CNVR1	83	1	88942731	88998960	gain	16859	52874641	130756529	Carcass_weight_(hot)	region1inRegion2	56229
CNVR2	88	1	91934647	91942954	loss	16859	52874641	130756529	Carcass_weight_(hot)	region1inRegion2	8307
CNVR3	92	1	95316086	95335767	gain	16859	52874641	130756529	Carcass_weight_(hot)	region1inRegion2	19681
CNVR4	99	1	99687218	99704572	gain	16859	52874641	130756529	Carcass_weight_(hot)	region1inRegion2	17354
CNVR5	101	1	99733649	99785971	gain	16859	52874641	130756529	Carcass_weight_(hot)	region1inRegion2	52322
CNVR6	103	1	100019309	100027849	gain	16859	52874641	130756529	Carcass_weight_(hot)	region1inRegion2	8540
CNVR1	83	1	88942731	88998960	gain	12711	52874641	169149638	Carcass_weight_(cold)	region1inRegion2	56229
CNVR2	88	1	91934647	91942954	loss	12711	52874641	169149638	Carcass_weight_(cold)	region1inRegion2	8307
CNVR3	92	1	95316086	95335767	gain	12711	52874641	169149638	Carcass_weight_(cold)	region1inRegion2	19681
CNVR4	99	1	99687218	99704572	gain	12711	52874641	169149638	Carcass_weight_(cold)	region1inRegion2	17354
CNVR5	101	1	99733649	99785971	gain	12711	52874641	169149638	Carcass_weight_(cold)	region1inRegion2	52322
CNVR6	103	1	100019309	100027849	gain	12711	52874641	169149638	Carcass_weight_(cold)	region1inRegion2	8540
CNVR7	141	1	145236440	145265439	gain	12711	52874641	169149638	Carcass_weight_(cold)	region1inRegion2	28999
CNVR1	83	1	88942731	88998960	gain	12719	52874641	169149638	External_fat_on_ham	region1inRegion2	56229
CNVR2	88	1	91934647	91942954	loss	12719	52874641	169149638	External_fat_on_ham	region1inRegion2	8307
CNVR3	92	1	95316086	95335767	gain	12719	52874641	169149638	External_fat_on_ham	region1inRegion2	19681
CNVR4	99	1	99687218	99704572	gain	12719	52874641	169149638	External_fat_on_ham	region1inRegion2	17354
CNVR5	101	1	99733649	99785971	gain	12719	52874641	169149638	External_fat_on_ham	region1inRegion2	52322
CNVR6	103	1	100019309	100027849	gain	12719	52874641	169149638	External_fat_on_ham	region1inRegion2	8540
CNVR7	141	1	145236440	145265439	gain	12719	52874641	169149638	External_fat_on_ham	region1inRegion2	28999
CNVR1	83	1	88942731	88998960	gain	12725	52874641	169149638	Backfat_weight	region1inRegion2	56229
CNVR2	88	1	91934647	91942954	loss	12725	52874641	169149638	Backfat_weight	region1inRegion2	8307
CNVR3	92	1	95316086	95335767	gain	12725	52874641	169149638	Backfat_weight	region1inRegion2	19681
CNVR4	99	1	99687218	99704572	gain	12725	52874641	169149638	Backfat_weight	region1inRegion2	17354
CNVR5	101	1	99733649	99785971	gain	12725	52874641	169149638	Backfat_weight	region1inRegion2	52322
CNVR6	103	1	100019309	100027849	gain	12725	52874641	169149638	Backfat_weight	region1inRegion2	8540
CNVR7	141	1	145236440	145265439	gain	12725	52874641	169149638	Backfat_weight	region1inRegion2	28999
CNVR1	83	1	88942731	88998960	gain	860	52874641	169149638	Head_weight	region1inRegion2	56229
CNVR2	88	1	91934647	91942954	loss	860	52874641	169149638	Head_weight	region1inRegion2	8307
CNVR3	92	1	95316086	95335767	gain	860	52874641	169149638	Head_weight	region1inRegion2	19681
CNVR4	99	1	99687218	99704572	gain	860	52874641	169149638	Head_weight	region1inRegion2	17354
CNVR5	101	1	99733649	99785971	gain	860	52874641	169149638	Head_weight	region1inRegion2	52322
CNVR6	103	1	100019309	100027849	gain	860	52874641	169149638	Head_weight	region1inRegion2	8540
CNVR7	141	1	145236440	145265439	gain	860	52874641	169149638	Head_weight	region1inRegion2	28999
CNVR1	83	1	88942731	88998960	gain	871	52874641	169149638	Feed_intake	region1inRegion2	56229
CNVR2	88	1	91934647	91942954	loss	871	52874641	169149638	Feed_intake	region1inRegion2	8307
CNVR3	92	1	95316086	95335767	gain	871	52874641	169149638	Feed_intake	region1inRegion2	19681
CNVR4	99	1	99687218	99704572	gain	871	52874641	169149638	Feed_intake	region1inRegion2	17354
CNVR5	101	1	99733649	99785971	gain	871	52874641	169149638	Feed_intake	region1inRegion2	52322
CNVR6	103	1	100019309	100027849	gain	871	52874641	169149638	Feed_intake	region1inRegion2	8540
CNVR7	141	1	145236440	145265439	gain	871	52874641	169149638	Feed_intake	region1inRegion2	28999
CNVR1	83	1	88942731	88998960	gain	872	52874641	169149638	Liver_weight	region1inRegion2	56229
CNVR2	88	1	91934647	91942954	loss	872	52874641	169149638	Liver_weight	region1inRegion2	8307
CNVR3	92	1	95316086	95335767	gain	872	52874641	169149638	Liver_weight	region1inRegion2	19681
CNVR4	99	1	99687218	99704572	gain	872	52874641	169149638	Liver_weight	region1inRegion2	17354
CNVR5	101	1	99733649	99785971	gain	872	52874641	169149638	Liver_weight	region1inRegion2	52322
CNVR6	103	1	100019309	100027849	gain	872	52874641	169149638	Liver_weight	region1inRegion2	8540
CNVR7	141	1	145236440	145265439	gain	872	52874641	169149638	Liver_weight	region1inRegion2	28999
CNVR1	83	1	88942731	88998960	gain	12715	52874641	233806417	External_fat_on_ham	region1inRegion2	56229
CNVR2	88	1	91934647	91942954	loss	12715	52874641	233806417	External_fat_on_ham	region1inRegion2	8307
CNVR3	92	1	95316086	95335767	gain	12715	52874641	233806417	External_fat_on_ham	region1inRegion2	19681
CNVR4	99	1	99687218	99704572	gain	12715	52874641	233806417	External_fat_on_ham	region1inRegion2	17354
CNVR5	101	1	99733649	99785971	gain	12715	52874641	233806417	External_fat_on_ham	region1inRegion2	52322
CNVR6	103	1	100019309	100027849	gain	12715	52874641	233806417	External_fat_on_ham	region1inRegion2	8540
CNVR7	141	1	145236440	145265439	gain	12715	52874641	233806417	External_fat_on_ham	region1inRegion2	28999
CNVR8	156	1	170118820	170121900	gain	12715	52874641	233806417	External_fat_on_ham	region1inRegion2	3080
CNVR9	191	1	197269718	197274380	gain	12715	52874641	233806417	External_fat_on_ham	region1inRegion2	4662
CNVR10	220	1	224697068	224765844	gain	12715	52874641	233806417	External_fat_on_ham	region1inRegion2	68776
CNVR1	83	1	88942731	88998960	gain	16886	52874641	233806417	Average_daily_gain	region1inRegion2	56229
CNVR2	88	1	91934647	91942954	loss	16886	52874641	233806417	Average_daily_gain	region1inRegion2	8307

CNVR3	92	1	95316086	95335767	gain	16886	52874641	233806417	Average_daily_gain	region1inRegion2	19681
CNVR4	99	1	99687218	99704572	gain	16886	52874641	233806417	Average_daily_gain	region1inRegion2	17354
CNVR5	101	1	99733649	99785971	gain	16886	52874641	233806417	Average_daily_gain	region1inRegion2	52322
CNVR6	103	1	100019309	100027849	gain	16886	52874641	233806417	Average_daily_gain	region1inRegion2	8540
CNVR7	141	1	145236440	145265439	gain	16886	52874641	233806417	Average_daily_gain	region1inRegion2	28999
CNVR8	156	1	170118820	170121900	gain	16886	52874641	233806417	Average_daily_gain	region1inRegion2	3080
CNVR9	191	1	197269718	197274380	gain	16886	52874641	233806417	Average_daily_gain	region1inRegion2	4662
CNVR10	220	1	224697068	224765844	gain	16886	52874641	233806417	Average_daily_gain	region1inRegion2	68776
CNVR1	83	1	88942731	88998960	gain	3673	62261447	133275468	Average_daily_gain	region1inRegion2	56229
CNVR2	88	1	91934647	91942954	loss	3673	62261447	133275468	Average_daily_gain	region1inRegion2	8307
CNVR3	92	1	95316086	95335767	gain	3673	62261447	133275468	Average_daily_gain	region1inRegion2	19681
CNVR4	99	1	99687218	99704572	gain	3673	62261447	133275468	Average_daily_gain	region1inRegion2	17354
CNVR5	101	1	99733649	99785971	gain	3673	62261447	133275468	Average_daily_gain	region1inRegion2	52322
CNVR6	103	1	100019309	100027849	gain	3673	62261447	133275468	Average_daily_gain	region1inRegion2	8540
CNVR1	83	1	88942731	88998960	gain	3693	62261447	133275468	PH_for_Longissmus_dorsi	region1inRegion2	56229
CNVR2	88	1	91934647	91942954	loss	3693	62261447	133275468	PH_for_Longissmus_dorsi	region1inRegion2	8307
CNVR3	92	1	95316086	95335767	gain	3693	62261447	133275468	PH_for_Longissmus_dorsi	region1inRegion2	19681
CNVR4	99	1	99687218	99704572	gain	3693	62261447	133275468	PH_for_Longissmus_dorsi	region1inRegion2	17354
CNVR5	101	1	99733649	99785971	gain	3693	62261447	133275468	PH_for_Longissmus_dorsi	region1inRegion2	52322
CNVR6	103	1	100019309	100027849	gain	3693	62261447	133275468	PH_for_Longissmus_dorsi	region1inRegion2	8540
CNVR1	83	1	88942731	88998960	gain	3694	62261447	133275468	PH_for_Longissmus_dorsi	region1inRegion2	56229
CNVR2	88	1	91934647	91942954	loss	3694	62261447	133275468	PH_for_Longissmus_dorsi	region1inRegion2	8307
CNVR3	92	1	95316086	95335767	gain	3694	62261447	133275468	PH_for_Longissmus_dorsi	region1inRegion2	19681
CNVR4	99	1	99687218	99704572	gain	3694	62261447	133275468	PH_for_Longissmus_dorsi	region1inRegion2	17354
CNVR5	101	1	99733649	99785971	gain	3694	62261447	133275468	PH_for_Longissmus_dorsi	region1inRegion2	52322
CNVR6	103	1	100019309	100027849	gain	3694	62261447	133275468	PH_for_Longissmus_dorsi	region1inRegion2	8540
CNVR1	83	1	88942731	88998960	gain	3695	62261447	133275468	Carcass_weight_(hot)	region1inRegion2	56229
CNVR2	88	1	91934647	91942954	loss	3695	62261447	133275468	Carcass_weight_(hot)	region1inRegion2	8307
CNVR3	92	1	95316086	95335767	gain	3695	62261447	133275468	Carcass_weight_(hot)	region1inRegion2	19681
CNVR4	99	1	99687218	99704572	gain	3695	62261447	133275468	Carcass_weight_(hot)	region1inRegion2	17354
CNVR5	101	1	99733649	99785971	gain	3695	62261447	133275468	Carcass_weight_(hot)	region1inRegion2	52322
CNVR6	103	1	100019309	100027849	gain	3695	62261447	133275468	Carcass_weight_(hot)	region1inRegion2	8540
CNVR1	83	1	88942731	88998960	gain	4107	62261447	133275468	backfat_at_P2_position	region1inRegion2	56229
CNVR2	88	1	91934647	91942954	loss	4107	62261447	133275468	backfat_at_P2_position	region1inRegion2	8307
CNVR3	92	1	95316086	95335767	gain	4107	62261447	133275468	backfat_at_P2_position	region1inRegion2	19681
CNVR4	99	1	99687218	99704572	gain	4107	62261447	133275468	backfat_at_P2_position	region1inRegion2	17354
CNVR5	101	1	99733649	99785971	gain	4107	62261447	133275468	backfat_at_P2_position	region1inRegion2	52322
CNVR6	103	1	100019309	100027849	gain	4107	62261447	133275468	backfat_at_P2_position	region1inRegion2	8540
CNVR1	83	1	88942731	88998960	gain	4108	62261447	133275468	backfat_at_P2_position	region1inRegion2	56229
CNVR2	88	1	91934647	91942954	loss	4108	62261447	133275468	backfat_at_P2_position	region1inRegion2	8307
CNVR3	92	1	95316086	95335767	gain	4108	62261447	133275468	backfat_at_P2_position	region1inRegion2	19681
CNVR4	99	1	99687218	99704572	gain	4108	62261447	133275468	backfat_at_P2_position	region1inRegion2	17354
CNVR5	101	1	99733649	99785971	gain	4108	62261447	133275468	backfat_at_P2_position	region1inRegion2	52322
CNVR6	103	1	100019309	100027849	gain	4108	62261447	133275468	backfat_at_P2_position	region1inRegion2	8540
CNVR1	83	1	88942731	88998960	gain	4109	62261447	133275468	Lean_meat_percentage	region1inRegion2	56229
CNVR2	88	1	91934647	91942954	loss	4109	62261447	133275468	Lean_meat_percentage	region1inRegion2	8307
CNVR3	92	1	95316086	95335767	gain	4109	62261447	133275468	Lean_meat_percentage	region1inRegion2	19681
CNVR4	99	1	99687218	99704572	gain	4109	62261447	133275468	Lean_meat_percentage	region1inRegion2	17354
CNVR5	101	1	99733649	99785971	gain	4109	62261447	133275468	Lean_meat_percentage	region1inRegion2	52322
CNVR6	103	1	100019309	100027849	gain	4109	62261447	133275468	Lean_meat_percentage	region1inRegion2	8540
CNVR1	83	1	88942731	88998960	gain	4110	62261447	133275468	PH_for_Longissmus_dorsi	region1inRegion2	56229
CNVR2	88	1	91934647	91942954	loss	4110	62261447	133275468	PH_for_Longissmus_dorsi	region1inRegion2	8307
CNVR3	92	1	95316086	95335767	gain	4110	62261447	133275468	PH_for_Longissmus_dorsi	region1inRegion2	19681
CNVR4	99	1	99687218	99704572	gain	4110	62261447	133275468	PH_for_Longissmus_dorsi	region1inRegion2	17354
CNVR5	101	1	99733649	99785971	gain	4110	62261447	133275468	PH_for_Longissmus_dorsi	region1inRegion2	52322
CNVR6	103	1	100019309	100027849	gain	4110	62261447	133275468	PH_for_Longissmus_dorsi	region1inRegion2	8540
CNVR1	83	1	88942731	88998960	gain	329	62261447	162073157	Average_daily_gain	region1inRegion2	56229
CNVR2	88	1	91934647	91942954	loss	329	62261447	162073157	Average_daily_gain	region1inRegion2	8307
CNVR3	92	1	95316086	95335767	gain	329	62261447	162073157	Average_daily_gain	region1inRegion2	19681
CNVR4	99	1	99687218	99704572	gain	329	62261447	162073157	Average_daily_gain	region1inRegion2	17354
CNVR5	101	1	99733649	99785971	gain	329	62261447	162073157	Average_daily_gain	region1inRegion2	52322
CNVR6	103	1	100019309	100027849	gain	329	62261447	162073157	Average_daily_gain	region1inRegion2	8540
CNVR7	141	1	145236440	145265439	gain	329	62261447	162073157	Average_daily_gain	region1inRegion2	28999
CNVR1	83	1	88942731	88998960	gain	611	62261447	233806417	White_blood_cell_counts	region1inRegion2	56229
CNVR2	88	1	91934647	91942954	loss	611	62261447	233806417	White_blood_cell_counts	region1inRegion2	8307
CNVR3	92	1	95316086	95335767	gain	611	62261447	233806417	White_blood_cell_counts	region1inRegion2	19681
CNVR4	99	1	99687218	99704572	gain	611	62261447	233806417	White_blood_cell_counts	region1inRegion2	17354
CNVR5	101	1	99733649	99785971	gain	611	62261447	233806417	White_blood_cell_counts	region1inRegion2	52322
CNVR6	103	1	100019309	100027849	gain	611	62261447	233806417	White_blood_cell_counts	region1inRegion2	8540
CNVR7	141	1	145236440	145265439	gain	611	62261447	233806417	White_blood_cell_counts	region1inRegion2	28999
CNVR8	156	1	170118820	170121900	gain	611	62261447	233806417	White_blood_cell_counts	region1inRegion2	3080
CNVR9	191	1	197269718	197274380	gain	611	62261447	233806417	White_blood_cell_counts	region1inRegion2	4662
CNVR10	220	1	224697068	224765844	gain	611	62261447	233806417	White_blood_cell_counts	region1inRegion2	68776
CNVR1	83	1	88942731	88998960	gain	8908	62261447	233806417	Front_feet_conformation	region1inRegion2	56229
CNVR2	88	1	91934647	91942954	loss	8908	62261447	233806417	Front_feet_conformation	region1inRegion2	8307
CNVR3	92	1	95316086	95335767	gain	8908	62261447	233806417	Front_feet_conformation	region1inRegion2	19681
CNVR4	99	1	99687218	99704572	gain	8908	62261447	233806417	Front_feet_conformation	region1inRegion2	17354
CNVR5	101	1	99733649	99785971	gain	8908	62261447	233806417	Front_feet_conformation	region1inRegion2	52322
CNVR6	103	1	100019309	100027849	gain	8908	62261447	233806417	Front_feet_conformation	region1inRegion2	8540
CNVR7	141	1	145236440	145265439	gain	8908	62261447	233806417	Front_feet_conformation	region1inRegion2	28999
CNVR8	156	1	170118820	170121900	gain	8908	62261447	233806417	Front_feet_conformation	region1inRegion2	3080
CNVR9	191	1	197269718	197274380	gain	8908	62261447	233806417	Front_feet_conformation	region1inRegion2	4662
CNVR10	220	1	224697068	224765844	gain	8908	62261447	233806417	Front_feet_conformation	region1inRegion2	68776
CNVR1	83	1	88942731	88998960	gain	8910	62261447	233806417	Hind_leg_conformation	region1inRegion2	56229
CNVR2	88	1	91934647	91942954	loss	8910	62261447	233806417	Hind_leg_conformation	region1inRegion2	8307
CNVR3	92	1	95316086	95335767	gain	8910	62261447	233806417	Hind_leg_conformation	region1inRegion2	19681
CNVR4	99	1	99687218	99704572	gain	8910	62261447	233806417	Hind_leg_conformation	region1inRegion2	17354
CNVR5	101	1	99733649	99785971	gain	8910	62261447	233806417	Hind_leg_conformation	region1inRegion2	52322
CNVR6	103	1	100019309	100027849	gain	8910	62261447	233806417	Hind_leg_conformation	region1inRegion2	8540
CNVR7	141	1	145236440	145265439	gain	8910	62261447	233806417	Hind_leg_conformation	region1inRegion2	28999
CNVR8	156	1	170118820	170121900	gain	8910	62261447	233806417	Hind_leg_conformation	region1inRegion2	3080
CNVR9	191	1	197269718	197274380	gain	8910	62261447	233806417	Hind_leg_conformation	region1inRegion2	4662
CNVR10	220	1	224697068	224765844	gain	8910	62261447	233806417	Hind_leg_conformation	region1inRegion2	68776
CNVR1	83	1	88942731	88998960	gain	5420	63012552	226764071	hematocrit	region1inRegion2	56229
CNVR2	88	1	91934647	91942954	loss	5420	63012552	226764071	hematocrit	region1inRegion2	8307
CNVR3	92	1	95316086	95335767	gain	5420	63012552	226764071	hematocrit	region1inRegion2	19681
CNVR4	99	1	99687218	99704572	gain	5420	63012552	226764071	hematocrit	region1inRegion2	17354
CNVR5	101	1	99733649	99785971	gain	5420	63012552	226764071	hematocrit	region1inRegion2	52322
CNVR6	103	1	100019309	100027849	gain	5420	63012552	226764071	hematocrit	region1inRegion2	8540
CNVR7	141	1	145236440	145265439	gain	5420	63012552	226764071	hematocrit	region1inRegion2	28999
CNVR8	156	1	170118820	170121900	gain	5420	63012552	226764071	hematocrit	region1inRegion2	3080
CNVR9	191	1	197269718	197274380	gain	5420	63012552	226764071	hematocrit	region1inRegion2	4662
CNVR10	220	1	224697068	224765844	gain	5420	63012552	226764071	hematocrit	region1inRegion2	68776
CNVR1	83	1	88942731	88998960	gain	5468	63012552	226764071	Lymphocyte_number	region1inRegion2	56229
CNVR2	88	1	91934647	91942954	loss	5468	63012552	226764071	Lymphocyte_number	region1inRegion2	8307
CNVR3	92	1	95316086	95335767	gain	5468	63012552	226764071	Lymphocyte_number	region1inRegion2	19681
CNVR4	99	1	99687218	99704572	gain	5468	63012552	226764071	Lymphocyte_number	region1inRegion2	17354
CNVR5	101	1	99733649	99785971	gain	5468	63012552	226764071	Lymphocyte_number	region1inRegion2	52322
CNVR6	103	1	100019309	100027849	gain	5468	63012552	226764071	Lymphocyte_number	region1inRegion2	8540
CNVR7	141	1	145236440	145265439	gain	5468	63012552	226764071	Lymphocyte_number	region1inRegion2	28999
CNVR8	156	1	170118820	170121900	gain	5468	63012552	226764071	Lymphocyte_number	region1inRegion2	3080
CNVR9	191	1	197269718	197274380	gain	5468	63012552	226764071	Lymphocyte_number	region1inRegion2	4662
CNVR10	220	1	224697068	224765844	gain	5468	63012552	226764071	Lymphocyte_number	region1inRegion2	68776
CNVR1	83	1	88942731	88998960	gain	6366	63012552	226764071	Creatinine_level	region1inRegion2	56229
CNVR2	88	1	91934647	91942954	loss	6366	63012552	226764071	Creatinine_level	region1inRegion2	8307
CNVR3	92	1	95316086	95335767	gain	6366	63012552	226764071	Creatinine_level	region1inRegion2	19681
CNVR4	99	1	99687218	99704572	gain	6366	63012552	226764071	Creatinine_level	region1inRegion2	17354
CNVR5	101	1	99733649	99785971	gain	6366	63012552	226764071	Creatinine_level	region1inRegion2	52322
CNVR6	103	1	100019309	100027849	gain	6366	63012552	226764071	Creatinine_level	region1inRegion2	8540
CNVR7	141	1	145236440	145265439	gain	6366	63012552	226764071	Creatinine_level	region1inRegion2	28999
CNVR8	156	1	170118820	170121900	gain	6366	63012552	226764071	Creatinine_level	region1inRegion2	3080
CNVR9	191	1	197269718	197274380	gain	6366	63012552	226764071	Creatinine_level	region1inRegion2	4662

CNVR10	220	1	224697068	224765844	gain	6366	63012552	226764071	Creatinine_level	region1inRegion2	68776
CNVR1	83	1	88942731	88998960	gain	6380	63012552	226764071	Potassium_level	region1inRegion2	56229
CNVR2	88	1	91934647	91942954	loss	6380	63012552	226764071	Potassium_level	region1inRegion2	8307
CNVR3	92	1	95316086	95335767	gain	6380	63012552	226764071	Potassium_level	region1inRegion2	19681
CNVR4	99	1	99687218	99704572	gain	6380	63012552	226764071	Potassium_level	region1inRegion2	17354
CNVR5	101	1	99733649	99785971	gain	6380	63012552	226764071	Potassium_level	region1inRegion2	52322
CNVR6	103	1	100019309	100027849	gain	6380	63012552	226764071	Potassium_level	region1inRegion2	8540
CNVR7	141	1	145236440	145265439	gain	6380	63012552	226764071	Potassium_level	region1inRegion2	28999
CNVR8	156	1	170118820	170121900	gain	6380	63012552	226764071	Potassium_level	region1inRegion2	3080
CNVR9	191	1	197269718	197274380	gain	6380	63012552	226764071	Potassium_level	region1inRegion2	4662
CNVR10	220	1	224697068	224765844	gain	6380	63012552	226764071	Potassium_level	region1inRegion2	68776
CNVR1	83	1	88942731	88998960	gain	7936	64660371	303244141	Drip_loss	region1inRegion2	56229
CNVR2	88	1	91934647	91942954	loss	7936	64660371	303244141	Drip_loss	region1inRegion2	8307
CNVR3	92	1	95316086	95335767	gain	7936	64660371	303244141	Drip_loss	region1inRegion2	19681
CNVR4	99	1	99687218	99704572	gain	7936	64660371	303244141	Drip_loss	region1inRegion2	17354
CNVR5	101	1	99733649	99785971	gain	7936	64660371	303244141	Drip_loss	region1inRegion2	52322
CNVR6	103	1	100019309	100027849	gain	7936	64660371	303244141	Drip_loss	region1inRegion2	8540
CNVR7	141	1	145236440	145265439	gain	7936	64660371	303244141	Drip_loss	region1inRegion2	28999
CNVR8	156	1	170118820	170121900	gain	7936	64660371	303244141	Drip_loss	region1inRegion2	3080
CNVR9	191	1	197269718	197274380	gain	7936	64660371	303244141	Drip_loss	region1inRegion2	4662
CNVR10	220	1	224697068	224765844	gain	7936	64660371	303244141	Drip_loss	region1inRegion2	68776
CNVR11	255	1	259697345	259705356	gain	7936	64660371	303244141	Drip_loss	region1inRegion2	8011
CNVR12	261	1	268054795	268056507	gain	7936	64660371	303244141	Drip_loss	region1inRegion2	1712
CNVR13	266	1	271529620	271536665	gain	7936	64660371	303244141	Drip_loss	region1inRegion2	7045
CNVR14	279	1	284447951	284512355	loss	7936	64660371	303244141	Drip_loss	region1inRegion2	64404
CNVR15	288	1	295235629	295379020	gain	7936	64660371	303244141	Drip_loss	region1inRegion2	143391
CNVR16	290	1	296193901	296202305	loss-gain	7936	64660371	303244141	Drip_loss	region1inRegion2	8404
CNVR17	292	1	296918534	296982162	loss	7936	64660371	303244141	Drip_loss	region1inRegion2	63628
CNVR1	83	1	88942731	88998960	gain	8063	64660371	303244141	Drip_loss	region1inRegion2	56229
CNVR2	88	1	91934647	91942954	loss	8063	64660371	303244141	Drip_loss	region1inRegion2	8307
CNVR3	92	1	95316086	95335767	gain	8063	64660371	303244141	Drip_loss	region1inRegion2	19681
CNVR4	99	1	99687218	99704572	gain	8063	64660371	303244141	Drip_loss	region1inRegion2	17354
CNVR5	101	1	99733649	99785971	gain	8063	64660371	303244141	Drip_loss	region1inRegion2	52322
CNVR6	103	1	100019309	100027849	gain	8063	64660371	303244141	Drip_loss	region1inRegion2	8540
CNVR7	141	1	145236440	145265439	gain	8063	64660371	303244141	Drip_loss	region1inRegion2	28999
CNVR8	156	1	170118820	170121900	gain	8063	64660371	303244141	Drip_loss	region1inRegion2	3080
CNVR9	191	1	197269718	197274380	gain	8063	64660371	303244141	Drip_loss	region1inRegion2	4662
CNVR10	220	1	224697068	224765844	gain	8063	64660371	303244141	Drip_loss	region1inRegion2	68776
CNVR11	255	1	259697345	259705356	gain	8063	64660371	303244141	Drip_loss	region1inRegion2	8011
CNVR12	261	1	268054795	268056507	gain	8063	64660371	303244141	Drip_loss	region1inRegion2	1712
CNVR13	266	1	271529620	271536665	gain	8063	64660371	303244141	Drip_loss	region1inRegion2	7045
CNVR14	279	1	284447951	284512355	loss	8063	64660371	303244141	Drip_loss	region1inRegion2	64404
CNVR15	288	1	295235629	295379020	gain	8063	64660371	303244141	Drip_loss	region1inRegion2	143391
CNVR16	290	1	296193901	296202305	loss-gain	8063	64660371	303244141	Drip_loss	region1inRegion2	8404
CNVR17	292	1	296918534	296982162	loss	8063	64660371	303244141	Drip_loss	region1inRegion2	63628
CNVR1	83	1	88942731	88998960	gain	8131	64660371	303244141	Drip_loss	region1inRegion2	56229
CNVR2	88	1	91934647	91942954	loss	8131	64660371	303244141	Drip_loss	region1inRegion2	8307
CNVR3	92	1	95316086	95335767	gain	8131	64660371	303244141	Drip_loss	region1inRegion2	19681
CNVR4	99	1	99687218	99704572	gain	8131	64660371	303244141	Drip_loss	region1inRegion2	17354
CNVR5	101	1	99733649	99785971	gain	8131	64660371	303244141	Drip_loss	region1inRegion2	52322
CNVR6	103	1	100019309	100027849	gain	8131	64660371	303244141	Drip_loss	region1inRegion2	8540
CNVR7	141	1	145236440	145265439	gain	8131	64660371	303244141	Drip_loss	region1inRegion2	28999
CNVR8	156	1	170118820	170121900	gain	8131	64660371	303244141	Drip_loss	region1inRegion2	3080
CNVR9	191	1	197269718	197274380	gain	8131	64660371	303244141	Drip_loss	region1inRegion2	4662
CNVR10	220	1	224697068	224765844	gain	8131	64660371	303244141	Drip_loss	region1inRegion2	68776
CNVR11	255	1	259697345	259705356	gain	8131	64660371	303244141	Drip_loss	region1inRegion2	8011
CNVR12	261	1	268054795	268056507	gain	8131	64660371	303244141	Drip_loss	region1inRegion2	1712
CNVR13	266	1	271529620	271536665	gain	8131	64660371	303244141	Drip_loss	region1inRegion2	7045
CNVR14	279	1	284447951	284512355	loss	8131	64660371	303244141	Drip_loss	region1inRegion2	64404
CNVR15	288	1	295235629	295379020	gain	8131	64660371	303244141	Drip_loss	region1inRegion2	143391
CNVR16	290	1	296193901	296202305	loss-gain	8131	64660371	303244141	Drip_loss	region1inRegion2	8404
CNVR17	292	1	296918534	296982162	loss	8131	64660371	303244141	Drip_loss	region1inRegion2	63628
CNVR1	83	1	88942731	88998960	gain	8300	64660371	303244141	Drip_loss	region1inRegion2	56229
CNVR2	88	1	91934647	91942954	loss	8300	64660371	303244141	Drip_loss	region1inRegion2	8307
CNVR3	92	1	95316086	95335767	gain	8300	64660371	303244141	Drip_loss	region1inRegion2	19681
CNVR4	99	1	99687218	99704572	gain	8300	64660371	303244141	Drip_loss	region1inRegion2	17354
CNVR5	101	1	99733649	99785971	gain	8300	64660371	303244141	Drip_loss	region1inRegion2	52322
CNVR6	103	1	100019309	100027849	gain	8300	64660371	303244141	Drip_loss	region1inRegion2	8540
CNVR7	141	1	145236440	145265439	gain	8300	64660371	303244141	Drip_loss	region1inRegion2	28999
CNVR8	156	1	170118820	170121900	gain	8300	64660371	303244141	Drip_loss	region1inRegion2	3080
CNVR9	191	1	197269718	197274380	gain	8300	64660371	303244141	Drip_loss	region1inRegion2	4662
CNVR10	220	1	224697068	224765844	gain	8300	64660371	303244141	Drip_loss	region1inRegion2	68776
CNVR11	255	1	259697345	259705356	gain	8300	64660371	303244141	Drip_loss	region1inRegion2	8011
CNVR12	261	1	268054795	268056507	gain	8300	64660371	303244141	Drip_loss	region1inRegion2	1712
CNVR13	266	1	271529620	271536665	gain	8300	64660371	303244141	Drip_loss	region1inRegion2	7045
CNVR14	279	1	284447951	284512355	loss	8300	64660371	303244141	Drip_loss	region1inRegion2	64404
CNVR15	288	1	295235629	295379020	gain	8300	64660371	303244141	Drip_loss	region1inRegion2	143391
CNVR16	290	1	296193901	296202305	loss-gain	8300	64660371	303244141	Drip_loss	region1inRegion2	8404
CNVR17	292	1	296918534	296982162	loss	8300	64660371	303244141	Drip_loss	region1inRegion2	63628
CNVR1	83	1	88942731	88998960	gain	8323	64660371	303244141	Drip_loss	region1inRegion2	56229
CNVR2	88	1	91934647	91942954	loss	8323	64660371	303244141	Drip_loss	region1inRegion2	8307
CNVR3	92	1	95316086	95335767	gain	8323	64660371	303244141	Drip_loss	region1inRegion2	19681
CNVR4	99	1	99687218	99704572	gain	8323	64660371	303244141	Drip_loss	region1inRegion2	17354
CNVR5	101	1	99733649	99785971	gain	8323	64660371	303244141	Drip_loss	region1inRegion2	52322
CNVR6	103	1	100019309	100027849	gain	8323	64660371	303244141	Drip_loss	region1inRegion2	8540
CNVR7	141	1	145236440	145265439	gain	8323	64660371	303244141	Drip_loss	region1inRegion2	28999
CNVR8	156	1	170118820	170121900	gain	8323	64660371	303244141	Drip_loss	region1inRegion2	3080
CNVR9	191	1	197269718	197274380	gain	8323	64660371	303244141	Drip_loss	region1inRegion2	4662
CNVR10	220	1	224697068	224765844	gain	8323	64660371	303244141	Drip_loss	region1inRegion2	68776
CNVR11	255	1	259697345	259705356	gain	8323	64660371	303244141	Drip_loss	region1inRegion2	8011
CNVR12	261	1	268054795	268056507	gain	8323	64660371	303244141	Drip_loss	region1inRegion2	1712
CNVR13	266	1	271529620	271536665	gain	8323	64660371	303244141	Drip_loss	region1inRegion2	7045
CNVR14	279	1	284447951	284512355	loss	8323	64660371	303244141	Drip_loss	region1inRegion2	64404
CNVR15	288	1	295235629	295379020	gain	8323	64660371	303244141	Drip_loss	region1inRegion2	143391
CNVR16	290	1	296193901	296202305	loss-gain	8323	64660371	303244141	Drip_loss	region1inRegion2	8404
CNVR17	292	1	296918534	296982162	loss	8323	64660371	303244141	Drip_loss	region1inRegion2	63628
CNVR1	83	1	88942731	88998960	gain	8382	64660371	303244141	Drip_loss	region1inRegion2	56229
CNVR2	88	1	91934647	91942954	loss	8382	64660371	303244141	Drip_loss	region1inRegion2	8307
CNVR3	92	1	95316086	95335767	gain	8382	64660371	303244141	Drip_loss	region1inRegion2	19681
CNVR4	99	1	99687218	99704572	gain	8382	64660371	303244141	Drip_loss	region1inRegion2	17354
CNVR5	101	1	99733649	99785971	gain	8382	64660371	303244141	Drip_loss	region1inRegion2	52322
CNVR6	103	1	100019309	100027849	gain	8382	64660371	303244141	Drip_loss	region1inRegion2	8540
CNVR7	141	1	145236440	145265439	gain	8382	64660371	303244141	Drip_loss	region1inRegion2	28999
CNVR8	156	1	170118820	170121900	gain	8382	64660371	303244141	Drip_loss	region1inRegion2	3080
CNVR9	191	1	197269718	197274380	gain	8382	64660371	303244141	Drip_loss	region1inRegion2	4662
CNVR10	220	1	224697068	224765844	gain	8382	64660371	303244141	Drip_loss	region1inRegion2	68776
CNVR11	255	1	259697345	259705356	gain	8382	64660371	303244141	Drip_loss	region1inRegion2	8011
CNVR12	261	1	268054795	268056507	gain	8382	64660371	303244141	Drip_loss	region1inRegion2	1712
CNVR13	266	1	271529620	271536665	gain	8382	64660371	303244141	Drip_loss	region1inRegion2	7045
CNVR14	279	1	284447951	284512355	loss	8382	64660371	303244141	Drip_loss	region1inRegion2	64404
CNVR15	288	1	295235629	295379020	gain	8382	64660371	303244141	Drip_loss	region1inRegion2	143391
CNVR16	290	1	296193901	296202305	loss-gain	8382	64660371	303244141	Drip_loss	region1inRegion2	8404
CNVR17	292	1	296918534	296982162	loss	8382	64660371	303244141	Drip_loss	region1inRegion2	63628
CNVR1	83	1	88942731	88998960	gain	8480	64660371	303244141	Drip_loss	region1inRegion2	56229
CNVR2	88	1	91934647	91942954	loss	8480	64660371	303244141	Drip_loss	region1inRegion2	8307
CNVR3	92	1	95316086	95335767	gain	8480	64660371	303244141	Drip_loss	region1inRegion2	19681
CNVR4	99	1	99687218	99704572	gain	8480	64660371	303244141	Drip_loss	region1inRegion2	17354
CNVR5	101	1	99733649	99785971	gain	8480	64660371	303244141	Drip_loss	region1inRegion2	52322
CNVR6	103	1	100019309	100027849	gain	8480	64660371	303244141	Drip_loss	region1inRegion2	8540
CNVR7	141	1	145236440	145265439	gain	8480	64660371	303244141	Drip_loss	region1inRegion2	28999
CNVR8	156	1	170118820	170121900	gain	8480	64660371	303244141	Drip_loss	region1inRegion2	3080
CNVR9	191	1	197269718	197274380	gain	8480	64660371	303244141	Drip_loss	region1inRegion2	4662

CNVR10	220	1	224697068	224765844	gain	8480	64660371	303244141	Drip_loss	region1inRegion2	68776
CNVR11	255	1	259697345	259705356	gain	8480	64660371	303244141	Drip_loss	region1inRegion2	8011
CNVR12	261	1	268054795	268056507	gain	8480	64660371	303244141	Drip_loss	region1inRegion2	1712
CNVR13	266	1	271529620	271536665	gain	8480	64660371	303244141	Drip_loss	region1inRegion2	7045
CNVR14	279	1	284447951	284512355	loss	8480	64660371	303244141	Drip_loss	region1inRegion2	64404
CNVR15	288	1	295235629	295379020	gain	8480	64660371	303244141	Drip_loss	region1inRegion2	143391
CNVR16	290	1	296193901	296202305	loss-gain	8480	64660371	303244141	Drip_loss	region1inRegion2	8404
CNVR17	292	1	296918534	296982162	loss	8480	64660371	303244141	Drip_loss	region1inRegion2	63628
CNVR1	83	1	88942731	88998960	gain	8564	64660371	303244141	Drip_loss	region1inRegion2	56229
CNVR2	88	1	91934647	91942954	loss	8564	64660371	303244141	Drip_loss	region1inRegion2	8307
CNVR3	92	1	95316086	95335767	gain	8564	64660371	303244141	Drip_loss	region1inRegion2	19681
CNVR4	99	1	99687218	99704572	gain	8564	64660371	303244141	Drip_loss	region1inRegion2	17354
CNVR5	101	1	99733649	99785971	gain	8564	64660371	303244141	Drip_loss	region1inRegion2	52322
CNVR6	103	1	100019309	100027849	gain	8564	64660371	303244141	Drip_loss	region1inRegion2	8540
CNVR7	141	1	145236440	145265439	gain	8564	64660371	303244141	Drip_loss	region1inRegion2	28999
CNVR8	156	1	170118820	170121900	gain	8564	64660371	303244141	Drip_loss	region1inRegion2	3080
CNVR9	191	1	197269718	197274380	gain	8564	64660371	303244141	Drip_loss	region1inRegion2	4662
CNVR10	220	1	224697068	224765844	gain	8564	64660371	303244141	Drip_loss	region1inRegion2	68776
CNVR11	255	1	259697345	259705356	gain	8564	64660371	303244141	Drip_loss	region1inRegion2	8011
CNVR12	261	1	268054795	268056507	gain	8564	64660371	303244141	Drip_loss	region1inRegion2	1712
CNVR13	266	1	271529620	271536665	gain	8564	64660371	303244141	Drip_loss	region1inRegion2	7045
CNVR14	279	1	284447951	284512355	loss	8564	64660371	303244141	Drip_loss	region1inRegion2	64404
CNVR15	288	1	295235629	295379020	gain	8564	64660371	303244141	Drip_loss	region1inRegion2	143391
CNVR16	290	1	296193901	296202305	loss-gain	8564	64660371	303244141	Drip_loss	region1inRegion2	8404
CNVR17	292	1	296918534	296982162	loss	8564	64660371	303244141	Drip_loss	region1inRegion2	63628
CNVR1	83	1	88942731	88998960	gain	8602	64660371	303244141	Drip_loss	region1inRegion2	56229
CNVR2	88	1	91934647	91942954	loss	8602	64660371	303244141	Drip_loss	region1inRegion2	8307
CNVR3	92	1	95316086	95335767	gain	8602	64660371	303244141	Drip_loss	region1inRegion2	19681
CNVR4	99	1	99687218	99704572	gain	8602	64660371	303244141	Drip_loss	region1inRegion2	17354
CNVR5	101	1	99733649	99785971	gain	8602	64660371	303244141	Drip_loss	region1inRegion2	52322
CNVR6	103	1	100019309	100027849	gain	8602	64660371	303244141	Drip_loss	region1inRegion2	8540
CNVR7	141	1	145236440	145265439	gain	8602	64660371	303244141	Drip_loss	region1inRegion2	28999
CNVR8	156	1	170118820	170121900	gain	8602	64660371	303244141	Drip_loss	region1inRegion2	3080
CNVR9	191	1	197269718	197274380	gain	8602	64660371	303244141	Drip_loss	region1inRegion2	4662
CNVR10	220	1	224697068	224765844	gain	8602	64660371	303244141	Drip_loss	region1inRegion2	68776
CNVR11	255	1	259697345	259705356	gain	8602	64660371	303244141	Drip_loss	region1inRegion2	8011
CNVR12	261	1	268054795	268056507	gain	8602	64660371	303244141	Drip_loss	region1inRegion2	1712
CNVR13	266	1	271529620	271536665	gain	8602	64660371	303244141	Drip_loss	region1inRegion2	7045
CNVR14	279	1	284447951	284512355	loss	8602	64660371	303244141	Drip_loss	region1inRegion2	64404
CNVR15	288	1	295235629	295379020	gain	8602	64660371	303244141	Drip_loss	region1inRegion2	143391
CNVR16	290	1	296193901	296202305	loss-gain	8602	64660371	303244141	Drip_loss	region1inRegion2	8404
CNVR17	292	1	296918534	296982162	loss	8602	64660371	303244141	Drip_loss	region1inRegion2	63628
CNVR1	83	1	88942731	88998960	gain	5206	70361295	292707685	backfat_at_mid-back	region1inRegion2	56229
CNVR2	88	1	91934647	91942954	loss	5206	70361295	292707685	backfat_at_mid-back	region1inRegion2	8307
CNVR3	92	1	95316086	95335767	gain	5206	70361295	292707685	backfat_at_mid-back	region1inRegion2	19681
CNVR4	99	1	99687218	99704572	gain	5206	70361295	292707685	backfat_at_mid-back	region1inRegion2	17354
CNVR5	101	1	99733649	99785971	gain	5206	70361295	292707685	backfat_at_mid-back	region1inRegion2	52322
CNVR6	103	1	100019309	100027849	gain	5206	70361295	292707685	backfat_at_mid-back	region1inRegion2	8540
CNVR7	141	1	145236440	145265439	gain	5206	70361295	292707685	backfat_at_mid-back	region1inRegion2	28999
CNVR8	156	1	170118820	170121900	gain	5206	70361295	292707685	backfat_at_mid-back	region1inRegion2	3080
CNVR9	191	1	197269718	197274380	gain	5206	70361295	292707685	backfat_at_mid-back	region1inRegion2	4662
CNVR10	220	1	224697068	224765844	gain	5206	70361295	292707685	backfat_at_mid-back	region1inRegion2	68776
CNVR11	255	1	259697345	259705356	gain	5206	70361295	292707685	backfat_at_mid-back	region1inRegion2	8011
CNVR12	261	1	268054795	268056507	gain	5206	70361295	292707685	backfat_at_mid-back	region1inRegion2	1712
CNVR13	266	1	271529620	271536665	gain	5206	70361295	292707685	backfat_at_mid-back	region1inRegion2	7045
CNVR14	279	1	284447951	284512355	loss	5206	70361295	292707685	backfat_at_mid-back	region1inRegion2	64404
CNVR1	83	1	88942731	88998960	gain	126114	72534652	95300134	Top_line_conformation	region1inRegion2	56229
CNVR2	88	1	91934647	91942954	loss	126114	72534652	95300134	Top_line_conformation	region1inRegion2	8307
CNVR1	83	1	88942731	88998960	gain	6481	85842604	162073157	Teat_number	region1inRegion2	56229
CNVR2	88	1	91934647	91942954	loss	6481	85842604	162073157	Teat_number	region1inRegion2	8307
CNVR3	92	1	95316086	95335767	gain	6481	85842604	162073157	Teat_number	region1inRegion2	19681
CNVR4	99	1	99687218	99704572	gain	6481	85842604	162073157	Teat_number	region1inRegion2	17354
CNVR5	101	1	99733649	99785971	gain	6481	85842604	162073157	Teat_number	region1inRegion2	52322
CNVR6	103	1	100019309	100027849	gain	6481	85842604	162073157	Teat_number	region1inRegion2	8540
CNVR7	141	1	145236440	145265439	gain	6481	85842604	162073157	Teat_number	region1inRegion2	28999
CNVR1	83	1	88942731	88998960	gain	8853	85842604	162073157	Ear_weight	region1inRegion2	56229
CNVR2	88	1	91934647	91942954	loss	8853	85842604	162073157	Ear_weight	region1inRegion2	8307
CNVR3	92	1	95316086	95335767	gain	8853	85842604	162073157	Ear_weight	region1inRegion2	19681
CNVR4	99	1	99687218	99704572	gain	8853	85842604	162073157	Ear_weight	region1inRegion2	17354
CNVR5	101	1	99733649	99785971	gain	8853	85842604	162073157	Ear_weight	region1inRegion2	52322
CNVR6	103	1	100019309	100027849	gain	8853	85842604	162073157	Ear_weight	region1inRegion2	8540
CNVR7	141	1	145236440	145265439	gain	8853	85842604	162073157	Ear_weight	region1inRegion2	28999
CNVR1	83	1	88942731	88998960	gain	8854	85842604	162073157	Ear_weight	region1inRegion2	56229
CNVR2	88	1	91934647	91942954	loss	8854	85842604	162073157	Ear_weight	region1inRegion2	8307
CNVR3	92	1	95316086	95335767	gain	8854	85842604	162073157	Ear_weight	region1inRegion2	19681
CNVR4	99	1	99687218	99704572	gain	8854	85842604	162073157	Ear_weight	region1inRegion2	17354
CNVR5	101	1	99733649	99785971	gain	8854	85842604	162073157	Ear_weight	region1inRegion2	52322
CNVR6	103	1	100019309	100027849	gain	8854	85842604	162073157	Ear_weight	region1inRegion2	8540
CNVR7	141	1	145236440	145265439	gain	8854	85842604	162073157	Ear_weight	region1inRegion2	28999
CNVR1	83	1	88942731	88998960	gain	8885	85842604	162073157	Ear_area	region1inRegion2	56229
CNVR2	88	1	91934647	91942954	loss	8885	85842604	162073157	Ear_area	region1inRegion2	8307
CNVR3	92	1	95316086	95335767	gain	8885	85842604	162073157	Ear_area	region1inRegion2	19681
CNVR4	99	1	99687218	99704572	gain	8885	85842604	162073157	Ear_area	region1inRegion2	17354
CNVR5	101	1	99733649	99785971	gain	8885	85842604	162073157	Ear_area	region1inRegion2	52322
CNVR6	103	1	100019309	100027849	gain	8885	85842604	162073157	Ear_area	region1inRegion2	8540
CNVR7	141	1	145236440	145265439	gain	8885	85842604	162073157	Ear_area	region1inRegion2	28999
CNVR1	83	1	88942731	88998960	gain	8886	85842604	162073157	Ear_area	region1inRegion2	56229
CNVR2	88	1	91934647	91942954	loss	8886	85842604	162073157	Ear_area	region1inRegion2	8307
CNVR3	92	1	95316086	95335767	gain	8886	85842604	162073157	Ear_area	region1inRegion2	19681
CNVR4	99	1	99687218	99704572	gain	8886	85842604	162073157	Ear_area	region1inRegion2	17354
CNVR5	101	1	99733649	99785971	gain	8886	85842604	162073157	Ear_area	region1inRegion2	52322
CNVR6	103	1	100019309	100027849	gain	8886	85842604	162073157	Ear_area	region1inRegion2	8540
CNVR7	141	1	145236440	145265439	gain	8886	85842604	162073157	Ear_area	region1inRegion2	28999
CNVR1	83	1	88942731	88998960	gain	651	85842604	257944209	Backfat_at_rump	region1inRegion2	56229
CNVR2	88	1	91934647	91942954	loss	651	85842604	257944209	Backfat_at_rump	region1inRegion2	8307
CNVR3	92	1	95316086	95335767	gain	651	85842604	257944209	Backfat_at_rump	region1inRegion2	19681
CNVR4	99	1	99687218	99704572	gain	651	85842604	257944209	Backfat_at_rump	region1inRegion2	17354
CNVR5	101	1	99733649	99785971	gain	651	85842604	257944209	Backfat_at_rump	region1inRegion2	52322
CNVR6	103	1	100019309	100027849	gain	651	85842604	257944209	Backfat_at_rump	region1inRegion2	8540
CNVR7	141	1	145236440	145265439	gain	651	85842604	257944209	Backfat_at_rump	region1inRegion2	28999
CNVR8	156	1	170118820	170121900	gain	651	85842604	257944209	Backfat_at_rump	region1inRegion2	3080
CNVR9	191	1	197269718	197274380	gain	651	85842604	257944209	Backfat_at_rump	region1inRegion2	4662
CNVR10	220	1	224697068	224765844	gain	651	85842604	257944209	Backfat_at_rump	region1inRegion2	68776
CNVR2	88	1	91934647	91942954	loss	18365	91662984	96901518	uterine_horn_length	region1inRegion2	8307
CNVR3	92	1	95316086	95335767	gain	18365	91662984	96901518	uterine_horn_length	region1inRegion2	19681
CNVR3	92	1	95316086	95335767	gain	32101	94225166	102147789	Linoleic_acid_content	region1inRegion2	19681
CNVR4	99	1	99687218	99704572	gain	32101	94225166	102147789	Linoleic_acid_content	region1inRegion2	17354
CNVR5	101	1	99733649	99785971	gain	32101	94225166	102147789	Linoleic_acid_content	region1inRegion2	52322
CNVR6	103	1	100019309	100027849	gain	32101	94225166	102147789	Linoleic_acid_content	region1inRegion2	8540
CNVR4	99	1	99687218	99704572	gain	4326	96293184	142302138	CIE-L*	region1inRegion2	17354
CNVR5	101	1	99733649	99785971	gain	4326	96293184	142302138	CIE-L*	region1inRegion2	52322
CNVR6	103	1	100019309	100027849	gain	4326	96293184	142302138	CIE-L*	region1inRegion2	8540
CNVR4	99	1	99687218	99704572	gain	2845	96293184	247820666	Belly_weight	region1inRegion2	17354
CNVR5	101	1	99733649	99785971	gain	2845	96293184	247820666	Belly_weight	region1inRegion2	52322
CNVR6	103	1	100019309	100027849	gain	2845	96293184	247820666	Belly_weight	region1inRegion2	8540
CNVR7	141	1	145236440	145265439	gain	2845	96293184	247820666	Belly_weight	region1inRegion2	28999
CNVR8	156	1	170118820	170121900	gain	2845	96293184	247820666	Belly_weight	region1inRegion2	3080
CNVR9	191	1	197269718	197274380	gain	2845	96293184	247820666	Belly_weight	region1inRegion2	4662
CNVR10	220	1	224697068	224765844	gain	2845	96293184	247820666	Belly_weight	region1inRegion2	68776
CNVR7	141	1	145236440	145265439	gain	17711	105086765	154329026	Cholesterol_level	region1inRegion2	28999
CNVR7	141	1	145236440	145265439	gain	17712	105086765	154329026	HDL/LDL_ratio	region1inRegion2	28999
CNVR7	141	1	145236440	145265439	gain	17713	105086765	154329026	LDL_cholesterol	region1inRegion2	28999

CNVR7	141	1	145236440	145265439	gain	5928	105086765	154329026	Average_daily_gain	region1inRegion2	28999
CNVR7	141	1	145236440	145265439	gain	5931	105086765	154329026	Body_weight_(weaning)	region1inRegion2	28999
CNVR7	141	1	145236440	145265439	gain	2930	105086765	169149638	Marbling	region1inRegion2	28999
CNVR7	141	1	145236440	145265439	gain	5664	105086765	210644363	PH_for_Longissmus_dorsi	region1inRegion2	28999
CNVR8	156	1	170118820	170121900	gain	5664	105086765	210644363	PH_for_Longissmus_dorsi	region1inRegion2	3080
CNVR9	191	1	197269718	197274380	gain	5664	105086765	210644363	PH_for_Longissmus_dorsi	region1inRegion2	4662
CNVR7	141	1	145236440	145265439	gain	5665	105086765	210644363	pH_for_Semimembranosus	region1inRegion2	28999
CNVR8	156	1	170118820	170121900	gain	5665	105086765	210644363	pH_for_Semimembranosus	region1inRegion2	3080
CNVR9	191	1	197269718	197274380	gain	5665	105086765	210644363	pH_for_Semimembranosus	region1inRegion2	4662
CNVR7	141	1	145236440	145265439	gain	5666	105086765	210644363	PH_for_Longissmus_dorsi	region1inRegion2	28999
CNVR8	156	1	170118820	170121900	gain	5666	105086765	210644363	PH_for_Longissmus_dorsi	region1inRegion2	3080
CNVR9	191	1	197269718	197274380	gain	5666	105086765	210644363	PH_for_Longissmus_dorsi	region1inRegion2	4662
CNVR7	141	1	145236440	145265439	gain	5668	105086765	210644363	Conductivity_24_hours_postmortem_(loin)	region1inRegion2	28999
CNVR8	156	1	170118820	170121900	gain	5668	105086765	210644363	Conductivity_24_hours_postmortem_(loin)	region1inRegion2	3080
CNVR9	191	1	197269718	197274380	gain	5668	105086765	210644363	Conductivity_24_hours_postmortem_(loin)	region1inRegion2	4662
CNVR7	141	1	145236440	145265439	gain	3795	105086765	228164160	Backfat_at_tenth_rib	region1inRegion2	28999
CNVR8	156	1	170118820	170121900	gain	3795	105086765	228164160	Backfat_at_tenth_rib	region1inRegion2	3080
CNVR9	191	1	197269718	197274380	gain	3795	105086765	228164160	Backfat_at_tenth_rib	region1inRegion2	4662
CNVR10	220	1	224697068	224765844	gain	3795	105086765	228164160	Backfat_at_tenth_rib	region1inRegion2	68776
CNVR7	141	1	145236440	145265439	gain	3796	105086765	228164160	Loin_muscle_area	region1inRegion2	28999
CNVR8	156	1	170118820	170121900	gain	3796	105086765	228164160	Loin_muscle_area	region1inRegion2	3080
CNVR9	191	1	197269718	197274380	gain	3796	105086765	228164160	Loin_muscle_area	region1inRegion2	4662
CNVR10	220	1	224697068	224765844	gain	3796	105086765	228164160	Loin_muscle_area	region1inRegion2	68776
CNVR7	141	1	145236440	145265439	gain	5667	105086765	252283830	Meat_color_OPTO	region1inRegion2	28999
CNVR8	156	1	170118820	170121900	gain	5667	105086765	252283830	Meat_color_OPTO	region1inRegion2	3080
CNVR9	191	1	197269718	197274380	gain	5667	105086765	252283830	Meat_color_OPTO	region1inRegion2	4662
CNVR10	220	1	224697068	224765844	gain	5667	105086765	252283830	Meat_color_OPTO	region1inRegion2	68776
CNVR7	141	1	145236440	145265439	gain	3793	105086765	274130998	Average_backfat_thickness	region1inRegion2	28999
CNVR8	156	1	170118820	170121900	gain	3793	105086765	274130998	Average_backfat_thickness	region1inRegion2	3080
CNVR9	191	1	197269718	197274380	gain	3793	105086765	274130998	Average_backfat_thickness	region1inRegion2	4662
CNVR10	220	1	224697068	224765844	gain	3793	105086765	274130998	Average_backfat_thickness	region1inRegion2	68776
CNVR11	255	1	259697345	259705356	gain	3793	105086765	274130998	Average_backfat_thickness	region1inRegion2	8011
CNVR12	261	1	268054795	268056507	gain	3793	105086765	274130998	Average_backfat_thickness	region1inRegion2	1712
CNVR13	266	1	271529620	271536665	gain	3793	105086765	274130998	Average_backfat_thickness	region1inRegion2	7045
CNVR7	141	1	145236440	145265439	gain	3794	105086765	274130998	backfat_at_last_rib	region1inRegion2	28999
CNVR8	156	1	170118820	170121900	gain	3794	105086765	274130998	backfat_at_last_rib	region1inRegion2	3080
CNVR9	191	1	197269718	197274380	gain	3794	105086765	274130998	backfat_at_last_rib	region1inRegion2	4662
CNVR10	220	1	224697068	224765844	gain	3794	105086765	274130998	backfat_at_last_rib	region1inRegion2	68776
CNVR11	255	1	259697345	259705356	gain	3794	105086765	274130998	backfat_at_last_rib	region1inRegion2	8011
CNVR12	261	1	268054795	268056507	gain	3794	105086765	274130998	backfat_at_last_rib	region1inRegion2	1712
CNVR13	266	1	271529620	271536665	gain	3794	105086765	274130998	backfat_at_last_rib	region1inRegion2	7045
CNVR7	141	1	145236440	145265439	gain	5669	105086765	274130998	Conductivity_24_hours_postmortem_(ham)	region1inRegion2	28999
CNVR8	156	1	170118820	170121900	gain	5669	105086765	274130998	Conductivity_24_hours_postmortem_(ham)	region1inRegion2	3080
CNVR9	191	1	197269718	197274380	gain	5669	105086765	274130998	Conductivity_24_hours_postmortem_(ham)	region1inRegion2	4662
CNVR10	220	1	224697068	224765844	gain	5669	105086765	274130998	Conductivity_24_hours_postmortem_(ham)	region1inRegion2	68776
CNVR11	255	1	259697345	259705356	gain	5669	105086765	274130998	Conductivity_24_hours_postmortem_(ham)	region1inRegion2	8011
CNVR12	261	1	268054795	268056507	gain	5669	105086765	274130998	Conductivity_24_hours_postmortem_(ham)	region1inRegion2	1712
CNVR13	266	1	271529620	271536665	gain	5669	105086765	274130998	Conductivity_24_hours_postmortem_(ham)	region1inRegion2	7045
CNVR7	141	1	145236440	145265439	gain	5255	105873529	290457491	Teat_number	region1inRegion2	28999
CNVR8	156	1	170118820	170121900	gain	5255	105873529	290457491	Teat_number	region1inRegion2	3080
CNVR9	191	1	197269718	197274380	gain	5255	105873529	290457491	Teat_number	region1inRegion2	4662
CNVR10	220	1	224697068	224765844	gain	5255	105873529	290457491	Teat_number	region1inRegion2	68776
CNVR11	255	1	259697345	259705356	gain	5255	105873529	290457491	Teat_number	region1inRegion2	8011
CNVR12	261	1	268054795	268056507	gain	5255	105873529	290457491	Teat_number	region1inRegion2	1712
CNVR13	266	1	271529620	271536665	gain	5255	105873529	290457491	Teat_number	region1inRegion2	7045
CNVR14	279	1	284447951	284512355	loss	5255	105873529	290457491	Teat_number	region1inRegion2	64404
CNVR7	141	1	145236440	145265439	gain	5640	106747450	233806417	Ear_size	region1inRegion2	28999
CNVR8	156	1	170118820	170121900	gain	5640	106747450	233806417	Ear_size	region1inRegion2	3080
CNVR9	191	1	197269718	197274380	gain	5640	106747450	233806417	Ear_size	region1inRegion2	4662
CNVR10	220	1	224697068	224765844	gain	5640	106747450	233806417	Ear_size	region1inRegion2	68776
CNVR7	141	1	145236440	145265439	gain	5646	106747450	233806417	Ear_erectness	region1inRegion2	28999
CNVR8	156	1	170118820	170121900	gain	5646	106747450	233806417	Ear_erectness	region1inRegion2	3080
CNVR9	191	1	197269718	197274380	gain	5646	106747450	233806417	Ear_erectness	region1inRegion2	4662
CNVR10	220	1	224697068	224765844	gain	5646	106747450	233806417	Ear_erectness	region1inRegion2	68776
CNVR7	141	1	145236440	145265439	gain	5651	106747450	233806417	Ear_erectness	region1inRegion2	28999
CNVR8	156	1	170118820	170121900	gain	5651	106747450	233806417	Ear_erectness	region1inRegion2	3080
CNVR9	191	1	197269718	197274380	gain	5651	106747450	233806417	Ear_erectness	region1inRegion2	4662
CNVR10	220	1	224697068	224765844	gain	5651	106747450	233806417	Ear_erectness	region1inRegion2	68776
CNVR7	141	1	145236440	145265439	gain	3171	108714563	220801047	Backfat_weight	region1inRegion2	28999
CNVR8	156	1	170118820	170121900	gain	3171	108714563	220801047	Backfat_weight	region1inRegion2	3080
CNVR9	191	1	197269718	197274380	gain	3171	108714563	220801047	Backfat_weight	region1inRegion2	4662
CNVR7	141	1	145236440	145265439	gain	5230	108714563	293277782	Body_weight_(weaning)	region1inRegion2	28999
CNVR8	156	1	170118820	170121900	gain	5230	108714563	293277782	Body_weight_(weaning)	region1inRegion2	3080
CNVR9	191	1	197269718	197274380	gain	5230	108714563	293277782	Body_weight_(weaning)	region1inRegion2	4662
CNVR10	220	1	224697068	224765844	gain	5230	108714563	293277782	Body_weight_(weaning)	region1inRegion2	68776
CNVR11	255	1	259697345	259705356	gain	5230	108714563	293277782	Body_weight_(weaning)	region1inRegion2	8011
CNVR12	261	1	268054795	268056507	gain	5230	108714563	293277782	Body_weight_(weaning)	region1inRegion2	1712
CNVR13	266	1	271529620	271536665	gain	5230	108714563	293277782	Body_weight_(weaning)	region1inRegion2	7045
CNVR14	279	1	284447951	284512355	loss	5230	108714563	293277782	Body_weight_(weaning)	region1inRegion2	64404
CNVR7	141	1	145236440	145265439	gain	7215	125024752	151349204	Hemoglobin	region1inRegion2	28999
CNVR7	141	1	145236440	145265439	gain	78	133275468	154329026	Marbling	region1inRegion2	28999
CNVR7	141	1	145236440	145265439	gain	45	133275468	220801047	Backfat_at_last_lumbar	region1inRegion2	28999
CNVR8	156	1	170118820	170121900	gain	45	133275468	220801047	Backfat_at_last_lumbar	region1inRegion2	3080
CNVR9	191	1	197269718	197274380	gain	45	133275468	220801047	Backfat_at_last_lumbar	region1inRegion2	4662
CNVR7	141	1	145236440	145265439	gain	12312	133275468	242497921	Platelet_distribution_width	region1inRegion2	28999
CNVR8	156	1	170118820	170121900	gain	12312	133275468	242497921	Platelet_distribution_width	region1inRegion2	3080
CNVR9	191	1	197269718	197274380	gain	12312	133275468	242497921	Platelet_distribution_width	region1inRegion2	4662
CNVR10	220	1	224697068	224765844	gain	12312	133275468	242497921	Platelet_distribution_width	region1inRegion2	68776
CNVR7	141	1	145236440	145265439	gain	4020	140728674	229018949	Marbling	region1inRegion2	28999
CNVR8	156	1	170118820	170121900	gain	4020	140728674	229018949	Marbling	region1inRegion2	3080
CNVR9	191	1	197269718	197274380	gain	4020	140728674	229018949	Marbling	region1inRegion2	4662
CNVR10	220	1	224697068	224765844	gain	4020	140728674	229018949	Marbling	region1inRegion2	68776
CNVR7	141	1	145236440	145265439	gain	13266	140742758	168586806	Liver_weight	region1inRegion2	28999
CNVR7	141	1	145236440	145265439	gain	17789	143819347	175004689	Average_glycolytic_potential	region1inRegion2	28999
CNVR8	156	1	170118820	170121900	gain	17789	143819347	175004689	Average_glycolytic_potential	region1inRegion2	3080
CNVR8	156	1	170118820	170121900	gain	4017	150567892	184999941	Muscle_fat_content	region1inRegion2	3080
CNVR8	156	1	170118820	170121900	gain	797	154143188	180898441	Average_backfat_thickness	region1inRegion2	3080
CNVR8	156	1	170118820	170121900	gain	5958	154329026	210644363	Side_fat_thickness	region1inRegion2	3080
CNVR9	191	1	197269718	197274380	gain	5958	154329026	210644363	Side_fat_thickness	region1inRegion2	4662
CNVR8	156	1	170118820	170121900	gain	8742	160345807	304195794	Umbilical_hernia	region1inRegion2	3080
CNVR9	191	1	197269718	197274380	gain	8742	160345807	304195794	Umbilical_hernia	region1inRegion2	4662
CNVR10	220	1	224697068	224765844	gain	8742	160345807	304195794	Umbilical_hernia	region1inRegion2	68776
CNVR11	255	1	259697345	259705356	gain	8742	160345807	304195794	Umbilical_hernia	region1inRegion2	8011
CNVR12	261	1	268054795	268056507	gain	8742	160345807	304195794	Umbilical_hernia	region1inRegion2	1712
CNVR13	266	1	271529620	271536665	gain	8742	160345807	304195794	Umbilical_hernia	region1inRegion2	7045
CNVR14	279	1	284447951	284512355	loss	8742	160345807	304195794	Umbilical_hernia	region1inRegion2	64404
CNVR15	288	1	295235629	295379020	gain	8742	160345807	304195794	Umbilical_hernia	region1inRegion2	143391
CNVR16	290	1	296193901	296202305	loss-gain	8742	160345807	304195794	Umbilical_hernia	region1inRegion2	8404
CNVR17	292	1	296918534	296982162	loss	8742	160345807	304195794	Umbilical_hernia	region1inRegion2	63628
CNVR8	156	1	170118820	170121900	gain	11557	162073157	233806417	Salmonella_count_in_spleen	region1inRegion2	3080
CNVR9	191	1	197269718	197274380	gain	11557	162073157	233806417	Salmonella_count_in_spleen	region1inRegion2	4662
CNVR10	220	1	224697068	224765844	gain	11557	162073157	233806417	Salmonella_count_in_spleen	region1inRegion2	68776
CNVR8	156	1	170118820	170121900	gain	138	162073157	233806417	Backfat_at_tenth_rib	region1inRegion2	3080
CNVR9	191	1	197269718	197274380	gain	138	162073157	233806417	Backfat_at_tenth_rib	region1inRegion2	4662
CNVR10	220	1	224697068	224765844	gain	138	162073157	233806417	Backfat_at_tenth_rib	region1inRegion2	68776
CNVR8	156	1	170118820	170121900	gain	139	162073157	233806417	Average_daily_gain	region1inRegion2	3080
CNVR9	191	1	197269718	197274380	gain	139	162073157	233806417	Average_daily_gain	region1inRegion2	4662
CNVR10	220	1	224697068	224765844	gain	139	162073157	233806417	Average_daily_gain	region1inRegion2	68776
CNVR8	156	1	170118820	170121900	gain	140	162073157	233806417	Average_daily_gain	region1inRegion2	3080
CNVR9	191	1	197269718	197274380	gain	140	162073157	233806417	Average_daily_gain	region1inRegion2	4662
CNVR10	220	1	224697068	224765844	gain	140	162073157	233806417	Average_daily_gain	region1inRegion2	68776
CNVR8	156	1	170118820	170121900	gain	141	162073157	233806417	Daily_feed_intake	region1inRegion2	3080

CNVR9	191	1	197269718	197274380	gain	141	162073157	233806417	Daily_feed_intake	region1inRegion2	4662
CNVR10	220	1	224697068	224765844	gain	141	162073157	233806417	Daily_feed_intake	region1inRegion2	68776
CNVR8	156	1	170118820	170121900	gain	8898	162073157	257944209	Ear_erectness	region1inRegion2	3080
CNVR9	191	1	197269718	197274380	gain	8898	162073157	257944209	Ear_erectness	region1inRegion2	4662
CNVR10	220	1	224697068	224765844	gain	8898	162073157	257944209	Ear_erectness	region1inRegion2	68776
CNVR8	156	1	170118820	170121900	gain	432	163919363	234502051	Head_weight	region1inRegion2	3080
CNVR9	191	1	197269718	197274380	gain	432	163919363	234502051	Head_weight	region1inRegion2	4662
CNVR10	220	1	224697068	224765844	gain	432	163919363	234502051	Head_weight	region1inRegion2	68776
CNVR8	156	1	170118820	170121900	gain	13264	165442839	249115896	Liver_weight	region1inRegion2	3080
CNVR9	191	1	197269718	197274380	gain	13264	165442839	249115896	Liver_weight	region1inRegion2	4662
CNVR10	220	1	224697068	224765844	gain	13264	165442839	249115896	Liver_weight	region1inRegion2	68776
CNVR8	156	1	170118820	170121900	gain	7453	165580236	257944209	Nonfunctional_nipples	region1inRegion2	3080
CNVR9	191	1	197269718	197274380	gain	7453	165580236	257944209	Nonfunctional_nipples	region1inRegion2	4662
CNVR10	220	1	224697068	224765844	gain	7453	165580236	257944209	Nonfunctional_nipples	region1inRegion2	68776
CNVR8	156	1	170118820	170121900	gain	3124	167565961	173258818	Total_number_born_alive	region1inRegion2	3080
CNVR8	156	1	170118820	170121900	gain	1271	168669767	210644363	Ham_fat_weight	region1inRegion2	3080
CNVR9	191	1	197269718	197274380	gain	1271	168669767	210644363	Ham_fat_weight	region1inRegion2	4662
CNVR8	156	1	170118820	170121900	gain	18028	169149638	226764071	Adipocyte_diameter	region1inRegion2	3080
CNVR9	191	1	197269718	197274380	gain	18028	169149638	226764071	Adipocyte_diameter	region1inRegion2	4662
CNVR10	220	1	224697068	224765844	gain	18028	169149638	226764071	Adipocyte_diameter	region1inRegion2	68776
CNVR8	156	1	170118820	170121900	gain	866	169149638	226764071	Feed_intake	region1inRegion2	3080
CNVR9	191	1	197269718	197274380	gain	866	169149638	226764071	Feed_intake	region1inRegion2	4662
CNVR10	220	1	224697068	224765844	gain	866	169149638	226764071	Feed_intake	region1inRegion2	68776
CNVR8	156	1	170118820	170121900	gain	2742	169149638	228164160	Loin_muscle_area	region1inRegion2	3080
CNVR9	191	1	197269718	197274380	gain	2742	169149638	228164160	Loin_muscle_area	region1inRegion2	4662
CNVR10	220	1	224697068	224765844	gain	2742	169149638	228164160	Loin_muscle_area	region1inRegion2	68776
CNVR8	156	1	170118820	170121900	gain	3640	169149638	228164160	Loin_muscle_area	region1inRegion2	3080
CNVR9	191	1	197269718	197274380	gain	3640	169149638	228164160	Loin_muscle_area	region1inRegion2	4662
CNVR10	220	1	224697068	224765844	gain	3640	169149638	228164160	Loin_muscle_area	region1inRegion2	68776
CNVR8	156	1	170118820	170121900	gain	12709	169149638	233806417	Carcass_weight_(cold)	region1inRegion2	3080
CNVR9	191	1	197269718	197274380	gain	12709	169149638	233806417	Carcass_weight_(cold)	region1inRegion2	4662
CNVR10	220	1	224697068	224765844	gain	12709	169149638	233806417	Carcass_weight_(cold)	region1inRegion2	68776
CNVR8	156	1	170118820	170121900	gain	12714	169149638	233806417	Abdominal_fat_weight	region1inRegion2	3080
CNVR9	191	1	197269718	197274380	gain	12714	169149638	233806417	Abdominal_fat_weight	region1inRegion2	4662
CNVR10	220	1	224697068	224765844	gain	12714	169149638	233806417	Abdominal_fat_weight	region1inRegion2	68776
CNVR8	156	1	170118820	170121900	gain	12717	169149638	233806417	External_fat_on_ham	region1inRegion2	3080
CNVR9	191	1	197269718	197274380	gain	12717	169149638	233806417	External_fat_on_ham	region1inRegion2	4662
CNVR10	220	1	224697068	224765844	gain	12717	169149638	233806417	External_fat_on_ham	region1inRegion2	68776
CNVR8	156	1	170118820	170121900	gain	12722	169149638	233806417	Shoulder_external_fat_weight	region1inRegion2	3080
CNVR9	191	1	197269718	197274380	gain	12722	169149638	233806417	Shoulder_external_fat_weight	region1inRegion2	4662
CNVR10	220	1	224697068	224765844	gain	12722	169149638	233806417	Shoulder_external_fat_weight	region1inRegion2	68776
CNVR8	156	1	170118820	170121900	gain	12724	169149638	233806417	Backfat_weight	region1inRegion2	3080
CNVR9	191	1	197269718	197274380	gain	12724	169149638	233806417	Backfat_weight	region1inRegion2	4662
CNVR10	220	1	224697068	224765844	gain	12724	169149638	233806417	Backfat_weight	region1inRegion2	68776
CNVR8	156	1	170118820	170121900	gain	12731	169149638	233806417	backfat_above_muscle_dorsi	region1inRegion2	3080
CNVR9	191	1	197269718	197274380	gain	12731	169149638	233806417	backfat_above_muscle_dorsi	region1inRegion2	4662
CNVR10	220	1	224697068	224765844	gain	12731	169149638	233806417	backfat_above_muscle_dorsi	region1inRegion2	68776
CNVR8	156	1	170118820	170121900	gain	12733	169149638	233806417	Backfat_at_tenth_rib	region1inRegion2	3080
CNVR9	191	1	197269718	197274380	gain	12733	169149638	233806417	Backfat_at_tenth_rib	region1inRegion2	4662
CNVR10	220	1	224697068	224765844	gain	12733	169149638	233806417	Backfat_at_tenth_rib	region1inRegion2	68776
CNVR8	156	1	170118820	170121900	gain	12736	169149638	233806417	Subcutanous_fat_area	region1inRegion2	3080
CNVR9	191	1	197269718	197274380	gain	12736	169149638	233806417	Subcutanous_fat_area	region1inRegion2	4662
CNVR10	220	1	224697068	224765844	gain	12736	169149638	233806417	Subcutanous_fat_area	region1inRegion2	68776
CNVR8	156	1	170118820	170121900	gain	12742	169149638	233806417	Fat_to_meat_ratio	region1inRegion2	3080
CNVR9	191	1	197269718	197274380	gain	12742	169149638	233806417	Fat_to_meat_ratio	region1inRegion2	4662
CNVR10	220	1	224697068	224765844	gain	12742	169149638	233806417	Fat_to_meat_ratio	region1inRegion2	68776
CNVR8	156	1	170118820	170121900	gain	44	169149638	257763699	backfat_at_last_rib	region1inRegion2	3080
CNVR9	191	1	197269718	197274380	gain	44	169149638	257763699	backfat_at_last_rib	region1inRegion2	4662
CNVR10	220	1	224697068	224765844	gain	44	169149638	257763699	backfat_at_last_rib	region1inRegion2	68776
CNVR8	156	1	170118820	170121900	gain	16897	169149638	265323610	Carcass_weight_(hot)	region1inRegion2	3080
CNVR9	191	1	197269718	197274380	gain	16897	169149638	265323610	Carcass_weight_(hot)	region1inRegion2	4662
CNVR10	220	1	224697068	224765844	gain	16897	169149638	265323610	Carcass_weight_(hot)	region1inRegion2	68776
CNVR11	255	1	259697345	259705356	gain	16897	169149638	265323610	Carcass_weight_(hot)	region1inRegion2	8011
CNVR8	156	1	170118820	170121900	gain	17996	169149638	265323610	Average_backfat_thickness	region1inRegion2	3080
CNVR9	191	1	197269718	197274380	gain	17996	169149638	265323610	Average_backfat_thickness	region1inRegion2	4662
CNVR10	220	1	224697068	224765844	gain	17996	169149638	265323610	Average_backfat_thickness	region1inRegion2	68776
CNVR11	255	1	259697345	259705356	gain	17996	169149638	265323610	Average_backfat_thickness	region1inRegion2	8011
CNVR8	156	1	170118820	170121900	gain	3914	169149638	292955427	Average_daily_gain	region1inRegion2	3080
CNVR9	191	1	197269718	197274380	gain	3914	169149638	292955427	Average_daily_gain	region1inRegion2	4662
CNVR10	220	1	224697068	224765844	gain	3914	169149638	292955427	Average_daily_gain	region1inRegion2	68776
CNVR11	255	1	259697345	259705356	gain	3914	169149638	292955427	Average_daily_gain	region1inRegion2	8011
CNVR12	261	1	268054795	268056507	gain	3914	169149638	292955427	Average_daily_gain	region1inRegion2	1712
CNVR13	266	1	271529620	271536665	gain	3914	169149638	292955427	Average_daily_gain	region1inRegion2	7045
CNVR14	279	1	284447951	284512355	loss	3914	169149638	292955427	Average_daily_gain	region1inRegion2	64404
CNVR8	156	1	170118820	170121900	gain	3917	169149638	292955427	Average_daily_gain	region1inRegion2	3080
CNVR9	191	1	197269718	197274380	gain	3917	169149638	292955427	Average_daily_gain	region1inRegion2	4662
CNVR10	220	1	224697068	224765844	gain	3917	169149638	292955427	Average_daily_gain	region1inRegion2	68776
CNVR11	255	1	259697345	259705356	gain	3917	169149638	292955427	Average_daily_gain	region1inRegion2	8011
CNVR12	261	1	268054795	268056507	gain	3917	169149638	292955427	Average_daily_gain	region1inRegion2	1712
CNVR13	266	1	271529620	271536665	gain	3917	169149638	292955427	Average_daily_gain	region1inRegion2	7045
CNVR14	279	1	284447951	284512355	loss	3917	169149638	292955427	Average_daily_gain	region1inRegion2	64404
CNVR9	191	1	197269718	197274380	gain	1269	174394489	230570794	Average_backfat_thickness	region1inRegion2	4662
CNVR10	220	1	224697068	224765844	gain	1269	174394489	230570794	Average_backfat_thickness	region1inRegion2	68776
CNVR9	191	1	197269718	197274380	gain	4016	175004689	199579088	Average_backfat_thickness	region1inRegion2	4662
CNVR9	191	1	197269718	197274380	gain	12663	180791273	255350469	CIE-a*	region1inRegion2	4662
CNVR10	220	1	224697068	224765844	gain	12663	180791273	255350469	CIE-a*	region1inRegion2	68776
CNVR9	191	1	197269718	197274380	gain	4021	181255237	212599407	Percentage_type_I_fibers	region1inRegion2	4662
CNVR9	191	1	197269718	197274380	gain	126118	195003570	217149267	Hind_leg_conformation	region1inRegion2	4662
CNVR10	220	1	224697068	224765844	gain	520	199128720	272692549	Age_at_puberty	region1inRegion2	68776
CNVR11	255	1	259697345	259705356	gain	520	199128720	272692549	Age_at_puberty	region1inRegion2	8011
CNVR12	261	1	268054795	268056507	gain	520	199128720	272692549	Age_at_puberty	region1inRegion2	1712
CNVR13	266	1	271529620	271536665	gain	520	199128720	272692549	Age_at_puberty	region1inRegion2	7045
CNVR10	220	1	224697068	224765844	gain	798	204653903	279476353	Backfat_weight	region1inRegion2	68776
CNVR11	255	1	259697345	259705356	gain	798	204653903	279476353	Backfat_weight	region1inRegion2	8011
CNVR12	261	1	268054795	268056507	gain	798	204653903	279476353	Backfat_weight	region1inRegion2	1712
CNVR13	266	1	271529620	271536665	gain	798	204653903	279476353	Backfat_weight	region1inRegion2	7045
CNVR10	220	1	224697068	224765844	gain	686	210644363	228164160	Linolenic_acid_content	region1inRegion2	68776
CNVR10	220	1	224697068	224765844	gain	5673	210644363	252283830	backfat_above_muscle_dorsi	region1inRegion2	68776
CNVR10	220	1	224697068	224765844	gain	5681	210644363	292955427	Average_daily_gain	region1inRegion2	68776
CNVR11	255	1	259697345	259705356	gain	5681	210644363	292955427	Average_daily_gain	region1inRegion2	8011
CNVR12	261	1	268054795	268056507	gain	5681	210644363	292955427	Average_daily_gain	region1inRegion2	1712
CNVR13	266	1	271529620	271536665	gain	5681	210644363	292955427	Average_daily_gain	region1inRegion2	7045
CNVR14	279	1	284447951	284512355	loss	5681	210644363	292955427	Average_daily_gain	region1inRegion2	64404
CNVR10	220	1	224697068	224765844	gain	478	213932992	280776363	Locomotion	region1inRegion2	68776
CNVR11	255	1	259697345	259705356	gain	478	213932992	280776363	Locomotion	region1inRegion2	8011
CNVR12	261	1	268054795	268056507	gain	478	213932992	280776363	Locomotion	region1inRegion2	1712
CNVR13	266	1	271529620	271536665	gain	478	213932992	280776363	Locomotion	region1inRegion2	7045
CNVR10	220	1	224697068	224765844	gain	16832	214736360	226764071	backfat_at_last_rib	region1inRegion2	68776
CNVR10	220	1	224697068	224765844	gain	16880	214736360	226764071	backfat_at_last_rib	region1inRegion2	68776
CNVR10	220	1	224697068	224765844	gain	16882	214736360	226764071	backfat_at_last_rib	region1inRegion2	68776
CNVR10	220	1	224697068	224765844	gain	16902	214736360	226764071	Carcass_weight_(hot)	region1inRegion2	68776
CNVR10	220	1	224697068	224765844	gain	16833	216112266	294659146	backfat_at_last_rib	region1inRegion2	68776
CNVR11	255	1	259697345	259705356	gain	16833	216112266	294659146	backfat_at_last_rib	region1inRegion2	8011
CNVR12	261	1	268054795	268056507	gain	16833	216112266	294659146	backfat_at_last_rib	region1inRegion2	1712
CNVR13	266	1	271529620	271536665	gain	16833	216112266	294659146	backfat_at_last_rib	region1inRegion2	7045
CNVR14	279	1	284447951	284512355	loss	16833	216112266	294659146	backfat_at_last_rib	region1inRegion2	64404
CNVR10	220	1	224697068	224765844	gain	18019	216112266	294659146	Adipocyte_diameter	region1inRegion2	68776
CNVR11	255	1	259697345	259705356	gain	18019	216112266	294659146	Adipocyte_diameter	region1inRegion2	8011
CNVR12	261	1	268054795	268056507	gain	18019	216112266	294659146	Adipocyte_diameter	region1inRegion2	1712
CNVR13	266	1	271529620	271536665	gain	18019	216112266	294659146	Adipocyte_diameter	region1inRegion2	7045
CNVR14	279	1	284447951	284512355	loss	18019	216112266	294659146	Adipocyte_diameter	region1inRegion2	64404
CNVR10	220	1	224697068	224765844	gain	18002	218980897	265323610	Subcutanous_fat_area	region1inRegion2	68776

CNVR11	255	1	259697345	259705356	gain	18002	218980897	265323610	Subcutanous_fat_area	region1inRegion2	8011
CNVR10	220	1	224697068	224765844	gain	1277	220801047	228164160	Ratio_of_glycolytic_muscles_weight_to_ham_meat_and_bone_we	region1inRegion2	68776
CNVR10	220	1	224697068	224765844	gain	2844	220801047	247820666	Average_backfat_thickness	region1inRegion2	68776
CNVR10	220	1	224697068	224765844	gain	2848	220801047	247820666	Protein_accretion_rate	region1inRegion2	68776
CNVR10	220	1	224697068	224765844	gain	1264	220801047	266165650	Lean_meat_percentage	region1inRegion2	68776
CNVR11	255	1	259697345	259705356	gain	1264	220801047	266165650	Lean_meat_percentage	region1inRegion2	8011
CNVR10	220	1	224697068	224765844	gain	1266	220801047	266165650	Lean_meat_+_bone_in_back	region1inRegion2	68776
CNVR11	255	1	259697345	259705356	gain	1266	220801047	266165650	Lean_meat_+_bone_in_back	region1inRegion2	8011
CNVR10	220	1	224697068	224765844	gain	1267	220801047	266165650	Backfat_weight	region1inRegion2	68776
CNVR11	255	1	259697345	259705356	gain	1267	220801047	266165650	Backfat_weight	region1inRegion2	8011
CNVR10	220	1	224697068	224765844	gain	1268	220801047	266165650	Backfat_percentage	region1inRegion2	68776
CNVR11	255	1	259697345	259705356	gain	1268	220801047	266165650	Backfat_percentage	region1inRegion2	8011
CNVR10	220	1	224697068	224765844	gain	1270	220801047	266165650	Ham_percentage	region1inRegion2	68776
CNVR11	255	1	259697345	259705356	gain	1270	220801047	266165650	Ham_percentage	region1inRegion2	8011
CNVR10	220	1	224697068	224765844	gain	1272	220801047	266165650	Ham_fat_percentage	region1inRegion2	68776
CNVR11	255	1	259697345	259705356	gain	1272	220801047	266165650	Ham_fat_percentage	region1inRegion2	8011
CNVR10	220	1	224697068	224765844	gain	2842	220801047	271800234	Jowl_weight	region1inRegion2	68776
CNVR11	255	1	259697345	259705356	gain	2842	220801047	271800234	Jowl_weight	region1inRegion2	8011
CNVR12	261	1	268054795	268056507	gain	2842	220801047	271800234	Jowl_weight	region1inRegion2	1712
CNVR13	266	1	271529620	271536665	gain	2842	220801047	271800234	Jowl_weight	region1inRegion2	7045
CNVR10	220	1	224697068	224765844	gain	2846	220801047	271800234	Average_daily_gain	region1inRegion2	68776
CNVR11	255	1	259697345	259705356	gain	2846	220801047	271800234	Average_daily_gain	region1inRegion2	8011
CNVR12	261	1	268054795	268056507	gain	2846	220801047	271800234	Average_daily_gain	region1inRegion2	1712
CNVR13	266	1	271529620	271536665	gain	2846	220801047	271800234	Average_daily_gain	region1inRegion2	7045
CNVR10	220	1	224697068	224765844	gain	2847	220801047	271800234	Average_daily_gain	region1inRegion2	68776
CNVR11	255	1	259697345	259705356	gain	2847	220801047	271800234	Average_daily_gain	region1inRegion2	8011
CNVR12	261	1	268054795	268056507	gain	2847	220801047	271800234	Average_daily_gain	region1inRegion2	1712
CNVR13	266	1	271529620	271536665	gain	2847	220801047	271800234	Average_daily_gain	region1inRegion2	7045
CNVR10	220	1	224697068	224765844	gain	2849	220801047	271800234	Protein_accretion_rate	region1inRegion2	68776
CNVR11	255	1	259697345	259705356	gain	2849	220801047	271800234	Protein_accretion_rate	region1inRegion2	8011
CNVR12	261	1	268054795	268056507	gain	2849	220801047	271800234	Protein_accretion_rate	region1inRegion2	1712
CNVR13	266	1	271529620	271536665	gain	2849	220801047	271800234	Protein_accretion_rate	region1inRegion2	7045
CNVR10	220	1	224697068	224765844	gain	274	220801047	286501984	Vertebra_number	region1inRegion2	68776
CNVR11	255	1	259697345	259705356	gain	274	220801047	286501984	Vertebra_number	region1inRegion2	8011
CNVR12	261	1	268054795	268056507	gain	274	220801047	286501984	Vertebra_number	region1inRegion2	1712
CNVR13	266	1	271529620	271536665	gain	274	220801047	286501984	Vertebra_number	region1inRegion2	7045
CNVR14	279	1	284447951	284512355	loss	274	220801047	286501984	Vertebra_number	region1inRegion2	64404
CNVR11	255	1	259697345	259705356	gain	12721	226764071	265323610	Shoulder_external_fat_weight	region1inRegion2	8011
CNVR11	255	1	259697345	259705356	gain	12729	226764071	265323610	Fat-cuts_percentage	region1inRegion2	8011
CNVR11	255	1	259697345	259705356	gain	12730	226764071	265323610	backfat_above_muscle_dorsi	region1inRegion2	8011
CNVR11	255	1	259697345	259705356	gain	12737	226764071	265323610	Subcutanous_fat_area	region1inRegion2	8011
CNVR11	255	1	259697345	259705356	gain	12741	226764071	265323610	Fat_to_meat_ratio	region1inRegion2	8011
CNVR11	255	1	259697345	259705356	gain	16845	226764071	265323610	Average_daily_gain	region1inRegion2	8011
CNVR11	255	1	259697345	259705356	gain	5480	226764071	265323610	Segmented_neutrophil_number	region1inRegion2	8011
CNVR11	255	1	259697345	259705356	gain	7480	226764071	265323610	Aspartate_amino_transferase_activity	region1inRegion2	8011
CNVR11	255	1	259697345	259705356	gain	835	226764071	265323610	Dressing_percentage	region1inRegion2	8011
CNVR11	255	1	259697345	259705356	gain	840	226764071	265323610	Shoulder_external_fat_weight	region1inRegion2	8011
CNVR11	255	1	259697345	259705356	gain	842	226764071	265323610	Fat-cuts_percentage	region1inRegion2	8011
CNVR11	255	1	259697345	259705356	gain	848	226764071	265323610	pH_24_hr_post-mortem_(loin)	region1inRegion2	8011
CNVR11	255	1	259697345	259705356	gain	850	226764071	265323610	backfat_at_last_rib	region1inRegion2	8011
CNVR11	255	1	259697345	259705356	gain	855	226764071	265323610	Fat-cuts_percentage	region1inRegion2	8011
CNVR11	255	1	259697345	259705356	gain	864	226764071	265323610	Average_backfat_thickness	region1inRegion2	8011
CNVR11	255	1	259697345	259705356	gain	867	226764071	265323610	Backfat_thickness_between_3rd_and_4th_rib	region1inRegion2	8011
CNVR11	255	1	259697345	259705356	gain	10617	226764071	274130998	Gestation_length	region1inRegion2	8011
CNVR12	261	1	268054795	268056507	gain	10617	226764071	274130998	Gestation_length	region1inRegion2	1712
CNVR13	266	1	271529620	271536665	gain	10617	226764071	274130998	Gestation_length	region1inRegion2	7045
CNVR11	255	1	259697345	259705356	gain	278	228164160	274130998	Body_weight_(birth)	region1inRegion2	8011
CNVR12	261	1	268054795	268056507	gain	278	228164160	274130998	Body_weight_(birth)	region1inRegion2	1712
CNVR13	266	1	271529620	271536665	gain	278	228164160	274130998	Body_weight_(birth)	region1inRegion2	7045
CNVR11	255	1	259697345	259705356	gain	3772	228164160	274130998	pH_48_hr_post-mortem_(loin)	region1inRegion2	8011
CNVR12	261	1	268054795	268056507	gain	3772	228164160	274130998	pH_48_hr_post-mortem_(loin)	region1inRegion2	1712
CNVR13	266	1	271529620	271536665	gain	3772	228164160	274130998	pH_48_hr_post-mortem_(loin)	region1inRegion2	7045
CNVR11	255	1	259697345	259705356	gain	21353	228164160	286501984	Spareribs_weight	region1inRegion2	8011
CNVR12	261	1	268054795	268056507	gain	21353	228164160	286501984	Spareribs_weight	region1inRegion2	1712
CNVR13	266	1	271529620	271536665	gain	21353	228164160	286501984	Spareribs_weight	region1inRegion2	7045
CNVR14	279	1	284447951	284512355	loss	21353	228164160	286501984	Spareribs_weight	region1inRegion2	64404
CNVR11	255	1	259697345	259705356	gain	3223	228164160	286501984	Spareribs_weight	region1inRegion2	8011
CNVR12	261	1	268054795	268056507	gain	3223	228164160	286501984	Spareribs_weight	region1inRegion2	1712
CNVR13	266	1	271529620	271536665	gain	3223	228164160	286501984	Spareribs_weight	region1inRegion2	7045
CNVR14	279	1	284447951	284512355	loss	3223	228164160	286501984	Spareribs_weight	region1inRegion2	64404
CNVR11	255	1	259697345	259705356	gain	3773	228164160	292955427	Meat_color_score	region1inRegion2	8011
CNVR12	261	1	268054795	268056507	gain	3773	228164160	292955427	Meat_color_score	region1inRegion2	1712
CNVR13	266	1	271529620	271536665	gain	3773	228164160	292955427	Meat_color_score	region1inRegion2	7045
CNVR14	279	1	284447951	284512355	loss	3773	228164160	292955427	Meat_color_score	region1inRegion2	64404
CNVR11	255	1	259697345	259705356	gain	18634	228164160	306268161	Fat_area_percentage_in_carcass	region1inRegion2	8011
CNVR12	261	1	268054795	268056507	gain	18634	228164160	306268161	Fat_area_percentage_in_carcass	region1inRegion2	1712
CNVR13	266	1	271529620	271536665	gain	18634	228164160	306268161	Fat_area_percentage_in_carcass	region1inRegion2	7045
CNVR14	279	1	284447951	284512355	loss	18634	228164160	306268161	Fat_area_percentage_in_carcass	region1inRegion2	64404
CNVR15	288	1	295235629	295379020	gain	18634	228164160	306268161	Fat_area_percentage_in_carcass	region1inRegion2	143391
CNVR16	290	1	296193901	296202305	loss-gain	18634	228164160	306268161	Fat_area_percentage_in_carcass	region1inRegion2	8404
CNVR17	292	1	296918534	296982162	loss	18634	228164160	306268161	Fat_area_percentage_in_carcass	region1inRegion2	63628
CNVR11	255	1	259697345	259705356	gain	16887	233806417	265323610	Average_daily_gain	region1inRegion2	8011
CNVR11	255	1	259697345	259705356	gain	9	242344389	266165650	Carcass_length	region1inRegion2	8011
CNVR11	255	1	259697345	259705356	gain	2794	242497921	292955427	Percentage_type_I_fibers	region1inRegion2	8011
CNVR12	261	1	268054795	268056507	gain	2794	242497921	292955427	Percentage_type_I_fibers	region1inRegion2	1712
CNVR13	266	1	271529620	271536665	gain	2794	242497921	292955427	Percentage_type_I_fibers	region1inRegion2	7045
CNVR14	279	1	284447951	284512355	loss	2794	242497921	292955427	Percentage_type_I_fibers	region1inRegion2	64404
CNVR11	255	1	259697345	259705356	gain	2795	242497921	292955427	Diameter_of_type_IIb_muscle_fibers	region1inRegion2	8011
CNVR12	261	1	268054795	268056507	gain	2795	242497921	292955427	Diameter_of_type_IIb_muscle_fibers	region1inRegion2	1712
CNVR13	266	1	271529620	271536665	gain	2795	242497921	292955427	Diameter_of_type_IIb_muscle_fibers	region1inRegion2	7045
CNVR14	279	1	284447951	284512355	loss	2795	242497921	292955427	Diameter_of_type_IIb_muscle_fibers	region1inRegion2	64404
CNVR1	83	1	88942731	88998960	gain	22269	249063294	78183912	Average_daily_gain	region2inRegion1	-170879382
CNVR2	88	1	91934647	91942954	loss	22269	249063294	78183912	Average_daily_gain	region2inRegion1	-170879382
CNVR3	92	1	95316086	95335767	gain	22269	249063294	78183912	Average_daily_gain	region2inRegion1	-170879382
CNVR4	99	1	99687218	99704572	gain	22269	249063294	78183912	Average_daily_gain	region2inRegion1	-170879382
CNVR5	101	1	99733649	99785971	gain	22269	249063294	78183912	Average_daily_gain	region2inRegion1	-170879382
CNVR6	103	1	100019309	100027849	gain	22269	249063294	78183912	Average_daily_gain	region2inRegion1	-170879382
CNVR7	141	1	145236440	145265439	gain	22269	249063294	78183912	Average_daily_gain	region2inRegion1	-170879382
CNVR8	156	1	170118820	170121900	gain	22269	249063294	78183912	Average_daily_gain	region2inRegion1	-170879382
CNVR9	191	1	197269718	197274380	gain	22269	249063294	78183912	Average_daily_gain	region2inRegion1	-170879382
CNVR10	220	1	224697068	224765844	gain	22269	249063294	78183912	Average_daily_gain	region2inRegion1	-170879382
CNVR11	255	1	259697345	259705356	gain	582	256785161	270836919	Teat_number	region1inRegion2	8011
CNVR12	261	1	268054795	268056507	gain	582	256785161	270836919	Teat_number	region1inRegion2	1712
CNVR11	255	1	259697345	259705356	gain	1275	257763699	271800234	Musculus_biceps_femoris_and_dissected_ham_ratio	region1inRegion2	8011
CNVR12	261	1	268054795	268056507	gain	1275	257763699	271800234	Musculus_biceps_femoris_and_dissected_ham_ratio	region1inRegion2	1712
CNVR13	266	1	271529620	271536665	gain	1275	257763699	271800234	Musculus_biceps_femoris_and_dissected_ham_ratio	region1inRegion2	7045
CNVR11	255	1	259697345	259705356	gain	7784	257900446	311492855	Drip_loss	region1inRegion2	8011
CNVR12	261	1	268054795	268056507	gain	7784	257900446	311492855	Drip_loss	region1inRegion2	1712
CNVR13	266	1	271529620	271536665	gain	7784	257900446	311492855	Drip_loss	region1inRegion2	7045
CNVR14	279	1	284447951	284512355	loss	7784	257900446	311492855	Drip_loss	region1inRegion2	64404
CNVR15	288	1	295235629	295379020	gain	7784	257900446	311492855	Drip_loss	region1inRegion2	143391
CNVR16	290	1	296193901	296202305	loss-gain	7784	257900446	311492855	Drip_loss	region1inRegion2	8404
CNVR17	292	1	296918534	296982162	loss	7784	257900446	311492855	Drip_loss	region1inRegion2	63628
CNVR11	255	1	259697345	259705356	gain	7861	257900446	311492855	Drip_loss	region1inRegion2	8011
CNVR12	261	1	268054795	268056507	gain	7861	257900446	311492855	Drip_loss	region1inRegion2	1712
CNVR13	266	1	271529620	271536665	gain	7861	257900446	311492855	Drip_loss	region1inRegion2	7045
CNVR14	279	1	284447951	284512355	loss	7861	257900446	311492855	Drip_loss	region1inRegion2	64404
CNVR15	288	1	295235629	295379020	gain	7861	257900446	311492855	Drip_loss	region1inRegion2	143391
CNVR16	290	1	296193901	296202305	loss-gain	7861	257900446	311492855	Drip_loss	region1inRegion2	8404
CNVR17	292	1	296918534	296982162	loss	7861	257900446	311492855	Drip_loss	region1inRegion2	63628
CNVR11	255	1	259697345	259705356	gain	8102	257900446	311492855	Drip_loss	region1inRegion2	8011
CNVR12	261	1	268054795	268056507	gain	8102	257900446	311492855	Drip_loss	region1inRegion2	1712

CNVR13	266	1	271529620	271536665	gain	8102	257900446	311492855	Drip_loss	region1inRegion2	7045
CNVR14	279	1	284447951	284512355	loss	8102	257900446	311492855	Drip_loss	region1inRegion2	64404
CNVR15	288	1	295235629	295379020	gain	8102	257900446	311492855	Drip_loss	region1inRegion2	143391
CNVR16	290	1	296193901	296202305	loss-gain	8102	257900446	311492855	Drip_loss	region1inRegion2	8404
CNVR17	292	1	296918534	296982162	loss	8102	257900446	311492855	Drip_loss	region1inRegion2	63628
CNVR11	255	1	259697345	259705356	gain	8156	257900446	311492855	Drip_loss	region1inRegion2	8011
CNVR12	261	1	268054795	268056507	gain	8156	257900446	311492855	Drip_loss	region1inRegion2	1712
CNVR13	266	1	271529620	271536665	gain	8156	257900446	311492855	Drip_loss	region1inRegion2	7045
CNVR14	279	1	284447951	284512355	loss	8156	257900446	311492855	Drip_loss	region1inRegion2	64404
CNVR15	288	1	295235629	295379020	gain	8156	257900446	311492855	Drip_loss	region1inRegion2	143391
CNVR16	290	1	296193901	296202305	loss-gain	8156	257900446	311492855	Drip_loss	region1inRegion2	8404
CNVR17	292	1	296918534	296982162	loss	8156	257900446	311492855	Drip_loss	region1inRegion2	63628
CNVR11	255	1	259697345	259705356	gain	8348	257900446	311492855	Drip_loss	region1inRegion2	8011
CNVR12	261	1	268054795	268056507	gain	8348	257900446	311492855	Drip_loss	region1inRegion2	1712
CNVR13	266	1	271529620	271536665	gain	8348	257900446	311492855	Drip_loss	region1inRegion2	7045
CNVR14	279	1	284447951	284512355	loss	8348	257900446	311492855	Drip_loss	region1inRegion2	64404
CNVR15	288	1	295235629	295379020	gain	8348	257900446	311492855	Drip_loss	region1inRegion2	143391
CNVR16	290	1	296193901	296202305	loss-gain	8348	257900446	311492855	Drip_loss	region1inRegion2	8404
CNVR17	292	1	296918534	296982162	loss	8348	257900446	311492855	Drip_loss	region1inRegion2	63628
CNVR11	255	1	259697345	259705356	gain	8907	257944209	271800234	Front_leg_conformation	region1inRegion2	8011
CNVR12	261	1	268054795	268056507	gain	8907	257944209	271800234	Front_leg_conformation	region1inRegion2	1712
CNVR13	266	1	271529620	271536665	gain	8907	257944209	271800234	Front_leg_conformation	region1inRegion2	7045
CNVR11	255	1	259697345	259705356	gain	8915	257944209	271800234	Gait_score_(hind)	region1inRegion2	8011
CNVR12	261	1	268054795	268056507	gain	8915	257944209	271800234	Gait_score_(hind)	region1inRegion2	1712
CNVR13	266	1	271529620	271536665	gain	8915	257944209	271800234	Gait_score_(hind)	region1inRegion2	7045
CNVR11	255	1	259697345	259705356	gain	8916	257944209	271800234	Physis_score	region1inRegion2	8011
CNVR12	261	1	268054795	268056507	gain	8916	257944209	271800234	Physis_score	region1inRegion2	1712
CNVR13	266	1	271529620	271536665	gain	8916	257944209	271800234	Physis_score	region1inRegion2	7045
CNVR11	255	1	259697345	259705356	gain	5655	257944209	292955427	Ear_erectness	region1inRegion2	8011
CNVR12	261	1	268054795	268056507	gain	5655	257944209	292955427	Ear_erectness	region1inRegion2	1712
CNVR13	266	1	271529620	271536665	gain	5655	257944209	292955427	Ear_erectness	region1inRegion2	7045
CNVR14	279	1	284447951	284512355	loss	5655	257944209	292955427	Ear_erectness	region1inRegion2	64404
CNVR11	255	1	259697345	259705356	gain	9611	257944209	292955427	C3c_concentration	region1inRegion2	8011
CNVR12	261	1	268054795	268056507	gain	9611	257944209	292955427	C3c_concentration	region1inRegion2	1712
CNVR13	266	1	271529620	271536665	gain	9611	257944209	292955427	C3c_concentration	region1inRegion2	7045
CNVR14	279	1	284447951	284512355	loss	9611	257944209	292955427	C3c_concentration	region1inRegion2	64404
CNVR11	255	1	259697345	259705356	gain	80	257944209	307576631	Drip_loss	region1inRegion2	8011
CNVR12	261	1	268054795	268056507	gain	80	257944209	307576631	Drip_loss	region1inRegion2	1712
CNVR13	266	1	271529620	271536665	gain	80	257944209	307576631	Drip_loss	region1inRegion2	7045
CNVR14	279	1	284447951	284512355	loss	80	257944209	307576631	Drip_loss	region1inRegion2	64404
CNVR15	288	1	295235629	295379020	gain	80	257944209	307576631	Drip_loss	region1inRegion2	143391
CNVR16	290	1	296193901	296202305	loss-gain	80	257944209	307576631	Drip_loss	region1inRegion2	8404
CNVR17	292	1	296918534	296982162	loss	80	257944209	307576631	Drip_loss	region1inRegion2	63628
CNVR12	261	1	268054795	268056507	gain	1250	265323610	279095904	Teat_number	region1inRegion2	1712
CNVR13	266	1	271529620	271536665	gain	1250	265323610	279095904	Teat_number	region1inRegion2	7045
CNVR12	261	1	268054795	268056507	gain	1251	265323610	279095904	Fat-cuts_percentage	region1inRegion2	1712
CNVR13	266	1	271529620	271536665	gain	1251	265323610	279095904	Fat-cuts_percentage	region1inRegion2	7045
CNVR12	261	1	268054795	268056507	gain	12726	265323610	279095904	Backfat_weight	region1inRegion2	1712
CNVR13	266	1	271529620	271536665	gain	12726	265323610	279095904	Backfat_weight	region1inRegion2	7045
CNVR12	261	1	268054795	268056507	gain	12803	265323610	279095904	Adipocyte_diameter	region1inRegion2	1712
CNVR13	266	1	271529620	271536665	gain	12803	265323610	279095904	Adipocyte_diameter	region1inRegion2	7045
CNVR12	261	1	268054795	268056507	gain	5456	265323610	279095904	White_blood_cell_counts	region1inRegion2	1712
CNVR13	266	1	271529620	271536665	gain	5456	265323610	279095904	White_blood_cell_counts	region1inRegion2	7045
CNVR12	261	1	268054795	268056507	gain	5461	265323610	279095904	White_blood_cell_counts	region1inRegion2	1712
CNVR13	266	1	271529620	271536665	gain	5461	265323610	279095904	White_blood_cell_counts	region1inRegion2	7045
CNVR12	261	1	268054795	268056507	gain	6352	265323610	279095904	Alkaline_phosphatase_activity	region1inRegion2	1712
CNVR13	266	1	271529620	271536665	gain	6352	265323610	279095904	Alkaline_phosphatase_activity	region1inRegion2	7045
CNVR12	261	1	268054795	268056507	gain	6355	265323610	279095904	Alkaline_phosphatase_activity	region1inRegion2	1712
CNVR13	266	1	271529620	271536665	gain	6355	265323610	279095904	Alkaline_phosphatase_activity	region1inRegion2	7045
CNVR12	261	1	268054795	268056507	gain	796	265323610	279095904	Fat_weight_(total)	region1inRegion2	1712
CNVR13	266	1	271529620	271536665	gain	796	265323610	279095904	Fat_weight_(total)	region1inRegion2	7045
CNVR12	261	1	268054795	268056507	gain	808	265323610	279095904	Lean_cuts_percentage	region1inRegion2	1712
CNVR13	266	1	271529620	271536665	gain	808	265323610	279095904	Lean_cuts_percentage	region1inRegion2	7045
CNVR12	261	1	268054795	268056507	gain	822	265323610	279095904	Teat_number	region1inRegion2	1712
CNVR13	266	1	271529620	271536665	gain	822	265323610	279095904	Teat_number	region1inRegion2	7045
CNVR12	261	1	268054795	268056507	gain	823	265323610	279095904	Fat-cuts_percentage	region1inRegion2	1712
CNVR13	266	1	271529620	271536665	gain	823	265323610	279095904	Fat-cuts_percentage	region1inRegion2	7045
CNVR12	261	1	268054795	268056507	gain	827	265323610	279095904	Fat_percentage_in_carcass	region1inRegion2	1712
CNVR13	266	1	271529620	271536665	gain	827	265323610	279095904	Fat_percentage_in_carcass	region1inRegion2	7045
CNVR12	261	1	268054795	268056507	gain	828	265323610	279095904	Carcass_length	region1inRegion2	1712
CNVR13	266	1	271529620	271536665	gain	828	265323610	279095904	Carcass_length	region1inRegion2	7045
CNVR12	261	1	268054795	268056507	gain	829	265323610	279095904	Fat_percentage_in_carcass	region1inRegion2	1712
CNVR13	266	1	271529620	271536665	gain	829	265323610	279095904	Fat_percentage_in_carcass	region1inRegion2	7045
CNVR12	261	1	268054795	268056507	gain	845	265323610	279095904	Teat_number	region1inRegion2	1712
CNVR13	266	1	271529620	271536665	gain	845	265323610	279095904	Teat_number	region1inRegion2	7045
CNVR12	261	1	268054795	268056507	gain	851	265323610	279095904	Carcass_length	region1inRegion2	1712
CNVR13	266	1	271529620	271536665	gain	851	265323610	279095904	Carcass_length	region1inRegion2	7045
CNVR12	261	1	268054795	268056507	gain	852	265323610	279095904	Dressing_percentage	region1inRegion2	1712
CNVR13	266	1	271529620	271536665	gain	852	265323610	279095904	Dressing_percentage	region1inRegion2	7045
CNVR12	261	1	268054795	268056507	gain	853	265323610	279095904	Lean_meat_percentage	region1inRegion2	1712
CNVR13	266	1	271529620	271536665	gain	853	265323610	279095904	Lean_meat_percentage	region1inRegion2	7045
CNVR12	261	1	268054795	268056507	gain	854	265323610	279095904	Backfat_weight	region1inRegion2	1712
CNVR13	266	1	271529620	271536665	gain	854	265323610	279095904	Backfat_weight	region1inRegion2	7045
CNVR12	261	1	268054795	268056507	gain	856	265323610	279095904	Ham_weight	region1inRegion2	1712
CNVR13	266	1	271529620	271536665	gain	856	265323610	279095904	Ham_weight	region1inRegion2	7045
CNVR12	261	1	268054795	268056507	gain	857	265323610	279095904	Carcass_length	region1inRegion2	1712
CNVR13	266	1	271529620	271536665	gain	857	265323610	279095904	Carcass_length	region1inRegion2	7045
CNVR12	261	1	268054795	268056507	gain	858	265323610	279095904	Shoulder_external_fat_weight	region1inRegion2	1712
CNVR13	266	1	271529620	271536665	gain	858	265323610	279095904	Shoulder_external_fat_weight	region1inRegion2	7045
CNVR12	261	1	268054795	268056507	gain	859	265323610	279095904	Loin_and_neck_meat_weight	region1inRegion2	1712
CNVR13	266	1	271529620	271536665	gain	859	265323610	279095904	Loin_and_neck_meat_weight	region1inRegion2	7045
CNVR12	261	1	268054795	268056507	gain	861	265323610	279095904	External_fat_on_ham	region1inRegion2	1712
CNVR13	266	1	271529620	271536665	gain	861	265323610	279095904	External_fat_on_ham	region1inRegion2	7045
CNVR12	261	1	268054795	268056507	gain	862	265323610	279095904	Body_weight_(slaughter)	region1inRegion2	1712
CNVR13	266	1	271529620	271536665	gain	862	265323610	279095904	Body_weight_(slaughter)	region1inRegion2	7045
CNVR12	261	1	268054795	268056507	gain	865	265323610	279095904	Loin_muscle_area	region1inRegion2	1712
CNVR13	266	1	271529620	271536665	gain	865	265323610	279095904	Loin_muscle_area	region1inRegion2	7045
CNVR12	261	1	268054795	268056507	gain	873	265323610	279095904	Ham_weight	region1inRegion2	1712
CNVR13	266	1	271529620	271536665	gain	873	265323610	279095904	Ham_weight	region1inRegion2	7045
CNVR12	261	1	268054795	268056507	gain	863	265323610	294659146	Fat-cuts_percentage	region1inRegion2	1712
CNVR13	266	1	271529620	271536665	gain	863	265323610	294659146	Fat-cuts_percentage	region1inRegion2	7045
CNVR14	279	1	284447951	284512355	loss	863	265323610	294659146	Fat-cuts_percentage	region1inRegion2	64404
CNVR12	261	1	268054795	268056507	gain	310	266165650	277108096	Average_backfat_thickness	region1inRegion2	1712
CNVR13	266	1	271529620	271536665	gain	310	266165650	277108096	Average_backfat_thickness	region1inRegion2	7045
CNVR12	261	1	268054795	268056507	gain	21302	266307014	287364389	Thoracic_vertebra_number	region1inRegion2	1712
CNVR13	266	1	271529620	271536665	gain	21302	266307014	287364389	Thoracic_vertebra_number	region1inRegion2	7045
CNVR14	279	1	284447951	284512355	loss	21302	266307014	287364389	Thoracic_vertebra_number	region1inRegion2	64404
CNVR12	261	1	268054795	268056507	gain	21301	267851900	288372626	Vertebra_number	region1inRegion2	1712
CNVR13	266	1	271529620	271536665	gain	21301	267851900	288372626	Vertebra_number	region1inRegion2	7045
CNVR14	279	1	284447951	284512355	loss	21301	267851900	288372626	Vertebra_number	region1inRegion2	64404
CNVR13	266	1	271529620	271536665	gain	21204	268926929	290883097	Average_backfat_thickness	region1inRegion2	7045
CNVR14	279	1	284447951	284512355	loss	21204	268926929	290883097	Average_backfat_thickness	region1inRegion2	64404
CNVR13	266	1	271529620	271536665	gain	107317	271083624	271978821	Red_blood_cell_count	region1inRegion2	7045
CNVR13	266	1	271529620	271536665	gain	12713	271232749	271800234	Abdominal_fat_weight	region1inRegion2	7045
CNVR13	266	1	271529620	271536665	gain	12723	271232749	271800234	Backfat_weight	region1inRegion2	7045
CNVR13	266	1	271529620	271536665	gain	5216	271512220	292845492	Shoulder_subcutaneous_fat_thickness	region1inRegion2	7045
CNVR14	279	1	284447951	284512355	loss	5216	271512220	292845492	Shoulder_subcutaneous_fat_thickness	region1inRegion2	64404
CNVR14	279	1	284447951	284512355	loss	21210	271800234	291609299	intestinal_fat_weight	region1inRegion2	64404
CNVR14	279	1	284447951	284512355	loss	2840	271800234	292955427	Loin_weight	region1inRegion2	64404
CNVR14	279	1	284447951	284512355	loss	8815	271800234	292955427	Age_at_puberty	region1inRegion2	64404
CNVR14	279	1	284447951	284512355	loss	16	271800234	306268161	Trimmed_wholesale_product_/_carcass_weight	region1inRegion2	64404
CNVR15	288	1	295235629	295379020	gain	16	271800234	306268161	Trimmed_wholesale_product_/_carcass_weight	region1inRegion2	143391

CNVR16	290	1	296193901	296202305	loss-gain	16	271800234	306268161	Trimmed_wholesale_product_/_carcass_weight	region1inRegion2	8404
CNVR17	292	1	296918534	296982162	loss	16	271800234	306268161	Trimmed_wholesale_product_/_carcass_weight	region1inRegion2	63628
CNVR14	279	1	284447951	284512355	loss	3132	271800234	306268161	Trimmed_wholesale_product_/_live_weight	region1inRegion2	64404
CNVR15	288	1	295235629	295379020	gain	3132	271800234	306268161	Trimmed_wholesale_product_/_live_weight	region1inRegion2	143391
CNVR16	290	1	296193901	296202305	loss-gain	3132	271800234	306268161	Trimmed_wholesale_product_/_live_weight	region1inRegion2	8404
CNVR17	292	1	296918534	296982162	loss	3132	271800234	306268161	Trimmed_wholesale_product_/_live_weight	region1inRegion2	63628
CNVR14	279	1	284447951	284512355	loss	3133	271800234	306268161	Average_daily_gain	region1inRegion2	64404
CNVR15	288	1	295235629	295379020	gain	3133	271800234	306268161	Average_daily_gain	region1inRegion2	143391
CNVR16	290	1	296193901	296202305	loss-gain	3133	271800234	306268161	Average_daily_gain	region1inRegion2	8404
CNVR17	292	1	296918534	296982162	loss	3133	271800234	306268161	Average_daily_gain	region1inRegion2	63628
CNVR14	279	1	284447951	284512355	loss	3145	271800234	306268161	Average_backfat_thickness	region1inRegion2	64404
CNVR15	288	1	295235629	295379020	gain	3145	271800234	306268161	Average_backfat_thickness	region1inRegion2	143391
CNVR16	290	1	296193901	296202305	loss-gain	3145	271800234	306268161	Average_backfat_thickness	region1inRegion2	8404
CNVR17	292	1	296918534	296982162	loss	3145	271800234	306268161	Average_backfat_thickness	region1inRegion2	63628
CNVR14	279	1	284447951	284512355	loss	3147	271800234	306268161	Loin_weight	region1inRegion2	64404
CNVR15	288	1	295235629	295379020	gain	3147	271800234	306268161	Loin_weight	region1inRegion2	143391
CNVR16	290	1	296193901	296202305	loss-gain	3147	271800234	306268161	Loin_weight	region1inRegion2	8404
CNVR17	292	1	296918534	296982162	loss	3147	271800234	306268161	Loin_weight	region1inRegion2	63628
CNVR14	279	1	284447951	284512355	loss	3148	271800234	306268161	Ham_weight	region1inRegion2	64404
CNVR15	288	1	295235629	295379020	gain	3148	271800234	306268161	Ham_weight	region1inRegion2	143391
CNVR16	290	1	296193901	296202305	loss-gain	3148	271800234	306268161	Ham_weight	region1inRegion2	8404
CNVR17	292	1	296918534	296982162	loss	3148	271800234	306268161	Ham_weight	region1inRegion2	63628
CNVR14	279	1	284447951	284512355	loss	3149	271800234	306268161	Backfat_weight	region1inRegion2	64404
CNVR15	288	1	295235629	295379020	gain	3149	271800234	306268161	Backfat_weight	region1inRegion2	143391
CNVR16	290	1	296193901	296202305	loss-gain	3149	271800234	306268161	Backfat_weight	region1inRegion2	8404
CNVR17	292	1	296918534	296982162	loss	3149	271800234	306268161	Backfat_weight	region1inRegion2	63628
CNVR14	279	1	284447951	284512355	loss	3150	271800234	306268161	Lean_meat_percentage	region1inRegion2	64404
CNVR15	288	1	295235629	295379020	gain	3150	271800234	306268161	Lean_meat_percentage	region1inRegion2	143391
CNVR16	290	1	296193901	296202305	loss-gain	3150	271800234	306268161	Lean_meat_percentage	region1inRegion2	8404
CNVR17	292	1	296918534	296982162	loss	3150	271800234	306268161	Lean_meat_percentage	region1inRegion2	63628
CNVR14	279	1	284447951	284512355	loss	3168	271800234	306268161	pH_for_Semispinalis_Dorsi	region1inRegion2	64404
CNVR15	288	1	295235629	295379020	gain	3168	271800234	306268161	pH_for_Semispinalis_Dorsi	region1inRegion2	143391
CNVR16	290	1	296193901	296202305	loss-gain	3168	271800234	306268161	pH_for_Semispinalis_Dorsi	region1inRegion2	8404
CNVR17	292	1	296918534	296982162	loss	3168	271800234	306268161	pH_for_Semispinalis_Dorsi	region1inRegion2	63628
CNVR14	279	1	284447951	284512355	loss	3169	271800234	306268161	PH_for_Longissmus_dorsi	region1inRegion2	64404
CNVR15	288	1	295235629	295379020	gain	3169	271800234	306268161	PH_for_Longissmus_dorsi	region1inRegion2	143391
CNVR16	290	1	296193901	296202305	loss-gain	3169	271800234	306268161	PH_for_Longissmus_dorsi	region1inRegion2	8404
CNVR17	292	1	296918534	296982162	loss	3169	271800234	306268161	PH_for_Longissmus_dorsi	region1inRegion2	63628
CNVR14	279	1	284447951	284512355	loss	3170	271800234	306268161	PH_for_Biceps_femoris	region1inRegion2	64404
CNVR15	288	1	295235629	295379020	gain	3170	271800234	306268161	PH_for_Biceps_femoris	region1inRegion2	143391
CNVR16	290	1	296193901	296202305	loss-gain	3170	271800234	306268161	PH_for_Biceps_femoris	region1inRegion2	8404
CNVR17	292	1	296918534	296982162	loss	3170	271800234	306268161	PH_for_Biceps_femoris	region1inRegion2	63628
CNVR14	279	1	284447951	284512355	loss	35	271800234	306268161	Average_backfat_thickness	region1inRegion2	64404
CNVR15	288	1	295235629	295379020	gain	35	271800234	306268161	Average_backfat_thickness	region1inRegion2	143391
CNVR16	290	1	296193901	296202305	loss-gain	35	271800234	306268161	Average_backfat_thickness	region1inRegion2	8404
CNVR17	292	1	296918534	296982162	loss	35	271800234	306268161	Average_backfat_thickness	region1inRegion2	63628
CNVR14	279	1	284447951	284512355	loss	659	271800234	307576631	Average_daily_gain	region1inRegion2	64404
CNVR15	288	1	295235629	295379020	gain	659	271800234	307576631	Average_daily_gain	region1inRegion2	143391
CNVR16	290	1	296193901	296202305	loss-gain	659	271800234	307576631	Average_daily_gain	region1inRegion2	8404
CNVR17	292	1	296918534	296982162	loss	659	271800234	307576631	Average_daily_gain	region1inRegion2	63628
CNVR14	279	1	284447951	284512355	loss	660	271800234	307576631	body_weight_(10_weeks)	region1inRegion2	64404
CNVR15	288	1	295235629	295379020	gain	660	271800234	307576631	body_weight_(10_weeks)	region1inRegion2	143391
CNVR16	290	1	296193901	296202305	loss-gain	660	271800234	307576631	body_weight_(10_weeks)	region1inRegion2	8404
CNVR17	292	1	296918534	296982162	loss	660	271800234	307576631	body_weight_(10_weeks)	region1inRegion2	63628
CNVR14	279	1	284447951	284512355	loss	661	271800234	307576631	body_weight_(13_weeks)	region1inRegion2	64404
CNVR15	288	1	295235629	295379020	gain	661	271800234	307576631	body_weight_(13_weeks)	region1inRegion2	143391
CNVR16	290	1	296193901	296202305	loss-gain	661	271800234	307576631	body_weight_(13_weeks)	region1inRegion2	8404
CNVR17	292	1	296918534	296982162	loss	661	271800234	307576631	body_weight_(13_weeks)	region1inRegion2	63628
CNVR14	279	1	284447951	284512355	loss	662	271800234	307576631	body_weight_(17_weeks)	region1inRegion2	64404
CNVR15	288	1	295235629	295379020	gain	662	271800234	307576631	body_weight_(17_weeks)	region1inRegion2	143391
CNVR16	290	1	296193901	296202305	loss-gain	662	271800234	307576631	body_weight_(17_weeks)	region1inRegion2	8404
CNVR17	292	1	296918534	296982162	loss	662	271800234	307576631	body_weight_(17_weeks)	region1inRegion2	63628
CNVR14	279	1	284447951	284512355	loss	2931	274130998	292955427	Marbling	region1inRegion2	64404
CNVR14	279	1	284447951	284512355	loss	446	274130998	292955427	Average_daily_gain	region1inRegion2	64404
CNVR14	279	1	284447951	284512355	loss	447	274130998	292955427	Average_daily_gain	region1inRegion2	64404
CNVR14	279	1	284447951	284512355	loss	448	274130998	292955427	Average_daily_gain	region1inRegion2	64404
CNVR14	279	1	284447951	284512355	loss	5930	274130998	292955427	Average_daily_gain	region1inRegion2	64404
CNVR14	279	1	284447951	284512355	loss	4	277108096	306268161	Loin_muscle_area	region1inRegion2	64404
CNVR15	288	1	295235629	295379020	gain	4	277108096	306268161	Loin_muscle_area	region1inRegion2	143391
CNVR16	290	1	296193901	296202305	loss-gain	4	277108096	306268161	Loin_muscle_area	region1inRegion2	8404
CNVR17	292	1	296918534	296982162	loss	4	277108096	306268161	Loin_muscle_area	region1inRegion2	63628
CNVR14	279	1	284447951	284512355	loss	21205	277978209	291709412	Backfat_at_first_rib	region1inRegion2	64404
CNVR14	279	1	284447951	284512355	loss	12727	279095904	294659146	Fat-cuts_percentage	region1inRegion2	64404
CNVR14	279	1	284447951	284512355	loss	12739	279095904	294659146	Fat_to_meat_ratio	region1inRegion2	64404
CNVR14	279	1	284447951	284512355	loss	824	279095904	294659146	Lean_meat_percentage	region1inRegion2	64404
CNVR14	279	1	284447951	284512355	loss	847	279095904	294659146	Conductivity_24_hours_post-mortem	region1inRegion2	64404
CNVR14	279	1	284447951	284512355	loss	27	279095904	306268161	backfat_at_last_rib	region1inRegion2	64404
CNVR15	288	1	295235629	295379020	gain	27	279095904	306268161	backfat_at_last_rib	region1inRegion2	143391
CNVR16	290	1	296193901	296202305	loss-gain	27	279095904	306268161	backfat_at_last_rib	region1inRegion2	8404
CNVR17	292	1	296918534	296982162	loss	27	279095904	306268161	backfat_at_last_rib	region1inRegion2	63628
CNVR14	279	1	284447951	284512355	loss	30	279095904	306268161	Backfat_at_last_lumbar	region1inRegion2	64404
CNVR15	288	1	295235629	295379020	gain	30	279095904	306268161	Backfat_at_last_lumbar	region1inRegion2	143391
CNVR16	290	1	296193901	296202305	loss-gain	30	279095904	306268161	Backfat_at_last_lumbar	region1inRegion2	8404
CNVR17	292	1	296918534	296982162	loss	30	279095904	306268161	Backfat_at_last_lumbar	region1inRegion2	63628
CNVR14	279	1	284447951	284512355	loss	5250	279476353	292955427	Shoulder_subcutaneous_fat_thickness	region1inRegion2	64404
CNVR14	279	1	284447951	284512355	loss	23	280547030	303972175	Backfat_at_tenth_rib	region1inRegion2	64404
CNVR15	288	1	295235629	295379020	gain	23	280547030	303972175	Backfat_at_tenth_rib	region1inRegion2	143391
CNVR16	290	1	296193901	296202305	loss-gain	23	280547030	303972175	Backfat_at_tenth_rib	region1inRegion2	8404
CNVR17	292	1	296918534	296982162	loss	23	280547030	303972175	Backfat_at_tenth_rib	region1inRegion2	63628
CNVR14	279	1	284447951	284512355	loss	13	280547030	306268161	Trimmed_wholesale_product_/_live_weight	region1inRegion2	64404
CNVR15	288	1	295235629	295379020	gain	13	280547030	306268161	Trimmed_wholesale_product_/_live_weight	region1inRegion2	143391
CNVR16	290	1	296193901	296202305	loss-gain	13	280547030	306268161	Trimmed_wholesale_product_/_live_weight	region1inRegion2	8404
CNVR17	292	1	296918534	296982162	loss	13	280547030	306268161	Trimmed_wholesale_product_/_live_weight	region1inRegion2	63628
CNVR15	288	1	295235629	295379020	gain	169	290462910	301130831	body_weight_(13_weeks)	region1inRegion2	143391
CNVR16	290	1	296193901	296202305	loss-gain	169	290462910	301130831	body_weight_(13_weeks)	region1inRegion2	8404
CNVR17	292	1	296918534	296982162	loss	169	290462910	301130831	body_weight_(13_weeks)	region1inRegion2	63628
CNVR15	288	1	295235629	295379020	gain	401	292695065	307576631	Backfat_weight	region1inRegion2	143391
CNVR16	290	1	296193901	296202305	loss-gain	401	292695065	307576631	Backfat_weight	region1inRegion2	8404
CNVR17	292	1	296918534	296982162	loss	401	292695065	307576631	Backfat_weight	region1inRegion2	63628
CNVR15	288	1	295235629	295379020	gain	376	293329173	311724687	Ham_weight	region1inRegion2	143391
CNVR16	290	1	296193901	296202305	loss-gain	376	293329173	311724687	Ham_weight	region1inRegion2	8404
CNVR17	292	1	296918534	296982162	loss	376	293329173	311724687	Ham_weight	region1inRegion2	63628
CNVR15	288	1	295235629	295379020	gain	375	294756973	311724687	Loin_weight	region1inRegion2	143391
CNVR16	290	1	296193901	296202305	loss-gain	375	294756973	311724687	Loin_weight	region1inRegion2	8404
CNVR17	292	1	296918534	296982162	loss	375	294756973	311724687	Loin_weight	region1inRegion2	63628
CNVR15	288	1	295235629	295379020	gain	402	294756973	311724687	Leaf_fat_weight	region1inRegion2	143391
CNVR16	290	1	296193901	296202305	loss-gain	402	294756973	311724687	Leaf_fat_weight	region1inRegion2	8404
CNVR17	292	1	296918534	296982162	loss	402	294756973	311724687	Leaf_fat_weight	region1inRegion2	63628
CNVR18	298	1	312363155	312415324	gain	403	306172623	315321322	Percentage_of_backfat_and_leaf_fat_in_carcass	region1inRegion2	52169
CNVR19	299	1	312415324	312437693	gain	403	306172623	315321322	Percentage_of_backfat_and_leaf_fat_in_carcass	region1inRegion2	22369
CNVR20	312	2	11511074	11530117	gain	379	609757	12263031	Loin_weight	region1inRegion2	19043
CNVR20	312	2	11511074	11530117	gain	5146	609757	12263031	Neck_fat_weight	region1inRegion2	19043
CNVR20	312	2	11511074	11530117	gain	381	609757	12560715	Estimated_carcass_lean_content	region1inRegion2	19043
CNVR21	314	2	12364565	12398203	loss	381	609757	12560715	Estimated_carcass_lean_content	region1inRegion2	33638
CNVR20	312	2	11511074	11530117	gain	404	609757	13002292	Backfat_weight	region1inRegion2	19043
CNVR21	314	2	12364565	12398203	loss	404	609757	13002292	Backfat_weight	region1inRegion2	33638
CNVR22	316	2	12689951	12700771	loss	404	609757	13002292	Backfat_weight	region1inRegion2	10820
CNVR20	312	2	11511074	11530117	gain	405	609757	13002292	Percentage_of_backfat_and_leaf_fat_in_carcass	region1inRegion2	19043
CNVR21	314	2	12364565	12398203	loss	405	609757	13002292	Percentage_of_backfat_and_leaf_fat_in_carcass	region1inRegion2	33638
CNVR22	316	2	12689951	12700771	loss	405	609757	13002292	Percentage_of_backfat_and_leaf_fat_in_carcass	region1inRegion2	10820
CNVR20	312	2	11511074	11530117	gain	380	609757	13080712	Loin_and_ham_percentage_in_carcass	region1inRegion2	19043
CNVR21	314	2	12364565	12398203	loss	380	609757	13080712	Loin_and_ham_percentage_in_carcass	region1inRegion2	33638

CNVR22	316	2	12689951	12700771	loss	380	609757	13080712	Loin_and_ham_percentage_in_carcass	region1inRegion2	10820
CNVR20	312	2	11511074	11530117	gain	16834	609757	13366532	backfat_at_last_rib	region1inRegion2	19043
CNVR21	314	2	12364565	12398203	loss	16834	609757	13366532	backfat_at_last_rib	region1inRegion2	33638
CNVR22	316	2	12689951	12700771	loss	16834	609757	13366532	backfat_at_last_rib	region1inRegion2	10820
CNVR20	312	2	11511074	11530117	gain	16846	609757	13366532	Average_daily_gain	region1inRegion2	19043
CNVR21	314	2	12364565	12398203	loss	16846	609757	13366532	Average_daily_gain	region1inRegion2	33638
CNVR22	316	2	12689951	12700771	loss	16846	609757	13366532	Average_daily_gain	region1inRegion2	10820
CNVR20	312	2	11511074	11530117	gain	17997	609757	13366532	Average_backfat_thickness	region1inRegion2	19043
CNVR21	314	2	12364565	12398203	loss	17997	609757	13366532	Average_backfat_thickness	region1inRegion2	33638
CNVR22	316	2	12689951	12700771	loss	17997	609757	13366532	Average_backfat_thickness	region1inRegion2	10820
CNVR20	312	2	11511074	11530117	gain	18029	609757	13366532	Adipocyte_diameter	region1inRegion2	19043
CNVR21	314	2	12364565	12398203	loss	18029	609757	13366532	Adipocyte_diameter	region1inRegion2	33638
CNVR22	316	2	12689951	12700771	loss	18029	609757	13366532	Adipocyte_diameter	region1inRegion2	10820
CNVR20	312	2	11511074	11530117	gain	349	609757	13366532	Average_backfat_thickness	region1inRegion2	19043
CNVR21	314	2	12364565	12398203	loss	349	609757	13366532	Average_backfat_thickness	region1inRegion2	33638
CNVR22	316	2	12689951	12700771	loss	349	609757	13366532	Average_backfat_thickness	region1inRegion2	10820
CNVR20	312	2	11511074	11530117	gain	3797	609757	13366532	Average_backfat_thickness	region1inRegion2	19043
CNVR21	314	2	12364565	12398203	loss	3797	609757	13366532	Average_backfat_thickness	region1inRegion2	33638
CNVR22	316	2	12689951	12700771	loss	3797	609757	13366532	Average_backfat_thickness	region1inRegion2	10820
CNVR20	312	2	11511074	11530117	gain	3798	609757	13366532	Backfat_at_tenth_rib	region1inRegion2	19043
CNVR21	314	2	12364565	12398203	loss	3798	609757	13366532	Backfat_at_tenth_rib	region1inRegion2	33638
CNVR22	316	2	12689951	12700771	loss	3798	609757	13366532	Backfat_at_tenth_rib	region1inRegion2	10820
CNVR20	312	2	11511074	11530117	gain	3799	609757	13366532	Backfat_at_last_lumbar	region1inRegion2	19043
CNVR21	314	2	12364565	12398203	loss	3799	609757	13366532	Backfat_at_last_lumbar	region1inRegion2	33638
CNVR22	316	2	12689951	12700771	loss	3799	609757	13366532	Backfat_at_last_lumbar	region1inRegion2	10820
CNVR20	312	2	11511074	11530117	gain	3800	609757	13366532	backfat_at_last_rib	region1inRegion2	19043
CNVR21	314	2	12364565	12398203	loss	3800	609757	13366532	backfat_at_last_rib	region1inRegion2	33638
CNVR22	316	2	12689951	12700771	loss	3800	609757	13366532	backfat_at_last_rib	region1inRegion2	10820
CNVR20	312	2	11511074	11530117	gain	3801	609757	13366532	Loin_muscle_area	region1inRegion2	19043
CNVR21	314	2	12364565	12398203	loss	3801	609757	13366532	Loin_muscle_area	region1inRegion2	33638
CNVR22	316	2	12689951	12700771	loss	3801	609757	13366532	Loin_muscle_area	region1inRegion2	10820
CNVR20	312	2	11511074	11530117	gain	618	609757	15441523	Average_backfat_thickness	region1inRegion2	19043
CNVR21	314	2	12364565	12398203	loss	618	609757	15441523	Average_backfat_thickness	region1inRegion2	33638
CNVR22	316	2	12689951	12700771	loss	618	609757	15441523	Average_backfat_thickness	region1inRegion2	10820
CNVR23	319	2	14000848	14032642	loss-gain	618	609757	15441523	Average_backfat_thickness	region1inRegion2	31794
CNVR24	320	2	14728311	14771718	gain	618	609757	15441523	Average_backfat_thickness	region1inRegion2	43407
CNVR25	321	2	15024071	15060448	loss	618	609757	15441523	Average_backfat_thickness	region1inRegion2	36377
CNVR20	312	2	11511074	11530117	gain	619	609757	15441523	Ham_percentage	region1inRegion2	19043
CNVR21	314	2	12364565	12398203	loss	619	609757	15441523	Ham_percentage	region1inRegion2	33638
CNVR22	316	2	12689951	12700771	loss	619	609757	15441523	Ham_percentage	region1inRegion2	10820
CNVR23	319	2	14000848	14032642	loss-gain	619	609757	15441523	Ham_percentage	region1inRegion2	31794
CNVR24	320	2	14728311	14771718	gain	619	609757	15441523	Ham_percentage	region1inRegion2	43407
CNVR25	321	2	15024071	15060448	loss	619	609757	15441523	Ham_percentage	region1inRegion2	36377
CNVR20	312	2	11511074	11530117	gain	620	609757	15441523	Loin_percentage	region1inRegion2	19043
CNVR21	314	2	12364565	12398203	loss	620	609757	15441523	Loin_percentage	region1inRegion2	33638
CNVR22	316	2	12689951	12700771	loss	620	609757	15441523	Loin_percentage	region1inRegion2	10820
CNVR23	319	2	14000848	14032642	loss-gain	620	609757	15441523	Loin_percentage	region1inRegion2	31794
CNVR24	320	2	14728311	14771718	gain	620	609757	15441523	Loin_percentage	region1inRegion2	43407
CNVR25	321	2	15024071	15060448	loss	620	609757	15441523	Loin_percentage	region1inRegion2	36377
CNVR20	312	2	11511074	11530117	gain	621	609757	15441523	Lean_meat_percentage	region1inRegion2	19043
CNVR21	314	2	12364565	12398203	loss	621	609757	15441523	Lean_meat_percentage	region1inRegion2	33638
CNVR22	316	2	12689951	12700771	loss	621	609757	15441523	Lean_meat_percentage	region1inRegion2	10820
CNVR23	319	2	14000848	14032642	loss-gain	621	609757	15441523	Lean_meat_percentage	region1inRegion2	31794
CNVR24	320	2	14728311	14771718	gain	621	609757	15441523	Lean_meat_percentage	region1inRegion2	43407
CNVR25	321	2	15024071	15060448	loss	621	609757	15441523	Lean_meat_percentage	region1inRegion2	36377
CNVR20	312	2	11511074	11530117	gain	622	609757	15441523	Backfat_percentage	region1inRegion2	19043
CNVR21	314	2	12364565	12398203	loss	622	609757	15441523	Backfat_percentage	region1inRegion2	33638
CNVR22	316	2	12689951	12700771	loss	622	609757	15441523	Backfat_percentage	region1inRegion2	10820
CNVR23	319	2	14000848	14032642	loss-gain	622	609757	15441523	Backfat_percentage	region1inRegion2	31794
CNVR24	320	2	14728311	14771718	gain	622	609757	15441523	Backfat_percentage	region1inRegion2	43407
CNVR25	321	2	15024071	15060448	loss	622	609757	15441523	Backfat_percentage	region1inRegion2	36377
CNVR20	312	2	11511074	11530117	gain	623	609757	15441523	Fat-cuts_percentage	region1inRegion2	19043
CNVR21	314	2	12364565	12398203	loss	623	609757	15441523	Fat-cuts_percentage	region1inRegion2	33638
CNVR22	316	2	12689951	12700771	loss	623	609757	15441523	Fat-cuts_percentage	region1inRegion2	10820
CNVR23	319	2	14000848	14032642	loss-gain	623	609757	15441523	Fat-cuts_percentage	region1inRegion2	31794
CNVR24	320	2	14728311	14771718	gain	623	609757	15441523	Fat-cuts_percentage	region1inRegion2	43407
CNVR25	321	2	15024071	15060448	loss	623	609757	15441523	Fat-cuts_percentage	region1inRegion2	36377
CNVR20	312	2	11511074	11530117	gain	197	609757	15823608	Average_backfat_thickness	region1inRegion2	19043
CNVR21	314	2	12364565	12398203	loss	197	609757	15823608	Average_backfat_thickness	region1inRegion2	33638
CNVR22	316	2	12689951	12700771	loss	197	609757	15823608	Average_backfat_thickness	region1inRegion2	10820
CNVR23	319	2	14000848	14032642	loss-gain	197	609757	15823608	Average_backfat_thickness	region1inRegion2	31794
CNVR24	320	2	14728311	14771718	gain	197	609757	15823608	Average_backfat_thickness	region1inRegion2	43407
CNVR25	321	2	15024071	15060448	loss	197	609757	15823608	Average_backfat_thickness	region1inRegion2	36377
CNVR20	312	2	11511074	11530117	gain	199	609757	15823608	Average_backfat_thickness	region1inRegion2	19043
CNVR21	314	2	12364565	12398203	loss	199	609757	15823608	Average_backfat_thickness	region1inRegion2	33638
CNVR22	316	2	12689951	12700771	loss	199	609757	15823608	Average_backfat_thickness	region1inRegion2	10820
CNVR23	319	2	14000848	14032642	loss-gain	199	609757	15823608	Average_backfat_thickness	region1inRegion2	31794
CNVR24	320	2	14728311	14771718	gain	199	609757	15823608	Average_backfat_thickness	region1inRegion2	43407
CNVR25	321	2	15024071	15060448	loss	199	609757	15823608	Average_backfat_thickness	region1inRegion2	36377
CNVR20	312	2	11511074	11530117	gain	198	609757	15946890	Average_backfat_thickness	region1inRegion2	19043
CNVR21	314	2	12364565	12398203	loss	198	609757	15946890	Average_backfat_thickness	region1inRegion2	33638
CNVR22	316	2	12689951	12700771	loss	198	609757	15946890	Average_backfat_thickness	region1inRegion2	10820
CNVR23	319	2	14000848	14032642	loss-gain	198	609757	15946890	Average_backfat_thickness	region1inRegion2	31794
CNVR24	320	2	14728311	14771718	gain	198	609757	15946890	Average_backfat_thickness	region1inRegion2	43407
CNVR25	321	2	15024071	15060448	loss	198	609757	15946890	Average_backfat_thickness	region1inRegion2	36377
CNVR20	312	2	11511074	11530117	gain	9591	609757	18209772	Hemolytic_complement_activity_(alternative_pathway)	region1inRegion2	19043
CNVR21	314	2	12364565	12398203	loss	9591	609757	18209772	Hemolytic_complement_activity_(alternative_pathway)	region1inRegion2	33638
CNVR22	316	2	12689951	12700771	loss	9591	609757	18209772	Hemolytic_complement_activity_(alternative_pathway)	region1inRegion2	10820
CNVR23	319	2	14000848	14032642	loss-gain	9591	609757	18209772	Hemolytic_complement_activity_(alternative_pathway)	region1inRegion2	31794
CNVR24	320	2	14728311	14771718	gain	9591	609757	18209772	Hemolytic_complement_activity_(alternative_pathway)	region1inRegion2	43407
CNVR25	321	2	15024071	15060448	loss	9591	609757	18209772	Hemolytic_complement_activity_(alternative_pathway)	region1inRegion2	36377
CNVR20	312	2	11511074	11530117	gain	9592	609757	18209772	Hemolytic_complement_activity_(alternative_pathway)	region1inRegion2	19043
CNVR21	314	2	12364565	12398203	loss	9592	609757	18209772	Hemolytic_complement_activity_(alternative_pathway)	region1inRegion2	33638
CNVR22	316	2	12689951	12700771	loss	9592	609757	18209772	Hemolytic_complement_activity_(alternative_pathway)	region1inRegion2	10820
CNVR23	319	2	14000848	14032642	loss-gain	9592	609757	18209772	Hemolytic_complement_activity_(alternative_pathway)	region1inRegion2	31794
CNVR24	320	2	14728311	14771718	gain	9592	609757	18209772	Hemolytic_complement_activity_(alternative_pathway)	region1inRegion2	43407
CNVR25	321	2	15024071	15060448	loss	9592	609757	18209772	Hemolytic_complement_activity_(alternative_pathway)	region1inRegion2	36377
CNVR20	312	2	11511074	11530117	gain	9593	609757	18209772	Hemolytic_complement_activity_(alternative_pathway)	region1inRegion2	19043
CNVR21	314	2	12364565	12398203	loss	9593	609757	18209772	Hemolytic_complement_activity_(alternative_pathway)	region1inRegion2	33638
CNVR22	316	2	12689951	12700771	loss	9593	609757	18209772	Hemolytic_complement_activity_(alternative_pathway)	region1inRegion2	10820
CNVR23	319	2	14000848	14032642	loss-gain	9593	609757	18209772	Hemolytic_complement_activity_(alternative_pathway)	region1inRegion2	31794
CNVR24	320	2	14728311	14771718	gain	9593	609757	18209772	Hemolytic_complement_activity_(alternative_pathway)	region1inRegion2	43407
CNVR25	321	2	15024071	15060448	loss	9593	609757	18209772	Hemolytic_complement_activity_(alternative_pathway)	region1inRegion2	36377
CNVR20	312	2	11511074	11530117	gain	407	609757	19340056	backfat_at_last_rib	region1inRegion2	19043
CNVR21	314	2	12364565	12398203	loss	407	609757	19340056	backfat_at_last_rib	region1inRegion2	33638
CNVR22	316	2	12689951	12700771	loss	407	609757	19340056	backfat_at_last_rib	region1inRegion2	10820
CNVR23	319	2	14000848	14032642	loss-gain	407	609757	19340056	backfat_at_last_rib	region1inRegion2	31794
CNVR24	320	2	14728311	14771718	gain	407	609757	19340056	backfat_at_last_rib	region1inRegion2	43407
CNVR25	321	2	15024071	15060448	loss	407	609757	19340056	backfat_at_last_rib	region1inRegion2	36377
CNVR20	312	2	11511074	11530117	gain	17602	609757	21136167	Interleukin_10_level	region1inRegion2	19043
CNVR21	314	2	12364565	12398203	loss	17602	609757	21136167	Interleukin_10_level	region1inRegion2	33638
CNVR22	316	2	12689951	12700771	loss	17602	609757	21136167	Interleukin_10_level	region1inRegion2	10820
CNVR23	319	2	14000848	14032642	loss-gain	17602	609757	21136167	Interleukin_10_level	region1inRegion2	31794
CNVR24	320	2	14728311	14771718	gain	17602	609757	21136167	Interleukin_10_level	region1inRegion2	43407
CNVR25	321	2	15024071	15060448	loss	17602	609757	21136167	Interleukin_10_level	region1inRegion2	36377
CNVR20	312	2	11511074	11530117	gain	17618	609757	21136167	Toll-like_receptor_9_level	region1inRegion2	19043
CNVR21	314	2	12364565	12398203	loss	17618	609757	21136167	Toll-like_receptor_9_level	region1inRegion2	33638
CNVR22	316	2	12689951	12700771	loss	17618	609757	21136167	Toll-like_receptor_9_level	region1inRegion2	10820
CNVR23	319	2	14000848	14032642	loss-gain	17618	609757	21136167	Toll-like_receptor_9_level	region1inRegion2	31794
CNVR24	320	2	14728311	14771718	gain	17618	609757	21136167	Toll-like_receptor_9_level	region1inRegion2	43407
CNVR25	321	2	15024071	15060448	loss	17618	609757	21136167	Toll-like_receptor_9_level	region1inRegion2	36377
CNVR20	312	2	11511074	11530117	gain	406	609757	21763657	Backfat_thickness_between_3rd_and_4th_rib	region1inRegion2	19043

CNVR21	314	2	12364565	12398203	loss	406	609757	21763657	Backfat_thickness_between_3rd_and_4th_rib	region1inRegion2	33638
CNVR22	316	2	12689951	12700771	loss	406	609757	21763657	Backfat_thickness_between_3rd_and_4th_rib	region1inRegion2	10820
CNVR23	319	2	14000848	14032642	loss-gain	406	609757	21763657	Backfat_thickness_between_3rd_and_4th_rib	region1inRegion2	31794
CNVR24	320	2	14728311	14771718	gain	406	609757	21763657	Backfat_thickness_between_3rd_and_4th_rib	region1inRegion2	43407
CNVR25	321	2	15024071	15060448	loss	406	609757	21763657	Backfat_thickness_between_3rd_and_4th_rib	region1inRegion2	36377
CNVR20	312	2	11511074	11530117	gain	3839	609757	25252518	muscle_protein_percentage	region1inRegion2	19043
CNVR21	314	2	12364565	12398203	loss	3839	609757	25252518	muscle_protein_percentage	region1inRegion2	33638
CNVR22	316	2	12689951	12700771	loss	3839	609757	25252518	muscle_protein_percentage	region1inRegion2	10820
CNVR23	319	2	14000848	14032642	loss-gain	3839	609757	25252518	muscle_protein_percentage	region1inRegion2	31794
CNVR24	320	2	14728311	14771718	gain	3839	609757	25252518	muscle_protein_percentage	region1inRegion2	43407
CNVR25	321	2	15024071	15060448	loss	3839	609757	25252518	muscle_protein_percentage	region1inRegion2	36377
CNVR20	312	2	11511074	11530117	gain	3890	609757	27552141	Feed_intake	region1inRegion2	19043
CNVR21	314	2	12364565	12398203	loss	3890	609757	27552141	Feed_intake	region1inRegion2	33638
CNVR22	316	2	12689951	12700771	loss	3890	609757	27552141	Feed_intake	region1inRegion2	10820
CNVR23	319	2	14000848	14032642	loss-gain	3890	609757	27552141	Feed_intake	region1inRegion2	31794
CNVR24	320	2	14728311	14771718	gain	3890	609757	27552141	Feed_intake	region1inRegion2	43407
CNVR25	321	2	15024071	15060448	loss	3890	609757	27552141	Feed_intake	region1inRegion2	36377
CNVR20	312	2	11511074	11530117	gain	364	609757	43738502	Average_backfat_thickness	region1inRegion2	19043
CNVR21	314	2	12364565	12398203	loss	364	609757	43738502	Average_backfat_thickness	region1inRegion2	33638
CNVR22	316	2	12689951	12700771	loss	364	609757	43738502	Average_backfat_thickness	region1inRegion2	10820
CNVR23	319	2	14000848	14032642	loss-gain	364	609757	43738502	Average_backfat_thickness	region1inRegion2	31794
CNVR24	320	2	14728311	14771718	gain	364	609757	43738502	Average_backfat_thickness	region1inRegion2	43407
CNVR25	321	2	15024071	15060448	loss	364	609757	43738502	Average_backfat_thickness	region1inRegion2	36377
CNVR20	312	2	11511074	11530117	gain	3941	609757	48775112	Backfat_at_first_rib	region1inRegion2	19043
CNVR21	314	2	12364565	12398203	loss	3941	609757	48775112	Backfat_at_first_rib	region1inRegion2	33638
CNVR22	316	2	12689951	12700771	loss	3941	609757	48775112	Backfat_at_first_rib	region1inRegion2	10820
CNVR23	319	2	14000848	14032642	loss-gain	3941	609757	48775112	Backfat_at_first_rib	region1inRegion2	31794
CNVR24	320	2	14728311	14771718	gain	3941	609757	48775112	Backfat_at_first_rib	region1inRegion2	43407
CNVR25	321	2	15024071	15060448	loss	3941	609757	48775112	Backfat_at_first_rib	region1inRegion2	36377
CNVR26	343	2	44800086	44803691	gain	3941	609757	48775112	Backfat_at_first_rib	region1inRegion2	3605
CNVR20	312	2	11511074	11530117	gain	16875	609757	85009446	backfat_at_last_rib	region1inRegion2	19043
CNVR21	314	2	12364565	12398203	loss	16875	609757	85009446	backfat_at_last_rib	region1inRegion2	33638
CNVR22	316	2	12689951	12700771	loss	16875	609757	85009446	backfat_at_last_rib	region1inRegion2	10820
CNVR23	319	2	14000848	14032642	loss-gain	16875	609757	85009446	backfat_at_last_rib	region1inRegion2	31794
CNVR24	320	2	14728311	14771718	gain	16875	609757	85009446	backfat_at_last_rib	region1inRegion2	43407
CNVR25	321	2	15024071	15060448	loss	16875	609757	85009446	backfat_at_last_rib	region1inRegion2	36377
CNVR26	343	2	44800086	44803691	gain	16875	609757	85009446	backfat_at_last_rib	region1inRegion2	3605
CNVR27	348	2	54685639	54717573	loss-gain	16875	609757	85009446	backfat_at_last_rib	region1inRegion2	31934
CNVR28	353	2	56935884	56944870	loss	16875	609757	85009446	backfat_at_last_rib	region1inRegion2	8986
CNVR29	355	2	58030910	58035496	gain	16875	609757	85009446	backfat_at_last_rib	region1inRegion2	4586
CNVR30	359	2	61357153	61369311	loss	16875	609757	85009446	backfat_at_last_rib	region1inRegion2	12158
CNVR31	362	2	62416028	62428337	gain	16875	609757	85009446	backfat_at_last_rib	region1inRegion2	12309
CNVR32	364	2	62628253	62738661	gain	16875	609757	85009446	backfat_at_last_rib	region1inRegion2	110408
CNVR33	378	2	67915859	67952389	gain	16875	609757	85009446	backfat_at_last_rib	region1inRegion2	36530
CNVR34	379	2	68061173	68181801	gain	16875	609757	85009446	backfat_at_last_rib	region1inRegion2	120628
CNVR20	312	2	11511074	11530117	gain	15090	609757	101614625	Creatine_kinase_level	region1inRegion2	19043
CNVR21	314	2	12364565	12398203	loss	15090	609757	101614625	Creatine_kinase_level	region1inRegion2	33638
CNVR22	316	2	12689951	12700771	loss	15090	609757	101614625	Creatine_kinase_level	region1inRegion2	10820
CNVR23	319	2	14000848	14032642	loss-gain	15090	609757	101614625	Creatine_kinase_level	region1inRegion2	31794
CNVR24	320	2	14728311	14771718	gain	15090	609757	101614625	Creatine_kinase_level	region1inRegion2	43407
CNVR25	321	2	15024071	15060448	loss	15090	609757	101614625	Creatine_kinase_level	region1inRegion2	36377
CNVR26	343	2	44800086	44803691	gain	15090	609757	101614625	Creatine_kinase_level	region1inRegion2	3605
CNVR27	348	2	54685639	54717573	loss-gain	15090	609757	101614625	Creatine_kinase_level	region1inRegion2	31934
CNVR28	353	2	56935884	56944870	loss	15090	609757	101614625	Creatine_kinase_level	region1inRegion2	8986
CNVR29	355	2	58030910	58035496	gain	15090	609757	101614625	Creatine_kinase_level	region1inRegion2	4586
CNVR30	359	2	61357153	61369311	loss	15090	609757	101614625	Creatine_kinase_level	region1inRegion2	12158
CNVR31	362	2	62416028	62428337	gain	15090	609757	101614625	Creatine_kinase_level	region1inRegion2	12309
CNVR32	364	2	62628253	62738661	gain	15090	609757	101614625	Creatine_kinase_level	region1inRegion2	110408
CNVR33	378	2	67915859	67952389	gain	15090	609757	101614625	Creatine_kinase_level	region1inRegion2	36530
CNVR34	379	2	68061173	68181801	gain	15090	609757	101614625	Creatine_kinase_level	region1inRegion2	120628
CNVR35	401	2	95897658	95915484	loss	15090	609757	101614625	Creatine_kinase_level	region1inRegion2	17826
CNVR36	408	2	96833054	96835454	loss	15090	609757	101614625	Creatine_kinase_level	region1inRegion2	2400
CNVR20	312	2	11511074	11530117	gain	5688	609757	133375944	Loin_muscle_area	region1inRegion2	19043
CNVR21	314	2	12364565	12398203	loss	5688	609757	133375944	Loin_muscle_area	region1inRegion2	33638
CNVR22	316	2	12689951	12700771	loss	5688	609757	133375944	Loin_muscle_area	region1inRegion2	10820
CNVR23	319	2	14000848	14032642	loss-gain	5688	609757	133375944	Loin_muscle_area	region1inRegion2	31794
CNVR24	320	2	14728311	14771718	gain	5688	609757	133375944	Loin_muscle_area	region1inRegion2	43407
CNVR25	321	2	15024071	15060448	loss	5688	609757	133375944	Loin_muscle_area	region1inRegion2	36377
CNVR26	343	2	44800086	44803691	gain	5688	609757	133375944	Loin_muscle_area	region1inRegion2	3605
CNVR27	348	2	54685639	54717573	loss-gain	5688	609757	133375944	Loin_muscle_area	region1inRegion2	31934
CNVR28	353	2	56935884	56944870	loss	5688	609757	133375944	Loin_muscle_area	region1inRegion2	8986
CNVR29	355	2	58030910	58035496	gain	5688	609757	133375944	Loin_muscle_area	region1inRegion2	4586
CNVR30	359	2	61357153	61369311	loss	5688	609757	133375944	Loin_muscle_area	region1inRegion2	12158
CNVR31	362	2	62416028	62428337	gain	5688	609757	133375944	Loin_muscle_area	region1inRegion2	12309
CNVR32	364	2	62628253	62738661	gain	5688	609757	133375944	Loin_muscle_area	region1inRegion2	110408
CNVR33	378	2	67915859	67952389	gain	5688	609757	133375944	Loin_muscle_area	region1inRegion2	36530
CNVR34	379	2	68061173	68181801	gain	5688	609757	133375944	Loin_muscle_area	region1inRegion2	120628
CNVR35	401	2	95897658	95915484	loss	5688	609757	133375944	Loin_muscle_area	region1inRegion2	17826
CNVR36	408	2	96833054	96835454	loss	5688	609757	133375944	Loin_muscle_area	region1inRegion2	2400
CNVR37	422	2	107437562	107451870	gain	5688	609757	133375944	Loin_muscle_area	region1inRegion2	14308
CNVR20	312	2	11511074	11530117	gain	37569	609757	152938765	Actinobacillus_pleuropneumoniae_susceptibility	region1inRegion2	19043
CNVR21	314	2	12364565	12398203	loss	37569	609757	152938765	Actinobacillus_pleuropneumoniae_susceptibility	region1inRegion2	33638
CNVR22	316	2	12689951	12700771	loss	37569	609757	152938765	Actinobacillus_pleuropneumoniae_susceptibility	region1inRegion2	10820
CNVR23	319	2	14000848	14032642	loss-gain	37569	609757	152938765	Actinobacillus_pleuropneumoniae_susceptibility	region1inRegion2	31794
CNVR24	320	2	14728311	14771718	gain	37569	609757	152938765	Actinobacillus_pleuropneumoniae_susceptibility	region1inRegion2	43407
CNVR25	321	2	15024071	15060448	loss	37569	609757	152938765	Actinobacillus_pleuropneumoniae_susceptibility	region1inRegion2	36377
CNVR26	343	2	44800086	44803691	gain	37569	609757	152938765	Actinobacillus_pleuropneumoniae_susceptibility	region1inRegion2	3605
CNVR27	348	2	54685639	54717573	loss-gain	37569	609757	152938765	Actinobacillus_pleuropneumoniae_susceptibility	region1inRegion2	31934
CNVR28	353	2	56935884	56944870	loss	37569	609757	152938765	Actinobacillus_pleuropneumoniae_susceptibility	region1inRegion2	8986
CNVR29	355	2	58030910	58035496	gain	37569	609757	152938765	Actinobacillus_pleuropneumoniae_susceptibility	region1inRegion2	4586
CNVR30	359	2	61357153	61369311	loss	37569	609757	152938765	Actinobacillus_pleuropneumoniae_susceptibility	region1inRegion2	12158
CNVR31	362	2	62416028	62428337	gain	37569	609757	152938765	Actinobacillus_pleuropneumoniae_susceptibility	region1inRegion2	12309
CNVR32	364	2	62628253	62738661	gain	37569	609757	152938765	Actinobacillus_pleuropneumoniae_susceptibility	region1inRegion2	110408
CNVR33	378	2	67915859	67952389	gain	37569	609757	152938765	Actinobacillus_pleuropneumoniae_susceptibility	region1inRegion2	36530
CNVR34	379	2	68061173	68181801	gain	37569	609757	152938765	Actinobacillus_pleuropneumoniae_susceptibility	region1inRegion2	120628
CNVR35	401	2	95897658	95915484	loss	37569	609757	152938765	Actinobacillus_pleuropneumoniae_susceptibility	region1inRegion2	17826
CNVR36	408	2	96833054	96835454	loss	37569	609757	152938765	Actinobacillus_pleuropneumoniae_susceptibility	region1inRegion2	2400
CNVR37	422	2	107437562	107451870	gain	37569	609757	152938765	Actinobacillus_pleuropneumoniae_susceptibility	region1inRegion2	14308
CNVR38	466	2	148904898	148913506	loss-gain	37569	609757	152938765	Actinobacillus_pleuropneumoniae_susceptibility	region1inRegion2	8608
CNVR39	467	2	149095217	149101135	loss	37569	609757	152938765	Actinobacillus_pleuropneumoniae_susceptibility	region1inRegion2	5918
CNVR20	312	2	11511074	11530117	gain	5689	609757	154953107	Fat_to_meat_ratio	region1inRegion2	19043
CNVR21	314	2	12364565	12398203	loss	5689	609757	154953107	Fat_to_meat_ratio	region1inRegion2	33638
CNVR22	316	2	12689951	12700771	loss	5689	609757	154953107	Fat_to_meat_ratio	region1inRegion2	10820
CNVR23	319	2	14000848	14032642	loss-gain	5689	609757	154953107	Fat_to_meat_ratio	region1inRegion2	31794
CNVR24	320	2	14728311	14771718	gain	5689	609757	154953107	Fat_to_meat_ratio	region1inRegion2	43407
CNVR25	321	2	15024071	15060448	loss	5689	609757	154953107	Fat_to_meat_ratio	region1inRegion2	36377
CNVR26	343	2	44800086	44803691	gain	5689	609757	154953107	Fat_to_meat_ratio	region1inRegion2	3605
CNVR27	348	2	54685639	54717573	loss-gain	5689	609757	154953107	Fat_to_meat_ratio	region1inRegion2	31934
CNVR28	353	2	56935884	56944870	loss	5689	609757	154953107	Fat_to_meat_ratio	region1inRegion2	8986
CNVR29	355	2	58030910	58035496	gain	5689	609757	154953107	Fat_to_meat_ratio	region1inRegion2	4586
CNVR30	359	2	61357153	61369311	loss	5689	609757	154953107	Fat_to_meat_ratio	region1inRegion2	12158
CNVR31	362	2	62416028	62428337	gain	5689	609757	154953107	Fat_to_meat_ratio	region1inRegion2	12309
CNVR32	364	2	62628253	62738661	gain	5689	609757	154953107	Fat_to_meat_ratio	region1inRegion2	110408
CNVR33	378	2	67915859	67952389	gain	5689	609757	154953107	Fat_to_meat_ratio	region1inRegion2	36530
CNVR34	379	2	68061173	68181801	gain	5689	609757	154953107	Fat_to_meat_ratio	region1inRegion2	120628
CNVR35	401	2	95897658	95915484	loss	5689	609757	154953107	Fat_to_meat_ratio	region1inRegion2	17826
CNVR36	408	2	96833054	96835454	loss	5689	609757	154953107	Fat_to_meat_ratio	region1inRegion2	2400
CNVR37	422	2	107437562	107451870	gain	5689	609757	154953107	Fat_to_meat_ratio	region1inRegion2	14308
CNVR38	466	2	148904898	148913506	loss-gain	5689	609757	154953107	Fat_to_meat_ratio	region1inRegion2	8608
CNVR39	467	2	149095217	149101135	loss	5689	609757	154953107	Fat_to_meat_ratio	region1inRegion2	5918
CNVR20	312	2	11511074	11530117	gain	5432	609757	157789611	Carcass_weight_(hot)	region1inRegion2	19043
CNVR21	314	2	12364565	12398203	loss	5432	609757	157789611	Carcass_weight_(hot)	region1inRegion2	33638

CNVR22	316	2	12689951	12700771	loss	5432	609757	157789611	Carcass_weight_(hot)	region1inRegion2	10820
CNVR23	319	2	14000848	14032642	loss-gain	5432	609757	157789611	Carcass_weight_(hot)	region1inRegion2	31794
CNVR24	320	2	14728311	14771718	gain	5432	609757	157789611	Carcass_weight_(hot)	region1inRegion2	43407
CNVR25	321	2	15024071	15060448	loss	5432	609757	157789611	Carcass_weight_(hot)	region1inRegion2	36377
CNVR26	343	2	44800086	44803691	gain	5432	609757	157789611	Carcass_weight_(hot)	region1inRegion2	3605
CNVR27	348	2	54685639	54717573	loss-gain	5432	609757	157789611	Carcass_weight_(hot)	region1inRegion2	31934
CNVR28	353	2	56935884	56944870	loss	5432	609757	157789611	Carcass_weight_(hot)	region1inRegion2	8986
CNVR29	355	2	58030910	58035496	gain	5432	609757	157789611	Carcass_weight_(hot)	region1inRegion2	4586
CNVR30	359	2	61357153	61369311	loss	5432	609757	157789611	Carcass_weight_(hot)	region1inRegion2	12158
CNVR31	362	2	62416028	62428337	gain	5432	609757	157789611	Carcass_weight_(hot)	region1inRegion2	12309
CNVR32	364	2	62628253	62738661	gain	5432	609757	157789611	Carcass_weight_(hot)	region1inRegion2	110408
CNVR33	378	2	67915859	67952389	gain	5432	609757	157789611	Carcass_weight_(hot)	region1inRegion2	36530
CNVR34	379	2	68061173	68181801	gain	5432	609757	157789611	Carcass_weight_(hot)	region1inRegion2	120628
CNVR35	401	2	95897658	95915484	loss	5432	609757	157789611	Carcass_weight_(hot)	region1inRegion2	17826
CNVR36	408	2	96833054	96835454	loss	5432	609757	157789611	Carcass_weight_(hot)	region1inRegion2	2400
CNVR37	422	2	107437562	107451870	gain	5432	609757	157789611	Carcass_weight_(hot)	region1inRegion2	14308
CNVR38	466	2	148904898	148913506	loss-gain	5432	609757	157789611	Carcass_weight_(hot)	region1inRegion2	8608
CNVR39	467	2	149095217	149101135	loss	5432	609757	157789611	Carcass_weight_(hot)	region1inRegion2	5918
CNVR40	471	2	156079099	156090392	gain	5432	609757	157789611	Carcass_weight_(hot)	region1inRegion2	11293
CNVR20	312	2	11511074	11530117	gain	5437	609757	157789611	Ham_weight	region1inRegion2	19043
CNVR21	314	2	12364565	12398203	loss	5437	609757	157789611	Ham_weight	region1inRegion2	33638
CNVR22	316	2	12689951	12700771	loss	5437	609757	157789611	Ham_weight	region1inRegion2	10820
CNVR23	319	2	14000848	14032642	loss-gain	5437	609757	157789611	Ham_weight	region1inRegion2	31794
CNVR24	320	2	14728311	14771718	gain	5437	609757	157789611	Ham_weight	region1inRegion2	43407
CNVR25	321	2	15024071	15060448	loss	5437	609757	157789611	Ham_weight	region1inRegion2	36377
CNVR26	343	2	44800086	44803691	gain	5437	609757	157789611	Ham_weight	region1inRegion2	3605
CNVR27	348	2	54685639	54717573	loss-gain	5437	609757	157789611	Ham_weight	region1inRegion2	31934
CNVR28	353	2	56935884	56944870	loss	5437	609757	157789611	Ham_weight	region1inRegion2	8986
CNVR29	355	2	58030910	58035496	gain	5437	609757	157789611	Ham_weight	region1inRegion2	4586
CNVR30	359	2	61357153	61369311	loss	5437	609757	157789611	Ham_weight	region1inRegion2	12158
CNVR31	362	2	62416028	62428337	gain	5437	609757	157789611	Ham_weight	region1inRegion2	12309
CNVR32	364	2	62628253	62738661	gain	5437	609757	157789611	Ham_weight	region1inRegion2	110408
CNVR33	378	2	67915859	67952389	gain	5437	609757	157789611	Ham_weight	region1inRegion2	36530
CNVR34	379	2	68061173	68181801	gain	5437	609757	157789611	Ham_weight	region1inRegion2	120628
CNVR35	401	2	95897658	95915484	loss	5437	609757	157789611	Ham_weight	region1inRegion2	17826
CNVR36	408	2	96833054	96835454	loss	5437	609757	157789611	Ham_weight	region1inRegion2	2400
CNVR37	422	2	107437562	107451870	gain	5437	609757	157789611	Ham_weight	region1inRegion2	14308
CNVR38	466	2	148904898	148913506	loss-gain	5437	609757	157789611	Ham_weight	region1inRegion2	8608
CNVR39	467	2	149095217	149101135	loss	5437	609757	157789611	Ham_weight	region1inRegion2	5918
CNVR40	471	2	156079099	156090392	gain	5437	609757	157789611	Ham_weight	region1inRegion2	11293
CNVR20	312	2	11511074	11530117	gain	5438	609757	157789611	Shoulder_weight	region1inRegion2	19043
CNVR21	314	2	12364565	12398203	loss	5438	609757	157789611	Shoulder_weight	region1inRegion2	33638
CNVR22	316	2	12689951	12700771	loss	5438	609757	157789611	Shoulder_weight	region1inRegion2	10820
CNVR23	319	2	14000848	14032642	loss-gain	5438	609757	157789611	Shoulder_weight	region1inRegion2	31794
CNVR24	320	2	14728311	14771718	gain	5438	609757	157789611	Shoulder_weight	region1inRegion2	43407
CNVR25	321	2	15024071	15060448	loss	5438	609757	157789611	Shoulder_weight	region1inRegion2	36377
CNVR26	343	2	44800086	44803691	gain	5438	609757	157789611	Shoulder_weight	region1inRegion2	3605
CNVR27	348	2	54685639	54717573	loss-gain	5438	609757	157789611	Shoulder_weight	region1inRegion2	31934
CNVR28	353	2	56935884	56944870	loss	5438	609757	157789611	Shoulder_weight	region1inRegion2	8986
CNVR29	355	2	58030910	58035496	gain	5438	609757	157789611	Shoulder_weight	region1inRegion2	4586
CNVR30	359	2	61357153	61369311	loss	5438	609757	157789611	Shoulder_weight	region1inRegion2	12158
CNVR31	362	2	62416028	62428337	gain	5438	609757	157789611	Shoulder_weight	region1inRegion2	12309
CNVR32	364	2	62628253	62738661	gain	5438	609757	157789611	Shoulder_weight	region1inRegion2	110408
CNVR33	378	2	67915859	67952389	gain	5438	609757	157789611	Shoulder_weight	region1inRegion2	36530
CNVR34	379	2	68061173	68181801	gain	5438	609757	157789611	Shoulder_weight	region1inRegion2	120628
CNVR35	401	2	95897658	95915484	loss	5438	609757	157789611	Shoulder_weight	region1inRegion2	17826
CNVR36	408	2	96833054	96835454	loss	5438	609757	157789611	Shoulder_weight	region1inRegion2	2400
CNVR37	422	2	107437562	107451870	gain	5438	609757	157789611	Shoulder_weight	region1inRegion2	14308
CNVR38	466	2	148904898	148913506	loss-gain	5438	609757	157789611	Shoulder_weight	region1inRegion2	8608
CNVR39	467	2	149095217	149101135	loss	5438	609757	157789611	Shoulder_weight	region1inRegion2	5918
CNVR40	471	2	156079099	156090392	gain	5438	609757	157789611	Shoulder_weight	region1inRegion2	11293
CNVR20	312	2	11511074	11530117	gain	5440	609757	157789611	Shear_firmness	region1inRegion2	19043
CNVR21	314	2	12364565	12398203	loss	5440	609757	157789611	Shear_firmness	region1inRegion2	33638
CNVR22	316	2	12689951	12700771	loss	5440	609757	157789611	Shear_firmness	region1inRegion2	10820
CNVR23	319	2	14000848	14032642	loss-gain	5440	609757	157789611	Shear_firmness	region1inRegion2	31794
CNVR24	320	2	14728311	14771718	gain	5440	609757	157789611	Shear_firmness	region1inRegion2	43407
CNVR25	321	2	15024071	15060448	loss	5440	609757	157789611	Shear_firmness	region1inRegion2	36377
CNVR26	343	2	44800086	44803691	gain	5440	609757	157789611	Shear_firmness	region1inRegion2	3605
CNVR27	348	2	54685639	54717573	loss-gain	5440	609757	157789611	Shear_firmness	region1inRegion2	31934
CNVR28	353	2	56935884	56944870	loss	5440	609757	157789611	Shear_firmness	region1inRegion2	8986
CNVR29	355	2	58030910	58035496	gain	5440	609757	157789611	Shear_firmness	region1inRegion2	4586
CNVR30	359	2	61357153	61369311	loss	5440	609757	157789611	Shear_firmness	region1inRegion2	12158
CNVR31	362	2	62416028	62428337	gain	5440	609757	157789611	Shear_firmness	region1inRegion2	12309
CNVR32	364	2	62628253	62738661	gain	5440	609757	157789611	Shear_firmness	region1inRegion2	110408
CNVR33	378	2	67915859	67952389	gain	5440	609757	157789611	Shear_firmness	region1inRegion2	36530
CNVR34	379	2	68061173	68181801	gain	5440	609757	157789611	Shear_firmness	region1inRegion2	120628
CNVR35	401	2	95897658	95915484	loss	5440	609757	157789611	Shear_firmness	region1inRegion2	17826
CNVR36	408	2	96833054	96835454	loss	5440	609757	157789611	Shear_firmness	region1inRegion2	2400
CNVR37	422	2	107437562	107451870	gain	5440	609757	157789611	Shear_firmness	region1inRegion2	14308
CNVR38	466	2	148904898	148913506	loss-gain	5440	609757	157789611	Shear_firmness	region1inRegion2	8608
CNVR39	467	2	149095217	149101135	loss	5440	609757	157789611	Shear_firmness	region1inRegion2	5918
CNVR40	471	2	156079099	156090392	gain	5440	609757	157789611	Shear_firmness	region1inRegion2	11293
CNVR20	312	2	11511074	11530117	gain	5496	609757	157789611	Cutlet_weight	region1inRegion2	19043
CNVR21	314	2	12364565	12398203	loss	5496	609757	157789611	Cutlet_weight	region1inRegion2	33638
CNVR22	316	2	12689951	12700771	loss	5496	609757	157789611	Cutlet_weight	region1inRegion2	10820
CNVR23	319	2	14000848	14032642	loss-gain	5496	609757	157789611	Cutlet_weight	region1inRegion2	31794
CNVR24	320	2	14728311	14771718	gain	5496	609757	157789611	Cutlet_weight	region1inRegion2	43407
CNVR25	321	2	15024071	15060448	loss	5496	609757	157789611	Cutlet_weight	region1inRegion2	36377
CNVR26	343	2	44800086	44803691	gain	5496	609757	157789611	Cutlet_weight	region1inRegion2	3605
CNVR27	348	2	54685639	54717573	loss-gain	5496	609757	157789611	Cutlet_weight	region1inRegion2	31934
CNVR28	353	2	56935884	56944870	loss	5496	609757	157789611	Cutlet_weight	region1inRegion2	8986
CNVR29	355	2	58030910	58035496	gain	5496	609757	157789611	Cutlet_weight	region1inRegion2	4586
CNVR30	359	2	61357153	61369311	loss	5496	609757	157789611	Cutlet_weight	region1inRegion2	12158
CNVR31	362	2	62416028	62428337	gain	5496	609757	157789611	Cutlet_weight	region1inRegion2	12309
CNVR32	364	2	62628253	62738661	gain	5496	609757	157789611	Cutlet_weight	region1inRegion2	110408
CNVR33	378	2	67915859	67952389	gain	5496	609757	157789611	Cutlet_weight	region1inRegion2	36530
CNVR34	379	2	68061173	68181801	gain	5496	609757	157789611	Cutlet_weight	region1inRegion2	120628
CNVR35	401	2	95897658	95915484	loss	5496	609757	157789611	Cutlet_weight	region1inRegion2	17826
CNVR36	408	2	96833054	96835454	loss	5496	609757	157789611	Cutlet_weight	region1inRegion2	2400
CNVR37	422	2	107437562	107451870	gain	5496	609757	157789611	Cutlet_weight	region1inRegion2	14308
CNVR38	466	2	148904898	148913506	loss-gain	5496	609757	157789611	Cutlet_weight	region1inRegion2	8608
CNVR39	467	2	149095217	149101135	loss	5496	609757	157789611	Cutlet_weight	region1inRegion2	5918
CNVR40	471	2	156079099	156090392	gain	5496	609757	157789611	Cutlet_weight	region1inRegion2	11293
CNVR20	312	2	11511074	11530117	gain	9612	609757	162569375	C3c_concentration	region1inRegion2	19043
CNVR21	314	2	12364565	12398203	loss	9612	609757	162569375	C3c_concentration	region1inRegion2	33638
CNVR22	316	2	12689951	12700771	loss	9612	609757	162569375	C3c_concentration	region1inRegion2	10820
CNVR23	319	2	14000848	14032642	loss-gain	9612	609757	162569375	C3c_concentration	region1inRegion2	31794
CNVR24	320	2	14728311	14771718	gain	9612	609757	162569375	C3c_concentration	region1inRegion2	43407
CNVR25	321	2	15024071	15060448	loss	9612	609757	162569375	C3c_concentration	region1inRegion2	36377
CNVR26	343	2	44800086	44803691	gain	9612	609757	162569375	C3c_concentration	region1inRegion2	3605
CNVR27	348	2	54685639	54717573	loss-gain	9612	609757	162569375	C3c_concentration	region1inRegion2	31934
CNVR28	353	2	56935884	56944870	loss	9612	609757	162569375	C3c_concentration	region1inRegion2	8986
CNVR29	355	2	58030910	58035496	gain	9612	609757	162569375	C3c_concentration	region1inRegion2	4586
CNVR30	359	2	61357153	61369311	loss	9612	609757	162569375	C3c_concentration	region1inRegion2	12158
CNVR31	362	2	62416028	62428337	gain	9612	609757	162569375	C3c_concentration	region1inRegion2	12309
CNVR32	364	2	62628253	62738661	gain	9612	609757	162569375	C3c_concentration	region1inRegion2	110408
CNVR33	378	2	67915859	67952389	gain	9612	609757	162569375	C3c_concentration	region1inRegion2	36530
CNVR34	379	2	68061173	68181801	gain	9612	609757	162569375	C3c_concentration	region1inRegion2	120628
CNVR35	401	2	95897658	95915484	loss	9612	609757	162569375	C3c_concentration	region1inRegion2	17826
CNVR36	408	2	96833054	96835454	loss	9612	609757	162569375	C3c_concentration	region1inRegion2	2400
CNVR37	422	2	107437562	107451870	gain	9612	609757	162569375	C3c_concentration	region1inRegion2	14308
CNVR38	466	2	148904898	148913506	loss-gain	9612	609757	162569375	C3c_concentration	region1inRegion2	8608

CNVR39	467	2	149095217	149101135	loss	9612	609757	162569375	C3c_concentration	region1inRegion2	5918
CNVR40	471	2	156079099	156090392	gain	9612	609757	162569375	C3c_concentration	region1inRegion2	11293
CNVR41	472	2	158544152	158558867	gain	9612	609757	162569375	C3c_concentration	region1inRegion2	14715
CNVR42	473	2	158605784	158612295	gain	9612	609757	162569375	C3c_concentration	region1inRegion2	6511
CNVR42	473	2	158605784	158629757	gain	9612	609757	162569375	C3c_concentration	region1inRegion2	23973
CNVR44	479	2	159094998	159110351	gain	9612	609757	162569375	C3c_concentration	region1inRegion2	15353
CNVR45	487	2	160893564	160895760	gain	9612	609757	162569375	C3c_concentration	region1inRegion2	2196
CNVR20	312	2	11511074	11530117	gain	9613	609757	162569375	C3c_concentration	region1inRegion2	19043
CNVR21	314	2	12364565	12398203	loss	9613	609757	162569375	C3c_concentration	region1inRegion2	33638
CNVR22	316	2	12689951	12700771	loss	9613	609757	162569375	C3c_concentration	region1inRegion2	10820
CNVR23	319	2	14000848	14032642	loss-gain	9613	609757	162569375	C3c_concentration	region1inRegion2	31794
CNVR24	320	2	14728311	14771718	gain	9613	609757	162569375	C3c_concentration	region1inRegion2	43407
CNVR25	321	2	15024071	15060448	loss	9613	609757	162569375	C3c_concentration	region1inRegion2	36377
CNVR26	343	2	44800086	44803691	gain	9613	609757	162569375	C3c_concentration	region1inRegion2	3605
CNVR27	348	2	54685639	54717573	loss-gain	9613	609757	162569375	C3c_concentration	region1inRegion2	31934
CNVR28	353	2	56935884	56944870	loss	9613	609757	162569375	C3c_concentration	region1inRegion2	8986
CNVR29	355	2	58030910	58035496	gain	9613	609757	162569375	C3c_concentration	region1inRegion2	4586
CNVR30	359	2	61357153	61369311	loss	9613	609757	162569375	C3c_concentration	region1inRegion2	12158
CNVR31	362	2	62416028	62428337	gain	9613	609757	162569375	C3c_concentration	region1inRegion2	12309
CNVR32	364	2	62628253	62738661	gain	9613	609757	162569375	C3c_concentration	region1inRegion2	110408
CNVR33	378	2	67915859	67952389	gain	9613	609757	162569375	C3c_concentration	region1inRegion2	36530
CNVR34	379	2	68061173	68181801	gain	9613	609757	162569375	C3c_concentration	region1inRegion2	120628
CNVR35	401	2	95897658	95915484	loss	9613	609757	162569375	C3c_concentration	region1inRegion2	17826
CNVR36	408	2	96833054	96835454	loss	9613	609757	162569375	C3c_concentration	region1inRegion2	2400
CNVR37	422	2	107437562	107451870	gain	9613	609757	162569375	C3c_concentration	region1inRegion2	14308
CNVR38	466	2	148904898	148913506	loss-gain	9613	609757	162569375	C3c_concentration	region1inRegion2	8608
CNVR39	467	2	149095217	149101135	loss	9613	609757	162569375	C3c_concentration	region1inRegion2	5918
CNVR40	471	2	156079099	156090392	gain	9613	609757	162569375	C3c_concentration	region1inRegion2	11293
CNVR41	472	2	158544152	158558867	gain	9613	609757	162569375	C3c_concentration	region1inRegion2	14715
CNVR42	473	2	158605784	158612295	gain	9613	609757	162569375	C3c_concentration	region1inRegion2	6511
CNVR42	473	2	158605784	158629757	gain	9613	609757	162569375	C3c_concentration	region1inRegion2	23973
CNVR44	479	2	159094998	159110351	gain	9613	609757	162569375	C3c_concentration	region1inRegion2	15353
CNVR45	487	2	160893564	160895760	gain	9613	609757	162569375	C3c_concentration	region1inRegion2	2196
CNVR20	312	2	11511074	11530117	gain	9614	609757	162569375	C3c_concentration	region1inRegion2	19043
CNVR21	314	2	12364565	12398203	loss	9614	609757	162569375	C3c_concentration	region1inRegion2	33638
CNVR22	316	2	12689951	12700771	loss	9614	609757	162569375	C3c_concentration	region1inRegion2	10820
CNVR23	319	2	14000848	14032642	loss-gain	9614	609757	162569375	C3c_concentration	region1inRegion2	31794
CNVR24	320	2	14728311	14771718	gain	9614	609757	162569375	C3c_concentration	region1inRegion2	43407
CNVR25	321	2	15024071	15060448	loss	9614	609757	162569375	C3c_concentration	region1inRegion2	36377
CNVR26	343	2	44800086	44803691	gain	9614	609757	162569375	C3c_concentration	region1inRegion2	3605
CNVR27	348	2	54685639	54717573	loss-gain	9614	609757	162569375	C3c_concentration	region1inRegion2	31934
CNVR28	353	2	56935884	56944870	loss	9614	609757	162569375	C3c_concentration	region1inRegion2	8986
CNVR29	355	2	58030910	58035496	gain	9614	609757	162569375	C3c_concentration	region1inRegion2	4586
CNVR30	359	2	61357153	61369311	loss	9614	609757	162569375	C3c_concentration	region1inRegion2	12158
CNVR31	362	2	62416028	62428337	gain	9614	609757	162569375	C3c_concentration	region1inRegion2	12309
CNVR32	364	2	62628253	62738661	gain	9614	609757	162569375	C3c_concentration	region1inRegion2	110408
CNVR33	378	2	67915859	67952389	gain	9614	609757	162569375	C3c_concentration	region1inRegion2	36530
CNVR34	379	2	68061173	68181801	gain	9614	609757	162569375	C3c_concentration	region1inRegion2	120628
CNVR35	401	2	95897658	95915484	loss	9614	609757	162569375	C3c_concentration	region1inRegion2	17826
CNVR36	408	2	96833054	96835454	loss	9614	609757	162569375	C3c_concentration	region1inRegion2	2400
CNVR37	422	2	107437562	107451870	gain	9614	609757	162569375	C3c_concentration	region1inRegion2	14308
CNVR38	466	2	148904898	148913506	loss-gain	9614	609757	162569375	C3c_concentration	region1inRegion2	8608
CNVR39	467	2	149095217	149101135	loss	9614	609757	162569375	C3c_concentration	region1inRegion2	5918
CNVR40	471	2	156079099	156090392	gain	9614	609757	162569375	C3c_concentration	region1inRegion2	11293
CNVR41	472	2	158544152	158558867	gain	9614	609757	162569375	C3c_concentration	region1inRegion2	14715
CNVR42	473	2	158605784	158612295	gain	9614	609757	162569375	C3c_concentration	region1inRegion2	6511
CNVR42	473	2	158605784	158629757	gain	9614	609757	162569375	C3c_concentration	region1inRegion2	23973
CNVR44	479	2	159094998	159110351	gain	9614	609757	162569375	C3c_concentration	region1inRegion2	15353
CNVR45	487	2	160893564	160895760	gain	9614	609757	162569375	C3c_concentration	region1inRegion2	2196
CNVR20	312	2	11511074	11530117	gain	12334	2387169	13366532	Interferon-gamma_level	region1inRegion2	19043
CNVR21	314	2	12364565	12398203	loss	12334	2387169	13366532	Interferon-gamma_level	region1inRegion2	33638
CNVR22	316	2	12689951	12700771	loss	12334	2387169	13366532	Interferon-gamma_level	region1inRegion2	10820
CNVR20	312	2	11511074	11530117	gain	5683	2387169	13366532	Drip_loss	region1inRegion2	19043
CNVR21	314	2	12364565	12398203	loss	5683	2387169	13366532	Drip_loss	region1inRegion2	33638
CNVR22	316	2	12689951	12700771	loss	5683	2387169	13366532	Drip_loss	region1inRegion2	10820
CNVR20	312	2	11511074	11530117	gain	5687	2387169	13366532	Dressing_percentage	region1inRegion2	19043
CNVR21	314	2	12364565	12398203	loss	5687	2387169	13366532	Dressing_percentage	region1inRegion2	33638
CNVR22	316	2	12689951	12700771	loss	5687	2387169	13366532	Dressing_percentage	region1inRegion2	10820
CNVR20	312	2	11511074	11530117	gain	5934	2387169	13366532	Body_weight_(birth)	region1inRegion2	19043
CNVR21	314	2	12364565	12398203	loss	5934	2387169	13366532	Body_weight_(birth)	region1inRegion2	33638
CNVR22	316	2	12689951	12700771	loss	5934	2387169	13366532	Body_weight_(birth)	region1inRegion2	10820
CNVR20	312	2	11511074	11530117	gain	5937	2387169	13366532	Carcass_weight_(hot)	region1inRegion2	19043
CNVR21	314	2	12364565	12398203	loss	5937	2387169	13366532	Carcass_weight_(hot)	region1inRegion2	33638
CNVR22	316	2	12689951	12700771	loss	5937	2387169	13366532	Carcass_weight_(hot)	region1inRegion2	10820
CNVR20	312	2	11511074	11530117	gain	5961	2387169	13366532	Shoulder_subcutaneous_fat_thickness	region1inRegion2	19043
CNVR21	314	2	12364565	12398203	loss	5961	2387169	13366532	Shoulder_subcutaneous_fat_thickness	region1inRegion2	33638
CNVR22	316	2	12689951	12700771	loss	5961	2387169	13366532	Shoulder_subcutaneous_fat_thickness	region1inRegion2	10820
CNVR20	312	2	11511074	11530117	gain	5966	2387169	13366532	backfat_at_last_rib	region1inRegion2	19043
CNVR21	314	2	12364565	12398203	loss	5966	2387169	13366532	backfat_at_last_rib	region1inRegion2	33638
CNVR22	316	2	12689951	12700771	loss	5966	2387169	13366532	backfat_at_last_rib	region1inRegion2	10820
CNVR20	312	2	11511074	11530117	gain	5967	2387169	13366532	Side_fat_thickness	region1inRegion2	19043
CNVR21	314	2	12364565	12398203	loss	5967	2387169	13366532	Side_fat_thickness	region1inRegion2	33638
CNVR22	316	2	12689951	12700771	loss	5967	2387169	13366532	Side_fat_thickness	region1inRegion2	10820
CNVR20	312	2	11511074	11530117	gain	5968	2387169	13366532	Loin_muscle_area	region1inRegion2	19043
CNVR21	314	2	12364565	12398203	loss	5968	2387169	13366532	Loin_muscle_area	region1inRegion2	33638
CNVR22	316	2	12689951	12700771	loss	5968	2387169	13366532	Loin_muscle_area	region1inRegion2	10820
CNVR20	312	2	11511074	11530117	gain	5969	2387169	13366532	Subcutanous_fat_area	region1inRegion2	19043
CNVR21	314	2	12364565	12398203	loss	5969	2387169	13366532	Subcutanous_fat_area	region1inRegion2	33638
CNVR22	316	2	12689951	12700771	loss	5969	2387169	13366532	Subcutanous_fat_area	region1inRegion2	10820
CNVR20	312	2	11511074	11530117	gain	5970	2387169	13366532	Fat_to_meat_ratio	region1inRegion2	19043
CNVR21	314	2	12364565	12398203	loss	5970	2387169	13366532	Fat_to_meat_ratio	region1inRegion2	33638
CNVR22	316	2	12689951	12700771	loss	5970	2387169	13366532	Fat_to_meat_ratio	region1inRegion2	10820
CNVR20	312	2	11511074	11530117	gain	5971	2387169	13366532	Belly_meat_content	region1inRegion2	19043
CNVR21	314	2	12364565	12398203	loss	5971	2387169	13366532	Belly_meat_content	region1inRegion2	33638
CNVR22	316	2	12689951	12700771	loss	5971	2387169	13366532	Belly_meat_content	region1inRegion2	10820
CNVR20	312	2	11511074	11530117	gain	5972	2387169	13366532	Estimated_carcass_lean_content	region1inRegion2	19043
CNVR21	314	2	12364565	12398203	loss	5972	2387169	13366532	Estimated_carcass_lean_content	region1inRegion2	33638
CNVR22	316	2	12689951	12700771	loss	5972	2387169	13366532	Estimated_carcass_lean_content	region1inRegion2	10820
CNVR20	312	2	11511074	11530117	gain	910	2387169	13366532	Loin_and_neck_meat_weight	region1inRegion2	19043
CNVR21	314	2	12364565	12398203	loss	910	2387169	13366532	Loin_and_neck_meat_weight	region1inRegion2	33638
CNVR22	316	2	12689951	12700771	loss	910	2387169	13366532	Loin_and_neck_meat_weight	region1inRegion2	10820
CNVR20	312	2	11511074	11530117	gain	916	2387169	13366532	Loin_and_neck_meat_weight	region1inRegion2	19043
CNVR21	314	2	12364565	12398203	loss	916	2387169	13366532	Loin_and_neck_meat_weight	region1inRegion2	33638
CNVR22	316	2	12689951	12700771	loss	916	2387169	13366532	Loin_and_neck_meat_weight	region1inRegion2	10820
CNVR20	312	2	11511074	11530117	gain	918	2387169	13366532	Shoulder_meat_weight	region1inRegion2	19043
CNVR21	314	2	12364565	12398203	loss	918	2387169	13366532	Shoulder_meat_weight	region1inRegion2	33638
CNVR22	316	2	12689951	12700771	loss	918	2387169	13366532	Shoulder_meat_weight	region1inRegion2	10820
CNVR20	312	2	11511074	11530117	gain	922	2387169	13366532	Loin_and_neck_meat_weight	region1inRegion2	19043
CNVR21	314	2	12364565	12398203	loss	922	2387169	13366532	Loin_and_neck_meat_weight	region1inRegion2	33638
CNVR22	316	2	12689951	12700771	loss	922	2387169	13366532	Loin_and_neck_meat_weight	region1inRegion2	10820
CNVR20	312	2	11511074	11530117	gain	923	2387169	13366532	Average_backfat_thickness	region1inRegion2	19043
CNVR21	314	2	12364565	12398203	loss	923	2387169	13366532	Average_backfat_thickness	region1inRegion2	33638
CNVR22	316	2	12689951	12700771	loss	923	2387169	13366532	Average_backfat_thickness	region1inRegion2	10820
CNVR20	312	2	11511074	11530117	gain	924	2387169	13366532	Carcass_length	region1inRegion2	19043
CNVR21	314	2	12364565	12398203	loss	924	2387169	13366532	Carcass_length	region1inRegion2	33638
CNVR22	316	2	12689951	12700771	loss	924	2387169	13366532	Carcass_length	region1inRegion2	10820
CNVR20	312	2	11511074	11530117	gain	928	2387169	13366532	Body_weight_(3_weeks)	region1inRegion2	19043
CNVR21	314	2	12364565	12398203	loss	928	2387169	13366532	Body_weight_(3_weeks)	region1inRegion2	33638
CNVR22	316	2	12689951	12700771	loss	928	2387169	13366532	Body_weight_(3_weeks)	region1inRegion2	10820
CNVR20	312	2	11511074	11530117	gain	930	2387169	13366532	Dressing_percentage	region1inRegion2	19043
CNVR21	314	2	12364565	12398203	loss	930	2387169	13366532	Dressing_percentage	region1inRegion2	33638
CNVR22	316	2	12689951	12700771	loss	930	2387169	13366532	Dressing_percentage	region1inRegion2	10820

CNVR20	312	2	11511074	11530117	gain	38051	2387169	21136167	Loin_muscle_area	region1inRegion2	19043
CNVR21	314	2	12364565	12398203	loss	38051	2387169	21136167	Loin_muscle_area	region1inRegion2	33638
CNVR22	316	2	12689951	12700771	loss	38051	2387169	21136167	Loin_muscle_area	region1inRegion2	10820
CNVR23	319	2	14000848	14032642	loss-gain	38051	2387169	21136167	Loin_muscle_area	region1inRegion2	31794
CNVR24	320	2	14728311	14771718	gain	38051	2387169	21136167	Loin_muscle_area	region1inRegion2	43407
CNVR25	321	2	15024071	15060448	loss	38051	2387169	21136167	Loin_muscle_area	region1inRegion2	36377
CNVR20	312	2	11511074	11530117	gain	914	2387169	21136167	Ham_weight	region1inRegion2	19043
CNVR21	314	2	12364565	12398203	loss	914	2387169	21136167	Ham_weight	region1inRegion2	33638
CNVR22	316	2	12689951	12700771	loss	914	2387169	21136167	Ham_weight	region1inRegion2	10820
CNVR23	319	2	14000848	14032642	loss-gain	914	2387169	21136167	Ham_weight	region1inRegion2	31794
CNVR24	320	2	14728311	14771718	gain	914	2387169	21136167	Ham_weight	region1inRegion2	43407
CNVR25	321	2	15024071	15060448	loss	914	2387169	21136167	Ham_weight	region1inRegion2	36377
CNVR20	312	2	11511074	11530117	gain	929	2387169	21136167	Average_daily_gain	region1inRegion2	19043
CNVR21	314	2	12364565	12398203	loss	929	2387169	21136167	Average_daily_gain	region1inRegion2	33638
CNVR22	316	2	12689951	12700771	loss	929	2387169	21136167	Average_daily_gain	region1inRegion2	10820
CNVR23	319	2	14000848	14032642	loss-gain	929	2387169	21136167	Average_daily_gain	region1inRegion2	31794
CNVR24	320	2	14728311	14771718	gain	929	2387169	21136167	Average_daily_gain	region1inRegion2	43407
CNVR25	321	2	15024071	15060448	loss	929	2387169	21136167	Average_daily_gain	region1inRegion2	36377
CNVR20	312	2	11511074	11530117	gain	5965	2387169	38367026	Average_backfat_thickness	region1inRegion2	19043
CNVR21	314	2	12364565	12398203	loss	5965	2387169	38367026	Average_backfat_thickness	region1inRegion2	33638
CNVR22	316	2	12689951	12700771	loss	5965	2387169	38367026	Average_backfat_thickness	region1inRegion2	10820
CNVR23	319	2	14000848	14032642	loss-gain	5965	2387169	38367026	Average_backfat_thickness	region1inRegion2	31794
CNVR24	320	2	14728311	14771718	gain	5965	2387169	38367026	Average_backfat_thickness	region1inRegion2	43407
CNVR25	321	2	15024071	15060448	loss	5965	2387169	38367026	Average_backfat_thickness	region1inRegion2	36377
CNVR20	312	2	11511074	11530117	gain	7576	2387169	133160620	Melanoma_susceptibility	region1inRegion2	19043
CNVR21	314	2	12364565	12398203	loss	7576	2387169	133160620	Melanoma_susceptibility	region1inRegion2	33638
CNVR22	316	2	12689951	12700771	loss	7576	2387169	133160620	Melanoma_susceptibility	region1inRegion2	10820
CNVR23	319	2	14000848	14032642	loss-gain	7576	2387169	133160620	Melanoma_susceptibility	region1inRegion2	31794
CNVR24	320	2	14728311	14771718	gain	7576	2387169	133160620	Melanoma_susceptibility	region1inRegion2	43407
CNVR25	321	2	15024071	15060448	loss	7576	2387169	133160620	Melanoma_susceptibility	region1inRegion2	36377
CNVR26	343	2	44800086	44803691	gain	7576	2387169	133160620	Melanoma_susceptibility	region1inRegion2	3605
CNVR27	348	2	54685639	54717573	loss-gain	7576	2387169	133160620	Melanoma_susceptibility	region1inRegion2	31934
CNVR28	353	2	56935884	56944870	loss	7576	2387169	133160620	Melanoma_susceptibility	region1inRegion2	8986
CNVR29	355	2	58030910	58035496	gain	7576	2387169	133160620	Melanoma_susceptibility	region1inRegion2	4586
CNVR30	359	2	61357153	61369311	loss	7576	2387169	133160620	Melanoma_susceptibility	region1inRegion2	12158
CNVR31	362	2	62416028	62428337	gain	7576	2387169	133160620	Melanoma_susceptibility	region1inRegion2	12309
CNVR32	364	2	62628253	62738661	gain	7576	2387169	133160620	Melanoma_susceptibility	region1inRegion2	110408
CNVR33	378	2	67915859	67952389	gain	7576	2387169	133160620	Melanoma_susceptibility	region1inRegion2	36530
CNVR34	379	2	68061173	68181801	gain	7576	2387169	133160620	Melanoma_susceptibility	region1inRegion2	120628
CNVR35	401	2	95897658	95915484	loss	7576	2387169	133160620	Melanoma_susceptibility	region1inRegion2	17826
CNVR36	408	2	96833054	96835454	loss	7576	2387169	133160620	Melanoma_susceptibility	region1inRegion2	2400
CNVR37	422	2	107437562	107451870	gain	7576	2387169	133160620	Melanoma_susceptibility	region1inRegion2	14308
CNVR20	312	2	11511074	11530117	gain	38074	2387169	139359663	Shear_force	region1inRegion2	19043
CNVR21	314	2	12364565	12398203	loss	38074	2387169	139359663	Shear_force	region1inRegion2	33638
CNVR22	316	2	12689951	12700771	loss	38074	2387169	139359663	Shear_force	region1inRegion2	10820
CNVR23	319	2	14000848	14032642	loss-gain	38074	2387169	139359663	Shear_force	region1inRegion2	31794
CNVR24	320	2	14728311	14771718	gain	38074	2387169	139359663	Shear_force	region1inRegion2	43407
CNVR25	321	2	15024071	15060448	loss	38074	2387169	139359663	Shear_force	region1inRegion2	36377
CNVR26	343	2	44800086	44803691	gain	38074	2387169	139359663	Shear_force	region1inRegion2	3605
CNVR27	348	2	54685639	54717573	loss-gain	38074	2387169	139359663	Shear_force	region1inRegion2	31934
CNVR28	353	2	56935884	56944870	loss	38074	2387169	139359663	Shear_force	region1inRegion2	8986
CNVR29	355	2	58030910	58035496	gain	38074	2387169	139359663	Shear_force	region1inRegion2	4586
CNVR30	359	2	61357153	61369311	loss	38074	2387169	139359663	Shear_force	region1inRegion2	12158
CNVR31	362	2	62416028	62428337	gain	38074	2387169	139359663	Shear_force	region1inRegion2	12309
CNVR32	364	2	62628253	62738661	gain	38074	2387169	139359663	Shear_force	region1inRegion2	110408
CNVR33	378	2	67915859	67952389	gain	38074	2387169	139359663	Shear_force	region1inRegion2	36530
CNVR34	379	2	68061173	68181801	gain	38074	2387169	139359663	Shear_force	region1inRegion2	120628
CNVR35	401	2	95897658	95915484	loss	38074	2387169	139359663	Shear_force	region1inRegion2	17826
CNVR36	408	2	96833054	96835454	loss	38074	2387169	139359663	Shear_force	region1inRegion2	2400
CNVR37	422	2	107437562	107451870	gain	38074	2387169	139359663	Shear_force	region1inRegion2	14308
CNVR20	312	2	11511074	11530117	gain	38076	2387169	139359663	CIE-L*	region1inRegion2	19043
CNVR21	314	2	12364565	12398203	loss	38076	2387169	139359663	CIE-L*	region1inRegion2	33638
CNVR22	316	2	12689951	12700771	loss	38076	2387169	139359663	CIE-L*	region1inRegion2	10820
CNVR23	319	2	14000848	14032642	loss-gain	38076	2387169	139359663	CIE-L*	region1inRegion2	31794
CNVR24	320	2	14728311	14771718	gain	38076	2387169	139359663	CIE-L*	region1inRegion2	43407
CNVR25	321	2	15024071	15060448	loss	38076	2387169	139359663	CIE-L*	region1inRegion2	36377
CNVR26	343	2	44800086	44803691	gain	38076	2387169	139359663	CIE-L*	region1inRegion2	3605
CNVR27	348	2	54685639	54717573	loss-gain	38076	2387169	139359663	CIE-L*	region1inRegion2	31934
CNVR28	353	2	56935884	56944870	loss	38076	2387169	139359663	CIE-L*	region1inRegion2	8986
CNVR29	355	2	58030910	58035496	gain	38076	2387169	139359663	CIE-L*	region1inRegion2	4586
CNVR30	359	2	61357153	61369311	loss	38076	2387169	139359663	CIE-L*	region1inRegion2	12158
CNVR31	362	2	62416028	62428337	gain	38076	2387169	139359663	CIE-L*	region1inRegion2	12309
CNVR32	364	2	62628253	62738661	gain	38076	2387169	139359663	CIE-L*	region1inRegion2	110408
CNVR33	378	2	67915859	67952389	gain	38076	2387169	139359663	CIE-L*	region1inRegion2	36530
CNVR34	379	2	68061173	68181801	gain	38076	2387169	139359663	CIE-L*	region1inRegion2	120628
CNVR35	401	2	95897658	95915484	loss	38076	2387169	139359663	CIE-L*	region1inRegion2	17826
CNVR36	408	2	96833054	96835454	loss	38076	2387169	139359663	CIE-L*	region1inRegion2	2400
CNVR37	422	2	107437562	107451870	gain	38076	2387169	139359663	CIE-L*	region1inRegion2	14308
CNVR20	312	2	11511074	11530117	gain	5690	2387169	154953107	Belly_meat_content	region1inRegion2	19043
CNVR21	314	2	12364565	12398203	loss	5690	2387169	154953107	Belly_meat_content	region1inRegion2	33638
CNVR22	316	2	12689951	12700771	loss	5690	2387169	154953107	Belly_meat_content	region1inRegion2	10820
CNVR23	319	2	14000848	14032642	loss-gain	5690	2387169	154953107	Belly_meat_content	region1inRegion2	31794
CNVR24	320	2	14728311	14771718	gain	5690	2387169	154953107	Belly_meat_content	region1inRegion2	43407
CNVR25	321	2	15024071	15060448	loss	5690	2387169	154953107	Belly_meat_content	region1inRegion2	36377
CNVR26	343	2	44800086	44803691	gain	5690	2387169	154953107	Belly_meat_content	region1inRegion2	3605
CNVR27	348	2	54685639	54717573	loss-gain	5690	2387169	154953107	Belly_meat_content	region1inRegion2	31934
CNVR28	353	2	56935884	56944870	loss	5690	2387169	154953107	Belly_meat_content	region1inRegion2	8986
CNVR29	355	2	58030910	58035496	gain	5690	2387169	154953107	Belly_meat_content	region1inRegion2	4586
CNVR30	359	2	61357153	61369311	loss	5690	2387169	154953107	Belly_meat_content	region1inRegion2	12158
CNVR31	362	2	62416028	62428337	gain	5690	2387169	154953107	Belly_meat_content	region1inRegion2	12309
CNVR32	364	2	62628253	62738661	gain	5690	2387169	154953107	Belly_meat_content	region1inRegion2	110408
CNVR33	378	2	67915859	67952389	gain	5690	2387169	154953107	Belly_meat_content	region1inRegion2	36530
CNVR34	379	2	68061173	68181801	gain	5690	2387169	154953107	Belly_meat_content	region1inRegion2	120628
CNVR35	401	2	95897658	95915484	loss	5690	2387169	154953107	Belly_meat_content	region1inRegion2	17826
CNVR36	408	2	96833054	96835454	loss	5690	2387169	154953107	Belly_meat_content	region1inRegion2	2400
CNVR37	422	2	107437562	107451870	gain	5690	2387169	154953107	Belly_meat_content	region1inRegion2	14308
CNVR38	466	2	148904898	148913506	loss-gain	5690	2387169	154953107	Belly_meat_content	region1inRegion2	8608
CNVR39	467	2	149095217	149101135	loss	5690	2387169	154953107	Belly_meat_content	region1inRegion2	5918
CNVR20	312	2	11511074	11530117	gain	5691	2387169	154953107	Estimated_carcass_lean_content	region1inRegion2	19043
CNVR21	314	2	12364565	12398203	loss	5691	2387169	154953107	Estimated_carcass_lean_content	region1inRegion2	33638
CNVR22	316	2	12689951	12700771	loss	5691	2387169	154953107	Estimated_carcass_lean_content	region1inRegion2	10820
CNVR23	319	2	14000848	14032642	loss-gain	5691	2387169	154953107	Estimated_carcass_lean_content	region1inRegion2	31794
CNVR24	320	2	14728311	14771718	gain	5691	2387169	154953107	Estimated_carcass_lean_content	region1inRegion2	43407
CNVR25	321	2	15024071	15060448	loss	5691	2387169	154953107	Estimated_carcass_lean_content	region1inRegion2	36377
CNVR26	343	2	44800086	44803691	gain	5691	2387169	154953107	Estimated_carcass_lean_content	region1inRegion2	3605
CNVR27	348	2	54685639	54717573	loss-gain	5691	2387169	154953107	Estimated_carcass_lean_content	region1inRegion2	31934
CNVR28	353	2	56935884	56944870	loss	5691	2387169	154953107	Estimated_carcass_lean_content	region1inRegion2	8986
CNVR29	355	2	58030910	58035496	gain	5691	2387169	154953107	Estimated_carcass_lean_content	region1inRegion2	4586
CNVR30	359	2	61357153	61369311	loss	5691	2387169	154953107	Estimated_carcass_lean_content	region1inRegion2	12158
CNVR31	362	2	62416028	62428337	gain	5691	2387169	154953107	Estimated_carcass_lean_content	region1inRegion2	12309
CNVR32	364	2	62628253	62738661	gain	5691	2387169	154953107	Estimated_carcass_lean_content	region1inRegion2	110408
CNVR33	378	2	67915859	67952389	gain	5691	2387169	154953107	Estimated_carcass_lean_content	region1inRegion2	36530
CNVR34	379	2	68061173	68181801	gain	5691	2387169	154953107	Estimated_carcass_lean_content	region1inRegion2	120628
CNVR35	401	2	95897658	95915484	loss	5691	2387169	154953107	Estimated_carcass_lean_content	region1inRegion2	17826
CNVR36	408	2	96833054	96835454	loss	5691	2387169	154953107	Estimated_carcass_lean_content	region1inRegion2	2400
CNVR37	422	2	107437562	107451870	gain	5691	2387169	154953107	Estimated_carcass_lean_content	region1inRegion2	14308
CNVR38	466	2	148904898	148913506	loss-gain	5691	2387169	154953107	Estimated_carcass_lean_content	region1inRegion2	8608
CNVR39	467	2	149095217	149101135	loss	5691	2387169	154953107	Estimated_carcass_lean_content	region1inRegion2	5918
CNVR20	312	2	11511074	11530117	gain	65002	2594130	132711982	Heart_weight	region1inRegion2	19043
CNVR21	314	2	12364565	12398203	loss	65002	2594130	132711982	Heart_weight	region1inRegion2	33638
CNVR22	316	2	12689951	12700771	loss	65002	2594130	132711982	Heart_weight	region1inRegion2	10820
CNVR23	319	2	14000848	14032642	loss-gain	65002	2594130	132711982	Heart_weight	region1inRegion2	31794

CNVR24	320	2	14728311	14771718	gain	65002	2594130	132711982	Heart_weight	region1inRegion2	43407
CNVR25	321	2	15024071	15060448	loss	65002	2594130	132711982	Heart_weight	region1inRegion2	36377
CNVR26	343	2	44800086	44803691	gain	65002	2594130	132711982	Heart_weight	region1inRegion2	3605
CNVR27	348	2	54685639	54717573	loss-gain	65002	2594130	132711982	Heart_weight	region1inRegion2	31934
CNVR28	353	2	56935884	56944870	loss	65002	2594130	132711982	Heart_weight	region1inRegion2	8986
CNVR29	355	2	58030910	58035496	gain	65002	2594130	132711982	Heart_weight	region1inRegion2	4586
CNVR30	359	2	61357153	61369311	loss	65002	2594130	132711982	Heart_weight	region1inRegion2	12158
CNVR31	362	2	62416028	62428337	gain	65002	2594130	132711982	Heart_weight	region1inRegion2	12309
CNVR32	364	2	62628253	62738661	gain	65002	2594130	132711982	Heart_weight	region1inRegion2	110408
CNVR33	378	2	67915859	67952389	gain	65002	2594130	132711982	Heart_weight	region1inRegion2	36530
CNVR34	379	2	68061173	68181801	gain	65002	2594130	132711982	Heart_weight	region1inRegion2	120628
CNVR35	401	2	95897658	95915484	loss	65002	2594130	132711982	Heart_weight	region1inRegion2	17826
CNVR36	408	2	96833054	96835454	loss	65002	2594130	132711982	Heart_weight	region1inRegion2	2400
CNVR37	422	2	107437562	107451870	gain	65002	2594130	132711982	Heart_weight	region1inRegion2	14308
CNVR20	312	2	11511074	11530117	gain	3942	2795608	27137109	Backfat_between_6th_and_7th_ribs	region1inRegion2	19043
CNVR21	314	2	12364565	12398203	loss	3942	2795608	27137109	Backfat_between_6th_and_7th_ribs	region1inRegion2	33638
CNVR22	316	2	12689951	12700771	loss	3942	2795608	27137109	Backfat_between_6th_and_7th_ribs	region1inRegion2	10820
CNVR23	319	2	14000848	14032642	loss-gain	3942	2795608	27137109	Backfat_between_6th_and_7th_ribs	region1inRegion2	31794
CNVR24	320	2	14728311	14771718	gain	3942	2795608	27137109	Backfat_between_6th_and_7th_ribs	region1inRegion2	43407
CNVR25	321	2	15024071	15060448	loss	3942	2795608	27137109	Backfat_between_6th_and_7th_ribs	region1inRegion2	36377
CNVR20	312	2	11511074	11530117	gain	18003	2948442	13366532	Subcutanous_fat_area	region1inRegion2	19043
CNVR21	314	2	12364565	12398203	loss	18003	2948442	13366532	Subcutanous_fat_area	region1inRegion2	33638
CNVR22	316	2	12689951	12700771	loss	18003	2948442	13366532	Subcutanous_fat_area	region1inRegion2	10820
CNVR20	312	2	11511074	11530117	gain	18010	2948442	13366532	Fat_protein_content	region1inRegion2	19043
CNVR21	314	2	12364565	12398203	loss	18010	2948442	13366532	Fat_protein_content	region1inRegion2	33638
CNVR22	316	2	12689951	12700771	loss	18010	2948442	13366532	Fat_protein_content	region1inRegion2	10820
CNVR20	312	2	11511074	11530117	gain	18016	2948442	13366532	NADP-malate_dehydrogenase_activity	region1inRegion2	19043
CNVR21	314	2	12364565	12398203	loss	18016	2948442	13366532	NADP-malate_dehydrogenase_activity	region1inRegion2	33638
CNVR22	316	2	12689951	12700771	loss	18016	2948442	13366532	NADP-malate_dehydrogenase_activity	region1inRegion2	10820
CNVR20	312	2	11511074	11530117	gain	311	2948442	13366532	Average_backfat_thickness	region1inRegion2	19043
CNVR21	314	2	12364565	12398203	loss	311	2948442	13366532	Average_backfat_thickness	region1inRegion2	33638
CNVR22	316	2	12689951	12700771	loss	311	2948442	13366532	Average_backfat_thickness	region1inRegion2	10820
CNVR20	312	2	11511074	11530117	gain	21217	2948442	15950969	Backfat_at_rump	region1inRegion2	19043
CNVR21	314	2	12364565	12398203	loss	21217	2948442	15950969	Backfat_at_rump	region1inRegion2	33638
CNVR22	316	2	12689951	12700771	loss	21217	2948442	15950969	Backfat_at_rump	region1inRegion2	10820
CNVR23	319	2	14000848	14032642	loss-gain	21217	2948442	15950969	Backfat_at_rump	region1inRegion2	31794
CNVR24	320	2	14728311	14771718	gain	21217	2948442	15950969	Backfat_at_rump	region1inRegion2	43407
CNVR25	321	2	15024071	15060448	loss	21217	2948442	15950969	Backfat_at_rump	region1inRegion2	36377
CNVR20	312	2	11511074	11530117	gain	21216	2948442	44995408	Backfat_at_first_rib	region1inRegion2	19043
CNVR21	314	2	12364565	12398203	loss	21216	2948442	44995408	Backfat_at_first_rib	region1inRegion2	33638
CNVR22	316	2	12689951	12700771	loss	21216	2948442	44995408	Backfat_at_first_rib	region1inRegion2	10820
CNVR23	319	2	14000848	14032642	loss-gain	21216	2948442	44995408	Backfat_at_first_rib	region1inRegion2	31794
CNVR24	320	2	14728311	14771718	gain	21216	2948442	44995408	Backfat_at_first_rib	region1inRegion2	43407
CNVR25	321	2	15024071	15060448	loss	21216	2948442	44995408	Backfat_at_first_rib	region1inRegion2	36377
CNVR26	343	2	44800086	44803691	gain	21216	2948442	44995408	Backfat_at_first_rib	region1inRegion2	3605
CNVR20	312	2	11511074	11530117	gain	21212	2948442	137276970	Average_backfat_thickness	region1inRegion2	19043
CNVR21	314	2	12364565	12398203	loss	21212	2948442	137276970	Average_backfat_thickness	region1inRegion2	33638
CNVR22	316	2	12689951	12700771	loss	21212	2948442	137276970	Average_backfat_thickness	region1inRegion2	10820
CNVR23	319	2	14000848	14032642	loss-gain	21212	2948442	137276970	Average_backfat_thickness	region1inRegion2	31794
CNVR24	320	2	14728311	14771718	gain	21212	2948442	137276970	Average_backfat_thickness	region1inRegion2	43407
CNVR25	321	2	15024071	15060448	loss	21212	2948442	137276970	Average_backfat_thickness	region1inRegion2	36377
CNVR26	343	2	44800086	44803691	gain	21212	2948442	137276970	Average_backfat_thickness	region1inRegion2	3605
CNVR27	348	2	54685639	54717573	loss-gain	21212	2948442	137276970	Average_backfat_thickness	region1inRegion2	31934
CNVR28	353	2	56935884	56944870	loss	21212	2948442	137276970	Average_backfat_thickness	region1inRegion2	8986
CNVR29	355	2	58030910	58035496	gain	21212	2948442	137276970	Average_backfat_thickness	region1inRegion2	4586
CNVR30	359	2	61357153	61369311	loss	21212	2948442	137276970	Average_backfat_thickness	region1inRegion2	12158
CNVR31	362	2	62416028	62428337	gain	21212	2948442	137276970	Average_backfat_thickness	region1inRegion2	12309
CNVR32	364	2	62628253	62738661	gain	21212	2948442	137276970	Average_backfat_thickness	region1inRegion2	110408
CNVR33	378	2	67915859	67952389	gain	21212	2948442	137276970	Average_backfat_thickness	region1inRegion2	36530
CNVR34	379	2	68061173	68181801	gain	21212	2948442	137276970	Average_backfat_thickness	region1inRegion2	120628
CNVR35	401	2	95897658	95915484	loss	21212	2948442	137276970	Average_backfat_thickness	region1inRegion2	17826
CNVR36	408	2	96833054	96835454	loss	21212	2948442	137276970	Average_backfat_thickness	region1inRegion2	2400
CNVR37	422	2	107437562	107451870	gain	21212	2948442	137276970	Average_backfat_thickness	region1inRegion2	14308
CNVR20	312	2	11511074	11530117	gain	21214	2948442	138050552	Abdominal_fat_weight	region1inRegion2	19043
CNVR21	314	2	12364565	12398203	loss	21214	2948442	138050552	Abdominal_fat_weight	region1inRegion2	33638
CNVR22	316	2	12689951	12700771	loss	21214	2948442	138050552	Abdominal_fat_weight	region1inRegion2	10820
CNVR23	319	2	14000848	14032642	loss-gain	21214	2948442	138050552	Abdominal_fat_weight	region1inRegion2	31794
CNVR24	320	2	14728311	14771718	gain	21214	2948442	138050552	Abdominal_fat_weight	region1inRegion2	43407
CNVR25	321	2	15024071	15060448	loss	21214	2948442	138050552	Abdominal_fat_weight	region1inRegion2	36377
CNVR26	343	2	44800086	44803691	gain	21214	2948442	138050552	Abdominal_fat_weight	region1inRegion2	3605
CNVR27	348	2	54685639	54717573	loss-gain	21214	2948442	138050552	Abdominal_fat_weight	region1inRegion2	31934
CNVR28	353	2	56935884	56944870	loss	21214	2948442	138050552	Abdominal_fat_weight	region1inRegion2	8986
CNVR29	355	2	58030910	58035496	gain	21214	2948442	138050552	Abdominal_fat_weight	region1inRegion2	4586
CNVR30	359	2	61357153	61369311	loss	21214	2948442	138050552	Abdominal_fat_weight	region1inRegion2	12158
CNVR31	362	2	62416028	62428337	gain	21214	2948442	138050552	Abdominal_fat_weight	region1inRegion2	12309
CNVR32	364	2	62628253	62738661	gain	21214	2948442	138050552	Abdominal_fat_weight	region1inRegion2	110408
CNVR33	378	2	67915859	67952389	gain	21214	2948442	138050552	Abdominal_fat_weight	region1inRegion2	36530
CNVR34	379	2	68061173	68181801	gain	21214	2948442	138050552	Abdominal_fat_weight	region1inRegion2	120628
CNVR35	401	2	95897658	95915484	loss	21214	2948442	138050552	Abdominal_fat_weight	region1inRegion2	17826
CNVR36	408	2	96833054	96835454	loss	21214	2948442	138050552	Abdominal_fat_weight	region1inRegion2	2400
CNVR37	422	2	107437562	107451870	gain	21214	2948442	138050552	Abdominal_fat_weight	region1inRegion2	14308
CNVR20	312	2	11511074	11530117	gain	21213	4798534	131599097	Abdominal_fat_weight	region1inRegion2	19043
CNVR21	314	2	12364565	12398203	loss	21213	4798534	131599097	Abdominal_fat_weight	region1inRegion2	33638
CNVR22	316	2	12689951	12700771	loss	21213	4798534	131599097	Abdominal_fat_weight	region1inRegion2	10820
CNVR23	319	2	14000848	14032642	loss-gain	21213	4798534	131599097	Abdominal_fat_weight	region1inRegion2	31794
CNVR24	320	2	14728311	14771718	gain	21213	4798534	131599097	Abdominal_fat_weight	region1inRegion2	43407
CNVR25	321	2	15024071	15060448	loss	21213	4798534	131599097	Abdominal_fat_weight	region1inRegion2	36377
CNVR26	343	2	44800086	44803691	gain	21213	4798534	131599097	Abdominal_fat_weight	region1inRegion2	3605
CNVR27	348	2	54685639	54717573	loss-gain	21213	4798534	131599097	Abdominal_fat_weight	region1inRegion2	31934
CNVR28	353	2	56935884	56944870	loss	21213	4798534	131599097	Abdominal_fat_weight	region1inRegion2	8986
CNVR29	355	2	58030910	58035496	gain	21213	4798534	131599097	Abdominal_fat_weight	region1inRegion2	4586
CNVR30	359	2	61357153	61369311	loss	21213	4798534	131599097	Abdominal_fat_weight	region1inRegion2	12158
CNVR31	362	2	62416028	62428337	gain	21213	4798534	131599097	Abdominal_fat_weight	region1inRegion2	12309
CNVR32	364	2	62628253	62738661	gain	21213	4798534	131599097	Abdominal_fat_weight	region1inRegion2	110408
CNVR33	378	2	67915859	67952389	gain	21213	4798534	131599097	Abdominal_fat_weight	region1inRegion2	36530
CNVR34	379	2	68061173	68181801	gain	21213	4798534	131599097	Abdominal_fat_weight	region1inRegion2	120628
CNVR35	401	2	95897658	95915484	loss	21213	4798534	131599097	Abdominal_fat_weight	region1inRegion2	17826
CNVR36	408	2	96833054	96835454	loss	21213	4798534	131599097	Abdominal_fat_weight	region1inRegion2	2400
CNVR37	422	2	107437562	107451870	gain	21213	4798534	131599097	Abdominal_fat_weight	region1inRegion2	14308
CNVR20	312	2	11511074	11530117	gain	5242	5101784	150528181	backfat_at_mid-back	region1inRegion2	19043
CNVR21	314	2	12364565	12398203	loss	5242	5101784	150528181	backfat_at_mid-back	region1inRegion2	33638
CNVR22	316	2	12689951	12700771	loss	5242	5101784	150528181	backfat_at_mid-back	region1inRegion2	10820
CNVR23	319	2	14000848	14032642	loss-gain	5242	5101784	150528181	backfat_at_mid-back	region1inRegion2	31794
CNVR24	320	2	14728311	14771718	gain	5242	5101784	150528181	backfat_at_mid-back	region1inRegion2	43407
CNVR25	321	2	15024071	15060448	loss	5242	5101784	150528181	backfat_at_mid-back	region1inRegion2	36377
CNVR26	343	2	44800086	44803691	gain	5242	5101784	150528181	backfat_at_mid-back	region1inRegion2	3605
CNVR27	348	2	54685639	54717573	loss-gain	5242	5101784	150528181	backfat_at_mid-back	region1inRegion2	31934
CNVR28	353	2	56935884	56944870	loss	5242	5101784	150528181	backfat_at_mid-back	region1inRegion2	8986
CNVR29	355	2	58030910	58035496	gain	5242	5101784	150528181	backfat_at_mid-back	region1inRegion2	4586
CNVR30	359	2	61357153	61369311	loss	5242	5101784	150528181	backfat_at_mid-back	region1inRegion2	12158
CNVR31	362	2	62416028	62428337	gain	5242	5101784	150528181	backfat_at_mid-back	region1inRegion2	12309
CNVR32	364	2	62628253	62738661	gain	5242	5101784	150528181	backfat_at_mid-back	region1inRegion2	110408
CNVR33	378	2	67915859	67952389	gain	5242	5101784	150528181	backfat_at_mid-back	region1inRegion2	36530
CNVR34	379	2	68061173	68181801	gain	5242	5101784	150528181	backfat_at_mid-back	region1inRegion2	120628
CNVR35	401	2	95897658	95915484	loss	5242	5101784	150528181	backfat_at_mid-back	region1inRegion2	17826
CNVR36	408	2	96833054	96835454	loss	5242	5101784	150528181	backfat_at_mid-back	region1inRegion2	2400
CNVR37	422	2	107437562	107451870	gain	5242	5101784	150528181	backfat_at_mid-back	region1inRegion2	14308
CNVR38	466	2	148904898	148913506	loss-gain	5242	5101784	150528181	backfat_at_mid-back	region1inRegion2	8608
CNVR39	467	2	149095217	149101135	loss	5242	5101784	150528181	backfat_at_mid-back	region1inRegion2	5918
CNVR20	312	2	11511074	11530117	gain	5147	6636615	12560715	External_fat_on_ham	region1inRegion2	19043
CNVR21	314	2	12364565	12398203	loss	5147	6636615	12560715	External_fat_on_ham	region1inRegion2	33638
CNVR20	312	2	11511074	11530117	gain	5134	6636615	17962639	Ham_weight	region1inRegion2	19043

CNVR21	314	2	12364565	12398203	loss	5134	6636615	17962639	Ham_weight	region1inRegion2	33638
CNVR22	316	2	12689951	12700771	loss	5134	6636615	17962639	Ham_weight	region1inRegion2	10820
CNVR23	319	2	14000848	14032642	loss-gain	5134	6636615	17962639	Ham_weight	region1inRegion2	31794
CNVR24	320	2	14728311	14771718	gain	5134	6636615	17962639	Ham_weight	region1inRegion2	43407
CNVR25	321	2	15024071	15060448	loss	5134	6636615	17962639	Ham_weight	region1inRegion2	36377
CNVR20	312	2	11511074	11530117	gain	5218	6662614	140912872	Shoulder_subcutaneous_fat_thickness	region1inRegion2	19043
CNVR21	314	2	12364565	12398203	loss	5218	6662614	140912872	Shoulder_subcutaneous_fat_thickness	region1inRegion2	33638
CNVR22	316	2	12689951	12700771	loss	5218	6662614	140912872	Shoulder_subcutaneous_fat_thickness	region1inRegion2	10820
CNVR23	319	2	14000848	14032642	loss-gain	5218	6662614	140912872	Shoulder_subcutaneous_fat_thickness	region1inRegion2	31794
CNVR24	320	2	14728311	14771718	gain	5218	6662614	140912872	Shoulder_subcutaneous_fat_thickness	region1inRegion2	43407
CNVR25	321	2	15024071	15060448	loss	5218	6662614	140912872	Shoulder_subcutaneous_fat_thickness	region1inRegion2	36377
CNVR26	343	2	44800086	44803691	gain	5218	6662614	140912872	Shoulder_subcutaneous_fat_thickness	region1inRegion2	3605
CNVR27	348	2	54685639	54717573	loss-gain	5218	6662614	140912872	Shoulder_subcutaneous_fat_thickness	region1inRegion2	31934
CNVR28	353	2	56935884	56944870	loss	5218	6662614	140912872	Shoulder_subcutaneous_fat_thickness	region1inRegion2	8986
CNVR29	355	2	58030910	58035496	gain	5218	6662614	140912872	Shoulder_subcutaneous_fat_thickness	region1inRegion2	4586
CNVR30	359	2	61357153	61369311	loss	5218	6662614	140912872	Shoulder_subcutaneous_fat_thickness	region1inRegion2	12158
CNVR31	362	2	62416028	62428337	gain	5218	6662614	140912872	Shoulder_subcutaneous_fat_thickness	region1inRegion2	12309
CNVR32	364	2	62628253	62738661	gain	5218	6662614	140912872	Shoulder_subcutaneous_fat_thickness	region1inRegion2	110408
CNVR33	378	2	67915859	67952389	gain	5218	6662614	140912872	Shoulder_subcutaneous_fat_thickness	region1inRegion2	36530
CNVR34	379	2	68061173	68181801	gain	5218	6662614	140912872	Shoulder_subcutaneous_fat_thickness	region1inRegion2	120628
CNVR35	401	2	95897658	95915484	loss	5218	6662614	140912872	Shoulder_subcutaneous_fat_thickness	region1inRegion2	17826
CNVR36	408	2	96833054	96835454	loss	5218	6662614	140912872	Shoulder_subcutaneous_fat_thickness	region1inRegion2	2400
CNVR37	422	2	107437562	107451870	gain	5218	6662614	140912872	Shoulder_subcutaneous_fat_thickness	region1inRegion2	14308
CNVR20	312	2	11511074	11530117	gain	5755	7389378	12537424	Marbling	region1inRegion2	19043
CNVR21	314	2	12364565	12398203	loss	5755	7389378	12537424	Marbling	region1inRegion2	33638
CNVR20	312	2	11511074	11530117	gain	12285	7440323	13366532	Mean_corpuscular_hemoglobin_concentration	region1inRegion2	19043
CNVR21	314	2	12364565	12398203	loss	12285	7440323	13366532	Mean_corpuscular_hemoglobin_concentration	region1inRegion2	33638
CNVR22	316	2	12689951	12700771	loss	12285	7440323	13366532	Mean_corpuscular_hemoglobin_concentration	region1inRegion2	10820
CNVR20	312	2	11511074	11530117	gain	37538	7440323	13366532	CSFV_antibody_level	region1inRegion2	19043
CNVR21	314	2	12364565	12398203	loss	37538	7440323	13366532	CSFV_antibody_level	region1inRegion2	33638
CNVR22	316	2	12689951	12700771	loss	37538	7440323	13366532	CSFV_antibody_level	region1inRegion2	10820
CNVR20	312	2	11511074	11530117	gain	86	7440323	38367026	Drip_loss	region1inRegion2	19043
CNVR21	314	2	12364565	12398203	loss	86	7440323	38367026	Drip_loss	region1inRegion2	33638
CNVR22	316	2	12689951	12700771	loss	86	7440323	38367026	Drip_loss	region1inRegion2	10820
CNVR23	319	2	14000848	14032642	loss-gain	86	7440323	38367026	Drip_loss	region1inRegion2	31794
CNVR24	320	2	14728311	14771718	gain	86	7440323	38367026	Drip_loss	region1inRegion2	43407
CNVR25	321	2	15024071	15060448	loss	86	7440323	38367026	Drip_loss	region1inRegion2	36377
CNVR20	312	2	11511074	11530117	gain	38075	7440323	131656956	Drip_loss	region1inRegion2	19043
CNVR21	314	2	12364565	12398203	loss	38075	7440323	131656956	Drip_loss	region1inRegion2	33638
CNVR22	316	2	12689951	12700771	loss	38075	7440323	131656956	Drip_loss	region1inRegion2	10820
CNVR23	319	2	14000848	14032642	loss-gain	38075	7440323	131656956	Drip_loss	region1inRegion2	31794
CNVR24	320	2	14728311	14771718	gain	38075	7440323	131656956	Drip_loss	region1inRegion2	43407
CNVR25	321	2	15024071	15060448	loss	38075	7440323	131656956	Drip_loss	region1inRegion2	36377
CNVR26	343	2	44800086	44803691	gain	38075	7440323	131656956	Drip_loss	region1inRegion2	3605
CNVR27	348	2	54685639	54717573	loss-gain	38075	7440323	131656956	Drip_loss	region1inRegion2	31934
CNVR28	353	2	56935884	56944870	loss	38075	7440323	131656956	Drip_loss	region1inRegion2	8986
CNVR29	355	2	58030910	58035496	gain	38075	7440323	131656956	Drip_loss	region1inRegion2	4586
CNVR30	359	2	61357153	61369311	loss	38075	7440323	131656956	Drip_loss	region1inRegion2	12158
CNVR31	362	2	62416028	62428337	gain	38075	7440323	131656956	Drip_loss	region1inRegion2	12309
CNVR32	364	2	62628253	62738661	gain	38075	7440323	131656956	Drip_loss	region1inRegion2	110408
CNVR33	378	2	67915859	67952389	gain	38075	7440323	131656956	Drip_loss	region1inRegion2	36530
CNVR34	379	2	68061173	68181801	gain	38075	7440323	131656956	Drip_loss	region1inRegion2	120628
CNVR35	401	2	95897658	95915484	loss	38075	7440323	131656956	Drip_loss	region1inRegion2	17826
CNVR36	408	2	96833054	96835454	loss	38075	7440323	131656956	Drip_loss	region1inRegion2	2400
CNVR37	422	2	107437562	107451870	gain	38075	7440323	131656956	Drip_loss	region1inRegion2	14308
CNVR20	312	2	11511074	11530117	gain	5208	7804743	150596593	backfat_at_mid-back	region1inRegion2	19043
CNVR21	314	2	12364565	12398203	loss	5208	7804743	150596593	backfat_at_mid-back	region1inRegion2	33638
CNVR22	316	2	12689951	12700771	loss	5208	7804743	150596593	backfat_at_mid-back	region1inRegion2	10820
CNVR23	319	2	14000848	14032642	loss-gain	5208	7804743	150596593	backfat_at_mid-back	region1inRegion2	31794
CNVR24	320	2	14728311	14771718	gain	5208	7804743	150596593	backfat_at_mid-back	region1inRegion2	43407
CNVR25	321	2	15024071	15060448	loss	5208	7804743	150596593	backfat_at_mid-back	region1inRegion2	36377
CNVR26	343	2	44800086	44803691	gain	5208	7804743	150596593	backfat_at_mid-back	region1inRegion2	3605
CNVR27	348	2	54685639	54717573	loss-gain	5208	7804743	150596593	backfat_at_mid-back	region1inRegion2	31934
CNVR28	353	2	56935884	56944870	loss	5208	7804743	150596593	backfat_at_mid-back	region1inRegion2	8986
CNVR29	355	2	58030910	58035496	gain	5208	7804743	150596593	backfat_at_mid-back	region1inRegion2	4586
CNVR30	359	2	61357153	61369311	loss	5208	7804743	150596593	backfat_at_mid-back	region1inRegion2	12158
CNVR31	362	2	62416028	62428337	gain	5208	7804743	150596593	backfat_at_mid-back	region1inRegion2	12309
CNVR32	364	2	62628253	62738661	gain	5208	7804743	150596593	backfat_at_mid-back	region1inRegion2	110408
CNVR33	378	2	67915859	67952389	gain	5208	7804743	150596593	backfat_at_mid-back	region1inRegion2	36530
CNVR34	379	2	68061173	68181801	gain	5208	7804743	150596593	backfat_at_mid-back	region1inRegion2	120628
CNVR35	401	2	95897658	95915484	loss	5208	7804743	150596593	backfat_at_mid-back	region1inRegion2	17826
CNVR36	408	2	96833054	96835454	loss	5208	7804743	150596593	backfat_at_mid-back	region1inRegion2	2400
CNVR37	422	2	107437562	107451870	gain	5208	7804743	150596593	backfat_at_mid-back	region1inRegion2	14308
CNVR38	466	2	148904898	148913506	loss-gain	5208	7804743	150596593	backfat_at_mid-back	region1inRegion2	8608
CNVR39	467	2	149095217	149101135	loss	5208	7804743	150596593	backfat_at_mid-back	region1inRegion2	5918
CNVR20	312	2	11511074	11530117	gain	5760	7990163	84282205	tenderness_score	region1inRegion2	19043
CNVR21	314	2	12364565	12398203	loss	5760	7990163	84282205	tenderness_score	region1inRegion2	33638
CNVR22	316	2	12689951	12700771	loss	5760	7990163	84282205	tenderness_score	region1inRegion2	10820
CNVR23	319	2	14000848	14032642	loss-gain	5760	7990163	84282205	tenderness_score	region1inRegion2	31794
CNVR24	320	2	14728311	14771718	gain	5760	7990163	84282205	tenderness_score	region1inRegion2	43407
CNVR25	321	2	15024071	15060448	loss	5760	7990163	84282205	tenderness_score	region1inRegion2	36377
CNVR26	343	2	44800086	44803691	gain	5760	7990163	84282205	tenderness_score	region1inRegion2	3605
CNVR27	348	2	54685639	54717573	loss-gain	5760	7990163	84282205	tenderness_score	region1inRegion2	31934
CNVR28	353	2	56935884	56944870	loss	5760	7990163	84282205	tenderness_score	region1inRegion2	8986
CNVR29	355	2	58030910	58035496	gain	5760	7990163	84282205	tenderness_score	region1inRegion2	4586
CNVR30	359	2	61357153	61369311	loss	5760	7990163	84282205	tenderness_score	region1inRegion2	12158
CNVR31	362	2	62416028	62428337	gain	5760	7990163	84282205	tenderness_score	region1inRegion2	12309
CNVR32	364	2	62628253	62738661	gain	5760	7990163	84282205	tenderness_score	region1inRegion2	110408
CNVR33	378	2	67915859	67952389	gain	5760	7990163	84282205	tenderness_score	region1inRegion2	36530
CNVR34	379	2	68061173	68181801	gain	5760	7990163	84282205	tenderness_score	region1inRegion2	120628
CNVR20	312	2	11511074	11530117	gain	37557	8499847	155788186	Actinobacillus_pleuropneumoniae_susceptibility	region1inRegion2	19043
CNVR21	314	2	12364565	12398203	loss	37557	8499847	155788186	Actinobacillus_pleuropneumoniae_susceptibility	region1inRegion2	33638
CNVR22	316	2	12689951	12700771	loss	37557	8499847	155788186	Actinobacillus_pleuropneumoniae_susceptibility	region1inRegion2	10820
CNVR23	319	2	14000848	14032642	loss-gain	37557	8499847	155788186	Actinobacillus_pleuropneumoniae_susceptibility	region1inRegion2	31794
CNVR24	320	2	14728311	14771718	gain	37557	8499847	155788186	Actinobacillus_pleuropneumoniae_susceptibility	region1inRegion2	43407
CNVR25	321	2	15024071	15060448	loss	37557	8499847	155788186	Actinobacillus_pleuropneumoniae_susceptibility	region1inRegion2	36377
CNVR26	343	2	44800086	44803691	gain	37557	8499847	155788186	Actinobacillus_pleuropneumoniae_susceptibility	region1inRegion2	3605
CNVR27	348	2	54685639	54717573	loss-gain	37557	8499847	155788186	Actinobacillus_pleuropneumoniae_susceptibility	region1inRegion2	31934
CNVR28	353	2	56935884	56944870	loss	37557	8499847	155788186	Actinobacillus_pleuropneumoniae_susceptibility	region1inRegion2	8986
CNVR29	355	2	58030910	58035496	gain	37557	8499847	155788186	Actinobacillus_pleuropneumoniae_susceptibility	region1inRegion2	4586
CNVR30	359	2	61357153	61369311	loss	37557	8499847	155788186	Actinobacillus_pleuropneumoniae_susceptibility	region1inRegion2	12158
CNVR31	362	2	62416028	62428337	gain	37557	8499847	155788186	Actinobacillus_pleuropneumoniae_susceptibility	region1inRegion2	12309
CNVR32	364	2	62628253	62738661	gain	37557	8499847	155788186	Actinobacillus_pleuropneumoniae_susceptibility	region1inRegion2	110408
CNVR33	378	2	67915859	67952389	gain	37557	8499847	155788186	Actinobacillus_pleuropneumoniae_susceptibility	region1inRegion2	36530
CNVR34	379	2	68061173	68181801	gain	37557	8499847	155788186	Actinobacillus_pleuropneumoniae_susceptibility	region1inRegion2	120628
CNVR35	401	2	95897658	95915484	loss	37557	8499847	155788186	Actinobacillus_pleuropneumoniae_susceptibility	region1inRegion2	17826
CNVR36	408	2	96833054	96835454	loss	37557	8499847	155788186	Actinobacillus_pleuropneumoniae_susceptibility	region1inRegion2	2400
CNVR37	422	2	107437562	107451870	gain	37557	8499847	155788186	Actinobacillus_pleuropneumoniae_susceptibility	region1inRegion2	14308
CNVR38	466	2	148904898	148913506	loss-gain	37557	8499847	155788186	Actinobacillus_pleuropneumoniae_susceptibility	region1inRegion2	8608
CNVR39	467	2	149095217	149101135	loss	37557	8499847	155788186	Actinobacillus_pleuropneumoniae_susceptibility	region1inRegion2	5918
CNVR20	312	2	11511074	11530117	gain	5199	9758855	150135089	Body_weight_(end_of_test)	region1inRegion2	19043
CNVR21	314	2	12364565	12398203	loss	5199	9758855	150135089	Body_weight_(end_of_test)	region1inRegion2	33638
CNVR22	316	2	12689951	12700771	loss	5199	9758855	150135089	Body_weight_(end_of_test)	region1inRegion2	10820
CNVR23	319	2	14000848	14032642	loss-gain	5199	9758855	150135089	Body_weight_(end_of_test)	region1inRegion2	31794
CNVR24	320	2	14728311	14771718	gain	5199	9758855	150135089	Body_weight_(end_of_test)	region1inRegion2	43407
CNVR25	321	2	15024071	15060448	loss	5199	9758855	150135089	Body_weight_(end_of_test)	region1inRegion2	36377
CNVR26	343	2	44800086	44803691	gain	5199	9758855	150135089	Body_weight_(end_of_test)	region1inRegion2	3605
CNVR27	348	2	54685639	54717573	loss-gain	5199	9758855	150135089	Body_weight_(end_of_test)	region1inRegion2	31934
CNVR28	353	2	56935884	56944870	loss	5199	9758855	150135089	Body_weight_(end_of_test)	region1inRegion2	8986
CNVR29	355	2	58030910	58035496	gain	5199	9758855	150135089	Body_weight_(end_of_test)	region1inRegion2	4586
CNVR30	359	2	61357153	61369311	loss	5199	9758855	150135089	Body_weight_(end_of_test)	region1inRegion2	12158
CNVR31	362	2	62416028	62428337	gain	5199	9758855	150135089	Body_weight_(end_of_test)	region1inRegion2	12309

CNVR32	364	2	62628253	62738661	gain	5199	9758855	150135089	Body_weight_(end_of_test)	region1inRegion2	110408
CNVR33	378	2	67915859	67952389	gain	5199	9758855	150135089	Body_weight_(end_of_test)	region1inRegion2	36530
CNVR34	379	2	68061173	68181801	gain	5199	9758855	150135089	Body_weight_(end_of_test)	region1inRegion2	120628
CNVR35	401	2	95897658	95915484	loss	5199	9758855	150135089	Body_weight_(end_of_test)	region1inRegion2	17826
CNVR36	408	2	96833054	96835454	loss	5199	9758855	150135089	Body_weight_(end_of_test)	region1inRegion2	2400
CNVR37	422	2	107437562	107451870	gain	5199	9758855	150135089	Body_weight_(end_of_test)	region1inRegion2	14308
CNVR38	466	2	148904898	148913506	loss-gain	5199	9758855	150135089	Body_weight_(end_of_test)	region1inRegion2	8608
CNVR39	467	2	149095217	149101135	loss	5199	9758855	150135089	Body_weight_(end_of_test)	region1inRegion2	5918
CNVR20	312	2	11511074	11530117	gain	17549	10677424	12726165	Body_weight_(weaning)	region1inRegion2	19043
CNVR21	314	2	12364565	12398203	loss	17549	10677424	12726165	Body_weight_(weaning)	region1inRegion2	33638
CNVR22	316	2	12689951	12700771	loss	17549	10677424	12726165	Body_weight_(weaning)	region1inRegion2	10820
CNVR20	312	2	11511074	11530117	gain	7311	10833649	18301063	Ham_weight	region1inRegion2	19043
CNVR21	314	2	12364565	12398203	loss	7311	10833649	18301063	Ham_weight	region1inRegion2	33638
CNVR22	316	2	12689951	12700771	loss	7311	10833649	18301063	Ham_weight	region1inRegion2	10820
CNVR23	319	2	14000848	14032642	loss-gain	7311	10833649	18301063	Ham_weight	region1inRegion2	31794
CNVR24	320	2	14728311	14771718	gain	7311	10833649	18301063	Ham_weight	region1inRegion2	43407
CNVR25	321	2	15024071	15060448	loss	7311	10833649	18301063	Ham_weight	region1inRegion2	36377
CNVR20	312	2	11511074	11530117	gain	21313	10846385	21136167	Mycoplasmal_pneumonia_susceptibility	region1inRegion2	19043
CNVR21	314	2	12364565	12398203	loss	21313	10846385	21136167	Mycoplasmal_pneumonia_susceptibility	region1inRegion2	33638
CNVR22	316	2	12689951	12700771	loss	21313	10846385	21136167	Mycoplasmal_pneumonia_susceptibility	region1inRegion2	10820
CNVR23	319	2	14000848	14032642	loss-gain	21313	10846385	21136167	Mycoplasmal_pneumonia_susceptibility	region1inRegion2	31794
CNVR24	320	2	14728311	14771718	gain	21313	10846385	21136167	Mycoplasmal_pneumonia_susceptibility	region1inRegion2	43407
CNVR25	321	2	15024071	15060448	loss	21313	10846385	21136167	Mycoplasmal_pneumonia_susceptibility	region1inRegion2	36377
CNVR20	312	2	11511074	11530117	gain	5394	10846385	21136167	Hemoglobin	region1inRegion2	19043
CNVR21	314	2	12364565	12398203	loss	5394	10846385	21136167	Hemoglobin	region1inRegion2	33638
CNVR22	316	2	12689951	12700771	loss	5394	10846385	21136167	Hemoglobin	region1inRegion2	10820
CNVR23	319	2	14000848	14032642	loss-gain	5394	10846385	21136167	Hemoglobin	region1inRegion2	31794
CNVR24	320	2	14728311	14771718	gain	5394	10846385	21136167	Hemoglobin	region1inRegion2	43407
CNVR25	321	2	15024071	15060448	loss	5394	10846385	21136167	Hemoglobin	region1inRegion2	36377
CNVR20	312	2	11511074	11530117	gain	5411	10846385	21136167	Red_blood_cell_count	region1inRegion2	19043
CNVR21	314	2	12364565	12398203	loss	5411	10846385	21136167	Red_blood_cell_count	region1inRegion2	33638
CNVR22	316	2	12689951	12700771	loss	5411	10846385	21136167	Red_blood_cell_count	region1inRegion2	10820
CNVR23	319	2	14000848	14032642	loss-gain	5411	10846385	21136167	Red_blood_cell_count	region1inRegion2	31794
CNVR24	320	2	14728311	14771718	gain	5411	10846385	21136167	Red_blood_cell_count	region1inRegion2	43407
CNVR25	321	2	15024071	15060448	loss	5411	10846385	21136167	Red_blood_cell_count	region1inRegion2	36377
CNVR20	312	2	11511074	11530117	gain	5422	10846385	21136167	hematocrit	region1inRegion2	19043
CNVR21	314	2	12364565	12398203	loss	5422	10846385	21136167	hematocrit	region1inRegion2	33638
CNVR22	316	2	12689951	12700771	loss	5422	10846385	21136167	hematocrit	region1inRegion2	10820
CNVR23	319	2	14000848	14032642	loss-gain	5422	10846385	21136167	hematocrit	region1inRegion2	31794
CNVR24	320	2	14728311	14771718	gain	5422	10846385	21136167	hematocrit	region1inRegion2	43407
CNVR25	321	2	15024071	15060448	loss	5422	10846385	21136167	hematocrit	region1inRegion2	36377
CNVR20	312	2	11511074	11530117	gain	6354	10846385	21136167	Alkaline_phosphatase_activity	region1inRegion2	19043
CNVR21	314	2	12364565	12398203	loss	6354	10846385	21136167	Alkaline_phosphatase_activity	region1inRegion2	33638
CNVR22	316	2	12689951	12700771	loss	6354	10846385	21136167	Alkaline_phosphatase_activity	region1inRegion2	10820
CNVR23	319	2	14000848	14032642	loss-gain	6354	10846385	21136167	Alkaline_phosphatase_activity	region1inRegion2	31794
CNVR24	320	2	14728311	14771718	gain	6354	10846385	21136167	Alkaline_phosphatase_activity	region1inRegion2	43407
CNVR25	321	2	15024071	15060448	loss	6354	10846385	21136167	Alkaline_phosphatase_activity	region1inRegion2	36377
CNVR20	312	2	11511074	11530117	gain	17550	11055674	26478024	Average_backfat_thickness	region1inRegion2	19043
CNVR21	314	2	12364565	12398203	loss	17550	11055674	26478024	Average_backfat_thickness	region1inRegion2	33638
CNVR22	316	2	12689951	12700771	loss	17550	11055674	26478024	Average_backfat_thickness	region1inRegion2	10820
CNVR23	319	2	14000848	14032642	loss-gain	17550	11055674	26478024	Average_backfat_thickness	region1inRegion2	31794
CNVR24	320	2	14728311	14771718	gain	17550	11055674	26478024	Average_backfat_thickness	region1inRegion2	43407
CNVR25	321	2	15024071	15060448	loss	17550	11055674	26478024	Average_backfat_thickness	region1inRegion2	36377
CNVR20	312	2	11511074	11530117	gain	17552	11327829	19559392	CIE-a*	region1inRegion2	19043
CNVR21	314	2	12364565	12398203	loss	17552	11327829	19559392	CIE-a*	region1inRegion2	33638
CNVR22	316	2	12689951	12700771	loss	17552	11327829	19559392	CIE-a*	region1inRegion2	10820
CNVR23	319	2	14000848	14032642	loss-gain	17552	11327829	19559392	CIE-a*	region1inRegion2	31794
CNVR24	320	2	14728311	14771718	gain	17552	11327829	19559392	CIE-a*	region1inRegion2	43407
CNVR25	321	2	15024071	15060448	loss	17552	11327829	19559392	CIE-a*	region1inRegion2	36377
CNVR20	312	2	11511074	11530117	gain	17551	11360950	27277479	Lean_meat_percentage	region1inRegion2	19043
CNVR21	314	2	12364565	12398203	loss	17551	11360950	27277479	Lean_meat_percentage	region1inRegion2	33638
CNVR22	316	2	12689951	12700771	loss	17551	11360950	27277479	Lean_meat_percentage	region1inRegion2	10820
CNVR23	319	2	14000848	14032642	loss-gain	17551	11360950	27277479	Lean_meat_percentage	region1inRegion2	31794
CNVR24	320	2	14728311	14771718	gain	17551	11360950	27277479	Lean_meat_percentage	region1inRegion2	43407
CNVR25	321	2	15024071	15060448	loss	17551	11360950	27277479	Lean_meat_percentage	region1inRegion2	36377
CNVR20	312	2	11511074	11530117	gain	8680	11496528	11806359	Sperm_per_ejaculate	region1inRegion2	19043
CNVR21	314	2	12364565	12398203	loss	21303	12184677	154346072	Lumbar_vertebra_number	region1inRegion2	33638
CNVR22	316	2	12689951	12700771	loss	21303	12184677	154346072	Lumbar_vertebra_number	region1inRegion2	10820
CNVR23	319	2	14000848	14032642	loss-gain	21303	12184677	154346072	Lumbar_vertebra_number	region1inRegion2	31794
CNVR24	320	2	14728311	14771718	gain	21303	12184677	154346072	Lumbar_vertebra_number	region1inRegion2	43407
CNVR25	321	2	15024071	15060448	loss	21303	12184677	154346072	Lumbar_vertebra_number	region1inRegion2	36377
CNVR26	343	2	44800086	44803691	gain	21303	12184677	154346072	Lumbar_vertebra_number	region1inRegion2	3605
CNVR27	348	2	54685639	54717573	loss-gain	21303	12184677	154346072	Lumbar_vertebra_number	region1inRegion2	31934
CNVR28	353	2	56935884	56944870	loss	21303	12184677	154346072	Lumbar_vertebra_number	region1inRegion2	8986
CNVR29	355	2	58030910	58035496	gain	21303	12184677	154346072	Lumbar_vertebra_number	region1inRegion2	4586
CNVR30	359	2	61357153	61369311	loss	21303	12184677	154346072	Lumbar_vertebra_number	region1inRegion2	12158
CNVR31	362	2	62416028	62428337	gain	21303	12184677	154346072	Lumbar_vertebra_number	region1inRegion2	12309
CNVR32	364	2	62628253	62738661	gain	21303	12184677	154346072	Lumbar_vertebra_number	region1inRegion2	110408
CNVR33	378	2	67915859	67952389	gain	21303	12184677	154346072	Lumbar_vertebra_number	region1inRegion2	36530
CNVR34	379	2	68061173	68181801	gain	21303	12184677	154346072	Lumbar_vertebra_number	region1inRegion2	120628
CNVR35	401	2	95897658	95915484	loss	21303	12184677	154346072	Lumbar_vertebra_number	region1inRegion2	17826
CNVR36	408	2	96833054	96835454	loss	21303	12184677	154346072	Lumbar_vertebra_number	region1inRegion2	2400
CNVR37	422	2	107437562	107451870	gain	21303	12184677	154346072	Lumbar_vertebra_number	region1inRegion2	14308
CNVR38	466	2	148904898	148913506	loss-gain	21303	12184677	154346072	Lumbar_vertebra_number	region1inRegion2	8608
CNVR39	467	2	149095217	149101135	loss	21303	12184677	154346072	Lumbar_vertebra_number	region1inRegion2	5918
CNVR21	314	2	12364565	12398203	loss	28216	12231327	12412044	Backfat_at_rump	region1inRegion2	33638
CNVR23	319	2	14000848	14032642	loss-gain	17558	12858082	26561459	Lean_meat_percentage	region1inRegion2	31794
CNVR24	320	2	14728311	14771718	gain	17558	12858082	26561459	Lean_meat_percentage	region1inRegion2	43407
CNVR25	321	2	15024071	15060448	loss	17558	12858082	26561459	Lean_meat_percentage	region1inRegion2	36377
CNVR23	319	2	14000848	14032642	loss-gain	3889	12897996	24656701	Daily_feed_intake	region1inRegion2	31794
CNVR24	320	2	14728311	14771718	gain	3889	12897996	24656701	Daily_feed_intake	region1inRegion2	43407
CNVR25	321	2	15024071	15060448	loss	3889	12897996	24656701	Daily_feed_intake	region1inRegion2	36377
CNVR23	319	2	14000848	14032642	loss-gain	12313	13366532	21136167	Platelet_distribution_width	region1inRegion2	31794
CNVR24	320	2	14728311	14771718	gain	12313	13366532	21136167	Platelet_distribution_width	region1inRegion2	43407
CNVR25	321	2	15024071	15060448	loss	12313	13366532	21136167	Platelet_distribution_width	region1inRegion2	36377
CNVR23	319	2	14000848	14032642	loss-gain	12314	13366532	21136167	Plateletcrit	region1inRegion2	31794
CNVR24	320	2	14728311	14771718	gain	12314	13366532	21136167	Plateletcrit	region1inRegion2	43407
CNVR25	321	2	15024071	15060448	loss	12314	13366532	21136167	Plateletcrit	region1inRegion2	36377
CNVR23	319	2	14000848	14032642	loss-gain	917	13366532	21136167	Heart_weight	region1inRegion2	31794
CNVR24	320	2	14728311	14771718	gain	917	13366532	21136167	Heart_weight	region1inRegion2	43407
CNVR25	321	2	15024071	15060448	loss	917	13366532	21136167	Heart_weight	region1inRegion2	36377
CNVR23	319	2	14000848	14032642	loss-gain	925	13366532	21136167	Backfat_at_tenth_rib	region1inRegion2	31794
CNVR24	320	2	14728311	14771718	gain	925	13366532	21136167	Backfat_at_tenth_rib	region1inRegion2	43407
CNVR25	321	2	15024071	15060448	loss	925	13366532	21136167	Backfat_at_tenth_rib	region1inRegion2	36377
CNVR23	319	2	14000848	14032642	loss-gain	926	13366532	21136167	Shoulder_subcutaneous_fat_thickness	region1inRegion2	31794
CNVR24	320	2	14728311	14771718	gain	926	13366532	21136167	Shoulder_subcutaneous_fat_thickness	region1inRegion2	43407
CNVR25	321	2	15024071	15060448	loss	926	13366532	21136167	Shoulder_subcutaneous_fat_thickness	region1inRegion2	36377
CNVR23	319	2	14000848	14032642	loss-gain	2937	13366532	34221548	Drip_loss	region1inRegion2	31794
CNVR24	320	2	14728311	14771718	gain	2937	13366532	34221548	Drip_loss	region1inRegion2	43407
CNVR25	321	2	15024071	15060448	loss	2937	13366532	34221548	Drip_loss	region1inRegion2	36377
CNVR23	319	2	14000848	14032642	loss-gain	2938	13366532	34221548	Off-Flavor_Score	region1inRegion2	31794
CNVR24	320	2	14728311	14771718	gain	2938	13366532	34221548	Off-Flavor_Score	region1inRegion2	43407
CNVR25	321	2	15024071	15060448	loss	2938	13366532	34221548	Off-Flavor_Score	region1inRegion2	36377
CNVR23	319	2	14000848	14032642	loss-gain	902	13366532	44483418	pH_24_hr_post-mortem_(loin)	region1inRegion2	31794
CNVR24	320	2	14728311	14771718	gain	902	13366532	44483418	pH_24_hr_post-mortem_(loin)	region1inRegion2	43407
CNVR25	321	2	15024071	15060448	loss	902	13366532	44483418	pH_24_hr_post-mortem_(loin)	region1inRegion2	36377
CNVR23	319	2	14000848	14032642	loss-gain	909	13366532	44483418	Teat_number	region1inRegion2	31794
CNVR24	320	2	14728311	14771718	gain	909	13366532	44483418	Teat_number	region1inRegion2	43407
CNVR25	321	2	15024071	15060448	loss	909	13366532	44483418	Teat_number	region1inRegion2	36377
CNVR23	319	2	14000848	14032642	loss-gain	21848	13366532	46840491	Drip_loss	region1inRegion2	31794
CNVR24	320	2	14728311	14771718	gain	21848	13366532	46840491	Drip_loss	region1inRegion2	43407
CNVR25	321	2	15024071	15060448	loss	21848	13366532	46840491	Drip_loss	region1inRegion2	36377

CNVR26	343	2	44800086	44803691	gain	21848	13366532	46840491	Drip_loss	region1inRegion2	3605
CNVR23	319	2	14000848	14032642	loss-gain	17998	13366532	75040316	Average_backfat_thickness	region1inRegion2	31794
CNVR24	320	2	14728311	14771718	gain	17998	13366532	75040316	Average_backfat_thickness	region1inRegion2	43407
CNVR25	321	2	15024071	15060448	loss	17998	13366532	75040316	Average_backfat_thickness	region1inRegion2	36377
CNVR26	343	2	44800086	44803691	gain	17998	13366532	75040316	Average_backfat_thickness	region1inRegion2	3605
CNVR27	348	2	54685639	54717573	loss-gain	17998	13366532	75040316	Average_backfat_thickness	region1inRegion2	31934
CNVR28	353	2	56935884	56944870	loss	17998	13366532	75040316	Average_backfat_thickness	region1inRegion2	8986
CNVR29	355	2	58030910	58035496	gain	17998	13366532	75040316	Average_backfat_thickness	region1inRegion2	4586
CNVR30	359	2	61357153	61369311	loss	17998	13366532	75040316	Average_backfat_thickness	region1inRegion2	12158
CNVR31	362	2	62416028	62428337	gain	17998	13366532	75040316	Average_backfat_thickness	region1inRegion2	12309
CNVR32	364	2	62628253	62738661	gain	17998	13366532	75040316	Average_backfat_thickness	region1inRegion2	110408
CNVR33	378	2	67915859	67952389	gain	17998	13366532	75040316	Average_backfat_thickness	region1inRegion2	36530
CNVR34	379	2	68061173	68181801	gain	17998	13366532	75040316	Average_backfat_thickness	region1inRegion2	120628
CNVR23	319	2	14000848	14032642	loss-gain	3780	13366532	100385940	Drip_loss	region1inRegion2	31794
CNVR24	320	2	14728311	14771718	gain	3780	13366532	100385940	Drip_loss	region1inRegion2	43407
CNVR25	321	2	15024071	15060448	loss	3780	13366532	100385940	Drip_loss	region1inRegion2	36377
CNVR26	343	2	44800086	44803691	gain	3780	13366532	100385940	Drip_loss	region1inRegion2	3605
CNVR27	348	2	54685639	54717573	loss-gain	3780	13366532	100385940	Drip_loss	region1inRegion2	31934
CNVR28	353	2	56935884	56944870	loss	3780	13366532	100385940	Drip_loss	region1inRegion2	8986
CNVR29	355	2	58030910	58035496	gain	3780	13366532	100385940	Drip_loss	region1inRegion2	4586
CNVR30	359	2	61357153	61369311	loss	3780	13366532	100385940	Drip_loss	region1inRegion2	12158
CNVR31	362	2	62416028	62428337	gain	3780	13366532	100385940	Drip_loss	region1inRegion2	12309
CNVR32	364	2	62628253	62738661	gain	3780	13366532	100385940	Drip_loss	region1inRegion2	110408
CNVR33	378	2	67915859	67952389	gain	3780	13366532	100385940	Drip_loss	region1inRegion2	36530
CNVR34	379	2	68061173	68181801	gain	3780	13366532	100385940	Drip_loss	region1inRegion2	120628
CNVR35	401	2	95897658	95915484	loss	3780	13366532	100385940	Drip_loss	region1inRegion2	17826
CNVR36	408	2	96833054	96835454	loss	3780	13366532	100385940	Drip_loss	region1inRegion2	2400
CNVR23	319	2	14000848	14032642	loss-gain	3809	13366532	100385940	Firmness	region1inRegion2	31794
CNVR24	320	2	14728311	14771718	gain	3809	13366532	100385940	Firmness	region1inRegion2	43407
CNVR25	321	2	15024071	15060448	loss	3809	13366532	100385940	Firmness	region1inRegion2	36377
CNVR26	343	2	44800086	44803691	gain	3809	13366532	100385940	Firmness	region1inRegion2	3605
CNVR27	348	2	54685639	54717573	loss-gain	3809	13366532	100385940	Firmness	region1inRegion2	31934
CNVR28	353	2	56935884	56944870	loss	3809	13366532	100385940	Firmness	region1inRegion2	8986
CNVR29	355	2	58030910	58035496	gain	3809	13366532	100385940	Firmness	region1inRegion2	4586
CNVR30	359	2	61357153	61369311	loss	3809	13366532	100385940	Firmness	region1inRegion2	12158
CNVR31	362	2	62416028	62428337	gain	3809	13366532	100385940	Firmness	region1inRegion2	12309
CNVR32	364	2	62628253	62738661	gain	3809	13366532	100385940	Firmness	region1inRegion2	110408
CNVR33	378	2	67915859	67952389	gain	3809	13366532	100385940	Firmness	region1inRegion2	36530
CNVR34	379	2	68061173	68181801	gain	3809	13366532	100385940	Firmness	region1inRegion2	120628
CNVR35	401	2	95897658	95915484	loss	3809	13366532	100385940	Firmness	region1inRegion2	17826
CNVR36	408	2	96833054	96835454	loss	3809	13366532	100385940	Firmness	region1inRegion2	2400
CNVR23	319	2	14000848	14032642	loss-gain	3810	13366532	100385940	Off-Flavor_Score	region1inRegion2	31794
CNVR24	320	2	14728311	14771718	gain	3810	13366532	100385940	Off-Flavor_Score	region1inRegion2	43407
CNVR25	321	2	15024071	15060448	loss	3810	13366532	100385940	Off-Flavor_Score	region1inRegion2	36377
CNVR26	343	2	44800086	44803691	gain	3810	13366532	100385940	Off-Flavor_Score	region1inRegion2	3605
CNVR27	348	2	54685639	54717573	loss-gain	3810	13366532	100385940	Off-Flavor_Score	region1inRegion2	31934
CNVR28	353	2	56935884	56944870	loss	3810	13366532	100385940	Off-Flavor_Score	region1inRegion2	8986
CNVR29	355	2	58030910	58035496	gain	3810	13366532	100385940	Off-Flavor_Score	region1inRegion2	4586
CNVR30	359	2	61357153	61369311	loss	3810	13366532	100385940	Off-Flavor_Score	region1inRegion2	12158
CNVR31	362	2	62416028	62428337	gain	3810	13366532	100385940	Off-Flavor_Score	region1inRegion2	12309
CNVR32	364	2	62628253	62738661	gain	3810	13366532	100385940	Off-Flavor_Score	region1inRegion2	110408
CNVR33	378	2	67915859	67952389	gain	3810	13366532	100385940	Off-Flavor_Score	region1inRegion2	36530
CNVR34	379	2	68061173	68181801	gain	3810	13366532	100385940	Off-Flavor_Score	region1inRegion2	120628
CNVR35	401	2	95897658	95915484	loss	3810	13366532	100385940	Off-Flavor_Score	region1inRegion2	17826
CNVR36	408	2	96833054	96835454	loss	3810	13366532	100385940	Off-Flavor_Score	region1inRegion2	2400
CNVR23	319	2	14000848	14032642	loss-gain	15066	13366532	131656956	Cholesterol_level	region1inRegion2	31794
CNVR24	320	2	14728311	14771718	gain	15066	13366532	131656956	Cholesterol_level	region1inRegion2	43407
CNVR25	321	2	15024071	15060448	loss	15066	13366532	131656956	Cholesterol_level	region1inRegion2	36377
CNVR26	343	2	44800086	44803691	gain	15066	13366532	131656956	Cholesterol_level	region1inRegion2	3605
CNVR27	348	2	54685639	54717573	loss-gain	15066	13366532	131656956	Cholesterol_level	region1inRegion2	31934
CNVR28	353	2	56935884	56944870	loss	15066	13366532	131656956	Cholesterol_level	region1inRegion2	8986
CNVR29	355	2	58030910	58035496	gain	15066	13366532	131656956	Cholesterol_level	region1inRegion2	4586
CNVR30	359	2	61357153	61369311	loss	15066	13366532	131656956	Cholesterol_level	region1inRegion2	12158
CNVR31	362	2	62416028	62428337	gain	15066	13366532	131656956	Cholesterol_level	region1inRegion2	12309
CNVR32	364	2	62628253	62738661	gain	15066	13366532	131656956	Cholesterol_level	region1inRegion2	110408
CNVR33	378	2	67915859	67952389	gain	15066	13366532	131656956	Cholesterol_level	region1inRegion2	36530
CNVR34	379	2	68061173	68181801	gain	15066	13366532	131656956	Cholesterol_level	region1inRegion2	120628
CNVR35	401	2	95897658	95915484	loss	15066	13366532	131656956	Cholesterol_level	region1inRegion2	17826
CNVR36	408	2	96833054	96835454	loss	15066	13366532	131656956	Cholesterol_level	region1inRegion2	2400
CNVR37	422	2	107437562	107451870	gain	15066	13366532	131656956	Cholesterol_level	region1inRegion2	14308
CNVR23	319	2	14000848	14032642	loss-gain	5685	13366532	154953107	backfat_at_last_rib	region1inRegion2	31794
CNVR24	320	2	14728311	14771718	gain	5685	13366532	154953107	backfat_at_last_rib	region1inRegion2	43407
CNVR25	321	2	15024071	15060448	loss	5685	13366532	154953107	backfat_at_last_rib	region1inRegion2	36377
CNVR26	343	2	44800086	44803691	gain	5685	13366532	154953107	backfat_at_last_rib	region1inRegion2	3605
CNVR27	348	2	54685639	54717573	loss-gain	5685	13366532	154953107	backfat_at_last_rib	region1inRegion2	31934
CNVR28	353	2	56935884	56944870	loss	5685	13366532	154953107	backfat_at_last_rib	region1inRegion2	8986
CNVR29	355	2	58030910	58035496	gain	5685	13366532	154953107	backfat_at_last_rib	region1inRegion2	4586
CNVR30	359	2	61357153	61369311	loss	5685	13366532	154953107	backfat_at_last_rib	region1inRegion2	12158
CNVR31	362	2	62416028	62428337	gain	5685	13366532	154953107	backfat_at_last_rib	region1inRegion2	12309
CNVR32	364	2	62628253	62738661	gain	5685	13366532	154953107	backfat_at_last_rib	region1inRegion2	110408
CNVR33	378	2	67915859	67952389	gain	5685	13366532	154953107	backfat_at_last_rib	region1inRegion2	36530
CNVR34	379	2	68061173	68181801	gain	5685	13366532	154953107	backfat_at_last_rib	region1inRegion2	120628
CNVR35	401	2	95897658	95915484	loss	5685	13366532	154953107	backfat_at_last_rib	region1inRegion2	17826
CNVR36	408	2	96833054	96835454	loss	5685	13366532	154953107	backfat_at_last_rib	region1inRegion2	2400
CNVR37	422	2	107437562	107451870	gain	5685	13366532	154953107	backfat_at_last_rib	region1inRegion2	14308
CNVR38	466	2	148904898	148913506	loss-gain	5685	13366532	154953107	backfat_at_last_rib	region1inRegion2	8608
CNVR39	467	2	149095217	149101135	loss	5685	13366532	154953107	backfat_at_last_rib	region1inRegion2	5918
CNVR26	343	2	44800086	44803691	gain	7596	15441523	50079118	Melanoma_susceptibility	region1inRegion2	3605
CNVR26	343	2	44800086	44803691	gain	37558	15946890	150651729	Actinobacillus_pleuropneumoniae_susceptibility	region1inRegion2	3605
CNVR27	348	2	54685639	54717573	loss-gain	37558	15946890	150651729	Actinobacillus_pleuropneumoniae_susceptibility	region1inRegion2	31934
CNVR28	353	2	56935884	56944870	loss	37558	15946890	150651729	Actinobacillus_pleuropneumoniae_susceptibility	region1inRegion2	8986
CNVR29	355	2	58030910	58035496	gain	37558	15946890	150651729	Actinobacillus_pleuropneumoniae_susceptibility	region1inRegion2	4586
CNVR30	359	2	61357153	61369311	loss	37558	15946890	150651729	Actinobacillus_pleuropneumoniae_susceptibility	region1inRegion2	12158
CNVR31	362	2	62416028	62428337	gain	37558	15946890	150651729	Actinobacillus_pleuropneumoniae_susceptibility	region1inRegion2	12309
CNVR32	364	2	62628253	62738661	gain	37558	15946890	150651729	Actinobacillus_pleuropneumoniae_susceptibility	region1inRegion2	110408
CNVR33	378	2	67915859	67952389	gain	37558	15946890	150651729	Actinobacillus_pleuropneumoniae_susceptibility	region1inRegion2	36530
CNVR34	379	2	68061173	68181801	gain	37558	15946890	150651729	Actinobacillus_pleuropneumoniae_susceptibility	region1inRegion2	120628
CNVR35	401	2	95897658	95915484	loss	37558	15946890	150651729	Actinobacillus_pleuropneumoniae_susceptibility	region1inRegion2	17826
CNVR36	408	2	96833054	96835454	loss	37558	15946890	150651729	Actinobacillus_pleuropneumoniae_susceptibility	region1inRegion2	2400
CNVR37	422	2	107437562	107451870	gain	37558	15946890	150651729	Actinobacillus_pleuropneumoniae_susceptibility	region1inRegion2	14308
CNVR38	466	2	148904898	148913506	loss-gain	37558	15946890	150651729	Actinobacillus_pleuropneumoniae_susceptibility	region1inRegion2	8608
CNVR39	467	2	149095217	149101135	loss	37558	15946890	150651729	Actinobacillus_pleuropneumoniae_susceptibility	region1inRegion2	5918
CNVR26	343	2	44800086	44803691	gain	37559	17473237	149212234	Actinobacillus_pleuropneumoniae_susceptibility	region1inRegion2	3605
CNVR27	348	2	54685639	54717573	loss-gain	37559	17473237	149212234	Actinobacillus_pleuropneumoniae_susceptibility	region1inRegion2	31934
CNVR28	353	2	56935884	56944870	loss	37559	17473237	149212234	Actinobacillus_pleuropneumoniae_susceptibility	region1inRegion2	8986
CNVR29	355	2	58030910	58035496	gain	37559	17473237	149212234	Actinobacillus_pleuropneumoniae_susceptibility	region1inRegion2	4586
CNVR30	359	2	61357153	61369311	loss	37559	17473237	149212234	Actinobacillus_pleuropneumoniae_susceptibility	region1inRegion2	12158
CNVR31	362	2	62416028	62428337	gain	37559	17473237	149212234	Actinobacillus_pleuropneumoniae_susceptibility	region1inRegion2	12309
CNVR32	364	2	62628253	62738661	gain	37559	17473237	149212234	Actinobacillus_pleuropneumoniae_susceptibility	region1inRegion2	110408
CNVR33	378	2	67915859	67952389	gain	37559	17473237	149212234	Actinobacillus_pleuropneumoniae_susceptibility	region1inRegion2	36530
CNVR34	379	2	68061173	68181801	gain	37559	17473237	149212234	Actinobacillus_pleuropneumoniae_susceptibility	region1inRegion2	120628
CNVR35	401	2	95897658	95915484	loss	37559	17473237	149212234	Actinobacillus_pleuropneumoniae_susceptibility	region1inRegion2	17826
CNVR36	408	2	96833054	96835454	loss	37559	17473237	149212234	Actinobacillus_pleuropneumoniae_susceptibility	region1inRegion2	2400
CNVR37	422	2	107437562	107451870	gain	37559	17473237	149212234	Actinobacillus_pleuropneumoniae_susceptibility	region1inRegion2	14308
CNVR38	466	2	148904898	148913506	loss-gain	37559	17473237	149212234	Actinobacillus_pleuropneumoniae_susceptibility	region1inRegion2	8608
CNVR39	467	2	149095217	149101135	loss	37559	17473237	149212234	Actinobacillus_pleuropneumoniae_susceptibility	region1inRegion2	5918
CNVR26	343	2	44800086	44803691	gain	21219	18576632	122130129	Shoulder_subcutaneous_fat_thickness	region1inRegion2	3605
CNVR27	348	2	54685639	54717573	loss-gain	21219	18576632	122130129	Shoulder_subcutaneous_fat_thickness	region1inRegion2	31934
CNVR28	353	2	56935884	56944870	loss	21219	18576632	122130129	Shoulder_subcutaneous_fat_thickness	region1inRegion2	8986
CNVR29	355	2	58030910	58035496	gain	21219	18576632	122130129	Shoulder_subcutaneous_fat_thickness	region1inRegion2	4586
CNVR30	359	2	61357153	61369311	loss	21219	18576632	122130129	Shoulder_subcutaneous_fat_thickness	region1inRegion2	12158
CNVR31	362	2	62416028	62428337	gain	21219	18576632	122130129	Shoulder_subcutaneous_fat_thickness	region1inRegion2	12309

CNVR32	364	2	62628253	62738661	gain	21219	18576632	122130129	Shoulder_subcutaneous_fat_thickness	region1inRegion2	110408
CNVR33	378	2	67915859	67952389	gain	21219	18576632	122130129	Shoulder_subcutaneous_fat_thickness	region1inRegion2	36530
CNVR34	379	2	68061173	68181801	gain	21219	18576632	122130129	Shoulder_subcutaneous_fat_thickness	region1inRegion2	120628
CNVR35	401	2	95897658	95915484	loss	21219	18576632	122130129	Shoulder_subcutaneous_fat_thickness	region1inRegion2	17826
CNVR36	408	2	96833054	96835454	loss	21219	18576632	122130129	Shoulder_subcutaneous_fat_thickness	region1inRegion2	2400
CNVR37	422	2	107437562	107451870	gain	21219	18576632	122130129	Shoulder_subcutaneous_fat_thickness	region1inRegion2	14308
CNVR26	343	2	44800086	44803691	gain	12318	19343556	150827358	Tetanus_antibody_titer	region1inRegion2	3605
CNVR27	348	2	54685639	54717573	loss-gain	12318	19343556	150827358	Tetanus_antibody_titer	region1inRegion2	31934
CNVR28	353	2	56935884	56944870	loss	12318	19343556	150827358	Tetanus_antibody_titer	region1inRegion2	8986
CNVR29	355	2	58030910	58035496	gain	12318	19343556	150827358	Tetanus_antibody_titer	region1inRegion2	4586
CNVR30	359	2	61357153	61369311	loss	12318	19343556	150827358	Tetanus_antibody_titer	region1inRegion2	12158
CNVR31	362	2	62416028	62428337	gain	12318	19343556	150827358	Tetanus_antibody_titer	region1inRegion2	12309
CNVR32	364	2	62628253	62738661	gain	12318	19343556	150827358	Tetanus_antibody_titer	region1inRegion2	110408
CNVR33	378	2	67915859	67952389	gain	12318	19343556	150827358	Tetanus_antibody_titer	region1inRegion2	36530
CNVR34	379	2	68061173	68181801	gain	12318	19343556	150827358	Tetanus_antibody_titer	region1inRegion2	120628
CNVR35	401	2	95897658	95915484	loss	12318	19343556	150827358	Tetanus_antibody_titer	region1inRegion2	17826
CNVR36	408	2	96833054	96835454	loss	12318	19343556	150827358	Tetanus_antibody_titer	region1inRegion2	2400
CNVR37	422	2	107437562	107451870	gain	12318	19343556	150827358	Tetanus_antibody_titer	region1inRegion2	14308
CNVR38	466	2	148904898	148913506	loss-gain	12318	19343556	150827358	Tetanus_antibody_titer	region1inRegion2	8608
CNVR39	467	2	149095217	149101135	loss	12318	19343556	150827358	Tetanus_antibody_titer	region1inRegion2	5918
CNVR26	343	2	44800086	44803691	gain	37568	19681588	149212234	Actinobacillus_pleuropneumoniae_susceptibility	region1inRegion2	3605
CNVR27	348	2	54685639	54717573	loss-gain	37568	19681588	149212234	Actinobacillus_pleuropneumoniae_susceptibility	region1inRegion2	31934
CNVR28	353	2	56935884	56944870	loss	37568	19681588	149212234	Actinobacillus_pleuropneumoniae_susceptibility	region1inRegion2	8986
CNVR29	355	2	58030910	58035496	gain	37568	19681588	149212234	Actinobacillus_pleuropneumoniae_susceptibility	region1inRegion2	4586
CNVR30	359	2	61357153	61369311	loss	37568	19681588	149212234	Actinobacillus_pleuropneumoniae_susceptibility	region1inRegion2	12158
CNVR31	362	2	62416028	62428337	gain	37568	19681588	149212234	Actinobacillus_pleuropneumoniae_susceptibility	region1inRegion2	12309
CNVR32	364	2	62628253	62738661	gain	37568	19681588	149212234	Actinobacillus_pleuropneumoniae_susceptibility	region1inRegion2	110408
CNVR33	378	2	67915859	67952389	gain	37568	19681588	149212234	Actinobacillus_pleuropneumoniae_susceptibility	region1inRegion2	36530
CNVR34	379	2	68061173	68181801	gain	37568	19681588	149212234	Actinobacillus_pleuropneumoniae_susceptibility	region1inRegion2	120628
CNVR35	401	2	95897658	95915484	loss	37568	19681588	149212234	Actinobacillus_pleuropneumoniae_susceptibility	region1inRegion2	17826
CNVR36	408	2	96833054	96835454	loss	37568	19681588	149212234	Actinobacillus_pleuropneumoniae_susceptibility	region1inRegion2	2400
CNVR37	422	2	107437562	107451870	gain	37568	19681588	149212234	Actinobacillus_pleuropneumoniae_susceptibility	region1inRegion2	14308
CNVR38	466	2	148904898	148913506	loss-gain	37568	19681588	149212234	Actinobacillus_pleuropneumoniae_susceptibility	region1inRegion2	8608
CNVR39	467	2	149095217	149101135	loss	37568	19681588	149212234	Actinobacillus_pleuropneumoniae_susceptibility	region1inRegion2	5918
CNVR26	343	2	44800086	44803691	gain	21324	21136167	53611728	White_blood_cell_counts	region1inRegion2	3605
CNVR26	343	2	44800086	44803691	gain	21331	21136167	53611728	White_blood_cell_counts	region1inRegion2	3605
CNVR26	343	2	44800086	44803691	gain	17610	21136167	59648728	Interferon-gamma_level	region1inRegion2	3605
CNVR27	348	2	54685639	54717573	loss-gain	17610	21136167	59648728	Interferon-gamma_level	region1inRegion2	31934
CNVR28	353	2	56935884	56944870	loss	17610	21136167	59648728	Interferon-gamma_level	region1inRegion2	8986
CNVR29	355	2	58030910	58035496	gain	17610	21136167	59648728	Interferon-gamma_level	region1inRegion2	4586
CNVR26	343	2	44800086	44803691	gain	307	21136167	59648728	Average_backfat_thickness	region1inRegion2	3605
CNVR27	348	2	54685639	54717573	loss-gain	307	21136167	59648728	Average_backfat_thickness	region1inRegion2	31934
CNVR28	353	2	56935884	56944870	loss	307	21136167	59648728	Average_backfat_thickness	region1inRegion2	8986
CNVR29	355	2	58030910	58035496	gain	307	21136167	59648728	Average_backfat_thickness	region1inRegion2	4586
CNVR26	343	2	44800086	44803691	gain	16883	21136167	75040316	backfat_at_last_rib	region1inRegion2	3605
CNVR27	348	2	54685639	54717573	loss-gain	16883	21136167	75040316	backfat_at_last_rib	region1inRegion2	31934
CNVR28	353	2	56935884	56944870	loss	16883	21136167	75040316	backfat_at_last_rib	region1inRegion2	8986
CNVR29	355	2	58030910	58035496	gain	16883	21136167	75040316	backfat_at_last_rib	region1inRegion2	4586
CNVR30	359	2	61357153	61369311	loss	16883	21136167	75040316	backfat_at_last_rib	region1inRegion2	12158
CNVR31	362	2	62416028	62428337	gain	16883	21136167	75040316	backfat_at_last_rib	region1inRegion2	12309
CNVR32	364	2	62628253	62738661	gain	16883	21136167	75040316	backfat_at_last_rib	region1inRegion2	110408
CNVR33	378	2	67915859	67952389	gain	16883	21136167	75040316	backfat_at_last_rib	region1inRegion2	36530
CNVR34	379	2	68061173	68181801	gain	16883	21136167	75040316	backfat_at_last_rib	region1inRegion2	120628
CNVR26	343	2	44800086	44803691	gain	16898	21136167	75040316	Carcass_weight_(hot)	region1inRegion2	3605
CNVR27	348	2	54685639	54717573	loss-gain	16898	21136167	75040316	Carcass_weight_(hot)	region1inRegion2	31934
CNVR28	353	2	56935884	56944870	loss	16898	21136167	75040316	Carcass_weight_(hot)	region1inRegion2	8986
CNVR29	355	2	58030910	58035496	gain	16898	21136167	75040316	Carcass_weight_(hot)	region1inRegion2	4586
CNVR30	359	2	61357153	61369311	loss	16898	21136167	75040316	Carcass_weight_(hot)	region1inRegion2	12158
CNVR31	362	2	62416028	62428337	gain	16898	21136167	75040316	Carcass_weight_(hot)	region1inRegion2	12309
CNVR32	364	2	62628253	62738661	gain	16898	21136167	75040316	Carcass_weight_(hot)	region1inRegion2	110408
CNVR33	378	2	67915859	67952389	gain	16898	21136167	75040316	Carcass_weight_(hot)	region1inRegion2	36530
CNVR34	379	2	68061173	68181801	gain	16898	21136167	75040316	Carcass_weight_(hot)	region1inRegion2	120628
CNVR26	343	2	44800086	44803691	gain	4025	21136167	75040316	Percentage_type_IIa_fibers	region1inRegion2	3605
CNVR27	348	2	54685639	54717573	loss-gain	4025	21136167	75040316	Percentage_type_IIa_fibers	region1inRegion2	31934
CNVR28	353	2	56935884	56944870	loss	4025	21136167	75040316	Percentage_type_IIa_fibers	region1inRegion2	8986
CNVR29	355	2	58030910	58035496	gain	4025	21136167	75040316	Percentage_type_IIa_fibers	region1inRegion2	4586
CNVR30	359	2	61357153	61369311	loss	4025	21136167	75040316	Percentage_type_IIa_fibers	region1inRegion2	12158
CNVR31	362	2	62416028	62428337	gain	4025	21136167	75040316	Percentage_type_IIa_fibers	region1inRegion2	12309
CNVR32	364	2	62628253	62738661	gain	4025	21136167	75040316	Percentage_type_IIa_fibers	region1inRegion2	110408
CNVR33	378	2	67915859	67952389	gain	4025	21136167	75040316	Percentage_type_IIa_fibers	region1inRegion2	36530
CNVR34	379	2	68061173	68181801	gain	4025	21136167	75040316	Percentage_type_IIa_fibers	region1inRegion2	120628
CNVR26	343	2	44800086	44803691	gain	12747	21136167	119359590	Fat-cuts_percentage	region1inRegion2	3605
CNVR27	348	2	54685639	54717573	loss-gain	12747	21136167	119359590	Fat-cuts_percentage	region1inRegion2	31934
CNVR28	353	2	56935884	56944870	loss	12747	21136167	119359590	Fat-cuts_percentage	region1inRegion2	8986
CNVR29	355	2	58030910	58035496	gain	12747	21136167	119359590	Fat-cuts_percentage	region1inRegion2	4586
CNVR30	359	2	61357153	61369311	loss	12747	21136167	119359590	Fat-cuts_percentage	region1inRegion2	12158
CNVR31	362	2	62416028	62428337	gain	12747	21136167	119359590	Fat-cuts_percentage	region1inRegion2	12309
CNVR32	364	2	62628253	62738661	gain	12747	21136167	119359590	Fat-cuts_percentage	region1inRegion2	110408
CNVR33	378	2	67915859	67952389	gain	12747	21136167	119359590	Fat-cuts_percentage	region1inRegion2	36530
CNVR34	379	2	68061173	68181801	gain	12747	21136167	119359590	Fat-cuts_percentage	region1inRegion2	120628
CNVR35	401	2	95897658	95915484	loss	12747	21136167	119359590	Fat-cuts_percentage	region1inRegion2	17826
CNVR36	408	2	96833054	96835454	loss	12747	21136167	119359590	Fat-cuts_percentage	region1inRegion2	2400
CNVR37	422	2	107437562	107451870	gain	12747	21136167	119359590	Fat-cuts_percentage	region1inRegion2	14308
CNVR26	343	2	44800086	44803691	gain	12748	21136167	119359590	Fat_to_meat_ratio	region1inRegion2	3605
CNVR27	348	2	54685639	54717573	loss-gain	12748	21136167	119359590	Fat_to_meat_ratio	region1inRegion2	31934
CNVR28	353	2	56935884	56944870	loss	12748	21136167	119359590	Fat_to_meat_ratio	region1inRegion2	8986
CNVR29	355	2	58030910	58035496	gain	12748	21136167	119359590	Fat_to_meat_ratio	region1inRegion2	4586
CNVR30	359	2	61357153	61369311	loss	12748	21136167	119359590	Fat_to_meat_ratio	region1inRegion2	12158
CNVR31	362	2	62416028	62428337	gain	12748	21136167	119359590	Fat_to_meat_ratio	region1inRegion2	12309
CNVR32	364	2	62628253	62738661	gain	12748	21136167	119359590	Fat_to_meat_ratio	region1inRegion2	110408
CNVR33	378	2	67915859	67952389	gain	12748	21136167	119359590	Fat_to_meat_ratio	region1inRegion2	36530
CNVR34	379	2	68061173	68181801	gain	12748	21136167	119359590	Fat_to_meat_ratio	region1inRegion2	120628
CNVR35	401	2	95897658	95915484	loss	12748	21136167	119359590	Fat_to_meat_ratio	region1inRegion2	17826
CNVR36	408	2	96833054	96835454	loss	12748	21136167	119359590	Fat_to_meat_ratio	region1inRegion2	2400
CNVR37	422	2	107437562	107451870	gain	12748	21136167	119359590	Fat_to_meat_ratio	region1inRegion2	14308
CNVR26	343	2	44800086	44803691	gain	16888	21136167	119359590	Average_daily_gain	region1inRegion2	3605
CNVR27	348	2	54685639	54717573	loss-gain	16888	21136167	119359590	Average_daily_gain	region1inRegion2	31934
CNVR28	353	2	56935884	56944870	loss	16888	21136167	119359590	Average_daily_gain	region1inRegion2	8986
CNVR29	355	2	58030910	58035496	gain	16888	21136167	119359590	Average_daily_gain	region1inRegion2	4586
CNVR30	359	2	61357153	61369311	loss	16888	21136167	119359590	Average_daily_gain	region1inRegion2	12158
CNVR31	362	2	62416028	62428337	gain	16888	21136167	119359590	Average_daily_gain	region1inRegion2	12309
CNVR32	364	2	62628253	62738661	gain	16888	21136167	119359590	Average_daily_gain	region1inRegion2	110408
CNVR33	378	2	67915859	67952389	gain	16888	21136167	119359590	Average_daily_gain	region1inRegion2	36530
CNVR34	379	2	68061173	68181801	gain	16888	21136167	119359590	Average_daily_gain	region1inRegion2	120628
CNVR35	401	2	95897658	95915484	loss	16888	21136167	119359590	Average_daily_gain	region1inRegion2	17826
CNVR36	408	2	96833054	96835454	loss	16888	21136167	119359590	Average_daily_gain	region1inRegion2	2400
CNVR37	422	2	107437562	107451870	gain	16888	21136167	119359590	Average_daily_gain	region1inRegion2	14308
CNVR26	343	2	44800086	44803691	gain	4205	21136167	122791407	androstenone,_laboratory	region1inRegion2	3605
CNVR27	348	2	54685639	54717573	loss-gain	4205	21136167	122791407	androstenone,_laboratory	region1inRegion2	31934
CNVR28	353	2	56935884	56944870	loss	4205	21136167	122791407	androstenone,_laboratory	region1inRegion2	8986
CNVR29	355	2	58030910	58035496	gain	4205	21136167	122791407	androstenone,_laboratory	region1inRegion2	4586
CNVR30	359	2	61357153	61369311	loss	4205	21136167	122791407	androstenone,_laboratory	region1inRegion2	12158
CNVR31	362	2	62416028	62428337	gain	4205	21136167	122791407	androstenone,_laboratory	region1inRegion2	12309
CNVR32	364	2	62628253	62738661	gain	4205	21136167	122791407	androstenone,_laboratory	region1inRegion2	110408
CNVR33	378	2	67915859	67952389	gain	4205	21136167	122791407	androstenone,_laboratory	region1inRegion2	36530
CNVR34	379	2	68061173	68181801	gain	4205	21136167	122791407	androstenone,_laboratory	region1inRegion2	120628
CNVR35	401	2	95897658	95915484	loss	4205	21136167	122791407	androstenone,_laboratory	region1inRegion2	17826
CNVR36	408	2	96833054	96835454	loss	4205	21136167	122791407	androstenone,_laboratory	region1inRegion2	2400
CNVR37	422	2	107437562	107451870	gain	4205	21136167	122791407	androstenone,_laboratory	region1inRegion2	14308
CNVR26	343	2	44800086	44803691	gain	29690	21136167	139359663	Carcass_length	region1inRegion2	3605
CNVR27	348	2	54685639	54717573	loss-gain	29690	21136167	139359663	Carcass_length	region1inRegion2	31934
CNVR28	353	2	56935884	56944870	loss	29690	21136167	139359663	Carcass_length	region1inRegion2	8986

CNVR29	355	2	58030910	58035496	gain	29690	21136167	139359663	Carcass_length	region1inRegion2	4586
CNVR30	359	2	61357153	61369311	loss	29690	21136167	139359663	Carcass_length	region1inRegion2	12158
CNVR31	362	2	62416028	62428337	gain	29690	21136167	139359663	Carcass_length	region1inRegion2	12309
CNVR32	364	2	62628253	62738661	gain	29690	21136167	139359663	Carcass_length	region1inRegion2	110408
CNVR33	378	2	67915859	67952389	gain	29690	21136167	139359663	Carcass_length	region1inRegion2	36530
CNVR34	379	2	68061173	68181801	gain	29690	21136167	139359663	Carcass_length	region1inRegion2	120628
CNVR35	401	2	95897658	95915484	loss	29690	21136167	139359663	Carcass_length	region1inRegion2	17826
CNVR36	408	2	96833054	96835454	loss	29690	21136167	139359663	Carcass_length	region1inRegion2	2400
CNVR37	422	2	107437562	107451870	gain	29690	21136167	139359663	Carcass_length	region1inRegion2	14308
CNVR26	343	2	44800086	44803691	gain	37560	23613810	149813245	Actinobacillus_pleuropneumoniae_susceptibility	region1inRegion2	3605
CNVR27	348	2	54685639	54717573	loss-gain	37560	23613810	149813245	Actinobacillus_pleuropneumoniae_susceptibility	region1inRegion2	31934
CNVR28	353	2	56935884	56944870	loss	37560	23613810	149813245	Actinobacillus_pleuropneumoniae_susceptibility	region1inRegion2	8986
CNVR29	355	2	58030910	58035496	gain	37560	23613810	149813245	Actinobacillus_pleuropneumoniae_susceptibility	region1inRegion2	4586
CNVR30	359	2	61357153	61369311	loss	37560	23613810	149813245	Actinobacillus_pleuropneumoniae_susceptibility	region1inRegion2	12158
CNVR31	362	2	62416028	62428337	gain	37560	23613810	149813245	Actinobacillus_pleuropneumoniae_susceptibility	region1inRegion2	12309
CNVR32	364	2	62628253	62738661	gain	37560	23613810	149813245	Actinobacillus_pleuropneumoniae_susceptibility	region1inRegion2	110408
CNVR33	378	2	67915859	67952389	gain	37560	23613810	149813245	Actinobacillus_pleuropneumoniae_susceptibility	region1inRegion2	36530
CNVR34	379	2	68061173	68181801	gain	37560	23613810	149813245	Actinobacillus_pleuropneumoniae_susceptibility	region1inRegion2	120628
CNVR35	401	2	95897658	95915484	loss	37560	23613810	149813245	Actinobacillus_pleuropneumoniae_susceptibility	region1inRegion2	17826
CNVR36	408	2	96833054	96835454	loss	37560	23613810	149813245	Actinobacillus_pleuropneumoniae_susceptibility	region1inRegion2	2400
CNVR37	422	2	107437562	107451870	gain	37560	23613810	149813245	Actinobacillus_pleuropneumoniae_susceptibility	region1inRegion2	14308
CNVR38	466	2	148904898	148913506	loss-gain	37560	23613810	149813245	Actinobacillus_pleuropneumoniae_susceptibility	region1inRegion2	8608
CNVR39	467	2	149095217	149101135	loss	37560	23613810	149813245	Actinobacillus_pleuropneumoniae_susceptibility	region1inRegion2	5918
CNVR26	343	2	44800086	44803691	gain	3940	25252518	48775112	Semimembranosus_angle	region1inRegion2	3605
CNVR26	343	2	44800086	44803691	gain	9580	25252518	64352885	Linoleic_acid_content	region1inRegion2	3605
CNVR27	348	2	54685639	54717573	loss-gain	9580	25252518	64352885	Linoleic_acid_content	region1inRegion2	31934
CNVR28	353	2	56935884	56944870	loss	9580	25252518	64352885	Linoleic_acid_content	region1inRegion2	8986
CNVR29	355	2	58030910	58035496	gain	9580	25252518	64352885	Linoleic_acid_content	region1inRegion2	4586
CNVR30	359	2	61357153	61369311	loss	9580	25252518	64352885	Linoleic_acid_content	region1inRegion2	12158
CNVR31	362	2	62416028	62428337	gain	9580	25252518	64352885	Linoleic_acid_content	region1inRegion2	12309
CNVR32	364	2	62628253	62738661	gain	9580	25252518	64352885	Linoleic_acid_content	region1inRegion2	110408
CNVR26	343	2	44800086	44803691	gain	37561	26478024	147163597	Actinobacillus_pleuropneumoniae_susceptibility	region1inRegion2	3605
CNVR27	348	2	54685639	54717573	loss-gain	37561	26478024	147163597	Actinobacillus_pleuropneumoniae_susceptibility	region1inRegion2	31934
CNVR28	353	2	56935884	56944870	loss	37561	26478024	147163597	Actinobacillus_pleuropneumoniae_susceptibility	region1inRegion2	8986
CNVR29	355	2	58030910	58035496	gain	37561	26478024	147163597	Actinobacillus_pleuropneumoniae_susceptibility	region1inRegion2	4586
CNVR30	359	2	61357153	61369311	loss	37561	26478024	147163597	Actinobacillus_pleuropneumoniae_susceptibility	region1inRegion2	12158
CNVR31	362	2	62416028	62428337	gain	37561	26478024	147163597	Actinobacillus_pleuropneumoniae_susceptibility	region1inRegion2	12309
CNVR32	364	2	62628253	62738661	gain	37561	26478024	147163597	Actinobacillus_pleuropneumoniae_susceptibility	region1inRegion2	110408
CNVR33	378	2	67915859	67952389	gain	37561	26478024	147163597	Actinobacillus_pleuropneumoniae_susceptibility	region1inRegion2	36530
CNVR34	379	2	68061173	68181801	gain	37561	26478024	147163597	Actinobacillus_pleuropneumoniae_susceptibility	region1inRegion2	120628
CNVR35	401	2	95897658	95915484	loss	37561	26478024	147163597	Actinobacillus_pleuropneumoniae_susceptibility	region1inRegion2	17826
CNVR36	408	2	96833054	96835454	loss	37561	26478024	147163597	Actinobacillus_pleuropneumoniae_susceptibility	region1inRegion2	2400
CNVR37	422	2	107437562	107451870	gain	37561	26478024	147163597	Actinobacillus_pleuropneumoniae_susceptibility	region1inRegion2	14308
CNVR26	343	2	44800086	44803691	gain	6313	29455997	91623387	Platelet_count	region1inRegion2	3605
CNVR27	348	2	54685639	54717573	loss-gain	6313	29455997	91623387	Platelet_count	region1inRegion2	31934
CNVR28	353	2	56935884	56944870	loss	6313	29455997	91623387	Platelet_count	region1inRegion2	8986
CNVR29	355	2	58030910	58035496	gain	6313	29455997	91623387	Platelet_count	region1inRegion2	4586
CNVR30	359	2	61357153	61369311	loss	6313	29455997	91623387	Platelet_count	region1inRegion2	12158
CNVR31	362	2	62416028	62428337	gain	6313	29455997	91623387	Platelet_count	region1inRegion2	12309
CNVR32	364	2	62628253	62738661	gain	6313	29455997	91623387	Platelet_count	region1inRegion2	110408
CNVR33	378	2	67915859	67952389	gain	6313	29455997	91623387	Platelet_count	region1inRegion2	36530
CNVR34	379	2	68061173	68181801	gain	6313	29455997	91623387	Platelet_count	region1inRegion2	120628
CNVR26	343	2	44800086	44803691	gain	7313	29455997	91623387	pH_24_hr_post_mortem_(ham)	region1inRegion2	3605
CNVR27	348	2	54685639	54717573	loss-gain	7313	29455997	91623387	pH_24_hr_post_mortem_(ham)	region1inRegion2	31934
CNVR28	353	2	56935884	56944870	loss	7313	29455997	91623387	pH_24_hr_post_mortem_(ham)	region1inRegion2	8986
CNVR29	355	2	58030910	58035496	gain	7313	29455997	91623387	pH_24_hr_post_mortem_(ham)	region1inRegion2	4586
CNVR30	359	2	61357153	61369311	loss	7313	29455997	91623387	pH_24_hr_post_mortem_(ham)	region1inRegion2	12158
CNVR31	362	2	62416028	62428337	gain	7313	29455997	91623387	pH_24_hr_post_mortem_(ham)	region1inRegion2	12309
CNVR32	364	2	62628253	62738661	gain	7313	29455997	91623387	pH_24_hr_post_mortem_(ham)	region1inRegion2	110408
CNVR33	378	2	67915859	67952389	gain	7313	29455997	91623387	pH_24_hr_post_mortem_(ham)	region1inRegion2	36530
CNVR34	379	2	68061173	68181801	gain	7313	29455997	91623387	pH_24_hr_post_mortem_(ham)	region1inRegion2	120628
CNVR26	343	2	44800086	44803691	gain	3107	29530258	53611728	Fat_to_meat_ratio	region1inRegion2	3605
CNVR26	343	2	44800086	44803691	gain	669	33031443	123480352	Loin_muscle_depth	region1inRegion2	3605
CNVR27	348	2	54685639	54717573	loss-gain	669	33031443	123480352	Loin_muscle_depth	region1inRegion2	31934
CNVR28	353	2	56935884	56944870	loss	669	33031443	123480352	Loin_muscle_depth	region1inRegion2	8986
CNVR29	355	2	58030910	58035496	gain	669	33031443	123480352	Loin_muscle_depth	region1inRegion2	4586
CNVR30	359	2	61357153	61369311	loss	669	33031443	123480352	Loin_muscle_depth	region1inRegion2	12158
CNVR31	362	2	62416028	62428337	gain	669	33031443	123480352	Loin_muscle_depth	region1inRegion2	12309
CNVR32	364	2	62628253	62738661	gain	669	33031443	123480352	Loin_muscle_depth	region1inRegion2	110408
CNVR33	378	2	67915859	67952389	gain	669	33031443	123480352	Loin_muscle_depth	region1inRegion2	36530
CNVR34	379	2	68061173	68181801	gain	669	33031443	123480352	Loin_muscle_depth	region1inRegion2	120628
CNVR35	401	2	95897658	95915484	loss	669	33031443	123480352	Loin_muscle_depth	region1inRegion2	17826
CNVR36	408	2	96833054	96835454	loss	669	33031443	123480352	Loin_muscle_depth	region1inRegion2	2400
CNVR37	422	2	107437562	107451870	gain	669	33031443	123480352	Loin_muscle_depth	region1inRegion2	14308
CNVR26	343	2	44800086	44803691	gain	15856	34221548	75082926	Stearic_acid_content	region1inRegion2	3605
CNVR27	348	2	54685639	54717573	loss-gain	15856	34221548	75082926	Stearic_acid_content	region1inRegion2	31934
CNVR28	353	2	56935884	56944870	loss	15856	34221548	75082926	Stearic_acid_content	region1inRegion2	8986
CNVR29	355	2	58030910	58035496	gain	15856	34221548	75082926	Stearic_acid_content	region1inRegion2	4586
CNVR30	359	2	61357153	61369311	loss	15856	34221548	75082926	Stearic_acid_content	region1inRegion2	12158
CNVR31	362	2	62416028	62428337	gain	15856	34221548	75082926	Stearic_acid_content	region1inRegion2	12309
CNVR32	364	2	62628253	62738661	gain	15856	34221548	75082926	Stearic_acid_content	region1inRegion2	110408
CNVR33	378	2	67915859	67952389	gain	15856	34221548	75082926	Stearic_acid_content	region1inRegion2	36530
CNVR34	379	2	68061173	68181801	gain	15856	34221548	75082926	Stearic_acid_content	region1inRegion2	120628
CNVR26	343	2	44800086	44803691	gain	15857	34221548	75082926	Linoleic_acid_content	region1inRegion2	3605
CNVR27	348	2	54685639	54717573	loss-gain	15857	34221548	75082926	Linoleic_acid_content	region1inRegion2	31934
CNVR28	353	2	56935884	56944870	loss	15857	34221548	75082926	Linoleic_acid_content	region1inRegion2	8986
CNVR29	355	2	58030910	58035496	gain	15857	34221548	75082926	Linoleic_acid_content	region1inRegion2	4586
CNVR30	359	2	61357153	61369311	loss	15857	34221548	75082926	Linoleic_acid_content	region1inRegion2	12158
CNVR31	362	2	62416028	62428337	gain	15857	34221548	75082926	Linoleic_acid_content	region1inRegion2	12309
CNVR32	364	2	62628253	62738661	gain	15857	34221548	75082926	Linoleic_acid_content	region1inRegion2	110408
CNVR33	378	2	67915859	67952389	gain	15857	34221548	75082926	Linoleic_acid_content	region1inRegion2	36530
CNVR34	379	2	68061173	68181801	gain	15857	34221548	75082926	Linoleic_acid_content	region1inRegion2	120628
CNVR26	343	2	44800086	44803691	gain	3778	34221548	100385940	Body_weight_(16_days)	region1inRegion2	3605
CNVR27	348	2	54685639	54717573	loss-gain	3778	34221548	100385940	Body_weight_(16_days)	region1inRegion2	31934
CNVR28	353	2	56935884	56944870	loss	3778	34221548	100385940	Body_weight_(16_days)	region1inRegion2	8986
CNVR29	355	2	58030910	58035496	gain	3778	34221548	100385940	Body_weight_(16_days)	region1inRegion2	4586
CNVR30	359	2	61357153	61369311	loss	3778	34221548	100385940	Body_weight_(16_days)	region1inRegion2	12158
CNVR31	362	2	62416028	62428337	gain	3778	34221548	100385940	Body_weight_(16_days)	region1inRegion2	12309
CNVR32	364	2	62628253	62738661	gain	3778	34221548	100385940	Body_weight_(16_days)	region1inRegion2	110408
CNVR33	378	2	67915859	67952389	gain	3778	34221548	100385940	Body_weight_(16_days)	region1inRegion2	36530
CNVR34	379	2	68061173	68181801	gain	3778	34221548	100385940	Body_weight_(16_days)	region1inRegion2	120628
CNVR35	401	2	95897658	95915484	loss	3778	34221548	100385940	Body_weight_(16_days)	region1inRegion2	17826
CNVR36	408	2	96833054	96835454	loss	3778	34221548	100385940	Body_weight_(16_days)	region1inRegion2	2400
CNVR26	343	2	44800086	44803691	gain	3779	34221548	133160620	Average_daily_gain	region1inRegion2	3605
CNVR27	348	2	54685639	54717573	loss-gain	3779	34221548	133160620	Average_daily_gain	region1inRegion2	31934
CNVR28	353	2	56935884	56944870	loss	3779	34221548	133160620	Average_daily_gain	region1inRegion2	8986
CNVR29	355	2	58030910	58035496	gain	3779	34221548	133160620	Average_daily_gain	region1inRegion2	4586
CNVR30	359	2	61357153	61369311	loss	3779	34221548	133160620	Average_daily_gain	region1inRegion2	12158
CNVR31	362	2	62416028	62428337	gain	3779	34221548	133160620	Average_daily_gain	region1inRegion2	12309
CNVR32	364	2	62628253	62738661	gain	3779	34221548	133160620	Average_daily_gain	region1inRegion2	110408
CNVR33	378	2	67915859	67952389	gain	3779	34221548	133160620	Average_daily_gain	region1inRegion2	36530
CNVR34	379	2	68061173	68181801	gain	3779	34221548	133160620	Average_daily_gain	region1inRegion2	120628
CNVR35	401	2	95897658	95915484	loss	3779	34221548	133160620	Average_daily_gain	region1inRegion2	17826
CNVR36	408	2	96833054	96835454	loss	3779	34221548	133160620	Average_daily_gain	region1inRegion2	2400
CNVR37	422	2	107437562	107451870	gain	3779	34221548	133160620	Average_daily_gain	region1inRegion2	14308
CNVR26	343	2	44800086	44803691	gain	21354	35687241	48775112	Juiciness_score	region1inRegion2	3605
CNVR26	343	2	44800086	44803691	gain	3224	35687241	48775112	Juiciness_score	region1inRegion2	3605
CNVR26	343	2	44800086	44803691	gain	7219	35744022	90402970	Red_cell_distribution_width	region1inRegion2	3605
CNVR27	348	2	54685639	54717573	loss-gain	7219	35744022	90402970	Red_cell_distribution_width	region1inRegion2	31934
CNVR28	353	2	56935884	56944870	loss	7219	35744022	90402970	Red_cell_distribution_width	region1inRegion2	8986
CNVR29	355	2	58030910	58035496	gain	7219	35744022	90402970	Red_cell_distribution_width	region1inRegion2	4586
CNVR30	359	2	61357153	61369311	loss	7219	35744022	90402970	Red_cell_distribution_width	region1inRegion2	12158

CNVR31	362	2	62416028	62428337	gain	7219	35744022	90402970	Red_cell_distribution_width	region1inRegion2	12309
CNVR32	364	2	62628253	62738661	gain	7219	35744022	90402970	Red_cell_distribution_width	region1inRegion2	110408
CNVR33	378	2	67915859	67952389	gain	7219	35744022	90402970	Red_cell_distribution_width	region1inRegion2	36530
CNVR34	379	2	68061173	68181801	gain	7219	35744022	90402970	Red_cell_distribution_width	region1inRegion2	120628
CNVR26	343	2	44800086	44803691	gain	5936	38367026	59648728	Body_weight_(slaughter)	region1inRegion2	3605
CNVR27	348	2	54685639	54717573	loss-gain	5936	38367026	59648728	Body_weight_(slaughter)	region1inRegion2	31934
CNVR28	353	2	56935884	56944870	loss	5936	38367026	59648728	Body_weight_(slaughter)	region1inRegion2	8986
CNVR29	355	2	58030910	58035496	gain	5936	38367026	59648728	Body_weight_(slaughter)	region1inRegion2	4586
CNVR26	343	2	44800086	44803691	gain	5962	38367026	59648728	Shoulder_subcutaneous_fat_thickness	region1inRegion2	3605
CNVR27	348	2	54685639	54717573	loss-gain	5962	38367026	59648728	Shoulder_subcutaneous_fat_thickness	region1inRegion2	31934
CNVR28	353	2	56935884	56944870	loss	5962	38367026	59648728	Shoulder_subcutaneous_fat_thickness	region1inRegion2	8986
CNVR29	355	2	58030910	58035496	gain	5962	38367026	59648728	Shoulder_subcutaneous_fat_thickness	region1inRegion2	4586
CNVR26	343	2	44800086	44803691	gain	88	38367026	100385940	Water_holding_capacity	region1inRegion2	3605
CNVR27	348	2	54685639	54717573	loss-gain	88	38367026	100385940	Water_holding_capacity	region1inRegion2	31934
CNVR28	353	2	56935884	56944870	loss	88	38367026	100385940	Water_holding_capacity	region1inRegion2	8986
CNVR29	355	2	58030910	58035496	gain	88	38367026	100385940	Water_holding_capacity	region1inRegion2	4586
CNVR30	359	2	61357153	61369311	loss	88	38367026	100385940	Water_holding_capacity	region1inRegion2	12158
CNVR31	362	2	62416028	62428337	gain	88	38367026	100385940	Water_holding_capacity	region1inRegion2	12309
CNVR32	364	2	62628253	62738661	gain	88	38367026	100385940	Water_holding_capacity	region1inRegion2	110408
CNVR33	378	2	67915859	67952389	gain	88	38367026	100385940	Water_holding_capacity	region1inRegion2	36530
CNVR34	379	2	68061173	68181801	gain	88	38367026	100385940	Water_holding_capacity	region1inRegion2	120628
CNVR35	401	2	95897658	95915484	loss	88	38367026	100385940	Water_holding_capacity	region1inRegion2	17826
CNVR36	408	2	96833054	96835454	loss	88	38367026	100385940	Water_holding_capacity	region1inRegion2	2400
CNVR26	343	2	44800086	44803691	gain	82	38367026	119359590	Meat_color-L	region1inRegion2	3605
CNVR27	348	2	54685639	54717573	loss-gain	82	38367026	119359590	Meat_color-L	region1inRegion2	31934
CNVR28	353	2	56935884	56944870	loss	82	38367026	119359590	Meat_color-L	region1inRegion2	8986
CNVR29	355	2	58030910	58035496	gain	82	38367026	119359590	Meat_color-L	region1inRegion2	4586
CNVR30	359	2	61357153	61369311	loss	82	38367026	119359590	Meat_color-L	region1inRegion2	12158
CNVR31	362	2	62416028	62428337	gain	82	38367026	119359590	Meat_color-L	region1inRegion2	12309
CNVR32	364	2	62628253	62738661	gain	82	38367026	119359590	Meat_color-L	region1inRegion2	110408
CNVR33	378	2	67915859	67952389	gain	82	38367026	119359590	Meat_color-L	region1inRegion2	36530
CNVR34	379	2	68061173	68181801	gain	82	38367026	119359590	Meat_color-L	region1inRegion2	120628
CNVR35	401	2	95897658	95915484	loss	82	38367026	119359590	Meat_color-L	region1inRegion2	17826
CNVR36	408	2	96833054	96835454	loss	82	38367026	119359590	Meat_color-L	region1inRegion2	2400
CNVR37	422	2	107437562	107451870	gain	82	38367026	119359590	Meat_color-L	region1inRegion2	14308
CNVR26	343	2	44800086	44803691	gain	12317	38367026	151463344	Mycoplasma_hyopneumoniae_antibody_titer	region1inRegion2	3605
CNVR27	348	2	54685639	54717573	loss-gain	12317	38367026	151463344	Mycoplasma_hyopneumoniae_antibody_titer	region1inRegion2	31934
CNVR28	353	2	56935884	56944870	loss	12317	38367026	151463344	Mycoplasma_hyopneumoniae_antibody_titer	region1inRegion2	8986
CNVR29	355	2	58030910	58035496	gain	12317	38367026	151463344	Mycoplasma_hyopneumoniae_antibody_titer	region1inRegion2	4586
CNVR30	359	2	61357153	61369311	loss	12317	38367026	151463344	Mycoplasma_hyopneumoniae_antibody_titer	region1inRegion2	12158
CNVR31	362	2	62416028	62428337	gain	12317	38367026	151463344	Mycoplasma_hyopneumoniae_antibody_titer	region1inRegion2	12309
CNVR32	364	2	62628253	62738661	gain	12317	38367026	151463344	Mycoplasma_hyopneumoniae_antibody_titer	region1inRegion2	110408
CNVR33	378	2	67915859	67952389	gain	12317	38367026	151463344	Mycoplasma_hyopneumoniae_antibody_titer	region1inRegion2	36530
CNVR34	379	2	68061173	68181801	gain	12317	38367026	151463344	Mycoplasma_hyopneumoniae_antibody_titer	region1inRegion2	120628
CNVR35	401	2	95897658	95915484	loss	12317	38367026	151463344	Mycoplasma_hyopneumoniae_antibody_titer	region1inRegion2	17826
CNVR36	408	2	96833054	96835454	loss	12317	38367026	151463344	Mycoplasma_hyopneumoniae_antibody_titer	region1inRegion2	2400
CNVR37	422	2	107437562	107451870	gain	12317	38367026	151463344	Mycoplasma_hyopneumoniae_antibody_titer	region1inRegion2	14308
CNVR38	466	2	148904898	148913506	loss-gain	12317	38367026	151463344	Mycoplasma_hyopneumoniae_antibody_titer	region1inRegion2	8608
CNVR39	467	2	149095217	149101135	loss	12317	38367026	151463344	Mycoplasma_hyopneumoniae_antibody_titer	region1inRegion2	5918
CNVR26	343	2	44800086	44803691	gain	5682	38367026	157789611	pH_for_Semimembranosus	region1inRegion2	3605
CNVR27	348	2	54685639	54717573	loss-gain	5682	38367026	157789611	pH_for_Semimembranosus	region1inRegion2	31934
CNVR28	353	2	56935884	56944870	loss	5682	38367026	157789611	pH_for_Semimembranosus	region1inRegion2	8986
CNVR29	355	2	58030910	58035496	gain	5682	38367026	157789611	pH_for_Semimembranosus	region1inRegion2	4586
CNVR30	359	2	61357153	61369311	loss	5682	38367026	157789611	pH_for_Semimembranosus	region1inRegion2	12158
CNVR31	362	2	62416028	62428337	gain	5682	38367026	157789611	pH_for_Semimembranosus	region1inRegion2	12309
CNVR32	364	2	62628253	62738661	gain	5682	38367026	157789611	pH_for_Semimembranosus	region1inRegion2	110408
CNVR33	378	2	67915859	67952389	gain	5682	38367026	157789611	pH_for_Semimembranosus	region1inRegion2	36530
CNVR34	379	2	68061173	68181801	gain	5682	38367026	157789611	pH_for_Semimembranosus	region1inRegion2	120628
CNVR35	401	2	95897658	95915484	loss	5682	38367026	157789611	pH_for_Semimembranosus	region1inRegion2	17826
CNVR36	408	2	96833054	96835454	loss	5682	38367026	157789611	pH_for_Semimembranosus	region1inRegion2	2400
CNVR37	422	2	107437562	107451870	gain	5682	38367026	157789611	pH_for_Semimembranosus	region1inRegion2	14308
CNVR38	466	2	148904898	148913506	loss-gain	5682	38367026	157789611	pH_for_Semimembranosus	region1inRegion2	8608
CNVR39	467	2	149095217	149101135	loss	5682	38367026	157789611	pH_for_Semimembranosus	region1inRegion2	5918
CNVR40	471	2	156079099	156090392	gain	5682	38367026	157789611	pH_for_Semimembranosus	region1inRegion2	11293
CNVR26	343	2	44800086	44803691	gain	9615	38367026	162569375	C3c_concentration	region1inRegion2	3605
CNVR27	348	2	54685639	54717573	loss-gain	9615	38367026	162569375	C3c_concentration	region1inRegion2	31934
CNVR28	353	2	56935884	56944870	loss	9615	38367026	162569375	C3c_concentration	region1inRegion2	8986
CNVR29	355	2	58030910	58035496	gain	9615	38367026	162569375	C3c_concentration	region1inRegion2	4586
CNVR30	359	2	61357153	61369311	loss	9615	38367026	162569375	C3c_concentration	region1inRegion2	12158
CNVR31	362	2	62416028	62428337	gain	9615	38367026	162569375	C3c_concentration	region1inRegion2	12309
CNVR32	364	2	62628253	62738661	gain	9615	38367026	162569375	C3c_concentration	region1inRegion2	110408
CNVR33	378	2	67915859	67952389	gain	9615	38367026	162569375	C3c_concentration	region1inRegion2	36530
CNVR34	379	2	68061173	68181801	gain	9615	38367026	162569375	C3c_concentration	region1inRegion2	120628
CNVR35	401	2	95897658	95915484	loss	9615	38367026	162569375	C3c_concentration	region1inRegion2	17826
CNVR36	408	2	96833054	96835454	loss	9615	38367026	162569375	C3c_concentration	region1inRegion2	2400
CNVR37	422	2	107437562	107451870	gain	9615	38367026	162569375	C3c_concentration	region1inRegion2	14308
CNVR38	466	2	148904898	148913506	loss-gain	9615	38367026	162569375	C3c_concentration	region1inRegion2	8608
CNVR39	467	2	149095217	149101135	loss	9615	38367026	162569375	C3c_concentration	region1inRegion2	5918
CNVR40	471	2	156079099	156090392	gain	9615	38367026	162569375	C3c_concentration	region1inRegion2	11293
CNVR41	472	2	158544152	158558867	gain	9615	38367026	162569375	C3c_concentration	region1inRegion2	14715
CNVR42	473	2	158605784	158612295	gain	9615	38367026	162569375	C3c_concentration	region1inRegion2	6511
CNVR42	473	2	158605784	158629757	gain	9615	38367026	162569375	C3c_concentration	region1inRegion2	23973
CNVR44	479	2	159094998	159110351	gain	9615	38367026	162569375	C3c_concentration	region1inRegion2	15353
CNVR45	487	2	160893564	160895760	gain	9615	38367026	162569375	C3c_concentration	region1inRegion2	2196
CNVR26	343	2	44800086	44803691	gain	3985	41353385	133375944	Cooking_loss	region1inRegion2	3605
CNVR27	348	2	54685639	54717573	loss-gain	3985	41353385	133375944	Cooking_loss	region1inRegion2	31934
CNVR28	353	2	56935884	56944870	loss	3985	41353385	133375944	Cooking_loss	region1inRegion2	8986
CNVR29	355	2	58030910	58035496	gain	3985	41353385	133375944	Cooking_loss	region1inRegion2	4586
CNVR30	359	2	61357153	61369311	loss	3985	41353385	133375944	Cooking_loss	region1inRegion2	12158
CNVR31	362	2	62416028	62428337	gain	3985	41353385	133375944	Cooking_loss	region1inRegion2	12309
CNVR32	364	2	62628253	62738661	gain	3985	41353385	133375944	Cooking_loss	region1inRegion2	110408
CNVR33	378	2	67915859	67952389	gain	3985	41353385	133375944	Cooking_loss	region1inRegion2	36530
CNVR34	379	2	68061173	68181801	gain	3985	41353385	133375944	Cooking_loss	region1inRegion2	120628
CNVR35	401	2	95897658	95915484	loss	3985	41353385	133375944	Cooking_loss	region1inRegion2	17826
CNVR36	408	2	96833054	96835454	loss	3985	41353385	133375944	Cooking_loss	region1inRegion2	2400
CNVR37	422	2	107437562	107451870	gain	3985	41353385	133375944	Cooking_loss	region1inRegion2	14308
CNVR26	343	2	44800086	44803691	gain	3938	41353385	157789611	Ham_fat_thickness	region1inRegion2	3605
CNVR27	348	2	54685639	54717573	loss-gain	3938	41353385	157789611	Ham_fat_thickness	region1inRegion2	31934
CNVR28	353	2	56935884	56944870	loss	3938	41353385	157789611	Ham_fat_thickness	region1inRegion2	8986
CNVR29	355	2	58030910	58035496	gain	3938	41353385	157789611	Ham_fat_thickness	region1inRegion2	4586
CNVR30	359	2	61357153	61369311	loss	3938	41353385	157789611	Ham_fat_thickness	region1inRegion2	12158
CNVR31	362	2	62416028	62428337	gain	3938	41353385	157789611	Ham_fat_thickness	region1inRegion2	12309
CNVR32	364	2	62628253	62738661	gain	3938	41353385	157789611	Ham_fat_thickness	region1inRegion2	110408
CNVR33	378	2	67915859	67952389	gain	3938	41353385	157789611	Ham_fat_thickness	region1inRegion2	36530
CNVR34	379	2	68061173	68181801	gain	3938	41353385	157789611	Ham_fat_thickness	region1inRegion2	120628
CNVR35	401	2	95897658	95915484	loss	3938	41353385	157789611	Ham_fat_thickness	region1inRegion2	17826
CNVR36	408	2	96833054	96835454	loss	3938	41353385	157789611	Ham_fat_thickness	region1inRegion2	2400
CNVR37	422	2	107437562	107451870	gain	3938	41353385	157789611	Ham_fat_thickness	region1inRegion2	14308
CNVR38	466	2	148904898	148913506	loss-gain	3938	41353385	157789611	Ham_fat_thickness	region1inRegion2	8608
CNVR39	467	2	149095217	149101135	loss	3938	41353385	157789611	Ham_fat_thickness	region1inRegion2	5918
CNVR40	471	2	156079099	156090392	gain	3938	41353385	157789611	Ham_fat_thickness	region1inRegion2	11293
CNVR26	343	2	44800086	44803691	gain	3982	41353385	157789611	CIE-b*	region1inRegion2	3605
CNVR27	348	2	54685639	54717573	loss-gain	3982	41353385	157789611	CIE-b*	region1inRegion2	31934
CNVR28	353	2	56935884	56944870	loss	3982	41353385	157789611	CIE-b*	region1inRegion2	8986
CNVR29	355	2	58030910	58035496	gain	3982	41353385	157789611	CIE-b*	region1inRegion2	4586
CNVR30	359	2	61357153	61369311	loss	3982	41353385	157789611	CIE-b*	region1inRegion2	12158
CNVR31	362	2	62416028	62428337	gain	3982	41353385	157789611	CIE-b*	region1inRegion2	12309
CNVR32	364	2	62628253	62738661	gain	3982	41353385	157789611	CIE-b*	region1inRegion2	110408
CNVR33	378	2	67915859	67952389	gain	3982	41353385	157789611	CIE-b*	region1inRegion2	36530
CNVR34	379	2	68061173	68181801	gain	3982	41353385	157789611	CIE-b*	region1inRegion2	120628
CNVR35	401	2	95897658	95915484	loss	3982	41353385	157789611	CIE-b*	region1inRegion2	17826
CNVR36	408	2	96833054	96835454	loss	3982	41353385	157789611	CIE-b*	region1inRegion2	2400

CNVR37	422	2	107437562	107451870	gain	3982	41353385	157789611	CIE-b*	region1inRegion2	14308
CNVR38	466	2	148904898	148913506	loss-gain	3982	41353385	157789611	CIE-b*	region1inRegion2	8608
CNVR39	467	2	149095217	149101135	loss	3982	41353385	157789611	CIE-b*	region1inRegion2	5918
CNVR40	471	2	156079099	156090392	gain	3982	41353385	157789611	CIE-b*	region1inRegion2	11293
CNVR26	343	2	44800086	44803691	gain	3984	41353385	157789611	Water_holding_capacity	region1inRegion2	3605
CNVR27	348	2	54685639	54717573	loss-gain	3984	41353385	157789611	Water_holding_capacity	region1inRegion2	31934
CNVR28	353	2	56935884	56944870	loss	3984	41353385	157789611	Water_holding_capacity	region1inRegion2	8986
CNVR29	355	2	58030910	58035496	gain	3984	41353385	157789611	Water_holding_capacity	region1inRegion2	4586
CNVR30	359	2	61357153	61369311	loss	3984	41353385	157789611	Water_holding_capacity	region1inRegion2	12158
CNVR31	362	2	62416028	62428337	gain	3984	41353385	157789611	Water_holding_capacity	region1inRegion2	12309
CNVR32	364	2	62628253	62738661	gain	3984	41353385	157789611	Water_holding_capacity	region1inRegion2	110408
CNVR33	378	2	67915859	67952389	gain	3984	41353385	157789611	Water_holding_capacity	region1inRegion2	36530
CNVR34	379	2	68061173	68181801	gain	3984	41353385	157789611	Water_holding_capacity	region1inRegion2	120628
CNVR35	401	2	95897658	95915484	loss	3984	41353385	157789611	Water_holding_capacity	region1inRegion2	17826
CNVR36	408	2	96833054	96835454	loss	3984	41353385	157789611	Water_holding_capacity	region1inRegion2	2400
CNVR37	422	2	107437562	107451870	gain	3984	41353385	157789611	Water_holding_capacity	region1inRegion2	14308
CNVR38	466	2	148904898	148913506	loss-gain	3984	41353385	157789611	Water_holding_capacity	region1inRegion2	8608
CNVR39	467	2	149095217	149101135	loss	3984	41353385	157789611	Water_holding_capacity	region1inRegion2	5918
CNVR40	471	2	156079099	156090392	gain	3984	41353385	157789611	Water_holding_capacity	region1inRegion2	11293
CNVR26	343	2	44800086	44803691	gain	4272	41690230	86250066	Firmness	region1inRegion2	3605
CNVR27	348	2	54685639	54717573	loss-gain	4272	41690230	86250066	Firmness	region1inRegion2	31934
CNVR28	353	2	56935884	56944870	loss	4272	41690230	86250066	Firmness	region1inRegion2	8986
CNVR29	355	2	58030910	58035496	gain	4272	41690230	86250066	Firmness	region1inRegion2	4586
CNVR30	359	2	61357153	61369311	loss	4272	41690230	86250066	Firmness	region1inRegion2	12158
CNVR31	362	2	62416028	62428337	gain	4272	41690230	86250066	Firmness	region1inRegion2	12309
CNVR32	364	2	62628253	62738661	gain	4272	41690230	86250066	Firmness	region1inRegion2	110408
CNVR33	378	2	67915859	67952389	gain	4272	41690230	86250066	Firmness	region1inRegion2	36530
CNVR34	379	2	68061173	68181801	gain	4272	41690230	86250066	Firmness	region1inRegion2	120628
CNVR26	343	2	44800086	44803691	gain	7016	43333303	75040316	Percentage_type_IIa_fibers	region1inRegion2	3605
CNVR27	348	2	54685639	54717573	loss-gain	7016	43333303	75040316	Percentage_type_IIa_fibers	region1inRegion2	31934
CNVR28	353	2	56935884	56944870	loss	7016	43333303	75040316	Percentage_type_IIa_fibers	region1inRegion2	8986
CNVR29	355	2	58030910	58035496	gain	7016	43333303	75040316	Percentage_type_IIa_fibers	region1inRegion2	4586
CNVR30	359	2	61357153	61369311	loss	7016	43333303	75040316	Percentage_type_IIa_fibers	region1inRegion2	12158
CNVR31	362	2	62416028	62428337	gain	7016	43333303	75040316	Percentage_type_IIa_fibers	region1inRegion2	12309
CNVR32	364	2	62628253	62738661	gain	7016	43333303	75040316	Percentage_type_IIa_fibers	region1inRegion2	110408
CNVR33	378	2	67915859	67952389	gain	7016	43333303	75040316	Percentage_type_IIa_fibers	region1inRegion2	36530
CNVR34	379	2	68061173	68181801	gain	7016	43333303	75040316	Percentage_type_IIa_fibers	region1inRegion2	120628
CNVR26	343	2	44800086	44803691	gain	18140	43664058	45003832	Gestation_length	region1inRegion2	3605
CNVR26	343	2	44800086	44803691	gain	5251	44409258	140000086	Shoulder_subcutaneous_fat_thickness	region1inRegion2	3605
CNVR27	348	2	54685639	54717573	loss-gain	5251	44409258	140000086	Shoulder_subcutaneous_fat_thickness	region1inRegion2	31934
CNVR28	353	2	56935884	56944870	loss	5251	44409258	140000086	Shoulder_subcutaneous_fat_thickness	region1inRegion2	8986
CNVR29	355	2	58030910	58035496	gain	5251	44409258	140000086	Shoulder_subcutaneous_fat_thickness	region1inRegion2	4586
CNVR30	359	2	61357153	61369311	loss	5251	44409258	140000086	Shoulder_subcutaneous_fat_thickness	region1inRegion2	12158
CNVR31	362	2	62416028	62428337	gain	5251	44409258	140000086	Shoulder_subcutaneous_fat_thickness	region1inRegion2	12309
CNVR32	364	2	62628253	62738661	gain	5251	44409258	140000086	Shoulder_subcutaneous_fat_thickness	region1inRegion2	110408
CNVR33	378	2	67915859	67952389	gain	5251	44409258	140000086	Shoulder_subcutaneous_fat_thickness	region1inRegion2	36530
CNVR34	379	2	68061173	68181801	gain	5251	44409258	140000086	Shoulder_subcutaneous_fat_thickness	region1inRegion2	120628
CNVR35	401	2	95897658	95915484	loss	5251	44409258	140000086	Shoulder_subcutaneous_fat_thickness	region1inRegion2	17826
CNVR36	408	2	96833054	96835454	loss	5251	44409258	140000086	Shoulder_subcutaneous_fat_thickness	region1inRegion2	2400
CNVR37	422	2	107437562	107451870	gain	5251	44409258	140000086	Shoulder_subcutaneous_fat_thickness	region1inRegion2	14308
CNVR26	343	2	44800086	44803691	gain	16893	44483418	64352885	Carcass_weight_(hot)	region1inRegion2	3605
CNVR27	348	2	54685639	54717573	loss-gain	16893	44483418	64352885	Carcass_weight_(hot)	region1inRegion2	31934
CNVR28	353	2	56935884	56944870	loss	16893	44483418	64352885	Carcass_weight_(hot)	region1inRegion2	8986
CNVR29	355	2	58030910	58035496	gain	16893	44483418	64352885	Carcass_weight_(hot)	region1inRegion2	4586
CNVR30	359	2	61357153	61369311	loss	16893	44483418	64352885	Carcass_weight_(hot)	region1inRegion2	12158
CNVR31	362	2	62416028	62428337	gain	16893	44483418	64352885	Carcass_weight_(hot)	region1inRegion2	12309
CNVR32	364	2	62628253	62738661	gain	16893	44483418	64352885	Carcass_weight_(hot)	region1inRegion2	110408
CNVR26	343	2	44800086	44803691	gain	16860	44483418	75040316	Carcass_weight_(hot)	region1inRegion2	3605
CNVR27	348	2	54685639	54717573	loss-gain	16860	44483418	75040316	Carcass_weight_(hot)	region1inRegion2	31934
CNVR28	353	2	56935884	56944870	loss	16860	44483418	75040316	Carcass_weight_(hot)	region1inRegion2	8986
CNVR29	355	2	58030910	58035496	gain	16860	44483418	75040316	Carcass_weight_(hot)	region1inRegion2	4586
CNVR30	359	2	61357153	61369311	loss	16860	44483418	75040316	Carcass_weight_(hot)	region1inRegion2	12158
CNVR31	362	2	62416028	62428337	gain	16860	44483418	75040316	Carcass_weight_(hot)	region1inRegion2	12309
CNVR32	364	2	62628253	62738661	gain	16860	44483418	75040316	Carcass_weight_(hot)	region1inRegion2	110408
CNVR33	378	2	67915859	67952389	gain	16860	44483418	75040316	Carcass_weight_(hot)	region1inRegion2	36530
CNVR34	379	2	68061173	68181801	gain	16860	44483418	75040316	Carcass_weight_(hot)	region1inRegion2	120628
CNVR26	343	2	44800086	44803691	gain	18004	44483418	75040316	Subcutanous_fat_area	region1inRegion2	3605
CNVR27	348	2	54685639	54717573	loss-gain	18004	44483418	75040316	Subcutanous_fat_area	region1inRegion2	31934
CNVR28	353	2	56935884	56944870	loss	18004	44483418	75040316	Subcutanous_fat_area	region1inRegion2	8986
CNVR29	355	2	58030910	58035496	gain	18004	44483418	75040316	Subcutanous_fat_area	region1inRegion2	4586
CNVR30	359	2	61357153	61369311	loss	18004	44483418	75040316	Subcutanous_fat_area	region1inRegion2	12158
CNVR31	362	2	62416028	62428337	gain	18004	44483418	75040316	Subcutanous_fat_area	region1inRegion2	12309
CNVR32	364	2	62628253	62738661	gain	18004	44483418	75040316	Subcutanous_fat_area	region1inRegion2	110408
CNVR33	378	2	67915859	67952389	gain	18004	44483418	75040316	Subcutanous_fat_area	region1inRegion2	36530
CNVR34	379	2	68061173	68181801	gain	18004	44483418	75040316	Subcutanous_fat_area	region1inRegion2	120628
CNVR26	343	2	44800086	44803691	gain	906	44483418	75040316	pH_24_hr_post-mortem_(loin)	region1inRegion2	3605
CNVR27	348	2	54685639	54717573	loss-gain	906	44483418	75040316	pH_24_hr_post-mortem_(loin)	region1inRegion2	31934
CNVR28	353	2	56935884	56944870	loss	906	44483418	75040316	pH_24_hr_post-mortem_(loin)	region1inRegion2	8986
CNVR29	355	2	58030910	58035496	gain	906	44483418	75040316	pH_24_hr_post-mortem_(loin)	region1inRegion2	4586
CNVR30	359	2	61357153	61369311	loss	906	44483418	75040316	pH_24_hr_post-mortem_(loin)	region1inRegion2	12158
CNVR31	362	2	62416028	62428337	gain	906	44483418	75040316	pH_24_hr_post-mortem_(loin)	region1inRegion2	12309
CNVR32	364	2	62628253	62738661	gain	906	44483418	75040316	pH_24_hr_post-mortem_(loin)	region1inRegion2	110408
CNVR33	378	2	67915859	67952389	gain	906	44483418	75040316	pH_24_hr_post-mortem_(loin)	region1inRegion2	36530
CNVR34	379	2	68061173	68181801	gain	906	44483418	75040316	pH_24_hr_post-mortem_(loin)	region1inRegion2	120628
CNVR26	343	2	44800086	44803691	gain	907	44483418	75040316	Abdominal_fat_weight	region1inRegion2	3605
CNVR27	348	2	54685639	54717573	loss-gain	907	44483418	75040316	Abdominal_fat_weight	region1inRegion2	31934
CNVR28	353	2	56935884	56944870	loss	907	44483418	75040316	Abdominal_fat_weight	region1inRegion2	8986
CNVR29	355	2	58030910	58035496	gain	907	44483418	75040316	Abdominal_fat_weight	region1inRegion2	4586
CNVR30	359	2	61357153	61369311	loss	907	44483418	75040316	Abdominal_fat_weight	region1inRegion2	12158
CNVR31	362	2	62416028	62428337	gain	907	44483418	75040316	Abdominal_fat_weight	region1inRegion2	12309
CNVR32	364	2	62628253	62738661	gain	907	44483418	75040316	Abdominal_fat_weight	region1inRegion2	110408
CNVR33	378	2	67915859	67952389	gain	907	44483418	75040316	Abdominal_fat_weight	region1inRegion2	36530
CNVR34	379	2	68061173	68181801	gain	907	44483418	75040316	Abdominal_fat_weight	region1inRegion2	120628
CNVR26	343	2	44800086	44803691	gain	908	44483418	75040316	Backfat_weight	region1inRegion2	3605
CNVR27	348	2	54685639	54717573	loss-gain	908	44483418	75040316	Backfat_weight	region1inRegion2	31934
CNVR28	353	2	56935884	56944870	loss	908	44483418	75040316	Backfat_weight	region1inRegion2	8986
CNVR29	355	2	58030910	58035496	gain	908	44483418	75040316	Backfat_weight	region1inRegion2	4586
CNVR30	359	2	61357153	61369311	loss	908	44483418	75040316	Backfat_weight	region1inRegion2	12158
CNVR31	362	2	62416028	62428337	gain	908	44483418	75040316	Backfat_weight	region1inRegion2	12309
CNVR32	364	2	62628253	62738661	gain	908	44483418	75040316	Backfat_weight	region1inRegion2	110408
CNVR33	378	2	67915859	67952389	gain	908	44483418	75040316	Backfat_weight	region1inRegion2	36530
CNVR34	379	2	68061173	68181801	gain	908	44483418	75040316	Backfat_weight	region1inRegion2	120628
CNVR26	343	2	44800086	44803691	gain	911	44483418	75040316	Liver_weight	region1inRegion2	3605
CNVR27	348	2	54685639	54717573	loss-gain	911	44483418	75040316	Liver_weight	region1inRegion2	31934
CNVR28	353	2	56935884	56944870	loss	911	44483418	75040316	Liver_weight	region1inRegion2	8986
CNVR29	355	2	58030910	58035496	gain	911	44483418	75040316	Liver_weight	region1inRegion2	4586
CNVR30	359	2	61357153	61369311	loss	911	44483418	75040316	Liver_weight	region1inRegion2	12158
CNVR31	362	2	62416028	62428337	gain	911	44483418	75040316	Liver_weight	region1inRegion2	12309
CNVR32	364	2	62628253	62738661	gain	911	44483418	75040316	Liver_weight	region1inRegion2	110408
CNVR33	378	2	67915859	67952389	gain	911	44483418	75040316	Liver_weight	region1inRegion2	36530
CNVR34	379	2	68061173	68181801	gain	911	44483418	75040316	Liver_weight	region1inRegion2	120628
CNVR26	343	2	44800086	44803691	gain	912	44483418	75040316	Shoulder_external_fat_weight	region1inRegion2	3605
CNVR27	348	2	54685639	54717573	loss-gain	912	44483418	75040316	Shoulder_external_fat_weight	region1inRegion2	31934
CNVR28	353	2	56935884	56944870	loss	912	44483418	75040316	Shoulder_external_fat_weight	region1inRegion2	8986
CNVR29	355	2	58030910	58035496	gain	912	44483418	75040316	Shoulder_external_fat_weight	region1inRegion2	4586
CNVR30	359	2	61357153	61369311	loss	912	44483418	75040316	Shoulder_external_fat_weight	region1inRegion2	12158
CNVR31	362	2	62416028	62428337	gain	912	44483418	75040316	Shoulder_external_fat_weight	region1inRegion2	12309
CNVR32	364	2	62628253	62738661	gain	912	44483418	75040316	Shoulder_external_fat_weight	region1inRegion2	110408
CNVR33	378	2	67915859	67952389	gain	912	44483418	75040316	Shoulder_external_fat_weight	region1inRegion2	36530
CNVR34	379	2	68061173	68181801	gain	912	44483418	75040316	Shoulder_external_fat_weight	region1inRegion2	120628
CNVR26	343	2	44800086	44803691	gain	913	44483418	75040316	Loin_muscle_area	region1inRegion2	3605
CNVR27	348	2	54685639	54717573	loss-gain	913	44483418	75040316	Loin_muscle_area	region1inRegion2	31934

CNVR28	353	2	56935884	56944870	loss	913	44483418	75040316	Loin_muscle_area	region1inRegion2	8986
CNVR29	355	2	58030910	58035496	gain	913	44483418	75040316	Loin_muscle_area	region1inRegion2	4586
CNVR30	359	2	61357153	61369311	loss	913	44483418	75040316	Loin_muscle_area	region1inRegion2	12158
CNVR31	362	2	62416028	62428337	gain	913	44483418	75040316	Loin_muscle_area	region1inRegion2	12309
CNVR32	364	2	62628253	62738661	gain	913	44483418	75040316	Loin_muscle_area	region1inRegion2	110408
CNVR33	378	2	67915859	67952389	gain	913	44483418	75040316	Loin_muscle_area	region1inRegion2	36530
CNVR34	379	2	68061173	68181801	gain	913	44483418	75040316	Loin_muscle_area	region1inRegion2	120628
CNVR27	348	2	54685639	54717573	loss-gain	16835	46505671	75040316	backfat_at_last_rib	region1inRegion2	31934
CNVR28	353	2	56935884	56944870	loss	16835	46505671	75040316	backfat_at_last_rib	region1inRegion2	8986
CNVR29	355	2	58030910	58035496	gain	16835	46505671	75040316	backfat_at_last_rib	region1inRegion2	4586
CNVR30	359	2	61357153	61369311	loss	16835	46505671	75040316	backfat_at_last_rib	region1inRegion2	12158
CNVR31	362	2	62416028	62428337	gain	16835	46505671	75040316	backfat_at_last_rib	region1inRegion2	12309
CNVR32	364	2	62628253	62738661	gain	16835	46505671	75040316	backfat_at_last_rib	region1inRegion2	110408
CNVR33	378	2	67915859	67952389	gain	16835	46505671	75040316	backfat_at_last_rib	region1inRegion2	36530
CNVR34	379	2	68061173	68181801	gain	16835	46505671	75040316	backfat_at_last_rib	region1inRegion2	120628
CNVR27	348	2	54685639	54717573	loss-gain	2973	48775112	75082926	Shear_force	region1inRegion2	31934
CNVR28	353	2	56935884	56944870	loss	2973	48775112	75082926	Shear_force	region1inRegion2	8986
CNVR29	355	2	58030910	58035496	gain	2973	48775112	75082926	Shear_force	region1inRegion2	4586
CNVR30	359	2	61357153	61369311	loss	2973	48775112	75082926	Shear_force	region1inRegion2	12158
CNVR31	362	2	62416028	62428337	gain	2973	48775112	75082926	Shear_force	region1inRegion2	12309
CNVR32	364	2	62628253	62738661	gain	2973	48775112	75082926	Shear_force	region1inRegion2	110408
CNVR33	378	2	67915859	67952389	gain	2973	48775112	75082926	Shear_force	region1inRegion2	36530
CNVR34	379	2	68061173	68181801	gain	2973	48775112	75082926	Shear_force	region1inRegion2	120628
CNVR27	348	2	54685639	54717573	loss-gain	2974	48775112	75082926	chew_score	region1inRegion2	31934
CNVR28	353	2	56935884	56944870	loss	2974	48775112	75082926	chew_score	region1inRegion2	8986
CNVR29	355	2	58030910	58035496	gain	2974	48775112	75082926	chew_score	region1inRegion2	4586
CNVR30	359	2	61357153	61369311	loss	2974	48775112	75082926	chew_score	region1inRegion2	12158
CNVR31	362	2	62416028	62428337	gain	2974	48775112	75082926	chew_score	region1inRegion2	12309
CNVR32	364	2	62628253	62738661	gain	2974	48775112	75082926	chew_score	region1inRegion2	110408
CNVR33	378	2	67915859	67952389	gain	2974	48775112	75082926	chew_score	region1inRegion2	36530
CNVR34	379	2	68061173	68181801	gain	2974	48775112	75082926	chew_score	region1inRegion2	120628
CNVR27	348	2	54685639	54717573	loss-gain	21355	48775112	131656956	Carcass_temperature_(45_minutes_post-mortem)	region1inRegion2	31934
CNVR28	353	2	56935884	56944870	loss	21355	48775112	131656956	Carcass_temperature_(45_minutes_post-mortem)	region1inRegion2	8986
CNVR29	355	2	58030910	58035496	gain	21355	48775112	131656956	Carcass_temperature_(45_minutes_post-mortem)	region1inRegion2	4586
CNVR30	359	2	61357153	61369311	loss	21355	48775112	131656956	Carcass_temperature_(45_minutes_post-mortem)	region1inRegion2	12158
CNVR31	362	2	62416028	62428337	gain	21355	48775112	131656956	Carcass_temperature_(45_minutes_post-mortem)	region1inRegion2	12309
CNVR32	364	2	62628253	62738661	gain	21355	48775112	131656956	Carcass_temperature_(45_minutes_post-mortem)	region1inRegion2	110408
CNVR33	378	2	67915859	67952389	gain	21355	48775112	131656956	Carcass_temperature_(45_minutes_post-mortem)	region1inRegion2	36530
CNVR34	379	2	68061173	68181801	gain	21355	48775112	131656956	Carcass_temperature_(45_minutes_post-mortem)	region1inRegion2	120628
CNVR35	401	2	95897658	95915484	loss	21355	48775112	131656956	Carcass_temperature_(45_minutes_post-mortem)	region1inRegion2	17826
CNVR36	408	2	96833054	96835454	loss	21355	48775112	131656956	Carcass_temperature_(45_minutes_post-mortem)	region1inRegion2	2400
CNVR37	422	2	107437562	107451870	gain	21355	48775112	131656956	Carcass_temperature_(45_minutes_post-mortem)	region1inRegion2	14308
CNVR27	348	2	54685639	54717573	loss-gain	3225	48775112	131656956	Off-Flavor_Score	region1inRegion2	31934
CNVR28	353	2	56935884	56944870	loss	3225	48775112	131656956	Off-Flavor_Score	region1inRegion2	8986
CNVR29	355	2	58030910	58035496	gain	3225	48775112	131656956	Off-Flavor_Score	region1inRegion2	4586
CNVR30	359	2	61357153	61369311	loss	3225	48775112	131656956	Off-Flavor_Score	region1inRegion2	12158
CNVR31	362	2	62416028	62428337	gain	3225	48775112	131656956	Off-Flavor_Score	region1inRegion2	12309
CNVR32	364	2	62628253	62738661	gain	3225	48775112	131656956	Off-Flavor_Score	region1inRegion2	110408
CNVR33	378	2	67915859	67952389	gain	3225	48775112	131656956	Off-Flavor_Score	region1inRegion2	36530
CNVR34	379	2	68061173	68181801	gain	3225	48775112	131656956	Off-Flavor_Score	region1inRegion2	120628
CNVR35	401	2	95897658	95915484	loss	3225	48775112	131656956	Off-Flavor_Score	region1inRegion2	17826
CNVR36	408	2	96833054	96835454	loss	3225	48775112	131656956	Off-Flavor_Score	region1inRegion2	2400
CNVR37	422	2	107437562	107451870	gain	3225	48775112	131656956	Off-Flavor_Score	region1inRegion2	14308
CNVR27	348	2	54685639	54717573	loss-gain	7471	51054270	59648728	Nonfunctional_nipples	region1inRegion2	31934
CNVR28	353	2	56935884	56944870	loss	7471	51054270	59648728	Nonfunctional_nipples	region1inRegion2	8986
CNVR29	355	2	58030910	58035496	gain	7471	51054270	59648728	Nonfunctional_nipples	region1inRegion2	4586
CNVR30	359	2	61357153	61369311	loss	12102	59648728	157789611	pH_24_hr_post-mortem_(loin)	region1inRegion2	12158
CNVR31	362	2	62416028	62428337	gain	12102	59648728	157789611	pH_24_hr_post-mortem_(loin)	region1inRegion2	12309
CNVR32	364	2	62628253	62738661	gain	12102	59648728	157789611	pH_24_hr_post-mortem_(loin)	region1inRegion2	110408
CNVR33	378	2	67915859	67952389	gain	12102	59648728	157789611	pH_24_hr_post-mortem_(loin)	region1inRegion2	36530
CNVR34	379	2	68061173	68181801	gain	12102	59648728	157789611	pH_24_hr_post-mortem_(loin)	region1inRegion2	120628
CNVR35	401	2	95897658	95915484	loss	12102	59648728	157789611	pH_24_hr_post-mortem_(loin)	region1inRegion2	17826
CNVR36	408	2	96833054	96835454	loss	12102	59648728	157789611	pH_24_hr_post-mortem_(loin)	region1inRegion2	2400
CNVR37	422	2	107437562	107451870	gain	12102	59648728	157789611	pH_24_hr_post-mortem_(loin)	region1inRegion2	14308
CNVR38	466	2	148904898	148913506	loss-gain	12102	59648728	157789611	pH_24_hr_post-mortem_(loin)	region1inRegion2	8608
CNVR39	467	2	149095217	149101135	loss	12102	59648728	157789611	pH_24_hr_post-mortem_(loin)	region1inRegion2	5918
CNVR40	471	2	156079099	156090392	gain	12102	59648728	157789611	pH_24_hr_post-mortem_(loin)	region1inRegion2	11293
CNVR30	359	2	61357153	61369311	loss	12103	59648728	157789611	Cooking_loss	region1inRegion2	12158
CNVR31	362	2	62416028	62428337	gain	12103	59648728	157789611	Cooking_loss	region1inRegion2	12309
CNVR32	364	2	62628253	62738661	gain	12103	59648728	157789611	Cooking_loss	region1inRegion2	110408
CNVR33	378	2	67915859	67952389	gain	12103	59648728	157789611	Cooking_loss	region1inRegion2	36530
CNVR34	379	2	68061173	68181801	gain	12103	59648728	157789611	Cooking_loss	region1inRegion2	120628
CNVR35	401	2	95897658	95915484	loss	12103	59648728	157789611	Cooking_loss	region1inRegion2	17826
CNVR36	408	2	96833054	96835454	loss	12103	59648728	157789611	Cooking_loss	region1inRegion2	2400
CNVR37	422	2	107437562	107451870	gain	12103	59648728	157789611	Cooking_loss	region1inRegion2	14308
CNVR38	466	2	148904898	148913506	loss-gain	12103	59648728	157789611	Cooking_loss	region1inRegion2	8608
CNVR39	467	2	149095217	149101135	loss	12103	59648728	157789611	Cooking_loss	region1inRegion2	5918
CNVR40	471	2	156079099	156090392	gain	12103	59648728	157789611	Cooking_loss	region1inRegion2	11293
CNVR30	359	2	61357153	61369311	loss	12104	59648728	157789611	Cooking_loss	region1inRegion2	12158
CNVR31	362	2	62416028	62428337	gain	12104	59648728	157789611	Cooking_loss	region1inRegion2	12309
CNVR32	364	2	62628253	62738661	gain	12104	59648728	157789611	Cooking_loss	region1inRegion2	110408
CNVR33	378	2	67915859	67952389	gain	12104	59648728	157789611	Cooking_loss	region1inRegion2	36530
CNVR34	379	2	68061173	68181801	gain	12104	59648728	157789611	Cooking_loss	region1inRegion2	120628
CNVR35	401	2	95897658	95915484	loss	12104	59648728	157789611	Cooking_loss	region1inRegion2	17826
CNVR36	408	2	96833054	96835454	loss	12104	59648728	157789611	Cooking_loss	region1inRegion2	2400
CNVR37	422	2	107437562	107451870	gain	12104	59648728	157789611	Cooking_loss	region1inRegion2	14308
CNVR38	466	2	148904898	148913506	loss-gain	12104	59648728	157789611	Cooking_loss	region1inRegion2	8608
CNVR39	467	2	149095217	149101135	loss	12104	59648728	157789611	Cooking_loss	region1inRegion2	5918
CNVR40	471	2	156079099	156090392	gain	12104	59648728	157789611	Cooking_loss	region1inRegion2	11293
CNVR30	359	2	61357153	61369311	loss	12105	59648728	157789611	Intramuscular_fat_content	region1inRegion2	12158
CNVR31	362	2	62416028	62428337	gain	12105	59648728	157789611	Intramuscular_fat_content	region1inRegion2	12309
CNVR32	364	2	62628253	62738661	gain	12105	59648728	157789611	Intramuscular_fat_content	region1inRegion2	110408
CNVR33	378	2	67915859	67952389	gain	12105	59648728	157789611	Intramuscular_fat_content	region1inRegion2	36530
CNVR34	379	2	68061173	68181801	gain	12105	59648728	157789611	Intramuscular_fat_content	region1inRegion2	120628
CNVR35	401	2	95897658	95915484	loss	12105	59648728	157789611	Intramuscular_fat_content	region1inRegion2	17826
CNVR36	408	2	96833054	96835454	loss	12105	59648728	157789611	Intramuscular_fat_content	region1inRegion2	2400
CNVR37	422	2	107437562	107451870	gain	12105	59648728	157789611	Intramuscular_fat_content	region1inRegion2	14308
CNVR38	466	2	148904898	148913506	loss-gain	12105	59648728	157789611	Intramuscular_fat_content	region1inRegion2	8608
CNVR39	467	2	149095217	149101135	loss	12105	59648728	157789611	Intramuscular_fat_content	region1inRegion2	5918
CNVR40	471	2	156079099	156090392	gain	12105	59648728	157789611	Intramuscular_fat_content	region1inRegion2	11293
CNVR30	359	2	61357153	61369311	loss	12106	59648728	157789611	Shear_force	region1inRegion2	12158
CNVR31	362	2	62416028	62428337	gain	12106	59648728	157789611	Shear_force	region1inRegion2	12309
CNVR32	364	2	62628253	62738661	gain	12106	59648728	157789611	Shear_force	region1inRegion2	110408
CNVR33	378	2	67915859	67952389	gain	12106	59648728	157789611	Shear_force	region1inRegion2	36530
CNVR34	379	2	68061173	68181801	gain	12106	59648728	157789611	Shear_force	region1inRegion2	120628
CNVR35	401	2	95897658	95915484	loss	12106	59648728	157789611	Shear_force	region1inRegion2	17826
CNVR36	408	2	96833054	96835454	loss	12106	59648728	157789611	Shear_force	region1inRegion2	2400
CNVR37	422	2	107437562	107451870	gain	12106	59648728	157789611	Shear_force	region1inRegion2	14308
CNVR38	466	2	148904898	148913506	loss-gain	12106	59648728	157789611	Shear_force	region1inRegion2	8608
CNVR39	467	2	149095217	149101135	loss	12106	59648728	157789611	Shear_force	region1inRegion2	5918
CNVR40	471	2	156079099	156090392	gain	12106	59648728	157789611	Shear_force	region1inRegion2	11293
CNVR30	359	2	61357153	61369311	loss	12107	59648728	157789611	Ham_weight	region1inRegion2	12158
CNVR31	362	2	62416028	62428337	gain	12107	59648728	157789611	Ham_weight	region1inRegion2	12309
CNVR32	364	2	62628253	62738661	gain	12107	59648728	157789611	Ham_weight	region1inRegion2	110408
CNVR33	378	2	67915859	67952389	gain	12107	59648728	157789611	Ham_weight	region1inRegion2	36530
CNVR34	379	2	68061173	68181801	gain	12107	59648728	157789611	Ham_weight	region1inRegion2	120628
CNVR35	401	2	95897658	95915484	loss	12107	59648728	157789611	Ham_weight	region1inRegion2	17826
CNVR36	408	2	96833054	96835454	loss	12107	59648728	157789611	Ham_weight	region1inRegion2	2400
CNVR37	422	2	107437562	107451870	gain	12107	59648728	157789611	Ham_weight	region1inRegion2	14308
CNVR38	466	2	148904898	148913506	loss-gain	12107	59648728	157789611	Ham_weight	region1inRegion2	8608
CNVR39	467	2	149095217	149101135	loss	12107	59648728	157789611	Ham_weight	region1inRegion2	5918
CNVR40	471	2	156079099	156090392	gain	12107	59648728	157789611	Ham_weight	region1inRegion2	11293

CNVR30	359	2	61357153	61369311	loss	12108	59648728	157789611	Estimated_carcass_lean_content	region1inRegion2	12158
CNVR31	362	2	62416028	62428337	gain	12108	59648728	157789611	Estimated_carcass_lean_content	region1inRegion2	12309
CNVR32	364	2	62628253	62738661	gain	12108	59648728	157789611	Estimated_carcass_lean_content	region1inRegion2	110408
CNVR33	378	2	67915859	67952389	gain	12108	59648728	157789611	Estimated_carcass_lean_content	region1inRegion2	36530
CNVR34	379	2	68061173	68181801	gain	12108	59648728	157789611	Estimated_carcass_lean_content	region1inRegion2	120628
CNVR35	401	2	95897658	95915484	loss	12108	59648728	157789611	Estimated_carcass_lean_content	region1inRegion2	17826
CNVR36	408	2	96833054	96835454	loss	12108	59648728	157789611	Estimated_carcass_lean_content	region1inRegion2	2400
CNVR37	422	2	107437562	107451870	gain	12108	59648728	157789611	Estimated_carcass_lean_content	region1inRegion2	14308
CNVR38	466	2	148904898	148913506	loss-gain	12108	59648728	157789611	Estimated_carcass_lean_content	region1inRegion2	8608
CNVR39	467	2	149095217	149101135	loss	12108	59648728	157789611	Estimated_carcass_lean_content	region1inRegion2	5918
CNVR40	471	2	156079099	156090392	gain	12108	59648728	157789611	Estimated_carcass_lean_content	region1inRegion2	11293
CNVR30	359	2	61357153	61369311	loss	12109	59648728	157789611	Ham_weight	region1inRegion2	12158
CNVR31	362	2	62416028	62428337	gain	12109	59648728	157789611	Ham_weight	region1inRegion2	12309
CNVR32	364	2	62628253	62738661	gain	12109	59648728	157789611	Ham_weight	region1inRegion2	110408
CNVR33	378	2	67915859	67952389	gain	12109	59648728	157789611	Ham_weight	region1inRegion2	36530
CNVR34	379	2	68061173	68181801	gain	12109	59648728	157789611	Ham_weight	region1inRegion2	120628
CNVR35	401	2	95897658	95915484	loss	12109	59648728	157789611	Ham_weight	region1inRegion2	17826
CNVR36	408	2	96833054	96835454	loss	12109	59648728	157789611	Ham_weight	region1inRegion2	2400
CNVR37	422	2	107437562	107451870	gain	12109	59648728	157789611	Ham_weight	region1inRegion2	14308
CNVR38	466	2	148904898	148913506	loss-gain	12109	59648728	157789611	Ham_weight	region1inRegion2	8608
CNVR39	467	2	149095217	149101135	loss	12109	59648728	157789611	Ham_weight	region1inRegion2	5918
CNVR40	471	2	156079099	156090392	gain	12109	59648728	157789611	Ham_weight	region1inRegion2	11293
CNVR30	359	2	61357153	61369311	loss	5684	59648728	157789611	Shear_force	region1inRegion2	12158
CNVR31	362	2	62416028	62428337	gain	5684	59648728	157789611	Shear_force	region1inRegion2	12309
CNVR32	364	2	62628253	62738661	gain	5684	59648728	157789611	Shear_force	region1inRegion2	110408
CNVR33	378	2	67915859	67952389	gain	5684	59648728	157789611	Shear_force	region1inRegion2	36530
CNVR34	379	2	68061173	68181801	gain	5684	59648728	157789611	Shear_force	region1inRegion2	120628
CNVR35	401	2	95897658	95915484	loss	5684	59648728	157789611	Shear_force	region1inRegion2	17826
CNVR36	408	2	96833054	96835454	loss	5684	59648728	157789611	Shear_force	region1inRegion2	2400
CNVR37	422	2	107437562	107451870	gain	5684	59648728	157789611	Shear_force	region1inRegion2	14308
CNVR38	466	2	148904898	148913506	loss-gain	5684	59648728	157789611	Shear_force	region1inRegion2	8608
CNVR39	467	2	149095217	149101135	loss	5684	59648728	157789611	Shear_force	region1inRegion2	5918
CNVR40	471	2	156079099	156090392	gain	5684	59648728	157789611	Shear_force	region1inRegion2	11293
CNVR33	378	2	67915859	67952389	gain	5825	65112913	97924522	PH_for_Longissmus_dorsi	region1inRegion2	36530
CNVR34	379	2	68061173	68181801	gain	5825	65112913	97924522	PH_for_Longissmus_dorsi	region1inRegion2	120628
CNVR35	401	2	95897658	95915484	loss	5825	65112913	97924522	PH_for_Longissmus_dorsi	region1inRegion2	17826
CNVR36	408	2	96833054	96835454	loss	5825	65112913	97924522	PH_for_Longissmus_dorsi	region1inRegion2	2400
CNVR34	379	2	68061173	68181801	gain	27312	68105444	68105484	Mean_corpuscular_hemoglobin_content	region2inRegion1	40
CNVR34	379	2	68061173	68181801	gain	27381	68105444	68105484	Mean_corpuscular_volume	region2inRegion1	40
CNVR35	401	2	95897658	95915484	loss	21215	70124078	125622966	Backfat_at_first_rib	region1inRegion2	17826
CNVR36	408	2	96833054	96835454	loss	21215	70124078	125622966	Backfat_at_first_rib	region1inRegion2	2400
CNVR37	422	2	107437562	107451870	gain	21215	70124078	125622966	Backfat_at_first_rib	region1inRegion2	14308
CNVR35	401	2	95897658	95915484	loss	670	72813988	122153206	Loin_muscle_area	region1inRegion2	17826
CNVR36	408	2	96833054	96835454	loss	670	72813988	122153206	Loin_muscle_area	region1inRegion2	2400
CNVR37	422	2	107437562	107451870	gain	670	72813988	122153206	Loin_muscle_area	region1inRegion2	14308
CNVR35	401	2	95897658	95915484	loss	12743	75040316	119359590	Carcass_weight_(cold)	region1inRegion2	17826
CNVR36	408	2	96833054	96835454	loss	12743	75040316	119359590	Carcass_weight_(cold)	region1inRegion2	2400
CNVR37	422	2	107437562	107451870	gain	12743	75040316	119359590	Carcass_weight_(cold)	region1inRegion2	14308
CNVR35	401	2	95897658	95915484	loss	12745	75040316	119359590	Shoulder_external_fat_weight	region1inRegion2	17826
CNVR36	408	2	96833054	96835454	loss	12745	75040316	119359590	Shoulder_external_fat_weight	region1inRegion2	2400
CNVR37	422	2	107437562	107451870	gain	12745	75040316	119359590	Shoulder_external_fat_weight	region1inRegion2	14308
CNVR35	401	2	95897658	95915484	loss	12746	75040316	119359590	Backfat_weight	region1inRegion2	17826
CNVR36	408	2	96833054	96835454	loss	12746	75040316	119359590	Backfat_weight	region1inRegion2	2400
CNVR37	422	2	107437562	107451870	gain	12746	75040316	119359590	Backfat_weight	region1inRegion2	14308
CNVR35	401	2	95897658	95915484	loss	11558	75040316	121864622	Salmonella_count_in_liver_and_spleen	region1inRegion2	17826
CNVR36	408	2	96833054	96835454	loss	11558	75040316	121864622	Salmonella_count_in_liver_and_spleen	region1inRegion2	2400
CNVR37	422	2	107437562	107451870	gain	11558	75040316	121864622	Salmonella_count_in_liver_and_spleen	region1inRegion2	14308
CNVR35	401	2	95897658	95915484	loss	11559	75040316	121864622	Salmonella_count_in_liver	region1inRegion2	17826
CNVR36	408	2	96833054	96835454	loss	11559	75040316	121864622	Salmonella_count_in_liver	region1inRegion2	2400
CNVR37	422	2	107437562	107451870	gain	11559	75040316	121864622	Salmonella_count_in_liver	region1inRegion2	14308
CNVR35	401	2	95897658	95915484	loss	7309	75040316	125047558	Carcass_weight_(hot)	region1inRegion2	17826
CNVR36	408	2	96833054	96835454	loss	7309	75040316	125047558	Carcass_weight_(hot)	region1inRegion2	2400
CNVR37	422	2	107437562	107451870	gain	7309	75040316	125047558	Carcass_weight_(hot)	region1inRegion2	14308
CNVR35	401	2	95897658	95915484	loss	7310	75040316	125047558	Loin_muscle_area	region1inRegion2	17826
CNVR36	408	2	96833054	96835454	loss	7310	75040316	125047558	Loin_muscle_area	region1inRegion2	2400
CNVR37	422	2	107437562	107451870	gain	7310	75040316	125047558	Loin_muscle_area	region1inRegion2	14308
CNVR35	401	2	95897658	95915484	loss	16899	75040316	135266453	Carcass_weight_(hot)	region1inRegion2	17826
CNVR36	408	2	96833054	96835454	loss	16899	75040316	135266453	Carcass_weight_(hot)	region1inRegion2	2400
CNVR37	422	2	107437562	107451870	gain	16899	75040316	135266453	Carcass_weight_(hot)	region1inRegion2	14308
CNVR35	401	2	95897658	95915484	loss	2975	75082926	100385940	Muscle_moisture_percentage	region1inRegion2	17826
CNVR36	408	2	96833054	96835454	loss	2975	75082926	100385940	Muscle_moisture_percentage	region1inRegion2	2400
CNVR35	401	2	95897658	95915484	loss	2976	75082926	100385940	PH_for_Longissmus_dorsi	region1inRegion2	17826
CNVR36	408	2	96833054	96835454	loss	2976	75082926	100385940	PH_for_Longissmus_dorsi	region1inRegion2	2400
CNVR35	401	2	95897658	95915484	loss	4327	75082926	100385940	tenderness_score	region1inRegion2	17826
CNVR36	408	2	96833054	96835454	loss	4327	75082926	100385940	tenderness_score	region1inRegion2	2400
CNVR35	401	2	95897658	95915484	loss	2796	82717608	128992198	Diameter_of_type_IIb_muscle_fibers	region1inRegion2	17826
CNVR36	408	2	96833054	96835454	loss	2796	82717608	128992198	Diameter_of_type_IIb_muscle_fibers	region1inRegion2	2400
CNVR37	422	2	107437562	107451870	gain	2796	82717608	128992198	Diameter_of_type_IIb_muscle_fibers	region1inRegion2	14308
CNVR35	401	2	95897658	95915484	loss	2797	82717608	128992198	Diameter_of_type_IIa_muscle_fibers	region1inRegion2	17826
CNVR36	408	2	96833054	96835454	loss	2797	82717608	128992198	Diameter_of_type_IIa_muscle_fibers	region1inRegion2	2400
CNVR37	422	2	107437562	107451870	gain	2797	82717608	128992198	Diameter_of_type_IIa_muscle_fibers	region1inRegion2	14308
CNVR35	401	2	95897658	95915484	loss	2798	82717608	128992198	Diameter_of_muscle_fibers	region1inRegion2	17826
CNVR36	408	2	96833054	96835454	loss	2798	82717608	128992198	Diameter_of_muscle_fibers	region1inRegion2	2400
CNVR37	422	2	107437562	107451870	gain	2798	82717608	128992198	Diameter_of_muscle_fibers	region1inRegion2	14308
CNVR35	401	2	95897658	95915484	loss	37562	83714126	150929247	Actinobacillus_pleuropneumoniae_susceptibility	region1inRegion2	17826
CNVR36	408	2	96833054	96835454	loss	37562	83714126	150929247	Actinobacillus_pleuropneumoniae_susceptibility	region1inRegion2	2400
CNVR37	422	2	107437562	107451870	gain	37562	83714126	150929247	Actinobacillus_pleuropneumoniae_susceptibility	region1inRegion2	14308
CNVR38	466	2	148904898	148913506	loss-gain	37562	83714126	150929247	Actinobacillus_pleuropneumoniae_susceptibility	region1inRegion2	8608
CNVR39	467	2	149095217	149101135	loss	37562	83714126	150929247	Actinobacillus_pleuropneumoniae_susceptibility	region1inRegion2	5918
CNVR35	401	2	95897658	95915484	loss	18655	83936085	102514481	androstenone,_laboratory	region1inRegion2	17826
CNVR36	408	2	96833054	96835454	loss	18655	83936085	102514481	androstenone,_laboratory	region1inRegion2	2400
CNVR35	401	2	95897658	95915484	loss	21218	85135844	149834563	Leaf_fat_weight	region1inRegion2	17826
CNVR36	408	2	96833054	96835454	loss	21218	85135844	149834563	Leaf_fat_weight	region1inRegion2	2400
CNVR37	422	2	107437562	107451870	gain	21218	85135844	149834563	Leaf_fat_weight	region1inRegion2	14308
CNVR38	466	2	148904898	148913506	loss-gain	21218	85135844	149834563	Leaf_fat_weight	region1inRegion2	8608
CNVR39	467	2	149095217	149101135	loss	21218	85135844	149834563	Leaf_fat_weight	region1inRegion2	5918
CNVR35	401	2	95897658	95915484	loss	21211	89755188	131594228	Average_backfat_thickness	region1inRegion2	17826
CNVR36	408	2	96833054	96835454	loss	21211	89755188	131594228	Average_backfat_thickness	region1inRegion2	2400
CNVR37	422	2	107437562	107451870	gain	21211	89755188	131594228	Average_backfat_thickness	region1inRegion2	14308
CNVR35	401	2	95897658	95915484	loss	5757	95157201	96901037	Juiciness_score	region1inRegion2	17826
CNVR36	408	2	96833054	96835454	loss	5757	95157201	96901037	Juiciness_score	region1inRegion2	2400
CNVR35	401	2	95897658	95915484	loss	18063	95595645	96335500	Total_number_born_alive	region1inRegion2	17826
CNVR37	422	2	107437562	107451870	gain	6240	97794151	125044122	Body_weight_(15_weeks)	region1inRegion2	14308
CNVR37	422	2	107437562	107451870	gain	1278	100385940	113025492	Ratio_of_back_bone_and_meat_weight_to_ham_meat_and_bone_	region1inRegion2	14308
CNVR37	422	2	107437562	107451870	gain	21837	100385940	125047558	Gestation_length	region1inRegion2	14308
CNVR37	422	2	107437562	107451870	gain	8899	100385940	125047558	Ear_erectness	region1inRegion2	14308
CNVR37	422	2	107437562	107451870	gain	5161	100385940	133375944	Feed_intake	region1inRegion2	14308
CNVR35	401	2	95897658	95915484	loss	64703	101494035	93175114	Thoracic_vertebra_number	region2inRegion1	-8318921
CNVR36	408	2	96833054	96835454	loss	64703	101494035	93175114	Thoracic_vertebra_number	region2inRegion1	-8318921
CNVR37	422	2	107437562	107451870	gain	4273	103753269	127713292	Average_instron_(star_probe)_force	region1inRegion2	14308
CNVR38	466	2	148904898	148913506	loss-gain	12316	133375944	151463344	Change_in_Mycoplasma_hyopneumoniae_antibody_titer	region1inRegion2	8608
CNVR39	467	2	149095217	149101135	loss	12316	133375944	151463344	Change_in_Mycoplasma_hyopneumoniae_antibody_titer	region1inRegion2	5918
CNVR38	466	2	148904898	148913506	loss-gain	85	133375944	154953107	Drip_loss	region1inRegion2	8608
CNVR39	467	2	149095217	149101135	loss	85	133375944	154953107	Drip_loss	region1inRegion2	5918
CNVR38	466	2	148904898	148913506	loss-gain	7483	135097924	152314760	Body_temperature	region1inRegion2	8608
CNVR39	467	2	149095217	149101135	loss	7483	135097924	152314760	Body_temperature	region1inRegion2	5918
CNVR38	466	2	148904898	148913506	loss-gain	7040	135266453	154953107	Percentage_type_IIb_fibers	region1inRegion2	8608
CNVR39	467	2	149095217	149101135	loss	7040	135266453	154953107	Percentage_type_IIb_fibers	region1inRegion2	5918
CNVR38	466	2	148904898	148913506	loss-gain	4255	139359663	150135089	Nonfunctional_nipples	region1inRegion2	8608
CNVR39	467	2	149095217	149101135	loss	4255	139359663	150135089	Nonfunctional_nipples	region1inRegion2	5918
CNVR38	466	2	148904898	148913506	loss-gain	434	142363071	155325316	Feet_weight	region1inRegion2	8608
CNVR39	467	2	149095217	149101135	loss	434	142363071	155325316	Feet_weight	region1inRegion2	5918

CNVR38	466	2	148904898	148913506	loss-gain	433	142363071	162569375	Head_weight	region1inRegion2	8608
CNVR39	467	2	149095217	149101135	loss	433	142363071	162569375	Head_weight	region1inRegion2	5918
CNVR40	471	2	156079099	156090392	gain	433	142363071	162569375	Head_weight	region1inRegion2	11293
CNVR41	472	2	158544152	158558867	gain	433	142363071	162569375	Head_weight	region1inRegion2	14715
CNVR42	473	2	158605784	158612295	gain	433	142363071	162569375	Head_weight	region1inRegion2	6511
CNVR42	473	2	158605784	158629757	gain	433	142363071	162569375	Head_weight	region1inRegion2	23973
CNVR44	479	2	159094998	159110351	gain	433	142363071	162569375	Head_weight	region1inRegion2	15353
CNVR45	487	2	160893564	160895760	gain	433	142363071	162569375	Head_weight	region1inRegion2	2196
CNVR38	466	2	148904898	148913506	loss-gain	17714	143420936	154953107	Triglyceride_level	region1inRegion2	8608
CNVR39	467	2	149095217	149101135	loss	17714	143420936	154953107	Triglyceride_level	region1inRegion2	5918
CNVR38	466	2	148904898	148913506	loss-gain	3802	143420936	154953107	Meat_color-L	region1inRegion2	8608
CNVR39	467	2	149095217	149101135	loss	3802	143420936	154953107	Meat_color-L	region1inRegion2	5918
CNVR38	466	2	148904898	148913506	loss-gain	3803	143420936	154953107	pH_48_hr_post-mortem_(loin)	region1inRegion2	8608
CNVR39	467	2	149095217	149101135	loss	3803	143420936	154953107	pH_48_hr_post-mortem_(loin)	region1inRegion2	5918
CNVR38	466	2	148904898	148913506	loss-gain	5933	143420936	154953107	Average_daily_gain	region1inRegion2	8608
CNVR39	467	2	149095217	149101135	loss	5933	143420936	154953107	Average_daily_gain	region1inRegion2	5918
CNVR38	466	2	148904898	148913506	loss-gain	87	143420936	154953107	Water_holding_capacity	region1inRegion2	8608
CNVR39	467	2	149095217	149101135	loss	87	143420936	154953107	Water_holding_capacity	region1inRegion2	5918
CNVR38	466	2	148904898	148913506	loss-gain	83	143420936	157789611	Meat_color-L	region1inRegion2	8608
CNVR39	467	2	149095217	149101135	loss	83	143420936	157789611	Meat_color-L	region1inRegion2	5918
CNVR40	471	2	156079099	156090392	gain	83	143420936	157789611	Meat_color-L	region1inRegion2	11293
CNVR38	466	2	148904898	148913506	loss-gain	84	143420936	157789611	Meat_color-L	region1inRegion2	8608
CNVR39	467	2	149095217	149101135	loss	84	143420936	157789611	Meat_color-L	region1inRegion2	5918
CNVR40	471	2	156079099	156090392	gain	84	143420936	157789611	Meat_color-L	region1inRegion2	11293
CNVR38	466	2	148904898	148913506	loss-gain	23169	144927181	148905198	Shoulder_weight	overlapHead	301
CNVR38	466	2	148904898	148913506	loss-gain	23174	145959095	151354953	Loin_weight	region1inRegion2	8608
CNVR39	467	2	149095217	149101135	loss	23174	145959095	151354953	Loin_weight	region1inRegion2	5918
CNVR38	466	2	148904898	148913506	loss-gain	23275	145959095	151354953	Backfat_at_rump	region1inRegion2	8608
CNVR39	467	2	149095217	149101135	loss	23275	145959095	151354953	Backfat_at_rump	region1inRegion2	5918
CNVR38	466	2	148904898	148913506	loss-gain	23279	145959095	151354953	Backfat_at_rump	region1inRegion2	8608
CNVR39	467	2	149095217	149101135	loss	23279	145959095	151354953	Backfat_at_rump	region1inRegion2	5918
CNVR38	466	2	148904898	148913506	loss-gain	23296	145959095	151354953	Backfat_at_rump	region1inRegion2	8608
CNVR39	467	2	149095217	149101135	loss	23296	145959095	151354953	Backfat_at_rump	region1inRegion2	5918
CNVR38	466	2	148904898	148913506	loss-gain	23343	145959095	151354953	Backfat_at_rump	region1inRegion2	8608
CNVR39	467	2	149095217	149101135	loss	23343	145959095	151354953	Backfat_at_rump	region1inRegion2	5918
CNVR38	466	2	148904898	148913506	loss-gain	23358	145959095	151354953	Backfat_at_rump	region1inRegion2	8608
CNVR39	467	2	149095217	149101135	loss	23358	145959095	151354953	Backfat_at_rump	region1inRegion2	5918
CNVR38	466	2	148904898	148913506	loss-gain	23371	145959095	151354953	Backfat_at_rump	region1inRegion2	8608
CNVR39	467	2	149095217	149101135	loss	23371	145959095	151354953	Backfat_at_rump	region1inRegion2	5918
CNVR38	466	2	148904898	148913506	loss-gain	23447	145959095	151354953	Interferon-gamma_level	region1inRegion2	8608
CNVR39	467	2	149095217	149101135	loss	23447	145959095	151354953	Interferon-gamma_level	region1inRegion2	5918
CNVR38	466	2	148904898	148913506	loss-gain	23474	145959095	151354953	Immunoglobulin_G_level	region1inRegion2	8608
CNVR39	467	2	149095217	149101135	loss	23474	145959095	151354953	Immunoglobulin_G_level	region1inRegion2	5918
CNVR38	466	2	148904898	148913506	loss-gain	23475	145959095	151354953	Immunoglobulin_G_level	region1inRegion2	8608
CNVR39	467	2	149095217	149101135	loss	23475	145959095	151354953	Immunoglobulin_G_level	region1inRegion2	5918
CNVR38	466	2	148904898	148913506	loss-gain	23476	145959095	151354953	Immunoglobulin_G_level	region1inRegion2	8608
CNVR39	467	2	149095217	149101135	loss	23476	145959095	151354953	Immunoglobulin_G_level	region1inRegion2	5918
CNVR38	466	2	148904898	148913506	loss-gain	23477	145959095	151354953	Immunoglobulin_G_level	region1inRegion2	8608
CNVR39	467	2	149095217	149101135	loss	23477	145959095	151354953	Immunoglobulin_G_level	region1inRegion2	5918
CNVR38	466	2	148904898	148913506	loss-gain	23484	145959095	151354953	LDL_cholesterol	region1inRegion2	8608
CNVR39	467	2	149095217	149101135	loss	23484	145959095	151354953	LDL_cholesterol	region1inRegion2	5918
CNVR38	466	2	148904898	148913506	loss-gain	23485	145959095	151354953	LDL_cholesterol	region1inRegion2	8608
CNVR39	467	2	149095217	149101135	loss	23485	145959095	151354953	LDL_cholesterol	region1inRegion2	5918
CNVR38	466	2	148904898	148913506	loss-gain	23486	145959095	151354953	LDL_cholesterol	region1inRegion2	8608
CNVR39	467	2	149095217	149101135	loss	23486	145959095	151354953	LDL_cholesterol	region1inRegion2	5918
CNVR38	466	2	148904898	148913506	loss-gain	23487	145959095	151354953	LDL_cholesterol	region1inRegion2	8608
CNVR39	467	2	149095217	149101135	loss	23487	145959095	151354953	LDL_cholesterol	region1inRegion2	5918
CNVR38	466	2	148904898	148913506	loss-gain	23488	145959095	151354953	LDL_cholesterol	region1inRegion2	8608
CNVR39	467	2	149095217	149101135	loss	23488	145959095	151354953	LDL_cholesterol	region1inRegion2	5918
CNVR38	466	2	148904898	148913506	loss-gain	23489	145959095	151354953	LDL_cholesterol	region1inRegion2	8608
CNVR39	467	2	149095217	149101135	loss	23489	145959095	151354953	LDL_cholesterol	region1inRegion2	5918
CNVR38	466	2	148904898	148913506	loss-gain	23490	145959095	151354953	LDL_cholesterol	region1inRegion2	8608
CNVR39	467	2	149095217	149101135	loss	23490	145959095	151354953	LDL_cholesterol	region1inRegion2	5918
CNVR38	466	2	148904898	148913506	loss-gain	23491	145959095	151354953	LDL_cholesterol	region1inRegion2	8608
CNVR39	467	2	149095217	149101135	loss	23491	145959095	151354953	LDL_cholesterol	region1inRegion2	5918
CNVR38	466	2	148904898	148913506	loss-gain	23492	145959095	151354953	LDL_cholesterol	region1inRegion2	8608
CNVR39	467	2	149095217	149101135	loss	23492	145959095	151354953	LDL_cholesterol	region1inRegion2	5918
CNVR38	466	2	148904898	148913506	loss-gain	23493	145959095	151354953	LDL_cholesterol	region1inRegion2	8608
CNVR39	467	2	149095217	149101135	loss	23493	145959095	151354953	LDL_cholesterol	region1inRegion2	5918
CNVR38	466	2	148904898	148913506	loss-gain	23494	145959095	151354953	LDL_cholesterol	region1inRegion2	8608
CNVR39	467	2	149095217	149101135	loss	23494	145959095	151354953	LDL_cholesterol	region1inRegion2	5918
CNVR38	466	2	148904898	148913506	loss-gain	23495	145959095	151354953	LDL_cholesterol	region1inRegion2	8608
CNVR39	467	2	149095217	149101135	loss	23495	145959095	151354953	LDL_cholesterol	region1inRegion2	5918
CNVR38	466	2	148904898	148913506	loss-gain	23496	145959095	151354953	LDL_cholesterol	region1inRegion2	8608
CNVR39	467	2	149095217	149101135	loss	23496	145959095	151354953	LDL_cholesterol	region1inRegion2	5918
CNVR38	466	2	148904898	148913506	loss-gain	23497	145959095	151354953	LDL_cholesterol	region1inRegion2	8608
CNVR39	467	2	149095217	149101135	loss	23497	145959095	151354953	LDL_cholesterol	region1inRegion2	5918
CNVR38	466	2	148904898	148913506	loss-gain	23503	145959095	151354953	Cholesterol_level	region1inRegion2	8608
CNVR39	467	2	149095217	149101135	loss	23503	145959095	151354953	Cholesterol_level	region1inRegion2	5918
CNVR38	466	2	148904898	148913506	loss-gain	23504	145959095	151354953	Cholesterol_level	region1inRegion2	8608
CNVR39	467	2	149095217	149101135	loss	23504	145959095	151354953	Cholesterol_level	region1inRegion2	5918
CNVR38	466	2	148904898	148913506	loss-gain	23505	145959095	151354953	Cholesterol_level	region1inRegion2	8608
CNVR39	467	2	149095217	149101135	loss	23505	145959095	151354953	Cholesterol_level	region1inRegion2	5918
CNVR38	466	2	148904898	148913506	loss-gain	23506	145959095	151354953	Cholesterol_level	region1inRegion2	8608
CNVR39	467	2	149095217	149101135	loss	23506	145959095	151354953	Cholesterol_level	region1inRegion2	5918
CNVR38	466	2	148904898	148913506	loss-gain	23507	145959095	151354953	Cholesterol_level	region1inRegion2	8608
CNVR39	467	2	149095217	149101135	loss	23507	145959095	151354953	Cholesterol_level	region1inRegion2	5918
CNVR38	466	2	148904898	148913506	loss-gain	23508	145959095	151354953	Cholesterol_level	region1inRegion2	8608
CNVR39	467	2	149095217	149101135	loss	23508	145959095	151354953	Cholesterol_level	region1inRegion2	5918
CNVR38	466	2	148904898	148913506	loss-gain	23509	145959095	151354953	Cholesterol_level	region1inRegion2	8608
CNVR39	467	2	149095217	149101135	loss	23509	145959095	151354953	Cholesterol_level	region1inRegion2	5918
CNVR38	466	2	148904898	148913506	loss-gain	23510	145959095	151354953	Cholesterol_level	region1inRegion2	8608
CNVR39	467	2	149095217	149101135	loss	23510	145959095	151354953	Cholesterol_level	region1inRegion2	5918
CNVR38	466	2	148904898	148913506	loss-gain	23511	145959095	151354953	Cholesterol_level	region1inRegion2	8608
CNVR39	467	2	149095217	149101135	loss	23511	145959095	151354953	Cholesterol_level	region1inRegion2	5918
CNVR38	466	2	148904898	148913506	loss-gain	23512	145959095	151354953	Cholesterol_level	region1inRegion2	8608
CNVR39	467	2	149095217	149101135	loss	23512	145959095	151354953	Cholesterol_level	region1inRegion2	5918
CNVR38	466	2	148904898	148913506	loss-gain	23513	145959095	151354953	Cholesterol_level	region1inRegion2	8608
CNVR39	467	2	149095217	149101135	loss	23513	145959095	151354953	Cholesterol_level	region1inRegion2	5918
CNVR38	466	2	148904898	148913506	loss-gain	23514	145959095	151354953	Cholesterol_level	region1inRegion2	8608
CNVR39	467	2	149095217	149101135	loss	23514	145959095	151354953	Cholesterol_level	region1inRegion2	5918
CNVR38	466	2	148904898	148913506	loss-gain	23515	145959095	151354953	Cholesterol_level	region1inRegion2	8608
CNVR39	467	2	149095217	149101135	loss	23515	145959095	151354953	Cholesterol_level	region1inRegion2	5918
CNVR38	466	2	148904898	148913506	loss-gain	23516	145959095	151354953	Cholesterol_level	region1inRegion2	8608
CNVR39	467	2	149095217	149101135	loss	23516	145959095	151354953	Cholesterol_level	region1inRegion2	5918
CNVR38	466	2	148904898	148913506	loss-gain	23517	145959095	151354953	Cholesterol_level	region1inRegion2	8608
CNVR39	467	2	149095217	149101135	loss	23517	145959095	151354953	Cholesterol_level	region1inRegion2	5918
CNVR38	466	2	148904898	148913506	loss-gain	23518	145959095	151354953	Docosatetraenoic_acid_content	region1inRegion2	8608
CNVR39	467	2	149095217	149101135	loss	23518	145959095	151354953	Docosatetraenoic_acid_content	region1inRegion2	5918
CNVR38	466	2	148904898	148913506	loss-gain	23519	145959095	151354953	Cholesterol_level	region1inRegion2	8608
CNVR39	467	2	149095217	149101135	loss	23519	145959095	151354953	Cholesterol_level	region1inRegion2	5918
CNVR38	466	2	148904898	148913506	loss-gain	23520	145959095	151354953	Cholesterol_level	region1inRegion2	8608
CNVR39	467	2	149095217	149101135	loss	23520	145959095	151354953	Cholesterol_level	region1inRegion2	5918
CNVR38	466	2	148904898	148913506	loss-gain	23521	145959095	151354953	Cholesterol_level	region1inRegion2	8608
CNVR39	467	2	149095217	149101135	loss	23521	145959095	151354953	Cholesterol_level	region1inRegion2	5918
CNVR38	466	2	148904898	148913506	loss-gain	23522	145959095	151354953	Cholesterol_level	region1inRegion2	8608
CNVR39	467	2	149095217	149101135	loss	23522	145959095	151354953	Cholesterol_level	region1inRegion2	5918
CNVR38	466	2	148904898	148913506	loss-gain	23523	145959095	151354953	Cholesterol_level	region1inRegion2	8608
CNVR39	467	2	149095217	149101135	loss	23523	145959095	151354953	Cholesterol_level	region1inRegion2	5918
CNVR38	466	2	148904898	148913506	loss-gain	23524	145959095	151354953	Cholesterol_level	region1inRegion2	8608
CNVR39	467	2	149095217	149101135	loss	23524	145959095	151354953	Cholesterol_level	region1inRegion2	5918
CNVR38	466	2	148904898	148913506	loss-gain	23527	145959095	151354953	LDL_cholesterol	region1inRegion2	8608

CNVR39	467	2	149095217	149101135	loss	23527	145959095	151354953	LDL_cholesterol			region1inRegion2	5918
CNVR38	466	2	148904898	148913506	loss-gain	23528	145959095	151354953	LDL_cholesterol			region1inRegion2	8608
CNVR39	467	2	149095217	149101135	loss	23528	145959095	151354953	LDL_cholesterol			region1inRegion2	5918
CNVR38	466	2	148904898	148913506	loss-gain	23529	145959095	151354953	LDL_cholesterol			region1inRegion2	8608
CNVR39	467	2	149095217	149101135	loss	23529	145959095	151354953	LDL_cholesterol			region1inRegion2	5918
CNVR38	466	2	148904898	148913506	loss-gain	23530	145959095	151354953	LDL_cholesterol			region1inRegion2	8608
CNVR39	467	2	149095217	149101135	loss	23530	145959095	151354953	LDL_cholesterol			region1inRegion2	5918
CNVR38	466	2	148904898	148913506	loss-gain	23531	145959095	151354953	LDL_cholesterol			region1inRegion2	8608
CNVR39	467	2	149095217	149101135	loss	23531	145959095	151354953	LDL_cholesterol			region1inRegion2	5918
CNVR38	466	2	148904898	148913506	loss-gain	23532	145959095	151354953	LDL_cholesterol			region1inRegion2	8608
CNVR39	467	2	149095217	149101135	loss	23532	145959095	151354953	LDL_cholesterol			region1inRegion2	5918
CNVR38	466	2	148904898	148913506	loss-gain	23533	145959095	151354953	LDL_cholesterol			region1inRegion2	8608
CNVR39	467	2	149095217	149101135	loss	23533	145959095	151354953	LDL_cholesterol			region1inRegion2	5918
CNVR38	466	2	148904898	148913506	loss-gain	23534	145959095	151354953	LDL_cholesterol			region1inRegion2	8608
CNVR39	467	2	149095217	149101135	loss	23534	145959095	151354953	LDL_cholesterol			region1inRegion2	5918
CNVR38	466	2	148904898	148913506	loss-gain	23535	145959095	151354953	LDL_cholesterol			region1inRegion2	8608
CNVR39	467	2	149095217	149101135	loss	23535	145959095	151354953	LDL_cholesterol			region1inRegion2	5918
CNVR38	466	2	148904898	148913506	loss-gain	23536	145959095	151354953	LDL_cholesterol			region1inRegion2	8608
CNVR39	467	2	149095217	149101135	loss	23536	145959095	151354953	LDL_cholesterol			region1inRegion2	5918
CNVR38	466	2	148904898	148913506	loss-gain	23537	145959095	151354953	LDL_cholesterol			region1inRegion2	8608
CNVR39	467	2	149095217	149101135	loss	23537	145959095	151354953	LDL_cholesterol			region1inRegion2	5918
CNVR38	466	2	148904898	148913506	loss-gain	23538	145959095	151354953	LDL_cholesterol			region1inRegion2	8608
CNVR39	467	2	149095217	149101135	loss	23538	145959095	151354953	LDL_cholesterol			region1inRegion2	5918
CNVR38	466	2	148904898	148913506	loss-gain	23539	145959095	151354953	LDL_cholesterol			region1inRegion2	8608
CNVR39	467	2	149095217	149101135	loss	23539	145959095	151354953	LDL_cholesterol			region1inRegion2	5918
CNVR38	466	2	148904898	148913506	loss-gain	23540	145959095	151354953	LDL_cholesterol			region1inRegion2	8608
CNVR39	467	2	149095217	149101135	loss	23540	145959095	151354953	LDL_cholesterol			region1inRegion2	5918
CNVR38	466	2	148904898	148913506	loss-gain	23541	145959095	151354953	LDL_cholesterol			region1inRegion2	8608
CNVR39	467	2	149095217	149101135	loss	23541	145959095	151354953	LDL_cholesterol			region1inRegion2	5918
CNVR38	466	2	148904898	148913506	loss-gain	23542	145959095	151354953	LDL_cholesterol			region1inRegion2	8608
CNVR39	467	2	149095217	149101135	loss	23542	145959095	151354953	LDL_cholesterol			region1inRegion2	5918
CNVR38	466	2	148904898	148913506	loss-gain	23543	145959095	151354953	LDL_cholesterol			region1inRegion2	8608
CNVR39	467	2	149095217	149101135	loss	23543	145959095	151354953	LDL_cholesterol			region1inRegion2	5918
CNVR38	466	2	148904898	148913506	loss-gain	23544	145959095	151354953	LDL_cholesterol			region1inRegion2	8608
CNVR39	467	2	149095217	149101135	loss	23544	145959095	151354953	LDL_cholesterol			region1inRegion2	5918
CNVR38	466	2	148904898	148913506	loss-gain	23545	145959095	151354953	LDL_cholesterol			region1inRegion2	8608
CNVR39	467	2	149095217	149101135	loss	23545	145959095	151354953	LDL_cholesterol			region1inRegion2	5918
CNVR38	466	2	148904898	148913506	loss-gain	23546	145959095	151354953	LDL_cholesterol			region1inRegion2	8608
CNVR39	467	2	149095217	149101135	loss	23546	145959095	151354953	LDL_cholesterol			region1inRegion2	5918
CNVR38	466	2	148904898	148913506	loss-gain	23547	145959095	151354953	LDL_cholesterol			region1inRegion2	8608
CNVR39	467	2	149095217	149101135	loss	23547	145959095	151354953	LDL_cholesterol			region1inRegion2	5918
CNVR38	466	2	148904898	148913506	loss-gain	23548	145959095	151354953	LDL_cholesterol			region1inRegion2	8608
CNVR39	467	2	149095217	149101135	loss	23548	145959095	151354953	LDL_cholesterol			region1inRegion2	5918
CNVR38	466	2	148904898	148913506	loss-gain	23549	145959095	151354953	LDL_cholesterol			region1inRegion2	8608
CNVR39	467	2	149095217	149101135	loss	23549	145959095	151354953	LDL_cholesterol			region1inRegion2	5918
CNVR38	466	2	148904898	148913506	loss-gain	23550	145959095	151354953	LDL_cholesterol			region1inRegion2	8608
CNVR39	467	2	149095217	149101135	loss	23550	145959095	151354953	LDL_cholesterol			region1inRegion2	5918
CNVR38	466	2	148904898	148913506	loss-gain	23551	145959095	151354953	LDL_cholesterol			region1inRegion2	8608
CNVR39	467	2	149095217	149101135	loss	23551	145959095	151354953	LDL_cholesterol			region1inRegion2	5918
CNVR38	466	2	148904898	148913506	loss-gain	23552	145959095	151354953	LDL_cholesterol			region1inRegion2	8608
CNVR39	467	2	149095217	149101135	loss	23552	145959095	151354953	LDL_cholesterol			region1inRegion2	5918
CNVR38	466	2	148904898	148913506	loss-gain	23553	145959095	151354953	LDL_cholesterol			region1inRegion2	8608
CNVR39	467	2	149095217	149101135	loss	23553	145959095	151354953	LDL_cholesterol			region1inRegion2	5918
CNVR38	466	2	148904898	148913506	loss-gain	23554	145959095	151354953	LDL_cholesterol			region1inRegion2	8608
CNVR39	467	2	149095217	149101135	loss	23554	145959095	151354953	LDL_cholesterol			region1inRegion2	5918
CNVR38	466	2	148904898	148913506	loss-gain	23555	145959095	151354953	LDL_cholesterol			region1inRegion2	8608
CNVR39	467	2	149095217	149101135	loss	23555	145959095	151354953	LDL_cholesterol			region1inRegion2	5918
CNVR38	466	2	148904898	148913506	loss-gain	23556	145959095	151354953	LDL_cholesterol			region1inRegion2	8608
CNVR39	467	2	149095217	149101135	loss	23556	145959095	151354953	LDL_cholesterol			region1inRegion2	5918
CNVR38	466	2	148904898	148913506	loss-gain	23557	145959095	151354953	LDL_cholesterol			region1inRegion2	8608
CNVR39	467	2	149095217	149101135	loss	23557	145959095	151354953	LDL_cholesterol			region1inRegion2	5918
CNVR38	466	2	148904898	148913506	loss-gain	23558	145959095	151354953	LDL_cholesterol			region1inRegion2	8608
CNVR39	467	2	149095217	149101135	loss	23558	145959095	151354953	LDL_cholesterol			region1inRegion2	5918
CNVR38	466	2	148904898	148913506	loss-gain	23559	145959095	151354953	LDL_cholesterol			region1inRegion2	8608
CNVR39	467	2	149095217	149101135	loss	23559	145959095	151354953	LDL_cholesterol			region1inRegion2	5918
CNVR38	466	2	148904898	148913506	loss-gain	23560	145959095	151354953	LDL_cholesterol			region1inRegion2	8608
CNVR39	467	2	149095217	149101135	loss	23560	145959095	151354953	LDL_cholesterol			region1inRegion2	5918
CNVR38	466	2	148904898	148913506	loss-gain	23561	145959095	151354953	LDL_cholesterol			region1inRegion2	8608
CNVR39	467	2	149095217	149101135	loss	23561	145959095	151354953	LDL_cholesterol			region1inRegion2	5918
CNVR38	466	2	148904898	148913506	loss-gain	23562	145959095	151354953	LDL_cholesterol			region1inRegion2	8608
CNVR39	467	2	149095217	149101135	loss	23562	145959095	151354953	LDL_cholesterol			region1inRegion2	5918
CNVR38	466	2	148904898	148913506	loss-gain	23563	145959095	151354953	LDL_cholesterol			region1inRegion2	8608
CNVR39	467	2	149095217	149101135	loss	23563	145959095	151354953	LDL_cholesterol			region1inRegion2	5918
CNVR38	466	2	148904898	148913506	loss-gain	23564	145959095	151354953	LDL_cholesterol			region1inRegion2	8608
CNVR39	467	2	149095217	149101135	loss	23564	145959095	151354953	LDL_cholesterol			region1inRegion2	5918
CNVR38	466	2	148904898	148913506	loss-gain	23565	145959095	151354953	LDL_cholesterol			region1inRegion2	8608
CNVR39	467	2	149095217	149101135	loss	23565	145959095	151354953	LDL_cholesterol			region1inRegion2	5918
CNVR38	466	2	148904898	148913506	loss-gain	23566	145959095	151354953	LDL_cholesterol			region1inRegion2	8608
CNVR39	467	2	149095217	149101135	loss	23566	145959095	151354953	LDL_cholesterol			region1inRegion2	5918
CNVR38	466	2	148904898	148913506	loss-gain	23567	145959095	151354953	LDL_cholesterol			region1inRegion2	8608
CNVR39	467	2	149095217	149101135	loss	23567	145959095	151354953	LDL_cholesterol			region1inRegion2	5918
CNVR38	466	2	148904898	148913506	loss-gain	23568	145959095	151354953	LDL_cholesterol			region1inRegion2	8608
CNVR39	467	2	149095217	149101135	loss	23568	145959095	151354953	LDL_cholesterol			region1inRegion2	5918
CNVR38	466	2	148904898	148913506	loss-gain	23569	145959095	151354953	LDL_cholesterol			region1inRegion2	8608
CNVR39	467	2	149095217	149101135	loss	23569	145959095	151354953	LDL_cholesterol			region1inRegion2	5918
CNVR38	466	2	148904898	148913506	loss-gain	23570	145959095	151354953	LDL_cholesterol			region1inRegion2	8608
CNVR39	467	2	149095217	149101135	loss	23570	145959095	151354953	LDL_cholesterol			region1inRegion2	5918
CNVR38	466	2	148904898	148913506	loss-gain	23571	145959095	151354953	LDL_cholesterol			region1inRegion2	8608
CNVR39	467	2	149095217	149101135	loss	23571	145959095	151354953	LDL_cholesterol			region1inRegion2	5918
CNVR38	466	2	148904898	148913506	loss-gain	23572	145959095	151354953	LDL_cholesterol			region1inRegion2	8608
CNVR39	467	2	149095217	149101135	loss	23572	145959095	151354953	LDL_cholesterol			region1inRegion2	5918
CNVR38	466	2	148904898	148913506	loss-gain	23573	145959095	151354953	LDL_cholesterol			region1inRegion2	8608
CNVR39	467	2	149095217	149101135	loss	23573	145959095	151354953	LDL_cholesterol			region1inRegion2	5918
CNVR38	466	2	148904898	148913506	loss-gain	23574	145959095	151354953	LDL_cholesterol			region1inRegion2	8608
CNVR39	467	2	149095217	149101135	loss	23574	145959095	151354953	LDL_cholesterol			region1inRegion2	5918
CNVR38	466	2	148904898	148913506	loss-gain	23575	145959095	151354953	LDL_cholesterol			region1inRegion2	8608
CNVR39	467	2	149095217	149101135	loss	23575	145959095	151354953	LDL_cholesterol			region1inRegion2	5918
CNVR38	466	2	148904898	148913506	loss-gain	23576	145959095	151354953	LDL_cholesterol			region1inRegion2	8608
CNVR39	467	2	149095217	149101135	loss	23576	145959095	151354953	LDL_cholesterol			region1inRegion2	5918
CNVR38	466	2	148904898	148913506	loss-gain	23577	145959095	151354953	LDL_cholesterol			region1inRegion2	8608
CNVR39	467	2	149095217	149101135	loss	23577	145959095	151354953	LDL_cholesterol			region1inRegion2	5918
CNVR38	466	2	148904898	148913506	loss-gain	23578	145959095	151354953	LDL_cholesterol			region1inRegion2	8608
CNVR39	467	2	149095217	149101135	loss	23578	145959095	151354953	LDL_cholesterol			region1inRegion2	5918
CNVR38	466	2	148904898	148913506	loss-gain	23579	145959095	151354953	LDL_cholesterol			region1inRegion2	8608
CNVR39	467	2	149095217	149101135	loss	23579	145959095	151354953	LDL_cholesterol			region1inRegion2	5918
CNVR38	466	2	148904898	148913506	loss-gain	23580	145959095	151354953	LDL_cholesterol			region1inRegion2	8608
CNVR39	467	2	149095217	149101135	loss	23580	145959095	151354953	LDL_cholesterol			region1inRegion2	5918
CNVR38	466	2	148904898	148913506	loss-gain	23581	145959095	151354953	LDL_cholesterol			region1inRegion2	8608
CNVR39	467	2	149095217	149101135	loss	23581	145959095	151354953	LDL_cholesterol			region1inRegion2	5918
CNVR38	466	2	148904898	148913506	loss-gain	23582	145959095	151354953	LDL_cholesterol			region1inRegion2	8608
CNVR39	467	2	149095217	149101135	loss	23582	145959095	151354953	LDL_cholesterol			region1inRegion2	5918
CNVR38	466	2	148904898	148913506	loss-gain	23583	145959095	151354953	LDL_cholesterol			region1inRegion2	8608
CNVR39	467	2	149095217	149101135	loss	23583	145959095	151354953	LDL_cholesterol			region1inRegion2	5918
CNVR38	466	2	148904898	148913506	loss-gain	23584	145959095	151354953	LDL_cholesterol			region1inRegion2	8608
CNVR39	467	2	149095217	149101135	loss	23584	145959095	151354953	LDL_cholesterol			region1inRegion2	5918
CNVR38	466	2	148904898	148913506	loss-gain	23585	145959095	151354953	LDL_cholesterol			region1inRegion2	8608
CNVR39	467	2	149095217	149101135	loss	23585	145959095	151354953	LDL_cholesterol			region1inRegion2	5918
CNVR38	466	2	148904898	148913506	loss-gain	23586	145959095	151354953	LDL_cholesterol			region1inRegion2	8608
CNVR39	467	2	149095217	149101135	loss	23586	145959095	151354953	LDL_cholesterol			region1inRegion2	5918
CNVR38	466	2	148904898	148913506	loss-gain	23587	145959095	151354953	LDL_cholesterol			region1inRegion2	8608
CNVR39	467	2	149095217	149101135	loss	23587	145959095	151354953	LDL_cholesterol			region1inRegion2	5918
CNVR38	466	2	148904898	148913506	loss-gain	23588	145959095	151354953	LDL_cholesterol			region1inRegion2	8608

CNVR39	467	2	149095217	149101135	loss	23588	145959095	151354953	LDL_cholesterol	region1inRegion2	5918
CNVR38	466	2	148904898	148913506	loss-gain	23589	145959095	151354953	LDL_cholesterol	region1inRegion2	8608
CNVR39	467	2	149095217	149101135	loss	23589	145959095	151354953	LDL_cholesterol	region1inRegion2	5918
CNVR38	466	2	148904898	148913506	loss-gain	23590	145959095	151354953	LDL_cholesterol	region1inRegion2	8608
CNVR39	467	2	149095217	149101135	loss	23590	145959095	151354953	LDL_cholesterol	region1inRegion2	5918
CNVR38	466	2	148904898	148913506	loss-gain	23591	145959095	151354953	LDL_cholesterol	region1inRegion2	8608
CNVR39	467	2	149095217	149101135	loss	23591	145959095	151354953	LDL_cholesterol	region1inRegion2	5918
CNVR38	466	2	148904898	148913506	loss-gain	23592	145959095	151354953	LDL_cholesterol	region1inRegion2	8608
CNVR39	467	2	149095217	149101135	loss	23592	145959095	151354953	LDL_cholesterol	region1inRegion2	5918
CNVR38	466	2	148904898	148913506	loss-gain	23593	145959095	151354953	LDL_cholesterol	region1inRegion2	8608
CNVR39	467	2	149095217	149101135	loss	23593	145959095	151354953	LDL_cholesterol	region1inRegion2	5918
CNVR38	466	2	148904898	148913506	loss-gain	23594	145959095	151354953	LDL_cholesterol	region1inRegion2	8608
CNVR39	467	2	149095217	149101135	loss	23594	145959095	151354953	LDL_cholesterol	region1inRegion2	5918
CNVR38	466	2	148904898	148913506	loss-gain	23595	145959095	151354953	LDL_cholesterol	region1inRegion2	8608
CNVR39	467	2	149095217	149101135	loss	23595	145959095	151354953	LDL_cholesterol	region1inRegion2	5918
CNVR38	466	2	148904898	148913506	loss-gain	23596	145959095	151354953	LDL_cholesterol	region1inRegion2	8608
CNVR39	467	2	149095217	149101135	loss	23596	145959095	151354953	LDL_cholesterol	region1inRegion2	5918
CNVR38	466	2	148904898	148913506	loss-gain	23601	145959095	151354953	HDL/LDL_ratio	region1inRegion2	8608
CNVR39	467	2	149095217	149101135	loss	23601	145959095	151354953	HDL/LDL_ratio	region1inRegion2	5918
CNVR38	466	2	148904898	148913506	loss-gain	23602	145959095	151354953	HDL/LDL_ratio	region1inRegion2	8608
CNVR39	467	2	149095217	149101135	loss	23602	145959095	151354953	HDL/LDL_ratio	region1inRegion2	5918
CNVR38	466	2	148904898	148913506	loss-gain	23603	145959095	151354953	HDL/LDL_ratio	region1inRegion2	8608
CNVR39	467	2	149095217	149101135	loss	23603	145959095	151354953	HDL/LDL_ratio	region1inRegion2	5918
CNVR38	466	2	148904898	148913506	loss-gain	23604	145959095	151354953	HDL/LDL_ratio	region1inRegion2	8608
CNVR39	467	2	149095217	149101135	loss	23604	145959095	151354953	HDL/LDL_ratio	region1inRegion2	5918
CNVR38	466	2	148904898	148913506	loss-gain	23605	145959095	151354953	HDL/LDL_ratio	region1inRegion2	8608
CNVR39	467	2	149095217	149101135	loss	23605	145959095	151354953	HDL/LDL_ratio	region1inRegion2	5918
CNVR38	466	2	148904898	148913506	loss-gain	23606	145959095	151354953	HDL/LDL_ratio	region1inRegion2	8608
CNVR39	467	2	149095217	149101135	loss	23606	145959095	151354953	HDL/LDL_ratio	region1inRegion2	5918
CNVR38	466	2	148904898	148913506	loss-gain	23607	145959095	151354953	HDL/LDL_ratio	region1inRegion2	8608
CNVR39	467	2	149095217	149101135	loss	23607	145959095	151354953	HDL/LDL_ratio	region1inRegion2	5918
CNVR38	466	2	148904898	148913506	loss-gain	23608	145959095	151354953	HDL/LDL_ratio	region1inRegion2	8608
CNVR39	467	2	149095217	149101135	loss	23608	145959095	151354953	HDL/LDL_ratio	region1inRegion2	5918
CNVR38	466	2	148904898	148913506	loss-gain	23609	145959095	151354953	HDL/LDL_ratio	region1inRegion2	8608
CNVR39	467	2	149095217	149101135	loss	23609	145959095	151354953	HDL/LDL_ratio	region1inRegion2	5918
CNVR38	466	2	148904898	148913506	loss-gain	24120	145959095	151354953	Cortisol_level	region1inRegion2	8608
CNVR39	467	2	149095217	149101135	loss	24120	145959095	151354953	Cortisol_level	region1inRegion2	5918
CNVR38	466	2	148904898	148913506	loss-gain	24121	145959095	151354953	Cortisol_level	region1inRegion2	8608
CNVR39	467	2	149095217	149101135	loss	24121	145959095	151354953	Cortisol_level	region1inRegion2	5918
CNVR38	466	2	148904898	148913506	loss-gain	24122	145959095	151354953	Cortisol_level	region1inRegion2	8608
CNVR39	467	2	149095217	149101135	loss	24122	145959095	151354953	Cortisol_level	region1inRegion2	5918
CNVR38	466	2	148904898	148913506	loss-gain	24123	145959095	151354953	Cortisol_level	region1inRegion2	8608
CNVR39	467	2	149095217	149101135	loss	24123	145959095	151354953	Cortisol_level	region1inRegion2	5918
CNVR38	466	2	148904898	148913506	loss-gain	24124	145959095	151354953	Cortisol_level	region1inRegion2	8608
CNVR39	467	2	149095217	149101135	loss	24124	145959095	151354953	Cortisol_level	region1inRegion2	5918
CNVR38	466	2	148904898	148913506	loss-gain	24125	145959095	151354953	Cortisol_level	region1inRegion2	8608
CNVR39	467	2	149095217	149101135	loss	24125	145959095	151354953	Cortisol_level	region1inRegion2	5918
CNVR38	466	2	148904898	148913506	loss-gain	24126	145959095	151354953	Cortisol_level	region1inRegion2	8608
CNVR39	467	2	149095217	149101135	loss	24126	145959095	151354953	Cortisol_level	region1inRegion2	5918
CNVR38	466	2	148904898	148913506	loss-gain	24127	145959095	151354953	Cortisol_level	region1inRegion2	8608
CNVR39	467	2	149095217	149101135	loss	24127	145959095	151354953	Cortisol_level	region1inRegion2	5918
CNVR38	466	2	148904898	148913506	loss-gain	24152	145959095	151354953	Adrenal_gland_weight	region1inRegion2	8608
CNVR39	467	2	149095217	149101135	loss	24152	145959095	151354953	Adrenal_gland_weight	region1inRegion2	5918
CNVR38	466	2	148904898	148913506	loss-gain	24153	145959095	151354953	Adrenal_gland_weight	region1inRegion2	8608
CNVR39	467	2	149095217	149101135	loss	24153	145959095	151354953	Adrenal_gland_weight	region1inRegion2	5918
CNVR38	466	2	148904898	148913506	loss-gain	24154	145959095	151354953	Adrenal_gland_weight	region1inRegion2	8608
CNVR39	467	2	149095217	149101135	loss	24154	145959095	151354953	Adrenal_gland_weight	region1inRegion2	5918
CNVR38	466	2	148904898	148913506	loss-gain	24155	145959095	151354953	Adrenal_gland_weight	region1inRegion2	8608
CNVR39	467	2	149095217	149101135	loss	24155	145959095	151354953	Adrenal_gland_weight	region1inRegion2	5918
CNVR38	466	2	148904898	148913506	loss-gain	24156	145959095	151354953	Adrenal_gland_weight	region1inRegion2	8608
CNVR39	467	2	149095217	149101135	loss	24156	145959095	151354953	Adrenal_gland_weight	region1inRegion2	5918
CNVR38	466	2	148904898	148913506	loss-gain	24157	145959095	151354953	Adrenal_gland_weight	region1inRegion2	8608
CNVR39	467	2	149095217	149101135	loss	24157	145959095	151354953	Adrenal_gland_weight	region1inRegion2	5918
CNVR38	466	2	148904898	148913506	loss-gain	24158	145959095	151354953	Adrenal_gland_weight	region1inRegion2	8608
CNVR39	467	2	149095217	149101135	loss	24158	145959095	151354953	Adrenal_gland_weight	region1inRegion2	5918
CNVR38	466	2	148904898	148913506	loss-gain	24159	145959095	151354953	Adrenal_gland_weight	region1inRegion2	8608
CNVR39	467	2	149095217	149101135	loss	24159	145959095	151354953	Adrenal_gland_weight	region1inRegion2	5918
CNVR38	466	2	148904898	148913506	loss-gain	24160	145959095	151354953	Adrenal_gland_weight	region1inRegion2	8608
CNVR39	467	2	149095217	149101135	loss	24160	145959095	151354953	Adrenal_gland_weight	region1inRegion2	5918
CNVR38	466	2	148904898	148913506	loss-gain	24161	145959095	151354953	Adrenal_gland_weight	region1inRegion2	8608
CNVR39	467	2	149095217	149101135	loss	24161	145959095	151354953	Adrenal_gland_weight	region1inRegion2	5918
CNVR38	466	2	148904898	148913506	loss-gain	24162	145959095	151354953	Adrenal_gland_weight	region1inRegion2	8608
CNVR39	467	2	149095217	149101135	loss	24162	145959095	151354953	Adrenal_gland_weight	region1inRegion2	5918
CNVR38	466	2	148904898	148913506	loss-gain	24163	145959095	151354953	Adrenal_gland_weight	region1inRegion2	8608
CNVR39	467	2	149095217	149101135	loss	24163	145959095	151354953	Adrenal_gland_weight	region1inRegion2	5918
CNVR38	466	2	148904898	148913506	loss-gain	24164	145959095	151354953	Adrenal_gland_weight	region1inRegion2	8608
CNVR39	467	2	149095217	149101135	loss	24164	145959095	151354953	Adrenal_gland_weight	region1inRegion2	5918
CNVR38	466	2	148904898	148913506	loss-gain	24165	145959095	151354953	Adrenal_gland_weight	region1inRegion2	8608
CNVR39	467	2	149095217	149101135	loss	24165	145959095	151354953	Adrenal_gland_weight	region1inRegion2	5918
CNVR38	466	2	148904898	148913506	loss-gain	24166	145959095	151354953	Adrenal_gland_weight	region1inRegion2	8608
CNVR39	467	2	149095217	149101135	loss	24166	145959095	151354953	Adrenal_gland_weight	region1inRegion2	5918
CNVR38	466	2	148904898	148913506	loss-gain	24167	145959095	151354953	Adrenal_gland_weight	region1inRegion2	8608
CNVR39	467	2	149095217	149101135	loss	24167	145959095	151354953	Adrenal_gland_weight	region1inRegion2	5918
CNVR38	466	2	148904898	148913506	loss-gain	24168	145959095	151354953	Adrenal_gland_weight	region1inRegion2	8608
CNVR39	467	2	149095217	149101135	loss	24168	145959095	151354953	Adrenal_gland_weight	region1inRegion2	5918
CNVR38	466	2	148904898	148913506	loss-gain	24169	145959095	151354953	Adrenal_gland_weight	region1inRegion2	8608
CNVR39	467	2	149095217	149101135	loss	24169	145959095	151354953	Adrenal_gland_weight	region1inRegion2	5918
CNVR38	466	2	148904898	148913506	loss-gain	24170	145959095	151354953	Adrenal_gland_weight	region1inRegion2	8608
CNVR39	467	2	149095217	149101135	loss	24170	145959095	151354953	Adrenal_gland_weight	region1inRegion2	5918
CNVR38	466	2	148904898	148913506	loss-gain	24171	145959095	151354953	Adrenal_gland_weight	region1inRegion2	8608
CNVR39	467	2	149095217	149101135	loss	24171	145959095	151354953	Adrenal_gland_weight	region1inRegion2	5918
CNVR38	466	2	148904898	148913506	loss-gain	24172	145959095	151354953	Adrenal_gland_weight	region1inRegion2	8608
CNVR39	467	2	149095217	149101135	loss	24172	145959095	151354953	Adrenal_gland_weight	region1inRegion2	5918
CNVR38	466	2	148904898	148913506	loss-gain	24173	145959095	151354953	Adrenal_gland_weight	region1inRegion2	8608
CNVR39	467	2	149095217	149101135	loss	24173	145959095	151354953	Adrenal_gland_weight	region1inRegion2	5918
CNVR38	466	2	148904898	148913506	loss-gain	24174	145959095	151354953	Adrenal_gland_weight	region1inRegion2	8608
CNVR39	467	2	149095217	149101135	loss	24174	145959095	151354953	Adrenal_gland_weight	region1inRegion2	5918
CNVR38	466	2	148904898	148913506	loss-gain	24175	145959095	151354953	Adrenal_gland_weight	region1inRegion2	8608
CNVR39	467	2	149095217	149101135	loss	24175	145959095	151354953	Adrenal_gland_weight	region1inRegion2	5918
CNVR38	466	2	148904898	148913506	loss-gain	24176	145959095	151354953	Adrenal_gland_weight	region1inRegion2	8608
CNVR39	467	2	149095217	149101135	loss	24176	145959095	151354953	Adrenal_gland_weight	region1inRegion2	5918
CNVR38	466	2	148904898	148913506	loss-gain	24177	145959095	151354953	Adrenal_gland_weight	region1inRegion2	8608
CNVR39	467	2	149095217	149101135	loss	24177	145959095	151354953	Adrenal_gland_weight	region1inRegion2	5918
CNVR38	466	2	148904898	148913506	loss-gain	24178	145959095	151354953	Adrenal_gland_weight	region1inRegion2	8608
CNVR39	467	2	149095217	149101135	loss	24178	145959095	151354953	Adrenal_gland_weight	region1inRegion2	5918
CNVR38	466	2	148904898	148913506	loss-gain	24179	145959095	151354953	Adrenal_gland_weight	region1inRegion2	8608
CNVR39	467	2	149095217	149101135	loss	24179	145959095	151354953	Adrenal_gland_weight	region1inRegion2	5918
CNVR38	466	2	148904898	148913506	loss-gain	24180	145959095	151354953	Adrenal_gland_weight	region1inRegion2	8608
CNVR39	467	2	149095217	149101135	loss	24180	145959095	151354953	Adrenal_gland_weight	region1inRegion2	5918
CNVR38	466	2	148904898	148913506	loss-gain	24181	145959095	151354953	Adrenal_gland_weight	region1inRegion2	8608
CNVR39	467	2	149095217	149101135	loss	24181	145959095	151354953	Adrenal_gland_weight	region1inRegion2	5918
CNVR38	466	2	148904898	148913506	loss-gain	24182	145959095	151354953	Adrenal_gland_weight	region1inRegion2	8608
CNVR39	467	2	149095217	149101135	loss	24182	145959095	151354953	Adrenal_gland_weight	region1inRegion2	5918
CNVR38	466	2	148904898	148913506	loss-gain	24183	145959095	151354953	Adrenal_gland_weight	region1inRegion2	8608
CNVR39	467	2	149095217	149101135	loss	24183	145959095	151354953	Adrenal_gland_weight	region1inRegion2	5918
CNVR38	466	2	148904898	148913506	loss-gain	24184	145959095	151354953	Adrenal_gland_weight	region1inRegion2	8608
CNVR39	467	2	149095217	149101135	loss	24184	145959095	151354953	Adrenal_gland_weight	region1inRegion2	5918
CNVR38	466	2	148904898	148913506	loss-gain	24185	145959095	151354953	Adrenal_gland_weight	region1inRegion2	8608
CNVR39	467	2	149095217	149101135	loss	24185	145959095	151354953	Adrenal_gland_weight	region1inRegion2	5918
CNVR38	466	2	148904898	148913506	loss-gain	24186	145959095	151354953	Adrenal_gland_weight	region1inRegion2	8608
CNVR39	467	2	149095217	149101135	loss	24186	145959095	151354953	Adrenal_gland_weight	region1inRegion2	5918
CNVR38	466	2	148904898	148913506	loss-gain	24187	145959095	151354953	Adrenal_gland_weight	region1inRegion2	8608

CNVR39	467	2	149095217	149101135	loss	24187	145959095	151354953	Adrenal_gland_weight	region1inRegion2	5918
CNVR38	466	2	148904898	148913506	loss-gain	24188	145959095	151354953	Adrenal_gland_weight	region1inRegion2	8608
CNVR39	467	2	149095217	149101135	loss	24188	145959095	151354953	Adrenal_gland_weight	region1inRegion2	5918
CNVR38	466	2	148904898	148913506	loss-gain	24189	145959095	151354953	Adrenal_gland_weight	region1inRegion2	8608
CNVR39	467	2	149095217	149101135	loss	24189	145959095	151354953	Adrenal_gland_weight	region1inRegion2	5918
CNVR38	466	2	148904898	148913506	loss-gain	24190	145959095	151354953	Adrenal_gland_weight	region1inRegion2	8608
CNVR39	467	2	149095217	149101135	loss	24190	145959095	151354953	Adrenal_gland_weight	region1inRegion2	5918
CNVR38	466	2	148904898	148913506	loss-gain	24191	145959095	151354953	Adrenal_gland_weight	region1inRegion2	8608
CNVR39	467	2	149095217	149101135	loss	24191	145959095	151354953	Adrenal_gland_weight	region1inRegion2	5918
CNVR38	466	2	148904898	148913506	loss-gain	24235	145959095	151354953	Drip_loss	region1inRegion2	8608
CNVR39	467	2	149095217	149101135	loss	24235	145959095	151354953	Drip_loss	region1inRegion2	5918
CNVR38	466	2	148904898	148913506	loss-gain	24236	145959095	151354953	leaf_fat_percentage	region1inRegion2	8608
CNVR39	467	2	149095217	149101135	loss	24236	145959095	151354953	leaf_fat_percentage	region1inRegion2	5918
CNVR38	466	2	148904898	148913506	loss-gain	6359	148081154	154953107	Lactate_level	region1inRegion2	8608
CNVR39	467	2	149095217	149101135	loss	6359	148081154	154953107	Lactate_level	region1inRegion2	5918
CNVR40	471	2	156079099	156090392	gain	171	151463344	158935864	Average_daily_gain	region1inRegion2	11293
CNVR41	472	2	158544152	158558867	gain	171	151463344	158935864	Average_daily_gain	region1inRegion2	14715
CNVR42	473	2	158605784	158612295	gain	171	151463344	158935864	Average_daily_gain	region1inRegion2	6511
CNVR42	473	2	158605784	158629757	gain	171	151463344	158935864	Average_daily_gain	region1inRegion2	23973
CNVR40	471	2	156079099	156090392	gain	89	154953107	157789611	chew_score	region1inRegion2	11293
CNVR40	471	2	156079099	156090392	gain	90	154953107	157789611	tenderness_score	region1inRegion2	11293
CNVR41	472	2	158544152	158558867	gain	2799	157916972	162569375	Total_muscle_fiber_number	region1inRegion2	14715
CNVR42	473	2	158605784	158612295	gain	2799	157916972	162569375	Total_muscle_fiber_number	region1inRegion2	6511
CNVR42	473	2	158605784	158629757	gain	2799	157916972	162569375	Total_muscle_fiber_number	region1inRegion2	23973
CNVR44	479	2	159094998	159110351	gain	2799	157916972	162569375	Total_muscle_fiber_number	region1inRegion2	15353
CNVR45	487	2	160893564	160895760	gain	2799	157916972	162569375	Total_muscle_fiber_number	region1inRegion2	2196
CNVR41	472	2	158544152	158558867	gain	2800	157916972	162569375	Number_of_muscle_fibers_per_unit_area	region1inRegion2	14715
CNVR42	473	2	158605784	158612295	gain	2800	157916972	162569375	Number_of_muscle_fibers_per_unit_area	region1inRegion2	6511
CNVR42	473	2	158605784	158629757	gain	2800	157916972	162569375	Number_of_muscle_fibers_per_unit_area	region1inRegion2	23973
CNVR44	479	2	159094998	159110351	gain	2800	157916972	162569375	Number_of_muscle_fibers_per_unit_area	region1inRegion2	15353
CNVR45	487	2	160893564	160895760	gain	2800	157916972	162569375	Number_of_muscle_fibers_per_unit_area	region1inRegion2	2196
CNVR46	504	3	45023435	45241189	gain	15085	1456046	98066155	Lactate_dehydrogenase_level	region1inRegion2	217754
CNVR47	510	3	56589884	56595835	loss	15085	1456046	98066155	Lactate_dehydrogenase_level	region1inRegion2	5951
CNVR48	519	3	61793403	61809935	loss	15085	1456046	98066155	Lactate_dehydrogenase_level	region1inRegion2	16532
CNVR49	524	3	67785626	67805349	gain	15085	1456046	98066155	Lactate_dehydrogenase_level	region1inRegion2	19723
CNVR50	539	3	83892565	83943514	gain	15085	1456046	98066155	Lactate_dehydrogenase_level	region1inRegion2	50949
CNVR46	504	3	45023435	45241189	gain	5224	1456046	130209174	Teat_number	region1inRegion2	217754
CNVR47	510	3	56589884	56595835	loss	5224	1456046	130209174	Teat_number	region1inRegion2	5951
CNVR48	519	3	61793403	61809935	loss	5224	1456046	130209174	Teat_number	region1inRegion2	16532
CNVR49	524	3	67785626	67805349	gain	5224	1456046	130209174	Teat_number	region1inRegion2	19723
CNVR50	539	3	83892565	83943514	gain	5224	1456046	130209174	Teat_number	region1inRegion2	50949
CNVR46	504	3	45023435	45241189	gain	38077	1456046	140360108	Loin_muscle_area	region1inRegion2	217754
CNVR47	510	3	56589884	56595835	loss	38077	1456046	140360108	Loin_muscle_area	region1inRegion2	5951
CNVR48	519	3	61793403	61809935	loss	38077	1456046	140360108	Loin_muscle_area	region1inRegion2	16532
CNVR49	524	3	67785626	67805349	gain	38077	1456046	140360108	Loin_muscle_area	region1inRegion2	19723
CNVR50	539	3	83892565	83943514	gain	38077	1456046	140360108	Loin_muscle_area	region1inRegion2	50949
CNVR46	504	3	45023435	45241189	gain	38078	1456046	140360108	Shear_force	region1inRegion2	217754
CNVR47	510	3	56589884	56595835	loss	38078	1456046	140360108	Shear_force	region1inRegion2	5951
CNVR48	519	3	61793403	61809935	loss	38078	1456046	140360108	Shear_force	region1inRegion2	16532
CNVR49	524	3	67785626	67805349	gain	38078	1456046	140360108	Shear_force	region1inRegion2	19723
CNVR50	539	3	83892565	83943514	gain	38078	1456046	140360108	Shear_force	region1inRegion2	50949
CNVR46	504	3	45023435	45241189	gain	38080	1456046	140360108	CIE-b*	region1inRegion2	217754
CNVR47	510	3	56589884	56595835	loss	38080	1456046	140360108	CIE-b*	region1inRegion2	5951
CNVR48	519	3	61793403	61809935	loss	38080	1456046	140360108	CIE-b*	region1inRegion2	16532
CNVR49	524	3	67785626	67805349	gain	38080	1456046	140360108	CIE-b*	region1inRegion2	19723
CNVR50	539	3	83892565	83943514	gain	38080	1456046	140360108	CIE-b*	region1inRegion2	50949
CNVR46	504	3	45023435	45241189	gain	38081	1456046	140360108	Meat_color_chroma	region1inRegion2	217754
CNVR47	510	3	56589884	56595835	loss	38081	1456046	140360108	Meat_color_chroma	region1inRegion2	5951
CNVR48	519	3	61793403	61809935	loss	38081	1456046	140360108	Meat_color_chroma	region1inRegion2	16532
CNVR49	524	3	67785626	67805349	gain	38081	1456046	140360108	Meat_color_chroma	region1inRegion2	19723
CNVR50	539	3	83892565	83943514	gain	38081	1456046	140360108	Meat_color_chroma	region1inRegion2	50949
CNVR46	504	3	45023435	45241189	gain	515	4571903	82495610	Corpus_luteum_number	region1inRegion2	217754
CNVR47	510	3	56589884	56595835	loss	515	4571903	82495610	Corpus_luteum_number	region1inRegion2	5951
CNVR48	519	3	61793403	61809935	loss	515	4571903	82495610	Corpus_luteum_number	region1inRegion2	16532
CNVR49	524	3	67785626	67805349	gain	515	4571903	82495610	Corpus_luteum_number	region1inRegion2	19723
CNVR46	504	3	45023435	45241189	gain	5234	4656907	140465858	Body_weight_(end_of_test)	region1inRegion2	217754
CNVR47	510	3	56589884	56595835	loss	5234	4656907	140465858	Body_weight_(end_of_test)	region1inRegion2	5951
CNVR48	519	3	61793403	61809935	loss	5234	4656907	140465858	Body_weight_(end_of_test)	region1inRegion2	16532
CNVR49	524	3	67785626	67805349	gain	5234	4656907	140465858	Body_weight_(end_of_test)	region1inRegion2	19723
CNVR50	539	3	83892565	83943514	gain	5234	4656907	140465858	Body_weight_(end_of_test)	region1inRegion2	50949
CNVR46	504	3	45023435	45241189	gain	38079	6188004	140360108	Cooking_loss	region1inRegion2	217754
CNVR47	510	3	56589884	56595835	loss	38079	6188004	140360108	Cooking_loss	region1inRegion2	5951
CNVR48	519	3	61793403	61809935	loss	38079	6188004	140360108	Cooking_loss	region1inRegion2	16532
CNVR49	524	3	67785626	67805349	gain	38079	6188004	140360108	Cooking_loss	region1inRegion2	19723
CNVR50	539	3	83892565	83943514	gain	38079	6188004	140360108	Cooking_loss	region1inRegion2	50949
CNVR46	504	3	45023435	45241189	gain	300	13949105	49051751	pH_24_hr_post-mortem_(loin)	region1inRegion2	217754
CNVR46	504	3	45023435	45241189	gain	17639	14776389	62780348	Osteochondrosis_score	region1inRegion2	217754
CNVR47	510	3	56589884	56595835	loss	17639	14776389	62780348	Osteochondrosis_score	region1inRegion2	5951
CNVR48	519	3	61793403	61809935	loss	17639	14776389	62780348	Osteochondrosis_score	region1inRegion2	16532
CNVR46	504	3	45023435	45241189	gain	17716	14776389	62780348	HDL_cholesterol	region1inRegion2	217754
CNVR47	510	3	56589884	56595835	loss	17716	14776389	62780348	HDL_cholesterol	region1inRegion2	5951
CNVR48	519	3	61793403	61809935	loss	17716	14776389	62780348	HDL_cholesterol	region1inRegion2	16532
CNVR46	504	3	45023435	45241189	gain	2802	14776389	62780348	Number_of_muscle_fibers_per_unit_area	region1inRegion2	217754
CNVR47	510	3	56589884	56595835	loss	2802	14776389	62780348	Number_of_muscle_fibers_per_unit_area	region1inRegion2	5951
CNVR48	519	3	61793403	61809935	loss	2802	14776389	62780348	Number_of_muscle_fibers_per_unit_area	region1inRegion2	16532
CNVR46	504	3	45023435	45241189	gain	2803	14776389	62780348	Number_of_capillaries_per_type_I_fiber	region1inRegion2	217754
CNVR47	510	3	56589884	56595835	loss	2803	14776389	62780348	Number_of_capillaries_per_type_I_fiber	region1inRegion2	5951
CNVR48	519	3	61793403	61809935	loss	2803	14776389	62780348	Number_of_capillaries_per_type_I_fiber	region1inRegion2	16532
CNVR46	504	3	45023435	45241189	gain	5692	14776389	62780348	Drip_loss	region1inRegion2	217754
CNVR47	510	3	56589884	56595835	loss	5692	14776389	62780348	Drip_loss	region1inRegion2	5951
CNVR48	519	3	61793403	61809935	loss	5692	14776389	62780348	Drip_loss	region1inRegion2	16532
CNVR46	504	3	45023435	45241189	gain	5694	14776389	62780348	Body_weight_(birth)	region1inRegion2	217754
CNVR47	510	3	56589884	56595835	loss	5694	14776389	62780348	Body_weight_(birth)	region1inRegion2	5951
CNVR48	519	3	61793403	61809935	loss	5694	14776389	62780348	Body_weight_(birth)	region1inRegion2	16532
CNVR46	504	3	45023435	45241189	gain	5938	14776389	62780348	Body_weight_(birth)	region1inRegion2	217754
CNVR47	510	3	56589884	56595835	loss	5938	14776389	62780348	Body_weight_(birth)	region1inRegion2	5951
CNVR48	519	3	61793403	61809935	loss	5938	14776389	62780348	Body_weight_(birth)	region1inRegion2	16532
CNVR46	504	3	45023435	45241189	gain	9642	14776389	62780348	Haptoglobin_concentration	region1inRegion2	217754
CNVR47	510	3	56589884	56595835	loss	9642	14776389	62780348	Haptoglobin_concentration	region1inRegion2	5951
CNVR48	519	3	61793403	61809935	loss	9642	14776389	62780348	Haptoglobin_concentration	region1inRegion2	16532
CNVR46	504	3	45023435	45241189	gain	7472	14776389	100216075	Nonfunctional_nipples	region1inRegion2	217754
CNVR47	510	3	56589884	56595835	loss	7472	14776389	100216075	Nonfunctional_nipples	region1inRegion2	5951
CNVR48	519	3	61793403	61809935	loss	7472	14776389	100216075	Nonfunctional_nipples	region1inRegion2	16532
CNVR49	524	3	67785626	67805349	gain	7472	14776389	100216075	Nonfunctional_nipples	region1inRegion2	19723
CNVR50	539	3	83892565	83943514	gain	7472	14776389	100216075	Nonfunctional_nipples	region1inRegion2	50949
CNVR46	504	3	45023435	45241189	gain	7455	14776389	102773204	Nonfunctional_nipples	region1inRegion2	217754
CNVR47	510	3	56589884	56595835	loss	7455	14776389	102773204	Nonfunctional_nipples	region1inRegion2	5951
CNVR48	519	3	61793403	61809935	loss	7455	14776389	102773204	Nonfunctional_nipples	region1inRegion2	16532
CNVR49	524	3	67785626	67805349	gain	7455	14776389	102773204	Nonfunctional_nipples	region1inRegion2	19723
CNVR50	539	3	83892565	83943514	gain	7455	14776389	102773204	Nonfunctional_nipples	region1inRegion2	50949
CNVR46	504	3	45023435	45241189	gain	5693	14776389	127042542	Side_fat_thickness	region1inRegion2	217754
CNVR47	510	3	56589884	56595835	loss	5693	14776389	127042542	Side_fat_thickness	region1inRegion2	5951
CNVR48	519	3	61793403	61809935	loss	5693	14776389	127042542	Side_fat_thickness	region1inRegion2	16532
CNVR49	524	3	67785626	67805349	gain	5693	14776389	127042542	Side_fat_thickness	region1inRegion2	19723
CNVR50	539	3	83892565	83943514	gain	5693	14776389	127042542	Side_fat_thickness	region1inRegion2	50949
CNVR46	504	3	45023435	45241189	gain	3227	15370065	131728891	pH_45_minutes_post_mortem	region1inRegion2	217754
CNVR47	510	3	56589884	56595835	loss	3227	15370065	131728891	pH_45_minutes_post_mortem	region1inRegion2	5951
CNVR48	519	3	61793403	61809935	loss	3227	15370065	131728891	pH_45_minutes_post_mortem	region1inRegion2	16532
CNVR49	524	3	67785626	67805349	gain	3227	15370065	131728891	pH_45_minutes_post_mortem	region1inRegion2	19723
CNVR50	539	3	83892565	83943514	gain	3227	15370065	131728891	pH_45_minutes_post_mortem	region1inRegion2	50949
CNVR46	504	3	45023435	45241189	gain	6465	17795067	104530182	Left_teat_number	region1inRegion2	217754
CNVR47	510	3	56589884	56595835	loss	6465	17795067	104530182	Left_teat_number	region1inRegion2	5951

CNVR48	519	3	61793403	61809935	loss	6465	17795067	104530182	Left_teat_number	region1inRegion2	16532
CNVR49	524	3	67785626	67805349	gain	6465	17795067	104530182	Left_teat_number	region1inRegion2	19723
CNVR50	539	3	83892565	83943514	gain	6465	17795067	104530182	Left_teat_number	region1inRegion2	50949
CNVR46	504	3	45023435	45241189	gain	172	20575481	76010586	body_weight_(17_weeks)	region1inRegion2	217754
CNVR47	510	3	56589884	56595835	loss	172	20575481	76010586	body_weight_(17_weeks)	region1inRegion2	5951
CNVR48	519	3	61793403	61809935	loss	172	20575481	76010586	body_weight_(17_weeks)	region1inRegion2	16532
CNVR49	524	3	67785626	67805349	gain	172	20575481	76010586	body_weight_(17_weeks)	region1inRegion2	19723
CNVR46	504	3	45023435	45241189	gain	21327	21824455	57222361	Cortisol_level	region1inRegion2	217754
CNVR47	510	3	56589884	56595835	loss	21327	21824455	57222361	Cortisol_level	region1inRegion2	5951
CNVR46	504	3	45023435	45241189	gain	21332	21824455	57222361	White_blood_cell_counts	region1inRegion2	217754
CNVR47	510	3	56589884	56595835	loss	21332	21824455	57222361	White_blood_cell_counts	region1inRegion2	5951
CNVR46	504	3	45023435	45241189	gain	3989	21824455	57222361	Total_shear_work	region1inRegion2	217754
CNVR47	510	3	56589884	56595835	loss	3989	21824455	57222361	Total_shear_work	region1inRegion2	5951
CNVR46	504	3	45023435	45241189	gain	3946	21824455	115140252	Carcass_length	region1inRegion2	217754
CNVR47	510	3	56589884	56595835	loss	3946	21824455	115140252	Carcass_length	region1inRegion2	5951
CNVR48	519	3	61793403	61809935	loss	3946	21824455	115140252	Carcass_length	region1inRegion2	16532
CNVR49	524	3	67785626	67805349	gain	3946	21824455	115140252	Carcass_length	region1inRegion2	19723
CNVR50	539	3	83892565	83943514	gain	3946	21824455	115140252	Carcass_length	region1inRegion2	50949
CNVR46	504	3	45023435	45241189	gain	3815	21824455	122295139	Off-Flavor_Score	region1inRegion2	217754
CNVR47	510	3	56589884	56595835	loss	3815	21824455	122295139	Off-Flavor_Score	region1inRegion2	5951
CNVR48	519	3	61793403	61809935	loss	3815	21824455	122295139	Off-Flavor_Score	region1inRegion2	16532
CNVR49	524	3	67785626	67805349	gain	3815	21824455	122295139	Off-Flavor_Score	region1inRegion2	19723
CNVR50	539	3	83892565	83943514	gain	3815	21824455	122295139	Off-Flavor_Score	region1inRegion2	50949
CNVR46	504	3	45023435	45241189	gain	21357	21947993	46839405	Ham_weight	region1inRegion2	217754
CNVR46	504	3	45023435	45241189	gain	6333	21947993	46839405	Bicarbonate_level	region1inRegion2	217754
CNVR46	504	3	45023435	45241189	gain	6336	21947993	46839405	Carbon_dioxide_level	region1inRegion2	217754
CNVR46	504	3	45023435	45241189	gain	7490	21947993	46839405	Creatine_kinase_level	region1inRegion2	217754
CNVR46	504	3	45023435	45241189	gain	340	21947993	57222361	Average_daily_gain	region1inRegion2	217754
CNVR47	510	3	56589884	56595835	loss	340	21947993	57222361	Average_daily_gain	region1inRegion2	5951
CNVR46	504	3	45023435	45241189	gain	874	21947993	57222361	Loin_and_neck_meat_weight	region1inRegion2	217754
CNVR47	510	3	56589884	56595835	loss	874	21947993	57222361	Loin_and_neck_meat_weight	region1inRegion2	5951
CNVR46	504	3	45023435	45241189	gain	876	21947993	57222361	Loin_muscle_area	region1inRegion2	217754
CNVR47	510	3	56589884	56595835	loss	876	21947993	57222361	Loin_muscle_area	region1inRegion2	5951
CNVR46	504	3	45023435	45241189	gain	896	21947993	57222361	pH_24_hr_post-mortem_(loin)	region1inRegion2	217754
CNVR47	510	3	56589884	56595835	loss	896	21947993	57222361	pH_24_hr_post-mortem_(loin)	region1inRegion2	5951
CNVR46	504	3	45023435	45241189	gain	3097	21947993	76622784	Loin_and_neck_meat_weight	region1inRegion2	217754
CNVR47	510	3	56589884	56595835	loss	3097	21947993	76622784	Loin_and_neck_meat_weight	region1inRegion2	5951
CNVR48	519	3	61793403	61809935	loss	3097	21947993	76622784	Loin_and_neck_meat_weight	region1inRegion2	16532
CNVR49	524	3	67785626	67805349	gain	3097	21947993	76622784	Loin_and_neck_meat_weight	region1inRegion2	19723
CNVR46	504	3	45023435	45241189	gain	3098	21947993	76622784	Shoulder_meat_weight	region1inRegion2	217754
CNVR47	510	3	56589884	56595835	loss	3098	21947993	76622784	Shoulder_meat_weight	region1inRegion2	5951
CNVR48	519	3	61793403	61809935	loss	3098	21947993	76622784	Shoulder_meat_weight	region1inRegion2	16532
CNVR49	524	3	67785626	67805349	gain	3098	21947993	76622784	Shoulder_meat_weight	region1inRegion2	19723
CNVR46	504	3	45023435	45241189	gain	3099	21947993	76622784	Average_daily_gain	region1inRegion2	217754
CNVR47	510	3	56589884	56595835	loss	3099	21947993	76622784	Average_daily_gain	region1inRegion2	5951
CNVR48	519	3	61793403	61809935	loss	3099	21947993	76622784	Average_daily_gain	region1inRegion2	16532
CNVR49	524	3	67785626	67805349	gain	3099	21947993	76622784	Average_daily_gain	region1inRegion2	19723
CNVR46	504	3	45023435	45241189	gain	3100	21947993	76622784	Carcass_length	region1inRegion2	217754
CNVR47	510	3	56589884	56595835	loss	3100	21947993	76622784	Carcass_length	region1inRegion2	5951
CNVR48	519	3	61793403	61809935	loss	3100	21947993	76622784	Carcass_length	region1inRegion2	16532
CNVR49	524	3	67785626	67805349	gain	3100	21947993	76622784	Carcass_length	region1inRegion2	19723
CNVR46	504	3	45023435	45241189	gain	3101	21947993	76622784	Loin_muscle_area	region1inRegion2	217754
CNVR47	510	3	56589884	56595835	loss	3101	21947993	76622784	Loin_muscle_area	region1inRegion2	5951
CNVR48	519	3	61793403	61809935	loss	3101	21947993	76622784	Loin_muscle_area	region1inRegion2	16532
CNVR49	524	3	67785626	67805349	gain	3101	21947993	76622784	Loin_muscle_area	region1inRegion2	19723
CNVR46	504	3	45023435	45241189	gain	3102	21947993	76622784	Ham_meat_weight	region1inRegion2	217754
CNVR47	510	3	56589884	56595835	loss	3102	21947993	76622784	Ham_meat_weight	region1inRegion2	5951
CNVR48	519	3	61793403	61809935	loss	3102	21947993	76622784	Ham_meat_weight	region1inRegion2	16532
CNVR49	524	3	67785626	67805349	gain	3102	21947993	76622784	Ham_meat_weight	region1inRegion2	19723
CNVR46	504	3	45023435	45241189	gain	3103	21947993	76622784	Carcass_weight_(cold)	region1inRegion2	217754
CNVR47	510	3	56589884	56595835	loss	3103	21947993	76622784	Carcass_weight_(cold)	region1inRegion2	5951
CNVR48	519	3	61793403	61809935	loss	3103	21947993	76622784	Carcass_weight_(cold)	region1inRegion2	16532
CNVR49	524	3	67785626	67805349	gain	3103	21947993	76622784	Carcass_weight_(cold)	region1inRegion2	19723
CNVR46	504	3	45023435	45241189	gain	3104	21947993	76622784	Ham_weight	region1inRegion2	217754
CNVR47	510	3	56589884	56595835	loss	3104	21947993	76622784	Ham_weight	region1inRegion2	5951
CNVR48	519	3	61793403	61809935	loss	3104	21947993	76622784	Ham_weight	region1inRegion2	16532
CNVR49	524	3	67785626	67805349	gain	3104	21947993	76622784	Ham_weight	region1inRegion2	19723
CNVR46	504	3	45023435	45241189	gain	3105	21947993	76622784	Head_weight	region1inRegion2	217754
CNVR47	510	3	56589884	56595835	loss	3105	21947993	76622784	Head_weight	region1inRegion2	5951
CNVR48	519	3	61793403	61809935	loss	3105	21947993	76622784	Head_weight	region1inRegion2	16532
CNVR49	524	3	67785626	67805349	gain	3105	21947993	76622784	Head_weight	region1inRegion2	19723
CNVR46	504	3	45023435	45241189	gain	3106	21947993	76622784	Feed_conversion_ratio	region1inRegion2	217754
CNVR47	510	3	56589884	56595835	loss	3106	21947993	76622784	Feed_conversion_ratio	region1inRegion2	5951
CNVR48	519	3	61793403	61809935	loss	3106	21947993	76622784	Feed_conversion_ratio	region1inRegion2	16532
CNVR49	524	3	67785626	67805349	gain	3106	21947993	76622784	Feed_conversion_ratio	region1inRegion2	19723
CNVR46	504	3	45023435	45241189	gain	3108	21947993	76622784	Lean_cuts_percentage	region1inRegion2	217754
CNVR47	510	3	56589884	56595835	loss	3108	21947993	76622784	Lean_cuts_percentage	region1inRegion2	5951
CNVR48	519	3	61793403	61809935	loss	3108	21947993	76622784	Lean_cuts_percentage	region1inRegion2	16532
CNVR49	524	3	67785626	67805349	gain	3108	21947993	76622784	Lean_cuts_percentage	region1inRegion2	19723
CNVR46	504	3	45023435	45241189	gain	879	21947993	76622784	Average_daily_gain	region1inRegion2	217754
CNVR47	510	3	56589884	56595835	loss	879	21947993	76622784	Average_daily_gain	region1inRegion2	5951
CNVR48	519	3	61793403	61809935	loss	879	21947993	76622784	Average_daily_gain	region1inRegion2	16532
CNVR49	524	3	67785626	67805349	gain	879	21947993	76622784	Average_daily_gain	region1inRegion2	19723
CNVR46	504	3	45023435	45241189	gain	7037	21947993	115061496	Diameter_of_type_IIb_muscle_fibers	region1inRegion2	217754
CNVR47	510	3	56589884	56595835	loss	7037	21947993	115061496	Diameter_of_type_IIb_muscle_fibers	region1inRegion2	5951
CNVR48	519	3	61793403	61809935	loss	7037	21947993	115061496	Diameter_of_type_IIb_muscle_fibers	region1inRegion2	16532
CNVR49	524	3	67785626	67805349	gain	7037	21947993	115061496	Diameter_of_type_IIb_muscle_fibers	region1inRegion2	19723
CNVR50	539	3	83892565	83943514	gain	7037	21947993	115061496	Diameter_of_type_IIb_muscle_fibers	region1inRegion2	50949
CNVR46	504	3	45023435	45241189	gain	21220	25777024	100508792	Body_weight_(34_weeks)	region1inRegion2	217754
CNVR47	510	3	56589884	56595835	loss	21220	25777024	100508792	Body_weight_(34_weeks)	region1inRegion2	5951
CNVR48	519	3	61793403	61809935	loss	21220	25777024	100508792	Body_weight_(34_weeks)	region1inRegion2	16532
CNVR49	524	3	67785626	67805349	gain	21220	25777024	100508792	Body_weight_(34_weeks)	region1inRegion2	19723
CNVR50	539	3	83892565	83943514	gain	21220	25777024	100508792	Body_weight_(34_weeks)	region1inRegion2	50949
CNVR46	504	3	45023435	45241189	gain	4038	28851635	51918004	CIE-b*	region1inRegion2	217754
CNVR46	504	3	45023435	45241189	gain	29691	31261211	93197958	Carcass_weight_(hot)	region1inRegion2	217754
CNVR47	510	3	56589884	56595835	loss	29691	31261211	93197958	Carcass_weight_(hot)	region1inRegion2	5951
CNVR48	519	3	61793403	61809935	loss	29691	31261211	93197958	Carcass_weight_(hot)	region1inRegion2	16532
CNVR49	524	3	67785626	67805349	gain	29691	31261211	93197958	Carcass_weight_(hot)	region1inRegion2	19723
CNVR50	539	3	83892565	83943514	gain	29691	31261211	93197958	Carcass_weight_(hot)	region1inRegion2	50949
CNVR46	504	3	45023435	45241189	gain	596	31261211	115061532	Fat_androstenone_level	region1inRegion2	217754
CNVR47	510	3	56589884	56595835	loss	596	31261211	115061532	Fat_androstenone_level	region1inRegion2	5951
CNVR48	519	3	61793403	61809935	loss	596	31261211	115061532	Fat_androstenone_level	region1inRegion2	16532
CNVR49	524	3	67785626	67805349	gain	596	31261211	115061532	Fat_androstenone_level	region1inRegion2	19723
CNVR50	539	3	83892565	83943514	gain	596	31261211	115061532	Fat_androstenone_level	region1inRegion2	50949
CNVR46	504	3	45023435	45241189	gain	597	31261211	115061532	Fat_androstenone_level	region1inRegion2	217754
CNVR47	510	3	56589884	56595835	loss	597	31261211	115061532	Fat_androstenone_level	region1inRegion2	5951
CNVR48	519	3	61793403	61809935	loss	597	31261211	115061532	Fat_androstenone_level	region1inRegion2	16532
CNVR49	524	3	67785626	67805349	gain	597	31261211	115061532	Fat_androstenone_level	region1inRegion2	19723
CNVR50	539	3	83892565	83943514	gain	597	31261211	115061532	Fat_androstenone_level	region1inRegion2	50949
CNVR46	504	3	45023435	45241189	gain	173	34143639	98066155	Average_daily_gain	region1inRegion2	217754
CNVR47	510	3	56589884	56595835	loss	173	34143639	98066155	Average_daily_gain	region1inRegion2	5951
CNVR48	519	3	61793403	61809935	loss	173	34143639	98066155	Average_daily_gain	region1inRegion2	16532
CNVR49	524	3	67785626	67805349	gain	173	34143639	98066155	Average_daily_gain	region1inRegion2	19723
CNVR50	539	3	83892565	83943514	gain	173	34143639	98066155	Average_daily_gain	region1inRegion2	50949
CNVR46	504	3	45023435	45241189	gain	12286	38238763	62780348	Mean_corpuscular_volume	region1inRegion2	217754
CNVR47	510	3	56589884	56595835	loss	12286	38238763	62780348	Mean_corpuscular_volume	region1inRegion2	5951
CNVR48	519	3	61793403	61809935	loss	12286	38238763	62780348	Mean_corpuscular_volume	region1inRegion2	16532
CNVR46	504	3	45023435	45241189	gain	16862	38238763	76622784	Carcass_weight_(hot)	region1inRegion2	217754
CNVR47	510	3	56589884	56595835	loss	16862	38238763	76622784	Carcass_weight_(hot)	region1inRegion2	5951
CNVR48	519	3	61793403	61809935	loss	16862	38238763	76622784	Carcass_weight_(hot)	region1inRegion2	16532
CNVR49	524	3	67785626	67805349	gain	16862	38238763	76622784	Carcass_weight_(hot)	region1inRegion2	19723
CNVR46	504	3	45023435	45241189	gain	22285	45069392	46343661	Average_backfat_thickness	overlapTail	171798

CNVR47	510	3	56589884	56595835	loss	16847	46610085	76622784	Average_daily_gain	region1inRegion2	5951
CNVR48	519	3	61793403	61809935	loss	16847	46610085	76622784	Average_daily_gain	region1inRegion2	16532
CNVR49	524	3	67785626	67805349	gain	16847	46610085	76622784	Average_daily_gain	region1inRegion2	19723
CNVR47	510	3	56589884	56595835	loss	16884	46610085	76622784	Average_daily_gain	region1inRegion2	5951
CNVR48	519	3	61793403	61809935	loss	16884	46610085	76622784	Average_daily_gain	region1inRegion2	16532
CNVR49	524	3	67785626	67805349	gain	16884	46610085	76622784	Average_daily_gain	region1inRegion2	19723
CNVR47	510	3	56589884	56595835	loss	6320	46839405	100216075	Blood_pH	region1inRegion2	5951
CNVR48	519	3	61793403	61809935	loss	6320	46839405	100216075	Blood_pH	region1inRegion2	16532
CNVR49	524	3	67785626	67805349	gain	6320	46839405	100216075	Blood_pH	region1inRegion2	19723
CNVR50	539	3	83892565	83943514	gain	6320	46839405	100216075	Blood_pH	region1inRegion2	50949
CNVR47	510	3	56589884	56595835	loss	6342	46839405	100216075	Base_excess	region1inRegion2	5951
CNVR48	519	3	61793403	61809935	loss	6342	46839405	100216075	Base_excess	region1inRegion2	16532
CNVR49	524	3	67785626	67805349	gain	6342	46839405	100216075	Base_excess	region1inRegion2	19723
CNVR50	539	3	83892565	83943514	gain	6342	46839405	100216075	Base_excess	region1inRegion2	50949
CNVR47	510	3	56589884	56595835	loss	6361	46839405	100216075	Lactate_level	region1inRegion2	5951
CNVR48	519	3	61793403	61809935	loss	6361	46839405	100216075	Lactate_level	region1inRegion2	16532
CNVR49	524	3	67785626	67805349	gain	6361	46839405	100216075	Lactate_level	region1inRegion2	19723
CNVR50	539	3	83892565	83943514	gain	6361	46839405	100216075	Lactate_level	region1inRegion2	50949
CNVR47	510	3	56589884	56595835	loss	11852	56047063	101551719	Feet_and_leg_conformation	region1inRegion2	5951
CNVR48	519	3	61793403	61809935	loss	11852	56047063	101551719	Feet_and_leg_conformation	region1inRegion2	16532
CNVR49	524	3	67785626	67805349	gain	11852	56047063	101551719	Feet_and_leg_conformation	region1inRegion2	19723
CNVR50	539	3	83892565	83943514	gain	11852	56047063	101551719	Feet_and_leg_conformation	region1inRegion2	50949
CNVR47	510	3	56589884	56595835	loss	126672	56530168	57370124	Teat_number	region1inRegion2	5951
CNVR48	519	3	61793403	61809935	loss	892	57222361	76622784	Liver_weight	region1inRegion2	16532
CNVR49	524	3	67785626	67805349	gain	892	57222361	76622784	Liver_weight	region1inRegion2	19723
CNVR48	519	3	61793403	61809935	loss	8946	57222361	115140252	Physis_score	region1inRegion2	16532
CNVR49	524	3	67785626	67805349	gain	8946	57222361	115140252	Physis_score	region1inRegion2	19723
CNVR50	539	3	83892565	83943514	gain	8946	57222361	115140252	Physis_score	region1inRegion2	50949
CNVR48	519	3	61793403	61809935	loss	895	57222361	119745261	pH_45_minutes_post_mortem	region1inRegion2	16532
CNVR49	524	3	67785626	67805349	gain	895	57222361	119745261	pH_45_minutes_post_mortem	region1inRegion2	19723
CNVR50	539	3	83892565	83943514	gain	895	57222361	119745261	pH_45_minutes_post_mortem	region1inRegion2	50949
CNVR48	519	3	61793403	61809935	loss	21590	61123471	61798608	45_min-24_h_pH_decline	overlapHead	5206
CNVR49	524	3	67785626	67805349	gain	12287	62780348	100216075	Mean_corpuscular_hemoglobin_concentration	region1inRegion2	19723
CNVR50	539	3	83892565	83943514	gain	12287	62780348	100216075	Mean_corpuscular_hemoglobin_concentration	region1inRegion2	50949
CNVR49	524	3	67785626	67805349	gain	17603	62780348	100216075	Interleukin_10_level	region1inRegion2	19723
CNVR50	539	3	83892565	83943514	gain	17603	62780348	100216075	Interleukin_10_level	region1inRegion2	50949
CNVR49	524	3	67785626	67805349	gain	17611	62780348	100216075	Interferon-gamma_level	region1inRegion2	19723
CNVR50	539	3	83892565	83943514	gain	17611	62780348	100216075	Interferon-gamma_level	region1inRegion2	50949
CNVR49	524	3	67785626	67805349	gain	9616	62780348	100216075	C3c_concentration	region1inRegion2	19723
CNVR50	539	3	83892565	83943514	gain	9616	62780348	100216075	C3c_concentration	region1inRegion2	50949
CNVR49	524	3	67785626	67805349	gain	15067	62780348	118796463	Triglyceride_level	region1inRegion2	19723
CNVR50	539	3	83892565	83943514	gain	15067	62780348	118796463	Triglyceride_level	region1inRegion2	50949
CNVR49	524	3	67785626	67805349	gain	126598	67608274	68788489	Left_teat_number	region1inRegion2	19723
CNVR50	539	3	83892565	83943514	gain	880	76622784	119745261	Fat_percentage_in_carcass	region1inRegion2	50949
CNVR50	539	3	83892565	83943514	gain	881	76622784	119745261	Backfat_thickness_between_3rd_and_4th_rib	region1inRegion2	50949
CNVR50	539	3	83892565	83943514	gain	882	76622784	119745261	Dressing_percentage	region1inRegion2	50949
CNVR50	539	3	83892565	83943514	gain	883	76622784	119745261	Dressing_percentage	region1inRegion2	50949
CNVR50	539	3	83892565	83943514	gain	884	76622784	119745261	Average_backfat_thickness	region1inRegion2	50949
CNVR50	539	3	83892565	83943514	gain	885	76622784	119745261	Backfat_weight	region1inRegion2	50949
CNVR50	539	3	83892565	83943514	gain	886	76622784	119745261	Lean_meat_percentage	region1inRegion2	50949
CNVR50	539	3	83892565	83943514	gain	887	76622784	119745261	Shoulder_external_fat_weight	region1inRegion2	50949
CNVR50	539	3	83892565	83943514	gain	889	76622784	119745261	External_fat_on_ham	region1inRegion2	50949
CNVR50	539	3	83892565	83943514	gain	894	76622784	119745261	Body_weight_(3_weeks)	region1inRegion2	50949
CNVR50	539	3	83892565	83943514	gain	18011	76622784	143758669	Fat_protein_content	region1inRegion2	50949
CNVR51	581	3	142899740	142925257	loss	18011	76622784	143758669	Fat_protein_content	region1inRegion2	25517
CNVR52	582	3	142999513	143009221	gain	18011	76622784	143758669	Fat_protein_content	region1inRegion2	9708
CNVR53	585	3	143590087	143597682	gain	18011	76622784	143758669	Fat_protein_content	region1inRegion2	7595
CNVR50	539	3	83892565	83943514	gain	18044	76884637	85433008	Corpus_luteum_number	region1inRegion2	50949
CNVR50	539	3	83892565	83943514	gain	3944	76884637	93197958	Semimembranosus_angle	region1inRegion2	50949
CNVR50	539	3	83892565	83943514	gain	3986	76884637	93197958	CIE-L*	region1inRegion2	50949
CNVR50	539	3	83892565	83943514	gain	3987	76884637	93197958	Shear_force	region1inRegion2	50949
CNVR50	539	3	83892565	83943514	gain	3988	76884637	93197958	Shear_force_at_first_peak	region1inRegion2	50949
CNVR50	539	3	83892565	83943514	gain	3935	76884637	143758669	Ham_fat_thickness	region1inRegion2	50949
CNVR51	581	3	142899740	142925257	loss	3935	76884637	143758669	Ham_fat_thickness	region1inRegion2	25517
CNVR52	582	3	142999513	143009221	gain	3935	76884637	143758669	Ham_fat_thickness	region1inRegion2	9708
CNVR53	585	3	143590087	143597682	gain	3935	76884637	143758669	Ham_fat_thickness	region1inRegion2	7595
CNVR51	581	3	142899740	142925257	loss	6343	100216075	143758669	Base_excess	region1inRegion2	25517
CNVR52	582	3	142999513	143009221	gain	6343	100216075	143758669	Base_excess	region1inRegion2	9708
CNVR53	585	3	143590087	143597682	gain	6343	100216075	143758669	Base_excess	region1inRegion2	7595
CNVR51	581	3	142899740	142925257	loss	6367	100216075	143758669	Creatinine_level	region1inRegion2	25517
CNVR52	582	3	142999513	143009221	gain	6367	100216075	143758669	Creatinine_level	region1inRegion2	9708
CNVR53	585	3	143590087	143597682	gain	6367	100216075	143758669	Creatinine_level	region1inRegion2	7595
CNVR51	581	3	142899740	142925257	loss	6387	100216075	143758669	Sodium_level	region1inRegion2	25517
CNVR52	582	3	142999513	143009221	gain	6387	100216075	143758669	Sodium_level	region1inRegion2	9708
CNVR53	585	3	143590087	143597682	gain	6387	100216075	143758669	Sodium_level	region1inRegion2	7595
CNVR51	581	3	142899740	142925257	loss	21315	119745261	143758669	rhinitis	region1inRegion2	25517
CNVR52	582	3	142999513	143009221	gain	21315	119745261	143758669	rhinitis	region1inRegion2	9708
CNVR53	585	3	143590087	143597682	gain	21315	119745261	143758669	rhinitis	region1inRegion2	7595
CNVR51	581	3	142899740	142925257	loss	4250	125343844	143758669	Teat_number	region1inRegion2	25517
CNVR52	582	3	142999513	143009221	gain	4250	125343844	143758669	Teat_number	region1inRegion2	9708
CNVR53	585	3	143590087	143597682	gain	4250	125343844	143758669	Teat_number	region1inRegion2	7595
CNVR51	581	3	142899740	142925257	loss	4256	125343844	143758669	Nonfunctional_nipples	region1inRegion2	25517
CNVR52	582	3	142999513	143009221	gain	4256	125343844	143758669	Nonfunctional_nipples	region1inRegion2	9708
CNVR53	585	3	143590087	143597682	gain	4256	125343844	143758669	Nonfunctional_nipples	region1inRegion2	7595
CNVR55	639	4	52447075	52460832	gain	12481	131971	62356198	backfat_at_last_rib	region1inRegion2	13757
CNVR56	649	4	55682568	55685476	loss	12481	131971	62356198	backfat_at_last_rib	region1inRegion2	2908
CNVR55	639	4	52447075	52460832	gain	3093	131971	75369864	Average_backfat_thickness	region1inRegion2	13757
CNVR56	649	4	55682568	55685476	loss	3093	131971	75369864	Average_backfat_thickness	region1inRegion2	2908
CNVR57	668	4	67469665	67474606	loss	3093	131971	75369864	Average_backfat_thickness	region1inRegion2	4941
CNVR55	639	4	52447075	52460832	gain	12492	131971	80511752	Heart_weight	region1inRegion2	13757
CNVR56	649	4	55682568	55685476	loss	12492	131971	80511752	Heart_weight	region1inRegion2	2908
CNVR57	668	4	67469665	67474606	loss	12492	131971	80511752	Heart_weight	region1inRegion2	4941
CNVR55	639	4	52447075	52460832	gain	3757	131971	134902449	Average_daily_gain	region1inRegion2	13757
CNVR56	649	4	55682568	55685476	loss	3757	131971	134902449	Average_daily_gain	region1inRegion2	2908
CNVR57	668	4	67469665	67474606	loss	3757	131971	134902449	Average_daily_gain	region1inRegion2	4941
CNVR58	683	4	88694499	88704809	gain	3757	131971	134902449	Average_daily_gain	region1inRegion2	10310
CNVR59	698	4	111405421	111446020	gain	3757	131971	134902449	Average_daily_gain	region1inRegion2	40599
CNVR55	639	4	52447075	52460832	gain	3758	131971	134902449	Average_daily_gain	region1inRegion2	13757
CNVR56	649	4	55682568	55685476	loss	3758	131971	134902449	Average_daily_gain	region1inRegion2	2908
CNVR57	668	4	67469665	67474606	loss	3758	131971	134902449	Average_daily_gain	region1inRegion2	4941
CNVR58	683	4	88694499	88704809	gain	3758	131971	134902449	Average_daily_gain	region1inRegion2	10310
CNVR59	698	4	111405421	111446020	gain	3758	131971	134902449	Average_daily_gain	region1inRegion2	40599
CNVR55	639	4	52447075	52460832	gain	3759	131971	134902449	Average_backfat_thickness	region1inRegion2	13757
CNVR56	649	4	55682568	55685476	loss	3759	131971	134902449	Average_backfat_thickness	region1inRegion2	2908
CNVR57	668	4	67469665	67474606	loss	3759	131971	134902449	Average_backfat_thickness	region1inRegion2	4941
CNVR58	683	4	88694499	88704809	gain	3759	131971	134902449	Average_backfat_thickness	region1inRegion2	10310
CNVR59	698	4	111405421	111446020	gain	3759	131971	134902449	Average_backfat_thickness	region1inRegion2	40599
CNVR55	639	4	52447075	52460832	gain	3771	131971	134902449	Average_daily_gain	region1inRegion2	13757
CNVR56	649	4	55682568	55685476	loss	3771	131971	134902449	Average_daily_gain	region1inRegion2	2908
CNVR57	668	4	67469665	67474606	loss	3771	131971	134902449	Average_daily_gain	region1inRegion2	4941
CNVR58	683	4	88694499	88704809	gain	3771	131971	134902449	Average_daily_gain	region1inRegion2	10310
CNVR59	698	4	111405421	111446020	gain	3771	131971	134902449	Average_daily_gain	region1inRegion2	40599
CNVR55	639	4	52447075	52460832	gain	161	4064787	52626380	Taste_intensity	region1inRegion2	13757
CNVR55	639	4	52447075	52460832	gain	5225	4064787	71152561	Teat_number	region1inRegion2	13757
CNVR56	649	4	55682568	55685476	loss	5225	4064787	71152561	Teat_number	region1inRegion2	2908
CNVR57	668	4	67469665	67474606	loss	5225	4064787	71152561	Teat_number	region1inRegion2	4941
CNVR55	639	4	52447075	52460832	gain	313	4064787	82084284	Intramuscular_fat_content	region1inRegion2	13757
CNVR56	649	4	55682568	55685476	loss	313	4064787	82084284	Intramuscular_fat_content	region1inRegion2	2908
CNVR57	668	4	67469665	67474606	loss	313	4064787	82084284	Intramuscular_fat_content	region1inRegion2	4941
CNVR55	639	4	52447075	52460832	gain	140341	4314853	124326847	PRRSV_susceptibility	region1inRegion2	13757
CNVR56	649	4	55682568	55685476	loss	140341	4314853	124326847	PRRSV_susceptibility	region1inRegion2	2908
CNVR57	668	4	67469665	67474606	loss	140341	4314853	124326847	PRRSV_susceptibility	region1inRegion2	4941

CNVR58	683	4	88694499	88704809	gain	140341	4314853	124326847	PRRSV_susceptibility	region1inRegion2	10310
CNVR59	698	4	111405421	111446020	gain	140341	4314853	124326847	PRRSV_susceptibility	region1inRegion2	40599
CNVR55	639	4	52447075	52460832	gain	29556	4314853	124326847	Feed_conversion_ratio	region1inRegion2	13757
CNVR56	649	4	55682568	55685476	loss	29556	4314853	124326847	Feed_conversion_ratio	region1inRegion2	2908
CNVR57	668	4	67469665	67474606	loss	29556	4314853	124326847	Feed_conversion_ratio	region1inRegion2	4941
CNVR58	683	4	88694499	88704809	gain	29556	4314853	124326847	Feed_conversion_ratio	region1inRegion2	10310
CNVR59	698	4	111405421	111446020	gain	29556	4314853	124326847	Feed_conversion_ratio	region1inRegion2	40599
CNVR55	639	4	52447075	52460832	gain	5256	5244479	95136118	Teat_number	region1inRegion2	13757
CNVR56	649	4	55682568	55685476	loss	5256	5244479	95136118	Teat_number	region1inRegion2	2908
CNVR57	668	4	67469665	67474606	loss	5256	5244479	95136118	Teat_number	region1inRegion2	4941
CNVR58	683	4	88694499	88704809	gain	5256	5244479	95136118	Teat_number	region1inRegion2	10310
CNVR55	639	4	52447075	52460832	gain	38084	7237639	134902449	Drip_loss	region1inRegion2	13757
CNVR56	649	4	55682568	55685476	loss	38084	7237639	134902449	Drip_loss	region1inRegion2	2908
CNVR57	668	4	67469665	67474606	loss	38084	7237639	134902449	Drip_loss	region1inRegion2	4941
CNVR58	683	4	88694499	88704809	gain	38084	7237639	134902449	Drip_loss	region1inRegion2	10310
CNVR59	698	4	111405421	111446020	gain	38084	7237639	134902449	Drip_loss	region1inRegion2	40599
CNVR55	639	4	52447075	52460832	gain	21233	7281654	95345742	intestinal_fat_weight	region1inRegion2	13757
CNVR56	649	4	55682568	55685476	loss	21233	7281654	95345742	intestinal_fat_weight	region1inRegion2	2908
CNVR57	668	4	67469665	67474606	loss	21233	7281654	95345742	intestinal_fat_weight	region1inRegion2	4941
CNVR58	683	4	88694499	88704809	gain	21233	7281654	95345742	intestinal_fat_weight	region1inRegion2	10310
CNVR55	639	4	52447075	52460832	gain	253	8745760	129031532	Small_intestine_length	region1inRegion2	13757
CNVR56	649	4	55682568	55685476	loss	253	8745760	129031532	Small_intestine_length	region1inRegion2	2908
CNVR57	668	4	67469665	67474606	loss	253	8745760	129031532	Small_intestine_length	region1inRegion2	4941
CNVR58	683	4	88694499	88704809	gain	253	8745760	129031532	Small_intestine_length	region1inRegion2	10310
CNVR59	698	4	111405421	111446020	gain	253	8745760	129031532	Small_intestine_length	region1inRegion2	40599
CNVR55	639	4	52447075	52460832	gain	17544	11844192	85834188	Head_weight	region1inRegion2	13757
CNVR56	649	4	55682568	55685476	loss	17544	11844192	85834188	Head_weight	region1inRegion2	2908
CNVR57	668	4	67469665	67474606	loss	17544	11844192	85834188	Head_weight	region1inRegion2	4941
CNVR55	639	4	52447075	52460832	gain	159	12618993	71310074	Subacid_smell	region1inRegion2	13757
CNVR56	649	4	55682568	55685476	loss	159	12618993	71310074	Subacid_smell	region1inRegion2	2908
CNVR57	668	4	67469665	67474606	loss	159	12618993	71310074	Subacid_smell	region1inRegion2	4941
CNVR55	639	4	52447075	52460832	gain	442	12618993	77094703	Average_daily_gain	region1inRegion2	13757
CNVR56	649	4	55682568	55685476	loss	442	12618993	77094703	Average_daily_gain	region1inRegion2	2908
CNVR57	668	4	67469665	67474606	loss	442	12618993	77094703	Average_daily_gain	region1inRegion2	4941
CNVR55	639	4	52447075	52460832	gain	443	12618993	102054630	Average_daily_gain	region1inRegion2	13757
CNVR56	649	4	55682568	55685476	loss	443	12618993	102054630	Average_daily_gain	region1inRegion2	2908
CNVR57	668	4	67469665	67474606	loss	443	12618993	102054630	Average_daily_gain	region1inRegion2	4941
CNVR58	683	4	88694499	88704809	gain	443	12618993	102054630	Average_daily_gain	region1inRegion2	10310
CNVR55	639	4	52447075	52460832	gain	16894	12618993	110539481	Carcass_weight_(hot)	region1inRegion2	13757
CNVR56	649	4	55682568	55685476	loss	16894	12618993	110539481	Carcass_weight_(hot)	region1inRegion2	2908
CNVR57	668	4	67469665	67474606	loss	16894	12618993	110539481	Carcass_weight_(hot)	region1inRegion2	4941
CNVR58	683	4	88694499	88704809	gain	16894	12618993	110539481	Carcass_weight_(hot)	region1inRegion2	10310
CNVR55	639	4	52447075	52460832	gain	370	12618993	123430356	Average_daily_gain	region1inRegion2	13757
CNVR56	649	4	55682568	55685476	loss	370	12618993	123430356	Average_daily_gain	region1inRegion2	2908
CNVR57	668	4	67469665	67474606	loss	370	12618993	123430356	Average_daily_gain	region1inRegion2	4941
CNVR58	683	4	88694499	88704809	gain	370	12618993	123430356	Average_daily_gain	region1inRegion2	10310
CNVR59	698	4	111405421	111446020	gain	370	12618993	123430356	Average_daily_gain	region1inRegion2	40599
CNVR55	639	4	52447075	52460832	gain	38082	12618993	134902449	Marbling	region1inRegion2	13757
CNVR56	649	4	55682568	55685476	loss	38082	12618993	134902449	Marbling	region1inRegion2	2908
CNVR57	668	4	67469665	67474606	loss	38082	12618993	134902449	Marbling	region1inRegion2	4941
CNVR58	683	4	88694499	88704809	gain	38082	12618993	134902449	Marbling	region1inRegion2	10310
CNVR59	698	4	111405421	111446020	gain	38082	12618993	134902449	Marbling	region1inRegion2	40599
CNVR55	639	4	52447075	52460832	gain	5243	14152941	130171595	backfat_at_mid-back	region1inRegion2	13757
CNVR56	649	4	55682568	55685476	loss	5243	14152941	130171595	backfat_at_mid-back	region1inRegion2	2908
CNVR57	668	4	67469665	67474606	loss	5243	14152941	130171595	backfat_at_mid-back	region1inRegion2	4941
CNVR58	683	4	88694499	88704809	gain	5243	14152941	130171595	backfat_at_mid-back	region1inRegion2	10310
CNVR59	698	4	111405421	111446020	gain	5243	14152941	130171595	backfat_at_mid-back	region1inRegion2	40599
CNVR55	639	4	52447075	52460832	gain	245	14152941	136991929	Average_daily_gain	region1inRegion2	13757
CNVR56	649	4	55682568	55685476	loss	245	14152941	136991929	Average_daily_gain	region1inRegion2	2908
CNVR57	668	4	67469665	67474606	loss	245	14152941	136991929	Average_daily_gain	region1inRegion2	4941
CNVR58	683	4	88694499	88704809	gain	245	14152941	136991929	Average_daily_gain	region1inRegion2	10310
CNVR59	698	4	111405421	111446020	gain	245	14152941	136991929	Average_daily_gain	region1inRegion2	40599
CNVR55	639	4	52447075	52460832	gain	4895	15219816	121807926	Ham_weight	region1inRegion2	13757
CNVR56	649	4	55682568	55685476	loss	4895	15219816	121807926	Ham_weight	region1inRegion2	2908
CNVR57	668	4	67469665	67474606	loss	4895	15219816	121807926	Ham_weight	region1inRegion2	4941
CNVR58	683	4	88694499	88704809	gain	4895	15219816	121807926	Ham_weight	region1inRegion2	10310
CNVR59	698	4	111405421	111446020	gain	4895	15219816	121807926	Ham_weight	region1inRegion2	40599
CNVR55	639	4	52447075	52460832	gain	5235	17203914	127117355	Body_weight_(end_of_test)	region1inRegion2	13757
CNVR56	649	4	55682568	55685476	loss	5235	17203914	127117355	Body_weight_(end_of_test)	region1inRegion2	2908
CNVR57	668	4	67469665	67474606	loss	5235	17203914	127117355	Body_weight_(end_of_test)	region1inRegion2	4941
CNVR58	683	4	88694499	88704809	gain	5235	17203914	127117355	Body_weight_(end_of_test)	region1inRegion2	10310
CNVR59	698	4	111405421	111446020	gain	5235	17203914	127117355	Body_weight_(end_of_test)	region1inRegion2	40599
CNVR55	639	4	52447075	52460832	gain	21227	17289621	135224471	Body_weight_(46_days)	region1inRegion2	13757
CNVR56	649	4	55682568	55685476	loss	21227	17289621	135224471	Body_weight_(46_days)	region1inRegion2	2908
CNVR57	668	4	67469665	67474606	loss	21227	17289621	135224471	Body_weight_(46_days)	region1inRegion2	4941
CNVR58	683	4	88694499	88704809	gain	21227	17289621	135224471	Body_weight_(46_days)	region1inRegion2	10310
CNVR59	698	4	111405421	111446020	gain	21227	17289621	135224471	Body_weight_(46_days)	region1inRegion2	40599
CNVR55	639	4	52447075	52460832	gain	3091	21473440	115001161	Average_daily_gain	region1inRegion2	13757
CNVR56	649	4	55682568	55685476	loss	3091	21473440	115001161	Average_daily_gain	region1inRegion2	2908
CNVR57	668	4	67469665	67474606	loss	3091	21473440	115001161	Average_daily_gain	region1inRegion2	4941
CNVR58	683	4	88694499	88704809	gain	3091	21473440	115001161	Average_daily_gain	region1inRegion2	10310
CNVR59	698	4	111405421	111446020	gain	3091	21473440	115001161	Average_daily_gain	region1inRegion2	40599
CNVR55	639	4	52447075	52460832	gain	21333	22799207	52626380	White_blood_cell_counts	region1inRegion2	13757
CNVR55	639	4	52447075	52460832	gain	270	23266114	93366673	Average_backfat_thickness	region1inRegion2	13757
CNVR56	649	4	55682568	55685476	loss	270	23266114	93366673	Average_backfat_thickness	region1inRegion2	2908
CNVR57	668	4	67469665	67474606	loss	270	23266114	93366673	Average_backfat_thickness	region1inRegion2	4941
CNVR58	683	4	88694499	88704809	gain	270	23266114	93366673	Average_backfat_thickness	region1inRegion2	10310
CNVR55	639	4	52447075	52460832	gain	8801	23809394	82084284	Body_weight	region1inRegion2	13757
CNVR56	649	4	55682568	55685476	loss	8801	23809394	82084284	Body_weight	region1inRegion2	2908
CNVR57	668	4	67469665	67474606	loss	8801	23809394	82084284	Body_weight	region1inRegion2	4941
CNVR55	639	4	52447075	52460832	gain	8802	23809394	82084284	Body_weight	region1inRegion2	13757
CNVR56	649	4	55682568	55685476	loss	8802	23809394	82084284	Body_weight	region1inRegion2	2908
CNVR57	668	4	67469665	67474606	loss	8802	23809394	82084284	Body_weight	region1inRegion2	4941
CNVR55	639	4	52447075	52460832	gain	8817	23809394	82084284	Corpus_luteum_number	region1inRegion2	13757
CNVR56	649	4	55682568	55685476	loss	8817	23809394	82084284	Corpus_luteum_number	region1inRegion2	2908
CNVR57	668	4	67469665	67474606	loss	8817	23809394	82084284	Corpus_luteum_number	region1inRegion2	4941
CNVR55	639	4	52447075	52460832	gain	8947	23809394	82084284	Physis_score	region1inRegion2	13757
CNVR56	649	4	55682568	55685476	loss	8947	23809394	82084284	Physis_score	region1inRegion2	2908
CNVR57	668	4	67469665	67474606	loss	8947	23809394	82084284	Physis_score	region1inRegion2	4941
CNVR55	639	4	52447075	52460832	gain	444	23809394	96936045	Average_daily_gain	region1inRegion2	13757
CNVR56	649	4	55682568	55685476	loss	444	23809394	96936045	Average_daily_gain	region1inRegion2	2908
CNVR57	668	4	67469665	67474606	loss	444	23809394	96936045	Average_daily_gain	region1inRegion2	4941
CNVR58	683	4	88694499	88704809	gain	444	23809394	96936045	Average_daily_gain	region1inRegion2	10310
CNVR55	639	4	52447075	52460832	gain	17717	23809394	102054630	HDL/LDL_ratio	region1inRegion2	13757
CNVR56	649	4	55682568	55685476	loss	17717	23809394	102054630	HDL/LDL_ratio	region1inRegion2	2908
CNVR57	668	4	67469665	67474606	loss	17717	23809394	102054630	HDL/LDL_ratio	region1inRegion2	4941
CNVR58	683	4	88694499	88704809	gain	17717	23809394	102054630	HDL/LDL_ratio	region1inRegion2	10310
CNVR55	639	4	52447075	52460832	gain	200	23809394	102054630	Average_backfat_thickness	region1inRegion2	13757
CNVR56	649	4	55682568	55685476	loss	200	23809394	102054630	Average_backfat_thickness	region1inRegion2	2908
CNVR57	668	4	67469665	67474606	loss	200	23809394	102054630	Average_backfat_thickness	region1inRegion2	4941
CNVR58	683	4	88694499	88704809	gain	200	23809394	102054630	Average_backfat_thickness	region1inRegion2	10310
CNVR55	639	4	52447075	52460832	gain	201	23809394	102054630	Average_backfat_thickness	region1inRegion2	13757
CNVR56	649	4	55682568	55685476	loss	201	23809394	102054630	Average_backfat_thickness	region1inRegion2	2908
CNVR57	668	4	67469665	67474606	loss	201	23809394	102054630	Average_backfat_thickness	region1inRegion2	4941
CNVR58	683	4	88694499	88704809	gain	201	23809394	102054630	Average_backfat_thickness	region1inRegion2	10310
CNVR55	639	4	52447075	52460832	gain	3032	23809394	102054630	Loin_muscle_area	region1inRegion2	13757
CNVR56	649	4	55682568	55685476	loss	3032	23809394	102054630	Loin_muscle_area	region1inRegion2	2908
CNVR57	668	4	67469665	67474606	loss	3032	23809394	102054630	Loin_muscle_area	region1inRegion2	4941
CNVR58	683	4	88694499	88704809	gain	3032	23809394	102054630	Loin_muscle_area	region1inRegion2	10310
CNVR55	639	4	52447075	52460832	gain	3033	23809394	102054630	Carcass_length	region1inRegion2	13757
CNVR56	649	4	55682568	55685476	loss	3033	23809394	102054630	Carcass_length	region1inRegion2	2908
CNVR57	668	4	67469665	67474606	loss	3033	23809394	102054630	Carcass_length	region1inRegion2	4941

CNVR58	683	4	88694499	88704809	gain	3033	23809394	102054630	Carcass_length	region1inRegion2	10310
CNVR55	639	4	52447075	52460832	gain	3034	23809394	102054630	Average_backfat_thickness	region1inRegion2	13757
CNVR56	649	4	55682568	55685476	loss	3034	23809394	102054630	Average_backfat_thickness	region1inRegion2	2908
CNVR57	668	4	67469665	67474606	loss	3034	23809394	102054630	Average_backfat_thickness	region1inRegion2	4941
CNVR58	683	4	88694499	88704809	gain	3034	23809394	102054630	Average_backfat_thickness	region1inRegion2	10310
CNVR55	639	4	52447075	52460832	gain	3035	23809394	102054630	Average_daily_gain	region1inRegion2	13757
CNVR56	649	4	55682568	55685476	loss	3035	23809394	102054630	Average_daily_gain	region1inRegion2	2908
CNVR57	668	4	67469665	67474606	loss	3035	23809394	102054630	Average_daily_gain	region1inRegion2	4941
CNVR58	683	4	88694499	88704809	gain	3035	23809394	102054630	Average_daily_gain	region1inRegion2	10310
CNVR55	639	4	52447075	52460832	gain	461	23809394	102054630	Loin_muscle_area	region1inRegion2	13757
CNVR56	649	4	55682568	55685476	loss	461	23809394	102054630	Loin_muscle_area	region1inRegion2	2908
CNVR57	668	4	67469665	67474606	loss	461	23809394	102054630	Loin_muscle_area	region1inRegion2	4941
CNVR58	683	4	88694499	88704809	gain	461	23809394	102054630	Loin_muscle_area	region1inRegion2	10310
CNVR55	639	4	52447075	52460832	gain	462	23809394	102054630	Carcass_length	region1inRegion2	13757
CNVR56	649	4	55682568	55685476	loss	462	23809394	102054630	Carcass_length	region1inRegion2	2908
CNVR57	668	4	67469665	67474606	loss	462	23809394	102054630	Carcass_length	region1inRegion2	4941
CNVR58	683	4	88694499	88704809	gain	462	23809394	102054630	Carcass_length	region1inRegion2	10310
CNVR55	639	4	52447075	52460832	gain	463	23809394	102054630	backfat_at_mid-back	region1inRegion2	13757
CNVR56	649	4	55682568	55685476	loss	463	23809394	102054630	backfat_at_mid-back	region1inRegion2	2908
CNVR57	668	4	67469665	67474606	loss	463	23809394	102054630	backfat_at_mid-back	region1inRegion2	4941
CNVR58	683	4	88694499	88704809	gain	463	23809394	102054630	backfat_at_mid-back	region1inRegion2	10310
CNVR55	639	4	52447075	52460832	gain	598	23809394	102054630	Fat_androstenone_level	region1inRegion2	13757
CNVR56	649	4	55682568	55685476	loss	598	23809394	102054630	Fat_androstenone_level	region1inRegion2	2908
CNVR57	668	4	67469665	67474606	loss	598	23809394	102054630	Fat_androstenone_level	region1inRegion2	4941
CNVR58	683	4	88694499	88704809	gain	598	23809394	102054630	Fat_androstenone_level	region1inRegion2	10310
CNVR55	639	4	52447075	52460832	gain	599	23809394	102054630	Fat_androstenone_level	region1inRegion2	13757
CNVR56	649	4	55682568	55685476	loss	599	23809394	102054630	Fat_androstenone_level	region1inRegion2	2908
CNVR57	668	4	67469665	67474606	loss	599	23809394	102054630	Fat_androstenone_level	region1inRegion2	4941
CNVR58	683	4	88694499	88704809	gain	599	23809394	102054630	Fat_androstenone_level	region1inRegion2	10310
CNVR55	639	4	52447075	52460832	gain	600	23809394	102054630	Fat_androstenone_level	region1inRegion2	13757
CNVR56	649	4	55682568	55685476	loss	600	23809394	102054630	Fat_androstenone_level	region1inRegion2	2908
CNVR57	668	4	67469665	67474606	loss	600	23809394	102054630	Fat_androstenone_level	region1inRegion2	4941
CNVR58	683	4	88694499	88704809	gain	600	23809394	102054630	Fat_androstenone_level	region1inRegion2	10310
CNVR55	639	4	52447075	52460832	gain	369	23809394	120509810	Body_weight_(birth)	region1inRegion2	13757
CNVR56	649	4	55682568	55685476	loss	369	23809394	120509810	Body_weight_(birth)	region1inRegion2	2908
CNVR57	668	4	67469665	67474606	loss	369	23809394	120509810	Body_weight_(birth)	region1inRegion2	4941
CNVR58	683	4	88694499	88704809	gain	369	23809394	120509810	Body_weight_(birth)	region1inRegion2	10310
CNVR59	698	4	111405421	111446020	gain	369	23809394	120509810	Body_weight_(birth)	region1inRegion2	40599
CNVR55	639	4	52447075	52460832	gain	21224	24232478	121166399	body_weight_(17_weeks)	region1inRegion2	13757
CNVR56	649	4	55682568	55685476	loss	21224	24232478	121166399	body_weight_(17_weeks)	region1inRegion2	2908
CNVR57	668	4	67469665	67474606	loss	21224	24232478	121166399	body_weight_(17_weeks)	region1inRegion2	4941
CNVR58	683	4	88694499	88704809	gain	21224	24232478	121166399	body_weight_(17_weeks)	region1inRegion2	10310
CNVR59	698	4	111405421	111446020	gain	21224	24232478	121166399	body_weight_(17_weeks)	region1inRegion2	40599
CNVR55	639	4	52447075	52460832	gain	4896	24630831	114573224	Ham_weight	region1inRegion2	13757
CNVR56	649	4	55682568	55685476	loss	4896	24630831	114573224	Ham_weight	region1inRegion2	2908
CNVR57	668	4	67469665	67474606	loss	4896	24630831	114573224	Ham_weight	region1inRegion2	4941
CNVR58	683	4	88694499	88704809	gain	4896	24630831	114573224	Ham_weight	region1inRegion2	10310
CNVR59	698	4	111405421	111446020	gain	4896	24630831	114573224	Ham_weight	region1inRegion2	40599
CNVR55	639	4	52447075	52460832	gain	261	25322475	106309555	Abdominal_fat_weight	region1inRegion2	13757
CNVR56	649	4	55682568	55685476	loss	261	25322475	106309555	Abdominal_fat_weight	region1inRegion2	2908
CNVR57	668	4	67469665	67474606	loss	261	25322475	106309555	Abdominal_fat_weight	region1inRegion2	4941
CNVR58	683	4	88694499	88704809	gain	261	25322475	106309555	Abdominal_fat_weight	region1inRegion2	10310
CNVR55	639	4	52447075	52460832	gain	409	25322475	115001161	Belly_weight	region1inRegion2	13757
CNVR56	649	4	55682568	55685476	loss	409	25322475	115001161	Belly_weight	region1inRegion2	2908
CNVR57	668	4	67469665	67474606	loss	409	25322475	115001161	Belly_weight	region1inRegion2	4941
CNVR58	683	4	88694499	88704809	gain	409	25322475	115001161	Belly_weight	region1inRegion2	10310
CNVR59	698	4	111405421	111446020	gain	409	25322475	115001161	Belly_weight	region1inRegion2	40599
CNVR55	639	4	52447075	52460832	gain	4890	26069212	85032058	Carcass_length	region1inRegion2	13757
CNVR56	649	4	55682568	55685476	loss	4890	26069212	85032058	Carcass_length	region1inRegion2	2908
CNVR57	668	4	67469665	67474606	loss	4890	26069212	85032058	Carcass_length	region1inRegion2	4941
CNVR55	639	4	52447075	52460832	gain	21225	26607063	69099745	Body_weight_(30_weeks)	region1inRegion2	13757
CNVR56	649	4	55682568	55685476	loss	21225	26607063	69099745	Body_weight_(30_weeks)	region1inRegion2	2908
CNVR57	668	4	67469665	67474606	loss	21225	26607063	69099745	Body_weight_(30_weeks)	region1inRegion2	4941
CNVR55	639	4	52447075	52460832	gain	17545	26607063	69623853	Body_weight_(birth)	region1inRegion2	13757
CNVR56	649	4	55682568	55685476	loss	17545	26607063	69623853	Body_weight_(birth)	region1inRegion2	2908
CNVR57	668	4	67469665	67474606	loss	17545	26607063	69623853	Body_weight_(birth)	region1inRegion2	4941
CNVR55	639	4	52447075	52460832	gain	65800	28031034	69941488	pH_45_minutes_post_mortem	region1inRegion2	13757
CNVR56	649	4	55682568	55685476	loss	65800	28031034	69941488	pH_45_minutes_post_mortem	region1inRegion2	2908
CNVR57	668	4	67469665	67474606	loss	65800	28031034	69941488	pH_45_minutes_post_mortem	region1inRegion2	4941
CNVR55	639	4	52447075	52460832	gain	65801	28031034	69941488	pH_24_hr_post-mortem_(loin)	region1inRegion2	13757
CNVR56	649	4	55682568	55685476	loss	65801	28031034	69941488	pH_24_hr_post-mortem_(loin)	region1inRegion2	2908
CNVR57	668	4	67469665	67474606	loss	65801	28031034	69941488	pH_24_hr_post-mortem_(loin)	region1inRegion2	4941
CNVR55	639	4	52447075	52460832	gain	633	28653406	116386061	Fat-cuts_percentage	region1inRegion2	13757
CNVR56	649	4	55682568	55685476	loss	633	28653406	116386061	Fat-cuts_percentage	region1inRegion2	2908
CNVR57	668	4	67469665	67474606	loss	633	28653406	116386061	Fat-cuts_percentage	region1inRegion2	4941
CNVR58	683	4	88694499	88704809	gain	633	28653406	116386061	Fat-cuts_percentage	region1inRegion2	10310
CNVR59	698	4	111405421	111446020	gain	633	28653406	116386061	Fat-cuts_percentage	region1inRegion2	40599
CNVR55	639	4	52447075	52460832	gain	634	28653406	116386061	Body_weight_(slaughter)	region1inRegion2	13757
CNVR56	649	4	55682568	55685476	loss	634	28653406	116386061	Body_weight_(slaughter)	region1inRegion2	2908
CNVR57	668	4	67469665	67474606	loss	634	28653406	116386061	Body_weight_(slaughter)	region1inRegion2	4941
CNVR58	683	4	88694499	88704809	gain	634	28653406	116386061	Body_weight_(slaughter)	region1inRegion2	10310
CNVR59	698	4	111405421	111446020	gain	634	28653406	116386061	Body_weight_(slaughter)	region1inRegion2	40599
CNVR55	639	4	52447075	52460832	gain	635	28653406	116386061	Carcass_fat-free_weight	region1inRegion2	13757
CNVR56	649	4	55682568	55685476	loss	635	28653406	116386061	Carcass_fat-free_weight	region1inRegion2	2908
CNVR57	668	4	67469665	67474606	loss	635	28653406	116386061	Carcass_fat-free_weight	region1inRegion2	4941
CNVR58	683	4	88694499	88704809	gain	635	28653406	116386061	Carcass_fat-free_weight	region1inRegion2	10310
CNVR59	698	4	111405421	111446020	gain	635	28653406	116386061	Carcass_fat-free_weight	region1inRegion2	40599
CNVR55	639	4	52447075	52460832	gain	638	28653406	116386061	Age_at_slaughter	region1inRegion2	13757
CNVR56	649	4	55682568	55685476	loss	638	28653406	116386061	Age_at_slaughter	region1inRegion2	2908
CNVR57	668	4	67469665	67474606	loss	638	28653406	116386061	Age_at_slaughter	region1inRegion2	4941
CNVR58	683	4	88694499	88704809	gain	638	28653406	116386061	Age_at_slaughter	region1inRegion2	10310
CNVR59	698	4	111405421	111446020	gain	638	28653406	116386061	Age_at_slaughter	region1inRegion2	40599
CNVR55	639	4	52447075	52460832	gain	371	29834177	120509810	Average_backfat_thickness	region1inRegion2	13757
CNVR56	649	4	55682568	55685476	loss	371	29834177	120509810	Average_backfat_thickness	region1inRegion2	2908
CNVR57	668	4	67469665	67474606	loss	371	29834177	120509810	Average_backfat_thickness	region1inRegion2	4941
CNVR58	683	4	88694499	88704809	gain	371	29834177	120509810	Average_backfat_thickness	region1inRegion2	10310
CNVR59	698	4	111405421	111446020	gain	371	29834177	120509810	Average_backfat_thickness	region1inRegion2	40599
CNVR55	639	4	52447075	52460832	gain	21226	32256842	76850856	Body_weight_(34_weeks)	region1inRegion2	13757
CNVR56	649	4	55682568	55685476	loss	21226	32256842	76850856	Body_weight_(34_weeks)	region1inRegion2	2908
CNVR57	668	4	67469665	67474606	loss	21226	32256842	76850856	Body_weight_(34_weeks)	region1inRegion2	4941
CNVR55	639	4	52447075	52460832	gain	5200	32909944	121166399	Body_weight_(end_of_test)	region1inRegion2	13757
CNVR56	649	4	55682568	55685476	loss	5200	32909944	121166399	Body_weight_(end_of_test)	region1inRegion2	2908
CNVR57	668	4	67469665	67474606	loss	5200	32909944	121166399	Body_weight_(end_of_test)	region1inRegion2	4941
CNVR58	683	4	88694499	88704809	gain	5200	32909944	121166399	Body_weight_(end_of_test)	region1inRegion2	10310
CNVR59	698	4	111405421	111446020	gain	5200	32909944	121166399	Body_weight_(end_of_test)	region1inRegion2	40599
CNVR55	639	4	52447075	52460832	gain	17546	33070836	78758261	Belly_weight	region1inRegion2	13757
CNVR56	649	4	55682568	55685476	loss	17546	33070836	78758261	Belly_weight	region1inRegion2	2908
CNVR57	668	4	67469665	67474606	loss	17546	33070836	78758261	Belly_weight	region1inRegion2	4941
CNVR55	639	4	52447075	52460832	gain	21232	33323681	96936045	Shoulder_subcutaneous_fat_thickness	region1inRegion2	13757
CNVR56	649	4	55682568	55685476	loss	21232	33323681	96936045	Shoulder_subcutaneous_fat_thickness	region1inRegion2	2908
CNVR57	668	4	67469665	67474606	loss	21232	33323681	96936045	Shoulder_subcutaneous_fat_thickness	region1inRegion2	4941
CNVR58	683	4	88694499	88704809	gain	21232	33323681	96936045	Shoulder_subcutaneous_fat_thickness	region1inRegion2	10310
CNVR55	639	4	52447075	52460832	gain	510	34252554	52626380	Body_weight_(weaning)	region1inRegion2	13757
CNVR55	639	4	52447075	52460832	gain	459	34252554	102054630	Fat_percentage_in_carcass	region1inRegion2	13757
CNVR56	649	4	55682568	55685476	loss	459	34252554	102054630	Fat_percentage_in_carcass	region1inRegion2	2908
CNVR57	668	4	67469665	67474606	loss	459	34252554	102054630	Fat_percentage_in_carcass	region1inRegion2	4941
CNVR58	683	4	88694499	88704809	gain	459	34252554	102054630	Fat_percentage_in_carcass	region1inRegion2	10310
CNVR55	639	4	52447075	52460832	gain	458	34252554	123430356	Average_daily_gain	region1inRegion2	13757
CNVR56	649	4	55682568	55685476	loss	458	34252554	123430356	Average_daily_gain	region1inRegion2	2908
CNVR57	668	4	67469665	67474606	loss	458	34252554	123430356	Average_daily_gain	region1inRegion2	4941
CNVR58	683	4	88694499	88704809	gain	458	34252554	123430356	Average_daily_gain	region1inRegion2	10310
CNVR59	698	4	111405421	111446020	gain	458	34252554	123430356	Average_daily_gain	region1inRegion2	40599

CNVR55	639	4	52447075	52460832	gain	5874	34869792	85779737	pH_for_Semimembranosus	region1inRegion2	13757
CNVR56	649	4	55682568	55685476	loss	5874	34869792	85779737	pH_for_Semimembranosus	region1inRegion2	2908
CNVR57	668	4	67469665	67474606	loss	5874	34869792	85779737	pH_for_Semimembranosus	region1inRegion2	4941
CNVR55	639	4	52447075	52460832	gain	7269	34869792	85779737	Shoulder_subcutaneous_fat_thickness	region1inRegion2	13757
CNVR56	649	4	55682568	55685476	loss	7269	34869792	85779737	Shoulder_subcutaneous_fat_thickness	region1inRegion2	2908
CNVR57	668	4	67469665	67474606	loss	7269	34869792	85779737	Shoulder_subcutaneous_fat_thickness	region1inRegion2	4941
CNVR55	639	4	52447075	52460832	gain	17561	35171154	64690588	Body_weight_(birth)	region1inRegion2	13757
CNVR56	649	4	55682568	55685476	loss	17561	35171154	64690588	Body_weight_(birth)	region1inRegion2	2908
CNVR55	639	4	52447075	52460832	gain	10	35289465	84951475	Carcass_length	region1inRegion2	13757
CNVR56	649	4	55682568	55685476	loss	10	35289465	84951475	Carcass_length	region1inRegion2	2908
CNVR57	668	4	67469665	67474606	loss	10	35289465	84951475	Carcass_length	region1inRegion2	4941
CNVR55	639	4	52447075	52460832	gain	408	35396630	104147497	Backfat_weight	region1inRegion2	13757
CNVR56	649	4	55682568	55685476	loss	408	35396630	104147497	Backfat_weight	region1inRegion2	2908
CNVR57	668	4	67469665	67474606	loss	408	35396630	104147497	Backfat_weight	region1inRegion2	4941
CNVR58	683	4	88694499	88704809	gain	408	35396630	104147497	Backfat_weight	region1inRegion2	10310
CNVR55	639	4	52447075	52460832	gain	175	35396630	120347078	Body_weight_(3_weeks)	region1inRegion2	13757
CNVR56	649	4	55682568	55685476	loss	175	35396630	120347078	Body_weight_(3_weeks)	region1inRegion2	2908
CNVR57	668	4	67469665	67474606	loss	175	35396630	120347078	Body_weight_(3_weeks)	region1inRegion2	4941
CNVR58	683	4	88694499	88704809	gain	175	35396630	120347078	Body_weight_(3_weeks)	region1inRegion2	10310
CNVR59	698	4	111405421	111446020	gain	175	35396630	120347078	Body_weight_(3_weeks)	region1inRegion2	40599
CNVR55	639	4	52447075	52460832	gain	5084	37144882	61134234	Backfat_at_first_rib	region1inRegion2	13757
CNVR56	649	4	55682568	55685476	loss	5084	37144882	61134234	Backfat_at_first_rib	region1inRegion2	2908
CNVR55	639	4	52447075	52460832	gain	5085	37144882	61134234	backfat_at_last_rib	region1inRegion2	13757
CNVR56	649	4	55682568	55685476	loss	5085	37144882	61134234	backfat_at_last_rib	region1inRegion2	2908
CNVR55	639	4	52447075	52460832	gain	5086	37144882	61134234	Backfat_between_3rd_and_4th_last_ribs	region1inRegion2	13757
CNVR56	649	4	55682568	55685476	loss	5086	37144882	61134234	Backfat_between_3rd_and_4th_last_ribs	region1inRegion2	2908
CNVR55	639	4	52447075	52460832	gain	5087	37144882	61134234	Shoulder_weight	region1inRegion2	13757
CNVR56	649	4	55682568	55685476	loss	5087	37144882	61134234	Shoulder_weight	region1inRegion2	2908
CNVR55	639	4	52447075	52460832	gain	5088	37144882	61134234	Carcass_length	region1inRegion2	13757
CNVR56	649	4	55682568	55685476	loss	5088	37144882	61134234	Carcass_length	region1inRegion2	2908
CNVR55	639	4	52447075	52460832	gain	5089	37144882	61134234	Body_weight_(slaughter)	region1inRegion2	13757
CNVR56	649	4	55682568	55685476	loss	5089	37144882	61134234	Body_weight_(slaughter)	region1inRegion2	2908
CNVR55	639	4	52447075	52460832	gain	12827	38684579	71310074	Adipocyte_diameter	region1inRegion2	13757
CNVR56	649	4	55682568	55685476	loss	12827	38684579	71310074	Adipocyte_diameter	region1inRegion2	2908
CNVR57	668	4	67469665	67474606	loss	12827	38684579	71310074	Adipocyte_diameter	region1inRegion2	4941
CNVR55	639	4	52447075	52460832	gain	12828	38684579	71310074	Adipocyte_diameter	region1inRegion2	13757
CNVR56	649	4	55682568	55685476	loss	12828	38684579	71310074	Adipocyte_diameter	region1inRegion2	2908
CNVR57	668	4	67469665	67474606	loss	12828	38684579	71310074	Adipocyte_diameter	region1inRegion2	4941
CNVR55	639	4	52447075	52460832	gain	21223	39855897	76850856	Abdominal_fat_weight	region1inRegion2	13757
CNVR56	649	4	55682568	55685476	loss	21223	39855897	76850856	Abdominal_fat_weight	region1inRegion2	2908
CNVR57	668	4	67469665	67474606	loss	21223	39855897	76850856	Abdominal_fat_weight	region1inRegion2	4941
CNVR55	639	4	52447075	52460832	gain	174	39855897	93366673	Body_weight_(birth)	region1inRegion2	13757
CNVR56	649	4	55682568	55685476	loss	174	39855897	93366673	Body_weight_(birth)	region1inRegion2	2908
CNVR57	668	4	67469665	67474606	loss	174	39855897	93366673	Body_weight_(birth)	region1inRegion2	4941
CNVR58	683	4	88694499	88704809	gain	174	39855897	93366673	Body_weight_(birth)	region1inRegion2	10310
CNVR55	639	4	52447075	52460832	gain	176	39855897	93366673	body_weight_(10_weeks)	region1inRegion2	13757
CNVR56	649	4	55682568	55685476	loss	176	39855897	93366673	body_weight_(10_weeks)	region1inRegion2	2908
CNVR57	668	4	67469665	67474606	loss	176	39855897	93366673	body_weight_(10_weeks)	region1inRegion2	4941
CNVR58	683	4	88694499	88704809	gain	176	39855897	93366673	body_weight_(10_weeks)	region1inRegion2	10310
CNVR55	639	4	52447075	52460832	gain	179	39855897	119947535	Average_daily_gain	region1inRegion2	13757
CNVR56	649	4	55682568	55685476	loss	179	39855897	119947535	Average_daily_gain	region1inRegion2	2908
CNVR57	668	4	67469665	67474606	loss	179	39855897	119947535	Average_daily_gain	region1inRegion2	4941
CNVR58	683	4	88694499	88704809	gain	179	39855897	119947535	Average_daily_gain	region1inRegion2	10310
CNVR59	698	4	111405421	111446020	gain	179	39855897	119947535	Average_daily_gain	region1inRegion2	40599
CNVR55	639	4	52447075	52460832	gain	17541	42385883	80099908	Average_backfat_thickness	region1inRegion2	13757
CNVR56	649	4	55682568	55685476	loss	17541	42385883	80099908	Average_backfat_thickness	region1inRegion2	2908
CNVR57	668	4	67469665	67474606	loss	17541	42385883	80099908	Average_backfat_thickness	region1inRegion2	4941
CNVR55	639	4	52447075	52460832	gain	178	42915373	104147497	body_weight_(17_weeks)	region1inRegion2	13757
CNVR56	649	4	55682568	55685476	loss	178	42915373	104147497	body_weight_(17_weeks)	region1inRegion2	2908
CNVR57	668	4	67469665	67474606	loss	178	42915373	104147497	body_weight_(17_weeks)	region1inRegion2	4941
CNVR58	683	4	88694499	88704809	gain	178	42915373	104147497	body_weight_(17_weeks)	region1inRegion2	10310
CNVR55	639	4	52447075	52460832	gain	17542	43581945	91749532	Average_daily_gain	region1inRegion2	13757
CNVR56	649	4	55682568	55685476	loss	17542	43581945	91749532	Average_daily_gain	region1inRegion2	2908
CNVR57	668	4	67469665	67474606	loss	17542	43581945	91749532	Average_daily_gain	region1inRegion2	4941
CNVR58	683	4	88694499	88704809	gain	17542	43581945	91749532	Average_daily_gain	region1inRegion2	10310
CNVR55	639	4	52447075	52460832	gain	10409	43815472	106510877	Average_daily_gain	region1inRegion2	13757
CNVR56	649	4	55682568	55685476	loss	10409	43815472	106510877	Average_daily_gain	region1inRegion2	2908
CNVR57	668	4	67469665	67474606	loss	10409	43815472	106510877	Average_daily_gain	region1inRegion2	4941
CNVR58	683	4	88694499	88704809	gain	10409	43815472	106510877	Average_daily_gain	region1inRegion2	10310
CNVR55	639	4	52447075	52460832	gain	10410	43815472	106510877	Backfat_at_rump	region1inRegion2	13757
CNVR56	649	4	55682568	55685476	loss	10410	43815472	106510877	Backfat_at_rump	region1inRegion2	2908
CNVR57	668	4	67469665	67474606	loss	10410	43815472	106510877	Backfat_at_rump	region1inRegion2	4941
CNVR58	683	4	88694499	88704809	gain	10410	43815472	106510877	Backfat_at_rump	region1inRegion2	10310
CNVR55	639	4	52447075	52460832	gain	10411	43815472	106510877	Feed_conversion_ratio	region1inRegion2	13757
CNVR56	649	4	55682568	55685476	loss	10411	43815472	106510877	Feed_conversion_ratio	region1inRegion2	2908
CNVR57	668	4	67469665	67474606	loss	10411	43815472	106510877	Feed_conversion_ratio	region1inRegion2	4941
CNVR58	683	4	88694499	88704809	gain	10411	43815472	106510877	Feed_conversion_ratio	region1inRegion2	10310
CNVR55	639	4	52447075	52460832	gain	410	44242778	95921536	Backfat_thickness_between_3rd_and_4th_rib	region1inRegion2	13757
CNVR56	649	4	55682568	55685476	loss	410	44242778	95921536	Backfat_thickness_between_3rd_and_4th_rib	region1inRegion2	2908
CNVR57	668	4	67469665	67474606	loss	410	44242778	95921536	Backfat_thickness_between_3rd_and_4th_rib	region1inRegion2	4941
CNVR58	683	4	88694499	88704809	gain	410	44242778	95921536	Backfat_thickness_between_3rd_and_4th_rib	region1inRegion2	10310
CNVR55	639	4	52447075	52460832	gain	6406	46783250	52963684	Arachidonic_acid_content	region1inRegion2	13757
CNVR55	639	4	52447075	52460832	gain	671	47498304	98720794	Carcass_length	region1inRegion2	13757
CNVR56	649	4	55682568	55685476	loss	671	47498304	98720794	Carcass_length	region1inRegion2	2908
CNVR57	668	4	67469665	67474606	loss	671	47498304	98720794	Carcass_length	region1inRegion2	4941
CNVR58	683	4	88694499	88704809	gain	671	47498304	98720794	Carcass_length	region1inRegion2	10310
CNVR55	639	4	52447075	52460832	gain	177	47498304	102006493	body_weight_(13_weeks)	region1inRegion2	13757
CNVR56	649	4	55682568	55685476	loss	177	47498304	102006493	body_weight_(13_weeks)	region1inRegion2	2908
CNVR57	668	4	67469665	67474606	loss	177	47498304	102006493	body_weight_(13_weeks)	region1inRegion2	4941
CNVR58	683	4	88694499	88704809	gain	177	47498304	102006493	body_weight_(13_weeks)	region1inRegion2	10310
CNVR55	639	4	52447075	52460832	gain	180	47805278	109026230	Average_daily_gain	region1inRegion2	13757
CNVR56	649	4	55682568	55685476	loss	180	47805278	109026230	Average_daily_gain	region1inRegion2	2908
CNVR57	668	4	67469665	67474606	loss	180	47805278	109026230	Average_daily_gain	region1inRegion2	4941
CNVR58	683	4	88694499	88704809	gain	180	47805278	109026230	Average_daily_gain	region1inRegion2	10310
CNVR55	639	4	52447075	52460832	gain	32105	49505974	54689956	Palmitoleic_acid_content	region1inRegion2	13757
CNVR56	649	4	55682568	55685476	loss	3669	52626380	71310074	backfat_at_last_rib	region1inRegion2	2908
CNVR57	668	4	67469665	67474606	loss	3669	52626380	71310074	backfat_at_last_rib	region1inRegion2	4941
CNVR56	649	4	55682568	55685476	loss	3670	52626380	71310074	Average_daily_gain	region1inRegion2	2908
CNVR57	668	4	67469665	67474606	loss	3670	52626380	71310074	Average_daily_gain	region1inRegion2	4941
CNVR56	649	4	55682568	55685476	loss	3681	52626380	71310074	Carcass_length	region1inRegion2	2908
CNVR57	668	4	67469665	67474606	loss	3681	52626380	71310074	Carcass_length	region1inRegion2	4941
CNVR56	649	4	55682568	55685476	loss	3682	52626380	71310074	Carcass_weight_(hot)	region1inRegion2	2908
CNVR57	668	4	67469665	67474606	loss	3682	52626380	71310074	Carcass_weight_(hot)	region1inRegion2	4941
CNVR56	649	4	55682568	55685476	loss	3683	52626380	71310074	pH_for_Semimembranosus	region1inRegion2	2908
CNVR57	668	4	67469665	67474606	loss	3683	52626380	71310074	pH_for_Semimembranosus	region1inRegion2	4941
CNVR56	649	4	55682568	55685476	loss	4125	52626380	71310074	Average_daily_gain	region1inRegion2	2908
CNVR57	668	4	67469665	67474606	loss	4125	52626380	71310074	Average_daily_gain	region1inRegion2	4941
CNVR56	649	4	55682568	55685476	loss	4126	52626380	71310074	backfat_at_P2_position	region1inRegion2	2908
CNVR57	668	4	67469665	67474606	loss	4126	52626380	71310074	backfat_at_P2_position	region1inRegion2	4941
CNVR56	649	4	55682568	55685476	loss	4127	52626380	71310074	backfat_at_P2_position	region1inRegion2	2908
CNVR57	668	4	67469665	67474606	loss	4127	52626380	71310074	backfat_at_P2_position	region1inRegion2	4941
CNVR56	649	4	55682568	55685476	loss	4128	52626380	71310074	backfat_at_P2_position	region1inRegion2	2908
CNVR57	668	4	67469665	67474606	loss	4128	52626380	71310074	backfat_at_P2_position	region1inRegion2	4941
CNVR56	649	4	55682568	55685476	loss	4129	52626380	71310074	Lean_meat_percentage	region1inRegion2	2908
CNVR57	668	4	67469665	67474606	loss	4129	52626380	71310074	Lean_meat_percentage	region1inRegion2	4941
CNVR56	649	4	55682568	55685476	loss	4130	52626380	71310074	pH_for_Semimembranosus	region1inRegion2	2908
CNVR57	668	4	67469665	67474606	loss	4130	52626380	71310074	pH_for_Semimembranosus	region1inRegion2	4941
CNVR56	649	4	55682568	55685476	loss	4179	52626380	71310074	Conductivity_24_hours_postmortem_(ham)	region1inRegion2	2908
CNVR57	668	4	67469665	67474606	loss	4179	52626380	71310074	Conductivity_24_hours_postmortem_(ham)	region1inRegion2	4941
CNVR56	649	4	55682568	55685476	loss	4246	52626380	71310074	Carcass_weight_(hot)	region1inRegion2	2908
CNVR57	668	4	67469665	67474606	loss	4246	52626380	71310074	Carcass_weight_(hot)	region1inRegion2	4941
CNVR56	649	4	55682568	55685476	loss	21325	52626380	102054630	White_blood_cell_counts	region1inRegion2	2908

CNVR57	668	4	67469665	67474606	loss	21325	52626380	102054630	White_blood_cell_counts	region1inRegion2	4941
CNVR58	683	4	88694499	88704809	gain	21325	52626380	102054630	White_blood_cell_counts	region1inRegion2	10310
CNVR56	649	4	55682568	55685476	loss	511	52626380	102054630	Average_daily_gain	region1inRegion2	2908
CNVR57	668	4	67469665	67474606	loss	511	52626380	102054630	Average_daily_gain	region1inRegion2	4941
CNVR58	683	4	88694499	88704809	gain	511	52626380	102054630	Average_daily_gain	region1inRegion2	10310
CNVR56	649	4	55682568	55685476	loss	631	52626380	102054630	Abdominal_fat_weight	region1inRegion2	2908
CNVR57	668	4	67469665	67474606	loss	631	52626380	102054630	Abdominal_fat_weight	region1inRegion2	4941
CNVR58	683	4	88694499	88704809	gain	631	52626380	102054630	Abdominal_fat_weight	region1inRegion2	10310
CNVR56	649	4	55682568	55685476	loss	632	52626380	102054630	Subcutaneous_fat_thickness	region1inRegion2	2908
CNVR57	668	4	67469665	67474606	loss	632	52626380	102054630	Subcutaneous_fat_thickness	region1inRegion2	4941
CNVR58	683	4	88694499	88704809	gain	632	52626380	102054630	Subcutaneous_fat_thickness	region1inRegion2	10310
CNVR56	649	4	55682568	55685476	loss	636	52626380	102054630	Average_daily_gain	region1inRegion2	2908
CNVR57	668	4	67469665	67474606	loss	636	52626380	102054630	Average_daily_gain	region1inRegion2	4941
CNVR58	683	4	88694499	88704809	gain	636	52626380	102054630	Average_daily_gain	region1inRegion2	10310
CNVR56	649	4	55682568	55685476	loss	637	52626380	102054630	Carcass_length	region1inRegion2	2908
CNVR57	668	4	67469665	67474606	loss	637	52626380	102054630	Carcass_length	region1inRegion2	4941
CNVR58	683	4	88694499	88704809	gain	637	52626380	102054630	Carcass_length	region1inRegion2	10310
CNVR56	649	4	55682568	55685476	loss	3092	52626380	107758168	Small_intestine_length	region1inRegion2	2908
CNVR57	668	4	67469665	67474606	loss	3092	52626380	107758168	Small_intestine_length	region1inRegion2	4941
CNVR58	683	4	88694499	88704809	gain	3092	52626380	107758168	Small_intestine_length	region1inRegion2	10310
CNVR56	649	4	55682568	55685476	loss	3871	52626380	140378854	Intramuscular_fat_content	region1inRegion2	2908
CNVR57	668	4	67469665	67474606	loss	3871	52626380	140378854	Intramuscular_fat_content	region1inRegion2	4941
CNVR58	683	4	88694499	88704809	gain	3871	52626380	140378854	Intramuscular_fat_content	region1inRegion2	10310
CNVR59	698	4	111405421	111446020	gain	3871	52626380	140378854	Intramuscular_fat_content	region1inRegion2	40599
CNVR56	649	4	55682568	55685476	loss	411	52974575	95921536	backfat_at_last_rib	region1inRegion2	2908
CNVR57	668	4	67469665	67474606	loss	411	52974575	95921536	backfat_at_last_rib	region1inRegion2	4941
CNVR58	683	4	88694499	88704809	gain	411	52974575	95921536	backfat_at_last_rib	region1inRegion2	10310
CNVR56	649	4	55682568	55685476	loss	32113	53777458	67224733	Linoleic_acid_content	region1inRegion2	2908
CNVR56	649	4	55682568	55685476	loss	17562	55355430	119974883	Average_daily_gain	region1inRegion2	2908
CNVR57	668	4	67469665	67474606	loss	17562	55355430	119974883	Average_daily_gain	region1inRegion2	4941
CNVR58	683	4	88694499	88704809	gain	17562	55355430	119974883	Average_daily_gain	region1inRegion2	10310
CNVR59	698	4	111405421	111446020	gain	17562	55355430	119974883	Average_daily_gain	region1inRegion2	40599
CNVR57	668	4	67469665	67474606	loss	350	61924032	82084284	Average_backfat_thickness	region1inRegion2	4941
CNVR57	668	4	67469665	67474606	loss	677	62356198	86249654	Backfat_at_first_rib	region1inRegion2	4941
CNVR57	668	4	67469665	67474606	loss	23160	63332317	68417452	Loin_weight	region1inRegion2	4941
CNVR57	668	4	67469665	67474606	loss	21231	65910585	82527275	backfat_at_last_rib	region1inRegion2	4941
CNVR57	668	4	67469665	67474606	loss	32114	67224733	68417452	Linoleic_acid_content	region1inRegion2	4941
CNVR57	668	4	67469665	67474606	loss	18098	67399289	67686972	Number_of_stillborn	region1inRegion2	4941
CNVR58	683	4	88694499	88704809	gain	4892	67509911	114514007	Body_weight_(slaughter)	region1inRegion2	10310
CNVR59	698	4	111405421	111446020	gain	4892	67509911	114514007	Body_weight_(slaughter)	region1inRegion2	40599
CNVR58	683	4	88694499	88704809	gain	309	71310074	89529616	Intramuscular_fat_content	region1inRegion2	10310
CNVR58	683	4	88694499	88704809	gain	976	71310074	89529616	Loin_muscle_area	region1inRegion2	10310
CNVR58	683	4	88694499	88704809	gain	3134	71310074	98720794	Body_weight_(birth)	region1inRegion2	10310
CNVR58	683	4	88694499	88704809	gain	3135	71310074	98720794	Trimmed_wholesale_product_/_live_weight	region1inRegion2	10310
CNVR58	683	4	88694499	88704809	gain	3136	71310074	98720794	Trimmed_wholesale_product_/_live_weight	region1inRegion2	10310
CNVR58	683	4	88694499	88704809	gain	3137	71310074	98720794	Average_daily_gain	region1inRegion2	10310
CNVR58	683	4	88694499	88704809	gain	3138	71310074	98720794	Average_daily_gain	region1inRegion2	10310
CNVR58	683	4	88694499	88704809	gain	3156	71310074	98720794	Backfat_weight	region1inRegion2	10310
CNVR58	683	4	88694499	88704809	gain	3157	71310074	98720794	Backfat_at_last_lumbar	region1inRegion2	10310
CNVR58	683	4	88694499	88704809	gain	3158	71310074	98720794	Backfat_thickness_between_3rd_and_4th_rib	region1inRegion2	10310
CNVR58	683	4	88694499	88704809	gain	160	71310074	123430356	Subacid_smell	region1inRegion2	10310
CNVR59	698	4	111405421	111446020	gain	160	71310074	123430356	Subacid_smell	region1inRegion2	40599
CNVR58	683	4	88694499	88704809	gain	162	71310074	134902449	Average_backfat_thickness	region1inRegion2	10310
CNVR59	698	4	111405421	111446020	gain	162	71310074	134902449	Average_backfat_thickness	region1inRegion2	40599
CNVR58	683	4	88694499	88704809	gain	3933	71310074	140378854	Shear_force_at_first_peak	region1inRegion2	10310
CNVR59	698	4	111405421	111446020	gain	3933	71310074	140378854	Shear_force_at_first_peak	region1inRegion2	40599
CNVR58	683	4	88694499	88704809	gain	3934	71310074	140378854	Total_shear_work	region1inRegion2	10310
CNVR59	698	4	111405421	111446020	gain	3934	71310074	140378854	Total_shear_work	region1inRegion2	40599
CNVR58	683	4	88694499	88704809	gain	2981	77094703	102054630	Firmness	region1inRegion2	10310
CNVR58	683	4	88694499	88704809	gain	2982	77094703	102054630	Intramuscular_fat_content	region1inRegion2	10310
CNVR58	683	4	88694499	88704809	gain	8856	77094703	106510877	Ear_weight	region1inRegion2	10310
CNVR58	683	4	88694499	88704809	gain	8857	77094703	106510877	Ear_weight	region1inRegion2	10310
CNVR58	683	4	88694499	88704809	gain	8887	77094703	106510877	Ear_area	region1inRegion2	10310
CNVR58	683	4	88694499	88704809	gain	8888	77094703	106510877	Ear_area	region1inRegion2	10310
CNVR58	683	4	88694499	88704809	gain	678	77553155	91640628	backfat_at_last_rib	region1inRegion2	10310
CNVR58	683	4	88694499	88704809	gain	675	77553155	111731603	backfat_at_last_rib	region1inRegion2	10310
CNVR59	698	4	111405421	111446020	gain	675	77553155	111731603	backfat_at_last_rib	region1inRegion2	40599
CNVR58	683	4	88694499	88704809	gain	535	78867164	109026230	Linoleic_acid_content	region1inRegion2	10310
CNVR58	683	4	88694499	88704809	gain	526	78867164	113773938	Backfat_weight	region1inRegion2	10310
CNVR59	698	4	111405421	111446020	gain	526	78867164	113773938	Backfat_weight	region1inRegion2	40599
CNVR58	683	4	88694499	88704809	gain	17836	80511752	106510877	CD4-positive/CD8-positive_leukocyte_ratio	region1inRegion2	10310
CNVR58	683	4	88694499	88704809	gain	37540	80511752	106510877	CSFV_antibody_level	region1inRegion2	10310
CNVR58	683	4	88694499	88704809	gain	672	80511752	120713724	Loin_muscle_area	region1inRegion2	10310
CNVR59	698	4	111405421	111446020	gain	672	80511752	120713724	Loin_muscle_area	region1inRegion2	40599
CNVR58	683	4	88694499	88704809	gain	12037	81078618	91651519	Small_intestine_length	region1inRegion2	10310
CNVR58	683	4	88694499	88704809	gain	8968	81078618	91651519	Head_weight	region1inRegion2	10310
CNVR58	683	4	88694499	88704809	gain	527	81701886	114514007	Average_backfat_thickness	region1inRegion2	10310
CNVR59	698	4	111405421	111446020	gain	527	81701886	114514007	Average_backfat_thickness	region1inRegion2	40599
CNVR58	683	4	88694499	88704809	gain	2807	81701886	121568996	Diameter_of_type_IIb_muscle_fibers	region1inRegion2	10310
CNVR59	698	4	111405421	111446020	gain	2807	81701886	121568996	Diameter_of_type_IIb_muscle_fibers	region1inRegion2	40599
CNVR58	683	4	88694499	88704809	gain	2808	81701886	121568996	Number_of_muscle_fibers_per_unit_area	region1inRegion2	10310
CNVR59	698	4	111405421	111446020	gain	2808	81701886	121568996	Number_of_muscle_fibers_per_unit_area	region1inRegion2	40599
CNVR58	683	4	88694499	88704809	gain	2809	81701886	121568996	Diameter_of_muscle_fibers	region1inRegion2	10310
CNVR59	698	4	111405421	111446020	gain	2809	81701886	121568996	Diameter_of_muscle_fibers	region1inRegion2	40599
CNVR58	683	4	88694499	88704809	gain	2810	81701886	121568996	Diameter_of_type_IIb_muscle_fibers	region1inRegion2	10310
CNVR59	698	4	111405421	111446020	gain	2810	81701886	121568996	Diameter_of_type_IIb_muscle_fibers	region1inRegion2	40599
CNVR58	683	4	88694499	88704809	gain	12749	82084284	89529616	Carcass_weight_(cold)	region1inRegion2	10310
CNVR58	683	4	88694499	88704809	gain	4203	82084284	106510877	Linoleic_acid_content	region1inRegion2	10310
CNVR58	683	4	88694499	88704809	gain	456	82084284	120509810	Average_daily_gain	region1inRegion2	10310
CNVR59	698	4	111405421	111446020	gain	456	82084284	120509810	Average_daily_gain	region1inRegion2	40599
CNVR58	683	4	88694499	88704809	gain	12667	82942126	96968716	pH_for_Semimembranosus	region1inRegion2	10310
CNVR58	683	4	88694499	88704809	gain	800	83693913	106309555	Backfat_weight	region1inRegion2	10310
CNVR58	683	4	88694499	88704809	gain	813	83693913	106309555	Lean_cuts_percentage	region1inRegion2	10310
CNVR58	683	4	88694499	88704809	gain	3833	84951475	96936045	Average_daily_gain	region1inRegion2	10310
CNVR58	683	4	88694499	88704809	gain	445	84951475	106510877	backfat_at_mid-back	region1inRegion2	10310
CNVR58	683	4	88694499	88704809	gain	3847	84951475	123430356	Water_holding_capacity	region1inRegion2	10310
CNVR59	698	4	111405421	111446020	gain	3847	84951475	123430356	Water_holding_capacity	region1inRegion2	40599
CNVR58	683	4	88694499	88704809	gain	7289	85347460	98261222	Carcass_length	region1inRegion2	10310
CNVR58	683	4	88694499	88704809	gain	32070	86728309	92100663	Eicosatrienoic_acid_content	region1inRegion2	10310
CNVR58	683	4	88694499	88704809	gain	12804	87063391	98261222	Adipocyte_diameter	region1inRegion2	10310
CNVR58	683	4	88694499	88704809	gain	12805	87063391	98261222	Adipocyte_diameter	region1inRegion2	10310
CNVR58	683	4	88694499	88704809	gain	7268	87063391	98261222	Shoulder_subcutaneous_fat_thickness	region1inRegion2	10310
CNVR58	683	4	88694499	88704809	gain	7275	87063391	98261222	backfat_at_last_rib	region1inRegion2	10310
CNVR58	683	4	88694499	88704809	gain	7281	87063391	98261222	Shoulder_weight	region1inRegion2	10310
CNVR58	683	4	88694499	88704809	gain	7288	87063391	98261222	Carcass_length	region1inRegion2	10310
CNVR58	683	4	88694499	88704809	gain	21850	87420715	107758168	pH_45_minutes_post_mortem	region1inRegion2	10310
CNVR58	683	4	88694499	88704809	gain	32104	88255212	89952657	Palmitic_acid_content	region1inRegion2	10310
CNVR59	698	4	111405421	111446020	gain	528	89529616	115508020	Loin_muscle_area	region1inRegion2	40599
CNVR59	698	4	111405421	111446020	gain	816	94356806	117376906	Loin_and_neck_meat_weight	region1inRegion2	40599
CNVR59	698	4	111405421	111446020	gain	15876	94392103	128776464	Palmitic_acid_content	region1inRegion2	40599
CNVR59	698	4	111405421	111446020	gain	4288	95453660	129049892	Japanese_color_scale	region1inRegion2	40599
CNVR59	698	4	111405421	111446020	gain	16848	95921536	111525472	Average_daily_gain	region1inRegion2	40599
CNVR59	698	4	111405421	111446020	gain	967	95921536	115357260	Loin_and_neck_meat_weight	region1inRegion2	40599
CNVR59	698	4	111405421	111446020	gain	973	95921536	115357260	Carcass_weight_(cold)	region1inRegion2	40599
CNVR59	698	4	111405421	111446020	gain	975	95921536	115357260	Body_weight_(slaughter)	region1inRegion2	40599
CNVR59	698	4	111405421	111446020	gain	676	95921536	120713724	Average_backfat_thickness	region1inRegion2	40599
CNVR59	698	4	111405421	111446020	gain	158	96936045	123430356	Margaric_acid_content	region1inRegion2	40599
CNVR59	698	4	111405421	111446020	gain	4207	96936045	123430356	subjective_pork_odor	region1inRegion2	40599
CNVR59	698	4	111405421	111446020	gain	31810	98313842	130318179	Skin_thickness	region1inRegion2	40599
CNVR59	698	4	111405421	111446020	gain	7577	98720794	112174179	Melanoma_susceptibility	region1inRegion2	40599
CNVR59	698	4	111405421	111446020	gain	294	98720794	128083236	Meat_color-a	region1inRegion2	40599
CNVR59	698	4	111405421	111446020	gain	4898	101461640	122987968	CIE-L*	region1inRegion2	40599

CNVR59	698	4	111405421	111446020	gain	16864	102006493	120509810	Carcass_weight_(hot)	region1inRegion2	40599
CNVR59	698	4	111405421	111446020	gain	15875	102054630	120509810	Oleic_acid_content	region1inRegion2	40599
CNVR59	698	4	111405421	111446020	gain	5975	102054630	134902449	Dressing_percentage	region1inRegion2	40599
CNVR59	698	4	111405421	111446020	gain	6003	102054630	134902449	Drip_loss	region1inRegion2	40599
CNVR59	698	4	111405421	111446020	gain	9595	102054630	134902449	Hemolytic_complement_activity_(alternative_pathway)	region1inRegion2	40599
CNVR59	698	4	111405421	111446020	gain	9602	102054630	134902449	Hemolytic_complement_activity_(classical_pathway)	region1inRegion2	40599
CNVR59	698	4	111405421	111446020	gain	9603	102054630	134902449	Hemolytic_complement_activity_(classical_pathway)	region1inRegion2	40599
CNVR59	698	4	111405421	111446020	gain	9604	102054630	134902449	Hemolytic_complement_activity_(classical_pathway)	region1inRegion2	40599
CNVR59	698	4	111405421	111446020	gain	9605	102054630	134902449	Hemolytic_complement_activity_(classical_pathway)	region1inRegion2	40599
CNVR59	698	4	111405421	111446020	gain	9606	102054630	134902449	Hemolytic_complement_activity_(classical_pathway)	region1inRegion2	40599
CNVR59	698	4	111405421	111446020	gain	9607	102054630	134902449	Hemolytic_complement_activity_(classical_pathway)	region1inRegion2	40599
CNVR59	698	4	111405421	111446020	gain	9617	102054630	134902449	C3c_concentration	region1inRegion2	40599
CNVR59	698	4	111405421	111446020	gain	460	102054630	135006664	Carcass_length	region1inRegion2	40599
CNVR59	698	4	111405421	111446020	gain	7473	102054630	140378854	Nonfunctional_nipples	region1inRegion2	40599
CNVR59	698	4	111405421	111446020	gain	49	106510877	124310794	Average_daily_gain	region1inRegion2	40599
CNVR59	698	4	111405421	111446020	gain	51	106510877	134902449	Loin_muscle_area	region1inRegion2	40599
CNVR59	698	4	111405421	111446020	gain	3804	106510877	140378854	Carcass_weight_(cold)	region1inRegion2	40599
CNVR59	698	4	111405421	111446020	gain	968	107758168	115357260	Shoulder_meat_weight	region1inRegion2	40599
CNVR59	698	4	111405421	111446020	gain	970	107758168	115357260	Ham_weight	region1inRegion2	40599
CNVR59	698	4	111405421	111446020	gain	971	107758168	115357260	Carcass_length	region1inRegion2	40599
CNVR59	698	4	111405421	111446020	gain	18020	107758168	124847231	Adipocyte_diameter	region1inRegion2	40599
CNVR59	698	4	111405421	111446020	gain	4899	109026230	119299797	CIE-a*	region1inRegion2	40599
CNVR59	698	4	111405421	111446020	gain	4900	109026230	121308784	Hematin_pigmentation	region1inRegion2	40599
CNVR59	698	4	111405421	111446020	gain	31853	119798655	110586450	Corpus_luteum_number	region2inRegion1	-9212205
CNVR60	727	5	18191073	18199920	gain	17792	844337	34660429	CIE-L*	region1inRegion2	8847
CNVR61	730	5	21242609	21260003	gain	17792	844337	34660429	CIE-L*	region1inRegion2	17394
CNVR62	731	5	21332629	21368821	gain	17792	844337	34660429	CIE-L*	region1inRegion2	36192
CNVR63	736	5	22004036	22012384	loss	17792	844337	34660429	CIE-L*	region1inRegion2	8348
CNVR64	739	5	22410640	22421100	gain	17792	844337	34660429	CIE-L*	region1inRegion2	10460
CNVR60	727	5	18191073	18199920	gain	2927	844337	34660429	Teat_number	region1inRegion2	8847
CNVR61	730	5	21242609	21260003	gain	2927	844337	34660429	Teat_number	region1inRegion2	17394
CNVR62	731	5	21332629	21368821	gain	2927	844337	34660429	Teat_number	region1inRegion2	36192
CNVR63	736	5	22004036	22012384	loss	2927	844337	34660429	Teat_number	region1inRegion2	8348
CNVR64	739	5	22410640	22421100	gain	2927	844337	34660429	Teat_number	region1inRegion2	10460
CNVR60	727	5	18191073	18199920	gain	21322	3688083	19558279	Hemolytic_complement_activity_(alternative_pathway)	region1inRegion2	8847
CNVR60	727	5	18191073	18199920	gain	979	3688083	34660429	Feed_intake	region1inRegion2	8847
CNVR61	730	5	21242609	21260003	gain	979	3688083	34660429	Feed_intake	region1inRegion2	17394
CNVR62	731	5	21332629	21368821	gain	979	3688083	34660429	Feed_intake	region1inRegion2	36192
CNVR63	736	5	22004036	22012384	loss	979	3688083	34660429	Feed_intake	region1inRegion2	8348
CNVR64	739	5	22410640	22421100	gain	979	3688083	34660429	Feed_intake	region1inRegion2	10460
CNVR60	727	5	18191073	18199920	gain	981	3688083	34660429	Backfat_at_tenth_rib	region1inRegion2	8847
CNVR61	730	5	21242609	21260003	gain	981	3688083	34660429	Backfat_at_tenth_rib	region1inRegion2	17394
CNVR62	731	5	21332629	21368821	gain	981	3688083	34660429	Backfat_at_tenth_rib	region1inRegion2	36192
CNVR63	736	5	22004036	22012384	loss	981	3688083	34660429	Backfat_at_tenth_rib	region1inRegion2	8348
CNVR64	739	5	22410640	22421100	gain	981	3688083	34660429	Backfat_at_tenth_rib	region1inRegion2	10460
CNVR60	727	5	18191073	18199920	gain	3805	3688083	35377762	Drip_loss	region1inRegion2	8847
CNVR61	730	5	21242609	21260003	gain	3805	3688083	35377762	Drip_loss	region1inRegion2	17394
CNVR62	731	5	21332629	21368821	gain	3805	3688083	35377762	Drip_loss	region1inRegion2	36192
CNVR63	736	5	22004036	22012384	loss	3805	3688083	35377762	Drip_loss	region1inRegion2	8348
CNVR64	739	5	22410640	22421100	gain	3805	3688083	35377762	Drip_loss	region1inRegion2	10460
CNVR60	727	5	18191073	18199920	gain	38088	3688083	91051340	CIE-L*	region1inRegion2	8847
CNVR61	730	5	21242609	21260003	gain	38088	3688083	91051340	CIE-L*	region1inRegion2	17394
CNVR62	731	5	21332629	21368821	gain	38088	3688083	91051340	CIE-L*	region1inRegion2	36192
CNVR63	736	5	22004036	22012384	loss	38088	3688083	91051340	CIE-L*	region1inRegion2	8348
CNVR64	739	5	22410640	22421100	gain	38088	3688083	91051340	CIE-L*	region1inRegion2	10460
CNVR65	755	5	39192570	39207538	gain	38088	3688083	91051340	CIE-L*	region1inRegion2	14968
CNVR66	766	5	52064895	52069376	loss	38088	3688083	91051340	CIE-L*	region1inRegion2	4481
CNVR67	800	5	79630280	79697936	gain	38088	3688083	91051340	CIE-L*	region1inRegion2	67656
CNVR60	727	5	18191073	18199920	gain	38085	3688083	94410037	Loin_muscle_area	region1inRegion2	8847
CNVR61	730	5	21242609	21260003	gain	38085	3688083	94410037	Loin_muscle_area	region1inRegion2	17394
CNVR62	731	5	21332629	21368821	gain	38085	3688083	94410037	Loin_muscle_area	region1inRegion2	36192
CNVR63	736	5	22004036	22012384	loss	38085	3688083	94410037	Loin_muscle_area	region1inRegion2	8348
CNVR64	739	5	22410640	22421100	gain	38085	3688083	94410037	Loin_muscle_area	region1inRegion2	10460
CNVR65	755	5	39192570	39207538	gain	38085	3688083	94410037	Loin_muscle_area	region1inRegion2	14968
CNVR66	766	5	52064895	52069376	loss	38085	3688083	94410037	Loin_muscle_area	region1inRegion2	4481
CNVR67	800	5	79630280	79697936	gain	38085	3688083	94410037	Loin_muscle_area	region1inRegion2	67656
CNVR60	727	5	18191073	18199920	gain	5238	3688083	97477986	backfat_above_muscle_dorsi	region1inRegion2	8847
CNVR61	730	5	21242609	21260003	gain	5238	3688083	97477986	backfat_above_muscle_dorsi	region1inRegion2	17394
CNVR62	731	5	21332629	21368821	gain	5238	3688083	97477986	backfat_above_muscle_dorsi	region1inRegion2	36192
CNVR63	736	5	22004036	22012384	loss	5238	3688083	97477986	backfat_above_muscle_dorsi	region1inRegion2	8348
CNVR64	739	5	22410640	22421100	gain	5238	3688083	97477986	backfat_above_muscle_dorsi	region1inRegion2	10460
CNVR65	755	5	39192570	39207538	gain	5238	3688083	97477986	backfat_above_muscle_dorsi	region1inRegion2	14968
CNVR66	766	5	52064895	52069376	loss	5238	3688083	97477986	backfat_above_muscle_dorsi	region1inRegion2	4481
CNVR67	800	5	79630280	79697936	gain	5238	3688083	97477986	backfat_above_muscle_dorsi	region1inRegion2	67656
CNVR60	727	5	18191073	18199920	gain	5203	3688083	99658388	backfat_above_muscle_dorsi	region1inRegion2	8847
CNVR61	730	5	21242609	21260003	gain	5203	3688083	99658388	backfat_above_muscle_dorsi	region1inRegion2	17394
CNVR62	731	5	21332629	21368821	gain	5203	3688083	99658388	backfat_above_muscle_dorsi	region1inRegion2	36192
CNVR63	736	5	22004036	22012384	loss	5203	3688083	99658388	backfat_above_muscle_dorsi	region1inRegion2	8348
CNVR64	739	5	22410640	22421100	gain	5203	3688083	99658388	backfat_above_muscle_dorsi	region1inRegion2	10460
CNVR65	755	5	39192570	39207538	gain	5203	3688083	99658388	backfat_above_muscle_dorsi	region1inRegion2	14968
CNVR66	766	5	52064895	52069376	loss	5203	3688083	99658388	backfat_above_muscle_dorsi	region1inRegion2	4481
CNVR67	800	5	79630280	79697936	gain	5203	3688083	99658388	backfat_above_muscle_dorsi	region1inRegion2	67656
CNVR60	727	5	18191073	18199920	gain	5219	3688083	106691183	Shoulder_subcutaneous_fat_thickness	region1inRegion2	8847
CNVR61	730	5	21242609	21260003	gain	5219	3688083	106691183	Shoulder_subcutaneous_fat_thickness	region1inRegion2	17394
CNVR62	731	5	21332629	21368821	gain	5219	3688083	106691183	Shoulder_subcutaneous_fat_thickness	region1inRegion2	36192
CNVR63	736	5	22004036	22012384	loss	5219	3688083	106691183	Shoulder_subcutaneous_fat_thickness	region1inRegion2	8348
CNVR64	739	5	22410640	22421100	gain	5219	3688083	106691183	Shoulder_subcutaneous_fat_thickness	region1inRegion2	10460
CNVR65	755	5	39192570	39207538	gain	5219	3688083	106691183	Shoulder_subcutaneous_fat_thickness	region1inRegion2	14968
CNVR66	766	5	52064895	52069376	loss	5219	3688083	106691183	Shoulder_subcutaneous_fat_thickness	region1inRegion2	4481
CNVR67	800	5	79630280	79697936	gain	5219	3688083	106691183	Shoulder_subcutaneous_fat_thickness	region1inRegion2	67656
CNVR60	727	5	18191073	18199920	gain	17840	3722572	34660429	CD4-positive_leukocyte_percentage	region1inRegion2	8847
CNVR61	730	5	21242609	21260003	gain	17840	3722572	34660429	CD4-positive_leukocyte_percentage	region1inRegion2	17394
CNVR62	731	5	21332629	21368821	gain	17840	3722572	34660429	CD4-positive_leukocyte_percentage	region1inRegion2	36192
CNVR63	736	5	22004036	22012384	loss	17840	3722572	34660429	CD4-positive_leukocyte_percentage	region1inRegion2	8348
CNVR64	739	5	22410640	22421100	gain	17840	3722572	34660429	CD4-positive_leukocyte_percentage	region1inRegion2	10460
CNVR60	727	5	18191073	18199920	gain	31	3722572	35377762	Backfat_at_last_lumbar	region1inRegion2	8847
CNVR61	730	5	21242609	21260003	gain	31	3722572	35377762	Backfat_at_last_lumbar	region1inRegion2	17394
CNVR62	731	5	21332629	21368821	gain	31	3722572	35377762	Backfat_at_last_lumbar	region1inRegion2	36192
CNVR63	736	5	22004036	22012384	loss	31	3722572	35377762	Backfat_at_last_lumbar	region1inRegion2	8348
CNVR64	739	5	22410640	22421100	gain	31	3722572	35377762	Backfat_at_last_lumbar	region1inRegion2	10460
CNVR60	727	5	18191073	18199920	gain	5210	6801841	106691183	backfat_at_mid-back	region1inRegion2	8847
CNVR61	730	5	21242609	21260003	gain	5210	6801841	106691183	backfat_at_mid-back	region1inRegion2	17394
CNVR62	731	5	21332629	21368821	gain	5210	6801841	106691183	backfat_at_mid-back	region1inRegion2	36192
CNVR63	736	5	22004036	22012384	loss	5210	6801841	106691183	backfat_at_mid-back	region1inRegion2	8348
CNVR64	739	5	22410640	22421100	gain	5210	6801841	106691183	backfat_at_mid-back	region1inRegion2	10460
CNVR65	755	5	39192570	39207538	gain	5210	6801841	106691183	backfat_at_mid-back	region1inRegion2	14968
CNVR66	766	5	52064895	52069376	loss	5210	6801841	106691183	backfat_at_mid-back	region1inRegion2	4481
CNVR67	800	5	79630280	79697936	gain	5210	6801841	106691183	backfat_at_mid-back	region1inRegion2	67656
CNVR60	727	5	18191073	18199920	gain	2983	7985523	19558279	Myofibril_fragmentation_index	region1inRegion2	8847
CNVR60	727	5	18191073	18199920	gain	5906	7985523	34660429	Time_spent_feeding	region1inRegion2	8847
CNVR61	730	5	21242609	21260003	gain	5906	7985523	34660429	Time_spent_feeding	region1inRegion2	17394
CNVR62	731	5	21332629	21368821	gain	5906	7985523	34660429	Time_spent_feeding	region1inRegion2	36192
CNVR63	736	5	22004036	22012384	loss	5906	7985523	34660429	Time_spent_feeding	region1inRegion2	8348
CNVR64	739	5	22410640	22421100	gain	5906	7985523	34660429	Time_spent_feeding	region1inRegion2	10460
CNVR60	727	5	18191073	18199920	gain	9620	7985523	44572091	C3c_concentration	region1inRegion2	8847
CNVR61	730	5	21242609	21260003	gain	9620	7985523	44572091	C3c_concentration	region1inRegion2	17394
CNVR62	731	5	21332629	21368821	gain	9620	7985523	44572091	C3c_concentration	region1inRegion2	36192
CNVR63	736	5	22004036	22012384	loss	9620	7985523	44572091	C3c_concentration	region1inRegion2	8348
CNVR64	739	5	22410640	22421100	gain	9620	7985523	44572091	C3c_concentration	region1inRegion2	10460
CNVR65	755	5	39192570	39207538	gain	9620	7985523	44572091	C3c_concentration	region1inRegion2	14968
CNVR60	727	5	18191073	18199920	gain	12083	7985523	63117217	Meat_color-b	region1inRegion2	8847
CNVR61	730	5	21242609	21260003	gain	12083	7985523	63117217	Meat_color-b	region1inRegion2	17394

CNVR62	731	5	21332629	21368821	gain	12083	7985523	63117217	Meat_color-b	region1inRegion2	36192
CNVR63	736	5	22004036	22012384	loss	12083	7985523	63117217	Meat_color-b	region1inRegion2	8348
CNVR64	739	5	22410640	22421100	gain	12083	7985523	63117217	Meat_color-b	region1inRegion2	10460
CNVR65	755	5	39192570	39207538	gain	12083	7985523	63117217	Meat_color-b	region1inRegion2	14968
CNVR66	766	5	52064895	52069376	loss	12083	7985523	63117217	Meat_color-b	region1inRegion2	4481
CNVR60	727	5	18191073	18199920	gain	12084	7985523	63117217	Meat_color-L	region1inRegion2	8847
CNVR61	730	5	21242609	21260003	gain	12084	7985523	63117217	Meat_color-L	region1inRegion2	17394
CNVR62	731	5	21332629	21368821	gain	12084	7985523	63117217	Meat_color-L	region1inRegion2	36192
CNVR63	736	5	22004036	22012384	loss	12084	7985523	63117217	Meat_color-L	region1inRegion2	8348
CNVR64	739	5	22410640	22421100	gain	12084	7985523	63117217	Meat_color-L	region1inRegion2	10460
CNVR65	755	5	39192570	39207538	gain	12084	7985523	63117217	Meat_color-L	region1inRegion2	14968
CNVR66	766	5	52064895	52069376	loss	12084	7985523	63117217	Meat_color-L	region1inRegion2	4481
CNVR60	727	5	18191073	18199920	gain	12085	7985523	63117217	pH_24_hr_post-mortem_(loin)	region1inRegion2	8847
CNVR61	730	5	21242609	21260003	gain	12085	7985523	63117217	pH_24_hr_post-mortem_(loin)	region1inRegion2	17394
CNVR62	731	5	21332629	21368821	gain	12085	7985523	63117217	pH_24_hr_post-mortem_(loin)	region1inRegion2	36192
CNVR63	736	5	22004036	22012384	loss	12085	7985523	63117217	pH_24_hr_post-mortem_(loin)	region1inRegion2	8348
CNVR64	739	5	22410640	22421100	gain	12085	7985523	63117217	pH_24_hr_post-mortem_(loin)	region1inRegion2	10460
CNVR65	755	5	39192570	39207538	gain	12085	7985523	63117217	pH_24_hr_post-mortem_(loin)	region1inRegion2	14968
CNVR66	766	5	52064895	52069376	loss	12085	7985523	63117217	pH_24_hr_post-mortem_(loin)	region1inRegion2	4481
CNVR60	727	5	18191073	18199920	gain	12086	7985523	63117217	Shear_force	region1inRegion2	8847
CNVR61	730	5	21242609	21260003	gain	12086	7985523	63117217	Shear_force	region1inRegion2	17394
CNVR62	731	5	21332629	21368821	gain	12086	7985523	63117217	Shear_force	region1inRegion2	36192
CNVR63	736	5	22004036	22012384	loss	12086	7985523	63117217	Shear_force	region1inRegion2	8348
CNVR64	739	5	22410640	22421100	gain	12086	7985523	63117217	Shear_force	region1inRegion2	10460
CNVR65	755	5	39192570	39207538	gain	12086	7985523	63117217	Shear_force	region1inRegion2	14968
CNVR66	766	5	52064895	52069376	loss	12086	7985523	63117217	Shear_force	region1inRegion2	4481
CNVR60	727	5	18191073	18199920	gain	29692	7985523	82096612	body_weight_(10_weeks)	region1inRegion2	8847
CNVR61	730	5	21242609	21260003	gain	29692	7985523	82096612	body_weight_(10_weeks)	region1inRegion2	17394
CNVR62	731	5	21332629	21368821	gain	29692	7985523	82096612	body_weight_(10_weeks)	region1inRegion2	36192
CNVR63	736	5	22004036	22012384	loss	29692	7985523	82096612	body_weight_(10_weeks)	region1inRegion2	8348
CNVR64	739	5	22410640	22421100	gain	29692	7985523	82096612	body_weight_(10_weeks)	region1inRegion2	10460
CNVR65	755	5	39192570	39207538	gain	29692	7985523	82096612	body_weight_(10_weeks)	region1inRegion2	14968
CNVR66	766	5	52064895	52069376	loss	29692	7985523	82096612	body_weight_(10_weeks)	region1inRegion2	4481
CNVR67	800	5	79630280	79697936	gain	29692	7985523	82096612	body_weight_(10_weeks)	region1inRegion2	67656
CNVR60	727	5	18191073	18199920	gain	29693	7985523	82096612	Body_weight_(20_weeks)	region1inRegion2	8847
CNVR61	730	5	21242609	21260003	gain	29693	7985523	82096612	Body_weight_(20_weeks)	region1inRegion2	17394
CNVR62	731	5	21332629	21368821	gain	29693	7985523	82096612	Body_weight_(20_weeks)	region1inRegion2	36192
CNVR63	736	5	22004036	22012384	loss	29693	7985523	82096612	Body_weight_(20_weeks)	region1inRegion2	8348
CNVR64	739	5	22410640	22421100	gain	29693	7985523	82096612	Body_weight_(20_weeks)	region1inRegion2	10460
CNVR65	755	5	39192570	39207538	gain	29693	7985523	82096612	Body_weight_(20_weeks)	region1inRegion2	14968
CNVR66	766	5	52064895	52069376	loss	29693	7985523	82096612	Body_weight_(20_weeks)	region1inRegion2	4481
CNVR67	800	5	79630280	79697936	gain	29693	7985523	82096612	Body_weight_(20_weeks)	region1inRegion2	67656
CNVR60	727	5	18191073	18199920	gain	38087	7985523	91051340	CIE-b*	region1inRegion2	8847
CNVR61	730	5	21242609	21260003	gain	38087	7985523	91051340	CIE-b*	region1inRegion2	17394
CNVR62	731	5	21332629	21368821	gain	38087	7985523	91051340	CIE-b*	region1inRegion2	36192
CNVR63	736	5	22004036	22012384	loss	38087	7985523	91051340	CIE-b*	region1inRegion2	8348
CNVR64	739	5	22410640	22421100	gain	38087	7985523	91051340	CIE-b*	region1inRegion2	10460
CNVR65	755	5	39192570	39207538	gain	38087	7985523	91051340	CIE-b*	region1inRegion2	14968
CNVR66	766	5	52064895	52069376	loss	38087	7985523	91051340	CIE-b*	region1inRegion2	4481
CNVR67	800	5	79630280	79697936	gain	38087	7985523	91051340	CIE-b*	region1inRegion2	67656
CNVR60	727	5	18191073	18199920	gain	38086	7985523	109562487	Marbling	region1inRegion2	8847
CNVR61	730	5	21242609	21260003	gain	38086	7985523	109562487	Marbling	region1inRegion2	17394
CNVR62	731	5	21332629	21368821	gain	38086	7985523	109562487	Marbling	region1inRegion2	36192
CNVR63	736	5	22004036	22012384	loss	38086	7985523	109562487	Marbling	region1inRegion2	8348
CNVR64	739	5	22410640	22421100	gain	38086	7985523	109562487	Marbling	region1inRegion2	10460
CNVR65	755	5	39192570	39207538	gain	38086	7985523	109562487	Marbling	region1inRegion2	14968
CNVR66	766	5	52064895	52069376	loss	38086	7985523	109562487	Marbling	region1inRegion2	4481
CNVR67	800	5	79630280	79697936	gain	38086	7985523	109562487	Marbling	region1inRegion2	67656
CNVR60	727	5	18191073	18199920	gain	5209	11303026	91671608	backfat_at_mid-back	region1inRegion2	8847
CNVR61	730	5	21242609	21260003	gain	5209	11303026	91671608	backfat_at_mid-back	region1inRegion2	17394
CNVR62	731	5	21332629	21368821	gain	5209	11303026	91671608	backfat_at_mid-back	region1inRegion2	36192
CNVR63	736	5	22004036	22012384	loss	5209	11303026	91671608	backfat_at_mid-back	region1inRegion2	8348
CNVR64	739	5	22410640	22421100	gain	5209	11303026	91671608	backfat_at_mid-back	region1inRegion2	10460
CNVR65	755	5	39192570	39207538	gain	5209	11303026	91671608	backfat_at_mid-back	region1inRegion2	14968
CNVR66	766	5	52064895	52069376	loss	5209	11303026	91671608	backfat_at_mid-back	region1inRegion2	4481
CNVR67	800	5	79630280	79697936	gain	5209	11303026	91671608	backfat_at_mid-back	region1inRegion2	67656
CNVR60	727	5	18191073	18199920	gain	24288	11496289	77598894	Teat_number	region1inRegion2	8847
CNVR61	730	5	21242609	21260003	gain	24288	11496289	77598894	Teat_number	region1inRegion2	17394
CNVR62	731	5	21332629	21368821	gain	24288	11496289	77598894	Teat_number	region1inRegion2	36192
CNVR63	736	5	22004036	22012384	loss	24288	11496289	77598894	Teat_number	region1inRegion2	8348
CNVR64	739	5	22410640	22421100	gain	24288	11496289	77598894	Teat_number	region1inRegion2	10460
CNVR65	755	5	39192570	39207538	gain	24288	11496289	77598894	Teat_number	region1inRegion2	14968
CNVR66	766	5	52064895	52069376	loss	24288	11496289	77598894	Teat_number	region1inRegion2	4481
CNVR60	727	5	18191073	18199920	gain	8931	11511665	34660429	Front_feet_conformation	region1inRegion2	8847
CNVR61	730	5	21242609	21260003	gain	8931	11511665	34660429	Front_feet_conformation	region1inRegion2	17394
CNVR62	731	5	21332629	21368821	gain	8931	11511665	34660429	Front_feet_conformation	region1inRegion2	36192
CNVR63	736	5	22004036	22012384	loss	8931	11511665	34660429	Front_feet_conformation	region1inRegion2	8348
CNVR64	739	5	22410640	22421100	gain	8931	11511665	34660429	Front_feet_conformation	region1inRegion2	10460
CNVR60	727	5	18191073	18199920	gain	412	12281569	69669779	Backfat_thickness_between_3rd_and_4th_rib	region1inRegion2	8847
CNVR61	730	5	21242609	21260003	gain	412	12281569	69669779	Backfat_thickness_between_3rd_and_4th_rib	region1inRegion2	17394
CNVR62	731	5	21332629	21368821	gain	412	12281569	69669779	Backfat_thickness_between_3rd_and_4th_rib	region1inRegion2	36192
CNVR63	736	5	22004036	22012384	loss	412	12281569	69669779	Backfat_thickness_between_3rd_and_4th_rib	region1inRegion2	8348
CNVR64	739	5	22410640	22421100	gain	412	12281569	69669779	Backfat_thickness_between_3rd_and_4th_rib	region1inRegion2	10460
CNVR65	755	5	39192570	39207538	gain	412	12281569	69669779	Backfat_thickness_between_3rd_and_4th_rib	region1inRegion2	14968
CNVR66	766	5	52064895	52069376	loss	412	12281569	69669779	Backfat_thickness_between_3rd_and_4th_rib	region1inRegion2	4481
CNVR60	727	5	18191073	18199920	gain	21236	12787566	95713319	Backfat_at_first_rib	region1inRegion2	8847
CNVR61	730	5	21242609	21260003	gain	21236	12787566	95713319	Backfat_at_first_rib	region1inRegion2	17394
CNVR62	731	5	21332629	21368821	gain	21236	12787566	95713319	Backfat_at_first_rib	region1inRegion2	36192
CNVR63	736	5	22004036	22012384	loss	21236	12787566	95713319	Backfat_at_first_rib	region1inRegion2	8348
CNVR64	739	5	22410640	22421100	gain	21236	12787566	95713319	Backfat_at_first_rib	region1inRegion2	10460
CNVR65	755	5	39192570	39207538	gain	21236	12787566	95713319	Backfat_at_first_rib	region1inRegion2	14968
CNVR66	766	5	52064895	52069376	loss	21236	12787566	95713319	Backfat_at_first_rib	region1inRegion2	4481
CNVR67	800	5	79630280	79697936	gain	21236	12787566	95713319	Backfat_at_first_rib	region1inRegion2	67656
CNVR60	727	5	18191073	18199920	gain	5652	12849046	87904294	Ear_erectness	region1inRegion2	8847
CNVR61	730	5	21242609	21260003	gain	5652	12849046	87904294	Ear_erectness	region1inRegion2	17394
CNVR62	731	5	21332629	21368821	gain	5652	12849046	87904294	Ear_erectness	region1inRegion2	36192
CNVR63	736	5	22004036	22012384	loss	5652	12849046	87904294	Ear_erectness	region1inRegion2	8348
CNVR64	739	5	22410640	22421100	gain	5652	12849046	87904294	Ear_erectness	region1inRegion2	10460
CNVR65	755	5	39192570	39207538	gain	5652	12849046	87904294	Ear_erectness	region1inRegion2	14968
CNVR66	766	5	52064895	52069376	loss	5652	12849046	87904294	Ear_erectness	region1inRegion2	4481
CNVR67	800	5	79630280	79697936	gain	5652	12849046	87904294	Ear_erectness	region1inRegion2	67656
CNVR60	727	5	18191073	18199920	gain	5647	14234697	86649149	Ear_erectness	region1inRegion2	8847
CNVR61	730	5	21242609	21260003	gain	5647	14234697	86649149	Ear_erectness	region1inRegion2	17394
CNVR62	731	5	21332629	21368821	gain	5647	14234697	86649149	Ear_erectness	region1inRegion2	36192
CNVR63	736	5	22004036	22012384	loss	5647	14234697	86649149	Ear_erectness	region1inRegion2	8348
CNVR64	739	5	22410640	22421100	gain	5647	14234697	86649149	Ear_erectness	region1inRegion2	10460
CNVR65	755	5	39192570	39207538	gain	5647	14234697	86649149	Ear_erectness	region1inRegion2	14968
CNVR66	766	5	52064895	52069376	loss	5647	14234697	86649149	Ear_erectness	region1inRegion2	4481
CNVR67	800	5	79630280	79697936	gain	5647	14234697	86649149	Ear_erectness	region1inRegion2	67656
CNVR60	727	5	18191073	18199920	gain	18378	14401823	67445352	uterine_horn_weight	region1inRegion2	8847
CNVR61	730	5	21242609	21260003	gain	18378	14401823	67445352	uterine_horn_weight	region1inRegion2	17394
CNVR62	731	5	21332629	21368821	gain	18378	14401823	67445352	uterine_horn_weight	region1inRegion2	36192
CNVR63	736	5	22004036	22012384	loss	18378	14401823	67445352	uterine_horn_weight	region1inRegion2	8348
CNVR64	739	5	22410640	22421100	gain	18378	14401823	67445352	uterine_horn_weight	region1inRegion2	10460
CNVR65	755	5	39192570	39207538	gain	18378	14401823	67445352	uterine_horn_weight	region1inRegion2	14968
CNVR66	766	5	52064895	52069376	loss	18378	14401823	67445352	uterine_horn_weight	region1inRegion2	4481
CNVR60	727	5	18191073	18199920	gain	18380	14401823	67445352	Reproductive_tract_weight	region1inRegion2	8847
CNVR61	730	5	21242609	21260003	gain	18380	14401823	67445352	Reproductive_tract_weight	region1inRegion2	17394
CNVR62	731	5	21332629	21368821	gain	18380	14401823	67445352	Reproductive_tract_weight	region1inRegion2	36192
CNVR63	736	5	22004036	22012384	loss	18380	14401823	67445352	Reproductive_tract_weight	region1inRegion2	8348
CNVR64	739	5	22410640	22421100	gain	18380	14401823	67445352	Reproductive_tract_weight	region1inRegion2	10460
CNVR65	755	5	39192570	39207538	gain	18380	14401823	67445352	Reproductive_tract_weight	region1inRegion2	14968

CNVR66	766	5	52064895	52069376	loss	18380	14401823	67445352	Reproductive_tract_weight	region1inRegion2	4481
CNVR60	727	5	18191073	18199920	gain	8718	15124756	67279568	Hind_leg_conformation	region1inRegion2	8847
CNVR61	730	5	21242609	21260003	gain	8718	15124756	67279568	Hind_leg_conformation	region1inRegion2	17394
CNVR62	731	5	21332629	21368821	gain	8718	15124756	67279568	Hind_leg_conformation	region1inRegion2	36192
CNVR63	736	5	22004036	22012384	loss	8718	15124756	67279568	Hind_leg_conformation	region1inRegion2	8348
CNVR64	739	5	22410640	22421100	gain	8718	15124756	67279568	Hind_leg_conformation	region1inRegion2	10460
CNVR65	755	5	39192570	39207538	gain	8718	15124756	67279568	Hind_leg_conformation	region1inRegion2	14968
CNVR66	766	5	52064895	52069376	loss	8718	15124756	67279568	Hind_leg_conformation	region1inRegion2	4481
CNVR60	727	5	18191073	18199920	gain	21237	17363583	95652135	Backfat_at_rump	region1inRegion2	8847
CNVR61	730	5	21242609	21260003	gain	21237	17363583	95652135	Backfat_at_rump	region1inRegion2	17394
CNVR62	731	5	21332629	21368821	gain	21237	17363583	95652135	Backfat_at_rump	region1inRegion2	36192
CNVR63	736	5	22004036	22012384	loss	21237	17363583	95652135	Backfat_at_rump	region1inRegion2	8348
CNVR64	739	5	22410640	22421100	gain	21237	17363583	95652135	Backfat_at_rump	region1inRegion2	10460
CNVR65	755	5	39192570	39207538	gain	21237	17363583	95652135	Backfat_at_rump	region1inRegion2	14968
CNVR66	766	5	52064895	52069376	loss	21237	17363583	95652135	Backfat_at_rump	region1inRegion2	4481
CNVR67	800	5	79630280	79697936	gain	21237	17363583	95652135	Backfat_at_rump	region1inRegion2	67656
CNVR60	727	5	18191073	18199920	gain	21239	18084252	100641204	Shoulder_subcutaneous_fat_thickness	region1inRegion2	8847
CNVR61	730	5	21242609	21260003	gain	21239	18084252	100641204	Shoulder_subcutaneous_fat_thickness	region1inRegion2	17394
CNVR62	731	5	21332629	21368821	gain	21239	18084252	100641204	Shoulder_subcutaneous_fat_thickness	region1inRegion2	36192
CNVR63	736	5	22004036	22012384	loss	21239	18084252	100641204	Shoulder_subcutaneous_fat_thickness	region1inRegion2	8348
CNVR64	739	5	22410640	22421100	gain	21239	18084252	100641204	Shoulder_subcutaneous_fat_thickness	region1inRegion2	10460
CNVR65	755	5	39192570	39207538	gain	21239	18084252	100641204	Shoulder_subcutaneous_fat_thickness	region1inRegion2	14968
CNVR66	766	5	52064895	52069376	loss	21239	18084252	100641204	Shoulder_subcutaneous_fat_thickness	region1inRegion2	4481
CNVR67	800	5	79630280	79697936	gain	21239	18084252	100641204	Shoulder_subcutaneous_fat_thickness	region1inRegion2	67656
CNVR61	730	5	21242609	21260003	gain	12337	19558279	34660429	Interferon-gamma_level	region1inRegion2	17394
CNVR62	731	5	21332629	21368821	gain	12337	19558279	34660429	Interferon-gamma_level	region1inRegion2	36192
CNVR63	736	5	22004036	22012384	loss	12337	19558279	34660429	Interferon-gamma_level	region1inRegion2	8348
CNVR64	739	5	22410640	22421100	gain	12337	19558279	34660429	Interferon-gamma_level	region1inRegion2	10460
CNVR61	730	5	21242609	21260003	gain	12338	19558279	34660429	Change_in_interferon-gamma_level	region1inRegion2	17394
CNVR62	731	5	21332629	21368821	gain	12338	19558279	34660429	Change_in_interferon-gamma_level	region1inRegion2	36192
CNVR63	736	5	22004036	22012384	loss	12338	19558279	34660429	Change_in_interferon-gamma_level	region1inRegion2	8348
CNVR64	739	5	22410640	22421100	gain	12338	19558279	34660429	Change_in_interferon-gamma_level	region1inRegion2	10460
CNVR61	730	5	21242609	21260003	gain	17619	19558279	34660429	Toll-like_receptor_9_level	region1inRegion2	17394
CNVR62	731	5	21332629	21368821	gain	17619	19558279	34660429	Toll-like_receptor_9_level	region1inRegion2	36192
CNVR63	736	5	22004036	22012384	loss	17619	19558279	34660429	Toll-like_receptor_9_level	region1inRegion2	8348
CNVR64	739	5	22410640	22421100	gain	17619	19558279	34660429	Toll-like_receptor_9_level	region1inRegion2	10460
CNVR61	730	5	21242609	21260003	gain	984	19558279	34660429	Teat_number	region1inRegion2	17394
CNVR62	731	5	21332629	21368821	gain	984	19558279	34660429	Teat_number	region1inRegion2	36192
CNVR63	736	5	22004036	22012384	loss	984	19558279	34660429	Teat_number	region1inRegion2	8348
CNVR64	739	5	22410640	22421100	gain	984	19558279	34660429	Teat_number	region1inRegion2	10460
CNVR61	730	5	21242609	21260003	gain	2984	19558279	35377762	CIE-b*	region1inRegion2	17394
CNVR62	731	5	21332629	21368821	gain	2984	19558279	35377762	CIE-b*	region1inRegion2	36192
CNVR63	736	5	22004036	22012384	loss	2984	19558279	35377762	CIE-b*	region1inRegion2	8348
CNVR64	739	5	22410640	22421100	gain	2984	19558279	35377762	CIE-b*	region1inRegion2	10460
CNVR61	730	5	21242609	21260003	gain	2985	19558279	35377762	Shear_force	region1inRegion2	17394
CNVR62	731	5	21332629	21368821	gain	2985	19558279	35377762	Shear_force	region1inRegion2	36192
CNVR63	736	5	22004036	22012384	loss	2985	19558279	35377762	Shear_force	region1inRegion2	8348
CNVR64	739	5	22410640	22421100	gain	2985	19558279	35377762	Shear_force	region1inRegion2	10460
CNVR61	730	5	21242609	21260003	gain	9847	19558279	44572091	Drip_loss	region1inRegion2	17394
CNVR62	731	5	21332629	21368821	gain	9847	19558279	44572091	Drip_loss	region1inRegion2	36192
CNVR63	736	5	22004036	22012384	loss	9847	19558279	44572091	Drip_loss	region1inRegion2	8348
CNVR64	739	5	22410640	22421100	gain	9847	19558279	44572091	Drip_loss	region1inRegion2	10460
CNVR65	755	5	39192570	39207538	gain	9847	19558279	44572091	Drip_loss	region1inRegion2	14968
CNVR61	730	5	21242609	21260003	gain	9848	19558279	44572091	Conductivity_24_hours_postmortem_(loin)	region1inRegion2	17394
CNVR62	731	5	21332629	21368821	gain	9848	19558279	44572091	Conductivity_24_hours_postmortem_(loin)	region1inRegion2	36192
CNVR63	736	5	22004036	22012384	loss	9848	19558279	44572091	Conductivity_24_hours_postmortem_(loin)	region1inRegion2	8348
CNVR64	739	5	22410640	22421100	gain	9848	19558279	44572091	Conductivity_24_hours_postmortem_(loin)	region1inRegion2	10460
CNVR65	755	5	39192570	39207538	gain	9848	19558279	44572091	Conductivity_24_hours_postmortem_(loin)	region1inRegion2	14968
CNVR61	730	5	21242609	21260003	gain	9849	19558279	44572091	Conductivity_24_hours_postmortem_(loin)	region1inRegion2	17394
CNVR62	731	5	21332629	21368821	gain	9849	19558279	44572091	Conductivity_24_hours_postmortem_(loin)	region1inRegion2	36192
CNVR63	736	5	22004036	22012384	loss	9849	19558279	44572091	Conductivity_24_hours_postmortem_(loin)	region1inRegion2	8348
CNVR64	739	5	22410640	22421100	gain	9849	19558279	44572091	Conductivity_24_hours_postmortem_(loin)	region1inRegion2	10460
CNVR65	755	5	39192570	39207538	gain	9849	19558279	44572091	Conductivity_24_hours_postmortem_(loin)	region1inRegion2	14968
CNVR61	730	5	21242609	21260003	gain	9850	19558279	44572091	pH_45_minutes_post_mortem	region1inRegion2	17394
CNVR62	731	5	21332629	21368821	gain	9850	19558279	44572091	pH_45_minutes_post_mortem	region1inRegion2	36192
CNVR63	736	5	22004036	22012384	loss	9850	19558279	44572091	pH_45_minutes_post_mortem	region1inRegion2	8348
CNVR64	739	5	22410640	22421100	gain	9850	19558279	44572091	pH_45_minutes_post_mortem	region1inRegion2	10460
CNVR65	755	5	39192570	39207538	gain	9850	19558279	44572091	pH_45_minutes_post_mortem	region1inRegion2	14968
CNVR61	730	5	21242609	21260003	gain	202	19558279	63117217	Average_backfat_thickness	region1inRegion2	17394
CNVR62	731	5	21332629	21368821	gain	202	19558279	63117217	Average_backfat_thickness	region1inRegion2	36192
CNVR63	736	5	22004036	22012384	loss	202	19558279	63117217	Average_backfat_thickness	region1inRegion2	8348
CNVR64	739	5	22410640	22421100	gain	202	19558279	63117217	Average_backfat_thickness	region1inRegion2	10460
CNVR65	755	5	39192570	39207538	gain	202	19558279	63117217	Average_backfat_thickness	region1inRegion2	14968
CNVR66	766	5	52064895	52069376	loss	202	19558279	63117217	Average_backfat_thickness	region1inRegion2	4481
CNVR61	730	5	21242609	21260003	gain	203	19558279	63117217	Average_backfat_thickness	region1inRegion2	17394
CNVR62	731	5	21332629	21368821	gain	203	19558279	63117217	Average_backfat_thickness	region1inRegion2	36192
CNVR63	736	5	22004036	22012384	loss	203	19558279	63117217	Average_backfat_thickness	region1inRegion2	8348
CNVR64	739	5	22410640	22421100	gain	203	19558279	63117217	Average_backfat_thickness	region1inRegion2	10460
CNVR65	755	5	39192570	39207538	gain	203	19558279	63117217	Average_backfat_thickness	region1inRegion2	14968
CNVR66	766	5	52064895	52069376	loss	203	19558279	63117217	Average_backfat_thickness	region1inRegion2	4481
CNVR61	730	5	21242609	21260003	gain	204	19558279	63117217	Average_backfat_thickness	region1inRegion2	17394
CNVR62	731	5	21332629	21368821	gain	204	19558279	63117217	Average_backfat_thickness	region1inRegion2	36192
CNVR63	736	5	22004036	22012384	loss	204	19558279	63117217	Average_backfat_thickness	region1inRegion2	8348
CNVR64	739	5	22410640	22421100	gain	204	19558279	63117217	Average_backfat_thickness	region1inRegion2	10460
CNVR65	755	5	39192570	39207538	gain	204	19558279	63117217	Average_backfat_thickness	region1inRegion2	14968
CNVR66	766	5	52064895	52069376	loss	204	19558279	63117217	Average_backfat_thickness	region1inRegion2	4481
CNVR61	730	5	21242609	21260003	gain	3991	19558279	63117217	CIE-a*	region1inRegion2	17394
CNVR62	731	5	21332629	21368821	gain	3991	19558279	63117217	CIE-a*	region1inRegion2	36192
CNVR63	736	5	22004036	22012384	loss	3991	19558279	63117217	CIE-a*	region1inRegion2	8348
CNVR64	739	5	22410640	22421100	gain	3991	19558279	63117217	CIE-a*	region1inRegion2	10460
CNVR65	755	5	39192570	39207538	gain	3991	19558279	63117217	CIE-a*	region1inRegion2	14968
CNVR66	766	5	52064895	52069376	loss	3991	19558279	63117217	CIE-a*	region1inRegion2	4481
CNVR61	730	5	21242609	21260003	gain	9573	19558279	63117217	Linoleic_acid_content	region1inRegion2	17394
CNVR62	731	5	21332629	21368821	gain	9573	19558279	63117217	Linoleic_acid_content	region1inRegion2	36192
CNVR63	736	5	22004036	22012384	loss	9573	19558279	63117217	Linoleic_acid_content	region1inRegion2	8348
CNVR64	739	5	22410640	22421100	gain	9573	19558279	63117217	Linoleic_acid_content	region1inRegion2	10460
CNVR65	755	5	39192570	39207538	gain	9573	19558279	63117217	Linoleic_acid_content	region1inRegion2	14968
CNVR66	766	5	52064895	52069376	loss	9573	19558279	63117217	Linoleic_acid_content	region1inRegion2	4481
CNVR61	730	5	21242609	21260003	gain	985	19558279	63117217	Liver_weight	region1inRegion2	17394
CNVR62	731	5	21332629	21368821	gain	985	19558279	63117217	Liver_weight	region1inRegion2	36192
CNVR63	736	5	22004036	22012384	loss	985	19558279	63117217	Liver_weight	region1inRegion2	8348
CNVR64	739	5	22410640	22421100	gain	985	19558279	63117217	Liver_weight	region1inRegion2	10460
CNVR65	755	5	39192570	39207538	gain	985	19558279	63117217	Liver_weight	region1inRegion2	14968
CNVR66	766	5	52064895	52069376	loss	985	19558279	63117217	Liver_weight	region1inRegion2	4481
CNVR61	730	5	21242609	21260003	gain	986	19558279	63117217	Shoulder_external_fat_weight	region1inRegion2	17394
CNVR62	731	5	21332629	21368821	gain	986	19558279	63117217	Shoulder_external_fat_weight	region1inRegion2	36192
CNVR63	736	5	22004036	22012384	loss	986	19558279	63117217	Shoulder_external_fat_weight	region1inRegion2	8348
CNVR64	739	5	22410640	22421100	gain	986	19558279	63117217	Shoulder_external_fat_weight	region1inRegion2	10460
CNVR65	755	5	39192570	39207538	gain	986	19558279	63117217	Shoulder_external_fat_weight	region1inRegion2	14968
CNVR66	766	5	52064895	52069376	loss	986	19558279	63117217	Shoulder_external_fat_weight	region1inRegion2	4481
CNVR61	730	5	21242609	21260003	gain	15049	19558279	82096612	White_blood_cell_counts	region1inRegion2	17394
CNVR62	731	5	21332629	21368821	gain	15049	19558279	82096612	White_blood_cell_counts	region1inRegion2	36192
CNVR63	736	5	22004036	22012384	loss	15049	19558279	82096612	White_blood_cell_counts	region1inRegion2	8348
CNVR64	739	5	22410640	22421100	gain	15049	19558279	82096612	White_blood_cell_counts	region1inRegion2	10460
CNVR65	755	5	39192570	39207538	gain	15049	19558279	82096612	White_blood_cell_counts	region1inRegion2	14968
CNVR66	766	5	52064895	52069376	loss	15049	19558279	82096612	White_blood_cell_counts	region1inRegion2	4481
CNVR67	800	5	79630280	79697936	gain	15049	19558279	82096612	White_blood_cell_counts	region1inRegion2	67656
CNVR61	730	5	21242609	21260003	gain	29694	19558279	91679838	Carcass_weight_(hot)	region1inRegion2	17394
CNVR62	731	5	21332629	21368821	gain	29694	19558279	91679838	Carcass_weight_(hot)	region1inRegion2	36192
CNVR63	736	5	22004036	22012384	loss	29694	19558279	91679838	Carcass_weight_(hot)	region1inRegion2	8348
CNVR64	739	5	22410640	22421100	gain	29694	19558279	91679838	Carcass_weight_(hot)	region1inRegion2	10460
CNVR65	755	5	39192570	39207538	gain	29694	19558279	91679838	Carcass_weight_(hot)	region1inRegion2	14968

CNVR66	766	5	52064895	52069376	loss	29694	19558279	91679838	Carcass_weight_(hot)	region1inRegion2	4481
CNVR67	800	5	79630280	79697936	gain	29694	19558279	91679838	Carcass_weight_(hot)	region1inRegion2	67656
CNVR61	730	5	21242609	21260003	gain	13313	20956059	24041095	Cervical_vertebra_length	region1inRegion2	17394
CNVR62	731	5	21332629	21368821	gain	13313	20956059	24041095	Cervical_vertebra_length	region1inRegion2	36192
CNVR63	736	5	22004036	22012384	loss	13313	20956059	24041095	Cervical_vertebra_length	region1inRegion2	8348
CNVR64	739	5	22410640	22421100	gain	13313	20956059	24041095	Cervical_vertebra_length	region1inRegion2	10460
CNVR63	736	5	22004036	22012384	loss	64717	21534750	22112704	Thoracic_vertebra_number	region1inRegion2	8348
CNVR64	739	5	22410640	22421100	gain	323	22412792	34660429	Average_daily_gain	overlapTail	8309
CNVR64	739	5	22410640	22421100	gain	10619	22412792	63117217	uterine_horn_length	overlapTail	8309
CNVR65	755	5	39192570	39207538	gain	10619	22412792	63117217	uterine_horn_length	region1inRegion2	14968
CNVR66	766	5	52064895	52069376	loss	10619	22412792	63117217	uterine_horn_length	region1inRegion2	4481
CNVR65	755	5	39192570	39207538	gain	3918	25236625	78579054	CIE-b*	region1inRegion2	14968
CNVR66	766	5	52064895	52069376	loss	3918	25236625	78579054	CIE-b*	region1inRegion2	4481
CNVR65	755	5	39192570	39207538	gain	3919	25236625	78579054	CIE-b*	region1inRegion2	14968
CNVR66	766	5	52064895	52069376	loss	3919	25236625	78579054	CIE-b*	region1inRegion2	4481
CNVR65	755	5	39192570	39207538	gain	3920	25236625	78579054	CIE-b*	region1inRegion2	14968
CNVR66	766	5	52064895	52069376	loss	3920	25236625	78579054	CIE-b*	region1inRegion2	4481
CNVR65	755	5	39192570	39207538	gain	101	33251630	94763337	pH_24_hr_post-mortem_(loin)	region1inRegion2	14968
CNVR66	766	5	52064895	52069376	loss	101	33251630	94763337	pH_24_hr_post-mortem_(loin)	region1inRegion2	4481
CNVR67	800	5	79630280	79697936	gain	101	33251630	94763337	pH_24_hr_post-mortem_(loin)	region1inRegion2	67656
CNVR65	755	5	39192570	39207538	gain	8858	34660429	86649149	Ear_weight	region1inRegion2	14968
CNVR66	766	5	52064895	52069376	loss	8858	34660429	86649149	Ear_weight	region1inRegion2	4481
CNVR67	800	5	79630280	79697936	gain	8858	34660429	86649149	Ear_weight	region1inRegion2	67656
CNVR65	755	5	39192570	39207538	gain	8859	34660429	86649149	Ear_weight	region1inRegion2	14968
CNVR66	766	5	52064895	52069376	loss	8859	34660429	86649149	Ear_weight	region1inRegion2	4481
CNVR67	800	5	79630280	79697936	gain	8859	34660429	86649149	Ear_weight	region1inRegion2	67656
CNVR65	755	5	39192570	39207538	gain	8860	34660429	86649149	Ear_area	region1inRegion2	14968
CNVR66	766	5	52064895	52069376	loss	8860	34660429	86649149	Ear_area	region1inRegion2	4481
CNVR67	800	5	79630280	79697936	gain	8860	34660429	86649149	Ear_area	region1inRegion2	67656
CNVR65	755	5	39192570	39207538	gain	8861	34660429	86649149	Ear_area	region1inRegion2	14968
CNVR66	766	5	52064895	52069376	loss	8861	34660429	86649149	Ear_area	region1inRegion2	4481
CNVR67	800	5	79630280	79697936	gain	8861	34660429	86649149	Ear_area	region1inRegion2	67656
CNVR65	755	5	39192570	39207538	gain	8862	34660429	86649149	Ear_erectness	region1inRegion2	14968
CNVR66	766	5	52064895	52069376	loss	8862	34660429	86649149	Ear_erectness	region1inRegion2	4481
CNVR67	800	5	79630280	79697936	gain	8862	34660429	86649149	Ear_erectness	region1inRegion2	67656
CNVR65	755	5	39192570	39207538	gain	3990	34883290	90130141	pH_24_hr_post-mortem_(loin)	region1inRegion2	14968
CNVR66	766	5	52064895	52069376	loss	3990	34883290	90130141	pH_24_hr_post-mortem_(loin)	region1inRegion2	4481
CNVR67	800	5	79630280	79697936	gain	3990	34883290	90130141	pH_24_hr_post-mortem_(loin)	region1inRegion2	67656
CNVR65	755	5	39192570	39207538	gain	2986	35377762	56295399	Muscle_moisture_percentage	region1inRegion2	14968
CNVR66	766	5	52064895	52069376	loss	2986	35377762	56295399	Muscle_moisture_percentage	region1inRegion2	4481
CNVR65	755	5	39192570	39207538	gain	2987	35377762	56295399	Taste_intensity	region1inRegion2	14968
CNVR66	766	5	52064895	52069376	loss	2987	35377762	56295399	Taste_intensity	region1inRegion2	4481
CNVR65	755	5	39192570	39207538	gain	12753	35377762	63117217	Shoulder_external_fat_weight	region1inRegion2	14968
CNVR66	766	5	52064895	52069376	loss	12753	35377762	63117217	Shoulder_external_fat_weight	region1inRegion2	4481
CNVR65	755	5	39192570	39207538	gain	3235	35377762	63117217	Backfat_at_first_rib	region1inRegion2	14968
CNVR66	766	5	52064895	52069376	loss	3235	35377762	63117217	Backfat_at_first_rib	region1inRegion2	4481
CNVR65	755	5	39192570	39207538	gain	6467	35386728	68379898	Left_teat_number	region1inRegion2	14968
CNVR66	766	5	52064895	52069376	loss	6467	35386728	68379898	Left_teat_number	region1inRegion2	4481
CNVR66	766	5	52064895	52069376	loss	5244	39602968	95972671	backfat_at_mid-back	region1inRegion2	4481
CNVR67	800	5	79630280	79697936	gain	5244	39602968	95972671	backfat_at_mid-back	region1inRegion2	67656
CNVR66	766	5	52064895	52069376	loss	9643	44572091	85756451	Haptoglobin_concentration	region1inRegion2	4481
CNVR67	800	5	79630280	79697936	gain	9643	44572091	85756451	Haptoglobin_concentration	region1inRegion2	67656
CNVR66	766	5	52064895	52069376	loss	21234	49740293	77889581	Average_backfat_thickness	region1inRegion2	4481
CNVR67	800	5	79630280	79697936	gain	15069	56295399	109562487	Cholesterol_level	region1inRegion2	67656
CNVR67	800	5	79630280	79697936	gain	12087	63117217	85756451	Meat_color-L	region1inRegion2	67656
CNVR67	800	5	79630280	79697936	gain	12088	63117217	85756451	Meat_color_OPTO	region1inRegion2	67656
CNVR67	800	5	79630280	79697936	gain	12089	63117217	85756451	Meat_color_OPTO	region1inRegion2	67656
CNVR67	800	5	79630280	79697936	gain	12090	63117217	85756451	Japanese_color_scale	region1inRegion2	67656
CNVR67	800	5	79630280	79697936	gain	12091	63117217	85756451	Loin_muscle_area	region1inRegion2	67656
CNVR67	800	5	79630280	79697936	gain	12092	63117217	85756451	Loin_weight	region1inRegion2	67656
CNVR67	800	5	79630280	79697936	gain	12093	63117217	85756451	Ham_weight	region1inRegion2	67656
CNVR67	800	5	79630280	79697936	gain	342	63117217	85756451	Average_daily_gain	region1inRegion2	67656
CNVR67	800	5	79630280	79697936	gain	351	63117217	85756451	Average_backfat_thickness	region1inRegion2	67656
CNVR67	800	5	79630280	79697936	gain	4208	63117217	85756451	subjective_abnormal_odor	region1inRegion2	67656
CNVR67	800	5	79630280	79697936	gain	5977	63117217	86649149	Fat_to_meat_ratio	region1inRegion2	67656
CNVR67	800	5	79630280	79697936	gain	5978	63117217	86649149	Belly_meat_content	region1inRegion2	67656
CNVR67	800	5	79630280	79697936	gain	5979	63117217	86649149	Estimated_carcass_lean_content	region1inRegion2	67656
CNVR67	800	5	79630280	79697936	gain	12752	63117217	90130141	Carcass_weight_(cold)	region1inRegion2	67656
CNVR67	800	5	79630280	79697936	gain	3949	63117217	90130141	Backfat_between_6th_and_7th_ribs	region1inRegion2	67656
CNVR67	800	5	79630280	79697936	gain	987	63117217	90130141	Conductivity_24_hours_post-mortem	region1inRegion2	67656
CNVR67	800	5	79630280	79697936	gain	21364	63117217	90673514	Carcass_temperature_(24_hr_post-mortem)_	region1inRegion2	67656
CNVR67	800	5	79630280	79697936	gain	3060	63117217	90673514	Inside_ham_weight	region1inRegion2	67656
CNVR67	800	5	79630280	79697936	gain	3307	63117217	90673514	Carcass_temperature_(24_hr_post-mortem)_	region1inRegion2	67656
CNVR67	800	5	79630280	79697936	gain	3644	63117217	90673514	Empty_body_protein_linear	region1inRegion2	67656
CNVR67	800	5	79630280	79697936	gain	6353	63117217	90673514	Alkaline_phosphatase_activity	region1inRegion2	67656
CNVR67	800	5	79630280	79697936	gain	23166	67162792	80149376	Backfat_thickness_between_3rd_and_4th_rib	region1inRegion2	67656
CNVR67	800	5	79630280	79697936	gain	5849	67333455	83811318	Carcass_temperature_(45_minutes_post-mortem)	region1inRegion2	67656
CNVR67	800	5	79630280	79697936	gain	8960	67360352	84349314	Loin_muscle_area	region1inRegion2	67656
CNVR67	800	5	79630280	79697936	gain	9029	67360352	84349314	Marbling	region1inRegion2	67656
CNVR67	800	5	79630280	79697936	gain	13334	76968997	87235520	Cervical_vertebra_length	region1inRegion2	67656
CNVR67	800	5	79630280	79697936	gain	9863	77037698	91498118	Water_holding_capacity	region1inRegion2	67656
CNVR67	800	5	79630280	79697936	gain	3113	77055629	82096612	Unsaturated_fatty_acid_content	region1inRegion2	67656
CNVR67	800	5	79630280	79697936	gain	3114	77055629	82096612	Saturated_fatty_acid_content	region1inRegion2	67656
CNVR67	800	5	79630280	79697936	gain	3128	77055629	82096612	Unsaturated_fatty_acid_content	region1inRegion2	67656
CNVR67	800	5	79630280	79697936	gain	383	77887905	97514833	Loin_and_ham_percentage_in_carcass	region1inRegion2	67656
CNVR67	800	5	79630280	79697936	gain	413	78463269	97514833	Belly_weight	region1inRegion2	67656
CNVR67	800	5	79630280	79697936	gain	55	78579054	109562487	backfat_at_last_rib	region1inRegion2	67656
CNVR67	800	5	79630280	79697936	gain	21235	79082328	95415220	Body_weight_(34_weeks)	region1inRegion2	67656
CNVR60	727	5	18191073	18199920	gain	18128	103715674	2706495	Number_of_stillborn	region2inRegion1	-101009179
CNVR61	730	5	21242609	21260003	gain	18128	103715674	2706495	Number_of_stillborn	region2inRegion1	-101009179
CNVR62	731	5	21332629	21368821	gain	18128	103715674	2706495	Number_of_stillborn	region2inRegion1	-101009179
CNVR63	736	5	22004036	22012384	loss	18128	103715674	2706495	Number_of_stillborn	region2inRegion1	-101009179
CNVR64	739	5	22410640	22421100	gain	18128	103715674	2706495	Number_of_stillborn	region2inRegion1	-101009179
CNVR65	755	5	39192570	39207538	gain	18128	103715674	2706495	Number_of_stillborn	region2inRegion1	-101009179
CNVR66	766	5	52064895	52069376	loss	18128	103715674	2706495	Number_of_stillborn	region2inRegion1	-101009179
CNVR67	800	5	79630280	79697936	gain	18128	103715674	2706495	Number_of_stillborn	region2inRegion1	-101009179
CNVR68	833	6	8471113	8494128	gain	145	1829370	134683685	Intramuscular_fat_content	region1inRegion2	23015
CNVR69	862	6	95466297	95473943	gain	145	1829370	134683685	Intramuscular_fat_content	region1inRegion2	7646
CNVR70	871	6	107004191	107039849	loss	145	1829370	134683685	Intramuscular_fat_content	region1inRegion2	35658
CNVR68	833	6	8471113	8494128	gain	18022	2352681	19536155	Adipocyte_diameter	region1inRegion2	23015
CNVR68	833	6	8471113	8494128	gain	5425	2352681	19536155	Mean_corpuscular_hemoglobin_content	region1inRegion2	23015
CNVR68	833	6	8471113	8494128	gain	5926	2352681	19536155	Time_spent_socializing	region1inRegion2	23015
CNVR68	833	6	8471113	8494128	gain	6329	2352681	19536155	CO2_partial_pressure	region1inRegion2	23015
CNVR68	833	6	8471113	8494128	gain	5698	2352681	29979924	Loin_muscle_area	region1inRegion2	23015
CNVR68	833	6	8471113	8494128	gain	5699	2352681	29979924	Average_daily_gain	region1inRegion2	23015
CNVR68	833	6	8471113	8494128	gain	5982	2352681	29979924	backfat_at_last_rib	region1inRegion2	23015
CNVR68	833	6	8471113	8494128	gain	5983	2352681	29979924	Subcutanous_fat_area	region1inRegion2	23015
CNVR68	833	6	8471113	8494128	gain	5984	2352681	29979924	Fat_to_meat_ratio	region1inRegion2	23015
CNVR68	833	6	8471113	8494128	gain	5985	2352681	29979924	Belly_meat_content	region1inRegion2	23015
CNVR68	833	6	8471113	8494128	gain	5986	2352681	29979924	Estimated_carcass_lean_content	region1inRegion2	23015
CNVR68	833	6	8471113	8494128	gain	6004	2352681	29979924	Conductivity_24_hours_postmortem_(loin)	region1inRegion2	23015
CNVR68	833	6	8471113	8494128	gain	6005	2352681	29979924	Drip_loss	region1inRegion2	23015
CNVR68	833	6	8471113	8494128	gain	24289	2352681	72944202	Teat_number	region1inRegion2	23015
CNVR68	833	6	8471113	8494128	gain	3921	2352681	129740986	CIE-a*	region1inRegion2	23015
CNVR69	862	6	95466297	95473943	gain	3921	2352681	129740986	CIE-a*	region1inRegion2	7646
CNVR70	871	6	107004191	107039849	loss	3921	2352681	129740986	CIE-a*	region1inRegion2	35658
CNVR68	833	6	8471113	8494128	gain	3922	2352681	129740986	CIE-a*	region1inRegion2	23015
CNVR69	862	6	95466297	95473943	gain	3922	2352681	129740986	CIE-a*	region1inRegion2	7646
CNVR70	871	6	107004191	107039849	loss	3922	2352681	129740986	CIE-a*	region1inRegion2	35658
CNVR68	833	6	8471113	8494128	gain	3923	2352681	129740986	CIE-a*	region1inRegion2	23015
CNVR69	862	6	95466297	95473943	gain	3923	2352681	129740986	CIE-a*	region1inRegion2	7646
CNVR70	871	6	107004191	107039849	loss	3923	2352681	129740986	CIE-a*	region1inRegion2	35658

CNVR68	833	6	8471113	8494128	gain	3924	2352681	129740986	CIE-a*	region1inRegion2	23015
CNVR69	862	6	95466297	95473943	gain	3924	2352681	129740986	CIE-a*	region1inRegion2	7646
CNVR70	871	6	107004191	107039849	loss	3924	2352681	129740986	CIE-a*	region1inRegion2	35658
CNVR68	833	6	8471113	8494128	gain	38089	2352681	146365886	Marbling	region1inRegion2	23015
CNVR69	862	6	95466297	95473943	gain	38089	2352681	146365886	Marbling	region1inRegion2	7646
CNVR70	871	6	107004191	107039849	loss	38089	2352681	146365886	Marbling	region1inRegion2	35658
CNVR68	833	6	8471113	8494128	gain	3063	3913775	9872228	Inside_ham_weight	region1inRegion2	23015
CNVR68	833	6	8471113	8494128	gain	367	3913775	17750243	Intramuscular_fat_content	region1inRegion2	23015
CNVR68	833	6	8471113	8494128	gain	3859	3913775	17750243	Backfat_at_tenth_rib	region1inRegion2	23015
CNVR68	833	6	8471113	8494128	gain	384	8056293	16512374	Loin_and_ham_percentage_in_carcass	region1inRegion2	23015
CNVR68	833	6	8471113	8494128	gain	21240	8247419	80284968	Body_weight_(46_days)	region1inRegion2	23015
CNVR68	833	6	8471113	8494128	gain	65397	8395456	10529959	Loin_muscle_area	region1inRegion2	23015
CNVR68	833	6	8471113	8494128	gain	65398	8395456	10529959	backfat_at_last_rib	region1inRegion2	23015
CNVR68	833	6	8471113	8494128	gain	65399	8395456	10529959	Backfat_at_rump	region1inRegion2	23015
CNVR68	833	6	8471113	8494128	gain	65400	8395456	10529959	Average_backfat_thickness	region1inRegion2	23015
CNVR68	833	6	8471113	8494128	gain	2864	8442460	17750243	Hind_hock_weight	region1inRegion2	23015
CNVR68	833	6	8471113	8494128	gain	3236	8442460	19526758	Meat_color-a	region1inRegion2	23015
CNVR68	833	6	8471113	8494128	gain	332	8442460	19536155	Average_daily_gain	region1inRegion2	23015
CNVR68	833	6	8471113	8494128	gain	343	8442460	19536155	Average_daily_gain	region1inRegion2	23015
CNVR68	833	6	8471113	8494128	gain	38054	8442460	66465212	Loin_muscle_area	region1inRegion2	23015
CNVR68	833	6	8471113	8494128	gain	3879	8453515	8589435	Intramuscular_fat_content	region1inRegion2	23015
CNVR69	862	6	95466297	95473943	gain	5201	9242013	138477175	Body_weight_(end_of_test)	region1inRegion2	7646
CNVR70	871	6	107004191	107039849	loss	5201	9242013	138477175	Body_weight_(end_of_test)	region1inRegion2	35658
CNVR69	862	6	95466297	95473943	gain	11562	9957192	129740986	Salmonella_count_in_spleen	region1inRegion2	7646
CNVR70	871	6	107004191	107039849	loss	11562	9957192	129740986	Salmonella_count_in_spleen	region1inRegion2	35658
CNVR69	862	6	95466297	95473943	gain	38090	17750243	146365886	Loin_fat_percentage	region1inRegion2	7646
CNVR70	871	6	107004191	107039849	loss	38090	17750243	146365886	Loin_fat_percentage	region1inRegion2	35658
CNVR69	862	6	95466297	95473943	gain	15097	17750243	152260387	Alkaline_phosphatase_activity	region1inRegion2	7646
CNVR70	871	6	107004191	107039849	loss	15097	17750243	152260387	Alkaline_phosphatase_activity	region1inRegion2	35658
CNVR71	917	6	151994674	152015484	gain	15097	17750243	152260387	Alkaline_phosphatase_activity	region1inRegion2	20810
CNVR69	862	6	95466297	95473943	gain	15099	18610219	123963269	Lactate_dehydrogenase_level	region1inRegion2	7646
CNVR70	871	6	107004191	107039849	loss	15099	18610219	123963269	Lactate_dehydrogenase_level	region1inRegion2	35658
CNVR69	862	6	95466297	95473943	gain	3873	18942762	122134922	Carcass_length	region1inRegion2	7646
CNVR70	871	6	107004191	107039849	loss	3873	18942762	122134922	Carcass_length	region1inRegion2	35658
CNVR69	862	6	95466297	95473943	gain	5226	19536155	157765593	Teat_number	region1inRegion2	7646
CNVR70	871	6	107004191	107039849	loss	5226	19536155	157765593	Teat_number	region1inRegion2	35658
CNVR71	917	6	151994674	152015484	gain	5226	19536155	157765593	Teat_number	region1inRegion2	20810
CNVR69	862	6	95466297	95473943	gain	38091	28253996	152260387	Shear_force	region1inRegion2	7646
CNVR70	871	6	107004191	107039849	loss	38091	28253996	152260387	Shear_force	region1inRegion2	35658
CNVR71	917	6	151994674	152015484	gain	38091	28253996	152260387	Shear_force	region1inRegion2	20810
CNVR69	862	6	95466297	95473943	gain	148	29239143	136971287	Average_backfat_thickness	region1inRegion2	7646
CNVR70	871	6	107004191	107039849	loss	148	29239143	136971287	Average_backfat_thickness	region1inRegion2	35658
CNVR69	862	6	95466297	95473943	gain	149	29239143	136971287	Average_backfat_thickness	region1inRegion2	7646
CNVR70	871	6	107004191	107039849	loss	149	29239143	136971287	Average_backfat_thickness	region1inRegion2	35658
CNVR69	862	6	95466297	95473943	gain	150	29239143	136971287	Intramuscular_fat_content	region1inRegion2	7646
CNVR70	871	6	107004191	107039849	loss	150	29239143	136971287	Intramuscular_fat_content	region1inRegion2	35658
CNVR69	862	6	95466297	95473943	gain	151	29239143	136971287	Intramuscular_fat_content	region1inRegion2	7646
CNVR70	871	6	107004191	107039849	loss	151	29239143	136971287	Intramuscular_fat_content	region1inRegion2	35658
CNVR69	862	6	95466297	95473943	gain	688	29979924	117131464	Smell_intensity	region1inRegion2	7646
CNVR70	871	6	107004191	107039849	loss	688	29979924	117131464	Smell_intensity	region1inRegion2	35658
CNVR69	862	6	95466297	95473943	gain	691	29979924	117131464	tenderness_score	region1inRegion2	7646
CNVR70	871	6	107004191	107039849	loss	691	29979924	117131464	tenderness_score	region1inRegion2	35658
CNVR69	862	6	95466297	95473943	gain	4167	30086351	127687961	Backfat_between_3rd_and_4th_last_ribs	region1inRegion2	7646
CNVR70	871	6	107004191	107039849	loss	4167	30086351	127687961	Backfat_between_3rd_and_4th_last_ribs	region1inRegion2	35658
CNVR69	862	6	95466297	95473943	gain	183	30938918	100619284	body_weight_(13_weeks)	region1inRegion2	7646
CNVR69	862	6	95466297	95473943	gain	184	30938918	115392938	body_weight_(17_weeks)	region1inRegion2	7646
CNVR70	871	6	107004191	107039849	loss	184	30938918	115392938	body_weight_(17_weeks)	region1inRegion2	35658
CNVR69	862	6	95466297	95473943	gain	4169	30978589	130369909	Intramuscular_fat_content	region1inRegion2	7646
CNVR70	871	6	107004191	107039849	loss	4169	30978589	130369909	Intramuscular_fat_content	region1inRegion2	35658
CNVR69	862	6	95466297	95473943	gain	5911	34464154	129740986	Time_spent_feeding	region1inRegion2	7646
CNVR70	871	6	107004191	107039849	loss	5911	34464154	129740986	Time_spent_feeding	region1inRegion2	35658
CNVR69	862	6	95466297	95473943	gain	6347	34464154	129740986	Alkaline_phosphatase_activity	region1inRegion2	7646
CNVR70	871	6	107004191	107039849	loss	6347	34464154	129740986	Alkaline_phosphatase_activity	region1inRegion2	35658
CNVR69	862	6	95466297	95473943	gain	6376	34464154	129740986	Calcium_level	region1inRegion2	7646
CNVR70	871	6	107004191	107039849	loss	6376	34464154	129740986	Calcium_level	region1inRegion2	35658
CNVR69	862	6	95466297	95473943	gain	7499	34464154	129740986	Platelet_count	region1inRegion2	7646
CNVR70	871	6	107004191	107039849	loss	7499	34464154	129740986	Platelet_count	region1inRegion2	35658
CNVR69	862	6	95466297	95473943	gain	7500	34464154	129740986	Platelet_count	region1inRegion2	7646
CNVR70	871	6	107004191	107039849	loss	7500	34464154	129740986	Platelet_count	region1inRegion2	35658
CNVR69	862	6	95466297	95473943	gain	29696	44290269	146365886	Body_weight_(20_weeks)	region1inRegion2	7646
CNVR70	871	6	107004191	107039849	loss	29696	44290269	146365886	Body_weight_(20_weeks)	region1inRegion2	35658
CNVR69	862	6	95466297	95473943	gain	29697	44290269	146365886	Carcass_weight_(hot)	region1inRegion2	7646
CNVR70	871	6	107004191	107039849	loss	29697	44290269	146365886	Carcass_weight_(hot)	region1inRegion2	35658
CNVR69	862	6	95466297	95473943	gain	4219	44290269	146365886	subjective_boar_flavor_in_lean	region1inRegion2	7646
CNVR70	871	6	107004191	107039849	loss	4219	44290269	146365886	subjective_boar_flavor_in_lean	region1inRegion2	35658
CNVR69	862	6	95466297	95473943	gain	7476	61924724	117131464	Nonfunctional_nipples	region1inRegion2	7646
CNVR70	871	6	107004191	107039849	loss	7476	61924724	117131464	Nonfunctional_nipples	region1inRegion2	35658
CNVR69	862	6	95466297	95473943	gain	17599	66465212	117131464	Interleukin_2_level	region1inRegion2	7646
CNVR70	871	6	107004191	107039849	loss	17599	66465212	117131464	Interleukin_2_level	region1inRegion2	35658
CNVR69	862	6	95466297	95473943	gain	17718	66465212	117131464	Cholesterol_level	region1inRegion2	7646
CNVR70	871	6	107004191	107039849	loss	17718	66465212	117131464	Cholesterol_level	region1inRegion2	35658
CNVR69	862	6	95466297	95473943	gain	9610	66465212	117131464	Hemolytic_complement_activity_(classical_pathway)	region1inRegion2	7646
CNVR70	871	6	107004191	107039849	loss	9610	66465212	117131464	Hemolytic_complement_activity_(classical_pathway)	region1inRegion2	35658
CNVR69	862	6	95466297	95473943	gain	4168	71886584	134073790	Loin_muscle_area	region1inRegion2	7646
CNVR70	871	6	107004191	107039849	loss	4168	71886584	134073790	Loin_muscle_area	region1inRegion2	35658
CNVR69	862	6	95466297	95473943	gain	2870	73298768	137240589	Shoulder_meat_weight	region1inRegion2	7646
CNVR70	871	6	107004191	107039849	loss	2870	73298768	137240589	Shoulder_meat_weight	region1inRegion2	35658
CNVR69	862	6	95466297	95473943	gain	673	73934339	95802584	Loin_muscle_depth	region1inRegion2	7646
CNVR69	862	6	95466297	95473943	gain	17547	74133109	108169894	Teat_number	region1inRegion2	7646
CNVR70	871	6	107004191	107039849	loss	17547	74133109	108169894	Teat_number	region1inRegion2	35658
CNVR69	862	6	95466297	95473943	gain	3674	74531339	117131464	Average_daily_gain	region1inRegion2	7646
CNVR70	871	6	107004191	107039849	loss	3674	74531339	117131464	Average_daily_gain	region1inRegion2	35658
CNVR69	862	6	95466297	95473943	gain	3675	74531339	117131464	backfat_at_last_rib	region1inRegion2	7646
CNVR70	871	6	107004191	107039849	loss	3675	74531339	117131464	backfat_at_last_rib	region1inRegion2	35658
CNVR69	862	6	95466297	95473943	gain	3696	74531339	117131464	Carcass_weight_(hot)	region1inRegion2	7646
CNVR70	871	6	107004191	107039849	loss	3696	74531339	117131464	Carcass_weight_(hot)	region1inRegion2	35658
CNVR69	862	6	95466297	95473943	gain	3697	74531339	117131464	Carcass_length	region1inRegion2	7646
CNVR70	871	6	107004191	107039849	loss	3697	74531339	117131464	Carcass_length	region1inRegion2	35658
CNVR69	862	6	95466297	95473943	gain	3698	74531339	117131464	PH_for_Longissmus_dorsi	region1inRegion2	7646
CNVR70	871	6	107004191	107039849	loss	3698	74531339	117131464	PH_for_Longissmus_dorsi	region1inRegion2	35658
CNVR69	862	6	95466297	95473943	gain	3699	74531339	117131464	pH_for_Semimembranosus	region1inRegion2	7646
CNVR70	871	6	107004191	107039849	loss	3699	74531339	117131464	pH_for_Semimembranosus	region1inRegion2	35658
CNVR69	862	6	95466297	95473943	gain	4131	74531339	117131464	Average_daily_gain	region1inRegion2	7646
CNVR70	871	6	107004191	107039849	loss	4131	74531339	117131464	Average_daily_gain	region1inRegion2	35658
CNVR69	862	6	95466297	95473943	gain	4132	74531339	117131464	Average_daily_gain	region1inRegion2	7646
CNVR70	871	6	107004191	107039849	loss	4132	74531339	117131464	Average_daily_gain	region1inRegion2	35658
CNVR69	862	6	95466297	95473943	gain	4133	74531339	117131464	Carcass_length	region1inRegion2	7646
CNVR70	871	6	107004191	107039849	loss	4133	74531339	117131464	Carcass_length	region1inRegion2	35658
CNVR69	862	6	95466297	95473943	gain	4134	74531339	117131464	Lean_meat_percentage	region1inRegion2	7646
CNVR70	871	6	107004191	107039849	loss	4134	74531339	117131464	Lean_meat_percentage	region1inRegion2	35658
CNVR69	862	6	95466297	95473943	gain	4135	74531339	117131464	PH_for_Longissmus_dorsi	region1inRegion2	7646
CNVR70	871	6	107004191	107039849	loss	4135	74531339	117131464	PH_for_Longissmus_dorsi	region1inRegion2	35658
CNVR69	862	6	95466297	95473943	gain	4136	74531339	117131464	PH_for_Longissmus_dorsi	region1inRegion2	7646
CNVR70	871	6	107004191	107039849	loss	4136	74531339	117131464	PH_for_Longissmus_dorsi	region1inRegion2	35658
CNVR69	862	6	95466297	95473943	gain	4137	74531339	117131464	pH_for_Semimembranosus	region1inRegion2	7646
CNVR70	871	6	107004191	107039849	loss	4137	74531339	117131464	pH_for_Semimembranosus	region1inRegion2	35658
CNVR69	862	6	95466297	95473943	gain	4138	74531339	117131464	pH_for_Semimembranosus	region1inRegion2	7646
CNVR70	871	6	107004191	107039849	loss	4138	74531339	117131464	pH_for_Semimembranosus	region1inRegion2	35658
CNVR69	862	6	95466297	95473943	gain	4139	74531339	117131464	PH_for_Longissmus_dorsi	region1inRegion2	7646
CNVR70	871	6	107004191	107039849	loss	4139	74531339	117131464	PH_for_Longissmus_dorsi	region1inRegion2	35658
CNVR69	862	6	95466297	95473943	gain	4140	74531339	117131464	Conductivity_45_minutes_post-mortem	region1inRegion2	7646
CNVR70	871	6	107004191	107039849	loss	4140	74531339	117131464	Conductivity_45_minutes_post-mortem	region1inRegion2	35658

CNVR69	862	6	95466297	95473943	gain	4141	74531339	117131464	Conductivity_24_hours_postmortem_(loin)	region1inRegion2	7646
CNVR70	871	6	107004191	107039849	loss	4141	74531339	117131464	Conductivity_24_hours_postmortem_(loin)	region1inRegion2	35658
CNVR69	862	6	95466297	95473943	gain	4193	74531339	117131464	Conductivity_24_hours_postmortem_(loin)	region1inRegion2	7646
CNVR70	871	6	107004191	107039849	loss	4193	74531339	117131464	Conductivity_24_hours_postmortem_(loin)	region1inRegion2	35658
CNVR69	862	6	95466297	95473943	gain	4194	74531339	117131464	Conductivity_45_minutes_post-mortem	region1inRegion2	7646
CNVR70	871	6	107004191	107039849	loss	4194	74531339	117131464	Conductivity_45_minutes_post-mortem	region1inRegion2	35658
CNVR69	862	6	95466297	95473943	gain	10620	74531339	129740986	Litter_size	region1inRegion2	7646
CNVR70	871	6	107004191	107039849	loss	10620	74531339	129740986	Litter_size	region1inRegion2	35658
CNVR69	862	6	95466297	95473943	gain	1032	74661313	117131464	backfat_at_last_rib	region1inRegion2	7646
CNVR70	871	6	107004191	107039849	loss	1032	74661313	117131464	backfat_at_last_rib	region1inRegion2	35658
CNVR69	862	6	95466297	95473943	gain	1033	74661313	117131464	Abdominal_fat_weight	region1inRegion2	7646
CNVR70	871	6	107004191	107039849	loss	1033	74661313	117131464	Abdominal_fat_weight	region1inRegion2	35658
CNVR69	862	6	95466297	95473943	gain	1037	74661313	117131464	Body_weight_(3_weeks)	region1inRegion2	7646
CNVR70	871	6	107004191	107039849	loss	1037	74661313	117131464	Body_weight_(3_weeks)	region1inRegion2	35658
CNVR69	862	6	95466297	95473943	gain	674	75960946	98288152	Loin_muscle_area	region1inRegion2	7646
CNVR69	862	6	95466297	95473943	gain	3950	78052071	129740986	Ham_fat_thickness	region1inRegion2	7646
CNVR70	871	6	107004191	107039849	loss	3950	78052071	129740986	Ham_fat_thickness	region1inRegion2	35658
CNVR69	862	6	95466297	95473943	gain	152	78052071	136971287	Loin_muscle_area	region1inRegion2	7646
CNVR70	871	6	107004191	107039849	loss	152	78052071	136971287	Loin_muscle_area	region1inRegion2	35658
CNVR69	862	6	95466297	95473943	gain	153	78052071	136971287	Loin_muscle_area	region1inRegion2	7646
CNVR70	871	6	107004191	107039849	loss	153	78052071	136971287	Loin_muscle_area	region1inRegion2	35658
CNVR69	862	6	95466297	95473943	gain	21326	79653393	104254876	White_blood_cell_counts	region1inRegion2	7646
CNVR69	862	6	95466297	95473943	gain	12290	79653393	117131464	Mean_corpuscular_hemoglobin_content	region1inRegion2	7646
CNVR70	871	6	107004191	107039849	loss	12290	79653393	117131464	Mean_corpuscular_hemoglobin_content	region1inRegion2	35658
CNVR69	862	6	95466297	95473943	gain	21706	79751436	98288152	Muscle_moisture_percentage	region1inRegion2	7646
CNVR69	862	6	95466297	95473943	gain	156	83821055	129740986	post-stress_mitogen_induced_IL-2_activity	region1inRegion2	7646
CNVR70	871	6	107004191	107039849	loss	156	83821055	129740986	post-stress_mitogen_induced_IL-2_activity	region1inRegion2	35658
CNVR69	862	6	95466297	95473943	gain	15050	83821055	146365886	Hemoglobin	region1inRegion2	7646
CNVR70	871	6	107004191	107039849	loss	15050	83821055	146365886	Hemoglobin	region1inRegion2	35658
CNVR69	862	6	95466297	95473943	gain	15051	83821055	146365886	hematocrit	region1inRegion2	7646
CNVR70	871	6	107004191	107039849	loss	15051	83821055	146365886	hematocrit	region1inRegion2	35658
CNVR69	862	6	95466297	95473943	gain	4579	85950097	129712784	Body_weight_(birth)	region1inRegion2	7646
CNVR70	871	6	107004191	107039849	loss	4579	85950097	129712784	Body_weight_(birth)	region1inRegion2	35658
CNVR69	862	6	95466297	95473943	gain	15879	85992952	146365886	Oleic_acid_content	region1inRegion2	7646
CNVR70	871	6	107004191	107039849	loss	15879	85992952	146365886	Oleic_acid_content	region1inRegion2	35658
CNVR69	862	6	95466297	95473943	gain	37542	91030131	117131464	CSFV_antibody_level	region1inRegion2	7646
CNVR70	871	6	107004191	107039849	loss	37542	91030131	117131464	CSFV_antibody_level	region1inRegion2	35658
CNVR69	862	6	95466297	95473943	gain	12057	91030131	146365886	Muscle_moisture_percentage	region1inRegion2	7646
CNVR70	871	6	107004191	107039849	loss	12057	91030131	146365886	Muscle_moisture_percentage	region1inRegion2	35658
CNVR69	862	6	95466297	95473943	gain	12058	91030131	146365886	muscle_protein_percentage	region1inRegion2	7646
CNVR70	871	6	107004191	107039849	loss	12058	91030131	146365886	muscle_protein_percentage	region1inRegion2	35658
CNVR69	862	6	95466297	95473943	gain	12059	91030131	146365886	Intramuscular_fat_content	region1inRegion2	7646
CNVR70	871	6	107004191	107039849	loss	12059	91030131	146365886	Intramuscular_fat_content	region1inRegion2	35658
CNVR69	862	6	95466297	95473943	gain	12060	91030131	146365886	Cholesterol_level_in_meat	region1inRegion2	7646
CNVR70	871	6	107004191	107039849	loss	12060	91030131	146365886	Cholesterol_level_in_meat	region1inRegion2	35658
CNVR69	862	6	95466297	95473943	gain	12061	91030131	146365886	Flavor_score	region1inRegion2	7646
CNVR70	871	6	107004191	107039849	loss	12061	91030131	146365886	Flavor_score	region1inRegion2	35658
CNVR69	862	6	95466297	95473943	gain	15046	91030131	146365886	Red_blood_cell_count	region1inRegion2	7646
CNVR70	871	6	107004191	107039849	loss	15046	91030131	146365886	Red_blood_cell_count	region1inRegion2	35658
CNVR69	862	6	95466297	95473943	gain	4214	91030131	146365886	skatole,_laboratory	region1inRegion2	7646
CNVR70	871	6	107004191	107039849	loss	4214	91030131	146365886	skatole,_laboratory	region1inRegion2	35658
CNVR69	862	6	95466297	95473943	gain	4218	91030131	146365886	skatole,_sensory_panel	region1inRegion2	7646
CNVR70	871	6	107004191	107039849	loss	4218	91030131	146365886	skatole,_sensory_panel	region1inRegion2	35658
CNVR69	862	6	95466297	95473943	gain	8820	91030131	146365886	Age_at_puberty	region1inRegion2	7646
CNVR70	871	6	107004191	107039849	loss	8820	91030131	146365886	Age_at_puberty	region1inRegion2	35658
CNVR69	862	6	95466297	95473943	gain	16851	91314414	131621758	Average_daily_gain	region1inRegion2	7646
CNVR70	871	6	107004191	107039849	loss	16851	91314414	131621758	Average_daily_gain	region1inRegion2	35658
CNVR69	862	6	95466297	95473943	gain	182	92257476	138477175	body_weight_(10_weeks)	region1inRegion2	7646
CNVR70	871	6	107004191	107039849	loss	182	92257476	138477175	body_weight_(10_weeks)	region1inRegion2	35658
CNVR69	862	6	95466297	95473943	gain	17780	94382869	137240589	backfat_at_last_rib	region1inRegion2	7646
CNVR70	871	6	107004191	107039849	loss	17780	94382869	137240589	backfat_at_last_rib	region1inRegion2	35658
CNVR69	862	6	95466297	95473943	gain	17781	94382869	137240589	backfat_at_last_rib	region1inRegion2	7646
CNVR70	871	6	107004191	107039849	loss	17781	94382869	137240589	backfat_at_last_rib	region1inRegion2	35658
CNVR69	862	6	95466297	95473943	gain	16920	94382869	146365886	Fat_weight_(total)	region1inRegion2	7646
CNVR70	871	6	107004191	107039849	loss	16920	94382869	146365886	Fat_weight_(total)	region1inRegion2	35658
CNVR69	862	6	95466297	95473943	gain	16921	94382869	146365886	Backfat_at_tenth_rib	region1inRegion2	7646
CNVR70	871	6	107004191	107039849	loss	16921	94382869	146365886	Backfat_at_tenth_rib	region1inRegion2	35658
CNVR69	862	6	95466297	95473943	gain	16922	94382869	146365886	Backfat_at_tenth_rib	region1inRegion2	7646
CNVR70	871	6	107004191	107039849	loss	16922	94382869	146365886	Backfat_at_tenth_rib	region1inRegion2	35658
CNVR69	862	6	95466297	95473943	gain	16923	94382869	146365886	Empty_body_lipid_content	region1inRegion2	7646
CNVR70	871	6	107004191	107039849	loss	16923	94382869	146365886	Empty_body_lipid_content	region1inRegion2	35658
CNVR69	862	6	95466297	95473943	gain	18630	94382869	146365886	Fat_area_percentage_in_carcass	region1inRegion2	7646
CNVR70	871	6	107004191	107039849	loss	18630	94382869	146365886	Fat_area_percentage_in_carcass	region1inRegion2	35658
CNVR69	862	6	95466297	95473943	gain	18635	94382869	146365886	Fat_area_percentage_in_carcass	region1inRegion2	7646
CNVR70	871	6	107004191	107039849	loss	18635	94382869	146365886	Fat_area_percentage_in_carcass	region1inRegion2	35658
CNVR69	862	6	95466297	95473943	gain	18637	94382869	146365886	Fat_area_percentage_in_carcass	region1inRegion2	7646
CNVR70	871	6	107004191	107039849	loss	18637	94382869	146365886	Fat_area_percentage_in_carcass	region1inRegion2	35658
CNVR69	862	6	95466297	95473943	gain	18642	94382869	146365886	Fat_area_percentage_in_carcass	region1inRegion2	7646
CNVR70	871	6	107004191	107039849	loss	18642	94382869	146365886	Fat_area_percentage_in_carcass	region1inRegion2	35658
CNVR69	862	6	95466297	95473943	gain	18643	94382869	146365886	Leptin_level	region1inRegion2	7646
CNVR70	871	6	107004191	107039849	loss	18643	94382869	146365886	Leptin_level	region1inRegion2	35658
CNVR69	862	6	95466297	95473943	gain	21375	94382869	146365886	Belly_weight	region1inRegion2	7646
CNVR70	871	6	107004191	107039849	loss	21375	94382869	146365886	Belly_weight	region1inRegion2	35658
CNVR69	862	6	95466297	95473943	gain	21376	94382869	146365886	Ham_weight	region1inRegion2	7646
CNVR70	871	6	107004191	107039849	loss	21376	94382869	146365886	Ham_weight	region1inRegion2	35658
CNVR69	862	6	95466297	95473943	gain	21377	94382869	146365886	backfat_at_last_rib	region1inRegion2	7646
CNVR70	871	6	107004191	107039849	loss	21377	94382869	146365886	backfat_at_last_rib	region1inRegion2	35658
CNVR69	862	6	95466297	95473943	gain	21378	94382869	146365886	Loin_muscle_area	region1inRegion2	7646
CNVR70	871	6	107004191	107039849	loss	21378	94382869	146365886	Loin_muscle_area	region1inRegion2	35658
CNVR69	862	6	95466297	95473943	gain	21379	94382869	146365886	muscle_protein_percentage	region1inRegion2	7646
CNVR70	871	6	107004191	107039849	loss	21379	94382869	146365886	muscle_protein_percentage	region1inRegion2	35658
CNVR69	862	6	95466297	95473943	gain	21380	94382869	146365886	Carcass_weight_(hot)	region1inRegion2	7646
CNVR70	871	6	107004191	107039849	loss	21380	94382869	146365886	Carcass_weight_(hot)	region1inRegion2	35658
CNVR69	862	6	95466297	95473943	gain	21381	94382869	146365886	Carcass_temperature_(24_hr_post-mortem)_	region1inRegion2	7646
CNVR70	871	6	107004191	107039849	loss	21381	94382869	146365886	Carcass_temperature_(24_hr_post-mortem)_	region1inRegion2	35658
CNVR69	862	6	95466297	95473943	gain	21724	94382869	146365886	Ear_erectness	region1inRegion2	7646
CNVR70	871	6	107004191	107039849	loss	21724	94382869	146365886	Ear_erectness	region1inRegion2	35658
CNVR69	862	6	95466297	95473943	gain	2854	94382869	146365886	Intramuscular_fat_content	region1inRegion2	7646
CNVR70	871	6	107004191	107039849	loss	2854	94382869	146365886	Intramuscular_fat_content	region1inRegion2	35658
CNVR69	862	6	95466297	95473943	gain	2857	94382869	146365886	External_fat_on_loin	region1inRegion2	7646
CNVR70	871	6	107004191	107039849	loss	2857	94382869	146365886	External_fat_on_loin	region1inRegion2	35658
CNVR69	862	6	95466297	95473943	gain	2859	94382869	146365886	Ham_weight	region1inRegion2	7646
CNVR70	871	6	107004191	107039849	loss	2859	94382869	146365886	Ham_weight	region1inRegion2	35658
CNVR69	862	6	95466297	95473943	gain	2869	94382869	146365886	Shoulder_weight	region1inRegion2	7646
CNVR70	871	6	107004191	107039849	loss	2869	94382869	146365886	Shoulder_weight	region1inRegion2	35658
CNVR69	862	6	95466297	95473943	gain	2879	94382869	146365886	Daily_feed_intake	region1inRegion2	7646
CNVR70	871	6	107004191	107039849	loss	2879	94382869	146365886	Daily_feed_intake	region1inRegion2	35658
CNVR69	862	6	95466297	95473943	gain	2880	94382869	146365886	Daily_feed_intake	region1inRegion2	7646
CNVR70	871	6	107004191	107039849	loss	2880	94382869	146365886	Daily_feed_intake	region1inRegion2	35658
CNVR69	862	6	95466297	95473943	gain	3188	94382869	146365886	Fat_weight_(total)	region1inRegion2	7646
CNVR70	871	6	107004191	107039849	loss	3188	94382869	146365886	Fat_weight_(total)	region1inRegion2	35658
CNVR69	862	6	95466297	95473943	gain	3195	94382869	146365886	Backfat_at_tenth_rib	region1inRegion2	7646
CNVR70	871	6	107004191	107039849	loss	3195	94382869	146365886	Backfat_at_tenth_rib	region1inRegion2	35658
CNVR69	862	6	95466297	95473943	gain	3196	94382869	146365886	Backfat_at_tenth_rib	region1inRegion2	7646
CNVR70	871	6	107004191	107039849	loss	3196	94382869	146365886	Backfat_at_tenth_rib	region1inRegion2	35658
CNVR69	862	6	95466297	95473943	gain	3197	94382869	146365886	backfat_at_last_rib	region1inRegion2	7646
CNVR70	871	6	107004191	107039849	loss	3197	94382869	146365886	backfat_at_last_rib	region1inRegion2	35658
CNVR69	862	6	95466297	95473943	gain	3198	94382869	146365886	backfat_at_last_rib	region1inRegion2	7646
CNVR70	871	6	107004191	107039849	loss	3198	94382869	146365886	backfat_at_last_rib	region1inRegion2	35658
CNVR69	862	6	95466297	95473943	gain	3199	94382869	146365886	backfat_at_last_rib	region1inRegion2	7646
CNVR70	871	6	107004191	107039849	loss	3199	94382869	146365886	backfat_at_last_rib	region1inRegion2	35658
CNVR69	862	6	95466297	95473943	gain	3200	94382869	146365886	Backfat_at_tenth_rib	region1inRegion2	7646

CNVR70	871	6	107004191	107039849	loss	3200	94382869	146365886	Backfat_at_tenth_rib	region1inRegion2	35658
CNVR69	862	6	95466297	95473943	gain	3201	94382869	146365886	backfat_at_last_rib	region1inRegion2	7646
CNVR70	871	6	107004191	107039849	loss	3201	94382869	146365886	backfat_at_last_rib	region1inRegion2	35658
CNVR69	862	6	95466297	95473943	gain	3202	94382869	146365886	Backfat_at_tenth_rib	region1inRegion2	7646
CNVR70	871	6	107004191	107039849	loss	3202	94382869	146365886	Backfat_at_tenth_rib	region1inRegion2	35658
CNVR69	862	6	95466297	95473943	gain	3203	94382869	146365886	Backfat_at_tenth_rib	region1inRegion2	7646
CNVR70	871	6	107004191	107039849	loss	3203	94382869	146365886	Backfat_at_tenth_rib	region1inRegion2	35658
CNVR69	862	6	95466297	95473943	gain	3204	94382869	146365886	Loin_muscle_area	region1inRegion2	7646
CNVR70	871	6	107004191	107039849	loss	3204	94382869	146365886	Loin_muscle_area	region1inRegion2	35658
CNVR69	862	6	95466297	95473943	gain	3244	94382869	146365886	Carcass_length	region1inRegion2	7646
CNVR70	871	6	107004191	107039849	loss	3244	94382869	146365886	Carcass_length	region1inRegion2	35658
CNVR69	862	6	95466297	95473943	gain	3245	94382869	146365886	Loin_weight	region1inRegion2	7646
CNVR70	871	6	107004191	107039849	loss	3245	94382869	146365886	Loin_weight	region1inRegion2	35658
CNVR69	862	6	95466297	95473943	gain	3246	94382869	146365886	backfat_at_last_rib	region1inRegion2	7646
CNVR70	871	6	107004191	107039849	loss	3246	94382869	146365886	backfat_at_last_rib	region1inRegion2	35658
CNVR69	862	6	95466297	95473943	gain	3247	94382869	146365886	muscle_protein_percentage	region1inRegion2	7646
CNVR70	871	6	107004191	107039849	loss	3247	94382869	146365886	muscle_protein_percentage	region1inRegion2	35658
CNVR69	862	6	95466297	95473943	gain	3248	94382869	146365886	Spareribs_weight	region1inRegion2	7646
CNVR70	871	6	107004191	107039849	loss	3248	94382869	146365886	Spareribs_weight	region1inRegion2	35658
CNVR69	862	6	95466297	95473943	gain	3249	94382869	146365886	Loin_muscle_area	region1inRegion2	7646
CNVR70	871	6	107004191	107039849	loss	3249	94382869	146365886	Loin_muscle_area	region1inRegion2	35658
CNVR69	862	6	95466297	95473943	gain	3317	94382869	146365886	Empty_body_lipid_content	region1inRegion2	7646
CNVR70	871	6	107004191	107039849	loss	3317	94382869	146365886	Empty_body_lipid_content	region1inRegion2	35658
CNVR69	862	6	95466297	95473943	gain	3649	94382869	146365886	backfat_at_last_rib	region1inRegion2	7646
CNVR70	871	6	107004191	107039849	loss	3649	94382869	146365886	backfat_at_last_rib	region1inRegion2	35658
CNVR69	862	6	95466297	95473943	gain	3650	94382869	146365886	Backfat_linear_at_last_rib	region1inRegion2	7646
CNVR70	871	6	107004191	107039849	loss	3650	94382869	146365886	Backfat_linear_at_last_rib	region1inRegion2	35658
CNVR69	862	6	95466297	95473943	gain	3651	94382869	146365886	Backfat_at_tenth_rib	region1inRegion2	7646
CNVR70	871	6	107004191	107039849	loss	3651	94382869	146365886	Backfat_at_tenth_rib	region1inRegion2	35658
CNVR69	862	6	95466297	95473943	gain	3652	94382869	146365886	Backfat_linear_at_tenth_rib	region1inRegion2	7646
CNVR70	871	6	107004191	107039849	loss	3652	94382869	146365886	Backfat_linear_at_tenth_rib	region1inRegion2	35658
CNVR69	862	6	95466297	95473943	gain	135626	94962183	95772705	Feed_conversion_ratio	region1inRegion2	7646
CNVR69	862	6	95466297	95473943	gain	66026	95437656	115984089	Fat_area_percentage_in_carcass	region1inRegion2	7646
CNVR70	871	6	107004191	107039849	loss	66026	95437656	115984089	Fat_area_percentage_in_carcass	region1inRegion2	35658
CNVR70	871	6	107004191	107039849	loss	66025	96195635	115984089	Fat_area_percentage_in_carcass	region1inRegion2	35658
CNVR70	871	6	107004191	107039849	loss	66024	97895295	112143612	Average_backfat_thickness	region1inRegion2	35658
CNVR70	871	6	107004191	107039849	loss	66027	97895295	112143612	Fat_area_percentage_in_carcass	region1inRegion2	35658
CNVR70	871	6	107004191	107039849	loss	66031	97895295	113072600	Fat_area_percentage_in_carcass	region1inRegion2	35658
CNVR70	871	6	107004191	107039849	loss	66028	97895295	115984089	Fat_area_percentage_in_carcass	region1inRegion2	35658
CNVR70	871	6	107004191	107039849	loss	66032	97895295	124113659	Leptin_level	region1inRegion2	35658
CNVR70	871	6	107004191	107039849	loss	181	98288152	135394314	Body_weight_(3_weeks)	region1inRegion2	35658
CNVR70	871	6	107004191	107039849	loss	66029	100040975	109546295	Fat_area_percentage_in_carcass	region1inRegion2	35658
CNVR70	871	6	107004191	107039849	loss	66030	100040975	117691313	Fat_area_percentage_in_carcass	region1inRegion2	35658
CNVR70	871	6	107004191	107039849	loss	17554	100619284	123602825	Body_weight_(birth)	region1inRegion2	35658
CNVR70	871	6	107004191	107039849	loss	583	105864781	134139072	Teat_number	region1inRegion2	35658
CNVR70	871	6	107004191	107039849	loss	64637	106626089	107447105	Thoracolumbar_vertebra_number	region1inRegion2	35658
CNVR70	871	6	107004191	107039849	loss	64638	106626089	107447105	Thoracic_vertebra_number	region1inRegion2	35658
CNVR71	917	6	151994674	152015484	gain	314	117131464	152260387	Intramuscular_fat_content	region1inRegion2	20810
CNVR69	862	6	95466297	95473943	gain	18156	135140880	25859176	Gestation_length	region2inRegion1	-109281704
CNVR70	871	6	107004191	107039849	loss	18156	135140880	25859176	Gestation_length	region2inRegion1	-109281704
CNVR69	862	6	95466297	95473943	gain	18158	135140880	73007048	Gestation_length	region2inRegion1	-62133832
CNVR70	871	6	107004191	107039849	loss	18158	135140880	73007048	Gestation_length	region2inRegion1	-62133832
CNVR71	917	6	151994674	152015484	gain	15071	136849116	152260387	Cholesterol_level	region1inRegion2	20810
CNVR71	917	6	151994674	152015484	gain	18657	145259351	153129074	indole,_laboratory	region1inRegion2	20810
CNVR71	917	6	151994674	152015484	gain	21708	145842773	155199218	muscle_protein_percentage	region1inRegion2	20810
CNVR71	917	6	151994674	152015484	gain	21709	145842773	155199218	Loin_muscle_area	region1inRegion2	20810
CNVR71	917	6	151994674	152015484	gain	21710	145842773	155199218	Loin_weight	region1inRegion2	20810
CNVR71	917	6	151994674	152015484	gain	3250	146365886	152260387	Muscle_moisture_percentage	region1inRegion2	20810
CNVR71	917	6	151994674	152015484	gain	3308	146365886	152260387	Carcass_temperature_(24_hr_post-mortem)_	region1inRegion2	20810
CNVR71	917	6	151994674	152015484	gain	8941	146365886	152260387	Gait_score_(hind)	region1inRegion2	20810
CNVR71	917	6	151994674	152015484	gain	3840	146365886	155890823	muscle_protein_percentage	region1inRegion2	20810
CNVR71	917	6	151994674	152015484	gain	3852	146365886	155890823	Muscle_moisture_percentage	region1inRegion2	20810
CNVR71	917	6	151994674	152015484	gain	3951	146365886	155890823	Semimembranosus_angle	region1inRegion2	20810
CNVR72	923	7	10444634	10478517	gain	7536	48748	11625414	Total_number_born_alive	region1inRegion2	33883
CNVR72	923	7	10444634	10478517	gain	21864	48748	33757808	Meat_color_score	region1inRegion2	33883
CNVR73	934	7	22207228	22236111	gain	21864	48748	33757808	Meat_color_score	region1inRegion2	28883
CNVR74	938	7	23307667	23658110	loss-gain	21864	48748	33757808	Meat_color_score	region1inRegion2	350443
CNVR75	939	7	23481211	23658110	loss-gain	21864	48748	33757808	Meat_color_score	region1inRegion2	176899
CNVR76	942	7	24648309	24653067	loss-gain	21864	48748	33757808	Meat_color_score	region1inRegion2	4758
CNVR77	943	7	24735665	24740397	gain	21864	48748	33757808	Meat_color_score	region1inRegion2	4732
CNVR78	944	7	25359190	25399904	gain	21864	48748	33757808	Meat_color_score	region1inRegion2	40714
CNVR79	945	7	25488796	25557156	gain	21864	48748	33757808	Meat_color_score	region1inRegion2	68360
CNVR80	946	7	25785853	25888939	gain	21864	48748	33757808	Meat_color_score	region1inRegion2	103086
CNVR81	949	7	26267062	26297673	gain	21864	48748	33757808	Meat_color_score	region1inRegion2	30611
CNVR82	951	7	26600964	26608495	gain	21864	48748	33757808	Meat_color_score	region1inRegion2	7531
CNVR83	957	7	28525787	28565312	gain	21864	48748	33757808	Meat_color_score	region1inRegion2	39525
CNVR72	923	7	10444634	10478517	gain	3756	48748	120853004	Average_daily_gain	region1inRegion2	33883
CNVR73	934	7	22207228	22236111	gain	3756	48748	120853004	Average_daily_gain	region1inRegion2	28883
CNVR74	938	7	23307667	23658110	loss-gain	3756	48748	120853004	Average_daily_gain	region1inRegion2	350443
CNVR75	939	7	23481211	23658110	loss-gain	3756	48748	120853004	Average_daily_gain	region1inRegion2	176899
CNVR76	942	7	24648309	24653067	loss-gain	3756	48748	120853004	Average_daily_gain	region1inRegion2	4758
CNVR77	943	7	24735665	24740397	gain	3756	48748	120853004	Average_daily_gain	region1inRegion2	4732
CNVR78	944	7	25359190	25399904	gain	3756	48748	120853004	Average_daily_gain	region1inRegion2	40714
CNVR79	945	7	25488796	25557156	gain	3756	48748	120853004	Average_daily_gain	region1inRegion2	68360
CNVR80	946	7	25785853	25888939	gain	3756	48748	120853004	Average_daily_gain	region1inRegion2	103086
CNVR81	949	7	26267062	26297673	gain	3756	48748	120853004	Average_daily_gain	region1inRegion2	30611
CNVR82	951	7	26600964	26608495	gain	3756	48748	120853004	Average_daily_gain	region1inRegion2	7531
CNVR83	957	7	28525787	28565312	gain	3756	48748	120853004	Average_daily_gain	region1inRegion2	39525
CNVR84	972	7	58579811	58584542	gain	3756	48748	120853004	Average_daily_gain	region1inRegion2	4731
CNVR85	975	7	59278633	59284219	loss	3756	48748	120853004	Average_daily_gain	region1inRegion2	5586
CNVR86	977	7	61355441	61417944	gain	3756	48748	120853004	Average_daily_gain	region1inRegion2	62503
CNVR87	994	7	82234623	82374446	gain	3756	48748	120853004	Average_daily_gain	region1inRegion2	139823
CNVR88	1002	7	84728780	84749202	gain	3756	48748	120853004	Average_daily_gain	region1inRegion2	20422
CNVR89	1011	7	102790067	102823288	gain	3756	48748	120853004	Average_daily_gain	region1inRegion2	33221
CNVR90	1021	7	111620166	111623056	loss	3756	48748	120853004	Average_daily_gain	region1inRegion2	2890
CNVR72	923	7	10444634	10478517	gain	3760	48748	120853004	Average_backfat_thickness	region1inRegion2	33883
CNVR73	934	7	22207228	22236111	gain	3760	48748	120853004	Average_backfat_thickness	region1inRegion2	28883
CNVR74	938	7	23307667	23658110	loss-gain	3760	48748	120853004	Average_backfat_thickness	region1inRegion2	350443
CNVR75	939	7	23481211	23658110	loss-gain	3760	48748	120853004	Average_backfat_thickness	region1inRegion2	176899
CNVR76	942	7	24648309	24653067	loss-gain	3760	48748	120853004	Average_backfat_thickness	region1inRegion2	4758
CNVR77	943	7	24735665	24740397	gain	3760	48748	120853004	Average_backfat_thickness	region1inRegion2	4732
CNVR78	944	7	25359190	25399904	gain	3760	48748	120853004	Average_backfat_thickness	region1inRegion2	40714
CNVR79	945	7	25488796	25557156	gain	3760	48748	120853004	Average_backfat_thickness	region1inRegion2	68360
CNVR80	946	7	25785853	25888939	gain	3760	48748	120853004	Average_backfat_thickness	region1inRegion2	103086
CNVR81	949	7	26267062	26297673	gain	3760	48748	120853004	Average_backfat_thickness	region1inRegion2	30611
CNVR82	951	7	26600964	26608495	gain	3760	48748	120853004	Average_backfat_thickness	region1inRegion2	7531
CNVR83	957	7	28525787	28565312	gain	3760	48748	120853004	Average_backfat_thickness	region1inRegion2	39525
CNVR84	972	7	58579811	58584542	gain	3760	48748	120853004	Average_backfat_thickness	region1inRegion2	4731
CNVR85	975	7	59278633	59284219	loss	3760	48748	120853004	Average_backfat_thickness	region1inRegion2	5586
CNVR86	977	7	61355441	61417944	gain	3760	48748	120853004	Average_backfat_thickness	region1inRegion2	62503
CNVR87	994	7	82234623	82374446	gain	3760	48748	120853004	Average_backfat_thickness	region1inRegion2	139823
CNVR88	1002	7	84728780	84749202	gain	3760	48748	120853004	Average_backfat_thickness	region1inRegion2	20422
CNVR89	1011	7	102790067	102823288	gain	3760	48748	120853004	Average_backfat_thickness	region1inRegion2	33221
CNVR90	1021	7	111620166	111623056	loss	3760	48748	120853004	Average_backfat_thickness	region1inRegion2	2890
CNVR72	923	7	10444634	10478517	gain	3762	48748	120853004	backfat_at_last_rib	region1inRegion2	33883
CNVR73	934	7	22207228	22236111	gain	3762	48748	120853004	backfat_at_last_rib	region1inRegion2	28883
CNVR74	938	7	23307667	23658110	loss-gain	3762	48748	120853004	backfat_at_last_rib	region1inRegion2	350443
CNVR75	939	7	23481211	23658110	loss-gain	3762	48748	120853004	backfat_at_last_rib	region1inRegion2	176899
CNVR76	942	7	24648309	24653067	loss-gain	3762	48748	120853004	backfat_at_last_rib	region1inRegion2	4758
CNVR77	943	7	24735665	24740397	gain	3762	48748	120853004	backfat_at_last_rib	region1inRegion2	4732
CNVR78	944	7	25359190	25399904	gain	3762	48748	120853004	backfat_at_last_rib	region1inRegion2	40714
CNVR79	945	7	25488796	25557156	gain	3762	48748	120853004	backfat_at_last_rib	region1inRegion2	68360

CNVR80	946	7	25785853	25888939	gain	3762	48748	120853004	backfat_at_last_rib	region1inRegion2	103086
CNVR81	949	7	26267062	26297673	gain	3762	48748	120853004	backfat_at_last_rib	region1inRegion2	30611
CNVR82	951	7	26600964	26608495	gain	3762	48748	120853004	backfat_at_last_rib	region1inRegion2	7531
CNVR83	957	7	28525787	28565312	gain	3762	48748	120853004	backfat_at_last_rib	region1inRegion2	39525
CNVR84	972	7	58579811	58584542	gain	3762	48748	120853004	backfat_at_last_rib	region1inRegion2	4731
CNVR85	975	7	59278633	59284219	loss	3762	48748	120853004	backfat_at_last_rib	region1inRegion2	5586
CNVR86	977	7	61355441	61417944	gain	3762	48748	120853004	backfat_at_last_rib	region1inRegion2	62503
CNVR87	994	7	82234623	82374446	gain	3762	48748	120853004	backfat_at_last_rib	region1inRegion2	139823
CNVR88	1002	7	84728780	84749202	gain	3762	48748	120853004	backfat_at_last_rib	region1inRegion2	20422
CNVR89	1011	7	102790067	102823288	gain	3762	48748	120853004	backfat_at_last_rib	region1inRegion2	33221
CNVR90	1021	7	111620166	111623056	loss	3762	48748	120853004	backfat_at_last_rib	region1inRegion2	2890
CNVR72	923	7	10444634	10478517	gain	3763	48748	120853004	Loin_muscle_depth	region1inRegion2	33883
CNVR73	934	7	22207228	22236111	gain	3763	48748	120853004	Loin_muscle_depth	region1inRegion2	28883
CNVR74	938	7	23307667	23658110	loss-gain	3763	48748	120853004	Loin_muscle_depth	region1inRegion2	350443
CNVR75	939	7	23481211	23658110	loss-gain	3763	48748	120853004	Loin_muscle_depth	region1inRegion2	176899
CNVR76	942	7	24648309	24653067	loss-gain	3763	48748	120853004	Loin_muscle_depth	region1inRegion2	4758
CNVR77	943	7	24735665	24740397	gain	3763	48748	120853004	Loin_muscle_depth	region1inRegion2	4732
CNVR78	944	7	25359190	25399904	gain	3763	48748	120853004	Loin_muscle_depth	region1inRegion2	40714
CNVR79	945	7	25488796	25557156	gain	3763	48748	120853004	Loin_muscle_depth	region1inRegion2	68360
CNVR80	946	7	25785853	25888939	gain	3763	48748	120853004	Loin_muscle_depth	region1inRegion2	103086
CNVR81	949	7	26267062	26297673	gain	3763	48748	120853004	Loin_muscle_depth	region1inRegion2	30611
CNVR82	951	7	26600964	26608495	gain	3763	48748	120853004	Loin_muscle_depth	region1inRegion2	7531
CNVR83	957	7	28525787	28565312	gain	3763	48748	120853004	Loin_muscle_depth	region1inRegion2	39525
CNVR84	972	7	58579811	58584542	gain	3763	48748	120853004	Loin_muscle_depth	region1inRegion2	4731
CNVR85	975	7	59278633	59284219	loss	3763	48748	120853004	Loin_muscle_depth	region1inRegion2	5586
CNVR86	977	7	61355441	61417944	gain	3763	48748	120853004	Loin_muscle_depth	region1inRegion2	62503
CNVR87	994	7	82234623	82374446	gain	3763	48748	120853004	Loin_muscle_depth	region1inRegion2	139823
CNVR88	1002	7	84728780	84749202	gain	3763	48748	120853004	Loin_muscle_depth	region1inRegion2	20422
CNVR89	1011	7	102790067	102823288	gain	3763	48748	120853004	Loin_muscle_depth	region1inRegion2	33221
CNVR90	1021	7	111620166	111623056	loss	3763	48748	120853004	Loin_muscle_depth	region1inRegion2	2890
CNVR72	923	7	10444634	10478517	gain	3765	48748	120853004	Loin_muscle_area	region1inRegion2	33883
CNVR73	934	7	22207228	22236111	gain	3765	48748	120853004	Loin_muscle_area	region1inRegion2	28883
CNVR74	938	7	23307667	23658110	loss-gain	3765	48748	120853004	Loin_muscle_area	region1inRegion2	350443
CNVR75	939	7	23481211	23658110	loss-gain	3765	48748	120853004	Loin_muscle_area	region1inRegion2	176899
CNVR76	942	7	24648309	24653067	loss-gain	3765	48748	120853004	Loin_muscle_area	region1inRegion2	4758
CNVR77	943	7	24735665	24740397	gain	3765	48748	120853004	Loin_muscle_area	region1inRegion2	4732
CNVR78	944	7	25359190	25399904	gain	3765	48748	120853004	Loin_muscle_area	region1inRegion2	40714
CNVR79	945	7	25488796	25557156	gain	3765	48748	120853004	Loin_muscle_area	region1inRegion2	68360
CNVR80	946	7	25785853	25888939	gain	3765	48748	120853004	Loin_muscle_area	region1inRegion2	103086
CNVR81	949	7	26267062	26297673	gain	3765	48748	120853004	Loin_muscle_area	region1inRegion2	30611
CNVR82	951	7	26600964	26608495	gain	3765	48748	120853004	Loin_muscle_area	region1inRegion2	7531
CNVR83	957	7	28525787	28565312	gain	3765	48748	120853004	Loin_muscle_area	region1inRegion2	39525
CNVR84	972	7	58579811	58584542	gain	3765	48748	120853004	Loin_muscle_area	region1inRegion2	4731
CNVR85	975	7	59278633	59284219	loss	3765	48748	120853004	Loin_muscle_area	region1inRegion2	5586
CNVR86	977	7	61355441	61417944	gain	3765	48748	120853004	Loin_muscle_area	region1inRegion2	62503
CNVR87	994	7	82234623	82374446	gain	3765	48748	120853004	Loin_muscle_area	region1inRegion2	139823
CNVR88	1002	7	84728780	84749202	gain	3765	48748	120853004	Loin_muscle_area	region1inRegion2	20422
CNVR89	1011	7	102790067	102823288	gain	3765	48748	120853004	Loin_muscle_area	region1inRegion2	33221
CNVR90	1021	7	111620166	111623056	loss	3765	48748	120853004	Loin_muscle_area	region1inRegion2	2890
CNVR72	923	7	10444634	10478517	gain	3766	48748	120853004	Average_daily_gain	region1inRegion2	33883
CNVR73	934	7	22207228	22236111	gain	3766	48748	120853004	Average_daily_gain	region1inRegion2	28883
CNVR74	938	7	23307667	23658110	loss-gain	3766	48748	120853004	Average_daily_gain	region1inRegion2	350443
CNVR75	939	7	23481211	23658110	loss-gain	3766	48748	120853004	Average_daily_gain	region1inRegion2	176899
CNVR76	942	7	24648309	24653067	loss-gain	3766	48748	120853004	Average_daily_gain	region1inRegion2	4758
CNVR77	943	7	24735665	24740397	gain	3766	48748	120853004	Average_daily_gain	region1inRegion2	4732
CNVR78	944	7	25359190	25399904	gain	3766	48748	120853004	Average_daily_gain	region1inRegion2	40714
CNVR79	945	7	25488796	25557156	gain	3766	48748	120853004	Average_daily_gain	region1inRegion2	68360
CNVR80	946	7	25785853	25888939	gain	3766	48748	120853004	Average_daily_gain	region1inRegion2	103086
CNVR81	949	7	26267062	26297673	gain	3766	48748	120853004	Average_daily_gain	region1inRegion2	30611
CNVR82	951	7	26600964	26608495	gain	3766	48748	120853004	Average_daily_gain	region1inRegion2	7531
CNVR83	957	7	28525787	28565312	gain	3766	48748	120853004	Average_daily_gain	region1inRegion2	39525
CNVR84	972	7	58579811	58584542	gain	3766	48748	120853004	Average_daily_gain	region1inRegion2	4731
CNVR85	975	7	59278633	59284219	loss	3766	48748	120853004	Average_daily_gain	region1inRegion2	5586
CNVR86	977	7	61355441	61417944	gain	3766	48748	120853004	Average_daily_gain	region1inRegion2	62503
CNVR87	994	7	82234623	82374446	gain	3766	48748	120853004	Average_daily_gain	region1inRegion2	139823
CNVR88	1002	7	84728780	84749202	gain	3766	48748	120853004	Average_daily_gain	region1inRegion2	20422
CNVR89	1011	7	102790067	102823288	gain	3766	48748	120853004	Average_daily_gain	region1inRegion2	33221
CNVR90	1021	7	111620166	111623056	loss	3766	48748	120853004	Average_daily_gain	region1inRegion2	2890
CNVR72	923	7	10444634	10478517	gain	3767	48748	120853004	Average_daily_gain	region1inRegion2	33883
CNVR73	934	7	22207228	22236111	gain	3767	48748	120853004	Average_daily_gain	region1inRegion2	28883
CNVR74	938	7	23307667	23658110	loss-gain	3767	48748	120853004	Average_daily_gain	region1inRegion2	350443
CNVR75	939	7	23481211	23658110	loss-gain	3767	48748	120853004	Average_daily_gain	region1inRegion2	176899
CNVR76	942	7	24648309	24653067	loss-gain	3767	48748	120853004	Average_daily_gain	region1inRegion2	4758
CNVR77	943	7	24735665	24740397	gain	3767	48748	120853004	Average_daily_gain	region1inRegion2	4732
CNVR78	944	7	25359190	25399904	gain	3767	48748	120853004	Average_daily_gain	region1inRegion2	40714
CNVR79	945	7	25488796	25557156	gain	3767	48748	120853004	Average_daily_gain	region1inRegion2	68360
CNVR80	946	7	25785853	25888939	gain	3767	48748	120853004	Average_daily_gain	region1inRegion2	103086
CNVR81	949	7	26267062	26297673	gain	3767	48748	120853004	Average_daily_gain	region1inRegion2	30611
CNVR82	951	7	26600964	26608495	gain	3767	48748	120853004	Average_daily_gain	region1inRegion2	7531
CNVR83	957	7	28525787	28565312	gain	3767	48748	120853004	Average_daily_gain	region1inRegion2	39525
CNVR84	972	7	58579811	58584542	gain	3767	48748	120853004	Average_daily_gain	region1inRegion2	4731
CNVR85	975	7	59278633	59284219	loss	3767	48748	120853004	Average_daily_gain	region1inRegion2	5586
CNVR86	977	7	61355441	61417944	gain	3767	48748	120853004	Average_daily_gain	region1inRegion2	62503
CNVR87	994	7	82234623	82374446	gain	3767	48748	120853004	Average_daily_gain	region1inRegion2	139823
CNVR88	1002	7	84728780	84749202	gain	3767	48748	120853004	Average_daily_gain	region1inRegion2	20422
CNVR89	1011	7	102790067	102823288	gain	3767	48748	120853004	Average_daily_gain	region1inRegion2	33221
CNVR90	1021	7	111620166	111623056	loss	3767	48748	120853004	Average_daily_gain	region1inRegion2	2890
CNVR72	923	7	10444634	10478517	gain	3769	48748	120853004	Lean_meat_percentage	region1inRegion2	33883
CNVR73	934	7	22207228	22236111	gain	3769	48748	120853004	Lean_meat_percentage	region1inRegion2	28883
CNVR74	938	7	23307667	23658110	loss-gain	3769	48748	120853004	Lean_meat_percentage	region1inRegion2	350443
CNVR75	939	7	23481211	23658110	loss-gain	3769	48748	120853004	Lean_meat_percentage	region1inRegion2	176899
CNVR76	942	7	24648309	24653067	loss-gain	3769	48748	120853004	Lean_meat_percentage	region1inRegion2	4758
CNVR77	943	7	24735665	24740397	gain	3769	48748	120853004	Lean_meat_percentage	region1inRegion2	4732
CNVR78	944	7	25359190	25399904	gain	3769	48748	120853004	Lean_meat_percentage	region1inRegion2	40714
CNVR79	945	7	25488796	25557156	gain	3769	48748	120853004	Lean_meat_percentage	region1inRegion2	68360
CNVR80	946	7	25785853	25888939	gain	3769	48748	120853004	Lean_meat_percentage	region1inRegion2	103086
CNVR81	949	7	26267062	26297673	gain	3769	48748	120853004	Lean_meat_percentage	region1inRegion2	30611
CNVR82	951	7	26600964	26608495	gain	3769	48748	120853004	Lean_meat_percentage	region1inRegion2	7531
CNVR83	957	7	28525787	28565312	gain	3769	48748	120853004	Lean_meat_percentage	region1inRegion2	39525
CNVR84	972	7	58579811	58584542	gain	3769	48748	120853004	Lean_meat_percentage	region1inRegion2	4731
CNVR85	975	7	59278633	59284219	loss	3769	48748	120853004	Lean_meat_percentage	region1inRegion2	5586
CNVR86	977	7	61355441	61417944	gain	3769	48748	120853004	Lean_meat_percentage	region1inRegion2	62503
CNVR87	994	7	82234623	82374446	gain	3769	48748	120853004	Lean_meat_percentage	region1inRegion2	139823
CNVR88	1002	7	84728780	84749202	gain	3769	48748	120853004	Lean_meat_percentage	region1inRegion2	20422
CNVR89	1011	7	102790067	102823288	gain	3769	48748	120853004	Lean_meat_percentage	region1inRegion2	33221
CNVR90	1021	7	111620166	111623056	loss	3769	48748	120853004	Lean_meat_percentage	region1inRegion2	2890
CNVR72	923	7	10444634	10478517	gain	3770	48748	120853004	Average_daily_gain	region1inRegion2	33883
CNVR73	934	7	22207228	22236111	gain	3770	48748	120853004	Average_daily_gain	region1inRegion2	28883
CNVR74	938	7	23307667	23658110	loss-gain	3770	48748	120853004	Average_daily_gain	region1inRegion2	350443
CNVR75	939	7	23481211	23658110	loss-gain	3770	48748	120853004	Average_daily_gain	region1inRegion2	176899
CNVR76	942	7	24648309	24653067	loss-gain	3770	48748	120853004	Average_daily_gain	region1inRegion2	4758
CNVR77	943	7	24735665	24740397	gain	3770	48748	120853004	Average_daily_gain	region1inRegion2	4732
CNVR78	944	7	25359190	25399904	gain	3770	48748	120853004	Average_daily_gain	region1inRegion2	40714
CNVR79	945	7	25488796	25557156	gain	3770	48748	120853004	Average_daily_gain	region1inRegion2	68360
CNVR80	946	7	25785853	25888939	gain	3770	48748	120853004	Average_daily_gain	region1inRegion2	103086
CNVR81	949	7	26267062	26297673	gain	3770	48748	120853004	Average_daily_gain	region1inRegion2	30611
CNVR82	951	7	26600964	26608495	gain	3770	48748	120853004	Average_daily_gain	region1inRegion2	7531
CNVR83	957	7	28525787	28565312	gain	3770	48748	120853004	Average_daily_gain	region1inRegion2	39525
CNVR84	972	7	58579811	58584542	gain	3770	48748	120853004	Average_daily_gain	region1inRegion2	4731
CNVR85	975	7	59278633	59284219	loss	3770	48748	120853004	Average_daily_gain	region1inRegion2	5586
CNVR86	977	7	61355441	61417944	gain	3770	48748	120853004	Average_daily_gain	region1inRegion2	62503
CNVR87	994	7	82234623	82374446	gain	3770	48748	120853004	Average_daily_gain	region1inRegion2	139823

CNVR88	1002	7	84728780	84749202	gain	3770	48748	120853004	Average_daily_gain	region1inRegion2	20422
CNVR89	1011	7	102790067	102823288	gain	3770	48748	120853004	Average_daily_gain	region1inRegion2	33221
CNVR90	1021	7	111620166	111623056	loss	3770	48748	120853004	Average_daily_gain	region1inRegion2	2890
CNVR72	923	7	10444634	10478517	gain	18658	2462665	35290880	androstenone,_laboratory	region1inRegion2	33883
CNVR73	934	7	22207228	22236111	gain	18658	2462665	35290880	androstenone,_laboratory	region1inRegion2	28883
CNVR74	938	7	23307667	23658110	loss-gain	18658	2462665	35290880	androstenone,_laboratory	region1inRegion2	350443
CNVR75	939	7	23481211	23658110	loss-gain	18658	2462665	35290880	androstenone,_laboratory	region1inRegion2	176899
CNVR76	942	7	24648309	24653067	loss-gain	18658	2462665	35290880	androstenone,_laboratory	region1inRegion2	4758
CNVR77	943	7	24735665	24740397	gain	18658	2462665	35290880	androstenone,_laboratory	region1inRegion2	4732
CNVR78	944	7	25359190	25399904	gain	18658	2462665	35290880	androstenone,_laboratory	region1inRegion2	40714
CNVR79	945	7	25488796	25557156	gain	18658	2462665	35290880	androstenone,_laboratory	region1inRegion2	68360
CNVR80	946	7	25785853	25888939	gain	18658	2462665	35290880	androstenone,_laboratory	region1inRegion2	103086
CNVR81	949	7	26267062	26297673	gain	18658	2462665	35290880	androstenone,_laboratory	region1inRegion2	30611
CNVR82	951	7	26600964	26608495	gain	18658	2462665	35290880	androstenone,_laboratory	region1inRegion2	7531
CNVR83	957	7	28525787	28565312	gain	18658	2462665	35290880	androstenone,_laboratory	region1inRegion2	39525
CNVR72	923	7	10444634	10478517	gain	1058	4252710	11625414	Shoulder_external_fat_weight	region1inRegion2	33883
CNVR72	923	7	10444634	10478517	gain	1064	4252710	11625414	Carcass_length	region1inRegion2	33883
CNVR72	923	7	10444634	10478517	gain	1081	4252710	11625414	backfat_at_last_rib	region1inRegion2	33883
CNVR72	923	7	10444634	10478517	gain	12320	4252710	11625414	Mycoplasma_hyopneumoniae_antibody_titer	region1inRegion2	33883
CNVR72	923	7	10444634	10478517	gain	21382	4252710	11625414	muscle_protein_percentage	region1inRegion2	33883
CNVR72	923	7	10444634	10478517	gain	3251	4252710	11625414	Meat_color-L	region1inRegion2	33883
CNVR72	923	7	10444634	10478517	gain	3252	4252710	11625414	Meat_color_score	region1inRegion2	33883
CNVR72	923	7	10444634	10478517	gain	3253	4252710	11625414	muscle_protein_percentage	region1inRegion2	33883
CNVR72	923	7	10444634	10478517	gain	3860	4252710	11625414	Backfat_at_tenth_rib	region1inRegion2	33883
CNVR72	923	7	10444634	10478517	gain	5922	4252710	11625414	Time_spent_walking	region1inRegion2	33883
CNVR72	923	7	10444634	10478517	gain	6325	4252710	11625414	CO2_partial_pressure	region1inRegion2	33883
CNVR72	923	7	10444634	10478517	gain	6375	4252710	11625414	Bilirubin_level	region1inRegion2	33883
CNVR72	923	7	10444634	10478517	gain	16867	4252710	12337141	Carcass_weight_(hot)	region1inRegion2	33883
CNVR72	923	7	10444634	10478517	gain	16881	4252710	33757808	backfat_at_last_rib	region1inRegion2	33883
CNVR73	934	7	22207228	22236111	gain	16881	4252710	33757808	backfat_at_last_rib	region1inRegion2	28883
CNVR74	938	7	23307667	23658110	loss-gain	16881	4252710	33757808	backfat_at_last_rib	region1inRegion2	350443
CNVR75	939	7	23481211	23658110	loss-gain	16881	4252710	33757808	backfat_at_last_rib	region1inRegion2	176899
CNVR76	942	7	24648309	24653067	loss-gain	16881	4252710	33757808	backfat_at_last_rib	region1inRegion2	4758
CNVR77	943	7	24735665	24740397	gain	16881	4252710	33757808	backfat_at_last_rib	region1inRegion2	4732
CNVR78	944	7	25359190	25399904	gain	16881	4252710	33757808	backfat_at_last_rib	region1inRegion2	40714
CNVR79	945	7	25488796	25557156	gain	16881	4252710	33757808	backfat_at_last_rib	region1inRegion2	68360
CNVR80	946	7	25785853	25888939	gain	16881	4252710	33757808	backfat_at_last_rib	region1inRegion2	103086
CNVR81	949	7	26267062	26297673	gain	16881	4252710	33757808	backfat_at_last_rib	region1inRegion2	30611
CNVR82	951	7	26600964	26608495	gain	16881	4252710	33757808	backfat_at_last_rib	region1inRegion2	7531
CNVR83	957	7	28525787	28565312	gain	16881	4252710	33757808	backfat_at_last_rib	region1inRegion2	39525
CNVR72	923	7	10444634	10478517	gain	5221	4252710	132120394	Shoulder_subcutaneous_fat_thickness	region1inRegion2	33883
CNVR73	934	7	22207228	22236111	gain	5221	4252710	132120394	Shoulder_subcutaneous_fat_thickness	region1inRegion2	28883
CNVR74	938	7	23307667	23658110	loss-gain	5221	4252710	132120394	Shoulder_subcutaneous_fat_thickness	region1inRegion2	350443
CNVR75	939	7	23481211	23658110	loss-gain	5221	4252710	132120394	Shoulder_subcutaneous_fat_thickness	region1inRegion2	176899
CNVR76	942	7	24648309	24653067	loss-gain	5221	4252710	132120394	Shoulder_subcutaneous_fat_thickness	region1inRegion2	4758
CNVR77	943	7	24735665	24740397	gain	5221	4252710	132120394	Shoulder_subcutaneous_fat_thickness	region1inRegion2	4732
CNVR78	944	7	25359190	25399904	gain	5221	4252710	132120394	Shoulder_subcutaneous_fat_thickness	region1inRegion2	40714
CNVR79	945	7	25488796	25557156	gain	5221	4252710	132120394	Shoulder_subcutaneous_fat_thickness	region1inRegion2	68360
CNVR80	946	7	25785853	25888939	gain	5221	4252710	132120394	Shoulder_subcutaneous_fat_thickness	region1inRegion2	103086
CNVR81	949	7	26267062	26297673	gain	5221	4252710	132120394	Shoulder_subcutaneous_fat_thickness	region1inRegion2	30611
CNVR82	951	7	26600964	26608495	gain	5221	4252710	132120394	Shoulder_subcutaneous_fat_thickness	region1inRegion2	7531
CNVR83	957	7	28525787	28565312	gain	5221	4252710	132120394	Shoulder_subcutaneous_fat_thickness	region1inRegion2	39525
CNVR84	972	7	58579811	58584542	gain	5221	4252710	132120394	Shoulder_subcutaneous_fat_thickness	region1inRegion2	4731
CNVR85	975	7	59278633	59284219	loss	5221	4252710	132120394	Shoulder_subcutaneous_fat_thickness	region1inRegion2	5586
CNVR86	977	7	61355441	61417944	gain	5221	4252710	132120394	Shoulder_subcutaneous_fat_thickness	region1inRegion2	62503
CNVR87	994	7	82234623	82374446	gain	5221	4252710	132120394	Shoulder_subcutaneous_fat_thickness	region1inRegion2	139823
CNVR88	1002	7	84728780	84749202	gain	5221	4252710	132120394	Shoulder_subcutaneous_fat_thickness	region1inRegion2	20422
CNVR89	1011	7	102790067	102823288	gain	5221	4252710	132120394	Shoulder_subcutaneous_fat_thickness	region1inRegion2	33221
CNVR90	1021	7	111620166	111623056	loss	5221	4252710	132120394	Shoulder_subcutaneous_fat_thickness	region1inRegion2	2890
CNVR91	1035	7	131449863	131460402	gain	5221	4252710	132120394	Shoulder_subcutaneous_fat_thickness	region1inRegion2	10539
CNVR72	923	7	10444634	10478517	gain	3954	7434494	20633784	Carcass_width_(minimum)	region1inRegion2	33883
CNVR72	923	7	10444634	10478517	gain	3955	7434494	20633784	Semimembranosus_angle	region1inRegion2	33883
CNVR72	923	7	10444634	10478517	gain	3956	7434494	20633784	Backfat_at_first_rib	region1inRegion2	33883
CNVR72	923	7	10444634	10478517	gain	3957	7434494	20633784	Backfat_between_6th_and_7th_ribs	region1inRegion2	33883
CNVR72	923	7	10444634	10478517	gain	38094	7434494	99614552	CIE-a*	region1inRegion2	33883
CNVR73	934	7	22207228	22236111	gain	38094	7434494	99614552	CIE-a*	region1inRegion2	28883
CNVR74	938	7	23307667	23658110	loss-gain	38094	7434494	99614552	CIE-a*	region1inRegion2	350443
CNVR75	939	7	23481211	23658110	loss-gain	38094	7434494	99614552	CIE-a*	region1inRegion2	176899
CNVR76	942	7	24648309	24653067	loss-gain	38094	7434494	99614552	CIE-a*	region1inRegion2	4758
CNVR77	943	7	24735665	24740397	gain	38094	7434494	99614552	CIE-a*	region1inRegion2	4732
CNVR78	944	7	25359190	25399904	gain	38094	7434494	99614552	CIE-a*	region1inRegion2	40714
CNVR79	945	7	25488796	25557156	gain	38094	7434494	99614552	CIE-a*	region1inRegion2	68360
CNVR80	946	7	25785853	25888939	gain	38094	7434494	99614552	CIE-a*	region1inRegion2	103086
CNVR81	949	7	26267062	26297673	gain	38094	7434494	99614552	CIE-a*	region1inRegion2	30611
CNVR82	951	7	26600964	26608495	gain	38094	7434494	99614552	CIE-a*	region1inRegion2	7531
CNVR83	957	7	28525787	28565312	gain	38094	7434494	99614552	CIE-a*	region1inRegion2	39525
CNVR84	972	7	58579811	58584542	gain	38094	7434494	99614552	CIE-a*	region1inRegion2	4731
CNVR85	975	7	59278633	59284219	loss	38094	7434494	99614552	CIE-a*	region1inRegion2	5586
CNVR86	977	7	61355441	61417944	gain	38094	7434494	99614552	CIE-a*	region1inRegion2	62503
CNVR87	994	7	82234623	82374446	gain	38094	7434494	99614552	CIE-a*	region1inRegion2	139823
CNVR88	1002	7	84728780	84749202	gain	38094	7434494	99614552	CIE-a*	region1inRegion2	20422
CNVR72	923	7	10444634	10478517	gain	38092	7434494	124300676	Loin_muscle_area	region1inRegion2	33883
CNVR73	934	7	22207228	22236111	gain	38092	7434494	124300676	Loin_muscle_area	region1inRegion2	28883
CNVR74	938	7	23307667	23658110	loss-gain	38092	7434494	124300676	Loin_muscle_area	region1inRegion2	350443
CNVR75	939	7	23481211	23658110	loss-gain	38092	7434494	124300676	Loin_muscle_area	region1inRegion2	176899
CNVR76	942	7	24648309	24653067	loss-gain	38092	7434494	124300676	Loin_muscle_area	region1inRegion2	4758
CNVR77	943	7	24735665	24740397	gain	38092	7434494	124300676	Loin_muscle_area	region1inRegion2	4732
CNVR78	944	7	25359190	25399904	gain	38092	7434494	124300676	Loin_muscle_area	region1inRegion2	40714
CNVR79	945	7	25488796	25557156	gain	38092	7434494	124300676	Loin_muscle_area	region1inRegion2	68360
CNVR80	946	7	25785853	25888939	gain	38092	7434494	124300676	Loin_muscle_area	region1inRegion2	103086
CNVR81	949	7	26267062	26297673	gain	38092	7434494	124300676	Loin_muscle_area	region1inRegion2	30611
CNVR82	951	7	26600964	26608495	gain	38092	7434494	124300676	Loin_muscle_area	region1inRegion2	7531
CNVR83	957	7	28525787	28565312	gain	38092	7434494	124300676	Loin_muscle_area	region1inRegion2	39525
CNVR84	972	7	58579811	58584542	gain	38092	7434494	124300676	Loin_muscle_area	region1inRegion2	4731
CNVR85	975	7	59278633	59284219	loss	38092	7434494	124300676	Loin_muscle_area	region1inRegion2	5586
CNVR86	977	7	61355441	61417944	gain	38092	7434494	124300676	Loin_muscle_area	region1inRegion2	62503
CNVR87	994	7	82234623	82374446	gain	38092	7434494	124300676	Loin_muscle_area	region1inRegion2	139823
CNVR88	1002	7	84728780	84749202	gain	38092	7434494	124300676	Loin_muscle_area	region1inRegion2	20422
CNVR89	1011	7	102790067	102823288	gain	38092	7434494	124300676	Loin_muscle_area	region1inRegion2	33221
CNVR90	1021	7	111620166	111623056	loss	38092	7434494	124300676	Loin_muscle_area	region1inRegion2	2890
CNVR72	923	7	10444634	10478517	gain	15102	7974684	11402680	Phosphate_level	region1inRegion2	33883
CNVR72	923	7	10444634	10478517	gain	24284	8014191	92220281	Corpus_luteum_number	region1inRegion2	33883
CNVR73	934	7	22207228	22236111	gain	24284	8014191	92220281	Corpus_luteum_number	region1inRegion2	28883
CNVR74	938	7	23307667	23658110	loss-gain	24284	8014191	92220281	Corpus_luteum_number	region1inRegion2	350443
CNVR75	939	7	23481211	23658110	loss-gain	24284	8014191	92220281	Corpus_luteum_number	region1inRegion2	176899
CNVR76	942	7	24648309	24653067	loss-gain	24284	8014191	92220281	Corpus_luteum_number	region1inRegion2	4758
CNVR77	943	7	24735665	24740397	gain	24284	8014191	92220281	Corpus_luteum_number	region1inRegion2	4732
CNVR78	944	7	25359190	25399904	gain	24284	8014191	92220281	Corpus_luteum_number	region1inRegion2	40714
CNVR79	945	7	25488796	25557156	gain	24284	8014191	92220281	Corpus_luteum_number	region1inRegion2	68360
CNVR80	946	7	25785853	25888939	gain	24284	8014191	92220281	Corpus_luteum_number	region1inRegion2	103086
CNVR81	949	7	26267062	26297673	gain	24284	8014191	92220281	Corpus_luteum_number	region1inRegion2	30611
CNVR82	951	7	26600964	26608495	gain	24284	8014191	92220281	Corpus_luteum_number	region1inRegion2	7531
CNVR83	957	7	28525787	28565312	gain	24284	8014191	92220281	Corpus_luteum_number	region1inRegion2	39525
CNVR84	972	7	58579811	58584542	gain	24284	8014191	92220281	Corpus_luteum_number	region1inRegion2	4731
CNVR85	975	7	59278633	59284219	loss	24284	8014191	92220281	Corpus_luteum_number	region1inRegion2	5586
CNVR86	977	7	61355441	61417944	gain	24284	8014191	92220281	Corpus_luteum_number	region1inRegion2	62503
CNVR87	994	7	82234623	82374446	gain	24284	8014191	92220281	Corpus_luteum_number	region1inRegion2	139823
CNVR88	1002	7	84728780	84749202	gain	24284	8014191	92220281	Corpus_luteum_number	region1inRegion2	20422
CNVR72	923	7	10444634	10478517	gain	4204	8764931	13812134	Linoleic_acid_content	region1inRegion2	33883
CNVR72	923	7	10444634	10478517	gain	3882	9663913	14781089	Average_backfat_thickness	region1inRegion2	33883
CNVR72	923	7	10444634	10478517	gain	9105	10242996	10457106	Adipocyte_volume	overlapHead	12473
CNVR72	923	7	10444634	10478517	gain	17817	10325548	10539659	Ear_size	region1inRegion2	33883

CNVR72	923	7	10444634	10478517	gain	17818	10325548	10539659	Ear_size	region1inRegion2	33883
CNVR72	923	7	10444634	10478517	gain	17819	10325548	10539659	Ear_size	region1inRegion2	33883
CNVR72	923	7	10444634	10478517	gain	17820	10325548	10539659	Ear_size	region1inRegion2	33883
CNVR72	923	7	10444634	10478517	gain	17821	10325548	10539659	Ear_size	region1inRegion2	33883
CNVR72	923	7	10444634	10478517	gain	17822	10325548	10539659	Ear_size	region1inRegion2	33883
CNVR72	923	7	10444634	10478517	gain	17823	10325548	10539659	Ear_size	region1inRegion2	33883
CNVR72	923	7	10444634	10478517	gain	31864	10464719	10584379	Corpus_luteum_number	overlapTail	13799
CNVR73	934	7	22207228	22236111	gain	16852	10665953	57665334	Average_daily_gain	region1inRegion2	28883
CNVR74	938	7	23307667	23658110	loss-gain	16852	10665953	57665334	Average_daily_gain	region1inRegion2	350443
CNVR75	939	7	23481211	23658110	loss-gain	16852	10665953	57665334	Average_daily_gain	region1inRegion2	176899
CNVR76	942	7	24648309	24653067	loss-gain	16852	10665953	57665334	Average_daily_gain	region1inRegion2	4758
CNVR77	943	7	24735665	24740397	gain	16852	10665953	57665334	Average_daily_gain	region1inRegion2	4732
CNVR78	944	7	25359190	25399904	gain	16852	10665953	57665334	Average_daily_gain	region1inRegion2	40714
CNVR79	945	7	25488796	25557156	gain	16852	10665953	57665334	Average_daily_gain	region1inRegion2	68360
CNVR80	946	7	25785853	25888939	gain	16852	10665953	57665334	Average_daily_gain	region1inRegion2	103086
CNVR81	949	7	26267062	26297673	gain	16852	10665953	57665334	Average_daily_gain	region1inRegion2	30611
CNVR82	951	7	26600964	26608495	gain	16852	10665953	57665334	Average_daily_gain	region1inRegion2	7531
CNVR83	957	7	28525787	28565312	gain	16852	10665953	57665334	Average_daily_gain	region1inRegion2	39525
CNVR73	934	7	22207228	22236111	gain	3768	10748505	45102432	Average_backfat_thickness	region1inRegion2	28883
CNVR74	938	7	23307667	23658110	loss-gain	3768	10748505	45102432	Average_backfat_thickness	region1inRegion2	350443
CNVR75	939	7	23481211	23658110	loss-gain	3768	10748505	45102432	Average_backfat_thickness	region1inRegion2	176899
CNVR76	942	7	24648309	24653067	loss-gain	3768	10748505	45102432	Average_backfat_thickness	region1inRegion2	4758
CNVR77	943	7	24735665	24740397	gain	3768	10748505	45102432	Average_backfat_thickness	region1inRegion2	4732
CNVR78	944	7	25359190	25399904	gain	3768	10748505	45102432	Average_backfat_thickness	region1inRegion2	40714
CNVR79	945	7	25488796	25557156	gain	3768	10748505	45102432	Average_backfat_thickness	region1inRegion2	68360
CNVR80	946	7	25785853	25888939	gain	3768	10748505	45102432	Average_backfat_thickness	region1inRegion2	103086
CNVR81	949	7	26267062	26297673	gain	3768	10748505	45102432	Average_backfat_thickness	region1inRegion2	30611
CNVR82	951	7	26600964	26608495	gain	3768	10748505	45102432	Average_backfat_thickness	region1inRegion2	7531
CNVR83	957	7	28525787	28565312	gain	3768	10748505	45102432	Average_backfat_thickness	region1inRegion2	39525
CNVR73	934	7	22207228	22236111	gain	308	10748505	64962096	Average_backfat_thickness	region1inRegion2	28883
CNVR74	938	7	23307667	23658110	loss-gain	308	10748505	64962096	Average_backfat_thickness	region1inRegion2	350443
CNVR75	939	7	23481211	23658110	loss-gain	308	10748505	64962096	Average_backfat_thickness	region1inRegion2	176899
CNVR76	942	7	24648309	24653067	loss-gain	308	10748505	64962096	Average_backfat_thickness	region1inRegion2	4758
CNVR77	943	7	24735665	24740397	gain	308	10748505	64962096	Average_backfat_thickness	region1inRegion2	4732
CNVR78	944	7	25359190	25399904	gain	308	10748505	64962096	Average_backfat_thickness	region1inRegion2	40714
CNVR79	945	7	25488796	25557156	gain	308	10748505	64962096	Average_backfat_thickness	region1inRegion2	68360
CNVR80	946	7	25785853	25888939	gain	308	10748505	64962096	Average_backfat_thickness	region1inRegion2	103086
CNVR81	949	7	26267062	26297673	gain	308	10748505	64962096	Average_backfat_thickness	region1inRegion2	30611
CNVR82	951	7	26600964	26608495	gain	308	10748505	64962096	Average_backfat_thickness	region1inRegion2	7531
CNVR83	957	7	28525787	28565312	gain	308	10748505	64962096	Average_backfat_thickness	region1inRegion2	39525
CNVR84	972	7	58579811	58584542	gain	308	10748505	64962096	Average_backfat_thickness	region1inRegion2	4731
CNVR85	975	7	59278633	59284219	loss	308	10748505	64962096	Average_backfat_thickness	region1inRegion2	5586
CNVR86	977	7	61355441	61417944	gain	308	10748505	64962096	Average_backfat_thickness	region1inRegion2	62503
CNVR73	934	7	22207228	22236111	gain	3	10748505	99614552	Carcass_weight_(hot)	region1inRegion2	28883
CNVR74	938	7	23307667	23658110	loss-gain	3	10748505	99614552	Carcass_weight_(hot)	region1inRegion2	350443
CNVR75	939	7	23481211	23658110	loss-gain	3	10748505	99614552	Carcass_weight_(hot)	region1inRegion2	176899
CNVR76	942	7	24648309	24653067	loss-gain	3	10748505	99614552	Carcass_weight_(hot)	region1inRegion2	4758
CNVR77	943	7	24735665	24740397	gain	3	10748505	99614552	Carcass_weight_(hot)	region1inRegion2	4732
CNVR78	944	7	25359190	25399904	gain	3	10748505	99614552	Carcass_weight_(hot)	region1inRegion2	40714
CNVR79	945	7	25488796	25557156	gain	3	10748505	99614552	Carcass_weight_(hot)	region1inRegion2	68360
CNVR80	946	7	25785853	25888939	gain	3	10748505	99614552	Carcass_weight_(hot)	region1inRegion2	103086
CNVR81	949	7	26267062	26297673	gain	3	10748505	99614552	Carcass_weight_(hot)	region1inRegion2	30611
CNVR82	951	7	26600964	26608495	gain	3	10748505	99614552	Carcass_weight_(hot)	region1inRegion2	7531
CNVR83	957	7	28525787	28565312	gain	3	10748505	99614552	Carcass_weight_(hot)	region1inRegion2	39525
CNVR84	972	7	58579811	58584542	gain	3	10748505	99614552	Carcass_weight_(hot)	region1inRegion2	4731
CNVR85	975	7	59278633	59284219	loss	3	10748505	99614552	Carcass_weight_(hot)	region1inRegion2	5586
CNVR86	977	7	61355441	61417944	gain	3	10748505	99614552	Carcass_weight_(hot)	region1inRegion2	62503
CNVR87	994	7	82234623	82374446	gain	3	10748505	99614552	Carcass_weight_(hot)	region1inRegion2	139823
CNVR88	1002	7	84728780	84749202	gain	3	10748505	99614552	Carcass_weight_(hot)	region1inRegion2	20422
CNVR73	934	7	22207228	22236111	gain	38055	10748505	124300676	Loin_fat_percentage	region1inRegion2	28883
CNVR74	938	7	23307667	23658110	loss-gain	38055	10748505	124300676	Loin_fat_percentage	region1inRegion2	350443
CNVR75	939	7	23481211	23658110	loss-gain	38055	10748505	124300676	Loin_fat_percentage	region1inRegion2	176899
CNVR76	942	7	24648309	24653067	loss-gain	38055	10748505	124300676	Loin_fat_percentage	region1inRegion2	4758
CNVR77	943	7	24735665	24740397	gain	38055	10748505	124300676	Loin_fat_percentage	region1inRegion2	4732
CNVR78	944	7	25359190	25399904	gain	38055	10748505	124300676	Loin_fat_percentage	region1inRegion2	40714
CNVR79	945	7	25488796	25557156	gain	38055	10748505	124300676	Loin_fat_percentage	region1inRegion2	68360
CNVR80	946	7	25785853	25888939	gain	38055	10748505	124300676	Loin_fat_percentage	region1inRegion2	103086
CNVR81	949	7	26267062	26297673	gain	38055	10748505	124300676	Loin_fat_percentage	region1inRegion2	30611
CNVR82	951	7	26600964	26608495	gain	38055	10748505	124300676	Loin_fat_percentage	region1inRegion2	7531
CNVR83	957	7	28525787	28565312	gain	38055	10748505	124300676	Loin_fat_percentage	region1inRegion2	39525
CNVR84	972	7	58579811	58584542	gain	38055	10748505	124300676	Loin_fat_percentage	region1inRegion2	4731
CNVR85	975	7	59278633	59284219	loss	38055	10748505	124300676	Loin_fat_percentage	region1inRegion2	5586
CNVR86	977	7	61355441	61417944	gain	38055	10748505	124300676	Loin_fat_percentage	region1inRegion2	62503
CNVR87	994	7	82234623	82374446	gain	38055	10748505	124300676	Loin_fat_percentage	region1inRegion2	139823
CNVR88	1002	7	84728780	84749202	gain	38055	10748505	124300676	Loin_fat_percentage	region1inRegion2	20422
CNVR89	1011	7	102790067	102823288	gain	38055	10748505	124300676	Loin_fat_percentage	region1inRegion2	33221
CNVR90	1021	7	111620166	111623056	loss	38055	10748505	124300676	Loin_fat_percentage	region1inRegion2	2890
CNVR73	934	7	22207228	22236111	gain	5257	10763543	117929721	Teat_number	region1inRegion2	28883
CNVR74	938	7	23307667	23658110	loss-gain	5257	10763543	117929721	Teat_number	region1inRegion2	350443
CNVR75	939	7	23481211	23658110	loss-gain	5257	10763543	117929721	Teat_number	region1inRegion2	176899
CNVR76	942	7	24648309	24653067	loss-gain	5257	10763543	117929721	Teat_number	region1inRegion2	4758
CNVR77	943	7	24735665	24740397	gain	5257	10763543	117929721	Teat_number	region1inRegion2	4732
CNVR78	944	7	25359190	25399904	gain	5257	10763543	117929721	Teat_number	region1inRegion2	40714
CNVR79	945	7	25488796	25557156	gain	5257	10763543	117929721	Teat_number	region1inRegion2	68360
CNVR80	946	7	25785853	25888939	gain	5257	10763543	117929721	Teat_number	region1inRegion2	103086
CNVR81	949	7	26267062	26297673	gain	5257	10763543	117929721	Teat_number	region1inRegion2	30611
CNVR82	951	7	26600964	26608495	gain	5257	10763543	117929721	Teat_number	region1inRegion2	7531
CNVR83	957	7	28525787	28565312	gain	5257	10763543	117929721	Teat_number	region1inRegion2	39525
CNVR84	972	7	58579811	58584542	gain	5257	10763543	117929721	Teat_number	region1inRegion2	4731
CNVR85	975	7	59278633	59284219	loss	5257	10763543	117929721	Teat_number	region1inRegion2	5586
CNVR86	977	7	61355441	61417944	gain	5257	10763543	117929721	Teat_number	region1inRegion2	62503
CNVR87	994	7	82234623	82374446	gain	5257	10763543	117929721	Teat_number	region1inRegion2	139823
CNVR88	1002	7	84728780	84749202	gain	5257	10763543	117929721	Teat_number	region1inRegion2	20422
CNVR89	1011	7	102790067	102823288	gain	5257	10763543	117929721	Teat_number	region1inRegion2	33221
CNVR90	1021	7	111620166	111623056	loss	5257	10763543	117929721	Teat_number	region1inRegion2	2890
CNVR73	934	7	22207228	22236111	gain	1048	11625414	30929840	Carcass_length	region1inRegion2	28883
CNVR74	938	7	23307667	23658110	loss-gain	1048	11625414	30929840	Carcass_length	region1inRegion2	350443
CNVR75	939	7	23481211	23658110	loss-gain	1048	11625414	30929840	Carcass_length	region1inRegion2	176899
CNVR76	942	7	24648309	24653067	loss-gain	1048	11625414	30929840	Carcass_length	region1inRegion2	4758
CNVR77	943	7	24735665	24740397	gain	1048	11625414	30929840	Carcass_length	region1inRegion2	4732
CNVR78	944	7	25359190	25399904	gain	1048	11625414	30929840	Carcass_length	region1inRegion2	40714
CNVR79	945	7	25488796	25557156	gain	1048	11625414	30929840	Carcass_length	region1inRegion2	68360
CNVR80	946	7	25785853	25888939	gain	1048	11625414	30929840	Carcass_length	region1inRegion2	103086
CNVR81	949	7	26267062	26297673	gain	1048	11625414	30929840	Carcass_length	region1inRegion2	30611
CNVR82	951	7	26600964	26608495	gain	1048	11625414	30929840	Carcass_length	region1inRegion2	7531
CNVR83	957	7	28525787	28565312	gain	1048	11625414	30929840	Carcass_length	region1inRegion2	39525
CNVR73	934	7	22207228	22236111	gain	1055	11625414	30929840	Liver_weight	region1inRegion2	28883
CNVR74	938	7	23307667	23658110	loss-gain	1055	11625414	30929840	Liver_weight	region1inRegion2	350443
CNVR75	939	7	23481211	23658110	loss-gain	1055	11625414	30929840	Liver_weight	region1inRegion2	176899
CNVR76	942	7	24648309	24653067	loss-gain	1055	11625414	30929840	Liver_weight	region1inRegion2	4758
CNVR77	943	7	24735665	24740397	gain	1055	11625414	30929840	Liver_weight	region1inRegion2	4732
CNVR78	944	7	25359190	25399904	gain	1055	11625414	30929840	Liver_weight	region1inRegion2	40714
CNVR79	945	7	25488796	25557156	gain	1055	11625414	30929840	Liver_weight	region1inRegion2	68360
CNVR80	946	7	25785853	25888939	gain	1055	11625414	30929840	Liver_weight	region1inRegion2	103086
CNVR81	949	7	26267062	26297673	gain	1055	11625414	30929840	Liver_weight	region1inRegion2	30611
CNVR82	951	7	26600964	26608495	gain	1055	11625414	30929840	Liver_weight	region1inRegion2	7531
CNVR83	957	7	28525787	28565312	gain	1055	11625414	30929840	Liver_weight	region1inRegion2	39525
CNVR73	934	7	22207228	22236111	gain	1057	11625414	30929840	Heart_weight	region1inRegion2	28883
CNVR74	938	7	23307667	23658110	loss-gain	1057	11625414	30929840	Heart_weight	region1inRegion2	350443
CNVR75	939	7	23481211	23658110	loss-gain	1057	11625414	30929840	Heart_weight	region1inRegion2	176899
CNVR76	942	7	24648309	24653067	loss-gain	1057	11625414	30929840	Heart_weight	region1inRegion2	4758
CNVR77	943	7	24735665	24740397	gain	1057	11625414	30929840	Heart_weight	region1inRegion2	4732

CNVR78	944	7	25359190	25399904	gain	1057	11625414	30929840	Heart_weight	region1inRegion2	40714
CNVR79	945	7	25488796	25557156	gain	1057	11625414	30929840	Heart_weight	region1inRegion2	68360
CNVR80	946	7	25785853	25888939	gain	1057	11625414	30929840	Heart_weight	region1inRegion2	103086
CNVR81	949	7	26267062	26297673	gain	1057	11625414	30929840	Heart_weight	region1inRegion2	30611
CNVR82	951	7	26600964	26608495	gain	1057	11625414	30929840	Heart_weight	region1inRegion2	7531
CNVR83	957	7	28525787	28565312	gain	1057	11625414	30929840	Heart_weight	region1inRegion2	39525
CNVR73	934	7	22207228	22236111	gain	1062	11625414	30929840	Abdominal_fat_weight	region1inRegion2	28883
CNVR74	938	7	23307667	23658110	loss-gain	1062	11625414	30929840	Abdominal_fat_weight	region1inRegion2	350443
CNVR75	939	7	23481211	23658110	loss-gain	1062	11625414	30929840	Abdominal_fat_weight	region1inRegion2	176899
CNVR76	942	7	24648309	24653067	loss-gain	1062	11625414	30929840	Abdominal_fat_weight	region1inRegion2	4758
CNVR77	943	7	24735665	24740397	gain	1062	11625414	30929840	Abdominal_fat_weight	region1inRegion2	4732
CNVR78	944	7	25359190	25399904	gain	1062	11625414	30929840	Abdominal_fat_weight	region1inRegion2	40714
CNVR79	945	7	25488796	25557156	gain	1062	11625414	30929840	Abdominal_fat_weight	region1inRegion2	68360
CNVR80	946	7	25785853	25888939	gain	1062	11625414	30929840	Abdominal_fat_weight	region1inRegion2	103086
CNVR81	949	7	26267062	26297673	gain	1062	11625414	30929840	Abdominal_fat_weight	region1inRegion2	30611
CNVR82	951	7	26600964	26608495	gain	1062	11625414	30929840	Abdominal_fat_weight	region1inRegion2	7531
CNVR83	957	7	28525787	28565312	gain	1062	11625414	30929840	Abdominal_fat_weight	region1inRegion2	39525
CNVR73	934	7	22207228	22236111	gain	16891	11625414	34042440	Average_daily_gain	region1inRegion2	28883
CNVR74	938	7	23307667	23658110	loss-gain	16891	11625414	34042440	Average_daily_gain	region1inRegion2	350443
CNVR75	939	7	23481211	23658110	loss-gain	16891	11625414	34042440	Average_daily_gain	region1inRegion2	176899
CNVR76	942	7	24648309	24653067	loss-gain	16891	11625414	34042440	Average_daily_gain	region1inRegion2	4758
CNVR77	943	7	24735665	24740397	gain	16891	11625414	34042440	Average_daily_gain	region1inRegion2	4732
CNVR78	944	7	25359190	25399904	gain	16891	11625414	34042440	Average_daily_gain	region1inRegion2	40714
CNVR79	945	7	25488796	25557156	gain	16891	11625414	34042440	Average_daily_gain	region1inRegion2	68360
CNVR80	946	7	25785853	25888939	gain	16891	11625414	34042440	Average_daily_gain	region1inRegion2	103086
CNVR81	949	7	26267062	26297673	gain	16891	11625414	34042440	Average_daily_gain	region1inRegion2	30611
CNVR82	951	7	26600964	26608495	gain	16891	11625414	34042440	Average_daily_gain	region1inRegion2	7531
CNVR83	957	7	28525787	28565312	gain	16891	11625414	34042440	Average_daily_gain	region1inRegion2	39525
CNVR73	934	7	22207228	22236111	gain	12777	11625414	36525998	Fat_protein_content	region1inRegion2	28883
CNVR74	938	7	23307667	23658110	loss-gain	12777	11625414	36525998	Fat_protein_content	region1inRegion2	350443
CNVR75	939	7	23481211	23658110	loss-gain	12777	11625414	36525998	Fat_protein_content	region1inRegion2	176899
CNVR76	942	7	24648309	24653067	loss-gain	12777	11625414	36525998	Fat_protein_content	region1inRegion2	4758
CNVR77	943	7	24735665	24740397	gain	12777	11625414	36525998	Fat_protein_content	region1inRegion2	4732
CNVR78	944	7	25359190	25399904	gain	12777	11625414	36525998	Fat_protein_content	region1inRegion2	40714
CNVR79	945	7	25488796	25557156	gain	12777	11625414	36525998	Fat_protein_content	region1inRegion2	68360
CNVR80	946	7	25785853	25888939	gain	12777	11625414	36525998	Fat_protein_content	region1inRegion2	103086
CNVR81	949	7	26267062	26297673	gain	12777	11625414	36525998	Fat_protein_content	region1inRegion2	30611
CNVR82	951	7	26600964	26608495	gain	12777	11625414	36525998	Fat_protein_content	region1inRegion2	7531
CNVR83	957	7	28525787	28565312	gain	12777	11625414	36525998	Fat_protein_content	region1inRegion2	39525
CNVR73	934	7	22207228	22236111	gain	12778	11625414	36525998	NADPH-generating_enzyme_activity	region1inRegion2	28883
CNVR74	938	7	23307667	23658110	loss-gain	12778	11625414	36525998	NADPH-generating_enzyme_activity	region1inRegion2	350443
CNVR75	939	7	23481211	23658110	loss-gain	12778	11625414	36525998	NADPH-generating_enzyme_activity	region1inRegion2	176899
CNVR76	942	7	24648309	24653067	loss-gain	12778	11625414	36525998	NADPH-generating_enzyme_activity	region1inRegion2	4758
CNVR77	943	7	24735665	24740397	gain	12778	11625414	36525998	NADPH-generating_enzyme_activity	region1inRegion2	4732
CNVR78	944	7	25359190	25399904	gain	12778	11625414	36525998	NADPH-generating_enzyme_activity	region1inRegion2	40714
CNVR79	945	7	25488796	25557156	gain	12778	11625414	36525998	NADPH-generating_enzyme_activity	region1inRegion2	68360
CNVR80	946	7	25785853	25888939	gain	12778	11625414	36525998	NADPH-generating_enzyme_activity	region1inRegion2	103086
CNVR81	949	7	26267062	26297673	gain	12778	11625414	36525998	NADPH-generating_enzyme_activity	region1inRegion2	30611
CNVR82	951	7	26600964	26608495	gain	12778	11625414	36525998	NADPH-generating_enzyme_activity	region1inRegion2	7531
CNVR83	957	7	28525787	28565312	gain	12778	11625414	36525998	NADPH-generating_enzyme_activity	region1inRegion2	39525
CNVR73	934	7	22207228	22236111	gain	18640	11625414	36993248	Fat_area_percentage_in_carcass	region1inRegion2	28883
CNVR74	938	7	23307667	23658110	loss-gain	18640	11625414	36993248	Fat_area_percentage_in_carcass	region1inRegion2	350443
CNVR75	939	7	23481211	23658110	loss-gain	18640	11625414	36993248	Fat_area_percentage_in_carcass	region1inRegion2	176899
CNVR76	942	7	24648309	24653067	loss-gain	18640	11625414	36993248	Fat_area_percentage_in_carcass	region1inRegion2	4758
CNVR77	943	7	24735665	24740397	gain	18640	11625414	36993248	Fat_area_percentage_in_carcass	region1inRegion2	4732
CNVR78	944	7	25359190	25399904	gain	18640	11625414	36993248	Fat_area_percentage_in_carcass	region1inRegion2	40714
CNVR79	945	7	25488796	25557156	gain	18640	11625414	36993248	Fat_area_percentage_in_carcass	region1inRegion2	68360
CNVR80	946	7	25785853	25888939	gain	18640	11625414	36993248	Fat_area_percentage_in_carcass	region1inRegion2	103086
CNVR81	949	7	26267062	26297673	gain	18640	11625414	36993248	Fat_area_percentage_in_carcass	region1inRegion2	30611
CNVR82	951	7	26600964	26608495	gain	18640	11625414	36993248	Fat_area_percentage_in_carcass	region1inRegion2	7531
CNVR83	957	7	28525787	28565312	gain	18640	11625414	36993248	Fat_area_percentage_in_carcass	region1inRegion2	39525
CNVR73	934	7	22207228	22236111	gain	5469	11625414	36993248	Lymphocyte_number	region1inRegion2	28883
CNVR74	938	7	23307667	23658110	loss-gain	5469	11625414	36993248	Lymphocyte_number	region1inRegion2	350443
CNVR75	939	7	23481211	23658110	loss-gain	5469	11625414	36993248	Lymphocyte_number	region1inRegion2	176899
CNVR76	942	7	24648309	24653067	loss-gain	5469	11625414	36993248	Lymphocyte_number	region1inRegion2	4758
CNVR77	943	7	24735665	24740397	gain	5469	11625414	36993248	Lymphocyte_number	region1inRegion2	4732
CNVR78	944	7	25359190	25399904	gain	5469	11625414	36993248	Lymphocyte_number	region1inRegion2	40714
CNVR79	945	7	25488796	25557156	gain	5469	11625414	36993248	Lymphocyte_number	region1inRegion2	68360
CNVR80	946	7	25785853	25888939	gain	5469	11625414	36993248	Lymphocyte_number	region1inRegion2	103086
CNVR81	949	7	26267062	26297673	gain	5469	11625414	36993248	Lymphocyte_number	region1inRegion2	30611
CNVR82	951	7	26600964	26608495	gain	5469	11625414	36993248	Lymphocyte_number	region1inRegion2	7531
CNVR83	957	7	28525787	28565312	gain	5469	11625414	36993248	Lymphocyte_number	region1inRegion2	39525
CNVR73	934	7	22207228	22236111	gain	5916	11625414	36993248	Time_spent_drinking	region1inRegion2	28883
CNVR74	938	7	23307667	23658110	loss-gain	5916	11625414	36993248	Time_spent_drinking	region1inRegion2	350443
CNVR75	939	7	23481211	23658110	loss-gain	5916	11625414	36993248	Time_spent_drinking	region1inRegion2	176899
CNVR76	942	7	24648309	24653067	loss-gain	5916	11625414	36993248	Time_spent_drinking	region1inRegion2	4758
CNVR77	943	7	24735665	24740397	gain	5916	11625414	36993248	Time_spent_drinking	region1inRegion2	4732
CNVR78	944	7	25359190	25399904	gain	5916	11625414	36993248	Time_spent_drinking	region1inRegion2	40714
CNVR79	945	7	25488796	25557156	gain	5916	11625414	36993248	Time_spent_drinking	region1inRegion2	68360
CNVR80	946	7	25785853	25888939	gain	5916	11625414	36993248	Time_spent_drinking	region1inRegion2	103086
CNVR81	949	7	26267062	26297673	gain	5916	11625414	36993248	Time_spent_drinking	region1inRegion2	30611
CNVR82	951	7	26600964	26608495	gain	5916	11625414	36993248	Time_spent_drinking	region1inRegion2	7531
CNVR83	957	7	28525787	28565312	gain	5916	11625414	36993248	Time_spent_drinking	region1inRegion2	39525
CNVR73	934	7	22207228	22236111	gain	5920	11625414	36993248	Time_spent_rooting	region1inRegion2	28883
CNVR74	938	7	23307667	23658110	loss-gain	5920	11625414	36993248	Time_spent_rooting	region1inRegion2	350443
CNVR75	939	7	23481211	23658110	loss-gain	5920	11625414	36993248	Time_spent_rooting	region1inRegion2	176899
CNVR76	942	7	24648309	24653067	loss-gain	5920	11625414	36993248	Time_spent_rooting	region1inRegion2	4758
CNVR77	943	7	24735665	24740397	gain	5920	11625414	36993248	Time_spent_rooting	region1inRegion2	4732
CNVR78	944	7	25359190	25399904	gain	5920	11625414	36993248	Time_spent_rooting	region1inRegion2	40714
CNVR79	945	7	25488796	25557156	gain	5920	11625414	36993248	Time_spent_rooting	region1inRegion2	68360
CNVR80	946	7	25785853	25888939	gain	5920	11625414	36993248	Time_spent_rooting	region1inRegion2	103086
CNVR81	949	7	26267062	26297673	gain	5920	11625414	36993248	Time_spent_rooting	region1inRegion2	30611
CNVR82	951	7	26600964	26608495	gain	5920	11625414	36993248	Time_spent_rooting	region1inRegion2	7531
CNVR83	957	7	28525787	28565312	gain	5920	11625414	36993248	Time_spent_rooting	region1inRegion2	39525
CNVR73	934	7	22207228	22236111	gain	5925	11625414	36993248	Time_spent_socializing	region1inRegion2	28883
CNVR74	938	7	23307667	23658110	loss-gain	5925	11625414	36993248	Time_spent_socializing	region1inRegion2	350443
CNVR75	939	7	23481211	23658110	loss-gain	5925	11625414	36993248	Time_spent_socializing	region1inRegion2	176899
CNVR76	942	7	24648309	24653067	loss-gain	5925	11625414	36993248	Time_spent_socializing	region1inRegion2	4758
CNVR77	943	7	24735665	24740397	gain	5925	11625414	36993248	Time_spent_socializing	region1inRegion2	4732
CNVR78	944	7	25359190	25399904	gain	5925	11625414	36993248	Time_spent_socializing	region1inRegion2	40714
CNVR79	945	7	25488796	25557156	gain	5925	11625414	36993248	Time_spent_socializing	region1inRegion2	68360
CNVR80	946	7	25785853	25888939	gain	5925	11625414	36993248	Time_spent_socializing	region1inRegion2	103086
CNVR81	949	7	26267062	26297673	gain	5925	11625414	36993248	Time_spent_socializing	region1inRegion2	30611
CNVR82	951	7	26600964	26608495	gain	5925	11625414	36993248	Time_spent_socializing	region1inRegion2	7531
CNVR83	957	7	28525787	28565312	gain	5925	11625414	36993248	Time_spent_socializing	region1inRegion2	39525
CNVR73	934	7	22207228	22236111	gain	6349	11625414	36993248	Alkaline_phosphatase_activity	region1inRegion2	28883
CNVR74	938	7	23307667	23658110	loss-gain	6349	11625414	36993248	Alkaline_phosphatase_activity	region1inRegion2	350443
CNVR75	939	7	23481211	23658110	loss-gain	6349	11625414	36993248	Alkaline_phosphatase_activity	region1inRegion2	176899
CNVR76	942	7	24648309	24653067	loss-gain	6349	11625414	36993248	Alkaline_phosphatase_activity	region1inRegion2	4758
CNVR77	943	7	24735665	24740397	gain	6349	11625414	36993248	Alkaline_phosphatase_activity	region1inRegion2	4732
CNVR78	944	7	25359190	25399904	gain	6349	11625414	36993248	Alkaline_phosphatase_activity	region1inRegion2	40714
CNVR79	945	7	25488796	25557156	gain	6349	11625414	36993248	Alkaline_phosphatase_activity	region1inRegion2	68360
CNVR80	946	7	25785853	25888939	gain	6349	11625414	36993248	Alkaline_phosphatase_activity	region1inRegion2	103086
CNVR81	949	7	26267062	26297673	gain	6349	11625414	36993248	Alkaline_phosphatase_activity	region1inRegion2	30611
CNVR82	951	7	26600964	26608495	gain	6349	11625414	36993248	Alkaline_phosphatase_activity	region1inRegion2	7531
CNVR83	957	7	28525787	28565312	gain	6349	11625414	36993248	Alkaline_phosphatase_activity	region1inRegion2	39525
CNVR73	934	7	22207228	22236111	gain	9596	11625414	38992356	Hemolytic_complement_activity_(alternative_pathway)	region1inRegion2	28883
CNVR74	938	7	23307667	23658110	loss-gain	9596	11625414	38992356	Hemolytic_complement_activity_(alternative_pathway)	region1inRegion2	350443
CNVR75	939	7	23481211	23658110	loss-gain	9596	11625414	38992356	Hemolytic_complement_activity_(alternative_pathway)	region1inRegion2	176899
CNVR76	942	7	24648309	24653067	loss-gain	9596	11625414	38992356	Hemolytic_complement_activity_(alternative_pathway)	region1inRegion2	4758
CNVR77	943	7	24735665	24740397	gain	9596	11625414	38992356	Hemolytic_complement_activity_(alternative_pathway)	region1inRegion2	4732
CNVR78	944	7	25359190	25399904	gain	9596	11625414	38992356	Hemolytic_complement_activity_(alternative_pathway)	region1inRegion2	40714

CNVR79	945	7	25488796	25557156	gain	9596	11625414	38992356	Hemolytic_complement_activity_(alternative_pathway)	region1inRegion2	68360
CNVR80	946	7	25785853	25888939	gain	9596	11625414	38992356	Hemolytic_complement_activity_(alternative_pathway)	region1inRegion2	103086
CNVR81	949	7	26267062	26297673	gain	9596	11625414	38992356	Hemolytic_complement_activity_(alternative_pathway)	region1inRegion2	30611
CNVR82	951	7	26600964	26608495	gain	9596	11625414	38992356	Hemolytic_complement_activity_(alternative_pathway)	region1inRegion2	7531
CNVR83	957	7	28525787	28565312	gain	9596	11625414	38992356	Hemolytic_complement_activity_(alternative_pathway)	region1inRegion2	39525
CNVR73	934	7	22207228	22236111	gain	12775	11625414	45102432	NADP-malate_dehydrogenase_activity	region1inRegion2	28883
CNVR74	938	7	23307667	23658110	loss-gain	12775	11625414	45102432	NADP-malate_dehydrogenase_activity	region1inRegion2	350443
CNVR75	939	7	23481211	23658110	loss-gain	12775	11625414	45102432	NADP-malate_dehydrogenase_activity	region1inRegion2	176899
CNVR76	942	7	24648309	24653067	loss-gain	12775	11625414	45102432	NADP-malate_dehydrogenase_activity	region1inRegion2	4758
CNVR77	943	7	24735665	24740397	gain	12775	11625414	45102432	NADP-malate_dehydrogenase_activity	region1inRegion2	4732
CNVR78	944	7	25359190	25399904	gain	12775	11625414	45102432	NADP-malate_dehydrogenase_activity	region1inRegion2	40714
CNVR79	945	7	25488796	25557156	gain	12775	11625414	45102432	NADP-malate_dehydrogenase_activity	region1inRegion2	68360
CNVR80	946	7	25785853	25888939	gain	12775	11625414	45102432	NADP-malate_dehydrogenase_activity	region1inRegion2	103086
CNVR81	949	7	26267062	26297673	gain	12775	11625414	45102432	NADP-malate_dehydrogenase_activity	region1inRegion2	30611
CNVR82	951	7	26600964	26608495	gain	12775	11625414	45102432	NADP-malate_dehydrogenase_activity	region1inRegion2	7531
CNVR83	957	7	28525787	28565312	gain	12775	11625414	45102432	NADP-malate_dehydrogenase_activity	region1inRegion2	39525
CNVR73	934	7	22207228	22236111	gain	12781	11625414	45102432	NADP-malate_dehydrogenase_activity	region1inRegion2	28883
CNVR74	938	7	23307667	23658110	loss-gain	12781	11625414	45102432	NADP-malate_dehydrogenase_activity	region1inRegion2	350443
CNVR75	939	7	23481211	23658110	loss-gain	12781	11625414	45102432	NADP-malate_dehydrogenase_activity	region1inRegion2	176899
CNVR76	942	7	24648309	24653067	loss-gain	12781	11625414	45102432	NADP-malate_dehydrogenase_activity	region1inRegion2	4758
CNVR77	943	7	24735665	24740397	gain	12781	11625414	45102432	NADP-malate_dehydrogenase_activity	region1inRegion2	4732
CNVR78	944	7	25359190	25399904	gain	12781	11625414	45102432	NADP-malate_dehydrogenase_activity	region1inRegion2	40714
CNVR79	945	7	25488796	25557156	gain	12781	11625414	45102432	NADP-malate_dehydrogenase_activity	region1inRegion2	68360
CNVR80	946	7	25785853	25888939	gain	12781	11625414	45102432	NADP-malate_dehydrogenase_activity	region1inRegion2	103086
CNVR81	949	7	26267062	26297673	gain	12781	11625414	45102432	NADP-malate_dehydrogenase_activity	region1inRegion2	30611
CNVR82	951	7	26600964	26608495	gain	12781	11625414	45102432	NADP-malate_dehydrogenase_activity	region1inRegion2	7531
CNVR83	957	7	28525787	28565312	gain	12781	11625414	45102432	NADP-malate_dehydrogenase_activity	region1inRegion2	39525
CNVR73	934	7	22207228	22236111	gain	12807	11625414	45102432	Adipocyte_diameter	region1inRegion2	28883
CNVR74	938	7	23307667	23658110	loss-gain	12807	11625414	45102432	Adipocyte_diameter	region1inRegion2	350443
CNVR75	939	7	23481211	23658110	loss-gain	12807	11625414	45102432	Adipocyte_diameter	region1inRegion2	176899
CNVR76	942	7	24648309	24653067	loss-gain	12807	11625414	45102432	Adipocyte_diameter	region1inRegion2	4758
CNVR77	943	7	24735665	24740397	gain	12807	11625414	45102432	Adipocyte_diameter	region1inRegion2	4732
CNVR78	944	7	25359190	25399904	gain	12807	11625414	45102432	Adipocyte_diameter	region1inRegion2	40714
CNVR79	945	7	25488796	25557156	gain	12807	11625414	45102432	Adipocyte_diameter	region1inRegion2	68360
CNVR80	946	7	25785853	25888939	gain	12807	11625414	45102432	Adipocyte_diameter	region1inRegion2	103086
CNVR81	949	7	26267062	26297673	gain	12807	11625414	45102432	Adipocyte_diameter	region1inRegion2	30611
CNVR82	951	7	26600964	26608495	gain	12807	11625414	45102432	Adipocyte_diameter	region1inRegion2	7531
CNVR83	957	7	28525787	28565312	gain	12807	11625414	45102432	Adipocyte_diameter	region1inRegion2	39525
CNVR73	934	7	22207228	22236111	gain	166	11625414	45102432	Smell_intensity_-_fat	region1inRegion2	28883
CNVR74	938	7	23307667	23658110	loss-gain	166	11625414	45102432	Smell_intensity_-_fat	region1inRegion2	350443
CNVR75	939	7	23481211	23658110	loss-gain	166	11625414	45102432	Smell_intensity_-_fat	region1inRegion2	176899
CNVR76	942	7	24648309	24653067	loss-gain	166	11625414	45102432	Smell_intensity_-_fat	region1inRegion2	4758
CNVR77	943	7	24735665	24740397	gain	166	11625414	45102432	Smell_intensity_-_fat	region1inRegion2	4732
CNVR78	944	7	25359190	25399904	gain	166	11625414	45102432	Smell_intensity_-_fat	region1inRegion2	40714
CNVR79	945	7	25488796	25557156	gain	166	11625414	45102432	Smell_intensity_-_fat	region1inRegion2	68360
CNVR80	946	7	25785853	25888939	gain	166	11625414	45102432	Smell_intensity_-_fat	region1inRegion2	103086
CNVR81	949	7	26267062	26297673	gain	166	11625414	45102432	Smell_intensity_-_fat	region1inRegion2	30611
CNVR82	951	7	26600964	26608495	gain	166	11625414	45102432	Smell_intensity_-_fat	region1inRegion2	7531
CNVR83	957	7	28525787	28565312	gain	166	11625414	45102432	Smell_intensity_-_fat	region1inRegion2	39525
CNVR73	934	7	22207228	22236111	gain	295	11625414	45102432	Meat_color-a	region1inRegion2	28883
CNVR74	938	7	23307667	23658110	loss-gain	295	11625414	45102432	Meat_color-a	region1inRegion2	350443
CNVR75	939	7	23481211	23658110	loss-gain	295	11625414	45102432	Meat_color-a	region1inRegion2	176899
CNVR76	942	7	24648309	24653067	loss-gain	295	11625414	45102432	Meat_color-a	region1inRegion2	4758
CNVR77	943	7	24735665	24740397	gain	295	11625414	45102432	Meat_color-a	region1inRegion2	4732
CNVR78	944	7	25359190	25399904	gain	295	11625414	45102432	Meat_color-a	region1inRegion2	40714
CNVR79	945	7	25488796	25557156	gain	295	11625414	45102432	Meat_color-a	region1inRegion2	68360
CNVR80	946	7	25785853	25888939	gain	295	11625414	45102432	Meat_color-a	region1inRegion2	103086
CNVR81	949	7	26267062	26297673	gain	295	11625414	45102432	Meat_color-a	region1inRegion2	30611
CNVR82	951	7	26600964	26608495	gain	295	11625414	45102432	Meat_color-a	region1inRegion2	7531
CNVR83	957	7	28525787	28565312	gain	295	11625414	45102432	Meat_color-a	region1inRegion2	39525
CNVR73	934	7	22207228	22236111	gain	312	11625414	45102432	Average_backfat_thickness	region1inRegion2	28883
CNVR74	938	7	23307667	23658110	loss-gain	312	11625414	45102432	Average_backfat_thickness	region1inRegion2	350443
CNVR75	939	7	23481211	23658110	loss-gain	312	11625414	45102432	Average_backfat_thickness	region1inRegion2	176899
CNVR76	942	7	24648309	24653067	loss-gain	312	11625414	45102432	Average_backfat_thickness	region1inRegion2	4758
CNVR77	943	7	24735665	24740397	gain	312	11625414	45102432	Average_backfat_thickness	region1inRegion2	4732
CNVR78	944	7	25359190	25399904	gain	312	11625414	45102432	Average_backfat_thickness	region1inRegion2	40714
CNVR79	945	7	25488796	25557156	gain	312	11625414	45102432	Average_backfat_thickness	region1inRegion2	68360
CNVR80	946	7	25785853	25888939	gain	312	11625414	45102432	Average_backfat_thickness	region1inRegion2	103086
CNVR81	949	7	26267062	26297673	gain	312	11625414	45102432	Average_backfat_thickness	region1inRegion2	30611
CNVR82	951	7	26600964	26608495	gain	312	11625414	45102432	Average_backfat_thickness	region1inRegion2	7531
CNVR83	957	7	28525787	28565312	gain	312	11625414	45102432	Average_backfat_thickness	region1inRegion2	39525
CNVR73	934	7	22207228	22236111	gain	4142	11625414	45102432	Average_daily_gain	region1inRegion2	28883
CNVR74	938	7	23307667	23658110	loss-gain	4142	11625414	45102432	Average_daily_gain	region1inRegion2	350443
CNVR75	939	7	23481211	23658110	loss-gain	4142	11625414	45102432	Average_daily_gain	region1inRegion2	176899
CNVR76	942	7	24648309	24653067	loss-gain	4142	11625414	45102432	Average_daily_gain	region1inRegion2	4758
CNVR77	943	7	24735665	24740397	gain	4142	11625414	45102432	Average_daily_gain	region1inRegion2	4732
CNVR78	944	7	25359190	25399904	gain	4142	11625414	45102432	Average_daily_gain	region1inRegion2	40714
CNVR79	945	7	25488796	25557156	gain	4142	11625414	45102432	Average_daily_gain	region1inRegion2	68360
CNVR80	946	7	25785853	25888939	gain	4142	11625414	45102432	Average_daily_gain	region1inRegion2	103086
CNVR81	949	7	26267062	26297673	gain	4142	11625414	45102432	Average_daily_gain	region1inRegion2	30611
CNVR82	951	7	26600964	26608495	gain	4142	11625414	45102432	Average_daily_gain	region1inRegion2	7531
CNVR83	957	7	28525787	28565312	gain	4142	11625414	45102432	Average_daily_gain	region1inRegion2	39525
CNVR73	934	7	22207228	22236111	gain	4143	11625414	45102432	Average_daily_gain	region1inRegion2	28883
CNVR74	938	7	23307667	23658110	loss-gain	4143	11625414	45102432	Average_daily_gain	region1inRegion2	350443
CNVR75	939	7	23481211	23658110	loss-gain	4143	11625414	45102432	Average_daily_gain	region1inRegion2	176899
CNVR76	942	7	24648309	24653067	loss-gain	4143	11625414	45102432	Average_daily_gain	region1inRegion2	4758
CNVR77	943	7	24735665	24740397	gain	4143	11625414	45102432	Average_daily_gain	region1inRegion2	4732
CNVR78	944	7	25359190	25399904	gain	4143	11625414	45102432	Average_daily_gain	region1inRegion2	40714
CNVR79	945	7	25488796	25557156	gain	4143	11625414	45102432	Average_daily_gain	region1inRegion2	68360
CNVR80	946	7	25785853	25888939	gain	4143	11625414	45102432	Average_daily_gain	region1inRegion2	103086
CNVR81	949	7	26267062	26297673	gain	4143	11625414	45102432	Average_daily_gain	region1inRegion2	30611
CNVR82	951	7	26600964	26608495	gain	4143	11625414	45102432	Average_daily_gain	region1inRegion2	7531
CNVR83	957	7	28525787	28565312	gain	4143	11625414	45102432	Average_daily_gain	region1inRegion2	39525
CNVR73	934	7	22207228	22236111	gain	4144	11625414	45102432	Average_daily_gain	region1inRegion2	28883
CNVR74	938	7	23307667	23658110	loss-gain	4144	11625414	45102432	Average_daily_gain	region1inRegion2	350443
CNVR75	939	7	23481211	23658110	loss-gain	4144	11625414	45102432	Average_daily_gain	region1inRegion2	176899
CNVR76	942	7	24648309	24653067	loss-gain	4144	11625414	45102432	Average_daily_gain	region1inRegion2	4758
CNVR77	943	7	24735665	24740397	gain	4144	11625414	45102432	Average_daily_gain	region1inRegion2	4732
CNVR78	944	7	25359190	25399904	gain	4144	11625414	45102432	Average_daily_gain	region1inRegion2	40714
CNVR79	945	7	25488796	25557156	gain	4144	11625414	45102432	Average_daily_gain	region1inRegion2	68360
CNVR80	946	7	25785853	25888939	gain	4144	11625414	45102432	Average_daily_gain	region1inRegion2	103086
CNVR81	949	7	26267062	26297673	gain	4144	11625414	45102432	Average_daily_gain	region1inRegion2	30611
CNVR82	951	7	26600964	26608495	gain	4144	11625414	45102432	Average_daily_gain	region1inRegion2	7531
CNVR83	957	7	28525787	28565312	gain	4144	11625414	45102432	Average_daily_gain	region1inRegion2	39525
CNVR73	934	7	22207228	22236111	gain	4145	11625414	45102432	PH_for_Longissmus_dorsi	region1inRegion2	28883
CNVR74	938	7	23307667	23658110	loss-gain	4145	11625414	45102432	PH_for_Longissmus_dorsi	region1inRegion2	350443
CNVR75	939	7	23481211	23658110	loss-gain	4145	11625414	45102432	PH_for_Longissmus_dorsi	region1inRegion2	176899
CNVR76	942	7	24648309	24653067	loss-gain	4145	11625414	45102432	PH_for_Longissmus_dorsi	region1inRegion2	4758
CNVR77	943	7	24735665	24740397	gain	4145	11625414	45102432	PH_for_Longissmus_dorsi	region1inRegion2	4732
CNVR78	944	7	25359190	25399904	gain	4145	11625414	45102432	PH_for_Longissmus_dorsi	region1inRegion2	40714
CNVR79	945	7	25488796	25557156	gain	4145	11625414	45102432	PH_for_Longissmus_dorsi	region1inRegion2	68360
CNVR80	946	7	25785853	25888939	gain	4145	11625414	45102432	PH_for_Longissmus_dorsi	region1inRegion2	103086
CNVR81	949	7	26267062	26297673	gain	4145	11625414	45102432	PH_for_Longissmus_dorsi	region1inRegion2	30611
CNVR82	951	7	26600964	26608495	gain	4145	11625414	45102432	PH_for_Longissmus_dorsi	region1inRegion2	7531
CNVR83	957	7	28525787	28565312	gain	4145	11625414	45102432	PH_for_Longissmus_dorsi	region1inRegion2	39525
CNVR73	934	7	22207228	22236111	gain	4146	11625414	45102432	pH_for_Semimembranosus	region1inRegion2	28883
CNVR74	938	7	23307667	23658110	loss-gain	4146	11625414	45102432	pH_for_Semimembranosus	region1inRegion2	350443
CNVR75	939	7	23481211	23658110	loss-gain	4146	11625414	45102432	pH_for_Semimembranosus	region1inRegion2	176899
CNVR76	942	7	24648309	24653067	loss-gain	4146	11625414	45102432	pH_for_Semimembranosus	region1inRegion2	4758
CNVR77	943	7	24735665	24740397	gain	4146	11625414	45102432	pH_for_Semimembranosus	region1inRegion2	4732
CNVR78	944	7	25359190	25399904	gain	4146	11625414	45102432	pH_for_Semimembranosus	region1inRegion2	40714
CNVR79	945	7	25488796	25557156	gain	4146	11625414	45102432	pH_for_Semimembranosus	region1inRegion2	68360

CNVR80	946	7	25785853	25888939	gain	4146	11625414	45102432	pH_for_Semimembranosus	region1inRegion2	103086
CNVR81	949	7	26267062	26297673	gain	4146	11625414	45102432	pH_for_Semimembranosus	region1inRegion2	30611
CNVR82	951	7	26600964	26608495	gain	4146	11625414	45102432	pH_for_Semimembranosus	region1inRegion2	7531
CNVR83	957	7	28525787	28565312	gain	4146	11625414	45102432	pH_for_Semimembranosus	region1inRegion2	39525
CNVR73	934	7	22207228	22236111	gain	4147	11625414	45102432	Conductivity_45_minutes_post-mortem	region1inRegion2	28883
CNVR74	938	7	23307667	23658110	loss-gain	4147	11625414	45102432	Conductivity_45_minutes_post-mortem	region1inRegion2	350443
CNVR75	939	7	23481211	23658110	loss-gain	4147	11625414	45102432	Conductivity_45_minutes_post-mortem	region1inRegion2	176899
CNVR76	942	7	24648309	24653067	loss-gain	4147	11625414	45102432	Conductivity_45_minutes_post-mortem	region1inRegion2	4758
CNVR77	943	7	24735665	24740397	gain	4147	11625414	45102432	Conductivity_45_minutes_post-mortem	region1inRegion2	4732
CNVR78	944	7	25359190	25399904	gain	4147	11625414	45102432	Conductivity_45_minutes_post-mortem	region1inRegion2	40714
CNVR79	945	7	25488796	25557156	gain	4147	11625414	45102432	Conductivity_45_minutes_post-mortem	region1inRegion2	68360
CNVR80	946	7	25785853	25888939	gain	4147	11625414	45102432	Conductivity_45_minutes_post-mortem	region1inRegion2	103086
CNVR81	949	7	26267062	26297673	gain	4147	11625414	45102432	Conductivity_45_minutes_post-mortem	region1inRegion2	30611
CNVR82	951	7	26600964	26608495	gain	4147	11625414	45102432	Conductivity_45_minutes_post-mortem	region1inRegion2	7531
CNVR83	957	7	28525787	28565312	gain	4147	11625414	45102432	Conductivity_45_minutes_post-mortem	region1inRegion2	39525
CNVR73	934	7	22207228	22236111	gain	4148	11625414	45102432	Conductivity_45_minutes_post-mortem	region1inRegion2	28883
CNVR74	938	7	23307667	23658110	loss-gain	4148	11625414	45102432	Conductivity_45_minutes_post-mortem	region1inRegion2	350443
CNVR75	939	7	23481211	23658110	loss-gain	4148	11625414	45102432	Conductivity_45_minutes_post-mortem	region1inRegion2	176899
CNVR76	942	7	24648309	24653067	loss-gain	4148	11625414	45102432	Conductivity_45_minutes_post-mortem	region1inRegion2	4758
CNVR77	943	7	24735665	24740397	gain	4148	11625414	45102432	Conductivity_45_minutes_post-mortem	region1inRegion2	4732
CNVR78	944	7	25359190	25399904	gain	4148	11625414	45102432	Conductivity_45_minutes_post-mortem	region1inRegion2	40714
CNVR79	945	7	25488796	25557156	gain	4148	11625414	45102432	Conductivity_45_minutes_post-mortem	region1inRegion2	68360
CNVR80	946	7	25785853	25888939	gain	4148	11625414	45102432	Conductivity_45_minutes_post-mortem	region1inRegion2	103086
CNVR81	949	7	26267062	26297673	gain	4148	11625414	45102432	Conductivity_45_minutes_post-mortem	region1inRegion2	30611
CNVR82	951	7	26600964	26608495	gain	4148	11625414	45102432	Conductivity_45_minutes_post-mortem	region1inRegion2	7531
CNVR83	957	7	28525787	28565312	gain	4148	11625414	45102432	Conductivity_45_minutes_post-mortem	region1inRegion2	39525
CNVR73	934	7	22207228	22236111	gain	4171	11625414	45102432	Average_daily_gain	region1inRegion2	28883
CNVR74	938	7	23307667	23658110	loss-gain	4171	11625414	45102432	Average_daily_gain	region1inRegion2	350443
CNVR75	939	7	23481211	23658110	loss-gain	4171	11625414	45102432	Average_daily_gain	region1inRegion2	176899
CNVR76	942	7	24648309	24653067	loss-gain	4171	11625414	45102432	Average_daily_gain	region1inRegion2	4758
CNVR77	943	7	24735665	24740397	gain	4171	11625414	45102432	Average_daily_gain	region1inRegion2	4732
CNVR78	944	7	25359190	25399904	gain	4171	11625414	45102432	Average_daily_gain	region1inRegion2	40714
CNVR79	945	7	25488796	25557156	gain	4171	11625414	45102432	Average_daily_gain	region1inRegion2	68360
CNVR80	946	7	25785853	25888939	gain	4171	11625414	45102432	Average_daily_gain	region1inRegion2	103086
CNVR81	949	7	26267062	26297673	gain	4171	11625414	45102432	Average_daily_gain	region1inRegion2	30611
CNVR82	951	7	26600964	26608495	gain	4171	11625414	45102432	Average_daily_gain	region1inRegion2	7531
CNVR83	957	7	28525787	28565312	gain	4171	11625414	45102432	Average_daily_gain	region1inRegion2	39525
CNVR73	934	7	22207228	22236111	gain	4172	11625414	45102432	Average_daily_gain	region1inRegion2	28883
CNVR74	938	7	23307667	23658110	loss-gain	4172	11625414	45102432	Average_daily_gain	region1inRegion2	350443
CNVR75	939	7	23481211	23658110	loss-gain	4172	11625414	45102432	Average_daily_gain	region1inRegion2	176899
CNVR76	942	7	24648309	24653067	loss-gain	4172	11625414	45102432	Average_daily_gain	region1inRegion2	4758
CNVR77	943	7	24735665	24740397	gain	4172	11625414	45102432	Average_daily_gain	region1inRegion2	4732
CNVR78	944	7	25359190	25399904	gain	4172	11625414	45102432	Average_daily_gain	region1inRegion2	40714
CNVR79	945	7	25488796	25557156	gain	4172	11625414	45102432	Average_daily_gain	region1inRegion2	68360
CNVR80	946	7	25785853	25888939	gain	4172	11625414	45102432	Average_daily_gain	region1inRegion2	103086
CNVR81	949	7	26267062	26297673	gain	4172	11625414	45102432	Average_daily_gain	region1inRegion2	30611
CNVR82	951	7	26600964	26608495	gain	4172	11625414	45102432	Average_daily_gain	region1inRegion2	7531
CNVR83	957	7	28525787	28565312	gain	4172	11625414	45102432	Average_daily_gain	region1inRegion2	39525
CNVR73	934	7	22207228	22236111	gain	4180	11625414	45102432	PH_for_Longissmus_dorsi	region1inRegion2	28883
CNVR74	938	7	23307667	23658110	loss-gain	4180	11625414	45102432	PH_for_Longissmus_dorsi	region1inRegion2	350443
CNVR75	939	7	23481211	23658110	loss-gain	4180	11625414	45102432	PH_for_Longissmus_dorsi	region1inRegion2	176899
CNVR76	942	7	24648309	24653067	loss-gain	4180	11625414	45102432	PH_for_Longissmus_dorsi	region1inRegion2	4758
CNVR77	943	7	24735665	24740397	gain	4180	11625414	45102432	PH_for_Longissmus_dorsi	region1inRegion2	4732
CNVR78	944	7	25359190	25399904	gain	4180	11625414	45102432	PH_for_Longissmus_dorsi	region1inRegion2	40714
CNVR79	945	7	25488796	25557156	gain	4180	11625414	45102432	PH_for_Longissmus_dorsi	region1inRegion2	68360
CNVR80	946	7	25785853	25888939	gain	4180	11625414	45102432	PH_for_Longissmus_dorsi	region1inRegion2	103086
CNVR81	949	7	26267062	26297673	gain	4180	11625414	45102432	PH_for_Longissmus_dorsi	region1inRegion2	30611
CNVR82	951	7	26600964	26608495	gain	4180	11625414	45102432	PH_for_Longissmus_dorsi	region1inRegion2	7531
CNVR83	957	7	28525787	28565312	gain	4180	11625414	45102432	PH_for_Longissmus_dorsi	region1inRegion2	39525
CNVR73	934	7	22207228	22236111	gain	4181	11625414	45102432	PH_for_Longissmus_dorsi	region1inRegion2	28883
CNVR74	938	7	23307667	23658110	loss-gain	4181	11625414	45102432	PH_for_Longissmus_dorsi	region1inRegion2	350443
CNVR75	939	7	23481211	23658110	loss-gain	4181	11625414	45102432	PH_for_Longissmus_dorsi	region1inRegion2	176899
CNVR76	942	7	24648309	24653067	loss-gain	4181	11625414	45102432	PH_for_Longissmus_dorsi	region1inRegion2	4758
CNVR77	943	7	24735665	24740397	gain	4181	11625414	45102432	PH_for_Longissmus_dorsi	region1inRegion2	4732
CNVR78	944	7	25359190	25399904	gain	4181	11625414	45102432	PH_for_Longissmus_dorsi	region1inRegion2	40714
CNVR79	945	7	25488796	25557156	gain	4181	11625414	45102432	PH_for_Longissmus_dorsi	region1inRegion2	68360
CNVR80	946	7	25785853	25888939	gain	4181	11625414	45102432	PH_for_Longissmus_dorsi	region1inRegion2	103086
CNVR81	949	7	26267062	26297673	gain	4181	11625414	45102432	PH_for_Longissmus_dorsi	region1inRegion2	30611
CNVR82	951	7	26600964	26608495	gain	4181	11625414	45102432	PH_for_Longissmus_dorsi	region1inRegion2	7531
CNVR83	957	7	28525787	28565312	gain	4181	11625414	45102432	PH_for_Longissmus_dorsi	region1inRegion2	39525
CNVR73	934	7	22207228	22236111	gain	4182	11625414	45102432	pH_for_Semimembranosus	region1inRegion2	28883
CNVR74	938	7	23307667	23658110	loss-gain	4182	11625414	45102432	pH_for_Semimembranosus	region1inRegion2	350443
CNVR75	939	7	23481211	23658110	loss-gain	4182	11625414	45102432	pH_for_Semimembranosus	region1inRegion2	176899
CNVR76	942	7	24648309	24653067	loss-gain	4182	11625414	45102432	pH_for_Semimembranosus	region1inRegion2	4758
CNVR77	943	7	24735665	24740397	gain	4182	11625414	45102432	pH_for_Semimembranosus	region1inRegion2	4732
CNVR78	944	7	25359190	25399904	gain	4182	11625414	45102432	pH_for_Semimembranosus	region1inRegion2	40714
CNVR79	945	7	25488796	25557156	gain	4182	11625414	45102432	pH_for_Semimembranosus	region1inRegion2	68360
CNVR80	946	7	25785853	25888939	gain	4182	11625414	45102432	pH_for_Semimembranosus	region1inRegion2	103086
CNVR81	949	7	26267062	26297673	gain	4182	11625414	45102432	pH_for_Semimembranosus	region1inRegion2	30611
CNVR82	951	7	26600964	26608495	gain	4182	11625414	45102432	pH_for_Semimembranosus	region1inRegion2	7531
CNVR83	957	7	28525787	28565312	gain	4182	11625414	45102432	pH_for_Semimembranosus	region1inRegion2	39525
CNVR73	934	7	22207228	22236111	gain	4183	11625414	45102432	Conductivity_45_minutes_post-mortem	region1inRegion2	28883
CNVR74	938	7	23307667	23658110	loss-gain	4183	11625414	45102432	Conductivity_45_minutes_post-mortem	region1inRegion2	350443
CNVR75	939	7	23481211	23658110	loss-gain	4183	11625414	45102432	Conductivity_45_minutes_post-mortem	region1inRegion2	176899
CNVR76	942	7	24648309	24653067	loss-gain	4183	11625414	45102432	Conductivity_45_minutes_post-mortem	region1inRegion2	4758
CNVR77	943	7	24735665	24740397	gain	4183	11625414	45102432	Conductivity_45_minutes_post-mortem	region1inRegion2	4732
CNVR78	944	7	25359190	25399904	gain	4183	11625414	45102432	Conductivity_45_minutes_post-mortem	region1inRegion2	40714
CNVR79	945	7	25488796	25557156	gain	4183	11625414	45102432	Conductivity_45_minutes_post-mortem	region1inRegion2	68360
CNVR80	946	7	25785853	25888939	gain	4183	11625414	45102432	Conductivity_45_minutes_post-mortem	region1inRegion2	103086
CNVR81	949	7	26267062	26297673	gain	4183	11625414	45102432	Conductivity_45_minutes_post-mortem	region1inRegion2	30611
CNVR82	951	7	26600964	26608495	gain	4183	11625414	45102432	Conductivity_45_minutes_post-mortem	region1inRegion2	7531
CNVR83	957	7	28525787	28565312	gain	4183	11625414	45102432	Conductivity_45_minutes_post-mortem	region1inRegion2	39525
CNVR73	934	7	22207228	22236111	gain	4184	11625414	45102432	Conductivity_45_minutes_post-mortem	region1inRegion2	28883
CNVR74	938	7	23307667	23658110	loss-gain	4184	11625414	45102432	Conductivity_45_minutes_post-mortem	region1inRegion2	350443
CNVR75	939	7	23481211	23658110	loss-gain	4184	11625414	45102432	Conductivity_45_minutes_post-mortem	region1inRegion2	176899
CNVR76	942	7	24648309	24653067	loss-gain	4184	11625414	45102432	Conductivity_45_minutes_post-mortem	region1inRegion2	4758
CNVR77	943	7	24735665	24740397	gain	4184	11625414	45102432	Conductivity_45_minutes_post-mortem	region1inRegion2	4732
CNVR78	944	7	25359190	25399904	gain	4184	11625414	45102432	Conductivity_45_minutes_post-mortem	region1inRegion2	40714
CNVR79	945	7	25488796	25557156	gain	4184	11625414	45102432	Conductivity_45_minutes_post-mortem	region1inRegion2	68360
CNVR80	946	7	25785853	25888939	gain	4184	11625414	45102432	Conductivity_45_minutes_post-mortem	region1inRegion2	103086
CNVR81	949	7	26267062	26297673	gain	4184	11625414	45102432	Conductivity_45_minutes_post-mortem	region1inRegion2	30611
CNVR82	951	7	26600964	26608495	gain	4184	11625414	45102432	Conductivity_45_minutes_post-mortem	region1inRegion2	7531
CNVR83	957	7	28525787	28565312	gain	4184	11625414	45102432	Conductivity_45_minutes_post-mortem	region1inRegion2	39525
CNVR73	934	7	22207228	22236111	gain	4185	11625414	45102432	Carcass_weight_(hot)	region1inRegion2	28883
CNVR74	938	7	23307667	23658110	loss-gain	4185	11625414	45102432	Carcass_weight_(hot)	region1inRegion2	350443
CNVR75	939	7	23481211	23658110	loss-gain	4185	11625414	45102432	Carcass_weight_(hot)	region1inRegion2	176899
CNVR76	942	7	24648309	24653067	loss-gain	4185	11625414	45102432	Carcass_weight_(hot)	region1inRegion2	4758
CNVR77	943	7	24735665	24740397	gain	4185	11625414	45102432	Carcass_weight_(hot)	region1inRegion2	4732
CNVR78	944	7	25359190	25399904	gain	4185	11625414	45102432	Carcass_weight_(hot)	region1inRegion2	40714
CNVR79	945	7	25488796	25557156	gain	4185	11625414	45102432	Carcass_weight_(hot)	region1inRegion2	68360
CNVR80	946	7	25785853	25888939	gain	4185	11625414	45102432	Carcass_weight_(hot)	region1inRegion2	103086
CNVR81	949	7	26267062	26297673	gain	4185	11625414	45102432	Carcass_weight_(hot)	region1inRegion2	30611
CNVR82	951	7	26600964	26608495	gain	4185	11625414	45102432	Carcass_weight_(hot)	region1inRegion2	7531
CNVR83	957	7	28525787	28565312	gain	4185	11625414	45102432	Carcass_weight_(hot)	region1inRegion2	39525
CNVR73	934	7	22207228	22236111	gain	4186	11625414	45102432	pH_for_Semimembranosus	region1inRegion2	28883
CNVR74	938	7	23307667	23658110	loss-gain	4186	11625414	45102432	pH_for_Semimembranosus	region1inRegion2	350443
CNVR75	939	7	23481211	23658110	loss-gain	4186	11625414	45102432	pH_for_Semimembranosus	region1inRegion2	176899
CNVR76	942	7	24648309	24653067	loss-gain	4186	11625414	45102432	pH_for_Semimembranosus	region1inRegion2	4758
CNVR77	943	7	24735665	24740397	gain	4186	11625414	45102432	pH_for_Semimembranosus	region1inRegion2	4732
CNVR78	944	7	25359190	25399904	gain	4186	11625414	45102432	pH_for_Semimembranosus	region1inRegion2	40714
CNVR79	945	7	25488796	25557156	gain	4186	11625414	45102432	pH_for_Semimembranosus	region1inRegion2	68360
CNVR80	946	7	25785853	25888939	gain	4186	11625414	45102432	pH_for_Semimembranosus	region1inRegion2	103086

CNVR81	949	7	26267062	26297673	gain	4186	11625414	45102432	pH_for_Semimembranosus	region1inRegion2	30611
CNVR82	951	7	26600964	26608495	gain	4186	11625414	45102432	pH_for_Semimembranosus	region1inRegion2	7531
CNVR83	957	7	28525787	28565312	gain	4186	11625414	45102432	pH_for_Semimembranosus	region1inRegion2	39525
CNVR73	934	7	22207228	22236111	gain	4187	11625414	45102432	Conductivity_45_minutes_post-mortem	region1inRegion2	28883
CNVR74	938	7	23307667	23658110	loss-gain	4187	11625414	45102432	Conductivity_45_minutes_post-mortem	region1inRegion2	350443
CNVR75	939	7	23481211	23658110	loss-gain	4187	11625414	45102432	Conductivity_45_minutes_post-mortem	region1inRegion2	176899
CNVR76	942	7	24648309	24653067	loss-gain	4187	11625414	45102432	Conductivity_45_minutes_post-mortem	region1inRegion2	4758
CNVR77	943	7	24735665	24740397	gain	4187	11625414	45102432	Conductivity_45_minutes_post-mortem	region1inRegion2	4732
CNVR78	944	7	25359190	25399904	gain	4187	11625414	45102432	Conductivity_45_minutes_post-mortem	region1inRegion2	40714
CNVR79	945	7	25488796	25557156	gain	4187	11625414	45102432	Conductivity_45_minutes_post-mortem	region1inRegion2	68360
CNVR80	946	7	25785853	25888939	gain	4187	11625414	45102432	Conductivity_45_minutes_post-mortem	region1inRegion2	103086
CNVR81	949	7	26267062	26297673	gain	4187	11625414	45102432	Conductivity_45_minutes_post-mortem	region1inRegion2	30611
CNVR82	951	7	26600964	26608495	gain	4187	11625414	45102432	Conductivity_45_minutes_post-mortem	region1inRegion2	7531
CNVR83	957	7	28525787	28565312	gain	4187	11625414	45102432	Conductivity_45_minutes_post-mortem	region1inRegion2	39525
CNVR73	934	7	22207228	22236111	gain	4188	11625414	45102432	Conductivity_45_minutes_post-mortem	region1inRegion2	28883
CNVR74	938	7	23307667	23658110	loss-gain	4188	11625414	45102432	Conductivity_45_minutes_post-mortem	region1inRegion2	350443
CNVR75	939	7	23481211	23658110	loss-gain	4188	11625414	45102432	Conductivity_45_minutes_post-mortem	region1inRegion2	176899
CNVR76	942	7	24648309	24653067	loss-gain	4188	11625414	45102432	Conductivity_45_minutes_post-mortem	region1inRegion2	4758
CNVR77	943	7	24735665	24740397	gain	4188	11625414	45102432	Conductivity_45_minutes_post-mortem	region1inRegion2	4732
CNVR78	944	7	25359190	25399904	gain	4188	11625414	45102432	Conductivity_45_minutes_post-mortem	region1inRegion2	40714
CNVR79	945	7	25488796	25557156	gain	4188	11625414	45102432	Conductivity_45_minutes_post-mortem	region1inRegion2	68360
CNVR80	946	7	25785853	25888939	gain	4188	11625414	45102432	Conductivity_45_minutes_post-mortem	region1inRegion2	103086
CNVR81	949	7	26267062	26297673	gain	4188	11625414	45102432	Conductivity_45_minutes_post-mortem	region1inRegion2	30611
CNVR82	951	7	26600964	26608495	gain	4188	11625414	45102432	Conductivity_45_minutes_post-mortem	region1inRegion2	7531
CNVR83	957	7	28525787	28565312	gain	4188	11625414	45102432	Conductivity_45_minutes_post-mortem	region1inRegion2	39525
CNVR73	934	7	22207228	22236111	gain	5943	11625414	45102432	Average_daily_gain	region1inRegion2	28883
CNVR74	938	7	23307667	23658110	loss-gain	5943	11625414	45102432	Average_daily_gain	region1inRegion2	350443
CNVR75	939	7	23481211	23658110	loss-gain	5943	11625414	45102432	Average_daily_gain	region1inRegion2	176899
CNVR76	942	7	24648309	24653067	loss-gain	5943	11625414	45102432	Average_daily_gain	region1inRegion2	4758
CNVR77	943	7	24735665	24740397	gain	5943	11625414	45102432	Average_daily_gain	region1inRegion2	4732
CNVR78	944	7	25359190	25399904	gain	5943	11625414	45102432	Average_daily_gain	region1inRegion2	40714
CNVR79	945	7	25488796	25557156	gain	5943	11625414	45102432	Average_daily_gain	region1inRegion2	68360
CNVR80	946	7	25785853	25888939	gain	5943	11625414	45102432	Average_daily_gain	region1inRegion2	103086
CNVR81	949	7	26267062	26297673	gain	5943	11625414	45102432	Average_daily_gain	region1inRegion2	30611
CNVR82	951	7	26600964	26608495	gain	5943	11625414	45102432	Average_daily_gain	region1inRegion2	7531
CNVR83	957	7	28525787	28565312	gain	5943	11625414	45102432	Average_daily_gain	region1inRegion2	39525
CNVR73	934	7	22207228	22236111	gain	5945	11625414	45102432	Average_daily_gain	region1inRegion2	28883
CNVR74	938	7	23307667	23658110	loss-gain	5945	11625414	45102432	Average_daily_gain	region1inRegion2	350443
CNVR75	939	7	23481211	23658110	loss-gain	5945	11625414	45102432	Average_daily_gain	region1inRegion2	176899
CNVR76	942	7	24648309	24653067	loss-gain	5945	11625414	45102432	Average_daily_gain	region1inRegion2	4758
CNVR77	943	7	24735665	24740397	gain	5945	11625414	45102432	Average_daily_gain	region1inRegion2	4732
CNVR78	944	7	25359190	25399904	gain	5945	11625414	45102432	Average_daily_gain	region1inRegion2	40714
CNVR79	945	7	25488796	25557156	gain	5945	11625414	45102432	Average_daily_gain	region1inRegion2	68360
CNVR80	946	7	25785853	25888939	gain	5945	11625414	45102432	Average_daily_gain	region1inRegion2	103086
CNVR81	949	7	26267062	26297673	gain	5945	11625414	45102432	Average_daily_gain	region1inRegion2	30611
CNVR82	951	7	26600964	26608495	gain	5945	11625414	45102432	Average_daily_gain	region1inRegion2	7531
CNVR83	957	7	28525787	28565312	gain	5945	11625414	45102432	Average_daily_gain	region1inRegion2	39525
CNVR73	934	7	22207228	22236111	gain	5946	11625414	45102432	Body_weight_(slaughter)	region1inRegion2	28883
CNVR74	938	7	23307667	23658110	loss-gain	5946	11625414	45102432	Body_weight_(slaughter)	region1inRegion2	350443
CNVR75	939	7	23481211	23658110	loss-gain	5946	11625414	45102432	Body_weight_(slaughter)	region1inRegion2	176899
CNVR76	942	7	24648309	24653067	loss-gain	5946	11625414	45102432	Body_weight_(slaughter)	region1inRegion2	4758
CNVR77	943	7	24735665	24740397	gain	5946	11625414	45102432	Body_weight_(slaughter)	region1inRegion2	4732
CNVR78	944	7	25359190	25399904	gain	5946	11625414	45102432	Body_weight_(slaughter)	region1inRegion2	40714
CNVR79	945	7	25488796	25557156	gain	5946	11625414	45102432	Body_weight_(slaughter)	region1inRegion2	68360
CNVR80	946	7	25785853	25888939	gain	5946	11625414	45102432	Body_weight_(slaughter)	region1inRegion2	103086
CNVR81	949	7	26267062	26297673	gain	5946	11625414	45102432	Body_weight_(slaughter)	region1inRegion2	30611
CNVR82	951	7	26600964	26608495	gain	5946	11625414	45102432	Body_weight_(slaughter)	region1inRegion2	7531
CNVR83	957	7	28525787	28565312	gain	5946	11625414	45102432	Body_weight_(slaughter)	region1inRegion2	39525
CNVR73	934	7	22207228	22236111	gain	36	11625414	48448805	Average_backfat_thickness	region1inRegion2	28883
CNVR74	938	7	23307667	23658110	loss-gain	36	11625414	48448805	Average_backfat_thickness	region1inRegion2	350443
CNVR75	939	7	23481211	23658110	loss-gain	36	11625414	48448805	Average_backfat_thickness	region1inRegion2	176899
CNVR76	942	7	24648309	24653067	loss-gain	36	11625414	48448805	Average_backfat_thickness	region1inRegion2	4758
CNVR77	943	7	24735665	24740397	gain	36	11625414	48448805	Average_backfat_thickness	region1inRegion2	4732
CNVR78	944	7	25359190	25399904	gain	36	11625414	48448805	Average_backfat_thickness	region1inRegion2	40714
CNVR79	945	7	25488796	25557156	gain	36	11625414	48448805	Average_backfat_thickness	region1inRegion2	68360
CNVR80	946	7	25785853	25888939	gain	36	11625414	48448805	Average_backfat_thickness	region1inRegion2	103086
CNVR81	949	7	26267062	26297673	gain	36	11625414	48448805	Average_backfat_thickness	region1inRegion2	30611
CNVR82	951	7	26600964	26608495	gain	36	11625414	48448805	Average_backfat_thickness	region1inRegion2	7531
CNVR83	957	7	28525787	28565312	gain	36	11625414	48448805	Average_backfat_thickness	region1inRegion2	39525
CNVR73	934	7	22207228	22236111	gain	11	11625414	50310118	Carcass_length	region1inRegion2	28883
CNVR74	938	7	23307667	23658110	loss-gain	11	11625414	50310118	Carcass_length	region1inRegion2	350443
CNVR75	939	7	23481211	23658110	loss-gain	11	11625414	50310118	Carcass_length	region1inRegion2	176899
CNVR76	942	7	24648309	24653067	loss-gain	11	11625414	50310118	Carcass_length	region1inRegion2	4758
CNVR77	943	7	24735665	24740397	gain	11	11625414	50310118	Carcass_length	region1inRegion2	4732
CNVR78	944	7	25359190	25399904	gain	11	11625414	50310118	Carcass_length	region1inRegion2	40714
CNVR79	945	7	25488796	25557156	gain	11	11625414	50310118	Carcass_length	region1inRegion2	68360
CNVR80	946	7	25785853	25888939	gain	11	11625414	50310118	Carcass_length	region1inRegion2	103086
CNVR81	949	7	26267062	26297673	gain	11	11625414	50310118	Carcass_length	region1inRegion2	30611
CNVR82	951	7	26600964	26608495	gain	11	11625414	50310118	Carcass_length	region1inRegion2	7531
CNVR83	957	7	28525787	28565312	gain	11	11625414	50310118	Carcass_length	region1inRegion2	39525
CNVR73	934	7	22207228	22236111	gain	2945	11625414	55731128	Backfat_at_last_lumbar	region1inRegion2	28883
CNVR74	938	7	23307667	23658110	loss-gain	2945	11625414	55731128	Backfat_at_last_lumbar	region1inRegion2	350443
CNVR75	939	7	23481211	23658110	loss-gain	2945	11625414	55731128	Backfat_at_last_lumbar	region1inRegion2	176899
CNVR76	942	7	24648309	24653067	loss-gain	2945	11625414	55731128	Backfat_at_last_lumbar	region1inRegion2	4758
CNVR77	943	7	24735665	24740397	gain	2945	11625414	55731128	Backfat_at_last_lumbar	region1inRegion2	4732
CNVR78	944	7	25359190	25399904	gain	2945	11625414	55731128	Backfat_at_last_lumbar	region1inRegion2	40714
CNVR79	945	7	25488796	25557156	gain	2945	11625414	55731128	Backfat_at_last_lumbar	region1inRegion2	68360
CNVR80	946	7	25785853	25888939	gain	2945	11625414	55731128	Backfat_at_last_lumbar	region1inRegion2	103086
CNVR81	949	7	26267062	26297673	gain	2945	11625414	55731128	Backfat_at_last_lumbar	region1inRegion2	30611
CNVR82	951	7	26600964	26608495	gain	2945	11625414	55731128	Backfat_at_last_lumbar	region1inRegion2	7531
CNVR83	957	7	28525787	28565312	gain	2945	11625414	55731128	Backfat_at_last_lumbar	region1inRegion2	39525
CNVR73	934	7	22207228	22236111	gain	365	11625414	55731128	Average_backfat_thickness	region1inRegion2	28883
CNVR74	938	7	23307667	23658110	loss-gain	365	11625414	55731128	Average_backfat_thickness	region1inRegion2	350443
CNVR75	939	7	23481211	23658110	loss-gain	365	11625414	55731128	Average_backfat_thickness	region1inRegion2	176899
CNVR76	942	7	24648309	24653067	loss-gain	365	11625414	55731128	Average_backfat_thickness	region1inRegion2	4758
CNVR77	943	7	24735665	24740397	gain	365	11625414	55731128	Average_backfat_thickness	region1inRegion2	4732
CNVR78	944	7	25359190	25399904	gain	365	11625414	55731128	Average_backfat_thickness	region1inRegion2	40714
CNVR79	945	7	25488796	25557156	gain	365	11625414	55731128	Average_backfat_thickness	region1inRegion2	68360
CNVR80	946	7	25785853	25888939	gain	365	11625414	55731128	Average_backfat_thickness	region1inRegion2	103086
CNVR81	949	7	26267062	26297673	gain	365	11625414	55731128	Average_backfat_thickness	region1inRegion2	30611
CNVR82	951	7	26600964	26608495	gain	365	11625414	55731128	Average_backfat_thickness	region1inRegion2	7531
CNVR83	957	7	28525787	28565312	gain	365	11625414	55731128	Average_backfat_thickness	region1inRegion2	39525
CNVR73	934	7	22207228	22236111	gain	366	11625414	55880470	Loin_muscle_depth	region1inRegion2	28883
CNVR74	938	7	23307667	23658110	loss-gain	366	11625414	55880470	Loin_muscle_depth	region1inRegion2	350443
CNVR75	939	7	23481211	23658110	loss-gain	366	11625414	55880470	Loin_muscle_depth	region1inRegion2	176899
CNVR76	942	7	24648309	24653067	loss-gain	366	11625414	55880470	Loin_muscle_depth	region1inRegion2	4758
CNVR77	943	7	24735665	24740397	gain	366	11625414	55880470	Loin_muscle_depth	region1inRegion2	4732
CNVR78	944	7	25359190	25399904	gain	366	11625414	55880470	Loin_muscle_depth	region1inRegion2	40714
CNVR79	945	7	25488796	25557156	gain	366	11625414	55880470	Loin_muscle_depth	region1inRegion2	68360
CNVR80	946	7	25785853	25888939	gain	366	11625414	55880470	Loin_muscle_depth	region1inRegion2	103086
CNVR81	949	7	26267062	26297673	gain	366	11625414	55880470	Loin_muscle_depth	region1inRegion2	30611
CNVR82	951	7	26600964	26608495	gain	366	11625414	55880470	Loin_muscle_depth	region1inRegion2	7531
CNVR83	957	7	28525787	28565312	gain	366	11625414	55880470	Loin_muscle_depth	region1inRegion2	39525
CNVR73	934	7	22207228	22236111	gain	28	11625414	69667766	backfat_at_last_rib	region1inRegion2	28883
CNVR74	938	7	23307667	23658110	loss-gain	28	11625414	69667766	backfat_at_last_rib	region1inRegion2	350443
CNVR75	939	7	23481211	23658110	loss-gain	28	11625414	69667766	backfat_at_last_rib	region1inRegion2	176899
CNVR76	942	7	24648309	24653067	loss-gain	28	11625414	69667766	backfat_at_last_rib	region1inRegion2	4758
CNVR77	943	7	24735665	24740397	gain	28	11625414	69667766	backfat_at_last_rib	region1inRegion2	4732
CNVR78	944	7	25359190	25399904	gain	28	11625414	69667766	backfat_at_last_rib	region1inRegion2	40714
CNVR79	945	7	25488796	25557156	gain	28	11625414	69667766	backfat_at_last_rib	region1inRegion2	68360
CNVR80	946	7	25785853	25888939	gain	28	11625414	69667766	backfat_at_last_rib	region1inRegion2	103086
CNVR81	949	7	26267062	26297673	gain	28	11625414	69667766	backfat_at_last_rib	region1inRegion2	30611

CNVR82	951	7	26600964	26608495	gain	28	11625414	69667766	backfat_at_last_rib	region1inRegion2	7531
CNVR83	957	7	28525787	28565312	gain	28	11625414	69667766	backfat_at_last_rib	region1inRegion2	39525
CNVR84	972	7	58579811	58584542	gain	28	11625414	69667766	backfat_at_last_rib	region1inRegion2	4731
CNVR85	975	7	59278633	59284219	loss	28	11625414	69667766	backfat_at_last_rib	region1inRegion2	5586
CNVR86	977	7	61355441	61417944	gain	28	11625414	69667766	backfat_at_last_rib	region1inRegion2	62503
CNVR73	934	7	22207228	22236111	gain	5700	11625414	101176460	Carcass_length	region1inRegion2	28883
CNVR74	938	7	23307667	23658110	loss-gain	5700	11625414	101176460	Carcass_length	region1inRegion2	350443
CNVR75	939	7	23481211	23658110	loss-gain	5700	11625414	101176460	Carcass_length	region1inRegion2	176899
CNVR76	942	7	24648309	24653067	loss-gain	5700	11625414	101176460	Carcass_length	region1inRegion2	4758
CNVR77	943	7	24735665	24740397	gain	5700	11625414	101176460	Carcass_length	region1inRegion2	4732
CNVR78	944	7	25359190	25399904	gain	5700	11625414	101176460	Carcass_length	region1inRegion2	40714
CNVR79	945	7	25488796	25557156	gain	5700	11625414	101176460	Carcass_length	region1inRegion2	68360
CNVR80	946	7	25785853	25888939	gain	5700	11625414	101176460	Carcass_length	region1inRegion2	103086
CNVR81	949	7	26267062	26297673	gain	5700	11625414	101176460	Carcass_length	region1inRegion2	30611
CNVR82	951	7	26600964	26608495	gain	5700	11625414	101176460	Carcass_length	region1inRegion2	7531
CNVR83	957	7	28525787	28565312	gain	5700	11625414	101176460	Carcass_length	region1inRegion2	39525
CNVR84	972	7	58579811	58584542	gain	5700	11625414	101176460	Carcass_length	region1inRegion2	4731
CNVR85	975	7	59278633	59284219	loss	5700	11625414	101176460	Carcass_length	region1inRegion2	5586
CNVR86	977	7	61355441	61417944	gain	5700	11625414	101176460	Carcass_length	region1inRegion2	62503
CNVR87	994	7	82234623	82374446	gain	5700	11625414	101176460	Carcass_length	region1inRegion2	139823
CNVR88	1002	7	84728780	84749202	gain	5700	11625414	101176460	Carcass_length	region1inRegion2	20422
CNVR73	934	7	22207228	22236111	gain	1072	12337141	30929840	Average_backfat_thickness	region1inRegion2	28883
CNVR74	938	7	23307667	23658110	loss-gain	1072	12337141	30929840	Average_backfat_thickness	region1inRegion2	350443
CNVR75	939	7	23481211	23658110	loss-gain	1072	12337141	30929840	Average_backfat_thickness	region1inRegion2	176899
CNVR76	942	7	24648309	24653067	loss-gain	1072	12337141	30929840	Average_backfat_thickness	region1inRegion2	4758
CNVR77	943	7	24735665	24740397	gain	1072	12337141	30929840	Average_backfat_thickness	region1inRegion2	4732
CNVR78	944	7	25359190	25399904	gain	1072	12337141	30929840	Average_backfat_thickness	region1inRegion2	40714
CNVR79	945	7	25488796	25557156	gain	1072	12337141	30929840	Average_backfat_thickness	region1inRegion2	68360
CNVR80	946	7	25785853	25888939	gain	1072	12337141	30929840	Average_backfat_thickness	region1inRegion2	103086
CNVR81	949	7	26267062	26297673	gain	1072	12337141	30929840	Average_backfat_thickness	region1inRegion2	30611
CNVR82	951	7	26600964	26608495	gain	1072	12337141	30929840	Average_backfat_thickness	region1inRegion2	7531
CNVR83	957	7	28525787	28565312	gain	1072	12337141	30929840	Average_backfat_thickness	region1inRegion2	39525
CNVR73	934	7	22207228	22236111	gain	3853	12337141	32380448	Muscle_moisture_percentage	region1inRegion2	28883
CNVR74	938	7	23307667	23658110	loss-gain	3853	12337141	32380448	Muscle_moisture_percentage	region1inRegion2	350443
CNVR75	939	7	23481211	23658110	loss-gain	3853	12337141	32380448	Muscle_moisture_percentage	region1inRegion2	176899
CNVR76	942	7	24648309	24653067	loss-gain	3853	12337141	32380448	Muscle_moisture_percentage	region1inRegion2	4758
CNVR77	943	7	24735665	24740397	gain	3853	12337141	32380448	Muscle_moisture_percentage	region1inRegion2	4732
CNVR78	944	7	25359190	25399904	gain	3853	12337141	32380448	Muscle_moisture_percentage	region1inRegion2	40714
CNVR79	945	7	25488796	25557156	gain	3853	12337141	32380448	Muscle_moisture_percentage	region1inRegion2	68360
CNVR80	946	7	25785853	25888939	gain	3853	12337141	32380448	Muscle_moisture_percentage	region1inRegion2	103086
CNVR81	949	7	26267062	26297673	gain	3853	12337141	32380448	Muscle_moisture_percentage	region1inRegion2	30611
CNVR82	951	7	26600964	26608495	gain	3853	12337141	32380448	Muscle_moisture_percentage	region1inRegion2	7531
CNVR83	957	7	28525787	28565312	gain	3853	12337141	32380448	Muscle_moisture_percentage	region1inRegion2	39525
CNVR73	934	7	22207228	22236111	gain	5506	12337141	32380448	Skin_percentage	region1inRegion2	28883
CNVR74	938	7	23307667	23658110	loss-gain	5506	12337141	32380448	Skin_percentage	region1inRegion2	350443
CNVR75	939	7	23481211	23658110	loss-gain	5506	12337141	32380448	Skin_percentage	region1inRegion2	176899
CNVR76	942	7	24648309	24653067	loss-gain	5506	12337141	32380448	Skin_percentage	region1inRegion2	4758
CNVR77	943	7	24735665	24740397	gain	5506	12337141	32380448	Skin_percentage	region1inRegion2	4732
CNVR78	944	7	25359190	25399904	gain	5506	12337141	32380448	Skin_percentage	region1inRegion2	40714
CNVR79	945	7	25488796	25557156	gain	5506	12337141	32380448	Skin_percentage	region1inRegion2	68360
CNVR80	946	7	25785853	25888939	gain	5506	12337141	32380448	Skin_percentage	region1inRegion2	103086
CNVR81	949	7	26267062	26297673	gain	5506	12337141	32380448	Skin_percentage	region1inRegion2	30611
CNVR82	951	7	26600964	26608495	gain	5506	12337141	32380448	Skin_percentage	region1inRegion2	7531
CNVR83	957	7	28525787	28565312	gain	5506	12337141	32380448	Skin_percentage	region1inRegion2	39525
CNVR73	934	7	22207228	22236111	gain	16903	12337141	36354046	Carcass_weight_(hot)	region1inRegion2	28883
CNVR74	938	7	23307667	23658110	loss-gain	16903	12337141	36354046	Carcass_weight_(hot)	region1inRegion2	350443
CNVR75	939	7	23481211	23658110	loss-gain	16903	12337141	36354046	Carcass_weight_(hot)	region1inRegion2	176899
CNVR76	942	7	24648309	24653067	loss-gain	16903	12337141	36354046	Carcass_weight_(hot)	region1inRegion2	4758
CNVR77	943	7	24735665	24740397	gain	16903	12337141	36354046	Carcass_weight_(hot)	region1inRegion2	4732
CNVR78	944	7	25359190	25399904	gain	16903	12337141	36354046	Carcass_weight_(hot)	region1inRegion2	40714
CNVR79	945	7	25488796	25557156	gain	16903	12337141	36354046	Carcass_weight_(hot)	region1inRegion2	68360
CNVR80	946	7	25785853	25888939	gain	16903	12337141	36354046	Carcass_weight_(hot)	region1inRegion2	103086
CNVR81	949	7	26267062	26297673	gain	16903	12337141	36354046	Carcass_weight_(hot)	region1inRegion2	30611
CNVR82	951	7	26600964	26608495	gain	16903	12337141	36354046	Carcass_weight_(hot)	region1inRegion2	7531
CNVR83	957	7	28525787	28565312	gain	16903	12337141	36354046	Carcass_weight_(hot)	region1inRegion2	39525
CNVR73	934	7	22207228	22236111	gain	12776	12337141	36525998	NADP-malate_dehydrogenase_activity	region1inRegion2	28883
CNVR74	938	7	23307667	23658110	loss-gain	12776	12337141	36525998	NADP-malate_dehydrogenase_activity	region1inRegion2	350443
CNVR75	939	7	23481211	23658110	loss-gain	12776	12337141	36525998	NADP-malate_dehydrogenase_activity	region1inRegion2	176899
CNVR76	942	7	24648309	24653067	loss-gain	12776	12337141	36525998	NADP-malate_dehydrogenase_activity	region1inRegion2	4758
CNVR77	943	7	24735665	24740397	gain	12776	12337141	36525998	NADP-malate_dehydrogenase_activity	region1inRegion2	4732
CNVR78	944	7	25359190	25399904	gain	12776	12337141	36525998	NADP-malate_dehydrogenase_activity	region1inRegion2	40714
CNVR79	945	7	25488796	25557156	gain	12776	12337141	36525998	NADP-malate_dehydrogenase_activity	region1inRegion2	68360
CNVR80	946	7	25785853	25888939	gain	12776	12337141	36525998	NADP-malate_dehydrogenase_activity	region1inRegion2	103086
CNVR81	949	7	26267062	26297673	gain	12776	12337141	36525998	NADP-malate_dehydrogenase_activity	region1inRegion2	30611
CNVR82	951	7	26600964	26608495	gain	12776	12337141	36525998	NADP-malate_dehydrogenase_activity	region1inRegion2	7531
CNVR83	957	7	28525787	28565312	gain	12776	12337141	36525998	NADP-malate_dehydrogenase_activity	region1inRegion2	39525
CNVR73	934	7	22207228	22236111	gain	12779	12337141	36525998	NADPH-generating_enzyme_activity	region1inRegion2	28883
CNVR74	938	7	23307667	23658110	loss-gain	12779	12337141	36525998	NADPH-generating_enzyme_activity	region1inRegion2	350443
CNVR75	939	7	23481211	23658110	loss-gain	12779	12337141	36525998	NADPH-generating_enzyme_activity	region1inRegion2	176899
CNVR76	942	7	24648309	24653067	loss-gain	12779	12337141	36525998	NADPH-generating_enzyme_activity	region1inRegion2	4758
CNVR77	943	7	24735665	24740397	gain	12779	12337141	36525998	NADPH-generating_enzyme_activity	region1inRegion2	4732
CNVR78	944	7	25359190	25399904	gain	12779	12337141	36525998	NADPH-generating_enzyme_activity	region1inRegion2	40714
CNVR79	945	7	25488796	25557156	gain	12779	12337141	36525998	NADPH-generating_enzyme_activity	region1inRegion2	68360
CNVR80	946	7	25785853	25888939	gain	12779	12337141	36525998	NADPH-generating_enzyme_activity	region1inRegion2	103086
CNVR81	949	7	26267062	26297673	gain	12779	12337141	36525998	NADPH-generating_enzyme_activity	region1inRegion2	30611
CNVR82	951	7	26600964	26608495	gain	12779	12337141	36525998	NADPH-generating_enzyme_activity	region1inRegion2	7531
CNVR83	957	7	28525787	28565312	gain	12779	12337141	36525998	NADPH-generating_enzyme_activity	region1inRegion2	39525
CNVR73	934	7	22207228	22236111	gain	12783	12337141	36525998	NADP-malate_dehydrogenase_activity	region1inRegion2	28883
CNVR74	938	7	23307667	23658110	loss-gain	12783	12337141	36525998	NADP-malate_dehydrogenase_activity	region1inRegion2	350443
CNVR75	939	7	23481211	23658110	loss-gain	12783	12337141	36525998	NADP-malate_dehydrogenase_activity	region1inRegion2	176899
CNVR76	942	7	24648309	24653067	loss-gain	12783	12337141	36525998	NADP-malate_dehydrogenase_activity	region1inRegion2	4758
CNVR77	943	7	24735665	24740397	gain	12783	12337141	36525998	NADP-malate_dehydrogenase_activity	region1inRegion2	4732
CNVR78	944	7	25359190	25399904	gain	12783	12337141	36525998	NADP-malate_dehydrogenase_activity	region1inRegion2	40714
CNVR79	945	7	25488796	25557156	gain	12783	12337141	36525998	NADP-malate_dehydrogenase_activity	region1inRegion2	68360
CNVR80	946	7	25785853	25888939	gain	12783	12337141	36525998	NADP-malate_dehydrogenase_activity	region1inRegion2	103086
CNVR81	949	7	26267062	26297673	gain	12783	12337141	36525998	NADP-malate_dehydrogenase_activity	region1inRegion2	30611
CNVR82	951	7	26600964	26608495	gain	12783	12337141	36525998	NADP-malate_dehydrogenase_activity	region1inRegion2	7531
CNVR83	957	7	28525787	28565312	gain	12783	12337141	36525998	NADP-malate_dehydrogenase_activity	region1inRegion2	39525
CNVR73	934	7	22207228	22236111	gain	12785	12337141	36525998	NADPH-generating_enzyme_activity	region1inRegion2	28883
CNVR74	938	7	23307667	23658110	loss-gain	12785	12337141	36525998	NADPH-generating_enzyme_activity	region1inRegion2	350443
CNVR75	939	7	23481211	23658110	loss-gain	12785	12337141	36525998	NADPH-generating_enzyme_activity	region1inRegion2	176899
CNVR76	942	7	24648309	24653067	loss-gain	12785	12337141	36525998	NADPH-generating_enzyme_activity	region1inRegion2	4758
CNVR77	943	7	24735665	24740397	gain	12785	12337141	36525998	NADPH-generating_enzyme_activity	region1inRegion2	4732
CNVR78	944	7	25359190	25399904	gain	12785	12337141	36525998	NADPH-generating_enzyme_activity	region1inRegion2	40714
CNVR79	945	7	25488796	25557156	gain	12785	12337141	36525998	NADPH-generating_enzyme_activity	region1inRegion2	68360
CNVR80	946	7	25785853	25888939	gain	12785	12337141	36525998	NADPH-generating_enzyme_activity	region1inRegion2	103086
CNVR81	949	7	26267062	26297673	gain	12785	12337141	36525998	NADPH-generating_enzyme_activity	region1inRegion2	30611
CNVR82	951	7	26600964	26608495	gain	12785	12337141	36525998	NADPH-generating_enzyme_activity	region1inRegion2	7531
CNVR83	957	7	28525787	28565312	gain	12785	12337141	36525998	NADPH-generating_enzyme_activity	region1inRegion2	39525
CNVR73	934	7	22207228	22236111	gain	1067	12337141	36993248	Head_weight	region1inRegion2	28883
CNVR74	938	7	23307667	23658110	loss-gain	1067	12337141	36993248	Head_weight	region1inRegion2	350443
CNVR75	939	7	23481211	23658110	loss-gain	1067	12337141	36993248	Head_weight	region1inRegion2	176899
CNVR76	942	7	24648309	24653067	loss-gain	1067	12337141	36993248	Head_weight	region1inRegion2	4758
CNVR77	943	7	24735665	24740397	gain	1067	12337141	36993248	Head_weight	region1inRegion2	4732
CNVR78	944	7	25359190	25399904	gain	1067	12337141	36993248	Head_weight	region1inRegion2	40714
CNVR79	945	7	25488796	25557156	gain	1067	12337141	36993248	Head_weight	region1inRegion2	68360
CNVR80	946	7	25785853	25888939	gain	1067	12337141	36993248	Head_weight	region1inRegion2	103086
CNVR81	949	7	26267062	26297673	gain	1067	12337141	36993248	Head_weight	region1inRegion2	30611
CNVR82	951	7	26600964	26608495	gain	1067	12337141	36993248	Head_weight	region1inRegion2	7531
CNVR83	957	7	28525787	28565312	gain	1067	12337141	36993248	Head_weight	region1inRegion2	39525
CNVR73	934	7	22207228	22236111	gain	1069	12337141	36993248	Backfat_at_tenth_rib	region1inRegion2	28883
CNVR74	938	7	23307667	23658110	loss-gain	1069	12337141	36993248	Backfat_at_tenth_rib	region1inRegion2	350443

CNVR75	939	7	23481211	23658110	loss-gain	1069	12337141	36993248	Backfat_at_tenth_rib	region1inRegion2	176899
CNVR76	942	7	24648309	24653067	loss-gain	1069	12337141	36993248	Backfat_at_tenth_rib	region1inRegion2	4758
CNVR77	943	7	24735665	24740397	gain	1069	12337141	36993248	Backfat_at_tenth_rib	region1inRegion2	4732
CNVR78	944	7	25359190	25399904	gain	1069	12337141	36993248	Backfat_at_tenth_rib	region1inRegion2	40714
CNVR79	945	7	25488796	25557156	gain	1069	12337141	36993248	Backfat_at_tenth_rib	region1inRegion2	68360
CNVR80	946	7	25785853	25888939	gain	1069	12337141	36993248	Backfat_at_tenth_rib	region1inRegion2	103086
CNVR81	949	7	26267062	26297673	gain	1069	12337141	36993248	Backfat_at_tenth_rib	region1inRegion2	30611
CNVR82	951	7	26600964	26608495	gain	1069	12337141	36993248	Backfat_at_tenth_rib	region1inRegion2	7531
CNVR83	957	7	28525787	28565312	gain	1069	12337141	36993248	Backfat_at_tenth_rib	region1inRegion2	39525
CNVR73	934	7	22207228	22236111	gain	1070	12337141	36993248	backfat_at_last_rib	region1inRegion2	28883
CNVR74	938	7	23307667	23658110	loss-gain	1070	12337141	36993248	backfat_at_last_rib	region1inRegion2	350443
CNVR75	939	7	23481211	23658110	loss-gain	1070	12337141	36993248	backfat_at_last_rib	region1inRegion2	176899
CNVR76	942	7	24648309	24653067	loss-gain	1070	12337141	36993248	backfat_at_last_rib	region1inRegion2	4758
CNVR77	943	7	24735665	24740397	gain	1070	12337141	36993248	backfat_at_last_rib	region1inRegion2	4732
CNVR78	944	7	25359190	25399904	gain	1070	12337141	36993248	backfat_at_last_rib	region1inRegion2	40714
CNVR79	945	7	25488796	25557156	gain	1070	12337141	36993248	backfat_at_last_rib	region1inRegion2	68360
CNVR80	946	7	25785853	25888939	gain	1070	12337141	36993248	backfat_at_last_rib	region1inRegion2	103086
CNVR81	949	7	26267062	26297673	gain	1070	12337141	36993248	backfat_at_last_rib	region1inRegion2	30611
CNVR82	951	7	26600964	26608495	gain	1070	12337141	36993248	backfat_at_last_rib	region1inRegion2	7531
CNVR83	957	7	28525787	28565312	gain	1070	12337141	36993248	backfat_at_last_rib	region1inRegion2	39525
CNVR73	934	7	22207228	22236111	gain	1079	12337141	36993248	Fat-cuts_percentage	region1inRegion2	28883
CNVR74	938	7	23307667	23658110	loss-gain	1079	12337141	36993248	Fat-cuts_percentage	region1inRegion2	350443
CNVR75	939	7	23481211	23658110	loss-gain	1079	12337141	36993248	Fat-cuts_percentage	region1inRegion2	176899
CNVR76	942	7	24648309	24653067	loss-gain	1079	12337141	36993248	Fat-cuts_percentage	region1inRegion2	4758
CNVR77	943	7	24735665	24740397	gain	1079	12337141	36993248	Fat-cuts_percentage	region1inRegion2	4732
CNVR78	944	7	25359190	25399904	gain	1079	12337141	36993248	Fat-cuts_percentage	region1inRegion2	40714
CNVR79	945	7	25488796	25557156	gain	1079	12337141	36993248	Fat-cuts_percentage	region1inRegion2	68360
CNVR80	946	7	25785853	25888939	gain	1079	12337141	36993248	Fat-cuts_percentage	region1inRegion2	103086
CNVR81	949	7	26267062	26297673	gain	1079	12337141	36993248	Fat-cuts_percentage	region1inRegion2	30611
CNVR82	951	7	26600964	26608495	gain	1079	12337141	36993248	Fat-cuts_percentage	region1inRegion2	7531
CNVR83	957	7	28525787	28565312	gain	1079	12337141	36993248	Fat-cuts_percentage	region1inRegion2	39525
CNVR73	934	7	22207228	22236111	gain	3836	12337141	36993248	Average_backfat_thickness	region1inRegion2	28883
CNVR74	938	7	23307667	23658110	loss-gain	3836	12337141	36993248	Average_backfat_thickness	region1inRegion2	350443
CNVR75	939	7	23481211	23658110	loss-gain	3836	12337141	36993248	Average_backfat_thickness	region1inRegion2	176899
CNVR76	942	7	24648309	24653067	loss-gain	3836	12337141	36993248	Average_backfat_thickness	region1inRegion2	4758
CNVR77	943	7	24735665	24740397	gain	3836	12337141	36993248	Average_backfat_thickness	region1inRegion2	4732
CNVR78	944	7	25359190	25399904	gain	3836	12337141	36993248	Average_backfat_thickness	region1inRegion2	40714
CNVR79	945	7	25488796	25557156	gain	3836	12337141	36993248	Average_backfat_thickness	region1inRegion2	68360
CNVR80	946	7	25785853	25888939	gain	3836	12337141	36993248	Average_backfat_thickness	region1inRegion2	103086
CNVR81	949	7	26267062	26297673	gain	3836	12337141	36993248	Average_backfat_thickness	region1inRegion2	30611
CNVR82	951	7	26600964	26608495	gain	3836	12337141	36993248	Average_backfat_thickness	region1inRegion2	7531
CNVR83	957	7	28525787	28565312	gain	3836	12337141	36993248	Average_backfat_thickness	region1inRegion2	39525
CNVR73	934	7	22207228	22236111	gain	32	12337141	38992356	Backfat_at_last_lumbar	region1inRegion2	28883
CNVR74	938	7	23307667	23658110	loss-gain	32	12337141	38992356	Backfat_at_last_lumbar	region1inRegion2	350443
CNVR75	939	7	23481211	23658110	loss-gain	32	12337141	38992356	Backfat_at_last_lumbar	region1inRegion2	176899
CNVR76	942	7	24648309	24653067	loss-gain	32	12337141	38992356	Backfat_at_last_lumbar	region1inRegion2	4758
CNVR77	943	7	24735665	24740397	gain	32	12337141	38992356	Backfat_at_last_lumbar	region1inRegion2	4732
CNVR78	944	7	25359190	25399904	gain	32	12337141	38992356	Backfat_at_last_lumbar	region1inRegion2	40714
CNVR79	945	7	25488796	25557156	gain	32	12337141	38992356	Backfat_at_last_lumbar	region1inRegion2	68360
CNVR80	946	7	25785853	25888939	gain	32	12337141	38992356	Backfat_at_last_lumbar	region1inRegion2	103086
CNVR81	949	7	26267062	26297673	gain	32	12337141	38992356	Backfat_at_last_lumbar	region1inRegion2	30611
CNVR82	951	7	26600964	26608495	gain	32	12337141	38992356	Backfat_at_last_lumbar	region1inRegion2	7531
CNVR83	957	7	28525787	28565312	gain	32	12337141	38992356	Backfat_at_last_lumbar	region1inRegion2	39525
CNVR73	934	7	22207228	22236111	gain	24	12337141	45102432	Backfat_at_tenth_rib	region1inRegion2	28883
CNVR74	938	7	23307667	23658110	loss-gain	24	12337141	45102432	Backfat_at_tenth_rib	region1inRegion2	350443
CNVR75	939	7	23481211	23658110	loss-gain	24	12337141	45102432	Backfat_at_tenth_rib	region1inRegion2	176899
CNVR76	942	7	24648309	24653067	loss-gain	24	12337141	45102432	Backfat_at_tenth_rib	region1inRegion2	4758
CNVR77	943	7	24735665	24740397	gain	24	12337141	45102432	Backfat_at_tenth_rib	region1inRegion2	4732
CNVR78	944	7	25359190	25399904	gain	24	12337141	45102432	Backfat_at_tenth_rib	region1inRegion2	40714
CNVR79	945	7	25488796	25557156	gain	24	12337141	45102432	Backfat_at_tenth_rib	region1inRegion2	68360
CNVR80	946	7	25785853	25888939	gain	24	12337141	45102432	Backfat_at_tenth_rib	region1inRegion2	103086
CNVR81	949	7	26267062	26297673	gain	24	12337141	45102432	Backfat_at_tenth_rib	region1inRegion2	30611
CNVR82	951	7	26600964	26608495	gain	24	12337141	45102432	Backfat_at_tenth_rib	region1inRegion2	7531
CNVR83	957	7	28525787	28565312	gain	24	12337141	45102432	Backfat_at_tenth_rib	region1inRegion2	39525
CNVR73	934	7	22207228	22236111	gain	39	12337141	48448805	Leaf_fat_weight	region1inRegion2	28883
CNVR74	938	7	23307667	23658110	loss-gain	39	12337141	48448805	Leaf_fat_weight	region1inRegion2	350443
CNVR75	939	7	23481211	23658110	loss-gain	39	12337141	48448805	Leaf_fat_weight	region1inRegion2	176899
CNVR76	942	7	24648309	24653067	loss-gain	39	12337141	48448805	Leaf_fat_weight	region1inRegion2	4758
CNVR77	943	7	24735665	24740397	gain	39	12337141	48448805	Leaf_fat_weight	region1inRegion2	4732
CNVR78	944	7	25359190	25399904	gain	39	12337141	48448805	Leaf_fat_weight	region1inRegion2	40714
CNVR79	945	7	25488796	25557156	gain	39	12337141	48448805	Leaf_fat_weight	region1inRegion2	68360
CNVR80	946	7	25785853	25888939	gain	39	12337141	48448805	Leaf_fat_weight	region1inRegion2	103086
CNVR81	949	7	26267062	26297673	gain	39	12337141	48448805	Leaf_fat_weight	region1inRegion2	30611
CNVR82	951	7	26600964	26608495	gain	39	12337141	48448805	Leaf_fat_weight	region1inRegion2	7531
CNVR83	957	7	28525787	28565312	gain	39	12337141	48448805	Leaf_fat_weight	region1inRegion2	39525
CNVR73	934	7	22207228	22236111	gain	20	12337141	50310118	Backfat_at_first_rib	region1inRegion2	28883
CNVR74	938	7	23307667	23658110	loss-gain	20	12337141	50310118	Backfat_at_first_rib	region1inRegion2	350443
CNVR75	939	7	23481211	23658110	loss-gain	20	12337141	50310118	Backfat_at_first_rib	region1inRegion2	176899
CNVR76	942	7	24648309	24653067	loss-gain	20	12337141	50310118	Backfat_at_first_rib	region1inRegion2	4758
CNVR77	943	7	24735665	24740397	gain	20	12337141	50310118	Backfat_at_first_rib	region1inRegion2	4732
CNVR78	944	7	25359190	25399904	gain	20	12337141	50310118	Backfat_at_first_rib	region1inRegion2	40714
CNVR79	945	7	25488796	25557156	gain	20	12337141	50310118	Backfat_at_first_rib	region1inRegion2	68360
CNVR80	946	7	25785853	25888939	gain	20	12337141	50310118	Backfat_at_first_rib	region1inRegion2	103086
CNVR81	949	7	26267062	26297673	gain	20	12337141	50310118	Backfat_at_first_rib	region1inRegion2	30611
CNVR82	951	7	26600964	26608495	gain	20	12337141	50310118	Backfat_at_first_rib	region1inRegion2	7531
CNVR83	957	7	28525787	28565312	gain	20	12337141	50310118	Backfat_at_first_rib	region1inRegion2	39525
CNVR73	934	7	22207228	22236111	gain	60	12337141	120698338	Backfat_at_last_lumbar	region1inRegion2	28883
CNVR74	938	7	23307667	23658110	loss-gain	60	12337141	120698338	Backfat_at_last_lumbar	region1inRegion2	350443
CNVR75	939	7	23481211	23658110	loss-gain	60	12337141	120698338	Backfat_at_last_lumbar	region1inRegion2	176899
CNVR76	942	7	24648309	24653067	loss-gain	60	12337141	120698338	Backfat_at_last_lumbar	region1inRegion2	4758
CNVR77	943	7	24735665	24740397	gain	60	12337141	120698338	Backfat_at_last_lumbar	region1inRegion2	4732
CNVR78	944	7	25359190	25399904	gain	60	12337141	120698338	Backfat_at_last_lumbar	region1inRegion2	40714
CNVR79	945	7	25488796	25557156	gain	60	12337141	120698338	Backfat_at_last_lumbar	region1inRegion2	68360
CNVR80	946	7	25785853	25888939	gain	60	12337141	120698338	Backfat_at_last_lumbar	region1inRegion2	103086
CNVR81	949	7	26267062	26297673	gain	60	12337141	120698338	Backfat_at_last_lumbar	region1inRegion2	30611
CNVR82	951	7	26600964	26608495	gain	60	12337141	120698338	Backfat_at_last_lumbar	region1inRegion2	7531
CNVR83	957	7	28525787	28565312	gain	60	12337141	120698338	Backfat_at_last_lumbar	region1inRegion2	39525
CNVR84	972	7	58579811	58584542	gain	60	12337141	120698338	Backfat_at_last_lumbar	region1inRegion2	4731
CNVR85	975	7	59278633	59284219	loss	60	12337141	120698338	Backfat_at_last_lumbar	region1inRegion2	5586
CNVR86	977	7	61355441	61417944	gain	60	12337141	120698338	Backfat_at_last_lumbar	region1inRegion2	62503
CNVR87	994	7	82234623	82374446	gain	60	12337141	120698338	Backfat_at_last_lumbar	region1inRegion2	139823
CNVR88	1002	7	84728780	84749202	gain	60	12337141	120698338	Backfat_at_last_lumbar	region1inRegion2	20422
CNVR89	1011	7	102790067	102823288	gain	60	12337141	120698338	Backfat_at_last_lumbar	region1inRegion2	33221
CNVR90	1021	7	111620166	111623056	loss	60	12337141	120698338	Backfat_at_last_lumbar	region1inRegion2	2890
CNVR73	934	7	22207228	22236111	gain	5236	12528514	123207566	Body_weight_(end_of_test)	region1inRegion2	28883
CNVR74	938	7	23307667	23658110	loss-gain	5236	12528514	123207566	Body_weight_(end_of_test)	region1inRegion2	350443
CNVR75	939	7	23481211	23658110	loss-gain	5236	12528514	123207566	Body_weight_(end_of_test)	region1inRegion2	176899
CNVR76	942	7	24648309	24653067	loss-gain	5236	12528514	123207566	Body_weight_(end_of_test)	region1inRegion2	4758
CNVR77	943	7	24735665	24740397	gain	5236	12528514	123207566	Body_weight_(end_of_test)	region1inRegion2	4732
CNVR78	944	7	25359190	25399904	gain	5236	12528514	123207566	Body_weight_(end_of_test)	region1inRegion2	40714
CNVR79	945	7	25488796	25557156	gain	5236	12528514	123207566	Body_weight_(end_of_test)	region1inRegion2	68360
CNVR80	946	7	25785853	25888939	gain	5236	12528514	123207566	Body_weight_(end_of_test)	region1inRegion2	103086
CNVR81	949	7	26267062	26297673	gain	5236	12528514	123207566	Body_weight_(end_of_test)	region1inRegion2	30611
CNVR82	951	7	26600964	26608495	gain	5236	12528514	123207566	Body_weight_(end_of_test)	region1inRegion2	7531
CNVR83	957	7	28525787	28565312	gain	5236	12528514	123207566	Body_weight_(end_of_test)	region1inRegion2	39525
CNVR84	972	7	58579811	58584542	gain	5236	12528514	123207566	Body_weight_(end_of_test)	region1inRegion2	4731
CNVR85	975	7	59278633	59284219	loss	5236	12528514	123207566	Body_weight_(end_of_test)	region1inRegion2	5586
CNVR86	977	7	61355441	61417944	gain	5236	12528514	123207566	Body_weight_(end_of_test)	region1inRegion2	62503
CNVR87	994	7	82234623	82374446	gain	5236	12528514	123207566	Body_weight_(end_of_test)	region1inRegion2	139823
CNVR88	1002	7	84728780	84749202	gain	5236	12528514	123207566	Body_weight_(end_of_test)	region1inRegion2	20422
CNVR89	1011	7	102790067	102823288	gain	5236	12528514	123207566	Body_weight_(end_of_test)	region1inRegion2	33221
CNVR90	1021	7	111620166	111623056	loss	5236	12528514	123207566	Body_weight_(end_of_test)	region1inRegion2	2890

CNVR73	934	7	22207228	22236111	gain	22159	16139041	39498491	Hemoglobin	region1inRegion2	28883
CNVR74	938	7	23307667	23658110	loss-gain	22159	16139041	39498491	Hemoglobin	region1inRegion2	350443
CNVR75	939	7	23481211	23658110	loss-gain	22159	16139041	39498491	Hemoglobin	region1inRegion2	176899
CNVR76	942	7	24648309	24653067	loss-gain	22159	16139041	39498491	Hemoglobin	region1inRegion2	4758
CNVR77	943	7	24735665	24740397	gain	22159	16139041	39498491	Hemoglobin	region1inRegion2	4732
CNVR78	944	7	25359190	25399904	gain	22159	16139041	39498491	Hemoglobin	region1inRegion2	40714
CNVR79	945	7	25488796	25557156	gain	22159	16139041	39498491	Hemoglobin	region1inRegion2	68360
CNVR80	946	7	25785853	25888939	gain	22159	16139041	39498491	Hemoglobin	region1inRegion2	103086
CNVR81	949	7	26267062	26297673	gain	22159	16139041	39498491	Hemoglobin	region1inRegion2	30611
CNVR82	951	7	26600964	26608495	gain	22159	16139041	39498491	Hemoglobin	region1inRegion2	7531
CNVR83	957	7	28525787	28565312	gain	22159	16139041	39498491	Hemoglobin	region1inRegion2	39525
CNVR73	934	7	22207228	22236111	gain	588	16365408	42509154	Age_at_puberty	region1inRegion2	28883
CNVR74	938	7	23307667	23658110	loss-gain	588	16365408	42509154	Age_at_puberty	region1inRegion2	350443
CNVR75	939	7	23481211	23658110	loss-gain	588	16365408	42509154	Age_at_puberty	region1inRegion2	176899
CNVR76	942	7	24648309	24653067	loss-gain	588	16365408	42509154	Age_at_puberty	region1inRegion2	4758
CNVR77	943	7	24735665	24740397	gain	588	16365408	42509154	Age_at_puberty	region1inRegion2	4732
CNVR78	944	7	25359190	25399904	gain	588	16365408	42509154	Age_at_puberty	region1inRegion2	40714
CNVR79	945	7	25488796	25557156	gain	588	16365408	42509154	Age_at_puberty	region1inRegion2	68360
CNVR80	946	7	25785853	25888939	gain	588	16365408	42509154	Age_at_puberty	region1inRegion2	103086
CNVR81	949	7	26267062	26297673	gain	588	16365408	42509154	Age_at_puberty	region1inRegion2	30611
CNVR82	951	7	26600964	26608495	gain	588	16365408	42509154	Age_at_puberty	region1inRegion2	7531
CNVR83	957	7	28525787	28565312	gain	588	16365408	42509154	Age_at_puberty	region1inRegion2	39525
CNVR73	934	7	22207228	22236111	gain	6036	16864628	24848007	backfat_between_the_last_3rd_and_4th_lumbar	region1inRegion2	28883
CNVR74	938	7	23307667	23658110	loss-gain	6036	16864628	24848007	backfat_between_the_last_3rd_and_4th_lumbar	region1inRegion2	350443
CNVR75	939	7	23481211	23658110	loss-gain	6036	16864628	24848007	backfat_between_the_last_3rd_and_4th_lumbar	region1inRegion2	176899
CNVR76	942	7	24648309	24653067	loss-gain	6036	16864628	24848007	backfat_between_the_last_3rd_and_4th_lumbar	region1inRegion2	4758
CNVR77	943	7	24735665	24740397	gain	6036	16864628	24848007	backfat_between_the_last_3rd_and_4th_lumbar	region1inRegion2	4732
CNVR73	934	7	22207228	22236111	gain	6037	16864628	24848007	backfat_at_last_rib	region1inRegion2	28883
CNVR74	938	7	23307667	23658110	loss-gain	6037	16864628	24848007	backfat_at_last_rib	region1inRegion2	350443
CNVR75	939	7	23481211	23658110	loss-gain	6037	16864628	24848007	backfat_at_last_rib	region1inRegion2	176899
CNVR76	942	7	24648309	24653067	loss-gain	6037	16864628	24848007	backfat_at_last_rib	region1inRegion2	4758
CNVR77	943	7	24735665	24740397	gain	6037	16864628	24848007	backfat_at_last_rib	region1inRegion2	4732
CNVR73	934	7	22207228	22236111	gain	135752	17460564	36092300	Backfat_at_first_rib	region1inRegion2	28883
CNVR74	938	7	23307667	23658110	loss-gain	135752	17460564	36092300	Backfat_at_first_rib	region1inRegion2	350443
CNVR75	939	7	23481211	23658110	loss-gain	135752	17460564	36092300	Backfat_at_first_rib	region1inRegion2	176899
CNVR76	942	7	24648309	24653067	loss-gain	135752	17460564	36092300	Backfat_at_first_rib	region1inRegion2	4758
CNVR77	943	7	24735665	24740397	gain	135752	17460564	36092300	Backfat_at_first_rib	region1inRegion2	4732
CNVR78	944	7	25359190	25399904	gain	135752	17460564	36092300	Backfat_at_first_rib	region1inRegion2	40714
CNVR79	945	7	25488796	25557156	gain	135752	17460564	36092300	Backfat_at_first_rib	region1inRegion2	68360
CNVR80	946	7	25785853	25888939	gain	135752	17460564	36092300	Backfat_at_first_rib	region1inRegion2	103086
CNVR81	949	7	26267062	26297673	gain	135752	17460564	36092300	Backfat_at_first_rib	region1inRegion2	30611
CNVR82	951	7	26600964	26608495	gain	135752	17460564	36092300	Backfat_at_first_rib	region1inRegion2	7531
CNVR83	957	7	28525787	28565312	gain	135752	17460564	36092300	Backfat_at_first_rib	region1inRegion2	39525
CNVR73	934	7	22207228	22236111	gain	18000	18153031	36993248	Average_backfat_thickness	region1inRegion2	28883
CNVR74	938	7	23307667	23658110	loss-gain	18000	18153031	36993248	Average_backfat_thickness	region1inRegion2	350443
CNVR75	939	7	23481211	23658110	loss-gain	18000	18153031	36993248	Average_backfat_thickness	region1inRegion2	176899
CNVR76	942	7	24648309	24653067	loss-gain	18000	18153031	36993248	Average_backfat_thickness	region1inRegion2	4758
CNVR77	943	7	24735665	24740397	gain	18000	18153031	36993248	Average_backfat_thickness	region1inRegion2	4732
CNVR78	944	7	25359190	25399904	gain	18000	18153031	36993248	Average_backfat_thickness	region1inRegion2	40714
CNVR79	945	7	25488796	25557156	gain	18000	18153031	36993248	Average_backfat_thickness	region1inRegion2	68360
CNVR80	946	7	25785853	25888939	gain	18000	18153031	36993248	Average_backfat_thickness	region1inRegion2	103086
CNVR81	949	7	26267062	26297673	gain	18000	18153031	36993248	Average_backfat_thickness	region1inRegion2	30611
CNVR82	951	7	26600964	26608495	gain	18000	18153031	36993248	Average_backfat_thickness	region1inRegion2	7531
CNVR83	957	7	28525787	28565312	gain	18000	18153031	36993248	Average_backfat_thickness	region1inRegion2	39525
CNVR73	934	7	22207228	22236111	gain	584	18392887	31164815	Teat_number	region1inRegion2	28883
CNVR74	938	7	23307667	23658110	loss-gain	584	18392887	31164815	Teat_number	region1inRegion2	350443
CNVR75	939	7	23481211	23658110	loss-gain	584	18392887	31164815	Teat_number	region1inRegion2	176899
CNVR76	942	7	24648309	24653067	loss-gain	584	18392887	31164815	Teat_number	region1inRegion2	4758
CNVR77	943	7	24735665	24740397	gain	584	18392887	31164815	Teat_number	region1inRegion2	4732
CNVR78	944	7	25359190	25399904	gain	584	18392887	31164815	Teat_number	region1inRegion2	40714
CNVR79	945	7	25488796	25557156	gain	584	18392887	31164815	Teat_number	region1inRegion2	68360
CNVR80	946	7	25785853	25888939	gain	584	18392887	31164815	Teat_number	region1inRegion2	103086
CNVR81	949	7	26267062	26297673	gain	584	18392887	31164815	Teat_number	region1inRegion2	30611
CNVR82	951	7	26600964	26608495	gain	584	18392887	31164815	Teat_number	region1inRegion2	7531
CNVR83	957	7	28525787	28565312	gain	584	18392887	31164815	Teat_number	region1inRegion2	39525
CNVR73	934	7	22207228	22236111	gain	3880	18418873	50310118	Leaf_fat_weight	region1inRegion2	28883
CNVR74	938	7	23307667	23658110	loss-gain	3880	18418873	50310118	Leaf_fat_weight	region1inRegion2	350443
CNVR75	939	7	23481211	23658110	loss-gain	3880	18418873	50310118	Leaf_fat_weight	region1inRegion2	176899
CNVR76	942	7	24648309	24653067	loss-gain	3880	18418873	50310118	Leaf_fat_weight	region1inRegion2	4758
CNVR77	943	7	24735665	24740397	gain	3880	18418873	50310118	Leaf_fat_weight	region1inRegion2	4732
CNVR78	944	7	25359190	25399904	gain	3880	18418873	50310118	Leaf_fat_weight	region1inRegion2	40714
CNVR79	945	7	25488796	25557156	gain	3880	18418873	50310118	Leaf_fat_weight	region1inRegion2	68360
CNVR80	946	7	25785853	25888939	gain	3880	18418873	50310118	Leaf_fat_weight	region1inRegion2	103086
CNVR81	949	7	26267062	26297673	gain	3880	18418873	50310118	Leaf_fat_weight	region1inRegion2	30611
CNVR82	951	7	26600964	26608495	gain	3880	18418873	50310118	Leaf_fat_weight	region1inRegion2	7531
CNVR83	957	7	28525787	28565312	gain	3880	18418873	50310118	Leaf_fat_weight	region1inRegion2	39525
CNVR73	934	7	22207228	22236111	gain	18012	18501425	45102432	Fat_protein_content	region1inRegion2	28883
CNVR74	938	7	23307667	23658110	loss-gain	18012	18501425	45102432	Fat_protein_content	region1inRegion2	350443
CNVR75	939	7	23481211	23658110	loss-gain	18012	18501425	45102432	Fat_protein_content	region1inRegion2	176899
CNVR76	942	7	24648309	24653067	loss-gain	18012	18501425	45102432	Fat_protein_content	region1inRegion2	4758
CNVR77	943	7	24735665	24740397	gain	18012	18501425	45102432	Fat_protein_content	region1inRegion2	4732
CNVR78	944	7	25359190	25399904	gain	18012	18501425	45102432	Fat_protein_content	region1inRegion2	40714
CNVR79	945	7	25488796	25557156	gain	18012	18501425	45102432	Fat_protein_content	region1inRegion2	68360
CNVR80	946	7	25785853	25888939	gain	18012	18501425	45102432	Fat_protein_content	region1inRegion2	103086
CNVR81	949	7	26267062	26297673	gain	18012	18501425	45102432	Fat_protein_content	region1inRegion2	30611
CNVR82	951	7	26600964	26608495	gain	18012	18501425	45102432	Fat_protein_content	region1inRegion2	7531
CNVR83	957	7	28525787	28565312	gain	18012	18501425	45102432	Fat_protein_content	region1inRegion2	39525
CNVR73	934	7	22207228	22236111	gain	18017	18501425	45102432	NADP-malate_dehydrogenase_activity	region1inRegion2	28883
CNVR74	938	7	23307667	23658110	loss-gain	18017	18501425	45102432	NADP-malate_dehydrogenase_activity	region1inRegion2	350443
CNVR75	939	7	23481211	23658110	loss-gain	18017	18501425	45102432	NADP-malate_dehydrogenase_activity	region1inRegion2	176899
CNVR76	942	7	24648309	24653067	loss-gain	18017	18501425	45102432	NADP-malate_dehydrogenase_activity	region1inRegion2	4758
CNVR77	943	7	24735665	24740397	gain	18017	18501425	45102432	NADP-malate_dehydrogenase_activity	region1inRegion2	4732
CNVR78	944	7	25359190	25399904	gain	18017	18501425	45102432	NADP-malate_dehydrogenase_activity	region1inRegion2	40714
CNVR79	945	7	25488796	25557156	gain	18017	18501425	45102432	NADP-malate_dehydrogenase_activity	region1inRegion2	68360
CNVR80	946	7	25785853	25888939	gain	18017	18501425	45102432	NADP-malate_dehydrogenase_activity	region1inRegion2	103086
CNVR81	949	7	26267062	26297673	gain	18017	18501425	45102432	NADP-malate_dehydrogenase_activity	region1inRegion2	30611
CNVR82	951	7	26600964	26608495	gain	18017	18501425	45102432	NADP-malate_dehydrogenase_activity	region1inRegion2	7531
CNVR83	957	7	28525787	28565312	gain	18017	18501425	45102432	NADP-malate_dehydrogenase_activity	region1inRegion2	39525
CNVR73	934	7	22207228	22236111	gain	18023	18501425	45102432	Adipocyte_diameter	region1inRegion2	28883
CNVR74	938	7	23307667	23658110	loss-gain	18023	18501425	45102432	Adipocyte_diameter	region1inRegion2	350443
CNVR75	939	7	23481211	23658110	loss-gain	18023	18501425	45102432	Adipocyte_diameter	region1inRegion2	176899
CNVR76	942	7	24648309	24653067	loss-gain	18023	18501425	45102432	Adipocyte_diameter	region1inRegion2	4758
CNVR77	943	7	24735665	24740397	gain	18023	18501425	45102432	Adipocyte_diameter	region1inRegion2	4732
CNVR78	944	7	25359190	25399904	gain	18023	18501425	45102432	Adipocyte_diameter	region1inRegion2	40714
CNVR79	945	7	25488796	25557156	gain	18023	18501425	45102432	Adipocyte_diameter	region1inRegion2	68360
CNVR80	946	7	25785853	25888939	gain	18023	18501425	45102432	Adipocyte_diameter	region1inRegion2	103086
CNVR81	949	7	26267062	26297673	gain	18023	18501425	45102432	Adipocyte_diameter	region1inRegion2	30611
CNVR82	951	7	26600964	26608495	gain	18023	18501425	45102432	Adipocyte_diameter	region1inRegion2	7531
CNVR83	957	7	28525787	28565312	gain	18023	18501425	45102432	Adipocyte_diameter	region1inRegion2	39525
CNVR73	934	7	22207228	22236111	gain	18031	18501425	45102432	Adipocyte_diameter	region1inRegion2	28883
CNVR74	938	7	23307667	23658110	loss-gain	18031	18501425	45102432	Adipocyte_diameter	region1inRegion2	350443
CNVR75	939	7	23481211	23658110	loss-gain	18031	18501425	45102432	Adipocyte_diameter	region1inRegion2	176899
CNVR76	942	7	24648309	24653067	loss-gain	18031	18501425	45102432	Adipocyte_diameter	region1inRegion2	4758
CNVR77	943	7	24735665	24740397	gain	18031	18501425	45102432	Adipocyte_diameter	region1inRegion2	4732
CNVR78	944	7	25359190	25399904	gain	18031	18501425	45102432	Adipocyte_diameter	region1inRegion2	40714
CNVR79	945	7	25488796	25557156	gain	18031	18501425	45102432	Adipocyte_diameter	region1inRegion2	68360
CNVR80	946	7	25785853	25888939	gain	18031	18501425	45102432	Adipocyte_diameter	region1inRegion2	103086
CNVR81	949	7	26267062	26297673	gain	18031	18501425	45102432	Adipocyte_diameter	region1inRegion2	30611
CNVR82	951	7	26600964	26608495	gain	18031	18501425	45102432	Adipocyte_diameter	region1inRegion2	7531
CNVR83	957	7	28525787	28565312	gain	18031	18501425	45102432	Adipocyte_diameter	region1inRegion2	39525
CNVR73	934	7	22207228	22236111	gain	5204	18670348	92531110	backfat_above_muscle_dorsi	region1inRegion2	28883
CNVR74	938	7	23307667	23658110	loss-gain	5204	18670348	92531110	backfat_above_muscle_dorsi	region1inRegion2	350443

CNVR75	939	7	23481211	23658110	loss-gain	5204	18670348	92531110	backfat_above_muscle_dorsi	region1inRegion2	176899
CNVR76	942	7	24648309	24653067	loss-gain	5204	18670348	92531110	backfat_above_muscle_dorsi	region1inRegion2	4758
CNVR77	943	7	24735665	24740397	gain	5204	18670348	92531110	backfat_above_muscle_dorsi	region1inRegion2	4732
CNVR78	944	7	25359190	25399904	gain	5204	18670348	92531110	backfat_above_muscle_dorsi	region1inRegion2	40714
CNVR79	945	7	25488796	25557156	gain	5204	18670348	92531110	backfat_above_muscle_dorsi	region1inRegion2	68360
CNVR80	946	7	25785853	25888939	gain	5204	18670348	92531110	backfat_above_muscle_dorsi	region1inRegion2	103086
CNVR81	949	7	26267062	26297673	gain	5204	18670348	92531110	backfat_above_muscle_dorsi	region1inRegion2	30611
CNVR82	951	7	26600964	26608495	gain	5204	18670348	92531110	backfat_above_muscle_dorsi	region1inRegion2	7531
CNVR83	957	7	28525787	28565312	gain	5204	18670348	92531110	backfat_above_muscle_dorsi	region1inRegion2	39525
CNVR84	972	7	58579811	58584542	gain	5204	18670348	92531110	backfat_above_muscle_dorsi	region1inRegion2	4731
CNVR85	975	7	59278633	59284219	loss	5204	18670348	92531110	backfat_above_muscle_dorsi	region1inRegion2	5586
CNVR86	977	7	61355441	61417944	gain	5204	18670348	92531110	backfat_above_muscle_dorsi	region1inRegion2	62503
CNVR87	994	7	82234623	82374446	gain	5204	18670348	92531110	backfat_above_muscle_dorsi	region1inRegion2	139823
CNVR88	1002	7	84728780	84749202	gain	5204	18670348	92531110	backfat_above_muscle_dorsi	region1inRegion2	20422
CNVR73	934	7	22207228	22236111	gain	135745	19288463	37710365	Backfat_at_rump	region1inRegion2	28883
CNVR74	938	7	23307667	23658110	loss-gain	135745	19288463	37710365	Backfat_at_rump	region1inRegion2	350443
CNVR75	939	7	23481211	23658110	loss-gain	135745	19288463	37710365	Backfat_at_rump	region1inRegion2	176899
CNVR76	942	7	24648309	24653067	loss-gain	135745	19288463	37710365	Backfat_at_rump	region1inRegion2	4758
CNVR77	943	7	24735665	24740397	gain	135745	19288463	37710365	Backfat_at_rump	region1inRegion2	4732
CNVR78	944	7	25359190	25399904	gain	135745	19288463	37710365	Backfat_at_rump	region1inRegion2	40714
CNVR79	945	7	25488796	25557156	gain	135745	19288463	37710365	Backfat_at_rump	region1inRegion2	68360
CNVR80	946	7	25785853	25888939	gain	135745	19288463	37710365	Backfat_at_rump	region1inRegion2	103086
CNVR81	949	7	26267062	26297673	gain	135745	19288463	37710365	Backfat_at_rump	region1inRegion2	30611
CNVR82	951	7	26600964	26608495	gain	135745	19288463	37710365	Backfat_at_rump	region1inRegion2	7531
CNVR83	957	7	28525787	28565312	gain	135745	19288463	37710365	Backfat_at_rump	region1inRegion2	39525
CNVR73	934	7	22207228	22236111	gain	135758	19615474	37710365	backfat_at_last_rib	region1inRegion2	28883
CNVR74	938	7	23307667	23658110	loss-gain	135758	19615474	37710365	backfat_at_last_rib	region1inRegion2	350443
CNVR75	939	7	23481211	23658110	loss-gain	135758	19615474	37710365	backfat_at_last_rib	region1inRegion2	176899
CNVR76	942	7	24648309	24653067	loss-gain	135758	19615474	37710365	backfat_at_last_rib	region1inRegion2	4758
CNVR77	943	7	24735665	24740397	gain	135758	19615474	37710365	backfat_at_last_rib	region1inRegion2	4732
CNVR78	944	7	25359190	25399904	gain	135758	19615474	37710365	backfat_at_last_rib	region1inRegion2	40714
CNVR79	945	7	25488796	25557156	gain	135758	19615474	37710365	backfat_at_last_rib	region1inRegion2	68360
CNVR80	946	7	25785853	25888939	gain	135758	19615474	37710365	backfat_at_last_rib	region1inRegion2	103086
CNVR81	949	7	26267062	26297673	gain	135758	19615474	37710365	backfat_at_last_rib	region1inRegion2	30611
CNVR82	951	7	26600964	26608495	gain	135758	19615474	37710365	backfat_at_last_rib	region1inRegion2	7531
CNVR83	957	7	28525787	28565312	gain	135758	19615474	37710365	backfat_at_last_rib	region1inRegion2	39525
CNVR73	934	7	22207228	22236111	gain	126123	19900584	32908117	Conformation_score	region1inRegion2	28883
CNVR74	938	7	23307667	23658110	loss-gain	126123	19900584	32908117	Conformation_score	region1inRegion2	350443
CNVR75	939	7	23481211	23658110	loss-gain	126123	19900584	32908117	Conformation_score	region1inRegion2	176899
CNVR76	942	7	24648309	24653067	loss-gain	126123	19900584	32908117	Conformation_score	region1inRegion2	4758
CNVR77	943	7	24735665	24740397	gain	126123	19900584	32908117	Conformation_score	region1inRegion2	4732
CNVR78	944	7	25359190	25399904	gain	126123	19900584	32908117	Conformation_score	region1inRegion2	40714
CNVR79	945	7	25488796	25557156	gain	126123	19900584	32908117	Conformation_score	region1inRegion2	68360
CNVR80	946	7	25785853	25888939	gain	126123	19900584	32908117	Conformation_score	region1inRegion2	103086
CNVR81	949	7	26267062	26297673	gain	126123	19900584	32908117	Conformation_score	region1inRegion2	30611
CNVR82	951	7	26600964	26608495	gain	126123	19900584	32908117	Conformation_score	region1inRegion2	7531
CNVR83	957	7	28525787	28565312	gain	126123	19900584	32908117	Conformation_score	region1inRegion2	39525
CNVR73	934	7	22207228	22236111	gain	126124	19900584	32908117	Conformation_score	region1inRegion2	28883
CNVR74	938	7	23307667	23658110	loss-gain	126124	19900584	32908117	Conformation_score	region1inRegion2	350443
CNVR75	939	7	23481211	23658110	loss-gain	126124	19900584	32908117	Conformation_score	region1inRegion2	176899
CNVR76	942	7	24648309	24653067	loss-gain	126124	19900584	32908117	Conformation_score	region1inRegion2	4758
CNVR77	943	7	24735665	24740397	gain	126124	19900584	32908117	Conformation_score	region1inRegion2	4732
CNVR78	944	7	25359190	25399904	gain	126124	19900584	32908117	Conformation_score	region1inRegion2	40714
CNVR79	945	7	25488796	25557156	gain	126124	19900584	32908117	Conformation_score	region1inRegion2	68360
CNVR80	946	7	25785853	25888939	gain	126124	19900584	32908117	Conformation_score	region1inRegion2	103086
CNVR81	949	7	26267062	26297673	gain	126124	19900584	32908117	Conformation_score	region1inRegion2	30611
CNVR82	951	7	26600964	26608495	gain	126124	19900584	32908117	Conformation_score	region1inRegion2	7531
CNVR83	957	7	28525787	28565312	gain	126124	19900584	32908117	Conformation_score	region1inRegion2	39525
CNVR73	934	7	22207228	22236111	gain	5220	20130643	132120394	Shoulder_subcutaneous_fat_thickness	region1inRegion2	28883
CNVR74	938	7	23307667	23658110	loss-gain	5220	20130643	132120394	Shoulder_subcutaneous_fat_thickness	region1inRegion2	350443
CNVR75	939	7	23481211	23658110	loss-gain	5220	20130643	132120394	Shoulder_subcutaneous_fat_thickness	region1inRegion2	176899
CNVR76	942	7	24648309	24653067	loss-gain	5220	20130643	132120394	Shoulder_subcutaneous_fat_thickness	region1inRegion2	4758
CNVR77	943	7	24735665	24740397	gain	5220	20130643	132120394	Shoulder_subcutaneous_fat_thickness	region1inRegion2	4732
CNVR78	944	7	25359190	25399904	gain	5220	20130643	132120394	Shoulder_subcutaneous_fat_thickness	region1inRegion2	40714
CNVR79	945	7	25488796	25557156	gain	5220	20130643	132120394	Shoulder_subcutaneous_fat_thickness	region1inRegion2	68360
CNVR80	946	7	25785853	25888939	gain	5220	20130643	132120394	Shoulder_subcutaneous_fat_thickness	region1inRegion2	103086
CNVR81	949	7	26267062	26297673	gain	5220	20130643	132120394	Shoulder_subcutaneous_fat_thickness	region1inRegion2	30611
CNVR82	951	7	26600964	26608495	gain	5220	20130643	132120394	Shoulder_subcutaneous_fat_thickness	region1inRegion2	7531
CNVR83	957	7	28525787	28565312	gain	5220	20130643	132120394	Shoulder_subcutaneous_fat_thickness	region1inRegion2	39525
CNVR84	972	7	58579811	58584542	gain	5220	20130643	132120394	Shoulder_subcutaneous_fat_thickness	region1inRegion2	4731
CNVR85	975	7	59278633	59284219	loss	5220	20130643	132120394	Shoulder_subcutaneous_fat_thickness	region1inRegion2	5586
CNVR86	977	7	61355441	61417944	gain	5220	20130643	132120394	Shoulder_subcutaneous_fat_thickness	region1inRegion2	62503
CNVR87	994	7	82234623	82374446	gain	5220	20130643	132120394	Shoulder_subcutaneous_fat_thickness	region1inRegion2	139823
CNVR88	1002	7	84728780	84749202	gain	5220	20130643	132120394	Shoulder_subcutaneous_fat_thickness	region1inRegion2	20422
CNVR89	1011	7	102790067	102823288	gain	5220	20130643	132120394	Shoulder_subcutaneous_fat_thickness	region1inRegion2	33221
CNVR90	1021	7	111620166	111623056	loss	5220	20130643	132120394	Shoulder_subcutaneous_fat_thickness	region1inRegion2	2890
CNVR91	1035	7	131449863	131460402	gain	5220	20130643	132120394	Shoulder_subcutaneous_fat_thickness	region1inRegion2	10539
CNVR73	934	7	22207228	22236111	gain	5211	20188985	58221858	backfat_at_mid-back	region1inRegion2	28883
CNVR74	938	7	23307667	23658110	loss-gain	5211	20188985	58221858	backfat_at_mid-back	region1inRegion2	350443
CNVR75	939	7	23481211	23658110	loss-gain	5211	20188985	58221858	backfat_at_mid-back	region1inRegion2	176899
CNVR76	942	7	24648309	24653067	loss-gain	5211	20188985	58221858	backfat_at_mid-back	region1inRegion2	4758
CNVR77	943	7	24735665	24740397	gain	5211	20188985	58221858	backfat_at_mid-back	region1inRegion2	4732
CNVR78	944	7	25359190	25399904	gain	5211	20188985	58221858	backfat_at_mid-back	region1inRegion2	40714
CNVR79	945	7	25488796	25557156	gain	5211	20188985	58221858	backfat_at_mid-back	region1inRegion2	68360
CNVR80	946	7	25785853	25888939	gain	5211	20188985	58221858	backfat_at_mid-back	region1inRegion2	103086
CNVR81	949	7	26267062	26297673	gain	5211	20188985	58221858	backfat_at_mid-back	region1inRegion2	30611
CNVR82	951	7	26600964	26608495	gain	5211	20188985	58221858	backfat_at_mid-back	region1inRegion2	7531
CNVR83	957	7	28525787	28565312	gain	5211	20188985	58221858	backfat_at_mid-back	region1inRegion2	39525
CNVR73	934	7	22207228	22236111	gain	5212	20320191	84675360	backfat_at_mid-back	region1inRegion2	28883
CNVR74	938	7	23307667	23658110	loss-gain	5212	20320191	84675360	backfat_at_mid-back	region1inRegion2	350443
CNVR75	939	7	23481211	23658110	loss-gain	5212	20320191	84675360	backfat_at_mid-back	region1inRegion2	176899
CNVR76	942	7	24648309	24653067	loss-gain	5212	20320191	84675360	backfat_at_mid-back	region1inRegion2	4758
CNVR77	943	7	24735665	24740397	gain	5212	20320191	84675360	backfat_at_mid-back	region1inRegion2	4732
CNVR78	944	7	25359190	25399904	gain	5212	20320191	84675360	backfat_at_mid-back	region1inRegion2	40714
CNVR79	945	7	25488796	25557156	gain	5212	20320191	84675360	backfat_at_mid-back	region1inRegion2	68360
CNVR80	946	7	25785853	25888939	gain	5212	20320191	84675360	backfat_at_mid-back	region1inRegion2	103086
CNVR81	949	7	26267062	26297673	gain	5212	20320191	84675360	backfat_at_mid-back	region1inRegion2	30611
CNVR82	951	7	26600964	26608495	gain	5212	20320191	84675360	backfat_at_mid-back	region1inRegion2	7531
CNVR83	957	7	28525787	28565312	gain	5212	20320191	84675360	backfat_at_mid-back	region1inRegion2	39525
CNVR84	972	7	58579811	58584542	gain	5212	20320191	84675360	backfat_at_mid-back	region1inRegion2	4731
CNVR85	975	7	59278633	59284219	loss	5212	20320191	84675360	backfat_at_mid-back	region1inRegion2	5586
CNVR86	977	7	61355441	61417944	gain	5212	20320191	84675360	backfat_at_mid-back	region1inRegion2	62503
CNVR87	994	7	82234623	82374446	gain	5212	20320191	84675360	backfat_at_mid-back	region1inRegion2	139823
CNVR73	934	7	22207228	22236111	gain	3993	20633784	32380448	Japanese_color_scale	region1inRegion2	28883
CNVR74	938	7	23307667	23658110	loss-gain	3993	20633784	32380448	Japanese_color_scale	region1inRegion2	350443
CNVR75	939	7	23481211	23658110	loss-gain	3993	20633784	32380448	Japanese_color_scale	region1inRegion2	176899
CNVR76	942	7	24648309	24653067	loss-gain	3993	20633784	32380448	Japanese_color_scale	region1inRegion2	4758
CNVR77	943	7	24735665	24740397	gain	3993	20633784	32380448	Japanese_color_scale	region1inRegion2	4732
CNVR78	944	7	25359190	25399904	gain	3993	20633784	32380448	Japanese_color_scale	region1inRegion2	40714
CNVR79	945	7	25488796	25557156	gain	3993	20633784	32380448	Japanese_color_scale	region1inRegion2	68360
CNVR80	946	7	25785853	25888939	gain	3993	20633784	32380448	Japanese_color_scale	region1inRegion2	103086
CNVR81	949	7	26267062	26297673	gain	3993	20633784	32380448	Japanese_color_scale	region1inRegion2	30611
CNVR82	951	7	26600964	26608495	gain	3993	20633784	32380448	Japanese_color_scale	region1inRegion2	7531
CNVR83	957	7	28525787	28565312	gain	3993	20633784	32380448	Japanese_color_scale	region1inRegion2	39525
CNVR73	934	7	22207228	22236111	gain	3995	20633784	32380448	CIE-L*	region1inRegion2	28883
CNVR74	938	7	23307667	23658110	loss-gain	3995	20633784	32380448	CIE-L*	region1inRegion2	350443
CNVR75	939	7	23481211	23658110	loss-gain	3995	20633784	32380448	CIE-L*	region1inRegion2	176899
CNVR76	942	7	24648309	24653067	loss-gain	3995	20633784	32380448	CIE-L*	region1inRegion2	4758
CNVR77	943	7	24735665	24740397	gain	3995	20633784	32380448	CIE-L*	region1inRegion2	4732
CNVR78	944	7	25359190	25399904	gain	3995	20633784	32380448	CIE-L*	region1inRegion2	40714
CNVR79	945	7	25488796	25557156	gain	3995	20633784	32380448	CIE-L*	region1inRegion2	68360
CNVR80	946	7	25785853	25888939	gain	3995	20633784	32380448	CIE-L*	region1inRegion2	103086

CNVR81	949	7	26267062	26297673	gain	3995	20633784	32380448	CIE-L*	region1inRegion2	30611
CNVR82	951	7	26600964	26608495	gain	3995	20633784	32380448	CIE-L*	region1inRegion2	7531
CNVR83	957	7	28525787	28565312	gain	3995	20633784	32380448	CIE-L*	region1inRegion2	39525
CNVR73	934	7	22207228	22236111	gain	3997	20633784	32380448	Shear_force_at_first_peak	region1inRegion2	28883
CNVR74	938	7	23307667	23658110	loss-gain	3997	20633784	32380448	Shear_force_at_first_peak	region1inRegion2	350443
CNVR75	939	7	23481211	23658110	loss-gain	3997	20633784	32380448	Shear_force_at_first_peak	region1inRegion2	176899
CNVR76	942	7	24648309	24653067	loss-gain	3997	20633784	32380448	Shear_force_at_first_peak	region1inRegion2	4758
CNVR77	943	7	24735665	24740397	gain	3997	20633784	32380448	Shear_force_at_first_peak	region1inRegion2	4732
CNVR78	944	7	25359190	25399904	gain	3997	20633784	32380448	Shear_force_at_first_peak	region1inRegion2	40714
CNVR79	945	7	25488796	25557156	gain	3997	20633784	32380448	Shear_force_at_first_peak	region1inRegion2	68360
CNVR80	946	7	25785853	25888939	gain	3997	20633784	32380448	Shear_force_at_first_peak	region1inRegion2	103086
CNVR81	949	7	26267062	26297673	gain	3997	20633784	32380448	Shear_force_at_first_peak	region1inRegion2	30611
CNVR82	951	7	26600964	26608495	gain	3997	20633784	32380448	Shear_force_at_first_peak	region1inRegion2	7531
CNVR83	957	7	28525787	28565312	gain	3997	20633784	32380448	Shear_force_at_first_peak	region1inRegion2	39525
CNVR73	934	7	22207228	22236111	gain	5436	20633784	36993248	backfat_at_last_rib	region1inRegion2	28883
CNVR74	938	7	23307667	23658110	loss-gain	5436	20633784	36993248	backfat_at_last_rib	region1inRegion2	350443
CNVR75	939	7	23481211	23658110	loss-gain	5436	20633784	36993248	backfat_at_last_rib	region1inRegion2	176899
CNVR76	942	7	24648309	24653067	loss-gain	5436	20633784	36993248	backfat_at_last_rib	region1inRegion2	4758
CNVR77	943	7	24735665	24740397	gain	5436	20633784	36993248	backfat_at_last_rib	region1inRegion2	4732
CNVR78	944	7	25359190	25399904	gain	5436	20633784	36993248	backfat_at_last_rib	region1inRegion2	40714
CNVR79	945	7	25488796	25557156	gain	5436	20633784	36993248	backfat_at_last_rib	region1inRegion2	68360
CNVR80	946	7	25785853	25888939	gain	5436	20633784	36993248	backfat_at_last_rib	region1inRegion2	103086
CNVR81	949	7	26267062	26297673	gain	5436	20633784	36993248	backfat_at_last_rib	region1inRegion2	30611
CNVR82	951	7	26600964	26608495	gain	5436	20633784	36993248	backfat_at_last_rib	region1inRegion2	7531
CNVR83	957	7	28525787	28565312	gain	5436	20633784	36993248	backfat_at_last_rib	region1inRegion2	39525
CNVR73	934	7	22207228	22236111	gain	12293	20633784	38992356	Red_cell_distribution_width	region1inRegion2	28883
CNVR74	938	7	23307667	23658110	loss-gain	12293	20633784	38992356	Red_cell_distribution_width	region1inRegion2	350443
CNVR75	939	7	23481211	23658110	loss-gain	12293	20633784	38992356	Red_cell_distribution_width	region1inRegion2	176899
CNVR76	942	7	24648309	24653067	loss-gain	12293	20633784	38992356	Red_cell_distribution_width	region1inRegion2	4758
CNVR77	943	7	24735665	24740397	gain	12293	20633784	38992356	Red_cell_distribution_width	region1inRegion2	4732
CNVR78	944	7	25359190	25399904	gain	12293	20633784	38992356	Red_cell_distribution_width	region1inRegion2	40714
CNVR79	945	7	25488796	25557156	gain	12293	20633784	38992356	Red_cell_distribution_width	region1inRegion2	68360
CNVR80	946	7	25785853	25888939	gain	12293	20633784	38992356	Red_cell_distribution_width	region1inRegion2	103086
CNVR81	949	7	26267062	26297673	gain	12293	20633784	38992356	Red_cell_distribution_width	region1inRegion2	30611
CNVR82	951	7	26600964	26608495	gain	12293	20633784	38992356	Red_cell_distribution_width	region1inRegion2	7531
CNVR83	957	7	28525787	28565312	gain	12293	20633784	38992356	Red_cell_distribution_width	region1inRegion2	39525
CNVR73	934	7	22207228	22236111	gain	207	20633784	45102432	Average_backfat_thickness	region1inRegion2	28883
CNVR74	938	7	23307667	23658110	loss-gain	207	20633784	45102432	Average_backfat_thickness	region1inRegion2	350443
CNVR75	939	7	23481211	23658110	loss-gain	207	20633784	45102432	Average_backfat_thickness	region1inRegion2	176899
CNVR76	942	7	24648309	24653067	loss-gain	207	20633784	45102432	Average_backfat_thickness	region1inRegion2	4758
CNVR77	943	7	24735665	24740397	gain	207	20633784	45102432	Average_backfat_thickness	region1inRegion2	4732
CNVR78	944	7	25359190	25399904	gain	207	20633784	45102432	Average_backfat_thickness	region1inRegion2	40714
CNVR79	945	7	25488796	25557156	gain	207	20633784	45102432	Average_backfat_thickness	region1inRegion2	68360
CNVR80	946	7	25785853	25888939	gain	207	20633784	45102432	Average_backfat_thickness	region1inRegion2	103086
CNVR81	949	7	26267062	26297673	gain	207	20633784	45102432	Average_backfat_thickness	region1inRegion2	30611
CNVR82	951	7	26600964	26608495	gain	207	20633784	45102432	Average_backfat_thickness	region1inRegion2	7531
CNVR83	957	7	28525787	28565312	gain	207	20633784	45102432	Average_backfat_thickness	region1inRegion2	39525
CNVR73	934	7	22207228	22236111	gain	208	20633784	45102432	Average_backfat_thickness	region1inRegion2	28883
CNVR74	938	7	23307667	23658110	loss-gain	208	20633784	45102432	Average_backfat_thickness	region1inRegion2	350443
CNVR75	939	7	23481211	23658110	loss-gain	208	20633784	45102432	Average_backfat_thickness	region1inRegion2	176899
CNVR76	942	7	24648309	24653067	loss-gain	208	20633784	45102432	Average_backfat_thickness	region1inRegion2	4758
CNVR77	943	7	24735665	24740397	gain	208	20633784	45102432	Average_backfat_thickness	region1inRegion2	4732
CNVR78	944	7	25359190	25399904	gain	208	20633784	45102432	Average_backfat_thickness	region1inRegion2	40714
CNVR79	945	7	25488796	25557156	gain	208	20633784	45102432	Average_backfat_thickness	region1inRegion2	68360
CNVR80	946	7	25785853	25888939	gain	208	20633784	45102432	Average_backfat_thickness	region1inRegion2	103086
CNVR81	949	7	26267062	26297673	gain	208	20633784	45102432	Average_backfat_thickness	region1inRegion2	30611
CNVR82	951	7	26600964	26608495	gain	208	20633784	45102432	Average_backfat_thickness	region1inRegion2	7531
CNVR83	957	7	28525787	28565312	gain	208	20633784	45102432	Average_backfat_thickness	region1inRegion2	39525
CNVR73	934	7	22207228	22236111	gain	209	20633784	45102432	Average_backfat_thickness	region1inRegion2	28883
CNVR74	938	7	23307667	23658110	loss-gain	209	20633784	45102432	Average_backfat_thickness	region1inRegion2	350443
CNVR75	939	7	23481211	23658110	loss-gain	209	20633784	45102432	Average_backfat_thickness	region1inRegion2	176899
CNVR76	942	7	24648309	24653067	loss-gain	209	20633784	45102432	Average_backfat_thickness	region1inRegion2	4758
CNVR77	943	7	24735665	24740397	gain	209	20633784	45102432	Average_backfat_thickness	region1inRegion2	4732
CNVR78	944	7	25359190	25399904	gain	209	20633784	45102432	Average_backfat_thickness	region1inRegion2	40714
CNVR79	945	7	25488796	25557156	gain	209	20633784	45102432	Average_backfat_thickness	region1inRegion2	68360
CNVR80	946	7	25785853	25888939	gain	209	20633784	45102432	Average_backfat_thickness	region1inRegion2	103086
CNVR81	949	7	26267062	26297673	gain	209	20633784	45102432	Average_backfat_thickness	region1inRegion2	30611
CNVR82	951	7	26600964	26608495	gain	209	20633784	45102432	Average_backfat_thickness	region1inRegion2	7531
CNVR83	957	7	28525787	28565312	gain	209	20633784	45102432	Average_backfat_thickness	region1inRegion2	39525
CNVR73	934	7	22207228	22236111	gain	210	20633784	45102432	Average_backfat_thickness	region1inRegion2	28883
CNVR74	938	7	23307667	23658110	loss-gain	210	20633784	45102432	Average_backfat_thickness	region1inRegion2	350443
CNVR75	939	7	23481211	23658110	loss-gain	210	20633784	45102432	Average_backfat_thickness	region1inRegion2	176899
CNVR76	942	7	24648309	24653067	loss-gain	210	20633784	45102432	Average_backfat_thickness	region1inRegion2	4758
CNVR77	943	7	24735665	24740397	gain	210	20633784	45102432	Average_backfat_thickness	region1inRegion2	4732
CNVR78	944	7	25359190	25399904	gain	210	20633784	45102432	Average_backfat_thickness	region1inRegion2	40714
CNVR79	945	7	25488796	25557156	gain	210	20633784	45102432	Average_backfat_thickness	region1inRegion2	68360
CNVR80	946	7	25785853	25888939	gain	210	20633784	45102432	Average_backfat_thickness	region1inRegion2	103086
CNVR81	949	7	26267062	26297673	gain	210	20633784	45102432	Average_backfat_thickness	region1inRegion2	30611
CNVR82	951	7	26600964	26608495	gain	210	20633784	45102432	Average_backfat_thickness	region1inRegion2	7531
CNVR83	957	7	28525787	28565312	gain	210	20633784	45102432	Average_backfat_thickness	region1inRegion2	39525
CNVR73	934	7	22207228	22236111	gain	211	20633784	45102432	Average_backfat_thickness	region1inRegion2	28883
CNVR74	938	7	23307667	23658110	loss-gain	211	20633784	45102432	Average_backfat_thickness	region1inRegion2	350443
CNVR75	939	7	23481211	23658110	loss-gain	211	20633784	45102432	Average_backfat_thickness	region1inRegion2	176899
CNVR76	942	7	24648309	24653067	loss-gain	211	20633784	45102432	Average_backfat_thickness	region1inRegion2	4758
CNVR77	943	7	24735665	24740397	gain	211	20633784	45102432	Average_backfat_thickness	region1inRegion2	4732
CNVR78	944	7	25359190	25399904	gain	211	20633784	45102432	Average_backfat_thickness	region1inRegion2	40714
CNVR79	945	7	25488796	25557156	gain	211	20633784	45102432	Average_backfat_thickness	region1inRegion2	68360
CNVR80	946	7	25785853	25888939	gain	211	20633784	45102432	Average_backfat_thickness	region1inRegion2	103086
CNVR81	949	7	26267062	26297673	gain	211	20633784	45102432	Average_backfat_thickness	region1inRegion2	30611
CNVR82	951	7	26600964	26608495	gain	211	20633784	45102432	Average_backfat_thickness	region1inRegion2	7531
CNVR83	957	7	28525787	28565312	gain	211	20633784	45102432	Average_backfat_thickness	region1inRegion2	39525
CNVR73	934	7	22207228	22236111	gain	3139	20633784	45102432	Body_weight_(birth)	region1inRegion2	28883
CNVR74	938	7	23307667	23658110	loss-gain	3139	20633784	45102432	Body_weight_(birth)	region1inRegion2	350443
CNVR75	939	7	23481211	23658110	loss-gain	3139	20633784	45102432	Body_weight_(birth)	region1inRegion2	176899
CNVR76	942	7	24648309	24653067	loss-gain	3139	20633784	45102432	Body_weight_(birth)	region1inRegion2	4758
CNVR77	943	7	24735665	24740397	gain	3139	20633784	45102432	Body_weight_(birth)	region1inRegion2	4732
CNVR78	944	7	25359190	25399904	gain	3139	20633784	45102432	Body_weight_(birth)	region1inRegion2	40714
CNVR79	945	7	25488796	25557156	gain	3139	20633784	45102432	Body_weight_(birth)	region1inRegion2	68360
CNVR80	946	7	25785853	25888939	gain	3139	20633784	45102432	Body_weight_(birth)	region1inRegion2	103086
CNVR81	949	7	26267062	26297673	gain	3139	20633784	45102432	Body_weight_(birth)	region1inRegion2	30611
CNVR82	951	7	26600964	26608495	gain	3139	20633784	45102432	Body_weight_(birth)	region1inRegion2	7531
CNVR83	957	7	28525787	28565312	gain	3139	20633784	45102432	Body_weight_(birth)	region1inRegion2	39525
CNVR73	934	7	22207228	22236111	gain	3140	20633784	45102432	Body_weight_(weaning)	region1inRegion2	28883
CNVR74	938	7	23307667	23658110	loss-gain	3140	20633784	45102432	Body_weight_(weaning)	region1inRegion2	350443
CNVR75	939	7	23481211	23658110	loss-gain	3140	20633784	45102432	Body_weight_(weaning)	region1inRegion2	176899
CNVR76	942	7	24648309	24653067	loss-gain	3140	20633784	45102432	Body_weight_(weaning)	region1inRegion2	4758
CNVR77	943	7	24735665	24740397	gain	3140	20633784	45102432	Body_weight_(weaning)	region1inRegion2	4732
CNVR78	944	7	25359190	25399904	gain	3140	20633784	45102432	Body_weight_(weaning)	region1inRegion2	40714
CNVR79	945	7	25488796	25557156	gain	3140	20633784	45102432	Body_weight_(weaning)	region1inRegion2	68360
CNVR80	946	7	25785853	25888939	gain	3140	20633784	45102432	Body_weight_(weaning)	region1inRegion2	103086
CNVR81	949	7	26267062	26297673	gain	3140	20633784	45102432	Body_weight_(weaning)	region1inRegion2	30611
CNVR82	951	7	26600964	26608495	gain	3140	20633784	45102432	Body_weight_(weaning)	region1inRegion2	7531
CNVR83	957	7	28525787	28565312	gain	3140	20633784	45102432	Body_weight_(weaning)	region1inRegion2	39525
CNVR73	934	7	22207228	22236111	gain	3141	20633784	45102432	Trimmed_wholesale_product_/_live_weight	region1inRegion2	28883
CNVR74	938	7	23307667	23658110	loss-gain	3141	20633784	45102432	Trimmed_wholesale_product_/_live_weight	region1inRegion2	350443
CNVR75	939	7	23481211	23658110	loss-gain	3141	20633784	45102432	Trimmed_wholesale_product_/_live_weight	region1inRegion2	176899
CNVR76	942	7	24648309	24653067	loss-gain	3141	20633784	45102432	Trimmed_wholesale_product_/_live_weight	region1inRegion2	4758
CNVR77	943	7	24735665	24740397	gain	3141	20633784	45102432	Trimmed_wholesale_product_/_live_weight	region1inRegion2	4732
CNVR78	944	7	25359190	25399904	gain	3141	20633784	45102432	Trimmed_wholesale_product_/_live_weight	region1inRegion2	40714
CNVR79	945	7	25488796	25557156	gain	3141	20633784	45102432	Trimmed_wholesale_product_/_live_weight	region1inRegion2	68360
CNVR80	946	7	25785853	25888939	gain	3141	20633784	45102432	Trimmed_wholesale_product_/_live_weight	region1inRegion2	103086
CNVR81	949	7	26267062	26297673	gain	3141	20633784	45102432	Trimmed_wholesale_product_/_live_weight	region1inRegion2	30611

CNVR82	951	7	26600964	26608495	gain	3141	20633784	45102432	Trimmed_wholesale_product_/_live_weight	region1inRegion2	7531
CNVR83	957	7	28525787	28565312	gain	3141	20633784	45102432	Trimmed_wholesale_product_/_live_weight	region1inRegion2	39525
CNVR73	934	7	22207228	22236111	gain	3142	20633784	45102432	Trimmed_wholesale_product_/_live_weight	region1inRegion2	28883
CNVR74	938	7	23307667	23658110	loss-gain	3142	20633784	45102432	Trimmed_wholesale_product_/_live_weight	region1inRegion2	350443
CNVR75	939	7	23481211	23658110	loss-gain	3142	20633784	45102432	Trimmed_wholesale_product_/_live_weight	region1inRegion2	176899
CNVR76	942	7	24648309	24653067	loss-gain	3142	20633784	45102432	Trimmed_wholesale_product_/_live_weight	region1inRegion2	4758
CNVR77	943	7	24735665	24740397	gain	3142	20633784	45102432	Trimmed_wholesale_product_/_live_weight	region1inRegion2	4732
CNVR78	944	7	25359190	25399904	gain	3142	20633784	45102432	Trimmed_wholesale_product_/_live_weight	region1inRegion2	40714
CNVR79	945	7	25488796	25557156	gain	3142	20633784	45102432	Trimmed_wholesale_product_/_live_weight	region1inRegion2	68360
CNVR80	946	7	25785853	25888939	gain	3142	20633784	45102432	Trimmed_wholesale_product_/_live_weight	region1inRegion2	103086
CNVR81	949	7	26267062	26297673	gain	3142	20633784	45102432	Trimmed_wholesale_product_/_live_weight	region1inRegion2	30611
CNVR82	951	7	26600964	26608495	gain	3142	20633784	45102432	Trimmed_wholesale_product_/_live_weight	region1inRegion2	7531
CNVR83	957	7	28525787	28565312	gain	3142	20633784	45102432	Trimmed_wholesale_product_/_live_weight	region1inRegion2	39525
CNVR73	934	7	22207228	22236111	gain	3143	20633784	45102432	Average_daily_gain	region1inRegion2	28883
CNVR74	938	7	23307667	23658110	loss-gain	3143	20633784	45102432	Average_daily_gain	region1inRegion2	350443
CNVR75	939	7	23481211	23658110	loss-gain	3143	20633784	45102432	Average_daily_gain	region1inRegion2	176899
CNVR76	942	7	24648309	24653067	loss-gain	3143	20633784	45102432	Average_daily_gain	region1inRegion2	4758
CNVR77	943	7	24735665	24740397	gain	3143	20633784	45102432	Average_daily_gain	region1inRegion2	4732
CNVR78	944	7	25359190	25399904	gain	3143	20633784	45102432	Average_daily_gain	region1inRegion2	40714
CNVR79	945	7	25488796	25557156	gain	3143	20633784	45102432	Average_daily_gain	region1inRegion2	68360
CNVR80	946	7	25785853	25888939	gain	3143	20633784	45102432	Average_daily_gain	region1inRegion2	103086
CNVR81	949	7	26267062	26297673	gain	3143	20633784	45102432	Average_daily_gain	region1inRegion2	30611
CNVR82	951	7	26600964	26608495	gain	3143	20633784	45102432	Average_daily_gain	region1inRegion2	7531
CNVR83	957	7	28525787	28565312	gain	3143	20633784	45102432	Average_daily_gain	region1inRegion2	39525
CNVR73	934	7	22207228	22236111	gain	3144	20633784	45102432	Average_daily_gain	region1inRegion2	28883
CNVR74	938	7	23307667	23658110	loss-gain	3144	20633784	45102432	Average_daily_gain	region1inRegion2	350443
CNVR75	939	7	23481211	23658110	loss-gain	3144	20633784	45102432	Average_daily_gain	region1inRegion2	176899
CNVR76	942	7	24648309	24653067	loss-gain	3144	20633784	45102432	Average_daily_gain	region1inRegion2	4758
CNVR77	943	7	24735665	24740397	gain	3144	20633784	45102432	Average_daily_gain	region1inRegion2	4732
CNVR78	944	7	25359190	25399904	gain	3144	20633784	45102432	Average_daily_gain	region1inRegion2	40714
CNVR79	945	7	25488796	25557156	gain	3144	20633784	45102432	Average_daily_gain	region1inRegion2	68360
CNVR80	946	7	25785853	25888939	gain	3144	20633784	45102432	Average_daily_gain	region1inRegion2	103086
CNVR81	949	7	26267062	26297673	gain	3144	20633784	45102432	Average_daily_gain	region1inRegion2	30611
CNVR82	951	7	26600964	26608495	gain	3144	20633784	45102432	Average_daily_gain	region1inRegion2	7531
CNVR83	957	7	28525787	28565312	gain	3144	20633784	45102432	Average_daily_gain	region1inRegion2	39525
CNVR73	934	7	22207228	22236111	gain	3146	20633784	45102432	Average_backfat_thickness	region1inRegion2	28883
CNVR74	938	7	23307667	23658110	loss-gain	3146	20633784	45102432	Average_backfat_thickness	region1inRegion2	350443
CNVR75	939	7	23481211	23658110	loss-gain	3146	20633784	45102432	Average_backfat_thickness	region1inRegion2	176899
CNVR76	942	7	24648309	24653067	loss-gain	3146	20633784	45102432	Average_backfat_thickness	region1inRegion2	4758
CNVR77	943	7	24735665	24740397	gain	3146	20633784	45102432	Average_backfat_thickness	region1inRegion2	4732
CNVR78	944	7	25359190	25399904	gain	3146	20633784	45102432	Average_backfat_thickness	region1inRegion2	40714
CNVR79	945	7	25488796	25557156	gain	3146	20633784	45102432	Average_backfat_thickness	region1inRegion2	68360
CNVR80	946	7	25785853	25888939	gain	3146	20633784	45102432	Average_backfat_thickness	region1inRegion2	103086
CNVR81	949	7	26267062	26297673	gain	3146	20633784	45102432	Average_backfat_thickness	region1inRegion2	30611
CNVR82	951	7	26600964	26608495	gain	3146	20633784	45102432	Average_backfat_thickness	region1inRegion2	7531
CNVR83	957	7	28525787	28565312	gain	3146	20633784	45102432	Average_backfat_thickness	region1inRegion2	39525
CNVR73	934	7	22207228	22236111	gain	3160	20633784	45102432	Ham_weight	region1inRegion2	28883
CNVR74	938	7	23307667	23658110	loss-gain	3160	20633784	45102432	Ham_weight	region1inRegion2	350443
CNVR75	939	7	23481211	23658110	loss-gain	3160	20633784	45102432	Ham_weight	region1inRegion2	176899
CNVR76	942	7	24648309	24653067	loss-gain	3160	20633784	45102432	Ham_weight	region1inRegion2	4758
CNVR77	943	7	24735665	24740397	gain	3160	20633784	45102432	Ham_weight	region1inRegion2	4732
CNVR78	944	7	25359190	25399904	gain	3160	20633784	45102432	Ham_weight	region1inRegion2	40714
CNVR79	945	7	25488796	25557156	gain	3160	20633784	45102432	Ham_weight	region1inRegion2	68360
CNVR80	946	7	25785853	25888939	gain	3160	20633784	45102432	Ham_weight	region1inRegion2	103086
CNVR81	949	7	26267062	26297673	gain	3160	20633784	45102432	Ham_weight	region1inRegion2	30611
CNVR82	951	7	26600964	26608495	gain	3160	20633784	45102432	Ham_weight	region1inRegion2	7531
CNVR83	957	7	28525787	28565312	gain	3160	20633784	45102432	Ham_weight	region1inRegion2	39525
CNVR73	934	7	22207228	22236111	gain	3161	20633784	45102432	Shoulder_weight	region1inRegion2	28883
CNVR74	938	7	23307667	23658110	loss-gain	3161	20633784	45102432	Shoulder_weight	region1inRegion2	350443
CNVR75	939	7	23481211	23658110	loss-gain	3161	20633784	45102432	Shoulder_weight	region1inRegion2	176899
CNVR76	942	7	24648309	24653067	loss-gain	3161	20633784	45102432	Shoulder_weight	region1inRegion2	4758
CNVR77	943	7	24735665	24740397	gain	3161	20633784	45102432	Shoulder_weight	region1inRegion2	4732
CNVR78	944	7	25359190	25399904	gain	3161	20633784	45102432	Shoulder_weight	region1inRegion2	40714
CNVR79	945	7	25488796	25557156	gain	3161	20633784	45102432	Shoulder_weight	region1inRegion2	68360
CNVR80	946	7	25785853	25888939	gain	3161	20633784	45102432	Shoulder_weight	region1inRegion2	103086
CNVR81	949	7	26267062	26297673	gain	3161	20633784	45102432	Shoulder_weight	region1inRegion2	30611
CNVR82	951	7	26600964	26608495	gain	3161	20633784	45102432	Shoulder_weight	region1inRegion2	7531
CNVR83	957	7	28525787	28565312	gain	3161	20633784	45102432	Shoulder_weight	region1inRegion2	39525
CNVR73	934	7	22207228	22236111	gain	3162	20633784	45102432	Backfat_weight	region1inRegion2	28883
CNVR74	938	7	23307667	23658110	loss-gain	3162	20633784	45102432	Backfat_weight	region1inRegion2	350443
CNVR75	939	7	23481211	23658110	loss-gain	3162	20633784	45102432	Backfat_weight	region1inRegion2	176899
CNVR76	942	7	24648309	24653067	loss-gain	3162	20633784	45102432	Backfat_weight	region1inRegion2	4758
CNVR77	943	7	24735665	24740397	gain	3162	20633784	45102432	Backfat_weight	region1inRegion2	4732
CNVR78	944	7	25359190	25399904	gain	3162	20633784	45102432	Backfat_weight	region1inRegion2	40714
CNVR79	945	7	25488796	25557156	gain	3162	20633784	45102432	Backfat_weight	region1inRegion2	68360
CNVR80	946	7	25785853	25888939	gain	3162	20633784	45102432	Backfat_weight	region1inRegion2	103086
CNVR81	949	7	26267062	26297673	gain	3162	20633784	45102432	Backfat_weight	region1inRegion2	30611
CNVR82	951	7	26600964	26608495	gain	3162	20633784	45102432	Backfat_weight	region1inRegion2	7531
CNVR83	957	7	28525787	28565312	gain	3162	20633784	45102432	Backfat_weight	region1inRegion2	39525
CNVR73	934	7	22207228	22236111	gain	3163	20633784	45102432	Feet_weight	region1inRegion2	28883
CNVR74	938	7	23307667	23658110	loss-gain	3163	20633784	45102432	Feet_weight	region1inRegion2	350443
CNVR75	939	7	23481211	23658110	loss-gain	3163	20633784	45102432	Feet_weight	region1inRegion2	176899
CNVR76	942	7	24648309	24653067	loss-gain	3163	20633784	45102432	Feet_weight	region1inRegion2	4758
CNVR77	943	7	24735665	24740397	gain	3163	20633784	45102432	Feet_weight	region1inRegion2	4732
CNVR78	944	7	25359190	25399904	gain	3163	20633784	45102432	Feet_weight	region1inRegion2	40714
CNVR79	945	7	25488796	25557156	gain	3163	20633784	45102432	Feet_weight	region1inRegion2	68360
CNVR80	946	7	25785853	25888939	gain	3163	20633784	45102432	Feet_weight	region1inRegion2	103086
CNVR81	949	7	26267062	26297673	gain	3163	20633784	45102432	Feet_weight	region1inRegion2	30611
CNVR82	951	7	26600964	26608495	gain	3163	20633784	45102432	Feet_weight	region1inRegion2	7531
CNVR83	957	7	28525787	28565312	gain	3163	20633784	45102432	Feet_weight	region1inRegion2	39525
CNVR73	934	7	22207228	22236111	gain	3164	20633784	45102432	Belly_weight	region1inRegion2	28883
CNVR74	938	7	23307667	23658110	loss-gain	3164	20633784	45102432	Belly_weight	region1inRegion2	350443
CNVR75	939	7	23481211	23658110	loss-gain	3164	20633784	45102432	Belly_weight	region1inRegion2	176899
CNVR76	942	7	24648309	24653067	loss-gain	3164	20633784	45102432	Belly_weight	region1inRegion2	4758
CNVR77	943	7	24735665	24740397	gain	3164	20633784	45102432	Belly_weight	region1inRegion2	4732
CNVR78	944	7	25359190	25399904	gain	3164	20633784	45102432	Belly_weight	region1inRegion2	40714
CNVR79	945	7	25488796	25557156	gain	3164	20633784	45102432	Belly_weight	region1inRegion2	68360
CNVR80	946	7	25785853	25888939	gain	3164	20633784	45102432	Belly_weight	region1inRegion2	103086
CNVR81	949	7	26267062	26297673	gain	3164	20633784	45102432	Belly_weight	region1inRegion2	30611
CNVR82	951	7	26600964	26608495	gain	3164	20633784	45102432	Belly_weight	region1inRegion2	7531
CNVR83	957	7	28525787	28565312	gain	3164	20633784	45102432	Belly_weight	region1inRegion2	39525
CNVR73	934	7	22207228	22236111	gain	3165	20633784	45102432	Backfat_at_last_lumbar	region1inRegion2	28883
CNVR74	938	7	23307667	23658110	loss-gain	3165	20633784	45102432	Backfat_at_last_lumbar	region1inRegion2	350443
CNVR75	939	7	23481211	23658110	loss-gain	3165	20633784	45102432	Backfat_at_last_lumbar	region1inRegion2	176899
CNVR76	942	7	24648309	24653067	loss-gain	3165	20633784	45102432	Backfat_at_last_lumbar	region1inRegion2	4758
CNVR77	943	7	24735665	24740397	gain	3165	20633784	45102432	Backfat_at_last_lumbar	region1inRegion2	4732
CNVR78	944	7	25359190	25399904	gain	3165	20633784	45102432	Backfat_at_last_lumbar	region1inRegion2	40714
CNVR79	945	7	25488796	25557156	gain	3165	20633784	45102432	Backfat_at_last_lumbar	region1inRegion2	68360
CNVR80	946	7	25785853	25888939	gain	3165	20633784	45102432	Backfat_at_last_lumbar	region1inRegion2	103086
CNVR81	949	7	26267062	26297673	gain	3165	20633784	45102432	Backfat_at_last_lumbar	region1inRegion2	30611
CNVR82	951	7	26600964	26608495	gain	3165	20633784	45102432	Backfat_at_last_lumbar	region1inRegion2	7531
CNVR83	957	7	28525787	28565312	gain	3165	20633784	45102432	Backfat_at_last_lumbar	region1inRegion2	39525
CNVR73	934	7	22207228	22236111	gain	3166	20633784	45102432	Backfat_thickness_between_3rd_and_4th_rib	region1inRegion2	28883
CNVR74	938	7	23307667	23658110	loss-gain	3166	20633784	45102432	Backfat_thickness_between_3rd_and_4th_rib	region1inRegion2	350443
CNVR75	939	7	23481211	23658110	loss-gain	3166	20633784	45102432	Backfat_thickness_between_3rd_and_4th_rib	region1inRegion2	176899
CNVR76	942	7	24648309	24653067	loss-gain	3166	20633784	45102432	Backfat_thickness_between_3rd_and_4th_rib	region1inRegion2	4758
CNVR77	943	7	24735665	24740397	gain	3166	20633784	45102432	Backfat_thickness_between_3rd_and_4th_rib	region1inRegion2	4732
CNVR78	944	7	25359190	25399904	gain	3166	20633784	45102432	Backfat_thickness_between_3rd_and_4th_rib	region1inRegion2	40714
CNVR79	945	7	25488796	25557156	gain	3166	20633784	45102432	Backfat_thickness_between_3rd_and_4th_rib	region1inRegion2	68360
CNVR80	946	7	25785853	25888939	gain	3166	20633784	45102432	Backfat_thickness_between_3rd_and_4th_rib	region1inRegion2	103086
CNVR81	949	7	26267062	26297673	gain	3166	20633784	45102432	Backfat_thickness_between_3rd_and_4th_rib	region1inRegion2	30611
CNVR82	951	7	26600964	26608495	gain	3166	20633784	45102432	Backfat_thickness_between_3rd_and_4th_rib	region1inRegion2	7531

CNVR83	957	7	28525787	28565312	gain	3166	20633784	45102432	Backfat_thickness_between_3rd_and_4th_rib	region1inRegion2	39525
CNVR73	934	7	22207228	22236111	gain	3167	20633784	45102432	Lean_meat_percentage	region1inRegion2	28883
CNVR74	938	7	23307667	23658110	loss-gain	3167	20633784	45102432	Lean_meat_percentage	region1inRegion2	350443
CNVR75	939	7	23481211	23658110	loss-gain	3167	20633784	45102432	Lean_meat_percentage	region1inRegion2	176899
CNVR76	942	7	24648309	24653067	loss-gain	3167	20633784	45102432	Lean_meat_percentage	region1inRegion2	4758
CNVR77	943	7	24735665	24740397	gain	3167	20633784	45102432	Lean_meat_percentage	region1inRegion2	4732
CNVR78	944	7	25359190	25399904	gain	3167	20633784	45102432	Lean_meat_percentage	region1inRegion2	40714
CNVR79	945	7	25488796	25557156	gain	3167	20633784	45102432	Lean_meat_percentage	region1inRegion2	68360
CNVR80	946	7	25785853	25888939	gain	3167	20633784	45102432	Lean_meat_percentage	region1inRegion2	103086
CNVR81	949	7	26267062	26297673	gain	3167	20633784	45102432	Lean_meat_percentage	region1inRegion2	30611
CNVR82	951	7	26600964	26608495	gain	3167	20633784	45102432	Lean_meat_percentage	region1inRegion2	7531
CNVR83	957	7	28525787	28565312	gain	3167	20633784	45102432	Lean_meat_percentage	region1inRegion2	39525
CNVR73	934	7	22207228	22236111	gain	601	20633784	45102432	Fat_androstenone_level	region1inRegion2	28883
CNVR74	938	7	23307667	23658110	loss-gain	601	20633784	45102432	Fat_androstenone_level	region1inRegion2	350443
CNVR75	939	7	23481211	23658110	loss-gain	601	20633784	45102432	Fat_androstenone_level	region1inRegion2	176899
CNVR76	942	7	24648309	24653067	loss-gain	601	20633784	45102432	Fat_androstenone_level	region1inRegion2	4758
CNVR77	943	7	24735665	24740397	gain	601	20633784	45102432	Fat_androstenone_level	region1inRegion2	4732
CNVR78	944	7	25359190	25399904	gain	601	20633784	45102432	Fat_androstenone_level	region1inRegion2	40714
CNVR79	945	7	25488796	25557156	gain	601	20633784	45102432	Fat_androstenone_level	region1inRegion2	68360
CNVR80	946	7	25785853	25888939	gain	601	20633784	45102432	Fat_androstenone_level	region1inRegion2	103086
CNVR81	949	7	26267062	26297673	gain	601	20633784	45102432	Fat_androstenone_level	region1inRegion2	30611
CNVR82	951	7	26600964	26608495	gain	601	20633784	45102432	Fat_androstenone_level	region1inRegion2	7531
CNVR83	957	7	28525787	28565312	gain	601	20633784	45102432	Fat_androstenone_level	region1inRegion2	39525
CNVR73	934	7	22207228	22236111	gain	602	20633784	45102432	Fat_androstenone_level	region1inRegion2	28883
CNVR74	938	7	23307667	23658110	loss-gain	602	20633784	45102432	Fat_androstenone_level	region1inRegion2	350443
CNVR75	939	7	23481211	23658110	loss-gain	602	20633784	45102432	Fat_androstenone_level	region1inRegion2	176899
CNVR76	942	7	24648309	24653067	loss-gain	602	20633784	45102432	Fat_androstenone_level	region1inRegion2	4758
CNVR77	943	7	24735665	24740397	gain	602	20633784	45102432	Fat_androstenone_level	region1inRegion2	4732
CNVR78	944	7	25359190	25399904	gain	602	20633784	45102432	Fat_androstenone_level	region1inRegion2	40714
CNVR79	945	7	25488796	25557156	gain	602	20633784	45102432	Fat_androstenone_level	region1inRegion2	68360
CNVR80	946	7	25785853	25888939	gain	602	20633784	45102432	Fat_androstenone_level	region1inRegion2	103086
CNVR81	949	7	26267062	26297673	gain	602	20633784	45102432	Fat_androstenone_level	region1inRegion2	30611
CNVR82	951	7	26600964	26608495	gain	602	20633784	45102432	Fat_androstenone_level	region1inRegion2	7531
CNVR83	957	7	28525787	28565312	gain	602	20633784	45102432	Fat_androstenone_level	region1inRegion2	39525
CNVR73	934	7	22207228	22236111	gain	603	20633784	45102432	Fat_androstenone_level	region1inRegion2	28883
CNVR74	938	7	23307667	23658110	loss-gain	603	20633784	45102432	Fat_androstenone_level	region1inRegion2	350443
CNVR75	939	7	23481211	23658110	loss-gain	603	20633784	45102432	Fat_androstenone_level	region1inRegion2	176899
CNVR76	942	7	24648309	24653067	loss-gain	603	20633784	45102432	Fat_androstenone_level	region1inRegion2	4758
CNVR77	943	7	24735665	24740397	gain	603	20633784	45102432	Fat_androstenone_level	region1inRegion2	4732
CNVR78	944	7	25359190	25399904	gain	603	20633784	45102432	Fat_androstenone_level	region1inRegion2	40714
CNVR79	945	7	25488796	25557156	gain	603	20633784	45102432	Fat_androstenone_level	region1inRegion2	68360
CNVR80	946	7	25785853	25888939	gain	603	20633784	45102432	Fat_androstenone_level	region1inRegion2	103086
CNVR81	949	7	26267062	26297673	gain	603	20633784	45102432	Fat_androstenone_level	region1inRegion2	30611
CNVR82	951	7	26600964	26608495	gain	603	20633784	45102432	Fat_androstenone_level	region1inRegion2	7531
CNVR83	957	7	28525787	28565312	gain	603	20633784	45102432	Fat_androstenone_level	region1inRegion2	39525
CNVR73	934	7	22207228	22236111	gain	604	20633784	45102432	Fat_androstenone_level	region1inRegion2	28883
CNVR74	938	7	23307667	23658110	loss-gain	604	20633784	45102432	Fat_androstenone_level	region1inRegion2	350443
CNVR75	939	7	23481211	23658110	loss-gain	604	20633784	45102432	Fat_androstenone_level	region1inRegion2	176899
CNVR76	942	7	24648309	24653067	loss-gain	604	20633784	45102432	Fat_androstenone_level	region1inRegion2	4758
CNVR77	943	7	24735665	24740397	gain	604	20633784	45102432	Fat_androstenone_level	region1inRegion2	4732
CNVR78	944	7	25359190	25399904	gain	604	20633784	45102432	Fat_androstenone_level	region1inRegion2	40714
CNVR79	945	7	25488796	25557156	gain	604	20633784	45102432	Fat_androstenone_level	region1inRegion2	68360
CNVR80	946	7	25785853	25888939	gain	604	20633784	45102432	Fat_androstenone_level	region1inRegion2	103086
CNVR81	949	7	26267062	26297673	gain	604	20633784	45102432	Fat_androstenone_level	region1inRegion2	30611
CNVR82	951	7	26600964	26608495	gain	604	20633784	45102432	Fat_androstenone_level	region1inRegion2	7531
CNVR83	957	7	28525787	28565312	gain	604	20633784	45102432	Fat_androstenone_level	region1inRegion2	39525
CNVR73	934	7	22207228	22236111	gain	605	20633784	45102432	Fat_androstenone_level	region1inRegion2	28883
CNVR74	938	7	23307667	23658110	loss-gain	605	20633784	45102432	Fat_androstenone_level	region1inRegion2	350443
CNVR75	939	7	23481211	23658110	loss-gain	605	20633784	45102432	Fat_androstenone_level	region1inRegion2	176899
CNVR76	942	7	24648309	24653067	loss-gain	605	20633784	45102432	Fat_androstenone_level	region1inRegion2	4758
CNVR77	943	7	24735665	24740397	gain	605	20633784	45102432	Fat_androstenone_level	region1inRegion2	4732
CNVR78	944	7	25359190	25399904	gain	605	20633784	45102432	Fat_androstenone_level	region1inRegion2	40714
CNVR79	945	7	25488796	25557156	gain	605	20633784	45102432	Fat_androstenone_level	region1inRegion2	68360
CNVR80	946	7	25785853	25888939	gain	605	20633784	45102432	Fat_androstenone_level	region1inRegion2	103086
CNVR81	949	7	26267062	26297673	gain	605	20633784	45102432	Fat_androstenone_level	region1inRegion2	30611
CNVR82	951	7	26600964	26608495	gain	605	20633784	45102432	Fat_androstenone_level	region1inRegion2	7531
CNVR83	957	7	28525787	28565312	gain	605	20633784	45102432	Fat_androstenone_level	region1inRegion2	39525
CNVR73	934	7	22207228	22236111	gain	3254	20633784	50310118	Carcass_length	region1inRegion2	28883
CNVR74	938	7	23307667	23658110	loss-gain	3254	20633784	50310118	Carcass_length	region1inRegion2	350443
CNVR75	939	7	23481211	23658110	loss-gain	3254	20633784	50310118	Carcass_length	region1inRegion2	176899
CNVR76	942	7	24648309	24653067	loss-gain	3254	20633784	50310118	Carcass_length	region1inRegion2	4758
CNVR77	943	7	24735665	24740397	gain	3254	20633784	50310118	Carcass_length	region1inRegion2	4732
CNVR78	944	7	25359190	25399904	gain	3254	20633784	50310118	Carcass_length	region1inRegion2	40714
CNVR79	945	7	25488796	25557156	gain	3254	20633784	50310118	Carcass_length	region1inRegion2	68360
CNVR80	946	7	25785853	25888939	gain	3254	20633784	50310118	Carcass_length	region1inRegion2	103086
CNVR81	949	7	26267062	26297673	gain	3254	20633784	50310118	Carcass_length	region1inRegion2	30611
CNVR82	951	7	26600964	26608495	gain	3254	20633784	50310118	Carcass_length	region1inRegion2	7531
CNVR83	957	7	28525787	28565312	gain	3254	20633784	50310118	Carcass_length	region1inRegion2	39525
CNVR73	934	7	22207228	22236111	gain	3255	20633784	50310118	Dressing_percentage	region1inRegion2	28883
CNVR74	938	7	23307667	23658110	loss-gain	3255	20633784	50310118	Dressing_percentage	region1inRegion2	350443
CNVR75	939	7	23481211	23658110	loss-gain	3255	20633784	50310118	Dressing_percentage	region1inRegion2	176899
CNVR76	942	7	24648309	24653067	loss-gain	3255	20633784	50310118	Dressing_percentage	region1inRegion2	4758
CNVR77	943	7	24735665	24740397	gain	3255	20633784	50310118	Dressing_percentage	region1inRegion2	4732
CNVR78	944	7	25359190	25399904	gain	3255	20633784	50310118	Dressing_percentage	region1inRegion2	40714
CNVR79	945	7	25488796	25557156	gain	3255	20633784	50310118	Dressing_percentage	region1inRegion2	68360
CNVR80	946	7	25785853	25888939	gain	3255	20633784	50310118	Dressing_percentage	region1inRegion2	103086
CNVR81	949	7	26267062	26297673	gain	3255	20633784	50310118	Dressing_percentage	region1inRegion2	30611
CNVR82	951	7	26600964	26608495	gain	3255	20633784	50310118	Dressing_percentage	region1inRegion2	7531
CNVR83	957	7	28525787	28565312	gain	3255	20633784	50310118	Dressing_percentage	region1inRegion2	39525
CNVR73	934	7	22207228	22236111	gain	36302	21220389	109122882	Percentage_type_I_fibers	region1inRegion2	28883
CNVR74	938	7	23307667	23658110	loss-gain	36302	21220389	109122882	Percentage_type_I_fibers	region1inRegion2	350443
CNVR75	939	7	23481211	23658110	loss-gain	36302	21220389	109122882	Percentage_type_I_fibers	region1inRegion2	176899
CNVR76	942	7	24648309	24653067	loss-gain	36302	21220389	109122882	Percentage_type_I_fibers	region1inRegion2	4758
CNVR77	943	7	24735665	24740397	gain	36302	21220389	109122882	Percentage_type_I_fibers	region1inRegion2	4732
CNVR78	944	7	25359190	25399904	gain	36302	21220389	109122882	Percentage_type_I_fibers	region1inRegion2	40714
CNVR79	945	7	25488796	25557156	gain	36302	21220389	109122882	Percentage_type_I_fibers	region1inRegion2	68360
CNVR80	946	7	25785853	25888939	gain	36302	21220389	109122882	Percentage_type_I_fibers	region1inRegion2	103086
CNVR81	949	7	26267062	26297673	gain	36302	21220389	109122882	Percentage_type_I_fibers	region1inRegion2	30611
CNVR82	951	7	26600964	26608495	gain	36302	21220389	109122882	Percentage_type_I_fibers	region1inRegion2	7531
CNVR83	957	7	28525787	28565312	gain	36302	21220389	109122882	Percentage_type_I_fibers	region1inRegion2	39525
CNVR84	972	7	58579811	58584542	gain	36302	21220389	109122882	Percentage_type_I_fibers	region1inRegion2	4731
CNVR85	975	7	59278633	59284219	loss	36302	21220389	109122882	Percentage_type_I_fibers	region1inRegion2	5586
CNVR86	977	7	61355441	61417944	gain	36302	21220389	109122882	Percentage_type_I_fibers	region1inRegion2	62503
CNVR87	994	7	82234623	82374446	gain	36302	21220389	109122882	Percentage_type_I_fibers	region1inRegion2	139823
CNVR88	1002	7	84728780	84749202	gain	36302	21220389	109122882	Percentage_type_I_fibers	region1inRegion2	20422
CNVR89	1011	7	102790067	102823288	gain	36302	21220389	109122882	Percentage_type_I_fibers	region1inRegion2	33221
CNVR73	934	7	22207228	22236111	gain	36303	21220389	109122882	Percentage_type_IIb_fibers	region1inRegion2	28883
CNVR74	938	7	23307667	23658110	loss-gain	36303	21220389	109122882	Percentage_type_IIb_fibers	region1inRegion2	350443
CNVR75	939	7	23481211	23658110	loss-gain	36303	21220389	109122882	Percentage_type_IIb_fibers	region1inRegion2	176899
CNVR76	942	7	24648309	24653067	loss-gain	36303	21220389	109122882	Percentage_type_IIb_fibers	region1inRegion2	4758
CNVR77	943	7	24735665	24740397	gain	36303	21220389	109122882	Percentage_type_IIb_fibers	region1inRegion2	4732
CNVR78	944	7	25359190	25399904	gain	36303	21220389	109122882	Percentage_type_IIb_fibers	region1inRegion2	40714
CNVR79	945	7	25488796	25557156	gain	36303	21220389	109122882	Percentage_type_IIb_fibers	region1inRegion2	68360
CNVR80	946	7	25785853	25888939	gain	36303	21220389	109122882	Percentage_type_IIb_fibers	region1inRegion2	103086
CNVR81	949	7	26267062	26297673	gain	36303	21220389	109122882	Percentage_type_IIb_fibers	region1inRegion2	30611
CNVR82	951	7	26600964	26608495	gain	36303	21220389	109122882	Percentage_type_IIb_fibers	region1inRegion2	7531
CNVR83	957	7	28525787	28565312	gain	36303	21220389	109122882	Percentage_type_IIb_fibers	region1inRegion2	39525
CNVR84	972	7	58579811	58584542	gain	36303	21220389	109122882	Percentage_type_IIb_fibers	region1inRegion2	4731
CNVR85	975	7	59278633	59284219	loss	36303	21220389	109122882	Percentage_type_IIb_fibers	region1inRegion2	5586
CNVR86	977	7	61355441	61417944	gain	36303	21220389	109122882	Percentage_type_IIb_fibers	region1inRegion2	62503
CNVR87	994	7	82234623	82374446	gain	36303	21220389	109122882	Percentage_type_IIb_fibers	region1inRegion2	139823
CNVR88	1002	7	84728780	84749202	gain	36303	21220389	109122882	Percentage_type_IIb_fibers	region1inRegion2	20422

CNVR89	1011	7	102790067	102823288	gain	36303	21220389	109122882	Percentage_type_IIb_fibers	region1inRegion2	33221
CNVR73	934	7	22207228	22236111	gain	36304	21220389	109122882	Drip_loss	region1inRegion2	28883
CNVR74	938	7	23307667	23658110	loss-gain	36304	21220389	109122882	Drip_loss	region1inRegion2	350443
CNVR75	939	7	23481211	23658110	loss-gain	36304	21220389	109122882	Drip_loss	region1inRegion2	176899
CNVR76	942	7	24648309	24653067	loss-gain	36304	21220389	109122882	Drip_loss	region1inRegion2	4758
CNVR77	943	7	24735665	24740397	gain	36304	21220389	109122882	Drip_loss	region1inRegion2	4732
CNVR78	944	7	25359190	25399904	gain	36304	21220389	109122882	Drip_loss	region1inRegion2	40714
CNVR79	945	7	25488796	25557156	gain	36304	21220389	109122882	Drip_loss	region1inRegion2	68360
CNVR80	946	7	25785853	25888939	gain	36304	21220389	109122882	Drip_loss	region1inRegion2	103086
CNVR81	949	7	26267062	26297673	gain	36304	21220389	109122882	Drip_loss	region1inRegion2	30611
CNVR82	951	7	26600964	26608495	gain	36304	21220389	109122882	Drip_loss	region1inRegion2	7531
CNVR83	957	7	28525787	28565312	gain	36304	21220389	109122882	Drip_loss	region1inRegion2	39525
CNVR84	972	7	58579811	58584542	gain	36304	21220389	109122882	Drip_loss	region1inRegion2	4731
CNVR85	975	7	59278633	59284219	loss	36304	21220389	109122882	Drip_loss	region1inRegion2	5586
CNVR86	977	7	61355441	61417944	gain	36304	21220389	109122882	Drip_loss	region1inRegion2	62503
CNVR87	994	7	82234623	82374446	gain	36304	21220389	109122882	Drip_loss	region1inRegion2	139823
CNVR88	1002	7	84728780	84749202	gain	36304	21220389	109122882	Drip_loss	region1inRegion2	20422
CNVR89	1011	7	102790067	102823288	gain	36304	21220389	109122882	Drip_loss	region1inRegion2	33221
CNVR73	934	7	22207228	22236111	gain	5816	21815631	22846794	Loin_muscle_area	region1inRegion2	28883
CNVR73	934	7	22207228	22236111	gain	7503	21815631	22846794	Parasite_load	region1inRegion2	28883
CNVR73	934	7	22207228	22236111	gain	803	21984648	45019880	Average_backfat_thickness	region1inRegion2	28883
CNVR74	938	7	23307667	23658110	loss-gain	803	21984648	45019880	Average_backfat_thickness	region1inRegion2	350443
CNVR75	939	7	23481211	23658110	loss-gain	803	21984648	45019880	Average_backfat_thickness	region1inRegion2	176899
CNVR76	942	7	24648309	24653067	loss-gain	803	21984648	45019880	Average_backfat_thickness	region1inRegion2	4758
CNVR77	943	7	24735665	24740397	gain	803	21984648	45019880	Average_backfat_thickness	region1inRegion2	4732
CNVR78	944	7	25359190	25399904	gain	803	21984648	45019880	Average_backfat_thickness	region1inRegion2	40714
CNVR79	945	7	25488796	25557156	gain	803	21984648	45019880	Average_backfat_thickness	region1inRegion2	68360
CNVR80	946	7	25785853	25888939	gain	803	21984648	45019880	Average_backfat_thickness	region1inRegion2	103086
CNVR81	949	7	26267062	26297673	gain	803	21984648	45019880	Average_backfat_thickness	region1inRegion2	30611
CNVR82	951	7	26600964	26608495	gain	803	21984648	45019880	Average_backfat_thickness	region1inRegion2	7531
CNVR83	957	7	28525787	28565312	gain	803	21984648	45019880	Average_backfat_thickness	region1inRegion2	39525
CNVR74	938	7	23307667	23658110	loss-gain	12261	22846794	24153455	Average_daily_gain	region1inRegion2	350443
CNVR75	939	7	23481211	23658110	loss-gain	12261	22846794	24153455	Average_daily_gain	region1inRegion2	176899
CNVR74	938	7	23307667	23658110	loss-gain	12262	22846794	24153455	Average_daily_gain	region1inRegion2	350443
CNVR75	939	7	23481211	23658110	loss-gain	12262	22846794	24153455	Average_daily_gain	region1inRegion2	176899
CNVR74	938	7	23307667	23658110	loss-gain	12263	22846794	24153455	Days_to_100_kg	region1inRegion2	350443
CNVR75	939	7	23481211	23658110	loss-gain	12263	22846794	24153455	Days_to_100_kg	region1inRegion2	176899
CNVR74	938	7	23307667	23658110	loss-gain	554	22871560	24532135	Body_weight_(26_weeks)	region1inRegion2	350443
CNVR75	939	7	23481211	23658110	loss-gain	554	22871560	24532135	Body_weight_(26_weeks)	region1inRegion2	176899
CNVR74	938	7	23307667	23658110	loss-gain	31803	23297612	24178503	PRRSV_susceptibility	region1inRegion2	350443
CNVR75	939	7	23481211	23658110	loss-gain	31803	23297612	24178503	PRRSV_susceptibility	region1inRegion2	176899
CNVR74	938	7	23307667	23658110	loss-gain	27765	23360672	23360712	Mean_corpuscular_volume	region2inRegion1	40
CNVR74	938	7	23307667	23658110	loss-gain	27766	23381438	23381478	Mean_corpuscular_volume	region2inRegion1	40
CNVR74	938	7	23307667	23658110	loss-gain	22485	23406029	23406069	Oleic_acid_content	region2inRegion1	40
CNVR74	938	7	23307667	23658110	loss-gain	27767	23406029	23406069	Mean_corpuscular_volume	region2inRegion1	40
CNVR74	938	7	23307667	23658110	loss-gain	27773	23444422	23444462	Mean_corpuscular_volume	region2inRegion1	40
CNVR74	938	7	23307667	23658110	loss-gain	27772	23460164	23460204	Mean_corpuscular_volume	region2inRegion1	40
CNVR74	938	7	23307667	23658110	loss-gain	27771	23481670	23481710	Mean_corpuscular_volume	region2inRegion1	40
CNVR75	939	7	23481211	23658110	loss-gain	27771	23481670	23481710	Mean_corpuscular_volume	region2inRegion1	40
CNVR74	938	7	23307667	23658110	loss-gain	95367	23481690	27310755	cis-11-Eicosenoic_acid_content	overlapTail	176421
CNVR75	939	7	23481211	23658110	loss-gain	95367	23481690	27310755	cis-11-Eicosenoic_acid_content	overlapTail	176421
CNVR76	942	7	24648309	24653067	loss-gain	95367	23481690	27310755	cis-11-Eicosenoic_acid_content	region1inRegion2	4758
CNVR77	943	7	24735665	24740397	gain	95367	23481690	27310755	cis-11-Eicosenoic_acid_content	region1inRegion2	4732
CNVR78	944	7	25359190	25399904	gain	95367	23481690	27310755	cis-11-Eicosenoic_acid_content	region1inRegion2	40714
CNVR79	945	7	25488796	25557156	gain	95367	23481690	27310755	cis-11-Eicosenoic_acid_content	region1inRegion2	68360
CNVR80	946	7	25785853	25888939	gain	95367	23481690	27310755	cis-11-Eicosenoic_acid_content	region1inRegion2	103086
CNVR81	949	7	26267062	26297673	gain	95367	23481690	27310755	cis-11-Eicosenoic_acid_content	region1inRegion2	30611
CNVR82	951	7	26600964	26608495	gain	95367	23481690	27310755	cis-11-Eicosenoic_acid_content	region1inRegion2	7531
CNVR74	938	7	23307667	23658110	loss-gain	27770	23504058	23504098	Mean_corpuscular_volume	region2inRegion1	40
CNVR75	939	7	23481211	23658110	loss-gain	27770	23504058	23504098	Mean_corpuscular_volume	region2inRegion1	40
CNVR74	938	7	23307667	23658110	loss-gain	27769	23515797	23515837	Mean_corpuscular_volume	region2inRegion1	40
CNVR75	939	7	23481211	23658110	loss-gain	27769	23515797	23515837	Mean_corpuscular_volume	region2inRegion1	40
CNVR74	938	7	23307667	23658110	loss-gain	12039	23520386	34555848	Small_intestine_length	overlapTail	137725
CNVR75	939	7	23481211	23658110	loss-gain	12039	23520386	34555848	Small_intestine_length	overlapTail	137725
CNVR76	942	7	24648309	24653067	loss-gain	12039	23520386	34555848	Small_intestine_length	region1inRegion2	4758
CNVR77	943	7	24735665	24740397	gain	12039	23520386	34555848	Small_intestine_length	region1inRegion2	4732
CNVR78	944	7	25359190	25399904	gain	12039	23520386	34555848	Small_intestine_length	region1inRegion2	40714
CNVR79	945	7	25488796	25557156	gain	12039	23520386	34555848	Small_intestine_length	region1inRegion2	68360
CNVR80	946	7	25785853	25888939	gain	12039	23520386	34555848	Small_intestine_length	region1inRegion2	103086
CNVR81	949	7	26267062	26297673	gain	12039	23520386	34555848	Small_intestine_length	region1inRegion2	30611
CNVR82	951	7	26600964	26608495	gain	12039	23520386	34555848	Small_intestine_length	region1inRegion2	7531
CNVR83	957	7	28525787	28565312	gain	12039	23520386	34555848	Small_intestine_length	region1inRegion2	39525
CNVR74	938	7	23307667	23658110	loss-gain	9007	23520386	34555848	Cervical_vertebra_length	overlapTail	137725
CNVR75	939	7	23481211	23658110	loss-gain	9007	23520386	34555848	Cervical_vertebra_length	overlapTail	137725
CNVR76	942	7	24648309	24653067	loss-gain	9007	23520386	34555848	Cervical_vertebra_length	region1inRegion2	4758
CNVR77	943	7	24735665	24740397	gain	9007	23520386	34555848	Cervical_vertebra_length	region1inRegion2	4732
CNVR78	944	7	25359190	25399904	gain	9007	23520386	34555848	Cervical_vertebra_length	region1inRegion2	40714
CNVR79	945	7	25488796	25557156	gain	9007	23520386	34555848	Cervical_vertebra_length	region1inRegion2	68360
CNVR80	946	7	25785853	25888939	gain	9007	23520386	34555848	Cervical_vertebra_length	region1inRegion2	103086
CNVR81	949	7	26267062	26297673	gain	9007	23520386	34555848	Cervical_vertebra_length	region1inRegion2	30611
CNVR82	951	7	26600964	26608495	gain	9007	23520386	34555848	Cervical_vertebra_length	region1inRegion2	7531
CNVR83	957	7	28525787	28565312	gain	9007	23520386	34555848	Cervical_vertebra_length	region1inRegion2	39525
CNVR74	938	7	23307667	23658110	loss-gain	9067	23520386	34555848	Meat_color_score	overlapTail	137725
CNVR75	939	7	23481211	23658110	loss-gain	9067	23520386	34555848	Meat_color_score	overlapTail	137725
CNVR76	942	7	24648309	24653067	loss-gain	9067	23520386	34555848	Meat_color_score	region1inRegion2	4758
CNVR77	943	7	24735665	24740397	gain	9067	23520386	34555848	Meat_color_score	region1inRegion2	4732
CNVR78	944	7	25359190	25399904	gain	9067	23520386	34555848	Meat_color_score	region1inRegion2	40714
CNVR79	945	7	25488796	25557156	gain	9067	23520386	34555848	Meat_color_score	region1inRegion2	68360
CNVR80	946	7	25785853	25888939	gain	9067	23520386	34555848	Meat_color_score	region1inRegion2	103086
CNVR81	949	7	26267062	26297673	gain	9067	23520386	34555848	Meat_color_score	region1inRegion2	30611
CNVR82	951	7	26600964	26608495	gain	9067	23520386	34555848	Meat_color_score	region1inRegion2	7531
CNVR83	957	7	28525787	28565312	gain	9067	23520386	34555848	Meat_color_score	region1inRegion2	39525
CNVR74	938	7	23307667	23658110	loss-gain	9069	23520386	34555848	CIE-L*	overlapTail	137725
CNVR75	939	7	23481211	23658110	loss-gain	9069	23520386	34555848	CIE-L*	overlapTail	137725
CNVR76	942	7	24648309	24653067	loss-gain	9069	23520386	34555848	CIE-L*	region1inRegion2	4758
CNVR77	943	7	24735665	24740397	gain	9069	23520386	34555848	CIE-L*	region1inRegion2	4732
CNVR78	944	7	25359190	25399904	gain	9069	23520386	34555848	CIE-L*	region1inRegion2	40714
CNVR79	945	7	25488796	25557156	gain	9069	23520386	34555848	CIE-L*	region1inRegion2	68360
CNVR80	946	7	25785853	25888939	gain	9069	23520386	34555848	CIE-L*	region1inRegion2	103086
CNVR81	949	7	26267062	26297673	gain	9069	23520386	34555848	CIE-L*	region1inRegion2	30611
CNVR82	951	7	26600964	26608495	gain	9069	23520386	34555848	CIE-L*	region1inRegion2	7531
CNVR83	957	7	28525787	28565312	gain	9069	23520386	34555848	CIE-L*	region1inRegion2	39525
CNVR74	938	7	23307667	23658110	loss-gain	9081	23520386	34555848	CIE-a*	overlapTail	137725
CNVR75	939	7	23481211	23658110	loss-gain	9081	23520386	34555848	CIE-a*	overlapTail	137725
CNVR76	942	7	24648309	24653067	loss-gain	9081	23520386	34555848	CIE-a*	region1inRegion2	4758
CNVR77	943	7	24735665	24740397	gain	9081	23520386	34555848	CIE-a*	region1inRegion2	4732
CNVR78	944	7	25359190	25399904	gain	9081	23520386	34555848	CIE-a*	region1inRegion2	40714
CNVR79	945	7	25488796	25557156	gain	9081	23520386	34555848	CIE-a*	region1inRegion2	68360
CNVR80	946	7	25785853	25888939	gain	9081	23520386	34555848	CIE-a*	region1inRegion2	103086
CNVR81	949	7	26267062	26297673	gain	9081	23520386	34555848	CIE-a*	region1inRegion2	30611
CNVR82	951	7	26600964	26608495	gain	9081	23520386	34555848	CIE-a*	region1inRegion2	7531
CNVR83	957	7	28525787	28565312	gain	9081	23520386	34555848	CIE-a*	region1inRegion2	39525
CNVR74	938	7	23307667	23658110	loss-gain	9089	23520386	34555848	CIE-a*	overlapTail	137725
CNVR75	939	7	23481211	23658110	loss-gain	9089	23520386	34555848	CIE-a*	overlapTail	137725
CNVR76	942	7	24648309	24653067	loss-gain	9089	23520386	34555848	CIE-a*	region1inRegion2	4758
CNVR77	943	7	24735665	24740397	gain	9089	23520386	34555848	CIE-a*	region1inRegion2	4732
CNVR78	944	7	25359190	25399904	gain	9089	23520386	34555848	CIE-a*	region1inRegion2	40714
CNVR79	945	7	25488796	25557156	gain	9089	23520386	34555848	CIE-a*	region1inRegion2	68360
CNVR80	946	7	25785853	25888939	gain	9089	23520386	34555848	CIE-a*	region1inRegion2	103086
CNVR81	949	7	26267062	26297673	gain	9089	23520386	34555848	CIE-a*	region1inRegion2	30611
CNVR82	951	7	26600964	26608495	gain	9089	23520386	34555848	CIE-a*	region1inRegion2	7531
CNVR83	957	7	28525787	28565312	gain	9089	23520386	34555848	CIE-a*	region1inRegion2	39525

CNVR74	938	7	23307667	23658110	loss-gain	9092	23520386	34555848	CIE-a*	overlapTail	137725
CNVR75	939	7	23481211	23658110	loss-gain	9092	23520386	34555848	CIE-a*	overlapTail	137725
CNVR76	942	7	24648309	24653067	loss-gain	9092	23520386	34555848	CIE-a*	region1inRegion2	4758
CNVR77	943	7	24735665	24740397	gain	9092	23520386	34555848	CIE-a*	region1inRegion2	4732
CNVR78	944	7	25359190	25399904	gain	9092	23520386	34555848	CIE-a*	region1inRegion2	40714
CNVR79	945	7	25488796	25557156	gain	9092	23520386	34555848	CIE-a*	region1inRegion2	68360
CNVR80	946	7	25785853	25888939	gain	9092	23520386	34555848	CIE-a*	region1inRegion2	103086
CNVR81	949	7	26267062	26297673	gain	9092	23520386	34555848	CIE-a*	region1inRegion2	30611
CNVR82	951	7	26600964	26608495	gain	9092	23520386	34555848	CIE-a*	region1inRegion2	7531
CNVR83	957	7	28525787	28565312	gain	9092	23520386	34555848	CIE-a*	region1inRegion2	39525
CNVR74	938	7	23307667	23658110	loss-gain	6470	23520386	35484503	Left_teat_number	overlapTail	137725
CNVR75	939	7	23481211	23658110	loss-gain	6470	23520386	35484503	Left_teat_number	overlapTail	137725
CNVR76	942	7	24648309	24653067	loss-gain	6470	23520386	35484503	Left_teat_number	region1inRegion2	4758
CNVR77	943	7	24735665	24740397	gain	6470	23520386	35484503	Left_teat_number	region1inRegion2	4732
CNVR78	944	7	25359190	25399904	gain	6470	23520386	35484503	Left_teat_number	region1inRegion2	40714
CNVR79	945	7	25488796	25557156	gain	6470	23520386	35484503	Left_teat_number	region1inRegion2	68360
CNVR80	946	7	25785853	25888939	gain	6470	23520386	35484503	Left_teat_number	region1inRegion2	103086
CNVR81	949	7	26267062	26297673	gain	6470	23520386	35484503	Left_teat_number	region1inRegion2	30611
CNVR82	951	7	26600964	26608495	gain	6470	23520386	35484503	Left_teat_number	region1inRegion2	7531
CNVR83	957	7	28525787	28565312	gain	6470	23520386	35484503	Left_teat_number	region1inRegion2	39525
CNVR74	938	7	23307667	23658110	loss-gain	6488	23520386	35484503	Teat_number	overlapTail	137725
CNVR75	939	7	23481211	23658110	loss-gain	6488	23520386	35484503	Teat_number	overlapTail	137725
CNVR76	942	7	24648309	24653067	loss-gain	6488	23520386	35484503	Teat_number	region1inRegion2	4758
CNVR77	943	7	24735665	24740397	gain	6488	23520386	35484503	Teat_number	region1inRegion2	4732
CNVR78	944	7	25359190	25399904	gain	6488	23520386	35484503	Teat_number	region1inRegion2	40714
CNVR79	945	7	25488796	25557156	gain	6488	23520386	35484503	Teat_number	region1inRegion2	68360
CNVR80	946	7	25785853	25888939	gain	6488	23520386	35484503	Teat_number	region1inRegion2	103086
CNVR81	949	7	26267062	26297673	gain	6488	23520386	35484503	Teat_number	region1inRegion2	30611
CNVR82	951	7	26600964	26608495	gain	6488	23520386	35484503	Teat_number	region1inRegion2	7531
CNVR83	957	7	28525787	28565312	gain	6488	23520386	35484503	Teat_number	region1inRegion2	39525
CNVR74	938	7	23307667	23658110	loss-gain	27768	23532732	23532772	Mean_corpuscular_volume	region2inRegion1	40
CNVR75	939	7	23481211	23658110	loss-gain	27768	23532732	23532772	Mean_corpuscular_volume	region2inRegion1	40
CNVR74	938	7	23307667	23658110	loss-gain	3761	23536897	45102432	backfat_at_last_rib	overlapTail	121214
CNVR75	939	7	23481211	23658110	loss-gain	3761	23536897	45102432	backfat_at_last_rib	overlapTail	121214
CNVR76	942	7	24648309	24653067	loss-gain	3761	23536897	45102432	backfat_at_last_rib	region1inRegion2	4758
CNVR77	943	7	24735665	24740397	gain	3761	23536897	45102432	backfat_at_last_rib	region1inRegion2	4732
CNVR78	944	7	25359190	25399904	gain	3761	23536897	45102432	backfat_at_last_rib	region1inRegion2	40714
CNVR79	945	7	25488796	25557156	gain	3761	23536897	45102432	backfat_at_last_rib	region1inRegion2	68360
CNVR80	946	7	25785853	25888939	gain	3761	23536897	45102432	backfat_at_last_rib	region1inRegion2	103086
CNVR81	949	7	26267062	26297673	gain	3761	23536897	45102432	backfat_at_last_rib	region1inRegion2	30611
CNVR82	951	7	26600964	26608495	gain	3761	23536897	45102432	backfat_at_last_rib	region1inRegion2	7531
CNVR83	957	7	28525787	28565312	gain	3761	23536897	45102432	backfat_at_last_rib	region1inRegion2	39525
CNVR74	938	7	23307667	23658110	loss-gain	282	23536897	50310118	backfat_at_mid-back	overlapTail	121214
CNVR75	939	7	23481211	23658110	loss-gain	282	23536897	50310118	backfat_at_mid-back	overlapTail	121214
CNVR76	942	7	24648309	24653067	loss-gain	282	23536897	50310118	backfat_at_mid-back	region1inRegion2	4758
CNVR77	943	7	24735665	24740397	gain	282	23536897	50310118	backfat_at_mid-back	region1inRegion2	4732
CNVR78	944	7	25359190	25399904	gain	282	23536897	50310118	backfat_at_mid-back	region1inRegion2	40714
CNVR79	945	7	25488796	25557156	gain	282	23536897	50310118	backfat_at_mid-back	region1inRegion2	68360
CNVR80	946	7	25785853	25888939	gain	282	23536897	50310118	backfat_at_mid-back	region1inRegion2	103086
CNVR81	949	7	26267062	26297673	gain	282	23536897	50310118	backfat_at_mid-back	region1inRegion2	30611
CNVR82	951	7	26600964	26608495	gain	282	23536897	50310118	backfat_at_mid-back	region1inRegion2	7531
CNVR83	957	7	28525787	28565312	gain	282	23536897	50310118	backfat_at_mid-back	region1inRegion2	39525
CNVR74	938	7	23307667	23658110	loss-gain	415	23536897	56995195	Backfat_weight	overlapTail	121214
CNVR75	939	7	23481211	23658110	loss-gain	415	23536897	56995195	Backfat_weight	overlapTail	121214
CNVR76	942	7	24648309	24653067	loss-gain	415	23536897	56995195	Backfat_weight	region1inRegion2	4758
CNVR77	943	7	24735665	24740397	gain	415	23536897	56995195	Backfat_weight	region1inRegion2	4732
CNVR78	944	7	25359190	25399904	gain	415	23536897	56995195	Backfat_weight	region1inRegion2	40714
CNVR79	945	7	25488796	25557156	gain	415	23536897	56995195	Backfat_weight	region1inRegion2	68360
CNVR80	946	7	25785853	25888939	gain	415	23536897	56995195	Backfat_weight	region1inRegion2	103086
CNVR81	949	7	26267062	26297673	gain	415	23536897	56995195	Backfat_weight	region1inRegion2	30611
CNVR82	951	7	26600964	26608495	gain	415	23536897	56995195	Backfat_weight	region1inRegion2	7531
CNVR83	957	7	28525787	28565312	gain	415	23536897	56995195	Backfat_weight	region1inRegion2	39525
CNVR74	938	7	23307667	23658110	loss-gain	419	23536897	56995195	Backfat_thickness_between_3rd_and_4th_rib	overlapTail	121214
CNVR75	939	7	23481211	23658110	loss-gain	419	23536897	56995195	Backfat_thickness_between_3rd_and_4th_rib	overlapTail	121214
CNVR76	942	7	24648309	24653067	loss-gain	419	23536897	56995195	Backfat_thickness_between_3rd_and_4th_rib	region1inRegion2	4758
CNVR77	943	7	24735665	24740397	gain	419	23536897	56995195	Backfat_thickness_between_3rd_and_4th_rib	region1inRegion2	4732
CNVR78	944	7	25359190	25399904	gain	419	23536897	56995195	Backfat_thickness_between_3rd_and_4th_rib	region1inRegion2	40714
CNVR79	945	7	25488796	25557156	gain	419	23536897	56995195	Backfat_thickness_between_3rd_and_4th_rib	region1inRegion2	68360
CNVR80	946	7	25785853	25888939	gain	419	23536897	56995195	Backfat_thickness_between_3rd_and_4th_rib	region1inRegion2	103086
CNVR81	949	7	26267062	26297673	gain	419	23536897	56995195	Backfat_thickness_between_3rd_and_4th_rib	region1inRegion2	30611
CNVR82	951	7	26600964	26608495	gain	419	23536897	56995195	Backfat_thickness_between_3rd_and_4th_rib	region1inRegion2	7531
CNVR83	957	7	28525787	28565312	gain	419	23536897	56995195	Backfat_thickness_between_3rd_and_4th_rib	region1inRegion2	39525
CNVR74	938	7	23307667	23658110	loss-gain	417	23536897	64274644	Belly_weight	overlapTail	121214
CNVR75	939	7	23481211	23658110	loss-gain	417	23536897	64274644	Belly_weight	overlapTail	121214
CNVR76	942	7	24648309	24653067	loss-gain	417	23536897	64274644	Belly_weight	region1inRegion2	4758
CNVR77	943	7	24735665	24740397	gain	417	23536897	64274644	Belly_weight	region1inRegion2	4732
CNVR78	944	7	25359190	25399904	gain	417	23536897	64274644	Belly_weight	region1inRegion2	40714
CNVR79	945	7	25488796	25557156	gain	417	23536897	64274644	Belly_weight	region1inRegion2	68360
CNVR80	946	7	25785853	25888939	gain	417	23536897	64274644	Belly_weight	region1inRegion2	103086
CNVR81	949	7	26267062	26297673	gain	417	23536897	64274644	Belly_weight	region1inRegion2	30611
CNVR82	951	7	26600964	26608495	gain	417	23536897	64274644	Belly_weight	region1inRegion2	7531
CNVR83	957	7	28525787	28565312	gain	417	23536897	64274644	Belly_weight	region1inRegion2	39525
CNVR84	972	7	58579811	58584542	gain	417	23536897	64274644	Belly_weight	region1inRegion2	4731
CNVR85	975	7	59278633	59284219	loss	417	23536897	64274644	Belly_weight	region1inRegion2	5586
CNVR86	977	7	61355441	61417944	gain	417	23536897	64274644	Belly_weight	region1inRegion2	62503
CNVR74	938	7	23307667	23658110	loss-gain	298	23536897	88946228	Meat_color-L	overlapTail	121214
CNVR75	939	7	23481211	23658110	loss-gain	298	23536897	88946228	Meat_color-L	overlapTail	121214
CNVR76	942	7	24648309	24653067	loss-gain	298	23536897	88946228	Meat_color-L	region1inRegion2	4758
CNVR77	943	7	24735665	24740397	gain	298	23536897	88946228	Meat_color-L	region1inRegion2	4732
CNVR78	944	7	25359190	25399904	gain	298	23536897	88946228	Meat_color-L	region1inRegion2	40714
CNVR79	945	7	25488796	25557156	gain	298	23536897	88946228	Meat_color-L	region1inRegion2	68360
CNVR80	946	7	25785853	25888939	gain	298	23536897	88946228	Meat_color-L	region1inRegion2	103086
CNVR81	949	7	26267062	26297673	gain	298	23536897	88946228	Meat_color-L	region1inRegion2	30611
CNVR82	951	7	26600964	26608495	gain	298	23536897	88946228	Meat_color-L	region1inRegion2	7531
CNVR83	957	7	28525787	28565312	gain	298	23536897	88946228	Meat_color-L	region1inRegion2	39525
CNVR84	972	7	58579811	58584542	gain	298	23536897	88946228	Meat_color-L	region1inRegion2	4731
CNVR85	975	7	59278633	59284219	loss	298	23536897	88946228	Meat_color-L	region1inRegion2	5586
CNVR86	977	7	61355441	61417944	gain	298	23536897	88946228	Meat_color-L	region1inRegion2	62503
CNVR87	994	7	82234623	82374446	gain	298	23536897	88946228	Meat_color-L	region1inRegion2	139823
CNVR88	1002	7	84728780	84749202	gain	298	23536897	88946228	Meat_color-L	region1inRegion2	20422
CNVR74	938	7	23307667	23658110	loss-gain	59	23536897	120698338	Average_backfat_thickness	overlapTail	121214
CNVR75	939	7	23481211	23658110	loss-gain	59	23536897	120698338	Average_backfat_thickness	overlapTail	121214
CNVR76	942	7	24648309	24653067	loss-gain	59	23536897	120698338	Average_backfat_thickness	region1inRegion2	4758
CNVR77	943	7	24735665	24740397	gain	59	23536897	120698338	Average_backfat_thickness	region1inRegion2	4732
CNVR78	944	7	25359190	25399904	gain	59	23536897	120698338	Average_backfat_thickness	region1inRegion2	40714
CNVR79	945	7	25488796	25557156	gain	59	23536897	120698338	Average_backfat_thickness	region1inRegion2	68360
CNVR80	946	7	25785853	25888939	gain	59	23536897	120698338	Average_backfat_thickness	region1inRegion2	103086
CNVR81	949	7	26267062	26297673	gain	59	23536897	120698338	Average_backfat_thickness	region1inRegion2	30611
CNVR82	951	7	26600964	26608495	gain	59	23536897	120698338	Average_backfat_thickness	region1inRegion2	7531
CNVR83	957	7	28525787	28565312	gain	59	23536897	120698338	Average_backfat_thickness	region1inRegion2	39525
CNVR84	972	7	58579811	58584542	gain	59	23536897	120698338	Average_backfat_thickness	region1inRegion2	4731
CNVR85	975	7	59278633	59284219	loss	59	23536897	120698338	Average_backfat_thickness	region1inRegion2	5586
CNVR86	977	7	61355441	61417944	gain	59	23536897	120698338	Average_backfat_thickness	region1inRegion2	62503
CNVR87	994	7	82234623	82374446	gain	59	23536897	120698338	Average_backfat_thickness	region1inRegion2	139823
CNVR88	1002	7	84728780	84749202	gain	59	23536897	120698338	Average_backfat_thickness	region1inRegion2	20422
CNVR89	1011	7	102790067	102823288	gain	59	23536897	120698338	Average_backfat_thickness	region1inRegion2	33221
CNVR90	1021	7	111620166	111623056	loss	59	23536897	120698338	Average_backfat_thickness	region1inRegion2	2890
CNVR74	938	7	23307667	23658110	loss-gain	27776	23632790	23632830	Mean_corpuscular_volume	region2inRegion1	40
CNVR75	939	7	23481211	23658110	loss-gain	27776	23632790	23632830	Mean_corpuscular_volume	region2inRegion1	40
CNVR76	942	7	24648309	24653067	loss-gain	66003	24153455	25229400	Average_backfat_thickness	region1inRegion2	4758
CNVR77	943	7	24735665	24740397	gain	66003	24153455	25229400	Average_backfat_thickness	region1inRegion2	4732
CNVR76	942	7	24648309	24653067	loss-gain	66004	24153455	25229400	Drip_loss	region1inRegion2	4758

CNVR77	943	7	24735665	24740397	gain	66004	24153455	25229400	Drip_loss	region1inRegion2	4732
CNVR76	942	7	24648309	24653067	loss-gain	66005	24153455	25229400	CIE-L*	region1inRegion2	4758
CNVR77	943	7	24735665	24740397	gain	66005	24153455	25229400	CIE-L*	region1inRegion2	4732
CNVR76	942	7	24648309	24653067	loss-gain	66006	24153455	25229400	Total_muscle_fiber_number	region1inRegion2	4758
CNVR77	943	7	24735665	24740397	gain	66006	24153455	25229400	Total_muscle_fiber_number	region1inRegion2	4732
CNVR76	942	7	24648309	24653067	loss-gain	66007	24153455	25229400	Loin_muscle_area	region1inRegion2	4758
CNVR77	943	7	24735665	24740397	gain	66007	24153455	25229400	Loin_muscle_area	region1inRegion2	4732
CNVR76	942	7	24648309	24653067	loss-gain	66008	24153455	25229400	pH_45_minutes_post_mortem	region1inRegion2	4758
CNVR77	943	7	24735665	24740397	gain	66008	24153455	25229400	pH_45_minutes_post_mortem	region1inRegion2	4732
CNVR78	944	7	25359190	25399904	gain	333	24749679	26754793	Average_daily_gain	region1inRegion2	40714
CNVR79	945	7	25488796	25557156	gain	333	24749679	26754793	Average_daily_gain	region1inRegion2	68360
CNVR80	946	7	25785853	25888939	gain	333	24749679	26754793	Average_daily_gain	region1inRegion2	103086
CNVR81	949	7	26267062	26297673	gain	333	24749679	26754793	Average_daily_gain	region1inRegion2	30611
CNVR82	951	7	26600964	26608495	gain	333	24749679	26754793	Average_daily_gain	region1inRegion2	7531
CNVR78	944	7	25359190	25399904	gain	345	24749679	26754793	Average_daily_gain	region1inRegion2	40714
CNVR79	945	7	25488796	25557156	gain	345	24749679	26754793	Average_daily_gain	region1inRegion2	68360
CNVR80	946	7	25785853	25888939	gain	345	24749679	26754793	Average_daily_gain	region1inRegion2	103086
CNVR81	949	7	26267062	26297673	gain	345	24749679	26754793	Average_daily_gain	region1inRegion2	30611
CNVR82	951	7	26600964	26608495	gain	345	24749679	26754793	Average_daily_gain	region1inRegion2	7531
CNVR78	944	7	25359190	25399904	gain	5502	24749679	26754793	Skin_weight	region1inRegion2	40714
CNVR79	945	7	25488796	25557156	gain	5502	24749679	26754793	Skin_weight	region1inRegion2	68360
CNVR80	946	7	25785853	25888939	gain	5502	24749679	26754793	Skin_weight	region1inRegion2	103086
CNVR81	949	7	26267062	26297673	gain	5502	24749679	26754793	Skin_weight	region1inRegion2	30611
CNVR82	951	7	26600964	26608495	gain	5502	24749679	26754793	Skin_weight	region1inRegion2	7531
CNVR78	944	7	25359190	25399904	gain	9842	24749679	32380448	Average_backfat_thickness	region1inRegion2	40714
CNVR79	945	7	25488796	25557156	gain	9842	24749679	32380448	Average_backfat_thickness	region1inRegion2	68360
CNVR80	946	7	25785853	25888939	gain	9842	24749679	32380448	Average_backfat_thickness	region1inRegion2	103086
CNVR81	949	7	26267062	26297673	gain	9842	24749679	32380448	Average_backfat_thickness	region1inRegion2	30611
CNVR82	951	7	26600964	26608495	gain	9842	24749679	32380448	Average_backfat_thickness	region1inRegion2	7531
CNVR83	957	7	28525787	28565312	gain	9842	24749679	32380448	Average_backfat_thickness	region1inRegion2	39525
CNVR78	944	7	25359190	25399904	gain	9843	24749679	32380448	Leaf_fat_weight	region1inRegion2	40714
CNVR79	945	7	25488796	25557156	gain	9843	24749679	32380448	Leaf_fat_weight	region1inRegion2	68360
CNVR80	946	7	25785853	25888939	gain	9843	24749679	32380448	Leaf_fat_weight	region1inRegion2	103086
CNVR81	949	7	26267062	26297673	gain	9843	24749679	32380448	Leaf_fat_weight	region1inRegion2	30611
CNVR82	951	7	26600964	26608495	gain	9843	24749679	32380448	Leaf_fat_weight	region1inRegion2	7531
CNVR83	957	7	28525787	28565312	gain	9843	24749679	32380448	Leaf_fat_weight	region1inRegion2	39525
CNVR78	944	7	25359190	25399904	gain	29698	24888146	107293999	Carcass_length	region1inRegion2	40714
CNVR79	945	7	25488796	25557156	gain	29698	24888146	107293999	Carcass_length	region1inRegion2	68360
CNVR80	946	7	25785853	25888939	gain	29698	24888146	107293999	Carcass_length	region1inRegion2	103086
CNVR81	949	7	26267062	26297673	gain	29698	24888146	107293999	Carcass_length	region1inRegion2	30611
CNVR82	951	7	26600964	26608495	gain	29698	24888146	107293999	Carcass_length	region1inRegion2	7531
CNVR83	957	7	28525787	28565312	gain	29698	24888146	107293999	Carcass_length	region1inRegion2	39525
CNVR84	972	7	58579811	58584542	gain	29698	24888146	107293999	Carcass_length	region1inRegion2	4731
CNVR85	975	7	59278633	59284219	loss	29698	24888146	107293999	Carcass_length	region1inRegion2	5586
CNVR86	977	7	61355441	61417944	gain	29698	24888146	107293999	Carcass_length	region1inRegion2	62503
CNVR87	994	7	82234623	82374446	gain	29698	24888146	107293999	Carcass_length	region1inRegion2	139823
CNVR88	1002	7	84728780	84749202	gain	29698	24888146	107293999	Carcass_length	region1inRegion2	20422
CNVR89	1011	7	102790067	102823288	gain	29698	24888146	107293999	Carcass_length	region1inRegion2	33221
CNVR78	944	7	25359190	25399904	gain	38093	24888146	124300676	Muscle_moisture_percentage	region1inRegion2	40714
CNVR79	945	7	25488796	25557156	gain	38093	24888146	124300676	Muscle_moisture_percentage	region1inRegion2	68360
CNVR80	946	7	25785853	25888939	gain	38093	24888146	124300676	Muscle_moisture_percentage	region1inRegion2	103086
CNVR81	949	7	26267062	26297673	gain	38093	24888146	124300676	Muscle_moisture_percentage	region1inRegion2	30611
CNVR82	951	7	26600964	26608495	gain	38093	24888146	124300676	Muscle_moisture_percentage	region1inRegion2	7531
CNVR83	957	7	28525787	28565312	gain	38093	24888146	124300676	Muscle_moisture_percentage	region1inRegion2	39525
CNVR84	972	7	58579811	58584542	gain	38093	24888146	124300676	Muscle_moisture_percentage	region1inRegion2	4731
CNVR85	975	7	59278633	59284219	loss	38093	24888146	124300676	Muscle_moisture_percentage	region1inRegion2	5586
CNVR86	977	7	61355441	61417944	gain	38093	24888146	124300676	Muscle_moisture_percentage	region1inRegion2	62503
CNVR87	994	7	82234623	82374446	gain	38093	24888146	124300676	Muscle_moisture_percentage	region1inRegion2	139823
CNVR88	1002	7	84728780	84749202	gain	38093	24888146	124300676	Muscle_moisture_percentage	region1inRegion2	20422
CNVR89	1011	7	102790067	102823288	gain	38093	24888146	124300676	Muscle_moisture_percentage	region1inRegion2	33221
CNVR90	1021	7	111620166	111623056	loss	38093	24888146	124300676	Muscle_moisture_percentage	region1inRegion2	2890
CNVR78	944	7	25359190	25399904	gain	18059	25122082	33757808	backfat_at_mid-back	region1inRegion2	40714
CNVR79	945	7	25488796	25557156	gain	18059	25122082	33757808	backfat_at_mid-back	region1inRegion2	68360
CNVR80	946	7	25785853	25888939	gain	18059	25122082	33757808	backfat_at_mid-back	region1inRegion2	103086
CNVR81	949	7	26267062	26297673	gain	18059	25122082	33757808	backfat_at_mid-back	region1inRegion2	30611
CNVR82	951	7	26600964	26608495	gain	18059	25122082	33757808	backfat_at_mid-back	region1inRegion2	7531
CNVR83	957	7	28525787	28565312	gain	18059	25122082	33757808	backfat_at_mid-back	region1inRegion2	39525
CNVR78	944	7	25359190	25399904	gain	95368	25176925	29136708	Myristic_acid_content	region1inRegion2	40714
CNVR79	945	7	25488796	25557156	gain	95368	25176925	29136708	Myristic_acid_content	region1inRegion2	68360
CNVR80	946	7	25785853	25888939	gain	95368	25176925	29136708	Myristic_acid_content	region1inRegion2	103086
CNVR81	949	7	26267062	26297673	gain	95368	25176925	29136708	Myristic_acid_content	region1inRegion2	30611
CNVR82	951	7	26600964	26608495	gain	95368	25176925	29136708	Myristic_acid_content	region1inRegion2	7531
CNVR83	957	7	28525787	28565312	gain	95368	25176925	29136708	Myristic_acid_content	region1inRegion2	39525
CNVR78	944	7	25359190	25399904	gain	95369	25176925	29560469	Palmitoleic_acid_content	region1inRegion2	40714
CNVR79	945	7	25488796	25557156	gain	95369	25176925	29560469	Palmitoleic_acid_content	region1inRegion2	68360
CNVR80	946	7	25785853	25888939	gain	95369	25176925	29560469	Palmitoleic_acid_content	region1inRegion2	103086
CNVR81	949	7	26267062	26297673	gain	95369	25176925	29560469	Palmitoleic_acid_content	region1inRegion2	30611
CNVR82	951	7	26600964	26608495	gain	95369	25176925	29560469	Palmitoleic_acid_content	region1inRegion2	7531
CNVR83	957	7	28525787	28565312	gain	95369	25176925	29560469	Palmitoleic_acid_content	region1inRegion2	39525
CNVR78	944	7	25359190	25399904	gain	95370	25176925	29560469	Eicosadienoic_acid_content	region1inRegion2	40714
CNVR79	945	7	25488796	25557156	gain	95370	25176925	29560469	Eicosadienoic_acid_content	region1inRegion2	68360
CNVR80	946	7	25785853	25888939	gain	95370	25176925	29560469	Eicosadienoic_acid_content	region1inRegion2	103086
CNVR81	949	7	26267062	26297673	gain	95370	25176925	29560469	Eicosadienoic_acid_content	region1inRegion2	30611
CNVR82	951	7	26600964	26608495	gain	95370	25176925	29560469	Eicosadienoic_acid_content	region1inRegion2	7531
CNVR83	957	7	28525787	28565312	gain	95370	25176925	29560469	Eicosadienoic_acid_content	region1inRegion2	39525
CNVR78	944	7	25359190	25399904	gain	65004	25176925	36677045	Liver_weight	region1inRegion2	40714
CNVR79	945	7	25488796	25557156	gain	65004	25176925	36677045	Liver_weight	region1inRegion2	68360
CNVR80	946	7	25785853	25888939	gain	65004	25176925	36677045	Liver_weight	region1inRegion2	103086
CNVR81	949	7	26267062	26297673	gain	65004	25176925	36677045	Liver_weight	region1inRegion2	30611
CNVR82	951	7	26600964	26608495	gain	65004	25176925	36677045	Liver_weight	region1inRegion2	7531
CNVR83	957	7	28525787	28565312	gain	65004	25176925	36677045	Liver_weight	region1inRegion2	39525
CNVR78	944	7	25359190	25399904	gain	6538	25379271	37026269	Mean_corpuscular_hemoglobin_content	overlapTail	20634
CNVR79	945	7	25488796	25557156	gain	6538	25379271	37026269	Mean_corpuscular_hemoglobin_content	region1inRegion2	68360
CNVR80	946	7	25785853	25888939	gain	6538	25379271	37026269	Mean_corpuscular_hemoglobin_content	region1inRegion2	103086
CNVR81	949	7	26267062	26297673	gain	6538	25379271	37026269	Mean_corpuscular_hemoglobin_content	region1inRegion2	30611
CNVR82	951	7	26600964	26608495	gain	6538	25379271	37026269	Mean_corpuscular_hemoglobin_content	region1inRegion2	7531
CNVR83	957	7	28525787	28565312	gain	6538	25379271	37026269	Mean_corpuscular_hemoglobin_content	region1inRegion2	39525
CNVR78	944	7	25359190	25399904	gain	6541	25379271	37026269	Mean_corpuscular_volume	overlapTail	20634
CNVR79	945	7	25488796	25557156	gain	6541	25379271	37026269	Mean_corpuscular_volume	region1inRegion2	68360
CNVR80	946	7	25785853	25888939	gain	6541	25379271	37026269	Mean_corpuscular_volume	region1inRegion2	103086
CNVR81	949	7	26267062	26297673	gain	6541	25379271	37026269	Mean_corpuscular_volume	region1inRegion2	30611
CNVR82	951	7	26600964	26608495	gain	6541	25379271	37026269	Mean_corpuscular_volume	region1inRegion2	7531
CNVR83	957	7	28525787	28565312	gain	6541	25379271	37026269	Mean_corpuscular_volume	region1inRegion2	39525
CNVR78	944	7	25359190	25399904	gain	9585	25379271	37026269	Feed_conversion_ratio	overlapTail	20634
CNVR79	945	7	25488796	25557156	gain	9585	25379271	37026269	Feed_conversion_ratio	region1inRegion2	68360
CNVR80	946	7	25785853	25888939	gain	9585	25379271	37026269	Feed_conversion_ratio	region1inRegion2	103086
CNVR81	949	7	26267062	26297673	gain	9585	25379271	37026269	Feed_conversion_ratio	region1inRegion2	30611
CNVR82	951	7	26600964	26608495	gain	9585	25379271	37026269	Feed_conversion_ratio	region1inRegion2	7531
CNVR83	957	7	28525787	28565312	gain	9585	25379271	37026269	Feed_conversion_ratio	region1inRegion2	39525
CNVR78	944	7	25359190	25399904	gain	101765	25384197	25384237	Eicosadienoic_acid_to_linoleic_acid_ratio	region2inRegion1	40
CNVR78	944	7	25359190	25399904	gain	127691	25384197	25384237	Mean_corpuscular_volume	region2inRegion1	40
CNVR79	945	7	25488796	25557156	gain	127735	25489726	25489766	Mean_corpuscular_volume	region2inRegion1	40
CNVR79	945	7	25488796	25557156	gain	127736	25516761	25516801	Mean_corpuscular_volume	region2inRegion1	40
CNVR80	946	7	25785853	25888939	gain	140316	25565917	26501975	PRRS_viral_load	region1inRegion2	103086
CNVR81	949	7	26267062	26297673	gain	140316	25565917	26501975	PRRS_viral_load	region1inRegion2	30611
CNVR80	946	7	25785853	25888939	gain	140324	25565917	26501975	PRRS_viral_load	region1inRegion2	103086
CNVR81	949	7	26267062	26297673	gain	140324	25565917	26501975	PRRS_viral_load	region1inRegion2	30611
CNVR80	946	7	25785853	25888939	gain	127737	25872490	25872530	Mean_corpuscular_volume	region2inRegion1	40
CNVR82	951	7	26600964	26608495	gain	135743	26331485	36280791	Shoulder_subcutaneous_fat_thickness	region1inRegion2	7531
CNVR83	957	7	28525787	28565312	gain	135743	26331485	36280791	Shoulder_subcutaneous_fat_thickness	region1inRegion2	39525
CNVR82	951	7	26600964	26608495	gain	135744	26331485	37710365	Backfat_at_first_rib	region1inRegion2	7531
CNVR83	957	7	28525787	28565312	gain	135744	26331485	37710365	Backfat_at_first_rib	region1inRegion2	39525

CNVR82	951	7	26600964	26608495	gain	65006	26433052	41454098	Heart_weight	region1inRegion2	7531
CNVR83	957	7	28525787	28565312	gain	65006	26433052	41454098	Heart_weight	region1inRegion2	39525
CNVR82	951	7	26600964	26608495	gain	22300	26466946	27562670	Loin_muscle_area	region1inRegion2	7531
CNVR82	951	7	26600964	26608495	gain	22301	26466946	27562670	Loin_muscle_area	region1inRegion2	7531
CNVR82	951	7	26600964	26608495	gain	22302	26466946	27562670	Loin_muscle_area	region1inRegion2	7531
CNVR82	951	7	26600964	26608495	gain	22303	26466946	27562670	Loin_muscle_area	region1inRegion2	7531
CNVR82	951	7	26600964	26608495	gain	22304	26466946	27562670	Loin_muscle_area	region1inRegion2	7531
CNVR82	951	7	26600964	26608495	gain	22305	26466946	27562670	Loin_muscle_area	region1inRegion2	7531
CNVR82	951	7	26600964	26608495	gain	22306	26466946	27562670	Loin_muscle_area	region1inRegion2	7531
CNVR82	951	7	26600964	26608495	gain	22307	26466946	27562670	Loin_muscle_area	region1inRegion2	7531
CNVR82	951	7	26600964	26608495	gain	22308	26466946	27562670	Loin_muscle_area	region1inRegion2	7531
CNVR82	951	7	26600964	26608495	gain	64731	26466946	27562670	Thoracolumbar_vertebra_number	region1inRegion2	7531
CNVR82	951	7	26600964	26608495	gain	135748	26501975	36120461	Backfat_at_rump	region1inRegion2	7531
CNVR83	957	7	28525787	28565312	gain	135748	26501975	36120461	Backfat_at_rump	region1inRegion2	39525
CNVR82	951	7	26600964	26608495	gain	135747	26501975	37710365	Backfat_at_first_rib	region1inRegion2	7531
CNVR83	957	7	28525787	28565312	gain	135747	26501975	37710365	Backfat_at_first_rib	region1inRegion2	39525
CNVR82	951	7	26600964	26608495	gain	135749	26501975	37710365	backfat_at_last_rib	region1inRegion2	7531
CNVR83	957	7	28525787	28565312	gain	135749	26501975	37710365	backfat_at_last_rib	region1inRegion2	39525
CNVR82	951	7	26600964	26608495	gain	65005	26501975	40992620	Liver_weight	region1inRegion2	7531
CNVR83	957	7	28525787	28565312	gain	65005	26501975	40992620	Liver_weight	region1inRegion2	39525
CNVR83	957	7	28525787	28565312	gain	135751	26697426	34772625	Shoulder_subcutaneous_fat_thickness	region1inRegion2	39525
CNVR83	957	7	28525787	28565312	gain	135750	26697426	36120461	Leaf_fat_weight	region1inRegion2	39525
CNVR83	957	7	28525787	28565312	gain	5643	26756689	50769724	Ear_size	region1inRegion2	39525
CNVR83	957	7	28525787	28565312	gain	18006	26756689	62374975	Subcutanous_fat_area	region1inRegion2	39525
CNVR84	972	7	58579811	58584542	gain	18006	26756689	62374975	Subcutanous_fat_area	region1inRegion2	4731
CNVR85	975	7	59278633	59284219	loss	18006	26756689	62374975	Subcutanous_fat_area	region1inRegion2	5586
CNVR86	977	7	61355441	61417944	gain	18006	26756689	62374975	Subcutanous_fat_area	region1inRegion2	62503
CNVR83	957	7	28525787	28565312	gain	187	27288146	69157768	Body_weight_(birth)	region1inRegion2	39525
CNVR84	972	7	58579811	58584542	gain	187	27288146	69157768	Body_weight_(birth)	region1inRegion2	4731
CNVR85	975	7	59278633	59284219	loss	187	27288146	69157768	Body_weight_(birth)	region1inRegion2	5586
CNVR86	977	7	61355441	61417944	gain	187	27288146	69157768	Body_weight_(birth)	region1inRegion2	62503
CNVR83	957	7	28525787	28565312	gain	135746	27487944	32492111	Abdominal_fat_weight	region1inRegion2	39525
CNVR83	957	7	28525787	28565312	gain	5817	28117351	30150257	Cannon_bone_circumference	region1inRegion2	39525
CNVR83	957	7	28525787	28565312	gain	5818	28117351	30150257	Cannon_bone_circumference	region1inRegion2	39525
CNVR83	957	7	28525787	28565312	gain	5819	28117351	30150257	Japanese_color_scale	region1inRegion2	39525
CNVR83	957	7	28525787	28565312	gain	9041	28117351	30150257	Muscle_moisture_percentage	region1inRegion2	39525
CNVR83	957	7	28525787	28565312	gain	9047	28117351	30150257	Hematin_pigmentation	region1inRegion2	39525
CNVR83	957	7	28525787	28565312	gain	9055	28117351	30150257	Meat_color_score	region1inRegion2	39525
CNVR83	957	7	28525787	28565312	gain	127821	28541754	28541794	Mean_corpuscular_hemoglobin_concentration	region2inRegion1	40
CNVR83	957	7	28525787	28565312	gain	127822	28559157	28559197	Mean_corpuscular_hemoglobin_concentration	region2inRegion1	40
CNVR84	972	7	58579811	58584542	gain	62	30781324	122982289	backfat_at_last_rib	region1inRegion2	4731
CNVR85	975	7	59278633	59284219	loss	62	30781324	122982289	backfat_at_last_rib	region1inRegion2	5586
CNVR86	977	7	61355441	61417944	gain	62	30781324	122982289	backfat_at_last_rib	region1inRegion2	62503
CNVR87	994	7	82234623	82374446	gain	62	30781324	122982289	backfat_at_last_rib	region1inRegion2	139823
CNVR88	1002	7	84728780	84749202	gain	62	30781324	122982289	backfat_at_last_rib	region1inRegion2	20422
CNVR89	1011	7	102790067	102823288	gain	62	30781324	122982289	backfat_at_last_rib	region1inRegion2	33221
CNVR90	1021	7	111620166	111623056	loss	62	30781324	122982289	backfat_at_last_rib	region1inRegion2	2890
CNVR84	972	7	58579811	58584542	gain	386	30847287	94411320	Ham_weight	region1inRegion2	4731
CNVR85	975	7	59278633	59284219	loss	386	30847287	94411320	Ham_weight	region1inRegion2	5586
CNVR86	977	7	61355441	61417944	gain	386	30847287	94411320	Ham_weight	region1inRegion2	62503
CNVR87	994	7	82234623	82374446	gain	386	30847287	94411320	Ham_weight	region1inRegion2	139823
CNVR88	1002	7	84728780	84749202	gain	386	30847287	94411320	Ham_weight	region1inRegion2	20422
CNVR84	972	7	58579811	58584542	gain	8864	32380448	69400776	Ear_weight	region1inRegion2	4731
CNVR85	975	7	59278633	59284219	loss	8864	32380448	69400776	Ear_weight	region1inRegion2	5586
CNVR86	977	7	61355441	61417944	gain	8864	32380448	69400776	Ear_weight	region1inRegion2	62503
CNVR84	972	7	58579811	58584542	gain	8865	32380448	69400776	Ear_weight	region1inRegion2	4731
CNVR85	975	7	59278633	59284219	loss	8865	32380448	69400776	Ear_weight	region1inRegion2	5586
CNVR86	977	7	61355441	61417944	gain	8865	32380448	69400776	Ear_weight	region1inRegion2	62503
CNVR84	972	7	58579811	58584542	gain	8866	32380448	69400776	Ear_area	region1inRegion2	4731
CNVR85	975	7	59278633	59284219	loss	8866	32380448	69400776	Ear_area	region1inRegion2	5586
CNVR86	977	7	61355441	61417944	gain	8866	32380448	69400776	Ear_area	region1inRegion2	62503
CNVR84	972	7	58579811	58584542	gain	8867	32380448	69400776	Ear_area	region1inRegion2	4731
CNVR85	975	7	59278633	59284219	loss	8867	32380448	69400776	Ear_area	region1inRegion2	5586
CNVR86	977	7	61355441	61417944	gain	8867	32380448	69400776	Ear_area	region1inRegion2	62503
CNVR84	972	7	58579811	58584542	gain	8868	32380448	69400776	Ear_erectness	region1inRegion2	4731
CNVR85	975	7	59278633	59284219	loss	8868	32380448	69400776	Ear_erectness	region1inRegion2	5586
CNVR86	977	7	61355441	61417944	gain	8868	32380448	69400776	Ear_erectness	region1inRegion2	62503
CNVR84	972	7	58579811	58584542	gain	17802	32380448	78541888	backfat_at_last_rib	region1inRegion2	4731
CNVR85	975	7	59278633	59284219	loss	17802	32380448	78541888	backfat_at_last_rib	region1inRegion2	5586
CNVR86	977	7	61355441	61417944	gain	17802	32380448	78541888	backfat_at_last_rib	region1inRegion2	62503
CNVR84	972	7	58579811	58584542	gain	5648	33110562	134238358	Ear_erectness	region1inRegion2	4731
CNVR85	975	7	59278633	59284219	loss	5648	33110562	134238358	Ear_erectness	region1inRegion2	5586
CNVR86	977	7	61355441	61417944	gain	5648	33110562	134238358	Ear_erectness	region1inRegion2	62503
CNVR87	994	7	82234623	82374446	gain	5648	33110562	134238358	Ear_erectness	region1inRegion2	139823
CNVR88	1002	7	84728780	84749202	gain	5648	33110562	134238358	Ear_erectness	region1inRegion2	20422
CNVR89	1011	7	102790067	102823288	gain	5648	33110562	134238358	Ear_erectness	region1inRegion2	33221
CNVR90	1021	7	111620166	111623056	loss	5648	33110562	134238358	Ear_erectness	region1inRegion2	2890
CNVR91	1035	7	131449863	131460402	gain	5648	33110562	134238358	Ear_erectness	region1inRegion2	10539
CNVR92	1040	7	132961208	132975832	loss	5648	33110562	134238358	Ear_erectness	region1inRegion2	14624
CNVR93	1041	7	132985953	133003168	loss	5648	33110562	134238358	Ear_erectness	region1inRegion2	17215
CNVR84	972	7	58579811	58584542	gain	140348	33826747	75045135	PRRSV_susceptibility	region1inRegion2	4731
CNVR85	975	7	59278633	59284219	loss	140348	33826747	75045135	PRRSV_susceptibility	region1inRegion2	5586
CNVR86	977	7	61355441	61417944	gain	140348	33826747	75045135	PRRSV_susceptibility	region1inRegion2	62503
CNVR84	972	7	58579811	58584542	gain	5239	34099350	101100192	backfat_above_muscle_dorsi	region1inRegion2	4731
CNVR85	975	7	59278633	59284219	loss	5239	34099350	101100192	backfat_above_muscle_dorsi	region1inRegion2	5586
CNVR86	977	7	61355441	61417944	gain	5239	34099350	101100192	backfat_above_muscle_dorsi	region1inRegion2	62503
CNVR87	994	7	82234623	82374446	gain	5239	34099350	101100192	backfat_above_muscle_dorsi	region1inRegion2	139823
CNVR88	1002	7	84728780	84749202	gain	5239	34099350	101100192	backfat_above_muscle_dorsi	region1inRegion2	20422
CNVR84	972	7	58579811	58584542	gain	5653	36271493	64962096	Ear_erectness	region1inRegion2	4731
CNVR85	975	7	59278633	59284219	loss	5653	36271493	64962096	Ear_erectness	region1inRegion2	5586
CNVR86	977	7	61355441	61417944	gain	5653	36271493	64962096	Ear_erectness	region1inRegion2	62503
CNVR84	972	7	58579811	58584542	gain	16837	36525998	63372652	backfat_at_last_rib	region1inRegion2	4731
CNVR85	975	7	59278633	59284219	loss	16837	36525998	63372652	backfat_at_last_rib	region1inRegion2	5586
CNVR86	977	7	61355441	61417944	gain	16837	36525998	63372652	backfat_at_last_rib	region1inRegion2	62503
CNVR84	972	7	58579811	58584542	gain	1059	36993248	63372652	Backfat_weight	region1inRegion2	4731
CNVR85	975	7	59278633	59284219	loss	1059	36993248	63372652	Backfat_weight	region1inRegion2	5586
CNVR86	977	7	61355441	61417944	gain	1059	36993248	63372652	Backfat_weight	region1inRegion2	62503
CNVR84	972	7	58579811	58584542	gain	4209	36993248	64962096	androstenone,_laboratory	region1inRegion2	4731
CNVR85	975	7	59278633	59284219	loss	4209	36993248	64962096	androstenone,_laboratory	region1inRegion2	5586
CNVR86	977	7	61355441	61417944	gain	4209	36993248	64962096	androstenone,_laboratory	region1inRegion2	62503
CNVR84	972	7	58579811	58584542	gain	8935	36993248	64962096	Hind_leg_conformation	region1inRegion2	4731
CNVR85	975	7	59278633	59284219	loss	8935	36993248	64962096	Hind_leg_conformation	region1inRegion2	5586
CNVR86	977	7	61355441	61417944	gain	8935	36993248	64962096	Hind_leg_conformation	region1inRegion2	62503
CNVR84	972	7	58579811	58584542	gain	8949	36993248	64962096	Physis_score	region1inRegion2	4731
CNVR85	975	7	59278633	59284219	loss	8949	36993248	64962096	Physis_score	region1inRegion2	5586
CNVR86	977	7	61355441	61417944	gain	8949	36993248	64962096	Physis_score	region1inRegion2	62503
CNVR84	972	7	58579811	58584542	gain	5389	36993248	99614552	Hemoglobin	region1inRegion2	4731
CNVR85	975	7	59278633	59284219	loss	5389	36993248	99614552	Hemoglobin	region1inRegion2	5586
CNVR86	977	7	61355441	61417944	gain	5389	36993248	99614552	Hemoglobin	region1inRegion2	62503
CNVR87	994	7	82234623	82374446	gain	5389	36993248	99614552	Hemoglobin	region1inRegion2	139823
CNVR88	1002	7	84728780	84749202	gain	5389	36993248	99614552	Hemoglobin	region1inRegion2	20422
CNVR84	972	7	58579811	58584542	gain	5397	36993248	99614552	Red_blood_cell_count	region1inRegion2	4731
CNVR85	975	7	59278633	59284219	loss	5397	36993248	99614552	Red_blood_cell_count	region1inRegion2	5586
CNVR86	977	7	61355441	61417944	gain	5397	36993248	99614552	Red_blood_cell_count	region1inRegion2	62503
CNVR87	994	7	82234623	82374446	gain	5397	36993248	99614552	Red_blood_cell_count	region1inRegion2	139823
CNVR88	1002	7	84728780	84749202	gain	5397	36993248	99614552	Red_blood_cell_count	region1inRegion2	20422
CNVR84	972	7	58579811	58584542	gain	5412	36993248	99614552	hematocrit	region1inRegion2	4731
CNVR85	975	7	59278633	59284219	loss	5412	36993248	99614552	hematocrit	region1inRegion2	5586
CNVR86	977	7	61355441	61417944	gain	5412	36993248	99614552	hematocrit	region1inRegion2	62503
CNVR87	994	7	82234623	82374446	gain	5412	36993248	99614552	hematocrit	region1inRegion2	139823
CNVR88	1002	7	84728780	84749202	gain	5412	36993248	99614552	hematocrit	region1inRegion2	20422
CNVR84	972	7	58579811	58584542	gain	5426	36993248	99614552	Mean_corpuscular_hemoglobin_content	region1inRegion2	4731

CNVR85	975	7	59278633	59284219	loss	5426	36993248	99614552	Mean_corpuscular_hemoglobin_content	region1inRegion2	5586
CNVR86	977	7	61355441	61417944	gain	5426	36993248	99614552	Mean_corpuscular_hemoglobin_content	region1inRegion2	62503
CNVR87	994	7	82234623	82374446	gain	5426	36993248	99614552	Mean_corpuscular_hemoglobin_content	region1inRegion2	139823
CNVR88	1002	7	84728780	84749202	gain	5426	36993248	99614552	Mean_corpuscular_hemoglobin_content	region1inRegion2	20422
CNVR84	972	7	58579811	58584542	gain	6331	36993248	99614552	Bicarbonate_level	region1inRegion2	4731
CNVR85	975	7	59278633	59284219	loss	6331	36993248	99614552	Bicarbonate_level	region1inRegion2	5586
CNVR86	977	7	61355441	61417944	gain	6331	36993248	99614552	Bicarbonate_level	region1inRegion2	62503
CNVR87	994	7	82234623	82374446	gain	6331	36993248	99614552	Bicarbonate_level	region1inRegion2	139823
CNVR88	1002	7	84728780	84749202	gain	6331	36993248	99614552	Bicarbonate_level	region1inRegion2	20422
CNVR84	972	7	58579811	58584542	gain	6334	36993248	99614552	Carbon_dioxide_level	region1inRegion2	4731
CNVR85	975	7	59278633	59284219	loss	6334	36993248	99614552	Carbon_dioxide_level	region1inRegion2	5586
CNVR86	977	7	61355441	61417944	gain	6334	36993248	99614552	Carbon_dioxide_level	region1inRegion2	62503
CNVR87	994	7	82234623	82374446	gain	6334	36993248	99614552	Carbon_dioxide_level	region1inRegion2	139823
CNVR88	1002	7	84728780	84749202	gain	6334	36993248	99614552	Carbon_dioxide_level	region1inRegion2	20422
CNVR84	972	7	58579811	58584542	gain	6337	36993248	99614552	Base_excess	region1inRegion2	4731
CNVR85	975	7	59278633	59284219	loss	6337	36993248	99614552	Base_excess	region1inRegion2	5586
CNVR86	977	7	61355441	61417944	gain	6337	36993248	99614552	Base_excess	region1inRegion2	62503
CNVR87	994	7	82234623	82374446	gain	6337	36993248	99614552	Base_excess	region1inRegion2	139823
CNVR88	1002	7	84728780	84749202	gain	6337	36993248	99614552	Base_excess	region1inRegion2	20422
CNVR84	972	7	58579811	58584542	gain	6346	36993248	99614552	Alkaline_phosphatase_activity	region1inRegion2	4731
CNVR85	975	7	59278633	59284219	loss	6346	36993248	99614552	Alkaline_phosphatase_activity	region1inRegion2	5586
CNVR86	977	7	61355441	61417944	gain	6346	36993248	99614552	Alkaline_phosphatase_activity	region1inRegion2	62503
CNVR87	994	7	82234623	82374446	gain	6346	36993248	99614552	Alkaline_phosphatase_activity	region1inRegion2	139823
CNVR88	1002	7	84728780	84749202	gain	6346	36993248	99614552	Alkaline_phosphatase_activity	region1inRegion2	20422
CNVR84	972	7	58579811	58584542	gain	6351	36993248	99614552	Alkaline_phosphatase_activity	region1inRegion2	4731
CNVR85	975	7	59278633	59284219	loss	6351	36993248	99614552	Alkaline_phosphatase_activity	region1inRegion2	5586
CNVR86	977	7	61355441	61417944	gain	6351	36993248	99614552	Alkaline_phosphatase_activity	region1inRegion2	62503
CNVR87	994	7	82234623	82374446	gain	6351	36993248	99614552	Alkaline_phosphatase_activity	region1inRegion2	139823
CNVR88	1002	7	84728780	84749202	gain	6351	36993248	99614552	Alkaline_phosphatase_activity	region1inRegion2	20422
CNVR84	972	7	58579811	58584542	gain	6371	36993248	99614552	Creatinine_level	region1inRegion2	4731
CNVR85	975	7	59278633	59284219	loss	6371	36993248	99614552	Creatinine_level	region1inRegion2	5586
CNVR86	977	7	61355441	61417944	gain	6371	36993248	99614552	Creatinine_level	region1inRegion2	62503
CNVR87	994	7	82234623	82374446	gain	6371	36993248	99614552	Creatinine_level	region1inRegion2	139823
CNVR88	1002	7	84728780	84749202	gain	6371	36993248	99614552	Creatinine_level	region1inRegion2	20422
CNVR84	972	7	58579811	58584542	gain	6379	36993248	99614552	Potassium_level	region1inRegion2	4731
CNVR85	975	7	59278633	59284219	loss	6379	36993248	99614552	Potassium_level	region1inRegion2	5586
CNVR86	977	7	61355441	61417944	gain	6379	36993248	99614552	Potassium_level	region1inRegion2	62503
CNVR87	994	7	82234623	82374446	gain	6379	36993248	99614552	Potassium_level	region1inRegion2	139823
CNVR88	1002	7	84728780	84749202	gain	6379	36993248	99614552	Potassium_level	region1inRegion2	20422
CNVR84	972	7	58579811	58584542	gain	9623	38992356	91621050	C3c_concentration	region1inRegion2	4731
CNVR85	975	7	59278633	59284219	loss	9623	38992356	91621050	C3c_concentration	region1inRegion2	5586
CNVR86	977	7	61355441	61417944	gain	9623	38992356	91621050	C3c_concentration	region1inRegion2	62503
CNVR87	994	7	82234623	82374446	gain	9623	38992356	91621050	C3c_concentration	region1inRegion2	139823
CNVR88	1002	7	84728780	84749202	gain	9623	38992356	91621050	C3c_concentration	region1inRegion2	20422
CNVR84	972	7	58579811	58584542	gain	21243	39401638	96585560	Body_weight_(birth)	region1inRegion2	4731
CNVR85	975	7	59278633	59284219	loss	21243	39401638	96585560	Body_weight_(birth)	region1inRegion2	5586
CNVR86	977	7	61355441	61417944	gain	21243	39401638	96585560	Body_weight_(birth)	region1inRegion2	62503
CNVR87	994	7	82234623	82374446	gain	21243	39401638	96585560	Body_weight_(birth)	region1inRegion2	139823
CNVR88	1002	7	84728780	84749202	gain	21243	39401638	96585560	Body_weight_(birth)	region1inRegion2	20422
CNVR84	972	7	58579811	58584542	gain	390	40700868	77016692	Loin_muscle_depth	region1inRegion2	4731
CNVR85	975	7	59278633	59284219	loss	390	40700868	77016692	Loin_muscle_depth	region1inRegion2	5586
CNVR86	977	7	61355441	61417944	gain	390	40700868	77016692	Loin_muscle_depth	region1inRegion2	62503
CNVR84	972	7	58579811	58584542	gain	387	41694849	69157768	Shoulder_weight	region1inRegion2	4731
CNVR85	975	7	59278633	59284219	loss	387	41694849	69157768	Shoulder_weight	region1inRegion2	5586
CNVR86	977	7	61355441	61417944	gain	387	41694849	69157768	Shoulder_weight	region1inRegion2	62503
CNVR84	972	7	58579811	58584542	gain	5197	43007205	132385084	Body_weight_(birth)	region1inRegion2	4731
CNVR85	975	7	59278633	59284219	loss	5197	43007205	132385084	Body_weight_(birth)	region1inRegion2	5586
CNVR86	977	7	61355441	61417944	gain	5197	43007205	132385084	Body_weight_(birth)	region1inRegion2	62503
CNVR87	994	7	82234623	82374446	gain	5197	43007205	132385084	Body_weight_(birth)	region1inRegion2	139823
CNVR88	1002	7	84728780	84749202	gain	5197	43007205	132385084	Body_weight_(birth)	region1inRegion2	20422
CNVR89	1011	7	102790067	102823288	gain	5197	43007205	132385084	Body_weight_(birth)	region1inRegion2	33221
CNVR90	1021	7	111620166	111623056	loss	5197	43007205	132385084	Body_weight_(birth)	region1inRegion2	2890
CNVR91	1035	7	131449863	131460402	gain	5197	43007205	132385084	Body_weight_(birth)	region1inRegion2	10539
CNVR84	972	7	58579811	58584542	gain	7349	44979276	133444277	Backfat_weight	region1inRegion2	4731
CNVR85	975	7	59278633	59284219	loss	7349	44979276	133444277	Backfat_weight	region1inRegion2	5586
CNVR86	977	7	61355441	61417944	gain	7349	44979276	133444277	Backfat_weight	region1inRegion2	62503
CNVR87	994	7	82234623	82374446	gain	7349	44979276	133444277	Backfat_weight	region1inRegion2	139823
CNVR88	1002	7	84728780	84749202	gain	7349	44979276	133444277	Backfat_weight	region1inRegion2	20422
CNVR89	1011	7	102790067	102823288	gain	7349	44979276	133444277	Backfat_weight	region1inRegion2	33221
CNVR90	1021	7	111620166	111623056	loss	7349	44979276	133444277	Backfat_weight	region1inRegion2	2890
CNVR91	1035	7	131449863	131460402	gain	7349	44979276	133444277	Backfat_weight	region1inRegion2	10539
CNVR92	1040	7	132961208	132975832	loss	7349	44979276	133444277	Backfat_weight	region1inRegion2	14624
CNVR93	1041	7	132985953	133003168	loss	7349	44979276	133444277	Backfat_weight	region1inRegion2	17215
CNVR84	972	7	58579811	58584542	gain	21308	45053573	117744862	Lumbar_vertebra_number	region1inRegion2	4731
CNVR85	975	7	59278633	59284219	loss	21308	45053573	117744862	Lumbar_vertebra_number	region1inRegion2	5586
CNVR86	977	7	61355441	61417944	gain	21308	45053573	117744862	Lumbar_vertebra_number	region1inRegion2	62503
CNVR87	994	7	82234623	82374446	gain	21308	45053573	117744862	Lumbar_vertebra_number	region1inRegion2	139823
CNVR88	1002	7	84728780	84749202	gain	21308	45053573	117744862	Lumbar_vertebra_number	region1inRegion2	20422
CNVR89	1011	7	102790067	102823288	gain	21308	45053573	117744862	Lumbar_vertebra_number	region1inRegion2	33221
CNVR90	1021	7	111620166	111623056	loss	21308	45053573	117744862	Lumbar_vertebra_number	region1inRegion2	2890
CNVR84	972	7	58579811	58584542	gain	1075	45102432	63372652	Conductivity_24_hours_post-mortem	region1inRegion2	4731
CNVR85	975	7	59278633	59284219	loss	1075	45102432	63372652	Conductivity_24_hours_post-mortem	region1inRegion2	5586
CNVR86	977	7	61355441	61417944	gain	1075	45102432	63372652	Conductivity_24_hours_post-mortem	region1inRegion2	62503
CNVR84	972	7	58579811	58584542	gain	12767	45102432	63372652	Abdominal_fat_weight	region1inRegion2	4731
CNVR85	975	7	59278633	59284219	loss	12767	45102432	63372652	Abdominal_fat_weight	region1inRegion2	5586
CNVR86	977	7	61355441	61417944	gain	12767	45102432	63372652	Abdominal_fat_weight	region1inRegion2	62503
CNVR84	972	7	58579811	58584542	gain	12826	45102432	63372652	Adipocyte_diameter	region1inRegion2	4731
CNVR85	975	7	59278633	59284219	loss	12826	45102432	63372652	Adipocyte_diameter	region1inRegion2	5586
CNVR86	977	7	61355441	61417944	gain	12826	45102432	63372652	Adipocyte_diameter	region1inRegion2	62503
CNVR84	972	7	58579811	58584542	gain	12836	45102432	63372652	Adipocyte_diameter	region1inRegion2	4731
CNVR85	975	7	59278633	59284219	loss	12836	45102432	63372652	Adipocyte_diameter	region1inRegion2	5586
CNVR86	977	7	61355441	61417944	gain	12836	45102432	63372652	Adipocyte_diameter	region1inRegion2	62503
CNVR84	972	7	58579811	58584542	gain	12842	45102432	63372652	Adipocyte_diameter	region1inRegion2	4731
CNVR85	975	7	59278633	59284219	loss	12842	45102432	63372652	Adipocyte_diameter	region1inRegion2	5586
CNVR86	977	7	61355441	61417944	gain	12842	45102432	63372652	Adipocyte_diameter	region1inRegion2	62503
CNVR84	972	7	58579811	58584542	gain	2790	45102432	101176460	Side_fat_thickness	region1inRegion2	4731
CNVR85	975	7	59278633	59284219	loss	2790	45102432	101176460	Side_fat_thickness	region1inRegion2	5586
CNVR86	977	7	61355441	61417944	gain	2790	45102432	101176460	Side_fat_thickness	region1inRegion2	62503
CNVR87	994	7	82234623	82374446	gain	2790	45102432	101176460	Side_fat_thickness	region1inRegion2	139823
CNVR88	1002	7	84728780	84749202	gain	2790	45102432	101176460	Side_fat_thickness	region1inRegion2	20422
CNVR84	972	7	58579811	58584542	gain	3159	45102432	107293999	Loin_weight	region1inRegion2	4731
CNVR85	975	7	59278633	59284219	loss	3159	45102432	107293999	Loin_weight	region1inRegion2	5586
CNVR86	977	7	61355441	61417944	gain	3159	45102432	107293999	Loin_weight	region1inRegion2	62503
CNVR87	994	7	82234623	82374446	gain	3159	45102432	107293999	Loin_weight	region1inRegion2	139823
CNVR88	1002	7	84728780	84749202	gain	3159	45102432	107293999	Loin_weight	region1inRegion2	20422
CNVR89	1011	7	102790067	102823288	gain	3159	45102432	107293999	Loin_weight	region1inRegion2	33221
CNVR84	972	7	58579811	58584542	gain	7360	46852015	134088187	lean_thickness_between_3rd_and_4th_last_ribs	region1inRegion2	4731
CNVR85	975	7	59278633	59284219	loss	7360	46852015	134088187	lean_thickness_between_3rd_and_4th_last_ribs	region1inRegion2	5586
CNVR86	977	7	61355441	61417944	gain	7360	46852015	134088187	lean_thickness_between_3rd_and_4th_last_ribs	region1inRegion2	62503
CNVR87	994	7	82234623	82374446	gain	7360	46852015	134088187	lean_thickness_between_3rd_and_4th_last_ribs	region1inRegion2	139823
CNVR88	1002	7	84728780	84749202	gain	7360	46852015	134088187	lean_thickness_between_3rd_and_4th_last_ribs	region1inRegion2	20422
CNVR89	1011	7	102790067	102823288	gain	7360	46852015	134088187	lean_thickness_between_3rd_and_4th_last_ribs	region1inRegion2	33221
CNVR90	1021	7	111620166	111623056	loss	7360	46852015	134088187	lean_thickness_between_3rd_and_4th_last_ribs	region1inRegion2	2890
CNVR91	1035	7	131449863	131460402	gain	7360	46852015	134088187	lean_thickness_between_3rd_and_4th_last_ribs	region1inRegion2	10539
CNVR92	1040	7	132961208	132975832	loss	7360	46852015	134088187	lean_thickness_between_3rd_and_4th_last_ribs	region1inRegion2	14624
CNVR93	1041	7	132985953	133003168	loss	7360	46852015	134088187	lean_thickness_between_3rd_and_4th_last_ribs	region1inRegion2	17215
CNVR84	972	7	58579811	58584542	gain	2989	48448805	99614552	Muscle_moisture_percentage	region1inRegion2	4731
CNVR85	975	7	59278633	59284219	loss	2989	48448805	99614552	Muscle_moisture_percentage	region1inRegion2	5586
CNVR86	977	7	61355441	61417944	gain	2989	48448805	99614552	Muscle_moisture_percentage	region1inRegion2	62503
CNVR87	994	7	82234623	82374446	gain	2989	48448805	99614552	Muscle_moisture_percentage	region1inRegion2	139823
CNVR88	1002	7	84728780	84749202	gain	2989	48448805	99614552	Muscle_moisture_percentage	region1inRegion2	20422
CNVR84	972	7	58579811	58584542	gain	16927	50310118	99225754	Loin_muscle_area	region1inRegion2	4731

CNVR85	975	7	59278633	59284219	loss	16927	50310118	99225754	Loin_muscle_area	region1inRegion2	5586
CNVR86	977	7	61355441	61417944	gain	16927	50310118	99225754	Loin_muscle_area	region1inRegion2	62503
CNVR87	994	7	82234623	82374446	gain	16927	50310118	99225754	Loin_muscle_area	region1inRegion2	139823
CNVR88	1002	7	84728780	84749202	gain	16927	50310118	99225754	Loin_muscle_area	region1inRegion2	20422
CNVR84	972	7	58579811	58584542	gain	21388	50310118	99225754	Marbling	region1inRegion2	4731
CNVR85	975	7	59278633	59284219	loss	21388	50310118	99225754	Marbling	region1inRegion2	5586
CNVR86	977	7	61355441	61417944	gain	21388	50310118	99225754	Marbling	region1inRegion2	62503
CNVR87	994	7	82234623	82374446	gain	21388	50310118	99225754	Marbling	region1inRegion2	139823
CNVR88	1002	7	84728780	84749202	gain	21388	50310118	99225754	Marbling	region1inRegion2	20422
CNVR84	972	7	58579811	58584542	gain	21389	50310118	99225754	Ham_weight	region1inRegion2	4731
CNVR85	975	7	59278633	59284219	loss	21389	50310118	99225754	Ham_weight	region1inRegion2	5586
CNVR86	977	7	61355441	61417944	gain	21389	50310118	99225754	Ham_weight	region1inRegion2	62503
CNVR87	994	7	82234623	82374446	gain	21389	50310118	99225754	Ham_weight	region1inRegion2	139823
CNVR88	1002	7	84728780	84749202	gain	21389	50310118	99225754	Ham_weight	region1inRegion2	20422
CNVR84	972	7	58579811	58584542	gain	21390	50310118	99225754	Loin_weight	region1inRegion2	4731
CNVR85	975	7	59278633	59284219	loss	21390	50310118	99225754	Loin_weight	region1inRegion2	5586
CNVR86	977	7	61355441	61417944	gain	21390	50310118	99225754	Loin_weight	region1inRegion2	62503
CNVR87	994	7	82234623	82374446	gain	21390	50310118	99225754	Loin_weight	region1inRegion2	139823
CNVR88	1002	7	84728780	84749202	gain	21390	50310118	99225754	Loin_weight	region1inRegion2	20422
CNVR84	972	7	58579811	58584542	gain	3256	50310118	101176460	Loin_muscle_area	region1inRegion2	4731
CNVR85	975	7	59278633	59284219	loss	3256	50310118	101176460	Loin_muscle_area	region1inRegion2	5586
CNVR86	977	7	61355441	61417944	gain	3256	50310118	101176460	Loin_muscle_area	region1inRegion2	62503
CNVR87	994	7	82234623	82374446	gain	3256	50310118	101176460	Loin_muscle_area	region1inRegion2	139823
CNVR88	1002	7	84728780	84749202	gain	3256	50310118	101176460	Loin_muscle_area	region1inRegion2	20422
CNVR84	972	7	58579811	58584542	gain	3257	50310118	101176460	Number_of_ribs	region1inRegion2	4731
CNVR85	975	7	59278633	59284219	loss	3257	50310118	101176460	Number_of_ribs	region1inRegion2	5586
CNVR86	977	7	61355441	61417944	gain	3257	50310118	101176460	Number_of_ribs	region1inRegion2	62503
CNVR87	994	7	82234623	82374446	gain	3257	50310118	101176460	Number_of_ribs	region1inRegion2	139823
CNVR88	1002	7	84728780	84749202	gain	3257	50310118	101176460	Number_of_ribs	region1inRegion2	20422
CNVR84	972	7	58579811	58584542	gain	3258	50310118	101176460	Spareribs_weight	region1inRegion2	4731
CNVR85	975	7	59278633	59284219	loss	3258	50310118	101176460	Spareribs_weight	region1inRegion2	5586
CNVR86	977	7	61355441	61417944	gain	3258	50310118	101176460	Spareribs_weight	region1inRegion2	62503
CNVR87	994	7	82234623	82374446	gain	3258	50310118	101176460	Spareribs_weight	region1inRegion2	139823
CNVR88	1002	7	84728780	84749202	gain	3258	50310118	101176460	Spareribs_weight	region1inRegion2	20422
CNVR84	972	7	58579811	58584542	gain	302	50310118	102304856	Hematin_pigmentation	region1inRegion2	4731
CNVR85	975	7	59278633	59284219	loss	302	50310118	102304856	Hematin_pigmentation	region1inRegion2	5586
CNVR86	977	7	61355441	61417944	gain	302	50310118	102304856	Hematin_pigmentation	region1inRegion2	62503
CNVR87	994	7	82234623	82374446	gain	302	50310118	102304856	Hematin_pigmentation	region1inRegion2	139823
CNVR88	1002	7	84728780	84749202	gain	302	50310118	102304856	Hematin_pigmentation	region1inRegion2	20422
CNVR84	972	7	58579811	58584542	gain	648	50310118	102304856	Average_daily_gain	region1inRegion2	4731
CNVR85	975	7	59278633	59284219	loss	648	50310118	102304856	Average_daily_gain	region1inRegion2	5586
CNVR86	977	7	61355441	61417944	gain	648	50310118	102304856	Average_daily_gain	region1inRegion2	62503
CNVR87	994	7	82234623	82374446	gain	648	50310118	102304856	Average_daily_gain	region1inRegion2	139823
CNVR88	1002	7	84728780	84749202	gain	648	50310118	102304856	Average_daily_gain	region1inRegion2	20422
CNVR84	972	7	58579811	58584542	gain	61	50310118	120698338	Backfat_at_tenth_rib	region1inRegion2	4731
CNVR85	975	7	59278633	59284219	loss	61	50310118	120698338	Backfat_at_tenth_rib	region1inRegion2	5586
CNVR86	977	7	61355441	61417944	gain	61	50310118	120698338	Backfat_at_tenth_rib	region1inRegion2	62503
CNVR87	994	7	82234623	82374446	gain	61	50310118	120698338	Backfat_at_tenth_rib	region1inRegion2	139823
CNVR88	1002	7	84728780	84749202	gain	61	50310118	120698338	Backfat_at_tenth_rib	region1inRegion2	20422
CNVR89	1011	7	102790067	102823288	gain	61	50310118	120698338	Backfat_at_tenth_rib	region1inRegion2	33221
CNVR90	1021	7	111620166	111623056	loss	61	50310118	120698338	Backfat_at_tenth_rib	region1inRegion2	2890
CNVR84	972	7	58579811	58584542	gain	613	52835025	134608551	conA-induced_cell_proliferation	region1inRegion2	4731
CNVR85	975	7	59278633	59284219	loss	613	52835025	134608551	conA-induced_cell_proliferation	region1inRegion2	5586
CNVR86	977	7	61355441	61417944	gain	613	52835025	134608551	conA-induced_cell_proliferation	region1inRegion2	62503
CNVR87	994	7	82234623	82374446	gain	613	52835025	134608551	conA-induced_cell_proliferation	region1inRegion2	139823
CNVR88	1002	7	84728780	84749202	gain	613	52835025	134608551	conA-induced_cell_proliferation	region1inRegion2	20422
CNVR89	1011	7	102790067	102823288	gain	613	52835025	134608551	conA-induced_cell_proliferation	region1inRegion2	33221
CNVR90	1021	7	111620166	111623056	loss	613	52835025	134608551	conA-induced_cell_proliferation	region1inRegion2	2890
CNVR91	1035	7	131449863	131460402	gain	613	52835025	134608551	conA-induced_cell_proliferation	region1inRegion2	10539
CNVR92	1040	7	132961208	132975832	loss	613	52835025	134608551	conA-induced_cell_proliferation	region1inRegion2	14624
CNVR93	1041	7	132985953	133003168	loss	613	52835025	134608551	conA-induced_cell_proliferation	region1inRegion2	17215
CNVR84	972	7	58579811	58584542	gain	7352	52835025	134608551	Average_daily_gain	region1inRegion2	4731
CNVR85	975	7	59278633	59284219	loss	7352	52835025	134608551	Average_daily_gain	region1inRegion2	5586
CNVR86	977	7	61355441	61417944	gain	7352	52835025	134608551	Average_daily_gain	region1inRegion2	62503
CNVR87	994	7	82234623	82374446	gain	7352	52835025	134608551	Average_daily_gain	region1inRegion2	139823
CNVR88	1002	7	84728780	84749202	gain	7352	52835025	134608551	Average_daily_gain	region1inRegion2	20422
CNVR89	1011	7	102790067	102823288	gain	7352	52835025	134608551	Average_daily_gain	region1inRegion2	33221
CNVR90	1021	7	111620166	111623056	loss	7352	52835025	134608551	Average_daily_gain	region1inRegion2	2890
CNVR91	1035	7	131449863	131460402	gain	7352	52835025	134608551	Average_daily_gain	region1inRegion2	10539
CNVR92	1040	7	132961208	132975832	loss	7352	52835025	134608551	Average_daily_gain	region1inRegion2	14624
CNVR93	1041	7	132985953	133003168	loss	7352	52835025	134608551	Average_daily_gain	region1inRegion2	17215
CNVR84	972	7	58579811	58584542	gain	2947	55731128	99225754	backfat_at_last_rib	region1inRegion2	4731
CNVR85	975	7	59278633	59284219	loss	2947	55731128	99225754	backfat_at_last_rib	region1inRegion2	5586
CNVR86	977	7	61355441	61417944	gain	2947	55731128	99225754	backfat_at_last_rib	region1inRegion2	62503
CNVR87	994	7	82234623	82374446	gain	2947	55731128	99225754	backfat_at_last_rib	region1inRegion2	139823
CNVR88	1002	7	84728780	84749202	gain	2947	55731128	99225754	backfat_at_last_rib	region1inRegion2	20422
CNVR84	972	7	58579811	58584542	gain	2948	55731128	99225754	Average_backfat_thickness	region1inRegion2	4731
CNVR85	975	7	59278633	59284219	loss	2948	55731128	99225754	Average_backfat_thickness	region1inRegion2	5586
CNVR86	977	7	61355441	61417944	gain	2948	55731128	99225754	Average_backfat_thickness	region1inRegion2	62503
CNVR87	994	7	82234623	82374446	gain	2948	55731128	99225754	Average_backfat_thickness	region1inRegion2	139823
CNVR88	1002	7	84728780	84749202	gain	2948	55731128	99225754	Average_backfat_thickness	region1inRegion2	20422
CNVR84	972	7	58579811	58584542	gain	17600	55880470	101176460	Interleukin_2_level	region1inRegion2	4731
CNVR85	975	7	59278633	59284219	loss	17600	55880470	101176460	Interleukin_2_level	region1inRegion2	5586
CNVR86	977	7	61355441	61417944	gain	17600	55880470	101176460	Interleukin_2_level	region1inRegion2	62503
CNVR87	994	7	82234623	82374446	gain	17600	55880470	101176460	Interleukin_2_level	region1inRegion2	139823
CNVR88	1002	7	84728780	84749202	gain	17600	55880470	101176460	Interleukin_2_level	region1inRegion2	20422
CNVR84	972	7	58579811	58584542	gain	17720	55880470	101176460	Cholesterol_level	region1inRegion2	4731
CNVR85	975	7	59278633	59284219	loss	17720	55880470	101176460	Cholesterol_level	region1inRegion2	5586
CNVR86	977	7	61355441	61417944	gain	17720	55880470	101176460	Cholesterol_level	region1inRegion2	62503
CNVR87	994	7	82234623	82374446	gain	17720	55880470	101176460	Cholesterol_level	region1inRegion2	139823
CNVR88	1002	7	84728780	84749202	gain	17720	55880470	101176460	Cholesterol_level	region1inRegion2	20422
CNVR84	972	7	58579811	58584542	gain	17721	55880470	101176460	LDL_cholesterol	region1inRegion2	4731
CNVR85	975	7	59278633	59284219	loss	17721	55880470	101176460	LDL_cholesterol	region1inRegion2	5586
CNVR86	977	7	61355441	61417944	gain	17721	55880470	101176460	LDL_cholesterol	region1inRegion2	62503
CNVR87	994	7	82234623	82374446	gain	17721	55880470	101176460	LDL_cholesterol	region1inRegion2	139823
CNVR88	1002	7	84728780	84749202	gain	17721	55880470	101176460	LDL_cholesterol	region1inRegion2	20422
CNVR84	972	7	58579811	58584542	gain	5701	55880470	101176460	Backfat_at_tenth_rib	region1inRegion2	4731
CNVR85	975	7	59278633	59284219	loss	5701	55880470	101176460	Backfat_at_tenth_rib	region1inRegion2	5586
CNVR86	977	7	61355441	61417944	gain	5701	55880470	101176460	Backfat_at_tenth_rib	region1inRegion2	62503
CNVR87	994	7	82234623	82374446	gain	5701	55880470	101176460	Backfat_at_tenth_rib	region1inRegion2	139823
CNVR88	1002	7	84728780	84749202	gain	5701	55880470	101176460	Backfat_at_tenth_rib	region1inRegion2	20422
CNVR84	972	7	58579811	58584542	gain	5989	55880470	101176460	Dressing_percentage	region1inRegion2	4731
CNVR85	975	7	59278633	59284219	loss	5989	55880470	101176460	Dressing_percentage	region1inRegion2	5586
CNVR86	977	7	61355441	61417944	gain	5989	55880470	101176460	Dressing_percentage	region1inRegion2	62503
CNVR87	994	7	82234623	82374446	gain	5989	55880470	101176460	Dressing_percentage	region1inRegion2	139823
CNVR88	1002	7	84728780	84749202	gain	5989	55880470	101176460	Dressing_percentage	region1inRegion2	20422
CNVR84	972	7	58579811	58584542	gain	9661	55880470	101176460	PRRSV_antibody_titer	region1inRegion2	4731
CNVR85	975	7	59278633	59284219	loss	9661	55880470	101176460	PRRSV_antibody_titer	region1inRegion2	5586
CNVR86	977	7	61355441	61417944	gain	9661	55880470	101176460	PRRSV_antibody_titer	region1inRegion2	62503
CNVR87	994	7	82234623	82374446	gain	9661	55880470	101176460	PRRSV_antibody_titer	region1inRegion2	139823
CNVR88	1002	7	84728780	84749202	gain	9661	55880470	101176460	PRRSV_antibody_titer	region1inRegion2	20422
CNVR84	972	7	58579811	58584542	gain	9853	55880470	101176460	Drip_loss	region1inRegion2	4731
CNVR85	975	7	59278633	59284219	loss	9853	55880470	101176460	Drip_loss	region1inRegion2	5586
CNVR86	977	7	61355441	61417944	gain	9853	55880470	101176460	Drip_loss	region1inRegion2	62503
CNVR87	994	7	82234623	82374446	gain	9853	55880470	101176460	Drip_loss	region1inRegion2	139823
CNVR88	1002	7	84728780	84749202	gain	9853	55880470	101176460	Drip_loss	region1inRegion2	20422
CNVR84	972	7	58579811	58584542	gain	9854	55880470	101176460	pH_24_hr_post-mortem_(loin)	region1inRegion2	4731
CNVR85	975	7	59278633	59284219	loss	9854	55880470	101176460	pH_24_hr_post-mortem_(loin)	region1inRegion2	5586
CNVR86	977	7	61355441	61417944	gain	9854	55880470	101176460	pH_24_hr_post-mortem_(loin)	region1inRegion2	62503
CNVR87	994	7	82234623	82374446	gain	9854	55880470	101176460	pH_24_hr_post-mortem_(loin)	region1inRegion2	139823
CNVR88	1002	7	84728780	84749202	gain	9854	55880470	101176460	pH_24_hr_post-mortem_(loin)	region1inRegion2	20422
CNVR84	972	7	58579811	58584542	gain	3994	55880470	107293999	CIE-b*	region1inRegion2	4731

CNVR85	975	7	59278633	59284219	loss	3994	55880470	107293999	CIE-b*	region1inRegion2	5586
CNVR86	977	7	61355441	61417944	gain	3994	55880470	107293999	CIE-b*	region1inRegion2	62503
CNVR87	994	7	82234623	82374446	gain	3994	55880470	107293999	CIE-b*	region1inRegion2	139823
CNVR88	1002	7	84728780	84749202	gain	3994	55880470	107293999	CIE-b*	region1inRegion2	20422
CNVR89	1011	7	102790067	102823288	gain	3994	55880470	107293999	CIE-b*	region1inRegion2	33221
CNVR84	972	7	58579811	58584542	gain	3996	55880470	107293999	Shear_force	region1inRegion2	4731
CNVR85	975	7	59278633	59284219	loss	3996	55880470	107293999	Shear_force	region1inRegion2	5586
CNVR86	977	7	61355441	61417944	gain	3996	55880470	107293999	Shear_force	region1inRegion2	62503
CNVR87	994	7	82234623	82374446	gain	3996	55880470	107293999	Shear_force	region1inRegion2	139823
CNVR88	1002	7	84728780	84749202	gain	3996	55880470	107293999	Shear_force	region1inRegion2	20422
CNVR89	1011	7	102790067	102823288	gain	3996	55880470	107293999	Shear_force	region1inRegion2	33221
CNVR84	972	7	58579811	58584542	gain	3998	55880470	107293999	Total_shear_work	region1inRegion2	4731
CNVR85	975	7	59278633	59284219	loss	3998	55880470	107293999	Total_shear_work	region1inRegion2	5586
CNVR86	977	7	61355441	61417944	gain	3998	55880470	107293999	Total_shear_work	region1inRegion2	62503
CNVR87	994	7	82234623	82374446	gain	3998	55880470	107293999	Total_shear_work	region1inRegion2	139823
CNVR88	1002	7	84728780	84749202	gain	3998	55880470	107293999	Total_shear_work	region1inRegion2	20422
CNVR89	1011	7	102790067	102823288	gain	3998	55880470	107293999	Total_shear_work	region1inRegion2	33221
CNVR84	972	7	58579811	58584542	gain	22315	58385473	59342923	Loin_muscle_area	region1inRegion2	4731
CNVR85	975	7	59278633	59284219	loss	22315	58385473	59342923	Loin_muscle_area	region1inRegion2	5586
CNVR86	977	7	61355441	61417944	gain	1260	59947461	64274644	Head_weight	region1inRegion2	62503
CNVR86	977	7	61355441	61417944	gain	277	60255640	99614552	Teat_number	region1inRegion2	62503
CNVR87	994	7	82234623	82374446	gain	277	60255640	99614552	Teat_number	region1inRegion2	139823
CNVR88	1002	7	84728780	84749202	gain	277	60255640	99614552	Teat_number	region1inRegion2	20422
CNVR87	994	7	82234623	82374446	gain	1065	64962096	101176460	pH_24_hr_post-mortem_(loin)	region1inRegion2	139823
CNVR88	1002	7	84728780	84749202	gain	1065	64962096	101176460	pH_24_hr_post-mortem_(loin)	region1inRegion2	20422
CNVR87	994	7	82234623	82374446	gain	12822	64962096	101176460	Adipocyte_diameter	region1inRegion2	139823
CNVR88	1002	7	84728780	84749202	gain	12822	64962096	101176460	Adipocyte_diameter	region1inRegion2	20422
CNVR87	994	7	82234623	82374446	gain	12823	64962096	101176460	Adipocyte_diameter	region1inRegion2	139823
CNVR88	1002	7	84728780	84749202	gain	12823	64962096	101176460	Adipocyte_diameter	region1inRegion2	20422
CNVR87	994	7	82234623	82374446	gain	12825	64962096	101176460	Adipocyte_diameter	region1inRegion2	139823
CNVR88	1002	7	84728780	84749202	gain	12825	64962096	101176460	Adipocyte_diameter	region1inRegion2	20422
CNVR87	994	7	82234623	82374446	gain	12837	64962096	101176460	Adipocyte_diameter	region1inRegion2	139823
CNVR88	1002	7	84728780	84749202	gain	12837	64962096	101176460	Adipocyte_diameter	region1inRegion2	20422
CNVR87	994	7	82234623	82374446	gain	12841	64962096	101176460	Adipocyte_diameter	region1inRegion2	139823
CNVR88	1002	7	84728780	84749202	gain	12841	64962096	101176460	Adipocyte_diameter	region1inRegion2	20422
CNVR87	994	7	82234623	82374446	gain	18024	64962096	101176460	Adipocyte_diameter	region1inRegion2	139823
CNVR88	1002	7	84728780	84749202	gain	18024	64962096	101176460	Adipocyte_diameter	region1inRegion2	20422
CNVR87	994	7	82234623	82374446	gain	5656	64962096	107293999	Ear_erectness	region1inRegion2	139823
CNVR88	1002	7	84728780	84749202	gain	5656	64962096	107293999	Ear_erectness	region1inRegion2	20422
CNVR89	1011	7	102790067	102823288	gain	5656	64962096	107293999	Ear_erectness	region1inRegion2	33221
CNVR87	994	7	82234623	82374446	gain	649	64962096	120698338	Carcass_length	region1inRegion2	139823
CNVR88	1002	7	84728780	84749202	gain	649	64962096	120698338	Carcass_length	region1inRegion2	20422
CNVR89	1011	7	102790067	102823288	gain	649	64962096	120698338	Carcass_length	region1inRegion2	33221
CNVR90	1021	7	111620166	111623056	loss	649	64962096	120698338	Carcass_length	region1inRegion2	2890
CNVR87	994	7	82234623	82374446	gain	650	64962096	120698338	Belly_percentage	region1inRegion2	139823
CNVR88	1002	7	84728780	84749202	gain	650	64962096	120698338	Belly_percentage	region1inRegion2	20422
CNVR89	1011	7	102790067	102823288	gain	650	64962096	120698338	Belly_percentage	region1inRegion2	33221
CNVR90	1021	7	111620166	111623056	loss	650	64962096	120698338	Belly_percentage	region1inRegion2	2890
CNVR87	994	7	82234623	82374446	gain	12265	65856079	99614552	Interferon-gamma_level	region1inRegion2	139823
CNVR88	1002	7	84728780	84749202	gain	12265	65856079	99614552	Interferon-gamma_level	region1inRegion2	20422
CNVR87	994	7	82234623	82374446	gain	385	65950585	107791845	Loin_weight	region1inRegion2	139823
CNVR88	1002	7	84728780	84749202	gain	385	65950585	107791845	Loin_weight	region1inRegion2	20422
CNVR89	1011	7	102790067	102823288	gain	385	65950585	107791845	Loin_weight	region1inRegion2	33221
CNVR87	994	7	82234623	82374446	gain	21713	71953904	100046036	Carcass_length	region1inRegion2	139823
CNVR88	1002	7	84728780	84749202	gain	21713	71953904	100046036	Carcass_length	region1inRegion2	20422
CNVR87	994	7	82234623	82374446	gain	21714	71953904	100046036	Loin_muscle_area	region1inRegion2	139823
CNVR88	1002	7	84728780	84749202	gain	21714	71953904	100046036	Loin_muscle_area	region1inRegion2	20422
CNVR87	994	7	82234623	82374446	gain	7357	74791577	86101177	Backfat_at_rump	region1inRegion2	139823
CNVR88	1002	7	84728780	84749202	gain	7357	74791577	86101177	Backfat_at_rump	region1inRegion2	20422
CNVR87	994	7	82234623	82374446	gain	768	80737961	87879272	Skin_weight	region1inRegion2	139823
CNVR88	1002	7	84728780	84749202	gain	768	80737961	87879272	Skin_weight	region1inRegion2	20422
CNVR87	994	7	82234623	82374446	gain	8754	80737961	89472433	Umbilical_hernia	region1inRegion2	139823
CNVR88	1002	7	84728780	84749202	gain	8754	80737961	89472433	Umbilical_hernia	region1inRegion2	20422
CNVR87	994	7	82234623	82374446	gain	37446	81209086	85125860	Teat_number	region1inRegion2	139823
CNVR88	1002	7	84728780	84749202	gain	37446	81209086	85125860	Teat_number	region1inRegion2	20422
CNVR88	1002	7	84728780	84749202	gain	106282	84047647	85068543	Monounsaturated_fatty_acid_content	region1inRegion2	20422
CNVR88	1002	7	84728780	84749202	gain	106283	84047647	85068543	Oleic_acid_content	region1inRegion2	20422
CNVR89	1011	7	102790067	102823288	gain	8804	88946228	107293999	Teat_number	region1inRegion2	33221
CNVR89	1011	7	102790067	102823288	gain	8805	88946228	107293999	Teat_number	region1inRegion2	33221
CNVR89	1011	7	102790067	102823288	gain	3791	99225754	106839376	Meat_color-L	region1inRegion2	33221
CNVR89	1011	7	102790067	102823288	gain	4251	99225754	106839376	Teat_number	region1inRegion2	33221
CNVR89	1011	7	102790067	102823288	gain	18631	99614552	106839376	Fat_area_percentage_in_carcass	region1inRegion2	33221
CNVR89	1011	7	102790067	102823288	gain	2990	99614552	107293999	Lean_meat_percentage	region1inRegion2	33221
CNVR89	1011	7	102790067	102823288	gain	16929	101176460	107293999	Loin_muscle_area	region1inRegion2	33221
CNVR89	1011	7	102790067	102823288	gain	21391	101176460	107293999	Number_of_ribs	region1inRegion2	33221
CNVR89	1011	7	102790067	102823288	gain	3205	101176460	112604047	Average_daily_gain	region1inRegion2	33221
CNVR90	1021	7	111620166	111623056	loss	3205	101176460	112604047	Average_daily_gain	region1inRegion2	2890
CNVR89	1011	7	102790067	102823288	gain	356	101176460	118398897	Number_of_white_fibers	region1inRegion2	33221
CNVR90	1021	7	111620166	111623056	loss	356	101176460	118398897	Number_of_white_fibers	region1inRegion2	2890
CNVR89	1011	7	102790067	102823288	gain	357	101176460	118398897	Percentage_type_IIb_fibers	region1inRegion2	33221
CNVR90	1021	7	111620166	111623056	loss	357	101176460	118398897	Percentage_type_IIb_fibers	region1inRegion2	2890
CNVR89	1011	7	102790067	102823288	gain	358	101176460	118398897	Diameter_of_type_IIb_muscle_fibers	region1inRegion2	33221
CNVR90	1021	7	111620166	111623056	loss	358	101176460	118398897	Diameter_of_type_IIb_muscle_fibers	region1inRegion2	2890
CNVR89	1011	7	102790067	102823288	gain	359	101176460	118398897	R-value	region1inRegion2	33221
CNVR90	1021	7	111620166	111623056	loss	359	101176460	118398897	R-value	region1inRegion2	2890
CNVR89	1011	7	102790067	102823288	gain	360	101176460	118398897	Average_lactate	region1inRegion2	33221
CNVR90	1021	7	111620166	111623056	loss	360	101176460	118398897	Average_lactate	region1inRegion2	2890
CNVR89	1011	7	102790067	102823288	gain	361	101176460	118398897	Average_glycolytic_potential	region1inRegion2	33221
CNVR90	1021	7	111620166	111623056	loss	361	101176460	118398897	Average_glycolytic_potential	region1inRegion2	2890
CNVR89	1011	7	102790067	102823288	gain	362	101176460	118398897	R-value_/_diameter_of_white_fibers	region1inRegion2	33221
CNVR90	1021	7	111620166	111623056	loss	362	101176460	118398897	R-value_/_diameter_of_white_fibers	region1inRegion2	2890
CNVR89	1011	7	102790067	102823288	gain	363	101176460	118398897	Lactate_/_diameter_of_white_fibers	region1inRegion2	33221
CNVR90	1021	7	111620166	111623056	loss	363	101176460	118398897	Lactate_/_diameter_of_white_fibers	region1inRegion2	2890
CNVR89	1011	7	102790067	102823288	gain	63	101176460	118398897	Carcass_weight_(cold)	region1inRegion2	33221
CNVR90	1021	7	111620166	111623056	loss	63	101176460	118398897	Carcass_weight_(cold)	region1inRegion2	2890
CNVR89	1011	7	102790067	102823288	gain	2812	101176460	120698338	Diameter_of_type_I_muscle_fibers	region1inRegion2	33221
CNVR90	1021	7	111620166	111623056	loss	2812	101176460	120698338	Diameter_of_type_I_muscle_fibers	region1inRegion2	2890
CNVR89	1011	7	102790067	102823288	gain	5988	101176460	120698338	Subcutanous_fat_area	region1inRegion2	33221
CNVR90	1021	7	111620166	111623056	loss	5988	101176460	120698338	Subcutanous_fat_area	region1inRegion2	2890
CNVR89	1011	7	102790067	102823288	gain	9624	101176460	120698338	C3c_concentration	region1inRegion2	33221
CNVR90	1021	7	111620166	111623056	loss	9624	101176460	120698338	C3c_concentration	region1inRegion2	2890
CNVR89	1011	7	102790067	102823288	gain	3881	101176460	128266828	Leaf_fat_weight	region1inRegion2	33221
CNVR90	1021	7	111620166	111623056	loss	3881	101176460	128266828	Leaf_fat_weight	region1inRegion2	2890
CNVR89	1011	7	102790067	102823288	gain	5500	102304856	118398897	Lean_meat_weight	region1inRegion2	33221
CNVR90	1021	7	111620166	111623056	loss	5500	102304856	118398897	Lean_meat_weight	region1inRegion2	2890
CNVR90	1021	7	111620166	111623056	loss	2915	104023972	114859873	Average_backfat_thickness	region1inRegion2	2890
CNVR90	1021	7	111620166	111623056	loss	3036	104023972	114859873	Average_backfat_thickness	region1inRegion2	2890
CNVR90	1021	7	111620166	111623056	loss	727	104023972	114859873	Leaf_fat_weight	region1inRegion2	2890
CNVR90	1021	7	111620166	111623056	loss	763	104023972	114859873	Loin_muscle_area	region1inRegion2	2890
CNVR90	1021	7	111620166	111623056	loss	5403	105380038	114697922	Red_blood_cell_count	region1inRegion2	2890
CNVR90	1021	7	111620166	111623056	loss	5407	105380038	114697922	Red_blood_cell_count	region1inRegion2	2890
CNVR90	1021	7	111620166	111623056	loss	5609	105380038	114697922	Vertebra_number	region1inRegion2	2890
CNVR90	1021	7	111620166	111623056	loss	8755	105380038	114697922	Umbilical_hernia	region1inRegion2	2890
CNVR90	1021	7	111620166	111623056	loss	3096	106839376	112604047	Intramuscular_fat_content	region1inRegion2	2890
CNVR90	1021	7	111620166	111623056	loss	703	106839376	112604047	Longissimus_muscle_length	region1inRegion2	2890
CNVR90	1021	7	111620166	111623056	loss	2946	106839376	120698338	Backfat_at_last_lumbar	region1inRegion2	2890
CNVR90	1021	7	111620166	111623056	loss	2949	106839376	120698338	Average_backfat_thickness	region1inRegion2	2890
CNVR90	1021	7	111620166	111623056	loss	8889	106839376	120698338	Ear_area	region1inRegion2	2890
CNVR90	1021	7	111620166	111623056	loss	8890	106839376	120698338	Ear_area	region1inRegion2	2890
CNVR90	1021	7	111620166	111623056	loss	10618	107293999	130259585	uterine_horn_length	region1inRegion2	2890
CNVR90	1021	7	111620166	111623056	loss	15072	107293999	130259585	Potassium_level	region1inRegion2	2890
CNVR90	1021	7	111620166	111623056	loss	482	107293999	130259585	Cortisol_level	region1inRegion2	2890

CNVR90	1021	7	111620166	111623056	loss	483	107293999	130259585	Cortisol_level	region1inRegion2	2890
CNVR90	1021	7	111620166	111623056	loss	139175	111423873	112412287	Lean_meat_percentage	region1inRegion2	2890
CNVR91	1035	7	131449863	131460402	gain	18018	126520300	134764511	NADP-malate_dehydrogenase_activity	region1inRegion2	10539
CNVR92	1040	7	132961208	132975832	loss	18018	126520300	134764511	NADP-malate_dehydrogenase_activity	region1inRegion2	14624
CNVR93	1041	7	132985953	133003168	loss	18018	126520300	134764511	NADP-malate_dehydrogenase_activity	region1inRegion2	17215
CNVR91	1035	7	131449863	131460402	gain	17879	128399007	132430483	Lean_meat_percentage	region1inRegion2	10539
CNVR91	1035	7	131449863	131460402	gain	17880	128399007	132430483	Meat_to_fat_ratio	region1inRegion2	10539
CNVR91	1035	7	131449863	131460402	gain	17881	128399007	132430483	Shoulder_subcutaneous_fat_thickness	region1inRegion2	10539
CNVR91	1035	7	131449863	131460402	gain	17882	128399007	132430483	Backfat_between_6th_and_7th_ribs	region1inRegion2	10539
CNVR91	1035	7	131449863	131460402	gain	17883	128399007	132430483	Backfat_at_rump	region1inRegion2	10539
CNVR91	1035	7	131449863	131460402	gain	17884	128399007	132430483	Loin_muscle_area	region1inRegion2	10539
CNVR91	1035	7	131449863	131460402	gain	17885	128399007	132430483	Drip_loss	region1inRegion2	10539
CNVR91	1035	7	131449863	131460402	gain	17886	128399007	132430483	Water_holding_capacity	region1inRegion2	10539
CNVR91	1035	7	131449863	131460402	gain	17887	128399007	132430483	Meat_color_score	region1inRegion2	10539
CNVR91	1035	7	131449863	131460402	gain	17888	128399007	132430483	Intramuscular_fat_content	region1inRegion2	10539
CNVR91	1035	7	131449863	131460402	gain	17889	128399007	132430483	Muscle_moisture_percentage	region1inRegion2	10539
CNVR94	1059	8	26029928	26034839	loss	1098	811090	26534249	Conductivity_45_minutes_post-mortem	region1inRegion2	4911
CNVR94	1059	8	26029928	26034839	loss	5921	811090	46427317	Time_spent_walking	region1inRegion2	4911
CNVR95	1068	8	30016900	30024587	loss	5921	811090	46427317	Time_spent_walking	region1inRegion2	7687
CNVR96	1086	8	44371710	44447879	gain	5921	811090	46427317	Time_spent_walking	region1inRegion2	76169
CNVR94	1059	8	26029928	26034839	loss	9645	811090	77429212	Haptoglobin_concentration	region1inRegion2	4911
CNVR95	1068	8	30016900	30024587	loss	9645	811090	77429212	Haptoglobin_concentration	region1inRegion2	7687
CNVR96	1086	8	44371710	44447879	gain	9645	811090	77429212	Haptoglobin_concentration	region1inRegion2	76169
CNVR97	1112	8	64473379	64515977	loss	9645	811090	77429212	Haptoglobin_concentration	region1inRegion2	42598
CNVR98	1116	8	68684763	68710413	gain	9645	811090	77429212	Haptoglobin_concentration	region1inRegion2	25650
CNVR94	1059	8	26029928	26034839	loss	38096	811090	108610930	Muscle_moisture_percentage	region1inRegion2	4911
CNVR95	1068	8	30016900	30024587	loss	38096	811090	108610930	Muscle_moisture_percentage	region1inRegion2	7687
CNVR96	1086	8	44371710	44447879	gain	38096	811090	108610930	Muscle_moisture_percentage	region1inRegion2	76169
CNVR97	1112	8	64473379	64515977	loss	38096	811090	108610930	Muscle_moisture_percentage	region1inRegion2	42598
CNVR98	1116	8	68684763	68710413	gain	38096	811090	108610930	Muscle_moisture_percentage	region1inRegion2	25650
CNVR99	1129	8	85727251	85735420	gain	38096	811090	108610930	Muscle_moisture_percentage	region1inRegion2	8169
CNVR94	1059	8	26029928	26034839	loss	5704	3470575	124156612	Loin_muscle_area	region1inRegion2	4911
CNVR95	1068	8	30016900	30024587	loss	5704	3470575	124156612	Loin_muscle_area	region1inRegion2	7687
CNVR96	1086	8	44371710	44447879	gain	5704	3470575	124156612	Loin_muscle_area	region1inRegion2	76169
CNVR97	1112	8	64473379	64515977	loss	5704	3470575	124156612	Loin_muscle_area	region1inRegion2	42598
CNVR98	1116	8	68684763	68710413	gain	5704	3470575	124156612	Loin_muscle_area	region1inRegion2	25650
CNVR99	1129	8	85727251	85735420	gain	5704	3470575	124156612	Loin_muscle_area	region1inRegion2	8169
CNVR100	1169	8	122108843	122121928	loss	5704	3470575	124156612	Loin_muscle_area	region1inRegion2	13085
CNVR94	1059	8	26029928	26034839	loss	7477	3470575	124156612	Nonfunctional_nipples	region1inRegion2	4911
CNVR95	1068	8	30016900	30024587	loss	7477	3470575	124156612	Nonfunctional_nipples	region1inRegion2	7687
CNVR96	1086	8	44371710	44447879	gain	7477	3470575	124156612	Nonfunctional_nipples	region1inRegion2	76169
CNVR97	1112	8	64473379	64515977	loss	7477	3470575	124156612	Nonfunctional_nipples	region1inRegion2	42598
CNVR98	1116	8	68684763	68710413	gain	7477	3470575	124156612	Nonfunctional_nipples	region1inRegion2	25650
CNVR99	1129	8	85727251	85735420	gain	7477	3470575	124156612	Nonfunctional_nipples	region1inRegion2	8169
CNVR100	1169	8	122108843	122121928	loss	7477	3470575	124156612	Nonfunctional_nipples	region1inRegion2	13085
CNVR94	1059	8	26029928	26034839	loss	24282	3470575	145599486	Litter_size	region1inRegion2	4911
CNVR95	1068	8	30016900	30024587	loss	24282	3470575	145599486	Litter_size	region1inRegion2	7687
CNVR96	1086	8	44371710	44447879	gain	24282	3470575	145599486	Litter_size	region1inRegion2	76169
CNVR97	1112	8	64473379	64515977	loss	24282	3470575	145599486	Litter_size	region1inRegion2	42598
CNVR98	1116	8	68684763	68710413	gain	24282	3470575	145599486	Litter_size	region1inRegion2	25650
CNVR99	1129	8	85727251	85735420	gain	24282	3470575	145599486	Litter_size	region1inRegion2	8169
CNVR100	1169	8	122108843	122121928	loss	24282	3470575	145599486	Litter_size	region1inRegion2	13085
CNVR101	1176	8	129139072	129153237	loss	24282	3470575	145599486	Litter_size	region1inRegion2	14165
CNVR94	1059	8	26029928	26034839	loss	21253	4904068	124426079	Body_weight_(30_weeks)	region1inRegion2	4911
CNVR95	1068	8	30016900	30024587	loss	21253	4904068	124426079	Body_weight_(30_weeks)	region1inRegion2	7687
CNVR96	1086	8	44371710	44447879	gain	21253	4904068	124426079	Body_weight_(30_weeks)	region1inRegion2	76169
CNVR97	1112	8	64473379	64515977	loss	21253	4904068	124426079	Body_weight_(30_weeks)	region1inRegion2	42598
CNVR98	1116	8	68684763	68710413	gain	21253	4904068	124426079	Body_weight_(30_weeks)	region1inRegion2	25650
CNVR99	1129	8	85727251	85735420	gain	21253	4904068	124426079	Body_weight_(30_weeks)	region1inRegion2	8169
CNVR100	1169	8	122108843	122121928	loss	21253	4904068	124426079	Body_weight_(30_weeks)	region1inRegion2	13085
CNVR94	1059	8	26029928	26034839	loss	21254	7495097	92309019	Body_weight_(34_weeks)	region1inRegion2	4911
CNVR95	1068	8	30016900	30024587	loss	21254	7495097	92309019	Body_weight_(34_weeks)	region1inRegion2	7687
CNVR96	1086	8	44371710	44447879	gain	21254	7495097	92309019	Body_weight_(34_weeks)	region1inRegion2	76169
CNVR97	1112	8	64473379	64515977	loss	21254	7495097	92309019	Body_weight_(34_weeks)	region1inRegion2	42598
CNVR98	1116	8	68684763	68710413	gain	21254	7495097	92309019	Body_weight_(34_weeks)	region1inRegion2	25650
CNVR99	1129	8	85727251	85735420	gain	21254	7495097	92309019	Body_weight_(34_weeks)	region1inRegion2	8169
CNVR94	1059	8	26029928	26034839	loss	38095	7900624	141371437	Loin_muscle_area	region1inRegion2	4911
CNVR95	1068	8	30016900	30024587	loss	38095	7900624	141371437	Loin_muscle_area	region1inRegion2	7687
CNVR96	1086	8	44371710	44447879	gain	38095	7900624	141371437	Loin_muscle_area	region1inRegion2	76169
CNVR97	1112	8	64473379	64515977	loss	38095	7900624	141371437	Loin_muscle_area	region1inRegion2	42598
CNVR98	1116	8	68684763	68710413	gain	38095	7900624	141371437	Loin_muscle_area	region1inRegion2	25650
CNVR99	1129	8	85727251	85735420	gain	38095	7900624	141371437	Loin_muscle_area	region1inRegion2	8169
CNVR100	1169	8	122108843	122121928	loss	38095	7900624	141371437	Loin_muscle_area	region1inRegion2	13085
CNVR101	1176	8	129139072	129153237	loss	38095	7900624	141371437	Loin_muscle_area	region1inRegion2	14165
CNVR94	1059	8	26029928	26034839	loss	21255	8803225	127786071	Backfat_at_rump	region1inRegion2	4911
CNVR95	1068	8	30016900	30024587	loss	21255	8803225	127786071	Backfat_at_rump	region1inRegion2	7687
CNVR96	1086	8	44371710	44447879	gain	21255	8803225	127786071	Backfat_at_rump	region1inRegion2	76169
CNVR97	1112	8	64473379	64515977	loss	21255	8803225	127786071	Backfat_at_rump	region1inRegion2	42598
CNVR98	1116	8	68684763	68710413	gain	21255	8803225	127786071	Backfat_at_rump	region1inRegion2	25650
CNVR99	1129	8	85727251	85735420	gain	21255	8803225	127786071	Backfat_at_rump	region1inRegion2	8169
CNVR100	1169	8	122108843	122121928	loss	21255	8803225	127786071	Backfat_at_rump	region1inRegion2	13085
CNVR94	1059	8	26029928	26034839	loss	439	9606098	99338918	Feet_weight	region1inRegion2	4911
CNVR95	1068	8	30016900	30024587	loss	439	9606098	99338918	Feet_weight	region1inRegion2	7687
CNVR96	1086	8	44371710	44447879	gain	439	9606098	99338918	Feet_weight	region1inRegion2	76169
CNVR97	1112	8	64473379	64515977	loss	439	9606098	99338918	Feet_weight	region1inRegion2	42598
CNVR98	1116	8	68684763	68710413	gain	439	9606098	99338918	Feet_weight	region1inRegion2	25650
CNVR99	1129	8	85727251	85735420	gain	439	9606098	99338918	Feet_weight	region1inRegion2	8169
CNVR94	1059	8	26029928	26034839	loss	21257	10192847	92906326	intestinal_fat_weight	region1inRegion2	4911
CNVR95	1068	8	30016900	30024587	loss	21257	10192847	92906326	intestinal_fat_weight	region1inRegion2	7687
CNVR96	1086	8	44371710	44447879	gain	21257	10192847	92906326	intestinal_fat_weight	region1inRegion2	76169
CNVR97	1112	8	64473379	64515977	loss	21257	10192847	92906326	intestinal_fat_weight	region1inRegion2	42598
CNVR98	1116	8	68684763	68710413	gain	21257	10192847	92906326	intestinal_fat_weight	region1inRegion2	25650
CNVR99	1129	8	85727251	85735420	gain	21257	10192847	92906326	intestinal_fat_weight	region1inRegion2	8169
CNVR94	1059	8	26029928	26034839	loss	1083	10844129	26534249	Head_weight	region1inRegion2	4911
CNVR94	1059	8	26029928	26034839	loss	1085	10844129	26534249	Head_weight	region1inRegion2	4911
CNVR94	1059	8	26029928	26034839	loss	1086	10844129	26534249	Loin_and_neck_meat_weight	region1inRegion2	4911
CNVR94	1059	8	26029928	26034839	loss	1087	10844129	26534249	Ham_weight	region1inRegion2	4911
CNVR94	1059	8	26029928	26034839	loss	1088	10844129	26534249	Ham_percentage	region1inRegion2	4911
CNVR94	1059	8	26029928	26034839	loss	1089	10844129	26534249	Body_weight_(slaughter)	region1inRegion2	4911
CNVR94	1059	8	26029928	26034839	loss	1090	10844129	26534249	Shoulder_meat_weight	region1inRegion2	4911
CNVR94	1059	8	26029928	26034839	loss	1091	10844129	26534249	Heart_weight	region1inRegion2	4911
CNVR94	1059	8	26029928	26034839	loss	1092	10844129	26534249	Average_daily_gain	region1inRegion2	4911
CNVR94	1059	8	26029928	26034839	loss	1093	10844129	26534249	Carcass_length	region1inRegion2	4911
CNVR94	1059	8	26029928	26034839	loss	1095	10844129	26534249	Carcass_weight_(cold)	region1inRegion2	4911
CNVR94	1059	8	26029928	26034839	loss	1096	10844129	26534249	Liver_weight	region1inRegion2	4911
CNVR94	1059	8	26029928	26034839	loss	1097	10844129	26534249	Creatine_kinase_level	region1inRegion2	4911
CNVR94	1059	8	26029928	26034839	loss	16853	10844129	26534249	Average_daily_gain	region1inRegion2	4911
CNVR94	1059	8	26029928	26034839	loss	16869	10844129	26534249	Carcass_weight_(hot)	region1inRegion2	4911
CNVR94	1059	8	26029928	26034839	loss	2992	10844129	29471436	PH_for_Longissmus_dorsi	region1inRegion2	4911
CNVR94	1059	8	26029928	26034839	loss	3671	10844129	29471436	Average_daily_gain	region1inRegion2	4911
CNVR94	1059	8	26029928	26034839	loss	3684	10844129	29471436	Carcass_length	region1inRegion2	4911
CNVR94	1059	8	26029928	26034839	loss	4149	10844129	29471436	Average_daily_gain	region1inRegion2	4911
CNVR94	1059	8	26029928	26034839	loss	4150	10844129	29471436	Average_daily_gain	region1inRegion2	4911
CNVR94	1059	8	26029928	26034839	loss	4151	10844129	29471436	backfat_at_P2_position	region1inRegion2	4911
CNVR94	1059	8	26029928	26034839	loss	4152	10844129	29471436	backfat_at_P2_position	region1inRegion2	4911
CNVR94	1059	8	26029928	26034839	loss	4153	10844129	29471436	Average_backfat_thickness	region1inRegion2	4911
CNVR94	1059	8	26029928	26034839	loss	4154	10844129	29471436	Lean_meat_percentage	region1inRegion2	4911
CNVR94	1059	8	26029928	26034839	loss	16895	10844129	38873367	Carcass_weight_(hot)	region1inRegion2	4911
CNVR95	1068	8	30016900	30024587	loss	16895	10844129	38873367	Carcass_weight_(hot)	region1inRegion2	7687
CNVR94	1059	8	26029928	26034839	loss	5129	10844129	51688517	Ham_weight	region1inRegion2	4911
CNVR95	1068	8	30016900	30024587	loss	5129	10844129	51688517	Ham_weight	region1inRegion2	7687
CNVR96	1086	8	44371710	44447879	gain	5129	10844129	51688517	Ham_weight	region1inRegion2	76169

CNVR94	1059	8	26029928	26034839	loss	7579	10844129	148491826	Melanoma_susceptibility	region1inRegion2	4911
CNVR95	1068	8	30016900	30024587	loss	7579	10844129	148491826	Melanoma_susceptibility	region1inRegion2	7687
CNVR96	1086	8	44371710	44447879	gain	7579	10844129	148491826	Melanoma_susceptibility	region1inRegion2	76169
CNVR97	1112	8	64473379	64515977	loss	7579	10844129	148491826	Melanoma_susceptibility	region1inRegion2	42598
CNVR98	1116	8	68684763	68710413	gain	7579	10844129	148491826	Melanoma_susceptibility	region1inRegion2	25650
CNVR99	1129	8	85727251	85735420	gain	7579	10844129	148491826	Melanoma_susceptibility	region1inRegion2	8169
CNVR100	1169	8	122108843	122121928	loss	7579	10844129	148491826	Melanoma_susceptibility	region1inRegion2	13085
CNVR101	1176	8	129139072	129153237	loss	7579	10844129	148491826	Melanoma_susceptibility	region1inRegion2	14165
CNVR94	1059	8	26029928	26034839	loss	40	11308413	26534249	Leaf_fat_weight	region1inRegion2	4911
CNVR94	1059	8	26029928	26034839	loss	29701	11308413	77429212	Body_weight_(20_weeks)	region1inRegion2	4911
CNVR95	1068	8	30016900	30024587	loss	29701	11308413	77429212	Body_weight_(20_weeks)	region1inRegion2	7687
CNVR96	1086	8	44371710	44447879	gain	29701	11308413	77429212	Body_weight_(20_weeks)	region1inRegion2	76169
CNVR97	1112	8	64473379	64515977	loss	29701	11308413	77429212	Body_weight_(20_weeks)	region1inRegion2	42598
CNVR98	1116	8	68684763	68710413	gain	29701	11308413	77429212	Body_weight_(20_weeks)	region1inRegion2	25650
CNVR94	1059	8	26029928	26034839	loss	29702	11308413	77429212	Carcass_weight_(hot)	region1inRegion2	4911
CNVR95	1068	8	30016900	30024587	loss	29702	11308413	77429212	Carcass_weight_(hot)	region1inRegion2	7687
CNVR96	1086	8	44371710	44447879	gain	29702	11308413	77429212	Carcass_weight_(hot)	region1inRegion2	76169
CNVR97	1112	8	64473379	64515977	loss	29702	11308413	77429212	Carcass_weight_(hot)	region1inRegion2	42598
CNVR98	1116	8	68684763	68710413	gain	29702	11308413	77429212	Carcass_weight_(hot)	region1inRegion2	25650
CNVR94	1059	8	26029928	26034839	loss	29700	11308413	108610930	Body_weight_(3_weeks)	region1inRegion2	4911
CNVR95	1068	8	30016900	30024587	loss	29700	11308413	108610930	Body_weight_(3_weeks)	region1inRegion2	7687
CNVR96	1086	8	44371710	44447879	gain	29700	11308413	108610930	Body_weight_(3_weeks)	region1inRegion2	76169
CNVR97	1112	8	64473379	64515977	loss	29700	11308413	108610930	Body_weight_(3_weeks)	region1inRegion2	42598
CNVR98	1116	8	68684763	68710413	gain	29700	11308413	108610930	Body_weight_(3_weeks)	region1inRegion2	25650
CNVR99	1129	8	85727251	85735420	gain	29700	11308413	108610930	Body_weight_(3_weeks)	region1inRegion2	8169
CNVR94	1059	8	26029928	26034839	loss	15105	11308413	124156612	Alkaline_phosphatase_activity	region1inRegion2	4911
CNVR95	1068	8	30016900	30024587	loss	15105	11308413	124156612	Alkaline_phosphatase_activity	region1inRegion2	7687
CNVR96	1086	8	44371710	44447879	gain	15105	11308413	124156612	Alkaline_phosphatase_activity	region1inRegion2	76169
CNVR97	1112	8	64473379	64515977	loss	15105	11308413	124156612	Alkaline_phosphatase_activity	region1inRegion2	42598
CNVR98	1116	8	68684763	68710413	gain	15105	11308413	124156612	Alkaline_phosphatase_activity	region1inRegion2	25650
CNVR99	1129	8	85727251	85735420	gain	15105	11308413	124156612	Alkaline_phosphatase_activity	region1inRegion2	8169
CNVR100	1169	8	122108843	122121928	loss	15105	11308413	124156612	Alkaline_phosphatase_activity	region1inRegion2	13085
CNVR94	1059	8	26029928	26034839	loss	21252	12243782	137590478	Abdominal_fat_weight	region1inRegion2	4911
CNVR95	1068	8	30016900	30024587	loss	21252	12243782	137590478	Abdominal_fat_weight	region1inRegion2	7687
CNVR96	1086	8	44371710	44447879	gain	21252	12243782	137590478	Abdominal_fat_weight	region1inRegion2	76169
CNVR97	1112	8	64473379	64515977	loss	21252	12243782	137590478	Abdominal_fat_weight	region1inRegion2	42598
CNVR98	1116	8	68684763	68710413	gain	21252	12243782	137590478	Abdominal_fat_weight	region1inRegion2	25650
CNVR99	1129	8	85727251	85735420	gain	21252	12243782	137590478	Abdominal_fat_weight	region1inRegion2	8169
CNVR100	1169	8	122108843	122121928	loss	21252	12243782	137590478	Abdominal_fat_weight	region1inRegion2	13085
CNVR101	1176	8	129139072	129153237	loss	21252	12243782	137590478	Abdominal_fat_weight	region1inRegion2	14165
CNVR94	1059	8	26029928	26034839	loss	4252	15448482	26534249	Teat_number	region1inRegion2	4911
CNVR94	1059	8	26029928	26034839	loss	334	15636344	72039691	Average_daily_gain	region1inRegion2	4911
CNVR95	1068	8	30016900	30024587	loss	334	15636344	72039691	Average_daily_gain	region1inRegion2	7687
CNVR96	1086	8	44371710	44447879	gain	334	15636344	72039691	Average_daily_gain	region1inRegion2	76169
CNVR97	1112	8	64473379	64515977	loss	334	15636344	72039691	Average_daily_gain	region1inRegion2	42598
CNVR98	1116	8	68684763	68710413	gain	334	15636344	72039691	Average_daily_gain	region1inRegion2	25650
CNVR94	1059	8	26029928	26034839	loss	21256	15655066	79969240	Leaf_fat_weight	region1inRegion2	4911
CNVR95	1068	8	30016900	30024587	loss	21256	15655066	79969240	Leaf_fat_weight	region1inRegion2	7687
CNVR96	1086	8	44371710	44447879	gain	21256	15655066	79969240	Leaf_fat_weight	region1inRegion2	76169
CNVR97	1112	8	64473379	64515977	loss	21256	15655066	79969240	Leaf_fat_weight	region1inRegion2	42598
CNVR98	1116	8	68684763	68710413	gain	21256	15655066	79969240	Leaf_fat_weight	region1inRegion2	25650
CNVR94	1059	8	26029928	26034839	loss	5148	19749801	39840424	Intramuscular_fat_content	region1inRegion2	4911
CNVR95	1068	8	30016900	30024587	loss	5148	19749801	39840424	Intramuscular_fat_content	region1inRegion2	7687
CNVR94	1059	8	26029928	26034839	loss	5467	19749801	46427317	Lymphocyte_number	region1inRegion2	4911
CNVR95	1068	8	30016900	30024587	loss	5467	19749801	46427317	Lymphocyte_number	region1inRegion2	7687
CNVR96	1086	8	44371710	44447879	gain	5467	19749801	46427317	Lymphocyte_number	region1inRegion2	76169
CNVR94	1059	8	26029928	26034839	loss	5478	19749801	46427317	Segmented_neutrophil_number	region1inRegion2	4911
CNVR95	1068	8	30016900	30024587	loss	5478	19749801	46427317	Segmented_neutrophil_number	region1inRegion2	7687
CNVR96	1086	8	44371710	44447879	gain	5478	19749801	46427317	Segmented_neutrophil_number	region1inRegion2	76169
CNVR94	1059	8	26029928	26034839	loss	5904	19749801	46427317	Activity_during_lying	region1inRegion2	4911
CNVR95	1068	8	30016900	30024587	loss	5904	19749801	46427317	Activity_during_lying	region1inRegion2	7687
CNVR96	1086	8	44371710	44447879	gain	5904	19749801	46427317	Activity_during_lying	region1inRegion2	76169
CNVR94	1059	8	26029928	26034839	loss	3263	19749801	72039691	Spareribs_weight	region1inRegion2	4911
CNVR95	1068	8	30016900	30024587	loss	3263	19749801	72039691	Spareribs_weight	region1inRegion2	7687
CNVR96	1086	8	44371710	44447879	gain	3263	19749801	72039691	Spareribs_weight	region1inRegion2	76169
CNVR97	1112	8	64473379	64515977	loss	3263	19749801	72039691	Spareribs_weight	region1inRegion2	42598
CNVR98	1116	8	68684763	68710413	gain	3263	19749801	72039691	Spareribs_weight	region1inRegion2	25650
CNVR94	1059	8	26029928	26034839	loss	5510	19896638	46427317	Loin_muscle_width	region1inRegion2	4911
CNVR95	1068	8	30016900	30024587	loss	5510	19896638	46427317	Loin_muscle_width	region1inRegion2	7687
CNVR96	1086	8	44371710	44447879	gain	5510	19896638	46427317	Loin_muscle_width	region1inRegion2	76169
CNVR94	1059	8	26029928	26034839	loss	15883	20979749	41588816	Stearic_acid_content	region1inRegion2	4911
CNVR95	1068	8	30016900	30024587	loss	15883	20979749	41588816	Stearic_acid_content	region1inRegion2	7687
CNVR95	1068	8	30016900	30024587	loss	1099	26534249	38873367	Meat_color-a	region1inRegion2	7687
CNVR95	1068	8	30016900	30024587	loss	12266	26534249	38873367	Interferon-gamma_level	region1inRegion2	7687
CNVR95	1068	8	30016900	30024587	loss	15055	26534249	77429212	Mean_corpuscular_hemoglobin_content	region1inRegion2	7687
CNVR96	1086	8	44371710	44447879	gain	15055	26534249	77429212	Mean_corpuscular_hemoglobin_content	region1inRegion2	76169
CNVR97	1112	8	64473379	64515977	loss	15055	26534249	77429212	Mean_corpuscular_hemoglobin_content	region1inRegion2	42598
CNVR98	1116	8	68684763	68710413	gain	15055	26534249	77429212	Mean_corpuscular_hemoglobin_content	region1inRegion2	25650
CNVR95	1068	8	30016900	30024587	loss	29703	26534249	77429212	Carcass_length	region1inRegion2	7687
CNVR96	1086	8	44371710	44447879	gain	29703	26534249	77429212	Carcass_length	region1inRegion2	76169
CNVR97	1112	8	64473379	64515977	loss	29703	26534249	77429212	Carcass_length	region1inRegion2	42598
CNVR98	1116	8	68684763	68710413	gain	29703	26534249	77429212	Carcass_length	region1inRegion2	25650
CNVR95	1068	8	30016900	30024587	loss	451	26534249	77429212	Corpus_luteum_number	region1inRegion2	7687
CNVR96	1086	8	44371710	44447879	gain	451	26534249	77429212	Corpus_luteum_number	region1inRegion2	76169
CNVR97	1112	8	64473379	64515977	loss	451	26534249	77429212	Corpus_luteum_number	region1inRegion2	42598
CNVR98	1116	8	68684763	68710413	gain	451	26534249	77429212	Corpus_luteum_number	region1inRegion2	25650
CNVR95	1068	8	30016900	30024587	loss	29699	26534249	90653103	Body_weight_(birth)	region1inRegion2	7687
CNVR96	1086	8	44371710	44447879	gain	29699	26534249	90653103	Body_weight_(birth)	region1inRegion2	76169
CNVR97	1112	8	64473379	64515977	loss	29699	26534249	90653103	Body_weight_(birth)	region1inRegion2	42598
CNVR98	1116	8	68684763	68710413	gain	29699	26534249	90653103	Body_weight_(birth)	region1inRegion2	25650
CNVR99	1129	8	85727251	85735420	gain	29699	26534249	90653103	Body_weight_(birth)	region1inRegion2	8169
CNVR95	1068	8	30016900	30024587	loss	15052	26534249	108610930	Platelet_count	region1inRegion2	7687
CNVR96	1086	8	44371710	44447879	gain	15052	26534249	108610930	Platelet_count	region1inRegion2	76169
CNVR97	1112	8	64473379	64515977	loss	15052	26534249	108610930	Platelet_count	region1inRegion2	42598
CNVR98	1116	8	68684763	68710413	gain	15052	26534249	108610930	Platelet_count	region1inRegion2	25650
CNVR99	1129	8	85727251	85735420	gain	15052	26534249	108610930	Platelet_count	region1inRegion2	8169
CNVR95	1068	8	30016900	30024587	loss	683	27450871	34473703	Teat_number	region1inRegion2	7687
CNVR95	1068	8	30016900	30024587	loss	5153	27756434	51578098	Lipid_accretion_rate	region1inRegion2	7687
CNVR96	1086	8	44371710	44447879	gain	5153	27756434	51578098	Lipid_accretion_rate	region1inRegion2	76169
CNVR95	1068	8	30016900	30024587	loss	2993	29471436	77429212	CIE-b*	region1inRegion2	7687
CNVR96	1086	8	44371710	44447879	gain	2993	29471436	77429212	CIE-b*	region1inRegion2	76169
CNVR97	1112	8	64473379	64515977	loss	2993	29471436	77429212	CIE-b*	region1inRegion2	42598
CNVR98	1116	8	68684763	68710413	gain	2993	29471436	77429212	CIE-b*	region1inRegion2	25650
CNVR95	1068	8	30016900	30024587	loss	22165	29607052	47692165	Red_blood_cell_count	region1inRegion2	7687
CNVR96	1086	8	44371710	44447879	gain	22165	29607052	47692165	Red_blood_cell_count	region1inRegion2	76169
CNVR95	1068	8	30016900	30024587	loss	474	29785347	34473703	Corpus_luteum_number	region1inRegion2	7687
CNVR96	1086	8	44371710	44447879	gain	22138	30046732	75402090	Mean_corpuscular_hemoglobin_concentration	region1inRegion2	76169
CNVR97	1112	8	64473379	64515977	loss	22138	30046732	75402090	Mean_corpuscular_hemoglobin_concentration	region1inRegion2	42598
CNVR98	1116	8	68684763	68710413	gain	22138	30046732	75402090	Mean_corpuscular_hemoglobin_concentration	region1inRegion2	25650
CNVR96	1086	8	44371710	44447879	gain	22145	30720808	47482649	Red_blood_cell_count	region1inRegion2	76169
CNVR96	1086	8	44371710	44447879	gain	421	31625772	114156769	Belly_weight	region1inRegion2	76169
CNVR97	1112	8	64473379	64515977	loss	421	31625772	114156769	Belly_weight	region1inRegion2	42598
CNVR98	1116	8	68684763	68710413	gain	421	31625772	114156769	Belly_weight	region1inRegion2	25650
CNVR99	1129	8	85727251	85735420	gain	421	31625772	114156769	Belly_weight	region1inRegion2	8169
CNVR96	1086	8	44371710	44447879	gain	391	31625772	124047324	Ham_weight	region1inRegion2	76169
CNVR97	1112	8	64473379	64515977	loss	391	31625772	124047324	Ham_weight	region1inRegion2	42598
CNVR98	1116	8	68684763	68710413	gain	391	31625772	124047324	Ham_weight	region1inRegion2	25650
CNVR99	1129	8	85727251	85735420	gain	391	31625772	124047324	Ham_weight	region1inRegion2	8169
CNVR100	1169	8	122108843	122121928	loss	391	31625772	124047324	Ham_weight	region1inRegion2	13085
CNVR96	1086	8	44371710	44447879	gain	22168	32779044	47692165	Platelet_distribution_width	region1inRegion2	76169
CNVR96	1086	8	44371710	44447879	gain	22147	32779044	47744169	Red_blood_cell_count	region1inRegion2	76169
CNVR96	1086	8	44371710	44447879	gain	22144	32779044	74641551	Mean_corpuscular_volume	region1inRegion2	76169

CNVR97	1112	8	64473379	64515977	loss	22144	32779044	74641551	Mean_corpuscular_volume	region1inRegion2	42598
CNVR98	1116	8	68684763	68710413	gain	22144	32779044	74641551	Mean_corpuscular_volume	region1inRegion2	25650
CNVR96	1086	8	44371710	44447879	gain	22164	32779044	79533086	Mean_corpuscular_volume	region1inRegion2	76169
CNVR97	1112	8	64473379	64515977	loss	22164	32779044	79533086	Mean_corpuscular_volume	region1inRegion2	42598
CNVR98	1116	8	68684763	68710413	gain	22164	32779044	79533086	Mean_corpuscular_volume	region1inRegion2	25650
CNVR96	1086	8	44371710	44447879	gain	22162	33248935	74705127	Mean_corpuscular_hemoglobin_concentration	region1inRegion2	76169
CNVR97	1112	8	64473379	64515977	loss	22162	33248935	74705127	Mean_corpuscular_hemoglobin_concentration	region1inRegion2	42598
CNVR98	1116	8	68684763	68710413	gain	22162	33248935	74705127	Mean_corpuscular_hemoglobin_concentration	region1inRegion2	25650
CNVR96	1086	8	44371710	44447879	gain	523	33339776	136437041	Uterine_capacity	region1inRegion2	76169
CNVR97	1112	8	64473379	64515977	loss	523	33339776	136437041	Uterine_capacity	region1inRegion2	42598
CNVR98	1116	8	68684763	68710413	gain	523	33339776	136437041	Uterine_capacity	region1inRegion2	25650
CNVR99	1129	8	85727251	85735420	gain	523	33339776	136437041	Uterine_capacity	region1inRegion2	8169
CNVR100	1169	8	122108843	122121928	loss	523	33339776	136437041	Uterine_capacity	region1inRegion2	13085
CNVR101	1176	8	129139072	129153237	loss	523	33339776	136437041	Uterine_capacity	region1inRegion2	14165
CNVR96	1086	8	44371710	44447879	gain	8730	33832138	60824182	Gait_score_(hind)	region1inRegion2	76169
CNVR96	1086	8	44371710	44447879	gain	22140	35266996	47744169	Mean_corpuscular_volume	region1inRegion2	76169
CNVR96	1086	8	44371710	44447879	gain	22136	37231380	47744169	Mean_corpuscular_hemoglobin_concentration	region1inRegion2	76169
CNVR96	1086	8	44371710	44447879	gain	7599	38873367	50483614	Melanoma_susceptibility	region1inRegion2	76169
CNVR96	1086	8	44371710	44447879	gain	4253	38873367	90653103	Teat_number	region1inRegion2	76169
CNVR97	1112	8	64473379	64515977	loss	4253	38873367	90653103	Teat_number	region1inRegion2	42598
CNVR98	1116	8	68684763	68710413	gain	4253	38873367	90653103	Teat_number	region1inRegion2	25650
CNVR99	1129	8	85727251	85735420	gain	4253	38873367	90653103	Teat_number	region1inRegion2	8169
CNVR96	1086	8	44371710	44447879	gain	1100	38873367	124156612	Teat_number	region1inRegion2	76169
CNVR97	1112	8	64473379	64515977	loss	1100	38873367	124156612	Teat_number	region1inRegion2	42598
CNVR98	1116	8	68684763	68710413	gain	1100	38873367	124156612	Teat_number	region1inRegion2	25650
CNVR99	1129	8	85727251	85735420	gain	1100	38873367	124156612	Teat_number	region1inRegion2	8169
CNVR100	1169	8	122108843	122121928	loss	1100	38873367	124156612	Teat_number	region1inRegion2	13085
CNVR96	1086	8	44371710	44447879	gain	15054	39840424	77429212	Mean_corpuscular_volume	region1inRegion2	76169
CNVR97	1112	8	64473379	64515977	loss	15054	39840424	77429212	Mean_corpuscular_volume	region1inRegion2	42598
CNVR98	1116	8	68684763	68710413	gain	15054	39840424	77429212	Mean_corpuscular_volume	region1inRegion2	25650
CNVR96	1086	8	44371710	44447879	gain	2950	39840424	77429212	Carcass_weight_(cold)	region1inRegion2	76169
CNVR97	1112	8	64473379	64515977	loss	2950	39840424	77429212	Carcass_weight_(cold)	region1inRegion2	42598
CNVR98	1116	8	68684763	68710413	gain	2950	39840424	77429212	Carcass_weight_(cold)	region1inRegion2	25650
CNVR96	1086	8	44371710	44447879	gain	4265	39840424	77429212	Lymphocyte_number	region1inRegion2	76169
CNVR97	1112	8	64473379	64515977	loss	4265	39840424	77429212	Lymphocyte_number	region1inRegion2	42598
CNVR98	1116	8	68684763	68710413	gain	4265	39840424	77429212	Lymphocyte_number	region1inRegion2	25650
CNVR96	1086	8	44371710	44447879	gain	4267	39840424	77429212	N1c-positive_leukocyte_number	region1inRegion2	76169
CNVR97	1112	8	64473379	64515977	loss	4267	39840424	77429212	N1c-positive_leukocyte_number	region1inRegion2	42598
CNVR98	1116	8	68684763	68710413	gain	4267	39840424	77429212	N1c-positive_leukocyte_number	region1inRegion2	25650
CNVR96	1086	8	44371710	44447879	gain	4268	39840424	77429212	IgM-positive_leukocyte_number	region1inRegion2	76169
CNVR97	1112	8	64473379	64515977	loss	4268	39840424	77429212	IgM-positive_leukocyte_number	region1inRegion2	42598
CNVR98	1116	8	68684763	68710413	gain	4268	39840424	77429212	IgM-positive_leukocyte_number	region1inRegion2	25650
CNVR96	1086	8	44371710	44447879	gain	4278	39840424	77429212	hematocrit	region1inRegion2	76169
CNVR97	1112	8	64473379	64515977	loss	4278	39840424	77429212	hematocrit	region1inRegion2	42598
CNVR98	1116	8	68684763	68710413	gain	4278	39840424	77429212	hematocrit	region1inRegion2	25650
CNVR96	1086	8	44371710	44447879	gain	15053	39840424	90653103	Red_blood_cell_count	region1inRegion2	76169
CNVR97	1112	8	64473379	64515977	loss	15053	39840424	90653103	Red_blood_cell_count	region1inRegion2	42598
CNVR98	1116	8	68684763	68710413	gain	15053	39840424	90653103	Red_blood_cell_count	region1inRegion2	25650
CNVR99	1129	8	85727251	85735420	gain	15053	39840424	90653103	Red_blood_cell_count	region1inRegion2	8169
CNVR96	1086	8	44371710	44447879	gain	4277	39840424	108610930	Hemoglobin	region1inRegion2	76169
CNVR97	1112	8	64473379	64515977	loss	4277	39840424	108610930	Hemoglobin	region1inRegion2	42598
CNVR98	1116	8	68684763	68710413	gain	4277	39840424	108610930	Hemoglobin	region1inRegion2	25650
CNVR99	1129	8	85727251	85735420	gain	4277	39840424	108610930	Hemoglobin	region1inRegion2	8169
CNVR96	1086	8	44371710	44447879	gain	15106	39840424	124156612	Glucose_level	region1inRegion2	76169
CNVR97	1112	8	64473379	64515977	loss	15106	39840424	124156612	Glucose_level	region1inRegion2	42598
CNVR98	1116	8	68684763	68710413	gain	15106	39840424	124156612	Glucose_level	region1inRegion2	25650
CNVR99	1129	8	85727251	85735420	gain	15106	39840424	124156612	Glucose_level	region1inRegion2	8169
CNVR100	1169	8	122108843	122121928	loss	15106	39840424	124156612	Glucose_level	region1inRegion2	13085
CNVR96	1086	8	44371710	44447879	gain	9095	43716890	48977449	CIE-b*	region1inRegion2	76169
CNVR96	1086	8	44371710	44447879	gain	438	43822094	135374495	Head_weight	region1inRegion2	76169
CNVR97	1112	8	64473379	64515977	loss	438	43822094	135374495	Head_weight	region1inRegion2	42598
CNVR98	1116	8	68684763	68710413	gain	438	43822094	135374495	Head_weight	region1inRegion2	25650
CNVR99	1129	8	85727251	85735420	gain	438	43822094	135374495	Head_weight	region1inRegion2	8169
CNVR100	1169	8	122108843	122121928	loss	438	43822094	135374495	Head_weight	region1inRegion2	13085
CNVR101	1176	8	129139072	129153237	loss	438	43822094	135374495	Head_weight	region1inRegion2	14165
CNVR96	1086	8	44371710	44447879	gain	21456	44439746	44439786	Mean_corpuscular_hemoglobin_content	region2inRegion1	40
CNVR96	1086	8	44371710	44447879	gain	21468	44439746	44439786	Mean_corpuscular_volume	region2inRegion1	40
CNVR97	1112	8	64473379	64515977	loss	6374	46427317	120532322	Bilirubin_level	region1inRegion2	42598
CNVR98	1116	8	68684763	68710413	gain	6374	46427317	120532322	Bilirubin_level	region1inRegion2	25650
CNVR99	1129	8	85727251	85735420	gain	6374	46427317	120532322	Bilirubin_level	region1inRegion2	8169
CNVR97	1112	8	64473379	64515977	loss	492	52718097	136739744	Corpus_luteum_number	region1inRegion2	42598
CNVR98	1116	8	68684763	68710413	gain	492	52718097	136739744	Corpus_luteum_number	region1inRegion2	25650
CNVR99	1129	8	85727251	85735420	gain	492	52718097	136739744	Corpus_luteum_number	region1inRegion2	8169
CNVR100	1169	8	122108843	122121928	loss	492	52718097	136739744	Corpus_luteum_number	region1inRegion2	13085
CNVR101	1176	8	129139072	129153237	loss	492	52718097	136739744	Corpus_luteum_number	region1inRegion2	14165
CNVR97	1112	8	64473379	64515977	loss	55873	60762560	68864355	Cryptorchidism	region1inRegion2	42598
CNVR98	1116	8	68684763	68710413	gain	55873	60762560	68864355	Cryptorchidism	region1inRegion2	25650
CNVR97	1112	8	64473379	64515977	loss	55874	60762560	68864355	Cryptorchidism	region1inRegion2	42598
CNVR98	1116	8	68684763	68710413	gain	55874	60762560	68864355	Cryptorchidism	region1inRegion2	25650
CNVR97	1112	8	64473379	64515977	loss	55875	60762560	68864355	Cryptorchidism	region1inRegion2	42598
CNVR98	1116	8	68684763	68710413	gain	55875	60762560	68864355	Cryptorchidism	region1inRegion2	25650
CNVR97	1112	8	64473379	64515977	loss	55857	61064094	79482023	Cryptorchidism	region1inRegion2	42598
CNVR98	1116	8	68684763	68710413	gain	55857	61064094	79482023	Cryptorchidism	region1inRegion2	25650
CNVR97	1112	8	64473379	64515977	loss	22148	62200211	70360798	Red_blood_cell_count	region1inRegion2	42598
CNVR98	1116	8	68684763	68710413	gain	22148	62200211	70360798	Red_blood_cell_count	region1inRegion2	25650
CNVR97	1112	8	64473379	64515977	loss	22149	62200211	70360798	Granulocyte_percentage	region1inRegion2	42598
CNVR98	1116	8	68684763	68710413	gain	22149	62200211	70360798	Granulocyte_percentage	region1inRegion2	25650
CNVR97	1112	8	64473379	64515977	loss	22150	62200211	70360798	Granulocyte_percentage	region1inRegion2	42598
CNVR98	1116	8	68684763	68710413	gain	22150	62200211	70360798	Granulocyte_percentage	region1inRegion2	25650
CNVR97	1112	8	64473379	64515977	loss	22169	62200211	74641551	Platelet_distribution_width	region1inRegion2	42598
CNVR98	1116	8	68684763	68710413	gain	22169	62200211	74641551	Platelet_distribution_width	region1inRegion2	25650
CNVR97	1112	8	64473379	64515977	loss	22141	62200211	74985007	Mean_corpuscular_volume	region1inRegion2	42598
CNVR98	1116	8	68684763	68710413	gain	22141	62200211	74985007	Mean_corpuscular_volume	region1inRegion2	25650
CNVR97	1112	8	64473379	64515977	loss	22142	62200211	74985007	Mean_corpuscular_volume	region1inRegion2	42598
CNVR98	1116	8	68684763	68710413	gain	22142	62200211	74985007	Mean_corpuscular_volume	region1inRegion2	25650
CNVR97	1112	8	64473379	64515977	loss	22143	62200211	74985007	Mean_corpuscular_volume	region1inRegion2	42598
CNVR98	1116	8	68684763	68710413	gain	22143	62200211	74985007	Mean_corpuscular_volume	region1inRegion2	25650
CNVR97	1112	8	64473379	64515977	loss	22146	62200211	79533086	Red_blood_cell_count	region1inRegion2	42598
CNVR98	1116	8	68684763	68710413	gain	22146	62200211	79533086	Red_blood_cell_count	region1inRegion2	25650
CNVR97	1112	8	64473379	64515977	loss	22166	62200211	80103382	Red_blood_cell_count	region1inRegion2	42598
CNVR98	1116	8	68684763	68710413	gain	22166	62200211	80103382	Red_blood_cell_count	region1inRegion2	25650
CNVR97	1112	8	64473379	64515977	loss	62291	63495558	100809900	Days_to_100_kg	region1inRegion2	42598
CNVR98	1116	8	68684763	68710413	gain	62291	63495558	100809900	Days_to_100_kg	region1inRegion2	25650
CNVR99	1129	8	85727251	85735420	gain	62291	63495558	100809900	Days_to_100_kg	region1inRegion2	8169
CNVR97	1112	8	64473379	64515977	loss	62292	63610798	100809900	Days_to_100_kg	region1inRegion2	42598
CNVR98	1116	8	68684763	68710413	gain	62292	63610798	100809900	Days_to_100_kg	region1inRegion2	25650
CNVR99	1129	8	85727251	85735420	gain	62292	63610798	100809900	Days_to_100_kg	region1inRegion2	8169
CNVR97	1112	8	64473379	64515977	loss	62293	63883183	100809900	Days_to_100_kg	region1inRegion2	42598
CNVR98	1116	8	68684763	68710413	gain	62293	63883183	100809900	Days_to_100_kg	region1inRegion2	25650
CNVR99	1129	8	85727251	85735420	gain	62293	63883183	100809900	Days_to_100_kg	region1inRegion2	8169
CNVR97	1112	8	64473379	64515977	loss	62294	63983428	100809900	Days_to_100_kg	region1inRegion2	42598
CNVR98	1116	8	68684763	68710413	gain	62294	63983428	100809900	Days_to_100_kg	region1inRegion2	25650
CNVR99	1129	8	85727251	85735420	gain	62294	63983428	100809900	Days_to_100_kg	region1inRegion2	8169
CNVR98	1116	8	68684763	68710413	gain	498	68687287	75933650	Average_daily_gain	overlapTail	23127
CNVR98	1116	8	68684763	68710413	gain	21508	68707136	68707176	Mean_corpuscular_volume	region2inRegion1	40
CNVR99	1129	8	85727251	85735420	gain	16930	72039691	120532322	Empty_body_lipid_content	region1inRegion2	8169
CNVR99	1129	8	85727251	85735420	gain	3264	72039691	120532322	Loin_muscle_area	region1inRegion2	8169
CNVR99	1129	8	85727251	85735420	gain	3999	72039691	135740155	Temperature_40_minutes_post_mortem_(loin)	region1inRegion2	8169
CNVR100	1169	8	122108843	122121928	loss	3999	72039691	135740155	Temperature_40_minutes_post_mortem_(loin)	region1inRegion2	13085
CNVR101	1176	8	129139072	129153237	loss	3999	72039691	135740155	Temperature_40_minutes_post_mortem_(loin)	region1inRegion2	14165
CNVR99	1129	8	85727251	85735420	gain	469	72039691	139007531	Palmitic_acid_content	region1inRegion2	8169
CNVR100	1169	8	122108843	122121928	loss	469	72039691	139007531	Palmitic_acid_content	region1inRegion2	13085

CNVR101	1176	8	129139072	129153237	loss	469	72039691	139007531	Palmitic_acid_content	region1inRegion2	14165
CNVR99	1129	8	85727251	85735420	gain	470	72039691	139007531	Palmitoleic_acid_content	region1inRegion2	8169
CNVR100	1169	8	122108843	122121928	loss	470	72039691	139007531	Palmitoleic_acid_content	region1inRegion2	13085
CNVR101	1176	8	129139072	129153237	loss	470	72039691	139007531	Palmitoleic_acid_content	region1inRegion2	14165
CNVR99	1129	8	85727251	85735420	gain	471	72039691	139007531	Average_chain_length	region1inRegion2	8169
CNVR100	1169	8	122108843	122121928	loss	471	72039691	139007531	Average_chain_length	region1inRegion2	13085
CNVR101	1176	8	129139072	129153237	loss	471	72039691	139007531	Average_chain_length	region1inRegion2	14165
CNVR99	1129	8	85727251	85735420	gain	589	76024128	111442127	Age_at_puberty	region1inRegion2	8169
CNVR99	1129	8	85727251	85735420	gain	17620	77429212	124156612	Toll-like_receptor_9_level	region1inRegion2	8169
CNVR100	1169	8	122108843	122121928	loss	17620	77429212	124156612	Toll-like_receptor_9_level	region1inRegion2	13085
CNVR99	1129	8	85727251	85735420	gain	5705	77429212	124156612	Fat_to_meat_ratio	region1inRegion2	8169
CNVR100	1169	8	122108843	122121928	loss	5705	77429212	124156612	Fat_to_meat_ratio	region1inRegion2	13085
CNVR99	1129	8	85727251	85735420	gain	5707	77429212	124156612	Estimated_carcass_lean_content	region1inRegion2	8169
CNVR100	1169	8	122108843	122121928	loss	5707	77429212	124156612	Estimated_carcass_lean_content	region1inRegion2	13085
CNVR99	1129	8	85727251	85735420	gain	17844	78781463	90653103	CD4-positive_leukocyte_percentage	region1inRegion2	8169
CNVR99	1129	8	85727251	85735420	gain	17845	78781463	90653103	CD4-positive/CD8-positive_leukocyte_ratio	region1inRegion2	8169
CNVR99	1129	8	85727251	85735420	gain	6553	84933990	85938521	Red_blood_cell_count	region1inRegion2	8169
CNVR99	1129	8	85727251	85735420	gain	18040	85006687	148491826	pH_24_hr_post_mortem_(ham)	region1inRegion2	8169
CNVR100	1169	8	122108843	122121928	loss	18040	85006687	148491826	pH_24_hr_post_mortem_(ham)	region1inRegion2	13085
CNVR101	1176	8	129139072	129153237	loss	18040	85006687	148491826	pH_24_hr_post_mortem_(ham)	region1inRegion2	14165
CNVR99	1129	8	85727251	85735420	gain	32116	85436831	86950848	Palmitic_acid_content	region1inRegion2	8169
CNVR100	1169	8	122108843	122121928	loss	8891	90653103	123337593	Ear_area	region1inRegion2	13085
CNVR100	1169	8	122108843	122121928	loss	8892	90653103	123337593	Ear_area	region1inRegion2	13085
CNVR100	1169	8	122108843	122121928	loss	296	99338918	130701968	Meat_color-a	region1inRegion2	13085
CNVR101	1176	8	129139072	129153237	loss	296	99338918	130701968	Meat_color-a	region1inRegion2	14165
CNVR100	1169	8	122108843	122121928	loss	18632	108610930	136396030	Fat_area_percentage_in_carcass	region1inRegion2	13085
CNVR101	1176	8	129139072	129153237	loss	18632	108610930	136396030	Fat_area_percentage_in_carcass	region1inRegion2	14165
CNVR100	1169	8	122108843	122121928	loss	8808	108610930	136396030	Teat_number	region1inRegion2	13085
CNVR101	1176	8	129139072	129153237	loss	8808	108610930	136396030	Teat_number	region1inRegion2	14165
CNVR100	1169	8	122108843	122121928	loss	17782	108610930	139007531	backfat_at_last_rib	region1inRegion2	13085
CNVR101	1176	8	129139072	129153237	loss	17782	108610930	139007531	backfat_at_last_rib	region1inRegion2	14165
CNVR100	1169	8	122108843	122121928	loss	21846	108610930	139007531	CIE-a*	region1inRegion2	13085
CNVR101	1176	8	129139072	129153237	loss	21846	108610930	139007531	CIE-a*	region1inRegion2	14165
CNVR100	1169	8	122108843	122121928	loss	8950	108610930	139007531	Physis_score	region1inRegion2	13085
CNVR101	1176	8	129139072	129153237	loss	8950	108610930	139007531	Physis_score	region1inRegion2	14165
CNVR100	1169	8	122108843	122121928	loss	2994	120532322	124156612	Myofibril_fragmentation_index	region1inRegion2	13085
CNVR100	1169	8	122108843	122121928	loss	3206	120532322	130701968	Backfat_at_tenth_rib	region1inRegion2	13085
CNVR101	1176	8	129139072	129153237	loss	3206	120532322	130701968	Backfat_at_tenth_rib	region1inRegion2	14165
CNVR100	1169	8	122108843	122121928	loss	3654	120532322	130701968	Backfat_linear_at_last_rib	region1inRegion2	13085
CNVR101	1176	8	129139072	129153237	loss	3654	120532322	130701968	Backfat_linear_at_last_rib	region1inRegion2	14165
CNVR100	1169	8	122108843	122121928	loss	3655	120532322	130701968	Total_body_fat_tissue_linear	region1inRegion2	13085
CNVR101	1176	8	129139072	129153237	loss	3655	120532322	130701968	Total_body_fat_tissue_linear	region1inRegion2	14165
CNVR100	1169	8	122108843	122121928	loss	5400	120532322	136739744	Red_blood_cell_count	region1inRegion2	13085
CNVR101	1176	8	129139072	129153237	loss	5400	120532322	136739744	Red_blood_cell_count	region1inRegion2	14165
CNVR100	1169	8	122108843	122121928	loss	5401	120532322	136739744	Red_blood_cell_count	region1inRegion2	13085
CNVR101	1176	8	129139072	129153237	loss	5401	120532322	136739744	Red_blood_cell_count	region1inRegion2	14165
CNVR100	1169	8	122108843	122121928	loss	5406	120532322	136739744	Red_blood_cell_count	region1inRegion2	13085
CNVR101	1176	8	129139072	129153237	loss	5406	120532322	136739744	Red_blood_cell_count	region1inRegion2	14165
CNVR100	1169	8	122108843	122121928	loss	5410	120532322	136739744	Red_blood_cell_count	region1inRegion2	13085
CNVR101	1176	8	129139072	129153237	loss	5410	120532322	136739744	Red_blood_cell_count	region1inRegion2	14165
CNVR100	1169	8	122108843	122121928	loss	5415	120532322	136739744	hematocrit	region1inRegion2	13085
CNVR101	1176	8	129139072	129153237	loss	5415	120532322	136739744	hematocrit	region1inRegion2	14165
CNVR100	1169	8	122108843	122121928	loss	5458	120532322	136739744	White_blood_cell_counts	region1inRegion2	13085
CNVR101	1176	8	129139072	129153237	loss	5458	120532322	136739744	White_blood_cell_counts	region1inRegion2	14165
CNVR100	1169	8	122108843	122121928	loss	7506	120532322	136739744	Sarcocystis_miescheriana_IgG_levels	region1inRegion2	13085
CNVR101	1176	8	129139072	129153237	loss	7506	120532322	136739744	Sarcocystis_miescheriana_IgG_levels	region1inRegion2	14165
CNVR100	1169	8	122108843	122121928	loss	9011	121362278	122208001	Cervical_vertebra_length	region1inRegion2	13085
CNVR100	1169	8	122108843	122121928	loss	32080	121443468	122235171	Palmitic_acid_content	region1inRegion2	13085
CNVR100	1169	8	122108843	122121928	loss	32121	121443468	125300406	Palmitic_acid_content	region1inRegion2	13085
CNVR100	1169	8	122108843	122121928	loss	62225	122210418	100809900	Feed_conversion_ratio	region2inRegion1	-21400518
CNVR100	1169	8	122108843	122121928	loss	62226	122235151	100809900	Feed_conversion_ratio	region2inRegion1	-21425251
CNVR100	1169	8	122108843	122121928	loss	62227	122257414	100809900	Feed_conversion_ratio	region2inRegion1	-21447514
CNVR101	1176	8	129139072	129153237	loss	8901	123337593	136739744	Ear_erectness	region1inRegion2	14165
CNVR101	1176	8	129139072	129153237	loss	684	123337593	147305244	Litter_size	region1inRegion2	14165
CNVR101	1176	8	129139072	129153237	loss	685	123337593	147305244	Total_number_born_alive	region1inRegion2	14165
CNVR101	1176	8	129139072	129153237	loss	5876	123381761	130795057	pH_for_Semimembranosus	region1inRegion2	14165
CNVR101	1176	8	129139072	129153237	loss	1084	124156612	133682376	Feed_conversion_ratio	region1inRegion2	14165
CNVR101	1176	8	129139072	129153237	loss	18025	124156612	139007531	Adipocyte_diameter	region1inRegion2	14165
CNVR101	1176	8	129139072	129153237	loss	2995	124156612	139007531	Marbling	region1inRegion2	14165
CNVR101	1176	8	129139072	129153237	loss	5702	124156612	139007531	Average_backfat_thickness	region1inRegion2	14165
CNVR101	1176	8	129139072	129153237	loss	5703	124156612	139007531	Shoulder_subcutaneous_fat_thickness	region1inRegion2	14165
CNVR101	1176	8	129139072	129153237	loss	5706	124156612	139007531	Belly_meat_content	region1inRegion2	14165
CNVR101	1176	8	129139072	129153237	loss	5708	124156612	139007531	Average_daily_gain	region1inRegion2	14165
CNVR101	1176	8	129139072	129153237	loss	5709	124156612	139007531	Dressing_percentage	region1inRegion2	14165
CNVR101	1176	8	129139072	129153237	loss	5947	124156612	139007531	Daily_feed_intake	region1inRegion2	14165
CNVR101	1176	8	129139072	129153237	loss	21715	124236417	138454719	CIE-b*	region1inRegion2	14165
CNVR100	1169	8	122108843	122121928	loss	62304	126044813	100809900	Days_to_100_kg	region2inRegion1	-25234913
CNVR101	1176	8	129139072	129153237	loss	590	127942565	136449791	Age_at_puberty	region1inRegion2	14165
CNVR101	1176	8	129139072	129153237	loss	8842	128204671	129329231	Number_of_stillborn	region1inRegion2	14165
CNVR100	1169	8	122108843	122121928	loss	62195	132448351	100809900	Average_daily_gain	region2inRegion1	-31638451
CNVR101	1176	8	129139072	129153237	loss	62195	132448351	100809900	Average_daily_gain	region2inRegion1	-31638451
CNVR100	1169	8	122108843	122121928	loss	62306	132448351	100809900	Days_to_100_kg	region2inRegion1	-31638451
CNVR101	1176	8	129139072	129153237	loss	62306	132448351	100809900	Days_to_100_kg	region2inRegion1	-31638451
CNVR100	1169	8	122108843	122121928	loss	62196	132518864	100809900	Average_daily_gain	region2inRegion1	-31708964
CNVR101	1176	8	129139072	129153237	loss	62196	132518864	100809900	Average_daily_gain	region2inRegion1	-31708964
CNVR100	1169	8	122108843	122121928	loss	62307	132518864	100809900	Days_to_100_kg	region2inRegion1	-31708964
CNVR101	1176	8	129139072	129153237	loss	62307	132518864	100809900	Days_to_100_kg	region2inRegion1	-31708964
CNVR100	1169	8	122108843	122121928	loss	62197	132553841	100809900	Average_daily_gain	region2inRegion1	-31743941
CNVR101	1176	8	129139072	129153237	loss	62197	132553841	100809900	Average_daily_gain	region2inRegion1	-31743941
CNVR100	1169	8	122108843	122121928	loss	62308	132553841	100809900	Days_to_100_kg	region2inRegion1	-31743941
CNVR101	1176	8	129139072	129153237	loss	62308	132553841	100809900	Days_to_100_kg	region2inRegion1	-31743941
CNVR100	1169	8	122108843	122121928	loss	62198	132773763	100809900	Average_daily_gain	region2inRegion1	-31963863
CNVR101	1176	8	129139072	129153237	loss	62198	132773763	100809900	Average_daily_gain	region2inRegion1	-31963863
CNVR100	1169	8	122108843	122121928	loss	62309	132773763	100809900	Days_to_100_kg	region2inRegion1	-31963863
CNVR101	1176	8	129139072	129153237	loss	62309	132773763	100809900	Days_to_100_kg	region2inRegion1	-31963863
CNVR100	1169	8	122108843	122121928	loss	62199	132795623	100809900	Average_daily_gain	region2inRegion1	-31985723
CNVR101	1176	8	129139072	129153237	loss	62199	132795623	100809900	Average_daily_gain	region2inRegion1	-31985723
CNVR100	1169	8	122108843	122121928	loss	62310	132795623	100809900	Days_to_100_kg	region2inRegion1	-31985723
CNVR101	1176	8	129139072	129153237	loss	62310	132795623	100809900	Days_to_100_kg	region2inRegion1	-31985723
CNVR100	1169	8	122108843	122121928	loss	62200	132934182	100809900	Average_daily_gain	region2inRegion1	-32124282
CNVR101	1176	8	129139072	129153237	loss	62200	132934182	100809900	Average_daily_gain	region2inRegion1	-32124282
CNVR100	1169	8	122108843	122121928	loss	62311	132934182	100809900	Days_to_100_kg	region2inRegion1	-32124282
CNVR101	1176	8	129139072	129153237	loss	62311	132934182	100809900	Days_to_100_kg	region2inRegion1	-32124282
CNVR100	1169	8	122108843	122121928	loss	62313	134615307	100809900	Days_to_100_kg	region2inRegion1	-33805407
CNVR101	1176	8	129139072	129153237	loss	62313	134615307	100809900	Days_to_100_kg	region2inRegion1	-33805407
CNVR100	1169	8	122108843	122121928	loss	62314	134978203	100809900	Days_to_100_kg	region2inRegion1	-34168303
CNVR101	1176	8	129139072	129153237	loss	62314	134978203	100809900	Days_to_100_kg	region2inRegion1	-34168303
CNVR102	1198	9	1963636	2010455	gain	16931	88508	11066889	body_weight_(22_weeks)	region1inRegion2	46819
CNVR103	1200	9	3614907	3618335	gain	16931	88508	11066889	body_weight_(22_weeks)	region1inRegion2	3428
CNVR104	1203	9	5206397	5225462	gain	16931	88508	11066889	body_weight_(22_weeks)	region1inRegion2	19065
CNVR105	1204	9	5488035	5492918	gain	16931	88508	11066889	body_weight_(22_weeks)	region1inRegion2	4883
CNVR106	1205	9	5747061	5810398	loss	16931	88508	11066889	body_weight_(22_weeks)	region1inRegion2	63337
CNVR102	1198	9	1963636	2010455	gain	16932	88508	11066889	Empty_body_lipid_content	region1inRegion2	46819
CNVR103	1200	9	3614907	3618335	gain	16932	88508	11066889	Empty_body_lipid_content	region1inRegion2	3428
CNVR104	1203	9	5206397	5225462	gain	16932	88508	11066889	Empty_body_lipid_content	region1inRegion2	19065
CNVR105	1204	9	5488035	5492918	gain	16932	88508	11066889	Empty_body_lipid_content	region1inRegion2	4883
CNVR106	1205	9	5747061	5810398	loss	16932	88508	11066889	Empty_body_lipid_content	region1inRegion2	63337
CNVR102	1198	9	1963636	2010455	gain	16933	88508	11066889	body_weight_(19_weeks)	region1inRegion2	46819
CNVR103	1200	9	3614907	3618335	gain	16933	88508	11066889	body_weight_(19_weeks)	region1inRegion2	3428
CNVR104	1203	9	5206397	5225462	gain	16933	88508	11066889	body_weight_(19_weeks)	region1inRegion2	19065
CNVR105	1204	9	5488035	5492918	gain	16933	88508	11066889	body_weight_(19_weeks)	region1inRegion2	4883
CNVR106	1205	9	5747061	5810398	loss	16933	88508	11066889	body_weight_(19_weeks)	region1inRegion2	63337

CNVR102	1198	9	1963636	2010455	gain	3186	88508	11066889	Empty_body_protein_content	region1inRegion2	46819
CNVR103	1200	9	3614907	3618335	gain	3186	88508	11066889	Empty_body_protein_content	region1inRegion2	3428
CNVR104	1203	9	5206397	5225462	gain	3186	88508	11066889	Empty_body_protein_content	region1inRegion2	19065
CNVR105	1204	9	5488035	5492918	gain	3186	88508	11066889	Empty_body_protein_content	region1inRegion2	4883
CNVR106	1205	9	5747061	5810398	loss	3186	88508	11066889	Empty_body_protein_content	region1inRegion2	63337
CNVR102	1198	9	1963636	2010455	gain	3187	88508	11066889	Days_to_105_kg	region1inRegion2	46819
CNVR103	1200	9	3614907	3618335	gain	3187	88508	11066889	Days_to_105_kg	region1inRegion2	3428
CNVR104	1203	9	5206397	5225462	gain	3187	88508	11066889	Days_to_105_kg	region1inRegion2	19065
CNVR105	1204	9	5488035	5492918	gain	3187	88508	11066889	Days_to_105_kg	region1inRegion2	4883
CNVR106	1205	9	5747061	5810398	loss	3187	88508	11066889	Days_to_105_kg	region1inRegion2	63337
CNVR102	1198	9	1963636	2010455	gain	3207	88508	11066889	Estimated_carcass_lean_content	region1inRegion2	46819
CNVR103	1200	9	3614907	3618335	gain	3207	88508	11066889	Estimated_carcass_lean_content	region1inRegion2	3428
CNVR104	1203	9	5206397	5225462	gain	3207	88508	11066889	Estimated_carcass_lean_content	region1inRegion2	19065
CNVR105	1204	9	5488035	5492918	gain	3207	88508	11066889	Estimated_carcass_lean_content	region1inRegion2	4883
CNVR106	1205	9	5747061	5810398	loss	3207	88508	11066889	Estimated_carcass_lean_content	region1inRegion2	63337
CNVR102	1198	9	1963636	2010455	gain	3266	88508	11066889	Drip_loss	region1inRegion2	46819
CNVR103	1200	9	3614907	3618335	gain	3266	88508	11066889	Drip_loss	region1inRegion2	3428
CNVR104	1203	9	5206397	5225462	gain	3266	88508	11066889	Drip_loss	region1inRegion2	19065
CNVR105	1204	9	5488035	5492918	gain	3266	88508	11066889	Drip_loss	region1inRegion2	4883
CNVR106	1205	9	5747061	5810398	loss	3266	88508	11066889	Drip_loss	region1inRegion2	63337
CNVR102	1198	9	1963636	2010455	gain	3267	88508	11066889	Carcass_weight_(hot)	region1inRegion2	46819
CNVR103	1200	9	3614907	3618335	gain	3267	88508	11066889	Carcass_weight_(hot)	region1inRegion2	3428
CNVR104	1203	9	5206397	5225462	gain	3267	88508	11066889	Carcass_weight_(hot)	region1inRegion2	19065
CNVR105	1204	9	5488035	5492918	gain	3267	88508	11066889	Carcass_weight_(hot)	region1inRegion2	4883
CNVR106	1205	9	5747061	5810398	loss	3267	88508	11066889	Carcass_weight_(hot)	region1inRegion2	63337
CNVR102	1198	9	1963636	2010455	gain	3268	88508	11066889	Body_weight_(slaughter)	region1inRegion2	46819
CNVR103	1200	9	3614907	3618335	gain	3268	88508	11066889	Body_weight_(slaughter)	region1inRegion2	3428
CNVR104	1203	9	5206397	5225462	gain	3268	88508	11066889	Body_weight_(slaughter)	region1inRegion2	19065
CNVR105	1204	9	5488035	5492918	gain	3268	88508	11066889	Body_weight_(slaughter)	region1inRegion2	4883
CNVR106	1205	9	5747061	5810398	loss	3268	88508	11066889	Body_weight_(slaughter)	region1inRegion2	63337
CNVR102	1198	9	1963636	2010455	gain	3269	88508	11066889	tenderness_score	region1inRegion2	46819
CNVR103	1200	9	3614907	3618335	gain	3269	88508	11066889	tenderness_score	region1inRegion2	3428
CNVR104	1203	9	5206397	5225462	gain	3269	88508	11066889	tenderness_score	region1inRegion2	19065
CNVR105	1204	9	5488035	5492918	gain	3269	88508	11066889	tenderness_score	region1inRegion2	4883
CNVR106	1205	9	5747061	5810398	loss	3269	88508	11066889	tenderness_score	region1inRegion2	63337
CNVR102	1198	9	1963636	2010455	gain	3270	88508	11066889	Ham_weight	region1inRegion2	46819
CNVR103	1200	9	3614907	3618335	gain	3270	88508	11066889	Ham_weight	region1inRegion2	3428
CNVR104	1203	9	5206397	5225462	gain	3270	88508	11066889	Ham_weight	region1inRegion2	19065
CNVR105	1204	9	5488035	5492918	gain	3270	88508	11066889	Ham_weight	region1inRegion2	4883
CNVR106	1205	9	5747061	5810398	loss	3270	88508	11066889	Ham_weight	region1inRegion2	63337
CNVR102	1198	9	1963636	2010455	gain	3309	88508	11066889	Carcass_temperature_(24_hr_post-mortem)_	region1inRegion2	46819
CNVR103	1200	9	3614907	3618335	gain	3309	88508	11066889	Carcass_temperature_(24_hr_post-mortem)_	region1inRegion2	3428
CNVR104	1203	9	5206397	5225462	gain	3309	88508	11066889	Carcass_temperature_(24_hr_post-mortem)_	region1inRegion2	19065
CNVR105	1204	9	5488035	5492918	gain	3309	88508	11066889	Carcass_temperature_(24_hr_post-mortem)_	region1inRegion2	4883
CNVR106	1205	9	5747061	5810398	loss	3309	88508	11066889	Carcass_temperature_(24_hr_post-mortem)_	region1inRegion2	63337
CNVR102	1198	9	1963636	2010455	gain	3315	88508	11066889	Fat_weight_(total)	region1inRegion2	46819
CNVR103	1200	9	3614907	3618335	gain	3315	88508	11066889	Fat_weight_(total)	region1inRegion2	3428
CNVR104	1203	9	5206397	5225462	gain	3315	88508	11066889	Fat_weight_(total)	region1inRegion2	19065
CNVR105	1204	9	5488035	5492918	gain	3315	88508	11066889	Fat_weight_(total)	region1inRegion2	4883
CNVR106	1205	9	5747061	5810398	loss	3315	88508	11066889	Fat_weight_(total)	region1inRegion2	63337
CNVR102	1198	9	1963636	2010455	gain	3318	88508	11066889	Empty_body_lipid_content	region1inRegion2	46819
CNVR103	1200	9	3614907	3618335	gain	3318	88508	11066889	Empty_body_lipid_content	region1inRegion2	3428
CNVR104	1203	9	5206397	5225462	gain	3318	88508	11066889	Empty_body_lipid_content	region1inRegion2	19065
CNVR105	1204	9	5488035	5492918	gain	3318	88508	11066889	Empty_body_lipid_content	region1inRegion2	4883
CNVR106	1205	9	5747061	5810398	loss	3318	88508	11066889	Empty_body_lipid_content	region1inRegion2	63337
CNVR102	1198	9	1963636	2010455	gain	3676	88508	11066889	backfat_at_last_rib	region1inRegion2	46819
CNVR103	1200	9	3614907	3618335	gain	3676	88508	11066889	backfat_at_last_rib	region1inRegion2	3428
CNVR104	1203	9	5206397	5225462	gain	3676	88508	11066889	backfat_at_last_rib	region1inRegion2	19065
CNVR105	1204	9	5488035	5492918	gain	3676	88508	11066889	backfat_at_last_rib	region1inRegion2	4883
CNVR106	1205	9	5747061	5810398	loss	3676	88508	11066889	backfat_at_last_rib	region1inRegion2	63337
CNVR102	1198	9	1963636	2010455	gain	3677	88508	11066889	Average_daily_gain	region1inRegion2	46819
CNVR103	1200	9	3614907	3618335	gain	3677	88508	11066889	Average_daily_gain	region1inRegion2	3428
CNVR104	1203	9	5206397	5225462	gain	3677	88508	11066889	Average_daily_gain	region1inRegion2	19065
CNVR105	1204	9	5488035	5492918	gain	3677	88508	11066889	Average_daily_gain	region1inRegion2	4883
CNVR106	1205	9	5747061	5810398	loss	3677	88508	11066889	Average_daily_gain	region1inRegion2	63337
CNVR102	1198	9	1963636	2010455	gain	3700	88508	11066889	Carcass_length	region1inRegion2	46819
CNVR103	1200	9	3614907	3618335	gain	3700	88508	11066889	Carcass_length	region1inRegion2	3428
CNVR104	1203	9	5206397	5225462	gain	3700	88508	11066889	Carcass_length	region1inRegion2	19065
CNVR105	1204	9	5488035	5492918	gain	3700	88508	11066889	Carcass_length	region1inRegion2	4883
CNVR106	1205	9	5747061	5810398	loss	3700	88508	11066889	Carcass_length	region1inRegion2	63337
CNVR102	1198	9	1963636	2010455	gain	3701	88508	11066889	Carcass_weight_(hot)	region1inRegion2	46819
CNVR103	1200	9	3614907	3618335	gain	3701	88508	11066889	Carcass_weight_(hot)	region1inRegion2	3428
CNVR104	1203	9	5206397	5225462	gain	3701	88508	11066889	Carcass_weight_(hot)	region1inRegion2	19065
CNVR105	1204	9	5488035	5492918	gain	3701	88508	11066889	Carcass_weight_(hot)	region1inRegion2	4883
CNVR106	1205	9	5747061	5810398	loss	3701	88508	11066889	Carcass_weight_(hot)	region1inRegion2	63337
CNVR102	1198	9	1963636	2010455	gain	3702	88508	11066889	PH_for_Longissmus_dorsi	region1inRegion2	46819
CNVR103	1200	9	3614907	3618335	gain	3702	88508	11066889	PH_for_Longissmus_dorsi	region1inRegion2	3428
CNVR104	1203	9	5206397	5225462	gain	3702	88508	11066889	PH_for_Longissmus_dorsi	region1inRegion2	19065
CNVR105	1204	9	5488035	5492918	gain	3702	88508	11066889	PH_for_Longissmus_dorsi	region1inRegion2	4883
CNVR106	1205	9	5747061	5810398	loss	3702	88508	11066889	PH_for_Longissmus_dorsi	region1inRegion2	63337
CNVR102	1198	9	1963636	2010455	gain	3703	88508	11066889	pH_for_Semimembranosus	region1inRegion2	46819
CNVR103	1200	9	3614907	3618335	gain	3703	88508	11066889	pH_for_Semimembranosus	region1inRegion2	3428
CNVR104	1203	9	5206397	5225462	gain	3703	88508	11066889	pH_for_Semimembranosus	region1inRegion2	19065
CNVR105	1204	9	5488035	5492918	gain	3703	88508	11066889	pH_for_Semimembranosus	region1inRegion2	4883
CNVR106	1205	9	5747061	5810398	loss	3703	88508	11066889	pH_for_Semimembranosus	region1inRegion2	63337
CNVR102	1198	9	1963636	2010455	gain	4155	88508	11066889	Average_daily_gain	region1inRegion2	46819
CNVR103	1200	9	3614907	3618335	gain	4155	88508	11066889	Average_daily_gain	region1inRegion2	3428
CNVR104	1203	9	5206397	5225462	gain	4155	88508	11066889	Average_daily_gain	region1inRegion2	19065
CNVR105	1204	9	5488035	5492918	gain	4155	88508	11066889	Average_daily_gain	region1inRegion2	4883
CNVR106	1205	9	5747061	5810398	loss	4155	88508	11066889	Average_daily_gain	region1inRegion2	63337
CNVR102	1198	9	1963636	2010455	gain	4156	88508	11066889	Lean_meat_percentage	region1inRegion2	46819
CNVR103	1200	9	3614907	3618335	gain	4156	88508	11066889	Lean_meat_percentage	region1inRegion2	3428
CNVR104	1203	9	5206397	5225462	gain	4156	88508	11066889	Lean_meat_percentage	region1inRegion2	19065
CNVR105	1204	9	5488035	5492918	gain	4156	88508	11066889	Lean_meat_percentage	region1inRegion2	4883
CNVR106	1205	9	5747061	5810398	loss	4156	88508	11066889	Lean_meat_percentage	region1inRegion2	63337
CNVR102	1198	9	1963636	2010455	gain	4157	88508	11066889	PH_for_Longissmus_dorsi	region1inRegion2	46819
CNVR103	1200	9	3614907	3618335	gain	4157	88508	11066889	PH_for_Longissmus_dorsi	region1inRegion2	3428
CNVR104	1203	9	5206397	5225462	gain	4157	88508	11066889	PH_for_Longissmus_dorsi	region1inRegion2	19065
CNVR105	1204	9	5488035	5492918	gain	4157	88508	11066889	PH_for_Longissmus_dorsi	region1inRegion2	4883
CNVR106	1205	9	5747061	5810398	loss	4157	88508	11066889	PH_for_Longissmus_dorsi	region1inRegion2	63337
CNVR102	1198	9	1963636	2010455	gain	4158	88508	11066889	pH_for_Semimembranosus	region1inRegion2	46819
CNVR103	1200	9	3614907	3618335	gain	4158	88508	11066889	pH_for_Semimembranosus	region1inRegion2	3428
CNVR104	1203	9	5206397	5225462	gain	4158	88508	11066889	pH_for_Semimembranosus	region1inRegion2	19065
CNVR105	1204	9	5488035	5492918	gain	4158	88508	11066889	pH_for_Semimembranosus	region1inRegion2	4883
CNVR106	1205	9	5747061	5810398	loss	4158	88508	11066889	pH_for_Semimembranosus	region1inRegion2	63337
CNVR102	1198	9	1963636	2010455	gain	4159	88508	11066889	Conductivity_24_hours_postmortem_(ham)	region1inRegion2	46819
CNVR103	1200	9	3614907	3618335	gain	4159	88508	11066889	Conductivity_24_hours_postmortem_(ham)	region1inRegion2	3428
CNVR104	1203	9	5206397	5225462	gain	4159	88508	11066889	Conductivity_24_hours_postmortem_(ham)	region1inRegion2	19065
CNVR105	1204	9	5488035	5492918	gain	4159	88508	11066889	Conductivity_24_hours_postmortem_(ham)	region1inRegion2	4883
CNVR106	1205	9	5747061	5810398	loss	4159	88508	11066889	Conductivity_24_hours_postmortem_(ham)	region1inRegion2	63337
CNVR102	1198	9	1963636	2010455	gain	4195	88508	11066889	Conductivity_45_minutes_post-mortem	region1inRegion2	46819
CNVR103	1200	9	3614907	3618335	gain	4195	88508	11066889	Conductivity_45_minutes_post-mortem	region1inRegion2	3428
CNVR104	1203	9	5206397	5225462	gain	4195	88508	11066889	Conductivity_45_minutes_post-mortem	region1inRegion2	19065
CNVR105	1204	9	5488035	5492918	gain	4195	88508	11066889	Conductivity_45_minutes_post-mortem	region1inRegion2	4883
CNVR106	1205	9	5747061	5810398	loss	4195	88508	11066889	Conductivity_45_minutes_post-mortem	region1inRegion2	63337
CNVR102	1198	9	1963636	2010455	gain	4196	88508	11066889	Conductivity_24_hours_postmortem_(ham)	region1inRegion2	46819
CNVR103	1200	9	3614907	3618335	gain	4196	88508	11066889	Conductivity_24_hours_postmortem_(ham)	region1inRegion2	3428
CNVR104	1203	9	5206397	5225462	gain	4196	88508	11066889	Conductivity_24_hours_postmortem_(ham)	region1inRegion2	19065
CNVR105	1204	9	5488035	5492918	gain	4196	88508	11066889	Conductivity_24_hours_postmortem_(ham)	region1inRegion2	4883
CNVR106	1205	9	5747061	5810398	loss	4196	88508	11066889	Conductivity_24_hours_postmortem_(ham)	region1inRegion2	63337
CNVR102	1198	9	1963636	2010455	gain	4247	88508	11066889	Body_weight_(slaughter)	region1inRegion2	46819
CNVR103	1200	9	3614907	3618335	gain	4247	88508	11066889	Body_weight_(slaughter)	region1inRegion2	3428

CNVR104	1203	9	5206397	5225462	gain	4247	88508	11066889	Body_weight_(slaughter)	region1inRegion2	19065
CNVR105	1204	9	5488035	5492918	gain	4247	88508	11066889	Body_weight_(slaughter)	region1inRegion2	4883
CNVR106	1205	9	5747061	5810398	loss	4247	88508	11066889	Body_weight_(slaughter)	region1inRegion2	63337
CNVR102	1198	9	1963636	2010455	gain	4248	88508	11066889	Carcass_weight_(hot)	region1inRegion2	46819
CNVR103	1200	9	3614907	3618335	gain	4248	88508	11066889	Carcass_weight_(hot)	region1inRegion2	3428
CNVR104	1203	9	5206397	5225462	gain	4248	88508	11066889	Carcass_weight_(hot)	region1inRegion2	19065
CNVR105	1204	9	5488035	5492918	gain	4248	88508	11066889	Carcass_weight_(hot)	region1inRegion2	4883
CNVR106	1205	9	5747061	5810398	loss	4248	88508	11066889	Carcass_weight_(hot)	region1inRegion2	63337
CNVR102	1198	9	1963636	2010455	gain	21396	88508	23144816	Ham_weight	region1inRegion2	46819
CNVR103	1200	9	3614907	3618335	gain	21396	88508	23144816	Ham_weight	region1inRegion2	3428
CNVR104	1203	9	5206397	5225462	gain	21396	88508	23144816	Ham_weight	region1inRegion2	19065
CNVR105	1204	9	5488035	5492918	gain	21396	88508	23144816	Ham_weight	region1inRegion2	4883
CNVR106	1205	9	5747061	5810398	loss	21396	88508	23144816	Ham_weight	region1inRegion2	63337
CNVR107	1227	9	21980507	21992085	gain	21396	88508	23144816	Ham_weight	region1inRegion2	11578
CNVR102	1198	9	1963636	2010455	gain	3271	88508	23144816	Shear_force	region1inRegion2	46819
CNVR103	1200	9	3614907	3618335	gain	3271	88508	23144816	Shear_force	region1inRegion2	3428
CNVR104	1203	9	5206397	5225462	gain	3271	88508	23144816	Shear_force	region1inRegion2	19065
CNVR105	1204	9	5488035	5492918	gain	3271	88508	23144816	Shear_force	region1inRegion2	4883
CNVR106	1205	9	5747061	5810398	loss	3271	88508	23144816	Shear_force	region1inRegion2	63337
CNVR107	1227	9	21980507	21992085	gain	3271	88508	23144816	Shear_force	region1inRegion2	11578
CNVR102	1198	9	1963636	2010455	gain	3272	88508	23144816	tenderness_score	region1inRegion2	46819
CNVR103	1200	9	3614907	3618335	gain	3272	88508	23144816	tenderness_score	region1inRegion2	3428
CNVR104	1203	9	5206397	5225462	gain	3272	88508	23144816	tenderness_score	region1inRegion2	19065
CNVR105	1204	9	5488035	5492918	gain	3272	88508	23144816	tenderness_score	region1inRegion2	4883
CNVR106	1205	9	5747061	5810398	loss	3272	88508	23144816	tenderness_score	region1inRegion2	63337
CNVR107	1227	9	21980507	21992085	gain	3272	88508	23144816	tenderness_score	region1inRegion2	11578
CNVR102	1198	9	1963636	2010455	gain	5213	88508	124560160	backfat_at_mid-back	region1inRegion2	46819
CNVR103	1200	9	3614907	3618335	gain	5213	88508	124560160	backfat_at_mid-back	region1inRegion2	3428
CNVR104	1203	9	5206397	5225462	gain	5213	88508	124560160	backfat_at_mid-back	region1inRegion2	19065
CNVR105	1204	9	5488035	5492918	gain	5213	88508	124560160	backfat_at_mid-back	region1inRegion2	4883
CNVR106	1205	9	5747061	5810398	loss	5213	88508	124560160	backfat_at_mid-back	region1inRegion2	63337
CNVR107	1227	9	21980507	21992085	gain	5213	88508	124560160	backfat_at_mid-back	region1inRegion2	11578
CNVR108	1234	9	27756709	27768971	gain	5213	88508	124560160	backfat_at_mid-back	region1inRegion2	12262
CNVR109	1261	9	56272660	56332060	gain	5213	88508	124560160	backfat_at_mid-back	region1inRegion2	59400
CNVR110	1262	9	56901872	56950458	loss	5213	88508	124560160	backfat_at_mid-back	region1inRegion2	48586
CNVR111	1316	9	108875949	108885208	gain	5213	88508	124560160	backfat_at_mid-back	region1inRegion2	9259
CNVR112	1331	9	119585370	119593230	gain	5213	88508	124560160	backfat_at_mid-back	region1inRegion2	7860
CNVR113	1338	9	124260883	124271069	gain	5213	88508	124560160	backfat_at_mid-back	region1inRegion2	10186
CNVR102	1198	9	1963636	2010455	gain	5246	88508	139717105	backfat_at_mid-back	region1inRegion2	46819
CNVR103	1200	9	3614907	3618335	gain	5246	88508	139717105	backfat_at_mid-back	region1inRegion2	3428
CNVR104	1203	9	5206397	5225462	gain	5246	88508	139717105	backfat_at_mid-back	region1inRegion2	19065
CNVR105	1204	9	5488035	5492918	gain	5246	88508	139717105	backfat_at_mid-back	region1inRegion2	4883
CNVR106	1205	9	5747061	5810398	loss	5246	88508	139717105	backfat_at_mid-back	region1inRegion2	63337
CNVR107	1227	9	21980507	21992085	gain	5246	88508	139717105	backfat_at_mid-back	region1inRegion2	11578
CNVR108	1234	9	27756709	27768971	gain	5246	88508	139717105	backfat_at_mid-back	region1inRegion2	12262
CNVR109	1261	9	56272660	56332060	gain	5246	88508	139717105	backfat_at_mid-back	region1inRegion2	59400
CNVR110	1262	9	56901872	56950458	loss	5246	88508	139717105	backfat_at_mid-back	region1inRegion2	48586
CNVR111	1316	9	108875949	108885208	gain	5246	88508	139717105	backfat_at_mid-back	region1inRegion2	9259
CNVR112	1331	9	119585370	119593230	gain	5246	88508	139717105	backfat_at_mid-back	region1inRegion2	7860
CNVR113	1338	9	124260883	124271069	gain	5246	88508	139717105	backfat_at_mid-back	region1inRegion2	10186
CNVR114	1339	9	127267298	127291319	gain	5246	88508	139717105	backfat_at_mid-back	region1inRegion2	24021
CNVR115	1346	9	136449280	136453167	gain	5246	88508	139717105	backfat_at_mid-back	region1inRegion2	3887
CNVR102	1198	9	1963636	2010455	gain	5205	88508	147173381	backfat_above_muscle_dorsi	region1inRegion2	46819
CNVR103	1200	9	3614907	3618335	gain	5205	88508	147173381	backfat_above_muscle_dorsi	region1inRegion2	3428
CNVR104	1203	9	5206397	5225462	gain	5205	88508	147173381	backfat_above_muscle_dorsi	region1inRegion2	19065
CNVR105	1204	9	5488035	5492918	gain	5205	88508	147173381	backfat_above_muscle_dorsi	region1inRegion2	4883
CNVR106	1205	9	5747061	5810398	loss	5205	88508	147173381	backfat_above_muscle_dorsi	region1inRegion2	63337
CNVR107	1227	9	21980507	21992085	gain	5205	88508	147173381	backfat_above_muscle_dorsi	region1inRegion2	11578
CNVR108	1234	9	27756709	27768971	gain	5205	88508	147173381	backfat_above_muscle_dorsi	region1inRegion2	12262
CNVR109	1261	9	56272660	56332060	gain	5205	88508	147173381	backfat_above_muscle_dorsi	region1inRegion2	59400
CNVR110	1262	9	56901872	56950458	loss	5205	88508	147173381	backfat_above_muscle_dorsi	region1inRegion2	48586
CNVR111	1316	9	108875949	108885208	gain	5205	88508	147173381	backfat_above_muscle_dorsi	region1inRegion2	9259
CNVR112	1331	9	119585370	119593230	gain	5205	88508	147173381	backfat_above_muscle_dorsi	region1inRegion2	7860
CNVR113	1338	9	124260883	124271069	gain	5205	88508	147173381	backfat_above_muscle_dorsi	region1inRegion2	10186
CNVR114	1339	9	127267298	127291319	gain	5205	88508	147173381	backfat_above_muscle_dorsi	region1inRegion2	24021
CNVR115	1346	9	136449280	136453167	gain	5205	88508	147173381	backfat_above_muscle_dorsi	region1inRegion2	3887
CNVR103	1200	9	3614907	3618335	gain	95478	3330061	4377887	Total_number_born_alive	region1inRegion2	3428
CNVR103	1200	9	3614907	3618335	gain	95689	3330061	4377887	backfat_between_last_rib_and_first_lumbar	region1inRegion2	3428
CNVR103	1200	9	3614907	3618335	gain	95690	3330061	4377887	Loin_muscle_area	region1inRegion2	3428
CNVR104	1203	9	5206397	5225462	gain	5872	4722562	5571409	pH_for_Semimembranosus	region1inRegion2	19065
CNVR105	1204	9	5488035	5492918	gain	5872	4722562	5571409	pH_for_Semimembranosus	region1inRegion2	4883
CNVR104	1203	9	5206397	5225462	gain	27503	5218767	5218807	hematocrit	region2inRegion1	40
CNVR106	1205	9	5747061	5810398	loss	95376	5631264	6815282	cis-11-Eicosenoic_acid_content	region1inRegion2	63337
CNVR106	1205	9	5747061	5810398	loss	27519	5784282	5784322	hematocrit	region2inRegion1	40
CNVR106	1205	9	5747061	5810398	loss	27830	5784282	5784322	Red_blood_cell_count	region2inRegion1	40
CNVR107	1227	9	21980507	21992085	gain	38097	7523504	147506758	Muscle_moisture_percentage	region1inRegion2	11578
CNVR108	1234	9	27756709	27768971	gain	38097	7523504	147506758	Muscle_moisture_percentage	region1inRegion2	12262
CNVR109	1261	9	56272660	56332060	gain	38097	7523504	147506758	Muscle_moisture_percentage	region1inRegion2	59400
CNVR110	1262	9	56901872	56950458	loss	38097	7523504	147506758	Muscle_moisture_percentage	region1inRegion2	48586
CNVR111	1316	9	108875949	108885208	gain	38097	7523504	147506758	Muscle_moisture_percentage	region1inRegion2	9259
CNVR112	1331	9	119585370	119593230	gain	38097	7523504	147506758	Muscle_moisture_percentage	region1inRegion2	7860
CNVR113	1338	9	124260883	124271069	gain	38097	7523504	147506758	Muscle_moisture_percentage	region1inRegion2	10186
CNVR114	1339	9	127267298	127291319	gain	38097	7523504	147506758	Muscle_moisture_percentage	region1inRegion2	24021
CNVR115	1346	9	136449280	136453167	gain	38097	7523504	147506758	Muscle_moisture_percentage	region1inRegion2	3887
CNVR107	1227	9	21980507	21992085	gain	38098	7523504	147506758	CIE-a*	region1inRegion2	11578
CNVR108	1234	9	27756709	27768971	gain	38098	7523504	147506758	CIE-a*	region1inRegion2	12262
CNVR109	1261	9	56272660	56332060	gain	38098	7523504	147506758	CIE-a*	region1inRegion2	59400
CNVR110	1262	9	56901872	56950458	loss	38098	7523504	147506758	CIE-a*	region1inRegion2	48586
CNVR111	1316	9	108875949	108885208	gain	38098	7523504	147506758	CIE-a*	region1inRegion2	9259
CNVR112	1331	9	119585370	119593230	gain	38098	7523504	147506758	CIE-a*	region1inRegion2	7860
CNVR113	1338	9	124260883	124271069	gain	38098	7523504	147506758	CIE-a*	region1inRegion2	10186
CNVR114	1339	9	127267298	127291319	gain	38098	7523504	147506758	CIE-a*	region1inRegion2	24021
CNVR115	1346	9	136449280	136453167	gain	38098	7523504	147506758	CIE-a*	region1inRegion2	3887
CNVR107	1227	9	21980507	21992085	gain	38099	7523504	147506758	CIE-L*	region1inRegion2	11578
CNVR108	1234	9	27756709	27768971	gain	38099	7523504	147506758	CIE-L*	region1inRegion2	12262
CNVR109	1261	9	56272660	56332060	gain	38099	7523504	147506758	CIE-L*	region1inRegion2	59400
CNVR110	1262	9	56901872	56950458	loss	38099	7523504	147506758	CIE-L*	region1inRegion2	48586
CNVR111	1316	9	108875949	108885208	gain	38099	7523504	147506758	CIE-L*	region1inRegion2	9259
CNVR112	1331	9	119585370	119593230	gain	38099	7523504	147506758	CIE-L*	region1inRegion2	7860
CNVR113	1338	9	124260883	124271069	gain	38099	7523504	147506758	CIE-L*	region1inRegion2	10186
CNVR114	1339	9	127267298	127291319	gain	38099	7523504	147506758	CIE-L*	region1inRegion2	24021
CNVR115	1346	9	136449280	136453167	gain	38099	7523504	147506758	CIE-L*	region1inRegion2	3887
CNVR107	1227	9	21980507	21992085	gain	18644	11066889	23144816	Leptin_level	region1inRegion2	11578
CNVR107	1227	9	21980507	21992085	gain	5149	11066889	23144816	Belly_fat_area	region1inRegion2	11578
CNVR107	1227	9	21980507	21992085	gain	5711	11066889	23144816	Body_weight_(weaning)	region1inRegion2	11578
CNVR107	1227	9	21980507	21992085	gain	5712	11066889	23144816	Average_daily_gain	region1inRegion2	11578
CNVR107	1227	9	21980507	21992085	gain	5713	11066889	23144816	Average_daily_gain	region1inRegion2	11578
CNVR107	1227	9	21980507	21992085	gain	5910	11066889	23144816	Time_spent_feeding	region1inRegion2	11578
CNVR107	1227	9	21980507	21992085	gain	5919	11066889	23144816	Time_spent_rooting	region1inRegion2	11578
CNVR107	1227	9	21980507	21992085	gain	5434	11066889	34499270	Average_backfat_thickness	region1inRegion2	11578
CNVR108	1234	9	27756709	27768971	gain	5434	11066889	34499270	Average_backfat_thickness	region1inRegion2	12262
CNVR107	1227	9	21980507	21992085	gain	8902	11066889	45282513	Ear_erectness	region1inRegion2	11578
CNVR108	1234	9	27756709	27768971	gain	8902	11066889	45282513	Ear_erectness	region1inRegion2	12262
CNVR107	1227	9	21980507	21992085	gain	7462	11066889	48711260	Nonfunctional_nipples	region1inRegion2	11578
CNVR108	1234	9	27756709	27768971	gain	7462	11066889	48711260	Nonfunctional_nipples	region1inRegion2	12262
CNVR107	1227	9	21980507	21992085	gain	18663	13715322	26786140	skatole,_laboratory	region1inRegion2	11578
CNVR107	1227	9	21980507	21992085	gain	21262	15638023	137469520	Leaf_fat_weight	region1inRegion2	11578
CNVR108	1234	9	27756709	27768971	gain	21262	15638023	137469520	Leaf_fat_weight	region1inRegion2	12262
CNVR109	1261	9	56272660	56332060	gain	21262	15638023	137469520	Leaf_fat_weight	region1inRegion2	59400
CNVR110	1262	9	56901872	56950458	loss	21262	15638023	137469520	Leaf_fat_weight	region1inRegion2	48586
CNVR111	1316	9	108875949	108885208	gain	21262	15638023	137469520	Leaf_fat_weight	region1inRegion2	9259
CNVR112	1331	9	119585370	119593230	gain	21262	15638023	137469520	Leaf_fat_weight	region1inRegion2	7860

CNVR113	1338	9	124260883	124271069	gain	21262	15638023	137469520	Leaf_fat_weight	region1inRegion2	10186
CNVR114	1339	9	127267298	127291319	gain	21262	15638023	137469520	Leaf_fat_weight	region1inRegion2	24021
CNVR115	1346	9	136449280	136453167	gain	21262	15638023	137469520	Leaf_fat_weight	region1inRegion2	3887
CNVR107	1227	9	21980507	21992085	gain	3782	18659348	23144816	Loin_muscle_area	region1inRegion2	11578
CNVR107	1227	9	21980507	21992085	gain	3783	18659348	23144816	Body_weight_(16_days)	region1inRegion2	11578
CNVR107	1227	9	21980507	21992085	gain	41	18659348	34499270	Leaf_fat_weight	region1inRegion2	11578
CNVR108	1234	9	27756709	27768971	gain	41	18659348	34499270	Leaf_fat_weight	region1inRegion2	12262
CNVR107	1227	9	21980507	21992085	gain	7721	21160262	24472004	HDL_cholesterol	region1inRegion2	11578
CNVR108	1234	9	27756709	27768971	gain	37543	23144816	36198230	CSFV_antibody_level	region1inRegion2	12262
CNVR108	1234	9	27756709	27768971	gain	1104	23144816	36456616	Liver_weight	region1inRegion2	12262
CNVR108	1234	9	27756709	27768971	gain	3273	23144816	45282513	Muscle_fat_content	region1inRegion2	12262
CNVR108	1234	9	27756709	27768971	gain	279	23144816	47461005	Average_daily_gain	region1inRegion2	12262
CNVR108	1234	9	27756709	27768971	gain	4210	23144816	65059079	androstenone,_laboratory	region1inRegion2	12262
CNVR109	1261	9	56272660	56332060	gain	4210	23144816	65059079	androstenone,_laboratory	region1inRegion2	59400
CNVR110	1262	9	56901872	56950458	loss	4210	23144816	65059079	androstenone,_laboratory	region1inRegion2	48586
CNVR108	1234	9	27756709	27768971	gain	4000	23144816	129142103	pH_40_minutes_post_mortem_(ham)	region1inRegion2	12262
CNVR109	1261	9	56272660	56332060	gain	4000	23144816	129142103	pH_40_minutes_post_mortem_(ham)	region1inRegion2	59400
CNVR110	1262	9	56901872	56950458	loss	4000	23144816	129142103	pH_40_minutes_post_mortem_(ham)	region1inRegion2	48586
CNVR111	1316	9	108875949	108885208	gain	4000	23144816	129142103	pH_40_minutes_post_mortem_(ham)	region1inRegion2	9259
CNVR112	1331	9	119585370	119593230	gain	4000	23144816	129142103	pH_40_minutes_post_mortem_(ham)	region1inRegion2	7860
CNVR113	1338	9	124260883	124271069	gain	4000	23144816	129142103	pH_40_minutes_post_mortem_(ham)	region1inRegion2	10186
CNVR114	1339	9	127267298	127291319	gain	4000	23144816	129142103	pH_40_minutes_post_mortem_(ham)	region1inRegion2	24021
CNVR108	1234	9	27756709	27768971	gain	7580	23144816	153670197	Melanoma_susceptibility	region1inRegion2	12262
CNVR109	1261	9	56272660	56332060	gain	7580	23144816	153670197	Melanoma_susceptibility	region1inRegion2	59400
CNVR110	1262	9	56901872	56950458	loss	7580	23144816	153670197	Melanoma_susceptibility	region1inRegion2	48586
CNVR111	1316	9	108875949	108885208	gain	7580	23144816	153670197	Melanoma_susceptibility	region1inRegion2	9259
CNVR112	1331	9	119585370	119593230	gain	7580	23144816	153670197	Melanoma_susceptibility	region1inRegion2	7860
CNVR113	1338	9	124260883	124271069	gain	7580	23144816	153670197	Melanoma_susceptibility	region1inRegion2	10186
CNVR114	1339	9	127267298	127291319	gain	7580	23144816	153670197	Melanoma_susceptibility	region1inRegion2	24021
CNVR115	1346	9	136449280	136453167	gain	7580	23144816	153670197	Melanoma_susceptibility	region1inRegion2	3887
CNVR108	1234	9	27756709	27768971	gain	15852	24624544	71387138	Palmitoleic_acid_content	region1inRegion2	12262
CNVR109	1261	9	56272660	56332060	gain	15852	24624544	71387138	Palmitoleic_acid_content	region1inRegion2	59400
CNVR110	1262	9	56901872	56950458	loss	15852	24624544	71387138	Palmitoleic_acid_content	region1inRegion2	48586
CNVR108	1234	9	27756709	27768971	gain	125483	27564748	98136454	Intramuscular_fat_content	region1inRegion2	12262
CNVR109	1261	9	56272660	56332060	gain	125483	27564748	98136454	Intramuscular_fat_content	region1inRegion2	59400
CNVR110	1262	9	56901872	56950458	loss	125483	27564748	98136454	Intramuscular_fat_content	region1inRegion2	48586
CNVR108	1234	9	27756709	27768971	gain	9035	27651964	28179642	Intramuscular_fat_content	region1inRegion2	12262
CNVR109	1261	9	56272660	56332060	gain	517	39568811	138751051	Corpus_luteum_number	region1inRegion2	59400
CNVR110	1262	9	56901872	56950458	loss	517	39568811	138751051	Corpus_luteum_number	region1inRegion2	48586
CNVR111	1316	9	108875949	108885208	gain	517	39568811	138751051	Corpus_luteum_number	region1inRegion2	9259
CNVR112	1331	9	119585370	119593230	gain	517	39568811	138751051	Corpus_luteum_number	region1inRegion2	7860
CNVR113	1338	9	124260883	124271069	gain	517	39568811	138751051	Corpus_luteum_number	region1inRegion2	10186
CNVR114	1339	9	127267298	127291319	gain	517	39568811	138751051	Corpus_luteum_number	region1inRegion2	24021
CNVR115	1346	9	136449280	136453167	gain	517	39568811	138751051	Corpus_luteum_number	region1inRegion2	3887
CNVR109	1261	9	56272660	56332060	gain	21261	39568811	143559357	Backfat_at_first_rib	region1inRegion2	59400
CNVR110	1262	9	56901872	56950458	loss	21261	39568811	143559357	Backfat_at_first_rib	region1inRegion2	48586
CNVR111	1316	9	108875949	108885208	gain	21261	39568811	143559357	Backfat_at_first_rib	region1inRegion2	9259
CNVR112	1331	9	119585370	119593230	gain	21261	39568811	143559357	Backfat_at_first_rib	region1inRegion2	7860
CNVR113	1338	9	124260883	124271069	gain	21261	39568811	143559357	Backfat_at_first_rib	region1inRegion2	10186
CNVR114	1339	9	127267298	127291319	gain	21261	39568811	143559357	Backfat_at_first_rib	region1inRegion2	24021
CNVR115	1346	9	136449280	136453167	gain	21261	39568811	143559357	Backfat_at_first_rib	region1inRegion2	3887
CNVR109	1261	9	56272660	56332060	gain	21258	40935645	144549282	Average_backfat_thickness	region1inRegion2	59400
CNVR110	1262	9	56901872	56950458	loss	21258	40935645	144549282	Average_backfat_thickness	region1inRegion2	48586
CNVR111	1316	9	108875949	108885208	gain	21258	40935645	144549282	Average_backfat_thickness	region1inRegion2	9259
CNVR112	1331	9	119585370	119593230	gain	21258	40935645	144549282	Average_backfat_thickness	region1inRegion2	7860
CNVR113	1338	9	124260883	124271069	gain	21258	40935645	144549282	Average_backfat_thickness	region1inRegion2	10186
CNVR114	1339	9	127267298	127291319	gain	21258	40935645	144549282	Average_backfat_thickness	region1inRegion2	24021
CNVR115	1346	9	136449280	136453167	gain	21258	40935645	144549282	Average_backfat_thickness	region1inRegion2	3887
CNVR109	1261	9	56272660	56332060	gain	5130	45282513	56664370	Shoulder_weight	region1inRegion2	59400
CNVR109	1261	9	56272660	56332060	gain	5131	45282513	56664370	Shoulder_weight	region1inRegion2	59400
CNVR109	1261	9	56272660	56332060	gain	3274	45282513	63050049	Muscle_moisture_percentage	region1inRegion2	59400
CNVR110	1262	9	56901872	56950458	loss	3274	45282513	63050049	Muscle_moisture_percentage	region1inRegion2	48586
CNVR109	1261	9	56272660	56332060	gain	3818	45282513	67062987	Off-Flavor_Score	region1inRegion2	59400
CNVR110	1262	9	56901872	56950458	loss	3818	45282513	67062987	Off-Flavor_Score	region1inRegion2	48586
CNVR109	1261	9	56272660	56332060	gain	5168	45282513	109605017	Shoulder_weight	region1inRegion2	59400
CNVR110	1262	9	56901872	56950458	loss	5168	45282513	109605017	Shoulder_weight	region1inRegion2	48586
CNVR111	1316	9	108875949	108885208	gain	5168	45282513	109605017	Shoulder_weight	region1inRegion2	9259
CNVR109	1261	9	56272660	56332060	gain	5178	45282513	109605017	Loin_muscle_area	region1inRegion2	59400
CNVR110	1262	9	56901872	56950458	loss	5178	45282513	109605017	Loin_muscle_area	region1inRegion2	48586
CNVR111	1316	9	108875949	108885208	gain	5178	45282513	109605017	Loin_muscle_area	region1inRegion2	9259
CNVR109	1261	9	56272660	56332060	gain	5179	45282513	109605017	Shoulder_weight	region1inRegion2	59400
CNVR110	1262	9	56901872	56950458	loss	5179	45282513	109605017	Shoulder_weight	region1inRegion2	48586
CNVR111	1316	9	108875949	108885208	gain	5179	45282513	109605017	Shoulder_weight	region1inRegion2	9259
CNVR109	1261	9	56272660	56332060	gain	5189	45282513	109605017	External_fat_on_loin	region1inRegion2	59400
CNVR110	1262	9	56901872	56950458	loss	5189	45282513	109605017	External_fat_on_loin	region1inRegion2	48586
CNVR111	1316	9	108875949	108885208	gain	5189	45282513	109605017	External_fat_on_loin	region1inRegion2	9259
CNVR109	1261	9	56272660	56332060	gain	5190	45282513	109605017	External_fat_on_ham	region1inRegion2	59400
CNVR110	1262	9	56901872	56950458	loss	5190	45282513	109605017	External_fat_on_ham	region1inRegion2	48586
CNVR111	1316	9	108875949	108885208	gain	5190	45282513	109605017	External_fat_on_ham	region1inRegion2	9259
CNVR109	1261	9	56272660	56332060	gain	5192	45282513	109605017	Side_fat_thickness	region1inRegion2	59400
CNVR110	1262	9	56901872	56950458	loss	5192	45282513	109605017	Side_fat_thickness	region1inRegion2	48586
CNVR111	1316	9	108875949	108885208	gain	5192	45282513	109605017	Side_fat_thickness	region1inRegion2	9259
CNVR109	1261	9	56272660	56332060	gain	5196	45282513	109605017	Average_daily_gain	region1inRegion2	59400
CNVR110	1262	9	56901872	56950458	loss	5196	45282513	109605017	Average_daily_gain	region1inRegion2	48586
CNVR111	1316	9	108875949	108885208	gain	5196	45282513	109605017	Average_daily_gain	region1inRegion2	9259
CNVR109	1261	9	56272660	56332060	gain	3812	45282513	128983346	Off-Flavor_Score	region1inRegion2	59400
CNVR110	1262	9	56901872	56950458	loss	3812	45282513	128983346	Off-Flavor_Score	region1inRegion2	48586
CNVR111	1316	9	108875949	108885208	gain	3812	45282513	128983346	Off-Flavor_Score	region1inRegion2	9259
CNVR112	1331	9	119585370	119593230	gain	3812	45282513	128983346	Off-Flavor_Score	region1inRegion2	7860
CNVR113	1338	9	124260883	124271069	gain	3812	45282513	128983346	Off-Flavor_Score	region1inRegion2	10186
CNVR114	1339	9	127267298	127291319	gain	3812	45282513	128983346	Off-Flavor_Score	region1inRegion2	24021
CNVR109	1261	9	56272660	56332060	gain	3806	45282513	139744874	Drip_loss	region1inRegion2	59400
CNVR110	1262	9	56901872	56950458	loss	3806	45282513	139744874	Drip_loss	region1inRegion2	48586
CNVR111	1316	9	108875949	108885208	gain	3806	45282513	139744874	Drip_loss	region1inRegion2	9259
CNVR112	1331	9	119585370	119593230	gain	3806	45282513	139744874	Drip_loss	region1inRegion2	7860
CNVR113	1338	9	124260883	124271069	gain	3806	45282513	139744874	Drip_loss	region1inRegion2	10186
CNVR114	1339	9	127267298	127291319	gain	3806	45282513	139744874	Drip_loss	region1inRegion2	24021
CNVR115	1346	9	136449280	136453167	gain	3806	45282513	139744874	Drip_loss	region1inRegion2	3887
CNVR109	1261	9	56272660	56332060	gain	3807	45282513	139744874	Meat_color-L	region1inRegion2	59400
CNVR110	1262	9	56901872	56950458	loss	3807	45282513	139744874	Meat_color-L	region1inRegion2	48586
CNVR111	1316	9	108875949	108885208	gain	3807	45282513	139744874	Meat_color-L	region1inRegion2	9259
CNVR112	1331	9	119585370	119593230	gain	3807	45282513	139744874	Meat_color-L	region1inRegion2	7860
CNVR113	1338	9	124260883	124271069	gain	3807	45282513	139744874	Meat_color-L	region1inRegion2	10186
CNVR114	1339	9	127267298	127291319	gain	3807	45282513	139744874	Meat_color-L	region1inRegion2	24021
CNVR115	1346	9	136449280	136453167	gain	3807	45282513	139744874	Meat_color-L	region1inRegion2	3887
CNVR109	1261	9	56272660	56332060	gain	3817	45282513	139744874	Meat_color-L	region1inRegion2	59400
CNVR110	1262	9	56901872	56950458	loss	3817	45282513	139744874	Meat_color-L	region1inRegion2	48586
CNVR111	1316	9	108875949	108885208	gain	3817	45282513	139744874	Meat_color-L	region1inRegion2	9259
CNVR112	1331	9	119585370	119593230	gain	3817	45282513	139744874	Meat_color-L	region1inRegion2	7860
CNVR113	1338	9	124260883	124271069	gain	3817	45282513	139744874	Meat_color-L	region1inRegion2	10186
CNVR114	1339	9	127267298	127291319	gain	3817	45282513	139744874	Meat_color-L	region1inRegion2	24021
CNVR115	1346	9	136449280	136453167	gain	3817	45282513	139744874	Meat_color-L	region1inRegion2	3887
CNVR109	1261	9	56272660	56332060	gain	3110	46682251	72981639	Palmitoleic_acid_content	region1inRegion2	59400
CNVR110	1262	9	56901872	56950458	loss	3110	46682251	72981639	Palmitoleic_acid_content	region1inRegion2	48586
CNVR109	1261	9	56272660	56332060	gain	3112	46682251	72981639	Stearic_acid_content	region1inRegion2	59400
CNVR110	1262	9	56901872	56950458	loss	3112	46682251	72981639	Stearic_acid_content	region1inRegion2	48586
CNVR109	1261	9	56272660	56332060	gain	3115	46682251	72981639	Saturated_fatty_acid_content	region1inRegion2	59400
CNVR110	1262	9	56901872	56950458	loss	3115	46682251	72981639	Saturated_fatty_acid_content	region1inRegion2	48586
CNVR109	1261	9	56272660	56332060	gain	3120	46682251	72981639	Stearic_acid_content	region1inRegion2	59400
CNVR110	1262	9	56901872	56950458	loss	3120	46682251	72981639	Stearic_acid_content	region1inRegion2	48586
CNVR109	1261	9	56272660	56332060	gain	16854	47461005	78039423	Average_daily_gain	region1inRegion2	59400
CNVR110	1262	9	56901872	56950458	loss	16854	47461005	78039423	Average_daily_gain	region1inRegion2	48586

CNVR109	1261	9	56272660	56332060	gain	5644	48711260	65059079	Ear_size	region1inRegion2	59400
CNVR110	1262	9	56901872	56950458	loss	5644	48711260	65059079	Ear_size	region1inRegion2	48586
CNVR109	1261	9	56272660	56332060	gain	1101	48711260	78039423	Feed_intake	region1inRegion2	59400
CNVR110	1262	9	56901872	56950458	loss	1101	48711260	78039423	Feed_intake	region1inRegion2	48586
CNVR109	1261	9	56272660	56332060	gain	392	52235142	130913986	Shoulder_weight	region1inRegion2	59400
CNVR110	1262	9	56901872	56950458	loss	392	52235142	130913986	Shoulder_weight	region1inRegion2	48586
CNVR111	1316	9	108875949	108885208	gain	392	52235142	130913986	Shoulder_weight	region1inRegion2	9259
CNVR112	1331	9	119585370	119593230	gain	392	52235142	130913986	Shoulder_weight	region1inRegion2	7860
CNVR113	1338	9	124260883	124271069	gain	392	52235142	130913986	Shoulder_weight	region1inRegion2	10186
CNVR114	1339	9	127267298	127291319	gain	392	52235142	130913986	Shoulder_weight	region1inRegion2	24021
CNVR109	1261	9	56272660	56332060	gain	6326	53311036	128983346	CO2_partial_pressure	region1inRegion2	59400
CNVR110	1262	9	56901872	56950458	loss	6326	53311036	128983346	CO2_partial_pressure	region1inRegion2	48586
CNVR111	1316	9	108875949	108885208	gain	6326	53311036	128983346	CO2_partial_pressure	region1inRegion2	9259
CNVR112	1331	9	119585370	119593230	gain	6326	53311036	128983346	CO2_partial_pressure	region1inRegion2	7860
CNVR113	1338	9	124260883	124271069	gain	6326	53311036	128983346	CO2_partial_pressure	region1inRegion2	10186
CNVR114	1339	9	127267298	127291319	gain	6326	53311036	128983346	CO2_partial_pressure	region1inRegion2	24021
CNVR109	1261	9	56272660	56332060	gain	3888	55759062	99689175	Shear_force	region1inRegion2	59400
CNVR110	1262	9	56901872	56950458	loss	3888	55759062	99689175	Shear_force	region1inRegion2	48586
CNVR109	1261	9	56272660	56332060	gain	126669	55939358	56830250	Teat_number,_difference_between_sides	region1inRegion2	59400
CNVR110	1262	9	56901872	56950458	loss	2813	56664370	126320226	Diameter_of_type_I_muscle_fibers	region1inRegion2	48586
CNVR111	1316	9	108875949	108885208	gain	2813	56664370	126320226	Diameter_of_type_I_muscle_fibers	region1inRegion2	9259
CNVR112	1331	9	119585370	119593230	gain	2813	56664370	126320226	Diameter_of_type_I_muscle_fibers	region1inRegion2	7860
CNVR113	1338	9	124260883	124271069	gain	2813	56664370	126320226	Diameter_of_type_I_muscle_fibers	region1inRegion2	10186
CNVR110	1262	9	56901872	56950458	loss	5710	56664370	126320226	Loin_muscle_area	region1inRegion2	48586
CNVR111	1316	9	108875949	108885208	gain	5710	56664370	126320226	Loin_muscle_area	region1inRegion2	9259
CNVR112	1331	9	119585370	119593230	gain	5710	56664370	126320226	Loin_muscle_area	region1inRegion2	7860
CNVR113	1338	9	124260883	124271069	gain	5710	56664370	126320226	Loin_muscle_area	region1inRegion2	10186
CNVR110	1262	9	56901872	56950458	loss	9646	56664370	126320226	Haptoglobin_concentration	region1inRegion2	48586
CNVR111	1316	9	108875949	108885208	gain	9646	56664370	126320226	Haptoglobin_concentration	region1inRegion2	9259
CNVR112	1331	9	119585370	119593230	gain	9646	56664370	126320226	Haptoglobin_concentration	region1inRegion2	7860
CNVR113	1338	9	124260883	124271069	gain	9646	56664370	126320226	Haptoglobin_concentration	region1inRegion2	10186
CNVR111	1316	9	108875949	108885208	gain	3208	63050049	123462124	Backfat_at_tenth_rib	region1inRegion2	9259
CNVR112	1331	9	119585370	119593230	gain	3208	63050049	123462124	Backfat_at_tenth_rib	region1inRegion2	7860
CNVR111	1316	9	108875949	108885208	gain	5649	65059079	126320226	Ear_erectness	region1inRegion2	9259
CNVR112	1331	9	119585370	119593230	gain	5649	65059079	126320226	Ear_erectness	region1inRegion2	7860
CNVR113	1338	9	124260883	124271069	gain	5649	65059079	126320226	Ear_erectness	region1inRegion2	10186
CNVR111	1316	9	108875949	108885208	gain	21260	66763557	141668522	Body_weight_(3_weeks)	region1inRegion2	9259
CNVR112	1331	9	119585370	119593230	gain	21260	66763557	141668522	Body_weight_(3_weeks)	region1inRegion2	7860
CNVR113	1338	9	124260883	124271069	gain	21260	66763557	141668522	Body_weight_(3_weeks)	region1inRegion2	10186
CNVR114	1339	9	127267298	127291319	gain	21260	66763557	141668522	Body_weight_(3_weeks)	region1inRegion2	24021
CNVR115	1346	9	136449280	136453167	gain	21260	66763557	141668522	Body_weight_(3_weeks)	region1inRegion2	3887
CNVR111	1316	9	108875949	108885208	gain	5150	71832311	118712399	External_fat_on_ham	region1inRegion2	9259
CNVR111	1316	9	108875949	108885208	gain	5143	71832311	119109362	Neck_weight	region1inRegion2	9259
CNVR111	1316	9	108875949	108885208	gain	21337	72981639	126320226	Immunoglobulin_G_level	region1inRegion2	9259
CNVR112	1331	9	119585370	119593230	gain	21337	72981639	126320226	Immunoglobulin_G_level	region1inRegion2	7860
CNVR113	1338	9	124260883	124271069	gain	21337	72981639	126320226	Immunoglobulin_G_level	region1inRegion2	10186
CNVR111	1316	9	108875949	108885208	gain	21259	74024794	140670466	Abdominal_fat_weight	region1inRegion2	9259
CNVR112	1331	9	119585370	119593230	gain	21259	74024794	140670466	Abdominal_fat_weight	region1inRegion2	7860
CNVR113	1338	9	124260883	124271069	gain	21259	74024794	140670466	Abdominal_fat_weight	region1inRegion2	10186
CNVR114	1339	9	127267298	127291319	gain	21259	74024794	140670466	Abdominal_fat_weight	region1inRegion2	24021
CNVR115	1346	9	136449280	136453167	gain	21259	74024794	140670466	Abdominal_fat_weight	region1inRegion2	3887
CNVR111	1316	9	108875949	108885208	gain	3887	77691779	129393898	Carcass_length	region1inRegion2	9259
CNVR112	1331	9	119585370	119593230	gain	3887	77691779	129393898	Carcass_length	region1inRegion2	7860
CNVR113	1338	9	124260883	124271069	gain	3887	77691779	129393898	Carcass_length	region1inRegion2	10186
CNVR114	1339	9	127267298	127291319	gain	3887	77691779	129393898	Carcass_length	region1inRegion2	24021
CNVR111	1316	9	108875949	108885208	gain	5163	77778360	125340885	Average_daily_gain	region1inRegion2	9259
CNVR112	1331	9	119585370	119593230	gain	5163	77778360	125340885	Average_daily_gain	region1inRegion2	7860
CNVR113	1338	9	124260883	124271069	gain	5163	77778360	125340885	Average_daily_gain	region1inRegion2	10186
CNVR111	1316	9	108875949	108885208	gain	5154	99846341	126482554	Protein_accretion_rate	region1inRegion2	9259
CNVR112	1331	9	119585370	119593230	gain	5154	99846341	126482554	Protein_accretion_rate	region1inRegion2	7860
CNVR113	1338	9	124260883	124271069	gain	5154	99846341	126482554	Protein_accretion_rate	region1inRegion2	10186
CNVR111	1316	9	108875949	108885208	gain	5151	100753612	110490081	Shoulder_external_fat_weight	region1inRegion2	9259
CNVR112	1331	9	119585370	119593230	gain	5155	114324299	123523128	Lipid_accretion_rate	region1inRegion2	7860
CNVR112	1331	9	119585370	119593230	gain	139189	119384249	120290466	Backfat_between_3rd_and_4th_last_ribs	region1inRegion2	7860
CNVR113	1338	9	124260883	124271069	gain	15851	121006106	129242149	Linoleic_acid_content	region1inRegion2	10186
CNVR114	1339	9	127267298	127291319	gain	15851	121006106	129242149	Linoleic_acid_content	region1inRegion2	24021
CNVR113	1338	9	124260883	124271069	gain	17848	123462124	128983346	CD4-positive_leukocyte_percentage	region1inRegion2	10186
CNVR114	1339	9	127267298	127291319	gain	17848	123462124	128983346	CD4-positive_leukocyte_percentage	region1inRegion2	24021
CNVR113	1338	9	124260883	124271069	gain	3275	123462124	142664837	Meat_color-b	region1inRegion2	10186
CNVR114	1339	9	127267298	127291319	gain	3275	123462124	142664837	Meat_color-b	region1inRegion2	24021
CNVR115	1346	9	136449280	136453167	gain	3275	123462124	142664837	Meat_color-b	region1inRegion2	3887
CNVR113	1338	9	124260883	124271069	gain	3310	123462124	142664837	Carcass_temperature_(45_minutes_post-mortem)	region1inRegion2	10186
CNVR114	1339	9	127267298	127291319	gain	3310	123462124	142664837	Carcass_temperature_(45_minutes_post-mortem)	region1inRegion2	24021
CNVR115	1346	9	136449280	136453167	gain	3310	123462124	142664837	Carcass_temperature_(45_minutes_post-mortem)	region1inRegion2	3887
CNVR114	1339	9	127267298	127291319	gain	55876	124804232	131826674	Cryptorchidism	region1inRegion2	24021
CNVR114	1339	9	127267298	127291319	gain	11568	126320226	139744874	Salmonella_count_in_spleen	region1inRegion2	24021
CNVR115	1346	9	136449280	136453167	gain	11568	126320226	139744874	Salmonella_count_in_spleen	region1inRegion2	3887
CNVR114	1339	9	127267298	127291319	gain	223	126320226	139744874	Shear_force	region1inRegion2	24021
CNVR115	1346	9	136449280	136453167	gain	223	126320226	139744874	Shear_force	region1inRegion2	3887
CNVR115	1346	9	136449280	136453167	gain	1106	127323435	139744874	Fat-cuts_percentage	region1inRegion2	3887
CNVR115	1346	9	136449280	136453167	gain	12294	128983346	139744874	Mean_corpuscular_volume	region1inRegion2	3887
CNVR115	1346	9	136449280	136453167	gain	21859	128983346	139744874	pH_24_hr_post-mortem_(loin)	region1inRegion2	3887
CNVR115	1346	9	136449280	136453167	gain	5156	128983346	139744874	Empty_body_protein_content	region1inRegion2	3887
CNVR115	1346	9	136449280	136453167	gain	5157	128983346	139744874	Empty_body_protein_content	region1inRegion2	3887
CNVR115	1346	9	136449280	136453167	gain	5158	128983346	139744874	Empty_body_lipid_content	region1inRegion2	3887
CNVR115	1346	9	136449280	136453167	gain	15056	128983346	147506758	Hemoglobin	region1inRegion2	3887
CNVR115	1346	9	136449280	136453167	gain	15057	128983346	147506758	Mean_corpuscular_volume	region1inRegion2	3887
CNVR115	1346	9	136449280	136453167	gain	15058	128983346	147506758	Mean_corpuscular_hemoglobin_content	region1inRegion2	3887
CNVR115	1346	9	136449280	136453167	gain	15059	128983346	147506758	Mean_corpuscular_hemoglobin_concentration	region1inRegion2	3887
CNVR115	1346	9	136449280	136453167	gain	29704	128983346	147506758	body_weight_(10_weeks)	region1inRegion2	3887
CNVR115	1346	9	136449280	136453167	gain	29705	128983346	147506758	Carcass_weight_(hot)	region1inRegion2	3887
CNVR115	1346	9	136449280	136453167	gain	29706	128983346	147506758	Carcass_length	region1inRegion2	3887
CNVR115	1346	9	136449280	136453167	gain	6382	128983346	147506758	Potassium_level	region1inRegion2	3887
CNVR115	1346	9	136449280	136453167	gain	7017	128983346	151394450	Diameter_of_type_IIa_muscle_fibers	region1inRegion2	3887
CNVR116	1380	10	22431073	22524502	gain	12339	664729	32088890	Change_in_interferon-gamma_level	region1inRegion2	93429
CNVR117	1381	10	23114909	23120232	loss-gain	12339	664729	32088890	Change_in_interferon-gamma_level	region1inRegion2	5323
CNVR116	1380	10	22431073	22524502	gain	17605	664729	32088890	Interleukin_10_level	region1inRegion2	93429
CNVR117	1381	10	23114909	23120232	loss-gain	17605	664729	32088890	Interleukin_10_level	region1inRegion2	5323
CNVR116	1380	10	22431073	22524502	gain	2814	664729	32088890	Number_of_capillaries_per_muscle_fiber	region1inRegion2	93429
CNVR117	1381	10	23114909	23120232	loss-gain	2814	664729	32088890	Number_of_capillaries_per_muscle_fiber	region1inRegion2	5323
CNVR116	1380	10	22431073	22524502	gain	2815	664729	32088890	Number_of_capillaries_per_type_IIa_fiber	region1inRegion2	93429
CNVR117	1381	10	23114909	23120232	loss-gain	2815	664729	32088890	Number_of_capillaries_per_type_IIa_fiber	region1inRegion2	5323
CNVR116	1380	10	22431073	22524502	gain	5715	664729	32088890	Average_daily_gain	region1inRegion2	93429
CNVR117	1381	10	23114909	23120232	loss-gain	5715	664729	32088890	Average_daily_gain	region1inRegion2	5323
CNVR116	1380	10	22431073	22524502	gain	5714	664729	61209673	Average_daily_gain	region1inRegion2	93429
CNVR117	1381	10	23114909	23120232	loss-gain	5714	664729	61209673	Average_daily_gain	region1inRegion2	5323
CNVR118	1416	10	55474331	55489205	gain	5714	664729	61209673	Average_daily_gain	region1inRegion2	14874
CNVR116	1380	10	22431073	22524502	gain	38101	664729	72237308	Shear_force	region1inRegion2	93429
CNVR117	1381	10	23114909	23120232	loss-gain	38101	664729	72237308	Shear_force	region1inRegion2	5323
CNVR118	1416	10	55474331	55489205	gain	38101	664729	72237308	Shear_force	region1inRegion2	14874
CNVR119	1427	10	71830224	71835029	gain	38101	664729	72237308	Shear_force	region1inRegion2	4805
CNVR116	1380	10	22431073	22524502	gain	38102	664729	72237308	CIE-a*	region1inRegion2	93429
CNVR117	1381	10	23114909	23120232	loss-gain	38102	664729	72237308	CIE-a*	region1inRegion2	5323
CNVR118	1416	10	55474331	55489205	gain	38102	664729	72237308	CIE-a*	region1inRegion2	14874
CNVR119	1427	10	71830224	71835029	gain	38102	664729	72237308	CIE-a*	region1inRegion2	4805
CNVR116	1380	10	22431073	22524502	gain	38103	664729	72237308	CIE-b*	region1inRegion2	93429
CNVR117	1381	10	23114909	23120232	loss-gain	38103	664729	72237308	CIE-b*	region1inRegion2	5323
CNVR118	1416	10	55474331	55489205	gain	38103	664729	72237308	CIE-b*	region1inRegion2	14874
CNVR119	1427	10	71830224	71835029	gain	38103	664729	72237308	CIE-b*	region1inRegion2	4805
CNVR116	1380	10	22431073	22524502	gain	127221	2303500	54996430	Mean_corpuscular_hemoglobin_concentration	region1inRegion2	93429
CNVR117	1381	10	23114909	23120232	loss-gain	127221	2303500	54996430	Mean_corpuscular_hemoglobin_concentration	region1inRegion2	5323
CNVR116	1380	10	22431073	22524502	gain	5253	3653029	70044415	Shoulder_subcutaneous_fat_thickness	region1inRegion2	93429

CNVR117	1381	10	23114909	23120232	loss-gain	5253	3653029	70044415	Shoulder_subcutaneous_fat_thickness	region1inRegion2	5323
CNVR118	1416	10	55474331	55489205	gain	5253	3653029	70044415	Shoulder_subcutaneous_fat_thickness	region1inRegion2	14874
CNVR116	1380	10	22431073	22524502	gain	5247	4388728	66625232	backfat_at_mid-back	region1inRegion2	93429
CNVR117	1381	10	23114909	23120232	loss-gain	5247	4388728	66625232	backfat_at_mid-back	region1inRegion2	5323
CNVR118	1416	10	55474331	55489205	gain	5247	4388728	66625232	backfat_at_mid-back	region1inRegion2	14874
CNVR116	1380	10	22431073	22524502	gain	7012	4476972	32088890	Percentage_type_I_fibers	region1inRegion2	93429
CNVR117	1381	10	23114909	23120232	loss-gain	7012	4476972	32088890	Percentage_type_I_fibers	region1inRegion2	5323
CNVR116	1380	10	22431073	22524502	gain	7026	4476972	32088890	Percentage_type_I_fibers	region1inRegion2	93429
CNVR117	1381	10	23114909	23120232	loss-gain	7026	4476972	32088890	Percentage_type_I_fibers	region1inRegion2	5323
CNVR116	1380	10	22431073	22524502	gain	7034	4476972	32088890	Percentage_type_IIa_fibers	region1inRegion2	93429
CNVR117	1381	10	23114909	23120232	loss-gain	7034	4476972	32088890	Percentage_type_IIa_fibers	region1inRegion2	5323
CNVR116	1380	10	22431073	22524502	gain	17642	5484101	68845076	Osteochondrosis_score	region1inRegion2	93429
CNVR117	1381	10	23114909	23120232	loss-gain	17642	5484101	68845076	Osteochondrosis_score	region1inRegion2	5323
CNVR118	1416	10	55474331	55489205	gain	17642	5484101	68845076	Osteochondrosis_score	region1inRegion2	14874
CNVR116	1380	10	22431073	22524502	gain	38100	6401430	72237308	Marbling	region1inRegion2	93429
CNVR117	1381	10	23114909	23120232	loss-gain	38100	6401430	72237308	Marbling	region1inRegion2	5323
CNVR118	1416	10	55474331	55489205	gain	38100	6401430	72237308	Marbling	region1inRegion2	14874
CNVR119	1427	10	71830224	71835029	gain	38100	6401430	72237308	Marbling	region1inRegion2	4805
CNVR116	1380	10	22431073	22524502	gain	7589	9308741	28168636	Melanoma_susceptibility	region1inRegion2	93429
CNVR117	1381	10	23114909	23120232	loss-gain	7589	9308741	28168636	Melanoma_susceptibility	region1inRegion2	5323
CNVR116	1380	10	22431073	22524502	gain	3939	9981187	25917876	Average_daily_lean_meat_gain	region1inRegion2	93429
CNVR117	1381	10	23114909	23120232	loss-gain	3939	9981187	25917876	Average_daily_lean_meat_gain	region1inRegion2	5323
CNVR116	1380	10	22431073	22524502	gain	130398	10002733	54996430	Litter_size	region1inRegion2	93429
CNVR117	1381	10	23114909	23120232	loss-gain	130398	10002733	54996430	Litter_size	region1inRegion2	5323
CNVR116	1380	10	22431073	22524502	gain	5259	10524710	44992400	Teat_number	region1inRegion2	93429
CNVR117	1381	10	23114909	23120232	loss-gain	5259	10524710	44992400	Teat_number	region1inRegion2	5323
CNVR116	1380	10	22431073	22524502	gain	21309	10565671	73121733	Vertebra_number	region1inRegion2	93429
CNVR117	1381	10	23114909	23120232	loss-gain	21309	10565671	73121733	Vertebra_number	region1inRegion2	5323
CNVR118	1416	10	55474331	55489205	gain	21309	10565671	73121733	Vertebra_number	region1inRegion2	14874
CNVR119	1427	10	71830224	71835029	gain	21309	10565671	73121733	Vertebra_number	region1inRegion2	4805
CNVR116	1380	10	22431073	22524502	gain	15863	10672278	25729904	Linoleic_acid_content	region1inRegion2	93429
CNVR117	1381	10	23114909	23120232	loss-gain	15863	10672278	25729904	Linoleic_acid_content	region1inRegion2	5323
CNVR116	1380	10	22431073	22524502	gain	3820	11093314	24563421	Marbling	region1inRegion2	93429
CNVR117	1381	10	23114909	23120232	loss-gain	3820	11093314	24563421	Marbling	region1inRegion2	5323
CNVR116	1380	10	22431073	22524502	gain	16856	11093314	31786469	Average_daily_gain	region1inRegion2	93429
CNVR117	1381	10	23114909	23120232	loss-gain	16856	11093314	31786469	Average_daily_gain	region1inRegion2	5323
CNVR116	1380	10	22431073	22524502	gain	16871	11093314	31786469	Carcass_weight_(hot)	region1inRegion2	93429
CNVR117	1381	10	23114909	23120232	loss-gain	16871	11093314	31786469	Carcass_weight_(hot)	region1inRegion2	5323
CNVR116	1380	10	22431073	22524502	gain	250	11732502	49141527	Average_daily_gain	region1inRegion2	93429
CNVR117	1381	10	23114909	23120232	loss-gain	250	11732502	49141527	Average_daily_gain	region1inRegion2	5323
CNVR116	1380	10	22431073	22524502	gain	15864	16987451	28168636	Stearic_acid_content	region1inRegion2	93429
CNVR117	1381	10	23114909	23120232	loss-gain	15864	16987451	28168636	Stearic_acid_content	region1inRegion2	5323
CNVR116	1380	10	22431073	22524502	gain	1108	16987451	34108676	Dressing_percentage	region1inRegion2	93429
CNVR117	1381	10	23114909	23120232	loss-gain	1108	16987451	34108676	Dressing_percentage	region1inRegion2	5323
CNVR116	1380	10	22431073	22524502	gain	8943	16987451	41334738	Gait_score_(hind)	region1inRegion2	93429
CNVR117	1381	10	23114909	23120232	loss-gain	8943	16987451	41334738	Gait_score_(hind)	region1inRegion2	5323
CNVR116	1380	10	22431073	22524502	gain	21310	17046083	72237308	Lumbar_vertebra_number	region1inRegion2	93429
CNVR117	1381	10	23114909	23120232	loss-gain	21310	17046083	72237308	Lumbar_vertebra_number	region1inRegion2	5323
CNVR118	1416	10	55474331	55489205	gain	21310	17046083	72237308	Lumbar_vertebra_number	region1inRegion2	14874
CNVR119	1427	10	71830224	71835029	gain	21310	17046083	72237308	Lumbar_vertebra_number	region1inRegion2	4805
CNVR116	1380	10	22431073	22524502	gain	15859	20117212	26483565	Stearic_acid_content	region1inRegion2	93429
CNVR117	1381	10	23114909	23120232	loss-gain	15859	20117212	26483565	Stearic_acid_content	region1inRegion2	5323
CNVR116	1380	10	22431073	22524502	gain	15860	20117212	28674613	Oleic_acid_content	region1inRegion2	93429
CNVR117	1381	10	23114909	23120232	loss-gain	15860	20117212	28674613	Oleic_acid_content	region1inRegion2	5323
CNVR118	1416	10	55474331	55489205	gain	3961	24563421	72237308	Semimembranosus_angle	region1inRegion2	14874
CNVR119	1427	10	71830224	71835029	gain	3961	24563421	72237308	Semimembranosus_angle	region1inRegion2	4805
CNVR118	1416	10	55474331	55489205	gain	518	25917876	67676064	Corpus_luteum_number	region1inRegion2	14874
CNVR118	1416	10	55474331	55489205	gain	15074	30374254	56168199	Creatinine_level	region1inRegion2	14874
CNVR118	1416	10	55474331	55489205	gain	5193	31508179	61045271	External_fat_on_loin	region1inRegion2	14874
CNVR118	1416	10	55474331	55489205	gain	5948	32088890	61209673	Body_weight_(slaughter)	region1inRegion2	14874
CNVR118	1416	10	55474331	55489205	gain	6010	32088890	61209673	Conductivity_24_hours_postmortem_(ham)	region1inRegion2	14874
CNVR118	1416	10	55474331	55489205	gain	6011	32088890	61209673	Shear_force	region1inRegion2	14874
CNVR118	1416	10	55474331	55489205	gain	7031	32088890	64951351	Percentage_type_IIa_fibers	region1inRegion2	14874
CNVR118	1416	10	55474331	55489205	gain	4211	41334738	61209673	subjective_abnormal_odor	region1inRegion2	14874
CNVR118	1416	10	55474331	55489205	gain	5393	41334738	61209673	Hemoglobin	region1inRegion2	14874
CNVR118	1416	10	55474331	55489205	gain	5419	41334738	61209673	hematocrit	region1inRegion2	14874
CNVR118	1416	10	55474331	55489205	gain	594	41334738	61209673	Teat_number	region1inRegion2	14874
CNVR118	1416	10	55474331	55489205	gain	15111	43498290	57049354	Lactate_dehydrogenase_level	region1inRegion2	14874
CNVR118	1416	10	55474331	55489205	gain	21263	43498290	60475548	Body_weight_(46_days)	region1inRegion2	14874
CNVR118	1416	10	55474331	55489205	gain	15861	46980658	61209673	Linoleic_acid_content	region1inRegion2	14874
CNVR118	1416	10	55474331	55489205	gain	2928	46980658	64951351	Teat_number	region1inRegion2	14874
CNVR118	1416	10	55474331	55489205	gain	18651	47362497	57943835	indole,_laboratory	region1inRegion2	14874
CNVR118	1416	10	55474331	55489205	gain	11847	50924853	60448721	Hind_leg_conformation	region1inRegion2	14874
CNVR118	1416	10	55474331	55489205	gain	327	50924853	60448721	Average_daily_gain	region1inRegion2	14874
CNVR118	1416	10	55474331	55489205	gain	7225	51074353	59991772	hematocrit	region1inRegion2	14874
CNVR118	1416	10	55474331	55489205	gain	21	51515088	58062668	Backfat_at_first_rib	region1inRegion2	14874
CNVR118	1416	10	55474331	55489205	gain	2952	52252366	64951351	backfat_at_last_rib	region1inRegion2	14874
CNVR118	1416	10	55474331	55489205	gain	3821	52252366	64951351	Loin_muscle_area	region1inRegion2	14874
CNVR118	1416	10	55474331	55489205	gain	5258	53056702	61390526	Teat_number	region1inRegion2	14874
CNVR118	1416	10	55474331	55489205	gain	21328	54177398	69339693	Cortisol_level	region1inRegion2	14874
CNVR118	1416	10	55474331	55489205	gain	14317	54435921	58456714	Umbilical_hernia	region1inRegion2	14874
CNVR118	1416	10	55474331	55489205	gain	65998	55093121	55946196	Juiciness_score	region1inRegion2	14874
CNVR118	1416	10	55474331	55489205	gain	130354	56421525	54996430	Litter_size	region2inRegion1	-1425095
CNVR118	1416	10	55474331	55489205	gain	130350	57913615	54996430	Litter_size	region2inRegion1	-2917185
CNVR118	1416	10	55474331	55489205	gain	130402	59665015	54996430	Litter_size	region2inRegion1	-4668585
CNVR119	1427	10	71830224	71835029	gain	9648	61130562	72237308	Haptoglobin_concentration	region1inRegion2	4805
CNVR119	1427	10	71830224	71835029	gain	1107	61209673	72237308	Teat_number	region1inRegion2	4805
CNVR119	1427	10	71830224	71835029	gain	5417	61209673	72237308	hematocrit	region1inRegion2	4805
CNVR119	1427	10	71830224	71835029	gain	6369	61209673	72237308	Creatinine_level	region1inRegion2	4805
CNVR119	1427	10	71830224	71835029	gain	7507	61209673	72237308	Aspartate_amino_transferase_activity	region1inRegion2	4805
CNVR119	1427	10	71830224	71835029	gain	3822	64951351	72237308	Cholesterol_level_in_meat	region1inRegion2	4805
CNVR118	1416	10	55474331	55489205	gain	127175	65925943	54996430	Hemoglobin	region2inRegion1	-10929513
CNVR119	1427	10	71830224	71835029	gain	521	66332574	73332866	Age_at_puberty	region1inRegion2	4805
CNVR118	1416	10	55474331	55489205	gain	29562	69049504	25199143	Feed_conversion_ratio	region2inRegion1	-43850361
CNVR119	1427	10	71830224	71835029	gain	4886	69339693	72237308	Age_at_puberty	region1inRegion2	4805
CNVR119	1427	10	71830224	71835029	gain	4887	69339693	72237308	Age_at_puberty	region1inRegion2	4805
CNVR121	1435	11	8243425	8246938	loss	3823	1111096	18695760	Carcass_length	region1inRegion2	3513
CNVR122	1436	11	8308242	8320582	gain	3823	1111096	18695760	Carcass_length	region1inRegion2	12340
CNVR123	1437	11	8815180	8845277	loss	3823	1111096	18695760	Carcass_length	region1inRegion2	30097
CNVR121	1435	11	8243425	8246938	loss	8893	1111096	18695760	Ear_area	region1inRegion2	3513
CNVR122	1436	11	8308242	8320582	gain	8893	1111096	18695760	Ear_area	region1inRegion2	12340
CNVR123	1437	11	8815180	8845277	loss	8893	1111096	18695760	Ear_area	region1inRegion2	30097
CNVR121	1435	11	8243425	8246938	loss	8903	1111096	18695760	Ear_erectness	region1inRegion2	3513
CNVR122	1436	11	8308242	8320582	gain	8903	1111096	18695760	Ear_erectness	region1inRegion2	12340
CNVR123	1437	11	8815180	8845277	loss	8903	1111096	18695760	Ear_erectness	region1inRegion2	30097
CNVR121	1435	11	8243425	8246938	loss	5427	1111096	26135817	Mean_corpuscular_hemoglobin_content	region1inRegion2	3513
CNVR122	1436	11	8308242	8320582	gain	5427	1111096	26135817	Mean_corpuscular_hemoglobin_content	region1inRegion2	12340
CNVR123	1437	11	8815180	8845277	loss	5427	1111096	26135817	Mean_corpuscular_hemoglobin_content	region1inRegion2	30097
CNVR124	1443	11	20978853	20984493	loss	5427	1111096	26135817	Mean_corpuscular_hemoglobin_content	region1inRegion2	5640
CNVR121	1435	11	8243425	8246938	loss	5466	1111096	26135817	Lymphocyte_number	region1inRegion2	3513
CNVR122	1436	11	8308242	8320582	gain	5466	1111096	26135817	Lymphocyte_number	region1inRegion2	12340
CNVR123	1437	11	8815180	8845277	loss	5466	1111096	26135817	Lymphocyte_number	region1inRegion2	30097
CNVR124	1443	11	20978853	20984493	loss	5466	1111096	26135817	Lymphocyte_number	region1inRegion2	5640
CNVR121	1435	11	8243425	8246938	loss	5477	1111096	26135817	Segmented_neutrophil_number	region1inRegion2	3513
CNVR122	1436	11	8308242	8320582	gain	5477	1111096	26135817	Segmented_neutrophil_number	region1inRegion2	12340
CNVR123	1437	11	8815180	8845277	loss	5477	1111096	26135817	Segmented_neutrophil_number	region1inRegion2	30097
CNVR124	1443	11	20978853	20984493	loss	5477	1111096	26135817	Segmented_neutrophil_number	region1inRegion2	5640
CNVR121	1435	11	8243425	8246938	loss	5493	1111096	26135817	Monocyte_number	region1inRegion2	3513
CNVR122	1436	11	8308242	8320582	gain	5493	1111096	26135817	Monocyte_number	region1inRegion2	12340
CNVR123	1437	11	8815180	8845277	loss	5493	1111096	26135817	Monocyte_number	region1inRegion2	30097
CNVR124	1443	11	20978853	20984493	loss	5493	1111096	26135817	Monocyte_number	region1inRegion2	5640
CNVR121	1435	11	8243425	8246938	loss	5923	1111096	26135817	Time_spent_socializing	region1inRegion2	3513

CNVR122	1436	11	8308242	8320582	gain	5923	1111096	26135817	Time_spent_socializing	region1inRegion2	12340
CNVR123	1437	11	8815180	8845277	loss	5923	1111096	26135817	Time_spent_socializing	region1inRegion2	30097
CNVR124	1443	11	20978853	20984493	loss	5923	1111096	26135817	Time_spent_socializing	region1inRegion2	5640
CNVR121	1435	11	8243425	8246938	loss	7020	1111096	41897196	Percentage_type_IIb_fibers	region1inRegion2	3513
CNVR122	1436	11	8308242	8320582	gain	7020	1111096	41897196	Percentage_type_IIb_fibers	region1inRegion2	12340
CNVR123	1437	11	8815180	8845277	loss	7020	1111096	41897196	Percentage_type_IIb_fibers	region1inRegion2	30097
CNVR124	1443	11	20978853	20984493	loss	7020	1111096	41897196	Percentage_type_IIb_fibers	region1inRegion2	5640
CNVR125	1457	11	32658621	32711857	loss	7020	1111096	41897196	Percentage_type_IIb_fibers	region1inRegion2	53236
CNVR126	1468	11	38006863	38074356	gain	7020	1111096	41897196	Percentage_type_IIb_fibers	region1inRegion2	67493
CNVR121	1435	11	8243425	8246938	loss	7023	1111096	41897196	Percentage_type_IIb_fibers	region1inRegion2	3513
CNVR122	1436	11	8308242	8320582	gain	7023	1111096	41897196	Percentage_type_IIb_fibers	region1inRegion2	12340
CNVR123	1437	11	8815180	8845277	loss	7023	1111096	41897196	Percentage_type_IIb_fibers	region1inRegion2	30097
CNVR124	1443	11	20978853	20984493	loss	7023	1111096	41897196	Percentage_type_IIb_fibers	region1inRegion2	5640
CNVR125	1457	11	32658621	32711857	loss	7023	1111096	41897196	Percentage_type_IIb_fibers	region1inRegion2	53236
CNVR126	1468	11	38006863	38074356	gain	7023	1111096	41897196	Percentage_type_IIb_fibers	region1inRegion2	67493
CNVR121	1435	11	8243425	8246938	loss	7030	1111096	41897196	Percentage_type_IIa_fibers	region1inRegion2	3513
CNVR122	1436	11	8308242	8320582	gain	7030	1111096	41897196	Percentage_type_IIa_fibers	region1inRegion2	12340
CNVR123	1437	11	8815180	8845277	loss	7030	1111096	41897196	Percentage_type_IIa_fibers	region1inRegion2	30097
CNVR124	1443	11	20978853	20984493	loss	7030	1111096	41897196	Percentage_type_IIa_fibers	region1inRegion2	5640
CNVR125	1457	11	32658621	32711857	loss	7030	1111096	41897196	Percentage_type_IIa_fibers	region1inRegion2	53236
CNVR126	1468	11	38006863	38074356	gain	7030	1111096	41897196	Percentage_type_IIa_fibers	region1inRegion2	67493
CNVR121	1435	11	8243425	8246938	loss	7042	1111096	41897196	Percentage_type_IIb_fibers	region1inRegion2	3513
CNVR122	1436	11	8308242	8320582	gain	7042	1111096	41897196	Percentage_type_IIb_fibers	region1inRegion2	12340
CNVR123	1437	11	8815180	8845277	loss	7042	1111096	41897196	Percentage_type_IIb_fibers	region1inRegion2	30097
CNVR124	1443	11	20978853	20984493	loss	7042	1111096	41897196	Percentage_type_IIb_fibers	region1inRegion2	5640
CNVR125	1457	11	32658621	32711857	loss	7042	1111096	41897196	Percentage_type_IIb_fibers	region1inRegion2	53236
CNVR126	1468	11	38006863	38074356	gain	7042	1111096	41897196	Percentage_type_IIb_fibers	region1inRegion2	67493
CNVR121	1435	11	8243425	8246938	loss	423	1111096	52754336	Percentage_of_backfat_and_leaf_fat_in_carcass	region1inRegion2	3513
CNVR122	1436	11	8308242	8320582	gain	423	1111096	52754336	Percentage_of_backfat_and_leaf_fat_in_carcass	region1inRegion2	12340
CNVR123	1437	11	8815180	8845277	loss	423	1111096	52754336	Percentage_of_backfat_and_leaf_fat_in_carcass	region1inRegion2	30097
CNVR124	1443	11	20978853	20984493	loss	423	1111096	52754336	Percentage_of_backfat_and_leaf_fat_in_carcass	region1inRegion2	5640
CNVR125	1457	11	32658621	32711857	loss	423	1111096	52754336	Percentage_of_backfat_and_leaf_fat_in_carcass	region1inRegion2	53236
CNVR126	1468	11	38006863	38074356	gain	423	1111096	52754336	Percentage_of_backfat_and_leaf_fat_in_carcass	region1inRegion2	67493
CNVR121	1435	11	8243425	8246938	loss	422	1111096	57368011	Backfat_weight	region1inRegion2	3513
CNVR122	1436	11	8308242	8320582	gain	422	1111096	57368011	Backfat_weight	region1inRegion2	12340
CNVR123	1437	11	8815180	8845277	loss	422	1111096	57368011	Backfat_weight	region1inRegion2	30097
CNVR124	1443	11	20978853	20984493	loss	422	1111096	57368011	Backfat_weight	region1inRegion2	5640
CNVR125	1457	11	32658621	32711857	loss	422	1111096	57368011	Backfat_weight	region1inRegion2	53236
CNVR126	1468	11	38006863	38074356	gain	422	1111096	57368011	Backfat_weight	region1inRegion2	67493
CNVR121	1435	11	8243425	8246938	loss	5260	1111096	68683528	Teat_number	region1inRegion2	3513
CNVR122	1436	11	8308242	8320582	gain	5260	1111096	68683528	Teat_number	region1inRegion2	12340
CNVR123	1437	11	8815180	8845277	loss	5260	1111096	68683528	Teat_number	region1inRegion2	30097
CNVR124	1443	11	20978853	20984493	loss	5260	1111096	68683528	Teat_number	region1inRegion2	5640
CNVR125	1457	11	32658621	32711857	loss	5260	1111096	68683528	Teat_number	region1inRegion2	53236
CNVR126	1468	11	38006863	38074356	gain	5260	1111096	68683528	Teat_number	region1inRegion2	67493
CNVR127	1505	11	61453183	61459945	gain	5260	1111096	68683528	Teat_number	region1inRegion2	6762
CNVR121	1435	11	8243425	8246938	loss	1	2933070	23432657	Belly_weight	region1inRegion2	3513
CNVR122	1436	11	8308242	8320582	gain	1	2933070	23432657	Belly_weight	region1inRegion2	12340
CNVR123	1437	11	8815180	8845277	loss	1	2933070	23432657	Belly_weight	region1inRegion2	30097
CNVR124	1443	11	20978853	20984493	loss	1	2933070	23432657	Belly_weight	region1inRegion2	5640
CNVR121	1435	11	8243425	8246938	loss	6	2933070	23432657	Loin_muscle_area	region1inRegion2	3513
CNVR122	1436	11	8308242	8320582	gain	6	2933070	23432657	Loin_muscle_area	region1inRegion2	12340
CNVR123	1437	11	8815180	8845277	loss	6	2933070	23432657	Loin_muscle_area	region1inRegion2	30097
CNVR124	1443	11	20978853	20984493	loss	6	2933070	23432657	Loin_muscle_area	region1inRegion2	5640
CNVR121	1435	11	8243425	8246938	loss	55880	3356496	13418361	Cryptorchidism	region1inRegion2	3513
CNVR122	1436	11	8308242	8320582	gain	55880	3356496	13418361	Cryptorchidism	region1inRegion2	12340
CNVR123	1437	11	8815180	8845277	loss	55880	3356496	13418361	Cryptorchidism	region1inRegion2	30097
CNVR121	1435	11	8243425	8246938	loss	55881	3356496	13418361	Cryptorchidism	region1inRegion2	3513
CNVR122	1436	11	8308242	8320582	gain	55881	3356496	13418361	Cryptorchidism	region1inRegion2	12340
CNVR123	1437	11	8815180	8845277	loss	55881	3356496	13418361	Cryptorchidism	region1inRegion2	30097
CNVR121	1435	11	8243425	8246938	loss	5578	7301324	10192096	Estrone,_laboratory	region1inRegion2	3513
CNVR122	1436	11	8308242	8320582	gain	5578	7301324	10192096	Estrone,_laboratory	region1inRegion2	12340
CNVR123	1437	11	8815180	8845277	loss	5578	7301324	10192096	Estrone,_laboratory	region1inRegion2	30097
CNVR121	1435	11	8243425	8246938	loss	4002	7902465	18695760	Conductivity_24_hours_postmortem_(loin)	region1inRegion2	3513
CNVR122	1436	11	8308242	8320582	gain	4002	7902465	18695760	Conductivity_24_hours_postmortem_(loin)	region1inRegion2	12340
CNVR123	1437	11	8815180	8845277	loss	4002	7902465	18695760	Conductivity_24_hours_postmortem_(loin)	region1inRegion2	30097
CNVR121	1435	11	8243425	8246938	loss	3220	7902465	41897196	backfat_at_last_rib	region1inRegion2	3513
CNVR122	1436	11	8308242	8320582	gain	3220	7902465	41897196	backfat_at_last_rib	region1inRegion2	12340
CNVR123	1437	11	8815180	8845277	loss	3220	7902465	41897196	backfat_at_last_rib	region1inRegion2	30097
CNVR124	1443	11	20978853	20984493	loss	3220	7902465	41897196	backfat_at_last_rib	region1inRegion2	5640
CNVR125	1457	11	32658621	32711857	loss	3220	7902465	41897196	backfat_at_last_rib	region1inRegion2	53236
CNVR126	1468	11	38006863	38074356	gain	3220	7902465	41897196	backfat_at_last_rib	region1inRegion2	67493
CNVR121	1435	11	8243425	8246938	loss	3657	7902465	41897196	Backfat_at_tenth_rib	region1inRegion2	3513
CNVR122	1436	11	8308242	8320582	gain	3657	7902465	41897196	Backfat_at_tenth_rib	region1inRegion2	12340
CNVR123	1437	11	8815180	8845277	loss	3657	7902465	41897196	Backfat_at_tenth_rib	region1inRegion2	30097
CNVR124	1443	11	20978853	20984493	loss	3657	7902465	41897196	Backfat_at_tenth_rib	region1inRegion2	5640
CNVR125	1457	11	32658621	32711857	loss	3657	7902465	41897196	Backfat_at_tenth_rib	region1inRegion2	53236
CNVR126	1468	11	38006863	38074356	gain	3657	7902465	41897196	Backfat_at_tenth_rib	region1inRegion2	67493
CNVR121	1435	11	8243425	8246938	loss	425	7902465	57368011	Backfat_thickness_between_3rd_and_4th_rib	region1inRegion2	3513
CNVR122	1436	11	8308242	8320582	gain	425	7902465	57368011	Backfat_thickness_between_3rd_and_4th_rib	region1inRegion2	12340
CNVR123	1437	11	8815180	8845277	loss	425	7902465	57368011	Backfat_thickness_between_3rd_and_4th_rib	region1inRegion2	30097
CNVR124	1443	11	20978853	20984493	loss	425	7902465	57368011	Backfat_thickness_between_3rd_and_4th_rib	region1inRegion2	5640
CNVR125	1457	11	32658621	32711857	loss	425	7902465	57368011	Backfat_thickness_between_3rd_and_4th_rib	region1inRegion2	53236
CNVR126	1468	11	38006863	38074356	gain	425	7902465	57368011	Backfat_thickness_between_3rd_and_4th_rib	region1inRegion2	67493
CNVR122	1436	11	8308242	8320582	gain	4314	8285934	10024160	Marbling	region1inRegion2	12340
CNVR123	1437	11	8815180	8845277	loss	4314	8285934	10024160	Marbling	region1inRegion2	30097
CNVR122	1436	11	8308242	8320582	gain	4315	8285934	10024160	Marbling	region1inRegion2	12340
CNVR123	1437	11	8815180	8845277	loss	4315	8285934	10024160	Marbling	region1inRegion2	30097
CNVR122	1436	11	8308242	8320582	gain	68	8300382	8595879	Carcass_length	region1inRegion2	12340
CNVR124	1443	11	20978853	20984493	loss	12323	18360382	23520623	Tetanus_antibody_titer	region1inRegion2	5640
CNVR124	1443	11	20978853	20984493	loss	12342	18360382	41897196	Change_in_interferon-gamma_level	region1inRegion2	5640
CNVR125	1457	11	32658621	32711857	loss	12342	18360382	41897196	Change_in_interferon-gamma_level	region1inRegion2	53236
CNVR126	1468	11	38006863	38074356	gain	12342	18360382	41897196	Change_in_interferon-gamma_level	region1inRegion2	67493
CNVR124	1443	11	20978853	20984493	loss	17601	18360382	41897196	Interleukin_2_level	region1inRegion2	5640
CNVR125	1457	11	32658621	32711857	loss	17601	18360382	41897196	Interleukin_2_level	region1inRegion2	53236
CNVR126	1468	11	38006863	38074356	gain	17601	18360382	41897196	Interleukin_2_level	region1inRegion2	67493
CNVR124	1443	11	20978853	20984493	loss	17606	18360382	41897196	Interleukin_10_level	region1inRegion2	5640
CNVR125	1457	11	32658621	32711857	loss	17606	18360382	41897196	Interleukin_10_level	region1inRegion2	53236
CNVR126	1468	11	38006863	38074356	gain	17606	18360382	41897196	Interleukin_10_level	region1inRegion2	67493
CNVR124	1443	11	20978853	20984493	loss	17612	18360382	41897196	Interferon-gamma_level	region1inRegion2	5640
CNVR125	1457	11	32658621	32711857	loss	17612	18360382	41897196	Interferon-gamma_level	region1inRegion2	53236
CNVR126	1468	11	38006863	38074356	gain	17612	18360382	41897196	Interferon-gamma_level	region1inRegion2	67493
CNVR124	1443	11	20978853	20984493	loss	17615	18360382	41897196	Toll-like_receptor_2_level	region1inRegion2	5640
CNVR125	1457	11	32658621	32711857	loss	17615	18360382	41897196	Toll-like_receptor_2_level	region1inRegion2	53236
CNVR126	1468	11	38006863	38074356	gain	17615	18360382	41897196	Toll-like_receptor_2_level	region1inRegion2	67493
CNVR124	1443	11	20978853	20984493	loss	17621	18360382	41897196	Toll-like_receptor_9_level	region1inRegion2	5640
CNVR125	1457	11	32658621	32711857	loss	17621	18360382	41897196	Toll-like_receptor_9_level	region1inRegion2	53236
CNVR126	1468	11	38006863	38074356	gain	17621	18360382	41897196	Toll-like_receptor_9_level	region1inRegion2	67493
CNVR124	1443	11	20978853	20984493	loss	17622	18360382	41897196	Toll-like_receptor_9_level	region1inRegion2	5640
CNVR125	1457	11	32658621	32711857	loss	17622	18360382	41897196	Toll-like_receptor_9_level	region1inRegion2	53236
CNVR126	1468	11	38006863	38074356	gain	17622	18360382	41897196	Toll-like_receptor_9_level	region1inRegion2	67493
CNVR124	1443	11	20978853	20984493	loss	17722	18360382	41897196	Triglyceride_level	region1inRegion2	5640
CNVR125	1457	11	32658621	32711857	loss	17722	18360382	41897196	Triglyceride_level	region1inRegion2	53236
CNVR126	1468	11	38006863	38074356	gain	17722	18360382	41897196	Triglyceride_level	region1inRegion2	67493
CNVR124	1443	11	20978853	20984493	loss	2816	18360382	41897196	Percentage_type_IIa_fibers	region1inRegion2	5640
CNVR125	1457	11	32658621	32711857	loss	2816	18360382	41897196	Percentage_type_IIa_fibers	region1inRegion2	53236
CNVR126	1468	11	38006863	38074356	gain	2816	18360382	41897196	Percentage_type_IIa_fibers	region1inRegion2	67493
CNVR124	1443	11	20978853	20984493	loss	9597	18360382	41897196	Hemolytic_complement_activity_(alternative_pathway)	region1inRegion2	5640
CNVR125	1457	11	32658621	32711857	loss	9597	18360382	41897196	Hemolytic_complement_activity_(alternative_pathway)	region1inRegion2	53236
CNVR126	1468	11	38006863	38074356	gain	9597	18360382	41897196	Hemolytic_complement_activity_(alternative_pathway)	region1inRegion2	67493
CNVR124	1443	11	20978853	20984493	loss	9598	18360382	41897196	Hemolytic_complement_activity_(alternative_pathway)	region1inRegion2	5640
CNVR125	1457	11	32658621	32711857	loss	9598	18360382	41897196	Hemolytic_complement_activity_(alternative_pathway)	region1inRegion2	53236

CNVR126	1468	11	38006863	38074356	gain	9598	18360382	41897196	Hemolytic_complement_activity_(alternative_pathway)	region1inRegion2	67493
CNVR124	1443	11	20978853	20984493	loss	17807	18360382	41959686	Loin_muscle_depth	region1inRegion2	5640
CNVR125	1457	11	32658621	32711857	loss	17807	18360382	41959686	Loin_muscle_depth	region1inRegion2	53236
CNVR126	1468	11	38006863	38074356	gain	17807	18360382	41959686	Loin_muscle_depth	region1inRegion2	67493
CNVR124	1443	11	20978853	20984493	loss	17808	18360382	41959686	Loin_muscle_area	region1inRegion2	5640
CNVR125	1457	11	32658621	32711857	loss	17808	18360382	41959686	Loin_muscle_area	region1inRegion2	53236
CNVR126	1468	11	38006863	38074356	gain	17808	18360382	41959686	Loin_muscle_area	region1inRegion2	67493
CNVR124	1443	11	20978853	20984493	loss	17809	18360382	41959686	Loin_muscle_depth	region1inRegion2	5640
CNVR125	1457	11	32658621	32711857	loss	17809	18360382	41959686	Loin_muscle_depth	region1inRegion2	53236
CNVR126	1468	11	38006863	38074356	gain	17809	18360382	41959686	Loin_muscle_depth	region1inRegion2	67493
CNVR124	1443	11	20978853	20984493	loss	3069	18695760	41897196	Loin_weight	region1inRegion2	5640
CNVR125	1457	11	32658621	32711857	loss	3069	18695760	41897196	Loin_weight	region1inRegion2	53236
CNVR126	1468	11	38006863	38074356	gain	3069	18695760	41897196	Loin_weight	region1inRegion2	67493
CNVR124	1443	11	20978853	20984493	loss	3070	18695760	41897196	Loin_muscle_depth	region1inRegion2	5640
CNVR125	1457	11	32658621	32711857	loss	3070	18695760	41897196	Loin_muscle_depth	region1inRegion2	53236
CNVR126	1468	11	38006863	38074356	gain	3070	18695760	41897196	Loin_muscle_depth	region1inRegion2	67493
CNVR124	1443	11	20978853	20984493	loss	3071	18695760	41897196	Loin_meat_weight	region1inRegion2	5640
CNVR125	1457	11	32658621	32711857	loss	3071	18695760	41897196	Loin_meat_weight	region1inRegion2	53236
CNVR126	1468	11	38006863	38074356	gain	3071	18695760	41897196	Loin_meat_weight	region1inRegion2	67493
CNVR124	1443	11	20978853	20984493	loss	1116	18695760	54245356	Loin_and_neck_meat_weight	region1inRegion2	5640
CNVR125	1457	11	32658621	32711857	loss	1116	18695760	54245356	Loin_and_neck_meat_weight	region1inRegion2	53236
CNVR126	1468	11	38006863	38074356	gain	1116	18695760	54245356	Loin_and_neck_meat_weight	region1inRegion2	67493
CNVR124	1443	11	20978853	20984493	loss	15060	18695760	75366022	Mean_corpuscular_hemoglobin_concentration	region1inRegion2	5640
CNVR125	1457	11	32658621	32711857	loss	15060	18695760	75366022	Mean_corpuscular_hemoglobin_concentration	region1inRegion2	53236
CNVR126	1468	11	38006863	38074356	gain	15060	18695760	75366022	Mean_corpuscular_hemoglobin_concentration	region1inRegion2	67493
CNVR127	1505	11	61453183	61459945	gain	15060	18695760	75366022	Mean_corpuscular_hemoglobin_concentration	region1inRegion2	6762
CNVR128	1514	11	69030920	69069509	loss	15060	18695760	75366022	Mean_corpuscular_hemoglobin_concentration	region1inRegion2	38589
CNVR129	1518	11	70689891	70709813	loss	15060	18695760	75366022	Mean_corpuscular_hemoglobin_concentration	region1inRegion2	19922
CNVR130	1520	11	71317528	71375741	loss	15060	18695760	75366022	Mean_corpuscular_hemoglobin_concentration	region1inRegion2	58213
CNVR131	1524	11	71878763	71887995	gain	15060	18695760	75366022	Mean_corpuscular_hemoglobin_concentration	region1inRegion2	9232
CNVR124	1443	11	20978853	20984493	loss	5214	18695760	79777182	backfat_at_mid-back	region1inRegion2	5640
CNVR125	1457	11	32658621	32711857	loss	5214	18695760	79777182	backfat_at_mid-back	region1inRegion2	53236
CNVR126	1468	11	38006863	38074356	gain	5214	18695760	79777182	backfat_at_mid-back	region1inRegion2	67493
CNVR127	1505	11	61453183	61459945	gain	5214	18695760	79777182	backfat_at_mid-back	region1inRegion2	6762
CNVR128	1514	11	69030920	69069509	loss	5214	18695760	79777182	backfat_at_mid-back	region1inRegion2	38589
CNVR129	1518	11	70689891	70709813	loss	5214	18695760	79777182	backfat_at_mid-back	region1inRegion2	19922
CNVR130	1520	11	71317528	71375741	loss	5214	18695760	79777182	backfat_at_mid-back	region1inRegion2	58213
CNVR131	1524	11	71878763	71887995	gain	5214	18695760	79777182	backfat_at_mid-back	region1inRegion2	9232
CNVR124	1443	11	20978853	20984493	loss	38104	18695760	84588432	Loin_muscle_area	region1inRegion2	5640
CNVR125	1457	11	32658621	32711857	loss	38104	18695760	84588432	Loin_muscle_area	region1inRegion2	53236
CNVR126	1468	11	38006863	38074356	gain	38104	18695760	84588432	Loin_muscle_area	region1inRegion2	67493
CNVR127	1505	11	61453183	61459945	gain	38104	18695760	84588432	Loin_muscle_area	region1inRegion2	6762
CNVR128	1514	11	69030920	69069509	loss	38104	18695760	84588432	Loin_muscle_area	region1inRegion2	38589
CNVR129	1518	11	70689891	70709813	loss	38104	18695760	84588432	Loin_muscle_area	region1inRegion2	19922
CNVR130	1520	11	71317528	71375741	loss	38104	18695760	84588432	Loin_muscle_area	region1inRegion2	58213
CNVR131	1524	11	71878763	71887995	gain	38104	18695760	84588432	Loin_muscle_area	region1inRegion2	9232
CNVR124	1443	11	20978853	20984493	loss	38105	18695760	84588432	CIE-b*	region1inRegion2	5640
CNVR125	1457	11	32658621	32711857	loss	38105	18695760	84588432	CIE-b*	region1inRegion2	53236
CNVR126	1468	11	38006863	38074356	gain	38105	18695760	84588432	CIE-b*	region1inRegion2	67493
CNVR127	1505	11	61453183	61459945	gain	38105	18695760	84588432	CIE-b*	region1inRegion2	6762
CNVR128	1514	11	69030920	69069509	loss	38105	18695760	84588432	CIE-b*	region1inRegion2	38589
CNVR129	1518	11	70689891	70709813	loss	38105	18695760	84588432	CIE-b*	region1inRegion2	19922
CNVR130	1520	11	71317528	71375741	loss	38105	18695760	84588432	CIE-b*	region1inRegion2	58213
CNVR131	1524	11	71878763	71887995	gain	38105	18695760	84588432	CIE-b*	region1inRegion2	9232
CNVR125	1457	11	32658621	32711857	loss	8894	26135817	41897196	Ear_area	region1inRegion2	53236
CNVR126	1468	11	38006863	38074356	gain	8894	26135817	41897196	Ear_area	region1inRegion2	67493
CNVR125	1457	11	32658621	32711857	loss	5914	26135817	79181648	Time_spent_drinking	region1inRegion2	53236
CNVR126	1468	11	38006863	38074356	gain	5914	26135817	79181648	Time_spent_drinking	region1inRegion2	67493
CNVR127	1505	11	61453183	61459945	gain	5914	26135817	79181648	Time_spent_drinking	region1inRegion2	6762
CNVR128	1514	11	69030920	69069509	loss	5914	26135817	79181648	Time_spent_drinking	region1inRegion2	38589
CNVR129	1518	11	70689891	70709813	loss	5914	26135817	79181648	Time_spent_drinking	region1inRegion2	19922
CNVR130	1520	11	71317528	71375741	loss	5914	26135817	79181648	Time_spent_drinking	region1inRegion2	58213
CNVR131	1524	11	71878763	71887995	gain	5914	26135817	79181648	Time_spent_drinking	region1inRegion2	9232
CNVR125	1457	11	32658621	32711857	loss	7508	26135817	79181648	Body_temperature	region1inRegion2	53236
CNVR126	1468	11	38006863	38074356	gain	7508	26135817	79181648	Body_temperature	region1inRegion2	67493
CNVR127	1505	11	61453183	61459945	gain	7508	26135817	79181648	Body_temperature	region1inRegion2	6762
CNVR128	1514	11	69030920	69069509	loss	7508	26135817	79181648	Body_temperature	region1inRegion2	38589
CNVR129	1518	11	70689891	70709813	loss	7508	26135817	79181648	Body_temperature	region1inRegion2	19922
CNVR130	1520	11	71317528	71375741	loss	7508	26135817	79181648	Body_temperature	region1inRegion2	58213
CNVR131	1524	11	71878763	71887995	gain	7508	26135817	79181648	Body_temperature	region1inRegion2	9232
CNVR125	1457	11	32658621	32711857	loss	7509	26135817	79181648	Eosinophil_number	region1inRegion2	53236
CNVR126	1468	11	38006863	38074356	gain	7509	26135817	79181648	Eosinophil_number	region1inRegion2	67493
CNVR127	1505	11	61453183	61459945	gain	7509	26135817	79181648	Eosinophil_number	region1inRegion2	6762
CNVR128	1514	11	69030920	69069509	loss	7509	26135817	79181648	Eosinophil_number	region1inRegion2	38589
CNVR129	1518	11	70689891	70709813	loss	7509	26135817	79181648	Eosinophil_number	region1inRegion2	19922
CNVR130	1520	11	71317528	71375741	loss	7509	26135817	79181648	Eosinophil_number	region1inRegion2	58213
CNVR131	1524	11	71878763	71887995	gain	7509	26135817	79181648	Eosinophil_number	region1inRegion2	9232
CNVR126	1468	11	38006863	38074356	gain	586	35252011	62047663	Teat_number	region1inRegion2	67493
CNVR127	1505	11	61453183	61459945	gain	586	35252011	62047663	Teat_number	region1inRegion2	6762
CNVR127	1505	11	61453183	61459945	gain	7027	41897196	75366022	Percentage_type_I_fibers	region1inRegion2	6762
CNVR128	1514	11	69030920	69069509	loss	7027	41897196	75366022	Percentage_type_I_fibers	region1inRegion2	38589
CNVR129	1518	11	70689891	70709813	loss	7027	41897196	75366022	Percentage_type_I_fibers	region1inRegion2	19922
CNVR130	1520	11	71317528	71375741	loss	7027	41897196	75366022	Percentage_type_I_fibers	region1inRegion2	58213
CNVR131	1524	11	71878763	71887995	gain	7027	41897196	75366022	Percentage_type_I_fibers	region1inRegion2	9232
CNVR127	1505	11	61453183	61459945	gain	7464	41897196	75366022	Nonfunctional_nipples	region1inRegion2	6762
CNVR128	1514	11	69030920	69069509	loss	7464	41897196	75366022	Nonfunctional_nipples	region1inRegion2	38589
CNVR129	1518	11	70689891	70709813	loss	7464	41897196	75366022	Nonfunctional_nipples	region1inRegion2	19922
CNVR130	1520	11	71317528	71375741	loss	7464	41897196	75366022	Nonfunctional_nipples	region1inRegion2	58213
CNVR131	1524	11	71878763	71887995	gain	7464	41897196	75366022	Nonfunctional_nipples	region1inRegion2	9232
CNVR127	1505	11	61453183	61459945	gain	2954	41897196	79769559	Meat_color_score	region1inRegion2	6762
CNVR128	1514	11	69030920	69069509	loss	2954	41897196	79769559	Meat_color_score	region1inRegion2	38589
CNVR129	1518	11	70689891	70709813	loss	2954	41897196	79769559	Meat_color_score	region1inRegion2	19922
CNVR130	1520	11	71317528	71375741	loss	2954	41897196	79769559	Meat_color_score	region1inRegion2	58213
CNVR131	1524	11	71878763	71887995	gain	2954	41897196	79769559	Meat_color_score	region1inRegion2	9232
CNVR127	1505	11	61453183	61459945	gain	2955	41897196	79769559	Drip_loss	region1inRegion2	6762
CNVR128	1514	11	69030920	69069509	loss	2955	41897196	79769559	Drip_loss	region1inRegion2	38589
CNVR129	1518	11	70689891	70709813	loss	2955	41897196	79769559	Drip_loss	region1inRegion2	19922
CNVR130	1520	11	71317528	71375741	loss	2955	41897196	79769559	Drip_loss	region1inRegion2	58213
CNVR131	1524	11	71878763	71887995	gain	2955	41897196	79769559	Drip_loss	region1inRegion2	9232
CNVR127	1505	11	61453183	61459945	gain	7534	52388584	79181648	Number_of_stillborn	region1inRegion2	6762
CNVR128	1514	11	69030920	69069509	loss	7534	52388584	79181648	Number_of_stillborn	region1inRegion2	38589
CNVR129	1518	11	70689891	70709813	loss	7534	52388584	79181648	Number_of_stillborn	region1inRegion2	19922
CNVR130	1520	11	71317528	71375741	loss	7534	52388584	79181648	Number_of_stillborn	region1inRegion2	58213
CNVR131	1524	11	71878763	71887995	gain	7534	52388584	79181648	Number_of_stillborn	region1inRegion2	9232
CNVR127	1505	11	61453183	61459945	gain	16934	52388584	81069519	backfat_at_last_rib	region1inRegion2	6762
CNVR128	1514	11	69030920	69069509	loss	16934	52388584	81069519	backfat_at_last_rib	region1inRegion2	38589
CNVR129	1518	11	70689891	70709813	loss	16934	52388584	81069519	backfat_at_last_rib	region1inRegion2	19922
CNVR130	1520	11	71317528	71375741	loss	16934	52388584	81069519	backfat_at_last_rib	region1inRegion2	58213
CNVR131	1524	11	71878763	71887995	gain	16934	52388584	81069519	backfat_at_last_rib	region1inRegion2	9232
CNVR127	1505	11	61453183	61459945	gain	16935	52388584	81069519	Backfat_at_tenth_rib	region1inRegion2	6762
CNVR128	1514	11	69030920	69069509	loss	16935	52388584	81069519	Backfat_at_tenth_rib	region1inRegion2	38589
CNVR129	1518	11	70689891	70709813	loss	16935	52388584	81069519	Backfat_at_tenth_rib	region1inRegion2	19922
CNVR130	1520	11	71317528	71375741	loss	16935	52388584	81069519	Backfat_at_tenth_rib	region1inRegion2	58213
CNVR131	1524	11	71878763	71887995	gain	16935	52388584	81069519	Backfat_at_tenth_rib	region1inRegion2	9232
CNVR127	1505	11	61453183	61459945	gain	16936	52388584	81069519	Empty_body_lipid_content	region1inRegion2	6762
CNVR128	1514	11	69030920	69069509	loss	16936	52388584	81069519	Empty_body_lipid_content	region1inRegion2	38589
CNVR129	1518	11	70689891	70709813	loss	16936	52388584	81069519	Empty_body_lipid_content	region1inRegion2	19922
CNVR130	1520	11	71317528	71375741	loss	16936	52388584	81069519	Empty_body_lipid_content	region1inRegion2	58213
CNVR131	1524	11	71878763	71887995	gain	16936	52388584	81069519	Empty_body_lipid_content	region1inRegion2	9232
CNVR127	1505	11	61453183	61459945	gain	3211	52388584	81069519	Backfat_at_tenth_rib	region1inRegion2	6762
CNVR128	1514	11	69030920	69069509	loss	3211	52388584	81069519	Backfat_at_tenth_rib	region1inRegion2	38589
CNVR129	1518	11	70689891	70709813	loss	3211	52388584	81069519	Backfat_at_tenth_rib	region1inRegion2	19922

CNVR130	1520	11	71317528	71375741	loss	3211	52388584	81069519	Backfat_at_tenth_rib	region1inRegion2	58213
CNVR131	1524	11	71878763	71887995	gain	3211	52388584	81069519	Backfat_at_tenth_rib	region1inRegion2	9232
CNVR127	1505	11	61453183	61459945	gain	3218	52388584	81069519	Backfat_at_tenth_rib	region1inRegion2	6762
CNVR128	1514	11	69030920	69069509	loss	3218	52388584	81069519	Backfat_at_tenth_rib	region1inRegion2	38589
CNVR129	1518	11	70689891	70709813	loss	3218	52388584	81069519	Backfat_at_tenth_rib	region1inRegion2	19922
CNVR130	1520	11	71317528	71375741	loss	3218	52388584	81069519	Backfat_at_tenth_rib	region1inRegion2	58213
CNVR131	1524	11	71878763	71887995	gain	3218	52388584	81069519	Backfat_at_tenth_rib	region1inRegion2	9232
CNVR127	1505	11	61453183	61459945	gain	3281	52388584	81069519	45_min-24_h_pH_decline	region1inRegion2	6762
CNVR128	1514	11	69030920	69069509	loss	3281	52388584	81069519	45_min-24_h_pH_decline	region1inRegion2	38589
CNVR129	1518	11	70689891	70709813	loss	3281	52388584	81069519	45_min-24_h_pH_decline	region1inRegion2	19922
CNVR130	1520	11	71317528	71375741	loss	3281	52388584	81069519	45_min-24_h_pH_decline	region1inRegion2	58213
CNVR131	1524	11	71878763	71887995	gain	3281	52388584	81069519	45_min-24_h_pH_decline	region1inRegion2	9232
CNVR127	1505	11	61453183	61459945	gain	3282	52388584	81069519	Muscle_fat_content	region1inRegion2	6762
CNVR128	1514	11	69030920	69069509	loss	3282	52388584	81069519	Muscle_fat_content	region1inRegion2	38589
CNVR129	1518	11	70689891	70709813	loss	3282	52388584	81069519	Muscle_fat_content	region1inRegion2	19922
CNVR130	1520	11	71317528	71375741	loss	3282	52388584	81069519	Muscle_fat_content	region1inRegion2	58213
CNVR131	1524	11	71878763	71887995	gain	3282	52388584	81069519	Muscle_fat_content	region1inRegion2	9232
CNVR127	1505	11	61453183	61459945	gain	3283	52388584	81069519	Muscle_moisture_percentage	region1inRegion2	6762
CNVR128	1514	11	69030920	69069509	loss	3283	52388584	81069519	Muscle_moisture_percentage	region1inRegion2	38589
CNVR129	1518	11	70689891	70709813	loss	3283	52388584	81069519	Muscle_moisture_percentage	region1inRegion2	19922
CNVR130	1520	11	71317528	71375741	loss	3283	52388584	81069519	Muscle_moisture_percentage	region1inRegion2	58213
CNVR131	1524	11	71878763	71887995	gain	3283	52388584	81069519	Muscle_moisture_percentage	region1inRegion2	9232
CNVR127	1505	11	61453183	61459945	gain	3319	52388584	81069519	Empty_body_lipid_content	region1inRegion2	6762
CNVR128	1514	11	69030920	69069509	loss	3319	52388584	81069519	Empty_body_lipid_content	region1inRegion2	38589
CNVR129	1518	11	70689891	70709813	loss	3319	52388584	81069519	Empty_body_lipid_content	region1inRegion2	19922
CNVR130	1520	11	71317528	71375741	loss	3319	52388584	81069519	Empty_body_lipid_content	region1inRegion2	58213
CNVR131	1524	11	71878763	71887995	gain	3319	52388584	81069519	Empty_body_lipid_content	region1inRegion2	9232
CNVR127	1505	11	61453183	61459945	gain	1115	54245356	75366022	Body_weight_(slaughter)	region1inRegion2	6762
CNVR128	1514	11	69030920	69069509	loss	1115	54245356	75366022	Body_weight_(slaughter)	region1inRegion2	38589
CNVR129	1518	11	70689891	70709813	loss	1115	54245356	75366022	Body_weight_(slaughter)	region1inRegion2	19922
CNVR130	1520	11	71317528	71375741	loss	1115	54245356	75366022	Body_weight_(slaughter)	region1inRegion2	58213
CNVR131	1524	11	71878763	71887995	gain	1115	54245356	75366022	Body_weight_(slaughter)	region1inRegion2	9232
CNVR127	1505	11	61453183	61459945	gain	1117	54245356	75366022	Average_backfat_thickness	region1inRegion2	6762
CNVR128	1514	11	69030920	69069509	loss	1117	54245356	75366022	Average_backfat_thickness	region1inRegion2	38589
CNVR129	1518	11	70689891	70709813	loss	1117	54245356	75366022	Average_backfat_thickness	region1inRegion2	19922
CNVR130	1520	11	71317528	71375741	loss	1117	54245356	75366022	Average_backfat_thickness	region1inRegion2	58213
CNVR131	1524	11	71878763	71887995	gain	1117	54245356	75366022	Average_backfat_thickness	region1inRegion2	9232
CNVR127	1505	11	61453183	61459945	gain	1118	54245356	75366022	Feed_intake	region1inRegion2	6762
CNVR128	1514	11	69030920	69069509	loss	1118	54245356	75366022	Feed_intake	region1inRegion2	38589
CNVR129	1518	11	70689891	70709813	loss	1118	54245356	75366022	Feed_intake	region1inRegion2	19922
CNVR130	1520	11	71317528	71375741	loss	1118	54245356	75366022	Feed_intake	region1inRegion2	58213
CNVR131	1524	11	71878763	71887995	gain	1118	54245356	75366022	Feed_intake	region1inRegion2	9232
CNVR127	1505	11	61453183	61459945	gain	16838	54245356	75366022	backfat_at_last_rib	region1inRegion2	6762
CNVR128	1514	11	69030920	69069509	loss	16838	54245356	75366022	backfat_at_last_rib	region1inRegion2	38589
CNVR129	1518	11	70689891	70709813	loss	16838	54245356	75366022	backfat_at_last_rib	region1inRegion2	19922
CNVR130	1520	11	71317528	71375741	loss	16838	54245356	75366022	backfat_at_last_rib	region1inRegion2	58213
CNVR131	1524	11	71878763	71887995	gain	16838	54245356	75366022	backfat_at_last_rib	region1inRegion2	9232
CNVR127	1505	11	61453183	61459945	gain	3963	60053411	73438559	Ham_fat_thickness	region1inRegion2	6762
CNVR128	1514	11	69030920	69069509	loss	3963	60053411	73438559	Ham_fat_thickness	region1inRegion2	38589
CNVR129	1518	11	70689891	70709813	loss	3963	60053411	73438559	Ham_fat_thickness	region1inRegion2	19922
CNVR130	1520	11	71317528	71375741	loss	3963	60053411	73438559	Ham_fat_thickness	region1inRegion2	58213
CNVR131	1524	11	71878763	71887995	gain	3963	60053411	73438559	Ham_fat_thickness	region1inRegion2	9232
CNVR127	1505	11	61453183	61459945	gain	17623	60053411	75366022	Toll-like_receptor_9_level	region1inRegion2	6762
CNVR128	1514	11	69030920	69069509	loss	17623	60053411	75366022	Toll-like_receptor_9_level	region1inRegion2	38589
CNVR129	1518	11	70689891	70709813	loss	17623	60053411	75366022	Toll-like_receptor_9_level	region1inRegion2	19922
CNVR130	1520	11	71317528	71375741	loss	17623	60053411	75366022	Toll-like_receptor_9_level	region1inRegion2	58213
CNVR131	1524	11	71878763	71887995	gain	17623	60053411	75366022	Toll-like_receptor_9_level	region1inRegion2	9232
CNVR127	1505	11	61453183	61459945	gain	7478	60053411	87690581	Nonfunctional_nipples	region1inRegion2	6762
CNVR128	1514	11	69030920	69069509	loss	7478	60053411	87690581	Nonfunctional_nipples	region1inRegion2	38589
CNVR129	1518	11	70689891	70709813	loss	7478	60053411	87690581	Nonfunctional_nipples	region1inRegion2	19922
CNVR130	1520	11	71317528	71375741	loss	7478	60053411	87690581	Nonfunctional_nipples	region1inRegion2	58213
CNVR131	1524	11	71878763	71887995	gain	7478	60053411	87690581	Nonfunctional_nipples	region1inRegion2	9232
CNVR128	1514	11	69030920	69069509	loss	3964	62760744	81069519	Lean_meat_percentage	region1inRegion2	38589
CNVR129	1518	11	70689891	70709813	loss	3964	62760744	81069519	Lean_meat_percentage	region1inRegion2	19922
CNVR130	1520	11	71317528	71375741	loss	3964	62760744	81069519	Lean_meat_percentage	region1inRegion2	58213
CNVR131	1524	11	71878763	71887995	gain	3964	62760744	81069519	Lean_meat_percentage	region1inRegion2	9232
CNVR128	1514	11	69030920	69069509	loss	4307	66840642	75703898	Average_daily_gain	region1inRegion2	38589
CNVR129	1518	11	70689891	70709813	loss	4307	66840642	75703898	Average_daily_gain	region1inRegion2	19922
CNVR130	1520	11	71317528	71375741	loss	4307	66840642	75703898	Average_daily_gain	region1inRegion2	58213
CNVR131	1524	11	71878763	71887995	gain	4307	66840642	75703898	Average_daily_gain	region1inRegion2	9232
CNVR128	1514	11	69030920	69069509	loss	7433	66840642	75985621	Double-bond_index	region1inRegion2	38589
CNVR129	1518	11	70689891	70709813	loss	7433	66840642	75985621	Double-bond_index	region1inRegion2	19922
CNVR130	1520	11	71317528	71375741	loss	7433	66840642	75985621	Double-bond_index	region1inRegion2	58213
CNVR131	1524	11	71878763	71887995	gain	7433	66840642	75985621	Double-bond_index	region1inRegion2	9232
CNVR128	1514	11	69030920	69069509	loss	64762	68781957	69737033	Thoracolumbar_vertebra_number	region1inRegion2	38589
CNVR128	1514	11	69030920	69069509	loss	10248	68877193	69699298	Cross-sectional_area_of_type_IIa_muscle_fibers	region1inRegion2	38589
CNVR129	1518	11	70689891	70709813	loss	66261	70702588	71552118	Daily_feed_intake	overlapTail	7226
CNVR130	1520	11	71317528	71375741	loss	66261	70702588	71552118	Daily_feed_intake	region1inRegion2	58213
CNVR129	1518	11	70689891	70709813	loss	126688	70702588	71698847	Teat_number	overlapTail	7226
CNVR130	1520	11	71317528	71375741	loss	126688	70702588	71698847	Teat_number	region1inRegion2	58213
CNVR131	1524	11	71878763	71887995	gain	18664	71386065	72664075	androstenone,_laboratory	region1inRegion2	9232
CNVR131	1524	11	71878763	71887995	gain	126617	71763167	72529341	Teat_number	region1inRegion2	9232
CNVR131	1524	11	71878763	71887995	gain	126683	71763167	72529341	Left_teat_number	region1inRegion2	9232
CNVR132	1555	12	50924636	50947287	gain	21312	114899	53297375	Thoracic_vertebra_number	region1inRegion2	22651
CNVR133	1556	12	51099806	51123003	gain	21312	114899	53297375	Thoracic_vertebra_number	region1inRegion2	23197
CNVR132	1555	12	50924636	50947287	gain	37567	114899	53743337	Actinobacillus_pleuropneumoniae_susceptibility	region1inRegion2	22651
CNVR133	1556	12	51099806	51123003	gain	37567	114899	53743337	Actinobacillus_pleuropneumoniae_susceptibility	region1inRegion2	23197
CNVR132	1555	12	50924636	50947287	gain	21311	114899	54374277	Vertebra_number	region1inRegion2	22651
CNVR133	1556	12	51099806	51123003	gain	21311	114899	54374277	Vertebra_number	region1inRegion2	23197
CNVR132	1555	12	50924636	50947287	gain	37576	114899	59478853	Actinobacillus_pleuropneumoniae_susceptibility	region1inRegion2	22651
CNVR133	1556	12	51099806	51123003	gain	37576	114899	59478853	Actinobacillus_pleuropneumoniae_susceptibility	region1inRegion2	23197
CNVR132	1555	12	50924636	50947287	gain	5227	4504025	63588571	Teat_number	region1inRegion2	22651
CNVR133	1556	12	51099806	51123003	gain	5227	4504025	63588571	Teat_number	region1inRegion2	23197
CNVR132	1555	12	50924636	50947287	gain	38106	4823589	54022075	Muscle_moisture_percentage	region1inRegion2	22651
CNVR133	1556	12	51099806	51123003	gain	38106	4823589	54022075	Muscle_moisture_percentage	region1inRegion2	23197
CNVR132	1555	12	50924636	50947287	gain	15076	4823589	62961334	Triglyceride_level	region1inRegion2	22651
CNVR133	1556	12	51099806	51123003	gain	15076	4823589	62961334	Triglyceride_level	region1inRegion2	23197
CNVR132	1555	12	50924636	50947287	gain	37565	7515272	59478853	Actinobacillus_pleuropneumoniae_susceptibility	region1inRegion2	22651
CNVR133	1556	12	51099806	51123003	gain	37565	7515272	59478853	Actinobacillus_pleuropneumoniae_susceptibility	region1inRegion2	23197
CNVR132	1555	12	50924636	50947287	gain	29707	9974951	62961334	body_weight_(10_weeks)	region1inRegion2	22651
CNVR133	1556	12	51099806	51123003	gain	29707	9974951	62961334	body_weight_(10_weeks)	region1inRegion2	23197
CNVR132	1555	12	50924636	50947287	gain	21264	10277143	53978267	Leaf_fat_weight	region1inRegion2	22651
CNVR133	1556	12	51099806	51123003	gain	21264	10277143	53978267	Leaf_fat_weight	region1inRegion2	23197
CNVR132	1555	12	50924636	50947287	gain	5261	10361588	63588571	Teat_number	region1inRegion2	22651
CNVR133	1556	12	51099806	51123003	gain	5261	10361588	63588571	Teat_number	region1inRegion2	23197
CNVR132	1555	12	50924636	50947287	gain	15115	15759023	53558899	Phosphate_level	region1inRegion2	22651
CNVR133	1556	12	51099806	51123003	gain	15115	15759023	53558899	Phosphate_level	region1inRegion2	23197
CNVR132	1555	12	50924636	50947287	gain	3966	19775991	60040247	Ham_fat_thickness	region1inRegion2	22651
CNVR133	1556	12	51099806	51123003	gain	3966	19775991	60040247	Ham_fat_thickness	region1inRegion2	23197
CNVR132	1555	12	50924636	50947287	gain	12115	23672762	60040247	Conductivity_24_hours_postmortem_(loin)	region1inRegion2	22651
CNVR133	1556	12	51099806	51123003	gain	12115	23672762	60040247	Conductivity_24_hours_postmortem_(loin)	region1inRegion2	23197
CNVR132	1555	12	50924636	50947287	gain	12116	23672762	60040247	Loin_muscle_depth	region1inRegion2	22651
CNVR133	1556	12	51099806	51123003	gain	12116	23672762	60040247	Loin_muscle_depth	region1inRegion2	23197
CNVR132	1555	12	50924636	50947287	gain	12117	23672762	60040247	Loin_weight	region1inRegion2	22651
CNVR133	1556	12	51099806	51123003	gain	12117	23672762	60040247	Loin_weight	region1inRegion2	23197
CNVR132	1555	12	50924636	50947287	gain	17608	23672762	60040247	Interleukin_10_level	region1inRegion2	22651
CNVR133	1556	12	51099806	51123003	gain	17608	23672762	60040247	Interleukin_10_level	region1inRegion2	23197
CNVR132	1555	12	50924636	50947287	gain	17616	23672762	60040247	Toll-like_receptor_2_level	region1inRegion2	22651
CNVR133	1556	12	51099806	51123003	gain	17616	23672762	60040247	Toll-like_receptor_2_level	region1inRegion2	23197
CNVR132	1555	12	50924636	50947287	gain	17625	23672762	60040247	Toll-like_receptor_9_level	region1inRegion2	22651
CNVR133	1556	12	51099806	51123003	gain	17625	23672762	60040247	Toll-like_receptor_9_level	region1inRegion2	23197
CNVR132	1555	12	50924636	50947287	gain	2818	23672762	60040247	Number_of_muscle_fibers_per_unit_area	region1inRegion2	22651

CNVR133	1556	12	51099806	51123003	gain	2818	23672762	60040247	Number_of_muscle_fibers_per_unit_area	region1inRegion2	23197
CNVR132	1555	12	50924636	50947287	gain	2819	23672762	60040247	Diameter_of_type_I_muscle_fibers	region1inRegion2	22651
CNVR133	1556	12	51099806	51123003	gain	2819	23672762	60040247	Diameter_of_type_I_muscle_fibers	region1inRegion2	23197
CNVR132	1555	12	50924636	50947287	gain	2820	23672762	60040247	Total_muscle_fiber_number	region1inRegion2	22651
CNVR133	1556	12	51099806	51123003	gain	2820	23672762	60040247	Total_muscle_fiber_number	region1inRegion2	23197
CNVR132	1555	12	50924636	50947287	gain	2821	23672762	60040247	Diameter_of_type_I_muscle_fibers	region1inRegion2	22651
CNVR133	1556	12	51099806	51123003	gain	2821	23672762	60040247	Diameter_of_type_I_muscle_fibers	region1inRegion2	23197
CNVR132	1555	12	50924636	50947287	gain	3085	23672762	60040247	Loin_muscle_area	region1inRegion2	22651
CNVR133	1556	12	51099806	51123003	gain	3085	23672762	60040247	Loin_muscle_area	region1inRegion2	23197
CNVR132	1555	12	50924636	50947287	gain	5717	23672762	60040247	Body_weight_(birth)	region1inRegion2	22651
CNVR133	1556	12	51099806	51123003	gain	5717	23672762	60040247	Body_weight_(birth)	region1inRegion2	23197
CNVR132	1555	12	50924636	50947287	gain	5949	23672762	60040247	Body_weight_(birth)	region1inRegion2	22651
CNVR133	1556	12	51099806	51123003	gain	5949	23672762	60040247	Body_weight_(birth)	region1inRegion2	23197
CNVR132	1555	12	50924636	50947287	gain	5990	23672762	60040247	Average_backfat_thickness	region1inRegion2	22651
CNVR133	1556	12	51099806	51123003	gain	5990	23672762	60040247	Average_backfat_thickness	region1inRegion2	23197
CNVR132	1555	12	50924636	50947287	gain	9844	23672762	60040247	pH_45_minutes_post_mortem	region1inRegion2	22651
CNVR133	1556	12	51099806	51123003	gain	9844	23672762	60040247	pH_45_minutes_post_mortem	region1inRegion2	23197
CNVR132	1555	12	50924636	50947287	gain	9845	23672762	60040247	pH_24_hr_post-mortem_(loin)	region1inRegion2	22651
CNVR133	1556	12	51099806	51123003	gain	9845	23672762	60040247	pH_24_hr_post-mortem_(loin)	region1inRegion2	23197
CNVR132	1555	12	50924636	50947287	gain	9846	23672762	60040247	Drip_loss	region1inRegion2	22651
CNVR133	1556	12	51099806	51123003	gain	9846	23672762	60040247	Drip_loss	region1inRegion2	23197
CNVR132	1555	12	50924636	50947287	gain	29708	26242726	54022075	Body_weight_(20_weeks)	region1inRegion2	22651
CNVR133	1556	12	51099806	51123003	gain	29708	26242726	54022075	Body_weight_(20_weeks)	region1inRegion2	23197
CNVR132	1555	12	50924636	50947287	gain	4212	38822400	51782740	subjective_abnormal_flavor_in_fat	region1inRegion2	22651
CNVR133	1556	12	51099806	51123003	gain	4212	38822400	51782740	subjective_abnormal_flavor_in_fat	region1inRegion2	23197
CNVR132	1555	12	50924636	50947287	gain	2958	38822400	52098502	Meat_color-L	region1inRegion2	22651
CNVR133	1556	12	51099806	51123003	gain	2958	38822400	52098502	Meat_color-L	region1inRegion2	23197
CNVR132	1555	12	50924636	50947287	gain	3288	38822400	59386165	Marbling	region1inRegion2	22651
CNVR133	1556	12	51099806	51123003	gain	3288	38822400	59386165	Marbling	region1inRegion2	23197
CNVR132	1555	12	50924636	50947287	gain	1125	38822400	62961334	Heart_weight	region1inRegion2	22651
CNVR133	1556	12	51099806	51123003	gain	1125	38822400	62961334	Heart_weight	region1inRegion2	23197
CNVR132	1555	12	50924636	50947287	gain	1126	38822400	62961334	Ham_weight	region1inRegion2	22651
CNVR133	1556	12	51099806	51123003	gain	1126	38822400	62961334	Ham_weight	region1inRegion2	23197
CNVR132	1555	12	50924636	50947287	gain	111	40771258	57583213	chew_score	region1inRegion2	22651
CNVR133	1556	12	51099806	51123003	gain	111	40771258	57583213	chew_score	region1inRegion2	23197
CNVR132	1555	12	50924636	50947287	gain	15075	42935689	62961334	Creatinine_level	region1inRegion2	22651
CNVR133	1556	12	51099806	51123003	gain	15075	42935689	62961334	Creatinine_level	region1inRegion2	23197
CNVR132	1555	12	50924636	50947287	gain	29709	42935689	62961334	Carcass_weight_(hot)	region1inRegion2	22651
CNVR133	1556	12	51099806	51123003	gain	29709	42935689	62961334	Carcass_weight_(hot)	region1inRegion2	23197
CNVR132	1555	12	50924636	50947287	gain	38066	42935689	62961334	Meat_color_chroma	region1inRegion2	22651
CNVR133	1556	12	51099806	51123003	gain	38066	42935689	62961334	Meat_color_chroma	region1inRegion2	23197
CNVR132	1555	12	50924636	50947287	gain	2957	47927603	52098502	Meat_color-L	region1inRegion2	22651
CNVR133	1556	12	51099806	51123003	gain	2957	47927603	52098502	Meat_color-L	region1inRegion2	23197
CNVR132	1555	12	50924636	50947287	gain	2959	47927603	52098502	Meat_color-L	region1inRegion2	22651
CNVR133	1556	12	51099806	51123003	gain	2959	47927603	52098502	Meat_color-L	region1inRegion2	23197
CNVR132	1555	12	50924636	50947287	gain	21403	47927603	59386165	CIE-a*	region1inRegion2	22651
CNVR133	1556	12	51099806	51123003	gain	21403	47927603	59386165	CIE-a*	region1inRegion2	23197
CNVR132	1555	12	50924636	50947287	gain	3788	47927603	59386165	Carcass_weight_(cold)	region1inRegion2	22651
CNVR133	1556	12	51099806	51123003	gain	3788	47927603	59386165	Carcass_weight_(cold)	region1inRegion2	23197
CNVR132	1555	12	50924636	50947287	gain	3824	47927603	59386165	Backfat_at_tenth_rib	region1inRegion2	22651
CNVR133	1556	12	51099806	51123003	gain	3824	47927603	59386165	Backfat_at_tenth_rib	region1inRegion2	23197
CNVR132	1555	12	50924636	50947287	gain	12844	47927603	62961334	Adipocyte_diameter	region1inRegion2	22651
CNVR133	1556	12	51099806	51123003	gain	12844	47927603	62961334	Adipocyte_diameter	region1inRegion2	23197
CNVR132	1555	12	50924636	50947287	gain	38057	47927603	62961334	Loin_muscle_area	region1inRegion2	22651
CNVR133	1556	12	51099806	51123003	gain	38057	47927603	62961334	Loin_muscle_area	region1inRegion2	23197
CNVR132	1555	12	50924636	50947287	gain	38058	47927603	62961334	Loin_muscle_area	region1inRegion2	22651
CNVR133	1556	12	51099806	51123003	gain	38058	47927603	62961334	Loin_muscle_area	region1inRegion2	23197
CNVR132	1555	12	50924636	50947287	gain	38059	47927603	62961334	Marbling	region1inRegion2	22651
CNVR133	1556	12	51099806	51123003	gain	38059	47927603	62961334	Marbling	region1inRegion2	23197
CNVR132	1555	12	50924636	50947287	gain	38060	47927603	62961334	Loin_fat_percentage	region1inRegion2	22651
CNVR133	1556	12	51099806	51123003	gain	38060	47927603	62961334	Loin_fat_percentage	region1inRegion2	23197
CNVR132	1555	12	50924636	50947287	gain	38061	47927603	62961334	Shear_force	region1inRegion2	22651
CNVR133	1556	12	51099806	51123003	gain	38061	47927603	62961334	Shear_force	region1inRegion2	23197
CNVR132	1555	12	50924636	50947287	gain	38062	47927603	62961334	Drip_loss	region1inRegion2	22651
CNVR133	1556	12	51099806	51123003	gain	38062	47927603	62961334	Drip_loss	region1inRegion2	23197
CNVR132	1555	12	50924636	50947287	gain	38064	47927603	62961334	CIE-a*	region1inRegion2	22651
CNVR133	1556	12	51099806	51123003	gain	38064	47927603	62961334	CIE-a*	region1inRegion2	23197
CNVR132	1555	12	50924636	50947287	gain	5424	47927603	62961334	Mean_corpuscular_hemoglobin_content	region1inRegion2	22651
CNVR133	1556	12	51099806	51123003	gain	5424	47927603	62961334	Mean_corpuscular_hemoglobin_content	region1inRegion2	23197
CNVR132	1555	12	50924636	50947287	gain	6341	47927603	62961334	Base_excess	region1inRegion2	22651
CNVR133	1556	12	51099806	51123003	gain	6341	47927603	62961334	Base_excess	region1inRegion2	23197
CNVR132	1555	12	50924636	50947287	gain	7021	47927603	62961334	Percentage_type_IIb_fibers	region1inRegion2	22651
CNVR133	1556	12	51099806	51123003	gain	7021	47927603	62961334	Percentage_type_IIb_fibers	region1inRegion2	23197
CNVR132	1555	12	50924636	50947287	gain	7036	47927603	62961334	Percentage_type_IIb_fibers	region1inRegion2	22651
CNVR133	1556	12	51099806	51123003	gain	7036	47927603	62961334	Percentage_type_IIb_fibers	region1inRegion2	23197
CNVR132	1555	12	50924636	50947287	gain	66300	50712041	52389776	Teat_number	region1inRegion2	22651
CNVR133	1556	12	51099806	51123003	gain	66300	50712041	52389776	Teat_number	region1inRegion2	23197
CNVR132	1555	12	50924636	50947287	gain	106186	50926475	50926515	Loin_muscle_area	region2inRegion1	40
CNVR132	1555	12	50924636	50947287	gain	124274	50926475	50926515	Coping_behavior	region2inRegion1	40
CNVR132	1555	12	50924636	50947287	gain	124458	50926475	50926515	Coping_behavior	region2inRegion1	40
CNVR132	1555	12	50924636	50947287	gain	124459	50926475	50926515	Coping_behavior	region2inRegion1	40
CNVR132	1555	12	50924636	50947287	gain	124770	50926475	50926515	Coping_behavior	region2inRegion1	40
CNVR132	1555	12	50924636	50947287	gain	124771	50926475	50926515	Coping_behavior	region2inRegion1	40
CNVR132	1555	12	50924636	50947287	gain	124959	50926475	50926515	Coping_behavior	region2inRegion1	40
CNVR132	1555	12	50924636	50947287	gain	124960	50926475	50926515	Coping_behavior	region2inRegion1	40
CNVR133	1556	12	51099806	51123003	gain	66278	51064712	51236261	Body_weight	region1inRegion2	23197
CNVR134	1573	13	16191826	16221816	gain	11574	88939	18698443	Salmonella_count_in_spleen	region1inRegion2	29990
CNVR134	1573	13	16191826	16221816	gain	493	88939	27210688	Corpus_luteum_number	region1inRegion2	29990
CNVR135	1578	13	19139627	19152872	gain	493	88939	27210688	Corpus_luteum_number	region1inRegion2	13245
CNVR134	1573	13	16191826	16221816	gain	652	88939	27210688	Average_backfat_thickness	region1inRegion2	29990
CNVR135	1578	13	19139627	19152872	gain	652	88939	27210688	Average_backfat_thickness	region1inRegion2	13245
CNVR134	1573	13	16191826	16221816	gain	244	88939	208183945	Average_daily_gain	region1inRegion2	29990
CNVR135	1578	13	19139627	19152872	gain	244	88939	208183945	Average_daily_gain	region1inRegion2	13245
CNVR136	1589	13	30640874	30643949	gain	244	88939	208183945	Average_daily_gain	region1inRegion2	3075
CNVR137	1593	13	40598705	40614627	gain	244	88939	208183945	Average_daily_gain	region1inRegion2	15922
CNVR138	1636	13	111201690	111225859	gain	244	88939	208183945	Average_daily_gain	region1inRegion2	24169
CNVR139	1656	13	131028348	131044332	gain	244	88939	208183945	Average_daily_gain	region1inRegion2	15984
CNVR140	1690	13	164895338	164898801	gain	244	88939	208183945	Average_daily_gain	region1inRegion2	3463
CNVR141	1693	13	166109149	166114547	gain	244	88939	208183945	Average_daily_gain	region1inRegion2	5398
CNVR142	1695	13	167567440	167600641	gain	244	88939	208183945	Average_daily_gain	region1inRegion2	33201
CNVR143	1699	13	170765271	170819670	gain	244	88939	208183945	Average_daily_gain	region1inRegion2	54399
CNVR144	1702	13	171285806	171311772	gain	244	88939	208183945	Average_daily_gain	region1inRegion2	25966
CNVR134	1573	13	16191826	16221816	gain	37578	88939	209907072	Actinobacillus_pleuropneumoniae_susceptibility	region1inRegion2	29990
CNVR135	1578	13	19139627	19152872	gain	37578	88939	209907072	Actinobacillus_pleuropneumoniae_susceptibility	region1inRegion2	13245
CNVR136	1589	13	30640874	30643949	gain	37578	88939	209907072	Actinobacillus_pleuropneumoniae_susceptibility	region1inRegion2	3075
CNVR137	1593	13	40598705	40614627	gain	37578	88939	209907072	Actinobacillus_pleuropneumoniae_susceptibility	region1inRegion2	15922
CNVR138	1636	13	111201690	111225859	gain	37578	88939	209907072	Actinobacillus_pleuropneumoniae_susceptibility	region1inRegion2	24169
CNVR139	1656	13	131028348	131044332	gain	37578	88939	209907072	Actinobacillus_pleuropneumoniae_susceptibility	region1inRegion2	15984
CNVR140	1690	13	164895338	164898801	gain	37578	88939	209907072	Actinobacillus_pleuropneumoniae_susceptibility	region1inRegion2	3463
CNVR141	1693	13	166109149	166114547	gain	37578	88939	209907072	Actinobacillus_pleuropneumoniae_susceptibility	region1inRegion2	5398
CNVR142	1695	13	167567440	167600641	gain	37578	88939	209907072	Actinobacillus_pleuropneumoniae_susceptibility	region1inRegion2	33201
CNVR143	1699	13	170765271	170819670	gain	37578	88939	209907072	Actinobacillus_pleuropneumoniae_susceptibility	region1inRegion2	54399
CNVR144	1702	13	171285806	171311772	gain	37578	88939	209907072	Actinobacillus_pleuropneumoniae_susceptibility	region1inRegion2	25966
CNVR134	1573	13	16191826	16221816	gain	238	5836383	16559958	Meat_color-b	region1inRegion2	29990
CNVR134	1573	13	16191826	16221816	gain	5718	5836383	27210688	Average_backfat_thickness	region1inRegion2	29990
CNVR135	1578	13	19139627	19152872	gain	5718	5836383	27210688	Average_backfat_thickness	region1inRegion2	13245
CNVR134	1573	13	16191826	16221816	gain	5719	5836383	27210688	Backfat_at_tenth_rib	region1inRegion2	29990
CNVR135	1578	13	19139627	19152872	gain	5719	5836383	27210688	Backfat_at_tenth_rib	region1inRegion2	13245
CNVR134	1573	13	16191826	16221816	gain	5721	5836383	27210688	Average_daily_gain	region1inRegion2	29990
CNVR135	1578	13	19139627	19152872	gain	5721	5836383	27210688	Average_daily_gain	region1inRegion2	13245
CNVR134	1573	13	16191826	16221816	gain	5924	5836383	27210688	Time_spent_socializing	region1inRegion2	29990
CNVR135	1578	13	19139627	19152872	gain	5924	5836383	27210688	Time_spent_socializing	region1inRegion2	13245

CNVR134	1573	13	16191826	16221816	gain	7514	5836383	27210688	Body_temperature	region1inRegion2	29990
CNVR135	1578	13	19139627	19152872	gain	7514	5836383	27210688	Body_temperature	region1inRegion2	13245
CNVR134	1573	13	16191826	16221816	gain	7515	5836383	27210688	Sarcocystis_miescheriana_IgM_levels	region1inRegion2	29990
CNVR135	1578	13	19139627	19152872	gain	7515	5836383	27210688	Sarcocystis_miescheriana_IgM_levels	region1inRegion2	13245
CNVR134	1573	13	16191826	16221816	gain	11578	5836383	145083047	Salmonella_count_in_spleen	region1inRegion2	29990
CNVR135	1578	13	19139627	19152872	gain	11578	5836383	145083047	Salmonella_count_in_spleen	region1inRegion2	13245
CNVR136	1589	13	30640874	30643949	gain	11578	5836383	145083047	Salmonella_count_in_spleen	region1inRegion2	3075
CNVR137	1593	13	40598705	40614627	gain	11578	5836383	145083047	Salmonella_count_in_spleen	region1inRegion2	15922
CNVR138	1636	13	111201690	111225859	gain	11578	5836383	145083047	Salmonella_count_in_spleen	region1inRegion2	24169
CNVR139	1656	13	131028348	131044332	gain	11578	5836383	145083047	Salmonella_count_in_spleen	region1inRegion2	15984
CNVR134	1573	13	16191826	16221816	gain	7479	5836383	188271972	Nonfunctional_nipples	region1inRegion2	29990
CNVR135	1578	13	19139627	19152872	gain	7479	5836383	188271972	Nonfunctional_nipples	region1inRegion2	13245
CNVR136	1589	13	30640874	30643949	gain	7479	5836383	188271972	Nonfunctional_nipples	region1inRegion2	3075
CNVR137	1593	13	40598705	40614627	gain	7479	5836383	188271972	Nonfunctional_nipples	region1inRegion2	15922
CNVR138	1636	13	111201690	111225859	gain	7479	5836383	188271972	Nonfunctional_nipples	region1inRegion2	24169
CNVR139	1656	13	131028348	131044332	gain	7479	5836383	188271972	Nonfunctional_nipples	region1inRegion2	15984
CNVR140	1690	13	164895338	164898801	gain	7479	5836383	188271972	Nonfunctional_nipples	region1inRegion2	3463
CNVR141	1693	13	166109149	166114547	gain	7479	5836383	188271972	Nonfunctional_nipples	region1inRegion2	5398
CNVR142	1695	13	167567440	167600641	gain	7479	5836383	188271972	Nonfunctional_nipples	region1inRegion2	33201
CNVR143	1699	13	170765271	170819670	gain	7479	5836383	188271972	Nonfunctional_nipples	region1inRegion2	54399
CNVR144	1702	13	171285806	171311772	gain	7479	5836383	188271972	Nonfunctional_nipples	region1inRegion2	25966
CNVR134	1573	13	16191826	16221816	gain	17725	5836383	194995520	LDL_cholesterol	region1inRegion2	29990
CNVR135	1578	13	19139627	19152872	gain	17725	5836383	194995520	LDL_cholesterol	region1inRegion2	13245
CNVR136	1589	13	30640874	30643949	gain	17725	5836383	194995520	LDL_cholesterol	region1inRegion2	3075
CNVR137	1593	13	40598705	40614627	gain	17725	5836383	194995520	LDL_cholesterol	region1inRegion2	15922
CNVR138	1636	13	111201690	111225859	gain	17725	5836383	194995520	LDL_cholesterol	region1inRegion2	24169
CNVR139	1656	13	131028348	131044332	gain	17725	5836383	194995520	LDL_cholesterol	region1inRegion2	15984
CNVR140	1690	13	164895338	164898801	gain	17725	5836383	194995520	LDL_cholesterol	region1inRegion2	3463
CNVR141	1693	13	166109149	166114547	gain	17725	5836383	194995520	LDL_cholesterol	region1inRegion2	5398
CNVR142	1695	13	167567440	167600641	gain	17725	5836383	194995520	LDL_cholesterol	region1inRegion2	33201
CNVR143	1699	13	170765271	170819670	gain	17725	5836383	194995520	LDL_cholesterol	region1inRegion2	54399
CNVR144	1702	13	171285806	171311772	gain	17725	5836383	194995520	LDL_cholesterol	region1inRegion2	25966
CNVR134	1573	13	16191826	16221816	gain	15118	7359728	131021249	Lactate_dehydrogenase_level	region1inRegion2	29990
CNVR135	1578	13	19139627	19152872	gain	15118	7359728	131021249	Lactate_dehydrogenase_level	region1inRegion2	13245
CNVR136	1589	13	30640874	30643949	gain	15118	7359728	131021249	Lactate_dehydrogenase_level	region1inRegion2	3075
CNVR137	1593	13	40598705	40614627	gain	15118	7359728	131021249	Lactate_dehydrogenase_level	region1inRegion2	15922
CNVR138	1636	13	111201690	111225859	gain	15118	7359728	131021249	Lactate_dehydrogenase_level	region1inRegion2	24169
CNVR134	1573	13	16191826	16221816	gain	38067	9843648	32577445	Loin_muscle_area	region1inRegion2	29990
CNVR135	1578	13	19139627	19152872	gain	38067	9843648	32577445	Loin_muscle_area	region1inRegion2	13245
CNVR136	1589	13	30640874	30643949	gain	38067	9843648	32577445	Loin_muscle_area	region1inRegion2	3075
CNVR134	1573	13	16191826	16221816	gain	8824	9843648	45496370	Age_at_puberty	region1inRegion2	29990
CNVR135	1578	13	19139627	19152872	gain	8824	9843648	45496370	Age_at_puberty	region1inRegion2	13245
CNVR136	1589	13	30640874	30643949	gain	8824	9843648	45496370	Age_at_puberty	region1inRegion2	3075
CNVR137	1593	13	40598705	40614627	gain	8824	9843648	45496370	Age_at_puberty	region1inRegion2	15922
CNVR134	1573	13	16191826	16221816	gain	8826	9843648	45496370	Corpus_luteum_number	region1inRegion2	29990
CNVR135	1578	13	19139627	19152872	gain	8826	9843648	45496370	Corpus_luteum_number	region1inRegion2	13245
CNVR136	1589	13	30640874	30643949	gain	8826	9843648	45496370	Corpus_luteum_number	region1inRegion2	3075
CNVR137	1593	13	40598705	40614627	gain	8826	9843648	45496370	Corpus_luteum_number	region1inRegion2	15922
CNVR134	1573	13	16191826	16221816	gain	38068	9843648	145083047	Loin_muscle_area	region1inRegion2	29990
CNVR135	1578	13	19139627	19152872	gain	38068	9843648	145083047	Loin_muscle_area	region1inRegion2	13245
CNVR136	1589	13	30640874	30643949	gain	38068	9843648	145083047	Loin_muscle_area	region1inRegion2	3075
CNVR137	1593	13	40598705	40614627	gain	38068	9843648	145083047	Loin_muscle_area	region1inRegion2	15922
CNVR138	1636	13	111201690	111225859	gain	38068	9843648	145083047	Loin_muscle_area	region1inRegion2	24169
CNVR139	1656	13	131028348	131044332	gain	38068	9843648	145083047	Loin_muscle_area	region1inRegion2	15984
CNVR134	1573	13	16191826	16221816	gain	15078	9843648	206710160	Triglyceride_level	region1inRegion2	29990
CNVR135	1578	13	19139627	19152872	gain	15078	9843648	206710160	Triglyceride_level	region1inRegion2	13245
CNVR136	1589	13	30640874	30643949	gain	15078	9843648	206710160	Triglyceride_level	region1inRegion2	3075
CNVR137	1593	13	40598705	40614627	gain	15078	9843648	206710160	Triglyceride_level	region1inRegion2	15922
CNVR138	1636	13	111201690	111225859	gain	15078	9843648	206710160	Triglyceride_level	region1inRegion2	24169
CNVR139	1656	13	131028348	131044332	gain	15078	9843648	206710160	Triglyceride_level	region1inRegion2	15984
CNVR140	1690	13	164895338	164898801	gain	15078	9843648	206710160	Triglyceride_level	region1inRegion2	3463
CNVR141	1693	13	166109149	166114547	gain	15078	9843648	206710160	Triglyceride_level	region1inRegion2	5398
CNVR142	1695	13	167567440	167600641	gain	15078	9843648	206710160	Triglyceride_level	region1inRegion2	33201
CNVR143	1699	13	170765271	170819670	gain	15078	9843648	206710160	Triglyceride_level	region1inRegion2	54399
CNVR144	1702	13	171285806	171311772	gain	15078	9843648	206710160	Triglyceride_level	region1inRegion2	25966
CNVR134	1573	13	16191826	16221816	gain	21843	12401583	32577445	Body_weight_(weaning)	region1inRegion2	29990
CNVR135	1578	13	19139627	19152872	gain	21843	12401583	32577445	Body_weight_(weaning)	region1inRegion2	13245
CNVR136	1589	13	30640874	30643949	gain	21843	12401583	32577445	Body_weight_(weaning)	region1inRegion2	3075
CNVR134	1573	13	16191826	16221816	gain	38107	12401583	185385437	Shear_force	region1inRegion2	29990
CNVR135	1578	13	19139627	19152872	gain	38107	12401583	185385437	Shear_force	region1inRegion2	13245
CNVR136	1589	13	30640874	30643949	gain	38107	12401583	185385437	Shear_force	region1inRegion2	3075
CNVR137	1593	13	40598705	40614627	gain	38107	12401583	185385437	Shear_force	region1inRegion2	15922
CNVR138	1636	13	111201690	111225859	gain	38107	12401583	185385437	Shear_force	region1inRegion2	24169
CNVR139	1656	13	131028348	131044332	gain	38107	12401583	185385437	Shear_force	region1inRegion2	15984
CNVR140	1690	13	164895338	164898801	gain	38107	12401583	185385437	Shear_force	region1inRegion2	3463
CNVR141	1693	13	166109149	166114547	gain	38107	12401583	185385437	Shear_force	region1inRegion2	5398
CNVR142	1695	13	167567440	167600641	gain	38107	12401583	185385437	Shear_force	region1inRegion2	33201
CNVR143	1699	13	170765271	170819670	gain	38107	12401583	185385437	Shear_force	region1inRegion2	54399
CNVR144	1702	13	171285806	171311772	gain	38107	12401583	185385437	Shear_force	region1inRegion2	25966
CNVR134	1573	13	16191826	16221816	gain	38109	12401583	203392786	CIE-a*	region1inRegion2	29990
CNVR135	1578	13	19139627	19152872	gain	38109	12401583	203392786	CIE-a*	region1inRegion2	13245
CNVR136	1589	13	30640874	30643949	gain	38109	12401583	203392786	CIE-a*	region1inRegion2	3075
CNVR137	1593	13	40598705	40614627	gain	38109	12401583	203392786	CIE-a*	region1inRegion2	15922
CNVR138	1636	13	111201690	111225859	gain	38109	12401583	203392786	CIE-a*	region1inRegion2	24169
CNVR139	1656	13	131028348	131044332	gain	38109	12401583	203392786	CIE-a*	region1inRegion2	15984
CNVR140	1690	13	164895338	164898801	gain	38109	12401583	203392786	CIE-a*	region1inRegion2	3463
CNVR141	1693	13	166109149	166114547	gain	38109	12401583	203392786	CIE-a*	region1inRegion2	5398
CNVR142	1695	13	167567440	167600641	gain	38109	12401583	203392786	CIE-a*	region1inRegion2	33201
CNVR143	1699	13	170765271	170819670	gain	38109	12401583	203392786	CIE-a*	region1inRegion2	54399
CNVR144	1702	13	171285806	171311772	gain	38109	12401583	203392786	CIE-a*	region1inRegion2	25966
CNVR134	1573	13	16191826	16221816	gain	3003	13078106	32577445	Juiciness_score	region1inRegion2	29990
CNVR135	1578	13	19139627	19152872	gain	3003	13078106	32577445	Juiciness_score	region1inRegion2	13245
CNVR136	1589	13	30640874	30643949	gain	3003	13078106	32577445	Juiciness_score	region1inRegion2	3075
CNVR134	1573	13	16191826	16221816	gain	3073	13078106	32577445	pH_45_minutes_post_mortem	region1inRegion2	29990
CNVR135	1578	13	19139627	19152872	gain	3073	13078106	32577445	pH_45_minutes_post_mortem	region1inRegion2	13245
CNVR136	1589	13	30640874	30643949	gain	3073	13078106	32577445	pH_45_minutes_post_mortem	region1inRegion2	3075
CNVR134	1573	13	16191826	16221816	gain	3075	13078106	32577445	Inside_ham_weight	region1inRegion2	29990
CNVR135	1578	13	19139627	19152872	gain	3075	13078106	32577445	Inside_ham_weight	region1inRegion2	13245
CNVR136	1589	13	30640874	30643949	gain	3075	13078106	32577445	Inside_ham_weight	region1inRegion2	3075
CNVR134	1573	13	16191826	16221816	gain	3078	13078106	32577445	Ham_meat_weight	region1inRegion2	29990
CNVR135	1578	13	19139627	19152872	gain	3078	13078106	32577445	Ham_meat_weight	region1inRegion2	13245
CNVR136	1589	13	30640874	30643949	gain	3078	13078106	32577445	Ham_meat_weight	region1inRegion2	3075
CNVR134	1573	13	16191826	16221816	gain	5509	14497580	32577445	Loin_muscle_depth	region1inRegion2	29990
CNVR135	1578	13	19139627	19152872	gain	5509	14497580	32577445	Loin_muscle_depth	region1inRegion2	13245
CNVR136	1589	13	30640874	30643949	gain	5509	14497580	32577445	Loin_muscle_depth	region1inRegion2	3075
CNVR134	1573	13	16191826	16221816	gain	38069	16163521	145083047	Loin_fat_percentage	region1inRegion2	29990
CNVR135	1578	13	19139627	19152872	gain	38069	16163521	145083047	Loin_fat_percentage	region1inRegion2	13245
CNVR136	1589	13	30640874	30643949	gain	38069	16163521	145083047	Loin_fat_percentage	region1inRegion2	3075
CNVR137	1593	13	40598705	40614627	gain	38069	16163521	145083047	Loin_fat_percentage	region1inRegion2	15922
CNVR138	1636	13	111201690	111225859	gain	38069	16163521	145083047	Loin_fat_percentage	region1inRegion2	24169
CNVR139	1656	13	131028348	131044332	gain	38069	16163521	145083047	Loin_fat_percentage	region1inRegion2	15984
CNVR134	1573	13	16191826	16221816	gain	38108	16163521	185385437	Muscle_moisture_percentage	region1inRegion2	29990
CNVR135	1578	13	19139627	19152872	gain	38108	16163521	185385437	Muscle_moisture_percentage	region1inRegion2	13245
CNVR136	1589	13	30640874	30643949	gain	38108	16163521	185385437	Muscle_moisture_percentage	region1inRegion2	3075
CNVR137	1593	13	40598705	40614627	gain	38108	16163521	185385437	Muscle_moisture_percentage	region1inRegion2	15922
CNVR138	1636	13	111201690	111225859	gain	38108	16163521	185385437	Muscle_moisture_percentage	region1inRegion2	24169
CNVR139	1656	13	131028348	131044332	gain	38108	16163521	185385437	Muscle_moisture_percentage	region1inRegion2	15984
CNVR140	1690	13	164895338	164898801	gain	38108	16163521	185385437	Muscle_moisture_percentage	region1inRegion2	3463
CNVR141	1693	13	166109149	166114547	gain	38108	16163521	185385437	Muscle_moisture_percentage	region1inRegion2	5398
CNVR142	1695	13	167567440	167600641	gain	38108	16163521	185385437	Muscle_moisture_percentage	region1inRegion2	33201
CNVR143	1699	13	170765271	170819670	gain	38108	16163521	185385437	Muscle_moisture_percentage	region1inRegion2	54399
CNVR144	1702	13	171285806	171311772	gain	38108	16163521	185385437	Muscle_moisture_percentage	region1inRegion2	25966

CNVR134	1573	13	16191826	16221816	gain	9075	16180362	16538167	CIE-L*	region1inRegion2	29990
CNVR135	1578	13	19139627	19152872	gain	1147	16559958	58927444	Conductivity_45_minutes_post-mortem	region1inRegion2	13245
CNVR136	1589	13	30640874	30643949	gain	1147	16559958	58927444	Conductivity_45_minutes_post-mortem	region1inRegion2	3075
CNVR137	1593	13	40598705	40614627	gain	1147	16559958	58927444	Conductivity_45_minutes_post-mortem	region1inRegion2	15922
CNVR135	1578	13	19139627	19152872	gain	16840	16559958	67299272	backfat_at_last_rib	region1inRegion2	13245
CNVR136	1589	13	30640874	30643949	gain	16840	16559958	67299272	backfat_at_last_rib	region1inRegion2	3075
CNVR137	1593	13	40598705	40614627	gain	16840	16559958	67299272	backfat_at_last_rib	region1inRegion2	15922
CNVR135	1578	13	19139627	19152872	gain	1132	16559958	130538722	Backfat_weight	region1inRegion2	13245
CNVR136	1589	13	30640874	30643949	gain	1132	16559958	130538722	Backfat_weight	region1inRegion2	3075
CNVR137	1593	13	40598705	40614627	gain	1132	16559958	130538722	Backfat_weight	region1inRegion2	15922
CNVR138	1636	13	111201690	111225859	gain	1132	16559958	130538722	Backfat_weight	region1inRegion2	24169
CNVR135	1578	13	19139627	19152872	gain	328	16559958	130538722	Average_daily_gain	region1inRegion2	13245
CNVR136	1589	13	30640874	30643949	gain	328	16559958	130538722	Average_daily_gain	region1inRegion2	3075
CNVR137	1593	13	40598705	40614627	gain	328	16559958	130538722	Average_daily_gain	region1inRegion2	15922
CNVR138	1636	13	111201690	111225859	gain	328	16559958	130538722	Average_daily_gain	region1inRegion2	24169
CNVR135	1578	13	19139627	19152872	gain	336	16559958	130538722	Average_daily_gain	region1inRegion2	13245
CNVR136	1589	13	30640874	30643949	gain	336	16559958	130538722	Average_daily_gain	region1inRegion2	3075
CNVR137	1593	13	40598705	40614627	gain	336	16559958	130538722	Average_daily_gain	region1inRegion2	15922
CNVR138	1636	13	111201690	111225859	gain	336	16559958	130538722	Average_daily_gain	region1inRegion2	24169
CNVR135	1578	13	19139627	19152872	gain	347	16559958	130538722	Average_daily_gain	region1inRegion2	13245
CNVR136	1589	13	30640874	30643949	gain	347	16559958	130538722	Average_daily_gain	region1inRegion2	3075
CNVR137	1593	13	40598705	40614627	gain	347	16559958	130538722	Average_daily_gain	region1inRegion2	15922
CNVR138	1636	13	111201690	111225859	gain	347	16559958	130538722	Average_daily_gain	region1inRegion2	24169
CNVR135	1578	13	19139627	19152872	gain	4226	16559958	130538722	skatole,_laboratory	region1inRegion2	13245
CNVR136	1589	13	30640874	30643949	gain	4226	16559958	130538722	skatole,_laboratory	region1inRegion2	3075
CNVR137	1593	13	40598705	40614627	gain	4226	16559958	130538722	skatole,_laboratory	region1inRegion2	15922
CNVR138	1636	13	111201690	111225859	gain	4226	16559958	130538722	skatole,_laboratory	region1inRegion2	24169
CNVR135	1578	13	19139627	19152872	gain	4227	16559958	130538722	androstenone,_sensory_panel	region1inRegion2	13245
CNVR136	1589	13	30640874	30643949	gain	4227	16559958	130538722	androstenone,_sensory_panel	region1inRegion2	3075
CNVR137	1593	13	40598705	40614627	gain	4227	16559958	130538722	androstenone,_sensory_panel	region1inRegion2	15922
CNVR138	1636	13	111201690	111225859	gain	4227	16559958	130538722	androstenone,_sensory_panel	region1inRegion2	24169
CNVR135	1578	13	19139627	19152872	gain	24285	18268056	206704152	Corpus_luteum_number	region1inRegion2	13245
CNVR136	1589	13	30640874	30643949	gain	24285	18268056	206704152	Corpus_luteum_number	region1inRegion2	3075
CNVR137	1593	13	40598705	40614627	gain	24285	18268056	206704152	Corpus_luteum_number	region1inRegion2	15922
CNVR138	1636	13	111201690	111225859	gain	24285	18268056	206704152	Corpus_luteum_number	region1inRegion2	24169
CNVR139	1656	13	131028348	131044332	gain	24285	18268056	206704152	Corpus_luteum_number	region1inRegion2	15984
CNVR140	1690	13	164895338	164898801	gain	24285	18268056	206704152	Corpus_luteum_number	region1inRegion2	3463
CNVR141	1693	13	166109149	166114547	gain	24285	18268056	206704152	Corpus_luteum_number	region1inRegion2	5398
CNVR142	1695	13	167567440	167600641	gain	24285	18268056	206704152	Corpus_luteum_number	region1inRegion2	33201
CNVR143	1699	13	170765271	170819670	gain	24285	18268056	206704152	Corpus_luteum_number	region1inRegion2	54399
CNVR144	1702	13	171285806	171311772	gain	24285	18268056	206704152	Corpus_luteum_number	region1inRegion2	25966
CNVR135	1578	13	19139627	19152872	gain	21265	18672597	97578060	intestinal_fat_weight	region1inRegion2	13245
CNVR136	1589	13	30640874	30643949	gain	21265	18672597	97578060	intestinal_fat_weight	region1inRegion2	3075
CNVR137	1593	13	40598705	40614627	gain	21265	18672597	97578060	intestinal_fat_weight	region1inRegion2	15922
CNVR135	1578	13	19139627	19152872	gain	21681	18698443	19329922	Conductivity_24_hours_postmortem_(ham)	region1inRegion2	13245
CNVR135	1578	13	19139627	19152872	gain	21682	18698443	19329922	Conductivity_24_hours_postmortem_(ham)	region1inRegion2	13245
CNVR135	1578	13	19139627	19152872	gain	3095	18698443	19329922	Average_daily_gain	region1inRegion2	13245
CNVR136	1589	13	30640874	30643949	gain	17850	27210688	58927444	CD4-negative,_CD8-positive_leukocyte_percentage	region1inRegion2	3075
CNVR137	1593	13	40598705	40614627	gain	17850	27210688	58927444	CD4-negative,_CD8-positive_leukocyte_percentage	region1inRegion2	15922
CNVR136	1589	13	30640874	30643949	gain	17851	27210688	58927444	CD4-negative,_CD8-negative_leukocyte_percentage	region1inRegion2	3075
CNVR137	1593	13	40598705	40614627	gain	17851	27210688	58927444	CD4-negative,_CD8-negative_leukocyte_percentage	region1inRegion2	15922
CNVR136	1589	13	30640874	30643949	gain	17852	27210688	58927444	CD8-positive_leukocyte_percentage	region1inRegion2	3075
CNVR137	1593	13	40598705	40614627	gain	17852	27210688	58927444	CD8-positive_leukocyte_percentage	region1inRegion2	15922
CNVR136	1589	13	30640874	30643949	gain	17853	27210688	58927444	CD4-positive/CD8-positive_leukocyte_ratio	region1inRegion2	3075
CNVR137	1593	13	40598705	40614627	gain	17853	27210688	58927444	CD4-positive/CD8-positive_leukocyte_ratio	region1inRegion2	15922
CNVR136	1589	13	30640874	30643949	gain	7466	27210688	91206297	Nonfunctional_nipples	region1inRegion2	3075
CNVR137	1593	13	40598705	40614627	gain	7466	27210688	91206297	Nonfunctional_nipples	region1inRegion2	15922
CNVR136	1589	13	30640874	30643949	gain	5392	27210688	159374767	Hemoglobin	region1inRegion2	3075
CNVR137	1593	13	40598705	40614627	gain	5392	27210688	159374767	Hemoglobin	region1inRegion2	15922
CNVR138	1636	13	111201690	111225859	gain	5392	27210688	159374767	Hemoglobin	region1inRegion2	24169
CNVR139	1656	13	131028348	131044332	gain	5392	27210688	159374767	Hemoglobin	region1inRegion2	15984
CNVR136	1589	13	30640874	30643949	gain	5398	27210688	159374767	Red_blood_cell_count	region1inRegion2	3075
CNVR137	1593	13	40598705	40614627	gain	5398	27210688	159374767	Red_blood_cell_count	region1inRegion2	15922
CNVR138	1636	13	111201690	111225859	gain	5398	27210688	159374767	Red_blood_cell_count	region1inRegion2	24169
CNVR139	1656	13	131028348	131044332	gain	5398	27210688	159374767	Red_blood_cell_count	region1inRegion2	15984
CNVR136	1589	13	30640874	30643949	gain	5409	27210688	159374767	Red_blood_cell_count	region1inRegion2	3075
CNVR137	1593	13	40598705	40614627	gain	5409	27210688	159374767	Red_blood_cell_count	region1inRegion2	15922
CNVR138	1636	13	111201690	111225859	gain	5409	27210688	159374767	Red_blood_cell_count	region1inRegion2	24169
CNVR139	1656	13	131028348	131044332	gain	5409	27210688	159374767	Red_blood_cell_count	region1inRegion2	15984
CNVR136	1589	13	30640874	30643949	gain	5421	27210688	159374767	hematocrit	region1inRegion2	3075
CNVR137	1593	13	40598705	40614627	gain	5421	27210688	159374767	hematocrit	region1inRegion2	15922
CNVR138	1636	13	111201690	111225859	gain	5421	27210688	159374767	hematocrit	region1inRegion2	24169
CNVR139	1656	13	131028348	131044332	gain	5421	27210688	159374767	hematocrit	region1inRegion2	15984
CNVR136	1589	13	30640874	30643949	gain	5491	27210688	159374767	Basophil_number	region1inRegion2	3075
CNVR137	1593	13	40598705	40614627	gain	5491	27210688	159374767	Basophil_number	region1inRegion2	15922
CNVR138	1636	13	111201690	111225859	gain	5491	27210688	159374767	Basophil_number	region1inRegion2	24169
CNVR139	1656	13	131028348	131044332	gain	5491	27210688	159374767	Basophil_number	region1inRegion2	15984
CNVR136	1589	13	30640874	30643949	gain	5720	27210688	194995520	Subcutanous_fat_area	region1inRegion2	3075
CNVR137	1593	13	40598705	40614627	gain	5720	27210688	194995520	Subcutanous_fat_area	region1inRegion2	15922
CNVR138	1636	13	111201690	111225859	gain	5720	27210688	194995520	Subcutanous_fat_area	region1inRegion2	24169
CNVR139	1656	13	131028348	131044332	gain	5720	27210688	194995520	Subcutanous_fat_area	region1inRegion2	15984
CNVR140	1690	13	164895338	164898801	gain	5720	27210688	194995520	Subcutanous_fat_area	region1inRegion2	3463
CNVR141	1693	13	166109149	166114547	gain	5720	27210688	194995520	Subcutanous_fat_area	region1inRegion2	5398
CNVR142	1695	13	167567440	167600641	gain	5720	27210688	194995520	Subcutanous_fat_area	region1inRegion2	33201
CNVR143	1699	13	170765271	170819670	gain	5720	27210688	194995520	Subcutanous_fat_area	region1inRegion2	54399
CNVR144	1702	13	171285806	171311772	gain	5720	27210688	194995520	Subcutanous_fat_area	region1inRegion2	25966
CNVR136	1589	13	30640874	30643949	gain	55898	30413447	33942128	Scrotal/inguinal_hernia	region1inRegion2	3075
CNVR136	1589	13	30640874	30643949	gain	55965	30413447	33942128	Drip_loss	region1inRegion2	3075
CNVR136	1589	13	30640874	30643949	gain	55992	30413447	33942128	LDL_cholesterol	region1inRegion2	3075
CNVR136	1589	13	30640874	30643949	gain	56002	30413447	33942128	LDL_cholesterol	region1inRegion2	3075
CNVR136	1589	13	30640874	30643949	gain	31884	30561494	30817916	Corpus_luteum_number	region1inRegion2	3075
CNVR137	1593	13	40598705	40614627	gain	1138	32577445	58927444	Conductivity_24_hours_post-mortem	region1inRegion2	15922
CNVR137	1593	13	40598705	40614627	gain	1141	32577445	58927444	Fat-cuts_percentage	region1inRegion2	15922
CNVR137	1593	13	40598705	40614627	gain	1131	32577445	130538722	Conductivity_45_minutes_post-mortem	region1inRegion2	15922
CNVR138	1636	13	111201690	111225859	gain	1131	32577445	130538722	Conductivity_45_minutes_post-mortem	region1inRegion2	24169
CNVR137	1593	13	40598705	40614627	gain	8827	35281335	44974795	Corpus_luteum_number	region1inRegion2	15922
CNVR137	1593	13	40598705	40614627	gain	16978	35281335	45267259	Carcass_length	region1inRegion2	15922
CNVR137	1593	13	40598705	40614627	gain	3004	40046563	130538722	Muscle_moisture_percentage	region1inRegion2	15922
CNVR138	1636	13	111201690	111225859	gain	3004	40046563	130538722	Muscle_moisture_percentage	region1inRegion2	24169
CNVR138	1636	13	111201690	111225859	gain	3970	45441954	209900460	Vertebra_number	region1inRegion2	24169
CNVR139	1656	13	131028348	131044332	gain	3970	45441954	209900460	Vertebra_number	region1inRegion2	15984
CNVR140	1690	13	164895338	164898801	gain	3970	45441954	209900460	Vertebra_number	region1inRegion2	3463
CNVR141	1693	13	166109149	166114547	gain	3970	45441954	209900460	Vertebra_number	region1inRegion2	5398
CNVR142	1695	13	167567440	167600641	gain	3970	45441954	209900460	Vertebra_number	region1inRegion2	33201
CNVR143	1699	13	170765271	170819670	gain	3970	45441954	209900460	Vertebra_number	region1inRegion2	54399
CNVR144	1702	13	171285806	171311772	gain	3970	45441954	209900460	Vertebra_number	region1inRegion2	25966
CNVR138	1636	13	111201690	111225859	gain	5780	48608490	141415709	Loin_muscle_area	region1inRegion2	24169
CNVR139	1656	13	131028348	131044332	gain	5780	48608490	141415709	Loin_muscle_area	region1inRegion2	15984
CNVR138	1636	13	111201690	111225859	gain	4005	54899442	203809783	Temperature_40_minutes_post_mortem_(loin)	region1inRegion2	24169
CNVR139	1656	13	131028348	131044332	gain	4005	54899442	203809783	Temperature_40_minutes_post_mortem_(loin)	region1inRegion2	15984
CNVR140	1690	13	164895338	164898801	gain	4005	54899442	203809783	Temperature_40_minutes_post_mortem_(loin)	region1inRegion2	3463
CNVR141	1693	13	166109149	166114547	gain	4005	54899442	203809783	Temperature_40_minutes_post_mortem_(loin)	region1inRegion2	5398
CNVR142	1695	13	167567440	167600641	gain	4005	54899442	203809783	Temperature_40_minutes_post_mortem_(loin)	region1inRegion2	33201
CNVR143	1699	13	170765271	170819670	gain	4005	54899442	203809783	Temperature_40_minutes_post_mortem_(loin)	region1inRegion2	54399
CNVR144	1702	13	171285806	171311772	gain	4005	54899442	203809783	Temperature_40_minutes_post_mortem_(loin)	region1inRegion2	25966
CNVR138	1636	13	111201690	111225859	gain	18357	58690821	157823757	Embryo_weight	region1inRegion2	24169
CNVR139	1656	13	131028348	131044332	gain	18357	58690821	157823757	Embryo_weight	region1inRegion2	15984
CNVR138	1636	13	111201690	111225859	gain	18641	58927444	130538722	Fat_area_percentage_in_carcass	region1inRegion2	24169
CNVR138	1636	13	111201690	111225859	gain	17810	58927444	172283983	Loin_weight	region1inRegion2	24169
CNVR139	1656	13	131028348	131044332	gain	17810	58927444	172283983	Loin_weight	region1inRegion2	15984
CNVR140	1690	13	164895338	164898801	gain	17810	58927444	172283983	Loin_weight	region1inRegion2	3463
CNVR141	1693	13	166109149	166114547	gain	17810	58927444	172283983	Loin_weight	region1inRegion2	5398

CNVR142	1695	13	167567440	167600641	gain	17810	58927444	172283983	Loin_weight	region1inRegion2	33201
CNVR143	1699	13	170765271	170819670	gain	17810	58927444	172283983	Loin_weight	region1inRegion2	54399
CNVR144	1702	13	171285806	171311772	gain	17810	58927444	172283983	Loin_weight	region1inRegion2	25966
CNVR138	1636	13	111201690	111225859	gain	17784	58927444	194995520	backfat_at_last_rib	region1inRegion2	24169
CNVR139	1656	13	131028348	131044332	gain	17784	58927444	194995520	backfat_at_last_rib	region1inRegion2	15984
CNVR140	1690	13	164895338	164898801	gain	17784	58927444	194995520	backfat_at_last_rib	region1inRegion2	3463
CNVR141	1693	13	166109149	166114547	gain	17784	58927444	194995520	backfat_at_last_rib	region1inRegion2	5398
CNVR142	1695	13	167567440	167600641	gain	17784	58927444	194995520	backfat_at_last_rib	region1inRegion2	33201
CNVR143	1699	13	170765271	170819670	gain	17784	58927444	194995520	backfat_at_last_rib	region1inRegion2	54399
CNVR144	1702	13	171285806	171311772	gain	17784	58927444	194995520	backfat_at_last_rib	region1inRegion2	25966
CNVR138	1636	13	111201690	111225859	gain	1144	67299272	130538722	Body_weight_(birth)	region1inRegion2	24169
CNVR138	1636	13	111201690	111225859	gain	1145	67299272	130538722	Shoulder_meat_weight	region1inRegion2	24169
CNVR138	1636	13	111201690	111225859	gain	3087	83599317	194995520	Side_fat_thickness	region1inRegion2	24169
CNVR139	1656	13	131028348	131044332	gain	3087	83599317	194995520	Side_fat_thickness	region1inRegion2	15984
CNVR140	1690	13	164895338	164898801	gain	3087	83599317	194995520	Side_fat_thickness	region1inRegion2	3463
CNVR141	1693	13	166109149	166114547	gain	3087	83599317	194995520	Side_fat_thickness	region1inRegion2	5398
CNVR142	1695	13	167567440	167600641	gain	3087	83599317	194995520	Side_fat_thickness	region1inRegion2	33201
CNVR143	1699	13	170765271	170819670	gain	3087	83599317	194995520	Side_fat_thickness	region1inRegion2	54399
CNVR144	1702	13	171285806	171311772	gain	3087	83599317	194995520	Side_fat_thickness	region1inRegion2	25966
CNVR138	1636	13	111201690	111225859	gain	13298	83773665	176190166	Spleen_weight	region1inRegion2	24169
CNVR139	1656	13	131028348	131044332	gain	13298	83773665	176190166	Spleen_weight	region1inRegion2	15984
CNVR140	1690	13	164895338	164898801	gain	13298	83773665	176190166	Spleen_weight	region1inRegion2	3463
CNVR141	1693	13	166109149	166114547	gain	13298	83773665	176190166	Spleen_weight	region1inRegion2	5398
CNVR142	1695	13	167567440	167600641	gain	13298	83773665	176190166	Spleen_weight	region1inRegion2	33201
CNVR143	1699	13	170765271	170819670	gain	13298	83773665	176190166	Spleen_weight	region1inRegion2	54399
CNVR144	1702	13	171285806	171311772	gain	13298	83773665	176190166	Spleen_weight	region1inRegion2	25966
CNVR138	1636	13	111201690	111225859	gain	5507	89503549	192233281	Skin_percentage	region1inRegion2	24169
CNVR139	1656	13	131028348	131044332	gain	5507	89503549	192233281	Skin_percentage	region1inRegion2	15984
CNVR140	1690	13	164895338	164898801	gain	5507	89503549	192233281	Skin_percentage	region1inRegion2	3463
CNVR141	1693	13	166109149	166114547	gain	5507	89503549	192233281	Skin_percentage	region1inRegion2	5398
CNVR142	1695	13	167567440	167600641	gain	5507	89503549	192233281	Skin_percentage	region1inRegion2	33201
CNVR143	1699	13	170765271	170819670	gain	5507	89503549	192233281	Skin_percentage	region1inRegion2	54399
CNVR144	1702	13	171285806	171311772	gain	5507	89503549	192233281	Skin_percentage	region1inRegion2	25966
CNVR138	1636	13	111201690	111225859	gain	3968	89503549	194995520	Ham_fat_thickness	region1inRegion2	24169
CNVR139	1656	13	131028348	131044332	gain	3968	89503549	194995520	Ham_fat_thickness	region1inRegion2	15984
CNVR140	1690	13	164895338	164898801	gain	3968	89503549	194995520	Ham_fat_thickness	region1inRegion2	3463
CNVR141	1693	13	166109149	166114547	gain	3968	89503549	194995520	Ham_fat_thickness	region1inRegion2	5398
CNVR142	1695	13	167567440	167600641	gain	3968	89503549	194995520	Ham_fat_thickness	region1inRegion2	33201
CNVR143	1699	13	170765271	170819670	gain	3968	89503549	194995520	Ham_fat_thickness	region1inRegion2	54399
CNVR144	1702	13	171285806	171311772	gain	3968	89503549	194995520	Ham_fat_thickness	region1inRegion2	25966
CNVR138	1636	13	111201690	111225859	gain	3969	89503549	194995520	Lean_meat_percentage	region1inRegion2	24169
CNVR139	1656	13	131028348	131044332	gain	3969	89503549	194995520	Lean_meat_percentage	region1inRegion2	15984
CNVR140	1690	13	164895338	164898801	gain	3969	89503549	194995520	Lean_meat_percentage	region1inRegion2	3463
CNVR141	1693	13	166109149	166114547	gain	3969	89503549	194995520	Lean_meat_percentage	region1inRegion2	5398
CNVR142	1695	13	167567440	167600641	gain	3969	89503549	194995520	Lean_meat_percentage	region1inRegion2	33201
CNVR143	1699	13	170765271	170819670	gain	3969	89503549	194995520	Lean_meat_percentage	region1inRegion2	54399
CNVR144	1702	13	171285806	171311772	gain	3969	89503549	194995520	Lean_meat_percentage	region1inRegion2	25966
CNVR138	1636	13	111201690	111225859	gain	4004	89503549	194995520	pH_40_minutes_post_mortem_(loin)	region1inRegion2	24169
CNVR139	1656	13	131028348	131044332	gain	4004	89503549	194995520	pH_40_minutes_post_mortem_(loin)	region1inRegion2	15984
CNVR140	1690	13	164895338	164898801	gain	4004	89503549	194995520	pH_40_minutes_post_mortem_(loin)	region1inRegion2	3463
CNVR141	1693	13	166109149	166114547	gain	4004	89503549	194995520	pH_40_minutes_post_mortem_(loin)	region1inRegion2	5398
CNVR142	1695	13	167567440	167600641	gain	4004	89503549	194995520	pH_40_minutes_post_mortem_(loin)	region1inRegion2	33201
CNVR143	1699	13	170765271	170819670	gain	4004	89503549	194995520	pH_40_minutes_post_mortem_(loin)	region1inRegion2	54399
CNVR144	1702	13	171285806	171311772	gain	4004	89503549	194995520	pH_40_minutes_post_mortem_(loin)	region1inRegion2	25966
CNVR138	1636	13	111201690	111225859	gain	7655	107529939	112833627	Body_weight_(birth)	region1inRegion2	24169
CNVR139	1656	13	131028348	131044332	gain	4006	112682060	145027202	CIE-a*	region1inRegion2	15984
CNVR139	1656	13	131028348	131044332	gain	21318	130538722	172283983	rhinitis	region1inRegion2	15984
CNVR140	1690	13	164895338	164898801	gain	21318	130538722	172283983	rhinitis	region1inRegion2	3463
CNVR141	1693	13	166109149	166114547	gain	21318	130538722	172283983	rhinitis	region1inRegion2	5398
CNVR142	1695	13	167567440	167600641	gain	21318	130538722	172283983	rhinitis	region1inRegion2	33201
CNVR143	1699	13	170765271	170819670	gain	21318	130538722	172283983	rhinitis	region1inRegion2	54399
CNVR144	1702	13	171285806	171311772	gain	21318	130538722	172283983	rhinitis	region1inRegion2	25966
CNVR139	1656	13	131028348	131044332	gain	1142	130538722	192233281	Ham_weight	region1inRegion2	15984
CNVR140	1690	13	164895338	164898801	gain	1142	130538722	192233281	Ham_weight	region1inRegion2	3463
CNVR141	1693	13	166109149	166114547	gain	1142	130538722	192233281	Ham_weight	region1inRegion2	5398
CNVR142	1695	13	167567440	167600641	gain	1142	130538722	192233281	Ham_weight	region1inRegion2	33201
CNVR143	1699	13	170765271	170819670	gain	1142	130538722	192233281	Ham_weight	region1inRegion2	54399
CNVR144	1702	13	171285806	171311772	gain	1142	130538722	192233281	Ham_weight	region1inRegion2	25966
CNVR139	1656	13	131028348	131044332	gain	1146	130538722	192233281	Dressing_percentage	region1inRegion2	15984
CNVR140	1690	13	164895338	164898801	gain	1146	130538722	192233281	Dressing_percentage	region1inRegion2	3463
CNVR141	1693	13	166109149	166114547	gain	1146	130538722	192233281	Dressing_percentage	region1inRegion2	5398
CNVR142	1695	13	167567440	167600641	gain	1146	130538722	192233281	Dressing_percentage	region1inRegion2	33201
CNVR143	1699	13	170765271	170819670	gain	1146	130538722	192233281	Dressing_percentage	region1inRegion2	54399
CNVR144	1702	13	171285806	171311772	gain	1146	130538722	192233281	Dressing_percentage	region1inRegion2	25966
CNVR139	1656	13	131028348	131044332	gain	2881	130538722	194995520	Loin_and_ham_percentage_in_carcass	region1inRegion2	15984
CNVR140	1690	13	164895338	164898801	gain	2881	130538722	194995520	Loin_and_ham_percentage_in_carcass	region1inRegion2	3463
CNVR141	1693	13	166109149	166114547	gain	2881	130538722	194995520	Loin_and_ham_percentage_in_carcass	region1inRegion2	5398
CNVR142	1695	13	167567440	167600641	gain	2881	130538722	194995520	Loin_and_ham_percentage_in_carcass	region1inRegion2	33201
CNVR143	1699	13	170765271	170819670	gain	2881	130538722	194995520	Loin_and_ham_percentage_in_carcass	region1inRegion2	54399
CNVR144	1702	13	171285806	171311772	gain	2881	130538722	194995520	Loin_and_ham_percentage_in_carcass	region1inRegion2	25966
CNVR139	1656	13	131028348	131044332	gain	2882	130538722	194995520	Loin_weight	region1inRegion2	15984
CNVR140	1690	13	164895338	164898801	gain	2882	130538722	194995520	Loin_weight	region1inRegion2	3463
CNVR141	1693	13	166109149	166114547	gain	2882	130538722	194995520	Loin_weight	region1inRegion2	5398
CNVR142	1695	13	167567440	167600641	gain	2882	130538722	194995520	Loin_weight	region1inRegion2	33201
CNVR143	1699	13	170765271	170819670	gain	2882	130538722	194995520	Loin_weight	region1inRegion2	54399
CNVR144	1702	13	171285806	171311772	gain	2882	130538722	194995520	Loin_weight	region1inRegion2	25966
CNVR139	1656	13	131028348	131044332	gain	3074	130538722	194995520	CIE-a*	region1inRegion2	15984
CNVR140	1690	13	164895338	164898801	gain	3074	130538722	194995520	CIE-a*	region1inRegion2	3463
CNVR141	1693	13	166109149	166114547	gain	3074	130538722	194995520	CIE-a*	region1inRegion2	5398
CNVR142	1695	13	167567440	167600641	gain	3074	130538722	194995520	CIE-a*	region1inRegion2	33201
CNVR143	1699	13	170765271	170819670	gain	3074	130538722	194995520	CIE-a*	region1inRegion2	54399
CNVR144	1702	13	171285806	171311772	gain	3074	130538722	194995520	CIE-a*	region1inRegion2	25966
CNVR139	1656	13	131028348	131044332	gain	3077	130538722	194995520	Loin_meat_weight	region1inRegion2	15984
CNVR140	1690	13	164895338	164898801	gain	3077	130538722	194995520	Loin_meat_weight	region1inRegion2	3463
CNVR141	1693	13	166109149	166114547	gain	3077	130538722	194995520	Loin_meat_weight	region1inRegion2	5398
CNVR142	1695	13	167567440	167600641	gain	3077	130538722	194995520	Loin_meat_weight	region1inRegion2	33201
CNVR143	1699	13	170765271	170819670	gain	3077	130538722	194995520	Loin_meat_weight	region1inRegion2	54399
CNVR144	1702	13	171285806	171311772	gain	3077	130538722	194995520	Loin_meat_weight	region1inRegion2	25966
CNVR139	1656	13	131028348	131044332	gain	3672	130538722	194995520	backfat_at_last_rib	region1inRegion2	15984
CNVR140	1690	13	164895338	164898801	gain	3672	130538722	194995520	backfat_at_last_rib	region1inRegion2	3463
CNVR141	1693	13	166109149	166114547	gain	3672	130538722	194995520	backfat_at_last_rib	region1inRegion2	5398
CNVR142	1695	13	167567440	167600641	gain	3672	130538722	194995520	backfat_at_last_rib	region1inRegion2	33201
CNVR143	1699	13	170765271	170819670	gain	3672	130538722	194995520	backfat_at_last_rib	region1inRegion2	54399
CNVR144	1702	13	171285806	171311772	gain	3672	130538722	194995520	backfat_at_last_rib	region1inRegion2	25966
CNVR139	1656	13	131028348	131044332	gain	3689	130538722	194995520	PH_for_Longissmus_dorsi	region1inRegion2	15984
CNVR140	1690	13	164895338	164898801	gain	3689	130538722	194995520	PH_for_Longissmus_dorsi	region1inRegion2	3463
CNVR141	1693	13	166109149	166114547	gain	3689	130538722	194995520	PH_for_Longissmus_dorsi	region1inRegion2	5398
CNVR142	1695	13	167567440	167600641	gain	3689	130538722	194995520	PH_for_Longissmus_dorsi	region1inRegion2	33201
CNVR143	1699	13	170765271	170819670	gain	3689	130538722	194995520	PH_for_Longissmus_dorsi	region1inRegion2	54399
CNVR144	1702	13	171285806	171311772	gain	3689	130538722	194995520	PH_for_Longissmus_dorsi	region1inRegion2	25966
CNVR139	1656	13	131028348	131044332	gain	3690	130538722	194995520	Carcass_length	region1inRegion2	15984
CNVR140	1690	13	164895338	164898801	gain	3690	130538722	194995520	Carcass_length	region1inRegion2	3463
CNVR141	1693	13	166109149	166114547	gain	3690	130538722	194995520	Carcass_length	region1inRegion2	5398
CNVR142	1695	13	167567440	167600641	gain	3690	130538722	194995520	Carcass_length	region1inRegion2	33201
CNVR143	1699	13	170765271	170819670	gain	3690	130538722	194995520	Carcass_length	region1inRegion2	54399
CNVR144	1702	13	171285806	171311772	gain	3690	130538722	194995520	Carcass_length	region1inRegion2	25966
CNVR139	1656	13	131028348	131044332	gain	3691	130538722	194995520	pH_for_Semimembranosus	region1inRegion2	15984
CNVR140	1690	13	164895338	164898801	gain	3691	130538722	194995520	pH_for_Semimembranosus	region1inRegion2	3463
CNVR141	1693	13	166109149	166114547	gain	3691	130538722	194995520	pH_for_Semimembranosus	region1inRegion2	5398
CNVR142	1695	13	167567440	167600641	gain	3691	130538722	194995520	pH_for_Semimembranosus	region1inRegion2	33201
CNVR143	1699	13	170765271	170819670	gain	3691	130538722	194995520	pH_for_Semimembranosus	region1inRegion2	54399
CNVR144	1702	13	171285806	171311772	gain	3691	130538722	194995520	pH_for_Semimembranosus	region1inRegion2	25966

CNVR139	1656	13	131028348	131044332	gain	3692	130538722	194995520	PH_for_Longissmus_dorsi	region1inRegion2	15984
CNVR140	1690	13	164895338	164898801	gain	3692	130538722	194995520	PH_for_Longissmus_dorsi	region1inRegion2	3463
CNVR141	1693	13	166109149	166114547	gain	3692	130538722	194995520	PH_for_Longissmus_dorsi	region1inRegion2	5398
CNVR142	1695	13	167567440	167600641	gain	3692	130538722	194995520	PH_for_Longissmus_dorsi	region1inRegion2	33201
CNVR143	1699	13	170765271	170819670	gain	3692	130538722	194995520	PH_for_Longissmus_dorsi	region1inRegion2	54399
CNVR144	1702	13	171285806	171311772	gain	3692	130538722	194995520	PH_for_Longissmus_dorsi	region1inRegion2	25966
CNVR139	1656	13	131028348	131044332	gain	4163	130538722	194995520	backfat_at_P2_position	region1inRegion2	15984
CNVR140	1690	13	164895338	164898801	gain	4163	130538722	194995520	backfat_at_P2_position	region1inRegion2	3463
CNVR141	1693	13	166109149	166114547	gain	4163	130538722	194995520	backfat_at_P2_position	region1inRegion2	5398
CNVR142	1695	13	167567440	167600641	gain	4163	130538722	194995520	backfat_at_P2_position	region1inRegion2	33201
CNVR143	1699	13	170765271	170819670	gain	4163	130538722	194995520	backfat_at_P2_position	region1inRegion2	54399
CNVR144	1702	13	171285806	171311772	gain	4163	130538722	194995520	backfat_at_P2_position	region1inRegion2	25966
CNVR139	1656	13	131028348	131044332	gain	4164	130538722	194995520	Carcass_length	region1inRegion2	15984
CNVR140	1690	13	164895338	164898801	gain	4164	130538722	194995520	Carcass_length	region1inRegion2	3463
CNVR141	1693	13	166109149	166114547	gain	4164	130538722	194995520	Carcass_length	region1inRegion2	5398
CNVR142	1695	13	167567440	167600641	gain	4164	130538722	194995520	Carcass_length	region1inRegion2	33201
CNVR143	1699	13	170765271	170819670	gain	4164	130538722	194995520	Carcass_length	region1inRegion2	54399
CNVR144	1702	13	171285806	171311772	gain	4164	130538722	194995520	Carcass_length	region1inRegion2	25966
CNVR139	1656	13	131028348	131044332	gain	4192	130538722	194995520	Conductivity_45_minutes_post-mortem	region1inRegion2	15984
CNVR140	1690	13	164895338	164898801	gain	4192	130538722	194995520	Conductivity_45_minutes_post-mortem	region1inRegion2	3463
CNVR141	1693	13	166109149	166114547	gain	4192	130538722	194995520	Conductivity_45_minutes_post-mortem	region1inRegion2	5398
CNVR142	1695	13	167567440	167600641	gain	4192	130538722	194995520	Conductivity_45_minutes_post-mortem	region1inRegion2	33201
CNVR143	1699	13	170765271	170819670	gain	4192	130538722	194995520	Conductivity_45_minutes_post-mortem	region1inRegion2	54399
CNVR144	1702	13	171285806	171311772	gain	4192	130538722	194995520	Conductivity_45_minutes_post-mortem	region1inRegion2	25966
CNVR139	1656	13	131028348	131044332	gain	4225	130538722	194995520	indole,_laboratory	region1inRegion2	15984
CNVR140	1690	13	164895338	164898801	gain	4225	130538722	194995520	indole,_laboratory	region1inRegion2	3463
CNVR141	1693	13	166109149	166114547	gain	4225	130538722	194995520	indole,_laboratory	region1inRegion2	5398
CNVR142	1695	13	167567440	167600641	gain	4225	130538722	194995520	indole,_laboratory	region1inRegion2	33201
CNVR143	1699	13	170765271	170819670	gain	4225	130538722	194995520	indole,_laboratory	region1inRegion2	54399
CNVR144	1702	13	171285806	171311772	gain	4225	130538722	194995520	indole,_laboratory	region1inRegion2	25966
CNVR139	1656	13	131028348	131044332	gain	8917	130538722	194995520	Front_leg_conformation	region1inRegion2	15984
CNVR140	1690	13	164895338	164898801	gain	8917	130538722	194995520	Front_leg_conformation	region1inRegion2	3463
CNVR141	1693	13	166109149	166114547	gain	8917	130538722	194995520	Front_leg_conformation	region1inRegion2	5398
CNVR142	1695	13	167567440	167600641	gain	8917	130538722	194995520	Front_leg_conformation	region1inRegion2	33201
CNVR143	1699	13	170765271	170819670	gain	8917	130538722	194995520	Front_leg_conformation	region1inRegion2	54399
CNVR144	1702	13	171285806	171311772	gain	8917	130538722	194995520	Front_leg_conformation	region1inRegion2	25966
CNVR139	1656	13	131028348	131044332	gain	8918	130538722	194995520	Front_feet_conformation	region1inRegion2	15984
CNVR140	1690	13	164895338	164898801	gain	8918	130538722	194995520	Front_feet_conformation	region1inRegion2	3463
CNVR141	1693	13	166109149	166114547	gain	8918	130538722	194995520	Front_feet_conformation	region1inRegion2	5398
CNVR142	1695	13	167567440	167600641	gain	8918	130538722	194995520	Front_feet_conformation	region1inRegion2	33201
CNVR143	1699	13	170765271	170819670	gain	8918	130538722	194995520	Front_feet_conformation	region1inRegion2	54399
CNVR144	1702	13	171285806	171311772	gain	8918	130538722	194995520	Front_feet_conformation	region1inRegion2	25966
CNVR139	1656	13	131028348	131044332	gain	8921	130538722	194995520	Gait_score_(hind)	region1inRegion2	15984
CNVR140	1690	13	164895338	164898801	gain	8921	130538722	194995520	Gait_score_(hind)	region1inRegion2	3463
CNVR141	1693	13	166109149	166114547	gain	8921	130538722	194995520	Gait_score_(hind)	region1inRegion2	5398
CNVR142	1695	13	167567440	167600641	gain	8921	130538722	194995520	Gait_score_(hind)	region1inRegion2	33201
CNVR143	1699	13	170765271	170819670	gain	8921	130538722	194995520	Gait_score_(hind)	region1inRegion2	54399
CNVR144	1702	13	171285806	171311772	gain	8921	130538722	194995520	Gait_score_(hind)	region1inRegion2	25966
CNVR139	1656	13	131028348	131044332	gain	8922	130538722	194995520	Physis_score	region1inRegion2	15984
CNVR140	1690	13	164895338	164898801	gain	8922	130538722	194995520	Physis_score	region1inRegion2	3463
CNVR141	1693	13	166109149	166114547	gain	8922	130538722	194995520	Physis_score	region1inRegion2	5398
CNVR142	1695	13	167567440	167600641	gain	8922	130538722	194995520	Physis_score	region1inRegion2	33201
CNVR143	1699	13	170765271	170819670	gain	8922	130538722	194995520	Physis_score	region1inRegion2	54399
CNVR144	1702	13	171285806	171311772	gain	8922	130538722	194995520	Physis_score	region1inRegion2	25966
CNVR139	1656	13	131028348	131044332	gain	107263	130598203	131352656	Mean_corpuscular_hemoglobin_content	region1inRegion2	15984
CNVR139	1656	13	131028348	131044332	gain	107287	130598203	131352656	Mean_corpuscular_volume	region1inRegion2	15984
CNVR139	1656	13	131028348	131044332	gain	107328	130598203	131352656	Red_cell_distribution_width	region1inRegion2	15984
CNVR140	1690	13	164895338	164898801	gain	2883	141415709	203809783	Protein_accretion_rate	region1inRegion2	3463
CNVR141	1693	13	166109149	166114547	gain	2883	141415709	203809783	Protein_accretion_rate	region1inRegion2	5398
CNVR142	1695	13	167567440	167600641	gain	2883	141415709	203809783	Protein_accretion_rate	region1inRegion2	33201
CNVR143	1699	13	170765271	170819670	gain	2883	141415709	203809783	Protein_accretion_rate	region1inRegion2	54399
CNVR144	1702	13	171285806	171311772	gain	2883	141415709	203809783	Protein_accretion_rate	region1inRegion2	25966
CNVR140	1690	13	164895338	164898801	gain	2884	141415709	203809783	Feed_conversion_ratio	region1inRegion2	3463
CNVR141	1693	13	166109149	166114547	gain	2884	141415709	203809783	Feed_conversion_ratio	region1inRegion2	5398
CNVR142	1695	13	167567440	167600641	gain	2884	141415709	203809783	Feed_conversion_ratio	region1inRegion2	33201
CNVR143	1699	13	170765271	170819670	gain	2884	141415709	203809783	Feed_conversion_ratio	region1inRegion2	54399
CNVR144	1702	13	171285806	171311772	gain	2884	141415709	203809783	Feed_conversion_ratio	region1inRegion2	25966
CNVR140	1690	13	164895338	164898801	gain	17744	143630911	195062883	Stearic_acid_content	region1inRegion2	3463
CNVR141	1693	13	166109149	166114547	gain	17744	143630911	195062883	Stearic_acid_content	region1inRegion2	5398
CNVR142	1695	13	167567440	167600641	gain	17744	143630911	195062883	Stearic_acid_content	region1inRegion2	33201
CNVR143	1699	13	170765271	170819670	gain	17744	143630911	195062883	Stearic_acid_content	region1inRegion2	54399
CNVR144	1702	13	171285806	171311772	gain	17744	143630911	195062883	Stearic_acid_content	region1inRegion2	25966
CNVR140	1690	13	164895338	164898801	gain	7708	143630911	195062883	HDL/LDL_ratio	region1inRegion2	3463
CNVR141	1693	13	166109149	166114547	gain	7708	143630911	195062883	HDL/LDL_ratio	region1inRegion2	5398
CNVR142	1695	13	167567440	167600641	gain	7708	143630911	195062883	HDL/LDL_ratio	region1inRegion2	33201
CNVR143	1699	13	170765271	170819670	gain	7708	143630911	195062883	HDL/LDL_ratio	region1inRegion2	54399
CNVR144	1702	13	171285806	171311772	gain	7708	143630911	195062883	HDL/LDL_ratio	region1inRegion2	25966
CNVR140	1690	13	164895338	164898801	gain	235	159374767	194995520	Meat_color-a	region1inRegion2	3463
CNVR141	1693	13	166109149	166114547	gain	235	159374767	194995520	Meat_color-a	region1inRegion2	5398
CNVR142	1695	13	167567440	167600641	gain	235	159374767	194995520	Meat_color-a	region1inRegion2	33201
CNVR143	1699	13	170765271	170819670	gain	235	159374767	194995520	Meat_color-a	region1inRegion2	54399
CNVR144	1702	13	171285806	171311772	gain	235	159374767	194995520	Meat_color-a	region1inRegion2	25966
CNVR140	1690	13	164895338	164898801	gain	5391	159374767	206658205	Hemoglobin	region1inRegion2	3463
CNVR141	1693	13	166109149	166114547	gain	5391	159374767	206658205	Hemoglobin	region1inRegion2	5398
CNVR142	1695	13	167567440	167600641	gain	5391	159374767	206658205	Hemoglobin	region1inRegion2	33201
CNVR143	1699	13	170765271	170819670	gain	5391	159374767	206658205	Hemoglobin	region1inRegion2	54399
CNVR144	1702	13	171285806	171311772	gain	5391	159374767	206658205	Hemoglobin	region1inRegion2	25966
CNVR140	1690	13	164895338	164898801	gain	5396	159374767	206658205	Hemoglobin	region1inRegion2	3463
CNVR141	1693	13	166109149	166114547	gain	5396	159374767	206658205	Hemoglobin	region1inRegion2	5398
CNVR142	1695	13	167567440	167600641	gain	5396	159374767	206658205	Hemoglobin	region1inRegion2	33201
CNVR143	1699	13	170765271	170819670	gain	5396	159374767	206658205	Hemoglobin	region1inRegion2	54399
CNVR144	1702	13	171285806	171311772	gain	5396	159374767	206658205	Hemoglobin	region1inRegion2	25966
CNVR140	1690	13	164895338	164898801	gain	5402	159374767	206658205	Red_blood_cell_count	region1inRegion2	3463
CNVR141	1693	13	166109149	166114547	gain	5402	159374767	206658205	Red_blood_cell_count	region1inRegion2	5398
CNVR142	1695	13	167567440	167600641	gain	5402	159374767	206658205	Red_blood_cell_count	region1inRegion2	33201
CNVR143	1699	13	170765271	170819670	gain	5402	159374767	206658205	Red_blood_cell_count	region1inRegion2	54399
CNVR144	1702	13	171285806	171311772	gain	5402	159374767	206658205	Red_blood_cell_count	region1inRegion2	25966
CNVR140	1690	13	164895338	164898801	gain	5414	159374767	206658205	hematocrit	region1inRegion2	3463
CNVR141	1693	13	166109149	166114547	gain	5414	159374767	206658205	hematocrit	region1inRegion2	5398
CNVR142	1695	13	167567440	167600641	gain	5414	159374767	206658205	hematocrit	region1inRegion2	33201
CNVR143	1699	13	170765271	170819670	gain	5414	159374767	206658205	hematocrit	region1inRegion2	54399
CNVR144	1702	13	171285806	171311772	gain	5414	159374767	206658205	hematocrit	region1inRegion2	25966
CNVR140	1690	13	164895338	164898801	gain	5416	159374767	206658205	hematocrit	region1inRegion2	3463
CNVR141	1693	13	166109149	166114547	gain	5416	159374767	206658205	hematocrit	region1inRegion2	5398
CNVR142	1695	13	167567440	167600641	gain	5416	159374767	206658205	hematocrit	region1inRegion2	33201
CNVR143	1699	13	170765271	170819670	gain	5416	159374767	206658205	hematocrit	region1inRegion2	54399
CNVR144	1702	13	171285806	171311772	gain	5416	159374767	206658205	hematocrit	region1inRegion2	25966
CNVR140	1690	13	164895338	164898801	gain	5428	159374767	206658205	Mean_corpuscular_hemoglobin_content	region1inRegion2	3463
CNVR141	1693	13	166109149	166114547	gain	5428	159374767	206658205	Mean_corpuscular_hemoglobin_content	region1inRegion2	5398
CNVR142	1695	13	167567440	167600641	gain	5428	159374767	206658205	Mean_corpuscular_hemoglobin_content	region1inRegion2	33201
CNVR143	1699	13	170765271	170819670	gain	5428	159374767	206658205	Mean_corpuscular_hemoglobin_content	region1inRegion2	54399
CNVR144	1702	13	171285806	171311772	gain	5428	159374767	206658205	Mean_corpuscular_hemoglobin_content	region1inRegion2	25966
CNVR140	1690	13	164895338	164898801	gain	6385	159374767	206658205	Sodium_level	region1inRegion2	3463
CNVR141	1693	13	166109149	166114547	gain	6385	159374767	206658205	Sodium_level	region1inRegion2	5398
CNVR142	1695	13	167567440	167600641	gain	6385	159374767	206658205	Sodium_level	region1inRegion2	33201
CNVR143	1699	13	170765271	170819670	gain	6385	159374767	206658205	Sodium_level	region1inRegion2	54399
CNVR144	1702	13	171285806	171311772	gain	6385	159374767	206658205	Sodium_level	region1inRegion2	25966
CNVR145	1777	14	2401741	2423505	gain	32128	1217660	12516762	Linoleic_acid_content	region1inRegion2	21764
CNVR146	1781	14	7794851	7822418	gain	32128	1217660	12516762	Linoleic_acid_content	region1inRegion2	27567
CNVR147	1782	14	8039487	8116098	gain	32128	1217660	12516762	Linoleic_acid_content	region1inRegion2	76611
CNVR146	1781	14	7794851	7822418	gain	17854	6898350	9756931	CD4-negative,_CD8-positive_leukocyte_percentage	region1inRegion2	27567
CNVR147	1782	14	8039487	8116098	gain	17854	6898350	9756931	CD4-negative,_CD8-positive_leukocyte_percentage	region1inRegion2	76611

CNVR146	1781	14	7794851	7822418	gain	3079	6898350	16066507	Marbling	region1inRegion2	27567
CNVR147	1782	14	8039487	8116098	gain	3079	6898350	16066507	Marbling	region1inRegion2	76611
CNVR146	1781	14	7794851	7822418	gain	4232	6898350	16066507	indole,_laboratory	region1inRegion2	27567
CNVR147	1782	14	8039487	8116098	gain	4232	6898350	16066507	indole,_laboratory	region1inRegion2	76611
CNVR146	1781	14	7794851	7822418	gain	4234	6898350	16066507	androstenone,_sensory_panel	region1inRegion2	27567
CNVR147	1782	14	8039487	8116098	gain	4234	6898350	16066507	androstenone,_sensory_panel	region1inRegion2	76611
CNVR146	1781	14	7794851	7822418	gain	8924	6898350	16066507	Front_feet_conformation	region1inRegion2	27567
CNVR147	1782	14	8039487	8116098	gain	8924	6898350	16066507	Front_feet_conformation	region1inRegion2	76611
CNVR146	1781	14	7794851	7822418	gain	8926	6898350	16066507	Hind_feet_conformation	region1inRegion2	27567
CNVR147	1782	14	8039487	8116098	gain	8926	6898350	16066507	Hind_feet_conformation	region1inRegion2	76611
CNVR146	1781	14	7794851	7822418	gain	16841	6898350	27531879	backfat_at_last_rib	region1inRegion2	27567
CNVR147	1782	14	8039487	8116098	gain	16841	6898350	27531879	backfat_at_last_rib	region1inRegion2	76611
CNVR148	1792	14	21068045	21091201	gain	16841	6898350	27531879	backfat_at_last_rib	region1inRegion2	23156
CNVR146	1781	14	7794851	7822418	gain	236	6898350	41217524	Meat_color-a	region1inRegion2	27567
CNVR147	1782	14	8039487	8116098	gain	236	6898350	41217524	Meat_color-a	region1inRegion2	76611
CNVR148	1792	14	21068045	21091201	gain	236	6898350	41217524	Meat_color-a	region1inRegion2	23156
CNVR146	1781	14	7794851	7822418	gain	354	6898350	41217524	Average_backfat_thickness	region1inRegion2	27567
CNVR147	1782	14	8039487	8116098	gain	354	6898350	41217524	Average_backfat_thickness	region1inRegion2	76611
CNVR148	1792	14	21068045	21091201	gain	354	6898350	41217524	Average_backfat_thickness	region1inRegion2	23156
CNVR146	1781	14	7794851	7822418	gain	3825	6898350	41487784	Meat_color-L	region1inRegion2	27567
CNVR147	1782	14	8039487	8116098	gain	3825	6898350	41487784	Meat_color-L	region1inRegion2	76611
CNVR148	1792	14	21068045	21091201	gain	3825	6898350	41487784	Meat_color-L	region1inRegion2	23156
CNVR146	1781	14	7794851	7822418	gain	15870	6898350	66305680	Stearic_acid_content	region1inRegion2	27567
CNVR147	1782	14	8039487	8116098	gain	15870	6898350	66305680	Stearic_acid_content	region1inRegion2	76611
CNVR148	1792	14	21068045	21091201	gain	15870	6898350	66305680	Stearic_acid_content	region1inRegion2	23156
CNVR149	1816	14	56382005	56403580	loss	15870	6898350	66305680	Stearic_acid_content	region1inRegion2	21575
CNVR150	1820	14	65589945	65600023	loss	15870	6898350	66305680	Stearic_acid_content	region1inRegion2	10078
CNVR146	1781	14	7794851	7822418	gain	15871	6898350	66305680	Linoleic_acid_content	region1inRegion2	27567
CNVR147	1782	14	8039487	8116098	gain	15871	6898350	66305680	Linoleic_acid_content	region1inRegion2	76611
CNVR148	1792	14	21068045	21091201	gain	15871	6898350	66305680	Linoleic_acid_content	region1inRegion2	23156
CNVR149	1816	14	56382005	56403580	loss	15871	6898350	66305680	Linoleic_acid_content	region1inRegion2	21575
CNVR150	1820	14	65589945	65600023	loss	15871	6898350	66305680	Linoleic_acid_content	region1inRegion2	10078
CNVR146	1781	14	7794851	7822418	gain	17643	6898350	98122568	Osteochondrosis_score	region1inRegion2	27567
CNVR147	1782	14	8039487	8116098	gain	17643	6898350	98122568	Osteochondrosis_score	region1inRegion2	76611
CNVR148	1792	14	21068045	21091201	gain	17643	6898350	98122568	Osteochondrosis_score	region1inRegion2	23156
CNVR149	1816	14	56382005	56403580	loss	17643	6898350	98122568	Osteochondrosis_score	region1inRegion2	21575
CNVR150	1820	14	65589945	65600023	loss	17643	6898350	98122568	Osteochondrosis_score	region1inRegion2	10078
CNVR151	1829	14	74107606	74126308	loss	17643	6898350	98122568	Osteochondrosis_score	region1inRegion2	18702
CNVR152	1838	14	81377028	81553876	gain	17643	6898350	98122568	Osteochondrosis_score	region1inRegion2	176848
CNVR153	1849	14	94594494	94629904	gain	17643	6898350	98122568	Osteochondrosis_score	region1inRegion2	35410
CNVR146	1781	14	7794851	7822418	gain	2822	6898350	132053949	Percentage_type_I_fibers	region1inRegion2	27567
CNVR147	1782	14	8039487	8116098	gain	2822	6898350	132053949	Percentage_type_I_fibers	region1inRegion2	76611
CNVR148	1792	14	21068045	21091201	gain	2822	6898350	132053949	Percentage_type_I_fibers	region1inRegion2	23156
CNVR149	1816	14	56382005	56403580	loss	2822	6898350	132053949	Percentage_type_I_fibers	region1inRegion2	21575
CNVR150	1820	14	65589945	65600023	loss	2822	6898350	132053949	Percentage_type_I_fibers	region1inRegion2	10078
CNVR151	1829	14	74107606	74126308	loss	2822	6898350	132053949	Percentage_type_I_fibers	region1inRegion2	18702
CNVR152	1838	14	81377028	81553876	gain	2822	6898350	132053949	Percentage_type_I_fibers	region1inRegion2	176848
CNVR153	1849	14	94594494	94629904	gain	2822	6898350	132053949	Percentage_type_I_fibers	region1inRegion2	35410
CNVR154	1873	14	120810562	120829324	gain	2822	6898350	132053949	Percentage_type_I_fibers	region1inRegion2	18762
CNVR155	1880	14	127501895	127526173	gain	2822	6898350	132053949	Percentage_type_I_fibers	region1inRegion2	24278
CNVR146	1781	14	7794851	7822418	gain	2823	6898350	132053949	Percentage_type_IIb_fibers	region1inRegion2	27567
CNVR147	1782	14	8039487	8116098	gain	2823	6898350	132053949	Percentage_type_IIb_fibers	region1inRegion2	76611
CNVR148	1792	14	21068045	21091201	gain	2823	6898350	132053949	Percentage_type_IIb_fibers	region1inRegion2	23156
CNVR149	1816	14	56382005	56403580	loss	2823	6898350	132053949	Percentage_type_IIb_fibers	region1inRegion2	21575
CNVR150	1820	14	65589945	65600023	loss	2823	6898350	132053949	Percentage_type_IIb_fibers	region1inRegion2	10078
CNVR151	1829	14	74107606	74126308	loss	2823	6898350	132053949	Percentage_type_IIb_fibers	region1inRegion2	18702
CNVR152	1838	14	81377028	81553876	gain	2823	6898350	132053949	Percentage_type_IIb_fibers	region1inRegion2	176848
CNVR153	1849	14	94594494	94629904	gain	2823	6898350	132053949	Percentage_type_IIb_fibers	region1inRegion2	35410
CNVR154	1873	14	120810562	120829324	gain	2823	6898350	132053949	Percentage_type_IIb_fibers	region1inRegion2	18762
CNVR155	1880	14	127501895	127526173	gain	2823	6898350	132053949	Percentage_type_IIb_fibers	region1inRegion2	24278
CNVR146	1781	14	7794851	7822418	gain	2824	6898350	132053949	Percentage_type_I_fibers	region1inRegion2	27567
CNVR147	1782	14	8039487	8116098	gain	2824	6898350	132053949	Percentage_type_I_fibers	region1inRegion2	76611
CNVR148	1792	14	21068045	21091201	gain	2824	6898350	132053949	Percentage_type_I_fibers	region1inRegion2	23156
CNVR149	1816	14	56382005	56403580	loss	2824	6898350	132053949	Percentage_type_I_fibers	region1inRegion2	21575
CNVR150	1820	14	65589945	65600023	loss	2824	6898350	132053949	Percentage_type_I_fibers	region1inRegion2	10078
CNVR151	1829	14	74107606	74126308	loss	2824	6898350	132053949	Percentage_type_I_fibers	region1inRegion2	18702
CNVR152	1838	14	81377028	81553876	gain	2824	6898350	132053949	Percentage_type_I_fibers	region1inRegion2	176848
CNVR153	1849	14	94594494	94629904	gain	2824	6898350	132053949	Percentage_type_I_fibers	region1inRegion2	35410
CNVR154	1873	14	120810562	120829324	gain	2824	6898350	132053949	Percentage_type_I_fibers	region1inRegion2	18762
CNVR155	1880	14	127501895	127526173	gain	2824	6898350	132053949	Percentage_type_I_fibers	region1inRegion2	24278
CNVR146	1781	14	7794851	7822418	gain	5722	6898350	132053949	Daily_feed_intake	region1inRegion2	27567
CNVR147	1782	14	8039487	8116098	gain	5722	6898350	132053949	Daily_feed_intake	region1inRegion2	76611
CNVR148	1792	14	21068045	21091201	gain	5722	6898350	132053949	Daily_feed_intake	region1inRegion2	23156
CNVR149	1816	14	56382005	56403580	loss	5722	6898350	132053949	Daily_feed_intake	region1inRegion2	21575
CNVR150	1820	14	65589945	65600023	loss	5722	6898350	132053949	Daily_feed_intake	region1inRegion2	10078
CNVR151	1829	14	74107606	74126308	loss	5722	6898350	132053949	Daily_feed_intake	region1inRegion2	18702
CNVR152	1838	14	81377028	81553876	gain	5722	6898350	132053949	Daily_feed_intake	region1inRegion2	176848
CNVR153	1849	14	94594494	94629904	gain	5722	6898350	132053949	Daily_feed_intake	region1inRegion2	35410
CNVR154	1873	14	120810562	120829324	gain	5722	6898350	132053949	Daily_feed_intake	region1inRegion2	18762
CNVR155	1880	14	127501895	127526173	gain	5722	6898350	132053949	Daily_feed_intake	region1inRegion2	24278
CNVR146	1781	14	7794851	7822418	gain	5991	6898350	132053949	backfat_above_muscle_dorsi	region1inRegion2	27567
CNVR147	1782	14	8039487	8116098	gain	5991	6898350	132053949	backfat_above_muscle_dorsi	region1inRegion2	76611
CNVR148	1792	14	21068045	21091201	gain	5991	6898350	132053949	backfat_above_muscle_dorsi	region1inRegion2	23156
CNVR149	1816	14	56382005	56403580	loss	5991	6898350	132053949	backfat_above_muscle_dorsi	region1inRegion2	21575
CNVR150	1820	14	65589945	65600023	loss	5991	6898350	132053949	backfat_above_muscle_dorsi	region1inRegion2	10078
CNVR151	1829	14	74107606	74126308	loss	5991	6898350	132053949	backfat_above_muscle_dorsi	region1inRegion2	18702
CNVR152	1838	14	81377028	81553876	gain	5991	6898350	132053949	backfat_above_muscle_dorsi	region1inRegion2	176848
CNVR153	1849	14	94594494	94629904	gain	5991	6898350	132053949	backfat_above_muscle_dorsi	region1inRegion2	35410
CNVR154	1873	14	120810562	120829324	gain	5991	6898350	132053949	backfat_above_muscle_dorsi	region1inRegion2	18762
CNVR155	1880	14	127501895	127526173	gain	5991	6898350	132053949	backfat_above_muscle_dorsi	region1inRegion2	24278
CNVR146	1781	14	7794851	7822418	gain	9630	6898350	132053949	C3c_concentration	region1inRegion2	27567
CNVR147	1782	14	8039487	8116098	gain	9630	6898350	132053949	C3c_concentration	region1inRegion2	76611
CNVR148	1792	14	21068045	21091201	gain	9630	6898350	132053949	C3c_concentration	region1inRegion2	23156
CNVR149	1816	14	56382005	56403580	loss	9630	6898350	132053949	C3c_concentration	region1inRegion2	21575
CNVR150	1820	14	65589945	65600023	loss	9630	6898350	132053949	C3c_concentration	region1inRegion2	10078
CNVR151	1829	14	74107606	74126308	loss	9630	6898350	132053949	C3c_concentration	region1inRegion2	18702
CNVR152	1838	14	81377028	81553876	gain	9630	6898350	132053949	C3c_concentration	region1inRegion2	176848
CNVR153	1849	14	94594494	94629904	gain	9630	6898350	132053949	C3c_concentration	region1inRegion2	35410
CNVR154	1873	14	120810562	120829324	gain	9630	6898350	132053949	C3c_concentration	region1inRegion2	18762
CNVR155	1880	14	127501895	127526173	gain	9630	6898350	132053949	C3c_concentration	region1inRegion2	24278
CNVR146	1781	14	7794851	7822418	gain	9653	6898350	132053949	Haptoglobin_concentration	region1inRegion2	27567
CNVR147	1782	14	8039487	8116098	gain	9653	6898350	132053949	Haptoglobin_concentration	region1inRegion2	76611
CNVR148	1792	14	21068045	21091201	gain	9653	6898350	132053949	Haptoglobin_concentration	region1inRegion2	23156
CNVR149	1816	14	56382005	56403580	loss	9653	6898350	132053949	Haptoglobin_concentration	region1inRegion2	21575
CNVR150	1820	14	65589945	65600023	loss	9653	6898350	132053949	Haptoglobin_concentration	region1inRegion2	10078
CNVR151	1829	14	74107606	74126308	loss	9653	6898350	132053949	Haptoglobin_concentration	region1inRegion2	18702
CNVR152	1838	14	81377028	81553876	gain	9653	6898350	132053949	Haptoglobin_concentration	region1inRegion2	176848
CNVR153	1849	14	94594494	94629904	gain	9653	6898350	132053949	Haptoglobin_concentration	region1inRegion2	35410
CNVR154	1873	14	120810562	120829324	gain	9653	6898350	132053949	Haptoglobin_concentration	region1inRegion2	18762
CNVR155	1880	14	127501895	127526173	gain	9653	6898350	132053949	Haptoglobin_concentration	region1inRegion2	24278
CNVR146	1781	14	7794851	7822418	gain	38110	6898350	149890749	CIE-L*	region1inRegion2	27567
CNVR147	1782	14	8039487	8116098	gain	38110	6898350	149890749	CIE-L*	region1inRegion2	76611
CNVR148	1792	14	21068045	21091201	gain	38110	6898350	149890749	CIE-L*	region1inRegion2	23156
CNVR149	1816	14	56382005	56403580	loss	38110	6898350	149890749	CIE-L*	region1inRegion2	21575
CNVR150	1820	14	65589945	65600023	loss	38110	6898350	149890749	CIE-L*	region1inRegion2	10078
CNVR151	1829	14	74107606	74126308	loss	38110	6898350	149890749	CIE-L*	region1inRegion2	18702
CNVR152	1838	14	81377028	81553876	gain	38110	6898350	149890749	CIE-L*	region1inRegion2	176848
CNVR153	1849	14	94594494	94629904	gain	38110	6898350	149890749	CIE-L*	region1inRegion2	35410
CNVR154	1873	14	120810562	120829324	gain	38110	6898350	149890749	CIE-L*	region1inRegion2	18762
CNVR155	1880	14	127501895	127526173	gain	38110	6898350	149890749	CIE-L*	region1inRegion2	24278
CNVR146	1781	14	7794851	7822418	gain	5233	7424787	152596119	Body_weight_(birth)	region1inRegion2	27567
CNVR147	1782	14	8039487	8116098	gain	5233	7424787	152596119	Body_weight_(birth)	region1inRegion2	76611

CNVR148	1792	14	21068045	21091201	gain	5233	7424787	152596119	Body_weight_(birth)	region1inRegion2	23156
CNVR149	1816	14	56382005	56403580	loss	5233	7424787	152596119	Body_weight_(birth)	region1inRegion2	21575
CNVR150	1820	14	65589945	65600023	loss	5233	7424787	152596119	Body_weight_(birth)	region1inRegion2	10078
CNVR151	1829	14	74107606	74126308	loss	5233	7424787	152596119	Body_weight_(birth)	region1inRegion2	18702
CNVR152	1838	14	81377028	81553876	gain	5233	7424787	152596119	Body_weight_(birth)	region1inRegion2	176848
CNVR153	1849	14	94594494	94629904	gain	5233	7424787	152596119	Body_weight_(birth)	region1inRegion2	35410
CNVR154	1873	14	120810562	120829324	gain	5233	7424787	152596119	Body_weight_(birth)	region1inRegion2	18762
CNVR155	1880	14	127501895	127526173	gain	5233	7424787	152596119	Body_weight_(birth)	region1inRegion2	24278
CNVR148	1792	14	21068045	21091201	gain	3925	8528044	143234735	Shear_force	region1inRegion2	23156
CNVR149	1816	14	56382005	56403580	loss	3925	8528044	143234735	Shear_force	region1inRegion2	21575
CNVR150	1820	14	65589945	65600023	loss	3925	8528044	143234735	Shear_force	region1inRegion2	10078
CNVR151	1829	14	74107606	74126308	loss	3925	8528044	143234735	Shear_force	region1inRegion2	18702
CNVR152	1838	14	81377028	81553876	gain	3925	8528044	143234735	Shear_force	region1inRegion2	176848
CNVR153	1849	14	94594494	94629904	gain	3925	8528044	143234735	Shear_force	region1inRegion2	35410
CNVR154	1873	14	120810562	120829324	gain	3925	8528044	143234735	Shear_force	region1inRegion2	18762
CNVR155	1880	14	127501895	127526173	gain	3925	8528044	143234735	Shear_force	region1inRegion2	24278
CNVR148	1792	14	21068045	21091201	gain	3929	8528044	149890749	Shear_force_at_first_peak	region1inRegion2	23156
CNVR149	1816	14	56382005	56403580	loss	3929	8528044	149890749	Shear_force_at_first_peak	region1inRegion2	21575
CNVR150	1820	14	65589945	65600023	loss	3929	8528044	149890749	Shear_force_at_first_peak	region1inRegion2	10078
CNVR151	1829	14	74107606	74126308	loss	3929	8528044	149890749	Shear_force_at_first_peak	region1inRegion2	18702
CNVR152	1838	14	81377028	81553876	gain	3929	8528044	149890749	Shear_force_at_first_peak	region1inRegion2	176848
CNVR153	1849	14	94594494	94629904	gain	3929	8528044	149890749	Shear_force_at_first_peak	region1inRegion2	35410
CNVR154	1873	14	120810562	120829324	gain	3929	8528044	149890749	Shear_force_at_first_peak	region1inRegion2	18762
CNVR155	1880	14	127501895	127526173	gain	3929	8528044	149890749	Shear_force_at_first_peak	region1inRegion2	24278
CNVR148	1792	14	21068045	21091201	gain	3930	8528044	149890749	Shear_force_at_first_peak	region1inRegion2	23156
CNVR149	1816	14	56382005	56403580	loss	3930	8528044	149890749	Shear_force_at_first_peak	region1inRegion2	21575
CNVR150	1820	14	65589945	65600023	loss	3930	8528044	149890749	Shear_force_at_first_peak	region1inRegion2	10078
CNVR151	1829	14	74107606	74126308	loss	3930	8528044	149890749	Shear_force_at_first_peak	region1inRegion2	18702
CNVR152	1838	14	81377028	81553876	gain	3930	8528044	149890749	Shear_force_at_first_peak	region1inRegion2	176848
CNVR153	1849	14	94594494	94629904	gain	3930	8528044	149890749	Shear_force_at_first_peak	region1inRegion2	35410
CNVR154	1873	14	120810562	120829324	gain	3930	8528044	149890749	Shear_force_at_first_peak	region1inRegion2	18762
CNVR155	1880	14	127501895	127526173	gain	3930	8528044	149890749	Shear_force_at_first_peak	region1inRegion2	24278
CNVR148	1792	14	21068045	21091201	gain	3931	8528044	149890749	Shear_force_at_first_peak	region1inRegion2	23156
CNVR149	1816	14	56382005	56403580	loss	3931	8528044	149890749	Shear_force_at_first_peak	region1inRegion2	21575
CNVR150	1820	14	65589945	65600023	loss	3931	8528044	149890749	Shear_force_at_first_peak	region1inRegion2	10078
CNVR151	1829	14	74107606	74126308	loss	3931	8528044	149890749	Shear_force_at_first_peak	region1inRegion2	18702
CNVR152	1838	14	81377028	81553876	gain	3931	8528044	149890749	Shear_force_at_first_peak	region1inRegion2	176848
CNVR153	1849	14	94594494	94629904	gain	3931	8528044	149890749	Shear_force_at_first_peak	region1inRegion2	35410
CNVR154	1873	14	120810562	120829324	gain	3931	8528044	149890749	Shear_force_at_first_peak	region1inRegion2	18762
CNVR155	1880	14	127501895	127526173	gain	3931	8528044	149890749	Shear_force_at_first_peak	region1inRegion2	24278
CNVR148	1792	14	21068045	21091201	gain	7	8628665	130615641	Loin_muscle_area	region1inRegion2	23156
CNVR149	1816	14	56382005	56403580	loss	7	8628665	130615641	Loin_muscle_area	region1inRegion2	21575
CNVR150	1820	14	65589945	65600023	loss	7	8628665	130615641	Loin_muscle_area	region1inRegion2	10078
CNVR151	1829	14	74107606	74126308	loss	7	8628665	130615641	Loin_muscle_area	region1inRegion2	18702
CNVR152	1838	14	81377028	81553876	gain	7	8628665	130615641	Loin_muscle_area	region1inRegion2	176848
CNVR153	1849	14	94594494	94629904	gain	7	8628665	130615641	Loin_muscle_area	region1inRegion2	35410
CNVR154	1873	14	120810562	120829324	gain	7	8628665	130615641	Loin_muscle_area	region1inRegion2	18762
CNVR155	1880	14	127501895	127526173	gain	7	8628665	130615641	Loin_muscle_area	region1inRegion2	24278
CNVR148	1792	14	21068045	21091201	gain	21267	10886535	83590642	Leaf_fat_weight	region1inRegion2	23156
CNVR149	1816	14	56382005	56403580	loss	21267	10886535	83590642	Leaf_fat_weight	region1inRegion2	21575
CNVR150	1820	14	65589945	65600023	loss	21267	10886535	83590642	Leaf_fat_weight	region1inRegion2	10078
CNVR151	1829	14	74107606	74126308	loss	21267	10886535	83590642	Leaf_fat_weight	region1inRegion2	18702
CNVR152	1838	14	81377028	81553876	gain	21267	10886535	83590642	Leaf_fat_weight	region1inRegion2	176848
CNVR148	1792	14	21068045	21091201	gain	21266	10886535	114812357	Abdominal_fat_weight	region1inRegion2	23156
CNVR149	1816	14	56382005	56403580	loss	21266	10886535	114812357	Abdominal_fat_weight	region1inRegion2	21575
CNVR150	1820	14	65589945	65600023	loss	21266	10886535	114812357	Abdominal_fat_weight	region1inRegion2	10078
CNVR151	1829	14	74107606	74126308	loss	21266	10886535	114812357	Abdominal_fat_weight	region1inRegion2	18702
CNVR152	1838	14	81377028	81553876	gain	21266	10886535	114812357	Abdominal_fat_weight	region1inRegion2	176848
CNVR153	1849	14	94594494	94629904	gain	21266	10886535	114812357	Abdominal_fat_weight	region1inRegion2	35410
CNVR148	1792	14	21068045	21091201	gain	7572	11240704	60644503	Melanoma_susceptibility	region1inRegion2	23156
CNVR149	1816	14	56382005	56403580	loss	7572	11240704	60644503	Melanoma_susceptibility	region1inRegion2	21575
CNVR148	1792	14	21068045	21091201	gain	21268	11905361	127445304	intestinal_fat_weight	region1inRegion2	23156
CNVR149	1816	14	56382005	56403580	loss	21268	11905361	127445304	intestinal_fat_weight	region1inRegion2	21575
CNVR150	1820	14	65589945	65600023	loss	21268	11905361	127445304	intestinal_fat_weight	region1inRegion2	10078
CNVR151	1829	14	74107606	74126308	loss	21268	11905361	127445304	intestinal_fat_weight	region1inRegion2	18702
CNVR152	1838	14	81377028	81553876	gain	21268	11905361	127445304	intestinal_fat_weight	region1inRegion2	176848
CNVR153	1849	14	94594494	94629904	gain	21268	11905361	127445304	intestinal_fat_weight	region1inRegion2	35410
CNVR154	1873	14	120810562	120829324	gain	21268	11905361	127445304	intestinal_fat_weight	region1inRegion2	18762
CNVR148	1792	14	21068045	21091201	gain	18007	13351847	27531879	Subcutanous_fat_area	region1inRegion2	23156
CNVR148	1792	14	21068045	21091201	gain	18032	13351847	27531879	Adipocyte_diameter	region1inRegion2	23156
CNVR148	1792	14	21068045	21091201	gain	15079	13351847	93372131	Calcium_level	region1inRegion2	23156
CNVR149	1816	14	56382005	56403580	loss	15079	13351847	93372131	Calcium_level	region1inRegion2	21575
CNVR150	1820	14	65589945	65600023	loss	15079	13351847	93372131	Calcium_level	region1inRegion2	10078
CNVR151	1829	14	74107606	74126308	loss	15079	13351847	93372131	Calcium_level	region1inRegion2	18702
CNVR152	1838	14	81377028	81553876	gain	15079	13351847	93372131	Calcium_level	region1inRegion2	176848
CNVR148	1792	14	21068045	21091201	gain	6362	13351847	93372131	Creatinine_level	region1inRegion2	23156
CNVR149	1816	14	56382005	56403580	loss	6362	13351847	93372131	Creatinine_level	region1inRegion2	21575
CNVR150	1820	14	65589945	65600023	loss	6362	13351847	93372131	Creatinine_level	region1inRegion2	10078
CNVR151	1829	14	74107606	74126308	loss	6362	13351847	93372131	Creatinine_level	region1inRegion2	18702
CNVR152	1838	14	81377028	81553876	gain	6362	13351847	93372131	Creatinine_level	region1inRegion2	176848
CNVR148	1792	14	21068045	21091201	gain	6364	13351847	93372131	Creatinine_level	region1inRegion2	23156
CNVR149	1816	14	56382005	56403580	loss	6364	13351847	93372131	Creatinine_level	region1inRegion2	21575
CNVR150	1820	14	65589945	65600023	loss	6364	13351847	93372131	Creatinine_level	region1inRegion2	10078
CNVR151	1829	14	74107606	74126308	loss	6364	13351847	93372131	Creatinine_level	region1inRegion2	18702
CNVR152	1838	14	81377028	81553876	gain	6364	13351847	93372131	Creatinine_level	region1inRegion2	176848
CNVR148	1792	14	21068045	21091201	gain	6384	13351847	93372131	Potassium_level	region1inRegion2	23156
CNVR149	1816	14	56382005	56403580	loss	6384	13351847	93372131	Potassium_level	region1inRegion2	21575
CNVR150	1820	14	65589945	65600023	loss	6384	13351847	93372131	Potassium_level	region1inRegion2	10078
CNVR151	1829	14	74107606	74126308	loss	6384	13351847	93372131	Potassium_level	region1inRegion2	18702
CNVR152	1838	14	81377028	81553876	gain	6384	13351847	93372131	Potassium_level	region1inRegion2	176848
CNVR148	1792	14	21068045	21091201	gain	18684	13669217	23648157	Reflectance_value	region1inRegion2	23156
CNVR148	1792	14	21068045	21091201	gain	18716	13669217	23648157	Reflectance_value	region1inRegion2	23156
CNVR148	1792	14	21068045	21091201	gain	18411	14113496	28261775	Reproductive_tract_weight	region1inRegion2	23156
CNVR148	1792	14	21068045	21091201	gain	606	14141727	132053949	Fat_androstenone_level	region1inRegion2	23156
CNVR149	1816	14	56382005	56403580	loss	606	14141727	132053949	Fat_androstenone_level	region1inRegion2	21575
CNVR150	1820	14	65589945	65600023	loss	606	14141727	132053949	Fat_androstenone_level	region1inRegion2	10078
CNVR151	1829	14	74107606	74126308	loss	606	14141727	132053949	Fat_androstenone_level	region1inRegion2	18702
CNVR152	1838	14	81377028	81553876	gain	606	14141727	132053949	Fat_androstenone_level	region1inRegion2	176848
CNVR153	1849	14	94594494	94629904	gain	606	14141727	132053949	Fat_androstenone_level	region1inRegion2	35410
CNVR154	1873	14	120810562	120829324	gain	606	14141727	132053949	Fat_androstenone_level	region1inRegion2	18762
CNVR155	1880	14	127501895	127526173	gain	606	14141727	132053949	Fat_androstenone_level	region1inRegion2	24278
CNVR148	1792	14	21068045	21091201	gain	607	14141727	132053949	Fat_androstenone_level	region1inRegion2	23156
CNVR149	1816	14	56382005	56403580	loss	607	14141727	132053949	Fat_androstenone_level	region1inRegion2	21575
CNVR150	1820	14	65589945	65600023	loss	607	14141727	132053949	Fat_androstenone_level	region1inRegion2	10078
CNVR151	1829	14	74107606	74126308	loss	607	14141727	132053949	Fat_androstenone_level	region1inRegion2	18702
CNVR152	1838	14	81377028	81553876	gain	607	14141727	132053949	Fat_androstenone_level	region1inRegion2	176848
CNVR153	1849	14	94594494	94629904	gain	607	14141727	132053949	Fat_androstenone_level	region1inRegion2	35410
CNVR154	1873	14	120810562	120829324	gain	607	14141727	132053949	Fat_androstenone_level	region1inRegion2	18762
CNVR155	1880	14	127501895	127526173	gain	607	14141727	132053949	Fat_androstenone_level	region1inRegion2	24278
CNVR148	1792	14	21068045	21091201	gain	608	14141727	132053949	Fat_androstenone_level	region1inRegion2	23156
CNVR149	1816	14	56382005	56403580	loss	608	14141727	132053949	Fat_androstenone_level	region1inRegion2	21575
CNVR150	1820	14	65589945	65600023	loss	608	14141727	132053949	Fat_androstenone_level	region1inRegion2	10078
CNVR151	1829	14	74107606	74126308	loss	608	14141727	132053949	Fat_androstenone_level	region1inRegion2	18702
CNVR152	1838	14	81377028	81553876	gain	608	14141727	132053949	Fat_androstenone_level	region1inRegion2	176848
CNVR153	1849	14	94594494	94629904	gain	608	14141727	132053949	Fat_androstenone_level	region1inRegion2	35410
CNVR154	1873	14	120810562	120829324	gain	608	14141727	132053949	Fat_androstenone_level	region1inRegion2	18762
CNVR155	1880	14	127501895	127526173	gain	608	14141727	132053949	Fat_androstenone_level	region1inRegion2	24278
CNVR148	1792	14	21068045	21091201	gain	609	14141727	132053949	Fat_androstenone_level	region1inRegion2	23156
CNVR149	1816	14	56382005	56403580	loss	609	14141727	132053949	Fat_androstenone_level	region1inRegion2	21575
CNVR150	1820	14	65589945	65600023	loss	609	14141727	132053949	Fat_androstenone_level	region1inRegion2	10078
CNVR151	1829	14	74107606	74126308	loss	609	14141727	132053949	Fat_androstenone_level	region1inRegion2	18702
CNVR152	1838	14	81377028	81553876	gain	609	14141727	132053949	Fat_androstenone_level	region1inRegion2	176848

CNVR153	1849	14	94594494	94629904	gain	609	14141727	132053949	Fat_androstenone_level	region1inRegion2	35410
CNVR154	1873	14	120810562	120829324	gain	609	14141727	132053949	Fat_androstenone_level	region1inRegion2	18762
CNVR155	1880	14	127501895	127526173	gain	609	14141727	132053949	Fat_androstenone_level	region1inRegion2	24278
CNVR148	1792	14	21068045	21091201	gain	31211	14258116	34459772	Total_number_born_alive	region1inRegion2	23156
CNVR148	1792	14	21068045	21091201	gain	31212	14258116	34459772	Total_number_born_alive	region1inRegion2	23156
CNVR148	1792	14	21068045	21091201	gain	31213	14258116	34459772	Total_number_born_alive	region1inRegion2	23156
CNVR148	1792	14	21068045	21091201	gain	4237	16066507	41217524	skatole,_sensory_panel	region1inRegion2	23156
CNVR148	1792	14	21068045	21091201	gain	4238	16066507	41217524	subjective_boar_flavor_in_lean	region1inRegion2	23156
CNVR148	1792	14	21068045	21091201	gain	4241	16066507	41217524	subjective_boar_flavor_in_fat	region1inRegion2	23156
CNVR148	1792	14	21068045	21091201	gain	4242	16066507	41217524	subjective_abnormal_odor	region1inRegion2	23156
CNVR148	1792	14	21068045	21091201	gain	8923	16066507	41217524	Front_leg_conformation	region1inRegion2	23156
CNVR148	1792	14	21068045	21091201	gain	3814	16066507	132053949	Average_instron_(star_probe)_force	region1inRegion2	23156
CNVR149	1816	14	56382005	56403580	loss	3814	16066507	132053949	Average_instron_(star_probe)_force	region1inRegion2	21575
CNVR150	1820	14	65589945	65600023	loss	3814	16066507	132053949	Average_instron_(star_probe)_force	region1inRegion2	10078
CNVR151	1829	14	74107606	74126308	loss	3814	16066507	132053949	Average_instron_(star_probe)_force	region1inRegion2	18702
CNVR152	1838	14	81377028	81553876	gain	3814	16066507	132053949	Average_instron_(star_probe)_force	region1inRegion2	176848
CNVR153	1849	14	94594494	94629904	gain	3814	16066507	132053949	Average_instron_(star_probe)_force	region1inRegion2	35410
CNVR154	1873	14	120810562	120829324	gain	3814	16066507	132053949	Average_instron_(star_probe)_force	region1inRegion2	18762
CNVR155	1880	14	127501895	127526173	gain	3814	16066507	132053949	Average_instron_(star_probe)_force	region1inRegion2	24278
CNVR148	1792	14	21068045	21091201	gain	7535	19260466	29711236	Number_of_stillborn	region1inRegion2	23156
CNVR148	1792	14	21068045	21091201	gain	14	19260466	60644503	Trimmed_wholesale_product_/_live_weight	region1inRegion2	23156
CNVR149	1816	14	56382005	56403580	loss	14	19260466	60644503	Trimmed_wholesale_product_/_live_weight	region1inRegion2	21575
CNVR148	1792	14	21068045	21091201	gain	3861	19260466	65129650	Loin_muscle_area	region1inRegion2	23156
CNVR149	1816	14	56382005	56403580	loss	3861	19260466	65129650	Loin_muscle_area	region1inRegion2	21575
CNVR148	1792	14	21068045	21091201	gain	7018	19260466	81745465	Diameter_of_type_IIa_muscle_fibers	region1inRegion2	23156
CNVR149	1816	14	56382005	56403580	loss	7018	19260466	81745465	Diameter_of_type_IIa_muscle_fibers	region1inRegion2	21575
CNVR150	1820	14	65589945	65600023	loss	7018	19260466	81745465	Diameter_of_type_IIa_muscle_fibers	region1inRegion2	10078
CNVR151	1829	14	74107606	74126308	loss	7018	19260466	81745465	Diameter_of_type_IIa_muscle_fibers	region1inRegion2	18702
CNVR152	1838	14	81377028	81553876	gain	7018	19260466	81745465	Diameter_of_type_IIa_muscle_fibers	region1inRegion2	176848
CNVR148	1792	14	21068045	21091201	gain	7024	19260466	81745465	Percentage_type_IIb_fibers	region1inRegion2	23156
CNVR149	1816	14	56382005	56403580	loss	7024	19260466	81745465	Percentage_type_IIb_fibers	region1inRegion2	21575
CNVR150	1820	14	65589945	65600023	loss	7024	19260466	81745465	Percentage_type_IIb_fibers	region1inRegion2	10078
CNVR151	1829	14	74107606	74126308	loss	7024	19260466	81745465	Percentage_type_IIb_fibers	region1inRegion2	18702
CNVR152	1838	14	81377028	81553876	gain	7024	19260466	81745465	Percentage_type_IIb_fibers	region1inRegion2	176848
CNVR148	1792	14	21068045	21091201	gain	7038	19260466	81745465	Diameter_of_type_IIb_muscle_fibers	region1inRegion2	23156
CNVR149	1816	14	56382005	56403580	loss	7038	19260466	81745465	Diameter_of_type_IIb_muscle_fibers	region1inRegion2	21575
CNVR150	1820	14	65589945	65600023	loss	7038	19260466	81745465	Diameter_of_type_IIb_muscle_fibers	region1inRegion2	10078
CNVR151	1829	14	74107606	74126308	loss	7038	19260466	81745465	Diameter_of_type_IIb_muscle_fibers	region1inRegion2	18702
CNVR152	1838	14	81377028	81553876	gain	7038	19260466	81745465	Diameter_of_type_IIb_muscle_fibers	region1inRegion2	176848
CNVR148	1792	14	21068045	21091201	gain	2924	20942196	21220482	Average_backfat_thickness	region1inRegion2	23156
CNVR149	1816	14	56382005	56403580	loss	18	24310612	115480974	Trimmed_wholesale_product_/_carcass_weight	region1inRegion2	21575
CNVR150	1820	14	65589945	65600023	loss	18	24310612	115480974	Trimmed_wholesale_product_/_carcass_weight	region1inRegion2	10078
CNVR151	1829	14	74107606	74126308	loss	18	24310612	115480974	Trimmed_wholesale_product_/_carcass_weight	region1inRegion2	18702
CNVR152	1838	14	81377028	81553876	gain	18	24310612	115480974	Trimmed_wholesale_product_/_carcass_weight	region1inRegion2	176848
CNVR153	1849	14	94594494	94629904	gain	18	24310612	115480974	Trimmed_wholesale_product_/_carcass_weight	region1inRegion2	35410
CNVR149	1816	14	56382005	56403580	loss	12272	27531879	60644503	Interferon-gamma_to_interleukin-10_ratio	region1inRegion2	21575
CNVR149	1816	14	56382005	56403580	loss	1165	27531879	81745465	Dressing_percentage	region1inRegion2	21575
CNVR150	1820	14	65589945	65600023	loss	1165	27531879	81745465	Dressing_percentage	region1inRegion2	10078
CNVR151	1829	14	74107606	74126308	loss	1165	27531879	81745465	Dressing_percentage	region1inRegion2	18702
CNVR152	1838	14	81377028	81553876	gain	1165	27531879	81745465	Dressing_percentage	region1inRegion2	176848
CNVR149	1816	14	56382005	56403580	loss	16873	27531879	132170772	Carcass_weight_(hot)	region1inRegion2	21575
CNVR150	1820	14	65589945	65600023	loss	16873	27531879	132170772	Carcass_weight_(hot)	region1inRegion2	10078
CNVR151	1829	14	74107606	74126308	loss	16873	27531879	132170772	Carcass_weight_(hot)	region1inRegion2	18702
CNVR152	1838	14	81377028	81553876	gain	16873	27531879	132170772	Carcass_weight_(hot)	region1inRegion2	176848
CNVR153	1849	14	94594494	94629904	gain	16873	27531879	132170772	Carcass_weight_(hot)	region1inRegion2	35410
CNVR154	1873	14	120810562	120829324	gain	16873	27531879	132170772	Carcass_weight_(hot)	region1inRegion2	18762
CNVR155	1880	14	127501895	127526173	gain	16873	27531879	132170772	Carcass_weight_(hot)	region1inRegion2	24278
CNVR149	1816	14	56382005	56403580	loss	37	27604850	60644503	Average_backfat_thickness	region1inRegion2	21575
CNVR149	1816	14	56382005	56403580	loss	33	27604850	66305680	Backfat_at_last_lumbar	region1inRegion2	21575
CNVR150	1820	14	65589945	65600023	loss	33	27604850	66305680	Backfat_at_last_lumbar	region1inRegion2	10078
CNVR149	1816	14	56382005	56403580	loss	8810	32915907	132053949	Body_weight	region1inRegion2	21575
CNVR150	1820	14	65589945	65600023	loss	8810	32915907	132053949	Body_weight	region1inRegion2	10078
CNVR151	1829	14	74107606	74126308	loss	8810	32915907	132053949	Body_weight	region1inRegion2	18702
CNVR152	1838	14	81377028	81553876	gain	8810	32915907	132053949	Body_weight	region1inRegion2	176848
CNVR153	1849	14	94594494	94629904	gain	8810	32915907	132053949	Body_weight	region1inRegion2	35410
CNVR154	1873	14	120810562	120829324	gain	8810	32915907	132053949	Body_weight	region1inRegion2	18762
CNVR155	1880	14	127501895	127526173	gain	8810	32915907	132053949	Body_weight	region1inRegion2	24278
CNVR149	1816	14	56382005	56403580	loss	234	41217524	81745465	Meat_color-L	region1inRegion2	21575
CNVR150	1820	14	65589945	65600023	loss	234	41217524	81745465	Meat_color-L	region1inRegion2	10078
CNVR151	1829	14	74107606	74126308	loss	234	41217524	81745465	Meat_color-L	region1inRegion2	18702
CNVR152	1838	14	81377028	81553876	gain	234	41217524	81745465	Meat_color-L	region1inRegion2	176848
CNVR149	1816	14	56382005	56403580	loss	4239	41217524	81745465	subjective_boar_flavor_in_lean	region1inRegion2	21575
CNVR150	1820	14	65589945	65600023	loss	4239	41217524	81745465	subjective_boar_flavor_in_lean	region1inRegion2	10078
CNVR151	1829	14	74107606	74126308	loss	4239	41217524	81745465	subjective_boar_flavor_in_lean	region1inRegion2	18702
CNVR152	1838	14	81377028	81553876	gain	4239	41217524	81745465	subjective_boar_flavor_in_lean	region1inRegion2	176848
CNVR149	1816	14	56382005	56403580	loss	8925	41217524	81745465	Hind_leg_conformation	region1inRegion2	21575
CNVR150	1820	14	65589945	65600023	loss	8925	41217524	81745465	Hind_leg_conformation	region1inRegion2	10078
CNVR151	1829	14	74107606	74126308	loss	8925	41217524	81745465	Hind_leg_conformation	region1inRegion2	18702
CNVR152	1838	14	81377028	81553876	gain	8925	41217524	81745465	Hind_leg_conformation	region1inRegion2	176848
CNVR149	1816	14	56382005	56403580	loss	13351	45381886	83901479	Cervical_vertebra_length	region1inRegion2	21575
CNVR150	1820	14	65589945	65600023	loss	13351	45381886	83901479	Cervical_vertebra_length	region1inRegion2	10078
CNVR151	1829	14	74107606	74126308	loss	13351	45381886	83901479	Cervical_vertebra_length	region1inRegion2	18702
CNVR152	1838	14	81377028	81553876	gain	13351	45381886	83901479	Cervical_vertebra_length	region1inRegion2	176848
CNVR149	1816	14	56382005	56403580	loss	30906	50181449	61727099	Linoleic_acid_content	region1inRegion2	21575
CNVR149	1816	14	56382005	56403580	loss	11579	53465935	81745465	Salmonella_count_in_liver_and_spleen	region1inRegion2	21575
CNVR150	1820	14	65589945	65600023	loss	11579	53465935	81745465	Salmonella_count_in_liver_and_spleen	region1inRegion2	10078
CNVR151	1829	14	74107606	74126308	loss	11579	53465935	81745465	Salmonella_count_in_liver_and_spleen	region1inRegion2	18702
CNVR152	1838	14	81377028	81553876	gain	11579	53465935	81745465	Salmonella_count_in_liver_and_spleen	region1inRegion2	176848
CNVR149	1816	14	56382005	56403580	loss	11580	53465935	81745465	Salmonella_count_in_liver	region1inRegion2	21575
CNVR150	1820	14	65589945	65600023	loss	11580	53465935	81745465	Salmonella_count_in_liver	region1inRegion2	10078
CNVR151	1829	14	74107606	74126308	loss	11580	53465935	81745465	Salmonella_count_in_liver	region1inRegion2	18702
CNVR152	1838	14	81377028	81553876	gain	11580	53465935	81745465	Salmonella_count_in_liver	region1inRegion2	176848
CNVR150	1820	14	65589945	65600023	loss	4035	57387449	80027337	Relative_area_of_type_IIb_fibers	region1inRegion2	10078
CNVR151	1829	14	74107606	74126308	loss	4035	57387449	80027337	Relative_area_of_type_IIb_fibers	region1inRegion2	18702
CNVR150	1820	14	65589945	65600023	loss	13318	59002615	109924185	Cervical_vertebra_length	region1inRegion2	10078
CNVR151	1829	14	74107606	74126308	loss	13318	59002615	109924185	Cervical_vertebra_length	region1inRegion2	18702
CNVR152	1838	14	81377028	81553876	gain	13318	59002615	109924185	Cervical_vertebra_length	region1inRegion2	176848
CNVR153	1849	14	94594494	94629904	gain	13318	59002615	109924185	Cervical_vertebra_length	region1inRegion2	35410
CNVR151	1829	14	74107606	74126308	loss	4022	69115424	114543972	Percentage_type_I_fibers	region1inRegion2	18702
CNVR152	1838	14	81377028	81553876	gain	4022	69115424	114543972	Percentage_type_I_fibers	region1inRegion2	176848
CNVR153	1849	14	94594494	94629904	gain	4022	69115424	114543972	Percentage_type_I_fibers	region1inRegion2	35410
CNVR151	1829	14	74107606	74126308	loss	4045	73412985	114757839	Percentage_type_IIb_fibers	region1inRegion2	18702
CNVR152	1838	14	81377028	81553876	gain	4045	73412985	114757839	Percentage_type_IIb_fibers	region1inRegion2	176848
CNVR153	1849	14	94594494	94629904	gain	4045	73412985	114757839	Percentage_type_IIb_fibers	region1inRegion2	35410
CNVR151	1829	14	74107606	74126308	loss	64662	74119670	74977277	Thoracolumbar_vertebra_number	overlapTail	6639
CNVR153	1849	14	94594494	94629904	gain	21405	81745465	108264282	CIE-a*	region1inRegion2	35410
CNVR153	1849	14	94594494	94629904	gain	21441	81745465	108264282	Shoulder_weight,_Boston	region1inRegion2	35410
CNVR153	1849	14	94594494	94629904	gain	3289	81745465	108264282	Meat_color-a	region1inRegion2	35410
CNVR153	1849	14	94594494	94629904	gain	337	81745465	132053949	Average_daily_gain	region1inRegion2	35410
CNVR154	1873	14	120810562	120829324	gain	337	81745465	132053949	Average_daily_gain	region1inRegion2	18762
CNVR155	1880	14	127501895	127526173	gain	337	81745465	132053949	Average_daily_gain	region1inRegion2	24278
CNVR153	1849	14	94594494	94629904	gain	3972	81745465	132053949	Semimembranosus_angle	region1inRegion2	35410
CNVR154	1873	14	120810562	120829324	gain	3972	81745465	132053949	Semimembranosus_angle	region1inRegion2	18762
CNVR155	1880	14	127501895	127526173	gain	3972	81745465	132053949	Semimembranosus_angle	region1inRegion2	24278
CNVR153	1849	14	94594494	94629904	gain	4007	81745465	132053949	CIE-b*	region1inRegion2	35410
CNVR154	1873	14	120810562	120829324	gain	4007	81745465	132053949	CIE-b*	region1inRegion2	18762
CNVR155	1880	14	127501895	127526173	gain	4007	81745465	132053949	CIE-b*	region1inRegion2	24278
CNVR153	1849	14	94594494	94629904	gain	4008	81745465	132053949	Total_shear_work	region1inRegion2	35410
CNVR154	1873	14	120810562	120829324	gain	4008	81745465	132053949	Total_shear_work	region1inRegion2	18762
CNVR155	1880	14	127501895	127526173	gain	4008	81745465	132053949	Total_shear_work	region1inRegion2	24278
CNVR153	1849	14	94594494	94629904	gain	1149	81745465	132170772	Ham_percentage	region1inRegion2	35410
CNVR154	1873	14	120810562	120829324	gain	1149	81745465	132170772	Ham_percentage	region1inRegion2	18762

CNVR155	1880	14	127501895	127526173	gain	1149	81745465	132170772	Ham_percentage	region1inRegion2	24278
CNVR153	1849	14	94594494	94629904	gain	1150	81745465	132170772	Fat-cuts_percentage	region1inRegion2	35410
CNVR154	1873	14	120810562	120829324	gain	1150	81745465	132170772	Fat-cuts_percentage	region1inRegion2	18762
CNVR155	1880	14	127501895	127526173	gain	1150	81745465	132170772	Fat-cuts_percentage	region1inRegion2	24278
CNVR153	1849	14	94594494	94629904	gain	1151	81745465	132170772	Dressing_percentage	region1inRegion2	35410
CNVR154	1873	14	120810562	120829324	gain	1151	81745465	132170772	Dressing_percentage	region1inRegion2	18762
CNVR155	1880	14	127501895	127526173	gain	1151	81745465	132170772	Dressing_percentage	region1inRegion2	24278
CNVR153	1849	14	94594494	94629904	gain	1152	81745465	132170772	Ham_percentage	region1inRegion2	35410
CNVR154	1873	14	120810562	120829324	gain	1152	81745465	132170772	Ham_percentage	region1inRegion2	18762
CNVR155	1880	14	127501895	127526173	gain	1152	81745465	132170772	Ham_percentage	region1inRegion2	24278
CNVR153	1849	14	94594494	94629904	gain	1153	81745465	132170772	Carcass_length	region1inRegion2	35410
CNVR154	1873	14	120810562	120829324	gain	1153	81745465	132170772	Carcass_length	region1inRegion2	18762
CNVR155	1880	14	127501895	127526173	gain	1153	81745465	132170772	Carcass_length	region1inRegion2	24278
CNVR153	1849	14	94594494	94629904	gain	1154	81745465	132170772	Loin_muscle_area	region1inRegion2	35410
CNVR154	1873	14	120810562	120829324	gain	1154	81745465	132170772	Loin_muscle_area	region1inRegion2	18762
CNVR155	1880	14	127501895	127526173	gain	1154	81745465	132170772	Loin_muscle_area	region1inRegion2	24278
CNVR153	1849	14	94594494	94629904	gain	1156	81745465	132170772	Carcass_weight_(cold)	region1inRegion2	35410
CNVR154	1873	14	120810562	120829324	gain	1156	81745465	132170772	Carcass_weight_(cold)	region1inRegion2	18762
CNVR155	1880	14	127501895	127526173	gain	1156	81745465	132170772	Carcass_weight_(cold)	region1inRegion2	24278
CNVR153	1849	14	94594494	94629904	gain	1157	81745465	132170772	Loin_and_neck_meat_weight	region1inRegion2	35410
CNVR154	1873	14	120810562	120829324	gain	1157	81745465	132170772	Loin_and_neck_meat_weight	region1inRegion2	18762
CNVR155	1880	14	127501895	127526173	gain	1157	81745465	132170772	Loin_and_neck_meat_weight	region1inRegion2	24278
CNVR153	1849	14	94594494	94629904	gain	1158	81745465	132170772	Ham_weight	region1inRegion2	35410
CNVR154	1873	14	120810562	120829324	gain	1158	81745465	132170772	Ham_weight	region1inRegion2	18762
CNVR155	1880	14	127501895	127526173	gain	1158	81745465	132170772	Ham_weight	region1inRegion2	24278
CNVR153	1849	14	94594494	94629904	gain	1159	81745465	132170772	Body_weight_(slaughter)	region1inRegion2	35410
CNVR154	1873	14	120810562	120829324	gain	1159	81745465	132170772	Body_weight_(slaughter)	region1inRegion2	18762
CNVR155	1880	14	127501895	127526173	gain	1159	81745465	132170772	Body_weight_(slaughter)	region1inRegion2	24278
CNVR153	1849	14	94594494	94629904	gain	1160	81745465	132170772	Shoulder_subcutaneous_fat_thickness	region1inRegion2	35410
CNVR154	1873	14	120810562	120829324	gain	1160	81745465	132170772	Shoulder_subcutaneous_fat_thickness	region1inRegion2	18762
CNVR155	1880	14	127501895	127526173	gain	1160	81745465	132170772	Shoulder_subcutaneous_fat_thickness	region1inRegion2	24278
CNVR153	1849	14	94594494	94629904	gain	1161	81745465	132170772	Average_backfat_thickness	region1inRegion2	35410
CNVR154	1873	14	120810562	120829324	gain	1161	81745465	132170772	Average_backfat_thickness	region1inRegion2	18762
CNVR155	1880	14	127501895	127526173	gain	1161	81745465	132170772	Average_backfat_thickness	region1inRegion2	24278
CNVR153	1849	14	94594494	94629904	gain	1163	81745465	132170772	backfat_at_last_rib	region1inRegion2	35410
CNVR154	1873	14	120810562	120829324	gain	1163	81745465	132170772	backfat_at_last_rib	region1inRegion2	18762
CNVR155	1880	14	127501895	127526173	gain	1163	81745465	132170772	backfat_at_last_rib	region1inRegion2	24278
CNVR153	1849	14	94594494	94629904	gain	1164	81745465	132170772	Feed_intake	region1inRegion2	35410
CNVR154	1873	14	120810562	120829324	gain	1164	81745465	132170772	Feed_intake	region1inRegion2	18762
CNVR155	1880	14	127501895	127526173	gain	1164	81745465	132170772	Feed_intake	region1inRegion2	24278
CNVR153	1849	14	94594494	94629904	gain	1166	81745465	132170772	Head_weight	region1inRegion2	35410
CNVR154	1873	14	120810562	120829324	gain	1166	81745465	132170772	Head_weight	region1inRegion2	18762
CNVR155	1880	14	127501895	127526173	gain	1166	81745465	132170772	Head_weight	region1inRegion2	24278
CNVR153	1849	14	94594494	94629904	gain	1167	81745465	132170772	External_fat_on_ham	region1inRegion2	35410
CNVR154	1873	14	120810562	120829324	gain	1167	81745465	132170772	External_fat_on_ham	region1inRegion2	18762
CNVR155	1880	14	127501895	127526173	gain	1167	81745465	132170772	External_fat_on_ham	region1inRegion2	24278
CNVR153	1849	14	94594494	94629904	gain	1168	81745465	132170772	Heart_weight	region1inRegion2	35410
CNVR154	1873	14	120810562	120829324	gain	1168	81745465	132170772	Heart_weight	region1inRegion2	18762
CNVR155	1880	14	127501895	127526173	gain	1168	81745465	132170772	Heart_weight	region1inRegion2	24278
CNVR153	1849	14	94594494	94629904	gain	1169	81745465	132170772	backfat_at_last_rib	region1inRegion2	35410
CNVR154	1873	14	120810562	120829324	gain	1169	81745465	132170772	backfat_at_last_rib	region1inRegion2	18762
CNVR155	1880	14	127501895	127526173	gain	1169	81745465	132170772	backfat_at_last_rib	region1inRegion2	24278
CNVR153	1849	14	94594494	94629904	gain	1242	81745465	132170772	Ham_weight	region1inRegion2	35410
CNVR154	1873	14	120810562	120829324	gain	1242	81745465	132170772	Ham_weight	region1inRegion2	18762
CNVR155	1880	14	127501895	127526173	gain	1242	81745465	132170772	Ham_weight	region1inRegion2	24278
CNVR153	1849	14	94594494	94629904	gain	1261	81745465	132170772	Carcass_length	region1inRegion2	35410
CNVR154	1873	14	120810562	120829324	gain	1261	81745465	132170772	Carcass_length	region1inRegion2	18762
CNVR155	1880	14	127501895	127526173	gain	1261	81745465	132170772	Carcass_length	region1inRegion2	24278
CNVR153	1849	14	94594494	94629904	gain	12850	81745465	132170772	Adipocyte_diameter	region1inRegion2	35410
CNVR154	1873	14	120810562	120829324	gain	12850	81745465	132170772	Adipocyte_diameter	region1inRegion2	18762
CNVR155	1880	14	127501895	127526173	gain	12850	81745465	132170772	Adipocyte_diameter	region1inRegion2	24278
CNVR153	1849	14	94594494	94629904	gain	12851	81745465	132170772	Adipocyte_diameter	region1inRegion2	35410
CNVR154	1873	14	120810562	120829324	gain	12851	81745465	132170772	Adipocyte_diameter	region1inRegion2	18762
CNVR155	1880	14	127501895	127526173	gain	12851	81745465	132170772	Adipocyte_diameter	region1inRegion2	24278
CNVR153	1849	14	94594494	94629904	gain	12852	81745465	132170772	Adipocyte_diameter	region1inRegion2	35410
CNVR154	1873	14	120810562	120829324	gain	12852	81745465	132170772	Adipocyte_diameter	region1inRegion2	18762
CNVR155	1880	14	127501895	127526173	gain	12852	81745465	132170772	Adipocyte_diameter	region1inRegion2	24278
CNVR153	1849	14	94594494	94629904	gain	11581	81745465	140166364	Salmonella_count_in_liver_and_spleen	region1inRegion2	35410
CNVR154	1873	14	120810562	120829324	gain	11581	81745465	140166364	Salmonella_count_in_liver_and_spleen	region1inRegion2	18762
CNVR155	1880	14	127501895	127526173	gain	11581	81745465	140166364	Salmonella_count_in_liver_and_spleen	region1inRegion2	24278
CNVR153	1849	14	94594494	94629904	gain	11582	81745465	140166364	Salmonella_count_in_liver	region1inRegion2	35410
CNVR154	1873	14	120810562	120829324	gain	11582	81745465	140166364	Salmonella_count_in_liver	region1inRegion2	18762
CNVR155	1880	14	127501895	127526173	gain	11582	81745465	140166364	Salmonella_count_in_liver	region1inRegion2	24278
CNVR153	1849	14	94594494	94629904	gain	11583	81745465	140166364	Salmonella_count_in_spleen	region1inRegion2	35410
CNVR154	1873	14	120810562	120829324	gain	11583	81745465	140166364	Salmonella_count_in_spleen	region1inRegion2	18762
CNVR155	1880	14	127501895	127526173	gain	11583	81745465	140166364	Salmonella_count_in_spleen	region1inRegion2	24278
CNVR153	1849	14	94594494	94629904	gain	1155	81745465	140815623	Shoulder_meat_weight	region1inRegion2	35410
CNVR154	1873	14	120810562	120829324	gain	1155	81745465	140815623	Shoulder_meat_weight	region1inRegion2	18762
CNVR155	1880	14	127501895	127526173	gain	1155	81745465	140815623	Shoulder_meat_weight	region1inRegion2	24278
CNVR153	1849	14	94594494	94629904	gain	18013	81745465	140815623	Fat_protein_content	region1inRegion2	35410
CNVR154	1873	14	120810562	120829324	gain	18013	81745465	140815623	Fat_protein_content	region1inRegion2	18762
CNVR155	1880	14	127501895	127526173	gain	18013	81745465	140815623	Fat_protein_content	region1inRegion2	24278
CNVR153	1849	14	94594494	94629904	gain	21861	81745465	149890749	pH_24_hr_post-mortem_(loin)	region1inRegion2	35410
CNVR154	1873	14	120810562	120829324	gain	21861	81745465	149890749	pH_24_hr_post-mortem_(loin)	region1inRegion2	18762
CNVR155	1880	14	127501895	127526173	gain	21861	81745465	149890749	pH_24_hr_post-mortem_(loin)	region1inRegion2	24278
CNVR153	1849	14	94594494	94629904	gain	7642	84652951	114800186	Gluteus_medius_depth	region1inRegion2	35410
CNVR153	1849	14	94594494	94629904	gain	15872	91580084	113959009	Stearic_acid_content	region1inRegion2	35410
CNVR153	1849	14	94594494	94629904	gain	12305	93372131	132053949	Mean_corpuscular_hemoglobin_content	region1inRegion2	35410
CNVR154	1873	14	120810562	120829324	gain	12305	93372131	132053949	Mean_corpuscular_hemoglobin_content	region1inRegion2	18762
CNVR155	1880	14	127501895	127526173	gain	12305	93372131	132053949	Mean_corpuscular_hemoglobin_content	region1inRegion2	24278
CNVR153	1849	14	94594494	94629904	gain	12306	93372131	132053949	Mean_corpuscular_hemoglobin_concentration	region1inRegion2	35410
CNVR154	1873	14	120810562	120829324	gain	12306	93372131	132053949	Mean_corpuscular_hemoglobin_concentration	region1inRegion2	18762
CNVR155	1880	14	127501895	127526173	gain	12306	93372131	132053949	Mean_corpuscular_hemoglobin_concentration	region1inRegion2	24278
CNVR153	1849	14	94594494	94629904	gain	12307	93372131	132053949	Red_cell_distribution_width	region1inRegion2	35410
CNVR154	1873	14	120810562	120829324	gain	12307	93372131	132053949	Red_cell_distribution_width	region1inRegion2	18762
CNVR155	1880	14	127501895	127526173	gain	12307	93372131	132053949	Red_cell_distribution_width	region1inRegion2	24278
CNVR153	1849	14	94594494	94629904	gain	21335	93372131	134456199	Granular_leukocyte_to_lymphocyte_ratio	region1inRegion2	35410
CNVR154	1873	14	120810562	120829324	gain	21335	93372131	134456199	Granular_leukocyte_to_lymphocyte_ratio	region1inRegion2	18762
CNVR155	1880	14	127501895	127526173	gain	21335	93372131	134456199	Granular_leukocyte_to_lymphocyte_ratio	region1inRegion2	24278
CNVR153	1849	14	94594494	94629904	gain	10223	93372131	137879539	Aspartate_amino_transferase_activity	region1inRegion2	35410
CNVR154	1873	14	120810562	120829324	gain	10223	93372131	137879539	Aspartate_amino_transferase_activity	region1inRegion2	18762
CNVR155	1880	14	127501895	127526173	gain	10223	93372131	137879539	Aspartate_amino_transferase_activity	region1inRegion2	24278
CNVR153	1849	14	94594494	94629904	gain	10224	93372131	137879539	Aspartate_amino_transferase_activity	region1inRegion2	35410
CNVR154	1873	14	120810562	120829324	gain	10224	93372131	137879539	Aspartate_amino_transferase_activity	region1inRegion2	18762
CNVR155	1880	14	127501895	127526173	gain	10224	93372131	137879539	Aspartate_amino_transferase_activity	region1inRegion2	24278
CNVR153	1849	14	94594494	94629904	gain	10225	93372131	137879539	Aspartate_amino_transferase_activity	region1inRegion2	35410
CNVR154	1873	14	120810562	120829324	gain	10225	93372131	137879539	Aspartate_amino_transferase_activity	region1inRegion2	18762
CNVR155	1880	14	127501895	127526173	gain	10225	93372131	137879539	Aspartate_amino_transferase_activity	region1inRegion2	24278
CNVR153	1849	14	94594494	94629904	gain	10226	93372131	137879539	Aspartate_amino_transferase_activity	region1inRegion2	35410
CNVR154	1873	14	120810562	120829324	gain	10226	93372131	137879539	Aspartate_amino_transferase_activity	region1inRegion2	18762
CNVR155	1880	14	127501895	127526173	gain	10226	93372131	137879539	Aspartate_amino_transferase_activity	region1inRegion2	24278
CNVR153	1849	14	94594494	94629904	gain	10227	93372131	137879539	Aspartate_amino_transferase_activity	region1inRegion2	35410
CNVR154	1873	14	120810562	120829324	gain	10227	93372131	137879539	Aspartate_amino_transferase_activity	region1inRegion2	18762
CNVR155	1880	14	127501895	127526173	gain	10227	93372131	137879539	Aspartate_amino_transferase_activity	region1inRegion2	24278
CNVR153	1849	14	94594494	94629904	gain	10228	93372131	137879539	Aspartate_amino_transferase_activity	region1inRegion2	35410
CNVR154	1873	14	120810562	120829324	gain	10228	93372131	137879539	Aspartate_amino_transferase_activity	region1inRegion2	18762
CNVR155	1880	14	127501895	127526173	gain	10228	93372131	137879539	Aspartate_amino_transferase_activity	region1inRegion2	24278
CNVR153	1849	14	94594494	94629904	gain	10229	93372131	137879539	Aspartate_amino_transferase_activity	region1inRegion2	35410
CNVR154	1873	14	120810562	120829324	gain	10229	93372131	137879539	Aspartate_amino_transferase_activity	region1inRegion2	18762
CNVR155	1880	14	127501895	127526173	gain	10229	93372131	137879539	Aspartate_amino_transferase_activity	region1inRegion2	24278
CNVR153	1849	14	94594494	94629904	gain	10230	93372131	137879539	Aspartate_amino_transferase_activity	region1inRegion2	35410
CNVR154	1873	14	120810562	120829324	gain	10230	93372131	137879539	Aspartate_amino_transferase_activity	region1inRegion2	18762

CNVR155	1880	14	127501895	127526173	gain	10230	93372131	137879539	Aspartate_amino_transferase_activity	region1inRegion2	24278
CNVR153	1849	14	94594494	94629904	gain	10231	93372131	137879539	Aspartate_amino_transferase_activity	region1inRegion2	35410
CNVR154	1873	14	120810562	120829324	gain	10231	93372131	137879539	Aspartate_amino_transferase_activity	region1inRegion2	18762
CNVR155	1880	14	127501895	127526173	gain	10231	93372131	137879539	Aspartate_amino_transferase_activity	region1inRegion2	24278
CNVR153	1849	14	94594494	94629904	gain	10232	93372131	137879539	Aspartate_amino_transferase_activity	region1inRegion2	35410
CNVR154	1873	14	120810562	120829324	gain	10232	93372131	137879539	Aspartate_amino_transferase_activity	region1inRegion2	18762
CNVR155	1880	14	127501895	127526173	gain	10232	93372131	137879539	Aspartate_amino_transferase_activity	region1inRegion2	24278
CNVR153	1849	14	94594494	94629904	gain	10233	93372131	137879539	Aspartate_amino_transferase_activity	region1inRegion2	35410
CNVR154	1873	14	120810562	120829324	gain	10233	93372131	137879539	Aspartate_amino_transferase_activity	region1inRegion2	18762
CNVR155	1880	14	127501895	127526173	gain	10233	93372131	137879539	Aspartate_amino_transferase_activity	region1inRegion2	24278
CNVR153	1849	14	94594494	94629904	gain	10234	93372131	137879539	Aspartate_amino_transferase_activity	region1inRegion2	35410
CNVR154	1873	14	120810562	120829324	gain	10234	93372131	137879539	Aspartate_amino_transferase_activity	region1inRegion2	18762
CNVR155	1880	14	127501895	127526173	gain	10234	93372131	137879539	Aspartate_amino_transferase_activity	region1inRegion2	24278
CNVR153	1849	14	94594494	94629904	gain	10235	93372131	137879539	Aspartate_amino_transferase_activity	region1inRegion2	35410
CNVR154	1873	14	120810562	120829324	gain	10235	93372131	137879539	Aspartate_amino_transferase_activity	region1inRegion2	18762
CNVR155	1880	14	127501895	127526173	gain	10235	93372131	137879539	Aspartate_amino_transferase_activity	region1inRegion2	24278
CNVR153	1849	14	94594494	94629904	gain	7516	93372131	137879539	Aspartate_amino_transferase_activity	region1inRegion2	35410
CNVR154	1873	14	120810562	120829324	gain	7516	93372131	137879539	Aspartate_amino_transferase_activity	region1inRegion2	18762
CNVR155	1880	14	127501895	127526173	gain	7516	93372131	137879539	Aspartate_amino_transferase_activity	region1inRegion2	24278
CNVR154	1873	14	120810562	120829324	gain	74	98726584	134652677	backfat_at_last_rib	region1inRegion2	18762
CNVR155	1880	14	127501895	127526173	gain	74	98726584	134652677	backfat_at_last_rib	region1inRegion2	24278
CNVR154	1873	14	120810562	120829324	gain	5132	102170112	135364437	Shoulder_weight	region1inRegion2	18762
CNVR155	1880	14	127501895	127526173	gain	5132	102170112	135364437	Shoulder_weight	region1inRegion2	24278
CNVR154	1873	14	120810562	120829324	gain	5145	102170112	135364437	Ham_meat_weight	region1inRegion2	18762
CNVR155	1880	14	127501895	127526173	gain	5145	102170112	135364437	Ham_meat_weight	region1inRegion2	24278
CNVR154	1873	14	120810562	120829324	gain	3971	108264282	137879539	Ham_fat_thickness	region1inRegion2	18762
CNVR155	1880	14	127501895	127526173	gain	3971	108264282	137879539	Ham_fat_thickness	region1inRegion2	24278
CNVR154	1873	14	120810562	120829324	gain	75	109966532	134904240	Carcass_weight_(cold)	region1inRegion2	18762
CNVR155	1880	14	127501895	127526173	gain	75	109966532	134904240	Carcass_weight_(cold)	region1inRegion2	24278
CNVR156	1968	15	82278814	82319824	gain	17765	2175824	157285085	pH_24_hr_post-mortem_(loin)	region1inRegion2	41010
CNVR157	1990	15	112634645	112649752	loss	17765	2175824	157285085	pH_24_hr_post-mortem_(loin)	region1inRegion2	15107
CNVR158	2026	15	156637663	156646003	gain	17765	2175824	157285085	pH_24_hr_post-mortem_(loin)	region1inRegion2	8340
CNVR156	1968	15	82278814	82319824	gain	17766	2175824	157285085	pH_24_hr_post_mortem_(ham)	region1inRegion2	41010
CNVR157	1990	15	112634645	112649752	loss	17766	2175824	157285085	pH_24_hr_post_mortem_(ham)	region1inRegion2	15107
CNVR158	2026	15	156637663	156646003	gain	17766	2175824	157285085	pH_24_hr_post_mortem_(ham)	region1inRegion2	8340
CNVR156	1968	15	82278814	82319824	gain	3976	5079243	157285085	Backfat_between_6th_and_7th_ribs	region1inRegion2	41010
CNVR157	1990	15	112634645	112649752	loss	3976	5079243	157285085	Backfat_between_6th_and_7th_ribs	region1inRegion2	15107
CNVR158	2026	15	156637663	156646003	gain	3976	5079243	157285085	Backfat_between_6th_and_7th_ribs	region1inRegion2	8340
CNVR156	1968	15	82278814	82319824	gain	5725	8000145	149797711	Backfat_at_tenth_rib	region1inRegion2	41010
CNVR157	1990	15	112634645	112649752	loss	5725	8000145	149797711	Backfat_at_tenth_rib	region1inRegion2	15107
CNVR156	1968	15	82278814	82319824	gain	38113	8000145	154567794	Shear_force	region1inRegion2	41010
CNVR157	1990	15	112634645	112649752	loss	38113	8000145	154567794	Shear_force	region1inRegion2	15107
CNVR156	1968	15	82278814	82319824	gain	24286	8483355	132040206	Corpus_luteum_number	region1inRegion2	41010
CNVR157	1990	15	112634645	112649752	loss	24286	8483355	132040206	Corpus_luteum_number	region1inRegion2	15107
CNVR156	1968	15	82278814	82319824	gain	21271	14384942	136254666	intestinal_fat_weight	region1inRegion2	41010
CNVR157	1990	15	112634645	112649752	loss	21271	14384942	136254666	intestinal_fat_weight	region1inRegion2	15107
CNVR156	1968	15	82278814	82319824	gain	5405	25021683	112762745	Red_blood_cell_count	region1inRegion2	41010
CNVR157	1990	15	112634645	112649752	loss	5405	25021683	112762745	Red_blood_cell_count	region1inRegion2	15107
CNVR156	1968	15	82278814	82319824	gain	15081	28179622	154567794	Creatinine_level	region1inRegion2	41010
CNVR157	1990	15	112634645	112649752	loss	15081	28179622	154567794	Creatinine_level	region1inRegion2	15107
CNVR156	1968	15	82278814	82319824	gain	123	33137272	115803475	pH_24_hr_post-mortem_(loin)	region1inRegion2	41010
CNVR157	1990	15	112634645	112649752	loss	123	33137272	115803475	pH_24_hr_post-mortem_(loin)	region1inRegion2	15107
CNVR156	1968	15	82278814	82319824	gain	12094	33137272	135157314	Meat_color_OPTO	region1inRegion2	41010
CNVR157	1990	15	112634645	112649752	loss	12094	33137272	135157314	Meat_color_OPTO	region1inRegion2	15107
CNVR156	1968	15	82278814	82319824	gain	12095	33137272	135157314	Conductivity_24_hours_postmortem_(loin)	region1inRegion2	41010
CNVR157	1990	15	112634645	112649752	loss	12095	33137272	135157314	Conductivity_24_hours_postmortem_(loin)	region1inRegion2	15107
CNVR156	1968	15	82278814	82319824	gain	12096	33137272	135157314	Lean_meat_percentage	region1inRegion2	41010
CNVR157	1990	15	112634645	112649752	loss	12096	33137272	135157314	Lean_meat_percentage	region1inRegion2	15107
CNVR156	1968	15	82278814	82319824	gain	12097	33137272	135157314	Loin_muscle_area	region1inRegion2	41010
CNVR157	1990	15	112634645	112649752	loss	12097	33137272	135157314	Loin_muscle_area	region1inRegion2	15107
CNVR156	1968	15	82278814	82319824	gain	2829	33137272	135157314	Total_muscle_fiber_number	region1inRegion2	41010
CNVR157	1990	15	112634645	112649752	loss	2829	33137272	135157314	Total_muscle_fiber_number	region1inRegion2	15107
CNVR156	1968	15	82278814	82319824	gain	2830	33137272	135157314	Percentage_angular_fibers	region1inRegion2	41010
CNVR157	1990	15	112634645	112649752	loss	2830	33137272	135157314	Percentage_angular_fibers	region1inRegion2	15107
CNVR156	1968	15	82278814	82319824	gain	2831	33137272	135157314	Diameter_of_giant_fibers	region1inRegion2	41010
CNVR157	1990	15	112634645	112649752	loss	2831	33137272	135157314	Diameter_of_giant_fibers	region1inRegion2	15107
CNVR156	1968	15	82278814	82319824	gain	2832	33137272	135157314	Diameter_of_type_I_muscle_fibers	region1inRegion2	41010
CNVR157	1990	15	112634645	112649752	loss	2832	33137272	135157314	Diameter_of_type_I_muscle_fibers	region1inRegion2	15107
CNVR156	1968	15	82278814	82319824	gain	2833	33137272	135157314	Diameter_of_type_IIa_muscle_fibers	region1inRegion2	41010
CNVR157	1990	15	112634645	112649752	loss	2833	33137272	135157314	Diameter_of_type_IIa_muscle_fibers	region1inRegion2	15107
CNVR156	1968	15	82278814	82319824	gain	5724	33137272	135157314	Average_backfat_thickness	region1inRegion2	41010
CNVR157	1990	15	112634645	112649752	loss	5724	33137272	135157314	Average_backfat_thickness	region1inRegion2	15107
CNVR156	1968	15	82278814	82319824	gain	5952	33137272	135157314	Body_weight_(weaning)	region1inRegion2	41010
CNVR157	1990	15	112634645	112649752	loss	5952	33137272	135157314	Body_weight_(weaning)	region1inRegion2	15107
CNVR156	1968	15	82278814	82319824	gain	5992	33137272	135157314	Backfat_at_tenth_rib	region1inRegion2	41010
CNVR157	1990	15	112634645	112649752	loss	5992	33137272	135157314	Backfat_at_tenth_rib	region1inRegion2	15107
CNVR156	1968	15	82278814	82319824	gain	6013	33137272	135157314	pH_for_Semimembranosus	region1inRegion2	41010
CNVR157	1990	15	112634645	112649752	loss	6013	33137272	135157314	pH_for_Semimembranosus	region1inRegion2	15107
CNVR156	1968	15	82278814	82319824	gain	5723	33137272	149797711	pH_for_Semimembranosus	region1inRegion2	41010
CNVR157	1990	15	112634645	112649752	loss	5723	33137272	149797711	pH_for_Semimembranosus	region1inRegion2	15107
CNVR156	1968	15	82278814	82319824	gain	7468	33137272	149797711	Nonfunctional_nipples	region1inRegion2	41010
CNVR157	1990	15	112634645	112649752	loss	7468	33137272	149797711	Nonfunctional_nipples	region1inRegion2	15107
CNVR156	1968	15	82278814	82319824	gain	21719	35772697	88894402	Loin_muscle_area	region1inRegion2	41010
CNVR156	1968	15	82278814	82319824	gain	21720	35772697	88894402	tenderness_score	region1inRegion2	41010
CNVR156	1968	15	82278814	82319824	gain	11586	37332503	149409685	Salmonella_count_in_liver_and_spleen	region1inRegion2	41010
CNVR157	1990	15	112634645	112649752	loss	11586	37332503	149409685	Salmonella_count_in_liver_and_spleen	region1inRegion2	15107
CNVR156	1968	15	82278814	82319824	gain	11587	37332503	149409685	Salmonella_count_in_spleen	region1inRegion2	41010
CNVR157	1990	15	112634645	112649752	loss	11587	37332503	149409685	Salmonella_count_in_spleen	region1inRegion2	15107
CNVR156	1968	15	82278814	82319824	gain	2964	43065120	99227812	pH_48_hr_post-mortem_(loin)	region1inRegion2	41010
CNVR156	1968	15	82278814	82319824	gain	3014	43065120	105746635	Drip_loss	region1inRegion2	41010
CNVR156	1968	15	82278814	82319824	gain	3015	43065120	105746635	Shear_force	region1inRegion2	41010
CNVR156	1968	15	82278814	82319824	gain	3827	43065120	140400767	tenderness_score	region1inRegion2	41010
CNVR157	1990	15	112634645	112649752	loss	3827	43065120	140400767	tenderness_score	region1inRegion2	15107
CNVR156	1968	15	82278814	82319824	gain	3915	46030500	149797711	Lean_meat_percentage	region1inRegion2	41010
CNVR157	1990	15	112634645	112649752	loss	3915	46030500	149797711	Lean_meat_percentage	region1inRegion2	15107
CNVR156	1968	15	82278814	82319824	gain	3916	46030500	149797711	Lean_meat_percentage	region1inRegion2	41010
CNVR157	1990	15	112634645	112649752	loss	3916	46030500	149797711	Lean_meat_percentage	region1inRegion2	15107
CNVR156	1968	15	82278814	82319824	gain	3975	46030500	149797711	Backfat_at_first_rib	region1inRegion2	41010
CNVR157	1990	15	112634645	112649752	loss	3975	46030500	149797711	Backfat_at_first_rib	region1inRegion2	15107
CNVR156	1968	15	82278814	82319824	gain	519	53604367	140194479	Corpus_luteum_number	region1inRegion2	41010
CNVR157	1990	15	112634645	112649752	loss	519	53604367	140194479	Corpus_luteum_number	region1inRegion2	15107
CNVR156	1968	15	82278814	82319824	gain	17855	57165536	127880313	CD4-positive/CD8-positive_leukocyte_ratio	region1inRegion2	41010
CNVR157	1990	15	112634645	112649752	loss	17855	57165536	127880313	CD4-positive/CD8-positive_leukocyte_ratio	region1inRegion2	15107
CNVR156	1968	15	82278814	82319824	gain	21270	57492424	120124351	Shoulder_subcutaneous_fat_thickness	region1inRegion2	41010
CNVR157	1990	15	112634645	112649752	loss	21270	57492424	120124351	Shoulder_subcutaneous_fat_thickness	region1inRegion2	15107
CNVR156	1968	15	82278814	82319824	gain	21721	60370996	115414717	CIE-a*	region1inRegion2	41010
CNVR157	1990	15	112634645	112649752	loss	21721	60370996	115414717	CIE-a*	region1inRegion2	15107
CNVR156	1968	15	82278814	82319824	gain	21723	60370996	115414717	CIE-L*	region1inRegion2	41010
CNVR157	1990	15	112634645	112649752	loss	21723	60370996	115414717	CIE-L*	region1inRegion2	15107
CNVR156	1968	15	82278814	82319824	gain	122	62954050	145436197	pH_24_hr_post-mortem_(loin)	region1inRegion2	41010
CNVR157	1990	15	112634645	112649752	loss	122	62954050	145436197	pH_24_hr_post-mortem_(loin)	region1inRegion2	15107
CNVR156	1968	15	82278814	82319824	gain	293	62954050	149797711	pH_24_hr_post-mortem_(loin)	region1inRegion2	41010
CNVR157	1990	15	112634645	112649752	loss	293	62954050	149797711	pH_24_hr_post-mortem_(loin)	region1inRegion2	15107
CNVR156	1968	15	82278814	82319824	gain	31812	65326564	133342560	Skin_thickness	region1inRegion2	41010
CNVR157	1990	15	112634645	112649752	loss	31812	65326564	133342560	Skin_thickness	region1inRegion2	15107
CNVR156	1968	15	82278814	82319824	gain	3974	66799112	99227812	Semimembranosus_angle	region1inRegion2	41010
CNVR156	1968	15	82278814	82319824	gain	4043	67847422	115213308	Intramuscular_fat_content	region1inRegion2	41010
CNVR157	1990	15	112634645	112649752	loss	4043	67847422	115213308	Intramuscular_fat_content	region1inRegion2	15107
CNVR156	1968	15	82278814	82319824	gain	16938	71973307	127880313	Loin_muscle_area	region1inRegion2	41010
CNVR157	1990	15	112634645	112649752	loss	16938	71973307	127880313	Loin_muscle_area	region1inRegion2	15107
CNVR156	1968	15	82278814	82319824	gain	21407	71973307	127880313	Loin_weight	region1inRegion2	41010
CNVR157	1990	15	112634645	112649752	loss	21407	71973307	127880313	Loin_weight	region1inRegion2	15107

CNVR156	1968	15	82278814	82319824	gain	21408	71973307	127880313	Backfat_at_first_rib	region1inRegion2	41010
CNVR157	1990	15	112634645	112649752	loss	21408	71973307	127880313	Backfat_at_first_rib	region1inRegion2	15107
CNVR156	1968	15	82278814	82319824	gain	21409	71973307	127880313	Backfat_at_tenth_rib	region1inRegion2	41010
CNVR157	1990	15	112634645	112649752	loss	21409	71973307	127880313	Backfat_at_tenth_rib	region1inRegion2	15107
CNVR156	1968	15	82278814	82319824	gain	3291	71973307	127880313	muscle_protein_percentage	region1inRegion2	41010
CNVR157	1990	15	112634645	112649752	loss	3291	71973307	127880313	muscle_protein_percentage	region1inRegion2	15107
CNVR156	1968	15	82278814	82319824	gain	3292	71973307	127880313	Meat_color-a	region1inRegion2	41010
CNVR157	1990	15	112634645	112649752	loss	3292	71973307	127880313	Meat_color-a	region1inRegion2	15107
CNVR156	1968	15	82278814	82319824	gain	3293	71973307	127880313	Meat_color_score	region1inRegion2	41010
CNVR157	1990	15	112634645	112649752	loss	3293	71973307	127880313	Meat_color_score	region1inRegion2	15107
CNVR156	1968	15	82278814	82319824	gain	3311	71973307	127880313	Meat_color-L	region1inRegion2	41010
CNVR157	1990	15	112634645	112649752	loss	3311	71973307	127880313	Meat_color-L	region1inRegion2	15107
CNVR156	1968	15	82278814	82319824	gain	237	71973307	135869562	Meat_color-a	region1inRegion2	41010
CNVR157	1990	15	112634645	112649752	loss	237	71973307	135869562	Meat_color-a	region1inRegion2	15107
CNVR156	1968	15	82278814	82319824	gain	16857	71973307	140400767	Average_daily_gain	region1inRegion2	41010
CNVR157	1990	15	112634645	112649752	loss	16857	71973307	140400767	Average_daily_gain	region1inRegion2	15107
CNVR156	1968	15	82278814	82319824	gain	16892	71973307	140400767	Average_daily_gain	region1inRegion2	41010
CNVR157	1990	15	112634645	112649752	loss	16892	71973307	140400767	Average_daily_gain	region1inRegion2	15107
CNVR157	1990	15	112634645	112649752	loss	290	88544663	140400767	Average_glycogen	region1inRegion2	15107
CNVR157	1990	15	112634645	112649752	loss	21269	90652605	144378710	Abdominal_fat_weight	region1inRegion2	15107
CNVR157	1990	15	112634645	112649752	loss	4042	92056199	129801145	pH_24_hr_post-mortem_(loin)	region1inRegion2	15107
CNVR157	1990	15	112634645	112649752	loss	4044	94979647	133676432	Intramuscular_fat_content	region1inRegion2	15107
CNVR157	1990	15	112634645	112649752	loss	4040	95004429	129801145	CIE-b*	region1inRegion2	15107
CNVR157	1990	15	112634645	112649752	loss	3790	99227812	127880313	Drip_loss	region1inRegion2	15107
CNVR157	1990	15	112634645	112649752	loss	3811	99227812	127880313	Off-Flavor_Score	region1inRegion2	15107
CNVR157	1990	15	112634645	112649752	loss	120	109367034	137874067	Meat_color-L	region1inRegion2	15107
CNVR157	1990	15	112634645	112649752	loss	21722	112594435	132343352	muscle_protein_percentage	region1inRegion2	15107
CNVR158	2026	15	156637663	156646003	gain	15127	136692942	157681621	Phosphate_level	region1inRegion2	8340
CNVR158	2026	15	156637663	156646003	gain	38111	154567794	157285085	Loin_muscle_area	region1inRegion2	8340
CNVR158	2026	15	156637663	156646003	gain	38112	154567794	157285085	Loin_muscle_area	region1inRegion2	8340
CNVR159	2034	16	6146177	6154424	loss	12532	342954	14462278	Bacon_depth	region1inRegion2	8247
CNVR160	2055	16	12229596	12241067	loss-gain	12532	342954	14462278	Bacon_depth	region1inRegion2	11471
CNVR159	2034	16	6146177	6154424	loss	7537	342954	18970761	Total_number_born_alive	region1inRegion2	8247
CNVR160	2055	16	12229596	12241067	loss-gain	7537	342954	18970761	Total_number_born_alive	region1inRegion2	11471
CNVR159	2034	16	6146177	6154424	loss	8937	342954	20168075	Hind_leg_conformation	region1inRegion2	8247
CNVR160	2055	16	12229596	12241067	loss-gain	8937	342954	20168075	Hind_leg_conformation	region1inRegion2	11471
CNVR159	2034	16	6146177	6154424	loss	21849	342954	22335189	Drip_loss	region1inRegion2	8247
CNVR160	2055	16	12229596	12241067	loss-gain	21849	342954	22335189	Drip_loss	region1inRegion2	11471
CNVR159	2034	16	6146177	6154424	loss	16874	342954	27084747	Carcass_weight_(hot)	region1inRegion2	8247
CNVR160	2055	16	12229596	12241067	loss-gain	16874	342954	27084747	Carcass_weight_(hot)	region1inRegion2	11471
CNVR159	2034	16	6146177	6154424	loss	16900	342954	27084747	Carcass_weight_(hot)	region1inRegion2	8247
CNVR160	2055	16	12229596	12241067	loss-gain	16900	342954	27084747	Carcass_weight_(hot)	region1inRegion2	11471
CNVR159	2034	16	6146177	6154424	loss	12533	342954	31276215	Backfat_at_last_lumbar	region1inRegion2	8247
CNVR160	2055	16	12229596	12241067	loss-gain	12533	342954	31276215	Backfat_at_last_lumbar	region1inRegion2	11471
CNVR161	2084	16	30196586	30215798	loss	12533	342954	31276215	Backfat_at_last_lumbar	region1inRegion2	19212
CNVR161	2084	16	30196586	30217974	loss	12533	342954	31276215	Backfat_at_last_lumbar	region1inRegion2	21388
CNVR159	2034	16	6146177	6154424	loss	12537	342954	42857275	Meat_color-a	region1inRegion2	8247
CNVR160	2055	16	12229596	12241067	loss-gain	12537	342954	42857275	Meat_color-a	region1inRegion2	11471
CNVR161	2084	16	30196586	30215798	loss	12537	342954	42857275	Meat_color-a	region1inRegion2	19212
CNVR161	2084	16	30196586	30217974	loss	12537	342954	42857275	Meat_color-a	region1inRegion2	21388
CNVR159	2034	16	6146177	6154424	loss	12535	342954	49483862	Lung_weight	region1inRegion2	8247
CNVR160	2055	16	12229596	12241067	loss-gain	12535	342954	49483862	Lung_weight	region1inRegion2	11471
CNVR161	2084	16	30196586	30215798	loss	12535	342954	49483862	Lung_weight	region1inRegion2	19212
CNVR161	2084	16	30196586	30217974	loss	12535	342954	49483862	Lung_weight	region1inRegion2	21388
CNVR159	2034	16	6146177	6154424	loss	12536	342954	67347086	Cooking_loss	region1inRegion2	8247
CNVR160	2055	16	12229596	12241067	loss-gain	12536	342954	67347086	Cooking_loss	region1inRegion2	11471
CNVR161	2084	16	30196586	30215798	loss	12536	342954	67347086	Cooking_loss	region1inRegion2	19212
CNVR161	2084	16	30196586	30217974	loss	12536	342954	67347086	Cooking_loss	region1inRegion2	21388
CNVR163	2109	16	53083153	53202189	gain	12536	342954	67347086	Cooking_loss	region1inRegion2	119036
CNVR164	2111	16	56657347	56707690	loss	12536	342954	67347086	Cooking_loss	region1inRegion2	50343
CNVR159	2034	16	6146177	6154424	loss	17627	342954	67649164	Toll-like_receptor_9_level	region1inRegion2	8247
CNVR160	2055	16	12229596	12241067	loss-gain	17627	342954	67649164	Toll-like_receptor_9_level	region1inRegion2	11471
CNVR161	2084	16	30196586	30215798	loss	17627	342954	67649164	Toll-like_receptor_9_level	region1inRegion2	19212
CNVR161	2084	16	30196586	30217974	loss	17627	342954	67649164	Toll-like_receptor_9_level	region1inRegion2	21388
CNVR163	2109	16	53083153	53202189	gain	17627	342954	67649164	Toll-like_receptor_9_level	region1inRegion2	119036
CNVR164	2111	16	56657347	56707690	loss	17627	342954	67649164	Toll-like_receptor_9_level	region1inRegion2	50343
CNVR159	2034	16	6146177	6154424	loss	17727	342954	67649164	HDL/LDL_ratio	region1inRegion2	8247
CNVR160	2055	16	12229596	12241067	loss-gain	17727	342954	67649164	HDL/LDL_ratio	region1inRegion2	11471
CNVR161	2084	16	30196586	30215798	loss	17727	342954	67649164	HDL/LDL_ratio	region1inRegion2	19212
CNVR161	2084	16	30196586	30217974	loss	17727	342954	67649164	HDL/LDL_ratio	region1inRegion2	21388
CNVR163	2109	16	53083153	53202189	gain	17727	342954	67649164	HDL/LDL_ratio	region1inRegion2	119036
CNVR164	2111	16	56657347	56707690	loss	17727	342954	67649164	HDL/LDL_ratio	region1inRegion2	50343
CNVR159	2034	16	6146177	6154424	loss	3926	342954	67649164	Conductivity_24_hours_postmortem_(ham)	region1inRegion2	8247
CNVR160	2055	16	12229596	12241067	loss-gain	3926	342954	67649164	Conductivity_24_hours_postmortem_(ham)	region1inRegion2	11471
CNVR161	2084	16	30196586	30215798	loss	3926	342954	67649164	Conductivity_24_hours_postmortem_(ham)	region1inRegion2	19212
CNVR161	2084	16	30196586	30217974	loss	3926	342954	67649164	Conductivity_24_hours_postmortem_(ham)	region1inRegion2	21388
CNVR163	2109	16	53083153	53202189	gain	3926	342954	67649164	Conductivity_24_hours_postmortem_(ham)	region1inRegion2	119036
CNVR164	2111	16	56657347	56707690	loss	3926	342954	67649164	Conductivity_24_hours_postmortem_(ham)	region1inRegion2	50343
CNVR159	2034	16	6146177	6154424	loss	3927	342954	67649164	Conductivity_24_hours_postmortem_(ham)	region1inRegion2	8247
CNVR160	2055	16	12229596	12241067	loss-gain	3927	342954	67649164	Conductivity_24_hours_postmortem_(ham)	region1inRegion2	11471
CNVR161	2084	16	30196586	30215798	loss	3927	342954	67649164	Conductivity_24_hours_postmortem_(ham)	region1inRegion2	19212
CNVR161	2084	16	30196586	30217974	loss	3927	342954	67649164	Conductivity_24_hours_postmortem_(ham)	region1inRegion2	21388
CNVR163	2109	16	53083153	53202189	gain	3927	342954	67649164	Conductivity_24_hours_postmortem_(ham)	region1inRegion2	119036
CNVR164	2111	16	56657347	56707690	loss	3927	342954	67649164	Conductivity_24_hours_postmortem_(ham)	region1inRegion2	50343
CNVR159	2034	16	6146177	6154424	loss	3928	342954	67649164	Conductivity_24_hours_postmortem_(ham)	region1inRegion2	8247
CNVR160	2055	16	12229596	12241067	loss-gain	3928	342954	67649164	Conductivity_24_hours_postmortem_(ham)	region1inRegion2	11471
CNVR161	2084	16	30196586	30215798	loss	3928	342954	67649164	Conductivity_24_hours_postmortem_(ham)	region1inRegion2	19212
CNVR161	2084	16	30196586	30217974	loss	3928	342954	67649164	Conductivity_24_hours_postmortem_(ham)	region1inRegion2	21388
CNVR163	2109	16	53083153	53202189	gain	3928	342954	67649164	Conductivity_24_hours_postmortem_(ham)	region1inRegion2	119036
CNVR164	2111	16	56657347	56707690	loss	3928	342954	67649164	Conductivity_24_hours_postmortem_(ham)	region1inRegion2	50343
CNVR159	2034	16	6146177	6154424	loss	5732	342954	67649164	Feed_conversion_ratio	region1inRegion2	8247
CNVR160	2055	16	12229596	12241067	loss-gain	5732	342954	67649164	Feed_conversion_ratio	region1inRegion2	11471
CNVR161	2084	16	30196586	30215798	loss	5732	342954	67649164	Feed_conversion_ratio	region1inRegion2	19212
CNVR161	2084	16	30196586	30217974	loss	5732	342954	67649164	Feed_conversion_ratio	region1inRegion2	21388
CNVR163	2109	16	53083153	53202189	gain	5732	342954	67649164	Feed_conversion_ratio	region1inRegion2	119036
CNVR164	2111	16	56657347	56707690	loss	5732	342954	67649164	Feed_conversion_ratio	region1inRegion2	50343
CNVR159	2034	16	6146177	6154424	loss	5953	342954	67649164	Daily_feed_intake	region1inRegion2	8247
CNVR160	2055	16	12229596	12241067	loss-gain	5953	342954	67649164	Daily_feed_intake	region1inRegion2	11471
CNVR161	2084	16	30196586	30215798	loss	5953	342954	67649164	Daily_feed_intake	region1inRegion2	19212
CNVR161	2084	16	30196586	30217974	loss	5953	342954	67649164	Daily_feed_intake	region1inRegion2	21388
CNVR163	2109	16	53083153	53202189	gain	5953	342954	67649164	Daily_feed_intake	region1inRegion2	119036
CNVR164	2111	16	56657347	56707690	loss	5953	342954	67649164	Daily_feed_intake	region1inRegion2	50343
CNVR159	2034	16	6146177	6154424	loss	5994	342954	67649164	backfat_above_muscle_dorsi	region1inRegion2	8247
CNVR160	2055	16	12229596	12241067	loss-gain	5994	342954	67649164	backfat_above_muscle_dorsi	region1inRegion2	11471
CNVR161	2084	16	30196586	30215798	loss	5994	342954	67649164	backfat_above_muscle_dorsi	region1inRegion2	19212
CNVR161	2084	16	30196586	30217974	loss	5994	342954	67649164	backfat_above_muscle_dorsi	region1inRegion2	21388
CNVR163	2109	16	53083153	53202189	gain	5994	342954	67649164	backfat_above_muscle_dorsi	region1inRegion2	119036
CNVR164	2111	16	56657347	56707690	loss	5994	342954	67649164	backfat_above_muscle_dorsi	region1inRegion2	50343
CNVR159	2034	16	6146177	6154424	loss	5995	342954	67649164	Shoulder_subcutaneous_fat_thickness	region1inRegion2	8247
CNVR160	2055	16	12229596	12241067	loss-gain	5995	342954	67649164	Shoulder_subcutaneous_fat_thickness	region1inRegion2	11471
CNVR161	2084	16	30196586	30215798	loss	5995	342954	67649164	Shoulder_subcutaneous_fat_thickness	region1inRegion2	19212
CNVR161	2084	16	30196586	30217974	loss	5995	342954	67649164	Shoulder_subcutaneous_fat_thickness	region1inRegion2	21388
CNVR163	2109	16	53083153	53202189	gain	5995	342954	67649164	Shoulder_subcutaneous_fat_thickness	region1inRegion2	119036
CNVR164	2111	16	56657347	56707690	loss	5995	342954	67649164	Shoulder_subcutaneous_fat_thickness	region1inRegion2	50343
CNVR159	2034	16	6146177	6154424	loss	5996	342954	67649164	Belly_meat_content	region1inRegion2	8247
CNVR160	2055	16	12229596	12241067	loss-gain	5996	342954	67649164	Belly_meat_content	region1inRegion2	11471
CNVR161	2084	16	30196586	30215798	loss	5996	342954	67649164	Belly_meat_content	region1inRegion2	19212
CNVR161	2084	16	30196586	30217974	loss	5996	342954	67649164	Belly_meat_content	region1inRegion2	21388
CNVR163	2109	16	53083153	53202189	gain	5996	342954	67649164	Belly_meat_content	region1inRegion2	119036
CNVR164	2111	16	56657347	56707690	loss	5996	342954	67649164	Belly_meat_content	region1inRegion2	50343
CNVR159	2034	16	6146177	6154424	loss	5997	342954	67649164	Estimated_carcass_lean_content	region1inRegion2	8247
CNVR160	2055	16	12229596	12241067	loss-gain	5997	342954	67649164	Estimated_carcass_lean_content	region1inRegion2	11471

CNVR161	2084	16	30196586	30215798	loss	5997	342954	67649164	Estimated_carcass_lean_content	region1inRegion2	19212
CNVR161	2084	16	30196586	30217974	loss	5997	342954	67649164	Estimated_carcass_lean_content	region1inRegion2	21388
CNVR163	2109	16	53083153	53202189	gain	5997	342954	67649164	Estimated_carcass_lean_content	region1inRegion2	119036
CNVR164	2111	16	56657347	56707690	loss	5997	342954	67649164	Estimated_carcass_lean_content	region1inRegion2	50343
CNVR159	2034	16	6146177	6154424	loss	5999	342954	67649164	Subcutanous_fat_area	region1inRegion2	8247
CNVR160	2055	16	12229596	12241067	loss-gain	5999	342954	67649164	Subcutanous_fat_area	region1inRegion2	11471
CNVR161	2084	16	30196586	30215798	loss	5999	342954	67649164	Subcutanous_fat_area	region1inRegion2	19212
CNVR161	2084	16	30196586	30217974	loss	5999	342954	67649164	Subcutanous_fat_area	region1inRegion2	21388
CNVR163	2109	16	53083153	53202189	gain	5999	342954	67649164	Subcutanous_fat_area	region1inRegion2	119036
CNVR164	2111	16	56657347	56707690	loss	5999	342954	67649164	Subcutanous_fat_area	region1inRegion2	50343
CNVR159	2034	16	6146177	6154424	loss	6014	342954	67649164	Cooking_loss	region1inRegion2	8247
CNVR160	2055	16	12229596	12241067	loss-gain	6014	342954	67649164	Cooking_loss	region1inRegion2	11471
CNVR161	2084	16	30196586	30215798	loss	6014	342954	67649164	Cooking_loss	region1inRegion2	19212
CNVR161	2084	16	30196586	30217974	loss	6014	342954	67649164	Cooking_loss	region1inRegion2	21388
CNVR163	2109	16	53083153	53202189	gain	6014	342954	67649164	Cooking_loss	region1inRegion2	119036
CNVR164	2111	16	56657347	56707690	loss	6014	342954	67649164	Cooking_loss	region1inRegion2	50343
CNVR159	2034	16	6146177	6154424	loss	6015	342954	67649164	Shear_force	region1inRegion2	8247
CNVR160	2055	16	12229596	12241067	loss-gain	6015	342954	67649164	Shear_force	region1inRegion2	11471
CNVR161	2084	16	30196586	30215798	loss	6015	342954	67649164	Shear_force	region1inRegion2	19212
CNVR161	2084	16	30196586	30217974	loss	6015	342954	67649164	Shear_force	region1inRegion2	21388
CNVR163	2109	16	53083153	53202189	gain	6015	342954	67649164	Shear_force	region1inRegion2	119036
CNVR164	2111	16	56657347	56707690	loss	6015	342954	67649164	Shear_force	region1inRegion2	50343
CNVR159	2034	16	6146177	6154424	loss	9633	342954	67649164	C3c_concentration	region1inRegion2	8247
CNVR160	2055	16	12229596	12241067	loss-gain	9633	342954	67649164	C3c_concentration	region1inRegion2	11471
CNVR161	2084	16	30196586	30215798	loss	9633	342954	67649164	C3c_concentration	region1inRegion2	19212
CNVR161	2084	16	30196586	30217974	loss	9633	342954	67649164	C3c_concentration	region1inRegion2	21388
CNVR163	2109	16	53083153	53202189	gain	9633	342954	67649164	C3c_concentration	region1inRegion2	119036
CNVR164	2111	16	56657347	56707690	loss	9633	342954	67649164	C3c_concentration	region1inRegion2	50343
CNVR159	2034	16	6146177	6154424	loss	9634	342954	67649164	C3c_concentration	region1inRegion2	8247
CNVR160	2055	16	12229596	12241067	loss-gain	9634	342954	67649164	C3c_concentration	region1inRegion2	11471
CNVR161	2084	16	30196586	30215798	loss	9634	342954	67649164	C3c_concentration	region1inRegion2	19212
CNVR161	2084	16	30196586	30217974	loss	9634	342954	67649164	C3c_concentration	region1inRegion2	21388
CNVR163	2109	16	53083153	53202189	gain	9634	342954	67649164	C3c_concentration	region1inRegion2	119036
CNVR164	2111	16	56657347	56707690	loss	9634	342954	67649164	C3c_concentration	region1inRegion2	50343
CNVR159	2034	16	6146177	6154424	loss	12534	342954	75493842	Heart_weight	region1inRegion2	8247
CNVR160	2055	16	12229596	12241067	loss-gain	12534	342954	75493842	Heart_weight	region1inRegion2	11471
CNVR161	2084	16	30196586	30215798	loss	12534	342954	75493842	Heart_weight	region1inRegion2	19212
CNVR161	2084	16	30196586	30217974	loss	12534	342954	75493842	Heart_weight	region1inRegion2	21388
CNVR163	2109	16	53083153	53202189	gain	12534	342954	75493842	Heart_weight	region1inRegion2	119036
CNVR164	2111	16	56657347	56707690	loss	12534	342954	75493842	Heart_weight	region1inRegion2	50343
CNVR159	2034	16	6146177	6154424	loss	37571	342954	78549461	Actinobacillus_pleuropneumoniae_susceptibility	region1inRegion2	8247
CNVR160	2055	16	12229596	12241067	loss-gain	37571	342954	78549461	Actinobacillus_pleuropneumoniae_susceptibility	region1inRegion2	11471
CNVR161	2084	16	30196586	30215798	loss	37571	342954	78549461	Actinobacillus_pleuropneumoniae_susceptibility	region1inRegion2	19212
CNVR161	2084	16	30196586	30217974	loss	37571	342954	78549461	Actinobacillus_pleuropneumoniae_susceptibility	region1inRegion2	21388
CNVR163	2109	16	53083153	53202189	gain	37571	342954	78549461	Actinobacillus_pleuropneumoniae_susceptibility	region1inRegion2	119036
CNVR164	2111	16	56657347	56707690	loss	37571	342954	78549461	Actinobacillus_pleuropneumoniae_susceptibility	region1inRegion2	50343
CNVR159	2034	16	6146177	6154424	loss	5228	342954	79624164	Teat_number	region1inRegion2	8247
CNVR160	2055	16	12229596	12241067	loss-gain	5228	342954	79624164	Teat_number	region1inRegion2	11471
CNVR161	2084	16	30196586	30215798	loss	5228	342954	79624164	Teat_number	region1inRegion2	19212
CNVR161	2084	16	30196586	30217974	loss	5228	342954	79624164	Teat_number	region1inRegion2	21388
CNVR163	2109	16	53083153	53202189	gain	5228	342954	79624164	Teat_number	region1inRegion2	119036
CNVR164	2111	16	56657347	56707690	loss	5228	342954	79624164	Teat_number	region1inRegion2	50343
CNVR159	2034	16	6146177	6154424	loss	38117	342954	80266973	Meat_color_chroma	region1inRegion2	8247
CNVR160	2055	16	12229596	12241067	loss-gain	38117	342954	80266973	Meat_color_chroma	region1inRegion2	11471
CNVR161	2084	16	30196586	30215798	loss	38117	342954	80266973	Meat_color_chroma	region1inRegion2	19212
CNVR161	2084	16	30196586	30217974	loss	38117	342954	80266973	Meat_color_chroma	region1inRegion2	21388
CNVR163	2109	16	53083153	53202189	gain	38117	342954	80266973	Meat_color_chroma	region1inRegion2	119036
CNVR164	2111	16	56657347	56707690	loss	38117	342954	80266973	Meat_color_chroma	region1inRegion2	50343
CNVR159	2034	16	6146177	6154424	loss	5198	342954	80266973	Body_weight_(birth)	region1inRegion2	8247
CNVR160	2055	16	12229596	12241067	loss-gain	5198	342954	80266973	Body_weight_(birth)	region1inRegion2	11471
CNVR161	2084	16	30196586	30215798	loss	5198	342954	80266973	Body_weight_(birth)	region1inRegion2	19212
CNVR161	2084	16	30196586	30217974	loss	5198	342954	80266973	Body_weight_(birth)	region1inRegion2	21388
CNVR163	2109	16	53083153	53202189	gain	5198	342954	80266973	Body_weight_(birth)	region1inRegion2	119036
CNVR164	2111	16	56657347	56707690	loss	5198	342954	80266973	Body_weight_(birth)	region1inRegion2	50343
CNVR159	2034	16	6146177	6154424	loss	12328	342954	80539062	Change_in_Mycoplasma_hyopneumoniae_antibody_titer	region1inRegion2	8247
CNVR160	2055	16	12229596	12241067	loss-gain	12328	342954	80539062	Change_in_Mycoplasma_hyopneumoniae_antibody_titer	region1inRegion2	11471
CNVR161	2084	16	30196586	30215798	loss	12328	342954	80539062	Change_in_Mycoplasma_hyopneumoniae_antibody_titer	region1inRegion2	19212
CNVR161	2084	16	30196586	30217974	loss	12328	342954	80539062	Change_in_Mycoplasma_hyopneumoniae_antibody_titer	region1inRegion2	21388
CNVR163	2109	16	53083153	53202189	gain	12328	342954	80539062	Change_in_Mycoplasma_hyopneumoniae_antibody_titer	region1inRegion2	119036
CNVR164	2111	16	56657347	56707690	loss	12328	342954	80539062	Change_in_Mycoplasma_hyopneumoniae_antibody_titer	region1inRegion2	50343
CNVR159	2034	16	6146177	6154424	loss	7647	4721435	6571190	Gluteus_medius_depth	region1inRegion2	8247
CNVR159	2034	16	6146177	6154424	loss	107244	5599229	6576317	hematocrit	region1inRegion2	8247
CNVR159	2034	16	6146177	6154424	loss	107323	5599229	6600720	Red_blood_cell_count	region1inRegion2	8247
CNVR159	2034	16	6146177	6154424	loss	17644	5861594	79725345	Front_feet_conformation	region1inRegion2	8247
CNVR160	2055	16	12229596	12241067	loss-gain	17644	5861594	79725345	Front_feet_conformation	region1inRegion2	11471
CNVR161	2084	16	30196586	30215798	loss	17644	5861594	79725345	Front_feet_conformation	region1inRegion2	19212
CNVR161	2084	16	30196586	30217974	loss	17644	5861594	79725345	Front_feet_conformation	region1inRegion2	21388
CNVR163	2109	16	53083153	53202189	gain	17644	5861594	79725345	Front_feet_conformation	region1inRegion2	119036
CNVR164	2111	16	56657347	56707690	loss	17644	5861594	79725345	Front_feet_conformation	region1inRegion2	50343
CNVR159	2034	16	6146177	6154424	loss	9575	6112871	6182280	Myristic_acid_content	region1inRegion2	8247
CNVR160	2055	16	12229596	12241067	loss-gain	12273	6890949	18970761	Interferon-gamma_to_interleukin-10_ratio	region1inRegion2	11471
CNVR160	2055	16	12229596	12241067	loss-gain	17856	6890949	18970761	CD4-positive,_CD8-positive_leukocyte_percentage	region1inRegion2	11471
CNVR160	2055	16	12229596	12241067	loss-gain	5484	6890949	22335189	Segmented_neutrophil_number	region1inRegion2	11471
CNVR160	2055	16	12229596	12241067	loss-gain	1179	7906590	22335189	Average_daily_gain	region1inRegion2	11471
CNVR160	2055	16	12229596	12241067	loss-gain	1180	7906590	22335189	Dressing_percentage	region1inRegion2	11471
CNVR160	2055	16	12229596	12241067	loss-gain	1181	7906590	22335189	Ham_percentage	region1inRegion2	11471
CNVR160	2055	16	12229596	12241067	loss-gain	1183	7906590	22335189	Feed_intake	region1inRegion2	11471
CNVR160	2055	16	12229596	12241067	loss-gain	17812	7906590	40229087	Shear_force	region1inRegion2	11471
CNVR161	2084	16	30196586	30215798	loss	17812	7906590	40229087	Shear_force	region1inRegion2	19212
CNVR161	2084	16	30196586	30217974	loss	17812	7906590	40229087	Shear_force	region1inRegion2	21388
CNVR160	2055	16	12229596	12241067	loss-gain	8877	7906590	43753218	Ear_weight	region1inRegion2	11471
CNVR161	2084	16	30196586	30215798	loss	8877	7906590	43753218	Ear_weight	region1inRegion2	19212
CNVR161	2084	16	30196586	30217974	loss	8877	7906590	43753218	Ear_weight	region1inRegion2	21388
CNVR160	2055	16	12229596	12241067	loss-gain	8878	7906590	43753218	Ear_weight	region1inRegion2	11471
CNVR161	2084	16	30196586	30215798	loss	8878	7906590	43753218	Ear_weight	region1inRegion2	19212
CNVR161	2084	16	30196586	30217974	loss	8878	7906590	43753218	Ear_weight	region1inRegion2	21388
CNVR160	2055	16	12229596	12241067	loss-gain	8879	7906590	43753218	Ear_area	region1inRegion2	11471
CNVR161	2084	16	30196586	30215798	loss	8879	7906590	43753218	Ear_area	region1inRegion2	19212
CNVR161	2084	16	30196586	30217974	loss	8879	7906590	43753218	Ear_area	region1inRegion2	21388
CNVR160	2055	16	12229596	12241067	loss-gain	8880	7906590	43753218	Ear_area	region1inRegion2	11471
CNVR161	2084	16	30196586	30215798	loss	8880	7906590	43753218	Ear_area	region1inRegion2	19212
CNVR161	2084	16	30196586	30217974	loss	8880	7906590	43753218	Ear_area	region1inRegion2	21388
CNVR160	2055	16	12229596	12241067	loss-gain	38071	7906590	83197113	CIE-b*	region1inRegion2	11471
CNVR161	2084	16	30196586	30215798	loss	38071	7906590	83197113	CIE-b*	region1inRegion2	19212
CNVR161	2084	16	30196586	30217974	loss	38071	7906590	83197113	CIE-b*	region1inRegion2	21388
CNVR163	2109	16	53083153	53202189	gain	38071	7906590	83197113	CIE-b*	region1inRegion2	119036
CNVR164	2111	16	56657347	56707690	loss	38071	7906590	83197113	CIE-b*	region1inRegion2	50343
CNVR160	2055	16	12229596	12241067	loss-gain	38115	7906590	83197113	Cooking_loss	region1inRegion2	11471
CNVR161	2084	16	30196586	30215798	loss	38115	7906590	83197113	Cooking_loss	region1inRegion2	19212
CNVR161	2084	16	30196586	30217974	loss	38115	7906590	83197113	Cooking_loss	region1inRegion2	21388
CNVR163	2109	16	53083153	53202189	gain	38115	7906590	83197113	Cooking_loss	region1inRegion2	119036
CNVR164	2111	16	56657347	56707690	loss	38115	7906590	83197113	Cooking_loss	region1inRegion2	50343
CNVR160	2055	16	12229596	12241067	loss-gain	38116	7906590	83197113	CIE-a*	region1inRegion2	11471
CNVR161	2084	16	30196586	30215798	loss	38116	7906590	83197113	CIE-a*	region1inRegion2	19212
CNVR161	2084	16	30196586	30217974	loss	38116	7906590	83197113	CIE-a*	region1inRegion2	21388
CNVR163	2109	16	53083153	53202189	gain	38116	7906590	83197113	CIE-a*	region1inRegion2	119036
CNVR164	2111	16	56657347	56707690	loss	38116	7906590	83197113	CIE-a*	region1inRegion2	50343
CNVR161	2084	16	30196586	30215798	loss	21423	22335189	42435187	Loin_muscle_area	region1inRegion2	19212
CNVR161	2084	16	30196586	30217974	loss	21423	22335189	42435187	Loin_muscle_area	region1inRegion2	21388
CNVR161	2084	16	30196586	30215798	loss	8812	22335189	65843170	Teat_number	region1inRegion2	19212
CNVR161	2084	16	30196586	30217974	loss	8812	22335189	65843170	Teat_number	region1inRegion2	21388
CNVR163	2109	16	53083153	53202189	gain	8812	22335189	65843170	Teat_number	region1inRegion2	119036

CNVR164	2111	16	56657347	56707690	loss	8812	22335189	65843170	Teat_number	region1inRegion2	50343
CNVR161	2084	16	30196586	30215798	loss	3212	22335189	71797057	backfat_at_last_rib	region1inRegion2	19212
CNVR161	2084	16	30196586	30217974	loss	3212	22335189	71797057	backfat_at_last_rib	region1inRegion2	21388
CNVR163	2109	16	53083153	53202189	gain	3212	22335189	71797057	backfat_at_last_rib	region1inRegion2	119036
CNVR164	2111	16	56657347	56707690	loss	3212	22335189	71797057	backfat_at_last_rib	region1inRegion2	50343
CNVR161	2084	16	30196586	30215798	loss	3659	22335189	71797057	Total_body_fat_tissue_linear	region1inRegion2	19212
CNVR161	2084	16	30196586	30217974	loss	3659	22335189	71797057	Total_body_fat_tissue_linear	region1inRegion2	21388
CNVR163	2109	16	53083153	53202189	gain	3659	22335189	71797057	Total_body_fat_tissue_linear	region1inRegion2	119036
CNVR164	2111	16	56657347	56707690	loss	3659	22335189	71797057	Total_body_fat_tissue_linear	region1inRegion2	50343
CNVR161	2084	16	30196586	30215798	loss	38070	22335189	71797057	Loin_fat_percentage	region1inRegion2	19212
CNVR161	2084	16	30196586	30217974	loss	38070	22335189	71797057	Loin_fat_percentage	region1inRegion2	21388
CNVR163	2109	16	53083153	53202189	gain	38070	22335189	71797057	Loin_fat_percentage	region1inRegion2	119036
CNVR164	2111	16	56657347	56707690	loss	38070	22335189	71797057	Loin_fat_percentage	region1inRegion2	50343
CNVR161	2084	16	30196586	30215798	loss	38114	22335189	83197113	Loin_muscle_area	region1inRegion2	19212
CNVR161	2084	16	30196586	30217974	loss	38114	22335189	83197113	Loin_muscle_area	region1inRegion2	21388
CNVR163	2109	16	53083153	53202189	gain	38114	22335189	83197113	Loin_muscle_area	region1inRegion2	119036
CNVR164	2111	16	56657347	56707690	loss	38114	22335189	83197113	Loin_muscle_area	region1inRegion2	50343
CNVR161	2084	16	30196586	30215798	loss	12309	22738076	31440121	Hemoglobin	region1inRegion2	19212
CNVR161	2084	16	30196586	30217974	loss	12309	22738076	31440121	Hemoglobin	region1inRegion2	21388
CNVR161	2084	16	30196586	30215798	loss	37546	22738076	31440121	CSFV_antibody_level	region1inRegion2	19212
CNVR161	2084	16	30196586	30217974	loss	37546	22738076	31440121	CSFV_antibody_level	region1inRegion2	21388
CNVR161	2084	16	30196586	30215798	loss	12815	23987098	67649164	Adipocyte_diameter	region1inRegion2	19212
CNVR161	2084	16	30196586	30217974	loss	12815	23987098	67649164	Adipocyte_diameter	region1inRegion2	21388
CNVR163	2109	16	53083153	53202189	gain	12815	23987098	67649164	Adipocyte_diameter	region1inRegion2	119036
CNVR164	2111	16	56657347	56707690	loss	12815	23987098	67649164	Adipocyte_diameter	region1inRegion2	50343
CNVR161	2084	16	30196586	30215798	loss	4257	24826912	48492012	Nonfunctional_nipples	region1inRegion2	19212
CNVR161	2084	16	30196586	30217974	loss	4257	24826912	48492012	Nonfunctional_nipples	region1inRegion2	21388
CNVR161	2084	16	30196586	30215798	loss	3978	25953165	35060804	Number_of_ribs	region1inRegion2	19212
CNVR161	2084	16	30196586	30217974	loss	3978	25953165	35060804	Number_of_ribs	region1inRegion2	21388
CNVR161	2084	16	30196586	30215798	loss	1182	25953165	67649164	Creatine_kinase_level	region1inRegion2	19212
CNVR161	2084	16	30196586	30217974	loss	1182	25953165	67649164	Creatine_kinase_level	region1inRegion2	21388
CNVR163	2109	16	53083153	53202189	gain	1182	25953165	67649164	Creatine_kinase_level	region1inRegion2	119036
CNVR164	2111	16	56657347	56707690	loss	1182	25953165	67649164	Creatine_kinase_level	region1inRegion2	50343
CNVR161	2084	16	30196586	30215798	loss	1178	25953165	83197113	Conductivity_24_hours_post-mortem	region1inRegion2	19212
CNVR161	2084	16	30196586	30217974	loss	1178	25953165	83197113	Conductivity_24_hours_post-mortem	region1inRegion2	21388
CNVR163	2109	16	53083153	53202189	gain	1178	25953165	83197113	Conductivity_24_hours_post-mortem	region1inRegion2	119036
CNVR164	2111	16	56657347	56707690	loss	1178	25953165	83197113	Conductivity_24_hours_post-mortem	region1inRegion2	50343
CNVR161	2084	16	30196586	30215798	loss	64667	29510913	30409709	Lumbar_vertebra_number	region1inRegion2	19212
CNVR161	2084	16	30196586	30217974	loss	64667	29510913	30409709	Lumbar_vertebra_number	region1inRegion2	21388
CNVR161	2084	16	30196586	30215798	loss	18286	29897368	30283095	mummified_pigs	region1inRegion2	19212
CNVR161	2084	16	30196586	30217974	loss	18286	29897368	30283095	mummified_pigs	region1inRegion2	21388
CNVR161	2084	16	30196586	30215798	loss	101593	30204508	30204548	Arachidic_acid_to_stearic_acid_ratio	region2inRegion1	40
CNVR161	2084	16	30196586	30217974	loss	101593	30204508	30204548	Arachidic_acid_to_stearic_acid_ratio	region2inRegion1	40
CNVR160	2055	16	12229596	12241067	loss-gain	22330	36353815	9287983	Loin_muscle_area	region2inRegion1	-27065832
CNVR161	2084	16	30196586	30215798	loss	22330	36353815	9287983	Loin_muscle_area	region2inRegion1	-27065832
CNVR161	2084	16	30196586	30217974	loss	22330	36353815	9287983	Loin_muscle_area	region2inRegion1	-27065832
CNVR163	2109	16	53083153	53202189	gain	6370	40229087	71797057	Creatinine_level	region1inRegion2	119036
CNVR164	2111	16	56657347	56707690	loss	6370	40229087	71797057	Creatinine_level	region1inRegion2	50343
CNVR163	2109	16	53083153	53202189	gain	7519	40229087	71797057	Body_temperature	region1inRegion2	119036
CNVR164	2111	16	56657347	56707690	loss	7519	40229087	71797057	Body_temperature	region1inRegion2	50343
CNVR163	2109	16	53083153	53202189	gain	21425	42435187	71797057	CIE-a*	region1inRegion2	119036
CNVR164	2111	16	56657347	56707690	loss	21425	42435187	71797057	CIE-a*	region1inRegion2	50343
CNVR163	2109	16	53083153	53202189	gain	21334	42857275	71797057	White_blood_cell_counts	region1inRegion2	119036
CNVR164	2111	16	56657347	56707690	loss	21334	42857275	71797057	White_blood_cell_counts	region1inRegion2	50343
CNVR163	2109	16	53083153	53202189	gain	9574	48492012	58970415	Myristic_acid_content	region1inRegion2	119036
CNVR164	2111	16	56657347	56707690	loss	9574	48492012	58970415	Myristic_acid_content	region1inRegion2	50343
CNVR164	2111	16	56657347	56707690	loss	131479	56666649	56666689	Eicosadienoic_acid_content	region2inRegion1	40
CNVR164	2111	16	56657347	56707690	loss	31898	56704434	56919380	Corpus_luteum_number	overlapTail	3257
CNVR166	2168	17	53147051	53158077	gain	5231	435637	69701581	Body_weight_(weaning)	region1inRegion2	11026
CNVR166	2168	17	53147051	53158077	gain	37564	4082851	64462043	Actinobacillus_pleuropneumoniae_susceptibility	region1inRegion2	11026
CNVR166	2168	17	53147051	53158077	gain	37575	4609122	66928144	Actinobacillus_pleuropneumoniae_susceptibility	region1inRegion2	11026
CNVR166	2168	17	53147051	53158077	gain	5229	16015836	69701581	Teat_number	region1inRegion2	11026
CNVR166	2168	17	53147051	53158077	gain	17728	32082161	66928144	Triglyceride_level	region1inRegion2	11026
CNVR166	2168	17	53147051	53158077	gain	9657	32082161	66928144	Haptoglobin_concentration	region1inRegion2	11026
CNVR166	2168	17	53147051	53158077	gain	12344	37174855	65306657	Interferon-gamma_level	region1inRegion2	11026
CNVR166	2168	17	53147051	53158077	gain	18026	45880990	57578641	Adipocyte_diameter	region1inRegion2	11026
CNVR166	2168	17	53147051	53158077	gain	16842	45880990	67855516	backfat_at_last_rib	region1inRegion2	11026
CNVR166	2168	17	53147051	53158077	gain	1186	46303048	67855516	Carcass_length	region1inRegion2	11026
CNVR166	2168	17	53147051	53158077	gain	1191	46303048	67855516	backfat_at_last_rib	region1inRegion2	11026
CNVR166	2168	17	53147051	53158077	gain	1192	46303048	67855516	Fat-cuts_percentage	region1inRegion2	11026
CNVR166	2168	17	53147051	53158077	gain	5471	48731060	66928144	Lymphocyte_number	region1inRegion2	11026
CNVR166	2168	17	53147051	53158077	gain	7521	48731060	66928144	Sarcocystis_miescheriana_IgG_levels	region1inRegion2	11026
CNVR166	2168	17	53147051	53158077	gain	3299	48731060	67855516	45_min-24_h_pH_decline	region1inRegion2	11026
CNVR166	2168	17	53147051	53158077	gain	2967	51909620	64337706	Meat_color_score	region1inRegion2	11026
CNVR166	2168	17	53147051	53158077	gain	2968	51909620	64337706	Meat_color-L	region1inRegion2	11026
CNVR166	2168	17	53147051	53158077	gain	2969	51909620	64337706	Meat_color-L	region1inRegion2	11026
CNVR166	2168	17	53147051	53158077	gain	4010	51909620	64337706	CIE-b*	region1inRegion2	11026
CNVR166	2168	17	53147051	53158077	gain	12274	51909620	67855516	Interferon-gamma_to_interleukin-10_ratio	region1inRegion2	11026
CNVR166	2168	17	53147051	53158077	gain	129	51909620	67855516	Meat_color_score	region1inRegion2	11026
CNVR166	2168	17	53147051	53158077	gain	130	51909620	67855516	Meat_color-L	region1inRegion2	11026
CNVR166	2168	17	53147051	53158077	gain	131	51909620	67855516	Meat_color-L	region1inRegion2	11026
CNVR166	2168	17	53147051	53158077	gain	132	51909620	67855516	Average_lactate	region1inRegion2	11026
CNVR166	2168	17	53147051	53158077	gain	133	51909620	67855516	Average_glycolytic_potential	region1inRegion2	11026
CNVR166	2168	17	53147051	53158077	gain	17858	51909620	67855516	CD4-positive,_CD8-positive_leukocyte_percentage	region1inRegion2	11026
