# Supplementary material for: Identification of copy number variations using high density whole-genome single nucleotide polymorphism markers in Chinese Dongxiang spotted pigs
Source: Asian-Australas J Anim Sci. 2019 Feb 7;32(12):1809–15. doi: 10.5713/ajas.18.0696 (PMC6819687; doi:10.5713/ajas.18.0696)
Supplement: Supplementary file 6 [file ajas-18-0696-suppl6.pdf]

Table S6. Unique CNVRs identified in this study and their overlapped QTLs

CNVR ID	CNV ID	Chr.	CNVR Start	CNVR End	State	QTL ID	QTL Start	QTL End	QTL Name	Relationship	OverlapLength	Gene
CNVR1	83	1	88942731	88998960	gain	126114	72534652	95300134	Top_line_conformation	region1inRegion2	56229	
CNVR2	88	1	91934647	91942954	loss	126114	72534652	95300134	Top_line_conformation	region1inRegion2	8307	NT5DC1
CNVR8	156	1	170118820	170121900	gain	1271	168669767	210644363	Ham_fat_weight	region1inRegion2	3080	
CNVR9	191	1	197269718	197274380	gain	1271	168669767	210644363	Ham_fat_weight	region1inRegion2	4662	
CNVR37	422	2	107437562	107451870	gain	6240	97794151	125044122	Body_weight_(15_weeks)	region1inRegion2	14308	LNPEP
CNVR37	422	2	107437562	107451870	gain	1278	100385940	113025492	Ratio_of_back_bone_and_meat_weight_to_ha	region1inRegion2	14308	LNPEP
CNVR47	510	3	56589884	56595835	loss	6320	46839405	100216075	Blood_pH	region1inRegion2	5951	
CNVR48	519	3	61793403	61809935	loss	6320	46839405	100216075	Blood_pH	region1inRegion2	16532	
CNVR49	524	3	67785626	67805349	gain	6320	46839405	100216075	Blood_pH	region1inRegion2	19723	
CNVR50	539	3	83892565	83943514	gain	6320	46839405	100216075	Blood_pH	region1inRegion2	50949	
CNVR47	510	3	56589884	56595835	loss	11852	56047063	101551719	Feet_and_leg_conformation	region1inRegion2	5951	
CNVR48	519	3	61793403	61809935	loss	11852	56047063	101551719	Feet_and_leg_conformation	region1inRegion2	16532	
CNVR49	524	3	67785626	67805349	gain	11852	56047063	101551719	Feet_and_leg_conformation	region1inRegion2	19723	
CNVR50	539	3	83892565	83943514	gain	11852	56047063	101551719	Feet_and_leg_conformation	region1inRegion2	50949	
CNVR55	639	4	52447075	52460832	gain	161	4064787	52626380	Taste_intensity	region1inRegion2	13757	
CNVR55	639	4	52447075	52460832	gain	6406	46783250	52963684	Arachidonic_acid_content	region1inRegion2	13757	
CNVR56	649	4	55682568	55685476	loss	632	52626380	102054630	Subcutaneous_fat_thickness	region1inRegion2	2908	ATP6V0D2
CNVR57	668	4	67469665	67474606	loss	632	52626380	102054630	Subcutaneous_fat_thickness	region1inRegion2	4941	
CNVR58	683	4	88694499	88704809	gain	632	52626380	102054630	Subcutaneous_fat_thickness	region1inRegion2	10310	KIFAP3
CNVR58	683	4	88694499	88704809	gain	32070	86728309	92100663	Eicosatrienoic_acid_content	region1inRegion2	10310	KIFAP3
CNVR60	727	5	18191073	18199920	gain	2983	7985523	19558279	Myofibril_fragmentation_index	region1inRegion2	8847	
CNVR65	755	5	39192570	39207538	gain	2987	35377762	56295399	Taste_intensity	region1inRegion2	14968	TRHDE
CNVR66	766	5	52064895	52069376	loss	2987	35377762	56295399	Taste_intensity	region1inRegion2	4481	
CNVR69	862	6	95466297	95473943	gain	688	29979924	117131464	Smell_intensity	region1inRegion2	7646	
CNVR70	871	6	107004191	107039849	loss	688	29979924	117131464	Smell_intensity	region1inRegion2	35658	
CNVR69	862	6	95466297	95473943	gain	156	83821055	129740986	post-stress_mitogen_induced_IL-2_activity	region1inRegion2	7646	
CNVR70	871	6	107004191	107039849	loss	156	83821055	129740986	post-stress_mitogen_induced_IL-2_activity	region1inRegion2	35658	
CNVR69	862	6	95466297	95473943	gain	12060	91030131	146365886	Cholesterol_level_in_meat	region1inRegion2	7646	
CNVR70	871	6	107004191	107039849	loss	12060	91030131	146365886	Cholesterol_level_in_meat	region1inRegion2	35658	
CNVR69	862	6	95466297	95473943	gain	12061	91030131	146365886	Flavor_score	region1inRegion2	7646	
CNVR70	871	6	107004191	107039849	loss	12061	91030131	146365886	Flavor_score	region1inRegion2	35658	
CNVR69	862	6	95466297	95473943	gain	18643	94382869	146365886	Leptin_level	region1inRegion2	7646	
CNVR70	871	6	107004191	107039849	loss	18643	94382869	146365886	Leptin_level	region1inRegion2	35658	
CNVR69	862	6	95466297	95473943	gain	3652	94382869	146365886	Backfat_linear_at_tenth_rib	region1inRegion2	7646	
CNVR70	871	6	107004191	107039849	loss	3652	94382869	146365886	Backfat_linear_at_tenth_rib	region1inRegion2	35658	
CNVR70	871	6	107004191	107039849	loss	66032	97895295	124113659	Leptin_level	region1inRegion2	35658	
CNVR72	923	7	10444634	10478517	gain	6375	4252710	11625414	Bilirubin_level	region1inRegion2	33883	
CNVR72	923	7	10444634	10478517	gain	3954	7434494	20633784	Carcass_width_(minimum)	region1inRegion2	33883	
CNVR72	923	7	10444634	10478517	gain	9105	10242996	10457106	Adipocyte_volume	overlapHead	12473	
CNVR83	957	7	28525787	28565312	gain	5817	28117351	30150257	Cannon_bone_circumference	region1inRegion2	39525	
CNVR83	957	7	28525787	28565312	gain	5818	28117351	30150257	Cannon_bone_circumference	region1inRegion2	39525	
CNVR88	1002	7	84728780	84749202	gain	106282	84047647	85068543	Monounsaturated_fatty_acid_content	region1inRegion2	20422	
CNVR96	1086	8	44371710	44447879	gain	4267	39840424	77429212	N1c-positive_leukocyte_number	region1inRegion2	76169	
CNVR97	1112	8	64473379	64515977	loss	4267	39840424	77429212	N1c-positive_leukocyte_number	region1inRegion2	42598	
CNVR98	1116	8	68684763	68710413	gain	4267	39840424	77429212	N1c-positive_leukocyte_number	region1inRegion2	25650	
CNVR96	1086	8	44371710	44447879	gain	4268	39840424	77429212	IgM-positive_leukocyte_number	region1inRegion2	76169	
CNVR97	1112	8	64473379	64515977	loss	4268	39840424	77429212	IgM-positive_leukocyte_number	region1inRegion2	42598	
CNVR98	1116	8	68684763	68710413	gain	4268	39840424	77429212	IgM-positive_leukocyte_number	region1inRegion2	25650	
CNVR96	1086	8	44371710	44447879	gain	15106	39840424	124156612	Glucose_level	region1inRegion2	76169	
CNVR97	1112	8	64473379	64515977	loss	15106	39840424	124156612	Glucose_level	region1inRegion2	42598	
CNVR98	1116	8	68684763	68710413	gain	15106	39840424	124156612	Glucose_level	region1inRegion2	25650	
CNVR99	1129	8	85727251	85735420	gain	15106	39840424	124156612	Glucose_level	region1inRegion2	8169	NR3C2
CNVR100	1169	8	122108843	122121928	loss	15106	39840424	124156612	Glucose_level	region1inRegion2	13085	LEF1
CNVR97	1112	8	64473379	64515977	loss	6374	46427317	120532322	Bilirubin_level	region1inRegion2	42598	
CNVR98	1116	8	68684763	68710413	gain	6374	46427317	120532322	Bilirubin_level	region1inRegion2	25650	
CNVR99	1129	8	85727251	85735420	gain	6374	46427317	120532322	Bilirubin_level	region1inRegion2	8169	NR3C2
CNVR97	1112	8	64473379	64515977	loss	22149	62200211	70360798	Granulocyte_percentage	region1inRegion2	42598	
CNVR98	1116	8	68684763	68710413	gain	22149	62200211	70360798	Granulocyte_percentage	region1inRegion2	25650	
CNVR97	1112	8	64473379	64515977	loss	22150	62200211	70360798	Granulocyte_percentage	region1inRegion2	42598	
CNVR98	1116	8	68684763	68710413	gain	22150	62200211	70360798	Granulocyte_percentage	region1inRegion2	25650	
CNVR100	1169	8	122108843	122121928	loss	2994	120532322	124156612	Myofibril_fragmentation_index	region1inRegion2	13085	LEF1
CNVR107	1227	9	21980507	21992085	gain	18644	11066889	23144816	Leptin_level	region1inRegion2	11578	SYTL2
CNVR107	1227	9	21980507	21992085	gain	5149	11066889	23144816	Belly_fat_area	region1inRegion2	11578	SYTL2
CNVR111	1316	9	108875949	108885208	gain	5143	71832311	119109362	Neck_weight	region1inRegion2	9259	
CNVR119	1427	10	71830224	71835029	gain	3822	64951351	72237308	Cholesterol_level_in_meat	region1inRegion2	4805	DDBX_BOVIN
CNVR134	1573	13	16191826	16221816	gain	7515	5836383	27210688	Sarcocystis_miescheriana_IgM_levels	region1inRegion2	29990	
CNVR135	1578	13	19139627	19152872	gain	7515	5836383	27210688	Sarcocystis_miescheriana_IgM_levels	region1inRegion2	13245	
CNVR138	1636	13	111201690	111225859	gain	18357	58690821	157823757	Embryo_weight	region1inRegion2	24169	
CNVR139	1656	13	131028348	131044332	gain	18357	58690821	157823757	Embryo_weight	region1inRegion2	15984	KLHL24
CNVR149	1816	14	56382005	56403580	loss	12272	27531879	60644503	Interferon-gamma_to_interleukin-10_ratio	region1inRegion2	21575	
CNVR160	2055	16	12229596	12241067	loss-gain	12273	6890949	18970761	Interferon-gamma_to_interleukin-10_ratio	region1inRegion2	11471	
CNVR160	2055	16	12229596	12241067	loss-gain	17856	6890949	18970761	CD4-positive,_CD8-positive_leukocyte_perce	region1inRegion2	11471	
CNVR166	2168	17	53147051	53158077	gain	12274	51909620	67855516	Interferon-gamma_to_interleukin-10_ratio	region1inRegion2	11026	
CNVR166	2168	17	53147051	53158077	gain	17858	51909620	67855516	CD4-positive,_CD8-positive_leukocyte_perce	region1inRegion2	11026	
